# Effect of dialyzer membrane materials on survival in chronic hemodialysis patients: Results from the annual survey of the Japanese Nationwide Dialysis Registry

**DOI:** 10.1371/journal.pone.0184424

**Published:** 2017-09-14

**Authors:** Masanori Abe, Takayuki Hamano, Atsushi Wada, Shigeru Nakai, Ikuto Masakane, on behalf of the Renal Data Registry Committee, Japanese Society for Dialysis Therapy

**Affiliations:** 1 Division of Nephrology, Hypertension and Endocrinology, Nihon University School of Medicine, Tokyo, Japan; 2 Department of Comprehensive Kidney Disease Research, Osaka University Graduate School of Medicine, Osaka, Japan; 3 Department of Nephrology, Kitasaito Hospital, Asahikawa, Japan; 4 Department of Clinical Engineering, Fujita Health University, Aichi, Japan; 5 Yabuki Hospital, Yamagata, Japan; Kaohsiung Medical University Hospital, TAIWAN

## Abstract

**Background:**

Little information is available regarding which type of dialyzer membrane results in good prognosis in patients on chronic hemodialysis. Therefore, we conducted a cohort study from a nationwide registry of hemodialysis patients in Japan to establish the association between different dialyzer membranes and mortality rates.

**Methods:**

We followed 142,412 patients on maintenance hemodialysis (female, 39.1%; mean age, 64.8 ± 12.3 years; median dialysis duration, 7 [4–12] years) for a year from 2008 to 2009. We included patients treated with seven types of high-flux dialyzer membranes at baseline, including cellulose triacetate (CTA), ethylene vinyl alcohol (EVAL), polyacrylonitrile (PAN), polyester polymer alloy (PEPA), polyethersulfone (PES), polymethylmethacrylate (PMMA), and polysulfone (PS). Cox regression was used to estimate the association between baseline dialyzers and all-cause mortality as hazard ratios (HRs) and 95% confidence intervals for 1-year mortality adjusting for potential confounders, and propensity score matching analysis was performed.

**Results:**

The distribution of patients treated with each membrane was as follows: PS (56.0%), CTA (17.3%), PES (12.0%), PEPA (7.5%), PMMA (4.9%), PAN (1.2%), and EVAL (1.1%). When data were adjusted using basic factors, with PS as a reference group, the mortality rate was significantly higher in all groups except for the PES group. When data were further adjusted for dialysis-related factors, HRs were significantly higher for the CTA, EVAL, and PEPA groups. When the data were further adjusted for nutrition-and inflammation-related factors, HRs were significantly lower for the PMMA and PES groups compared with the PS group. After propensity score matching, HRs were significantly lower for the PMMA group than for the PS group.

**Conclusion:**

The results suggest that the use of different membrane types may affect mortality in hemodialysis patients. However, further long-term prospective studies are needed to clarify these findings, including whether the use of the PMMA membrane can improve prognosis.

## Introduction

Dialyzer technology is undergoing development and moving towards high permeability and biocompatibility; therefore, there is an urgent need for robust evidence regarding the performance of various types of dialyzers. Dialyzers are classified to two types, low-flux and high-flux membrane dialyzers. High-flux dialyzers are recommended for good outcomes in hemodialysis patients [1,2]. The Kidney Disease Outcomes Quality Initiative guidelines discourage the use of cellulose membranes with poor biocompatibility [3]. The European Renal Best Practice guidelines recommend the use of high-flux membranes with large pores and high biocompatibility to improve morbidity and mortality [4]. Low-flux dialyzers are defined by an ultrafiltration rate <15 mL/mmHg/h and β_2_-microglobulin (β2M) clearance <15 mL/min, while high-flux dialyzers are defined by an ultrafiltration rate ≥15 mL/mmHg/h and β2M clearance ≥15 mL/min [1]. However, in Japan before 2016, dialyzers were classified from types I to V based on their β2M clearance of <10, <30 <50, <70, and >70 mL/min, respectively, at a blood flow rate of 200 mL/min and a dialysate flow rate of 500 mL/min [5]. Type II to V dialyzers are classified as high-flux membranes; only type I dialyzers are classified as low-flux membranes. High-performance membrane (HPM) dialyzers are quickly becoming conventional in Japan, but this is not the case in other countries. Moreover, the selection criteria for dialyzer membranes in the Japanese guidelines differ distinctly from those used in other countries [1]. The main purpose of HPMs is to eliminate uremic substances with molecular weights of 10,000–30,000 Da. The HPM concept, therefore, comprises all of the following characteristics: (1) high hydraulic permeability; (2) high solute permeability, especially for “middle molecules”; and (3) high biocompatibility [5]. In particular, types IV and V, which are classified as HPM dialyzers and composed of synthetic membranes, are used for >90% of Japanese hemodialysis patients [1]. Therefore, many Japanese patients are currently treated with HPM dialyzers, including high-flux membrane dialyzers. Furthermore, several different membrane materials are used in HPM dialyzers. However, there is little information available regarding which type of membrane results in good prognosis. Therefore, we conducted a cohort study from a nationwide registry of hemodialysis patients in Japan to clarify the association between different HPM dialyzers and mortality rates.

## Subjects and methods

The protocol of this study was approved by the Ethics Committee of the JSDT (JRDR-SAF-15009), and all procedures fully adhered to the Declaration of Helsinki. The study was registered with the University Hospital Medical Information Network (UMIN000018641).

### Database creation

The data were obtained from annual nationwide surveys of dialysis patients, which comprise the Japanese Renal Data Registry (JRDR) system, conducted by the Japanese Society for Dialysis Therapy (JSDT). The JSDT has been conducting an annual questionnaire survey of dialysis facilities throughout Japan since 1968, and several papers based on these surveys have been published [6–9]. Since 1983, the JSDT has been compiling a computer-based registry. Details regarding the inception, limitations, validity, variables, and questionnaires used in the study are available online at the JSDT homepage, (http://www.jsdt.or.jp). Year-end survey questionnaires are sent to all dialysis facilities in Japan each year. The questionnaires are administered by volunteers from among the doctors and staff of the facilities, the principal investigators in each prefecture, and JSDT committee members. In addition to the regular questionnaire, new survey items are added each year. In 2008, new questions were added regarding the dialyzers used. Data covered 282,622 patients undergoing dialysis at 4,072 facilities in the 2008 survey, and 290,675 patients at 4,125 facilities in the 2009 survey [10,11]. The study population consisted of patients who underwent maintenance dialysis between December 2008 and December 2009. We included patients who underwent maintenance hemodialysis 3 times a week, who had received maintenance dialysis for at least 2 years at the end of the year 2008 and were treated with one of the seven major dialyzers, namely, cellulose triacetate (CTA), ethylene vinyl alcohol (EVAL), polyacrylonitrile (PAN), polyester polymer alloy (PEPA), polyethersulfone (PES), polymethylmethacrylate (PMMA), and polysulfone (PS) membranes (S1 and S2 Tables). Patients were followed for outcomes through 31 December 2009 and 1-year all-cause mortality was analyzed retrospectively. We excluded patients who had been dialyzed fewer than 3 times a week or fewer than 2 h per treatment, those who had been treated with dialyzers other than the abovementioned seven dialyzers, those who had received peritoneal dialysis, those with a history of organ transplantation, those aged <20 years, and those whose records covering date of birth, dialysis initiation, or outcomes were incomplete.

Blood samples were drawn and analyzed at each dialysis center, typically within 24 h of the sample being taken. The most recent values, including serum albumin, hemoglobin, calcium, phosphate, total cholesterol, C-reactive protein (CRP), β2M, adequacy of dialysis treatment (single-pool Kt/V), normalized protein catabolic rate (nPCR), and % creatinine generation rate (%CGR) at the time of the survey were collected. These variables were measured at least monthly in many facilities because doing so is recommended by the JSDT guidelines [1].

**Fig 1:**
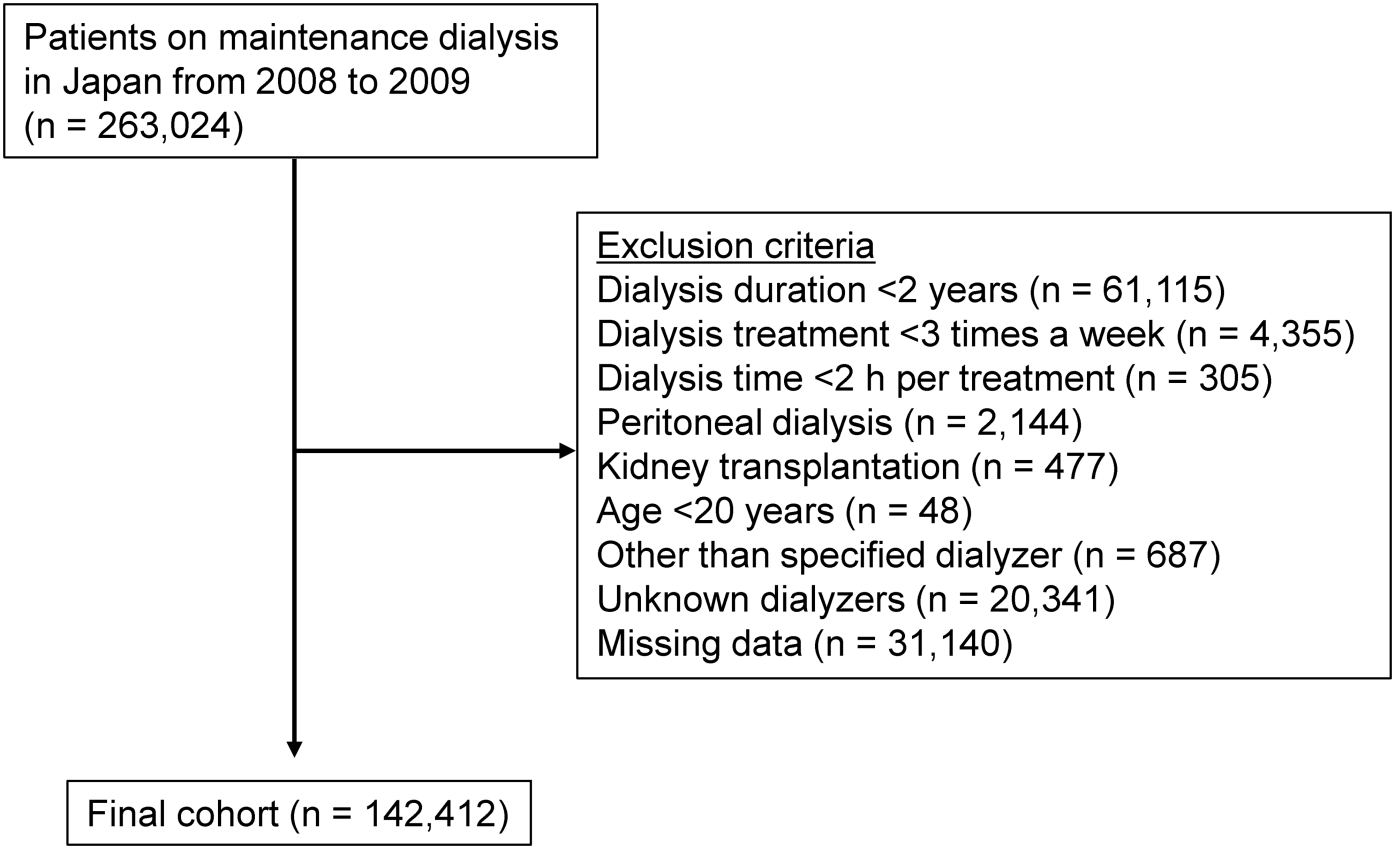
Flowchart of study participants.

Overall, 263,024 patients were registered at the end of year 2008. After exclusions, 142,412 patients remained (Fig 1). Demographic data and details of medical history were collected, with information on age, sex, dialysis duration, primary diseases of end-stage kidney disease, height, post-dialysis body weight, types of dialyzers, and history of vascular complications (cerebral infarction, cerebral hemorrhage, myocardial infarction, or limb amputation). Outcomes were examined at the end of 2009 using the JSDT 2009 database ID number and other pertinent identifiers to determine outcome such as death, kidney transplantation, and withdrawal from the registry.

### Statistical methods

Data were summarized using proportions, with mean ± standard deviation or median [interquartile range] as appropriate. Categorical variables were analyzed using the chi-square test, and continuous variables were compared using the *t*-test, as appropriate. Categorical data were compared between groups by using repeated-measures analysis of variance and Tukey’s honestly significant difference test or the Kruskal–Wallis test, as appropriate. Laboratory data were refined using these limits: height, 120–200 cm; body weight, 20–150 kg; albumin, 1.0–5.5 g/dL; hemoglobin, 5.0–20.0 g/dL; Kt/V, 0.5–4.0; and nPCR, 0.3–2.0.

### Outcome analysis by basic factors, dialysis dose, and nutrition factors

Survival analyses with Cox proportional hazards regression model were used to examine whether baseline basic factors including age, sex, dialysis duration, and presence or absence of diabetes and cardiovascular comorbidity predicted survival for up to 1 year of follow-up. We divided patients into seven a priori categories based on dialysis duration (2 to <5, 5 to <10, 10 to <15, 15 to <20, 20 to <25, 25 to <30, and ≥30 years) to examine the dose–response association between dialysis duration categories and death risk. Additional analyses were performed and adjusted for dialysis-related factors such as dialysis dose as assessed by Kt/V, and high- or low-flux membranes. We divided patients into eight a priori categories based on single-pool Kt/V: <0.8 and ≥2.0, with increments of 0.2 in between, to examine the dose–response association between Kt/V categories and death risk. Each dialyzer was divided into two types according to criteria for high-flux (β2M clearance ≥15 mL/min and ultrafiltration rate ≥15 mL/mmHg/h) and for low-flux membranes (β2M clearance <15 mL/min and ultrafiltration rate <15 mL/mmHg/h). Additional analyses were performed with adjustment for nutrition and inflammation factors, including serum albumin values, hemoglobin levels, total cholesterol values, body mass index (BMI), nPCR, %CGR, and CRP levels. To examine the dose–response associations between these categories and death risk, we divided patients into six a priori categories based on nPCR <0.5 and ≥1.3 g/kg/day, with increments of 0.2 g/kg/day in between; serum albumin <3.0 and ≥4.5 g/dL, with increments of 0.5 g/dL in between; calcium adjusted for serum albumin <8.5 and ≥10.5 mg/dL, with increments of 1.0 mg/dL in between; phosphate <3.5 and ≥8.5 mg/dL, with increments of 1.0 mg/dL in between; total cholesterol <80 and ≥240 mg/dL, with increments of 40 mg/dL in between; BMI: <16 and ≥28 kg/m^2^, with increments of 2 kg/m^2^ in between; and %CGR: <60% and ≥130%, with increments of 10% in between [12–15]. Age, CRP levels, and hemoglobin levels were analyzed as continuous variables.

### Outcome analysis by seven types of dialyzer membranes

Survival analyses with Cox proportional hazards regression were used to examine whether different types of dialyzer membranes predicted survival for up to 1 year of follow-up. The final analysis examined the associations between types of dialyzer membranes and all-cause mortality. We divided the patients into seven groups based on which dialyzer membrane was used. Models were analyzed when adjusted for the abovementioned basic factors, dialysis dose, and nutritional- and inflammation-factors measured at the study baseline (S1 File). The PS group was defined as the reference group because it is the most widely used dialyzer worldwide.

To reduce potential confounding and treatment selection bias, we adjusted the significant difference in baseline covariates by propensity score matching. We calculated the propensity score for factors contributing to mortality including the abovementioned basic factors, dialysis dose, and nutritional- and inflammation factors, which were examined using univariate Cox proportional hazards regression analysis. The score was then used to match patients in the PS group as a reference with patients in the other membrane groups in a 1:1 ratio, resulting in 10,592, 571, 764, 4,683, 7,480, and 3,835 matched pairs (CTA, EVAL, PAN, PEPA, PES, and PMMA membranes, respectively). Moreover, all-cause mortality was compared for propensity score-matched patients.

Missing covariate data were imputed by the conventional method for multivariate regression as appropriate. For all analyses, results were considered statistically significant if P < 0.05. All analyses were carried out using JMP^®^ version 13.0 (SAS Institute, Cary, NC).

## Results

### Study characteristics

The baseline characteristics of the patients in our cohort are shown in Table 1. In this cohort, which comprised 142,412 patients, average values were as follows: age, 64.8 ± 12.3 years; dialysis duration, 7 [4–12] years; female patients, 39.1%; BMI, 21.1 ± 3.5; comorbidity of cardiovascular diseases (CVD), including coronary artery disease, stroke, and limb amputation, 21.8%; albumin, 3.7 ± 0.4 g/dL; and hemoglobin, 10.4 ± 1.2 g/dL. During the 1-year observational period (December 2008-December 2009), 10,163 patients (7.1%) died, and 132,249 patients (92.9%) patients were alive at the end of the observation period.

**Table 1:** Demographic, clinical, and laboratory values in 142,412 hemodialysis patients.

Variables	All patients
n	142,412
Age (years)	64.8 ± 12.3
Sex (% female)	39.1
HD duration (years) ^a^	7 [4–12]
2–5 years (%)	32.0
5–10 years (%)	32.8
10–15 years (%)	26.5
15–20 years (%)	8.5
20–25 years (%)	5.0
25–30 years (%)	3.2
≥ 30 years (%)	2.0
Characteristics of dialyzers (%)	
Low flux	0.7
High flux	99.3
Surface area of dialyzers (m^2^)	1.69 ± 0.34
Presence of DM (%)	30.8
Comorbidity of CVD (%)	21.8
Body mass index (kg/m^2^)	21.1 ± 3.5
Hemoglobin (g/dL)	10.4 ± 1.2
Serum albumin (g/dL)	3.7 ± 0.4
Total cholesterol (mg/dL)	153 ± 36
Calcium (mg/dL)	9.1 ± 0.8
Phosphate (mg/dL)	5.3 ± 1.4
β_2_-microglobulin (mg/L)	27.5 ± 6.7
C-reactive protein (mg/dL)	0.11 [0.05–0.37]
Kt/V	1.43 ± 0.29
nPCR (g/kg/day)	0.89 ± 0.18
%CGR (%)	100 ± 25

^a^ Ranges are inclusive on the lower end and exclusive on the upper end.

CVD, cardiovascular disease; nPCR, normalized protein catabolic rate; %CGR, % creatinine generation rate.

### All-cause mortality according to basic factors, dialysis dose, and nutrition factors at enrollment

The hazard ratios (HRs) for variables evaluated as potential predictors of mortality in all the patients are presented in Table 2. Male sex, increasing age, dialysis duration, presence of DM, and comorbidity of CVD were significant predictors of mortality. Higher dialysis dose, as assessed by single-pool Kt/V was associated with lower mortality risk. Furthermore, poorer nutritional status and increased inflammatory status, such as lower nPCR, lower serum albumin, lower hemoglobin, lower total cholesterol, lower BMI, lower %CGR, and higher CRP were also associated with higher mortality rate in patients on hemodialysis.

**Table 2:** HRs and 95% CIs for variables evaluated as potential predictors of mortality among all the patients.

Factors	HR	95% CI	P value
Sex			
Male	1.00	Reference	Reference
Female	0.90	0.86–0.94	<0.0001
Age			
1 year increase	1.07	1.06–1.07	<0.0001
Hemodialysis duration (years) ^a^			
2–5 years (%)	0.93	0.89–0.98	0.003
5–10 years (%)	1.00	Reference	Reference
10–15 years (%)	1.12	1.06–1.19	<0.0001
15–20 years (%)	1.37	1.26–1.48	<0.0001
20–25 years (%)	1.47	1.32–1.63	<0.0001
25–30 years (%)	1.37	1.21–1.56	<0.0001
≥ 30 years (%)	1.22	1.05–1.41	0.008
Presence of DM			
non-DM	1.00	Reference	Reference
DM	1.53	1.47–1.59	<0.0001
Comorbidity of CVD			
No comorbidity of CVD	1.00	Reference	Reference
Comorbidity of CVD	1.60	1.53–1.67	<0.0001
Hemoglobin			
1 g/dL increase	0.89	0.87–0.90	<0.0001
C-reactive protein			
1 mg/dL increase	1.10	1.09–1.11	<0.0001
Kt/V ^a^			
<0.8	2.26	1.91–2.67	<0.0001
0.8–1.0	1.50	1.37–1.65	<0.0001
1.0–1.2	1.17	1.10–1.24	<0.0001
1.2–1.4	1.00	Reference	Reference
1.4–1.6	0.98	0.93–1.03	0.442
1.6–1.8	0.91	0.86–0.97	0.005
1.8–2.0	0.86	0.78–0.94	0.001
≥ 2.0	0.79	0.70–0.89	<0.0001
Normalized protein catabolic rate (g/kg/day) ^a^			
<0.5	2.86	2.49–3.28	<0.0001
0.5–0.7	1.39	1.31–1.47	<0.0001
0.7–0.9	1.00	Reference	Reference
0.9–1.1	0.92	0.87–0.96	0.00020
1.1–1.3	0.82	0.77–0.89	<0.0001
≥ 1.3	0.95	0.82–1.11	0.546
Serum albumin (g/dL) ^a^			
< 3.0	3.86	3.62–4.12	<0.0001
3.0–3.5	1.72	1.64–1.81	<0.0001
3.5–4.0	1.00	Reference	Reference
4.0–4.5	0.86	0.81–0.91	<0.0001
≥ 4.5	1.03	0.87–1.20	0.757
Calcium (mg/dL) ^a^			
< 8.5	1.30	1.23–1.37	<0.0001
8.5–9.5	1.00	Reference	Reference
9.5–10.5	0.97	0.92–1.02	0.256
≥ 10.5	1.16	1.04–1.28	0.006
Phosphate (mg/dL) ^a^			
<3.5	1.69	1.59–1.80	<0.0001
3.5–4.5	1.14	1.08–1.21	<0.0001
4.5–5.5	1.00	Reference	Reference
5.5–6.5	0.94	0.82–1.07	0.351
6.5–7.5	1.08	0.97–1.20	0.166
7.5–8.5	1.14	1.08–1.21	<0.0001
≥8.5	1.22	1.13–1.31	<0.0001
Total cholesterol (mg/dL) ^a^			
< 80	1.61	1.33–1.95	<0.0001
80–120	1.24	1.17–1.31	<0.0001
120–160	1.00	Reference	Reference
160–200	0.91	0.86–0.95	<0.0001
200–240	0.86	0.79–0.94	0.0003
≥ 240	1.04	0.88–1.23	0.621
Body mass index (m/kg^2^) ^a^			
< 16.0	2.40	2.22–2.60	<0.0001
16–18	1.50	1.41–1.60	<0.0001
18–20	1.15	1.09–1.22	<0.0001
20–22	1.00	Reference	Reference
22–24	0.93	0.87–0.99	0.038
24–26	0.90	0.83–0.98	0.011
26–28	0.90	0.80–1.00	0.051
≥ 28	0.90	0.77–1.02	0.095
% creatinine generation rate (%) ^a^			
< 60	2.55	2.37–2.75	<0.0001
60–70	1.75	1.61–1.91	<0.0001
70–80	1.44	1.33–1.56	<0.0001
80–90	1.14	1.05–1.23	0.001
90–100	1.00	Reference	Reference
100–110	0.96	0.89–1.00	0.284
110–120	0.86	0.79–0.93	<0.0001
120–130	0.80	0.73–0.87	<0.001
≥ 130	0.79	0.73–0.87	<0.0001

^a^ Ranges are inclusive on the lower end and exclusive on the upper end.

CI, confidence interval; CVD, cardiovascular disease; DM, diabetes mellitus; HR, hazard ratio.

### Clinical and demographic characteristics according to type of dialyzer membrane material

The patients were divided into seven groups according to the dialyzer membrane material. The demographics and characteristics of each group are shown in Table 3. More than half of the patients (56.0%) underwent hemodialysis with PS membrane, followed by CTA (17.3%), PES (12.0%), PEPA (7.5%), PMMA (4.9%), PAN (1.2%), and EVAL (1.1%). The patients treated with EVAL showed the following features: older age, fewer males, shorter hemodialysis duration, higher rate of CVD comorbidity, and poor nutritional status. Conversely, the patients treated with PS and PES were associated with younger age, more males, lower rates of presence of diabetes and CVD, and higher Kt/V, nPCR, %CGR, and higher surface area of membrane.

**Table 3:** Demographic, clinical, and laboratory values in 142,412 according to type of dialyzer membrane.

	CTA	EVAL	PAN	PEPA	PES	PMMA	PS	P value
n (%)	24,572 (17.3)	1,604 (1.1)	1,703 (1.2)	10,677 (7.5)	17,060 (12.0)	6,954 (4.9)	79,842 (56.0)	
Total deaths (n)	2,167	270	144	889	951	603	5,139	
Death rate (per 100 patient-years)	8.82	16.8	8.45	8.33	5.57	8.67	6.44	<0.0001
Age (years)	67 ± 12	72 ± 11	67 ± 12	66 ± 12	63 ± 12	68 ± 12	64 ± 12	<0.0001
Sex (% female)	39.5	55.7	46.7	41.4	35.2	43	38.7	<0.0001
Dialysis duration (years)	6 [3–10]	5 [3–9]	7 [4–12]	6 [3–11]	7 [4–13]	6 [4–11]	7 [4–13]	<0.0001
Presence of DM (%)	35.7	35.5	31.4	32.3	28.6	33.2	29.2	<0.0001
Comorbidity of CVD (%)	26.7	33.8	24.7	25.6	22.4	28.8	23.9	<0.0001
Body mass index (kg/m^2^)	21.0 ± 3.6	20.0 ± 3.4	20.7 ± 3.3	20.9 ± 3.4	21.4 ± 3.5	20.6 ± 3.2	21.1 ± 3.5	<0.0001
Hemoglobin (g/dL)	10.4 ± 1.3	10.2 ± 1.3	10.2 ± 1.2	10.4 ± 1.3	10.5 ± 1.2	10.2 ± 1.2	10.5 ± 1.2	<0.0001
Serum albumin (g/dL)	3.7 ± 0.4	3.5 ± 0.5	3.7 ± 0.4	3.6 ± 0.4	3.8 ± 0.4	3.6 ± 0.4	3.7 ± 0.4	<0.0001
Total cholesterol (mg/dL)	155 ± 36	162 ± 40	153 ± 35	157 ± 37	152 ± 35	162 ± 38	151 ± 36	<0.0001
Calcium (mg/dL)	9.0 ± 0.8	8.9 ± 0.8	9.1 ± 0.9	9.0 ± 0.8	9.1 ± 0.8	8.9 ± 0.8	9.1 ± 0.8	<0.0001
Phosphate (mg/dL)	5.3 ± 1.4	5.0 ± 1.4	5.3 ± 1.5	5.3 ± 1.4	5.4 ± 1.5	5.2 ± 1.4	5.3 ± 1.4	<0.0001
β_2_-microglobulin (mg/L)	27.8 ± 7.0	29.8 ± 8.0	30.0 ± 7.0	27.1 ± 6.5	27.2 ± 6.5	29.5 ± 7.0	27.2 ± 6.5	<0.0001
C-reactive protein (mg/dL)	0.13 [0.06–0.4]	0.16 [0.06–0.59]	0.13 [0.06–0.39]	0.12 [0.05–0.38]	0.10 [0.05–0.31]	0.13 [0.05–0.40]	0.11 [0.05–0.36]	<0.0001
Surface area of dialyzer (m^2^)	1.6 ± 0.4	1.4 ± 0.3	1.4 ± 0.2	1.6 ± 0.3	1.8 ± 0.3	1.6 ± 0.3	1.7 ± 0.3	<0.0001
Characteristic of dialyzer (%)								<0.0001
Low flux	1.7	36.1	0	0	0	1.0	0	
High flux	98.3	63.9	100	100	100	99.0	100	
Kt/V	1.41 ± 0.28	1.30 ± 0.28	1.39 ± 0.29	1.42 ± 0.28	1.46 ± 0.29	1.39 ± 0.27	1.45 ± 0.29	<0.0001
nPCR (g/kg/day)	0.87 ± 0.18	0.85 ± 0.18	0.88 ± 0.18	0.88 ± 0.18	0.90 ± 0.18	0.86 ± 0.18	0.90 ± 0.18	<0.0001
%CGR (%)	97 ± 26	85 ± 30	96 ± 27	99 ± 26	102 ± 24	96 ± 26	101 ± 25	<0.0001

CTA, cellulose triacetate; CVD, cardiovascular disease; DM, diabetes mellitus; EVAL, ethylene vinyl alcohol; nPCR, normalized protein catabolic rate; PAN, polyacrylonitrile; PEPA, polyester polymer alloy; PES, polyethersulfone; PMMA, polymethylmethacrylate; PS, polysulfone, %CGR, % creatinine generation rate.

### All-cause mortality according to type of dialyzer membrane material

**Fig 2:**
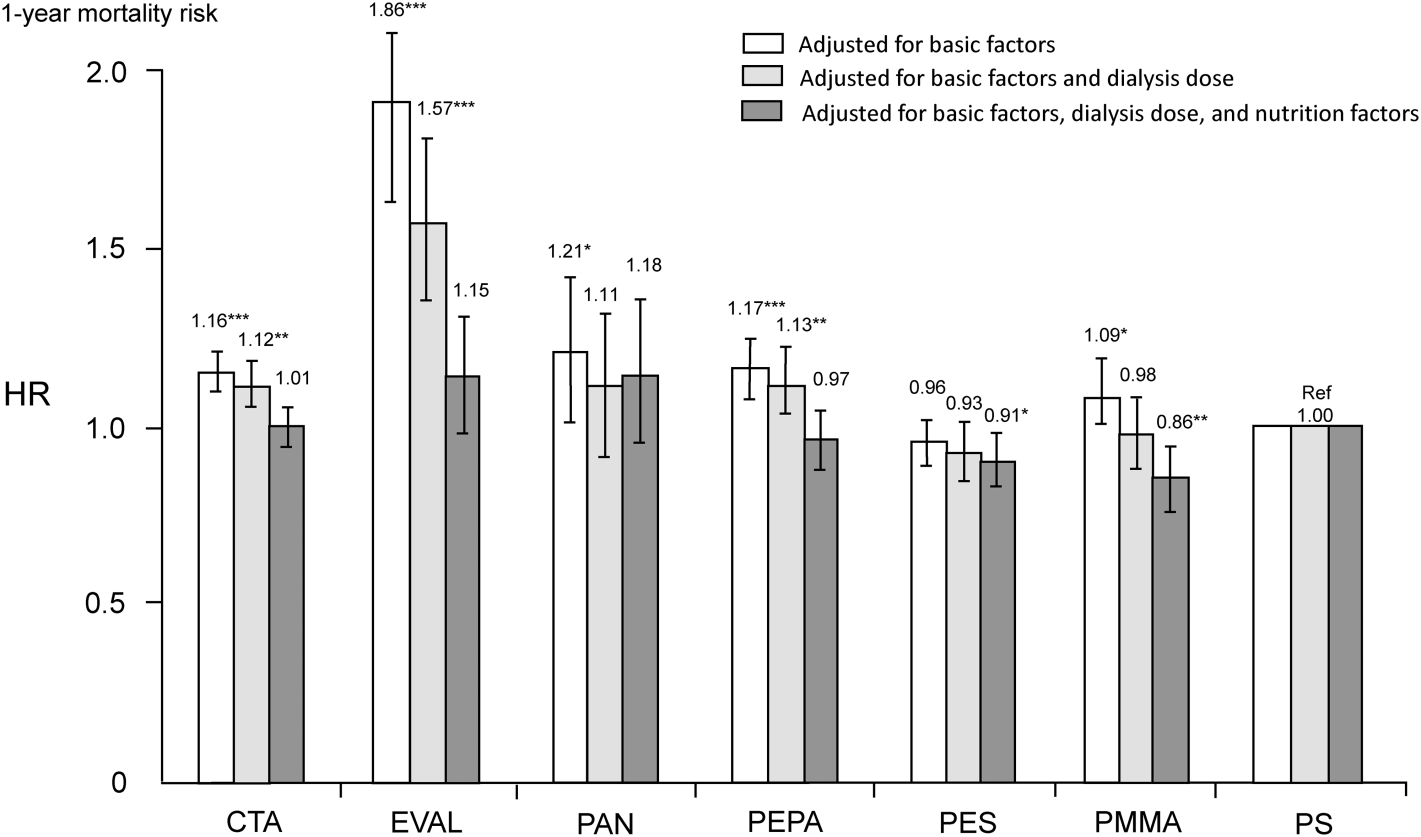
HRs of all-cause mortality between seven types of dialyzer membranes in 142,412 patients undergoing maintenance hemodialysis using standard Cox proportional hazards regression. White bars are adjusted for age, sex, dialysis duration, presence or absence of diabetes, and presence or absence of cardiovascular comorbidity. Gray bars are adjusted for basic factors and dialysis dose as indicated by single-pool Kt/V. Black bars are adjusted for basic factors, dialysis dose, and nutrition factors, including body mass index, serum levels of albumin, total cholesterol levels, normalized protein catabolic rate, and % creatinine generation rate. *P < 0.05, **P < 0.01, ***P < 0.001 vs. PS. CTA, cellulose triacetate; EVAL, ethylene vinyl alcohol; HR, hazard ratio; PAN, polyacrylonitrile; PEPA, polyester polymer alloy; PES, polyethersulfone; PMMA, polymethylmethacrylate; PS, polysulfone.

Unadjusted all-cause mortality HRs for CTA, EVAL, PAN, PEPA, and PMMA groups, as compared with the PS group (reference), were 1.39 (1.32–1.46), 2.78 (2.45–3.14), 1.32 (1.12–1.56), 1.30 (1.21–1.40), and 1.36 (1.25–1.48), respectively (S3 Table). In contrast, only the PES group had a significantly lower HR of 0.86 (0.80–0.92) compared with the PS group (reference). Fig 2 shows adjusted all-cause mortality HRs for each group. After adjusting for basic factors, five groups had higher HRs as compared with the PS group, as well as in the unadjusted model. However, there was no significant difference between the PES and PS groups. After adjusting for dialysis-related factors, such as dialysis dose and high- or low-flux membrane in addition to basic factors, the higher HRs for the CTA, EVAL, and PEPA groups persisted. However, there were no significant differences in the HRs of the PAN and PMMA groups as compared with the PS group. Finally, after adjusting for nutrition and inflammation factors in addition to basic factors and dialysis-related factors, there were no significant differences in the HRs of the CTA, EVAL, PAN, and PEPA groups compared with the PS group. However, HRs for the PES and PMMA groups were significantly lower than that for the PS group.

### Propensity score matching analysis for all-cause mortality according to type of dialyzer membrane material

**Fig 3:**
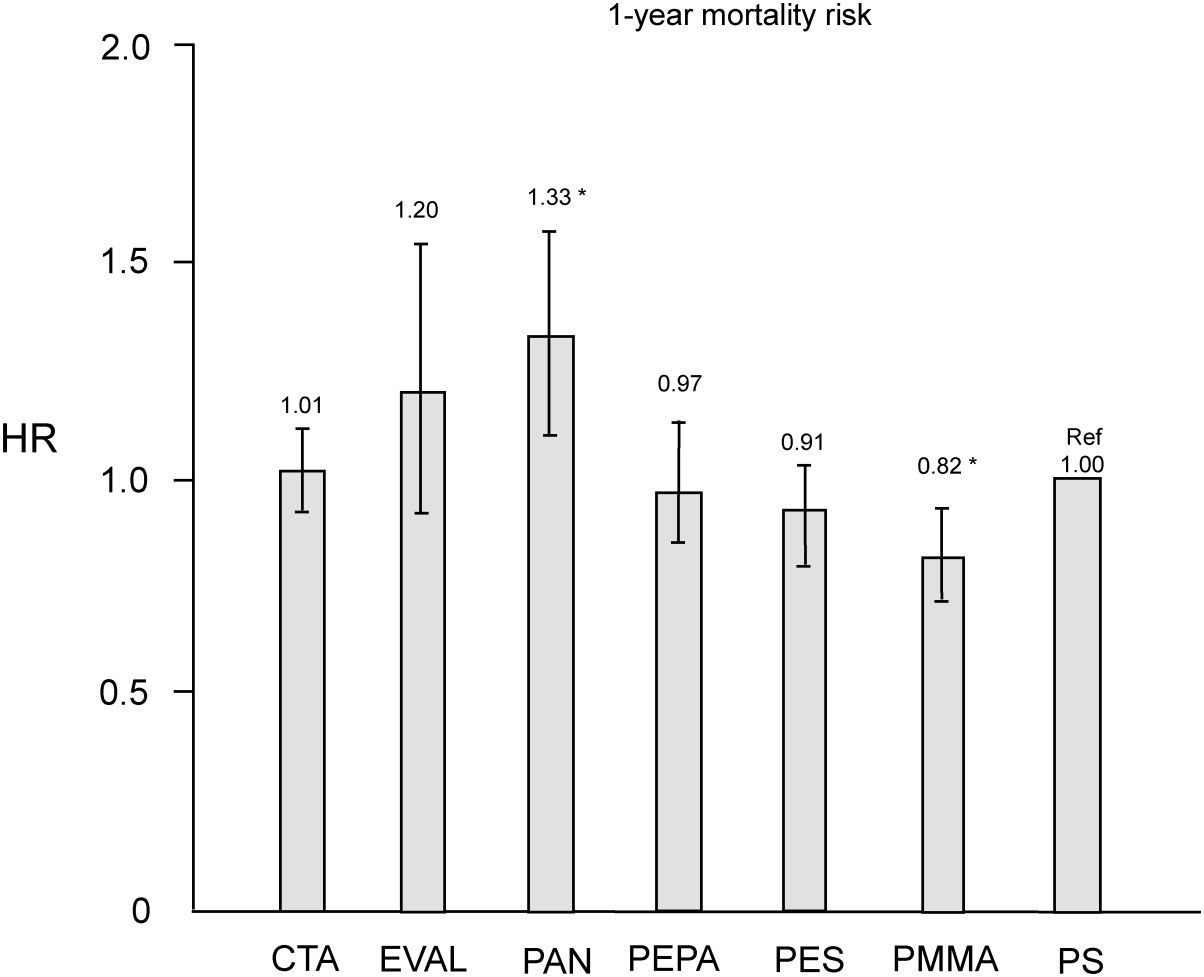
HRs of all-cause mortality after propensity score matching for six types of dialyzer groups compared to the PS group using Cox proportional hazards regression. *P < 0.01 vs. PS. CTA, cellulose triacetate; EVAL, ethylene vinyl alcohol; HR, hazard ratio; PAN, polyacrylonitrile; PEPA, polyester polymer alloy; PES, polyethersulfone; PMMA, polymethylmethacrylate; PS, polysulfone.

Table 4 shows patient characteristics and baseline data in the PS and each corresponding group after propensity score matching. No significant differences were noted in all variables. As shown in Fig 3, HRs of CTA, EVAL, PEPA, and PES groups did not differ significantly compared with the PS group. The HR for the PMMA group (0.82 [0.71–0.94]) was significantly lower than that for the PS group (P < 0.01).

**Table 4:** Baseline characteristics after propensity score matching between PS and other groups.

	CTA	PS	P value	EVAL	PS	P value	PAN	PS	P value	PEPA	PS	P value	PES	PS	P value	PMMA	PS	P value
n (%)	10,592	10,592		571	571		764	764		4,683	4,683		7,480	7,480		3,835	3,835	
Age (years)	67 ± 12	67 ± 12	0.539	70 ± 11	70 ± 11	0.689	66 ± 12	66 ± 12	0.283	66 ± 12	66 ± 12	0.283	62 ± 12	62 ± 12	0.835	68 ± 11	68 ± 11	0.081
Sex (% Female)	38.0	37.4	0.356	53.4	52	0.635	40.9	41.6	0.426	40.9	41.6	0.426	35.8	35.3	0.528	42.6	42.9	0.764
HD duration (years)	6 [3–10]	6 [3–10]	0.364	5 [3–10]	5 [3–9]	0.426	6 [4–11]	6 [4–11]	0.978	6 [4–11]	6 [4–11]	0.978	7 [4–12]	7 [4–12]	0.531	6 [3–11]	6 [4–11]	0.456
Presence of DM	37.2	36.5	0.339	36.4	36.6	0.859	32.4	32.2	0.859	32.4	32.2	0.859	29.0	28.6	0.563	33.0	33.0	1.00
Comorbidity of CVD	27.6	27.1	0.405	30.6	31.0	0.898	24.1	23.6	0.577	24.1	23.6	0.577	22.8	23.3	0.393	29.7	29.7	0.942
BMI (kg/m^2^)	21.1 ± 3.6	21.1 ± 3.5	0.355	20.2 ± 3.4	20.1 ± 3.5	0.682	20.8 ± 3.3	20.8 ± 3.3	0.487	20.8 ± 3.3	20.8 ± 3.3	0.487	21.4 ± 3.5	21.4 ± 3.6	0.442	20.5 ± 3.2	20.6 ± 3.3	0.859
Hb (g/dL)	10.5 ± 1.2	10.5 ± 1.2	0.469	10.3 ± 1.2	10.3 ± 1.6	0.946	10.4 ± 1.3	10.4 ± 1.2	0.919	10.4 ± 1.3	10.4 ± 1.2	0.919	10.5 ± 1.2	10.5 ± 1.2	0.712	10.2 ± 1.2	10.3 ± 1.2	0.142
Alb (g/dL)	3.6 ± 0.4	3.6 ± 0.5	0.072	3.5 ± 0.4	3.5 ± 0.5	0.885	3.6 ± 0.4	3.6 ± 0.4	0.247	3.6 ± 0.4	3.6 ± 0.4	0.247	3.8 ± 0.4	3.8 ± 0.4	0.502	3.6 ± 0.4	3.6 ± 0.4	0.691
TC (mg/dL)	155 ± 35	155 ± 36	0.230	158 ± 39	159 ± 39	0.845	157 ± 36	157 ± 36	0.485	157 ± 36	157 ± 36	0.485	153 ± 33	153 ± 34	0.367	162 ± 37	163 ± 38	0.183
Ca (mg/dL)	9.0 ± 0.8	9.0 ± 0.8	0.068	8.9 ± 0.8	8.9 ± 0.8	0.517	9.0 ± 0.8	9.0 ± 0.8	0.517	9.0 ± 0.8	9.0 ± 0.8	0.517	9.1 ± 0.8	9.1 ± 0.8	0.528	8.9 ± 0.8	8.9 ± 0.8	0.604
P (mg/dL)	5.3 ± 1.4	5.3 ± 1.4	0.053	5.0 ± 1.4	5.0 ± 1.4	0.864	5.3 ± 1.4	5.3 ± 1.4	0.641	5.3 ± 1.4	5.3 ± 1.4	0.641	5.4 ± 1.5	5.4 ± 1.5	0.801	5.2 ± 1.4	5.2 ± 1.4	0.562
β2M (mg/L)	27.8 ± 7.0	27.9 ± 6.8	0.414	29.6 ± 7.7	29.4 ± 8.4	0.731	27.2 ± 6.5	27.0 ± 6.3	0.197	27.2 ± 6.5	27.0 ± 6.3	0.197	27.2 ± 6.4	27.2 ± 6.2	0.961	29.3 ± 6.9	29.4 ± 7.2	0.574
CRP (mg/dL)	0.13 [0.05–0.40]	0.12 [0.06–0.38]	0.530	0.13 [0.05–0.43]	0.16 [0.06–0.47]	0.583	0.11 [0.05–0.35]	0.11 [0.05–0.32]	0.583	0.11 [0.05–0.35]	0.11 [0.05–0.32]	0.583	0.11 [0.05–0.30]	0.11 [0.05–0.34]	0.365	0.12 [0.06–0.40]	0.14 [0.06–0.41]	0.691
Kt/V	1.43 ± 0.27	1.43 ± 0.29	0.191	1.34 ± 0.28	1.33 ± 0.27	0.501	1.44 ± 0.27	1.45 ± 0.29	0.501	1.44 ± 0.27	1.45 ± 0.29	0.501	1.47 ± 0.29	1.47 ± 0.29	0.946	1.40 ± 0.27	1.40 ± 0.27	0.832
nPCR (g/kg/day)	0.87 ± 0.17	0.87 ± 0.17	0.858	0.84 ± 0.17	0.83 ± 0.17	0.649	0.88 ± 0.17	0.88 ± 0.17	0.672	0.88 ± 0.17	0.88 ± 0.17	0.672	0.90 ± 0.18	0.90 ± 0.18	0.512	0.86 ± 0.17	0.85 ± 0.18	0.125
%CGR (%)	98 ± 26	98 ± 26	0.663	87 ± 27	87 ± 28	0.975	100 ± 26	100 ± 29	0.975	100 ± 26	100 ± 29	0.975	103 ± 24	103 ± 27	0.947	96 ± 26	96 ± 26	0.621

Alb, serum albumin; β2M, β_2_-microglobulin; BMI, body mass index; Ca, calcium; CRP, C-reactive protein; CTA, cellulose triacetate; CVD, cardiovascular disease; DM, diabetes mellitus; EVAL, ethylene vinyl alcohol; nPCR, normalized protein catabolic rate; P, phosphate; PAN, polyacrylonitrile; PEPA, polyester polymer alloy; PES, polyethersulfone; PMMA, polymethylmethacrylate; PS, polysulfone, %CGR, % creatinine generation rate.

## Discussion

In this study, we first confirmed the predictors for 1-year mortality in hemodialysis patients. As reported previously, basic factors such as male sex, dialysis duration, increasing age, dialysis duration, and presence of diabetes and comorbidity of CVD were included as predictors [16,17]. In addition, dialysis-related factors and nutritional- and inflammation-factors were also included as predictors. Second, we compared the mortality rate between seven types of dialyzers membrane materials adjusted for these predicting factors. After fully adjusting for these factors and propensity score matching, the HR for the PMMA membrane group was significantly lower than that for the PS membrane group used as a reference. This is the first study to suggest that the mortality risk for hemodialysis patients might differ by the type of dialyzer membrane material used.

Several cross-sectional and cohort studies have shown that efficiency of removal of small uremic toxins during each dialysis session, particularly the normalized urea clearance (Kt/V), is a useful prognostic factor in dialysis patients. These studies evaluated the role of targeting Kt/V in reducing the mortality rate [18–20]. Furthermore, Kt/V was recently identified as an independent prognostic factor following an analysis by the JRDR [1]. In these patients, nutritional status is an important prognostic factor and has shown potential as a prognostic index in medium- to long-term dialysis. Another independent prognostic factor is the estimated muscle mass from %CGR [13]. In dialysis patients, malnutrition is a complex disorder complicated by uremic toxin accumulation, weak inflammatory response due to poor membrane biocompatibility, anorexia, and loss of glucose, amino acids, and other essential nutrients during dialysis [21]. Thus, thorough assessment of treatment effects is requisite. This can be achieved by monitoring medium- to long-term indices, including maintenance levels of uremic toxins (predialysis serum β2M concentration) and nutritional status, including nPCR, serum albumin, BMI, and %CGR; these are well established indices of dialysis dose and prognosis. Although higher total cholesterol was associated with increased mortality in hemodialysis patients without a history of coronary artery disease, lower total cholesterol was associated with increased mortality in patients both with and without a history of coronary artery disease [22]. Furthermore, the presence of protein energy wasting and/or inflammation may explain the paradoxical association between total cholesterol and mortality risk in the dialysis population, because total cholesterol levels are decreased in such conditions [23]. Although lower total cholesterol levels were associated with higher mortality in the present study, care should be taken regarding the interpretation of this result since we could not collect data on the use of statins.

To minimize the inflammatory responses, it is important to select highly biocompatible membranes and purified dialysate. Several guidelines also discourage the use of dialysis membranes that promote rapid activation of the complement system, leukocytosis, and inflammatory response [3,4]. MacLeod et al. conducted a meta-analysis, which failed to establish the superiority of synthetic polymer membranes [24]. However, comparison between high-flux and low-flux membranes in a randomized controlled trial reported better prognosis with the use of high-flux membranes for hemodialysis patients with diabetic nephropathy and patients with serum albumin <4.0 g/dL [25]. Furthermore, the mortality rate is lower for patients with predialysis serum β2M concentrations of 27.5–34 mg/L, as reported by the HEMO study and Okuno et al., suggesting that the decrease in concentrations of uremic substances, including β2M, is important in dialysis therapy [26–28]. The JRDR report states that predialysis serum β2M concentration was <30 mg/L in about 71% of patients [1].

In Japan, the HPM concept became popular around the early 1980s. Membranes with a high ultrafiltration rate, including high-flux membranes and high-permeability membranes, were considered high-efficiency membranes in the past. However, low-molecular-weight proteins that are smaller than albumin have been focused on as uremic toxins, and β2M was identified as the amyloid precursor protein in dialysis-related amyloidosis (DRA) in 1985 [29]. Therefore, dialyzers that function to eliminate solutes by adsorption and those with high biocompatibility that reduce inflammatory response are also considered to be HPM dialyzers. HPM dialyzers have much higher biocompatibility and eliminate solutes that cannot otherwise be eliminated by conventional dialyzers, thus, they are predicted to have favorable long-term clinical outcomes for hemodialysis patients. These include improved prognosis, and ameliorating conditions like malnutrition, DRA-associated syndromes, renal anemia, pruritus, and restless leg syndrome [30–34]. Several dialyzers currently in use can efficiently eliminate low-molecular-weight proteins; in Japan, the standard definition for HPM dialyzers is those membranes providing β2M clearance of at least 10 mL/min in actual clinical use [1,5]. The present study showed the PMMA membrane might be superior to other HPMs in terms of mortality, because 99.3% of the patients were treated with HPMs in the present study. PMMA is a symmetric membrane, in which the whole thickness is involved in the separation process, allowing high-efficiency adsorption of both middle- and high-molecular-weight molecules such as β2M and free light chains, and inflammatory cytokines [35–37]. Masakane reported that the body weights of patients for whom the dialyzer membrane was changed from PMMA to PS tended to decrease, while a reverse tendency was observed when the membrane was changed from PS to PMMA [38]. Similarly, Kreusser et al. reported that the cumulative 5-year survival rate for dialysis patients treated with the PMMA membrane is higher than that of patients treated with the PS membrane (68% vs. 54%) [39]. However, further long-term prospective large-scale studies are required to confirm these findings since the abovementioned studies had small sample sizes.

This study has several limitations. First, because of the nature of any annual survey and observational cohort study, the numbers of the patients differed between the seven groups. Therefore, mortality may vary between centers due to differences in center practices and patient populations. Second, we confirmed that all patients used the same membranes for 1 year after inclusion, but by the end of the study we did not have information about the types of dialyzers used. To investigate outcomes associated with the use of different dialyzers, further long-term studies with matched baseline characteristics of the patients are needed. However, we consider that the present study revealed and matched the actual clinical setting for hemodialysis treatment in Japan. Finally, we had no information about residual renal function, which could be a possible confounder. However, since the reported loss of renal function after starting dialysis was about 2.0 mL/min/year [40] and the mean estimated glomerular filtration rate at dialysis initiation was 6.52 mL/min/1.73 m^2^ in 2007 throughout Japan [41], the impact of residual renal function may be negligible because the median dialysis duration was 7 years in our cohort.

The results of this study suggest that the use of different membrane material types may have an impact on mortality in hemodialysis patients. However, further long-term prospective studies are needed to clarify these findings, including whether the PMMA membrane can improve prognosis.

## Supporting information

S1 Table Class according to the Japanese reimbursement system. (DOCX)

S2 Table Characteristics of the high-performance membrane dialyzers available in the present study. All data were provided by the manufacturer. ^a^ Plasma was used as a pseudo blood in the experiment. ^b^ Clearances were measured under the following conditions: blood flow rate = 200 mL/min, dialysis fluid flow rate = 500 mL/min, and ultrafiltration flow rate per unit surface area = 10 mL/min/m^2^. CTA, cellulose triacetate; EVAL, ethylene vinyl alcohol; PAN, polyacrylonitrile; PEPA, polyester polymer alloy; PES, polyethersulfone; PMMA, polymethylmethacrylate; PS, polysulfone.
(DOCX)

S3 Table HRs (95% CIs) of all-cause mortality between seven types of dialyzers in 142,412 maintenance hemodialysis patients using standard Cox proportional hazards regression. ^a^Adjusted for age, sex, dialysis duration, presence or absence of diabetes and cardiovascular disease. ^b^Adjusted for basic factors and Kt/V. ^c^Adjusted for basic factors, Kt/V, normalized protein catabolic rate, serum albumin, total cholesterol, body mass index, and % creatinine generation rate. CI, confidence interval; CTA, cellulose triacetate; EVAL, ethylene vinyl alcohol; HR, hazard ratio; PAN, polyacrylonitrile; PEPA, polyester polymer alloy; PES, polyethersulfone; PMMA, polymethylmethacrylate; PS, polysulfone.
(DOCX)

S1 File Data set of all patients who were included in the final cohort. Alb, serum albumin; β2M, β_2_-microglobulin; BMI, body mass index; Ca, serum calcium; CRP, C-reactive protein; CTA, cellulose triacetate; EVAL, ethylene vinyl alcohol; Hb, hemoglobin; HD, hemodialysis; nPCR, normalized protein catabolic rate; P, serum phosphate; PAN, polyacrylonitrile; PEPA, polyester polymer alloy; PES, polyethersulfone; PMMA, polymethylmethacrylate; PS, polysulfone; %CGR, % creatinine generation rate; TC, total cholesterol.
(ZIP)
