# Supplementary material for: Effect of dialyzer membrane materials on survival in chronic hemodialysis patients: Results from the annual survey of the Japanese Nationwide Dialysis Registry
Source: PLoS One. 2017 Sep 14;12(9):e0184424. doi: 10.1371/journal.pone.0184424 (PMC5598977; doi:10.1371/journal.pone.0184424)
Supplement: S1 Table — (DOCX) [file pone.0184424.s001.docx]

**Supporting information**

**S1 Table. Class according to the Japanese reimbursement system**

| Class | β_2_-MG clearance (mL/min) |
| --- | --- |
| I | < 10 |
| II | ≥ 10 and < 30 |
| III | ≥ 30 and < 50 |
| IV | ≥ 50 and < 70 |
| V | ≥ 70 |
