# Supplementary material for: Effect of dialyzer membrane materials on survival in chronic hemodialysis patients: Results from the annual survey of the Japanese Nationwide Dialysis Registry
Source: PLoS One. 2017 Sep 14;12(9):e0184424. doi: 10.1371/journal.pone.0184424 (PMC5598977; doi:10.1371/journal.pone.0184424)
Supplement: S2 Table — All data were provided by the manufacturer. a Plasma was used as a pseudo blood in the experiment. b Clearances were measured under the following conditions: blood flow rate = 200 mL/min, dialysis fluid flow rate = 500 mL/min, and ultrafiltration flow rate per unit surface area = 10 mL/min/m2. CTA, cellulose triacetate; EVAL, ethylene vinyl alcohol; PAN, polyacrylonitrile; PEPA, polyester polymer alloy; PES, polyethersulfone; PMMA, polymethylmethacrylate; PS, polysulfone. (DOCX) [file pone.0184424.s002.docx]

**Supporting information**

**S2 Table. Characteristics of the high-performance membrane dialyzers available in the present study**

| Surface | Company | Japanese brand name | Surface area (m^2^) | UFR ^a^ (mL/mmHg/hr) | Clearance (mL/min) ^a,b^ | |  |  | Sieving coefficient | | Class according to the Japanese reimbursement system |
| --- | --- | --- | --- | --- | --- | --- | --- | --- | --- | --- | --- |
|  |  |  |  |  | UN | Cr | β_2_-MG |  | β_2_-MG | Albumin |  |
| CTA | Nipro Corp. | FB-EG | 1.5 | 12.8 | 191 | 180 | 8 |  | 0.33 | NA | I |
|  |  | FB-E, FB-G | 1.5 | 31.2 | 197 | 192 | 20 |  | 0.62 | NA | II |
|  |  | FB-Pβ | 1.5 | 58.4 | 198 | 192 | 53 |  | 0.80 | NA | III |
|  |  | FB-Uβ | 1.5 | 60.1 | 198 | 192 | 65 |  | 0.99 | 0.01 | IV |
|  |  |  |  |  |  |  |  |  |  |  |  |
| EVAL | Kawasumi Laboratories, Inc. | KF | 1.5 | 8.7 | 179 | 157 | 17.5 |  | NA | 0.02 | I |
|  |  | kf-m | 1.5 | 11.5 | 175 | 155 | 16.6 |  | 0.67 | 0.03 | II |
|  |  | EK | 1.6 | 37.7 | 185 | 176 | 44.6 |  | NA | <0.01 | III |
|  |  |  |  |  |  |  |  |  |  |  |  |
| PAN | Baxter | H12-4000 | 1.53 | 80.0 | 182 | 189 |  |  | NA | 0.001 |  |
|  |  |  |  |  |  |  |  |  |  |  |  |
| PEPA | Nikkiso Co., Ltd. | FLX, FDY | 1.5 | 50.0 | 190 | 183 | 65 |  | NA | NA | IV |
|  |  | FDY-GW | 2.1 | 63.0 | 194 | 189 | 71 |  | NA | NA | V |
|  |  |  |  |  |  |  |  |  |  |  |  |
| PES | JMS　Co., Ltd. | BP-N | 1.5 | 43.0 | 190 | 178 | 75 |  | NA | 0.01 | V |
|  | Nipro Corp. | PES-DS | 1.5 | 34.7 | 190 | 181 | 48 |  | 0.98 | 0.01 | III |
|  |  | PES-Eα | 1.5 | 57.9 | 194 | 192 | 65 |  | 0.99 | <0.01 | IV |
|  |  | PES-Dα | 1.5 | 86.9 | 199 | 198 | 77 |  | 0.99 | <0.01 | V |
|  |  |  |  |  |  |  |  |  |  |  |  |
| PMMA | Toray Industries, Inc. | BK-U | 1.6 | 31.0 | 187 | 169 | 51 |  | NA | 0.03 | III |
|  |  | BG-U, BG-PQ | 1.6 | 31.0 | 190 | 172 | 55 |  | NA | 0.009 | IV |
|  |  |  |  |  |  |  |  |  |  |  |  |
| PS | Fresenius Medical Care Japan K.K. | FX | 1.4 | 49.0 | 197.5 | 182.1 | 48.7 |  | 0.8 | NA | IV |
|  |  | FX-S | 1.4 | 50.0 | 195.6 | 182.4 | 68.1 |  | 0.945 | NA | V |
|  | Kawasumi Laboratories, Inc. | PS-H | 1.6 | 68.0 | 187 | 177 | 62.6 |  | NA | NA | IV |
|  |  | PS-UW | 1.6 | 66.0 | 192 | 179 | 56.2 |  | 0.83 | < 0.01 | IV |
|  |  | PS-MW | 1.6 | 58.0 | 191 | 178 | 48.4 |  | NA | < 0.01 | III |
|  | Asahi Kasei-Kuraray Medical Co. Ltd | APS-UA | 1.5 | 42.0 | 196 | 191 | 48 |  | 0.7 | <0.002 | III |
|  |  | APS-SA | 1.5 | 63.0 | 196 | 191 | 68 |  | 0.85 | 0.002 | IV |
|  |  | APS-E | 1.5 | 66.0 | 190 | 181 | 74 |  | 0.87 | 0.008 | V |
|  |  | VPS | 1.5 | 63.0 | 196 | 190 | 62 |  | 0.82 | 0.002 | IV |
|  | Toray Industries, Inc. | TS | 1.6 | 54.0 | 194 | 183 | 59 |  | NA | 0.006 | IV |
|  |  | TS-PLX | 1.6 | 50.0 | 196 | 187 | 76 |  | NA | 0.011 | V |

All data were provided by the manufacturer.

^a^ Plasma was used as a pseudo blood in the experiment.

^b^ Clearances were measured under the following conditions: blood flow rate = 200 mL/min, dialysis fluid flow rate = 500 mL/min, and ultrafiltration flow rate per unit surface area = 10 mL/min/m^2^.

CTA, cellulose triacetate; EVAL, ethylene vinyl alcohol; PAN, polyacrylonitrile; PEPA, polyester polymer alloy; PES, polyethersulfone; PMMA, polymethylmethacrylate; PS, polysulfone.
