# Supplementary material for: Effect of dialyzer membrane materials on survival in chronic hemodialysis patients: Results from the annual survey of the Japanese Nationwide Dialysis Registry
Source: PLoS One. 2017 Sep 14;12(9):e0184424. doi: 10.1371/journal.pone.0184424 (PMC5598977; doi:10.1371/journal.pone.0184424)
Supplement: S3 Table — aAdjusted for age, sex, dialysis duration, presence or absence of diabetes and cardiovascular disease. bAdjusted for basic factors and Kt/V. cAdjusted for basic factors, Kt/V, normalized protein catabolic rate, serum albumin, total cholesterol, body mass index, and % creatinine generation rate. CI, confidence interval; CTA, cellulose triacetate; EVAL, ethylene vinyl alcohol; HR, hazard ratio; PAN, polyacrylonitrile; PEPA, polyester polymer alloy; PES, polyethersulfone; PMMA, polymethylmethacrylate; PS, polysulfone. (DOCX) [file pone.0184424.s003.docx]

**Supporting information**

**S3 Table. HRs (95% CIs) of all-cause mortality between seven types of dialyzers in 142,412 maintenance hemodialysis patients using standard Cox proportional hazards regression.**

| Dialyzer | Unadjusted | | |  | Adjusted for basic factors ^a^ | | |  | Adjusted for basic factors and dialysis dose ^b^ | | |  | Adjusted for basic factors, dialysis dose, and nutrition factors ^c^ | | |
| --- | --- | --- | --- | --- | --- | --- | --- | --- | --- | --- | --- | --- | --- | --- | --- |
|  | HR | 95% CI | P value |  | HR | 95% CI | P value |  | HR | 95% CI | P value |  | HR | 95% CI | P value |
| CTA | 1.39 | 1.32-1.46 | <0.0001 |  | 1.16 | 1.10-1.22 | <0.0001 |  | 1.12 | 1.06-1.19 | 0.0002 |  | 1.01 | 0.95-1.07 | 0.834 |
| EVAL | 2.78 | 2.45-3.14 | <0.0001 |  | 1.86 | 1.63-2.13 | <0.0001 |  | 1.57 | 1.35-1.82 | <0.0001 |  | 1.15 | 0.98-1.33 | 0.083 |
| PAN | 1.32 | 1.12-1.56 | 0.0013 |  | 1.21 | 1.01-1.43 | 0.029 |  | 1.11 | 0.91-1.33 | 0.309 |  | 1.18 | 0.96-1.46 | 0.122 |
| PEPA | 1.30 | 1.21-1.40 | <0.0001 |  | 1.17 | 1.08-1.26 | <0.0001 |  | 1.13 | 1.04-1.23 | 0.004 |  | 0.97 | 0.89-1.05 | 0.516 |
| PES | 0.86 | 0.80-0.92 | <0.0001 |  | 0.96 | 0.89-1.03 | 0.246 |  | 0.93 | 0.86-1.01 | 0.076 |  | 0.91 | 0.83-0.99 | 0.032 |
| PMMA | 1.36 | 1.25-1.48 | <0.0001 |  | 1.09 | 1.01-1.20 | 0.041 |  | 0.98 | 0.89-1.09 | 0.396 |  | 0.86 | 0.76-0.94 | 0.002 |
| PS | 1.00 | Reference | Reference |  | 1.00 | Reference | Reference |  | 1.00 | Reference | Reference |  | 1.00 | Reference | Reference |

^a^Adjusted for age, sex, dialysis duration, presence or absence of diabetes and cardiovascular disease.

^b^Adjusted for basic factors and Kt/V.

^c^Adjusted for basic factors, Kt/V, normalized protein catabolic rate, serum albumin, total cholesterol, body mass index, and % creatinine generation rate.

CI, confidence interval; CTA, cellulose triacetate; EVAL, ethylene vinyl alcohol; HR, hazard ratio; PAN, polyacrylonitrile; PEPA, polyester polymer alloy; PES, polyethersulfone; PMMA, polymethylmethacrylate; PS, polysulfone.
