# Supplementary material for: Effect of dialyzer membrane materials on survival in chronic hemodialysis patients: Results from the annual survey of the Japanese Nationwide Dialysis Registry
Source: PLoS One. 2017 Sep 14;12(9):e0184424. doi: 10.1371/journal.pone.0184424 (PMC5598977; doi:10.1371/journal.pone.0184424)
Supplement: S1 File — Alb, serum albumin; β2M, β2-microglobulin; BMI, body mass index; Ca, serum calcium; CRP, C-reactive protein; CTA, cellulose triacetate; EVAL, ethylene vinyl alcohol; Hb, hemoglobin; HD, hemodialysis; nPCR, normalized protein catabolic rate; P, serum phosphate; PAN, polyacrylonitrile; PEPA, polyester polymer alloy; PES, polyethersulfone; PMMA, polymethylmethacrylate; PS, polysulfone; %CGR, % creatinine generation rate; TC, total cholesterol. (ZIP) [file Dataset.pdf › Dataset.pdf]

Dialyzer	Age	Sex	HD duration	Died=0	BMI	Alb	TC	KT/V	nPCR	%CGR	Surface	high or low	Ca	P	CRP	β2M	Hb
PS	60	M	36	1	17.7	3.8	138	1.4	0.87	91.57	1.5	high	7.9	4.6	0.06	39	9.6
PS	53	M	28	1	19.9	3.2	145	1.12	0.65	52.68	1.5	high	8.1	6.1	0.06	24	11
PS	64	F	25	1	14.6	3.5	128	1.18	0.85	100.4	1.6	high	9.5	5.5	0.19	27	12.9
PS	72	M	23	1	18	3.8	173	1.35	1.14	99.82	1.4	high	11.4	6.5	0.1		11.6
PS	47	M	23	1	24.6	4	155	1.66	1.1	106.5	1.6	high	10.5	5.9	0.11	30	10.2
PS	58	M	22	1	17.7	3.6	196	1.6	1.28	92.08	1.4	high	8.1	3.1	0.04		8.3
PS	36	M	20	1	23.4	3.8	124	1.47	1.14	102.5	2.2	high	9.5	3.7	0.13	38	11
PS	68	F	20	1	20.5	3.6	135	1.41	0.82	118	2.2	high	9.2	4.4	0.1	28	9
PS	59	F	17	1	22	3.3	193	1.49	1.06	93.57	1	high	9	6	0.05	30	12.1
PS	56	M	17	1	20.6	3.6	224	1.76	1.14	126.9	1.8	high	8.3	3.6	3.06	32	9.4
PS	77	F	16	1	21.6	3.2	124	1.09	0.69	120.6	1.3	high	9	3.9	0.42	39	11.5
PS	52	F	16	1	20.2	3.3	191	1.98	0.81	106.7	2.1	high	9.1	5.2		25	10.4
PS	76	M	16	1	20.1	3.4	158	1.18	0.99	131.4	1.5	high	9.9	7.5		32	9.6
PS	69	M	14	1	18.7	3.4	151	1.63	1.25	122.1	2.2	high	9.5	5	0.12	24	10.9
PS	63	M	14	1	17.1	3.6	157	1.53	1.11	98.37	1	high	8.9	4.7	0.05	21	12.5
PS	79	F	13	1	17.7	3.3	157	1.18	0.86	97.6	1.8	high	11.7	6.4	0.3	28	9.5
PS	60	F	12	1	14.2	3.7	124	1.17	0.93	132.3	1	high	8.4	3.9	0.05	28	11.5
PS	59	M	12	1	23.8	3.8		1.63	1.05	120.3	2.1	high	8.1	5.4	0.05	25	10.4
PS	64	M	9	1	20.6	3.3	201	1.47	0.98	90.43	1.8	high	10.4	4.7		24	9.9
PS	76	F	9	1	24.2	3.8	166	1.45	1.18	107.8	1.5	high		5.4	0.3	25	9.3
PS	46	F	9	1	18	3.1	149	1.02	0.99	122.2	1.3	high	8.3	3.8	0.13	13	14.1
PS	39	M	8	1	20.1	4	140	1.85	0.96	108.1	1.8	high	9.5	5.6	0.1	28	9.7
PS	63	F	7	1	17.1	3.8		1.3	0.92	71.78	2.1	high	9.6	4.9	1	22	14
PS	60	F	6	1	26.8	3.5	129	1.48	0.93	111.2	1.5	high	9.5	5.3	0.32	32	11.2
PS	64	M	6	1	20.8	3.5		2.01	0.8	101.5	2.1	high	8.8	4.7	0.02	28	10.5
PS	59	M	6	1	26	3.3	141	1.63	0.69	106	1.8	high	9.6	4.9	0.07	23	11.4
PS	58	F	5	1	16.2	3.4	119	1.22	1.22	62.08	1.3	high	9.6	0.9		28	10.9
PS	27	M	5	1	29.3	3.5	186	2.11	1.15	127.3	2.1	high	9.6	4.6	0.1	29	10.1
PS	60	M	5	1	16.6	3.5	145	1.12	1.06	89.22	2.1	high	9.4	3.9	0		10
PS	62	F	4	1	24.8	3.5	206	1.29	0.93	119.6	2.1	high	8.6	4.5	0.02	17	9.9
PS	68	F	3	1	25	3.2	183	1.61	0.94	109.3	1.4	high	9.2	6	3.9		9.5
PS	56	M	3	1	22.9	3.8		1.67	0.68	70.92	2.1	high	8.6	4.5	0.2	25	10.1
PS	57	M	3	1	22.5	3.7	171	0.7	0.86	51.17	1.6	high	8.7	4.7	0.07	44	10.2
PS	61	M	3	1	17.2	3.1	180	1.46	0.64	68.61	1.6	high	9.9	5.2	0.07	27	11.1
PS	72	M	2	1	17.6	3.6	178	1.43	1.14	88.59	1.3	high	8.2	2.2	0.2	26	10.3
PS	35	M	2	1	19.6	4.2	137	1.41	0.8	115.8	1	high	8.7	6.2	4.21	35	14.6
PS	60	F	32	1	14.5	3.8	205	1.47	1.29	105	2.1	high		3.8		27	11.3
PS	68	F	24	1	23.2	3.6	211	1.72	0.9	86.03	1.8	high	9.1	6		24	11.6
PS	55	F	22	1	17.6	4		1.44	1.13	99.33	2.1	high	11	5.4	0.5	26	9.6
PS	63	M	20	1	20.2	6.1	161	1.45	0.91	115.6	2.5	high	10.7	4.5	0.02	27	11.6
PS	63	M	13	1	18.4	3.8	118	1.63	0.91	113.9	1.8	high	9.4	3.9	0.04	25	10.6
PS	70	M	12	1	20.2	4.1	237	1.5	1.08	98.71	2.1	high	8	4.5	0.14	19	8.8
PS	76	M	9	1	22.7	3.6	126	1.61	1.11	145.4	1.5	high	9.1	4.5		28	10.3
PS	54	M	8	1	21.3	3.9	140	1.57	1.18	113.6	1.3	high	9.3	3.9	0.26	31	11.5
PS	66	F	8	1	25.8	3.6	135	1.85	1.07	159.3	1.5	high	9.8	7		31	11.1
PS	66	M	8	1	21.6	4.2	179	1.39	1.06	131	1.8	high	7.1	5.3		25	9.4
PS	48	M	8	1	27.2	4	137	1.31	1.2	156.4	2.1	high	10.4	5.8	0.06	25	10.5
PS	58	F	7	1	22.6	4.2	124	1.6	1.18	88.95	2.1	high	8.7	3.9	0.06	24	9.7
PS	49	M	7	1	19.9	4.2	141	1.5	0.86	108.6	2.1	high	9.7	5.2	0.01	32	9.8
PS	61	M	7	1	21.8	3.9	148	1.54	0.96	116.3	1.8	high	8	4.9	0.02	27	8.8
PS	71	F	7	1	22	3.6	218	1.42	0.99	148.2	1.8	high	8.6	5.9	0.03	27	11.3
PS	34	F	7	1	19.5	4.2	90	1.16	0.82	111.8	1.5	high	6.9	5.5	0.19	28	11.5
PS	60	F	7	1	28.8	3.9	151	2.06	0.8	141.5	1.8	high	10.4	6.1			11.6
PS	50	F	7	1	19.6	3.9	179	1.64	0.67	116.5	1.8	high	9.3	4.2	0.06	29	10.9
PS	76	M	7	1	19.8	3.3		1.88	0.86	154.8	1.6	high	9.6	6.3	0.23	33	10.7
PS	59	M	7	1	21.2	4.2	86	1.37	1.03	141.8	1.8	high	8.2	3.7	3.58	33	8.4
PS	51	M	6	1	24.2	4.1	119	1.29	1.1	131.7	2.5	high	9.2	6.3	0.05	23	9.6

PS	73	M	6	1	25.3	3.8	183	1.72	1.05	141.6	1.1	high	9.6	6.2	0.3	28	13.6
PS	79	M	6	1	18	3.4	199	1.84	0.7	92.94	1.5	high		7.9	0.34	19	11.9
PS	61	M	6	1	20.4	4.6	220	1.33	1.17	132.2	1.6	high	8.9	5.4	0.05	27	12.8
PS	48	F	5	1	19.6	3.6	298	1.99	1.11	122.1	1.8	high	9.8	4.2	0.04	35	11.6
PS	70	F	5	1	18.6	3.7	287	1.14	0.88	88.72	2.1	high	10	5.4	0.22	31	10.4
PS	66	F	5	1	22.9	3.5	120	1.54	0.82	101.3	1.8	high	9.1	4.8	0.05	21	10.6
PS	73	F	5	1	19.6	3.9	134	1.08	1.21	142.1	2.1	high	9.4	8.5	0.2	26	11.4
PS	78	M	5	1	23.1	3.9	162	1.08	1.38	71.11	1.3	high		3.5	0.1	22	11.5
PS	55	M	4	1	24.3	4	212	0.81	0.91	118.3	1.8	high	7.6	4.6	0.28	37	9.7
PS	41	M	4	1	27.6	4.1	143	1.37	1.2	108.8	1.8	high	9.6	5.1	0.03	26	11.3
PS	59	M	4	1	24.4	3.8	150	1.68	0.83	99.62	1.5	high	8.6	3.9	0.04		10.3
PS	41	F	4	1	19.5	3.7	152	1.35	0.91	102.2	1.8	high	8.9	4.6	0.04	31	10.6
PS	61	M	4	1	20	3.8	121	1.65	1.03	84.53	1.6	high	9.7	5.1	0.12	34	10.2
PS	56	M	3	1	28.2	4.2	131	1.28	1.02	115	1.6	high	9.7	5.2	0.38	25	10.2
PS	64	M	3	1	19.2	3.3	283	1.27	0.94	67.19	1.8	high	9.3	3.9	0.42	23	10.5
PS	46	F	3	1	20.8	4.4	164	1.79	0.88	113.1	1.3	high	8.4	2.7	1		9.6
PS	58	M	3	1	22.3	4.4	105	1.28	0.96	144.6	1.5	high	9.5	5.3		30	9.6
PS	45	M	2	1	31	4.3	149	1.92	1.08	119.7	1.5	high	8.3	5.3	0.07	20	12
PS	74	F	2	1	21	4	114	1.16	0.92	123.6	2.1	high	10.4	7.1	0.63	27	10.4
PS	71	M	2	1	22	3.9	163	1.52	0.99	77.68	1.8	high	8.7	2.1	0.09	28	9.6
PS	72	M	2	1	23.8	3.5	133	0.79	0.88	116.4	2.1	high	7.8	6.6	0.18	45	11.5
PS	46	F	9	1	21.2	3.7	120	1.37	1.11	107.9	1.6	high	7.4	4.2	0	32	7.8
PS	62	M	5	1	18.9	3.3	156	1.21	0.8	65.42	1.5	high	10.1	4.9	0.05	27	10.3
PS	77	M	2	1	20.3	3.9	191	1.92	0.82	104.7	1.6	high	9.6	4.9	0.2	18	10.6
PS	60	F	5	1	32.9	4		2.04	0.65	98.13	2.1	high	8.9	4.6	0.1	32	11.8
PS	70	M	6	1	20.2		102	1.12	1.06	131.1	2.2	high		4.5	1.15	26	9.2
PS	67	M	5	1	25.3			1.49	0.78	128.3	2.1	high	8.8	5.1	0.1	28	11.6
PS	78	M	9	1	20.8			1.1	1.07	88.38	2.1	high	9.3	6.7	0.1	19	11.6
PS	54	F	7	1	23		125	1.64	1.17	121.8	2.1	high		5	1.43		8.9
PS	66	M	11	1	20.2			1.49	1.19	119.8	2.1	high	8.2	5.6	0.05	30	10.3
PS	77	M	9	1	21.8			1.46	1.18	110.3	2.1	high	9.7	5.8	0.45	26	11.3
PS	47	F	4	1	19.1		187	1.3	1.04	95.32	1.3	high		5.3	0.2	27	9.2
PS	59	F	3	1	27		111	1.21	0.9	54.37	1.3	high	8	4.2	0.17	26	10.1
PS	69	M	3	1	21.6	3.6	184	1.59	0.81	114.7	1.5	high	9.9	5.5	0.1	28	9
PS	59	F	6	1	16.3	3.6	108	2.09	1.08	110.8	1.5	high	9.1	6.7	0.3	26	11.9
PS	52	F	3	1	18.3	3.8	118	1.42	0.81	69.92	1.5	high	9.6	3.5	0.16	25	10.4
PS	71	M	10	1	18.4	3.5		1.89	0.79	102.3	2.1	high	8.6	6.8	0.19	35	11.9
PS	71	F	9	1	22.4	3.6	197	1.26	0.86	112.4	1.8	high		5.5	0.55		11.8
PS	71	M	8	1	21.4	3.8	157	0.89	0.61	15.2	1.8	high	8.5	6	0.13	25	12.3
PS	74	M	3	1	17.7	3.8	184	1.5	1.01	68.61	1.5	high	8.9	4.8	0.1	29	10.8
PS	61	M	5	1	19	3.4		1.53	1.16	118.9	2.1	high	7.9	3.7	0.1	30	11.4
PS	61	F	5	1	20.1	4	206	0.99	0.77	98.76	1.8	high	9.7	7.2	0.62	19	12.5
PS	61	F	5	1	22.4	3.8	183	1.28	0.8	126.1	1.5	high	7.5	7	0.8	24	11.2
PS	63	M	9	1	22.5	3.7	149	1.24	0.98	125	2.1	high	8.9	3.4	1.35	28	10
PS	60	M	5	1	24.3	2.9	154	1.27	1.03	126.7	1.6	high	9.2	6.6	0.6	17	11
PS	74	M	2	1	21.7	3.5	132	1.59	0.81	92.08	1.4	high	8.1	7.5			9.6
PS	41	F	19	1	24.8	3.9	283	1.68	0.95	133.8	1.8	high	9.9	5.5	0.09	23	11.2
PS	55	M	3	1	24.7	4.2	128	1.3	0.94	120.4	1.3	high		6.1	0.26	28	9.6
PS	55	F	2	1	28.5	3.7	169	1.4	0.62	72.63	1.8	high	8.8	5.4	0.08	19	9.7
PS	44	M	4	1	21.9	4.4	149	1.2	0.96	115.1	2.2	high		6.7		25	11.4
PS	40	F	10	1	18.6	4.3	128	1.4	1.06	117.6	1.5	high	9.8	3.7	0.02	26	12.1
PS	65	M	6	1	20.2	3.7	136	1.86	0.84	113.6	1.8	high		6.1	0.62	30	9.4
PS	61	M	18	1	23.3	4.1	137	0.99	0.85	104	2.1	high		6	1.33		12.4
PS	58	M	16	1	17.8	4.4		1.32	1.01	114	1.8	high		3.1			10.8
PS	69	M	4	1	21.3	4.2	162	1.1	0.86	106.1	2.1	high	9.7	2	12.55	31	8.4
PS	79	M	4	1	22	3.7	112	1.67	0.78	72.38	1.8	high	9.3	4.4		35	10.2
PS	55	F	7	1	24.2	4	179	1.09	0.87	121.6	1	high	8.5	4.6	0.05	20	9.9
PS	74	M	2	1	21.9	3.7	130	1.38	0.88	99.11	2.5	high	9.3	4.1		37	10.1
PS	60	F	4	1	23.9	3.7	179	1.13	0.94	86.57	1.8	high	9.7	4.2			11.2

PS	68	F	4	1	20.4	3.8	109	1.34	0.69	92.64	2.1	high	10	5.2	0		11.4
PS	72	M	15	1	16.5	3.1	153	0.65	0.8	83.6	2.1	high		3.8			11.6
PS	62	M	2	1	23.1	3.6	190	1.59	0.86	78.64	1.3	high	8.9	6	0	23	9.1
PS	62	M	2	1	22	3.5		1.43	0.99	86.41	2.1	high	9.4	4.7	0.1	23	11.7
PS	68	F	2	1	19.2	4.1	141	1.52	0.96	99.19	1.4	high		5		28	11.4
PS	60	M	8	1	23.6	3.7	175	2	0.65	102	1.8	high	9.2	6.4	0	29	10.6
PS	70	M	7	1	23.8	3.9	147	1.22	1.16	106.6	1.3	high	8.8	4.5	0.63	36	10.7
PS	69	M	6	1	22.2	3.3	166	1.54	0.96	118.5	1.6	high		3.1	0.25	46	8.2
PS	64	M	29	1	24.8	3.5	196	1.71	0.96	78.57	2.2	high	10.2	4.9			13.1
PS	53	M	27	1	21.7	3.7	123	1.66	0.87	94.58	1.6	high	9.5	5.3			10.6
PS	49	M	27	1	22.4	3.8	119	1.58	1.06	86.36	2.1	high	9	6.9			10.7
PS	53	M	24	1	21.4	3.7	177	1.67	1.08	110.3	1.6	high	9.8	6.8			10.7
PS	49	M	23	1	22.5	3.8	131	2.03	1.21	143.1	1.5	high	9.6	4.8		29	11.6
PS	45	M	22	1	22.7	3.7	163	1.27	1.08	117.9	1.8	high	9.3	4.8		21	11.4
PS	76	M	21	1	21.1	3.4	129	1.76	1.04	85.98	1.6	high	9.3	4.4			10.2
PS	46	F	19	1	15	3.7	111	1.73	1.01	88.49	1.6	high	8.9	4.3			9
PS	65	F	15	1	22.3	3.6		1.4	1.33	93.83	2.1	high	8	6	0.05	23	11.9
PS	60	F	14	1	18.1	3.5	114	1.23	1.4	70.35	1.5	high	8.6	5.6	0.01	23	10
PS	47	M	12	1	22.4	3.3	142	1.33	0.97	73.8	2.1	high	9.6	4.6	1.17	34	9.2
PS	55	M	11	1	17.9	3.8	153	1.38	0.92	123	2.1	high	9.4	4.9		32	11.1
PS	69	F	11	1	22.1	3.7	173	1.34	1.24	102.9	1.1	high	10.5	4	0.33	29	12.5
PS	56	F	10	1	24.7	3.7		1.28	1.33	126.6	1.8	high	8.5	4.4	0.8	32	10.5
PS	83	F	9	1	14.5	3.1	101	1.53	1.1	67.3	2.2	high		5.6	0.29		11.4
PS	72	F	9	1	17.6	3.1		1.14	1.05	75.46	2.1	high	8.9	5.5	0.1	22	11.3
PS	65	M	8	1	19.1	3.6	169	1.64	1.2	109.9	1.5	high	10.6	5	0.17	26	10.9
PS	57	M	8	1	20.8	4	78	1.2	1.02	119.7	0.8	high		5	8.54	43	10
PS	75	F	7	1	21.2	3.8	105	0.79	0.83	100.7	1.8	high	7.1	6.1	7.17	24	8.7
PS	53	F	6	1	29.1	3.6		2.18	0.87	111.5	1.5	high	8.5	7.3	0.2	27	10.1
PS	53	F	5	1	20.8	4.3		1.39	1	105.3	1.4	high	9.9	5.4		27	12.1
PS	42	F	5	1	21.2	4	206	1.47	0.95	91.21	1.8	high	10.6	4.8	0.15	30	9.3
PS	82	F	4	1	20.8	3.3	195	1.38	1.3	124.2	1.5	high	8.9	3.3	0.4	32	11.1
PS	65	F	4	1	19.6	3.8	172	1.5	0.88	110.5	1.5	high	10.3	4.8	0.15	26	9.8
PS	21	F	4	1	16.4	3.7		1.02	0.71	30.59	1.5	high	8.2	7.7			
PS	72	M	3	1	19.5	3	202	1.72	0.95	94.19	1.4	high	9.6	4.8	0.1		9.4
PS	60	M	3	1	22.7	3.6	200	1.87	0.9	132.1	1.8	high	8.6	3.4	0.15	24	10
PS	52	F	2	1	22.5	3.7	192	1.34	1.12	112.1	1.6	high	9.6	2.9	0.11	25	10.1
PS	54	M	2	1	23.4	3.7	172	1.72	1.08	125.8	1.5	high	8.8	3.6	8.38	34	10.5
PS	75	M	2	1	25.4	3.5	125	1.22	0.83	68.14	2.1	high	9.1	4.7	0	17	11.2
PS	69	F	2	1	23.2	3.7	193	1.66	1.26	114	1.5	high	7.4	4.1	0.1	29	11.6
PS	75	M	5	1	20.2	3.7	130	1.35	0.95	90.77	1.3	high	9	5	0.1	28	10.3
PS	73	F	29	1	18.9			2.09	1.23	70.37	1.8	high		5.8			11.1
PS	86	M	4	1	21.6	3.8	141	1.51	0.82	145.8	2.1	high	8.2	7.6	0.43	48	11
PS	51	F	4	1	17.5	3.9		1.53	1.36	77.97	1.3	high	10.1	4.5		20	
PS	71	F	16	1	19.7	4	166	1.7	0.96	134.1	1.8	high	10.5	5.5	0.05	23	10.6
PS	62	F	6	1	27.3	4.2	154	1.25	1.17	83.98	1.5	high	9.1	6.5	0.05	21	10.3
PS	68	M	6	1	20.1	3.7		0.87	1.12	92.8	2.5	high		6.6	0.01	30	9.6
PS	59	M	9	1	22.6	3.5	162	1.92	0.97	102.8	1.1	high		4.1	0.05	32	10.8
PS	64	F	5	1	22.9	3.5	129	1.25	0.99	103.7	1.8	high	9.4	5.7	0.15		8.6
PS	62	F	13	1	19.2	4	116	1.33	1.04	116.6	1.8	high		6.3		27	9.1
PS	90	M	12	1	24.9	3.5	169	1.86	0.64	148.9	1.4	high	9.6	3.9	0.1	28	10.2
PS	64	F	10	1	18.8	4.5	213	2.03	0.98	120.8	1	high	8.1	6.9	1.82	32	9
PS	65	M	8	1	22.8	4.1		1.04	0.97	102.3	1.5	high		5.8	0.15	26	9.1
PS	75	F	5	1	26	3.6		1.33	0.89	101.6	2.1	high	10.1	6.7	0.19	24	8.7
PS	62	M	4	1	20	3.6		1.65	0.78	82.49	2.2	high		3.3		21	10.4
PS	57	M	4	1	23	4	101	1.21	0.99	114.8	1.4	high	9.4	2.6		32	9.8
PS	67	M	4	1	23	3.9	166	1.19	1.1	97.17	2.2	high		6.7		30	8.5
PS	68	F	4	1	19.1	4.3	174	1.4	0.98	67.08	1	high	8.9	4.4	0.05	14	8.9
PS	92	F	4	1	18.7	3.8	152	1.17	0.87	84.8	2.1	high	8.3	5.6	0.89	26	9.9
PS	53	F	3	1	17.9	3.5	168	1.75	0.82	78.63	1.4	high	8.4	5.6	0.25	26	11.5

PS	65	M	3	1	23.3	3.9	151	1.39	1.06	107.1	2.2	high	8.7	3.5	0.06	26	9.1
PS	83	F	3	1	22.4	3.5		1.16	0.74	75.69	1.1	high		6.1	0.62	28	8.5
PS	90	M	3	1	18.7	3.5	151	1.54	0.8	65.02	1.6	high	9	4	0.03	28	11.1
PS	85	F	2	1	19.6	2.9	147	2.09	0.55	87.97	1.8	high	9.8	4.9	0.04	37	10.7
PS	73	M	2	1	25	3.8	195	1.4	0.89	71.37	1.8	high	8.3	4.8	0.55		10.3
PS	58	M	2	1	30.6	3.8		1.63	0.91	111	1.5	high	9.1	3.4	0.03	21	10.3
PS	45	F	19	1	15.7	4	183	1.61	0.92	118.7	1.8	high	9.4	4.9	0.42	28	10.9
PS	66	F	17	1	20.1	3.8	108	1.28	0.83	132.8	1.6	high	8.6	3.1	0.72	38	9
PS	35	M	12	1	27.1	4.5	131	1.67	0.98	111.8	2.1	high	9.7	3.7	0.3	31	10
PS	82	F	8	1	16.8	4.3	160	1.64	1.02	120.4	1.3	high	8.3	5.7	0.2	27	10
PS	65	F	7	1	21.5	3.9	97	1.3	0.82	134.3	1.5	high	8.6	5.6	0.3	35	10.6
PS	66	F	7	1	21.2	4.4	186	1.56	1.16	140.4	1	high	9.1	4.4	0.2	26	10.3
PS	68	M	6	1	21.3	4.1	130	1.49	1.24	114	1.5	high	9.1	5.9	0.1		11
PS	56	M	6	1	24.1	2.7	111	1.12	0.73	130.1	1.8	high	10.5	5.6	0.31	37	10.4
PS	65	F	5	1	23.2	4	133	1.39	0.96	112.8	1.5	high		4.6	0	32	12.1
PS	53	F	5	1	19.1	4.3	151	1.7	0.8	120.6	1	high	6.8	5.3	0.93	26	10.1
PS	80	M	4	1	18.3	4	116	1.13	0.86	142.5	1.6	high	8.6	5.3		29	9.3
PS	76	M	4	1	19.4	3		1.25	0.63	78.41	2.1	high	8.7	5.2	0.03	22	10.6
PS	65	M	4	1	21.6	3.4	119	1.28	0.96	122.7	1.8	high	9.9	6.4		29	11.1
PS	77	F	4	1	26.9	3.9		1.19	0.95	105.1	2.1	high	9.5	3.7	0.03	25	11.9
PS	67	F	4	1	17.4	4	185	1.97	1	65.31	1.4	high	9	6.2	0.02	31	10.3
PS	67	M	3	1	16.6	4.8	176	1.5	0.97	100.1	1.3	high	8.2	5.8	0.2	20	11.9
PS	76	M	3	1	16.6	3.2		1.4	0.53	95.66	2.1	high	8.9	6	1.2	32	13.1
PS	68	M	3	1	24.5	4.2	149	1.84	0.98	83.99	1.5	high	9.4	4.7	0.12	35	10.1
PS	70	F	3	1	22.9	2.2	186	2.43	1.04	63.56	1.8	high	9.5	4.8	0.2	29	8.6
PS	64	F	2	1	20.9	4	176	1.21	0.79	113.3	2.2	high	10.2	6.1	3.3	27	9.8
PS	68	M	2	1	15.4	4	112	2.11	0.99	91.48	1.6	high	8.3	5.6	0	37	11.5
PS	47	M	2	1	22.4	4.2	157	1.79	1.02	77.45	2.1	high	8	7	0	29	11
PS	69	M	6	1	23.8	3.9	165	1.52	0.91	106.7	1.4	high		7.1	0.05	27	12.9
PS	74	F	3	1	18.1	3.7		0.86	0.78	58.87	2.1	high	8.5	6.3	0.24	26	11.4
PS	82	F	3	1	19.5	3.7		1.8	0.75	51.4	1.5	high		6	0.07	28	9.9
PS	63	M	2	1	17.6	4.1	121	1.39	0.81	92.19	2.1	high	9.1	6.5	0.2	19	11.4
PS	55	F	28	1	23	3.6	96	1.01	1.1	109.6	1.8	high		4	0.15	25	10.3
PS	79	M	4	1	21.1	3.6	109	1.17	0.87	80.41	1.5	high	9.4	5	0		10.7
PS	62	F	4	1	18.9			1.26	0.91	136.2	2.5	high	9	3.2	0.11	18	11.9
PS	68	M	4	1	26.3	3.3	157	1.69	0.95	92.05	1.4	high		4.1		27	9.8
PS	54	M	5	1	21.6	3.6	136	1.27	0.79	92.42	1.5	high	9.9	4.7	0.05	31	11
PS	82	F	3	1	19.8	3.9	190	1.71	0.81	86.26	1.8	high	8.5	4.9		21	11.4
PS	65	M	4	1	22.4	3.8	149	0.83	0.76	57.42	2.1	high	9.2	6.6	0.1	27	11.4
PS	70	M	8	1	24.1	3.5	144	1.64	0.71	116.2	2.1	high	10.3	5.3	0.1	26	9.8
PS	68	F	9	1	26.7	3.8	178	1.7	0.96	84.44	2.1	high	9.4	7.3	0.1	30	12.2
PS	68	M	7	1	21.6		146	1.51	1.15	138.2	1.5	high	7.8	6.8	0.5	29	13.1
PS	59	M	33	1	20.8	3.4	230	1.74	1.15	138.8	1	high	9.6	6.6		25	10.2
PS	61	F	25	1	23.6	3.5	169	1.44	1.11	128.5	1.8	high	10.9	5.4		27	11.1
PS	52	F	15	1	13.4	3.3	126	1.49	0.82	89.83	1.3	high	8.3	6.9	0.11	24	10.7
PS	43	M	11	1	25.9	3.8	191	1.54	1	127.7	1.5	high	10.2	5.4	0.12	29	10.1
PS	63	F	10	1	19		153	1.36	0.88		1.5	high	9.9	4.4			10.9
PS	82	F	10	1	19.5	3.8	130	1.23	0.8	141.9	0.6	high	8.5	3.5	0.61	36	9.4
PS	64	M	9	1	20.1	4.5		1.36	0.84	116	2.1	high	8.3	7.8	0.13	27	10.7
PS	77	F	9	1	19	3.5		1.51	1.05	133.8	2.1	high	10	4.6	1.63	29	9.9
PS	71	F	7	1	23.9	3.7	98	1.66	0.92	101.5	1.8	high	8.4	5.1	0.1	22	10.5
PS	68	M	7	1	20.1	3.6	155	1.15	0.87	137.6	2.1	high	8.7	7.8	0.44		
PS	67	M	6	1	14.7	3		1.72	1.05	44.77	2.1	high	8.8	3.5	0.16	44	8.7
PS	47	M	6	1	37.6	4.4	180	1.36	0.75	95.53	1.1	high	7.7	4.8	0.05	21	9.5
PS	72	F	5	1	27	3	120	1.59	0.74	106.1	1.5	high	8.7	4	0.4	44	11.1
PS	60	M	4	1	20.2	4.2	115	1.29	0.92	120.8	1.4	high	9.4	3.8	0.5		9.8
PS	57	M	4	1	22	3.6	129	1.06	1.08	135.3	2.5	high	8.7	5.4	0.6	37	11.2
PS	60	M	4	1	22.5	2.3		1.86	1.21	113.5	1.6	high	8.6	3.7	0.12	26	10.5
PS	66	M	3	1	20.6	3.8	176	1.29	0.76	93.82	1.8	high	8.7	5.9			11.7

PS	36	F	3	1	16.6	4	138	1.3	1.19	113.3	1.8	high	8.6	6.6	1.2	38	10.7
PS	66	F	3	1	15.5	3.5	120	1.93	0.66	81.22	2.1	high	8.7	5.7	0.11	1	12.5
PS	63	F	3	1	20	3.6	154	1.51	0.87	97.79	2.1	high		6.1	0.05	27	9
PS	47	M	2	1	24	4	111	1.49	0.68	93.92	1.5	high	8.7	5.5	0.17	27	12
PS	63	F	2	1	22.2	3.7		1.47	0.88	131.2	2.1	high	9	3.7	0.18	40	9
PS	42	F	2	1	22.6	3.8	120	1.18	0.72	55.82	1.1	high	8	3.9		20	10.4
PS	62	M	2	1	23.8	3.7	159	1.5	0.79	59.77	1.8	high	8.3	4.5		31	11.6
PS	55	M	2	1	24.5	3.6	100	1.5	0.69	82.73	1.6	high	9	4.6		29	9.1
PS	83	F	2	1	19.1	2.4	119	1.37	0.98	92.1	1	high		6.1	0.09	23	7.7
PS	66	F	15	1	25.4	3.8	155	1.39	0.86	119	2.1	high	9.3	5.3	0.42	30	11.8
PS	79	M	10	1	20.8	4.4	195	1.6	1.01	95.19	1.8	high	9.7	3	0.08		9.9
PS	68	M	24	1	21.2	3.4	166	1.07	1.01	108.7	2.2	high	9.2	6.4	0.04	29	11.4
PS	52	M	17	1	17.6		142	1.29	0.85		2.1	high	9.3	6.8	0.37	30	10.6
PS	59	F	3	1	26.8	3.8	229	2.03	0.9	107.2	1.8	high	9.1	3.9		25	9.5
PS	55	F	16	1	17.2	2.4	137	0.9	1.08	59.58	1.3	high	9.8	3.7		22	10.9
PS	72	M	13	1	19.8	4.1	195	1.7	0.72	86.76	1.3	high		3.3	1.4	29	11.7
PS	53	F	10	1	20.4	3.9	120	1.33	0.97	142.8	1.6	high	9	6.6	0	23	12.3
PS	51	M	6	1	20.9	4	122	1.6	0.92	115	1.8	high		5.3	0.05	31	10.4
PS	60	F	6	1	20.9	4	179	1.86	0.71	95.3	1.6	high	8.8	6.4	0.03	39	10.2
PS	54	F	3	1	19.3	3.9	143	1.64	0.68	63.07	2.1	high	8.2	4.1	0.84	33	11
PS	40	F	3	1	18	4.4	175	1.84	0.76	110.4	1.5	high		5.5	0.05	26	10.1
PS	43	M	27	1	17.2	3.7	114	1.64	0.69	96.37	2.2	high	10.2	3.7		17	10.5
PS	61	F	20	1	16.7	4	112	1.62	0.87	99	1.5	high	9.2	4.3	0.12	32	10.7
PS	65	F	9	1	24.2	4.2	88	1.14	0.85	72.26	2.1	high		6.4			7.5
PS	62	M	9	1	19.4	3.9	139	1.92	0.92	142.7	1.3	high	9.4	5.7	0.1	30	7.4
PS	74	F	3	1	21.9	3.7	160	1.67	0.84	104.6	1.5	high	9.2	3.7	0.04	28	10
PS	86	F	3	1	16	3.4	159	1.53	0.48	25.4	1	high		4.7	2.08	23	10.2
PS	76	F	2	1	13.8	3.1	150	1.18	0.81	76.21	1.5	high	9.9	4.6	0.02	28	10.1
PS	52	M	29	1	19.8	2.8	122	1.4		261.6	2.2	high	9.2	7.2			8.3
PS	60	F	24	1	19.4		157	1.55	0.92	70.97	1.6	high	9.7	6	0.05	25	12.8
PS	75	F	17	1	19.6		150	1.55	0.83	93.99	2.2	high	9.2	5.1	0.8		10.1
PS	70	M	12	1	20.8		162	1.7	0.87	83.25	2.1	high	9.8	4.7	0.12	29	11.5
PS	60	M	7	1	21		202	1.45	1.05	88.85	1	high	7.9	7	8.8	25	9.9
PS	46	M	6	1	22.1		130	1.42	0.68	104.8	1.4	high	8.5	6.7	0.91	31	10.8
PS	70	M	6	1	26		201	1.38	0.91	112.2	1.1	high	8.3	5.4	0.8	23	9.9
PS	59	M	3	1	21.3		128	1.27	0.8	90.61	1.8	high	8.9	5.7		20	10.1
PS	50	F	2	1	22		118	1.36	1.27	94.22	2.1	high	9.1	3.8		41	9.2
PS	61	F	5	1	19.7	3.8	119	1.01	0.64	43.16	1.5	high	7.3	3.8	0.09	15	9
PS	67	M	10	1	24.1	3.3	158	1.49	0.75	123.1	1.8	high	8.5	7.4	0.05	20	8.9
PS	70	F	4	1	27.1	3.6	132	1.69	0.97	77.88	1.4	high		4.4		35	11
PS	33	M	3	1	20.2	4.6	189	1.89	0.95	129.5	1.4	high	9.3	4.4	0.18	35	10
PS	50	M	4	1	23.8	2.4	108	1.67	0.78	101.7	2.1	high	9.9	6.8	0.1	26	8.6
PS	35	M	12	1	22.2	4.3	228	1.52	1.2	111.3	1.3	high	9.8	5.2	0		10.7
PS	57	M	10	1	22.6	4.4	227	1.44	1.09	127.9	1.5	high	8.9	7	0.16	25	11.4
PS	40	M	8	1	25.1	4.1	158	1.27	0.8	119.6	2.1	high	8.4	4.7	4.85	30	9.6
PS	53	M	6	1	20.7	4		1.85	1.28	130.7	2.5	high	8.1	4.5	0.1	22	10.6
PS	63	M	2	1	23	3.8	197	1.27	0.79	119	1.8	high	8.2	3.7	0.05	27	10.7
PS	66	F	5	1	17.4	4	152	1.52	0.83	40.93	1.5	high		6.4	0.04	25	9.2
PS	67	F	11	1	22.6	3.8	187	1.29	1.05	128.7	2.1	high	8.1	5		23	11.6
PS	47	M	30	1	17.8	3.5	156	1.65	1.11	71.88	2.2	high	10.3	5	0.1		11
PS	46	M	16	1	23	4.3	126	1.07	1.01	133.9	2.5	high	9.2	5.6	0.08	23	10.3
PS	63	F	12	1	18.3	4	151	1.49	1.43	126.2	1.3	high	9.1	6.2	0.08	29	10.7
PS	69	F	12	1	16.5	3.3	153	1.48	0.66	86.44	1.5	high	9.1	7	0.08	33	10.4
PS	70	F	11	1	22	3.2	190	1.17	1.13	124.3	1.5	high	10	5		22	12.7
PS	66	F	19	1	18.9	3.8	216	1.25	0.77	109.4	1.3	high	9.8	3.9	1.33	20	10
PS	68	F	16	1	24.1			1.11	1.03	134.5	1.5	high					
PS	43	M	7	1	31.5	3.9	153	2.03	0.87	88.18	1.8	high	8.9	5.8	0.41	34	11.2
PS	60	M	3	1	22.6	3.8	207	1.23	0.91	95.72	1.8	high	9.4	6	0.5	29	12.2
PS	41	M	2	1	24.5	3.8		1.38	0.85	64.56	2.5	high	9.5	5.9		22	

PS	67	F	2	1	28.8	3.7	153	1.44	0.93	72.73	2.1	high		5.7	0.05	25	9.5
PS	50	F	21	1	16.5	3.8	119	1.3	1.11	107.6	1	high	8.7	6.4	0.2		10.6
PS	52	F	17	1	21.5	1.8		1.55	0.98	112.8	2.1	high	8	2.7	0.1	17	9.7
PS	66	M	14	1	20.1	1.9	148	1.35	0.98	123.2	1.3	high	9.2	3.3	0.08	18	9
PS	65	F	11	1	16.4	1.4	108	1.35	0.79	64.67	2.1	high	8.8	6.2	0.03	17	12.1
PS	74	M	10	1	21.8	1.4	130	1.57	0.62	111.5	1.6	high	8.8	3.5	0.17	23	10.8
PS	73	F	10	1	17.4	4	158	1.48	0.99	118.5	1.5	high	9.4	4.5	0.08	24	9
PS	72	F	9	1	16.2	1.8	156	1.78	0.74	100.6	2.1	high	10	6.9	0.4	29	10.1
PS	79	M	8	1	15.6	3.4	140	1.1	0.93	106.9	1.6	high		7.2	0.05	28	9.1
PS	39	M	8	1	20.6	4	151	1.62	0.79	74.48	2	high	8.9	5.1	1.76	29	11.5
PS	65	F	7	1	23.3	3.9	168	1.6	0.86	93.75	1.8	high	9.6	7.7	0	31	12
PS	92	M	5	1	16.5	2.8	169	1.25	0.85	70.69	1.6	high		5	0.81	28	12
PS	56	M	10	1	22.9	4.3	102	1.62	1.05	132.2	1.4	high	8.5	3.3	3.92	30	10.9
PS	54	M	8	1	24	4.1	174	1.57	0.8	124.6	2.1	high	8.8	3.8	0.4	21	11.7
PS	54	M	3	1	19.6	4	89	1.63	1.06	128.3	2.2	high	8.6	5	0.1	30	10.2
PS	70	F	14	1	15.2	3.8	133	1.45	1.17	96.1	1.8	high	9.9	3.3	0.3	31	10.8
PS	71	F	11	1	24.5	3.9	140	1.55	0.72	114.6	1.5	high	9.6	6	2.9	24	9.9
PS	67	M	8	1	24.4	4.3	124	1.41	0.77	117.7	1.8	high	9.3	5.3		22	10.4
PS	84	M	5	1	19.4	3.8	150	1.73	1	77.61	1.5	high	7.1	5.1	0.81	22	8.7
PS	83	M	2	1	19	3.7	154	1.24	0.77	77.06	2.1	high	9	6.5	0.11	26	8.5
PS	61	M	2	1	20.2	3.6	156	2.14	0.84	76.34	1.5	high	8.9	4.5	0.03	29	10.6
PS	50	F	8	1	18.5	4.1	110	1.36	0.81	119.2	1.8	high		5.2	0.53		11
PS	64	F	2	1	26.8	4.1	124	1.69	0.84	61.2	1.8	high	8.6	4.2	3.2	32	7.3
PS	58	F	8	1	19.4	1	153	1.83	0.87	109.6	1.5	high	9.8	4.5	0.05	25	10.3
PS	83	F	5	1	21.2	3.9	176	1.4	1.44	109.6	1.5	high	6.9	6.5	0.04		11.1
PS	53	M	7	1	18.3	4	121	1.68	0.99	87.34	1.5	high	8.4	5.2	0	26	11.4
PS	53	F	14	1	19.3	3	103	1.64	0.98	98.29	2.2	high	9.3	2.9	0.1	25	11.3
PS	68	M	2	1	21.4	3.7	230	1.69	0.83	118.3	1.8	high	9.4	4.8	0.21	21	9.8
PS	59	M	8	1	20.5	3.8	127	1.15	1.07	117.8	1.5	high	7.9	3		22	9.7
PS	69	F	29	1	17.6	4.4	206	1.44	1.13	110.7	1.4	high	9.9	10.2	3.5		9.8
PS	53	M	24	1	19.5	3.9	84	1.24	1.22	115	2.2	high	7.4	6.2	0.27	29	10.7
PS	67	M	6	1	18.7	3.7	140	1.82	0.78	91.38	2.2	high	10.8	7		32	10.5
PS	60	F	23	1	18.2	3.8	175	1.39	1.23	109	1	high	8.9	5.3	0.08	42	12.4
PS	76	F	10	1	16.6	3.8		1.48	0.81	102.4	1.5	high	7.9	4.6			11
PS	88	M	9	1	19.5	3.9	91	1.39	0.75	100.5	1.8	high	11.2	5.7	0.4	26	12.2
PS	87	F	8	1	22.4	3.8	227	1.94	1	99.28	1	high	9.3	4.1	0.1	20	8.2
PS	57	F	8	1	21.8	3.8	216	1.58	0.81	99.23	1.5	high		5.6	0.02	31	11.1
PS	53	F	7	1	19.2	4.1	84	1.23	1.16	110.4	2.1	high	8	5.2	0.03	41	10.5
PS	41	F	7	1	19.5	4.5	151	1.4	0.86	123.4	1.8	high	8.5	4.4	0.1	13	11.1
PS	59	F	6	1	17.1	4.4	209	1.22	1.11	144	1.8	high	10.1	5.8	0.22		11.4
PS	65	M	6	1	19.1	3.5	131	1.43	0.89	113.2	1.3	high	8.3	4.7	0.14	22	10.9
PS	68	F	5	1	23.2	4	132	1.44	1.01	110.8	1.5	high	8.3	4.4	0.17	28	9.2
PS	74	F	3	1	20.8	3.1		2.25	0.86	86.79	1.5	high	9.2	6.4		34	10
PS	67	M	23	1	25.4	4.2	120	1.74	0.82	123.7	1.8	high	10	5.3	0.2	28	8.5
PS	55	M	20	1	18.1	3.1	205	1.4	0.87	105.7	1	high	11	4.1	0.1	21	10.9
PS	70	F	20	0	17.9	3.4	132	1.36	1.25	123.8	1.8	high		3.1		25	10.2
PS	64	M	18	1	15.7	3.4	132	1.31	0.85	91.27	1	high		5.4	0.05	32	9.5
PS	61	F	18	1	17.9	3.7	154	1.14	0.98	119.2	1.8	high	9	4.6	0.1	18	11.3
PS	58	M	17	1	20.1	4.3	122	1.57	0.79	98.93	1.8	high	9.2	4	0.1	25	10.7
PS	46	F	12	1	23.4	4	207	1.12	0.73	117.1	2.2	high	8.7	7.7		21	10.9
PS	50	M	12	1	23.9	4.1		1.82	0.88	110	2.1	high	7.8	3.1	0.6	29	8.3
PS	52	F	11	1	24	3.8	175	1.11	1.05	126.7	2.2	high		5.4		27	10.6
PS	54	M	7	1	24.1	4.1		1.01	1.11	111	1.8	high	9.4	3.2	7.24	23	9.9
PS	65	M	7	1	23.2	3.9	151	1.63	1.11	138.3	1.4	high	10.2	5.7		28	9.9
PS	54	F	7	1	20.8	3.6	142	1.63	1.01	114	2.1	high	9.8	5.7		28	11.7
PS	56	M	7	1	20.4	4.5	202	1.4	0.86	86.07	2.1	high	9.1	4.9	0.44	30	9.3
PS	61	M	6	1	23.1	4.4	162	1.85	0.76	92.87	0.8	high	8.1	2.7	0.07	30	9.6
PS	61	M	6	1	18.8	4	129	1.6	0.85	90.45	1.8	high	8.6	6	0	31	10.3
PS	64	F	6	1	19.3	3.9	150	1.39	0.88	89.87	1.3	high	8.3	6.3	2.45	28	9.6

PS	49	M	5	1	28.5	4.2	175	1.88	0.96	89.04	1.8	high	9.8	7.7	0.51	24	9.2
PS	69	M	5	1	16.4	4.1	186	1.41	1.05	77.23	1.4	high		4	0.83	28	9.8
PS	60	M	4	1	20	4	175	1.13	0.86	132.5	2.5	high	8.8	4.2	0	25	11.1
PS	60	M	3	1	20	3.7		0.79	1.22	76.07	2.1	high	8.2	5.2	0.07	20	10.7
PS	76	M	3	1	24.9	4		0.94	1.3	115.5	1.8	high	7.5	4.7	0.93	18	10.6
PS	71	F	3	1	16.7	4	154	1.78	1.28	99	1.4	high	9.6	3.4	0.4	30	10.4
PS	53	F	3	1	27.9	3.1	163	1.21	0.94	125.2	1.8	high	9.2	6.4	2.68		8.1
PS	56	M	2	1	20.5	4.1	100	1.93	0.88	78.76	1.3	high	8.4	5.8	0.05	21	9.3
PS	58	M	2	1	23.4	4.1	163	1.57	0.65	53.78	1.5	high		4.7		25	12
PS	35	M	2	1	21.9	3.6		1.43	1	100.2	1.5	high	9.5	3.5		31	10.3
PS	66	M	2	1	22.4	3.9	125	1.31	0.86	155.4	0.6	high	9	6.2	1	50	10.1
PS	74	F	2	1	24.5	3.4	171	1.37	0.72	56.78	1.3	high	9.6	4.7	0		8.5
PS	58	F	2	1	22.5	4	211	1.38	1.16	46.62	2.1	high	10.6	10.6	0.3	25	10.4
PS	51	M	29	1	18.5	3.7	155	1.48	0.78	94.25	1.6	high	8.7	5.9	0.08	26	10.2
PS	57	M	22	1	20.4	3.8	147	1.19	0.92	128.9	2.2	high	8.7	6.1	0.77	22	9.4
PS	76	M	17	1	18.7	3.8	158	1.56	0.77	121.2	1.8	high		6.9	0.36	37	9.3
PS	69	M	16	1	17.3	3.6	233	1.72	0.89	91.45	2.1	high		4.8		25	10.3
PS	52	M	14	1	20.6	3.6	201	1.42	0.91	111	1.4	high	10.6	8.7	3	30	9.8
PS	62	M	6	1	21.2	3.6	191	1.79	0.77	122.8	1.8	high	8.8	4.8	0.1	30	10.5
PS	58	M	3	1	21.3	3.6	145	1.17	0.95	93.03	2.2	high		7.8	0.06	27	12.1
PS	77	F	15	1	18.3	4.2	143	1.29	0.81	103.6	1.8	high	9.4	5.8	7.24	27	11
PS	46	F	17	1	18.6	3.9	142	1.35	0.68	66.04	1.5	high		5.8	0.5	31	11.4
PS	75	F	8	1	18.6		174	1.74	0.99	132.5	1	high	10	5.8	0.3	28	10.7
PS	75	M	3	1	23	3.5	136	1.45	0.72	149.6	1.8	high		6.5	0.05	28	9.9
PS	72	M	6	1	17.6	3.3	175	1.88	0.74	74.67	1.5	high	8.5	5.2	0.09	32	9.5
PS	92	F	5	1	22.8	3.8	136	1.37	1	159.7	1.5	high	10	6.7	0.96	28	8.8
PS	59	F	29	1	20.3		153	1.57	0.82	100.4	1	high	10	5.6	0.4	29	8.5
PS	64	F	15	1	20.3		204	1.63	1.08	108.7	1.8	high	9.9	5	0.1	29	11.7
PS	64	M	10	1	21.7		133	1.29	0.96	123.6	1	high	8.5	4	0.67	36	9.5
PS	36	M	9	1	17.9		85	1.2	1.03	101.2	1.8	high	8.2	4.2	0.4	10	
PS	69	M	6	1	25.2		156	1.51	1.01	108.5	1.8	high	8	5.1	0.17		10.1
PS	55	F	10	1	31.2	4	205	1.52	1.13	147.5	1.3	high	9.6	4.6	0	29	10.7
PS	75	M	5	1	20	3.5	141	1.31	0.96	112.8	1.5	high	9.8	6.7	0.1	26	14.3
PS	61	F	5	1	18.1	4	150	1.57	0.82	48.54	2.1	high	8.3	5.5	0.11	27	11.8
PS	49	M	3	1	22	4.3	166	1.75	0.75	89.26	1.5	high	8.4	5	1.31	34	11.1
PS	53	M	7	1	31.1	3.8	166	1.61	1.04	107.5	1.3	high	10.2	5.9	0.1	20	8
PS	58	F	14	1	17.9	4		1.56	0.91	105.4	2.1	high	10.3	8.3	0.74	31	11.8
PS	65	M	8	1	26.3	3.2	170	2.12	0.93	122.8	1.3	high	8.5	3.5	0.7	26	7.3
PS	58	M	4	1	21.1	3.9	120	1.32	0.82	116.4	1.8	high		5.6	0.55	19	10.8
PS	68	F	5	1	22.6		145	1.98	0.98	65.22	1.8	high	10.6	6.5	0.1	25	10.1
PS	81	M	6	1	20.4	3.5	221	1.45	0.83	103.8	1.5	high	10.2	4.3	0.03	26	11.4
PS	79	M	5	1	19.4	3.8	173	1.39	0.84	88.49	1.8	high	9	4.9	0.12	24	11.8
PS	59	F	10	1	23.2	3.6	142	1.75	1.13	104.1	1.8	high	9.8	6.2	0	21	6.5
PS	67	M	4	1	20.8	3.4	139	1.55	0.66	84.48	0.8	high	10.1	3.4		37	10
PS	59	M	7	1	23.5	3.9		1.54	1.04	119.9	1.8	high	9.4	6	0.03	25	10.9
PS	53	M	6	1	19.6	4.7	136	1.41	1.09	86.41	1.8	high	10.4	6.5	0		8.9
PS	62	M	22	1	23.6	4.4		1.29	0.96	131.9	1.6	high	9.2	5.6	8.44	29	8.7
PS	65	F	15	1	17.2	4	139	1.47	1.31	115.4	1.5	high	8.7	5.6	0.2	32	9.2
PS	67	M	2	1	24.3	3.9	141	1.57	0.82	81.66	2.1	high	9.2	6.1	0.02	34	12.1
PS	70	F	14	1	22.6	3.6	183	1.43	0.73	90.43	1.8	high	7.1	6.4		23	12
PS	54	M	5	1	22.7	3.7	165	1.27	0.84	104.9	1.5	high	7.6	6.3	0.5	19	12.6
PS	79	F	3	1	18.6	3.7	117	1.4	0.75	85.75	1.4	high	8.1	3	1.6	26	8.5
PS	64	M	2	1	21.6	4.1	127	1.33	0.74	98.64	1.8	high	9	6.2	0.15		13.3
PS	28	M	23	1	19.9	4.1	216	1.15	0.99	108.6	2.1	high	9.9	5.1	0.58	23	9.8
PS	62	F	14	1	18.3	4.3	170	1.3	1.13	98.84	1.8	high	9.2	3.5	1.6	28	10.1
PS	57	F	12	1	24	4.3		1.44	0.96	119.1	2.1	high	9.3	5.2	0.05	26	10.6
PS	65	F	8	1	16.6	4.1	154	1.81	1.07	131.3	1.5	high	8.6	4.8		21	
PS	73	F	5	1	19.6	3.8	132	1.23	1.26	108.7	1.5	high	10.4	5.2		29	10.2
PS	70	F	4	1	19.8	3.5		1.95	0.79	108.2	2.1	high	8.7	6	0.1	44	11.9

PS	44	M	22	1	19.4	3.6	203	1.25	0.98	123	1.8	high	11.1	7.7	0.2	31	11.1
PS	58	F	18	1	18.7	3.4	202	1.8	1.08	126.1	1.8	high	8	4.2	0.05	24	10.5
PS	58	M	16	1	19.7	4.3	123	1.47	1.17	103.1	2.2	high	9.9	4.7	0.05	24	10.2
PS	64	M	6	1	18.6	4.4	148	0.98	1.14	145.5	1.8	high		6.6	0.49	24	10.1
PS	64	F	18	1	20.7	3	215	1.69	1.16	119.7	2.2	high	9.4	7.8	0.06		12.8
PS	49	M	13	1	20.3	4.1	117	1.84	1.14	96.67	1.5	high	8.8	4.2	0.06	27	11.3
PS	33	M	7	1	19.7	4.1	141	1.12	0.98	89.54	1.3	high		7.2	0	28	9.8
PS	75	F	4	1	16.7	4.1	187	1.36	0.97	127.3	2.1	high		3.8	0.29		12
PS	73	F	4	1	18	3.9	128	1.51	0.97	130.3	1.5	high	9.3	6.2	0.06	29	11.4
PS	72	F	4	1	19.2	3.7	157	1.27	1.09	79.72	1.8	high	9.6	4.5	0	22	7
PS	58	F	3	1	25.6	4	153	1.56	1.49	103.1	1.8	high	8.6	5.6	0.03	23	10.2
PS	55	M	2	1	16.3	4.4		1.54	0.86	72.03	2.1	high	10.2	5.2		23	
PS	63	M	2	1	24.6	4.3	139	1.63	1.13	95.87	1.5	high	8.4	4.1	0.1	25	10.1
PS	59	M	21	1	20.8	4	172	1.7	0.8	112.1	1.6	high	10.1	6.2	0.15	23	12
PS	37	M	13	1	25.9	3.8		1.4	0.65	107.6	2.1	high	10.2	6.7	0.11	24	11.9
PS	75	F	3	1	18.8	3.4	153	1.74	0.87	128.7	1.8	high		5.1	0.09	23	8
PS	37	M	10	1	27.9	3.8	141	1.41	1.01	136.2	1.8	high	9	6.1	0.04	19	11.2
PS	47	F	2	1	21.9	3.6	141	1.8	1.02	123.9	1.8	high	9.2	4.2	0		12.5
PS	65	F	28	1	18.4	3.9	124	1.34	0.84	83.3	1.8	high	8.8	7.8		28	8.7
PS	72	M	25	1	19.2	3.5	151	1.79	0.97	98.7	1.8	high	8.4	7.3	0.5	9	
PS	50	M	23	1	23.8	3.9	182	1.33	0.9	112.2	1.6	high	9.3	5	0.21	21	10.5
PS	61	M	20	1	19.4	4.2	189	1.75	1.21	98.86	1.8	high	9	4.3		20	10.4
PS	65	M	9	1	19.9	4	132	1.61	1.25	102.9	1.5	high	9.4	7.1	0.1	33	9.3
PS	79	M	7	1	20.9	3.3	137	1.4	0.79	106	2.1	high	9.8	4.9	0.08	28	10.9
PS	33	F	7	1	22.7	3.9	108	1.72	0.94	101.1	1.8	high	10	4.9	0.07	28	11.1
PS	62	F	3	1	20.2	3.5	178	1.2	1.03	85.11	1	high	8.2	3.5	0.04	16	12.1
PS	73	M	2	1	21.6	3.7	160	1.73	1.02	113.6	1.4	high	9.1	5.4	0	18	12.9
PS	62	M	2	1	23.8	4.9		1.25	0.82	90.29	1.8	high	8.6	4.9	0	23	9.3
PS	74	F	2	1	19.4	4	221	1.35	0.83	54.79	1.5	high		3.7	0.05	21	9.2
PS	67	M	11	1	20.5		104	1.45	1.19	130.6	1.8	high			0.19		
PS	47	F	6	1	19.3		162	1.71	1.12	101.3	2.1	high	10.3	6.8	0.11	30	11.1
PS	59	M	4	1	24.1		178	1.32	0.78	79.23	2.1	high	10.7	6	0.1	24	11.2
PS	58	M	3	1	22		224	1.36	1.02	118.9	1.5	high		5.3	0.1	18	9.6
PS	70	M	2	1	22.7			1.42	0.76	59.34	2.1	high	7.9	5.1	0.03	53	12.2
PS	67	M	3	1	18.8	6.8	131	0.83	0.9	100.6	2.1	high	9.5	4.7	0.14	27	9.3
PS	55	M	25	1	22.1	3.7		1.4	1.02	122.8	1.8	high		4.3			8.1
PS	67	F	22	1	17	3.5	129	1.33	0.89	86.74	1.6	high	8.3	5.8	0.78		8.4
PS	55	F	21	1	18.8	4.2	114	1.09	1.2	126.3	1.8	high	7.6	4.4	5	30	7.3
PS	68	M	19	1	18.7	3.5	178	1.23	0.81	106	1.8	high	8.9	6		28	10.3
PS	51	M	15	1	21.8	4.1	167	1.38	0.79	122.4	2.2	high		4.4	0	23	10.8
PS	75	M	15	1	21.3	4	182	1.91	0.91	115.4	1.5	high	9.9	5.8	0.1	29	8.5
PS	56	M	13	1	24.6	4.1		1.48	0.87	108.3	2.2	high	9.1	5	0.04	27	
PS	49	F	13	1	22	3.9	96	0.79	1.02	113.2	2.1	high		6.7			8.6
PS	52	F	13	1	20.7	3.9	227	1.58	0.98	129.7	1.8	high	9.5	5.2	0.19	16	8.2
PS	58	F	12	1	23.1	3.8	178	1.31	1.04	109.4	2.1	high	8.8	7	0.16	35	9.9
PS	68	F	11	1	17.2	3.8		1.41	0.57	68.14	1.4	high	8.5	5.3		25	11.8
PS	45	F	11	1	17.3	4.1	170	1.22	0.91	107.8	2.1	high	9.2	6.9	0.53	30	11.9
PS	62	M	10	1	18.4	3.6	147	1.34	0.77	109.2	2.1	high		3.5	0.05	23	10
PS	58	F	10	1	21.7	3.6	159	1.08	0.94	122.9	2.5	high	10.5	7.3	0.03	31	11.9
PS	58	F	9	1	18.9	3.5	149	1.24	0.85	108.3	2.1	high	8.6	6.5	0.33	31	10.5
PS	78	F	6	1	25.7	3.5	156	1.43	0.8	106.5	1.5	high	9.6	3.8	0.03	26	10.1
PS	72	F	6	1	21.3	3.7	109	1.53	0.96	130.1	2.1	high		8.1	0.1	30	10.5
PS	77	M	5	1	21.7	3.5	150	1.42	0.9	96.34	2.1	high	9.6	6.4	0.04	32	9.8
PS	69	F	5	1	21.7	3.9	149	1.23	0.98	103.9	1.3	high	9.6	4.8	0.1	29	11.7
PS	66	M	5	1	21.5	3.9	269	1.39	0.75	150.3	1.5	high	9	7.7	0.2	27	9
PS	64	M	5	1	18.3	3.5	103	1.43	0.92	105	1.8	high	8.3	3.5	11	39	8.1
PS	75	M	5	1	19.9	3.8	192	1.59	0.77	74.53	1.5	high	9.5	5	0.03	21	9
PS	74	F	5	1	24.2	3.6	173	1.32	1.05	132	2.1	high	10.1	4.4	0.13	36	12.7
PS	53	M	5	1	22.1	3.9	115	1.68	0.65	89.66	1.8	high	10.2	7.5	0.21	30	10.6

PS	74	M	4	1	21.9	4.2	152	0.82	0.78	105.7	0.8	high	7.8	11.1	0.6	32	10.2
PS	72	F	4	1	20.8	3.3	199	1.33	0.9	74.71	2.1	high	10.2	4.9	0.09	37	10.3
PS	75	M	4	1	24.6	3.8	142	1.86	0.71	95.35	1.8	high	7.8	4.5	4.1	30	6.4
PS	64	M	4	1	22.6	4	219	1.42	0.76	104.2	1.5	high	7.9	9	0.1	28	8.8
PS	68	M	4	1	19.9	3.6	189	1.61	0.59	101.1	1.8	high	8.4	6.7	0.02	22	11.4
PS	79	M	3	1	16	3.7	155	1.77	0.81	102.6	1.6	high	9.6	5.7	0.38	35	10.6
PS	80	F	2	1	15.4	3.6	168	2	0.73	117.1	1.8	high	9.2	6.2	0.17	26	10
PS	71	M	2	1	18.3	3.2	166	1.61	0.83	108.9	1.8	high	9.1	3.9	0.27	27	10
PS	74	F	2	1	19	4.4	203	1.97	1.09	106.4	1.5	high	8.9	3.1	0.29	27	11.2
PS	58	M	9	1	18.3	3.4		1.12	0.76	84.39	2.1	high	9.5	3.3		35	8.8
PS	59	F	2	1	21.5	3.5	164	1.51	1.16	76.17	1.5	high	9.5	2.8	0.2	32	10
PS	63	M	17	1	16.5	3.7	189	1.34	1.26	99.17	2.1	high		5.7	0.1	13	11.5
PS	62	M	3	1	22.4	3.8	211	1.11	0.96	99.83	2.1	high	8.5	6.3	0.01	30	12.1
PS	64	M	2	1	22.2	3.3	199	1.56	0.73	47.43	1.8	high	9	7.1	0.02	27	11.8
PS	59	M	24	1	24.4	3.5	197	1.25	1.07	78.68	1	high	10.2	3	0.42		7
PS	64	M	9	1	21.6	4.1	148	1.09	0.97	131.8	2.1	high	9	6	0.3	27	11.3
PS	61	M	2	1	27.2	4.3	151	1.89	1.15	93.28	1.5	high	9	6.4	0.16	30	12.9
PS	77	M	4	1	19	3.8		1.62	1.14	111.3	1.8	high	9.4	6.8	1.24	27	10
PS	60	F	29	1	18.5	3.8	117	1.58	0.88	70.2	1.9	high	8.7	4	0.01	28	10.1
PS	52	M	28	1	17.6	4	101	1.52	0.97	98.42	1.3	high		2.8	3.63	38	8.4
PS	64	M	26	1	19.8	3.6	156	1.31	0.82	95.53	1.9	high	8.7	4.3	0.16	28	10.3
PS	51	F	25	1	20.1	3.8	145	1.32	1.17	130.5	2.1	high	9.7	5.1	0.25	35	12
PS	66	F	22	1	19.9	3.5	111	1.38	0.97	120.4	1.9	high	9.1	3.1	0.18	31	11.1
PS	64	M	21	1	18.8	4	173	1.61	0.89	115.1	1.6	high	8.6	5.7	0.08	28	10.7
PS	64	F	17	1	21.2	4	90	1.38	1.01	120.9	1.9	high	9.1	5.1	0.06	35	10.4
PS	61	M	13	1	19.9	4	119	1.33	1.01	93.15	1.8	high	8.4	8.4	0.2	27	9.9
PS	85	M	10	1	17.1	4.3	129	1.81	0.86	84.72	2.1	high		7.9	0.2	18	10.8
PS	60	F	9	1	17.6	3.9	118	1.18	0.74	93.61	2.1	high	8.5	6.1	0.11		8.3
PS	46	M	7	1	23.3	4.3		1.59	0.75	72.22	1.4	high	9.3	5.8	0.11	24	8
PS	62	M	5	1	19.7	4	199	1.67	0.89	120.1	1.8	high		4.7	0	15	9.6
PS	73	M	4	1	25.5	4.5	257	1.74	0.73	75.84	2.1	high	9.8	4.9	0.15	28	10.8
PS	69	F	3	1	17.2	3.7	175	1.32	1.1	128.9	2.1	high		9.6	0.2	17	12.9
PS	49	F	18	1	20.4	4.1	133	1.39	0.89	116.5	1.6	high	8.8	5.9	0.11	33	11.5
PS	48	F	10	1	17.4	4	139	1.21	0.74	96.54	2.1	high	9.1	4.6	0.05	16	9.6
PS	61	F	2	1	20.8	4.4	195	1.48	1.05	104.8	1.5	high		4.6	0.1	18	11.5
PS	72	M	2	1	23.1	3.6	119	1.42	0.73	157.6	1.6	high	6.3	5.2	0.25	41	10.4
PS	67	M	3	1	25.9	4.2	100	1.57	0.8	136	1.8	high	8.9	6.3	0.07	25	10.4
PS	59	M	5	1	18.7	3.8	128	1.24	0.75	134.5	1.5	high	8.6	4.1	0.15	27	9.5
PS	60	M	2	1	24.5	3.7	151	1.73	0.8	123.3	1.5	high		6.8	0.02	31	8.7
PS	81	F	7	1	27.6	4	115	1.42	0.85	136.5	1.5	high		4	1.81	29	12.8
PS	71	F	3	1	19.4	4.1	113	1.39	0.97	113.7	1.3	high	9.1	5.9	0.34		10.9
PS	48	M	3	1	22	4	117	1.16	0.63	113.1	2.2	high	9	8.8	0.45	28	9.9
PS	50	M	2	1	20.5	4	190	1.55	0.89	113.8	1.3	high	9.4	5.9		19	8.7
PS	55	F	3	1	19	4.4	159	1.29	0.89	102.8	1.8	high	7.5	5.7	0.51	24	9.3
PS	52	M	23	1	21.9	4.3	123	1.42	0.97	114.1	1.8	high		4.9	0.2	24	10.5
PS	67	F	7	1	16.8	4.4	162	1.29	0.76	100.8	1.5	high		5.1	0.1	24	10.2
PS	34	M	13	1	24.3	4.4	151	1.29	0.94	117.3	1.5	high	8.3	4.3	0.12	35	9.5
PS	73	M	15	1	21.3	3.7	114	1.45	0.85	120.3	1.5	high		4.6	0.1	25	12.3
PS	54	M	11	1	20.8	4.2	172	1.19	0.83	112.4	2.1	high		4.2	0.1	19	10.6
PS	54	M	4	1	23.2	4	95	1.65	1.02	113.9	1.5	high	10.2	5.1	0.04	28	10.9
PS	60	M	2	1	25.1	4.4	171	1.29	0.56	106.8	2.1	high		8.4	0.2	28	12
PS	52	M	12	1	26.2	3.8	162	1.57	0.74	117.8	1.5	high		5.5	0.1	24	11.3
PS	79	F	4	1	17.8	4.1	172	1.51	0.91	94.56	1.6	high	8.9	5.8	0.45	37	11.8
PS	57	M	3	1	21.6	4.1	130	1.42	0.78	104.8	2.1	high	8.9	4.2	0.06	34	9.5
PS	75	F	7	1	18.1	4	155	1.33	0.76	98.73	2.1	high		5.7	0.1	19	13
PS	54	F	3	1	18.8	3.9	147	1.71	0.75	79.55	1.5	high	10.3	3.9	0.84	23	9.5
PS	65	M	3	1	22.9	4.2	198	1.2	0.83	93.13	1.8	high	8	5.6	3.1	30	10.5
PS	81	F	8	1	16.9	4.3	143	1.47	0.67	110.1	1.5	high		4.3	0.2	25	9.5
PS	52	M	7	1	22.6	3.9	169	1.49	1.12	129.5	1.5	high		6.2	0.3	21	9.6

PS	65	M	4	1	22	4	136	1.28	1.59	78.58	1.8	high	8.6	7.2	0.04	28	11.6
PS	50	M	4	1	20.3	4.2	139	1.77	1.05	137.5	1.8	high	8.4	3.9	0.1	22	8.3
PS	75	M	3	1	19.3	4.2	172	1.65	0.71	64.64	1.5	high		5.6	0.1	22	10
PS	60	M	3	1	19.9	4.3	143	1.63	1.01	87.46	1.8	high	8.9	4	0.1	48	9.7
PS	34	M	2	1	21.6	4.1	144	1.34	1.03	99.32	2.1	high	10.2	5.5	0.06	28	9.3
PS	71	F	4	1	22.7	3.7	137	1.46	0.9	67.65	2.1	high		8.5	0	21	11.5
PS	83	F	2	1	20.3	3.7		1.57	0.87	116.7	2.1	high	9.2	4.8	0.1	19	11.2
PS	65	M	34	1	18.8	3.5	135	0.78	0.89	79.05	1.8	high	8.6	5.8		26	10
PS	53	F	8	1	22.5	4.2	210	2.08	0.86	137.1	2.1	high	9.6	5	0.1	30	9.6
PS	63	M	7	1	21.3	4.1	174	1.36	0.69	112.3	1.8	high	9.7	5.6	0.02	22	9.6
PS	62	M	5	1	24.8	3.8	159	1.52	0.89	100.1	1.1	high	10	5.3		24	14.2
PS	62	F	3	1	23.1	4.3	179	1.46	1.01	148.6	2.1	high		8.3	0	18	12.1
PS	48	F	3	1	36.5	4.2	139	1.51	0.67	45.14	2.1	high		3.8	0.1	49	10.2
PS	43	M	4	1	17	4.1	146	1.46	0.99	97.58	2.1	high	8.4	8.7	0.12	28	9.1
PS	67	M	7	1	19.5	3.8	185	1.16	0.85	141.2	2.1	high	9.4	6.4	0.47	20	10.1
PS	58	M	8	1	22.3	4.4	176	0.92	0.96	129.6	1.8	high		3.7		26	8.8
PS	68	F	9	1	17.9	4.1	147	1.56	1.08	118.7	1.8	high	9.7	5.3	0.05	29	10.5
PS	70	M	7	1	22	4.3	125	1.29	1.11	112.7	1	high	9.5	6.9	0.35		9.8
PS	59	M	9	1	19.3	4.1	111	1.56	1.01	102.8	2.1	high	9.2	5.5	0.1	22	8.9
PS	59	F	10	1	17	3.8		2.07	0.92	81.05	1.5	high	9	4.8	0.09	19	12.5
PS	32	F	2	1	20.6	3.8	137	1.48	0.62	67.48	1.3	high		6.3	0.1	25	9.5
PS	76	F	5	1	21.4	3.5	166	1.71	0.74	108.8	1.5	high		7.3	0	19	9.4
PS	65	M	5	1	20.2	4.2	116	1.68	1.07	106.8	0.8	high		5.5	0.47		9.1
PS	38	F	4	1	19.7	4.3	188	1.55	0.85	72.22	1.5	high		5.5	0.05		10.2
PS	74	M	3	1	21.4	3.8	149	1.52	0.7	104.7	1.6	high	10.5	5.4	0.07	35	8.5
PS	57	M	2	1	20.8	4		1.56	1.02	129.3	1.5	high	9.9	6.1			11
PS	65	M	2	1	16	4.3	121	1.57	1.01	86.92	2.1	high	9.5	5.7	0.03	40	11.4
PS	59	M	2	1	22.8	3.8	221	1.39	0.91	89	2.1	high	9.7	6.1	0.42	29	10.5
PS	68	M	30	1	19.6	3.8	142	1.36	1.29	97.81	1	high	7.5	4.4	2.7	26	9.7
PS	62	M	14	1	17.6	3.9	182	1.46	1.11	125	2.1	high		5.7	0	20	9.2
PS	66	F	13	1	23.8	3.8	236	1.57	1.09	103.1	1.5	high	9.2	5.1	0.72	29	10.8
PS	48	M	13	1	18.4	4.3	125	1.01	1.4	111.8	1.5	high	8.1	2.6	5.21	15	9.4
PS	58	F	12	1	17.5	3.8	280	2.04	1.49	104.5	1.5	high	9.2	4.6			10.8
PS	47	M	10	1	17.8	4.4	172	1.51	0.96	82.7	2	high	8.5	3.3	0.22	28	8.3
PS	58	M	9	1	30.9	3.6	148	1.2	0.79	94.99	1.8	high	9.4	4.6	0.03	22	9.9
PS	51	M	9	1	21.4	3	180	1.66	0.81	91.13	2.1	high	8.9	4.8	0.03	29	10.1
PS	70	F	8	1	24.1	3.6	170	1.74	0.99	108.2	1.5	high		7.6		27	10.8
PS	67	M	7	1	23.4	3.8	156	1.2	1.15	110	1.5	high		2.3	0.2	23	10.4
PS	62	M	6	1	20	4.2	188	1.56	1.15	167.6	1.5	high		2.8	0	19	11.5
PS	37	F	4	1	17.9	3.7	105	1.31	1.11	88.01	1.8	high		6	0.1	26	10.4
PS	60	M	4	1	22.6	4.1	145	1.69	0.87	147.6	1.5	high		4.8	0	21	11.1
PS	69	F	4	1	21.5	3.3	135	1.86	0.96	76.01	1.5	high	9.7	5.3	0.15	26	10.3
PS	64	M	3	1	22.7	4	151	1.56	1.06	131.6	1.5	high		6.2	0.2	27	10.2
PS	54	M	3	1	22.2	4.2	131	1.76	1.02	126.5	1.6	high		3.8	0.22	48	9.8
PS	68	F	15	1	19.6	3.8	102	0.97	0.92	137.5	1.8	high		6.7		27	9.8
PS	74	M	5	1	23.7		100	1.55	1.04	112	1.8	high	10.3	5.4	0.02	57	10.7
PS	67	F	7	1	21.6	4.1	170	1.32	1.13	118.6	1.5	high		5.2	0.58	36	11
PS	66	M	6	1	15.6	3.6	195	0.93	0.63	86.77	1.5	high	9.9	6	0.1	52	11.4
PS	33	F	2	1	19.4	3.8	114	1.33	0.88	119.6	2.1	high	8.8	6.3	0.21	22	
PS	68	M	7	1	21.6		196	1.66	0.9	100.7	1.3	high	8.9	6.2	0.09	27	13.6
PS	63	F	23	1	17.6	3.8	273	1.13	0.94	97.08	2.1	high	9.8	6.3	0.3	28	11.8
PS	65	F	22	1	15.3	4.1	156	1.34	1.03	85.78	1	high	10.1	5.4	0.1	19	10.6
PS	60	M	18	1	17.5	3.9	132	1.19	0.93	98.88	2.1	high	8.6	4.6	0.05	26	9.3
PS	38	M	16	1	26.8	3.9	114	0.84	0.83	90.94	2.5	high		7.8	0.29	20	12.7
PS	64	M	14	1	20.6	3.3	206	1.83	0.83	75.11	0.6	high	9.3	3.9	0.1	23	10.3
PS	66	F	12	1	20.4	3.6	185	1.47	0.8	85.91	1.3	high	8.9	5.9	6.3		9.6
PS	80	F	11	1	19.9	3.3	140	1.24	0.96	85.27	1.8	high	9.3	5.4	0.1	17	10.7
PS	48	M	11	1	19.7	3.6		1.42	1.04	85.54	2.1	high	10.1	6.2	0.1	16	11.9
PS	76	M	10	1	22	4.3	170	1.72	0.98	140.3	2.1	high	9.9	5.8	0.42	43	10.2

PS	77	M	7	1	26.7	3.7		1.26	0.79	134.9	1.5	high		4.7			
PS	34	F	5	1	23.2	3.8	186	1.45	0.93	112.8	1.4	high	10.2	5.9	0.1	15	10.7
PS	60	M	5	1	22.3	3.7	178	1.05	0.72	101.8	1	high	9	4.5	0.05	32	10.7
PS	41	F	4	1	23.7	3.6	156	1.51	0.98	102.4	1.8	high		6.8	0.07	27	9.3
PS	58	M	3	1	18.5	3.3		1.03	0.91	95.43	2.4	high	10.1	6	0.15	32	11.8
PS	73	F	3	1	24.6	3.8	121	0.95	0.87	150.9	1.8	high	9.8	5.1	0.01	36	10.3
PS	40	M	3	1	19.8	4.1	140	0.98	1.01	116.8	1.3	high	9.4	5.3	0.1	25	10.3
PS	82	F	3	1	20.6	3.7		1.3	0.94	127.9	2.1	high	8.8	5.1		36	11.3
PS	74	M	2	1	19.3	4.2		1.3	0.83	77.27	1.3	high	9.9	8.6	0.08	22	10.5
PS	62	F	2	1	27.2	3.6	142	1.67	0.78	59.56	2.1	high	7.7	6		31	9.1
PS	28	M	2	1	28.7	3.7	170	1.64	0.91	105.7	1.3	high	8	4.1	0.2	27	10.5
PS	68	M	10	1	21.8	3.9		1.39	0.72	114.4	2.1	high	8.9	6.9	0	25	10.9
PS	51	M	6	1	18	4.4		1.06	1.12	133.5	1.6	high	7.8	2.5	4.3	20	9.3
PS	62	M	13	1	21.9			1.69	0.79	118.5	1.8	high	8.3	5.5	0.1	23	9.5
PS	58	M	10	1	21.2		261	1.89	0.95	116.8	1.6	high	10.1	7.3	0.05	26	11.7
PS	64	M	5	1	25.6		214	2.02	0.89	106.6	2.1	high	6.6	6.9			
PS	65	F	3	1	29.3		137	1.71	0.92	80.67	1.3	high	8.2	4.7	0.04	28	9.8
PS	63	F	2	1	19.6	3.7	151	1.14	0.63	111.4	1.3	high	8.3	4	1.42	29	12.3
PS	71	F	15	1	23.7	3.9	123	1.71	0.86	142.3	1.5	high	10.2	7.7	0.19	29	11.4
PS	60	M	31	1	17	4	159	1.65	0.85	99.81	1.5	high		3.8		27	11
PS	54	F	8	1	23.3	3.5	185	1.63	0.87	98.45	2.1	high	8.6	7.6	0.12	32	11.7
PS	81	F	8	1	18.1	4	118	1.03	0.73	124	2.1	high	8	6.8		30	11.9
PS	59	M	7	1	19.8	3	203	2	0.53	73.21	2.1	high	9.1	4.7		22	10.1
PS	83	F	5	1	19.6	4.4	207	1.8	0.94	125.9	0.8	high	10.7	4.6	0.26	26	10.8
PS	75	M	5	1	17.8	4.1	120	1.24	0.84	67.62	1.8	high	6.4	5.4	5.4	59	9.4
PS	55	M	4	1	21.5	5	160	1.23	0.66	42.03	1.1	high	9.4	6.2	0.35	28	9.4
PS	60	M	3	1	24	3.4		1.82	0.68	166.9	1.3	high	9.9	4.3	0.08	20	11.1
PS	70	M	2	1	19.7	4.4	147	1.32	0.56	56.37	1.8	high		7.9	0.33	25	9.7
PS	74	F	2	1	23.7	3.6	124	1.48	0.69	70.1	2.1	high		6.4	0.1	32	11.2
PS	53	M	6	1	19.6	4.1	202	1.32	1.05	87.05	1.5	high	10.4	5.7	0.07	23	11.4
PS	74	F	13	1	19.7	3.4	136	0.96	1.01	124.8	1.5	high	8.6	3.9	0.2	19	10.1
PS	69	M	9	1	21.3	4.1	160	1.77	1.05	107.7	2.1	high	9.6	6.1		19	9
PS	54	M	9	1	19.8	3.9	142	1.26	0.69	127.2	2.1	high	10.6	8.4	0	29	11
PS	81	M	7	1	28.7	3.7	143	1.28	0.71	86.49	1.5	high	9	5.6	0.1	30	8
PS	83	M	4	1	23	3.4	111	1.48	0.68	47.49	1.5	high	10.7	4			7.5
PS	80	F	4	1	19.1	3.5	176	1.69	0.95	117.5	1.3	high	8.1	7.1	11.01	24	8.3
PS	73	M	2	1	22.5	3.6	185	1.48	0.9	85.58	1.5	high		5.2		38	12.3
PS	76	F	2	1	21.1	3.8	209	1.2	0.93	59.92	1.4	high	9.2	3.6	0.09	12	10
PS	67	F	14	1	17.4	3.8	189	1.21	1.02	116.3	2.1	high		4.1	0.4	22	8.6
PS	71	M	26	1	23.9	3.7	179	1.93	0.75	90.49	1.6	high	11.3	6.4	0.03	31	11.4
PS	58	M	15	1	20.6	4	146	1.51	1.18	134.4	1.8	high	8.2	7.1	0.1	22	9.5
PS	79	F	12	1	15	3.9	188	1.14	1.05	106.8	2.1	high	8.7	5.4	0.4	31	9.1
PS	65	M	7	1	23.1	3.9	146	1.54	0.82	102.2	0.8	high	9.6	4.9	0.13	16	10
PS	78	F	2	1	23.3	3.8	116	1.8	0.87	111.3	2.1	high		6.1	0	30	10.6
PS	58	M	10	1	22.3	4.1	170	1.22	1.02	150.1	2.1	high		3.9	0.1	33	10.7
PS	59	M	10	1	20	4.1	166	1.24	0.74	125.5	1.8	high	8.5	8.5		26	11.1
PS	60	M	6	1	20	3.4	149	1.82	0.76	103.5	1.8	high	8.1	3.7	0.18	33	9.2
PS	67	M	6	1	19.9	3.9	187	1.2	1.54	102.8	2.1	high	8.3	5.5	0.1	25	9.9
PS	73	M	6	1	18.4	3.6	134	1.27	0.88	116.4	2.1	high	9.1	5.3	0.04	27	10.7
PS	73	F	4	1	20.2	3.9	142	1.76	0.76	102.6	1.5	high	8.3	6.7	0.26	26	11.4
PS	77	F	4	1	18.2	3.6	162	1.49	0.6	96.3	1.6	high	9.3	7.3		30	12.5
PS	68	M	4	1	24.3	3.6	155	1.6	0.83	140.7	1.5	high	8.8	4.7			8.9
PS	62	M	2	1	19	3.8	182	1.51	1.18	119.5	0.8	high	9.1	4	0.02	32	10.2
PS	76	M	2	1	23.5	3.6	160	1.16	0.69	102.6	2.1	high		10.3	0.57	24	10.4
PS	69	F	25	1	23	3.9	116	1.24	1.05	121.7	1.8	high		5.2	0.05	28	8.2
PS	63	F	19	1	20.6	2.2	121	1.13	1.14	76.79	2.1	high		10.9	1.1	25	10.5
PS	72	F	19	1	15.3	2.2	143	1.49	1.06	109.5	1.5	high		4.2	0	24	12.4
PS	54	M	18	1	23.4	3.8	201	1.58	0.73	128.5	1.5	high		4.7	0	25	10.5
PS	58	M	17	1	17.8	3.8	129	1.38	0.84	103.4	2.2	high		5.8	0.96	31	10.1

PS	43	M	17	1	20.3	4.1	188	1.24	0.81	69.44	1.3	high	9.3	4.8	0.23	28	10.1
PS	64	M	15	1	19.5	3.5		1.18	1.23	95.95	2.1	high	9.3	5.1	0.05	25	11.1
PS	76	M	11	1	19.6	3.4	219	1.28	0.89	78.97	2.2	high		6.4	0	30	10.4
PS	48	F	13	1	21.8	4		1.5	1.36	102.7	1.8	high	10.8	5.6	0.06	27	13.3
PS	54	M	11	1	22.9	3.8	138	1.58	1.11	88.23	2.1	high	9.1	5.5	0.06	34	8.7
PS	73	F	10	1	27.4	3.5	115	1.24	1.03	127	1	high		8.5	0.05		10.2
PS	40	M	9	1	20.9	4.6	142	1.37	1.13	114.4	1.4	high	8.1	5.6	0.2	24	8.1
PS	59	F	8	1	20.6	3.1	152	1.28	1.13	117.7	2.1	high		10	1.4	23	10.8
PS	76	M	7	1	22.5	3.5	214	1.9	0.95	83.76	1.4	high	10.2	5	0.16	30	9.5
PS	65	F	7	1	18.8	3.5	183	1.04	1.07	126.3	1.4	high		5.7		30	9.6
PS	70	F	6	1	31.4	3.6	169	1.49	1.11	92.58	2.5	high	9.4	4.6	7.48	27	11
PS	54	M	6	1	22.5	4	104	1.48	1.11	103.7	1.5	high		4.3	0.2	24	10.3
PS	76	M	5	1	22.5	3.6	199	1.69	0.73	96.33	1.3	high	9.5	4.9	0.36	25	10.2
PS	67	F	4	1	21.4	3.9	192	1.6	0.95	72.13	1.8	high	9.8	5.8	0.04	27	10.3
PS	71	M	4	1	19.1	4	139	1.46	0.87	88.43	1.8	high	10	5.4	0.09	30	9.5
PS	68	F	4	1	19.6	3	203	1.52	1.06	86.62	1	high	7.7	7.5	6.31	35	9.1
PS	52	M	4	1	17.9	1.5	211	1.9	1.12	61.86	1.3	high	9.7	7.5	3.32	25	11.6
PS	59	F	4	1	22.7	3.8	169	1.38	1.18	100.8	1.8	high		3.1	0.29	31	11.3
PS	63	M	4	1	30.7	4.2	128	1.31	0.83	86.48	1.3	high	9.7	6.6		24	11.4
PS	56	F	2	1	21.1	4.1	152	1.46	0.86	72.85	1.8	high	9.4	3.9	0.46	54	11.6
PS	72	F	3	1	25.1	3.5	175	1.63	0.95	74.49	1.5	high	8.4	5.9	0.2	22	11.5
PS	65	M	3	1	18.7	4.2	159	1.47	1.03	74.51	2.1	high		5.6	0	41	11.4
PS	61	M	3	1	25.5	3.7	115	1.18	0.96	85.36	2.1	high		3.9	0.02	18	10.4
PS	85	M	2	1	19.9	3.7	119	1.5	1.08	70.01	2.1	high	7.8	6.3	3.12	36	7.9
PS	46	M	2	1	24.7	3.6	142	1.72	0.91	98.17	1.8	high	11	4.6		18	10.5
PS	53	M	2	1	26.5	3.9	153	1.11	0.96	96.63	1.5	high	8.6	6.5		29	9.9
PS	73	F	2	1	23.5	4	142	1.26	1.16	100	1.5	high	11.3	8.3	0.22	44	12.9
PS	67	M	2	1	18.6	2.2	160	1.73	0.75	74.69	1.5	high		6	0.3	29	10.5
PS	65	M	14	1	26.6	4.1	178	1.69	0.83	129.4	2.1	high		6.2	0	41	10.1
PS	78	M	9	1	21.8	3.6	136	1.32	0.82	144	2.5	high	9.9	5.4	0.07	16	10.3
PS	59	F	5	1	21	3.9	99	1.3	1.11	103	1.3	high	7.8	3.3	0	38	8.8
PS	67	F	22	1	18.3	3.8	134	1.18	1.14	126.7	1.8	high	8.4	3.7	0.67	28	10.7
PS	67	M	22	1	18.4	4	102	1.35	1.04	124.8	1.5	high	9.9	6.5		31	8.2
PS	52	F	18	1	18.2	4.2	178	1.22	1.09	119.8	1.4	high	9.6	5.1	0.53	25	9.8
PS	36	M	15	1	18.1	4.1	85	1.48	1.05	124	1.8	high	8.5	5.6	0.16	26	10.7
PS	45	M	13	1	15.8	3.7	114	1.46	1.31	95.88	1.4	high	8.8	7	0.82	36	8.3
PS	61	M	12	1	24.9	3.4	190	1.66	0.81	104	1.5	high	8.9	4.4	0.05	23	9.4
PS	70	M	12	1	22.7	3.9	111	1.04	0.79	128.1	1.4	high	12.1	3.1	0.1	28	9.7
PS	53	M	11	1	20	3.7	178	1.63	1.12	90.24	1.5	high	9.1	4.2	0.05	33	11.2
PS	38	F	9	1	21.7	3	130	1.33	0.93	84.44	1.8	high	8.8	4.5	8.68	30	9
PS	52	M	7	1	23.4	4.4	142	1.26	0.79	124.1	2.2	high	9	4	1.82	26	9.5
PS	53	M	7	1	20.4	4	126	1.47	1.05	122.8	1.5	high	10.2	7.4	0.04	19	10.5
PS	44	M	5	1	22.2	3.8	111	1	0.79	95.81	1.8	high		5.1	0.2	32	10.2
PS	65	F	5	1	22.6	3.9		1.31	0.8	111.2	1.8	high	9.3	5.2		38	15
PS	61	F	5	1	19.1	3.5	151	1.53	1.12	110.2	1	high	8.8	2.8	0.2		10.5
PS	51	F	4	1	25.8	3.7	176	1.36	0.96	108.4	1.5	high	8.3	3.4	0.19		10.7
PS	59	F	4	1	26.4	3.9	108	1.26	0.89	100.7	2.1	high	11.4	7.5	0.01	23	6.2
PS	37	M	4	1	27	4.3		1.54	0.85	92.54	1.5	high	9.2	3.9	0.06	30	12.3
PS	32	M	4	1	23.8	3.9		1.37	0.9	100.8	2.5	high	8.8	5.7	0.09	18	10.6
PS	68	F	3	1	23.5	4.1	208	1.16	0.89	94.69	2.2	high	7.7	9.4	0.6	25	11.7
PS	57	M	3	1	27.6	3.9	178	1.46	0.97	77.44	1.5	high		5.3		24	9.3
PS	67	M	2	1	21.5	4.1	201	1.46	1.04	127.3	0.8	high	9.6	4	0.16	28	9.6
PS	61	M	2	1	19.9	3.8	141	1.35	0.84	126.3	1.8	high		5.2	0.04		10.7
PS	39	F	14	1	18.8	3.4	178	1.16	1.05	115.9	1.8	high	8.5	3.9	0.05	21	9.7
PS	72	F	12	1	19.5	3.5	131	1.25	0.73	95.12	1.8	high	8.6	7.6	1.1	34	
PS	72	M	7	1	19.9	3.6	123	1.46	1.04	149.9	2.1	high	9.8	5.3	0.07	30	10.2
PS	66	M	4	1	22.7	3.9	160	1.29	0.74	98.14	1	high		4.2		32	10.4
PS	66	F	4	1	20.9	3.9	117	1.46	1.07	107.4	2.1	high	10.5	4.9	0.1	32	7.8
PS	49	M	28	1	17.1	4.1	124	1.71	0.77	84.47	1.8	high		6.8	0.62	37	12

PS	57	F	27	1	16.3	4.1		1.33	1.28	89.13	1.4	high	8.6	5.7	0.03		10.3
PS	67	F	24	1	20.1	3.7	111	1.06	0.89	124	1.6	high	8.3	5.2	0.13	26	11.1
PS	71	F	22	1	15.8	3.5	132	1.26	0.83	100.2	0.8	high	10.3	4.8		24	9.5
PS	61	F	21	1	19.2	4	111	1.17	1.01	113	2.5	high	9.8	6.1	0.01	24	11.2
PS	78	M	19	1	21.7	4.1	122	1.23	1.05	122.2	1.8	high		6.1	4.51	40	8.8
PS	66	M	18	1	17.9	3.6	113	1.3	0.85	103.9	2.1	high			0.03		
PS	69	F	17	1	18.2	3.2	102	1.43	0.91	93.1	1.5	high	8.4	4.9	0.3	20	11
PS	44	F	13	1	19.4	4.1	157	1.14	0.8	83.5	1	high		5.2	0.08	39	11.2
PS	29	F	13	1	20.6	4.1	130	1.39	0.97	99.84	1.5	high	8.8	3	1.01	32	8.6
PS	43	F	12	1	27.2	3.9	184	1.56	1.03	129.4	1.6	high	10.2	6.7	0.3	0	10.1
PS	75	M	12	1	22.1	4	137	1.52	0.86	96.53	1.5	high		4.7		24	10.9
PS	59	F	12	1	19.6	4.1	88	2.19	0.97	148.9	1.4	high	7.6	4.1	0.08	31	9.1
PS	59	F	12	1	18.9	3.8	104	2.27	1.09	114.6	1.4	high	7.9	2.7	0.6	27	12
PS	68	M	8	1	22.8	3.9	190	1.74	0.73	97.18	2.1	high	10.3	6	0.05		10
PS	74	M	7	1	21.3	3.6	172	1.75	0.85	119.8	0.8	high	13.8	4.6	0.62	24	11.3
PS	74	F	7	1	21.1	4	128	1.26	0.92	114.4	1.5	high	8	4.9	0.19	27	10.3
PS	62	F	6	1	21.1	3.5	157	1.42	1.15	59.29	2.1	high	9.2	4.5	0.02	26	10.8
PS	48	F	3	1	17.8	3.8	153	1.39	0.91	104.1	2.1	high		7	0.2	21	10
PS	72	M	3	1	23.6	3.9	166	1.63	0.89	91.57	1.3	high	10.7	3.8	0.07	29	11
PS	40	M	2	1	18.4	4.2	136	1.73	0.87	82.87	2.1	high		6.2	0.15	36	10.8
PS	49	F	31	1	16.9	3.5	138	1.72	1.23	83.36	1.6	high	9.9	6.2	0.01	15	9
PS	58	F	31	1	19.3	3.4	153	1.4	1.24	83.9	1.6	high	9.1	7.3	0.01	27	12.8
PS	55	M	28	1	20.7	4	121	1.39	1.21	105.9	1.9	high	9.5	5.6	0.01	18	10
PS	56	F	27	1	28.1	3.7	160	1.13	1.02	87.99	2.1	high		7.1			12.1
PS	71	F	26	1	22.2	3.5	139	1.57	0.75	130.9	1.9	high	9.8	4.4			
PS	55	M	21	1	25.4	3.8	113	1.29	0.89	141	1.4	high	9.4	4.7	0.05	28	11.1
PS	60	M	21	1	22.7	3.5	118	1.51	1.18	124	1.9	high	9.5	4.6			
PS	71	M	20	1	23.2	3.3	203	1.35	0.68	111.3	1.6	high	10	5.4			
PS	72	M	20	1	19.6	3.7	64	1.13	1.12	117.6	2.2	high	8.1	3	1.18		7.2
PS	47	M	18	1	21	4.1	184	2.09	1.01	142.2	1.6	high	9.5	6.4	0.38	36	11.1
PS	64	F	18	1	17.9	3.9	183	1.6	1.04	156.3	2.2	high	8.8	4.3			
PS	46	F	18	1	19.5	3.7	84	1.19	0.94	100.5	2.1	high		6.2	0	23	10.7
PS	76	M	17	1	18.4	3.8	116	1.59	0.82	100.4	1	high		5.7	0.05		9.1
PS	74	F	17	1	20.8	3.9	136	1.16	0.76	121.4	1.8	high	9.9	6.9		27	10.1
PS	79	M	16	1	17.1	3.8	123	1.43	0.78	96.71	1.6	high	9.8	6.3	0.41	31	10.9
PS	57	F	16	1	22.5	4	139	1.5	0.99	135.3	1.4	high	8.6	3.1	0.1	25	12
PS	44	F	16	1	17.5	4	118	1.14	1.03	127.5	2.2	high	8.7	3.6	0.1	23	8.7
PS	61	F	15	1	17.5	4.2	135	1.45	0.92	148.7	1.4	high	9.2	4.3	0.37	24	8.1
PS	69	M	15	1	19.3	3.6	149	1.36	1.22	132.3	1.5	high	7.9	4.9	3.29	27	8.3
PS	57	M	14	1	16.9	3.9	169	1.68	1.1	123.9	1	high	8.4	4.1	0.06		11.2
PS	46	M	14	1	23	4	199	1.52	1.03	71.81	2.1	high		4.4		26	10.7
PS	65	F	14	1	20.7	4	121	1.57	0.98	107.2	1.4	high	9.2	4.9	0.06	17	11.1
PS	50	M	13	1	23.2	3.9	140	1.14	1.1	117.5	1.6	high	9.5	4.7	0.05	22	8.7
PS	63	F	12	1	22.7	3.8	178	1.09	0.82	89.72	1.8	high	9.5	5.2		23	10.6
PS	66	F	11	1	19.6	3.6	155	2.03	0.94	142.4	1.4	high	8.7	3.1	0.2	26	9.2
PS	57	M	11	1	20	3.7	134	1.26	0.92	156.1	1.8	high	9	6		27	11.5
PS	72	M	11	1	21.7	3.9	153	0.96	1	145.7	2.1	high		5.6			11.2
PS	66	M	11	1	22	3.7	171	1.32	1.05	148.1	1.8	high	9.1	3.5		25	9.2
PS	69	F	10	1	17.9	3.8	142	1.79	1.11	141.1	1.4	high	9.1	4	3.69	32	11.1
PS	69	M	10	1	19.1	4.1		1.72	1.21	118.8	1.5	high		5.4			10.1
PS	69	M	10	1	17	3.5	259	1.76	0.95	121.5	1	high	9.4	5.9	0.1	25	12
PS	64	M	9	1	22.6	4.1	130	0.99	0.75	104.5	2.1	high	9.5	7.9	0.09	29	11.3
PS	45	F	8	1	18.7	3.8	173	1.41	0.99	114	1.4	high	9.4	5.6	0.2	22	11
PS	69	M	7	1	21.8	4	158	1.68	0.81	141.3	1.8	high	8.7	4.9	0.2	26	10.4
PS	60	M	7	1	25.3	4	229	1.82	1.1	125.8	1.3	high	8.7	5.1	0.02	21	8.3
PS	62	M	6	1	21.6	3.8	152	1.41	0.71	91.53	1.4	high	8.4	5	0.02	28	9.4
PS	62	M	6	1	25.6	3.6	102	1.18	1.03	118.1	1.4	high	8.5	5.1	0.2	13	9.3
PS	80	M	5	1	21.2	3.6	140	1.45	0.78	119.9	2.2	high	9.7	3.8			10.6
PS	70	F	5	1	22.1	3.6	89	1.4	1.1	123.4	2.1	high	8.8	6.1			

PS	56	M	4	1	25.3	3.7	157	1.24	1.09	154.2	1.4	high	9.3	5.3	0.9	34	10.4
PS	48	M	4	1	17.4	4	141	1.64	0.81	69.64	1.8	high	8.7	5.4			10
PS	79	F	4	1	19.4	3.7	153	1.54	0.83	107.9	1.8	high	9.2	6.2	0.1	22	9.7
PS	76	M	4	1	24.3	3.8	172	1.49	0.74	71.78	1.8	high	9.6	6.4			11.9
PS	54	F	4	1	14.6	3.7	128	1.93	1.13	116.8	1.4	high	8.9	4.9			8.3
PS	67	M	4	1	21.7	3.4	219	1.34	0.89	100.8	1.3	high	9.4	5.5		29	10.5
PS	73	M	3	1	18	3.6	214	1.15	0.92	126.9	1.5	high	8.8	5.1	0.14	23	10.2
PS	65	F	3	1	19.8	3.8	160	1.19	1.04	117.2	1.8	high		7.1			
PS	60	M	3	1	22.4	4.1	174	1.38	1.04	102.1	1.8	high		8.7	0.7	28	14.7
PS	80	M	2	1	19.6	3.6	151	1.51	0.83	101.4	1.4	high	9.3	3.6	0.07	25	10.5
PS	57	F	2	1	22.4	3.7	97	1.44	0.91	80.5	1.5	high	11	3.8	0.4	39	10.9
PS	66	M	2	1	23.2	3.9	127	1.25	1.08	133.6	2.2	high	9.7	6.3		39	11.2
PS	52	F	2	1	21.7	4.2	127	1.27	0.98	106.5	2.1	high	8.8	4.3			
PS	68	M	2	1	18.6	4	229	1.36	0.84	77.49	2.1	high	10.1	8.2		27	10
PS	81	F	2	1	27.1	3.5		1.46	0.89	128.8	1.5	high	8.3	4.2	0.1	25	10.5
PS	61	M	2	1	20.5	3.9	249	0.77	1.04	82.59	0.8	high	8.2	4	0.1	11	12.2
PS	48	M	2	1	25.1	3.5	137	1.54	1.23	92.79	1.1	high	8.6	4	1.14	33	10.1
PS	67	M	9	1	20.8	3.7	177	1.69	0.92	118.1	1.5	high	9.7	6		29	10
PS	71	F	14	1	20.3	3.9		2.08	0.91	155.4	1.4	high		7.5	0.1	36	9.4
PS	52	F	26	1	19.7	4.3	149	1.56	0.35	147.2	2.1	high	9.8	6.2	0.05	25	9.4
PS	71	F	19	1	19.1	4	146	1.18	0.98	126.7	1.8	high	8.9	7.6	0.2	28	10.8
PS	75	F	19	1	16.5	4.6	127	1.15	1.16	112.5	2.1	high		6.7	0.3	13	11.6
PS	64	F	13	1	18.4	4	143	1.3	0.91	105.3	2.1	high	8.9	5.8	0		12.3
PS	54	M	13	1	20.1	4.1	107	1.2	1.02	126.7	1.6	high	9.8	4.2	0.04	31	9.4
PS	77	F	7	1	26.9	4	137	1.6	0.9	127.1	1.4	high	9	3.9	0.06	27	11
PS	59	F	6	1	20.7	4	154	1.53	1.13	117.9	1.5	high	9.2	4.9	0.06	30	10.6
PS	63	F	6	1	23.2	4.2	222	1.69	0.99	110.2	1.3	high	8.8	5.3	0		9.7
PS	62	M	5	1	18	4.3	106	1.38	0.94	106.4	1.8	high	8.3	5.1			10.5
PS	66	M	5	1	22.5	4.2	151	1.12	1.05	105.5	1.8	high	9.5	6.8		32	12.5
PS	68	F	5	1	25.9	3.8	130	1.75	1.11	129	2.1	high	7.8	4.7	0.21	30	10.4
PS	74	M	4	1	18.4	3.8		1.27	1.15	107.7	1.8	high		4.6	0.15	32	10.2
PS	81	M	4	1	21.3	4	194	1.66	0.94	119.1	1.5	high		6.4	0	19	11.4
PS	50	M	2	1	21.6	4.5		1.3	1.18	84.5	2.1	high	8	7.5	1.05	33	9.2
PS	60	M	23	1	19.8	4.1	143	1.22	1.1	83.17	2.2	high		6.9	0.13	31	9.8
PS	67	F	22	1	21.1	4	215	0.95	0.87	112.3	1.4	high	8.8	4.9	2.17	27	10.9
PS	62	M	22	1	21	3.9	215	1.46	0.73	122.6	1.4	high		3.8	0.14	26	10.9
PS	69	M	17	1	19.5	4	97	1.47	0.83	108.2	1.8	high	9	3.1	0.1	29	10.4
PS	58	F	15	1	23.5	4.4	119	1.48	1.49	120	2.2	high	8.4	5.7	0.1	28	12.9
PS	49	M	10	1	20.8	4.2	148	1.49	1.12	136.4	2.2	high	8	3.6	0.1	16	10.4
PS	66	F	10	1	19.2	3.7	161	2.29	0.78	150.3	1.4	high	8.8	6.2	0.1	23	8.8
PS	73	M	9	1	24.6	4.4	170	1.63	0.71	123.9	1.5	high	6.7	4	0.08	27	8.3
PS	64	M	7	1	21.7	2.8	115	1.64	0.49	25.21	1.8	high	9	4.1			10.9
PS	73	F	6	1	22.6	3.6	111	1.31	1.03	141.9	2.1	high		5.4			11.2
PS	56	M	4	1	18.3	4.1	177	1.49	0.78	70.04	1.6	high	8.1	4	0.08	28	10.3
PS	58	M	3	1	21.6	3.6	105	1.66	1.05	133.3	1.4	high	8.5	3.5	6.96	31	10.6
PS	49	F	7	1	20.9	4.1	112	1.44	1.11	118.3	2.2	high	8.6	6	0.17	25	12.7
PS	58	F	32	1	19.2	3.3	117	1.22	0.85	88.13	2.1	high	7.5	5.1	0.08	24	11
PS	54	F	25	1	26.5	3.9	162	1.22	0.82	87.93	1.8	high	9	6.9	0.05	31	10.7
PS	69	M	16	1	21.7	3.4	144	1.32	0.88	138.7	1.4	high	9.2	5.9	6.72	34	10
PS	26	F	11	1	17	4.3		1.12	1.32	113.3	1.8	high	9.6	3	0.6	15	9.9
PS	54	F	14	1	18.9	4	168	1.36	1.03	105.3	1.8	high	8.7	4.2	0.42	28	
PS	72	M	14	1	20.3	3.6	177	0.89	1.12	108.9	1.4	high	9.4	3.4		38	10.1
PS	77	M	14	1	19.2	4	121	1.11	0.92	106.2	2.1	high	8.4	7.8	0.04	25	11.9
PS	60	M	14	1	21.2	4.1	145	1.65	1	106.9	1.4	high		3.9		31	10.1
PS	71	M	13	1	16.8	4	130	1.56	1.06	92.72	2.1	high		5.7		20	9.8
PS	67	M	12	1	18.3	3.5	182	2.07	0.91	107.2	1.5	high		4.4		29	9.9
PS	47	F	12	1	18.4	4.3	154	1.55	0.73	120	1.4	high		2.8			11.1
PS	46	M	12	1	17.3	4.5	203	1.52	0.91	88.11	1.5	high	8.7	6.5	1.27	25	9.4
PS	78	M	10	1	22.7	3.5	165	1.44	0.99	132.4	1.4	high		4.9		26	9.7

PS	77	M	10	1	19.1	4.2	232	1.38	0.87	130.9	1.5	high	9.2	6	0.08	22	6.2
PS	73	F	10	1	22.2	4.1	203	1.02	1.03	131.6	2.2	high		4.6	0.61	25	11
PS	43	M	9	1	26.3	4.1	129	1.57	1.12	88.27	2.1	high	8.3	6.1	0.12	39	11
PS	68	M	9	1	20.7	3.9	146	1.55	0.78	124.8	1.5	high		8.7		26	10.5
PS	61	M	9	1	21.8	3.9	149	1.35	0.86	132.7	1.8	high	10.4	5.7	0.09	31	9.5
PS	54	F	9	1	15.7	4.3		1.49	1.08	134.2	1.8	high	9.1	4.3	0.1	25	11
PS	54	F	9	1	14.7	4.3	164	1.02	1.33	86.43	1.8	high	7.7	2.8	0.23	25	11.5
PS	44	F	8	1	18.3	4	155	1.03	1.06	88.09	1.3	high	8.7	4.7	0	22	9.9
PS	29	M	8	1	17.8	4.1		1.76	0.97	114.6	1.8	high	9.1	4.6	0.1	28	9.8
PS	83	M	7	1	16.8	4.1	186	1.09	1.12	153.5	1.8	high	8.5	4.4	0.28		7.9
PS	53	M	7	1	25.3	4	172	2.03	0.82	106.1	1.5	high	10.2	5	0.08	25	11.6
PS	51	F	7	1	20	4.3		2.02	1.26	113.5	1.8	high	9.3	6.9	0.08	29	10.3
PS	53	M	6	1	30.5	4.2	187	1.24	0.89	78.39	1.6	high	9.2	7.5	0.31	21	10.5
PS	59	M	6	1	20.8	4.3	151	1.94	1.12	130.9	1	high	9.7	5.7	0.27	27	9.6
PS	78	F	6	1	16.6	4		1.17	1.16	140.8	1.8	high	8.4	7.7	0.26	24	8.9
PS	70	M	5	1	21.5	3.6		1.51	0.86	108.5	1.8	high	8.4	5	0.22		12.2
PS	56	M	5	1	23.6	3.9	125	1.77	0.95	113.8	1.5	high	8	3.4	0.04	27	8.4
PS	71	F	4	1	18.6	4.1	102	1.49	0.99	97.61	1.5	high	8.4	4.2	0.05	40	10.1
PS	52	M	4	1	23.1	4.6	171	1.52	0.78	53.27	1.5	high	8.6	6.8	0.17	0	10.8
PS	69	F	3	1	20.6	3.5	160	1.2	0.74	94.17	1.6	high		5.3	0.05	18	8.5
PS	67	F	2	1	19.2	4	139	1.21	0.69	87.19	1.8	high	8.9	4.9	0.11	25	11.6
PS	56	M	2	1	22.3	3.7	158	1.83	0.82	122	1.6	high	10.1	7.7	0.05		
PS	50	F	2	1	25.9	4.2	181	1.69	0.97	102.5	1.5	high	9.3	5.6	0.12	28	9.6
PS	65	M	2	1	19.8	3.8	111	1.34	0.76	104.2	1.5	high	9.9	6.9	0.12	30	11.3
PS	69	F	28	1	21.1	3.8	177	1.24	0.86	112.5	1.8	high	8.2	5.8	0.19	31	11.3
PS	53	M	22	1	19.6	4.2	152	1.85	0.84	97.94	2.1	high	9.4	6.9	0	20	12.2
PS	60	F	15	1	18.4	4.1	125	1.29	0.68	119.8	1.8	high		4.5	0.26		11.6
PS	65	M	13	1	20.6	4.4	240	1.2	1.2	132.8	1.8	high	8.6	5.8	0.02	31	10.1
PS	72	M	13	1	20.9	4	98	1.5	0.84	112.3	1.8	high	8	3.5	0.14	39	11.9
PS	71	M	12	1	18.6	3.9	113	1.26	0.76	83.2	1.3	high		3.3	0.08	44	10.8
PS	58	M	11	1	21.4	4.2	161	1.5	0.88	135.6	1.3	high	8.8	7.2	0.17	34	10.9
PS	64	F	10	1	20.9	4.2	157	1.34	1.12	122.2	1.5	high	9.2	3.6	0.33	27	13.1
PS	67	F	8	1	20.3	3.9	171	1.17	1.05	121.7	1.3	high	9.4	4.9	4.97	28	13.3
PS	75	M	8	1	24.9	3.9	176	1.03	0.77	118.8	1.1	high	9.6	3.2	2.02	43	10.8
PS	57	F	6	1	19.9	4.1	130	1.51	1	73.31	1.5	high	10.3	7.9	0.05	43	7.4
PS	71	M	6	1	22	4.2	181	1.55	1.19	133.5	0.8	high	8.8	4.8	0.12	18	10.9
PS	73	F	4	1	24.4	3.7	136	1.41	0.74	78.82	1.3	high		1.9	0.63	31	8.3
PS	79	M	4	1	17.3	4.3		1.58	0.85	110.9	1.8	high	9.8	5.7	0.29	26	9.8
PS	60	M	3	1	21.8	4.2	170	1.66	0.92	90.26	1.5	high	8.5	5.8	0.02	24	12.6
PS	68	M	3	1	23.2	4.1	163	1.13	0.86	123.5	2.1	high	9.8	5.4	0.05	23	10
PS	52	F	3	1	18	4	105	1.34	0.96	104.5	1.5	high	9.5	2.9	0.17	23	11
PS	65	F	2	1	21.6	4	171	1.58	0.99	86.03	1.8	high	9	6	0.02	33	11.4
PS	64	M	24	1	20.9	3.8	240	1.56	0.79	110.9	1.8	high		3.7	0.12	29	10.2
PS	60	F	23	1	16.4	3.7	133	1.65	0.9	97.37	1.4	high	9.5	3.6	3.09	19	8.8
PS	59	F	21	1	23.5	3.5		2.16	1.09	139.5	1	high	8.7	4.8	0.21	24	11.1
PS	35	F	18	1	18.9	4	155	1.26	0.76	95.46	1.8	high	8.8	7.7	0.15	44	10.6
PS	81	F	16	1	16	3.3	94	1.3	0.91	77.22	2.1	high	9.3	6.6		25	11.4
PS	64	M	16	1	21.9	3.8	149	1.63	0.79	100.3	1	high	9.6	3	1.25	28	8.5
PS	35	F	12	1	19.2	3.8	219	1.42	1.34	118.5	1.4	high	10.5	5.7	4.17	29	10.2
PS	59	F	12	1	18.3	3.8	190	1.82	1.08	94.75	1.1	high	9	5.8	3.3	28	10.7
PS	64	F	10	1	17	3.7	111	1.37	1.03	146	1.8	high	8.5	5.3	0.26	27	9.7
PS	64	M	9	1	19.3	3.4	189	0.99	0.87	102.4	1.8	high	6.8	4.5	0.2	25	9.9
PS	74	F	7	1	15.8	3.2	134	1.46	0.83	92.7	1.3	high	9.4	5.5		29	9.1
PS	69	F	6	1	20.8	3.6	112	1.36	0.83	85.26	1.5	high	9.3	3.9	0.02	34	10.7
PS	78	M	6	1	22.5	3.7	187	1.35	1.04	119.2	2.2	high	10	7.1	0.36	26	12.7
PS	71	F	6	1	18.4	3.6	172	1.28	0.93	133.8	1.8	high		6.8			12.9
PS	60	F	6	1	16.4	3.5	113	1.56	0.8	76.36	1.1	high	9.8	6.7		19	9.6
PS	65	M	4	1	19.4	3.7	126	1.15	0.61	112	2.2	high	9.5	5.7	0.2	35	9.8
PS	84	M	3	1	20.4	2.8	154	1.96	0.45	98.77	1	high	8.7	5		28	10.5

PS	67	M	3	1	23	3.5	153	1.01	0.89	89.57	1	high	8.5	6.3	2.37	32	9.3
PS	50	F	3	1	21.4	3.9	101	1.07	0.89	97.8	1.8	high	8	6.3	1.66	29	7.8
PS	65	M	2	1	18.9	3.5	215	1.45	0.93	110.3	1.6	high	8.4	6.7	0.36	30	11.6
PS	65	M	2	1	22.8	3.9	198	1.37	1.05	91.2	2.1	high	9.1	2.7	0.05		10.8
PS	71	M	28	1	17	3.7	113	1.6	1.21	126.3	1.4	high	9.9	5.6	0.18	33	8.6
PS	71	F	25	1	22.3	3.8	150	1.07	1.27	112.1	1.8	high	8.6	2.7	4.21	35	9.4
PS	62	F	24	1	18	4.2	177	1.46	0.93	92.79	2.5	high	9.6	6.5	0.4	19	10.3
PS	38	M	23	1	15.4	4	167	1.59	0.95	102.7	1	high	9.6	5	0.07	26	11.6
PS	70	F	22	1	15.9	3.8		1.28	0.89	102.4	1.4	high	8.4	4.7	0.04		11
PS	53	F	22	1	22.8	4.2	180	1.48	0.93	118.7	1	high	10.1	4.2	0.2	30	9.3
PS	58	M	21	1	21.1	4.3	186	1.44	1.2	139.1	1.4	high	7.7	2.1	0.09	28	9.5
PS	62	M	21	1	22.8	4.4	133	1.51	0.65	98.48	1.4	high	9.4	4.6	0.08	25	10.1
PS	70	M	21	1	23	3.9	117	1.25	0.88	121.2	2.2	high	9.8	6.7	0.05	18	9.6
PS	61	M	20	1	21.5	4.1	104	1.58	1.29	119.6	1	high	9.5	4.8	0.42	26	10
PS	66	M	17	1	21.6	4.3	140	1.17	0.82	163.2	1.8	high		6.2		29	9.1
PS	65	F	17	1	25.8	3.9	165	1.44	1.1	97.99	1.8	high	8.4	4	0.5	32	10
PS	60	M	12	1	18.9	3.8	92	1.58	0.94	110.4	2.2	high	10.4	5.2		27	10
PS	61	F	11	1	17.8	4.2	128	0.95	1.05	125.7	2.2	high	8.7	3	4.49	26	10.8
PS	46	M	11	1	19.2	4.6	135	1.12	0.91	117	1.6	high	9.4	5.2		23	9.9
PS	55	M	8	1	27.3	4.2	177	1.36	1.11	115.9	1.5	high	8.5	4.7	0.1	31	10.1
PS	41	M	7	1	21.2	4.3	124	1.24	0.68	65.94	1.8	high	8.6	4.3	0.31	23	9.1
PS	29	M	7	1	17.4	4.2	107	1.34	0.96	130.5	1.8	high	8.6	4.7	0.09	25	10.5
PS	63	M	5	1	20.1	3.7	152	1.62	1.03	145.3	1.3	high		4.2	0.83	30	10.6
PS	54	F	5	1	20.2	4.3	143	1.99	1.05	121.5	1.4	high	9	5	7	27	10.4
PS	60	M	3	1	20	3.8	135	1.29	0.83	106.7	1.4	high	8	5.4	0.43	36	10.7
PS	71	M	16	1	24.1	3.7		1.49	1.04	96.31	1.8	high	9.1	4.7	0.62	32	8.2
PS	57	F	14	1	17.7	4.1	115	1.44	0.94	89.78	1	high	8.7	4.9	0.56	43	10.6
PS	57	M	13	1	22	3.7	156	1.35	1.21	117.2	1.8	high		5.3	0.05	20	9.4
PS	80	M	10	1	20.4	3.4	175	1.57	0.96	135.9	1	high	8.9	4.8	0.11	23	10.5
PS	50	M	7	1	17.6	4.3		1.57	0.93	76.63	2.5	high	9.4	5.3		25	
PS	64	M	7	1	19.5	3.4	132	1.54	1.3	54.56	2.2	high	8.4	4.7	0.1	20	10.8
PS	74	M	6	1	24.9	3.7	133	1.47	0.61	98.74	2.2	high	7.9	3.4	0.1	19	9.9
PS	65	M	6	1	22.8	4.1	134	1.78	1.15	128.3	1.4	high	7.7	3.9	0.12	34	12.3
PS	76	F	6	1	22.1	3.9	171	1.36	1.01	136.3	2.2	high	9.4	5.8	0.1	18	10.4
PS	71	M	5	1	22.1	4	177	1.59	0.85	105.7	1	high	8	7	0.1	29	10.6
PS	50	F	4	1	21.4	3.9	126	1.51	0.93	97.16	1.8	high	8.8	4.4	0.1	30	11.4
PS	35	M	3	1	17.1	4.3	125	1.56	0.68	92.02	2.1	high	8.8	6	0.26	33	9.5
PS	59	F	2	1	20.3	4.6	166	1.11	1.07	99.57	1	high		7.8	0.23		7.5
PS	69	M	14	1	21	4.3	189	1.22	0.85	118.2	1.4	high	9.3	3.9		11	11.2
PS	63	F	6	1	17.8	3.9	128	1.73	0.97	61.77	1.3	high		4.3	0.02	34	10.3
PS	50	F	9	1	17.5	4.7	137	1.22	1.15	117	1.4	high	8.9	3.5	0.9	29	9
PS	73	M	7	1	20.1	3.6	98	1.54	0.96	109.1	1.5	high	9.4	4.7	1	31	9.1
PS	67	M	5	1	20.5	3.8	92	1.48	0.73	95.03	2.1	high		5	0.2	16	10.3
PS	61	F	6	1	19	4	106	1.51	1.27	123.6	1.5	high	8.6	4.3	0.05	24	10.2
PS	64	F	14	1	26.1	4.3	124	1.02	1.06	119	1.8	high	8.9	7.1		24	9.9
PS	71	F	11	1	15.9	3.8	125	1.41	1.31	120.6	2.2	high	9.4	4.6	0.11	20	11
PS	61	F	3	1	22.7	3.6	185	1.45	0.92	82.05	1.4	high		6.8		29	9.9
PS	52	M	2	1	23.5	3.7	188	1.36	0.98	115	1.5	high	8.9	6.6	0.09	35	10.3
PS	61	M	5	1	17.6	3.9	172	1.67	0.98	64.27	1.3	high	8.4	3.3	0	26	10.7
PS	63	M	2	1	24.5	4	176	1.15	1.12	98.46	1.8	high	8.9	7.3	0.6	30	15
PS	61	M	5	1	19.2	3.8	132	1.63	1.36	111	2.2	high		6.3	0.39	29	9.9
PS	65	F	11	1	21.5	3.9	156	1.23	0.84	104.6	2.1	high	8.7	6.5	0.18	34	10.4
PS	57	M	6	1	27.2	4.2	148	1.58	1.08	135.2	1.5	high		6.1	0.56	37	10.6
PS	40	M	6	1	32.5	4.4	138	1.62	0.75	139.4	1.5	high		7.2	0.2	21	10.8
PS	48	M	5	1	23.2		168	2.16	1.28		1.8	high	8.7	3.5	0		10.9
PS	61	M	5	1	25.1	4.4	179	1.32	0.99	108.6	1.5	high		10.5	0	22	10.9
PS	61	F	5	1	17.8	4	152	1.28	0.97	92.18	2.1	high		5.7	0	19	10.2
PS	60	M	4	1	21.8	4.3	166	1.3	0.75	73.63	2.1	high		6	0.5	16	10.9
PS	71	M	2	1	19.3	4.4	171	1.49	0.73	80.11	2.1	high	8.5	3.8	0.11	14	11.5

PS	55	M	33	1	17.9	4	171	1.29	0.84	99.43	2.1	high		5.7	0.31	24	10.1
PS	60	F	28	1	17.4	3.8	124	1.28	1.03	114	1.6	high	9.9	4.8	0.6	25	12
PS	72	M	27	1	24.1	3.4	141	1.87	0.83	145.6	1.6	high	9.1	4.5	0.2	34	9.2
PS	70	M	24	1	20.5	3.1	228	1.43	0.9	119.6	1.5	high	8.7	5.6		29	11.6
PS	69	M	4	1	21.7	3.8	161	1.34	0.76	86.47	2.5	high	9.2	5.5	0.23	41	8.7
PS	64	F	22	1	19.3	3.6	170	1.13	1.06	110.4	1	high	8.6	3.7		19	11
PS	73	F	21	1	23.2	3.8	106	1.39	0.95	126.2	1.4	high	9.3	4.5		25	9.6
PS	68	M	19	1	24.6	3.9	140	1.45	0.82	150.1	2	high	9	5.9		36	10.2
PS	53	M	19	1	18.8	4	210	1.73	0.85	126.1	1.5	high		4	0.05	21	9.4
PS	74	F	19	1	18.6	3.8	209	1.95	0.92	137.3	1.3	high	8.5	4.5	1.42	24	10
PS	59	M	18	1	24.2	3.7	131	1.53	0.83	114.9	2.1	high	9.1	5.2	0.16	16	11.5
PS	62	M	18	1	21.2	3.5	199	1.55	0.97	101.8	1.4	high	8.9	4.5		32	
PS	69	F	17	1	18.3	4	165	1.34	0.97	130.9	1.5	high	8.6	4.6	0.03	24	10
PS	37	M	18	1	19.6	4.2	172	1.89	0.96	95.5	1.5	high	8.8	4.3	0.03	30	11.4
PS	37	M	25	1	19.8	4.3	150	1.42	1.11	104.7	1.8	high	10.3	5.3	0.16	20	10.3
PS	67	M	17	1	19.7	3.3	174	1.64	0.83	129.8	2.1	high	9.3	6.8	0.22	24	10.9
PS	56	M	17	1	20.1	3.9	190	1.23	0.9	92.84	2.1	high		6.7	0.04	28	10.7
PS	53	M	16	1	21.5	3.9	113	0.99	0.95	153	2	high	9.1	6.7	0.08	23	11.2
PS	62	M	16	1	21.1	3.9	103	1.35	1.07	89.6	1.5	high	9.7	3.2			10.9
PS	64	M	16	1	22.4	3.7	129	1.52	0.83	144.4	1.8	high	9.6	6.6	0.19	25	11.1
PS	71	F	13	1	27	4.2	163	1.06	0.72	97.24	1.5	high	10.2	5.4	0.05	26	9
PS	74	M	10	1	23.1		145	1.73	0.71	106.1	1.9	high	9.7	5.7	0.01	16	10
PS	66	F	16	1	23.1	3.8	159	1.55	1.09	123	1.4	high	8.3	5.6	0.1		10
PS	76	F	15	1	19.3	4.5	154	1.49	0.93	139.7	1.5	high	9.2	6.4	0.37	30	13
PS	65	M	15	1	17.3	3.6	146	1.32	0.83	149.9	1.8	high	9.6	5.2		30	
PS	54	M	15	1	22.4	4.2	135	1.6	0.78	111.3	2.1	high	9.3	3.6	0.18	25	8.9
PS	59	F	27	1	24.5	3.9	130	1.35	0.76	138.6	1	high	10.5	4.8	0.05		9.7
PS	84	M	15	1	19.2	3.7	203	1.2	0.82	133.3	1.6	high	8	6.3		16	10
PS	65	M	15	1	15.4	3.9	100	0.97	1.08	137.1	1.8	high		6.4	2.5	20	9.6
PS	48	M	15	1	19.3	3.6	129	1.52	0.86	109.9	1.8	high	9.6	4.3	0.13	32	9.7
PS	65	F	14	1	20.5	3.9	243	2.09	1.03	133.7	1.5	high	9.3	3	0.05	25	10.4
PS	72	M	14	1	20.7	3.8	182	1.44	0.94	145.5	1.8	high	9.4	4.9	0	27	
PS	57	M	14	1	21.5	4	153	1.31	0.98	127.6	1.5	high	9.8	7.7	0.21	28	10
PS	88	M	14	1	19.6	3.6	163	1.48	0.8	107.9	1.8	high		6.1	0	29	12.5
PS	55	F	14	1	21.4	3.7		1.25	0.97	109	1.8	high	7.8	5.7			9.2
PS	61	F	17	1	20.5	3.7	162	1.21	1.01	119.9	1.3	high	9.7	4.6	0.07	29	10
PS	47	M	13	1	20.7	4.2	107	1.38	0.85	107.5	1.5	high	9.7	7.2	0.22	27	10.9
PS	64	F	13	1	20.3	3.9	163	1.02	1.13	126.9	1.4	high	7.5	2.4	1.17	26	8.2
PS	68	M	21	1	17.5	3.9	128	1.29	1.11	139.4	2.1	high	7.2	9.6	0.05	30	10.5
PS	62	M	12	1	19	4.2	116	1.22	1.08	121.3	1.4	high	8.3	6.6	0.45	24	11.6
PS	54	M	12	1	17.7	3.9	95	1.49	0.75	121.1	1.8	high	10.2	5.7	0.05	25	10.4
PS	71	M	12	1	18.7	3.4	206	1.13	0.72	159.9	2.1	high	8.1	5.6	0.13	27	11.6
PS	62	M	12	1	21.8	3.8	112	1.66	0.51	134.7	1.5	high	8.2	4.8	0.19	21	11.3
PS	51	F	12	1	19.3	4	131	1.19	0.95	79.91	1.5	high	9.1	3.4	0.14	21	10
PS	66	M	12	1	22.1	3.9	139	1.54	0.98	127.9	1.5	high	8.7	6.1	0.12	26	10.6
PS	68	M	12	1	20.5	3.9	156	1.27	1.04	152.5	1.5	high	9.4	5.1	0.09	24	9.5
PS	61	M	11	1	17.2	4	149	1.14	1.04	119.6	1.8	high	9.2	4.6	0	24	10
PS	84	M	11	1	17.2	3.8	139	1.56	0.96	110.5	1.6	high		4.4	0.2	27	10.9
PS	64	M	10	1	19.6	3.9	162	1.83	1.1	121.9	1.5	high	8.9	3.2	1.62	20	7.2
PS	31	M	10	1	20.2	4.2	120	1.62	0.92	110.8	1.5	high	8.4	5	0.11	30	10.3
PS	58	F	10	1	24.4	4.1	154	1.12	0.79	110.7	1.3	high	10	6.7		33	12.2
PS	53	M	10	1	19.8	3.8	159	1.22	0.7	99.69	1.5	high	10	4.5	0.03	26	9.9
PS	42	M	10	1	24.8	3.9	101	1.17	0.92	112.5	2.1	high		4.8	0.5	18	9.4
PS	68	F	9	1	18.6	3.9	154	1.47	0.81	126.4	1.5	high	7.4	3.1	2.9	41	7
PS	69	M	9	1	22.9		165	1.23	0.85	157.8	1.8	high	10	4.9	0.27	27	9.8
PS	68	M	9	1	22.4	3.8	112	1.33	0.77	126.2	1.8	high	9.8	5.4		28	10.9
PS	49	M	9	1	18.4	4	131	2.05	1.17	129.2	1.6	high	8.7	5.3	0.13		11.1
PS	55	M	9	1	22.7	4	185	1.48	0.88	129.1	2.1	high	8.4	6.3	0.05	17	10.7
PS	58	M	9	1	22.1	3.5	131	2.45	0.76	104.8	1.5	high	11	4.6		28	12.6

PS	69	F	9	1	17.8	4	175	1.43	1.41	125.3	1.8	high	8.6	6.2	2.28		12.1
PS	59	M	9	1	21.3	3.8	111	1.32	0.94	124.5	2.1	high	8.6	6.9	0.16	31	11.4
PS	47	M	9	1	21.5	4.1	188	1.13	0.94	128.7	2.1	high	8.7	7.7	0.28	26	10.8
PS	68	F	8	1	18.8	4.1	237	1.21	1.1	114	1.8	high	8.9	7.2	0.35	28	10.5
PS	51	M	8	1	22	3.9	92	1.65	0.89	115	1.3	high	8.7	3.1	0	28	10.2
PS	65	M	8	1	24.8	3.7	161	1.72	0.73	119.4	1.8	high	8.4	4.7	2.75	20	8.2
PS	68	M	8	1	17.1	3.9	119	1.05	0.78	107.1	1.5	high	8.5	5.3	0.1	23	
PS	64	M	8	1	20.5	4.2	219	1.45	0.92	118.1	2.1	high	9.1	3.3	0.05	25	9.1
PS	64	F	8	1	16.8	3.9	141	1.89	1.12	143.4	1	high		5.7	1	40	11.1
PS	56	F	7	1	20.1	3.6	175	1.4	0.8	128.7	1.8	high	9.1	6.5	0.44	24	12.3
PS	65	M	7	1	22.8	3.9	78	1.23	0.7	143.6	1.8	high	8.7	6.2		32	10.6
PS	57	F	7	1	20	4	81	1.22	1	142.2	2.2	high	9.3	5.9	1.18	31	9.7
PS	47	M	7	1	27.3	4.2		1.57	0.72	112.8	1	high	9.4	2.9	0.56	31	10.1
PS	55	M	7	1	33.5	4	138	1.16	0.82	127.1	1.6	high	10.4	6.2			10.2
PS	71	F	7	1	24.1	3.6	157	1.68	1.03	119.8	1.5	high	8.6	4.2		24	9.6
PS	55	M	6	1	23.9	4	122	1.65	0.88	128.8	1.5	high	8.5	7.2	0.04	27	10.3
PS	59	M	6	1	22.5	3.5	199	1.12	0.77	126.4	1.5	high	8.5	5.2	0.1	22	10.8
PS	66	F	6	1	20.3	3.8	129	1.54	0.94	127.4	1.5	high	10.4	7.3			10
PS	72	M	6	1	21.9	3.7	112	1.12	0.73	131.3	1.8	high		4.2	0.3	31	9.7
PS	37	F	6	1	20		143	1.19	1.04	121.2	1.5	high	9.1	4.3	0.17	31	11.6
PS	56	M	6	1	19.2	4.2	135	1.43	0.96	145.6	1.5	high	9.6	5.9	0.16	30	11.1
PS	50	M	16	1	22.2	3.9	85	1.29	0.91	144.3	1	high		5.2	0.1	24	9.2
PS	64	F	6	1	22.2	4.2	133	1.17	1.01	100.2	2.1	high	9.2	4.8	3.9	28	7.8
PS	52	F	5	1	20.6	4	146	1.54	0.98	90.6	1.8	high	9.6	6.6	0.24	27	10.7
PS	73	M	5	1	19.7	3.5	97	1.02	0.93	69.99	2.1	high		7.1	0.2	29	11.1
PS	52	F	5	1	29.6	4	151	1.9	0.75	113.6	1.6	high		7.4	1.9	29	9.8
PS	49	M	5	1	24.4	3.8	107	0.98	0.92	111.8	1	high	9.2	5.2		22	
PS	67	M	5	1	20.4	3.8	114	0.88	1.23	115.9	1.8	high		3	1.5	30	8.1
PS	60	M	5	1	22.8	4	144	1.33	0.93	118.5	1.8	high	10.2	6.2	0.21	30	10.8
PS	66	M	5	1	22.8	3.7	256	1.38	0.72	119.9	1.5	high	8.3	4.9	0.1	19	10.5
PS	67	F	5	1	25.6	4.1	171	1.46	0.96	138	1.5	high	10.1	7.2	0.15	32	10.4
PS	56	M	4	1	21.7	4.1	149	1.45	0.95	107	1.3	high	8.5	4.9	0.2	30	10.1
PS	70	F	4	1	27.5	3.8	115	1.3	1.04	91.94	1.5	high	8	5		26	9.4
PS	52	M	4	1	34.6	4.2	102	1.27	0.69	97.45	1.4	high	9.2	7.3		30	
PS	60	M	4	1	27.3	4.1	126	1.37	0.71	127.6	1.5	high	8.8	6.2	0.12	25	10.5
PS	63	M	4	1	23.1	3.6	186	1.26	0.86	86.24	1.6	high	9.3	7.6	0		
PS	80	M	4	1	18.3	3.7	171	1.56	0.73	91.97	1.5	high	8.9	5.5		25	10.7
PS	64	M	4	1	25.3			1.24	0.82	91.61	1.8	high	8.1	7		27	10.9
PS	45	M	3	1	36.2	3.8	141	0.83	0.75	117.5	1.3	high	9	8.4	0.2		9.3
PS	81	F	3	1	22.9	3.8	121	1.06	1.04	95.28	1.1	high	8.4	6	0.1	24	10.4
PS	49	M	5	1	21.6	4.5	112	1.53	0.93	133.5	1.5	high	8.6	4.9	0	31	11.8
PS	76	M	5	1	26.5	3.9	154	1.5	0.84	85.52	1.5	high	8.8	5.8	0.05	34	13
PS	60	M	2	1	21.8	3.7	212	1.52	0.9	124.8	1.5	high	9.1	7	2.86	18	10
PS	87	F	2	1	18.5	3.6	163	1.79	0.74	81.94	1.9	high	9.1	4.3	0.04	19	9.8
PS	64	F	2	1	18.9	3.8	122	1.73	0.89	74.53	1.8	high	8.4	4.2	0.46		10.3
PS	79	F	6	1	22.8	4.2	166	1.48	1.11	140.7	2.1	high	9.3	1.6		23	10.1
PS	61	M	27	1	24	4	93	1.45	0.99	104.1	1.6	high		2.7			9.4
PS	63	M	9	1	23.8	4.2	110	1.14	1.17	122	2.2	high	9.6	4	7.75	17	10.2
PS	54	M	7	1	21.8	4.4	117	1.3	1.02	113.6	2.1	high		4.8	0.1	18	10.5
PS	48	M	3	1	23.2	4.5	209	1.77	0.71	88.63	1.5	high	8.6	7.2	0.89	40	12.2
PS	63	F	3	1	19.9	3.9	124	1.14	0.8	98.15	1.8	high	8.7	4.2	0.19	21	9.9
PS	47	M	3	1	21.1	4.2	132	1.41	0.89	96.61	2.1	high		5.5	0.06	28	12.1
PS	66	M	20	1	18.5	3.7	170	1.35	0.78	102.9	1.6	high	9.5	8	0.1	31	9.8
PS	54	F	18	1	18.1	3.6	178	1.36	0.99	92.12	2.1	high	9.1	4.7		29	10.8
PS	60	F	12	1	18.9	3.6	179	1.57	0.66	94.13	1.5	high	9	3.7		31	9.1
PS	72	M	7	1	19.4	4.1	119	0.9	0.85	110.9	2.1	high	8.9	5.5			
PS	35	M	6	1	19.5	4.1		1.51	0.7	71.78	2.1	high	9.9	5.1	0.11	22	11.8
PS	59	M	5	1	21.3	4	142	1.39	0.85	105	1.5	high	8.2	3		26	10.1
PS	77	M	2	1	20.7	3.3		1.23	0.68	49.01	1.3	high	8.1	5.8	0.05	26	11.7

PS	64	F	14	1	22.7	3.7	203	1.55	0.96	114	1	high	10.3	5.2		22	10.2
PS	59	M	6	1	25.4	3.8	128	1.49	0.74	93.12	1.3	high	8.1	5.6	0.5	35	11.1
PS	79	M	2	1	19.6	4.2	188	1.04	0.8	88.96	1.6	high		5.8	0	33	14.4
PS	54	M	28	1	21.2	3.9	174	1.66	0.83	111.8	1.3	high	8.3	4.2	0.21	26	9.6
PS	37	M	7	1	21.8	4.5	164	1.7	1.2	105.7	2.1	high	8.4	7.3	0.1		12.2
PS	64	M	5	1	19.2	4.2	154	1.47	0.86	109.3	1.8	high	9.9	5.7	1.08	25	8.9
PS	74	M	5	1	22.8	3.8		1.46	0.72	115.9	2.1	high	9.1	4.2	0.1	25	10.9
PS	74	M	20	1	23.2	3.7	102	1.45	0.67	83.7	2.1	high		4.5	0	20	8.9
PS	42	M	23	1	16.4	3.9	84	1.83	1.3	116.9	1.8	high	8.5	4.9	0.21	32	12.2
PS	74	F	23	1	21	3.8	154	1.23	0.67	92.33	1.6	high	9	4.9	0.05	19	11.6
PS	67	M	12	1	18.4	4	132	1.33	1.17	116.6	1.4	high	7.9	8	2.81	27	6.7
PS	62	M	12	1	22	4.4		1.92	1.14	123.7	1.3	high	8.8	7.5		18	10.9
PS	48	M	9	1	21.9	4.4		1.68	0.92	91.74	1.8	high	10	5.1		26	9.2
PS	60	M	7	1	21.5	4.1	174	1.31	1.05	106.4	1.8	high	8.6	5.1	0.44	17	8.9
PS	44	M	7	1	13.6	4	157	1.56	1.11	97.87	1.3	high	9.1	3.2	0.16	35	8.6
PS	60	M	4	1	26.6	4	203	1.26	0.81	156.8	1.8	high	7.2	4.5	0.44	19	10.6
PS	54	M	2	1	21.8	4.3	157	1.34	0.69	71.82	1.5	high	10.3	5.7	0.02	27	7.5
PS	51	M	2	1	22.8	4.4	113	1.08	0.9	103.9	2.1	high	9.8	8.4	6.8	37	10.4
PS	84	M	3	1	17.6	3.7	107	1.83	0.76	83.26	1.3	high		3.8	5.1	32	9.3
PS	35	F	14	1	18.6	4	195	1.78	1.08	108.8	1.8	high	9.5	6.6	0.15	27	10
PS	60	M	11	1	18.8	3.7	180	1.32	0.87	120.2	2.1	high	10.1	5.5	0.1		10.9
PS	61	F	3	1	21.3	3.9	103	1.53	0.99	108.6	1.5	high	9.8	6.3	0.15	26	10.2
PS	81	M	5	1	16.6	1.9		1.51	0.8	84.51	2.1	high	9.6	3.5	0.18	28	9.7
PS	81	F	5	1	17.7	2.5		1.62	1.18	112	1.6	high	9.8	5.8	0.03	22	10.8
PS	74	M	2	1	21.4	4.3	120	1.52	1.01	87.81	1.3	high	9.3	3.3	0.67	20	11.9
PS	50	F	16	1	20.6	3.9	143	1.54	1.28	124.2	1.8	high	8.2	5.6	0.1	21	10.9
PS	60	F	4	1	17.5	4	162	1.52	0.84	110.4	1.4	high	9	3.9	9.29	32	11
PS	61	M	18	1	22.5	4.1	82	0.88	0.86	119.7	1.8	high	8.3	5	0.23	22	8.4
PS	65	F	13	1	16.6	3.8		1.7	0.94	135.4	1.8	high	9.7	4.3		19	
PS	60	M	13	1	17.2	3.7	209	1.52	0.98	104.2	1	high	9.4	4.9	0.6	45	9.3
PS	61	M	12	1	20.3	4	142	1.63	1.03	122.6	1.4	high	7.6	4.2	0.05	23	10.8
PS	41	M	12	1	22.3	4.4		1.51	0.75	68.98	1.8	high	8.9	6.6		23	10.4
PS	73	M	8	1	23.9	4		1.29	0.92	107.6	2.1	high	9.6	7.6		21	12.3
PS	74	M	6	1	16.5	3.9		1.21	0.74	124.8	2.1	high	9	4.3		24	11.1
PS	42	M	6	1	29.5	4.2	180	1.65	1.09	106.5	1.6	high	8.5	6	1.14	30	10.3
PS	54	F	6	1	19.5	4.2		1.34	0.76	123.5	1.8	high	8.1	5.5		21	9.9
PS	61	M	5	1	23.9	4		1.8	0.82	110	1.5	high	8.7	6.2		20	9.9
PS	60	M	4	1	18.9	3.8		1.17	1	99.73	2.1	high	7.2	5.7		24	11.2
PS	51	M	3	1	23.1	4.3	102	2.1	0.75	46.82	1.5	high	9.6	5.9	0.52	26	11.6
PS	46	M	2	1	17	4.1	173	1.51	0.89	68.82	1.6	high		6.4	0	21	8.5
PS	59	M	2	1	19.3	4.2	168	2.42	1.1	113.5	1.3	high	9.1	4.2	0.05	18	11.1
PS	68	M	25	1	18.3	2.9	257	1.64	0.88	100.8	2.1	high	9.4	5	0.1	24	10.4
PS	53	M	9	1	35.1	4.1		1.29	1.05	88.19	1.8	high	8.2	9		26	10.7
PS	60	M	31	1	19.1	3.9		1.77	0.94	84.93	1.4	high	8.7	5.1	0.16		11.3
PS	49	M	30	1	19.9	4.5	214	1.62	0.97	94.84	1.8	high	8.2	4.8	1.23	23	9
PS	49	M	27	1	20.3	3.9	131	1.18	1.1	133.2	1.7	high	8.4	6.3	0.07	24	9
PS	70	M	24	1	21.9	4.1	149	1.36	0.99	133.3	2.2	high	7.8	5.8			10.7
PS	62	F	23	1	17.6	3.6	133	1.75	0.59	73.35	1.9	high	10.4	5.9	0.2	35	13.9
PS	62	M	22	1	22.4	5.1	150	1.52	0.82	103.5	1.9	high	11.3	6	0	31	9
PS	62	M	21	1	22.5	4.2	147	1.16	1.02	128.2	1.3	high	8	3.7	0	26	8.9
PS	68	M	21	1	24.2	3.5		1.86	0.68	65.68	1.3	high	9.7	4.9	0	28	9.4
PS	61	M	20	1	20	3.8	167	1.25	1.01	129.3	1.8	high	9.3	6.4	0.1	26	11.5
PS	53	M	20	1	20	4.4		1.58	0.95	123.7	1.3	high	9.1	4	0.1	27	9.5
PS	38	M	19	1	16	4		1.3	1.17	108.7	2.1	high	10.2	3.9	0.05	22	9.9
PS	57	M	19	1	23.3	3.9	129	1.41	0.96	131.3	1.6	high	9.2	5.8	0.13		10.7
PS	53	F	18	1	20.9	3.9	167	1.66	1.2	129.1	1	high	8.9	4.9	0.1	19	12.3
PS	65	M	18	1	20.2	3.4		1.71	0.93	119.6	1.8	high	10.1	7	0	17	9
PS	63	M	16	1	23.1	4.2	120	1.29	1.06	108.3	1.5	high	8.6	5	0.02	21	11.2
PS	68	F	16	1	17.1	4.2	201	0.81	1.2	102.4	1.4	high		6.9	0.26	24	8.6

PS	61	M	15	1	19.7	4	148	1.26	0.96	126.4	1	high	9.7	6.5	0.1	27	12.7
PS	49	M	15	1	19.2	4.2	189	1.66	0.91	113.7	1.4	high	9.3	6	5.33	22	8.7
PS	64	M	13	1	18.9		129	1.19	0.51	83.98	1.8	high		5.7	0.38	26	8.9
PS	64	F	13	1	18.6	3.8	157	1.34	1.01	89.48	1.8	high	9.2	4.3	0.1	22	11.6
PS	44	M	11	1	21.6	4	97	1.07	1.09	149.6	1	high	7.7	3	1.75	44	7.7
PS	57	M	11	1	20.5	3.9	146	1.43	1.08	120.3	1.5	high		6.9	0.2	21	12.5
PS	65	M	10	1	23.2	3.8		1.27	0.88	130.4	1.8	high	9.5	6.2		18	12.4
PS	68	F	9	1	23.1	3.8	111	0.82	0.83	128.1	1	high		5.2		26	11.3
PS	57	F	8	1	26.7	4.1		0.95	0.97	117	1.8	high	9.2	7.4			8.4
PS	34	M	7	1	20.7	4.3		1.85	0.96	127.7	1.8	high	9.1	5.4	0.05	26	11.3
PS	55	M	7	1	20.8	4.1	136	2.07	1.07	117.4	1.8	high		3.7	0.01	29	11.3
PS	55	M	7	1	20.6	4.1	201	1.24	1.02	141	1.8	high		5.9	1		10.6
PS	38	M	6	1	18.1	4.3	216	1.41	1.06	133	2.1	high	8.5	6.6	0.29	30	10.3
PS	73	M	6	1	19.7	3.8	126	1.49	0.86	134.4	1.8	high		3	0.1		10.1
PS	60	M	5	1	21.3	3.3	97	1.67	0.94	115.6	1.8	high	11.4	3.6	1.49		10.1
PS	66	M	4	1	25.1	4	149	1.41	0.85	126.4	2.1	high	7.6	5.3	0.8	34	9.9
PS	46	M	4	1	33	4.1	144	1.45	0.83	103.8	1	high		5.4		26	10.1
PS	62	F	3	1	22.9	4.3		1.43	0.87	138	2.1	high	8.5	8.6		26	10.4
PS	65	M	2	1	21.6	4.3	112	2.16	0.94	112.1	1.4	high	8.7	3.2	1.4	25	9.3
PS	57	M	2	1	24.9	3.9	110	1.36	0.83	134.1	1.8	high	8.4	7.2		30	12
PS	78	F	2	1	24.8	3.2	109	1.39	0.82	104.8	1.8	high		5.3		25	7.5
PS	60	M	2	1	20.6	4.3	231	1.24	0.97	121	2.1	high	8	4.1		29	10.7
PS	74	F	2	1	25.2	3.8	215	1.59	0.63	77.09	2.1	high	9.7	6.4	0.04	19	11
PS	52	F	26	1	23	4.2	136	1.08	0.99	126.2	1.6	high	8.6	7.1	0.63	30	10.8
PS	61	F	24	1	26.3	3.8	189	1.53	1.18	133.9	2.2	high	9.7	5.6	0.06	20	9.1
PS	53	M	18	1	18.8	4.4	98	1.41	1.05	102.7	1.4	high	8.1	3.9	0.23	23	9.1
PS	57	M	16	1	19.4	4	156	1.71	0.94	122.9	1.5	high	9.2	4.2	1.62		11.7
PS	69	F	16	1	17.1	3.8	213	1.7	0.89	115	1.4	high	8.7	6.3			9.7
PS	61	M	15	1	30.2	4.3	131	1.58	1	108.2	2.1	high		8	0.1	23	12.6
PS	65	F	15	1	20.7	3.8	199	1.8	0.96	94.61	1.8	high	9.1	4.8		40	
PS	48	M	10	1	22.8	4.3		1.33	0.93	132.9	1.4	high	9	4.8	0.23	33	12.1
PS	74	M	10	1	25.3	4.3	124	1.43	1.05	117	2.2	high	9	7.3	0.1	31	10.4
PS	48	M	10	1	18.8	4.4	216	1.33	1.02	129.5	1.3	high	8.4	3.2	0.09	27	10.9
PS	56	M	9	1	21.4	4.5	154	1.5	0.92	134.5	1.5	high	8.7	5	0.09	25	10.7
PS	61	M	9	1	22.6	4.4	169	1.58	1.08	156.7	1	high	8.5	2.9	0.12	22	9.5
PS	66	F	8	1	19.2	4	162	1.53	1.46	94.88	1.8	high		4.7	0	27	9.2
PS	67	M	7	1	20.8	3.7	186	1.41	0.78	107.9	1.6	high	10.1	6	0.21	31	10.5
PS	56	F	6	1	24.5	3.9	127	0.74	1.06	90.66	2.1	high		4.5	4.1	22	8.1
PS	69	M	6	1	24.8	3.7	151	1.42	0.73	100.2	1.8	high	9.8	5.3	0.5	27	10.3
PS	61	M	5	1	19.8	3.7	140	1.53	0.9	86.66	1.8	high	8.4	5	0.04	21	11
PS	71	M	5	1	21.3	4.4	160	1.45	0.89	105.2	1.8	high		6.2	0.15	26	10.4
PS	34	M	5	1	17.5	4.1	113	1.82	1.08	79.97	1	high	8	4.6	1.79	42	9.4
PS	59	M	4	1	21.7	4.2	167	1.11	0.94	128.8	1.4	high		3.9	0.55	23	9.8
PS	34	F	3	1	20	4.6	161	1.47	0.95	113.8	2.1	high	8.6	9.2	0.12	38	12.2
PS	82	M	2	1	19.6	3.9	141	1.15	0.73	88.94	1	high		3.9	0.1		8.7
PS	58	F	2	1	18.8	4.2		1.8	1.19	115.4	1.8	high	8.6	5.1	0.09	25	10.3
PS	55	F	34	1	20.7	3.8	151	1.34	1.01	79.34	1.4	high		5	0.09	25	8.9
PS	58	F	31	1	21.1	3.8	117	1.3	0.99	115.1	1.8	high	10.8	4.5	0	43	10.3
PS	61	F	26	1	20.7	3.9	169	1.51	1.24	114.2	1.4	high	9	3.4		44	10.2
PS	51	F	24	1	22.2	3.8	168	1.54	0.94	110.4	2.1	high	8.4	11.6	0.02	20	9.4
PS	56	M	20	1	19.7	4	182	1.39	0.98	130.1	1.5	high	9.3	3.5	0.97	16	11.1
PS	62	M	19	1	21.4	3.5	122	1.14	1.05	112.1	1.4	high	7.3	4.9	3.3	35	
PS	40	M	19	1	18	3.9	188	1.31	0.92	103.7	1.9	high	9.1	5		25	11
PS	56	F	16	1	23.2	3.9		1.89	1.11	102.5	1.8	high		4.4			9.3
PS	62	M	15	1	22.6	3.5	197	1.53	1.06	119.2	1.6	high	9.4	4.7	0.04	25	11.8
PS	55	F	14	1	20.8	3.6	138	1.71	1.06	102.6	1.4	high	8.2	4.1	1.6	25	10.8
PS	53	F	14	1	17.7	3.9	199	2.09	1.24	137.3	1.4	high	9.5	4.3	0.09	35	11
PS	50	M	12	1	19.7	3.6	209	1.81	0.78	108.3	1.3	high	9.8	2.3	0.09	30	8.9
PS	83	F	11	1	20.7	3.1	150	1.55	0.91	109.5	1.1	high	8.8	5.6			

PS	43	M	9	1	22.1	3.6	150	1.56	0.93	124	2.1	high	8.5	4.9	0.14	33	9.6
PS	69	M	9	1	21.8	3.5	138	1.41	0.69	111	1	high	8.5	3	0.05	25	6.5
PS	60	M	8	1	24.4	4		2	1.04	127.8	1.4	high	9.6	3.2	0.1		11.9
PS	73	F	5	1	26.8	3.6		1.36	0.78	102.9	1.8	high	9.6	4.7	0.04	29	12.7
PS	71	F	5	1	19.8	4.1	156	1.29	0.88	84.39	2.1	high	8.8	5.6	0.72	34	9.2
PS	55	M	5	1	22.4	4.3	156	1.35	1.01	100.3	1.8	high	9.4	5.3			11.7
PS	70	M	4	1	21.8	3.3	134	1.93	0.88	106.7	1	high	8.5	3.7	0.1	33	9.8
PS	62	F	4	1	20.5	3.7	166	1.76	1.14	143.4	1.4	high		3	1.02	25	10.8
PS	56	M	3	1	20.1	3.7	176	1.41	1.18	110.9	2.2	high	8.9	5.4	0.4		6.9
PS	58	F	3	1	22	3.7	154	1.45	1.04	119.6	1	high	8.7	3.6	2	28	10.7
PS	56	M	4	1	19.1	3.9	161	1.38	0.73	58.5	1.5	high	8.5	4.7	3.69	33	8.5
PS	61	M	23	1	20.5	4.8	153	1.48	1.06	102.7	1	high	8.5	4.9	0.1	25	9.4
PS	62	F	21	1	16.2	4.3	154	1.26	0.99	108.5	1.5	high		4		28	10
PS	65	F	16	1	23	4.2	151	1.51	0.97	137.6	1.5	high		9	0.4	40	11.1
PS	53	M	15	1	19.2	4.5	178	1.27	0.9	84.22	2.2	high	8.2	6.8	0.07	21	11.2
PS	62	F	14	1	17.9	4.7	144	1.45	1.31	104.9	1.5	high	9	7.4	0.27		12.3
PS	55	F	11	1	28.2	4.1	162	0.96	0.83	106.1	1.4	high	7.9	5.8	0.1	16	11
PS	65	M	11	1	21.4	4.1	102	1.08	0.93	119.9	2.2	high	9	7.6	0.18	21	9.1
PS	48	M	11	1	33.3	4.6	127	1.45	0.73	104.8	1.8	high	9.5	4.2	1.88	25	7.9
PS	74	F	10	1	19.9	3.9	211	1.82	0.96	131.6	1.5	high		3.8	0.4	18	11.9
PS	42	M	9	1	18.2	4.5		1.72	0.84	99.97	1.8	high	8.9	5.8	0.05	29	10.4
PS	72	M	9	1	25.3	3.8	111	1.92	0.67	82.53	1.5	high	8.8	4.5	0.04	17	10
PS	53	M	8	1	19.2	4.3	137	1.08	1.04	112.9	2.2	high		7.2	0.28	29	11.2
PS	58	M	8	1	17.2	4.4	149	1.17	1.01	94	1.5	high		4.2	2.7	14	10.4
PS	69	M	8	1	21	3.6	216	1.91	0.79	105.5	1.1	high	8.6	7.2	0.66	25	11.2
PS	60	M	7	1	21.8	3.9	114	1.61	0.87	127.1	2.1	high	8.6	5.9	0.8	28	10.2
PS	53	F	7	1	27.6	4.8	132	1.43	1.14	107.7	1.3	high	11.2	5	0.34	35	9.7
PS	57	M	4	1	23.7	4.9		1.68	0.86	104.3	2.1	high	8.3	3.9	0.3	37	10.9
PS	68	M	3	1	21	3.9	168	1.31	0.74	97.1	2.1	high	8.1	6.7	0.14	28	10.4
PS	44	F	3	1	22	4.1	127	1.23	1	102.6	2.1	high	9	4.2		29	11.3
PS	76	M	2	1	17.2	4.1	200	0.86	0.97	83.36	1.4	high		5.4	0.3	18	11.5
PS	64	M	11	1	20.3	3.7	172	1.4	0.79	107.3	2.5	high	9.5	8.7	0.1	25	10.1
PS	68	F	24	1	18	3.5	192	1.32	1.27	98.54	1.4	high	8.8	2.6	0.1	32	8.9
PS	55	F	37	1	16.9	3.6	232	1.75	1.09	95.81	1.6	high	8.5	5.8		20	12.1
PS	59	M	32	1	17.7	3.5	235	1.5	0.83	111.1	1.9	high	9.3	4.3			
PS	59	M	29	1	22.6	3.9	131	1.38	0.89	81.2	1.9	high	8.6	6.4	0	27	10.3
PS	60	F	29	1	21.7	3.8	152	2.37	0.87	89.32	1.6	high	11	4.1	0.23	27	9.4
PS	62	F	26	1	17.4	3.5	186	1.32	0.91	102.7	1.6	high	8.3	5.1	0.14	40	9.6
PS	51	M	26	1	17.6	3.8	121	1.16	0.91	129.4	1.8	high		4.9	0.11	23	12
PS	43	M	25	1	21.2	3.8		1.78	0.96	98	1.4	high	9.5	3.9	0	37	11.2
PS	46	F	23	1	20.6	3.6	161	1.7	1.25	122.2	1.4	high	10	4.4		19	8.6
PS	75	F	19	1	19.5	3.7	138	1.39	1.09	116.1	1.8	high		5.5	0.05	25	9.5
PS	61	M	19	1	18.1	3.8	161	1.69	1.2	123.7	1.9	high	7.7	2.1	0.07		10.5
PS	69	M	13	1	17.4	3.9	122	1.22	0.97	135.8	1.4	high	8.8	4		19	10.9
PS	78	F	7	1	16.8	4	125	2.38	0.94	141.1	1.4	high	7.7	3.3	0.26	26	12.5
PS	60	F	6	1	20.1	3.7	153	1.02	1.11	116	1	high	10.1	6.6		20	9.6
PS	70	F	2	1	24	3.5	110	1.25	0.95	103.7	2.1	high	9	5.9	0.01	16	11.4
PS	79	F	2	1	21.2	3.5	146	2.02	0.83	77.93	1.4	high	9.7	4.3	0.13	36	9.9
PS	74	F	7	1	20.7	3.8	197	2.22	0.83	153	1.4	high	9.1	5.9	0.05	23	11.6
PS	71	F	5	1	22.8	3.7	142	1.47	0.87	111.5	1.3	high	9.2	4.3	0.03	30	12.9
PS	57	M	2	1	20.7	3.6	111	1.03	0.79	111.2	1.5	high		5	0.22	29	10.8
PS	55	F	26	1	17.2	4	218	1.28	1.3	134.7	1.4	high	9.2	8.6	0.03	17	11.8
PS	29	M	17	1	20.9	4.6	132	1.54	1.1	78.46	1.8	high	8.6	6.3	0.67	29	10.9
PS	47	M	16	1	20.6	4.2	215	1.48	1	124.3	1.8	high	9.2	3.8			12.8
PS	62	M	16	1	18.7	3.9	180	1.73	0.83	127.2	1.6	high	9	5.4	0.04	31	9.5
PS	39	M	14	1	26.2	4.1	162	1.3	0.93	107.4	1.6	high	8	4.3	0.1	31	8.9
PS	44	M	24	1	13.5	3.9	169	1.16	0.59	99.91	2.1	high		5.7	0.4	11	10.8
PS	78	F	24	1	15.3	3.2	122	1.43	0.54	61.94	1.5	high		4.9	0	21	10.8
PS	73	F	23	1	18.6	3.7	127	1.3	0.48	70.05	2.2	high	7.5	5.3	0.14		11.3

PS	70	M	20	1	16.1	3.8	178	1.84	0.73	112.3	1.9	high	8.5	5.6	0.08	16	10
PS	67	F	20	1	17.6	4	135	1.34	0.95	142.9	2.1	high		7.4	0.6	16	10.6
PS	56	M	20	1	24	4.3	224	1.8	0.78	79.01	1.6	high	8.2	4.4		27	11
PS	51	M	20	1	18.5	4.5	123	1.07	0.97	92.77	2.1	high		4.6	0.4	22	13.7
PS	58	F	20	1	24.9	3.9	116	1.09	0.88	126.4	1.4	high	8	4			9.7
PS	42	M	15	1	14.7	3.8		1.45	0.68	86.66	1.8	high		4.2			10
PS	61	F	15	1	27.8	4	119	0.98	1.02	118.7	2.1	high		6.7	0.1	32	10.3
PS	52	M	15	1	17.7	4.1	165	1.32	0.89	82.69	2.1	high	9.2	5.8	0.12	23	10
PS	58	F	15	1	20	4.2	130	1.41	0.77	127.4	1.8	high		4.6	0.1	18	10.8
PS	67	M	15	1	20.9	4.4	157	1.2	1.41	123.6	1.4	high	9.5	5.9		47	11.4
PS	63	F	14	1	20.8	4.2	135	0.66	0.88	109.2	2.1	high		4.9	0.8	10	11.2
PS	55	F	14	1	21.6	4		1.4	0.84	101.2	2.5	high	9.6	8.5	0.1	21	13.1
PS	70	F	13	1	21.5	3.7	142	1.34	0.78	129.1	2.1	high		6.2	0.1	18	12.7
PS	57	M	13	1	19.3	4.4	200	1.4	0.78	95.14	1.5	high		4.9	0.1	14	10.4
PS	71	M	12	1	19.2	4	189	1.45	0.71	85.71	2.1	high	9.5	5.6	0.57	29	11.4
PS	57	F	11	1	31.2	3.8	181	1.67	0.86	104.1	2.1	high	9.7	10.1		22	8.9
PS	59	F	11	1	25.2	4.2	209	1.65	0.85	98.81	1.5	high		5.7	0	16	11.5
PS	60	M	11	1	25.2	3.4	128	1.58	0.76	97.22	1.8	high	9.7	3.9	0.01	33	8.8
PS	58	M	10	1	29.5	3.3		1.5	0.59	114.3	2.1	high	9.1	2.8	0.63	26	8.1
PS	53	M	10	1	23	4.3		0.99	0.83	103.8	1.5	high	7.5	3.4		24	12
PS	74	F	9	1	23.2	3.9	123	1.25	0.78	110.3	1.8	high		4.9	0.29	24	10.6
PS	66	M	9	1	20.8	4.1	127	1.15	0.97	104.8	2.1	high		6.7	0.1	16	11.1
PS	76	M	9	1	16.3	3.7	153	1.76	0.94	207.4	2.1	high	10.9	5.6	0.1	22	9.5
PS	27	M	7	1	26.3	4.6	117	1.29	1.22	104.3	0.6	high	9.7	2.6	1.1	29	7.5
PS	58	M	7	1	24.3	4.1	135	1.38	0.73	83.55	1.1	high	8.9	3.8	0.09	31	11.8
PS	52	F	6	1	18.7	4.3	137	1.45	1.12	97.51	1.4	high	8.5	4.5	0.03		9.9
PS	54	M	5	1	25.9	4.5	171	1.6	0.79	112.5	1.8	high	9.4	5.1	0.05	29	11.5
PS	50	F	5	1	26.8	4	194	1.19	0.9	106.7	2.1	high	8.6	6	0.1	31	10.8
PS	80	F	5	1	22.8	4	216	1.32	1.06	99.41	1.8	high	9.2	6.7		30	9.7
PS	71	M	4	1	25.2	3.8	145	1.36	0.81	92.54	1	high	7.5	4.8	0	23	8.4
PS	79	F	4	1	19.8	3.7	120	1.23	0.84	111.8	1.4	high		6			12.1
PS	58	M	4	1	20.2	4.4	164	1.22	0.91	92.25	1.4	high		5.1		14	11
PS	61	M	4	1	21.5	4.5	140	1.66	0.75	79.34	0.6	high	7.6	3	0.7	31	11.3
PS	86	F	4	1	15.5	4.6	135	1.44	0.93	103.2	1.8	high	10.1	6.4		31	12.7
PS	80	M	4	1	19.5	3.5		1.94	0.66	45.92	2.1	high	8.9	7		23	
PS	56	F	4	1	23.7	3.8	124	1.15	0.87	92.73	2.1	high		5.5	0.1	16	12
PS	79	M	4	1	26.2	3.8	99	1.06	1	112.6	1	high	7.9	4	0.9	40	11.3
PS	68	F	4	1	22.5	4	187	1.57	0.64	61.32	1.5	high	9.5	6.2	0.5	25	13.4
PS	86	F	4	1	16.7	3.8	134	1.21	0.93	155.3	2.1	high	9.9	6.1	0	29	12.3
PS	65	M	4	1	26.8	3.5	155	1.6	0.64	121.7	2.1	high	9.4	5.6	0.01	25	9.8
PS	72	M	4	1	21.5	4	154	1.43	0.86	127.1	1.8	high	9.9	6.5	0.11	28	12.7
PS	46	M	3	1	27.3	4.1	191	1.72	0.83	90.03	1.8	high	8.7	4.1			10.7
PS	68	M	3	1	29.7	3.7	141	1.37	0.94	81.59	2.1	high		4.9	0.02		11.1
PS	64	M	3	1	21.8	4	176	1.86	0.93	90.07	1.8	high	8.9	7.2		24	11
PS	55	M	3	1	19.8	4.2	145	1.37	0.96	89.4	1.8	high	9.1	7		21	11.9
PS	55	M	3	1	23.5	4.2	141	0.76	0.72	92.39	1.8	high	10.6	6.5	0.04	28	11.3
PS	61	M	2	1	24.1	4.2	150	1.43	1.04	122.9	1.8	high	10.9	6.2	3.8	31	9.1
PS	49	M	2	1	21.7	4.1	160	2.13	0.76	82.35	1.6	high	7.9	4	0.2	29	9.3
PS	77	M	2	1	21.6	4	222	1.69	0.87	73.6	1.8	high	9.2	5.6		20	11
PS	79	M	2	1	22.4	3.9	154	1.15	0.86	115.8	1.4	high	9	5.4		25	10.5
PS	41	M	2	1	21.6	4.7	141	1.46	0.87	118.1	2.1	high		4.9	0.02	31	11.6
PS	61	F	2	1	19	4.2	125	1.02	0.76	88.25	2.2	high	9.4	4.9	0.37	21	10.9
PS	50	M	2	1	26.9	4.2	102	1.3	0.85	108.4	2.2	high	9.6	7.4	0.08	26	10.9
PS	60	M	2	1	19.7	4.4	150	1.27	0.84	76.65	1.8	high	7.8	4.9			9.9
PS	70	F	2	1	21.2	4.1	133	1.55	1.01	100.7	1.8	high	8.7	5.7		33	10.9
PS	47	F	2	1	28.5	4.1	139	2.29	0.76	83.62	1.3	high	8.8	6.8	0.6	25	9.2
PS	58	F	13	1	24.7	3.9	141	1.58	1.19	108.7	1.5	high	9	7.3	0.3	27	11.8
PS	60	F	3	1	16.1	3.7	233	1.09	1.01	56.11	2.1	high	9.6	7.6	0.2	28	11.3
PS	52	M	3	1	31	3.9	141	1.36	0.76	24.45	1.8	high	9.1	5.5	0.14	32	11.8

PS	46	M	2	1	27.8	4	183	1.7	0.66	77.3	1.8	high	9.6	3.1	0.01	24	10
PS	78	M	3	1	18.5	3.9	123	1.34	1.13	88.56	1	high	8.4	3.8	0.02	23	9.8
PS	75	M	2	1	19.9	3.8	153	1.98	0.63	79.7	1.3	high	9.9	5.4	0.2	40	10.7
PS	72	F	5	1	18	3.5	206	1.51	0.75	92.17	2.1	high	8.8	4.9	0.08	23	10.3
PS	52	F	5	1	18	4.6	136	1.14	0.91	67.94	2.1	high	8.4	6.3	0.05	29	13.6
PS	55	F	11	1	22.1		209	0.89	1.24	90.44	1	high		6.8	1.5	38	12.4
PS	73	M	8	1	22.3		197	1.72	1.01	116.2	1.4	high	9.1	5	0.05	25	10.3
PS	77	M	7	1	18.6		183	2.36	0.88	84.49	1.5	high	8.8	5.7	0.09	25	10.1
PS	68	F	7	1	23.9			1.04	0.75	75.86	2.1	high	8.1	5.3	0.45	30	12.3
PS	61	M	28	1	22.4	3.8	139	1.42	1.07	93.23	2.1	high	9.1	5.8			10.7
PS	43	F	27	1	19.1	4.6	101	1.29	1.26	96.86	1.8	high	10.3	7.2	0.04	31	11.5
PS	39	F	25	1	23.4	3.7	112	1.25	0.96	95.31	1.3	high	8.2	5.2	0.03	32	9.9
PS	54	M	24	1	21	4.6	123	1.43	0.82	85.49	2.2	high	9	6.6			9.2
PS	54	M	19	1	23.9	4.4	141	1.31	1.04	114.2	1.5	high	7.9	5	0.03	18	10.3
PS	40	M	17	1	26.7	4	221	1.73	1.12	123.8	2.1	high	8.2	5.1	0.04	26	10.2
PS	37	M	16	1	20.2	4.4	146	1.8	1.24	98.27	2.1	high	8.7	3.4	1.14		10.9
PS	44	F	14	1	22.3	4.6	131	1.6	1.21	101.1	2.1	high	8.2	4		39	9.6
PS	50	F	13	1	21.5	4.5	199	1.7	0.94	124.5	2.1	high	8.8	4	0.13	22	9.4
PS	63	M	13	1	20.6	4.3	110	1.59	0.9	93.93	2.1	high	9.6	5.3	3.8	30	10.3
PS	68	M	13	1	20.9	4.3	202	1.62	1.1	126.2	2.1	high	8.3	6.6		23	11.4
PS	63	F	13	1	18.2	4.3		1.02	0.86	78	1.6	high		5.4		18	10.9
PS	60	F	12	1	19.1	3.6	137	2.09	0.72	90.35	1.5	high	9.2	7	0	23	10.3
PS	75	M	12	1	18.3	4	122	1.53	1.47	103.1	1.5	high	7.6	4.5	0.3	30	8.9
PS	55	F	12	1	21.6	3.9	164	0.89	0.94	93.86	1.5	high	8.4	5.6	3.97	40	8.9
PS	61	F	11	1	22.2	4	107	1.42	0.9	98.36	1.5	high		4.2	0	30	10.2
PS	57	M	10	1	21.1	4.3	172	1.43	1.26	76.96	2.1	high	9.1	4.9		23	10.7
PS	54	M	10	1	19.6	4	205	0.9	0.94	126	2.1	high	8.3	4.7	0.03	22	10.1
PS	42	M	9	1	17.4	4.7	145	1.43	0.98	108.9	2.5	high	9.8	6.4	0.21	31	12.7
PS	59	M	9	1	20.1	4.3	222	1.1	0.97	103.4	1.5	high	8.9	3.6			8.3
PS	58	M	8	1	23.6	3.8	179	1.65	0.87	117.4	1.6	high	10	5.2	0.35	26	10.6
PS	63	M	8	1	22.4	4.4	109	1.53	1.18	138.9	1.3	high	8.8	6.2	0.05	26	9.2
PS	60	M	7	1	20.6	3.8	185	1.28	0.86	129.4	1.8	high	8.3	4.4			9.8
PS	56	M	7	1	30.9	4	143	1.41	0.82	103.3	1.8	high	9.5	4.8	0.1	29	11.5
PS	68	F	7	1	23.5	4		1.54	0.84	133.6	2.5	high	7.7	3.2	0.3	34	12.6
PS	32	M	6	1	23.9	3.6	172	1.73	1.1	63.01	1.3	high	8	6.3	0.77	21	9.8
PS	65	M	6	1	25.4	4	265	1.63	0.71	119.7	1.5	high	9	4.1	0.09	22	10.1
PS	70	M	6	1	22.8	3.9	192	1.74	0.74	99.29	1.6	high	9.8	5.2	0.02	18	8.5
PS	59	M	5	1	20.1	3.9	138	1.55	1.13	116.3	1.8	high	8.5	4.7			11.5
PS	61	M	4	1	18.8	4	198	1.77	0.88	111.8	1.3	high	8.6	5.2	0.03	25	10.2
PS	55	M	3	1	29.1	4.2		1.78	0.95	133.8	1.5	high	8.9	6.7	0.1	18	10.7
PS	61	M	2	1	19.9	4.3	163	1.64	0.73	113.8	1.8	high	9.5	6.7	0.16	25	12
PS	62	F	22	1	17.4	3.9	207	1.45	0.81	101.6	2.1	high	10.8	7.7		25	9.1
PS	70	F	21	1	17	3.6	165	1.31	0.89	120.5	1.8	high	8.7	5.2	0.8		9.8
PS	39	M	11	1	34	4.3	111	1.19	0.95	104.7	1.8	high	10.5	5.6	0.01		
PS	54	M	6	1	23.7	3.8	166	1.6	1.17	79.63	1.3	high	8.7	7.3	0.1	25	9.6
PS	56	F	6	1	16.1	3.8		1.75	0.83	97.45	1.1	high	8.7	5.4	0.05		14.3
PS	60	M	2	1	30	4.1		1.85	0.6	101.3	2	high	6.9	5.1			9.5
PS	25	M	2	1	17.3	4	115	1.96	1.11	101.6	2	high	10.4	5.6	0.11	36	10.8
PS	59	M	36	1	20	3.6	186	1.19	1.02	51.43	1.4	high	6.9	7.1		32	9.4
PS	52	M	17	1	16.7	3.4	171	1.16	0.75	119.1	2.1	high		6.4		22	10.5
PS	41	M	14	1	17.4	3.6	134	1.51	0.72	89.96	1.5	high	8.4	4.9	0.05	31	9.1
PS	63	F	9	1	15.9	3.9	179	2.21	0.98	80.4	1.8	high	9.4	5.9		29	11.1
PS	70	F	8	1	23.7	3.7	129	1.4	0.83	114.6	2.1	high	9	5	0.5	32	10.1
PS	71	M	4	1	30.2	3.7	172	1.49	0.76	125.4	1.5	high	8.5	5	0.13	24	10
PS	59	M	4	1	21.8	4	173	1.46	0.8	104.8	2.1	high	8.7	4.1	0.4	24	10.7
PS	60	F	4	1	20.6	4.1	134	1.22	0.84	78.58	2.1	high	9.8	7.4		29	11.4
PS	79	M	4	1	22.4	3.8	184	1.36	1.07	92.26	1.6	high		4.6	0.23	24	8.6
PS	63	F	4	1	28.3	3.6	91	1.39	0.75	98.21	1.8	high	9.8	3.8	0	17	11.3
PS	68	M	4	1	24.9	3.4	175	2.02	0.61	115.7	1.5	high	10.2	5.2	0.34	24	10.8

PS	75	M	4	1	22.4	3.7	98	1.23	0.75	72.53	1.1	high	8	6	0.9	44	6.2
PS	72	F	4	1	19.1	3.6	156	1.61	0.9	114.9	1.4	high		4.3		35	10
PS	50	M	4	1	20.3	3.4	102	0.93	0.67	90.93	1.8	high		7.3	0.1	22	9
PS	76	F	3	1	17.3	3.5	115	1.37	1.2	143	1.1	high		3.9			11.8
PS	65	M	3	1	21.7	3.7	127	1.1	0.71	92.09	2.5	high	9.3	6.1		33	9.2
PS	49	F	3	1	31.1	3.7	125	1.48	1.23	84.45	2.1	high	8	4.1	0.1	31	10.4
PS	77	M	3	1	17.7	3.9	129	1.32	0.77	86.34	2.2	high	10	7.9		35	12.5
PS	76	M	3	1	20.4	3.6	133	1.73	0.63	72.24	1.5	high	8.5	6.5	0.1	35	12.3
PS	72	F	2	1	21.7	3.5	132	1.43	0.98	103.4	1	high	9.3	3.9	0.3	35	9.9
PS	62	M	2	1	22.5	3.9	191	1.56	1	111.9	2.1	high	8.8	4	0.1	35	12.3
PS	79	F	2	1	25.6	3.7	190	1.35	0.73	99.89	1.4	high	8.7	5			9.7
PS	73	M	2	1	23.3	3.8		1.81	0.95	135.7	1.3	high	9.5	5.4		19	
PS	66	M	7	1	24.6	3.9		1.83	0.98	147.6	2.1	high	8.9	3.9	0.04	22	10.3
PS	64	F	30	1	19.4	4	142	1.35	1.11	97.87	1	high	9.2	3.2			11.4
PS	62	M	24	1	22.5	3.6	179	1.25	1.23	140.8	1.4	high		7.1	0.57	33	11
PS	65	M	22	1	23.6	3.6	150	1.91	0.96	128.9	1.9	high	6.2	4.1		26	10.4
PS	43	F	17	1	19	3.7	199	1.52	0.81	87.25	1.8	high	9.3	3		17	11.9
PS	63	M	17	1	19	3.9	235	2.08	0.88	75.34	2.2	high	9.8	6.1	0.09	22	10.7
PS	70	M	17	1	19.4	4.1	154	1.28	1.06	107.8	1.8	high	9	3.8	0.07	25	10.4
PS	63	M	16	1	20.7	3.6	116	0.92	0.8	110.1	1	high	11	8.5	0.35		7.5
PS	51	F	10	1	16.6	4.5	93	1.3	1.15	102.2	1.8	high	9.5	5.1			
PS	55	M	9	1	21.8	3.7	101	1.22	0.85	111.2	1.5	high	9.7	3.6	0.16	27	12.6
PS	76	F	8	1	26.1	3.7		1.34	0.95	74.61	2.1	high	9.4	5.1	0.05	29	
PS	82	F	7	1	16.3	3.4	143	1.85	0.72	43.4	1	high	7.8	5.9			8.8
PS	59	M	7	1	19.8	3.9	133	1.93	1.09	130.7	1.5	high	8.8	5.9	0	29	10.1
PS	56	M	6	1	20.1	4.1	142	1.37	0.97	138.1	1.1	high	7.6	6.1	0.33	30	13.6
PS	62	M	6	1	17.9	3.5		1.41	0.79	75.01	1	high	9	4.8	0.04		7.7
PS	47	F	6	1	21.3	3.4	177	1.34	0.85	76.66	2.1	high	10.7	8.3	0.33		11.7
PS	56	M	5	1	23	4.2	110	1.53	0.84	117.4	1.5	high	8.5	5	0.01	24	10.3
PS	48	F	5	1	21.6	3.6	171	1.51	1.12	112.6	2.1	high			0.1	29	12.2
PS	75	M	5	1	22.8	3.4	143	1.63	0.93	84.32	1.5	high	10.9	6.5	0.05	22	12
PS	82	M	4	1	20.8	3.1	171	1.94	1.01	81.93	1.5	high	8.6	4.2	1.17	29	9.2
PS	77	M	4	1	24.5	4	71	1.75	1.23	124.4	1	high	7.5	4.3	0.1		11.3
PS	59	M	4	1	21.7	3.5	106	0.75	0.82	103.9	1.3	high	7.4	6.1	0	21	9.3
PS	74	M	3	1	28.6	3.5	203	1.82	0.71	100.1	1.8	high	8.8	5.4	0		11.2
PS	78	M	3	1	20.2	3.2	128	1.14	1.23	93.93	1.5	high		4	0.27		10.9
PS	67	M	2	1	22.9	3.8	95	1.36	0.87	73.41	2.2	high	9.2	4.8	0.08	48	11.4
PS	73	M	2	1	21.6	3.9	153	1.59	1.19	95.56	1.4	high	8.4	4.4		32	12.7
PS	55	M	13	1	23.2	4.1	121	1.27	0.66	94.81	1.8	high	8.9	7.7	0.4	51	11.2
PS	77	F	2	1	19.3	4.2	225	1.69	1.42	126.1	2.1	high	9.7	4.6	0.1	23	9.8
PS	57	M	9	1	20.9	3.4	167	1.39	0.65	112.9	1	high	9.5	4.7	0.33	18	12.2
PS	60	F	23	1	19.9	3.8	181	2.07	1.12	111.9	1.9	high	10	3.3		31	10.1
PS	54	M	35	1	18.4	4.5	173	1.1	0.86	98.86	1.8	high	8	5.4	3.78		10
PS	64	F	7	1	23.6	3.7	174	1.77	1.18	101.9	1.6	high	8.4	5.5	0.37	35	10.6
PS	71	F	3	1	21.8	4	122	1.37	0.68	111.3	1	high	9.1	4.4	1.1	28	8.1
PS	56	F	34	1	20.4	4.3	165	1.6	1.12	96.19	1.1	high	8.5	5.7	0.05	24	9.3
PS	55	F	27	1	16	3.7	95	1.11	0.93	72.58	1.8	high	9.5	7.3	0		11.1
PS	66	M	23	1	19.7	3.5	121	1.3	1.18	81.44	2.1	high		8.9		25	9.6
PS	53	M	22	1	17.1	3.7		1.41	0.88	91.07	2.1	high	8.4	4.6	0.07	19	10.4
PS	66	M	9	1	20.7	3.8	168	1.6	0.97	116.5	1.3	high	9.1	5.7		30	7.5
PS	43	M	8	1	27.5	3.9	115	0.85	0.3	5.22	2.1	high	8.6	3.4		21	11.6
PS	51	F	8	1	20.1	4.1	163	1.51	0.94	129.9	1.4	high	7.9	4.3	0	39	7.5
PS	70	M	5	1	19.2	3.3	147	1.58	0.99	71.52	2.1	high	8.8	5.9		19	10.6
PS	52	F	25	1	23.7	4.1	128	2.42	0.93	113.6	1.9	high	10	4.7		37	10.5
PS	46	F	25	1	16.1	3.6	187	2.18	1.06	118	1.9	high	10.1	5.3		29	9.2
PS	41	F	24	1	24.8	3.8	160	2.33	1.06	130.6	1.9	high	9.5	4.1		32	11.8
PS	55	F	21	1	17.9	4.2	159	2.01	1.16	112.6	1.9	high	9	6.2		32	11.3
PS	73	M	19	1	15.7	4	138	1.54	0.81	67.96	1.8	high	9	4.4	0.09		11.2
PS	59	F	19	1	19.8	3.8	160	2.1	0.98	116.4	1.8	high	9.1	1.9			9.9

PS	49	M	17	1	17.8	4.2	158	1.14	0.95	121.7	1.4	high	8.2	5	0.6		9.6
PS	66	M	13	1	22.6	3.8	137	1.54	0.91	105.3	1.4	high		3.8	0.2	23	10.2
PS	48	F	11	1	28.2	4.1	205	0.85	1.04	143.8	2.2	high	8.3	3.3	16	22	8.3
PS	56	M	10	1	25.5	4.5	150	1.22	0.85	99.35	2.1	high	8.1	6.3	0.1	22	10.8
PS	67	M	10	1	22.9	3.6		1.32	0.56	95.23	1.4	high	8.5	2	6.96		10
PS	59	M	10	1	21.1	3.8	184	1.57	0.89	121.9	2.1	high	7.9	3.7	18.1	19	10.4
PS	59	M	6	1	20.1	3.6	156	2.19	0.94	120.6	1.5	high	10.9	6.1		28	8.4
PS	61	M	6	1	26.6	4.1	121	1.29	0.89	108.3	2.1	high	10.2	6	0.1	29	11.1
PS	39	M	6	1	20.7	4.3	208	1.1	0.78	108.1	1	high	8.4	5.4	0.16		9.8
PS	58	M	6	1	20.2	3.8	133	1.24	1	105.8	2.1	high	10.6	4.1	0.02	27	9.2
PS	69	M	5	1	22.5	3.4	177	1.24	0.56	107	2.1	high	8.7	4.1	0	19	10.6
PS	58	M	5	1	18.7	3.9		1.85	1.02	137.6	1.5	high	9.1	7	0.67	30	10.7
PS	72	M	5	1	24.1	3.7	160	1.61	0.97	80.82	1.8	high	8.9	4.5	0	36	9.6
PS	57	M	4	1	23	4.2	155	1.73	0.67	90.75	1.5	high	8.5	5.6	0.1	25	11.4
PS	84	F	3	1	17.1	3.7	216	1.13	0.73	92.06	0.6	high	7.3	7.3	0.43	40	10.9
PS	76	F	3	1	21.8		171	1.73	0.82	70.04	2.2	high	9.8	6.4	0	28	12.3
PS	77	M	3	1	17.1	3.7	179	1.23	0.72	86.18	1.1	high	8.3	4.2	0.1	39	11.2
PS	63	M	3	1	20.8	4.2	135	1.37	0.86	89.76	1.5	high	8.8	4.4	0.2	24	10.7
PS	46	M	3	1	20.5		172	1.46	0.78	97.5	1.8	high	9.7	3.2	0.3	35	12.1
PS	78	F	2	1	21	3.9	202	1.28	0.52	59.04	1.5	high	9	4.5	0.5	25	10.1
PS	58	F	22	1	20.1	2.5	149	1.31	1.05	112.7	1.8	high	9.2	3.9	0		10.1
PS	45	M	10	1	18.9	2.4	199	1.51	0.97	98.35	1.5	high	10	5.7			12.1
PS	50	M	8	1	19	3.7	130	1.2	0.9	106.9	2.1	high	8.7	5.6	0.28	27	10.6
PS	26	M	8	1	21.4	3.7	140	1.47	0.99	122.9	1.6	high	8.3	5.7	0	21	10.6
PS	60	M	8	1	19.4	4	198	1.38	1.03	110.6	2.1	high	8.3	3.6		24	9.9
PS	71	F	8	1	26.1	3.9	119	1.41	0.99	90.72	1.6	high	8.6	5.4	1.21	35	9.8
PS	45	M	6	1	23.2	3.6		2.17	1.08	79.84	1.3	high	8.1	4.1		31	11.5
PS	31	M	5	1	22.8	4.3	175	1.08	0.91	106.3	1.5	high		10.1	0.2	22	10.9
PS	61	M	5	1	21.3	3.7	127	1.21	0.8	101.3	1.8	high	7.8	7.3	2.36	29	11.1
PS	75	M	5	1	25.2	3.5	162	1.67	0.84	112	2.1	high	8.7	5.1	0	24	9.1
PS	46	F	4	1	25.6	3.7	177	1.75	0.75	94.53	2.1	high		6.3		30	11.8
PS	65	M	4	1	23.9	2.8	184	1.3	1.09	133.2	2.1	high	9.2	5	0.06	18	10.9
PS	44	M	3	1	26.6	3.9		1.56	0.85	121.6	2.1	high	9	5.6	0.03	25	11.6
PS	60	F	3	1	27.5	4	170	1.24	0.82	50.62	2.1	high	8.9	6.3	0.12	21	11.1
PS	36	F	3	1	26.8	4.3	142	1.16	0.97	85.49	1.8	high	9.5	4.4		29	9.4
PS	69	F	2	1	30.5	3.7	195	1.71	0.95	90.68	2.1	high	9.5	4.5	0.17	20	9.5
PS	30	M	2	1	24.5	4.2	145	1.4	1	109.6	1.8	high		6.6	0	28	8.4
PS	77	F	2	1	26.5	3.8	149	1.27	0.62	114.6	1.5	high	9.6	4.6	0.2		9.4
PS	46	F	2	1	32.5	3.9	190	1.35	0.95	121.1	1.6	high	10.1	4.1		25	10.7
PS	71	M	2	1	27.3	3.2	196	1.26	0.7	74.55	1.6	high	11.5	4.3	0.04		11.8
PS	57	M	2	1	16.3	3.9		1.91	0.73	111	1	high	8.2	5	0.87		10.1
PS	55	F	2	1	22.8	4.2	121	1.16	1.41	118.1	1.5	high		5.8	0.43	28	9.2
PS	72	M	2	1	22.4	4.3	128	1.69	0.8	77.71	1.8	high	8.2	5.6	0	18	9.7
PS	33	M	2	1	20.5	4.2	195	1.55	0.99	74.62	1.5	high	10.3	8.2	0.06	37	11.2
PS	39	M	2	1	18.9	4.1	107	1.3	0.68	53.49	2.1	high	10.4	5.3	0.2	19	9.6
PS	56	M	5	1	18.1	3.3		1.46	1.26	102.5	2.1	high	8.7	6	0.1	18	10.5
PS	66	F	12	1	17.1		153	1.23	1.11	98.06	2.1	high		6.1		23	10.4
PS	72	F	10	1	16.8	3.6	147	1.27	1.06	61.09	1.6	high		3.7	3.53	29	9
PS	72	F	9	1	21.5	3.8	153	1.45	0.91	82.73	1.5	high	10	3.2	7.85	46	9.6
PS	64	F	8	1	21.1	4.1	259	0.98	0.79	114.5	2.1	high		5.2		33	8.8
PS	69	M	7	1	22.6	3.9	227	1.31	0.84	129.4	1.8	high	9.2	5.3	2.5	27	10.4
PS	59	F	7	1	20.8	3.9	105	1.47	0.92	117.5	1.5	high	9.7	8.4	1.73		8.9
PS	59	F	3	1	26.8	3.9	141	1.35	0.74	88.58	1.5	high	9	3.6	0.02	28	10.1
PS	69	M	3	1	25.7	3.7	165	1.21	0.76	93.08	1.6	high		7.8	0.1	28	10.4
PS	69	M	12	1	18.8	3.6	169	1.46	0.71	71.57	1.8	high	8.3	5.1	0.1	27	10.9
PS	71	M	2	1	20.1	3.8	147	1.38	0.65	59.07	1	high	9	2.7	0.1		10.3
PS	74	M	12	1	18.7	4.1	161	1.58	0.96	99.23	1.8	high		4.8	0.5		9.6
PS	72	F	22	1	16.9	3.8	164	0.91	1.12	97.12	0.8	high	8.7	3.8	0.92	42	10.3
PS	71	M	5	1	22.5	4	142	1.97	0.73	123.9	2.1	high	7.6	3.7	0.26	24	11.2

PS	56	M	25	1	22.4	3.7	136	1.43	0.81	91.1	1.8	high		5.1	0.12	22	12.6
PS	73	M	13	1	22.4	3.6	192	1.84	1.06	114.1	1.5	high	9.2	5.3	0.25	31	9.2
PS	75	F	11	1	19.6	3.8	147	1.33	0.81	111	1.5	high		4.1			11
PS	77	M	2	1	19.6	4.4	161	1.08	0.64	77.67	1.4	high	9.9	3.8	0.6	39	9.9
PS	58	F	9	1	18.2	3.8	163	1.21	1.14	123.2	2.1	high		5.6	0.02	25	10.1
PS	44	F	18	1	18.2	3.3	129	1.11	1.17	62.09	2.1	high		4.8	0	28	9.5
PS	78	F	17	1	21	3.4	113	1.29	1.16	113.4	1.8	high		6.5	0.06	28	11.2
PS	45	M	17	1	24.3	3.8	108	1.18	0.66	89.21	2.2	high		8.2	0.11		10.7
PS	63	M	16	1	20.2	3.7	172	1.52	0.79	105	1.8	high		6.6	0.1	30	11.5
PS	49	M	16	1	25.7	3.9	255	1.49	0.95	79.75	1.8	high		9.1	0.69		11.3
PS	68	F	16	1	19.6	3.5	136	1.33	0.84	106.6	1.8	high		6.9	0.09		12.5
PS	53	F	15	1	19.7	4.3	208	1.76	1.03	117.1	1.5	high	9.9	4.6	0.03	19	11.1
PS	53	F	15	1	16.2	3.7	114	1.51	0.66	98.52	1.8	high		4.8	0.1		10.1
PS	67	M	13	1	29.7	3.8	172	1.16	0.93	129.7	1.8	high		5.7	0.62		11.8
PS	43	M	13	1	18.9	3.9	146	1.79	0.7	82.48	2.2	high	9.3	5		30	10.5
PS	67	M	11	1	18.3	3.9	210	1.7	0.98	120.8	1.3	high	9.1	4.7	0	20	10
PS	31	F	9	1	16.1	4.1	140	1.81	1.01	121.4	1.4	high	10.5	2.8	0.1	24	8.6
PS	55	M	8	1	22.1	4.3	126	1.37	0.8	92.45	1.8	high	8.8	6.2	0.03	24	12
PS	59	M	8	1	23.2	4.1	138	1.06	0.67	73.23	1.8	high	8.5	3.6	0.03	21	11.3
PS	51	F	8	1	20.4	4.1	123	1.44	0.91	76.21	1.3	high	8.9	3.9		24	9.9
PS	49	M	8	1	20.8	4.2		1.3	0.94	104.2	1.8	high	8	5.6			11.2
PS	51	F	7	1	18.3	3.8	99	1.08	0.76	95.95	2.5	high	9.7	5.8	0.33	31	11.5
PS	49	M	7	1	26.9	4.3	158	2.11	0.67	116.2	1.5	high		5.5			
PS	62	M	6	1	15.3	3.8	165	1.48	0.84	78.29	1.8	high	8.8	5.1	0.08	27	11.8
PS	61	M	5	1	21.6	4.3		1.2	0.8	102.4	1.8	high	8.5	5.8			
PS	54	F	5	1	25.3	3.8	181	1.31	0.66	68.09	1.5	high	8.1	4.7		32	12.7
PS	66	M	4	1	22.6	4.1	196	1.37	0.83	105.7	2.2	high	8.9	7.2	0.02	22	12.4
PS	66	F	3	1	17.2	4.3	170	1.5	0.68	78.27	2.1	high	9	3.9	0.05	27	10.1
PS	85	F	2	1	19.7	3.6	146	1.13	0.93	96.11	1.3	high		5.8	0.59		10.6
PS	66	M	2	1	21.2	4	132	1.26	0.73	83.01	1.6	high		6.4	0.17	33	11.1
PS	62	F	9	1	19.2	3.7	173	1.62	0.87	106.9	1.5	high	8.4	6.1	0.24	39	10.8
PS	50	M	13	1	24.1	3.9	158	1.45	0.74	89.85	1.8	high	10	5.8	0	18	10.9
PS	69	M	4	1	21.5	4.4		1.71	0.74	113.4	1.8	high	9.3	6.2	0.12	30	10.6
PS	40	M	30	1	17.7	4.4	129	1.42	1.16	70.4	1.6	high	8.8	6	0.14	27	11.3
PS	70	F	21	1	16.3	4	148	1.64	1.51	81.04	1.6	high	8.8	5.7	0.1	27	9.5
PS	54	M	3	1	21.2	4	129	1.29	0.92	92.8	2.2	high	9.7	6	0.02	29	14.8
PS	54	M	2	1	21.4	4.5	120	1.67	0.88	110.5	2.1	high		4.6	0.09	29	9.7
PS	75	M	19	1	20.1	3.7	104	2.11	0.75	98.91	1.6	high	10	4.3	0.09	25	9.3
PS	72	M	3	1	22.2	3.7	166	1.58	0.85	98.86	1.8	high	9.1	4.2	0.05	28	11.1
PS	69	F	17	1	21	3.3	135	1.45	0.84	93.52	2.1	high	8.1	4.2	0.1	30	14.2
PS	66	M	20	1	16.9	4.3	114	1.71	1.09	113.2	1.5	high	9.2	6.5		31	9.1
PS	44	M	11	1	25.4		130	1.37	1.45		1.3	high		6.4	0.2	31	10.2
PS	75	M	7	1	22.2	3.8	116	1.55	0.73	105.3	1.5	high	9.4	4.7	0.1	19	11.7
PS	55	F	6	1	22.4	3.6	143	1.03	1.06	145	1.3	high	8.3	4.1	0.42	31	10.9
PS	62	F	5	1	21.2	3.8	107	1.29	0.95	85.89	1.5	high	8.2	5.2	1.2		8.2
PS	75	M	2	1	23.6	3.1	115	1.34	0.79	97.82	1.8	high	8.1	2.6	1.12	24	9
PS	79	F	3	1	14.4	3.5	102	1.22	0.92	67.05	2.2	high	8.8	4.8	1.13	34	12.2
PS	72	M	4	1	21	3.6	183	1.43	0.88	124.8	1.5	high	9.5	5.3	0.24	22	9.7
PS	72	M	20	1	19.4	3.8	151	1.55	1.03	118	2.1	high	9	5.7	0.2	24	10.9
PS	59	F	14	1	21.1	3.8		1.13	1.12	121	1.8	high		4.8			7.5
PS	59	F	12	1	21.8	3.9	121	1.8	1.1	130.8	1.8	high	8.4	4.8	0.11	32	9.6
PS	74	F	10	1	18.8	3.8	219	1.35	0.94	136.1	1.1	high	7.9	9.3	3.4	29	11.4
PS	63	F	8	1	23.5	3.9	134	1.01	0.99	105.4	1.6	high	9.3	4.8	0.05	23	10.7
PS	76	M	7	1	21.3	3.4	170	1.49	1.14	134.4	1.5	high	9.2	4.3	0.05	29	10.5
PS	78	M	7	1	24.8	3.6	190	1.73	1.07	149.5	2	high	10.1	6.2	0.4		9.5
PS	63	M	6	1	19.2	3.3	193	1.25	0.81	125.4	1	high		5.8	0.1	23	9.3
PS	58	F	6	1	22.2	3.7	154	0.87	1.17	129.1	0.8	high	7.5	4.7	0.07	14	10.4
PS	68	M	6	1	21.4	3.8	191	1.77	1.16	109.5	1.6	high	7.8	4.8	0.85	30	9.5
PS	57	F	6	1	18.6	3.9	211	1.28	0.99	92.9	2.1	high	8.9	4.7	0.55	24	11.1

PS	41	F	6	1	16.7	3.8	146	1.19	1.23	71.26	2.1	high	9.2	5.2	0.4	21	10.3
PS	61	M	5	1	19.6	4	121	1.02	0.97	110.1	2.1	high	8.3	8.2	0.31	31	11.7
PS	63	M	4	1	25	4.2	127	1.25	1.06	72.64	2.1	high	9.1	7.4	0.05	32	10
PS	38	M	4	1	24	4	163	1.44	0.79	101	1.5	high	9.5	6.4	0.06	29	12
PS	54	F	4	1	19.2	4.1	241	1.36	0.81	85.6	2.5	high	10	5.8	0.2	23	9.4
PS	72	M	4	1	23	3.7	141	1.37	1.02	109.5	2.1	high	9.9	4.9	0.02	36	9.7
PS	60	M	3	1	24.8	3.9	106	1.26	0.96	78.69	2.1	high	8.8	6.3	0.03	40	12.1
PS	68	M	3	1	20.7	4	161	1.18	0.93	79.08	2.1	high	9.5	5.9	0.01	28	14
PS	55	M	2	1	25.5	3.9	126	1.45	0.98	129.2	1.8	high	8.7	7.1	1.44	29	10.3
PS	53	F	2	1	19	4	192	1.2	0.81	65.46	2.1	high	8.7	7.3	0.81	19	11
PS	67	M	2	1	23.7	2	161	1.22	1.13	125.5	1.3	high	8.7	6	0	19	10.5
PS	65	M	2	1	24.4	4.1	153	1.31	0.8	126.5	2.1	high	7.7	4.8	1.1	26	7.2
PS	59	F	2	1	21.2	3.5		1.52	0.74	107.4	1.5	high	9.8	5.8	0.03	34	10.4
PS	63	M	5	1	21.6	3.7		1.93	0.77	107.4	1.5	high	10.7	6.1	0.07		10.6
PS	71	M	2	1	21.4	4		1.3	1.02	96.65	1.5	high	8.4	6.2	1.32	29	10.8
PS	34	F	8	1	17.4	3.6	216	0.78	0.92	106.4	1.3	high	10.5	3.5	0.1	16	10.7
PS	53	M	33	1	24.3	3.3	224	1.51	0.81	68.99	1.8	high	9.8	6	0.04	24	11.1
PS	69	F	31	1	20.6	3.4	180	1.27	1.33	105.2	1.8	high	8.7	7.1	0.11	21	11.8
PS	49	F	30	1	21.5	3.1	153	1.04	1.19	103.9	2.2	high	8.9	4.4	0.33		8.9
PS	59	F	27	1	20.7	3.5	174	0.92	0.91	80	2.1	high		4			9.6
PS	61	F	24	1	23.8	3.4	133	1.72	1.11	117.9	2.2	high	9.6	5.8			10.9
PS	78	F	19	1	19.5	3.4	149	1.29	0.96	75.92	1.8	high	8.3	5.9			8.7
PS	57	M	18	1	19.7	4.1	152	1.69	0.92	111.7	1.6	high	8.9	6.4	0.07	31	10.2
PS	66	F	17	1	16.8	3.4	115	1.16	0.87	149.4	2.2	high	9.3	8			8.8
PS	68	M	16	1	21.2	3.6	140	1.28	1.07	142.1	1.8	high	9	5.8	0.05	24	10.3
PS	57	F	16	1	18.4	3.7	157	1.71	1.21	109.4	1.6	high	10.2	5.8	0.15	19	12
PS	47	F	15	1	17.5	4.3	113	1.44	0.99	112.7	1.8	high	9.1	2.7			9.4
PS	67	M	15	1	29.8	4.3	194	0.96	1.12	113.2	2.1	high	8.8	7	1.5		10.9
PS	45	F	15	1	18.4	3.8	182	1.06	0.86	83.23	1.3	high		2.3	0.24	31	10.4
PS	61	M	15	1	19.5	3.4	147	1.08	0.82	95.84	2.1	high	8.5	6.9	0.1		10.7
PS	77	F	13	1	25.9	3.6	147	1.45	0.95	96.52	1.3	high	8.9	4.7	0.18	23	10.8
PS	71	M	13	1	20.3	4	107	1.34	1.02	124.8	2.2	high	9.2	6.4	1.45	33	11
PS	67	M	12	1	18.4	4.2	160	1.98	1.14	93.38	1.6	high	9.3	5.8	0.07	27	9.5
PS	64	F	9	1	20.4	1.6		1.67	0.77	120.4	2.1	high	10	3.6	0.1	20	10.2
PS	59	F	9	1	19.6	1.6	127	1.41	1.1	89.08	1.5	high	8.6	4.1	0.3	18	8.9
PS	70	M	8	1	20.2	3.8	202	1.27	0.95	104.1	1.5	high	9.6	5.4	0.22	20	10.8
PS	75	M	7	1	23.4	3.8	166	1.39	0.82	118.8	2.2	high	9.4	5.4	0.01	19	10.5
PS	66	M	7	1	21	3.3	127	1.36	0.86	92.2	1.5	high	9.9	7.3	0.1	27	10.6
PS	69	F	7	1	17	3.7		1.26	0.92	66.36	2.1	high	7.3	3	0.38	32	9.4
PS	81	F	6	1	28.4	3.5	247	1.27	0.96	104.4	2.2	high		5.6	1.03	30	12
PS	71	M	6	1	19.5	3.2	173	0.86	0.62	96.76	1.3	high	8.2	3.7	0.08	14	11.3
PS	78	F	6	1	18.7	3.9	187	1.77	0.98	101.2	2.2	high	9	4.7	0.05	28	9.6
PS	56	F	5	1	29.7	3.4	107	1.68	0.77	111.6	1.3	high	9	5.4	0.73	25	10.6
PS	74	F	5	1	22.4	3.6		1.72	1.46	86.28	2.1	high	8.1	3.9	0.07	29	12.5
PS	67	M	5	1	16.1	4	146	1.32	0.72	93.04	1.5	high	9.4	3.8		36	8.8
PS	61	F	5	1	29.3	3.6	91	1.43	0.81	102.9	1.5	high	9.1	4.1	0.15		11.4
PS	66	M	4	1	22.7	3.6		1.77	1.04	132.8	2.1	high	8.1	4.9	0.03	27	9.7
PS	72	F	4	1	25	3.2	105	1.46	1.18	143.9	2.2	high	8.9	4.1	0.06	21	10.4
PS	66	M	4	1	21.3	3.5	144	1.64	0.86	82.74	1.6	high	8.8	4.6	0.03	27	10.4
PS	78	M	3	1	19.7	3.7	148	1.84	1.04	123.3	2.2	high	8.4	7.6	0.06	32	11
PS	69	M	3	1	24.3	4		1.56	0.91	107.8	2.1	high	8.3	3.9	0.25	32	11.4
PS	50	F	2	1	17.4	3.5	157	1.69	0.81	106.1	2.2	high	8.3	5.8	0.02	24	10.8
PS	46	M	2	1	23.1	4	118	1.31	0.82	152.1	1	high	8.4	5.5			8.9
PS	69	F	2	1	21.7	3.6	172	1.62	0.77	86.13	2.5	high		7.3			11.3
PS	75	M	7	1	19.3	3.6	168	2	0.7	101.7	1.5	high	7.9	4.7	0.04	32	9.8
PS	56	M	30	1	19.8	4	126	1.35	1.05	96.68	2.2	high	9	5	0.11	36	11.2
PS	68	M	29	1	25.7	3.8	111	1.41	1.02	93.69	2.1	high		6			8.9
PS	61	M	15	1	21.7	3.5	123	1.36	0.71	95.31	2.2	high	9.1	4	0.02	25	11.2
PS	67	F	12	1	17.8	3.8	154	1.43	0.9	109.6	2.1	high		4.4			9.6

PS	54	M	11	1	17.6	4.1	174	1.35	1.06	120.7	2.2	high		6.2	1.02	26	10
PS	40	M	10	1	19.1	4.3	172	1.36	1.05	130.5	1.4	high	7.6	4.7	0.16	35	11.6
PS	40	M	9	1	21.4	4.3	193	1.52	0.57	103.9	1.8	high	9.8	9.2	0.12	21	11.1
PS	71	M	9	1	23.7	3.5	136	1.21	0.65	93.9	2.1	high		5.4		29	10.2
PS	57	F	8	1	17.1	4.5	98	1.46	1.19	116.3	1	high		5.2	0.1	21	9.4
PS	67	M	8	1	22.4		164	1.12	0.92	126.6	1.8	high	9.6	5.6	0.05	25	11.3
PS	63	F	8	1	15.8	3.8	140	1.22	0.89	126.3	2.2	high	7.6	4.6	0.57	34	12.7
PS	49	M	8	1	22.4	4.5	162	1.18	0.85	131.8	1	high		4.7	0.19	23	11.4
PS	60	M	8	1	19.1	4.2	82	1.8	1.04	73.5	1.8	high	8.6	4.9	0.36	51	8.7
PS	59	M	7	1	18.9	3.5	148	1.75	0.83	92.99	1.8	high	9.2	5.2	0.03	26	10.7
PS	68	M	7	1	16.6	4.2		1.62	1.08	121.6	2.1	high	8.5	4.6	0.05	21	10.4
PS	68	M	7	1	24	3.9	142	1.25	0.72	139.4	1.5	high	9.3	4.4	0.04	30	8.8
PS	74	F	7	1	21.2	3.3	92	1.44	0.9	101.7	2.5	high		4.7			8.5
PS	58	M	7	1	21.3	3.9	142	1.38	1	92.13	2.2	high	8.8	6.3	0.4	28	11.4
PS	66	F	6	1	18.2	3.9	167	1.41	1.02	99.45	1.5	high	10.1	5.9	0.05	25	12.4
PS	68	M	6	1	18.8	3.1		1.5	0.96	109.2	2.1	high	9.5	5.3	0.06	24	11.6
PS	46	F	6	1	29.1	4.2		1.16	0.88	115	2.1	high	9.3	4.7	0.15	20	10.7
PS	58	M	6	1	23.8	3.7	153	1.49	0.83	111.1	2.1	high	10.3	7.3	0.06	22	10.3
PS	58	F	6	1	16.3	4.5	132	1.56	1.04	105.1	2.2	high	9.2	6	0.4	35	10.3
PS	62	F	6	1	14.9	4.2	141	1.5	1.04	123.9	1.5	high		6.7	0.3		10.8
PS	57	M	5	1	24.4	3.8	134	1.86	0.55	78.03	1.8	high	9.2	6.7	0.04	27	11.8
PS	53	F	5	1	43.6	3.6	138	1.61	0.83	136	2.1	high		6.1			8.4
PS	56	F	5	1	21.6	4.2	175	1.71	0.97	138.4	1.3	high		6.5	0.07		10.8
PS	59	M	5	1	22.4	3.7	162	1.51	0.74	96.1	1.8	high	9.1	4.7	2.37	26	9.3
PS	69	M	13	1	19.6	4.2	133	1.54	1.06	113.7	1.5	high	9.8	5.6		34	11.4
PS	40	M	4	1	27.1	4.3	132	1.37	0.87	112.1	2.5	high	9.9	5.5	0	22	9.7
PS	66	F	4	1	17.9	3.8	107	1.08	0.87	132.5	1.8	high	8.9	5.2	0.1		9.6
PS	45	F	4	1	17.7	2.3	151	1.24	0.63	81.53	2.2	high	10.4	5.8	0.4	29	11.2
PS	56	M	4	1	20.5	4.3	180	1.65	0.6	65.54	1.5	high	8.9	5.5	0.7	29	10
PS	61	F	4	1	17.3	3.6		1.75	1.02	78.53	2.1	high	9.3	5		21	
PS	65	M	3	1	19.7	4	245	1.97	0.73	83.11	1.5	high	8.9	3.6	1.01	27	10.7
PS	78	F	3	1	17.6	4	202	0.73	1	120.1	2.2	high	9.4	5.2	0.41	20	13.7
PS	61	M	3	1	23.3	4.2	213	1.66	0.81	65.64	2.1	high	8.8	4.3	0.23	27	10.9
PS	64	M	2	1	19	3.9	172	1.41	0.73	109.4	2.1	high	8.6	5.2	0.4		10.9
PS	62	M	2	1	25.5	3.8	224	1.39	0.9	122.9	2.1	high		5.8			11.5
PS	47	M	2	1	18.7	4	129	1.66	0.98	103.9	1.8	high	9	5.6	0.02	30	9.3
PS	49	M	2	1	24.9	4.4	148	1.62	0.88	115.4	1.8	high		5	0.11	31	11.1
PS	68	M	7	1	26.1	4		1.15	0.81	126.3	2.1	high	7.7	2.7		29	8.2
PS	62	M	4	1	22.5	4.2	188	1.29	1.02	75.99	1.8	high	8.4	8.4	0.13	26	9.7
PS	44	M	22	1	20.2	3.5	124	1.5	0.86	71.7	2.1	high	9.3	7.1	0.11		12.4
PS	41	F	21	1	20.9	3.8	179	1.23	1.2	89.74	2.2	high	9.2	5.3	0.4	27	10.6
PS	64	M	13	1	22.9	3.9	166	1.49	0.82	144.1	2.1	high	8.8	4.5	0.2	22	10.6
PS	63	F	9	1	19.8	4	151	1.2	1	111.6	1.5	high	9.4	5.5		30	9.8
PS	67	M	7	1	19.8	3.8	163	1.45	1.17	131.2	1.8	high	8.4	5.8	0.06		11.4
PS	71	M	5	1	20.3	3.7	134	1.68	0.8	45.17	1.6	high	8.1	8.5	0.23	37	11.9
PS	46	M	5	1	24.8	3.6	191	1.73	0.68	95.02	1.6	high	8.2	6	0.34		11.4
PS	62	M	4	1	16.6	2.8	144	1.11	0.54	46.88	1.5	high	9.2	4.8		17	11.2
PS	84	F	4	1	21.1	3.5	175	1.4	0.66	96.16	2.2	high	10.2	5.7	0.4	27	10.3
PS	80	M	3	1	20.3	3.7		1.47	0.87	109.8	2.1	high	9.3	5.1		18	10.1
PS	53	M	2	1	12.6	3.4		1.62	0.89	71.25	1.8	high	8.9	5.2	0.35	26	10.7
PS	38	M	2	1	22	4	168	1.58	0.86	52.34	1.6	high	7.9	6.2	0.07	23	12.8
PS	70	M	5	1	19.8	3.7	167	1.87	0.82	110.5	1.6	high	9.7	3.9	0.1	32	
PS	59	F	16	1	15.7	3.4	171	1.59	0.9	77.24	1.4	high	8.5	7.1	0	24	11.4
PS	60	M	4	1	23.5	4		1.35	1	74.72	2.1	high	9	4.7		28	8.8
PS	57	M	4	1	21.1	3.9		1.27	0.81	79.67	2.1	high	9.4	5.8		24	9.7
PS	47	F	26	1	18.6	4.1	117	2.35	0.86	125.7	1.6	high	8.4	3.2		45	9.4
PS	65	M	15	1	15.3	3.4	199	1.56	0.96	107.2	2.2	high	9.5	7.3	0.01	26	10.5
PS	73	M	11	1	21.3	3.7	114	1.36	1.1	139.5	2.2	high	7.6	6.2	0.29	26	10.3
PS	72	M	6	1	19.2	4.1		1.34	0.7	119.5	2.5	high	9.6	7.1	0.16	27	10.8

PS	80	F	6	1	18.5	3.8	128	1.93	0.86	98.4	1.6	high	8.2	5.3	0.1		11.5
PS	80	M	5	1	23.5	4	176	1.85	0.76	140.2	1.6	high	8.2	5.9	0		10.7
PS	83	M	4	1	19.9	4	143	1.9	0.85	102.1	1.6	high	8.7	4	0.1		10.3
PS	73	M	4	1	21.7	3.7	159	1.71	0.79	93.75	2.1	high	7.5	3.5	2.11	27	9.8
PS	40	F	4	1	19.1	4	161	1.08	0.65	105.2	2.2	high	9	6		24	11
PS	71	F	12	1	20.4	3.5	185	1.17	1.15	107.6	2.1	high	9.2	6.3	0.3	27	10.8
PS	71	F	8	1	25.3	3.6	170	1.77	1.15	92.98	1.5	high	8.8	4.4	0.5	20	12.4
PS	70	M	5	1	21.7		145	1.63	0.83	124.2	2.1	high	8.6	3.7	0.26	27	9
PS	67	M	15	1	19.7	3.5	215	1.26	0.89	102.9	2.2	high	8.5	6.4		26	11.1
PS	70	M	12	1	18.3	3.4	109	1.18	0.97	83.8	2.2	high	7.7	6.1		31	11.4
PS	75	F	11	1	22.9	3.2		1.23	0.98	106.6	2.1	high	9.5	6.3		31	11.3
PS	72	M	4	1	18.7	3.6	177	1.48	1.21	94.95	2.2	high	7.8	6.5		21	12.6
PS	60	M	24	1	24.2	4	146	2.03	1	125.4	1.5	high	9.2	6.4	0.2	28	11.6
PS	58	M	21	1	19.6	4.1	138	1.51	1.09	111.2	2.1	high	8.6	5		22	8.8
PS	69	M	17	1	20.4	3.4	181	1.78	0.77	69.4	1.8	high	9.2	4.4	0.12	19	9.9
PS	68	M	16	1	25	4.1	139	1.7	0.73	91.64	1.5	high	15	6		30	11
PS	68	F	16	1	17.8	1.9	152	1.73	1.03	104.9	1.5	high	9.7	3.1		36	10.3
PS	58	M	15	1	20.2	4.1	133	1.17	0.78	111.5	1.5	high	8.4	9.1		26	10.3
PS	79	F	13	1	15.7	1.9	89	1.71	1.1	82.96	2.5	high	10.7	6.1		29	9.9
PS	39	F	12	1	17.2	3.9	144	1.16	0.93	110.5	1.8	high	8.9	6.8		26	10.2
PS	65	M	10	1	20.6	3.9	152	1.43	1.15	151.4	1.3	high	9	5.2		22	8
PS	42	M	11	1	18.5	3.9	190	1.9	0.99	125.2	2.1	high	9.4	3.4		37	11.2
PS	79	M	9	1	19.2	3.2	147	1.26	0.84	94.85	1.8	high	8.2	4.7	0.94	32	12.7
PS	78	F	9	1	15.6	3.4	116	1.36	1.17	121.9	1.5	high	7.3	5.1		30	10.8
PS	70	F	8	1	18.5	2.9	172	1.42	0.82	65.98	2.1	high	9.7	5.3	0.06	24	10.9
PS	81	F	8	1	24.7	3.3	110	1.6	0.73	102.4	2.5	high	10.3	6.7		24	10.6
PS	66	M	8	1	22	3.6	117	0.74	0.96	113	1.5	high		4		17	9.4
PS	72	M	7	1	14.9	3.5	94	1.54	0.64	76.36	2.1	high	10.2	6.2		30	9.9
PS	82	M	7	1	17.2	3.5	146	1.39	0.88	112.1	2.1	high	8.4	3.7	0	21	10.1
PS	70	F	6	1	22.8	1.8	187	1.56	1.38	76.44	1.5	high	9.4	2.9	0.12	21	10.9
PS	62	F	6	1	28	3.8	122	1.19	0.94	103.4	1.5	high		4.1	8.38	19	10.6
PS	60	M	6	1	21.6	4	138	1.35	0.93	144.8	2.1	high	10.1	5.2		23	11.4
PS	78	F	5	1	17.4	3.1	171	2.03	0.89	107.9	1.8	high	9.3	5.3	0.22	30	8.4
PS	66	F	5	1	24.2	3.4	147	1.4	0.77	84.54	1.8	high	8.4	3.8	0.2	19	11
PS	45	M	5	1	19.6	4.6	165	1.16	0.8	121	1.5	high	8.6	10.9	0.36	16	8.9
PS	61	M	4	1	25.4	3.8	128	1.44	0.96	95.19	1.6	high	9	4.5		46	9.3
PS	47	M	4	1	20.1	3.7	89	1.42	0.74	86.05	1.8	high	8.8	6.7	0.6	25	10.9
PS	55	M	4	1	20.1	1.9	128	1.3	0.75	112.3	1.8	high	8.8	4.4	0.15	25	9.7
PS	81	M	4	1	23.3	3.6	140	1.44	0.9	87.7	1.6	high		2.2	0.79	22	9.1
PS	61	M	4	1	24	3.8	209	1.7	0.78	122.1	1.5	high		4.8	0.05	27	10.3
PS	70	F	4	1	16.8	3.6	185	1.28	0.79	85.14	2.5	high	8.9	7.5		28	11.4
PS	66	M	3	1	23.8	3.8	156	1.32	0.79	82.21	1.8	high		2.9	0.21	29	11.1
PS	80	M	3	1	15.2	3.8	127	1.23	0.96	94.7	1.8	high	10.4	4.3		29	10.2
PS	41	M	3	1	26.1	3.2	162	1.23	0.94	124.6	1.8	high		7	0.48	35	11.5
PS	78	F	3	1	23.1	3.1	182	1.57	1.13	98.09	1.8	high	10.5	3.1		26	10.4
PS	74	F	3	1	22.4	3.8	160	1.45	0.92	57.29	1.3	high		6.4	0.02	28	11.5
PS	56	F	3	1	19.4	4	128	1.09	0.88	72.63	1.8	high	7.3	3.8	0.4	49	7
PS	51	F	2	1	22	2.8	136	1.8	0.71	93.74	1.3	high	8.4	3.7	0.2	32	9.6
PS	78	M	2	1	21.8	3	138	1.19	0.96	140.7	1.5	high	8.9	4.1	0.05	16	10.4
PS	82	F	2	1	18.5	3.6	191	1.47	1.12	44.63	1.5	high	9.2	6.3	0.02	20	9.4
PS	56	M	2	1	24.7	4	118	1.42	0.94	155.2	1.5	high	8	4.6	0.05		10.4
PS	69	F	9	1	15.8	4.1	176	1.32	0.96	120.2	1.3	high	8.5	3.9	0.1	26	10.5
PS	56	F	8	1	15.2	4.8	325	1.27	1.23	98.25	2.1	high	10.7	5.8	0	23	9.7
PS	69	F	4	1	19.6	4.1	210	1.41	0.91	101	2.1	high	8.2	1.9	0	20	10.1
PS	52	F	28	1	19.9	2.1	136	1.83	0.95	121.2	2.1	high	8.8	5.6	0	35	11.6
PS	58	M	27	1	20.7	4.4	181	2	0.79	84.78	1.9	high	9.7	3.6		29	10
PS	58	M	27	1	19	3.5	172	1.69	0.94	114.1	1.8	high	9.7	4.1	0.1	45	10.4
PS	52	F	24	1	23.9	2.6	141	1.84	0.96	100.8	1.8	high	9.3	4.3	0.1	27	9.2
PS	68	F	22	1	20.4	2	216	2.96	0.76	112.9	1.9	high	10.9	5		26	10.1

PS	60	M	22	1	17.3	2.5	155	2.48	0.92	121.3	1.9	high	9.2	3.6		39	10
PS	65	F	21	1	19.1	1.7	177	1.67	0.72	106.7	1.8	high	8.3	5.4	0	27	10.7
PS	62	F	19	1	18.2	3.1	141	1.2	0.72	84.67	1.8	high		5.3	0.04	17	10.9
PS	38	M	18	1	17.4	2.6	155	1.56	0.74	93.56	1.8	high	8.7	6.3	0.02	27	8.6
PS	70	F	16	1	17.9	3.3	208	1.59	0.91	101.4	2.2	high	8.9	3.8		19	11.9
PS	73	F	15	1	19.4	3		1.37	1.02	101.9	1.8	high	7	7	0.14	24	11.8
PS	67	M	12	1	22.2	3.3	158	1.63	0.91	125.4	1.3	high	8.4	3.5	0.3	29	11.2
PS	61	M	12	1	25.2	3.6	162	1.43	0.77	142.2	2.2	high	9.5	4.6		26	13.8
PS	34	M	11	1	22	3.1	180	0.97	0.73	108.8	2.1	high	9.3	4.7	0.04	23	11.2
PS	42	F	9	1	17.6	3.7	249	1.25	0.82	117.1	1.8	high	9	4.5	0.09	24	9.7
PS	61	M	9	1	24.8	3.3	133	1.46	0.93	95.24	2.1	high	9.8	6.4	0.06	25	11.3
PS	60	F	9	1	20.9	3.3	110	1.43	0.91	115.8	2.1	high		5.1	0.01	22	11.2
PS	62	F	8	1	23.7	3.8	162	1.23	0.82	98.58	1.8	high	10.3	5.3	1.36		8.8
PS	71	M	8	1	18.9	3	208	1.51	0.84	98.97	1.3	high	10	5.8	0.67	26	10.6
PS	73	M	8	1	19.9	3.4	133	1.27	1.07	141.6	1.3	high	8.8	5	0.05	27	10.9
PS	73	F	7	1	19.4	3.5		1.99	0.62	113.5	1.8	high	9	5.4	0.25		10.7
PS	48	M	6	1	22.8	3.3		1.38	0.71	104.1	2.1	high	8.7	5.4		38	11.7
PS	65	M	6	1	27.2	3.6	146	1.3	0.81	104.4	1.8	high	8.7	8.4	0.06	33	12.5
PS	67	M	6	1	22.7	3.7	203	2.01	0.78	79.87	2	high	8.2	5.1	0.4	35	11.7
PS	78	F	6	1	16.2	2.8	161	1.2	0.97	96.98	1.5	high		6.7	3.39	13	10.3
PS	63	M	6	1	24.1	3.4	185	1.78	0.85	94.12	2.1	high	10	3.9		21	9.9
PS	61	M	6	1	20.3	3.7		1.25	0.62	65.69	1.8	high	9.2	7.9		34	9.3
PS	66	M	5	1	22.8	3.1		0.97	0.65	132.5	2.1	high	8.6	9.7	0.45	26	10.5
PS	60	M	5	1	20.7	3.3	175	1.06	0.82	104.8	2.2	high	9.1	5.8	0	32	11.2
PS	64	M	5	1	20.9	4.1	115	1.52	0.76	117.5	2.1	high	8	3.5	0.46	28	9
PS	47	M	5	1	20.3	3.6	135	1.74	0.9	105.8	1.3	high		4.5	1	33	12.2
PS	74	F	5	1	27.1	2.2	141	1.31	0.52	68.85	1.6	high	9.1	6	0.15	21	9.8
PS	65	M	3	1	19.9	3.2	111	1.56	0.88	95.29	1.6	high	9.5	5.6	0.12	25	10.3
PS	32	F	3	1	19.5	4	135	1.75	1.26	99.95	1.5	high		4.1	0.08	27	9.8
PS	85	F	3	1	20.7	2.7	196	1.57	1.14	96.19	1.5	high	9.1	1.6	0.83	25	10.8
PS	50	M	2	1	27.2	3.7	129	1.49	0.93	108.3	1.8	high	8.4	6.6		39	8.6
PS	59	M	3	1	35	3.8		1.87	0.86	89.5	2.1	high	8.7	6.1		34	11.1
PS	57	M	8	1	20.1	3.6	163	1.26	0.84	134.4	1.8	high	10.5	6.4	0.05	30	9.7
PS	76	F	4	1	22	4	157	1.45	0.8	108.7	2.1	high	8.9	6.5	0.29	29	12.3
PS	70	F	30	1	21.2	3.9	142	1.23	0.96	77.97	2.1	high	10.3	6.2	0.05	28	10.4
PS	62	M	28	1	18.8	3.5	125	1.96	0.75	59.84	1.4	high	8.4	4.3	0.4	30	8.9
PS	56	M	26	1	18.5	3.8	133	1.62	0.91	118.1	1.4	high		3.6			8.6
PS	59	F	25	1	17.5	3.7	121	1.36	0.89	92.47	2.2	high	9.4	4.3			10.1
PS	57	M	20	1	18.5	3.6	176	1.49	0.9	116.7	1.3	high	8.6	4.8	0.05	23	11.9
PS	61	M	18	1	17.2	3.8	195	1.14	0.73	102.6	2.2	high		8.1	0.14		12.1
PS	64	M	18	1	20.2	4.1	135	1.26	0.83	97.19	2.2	high	9.4	5.2			10.2
PS	40	M	17	1	21.6	4.4	176	1.41	0.74	91.07	1.3	high	9.9	5.6	0.05	34	9.9
PS	56	M	17	1	18.7	4.4	131	1.8	0.8	104.6	2.2	high	11.3	3.7	0.06	32	14.4
PS	59	M	14	1	22.5	3.5	134	1.41	0.91	89.55	1.5	high	9	4.9	0.05	29	
PS	58	M	12	1	22.6	4.2	107	1.26	0.85	88.08	1.8	high	8.1	5.6	0.3	30	11.3
PS	59	F	12	1	18.2	3.5	144	1.43	0.79	107.2	1.8	high	9	7.2		25	8.9
PS	57	F	12	1	21.5	4.1	103	0.68	0.66	99.67	2.2	high		5.6		31	11
PS	74	F	12	1	22.9	3.5	155	0.95	0.69	96.15	2.1	high	8.3	6.2		34	10.3
PS	69	F	11	1	18.6	4	105	1.59	0.73	100.8	1.4	high	9.2	5.5	0.1	39	10.9
PS	57	F	11	1	24.7	3.8	208	1.26	0.83	88.15	1.3	high	8.1	3.4	0.05	20	5.5
PS	53	M	9	1	16.6	4	180	1.49	0.97	81.72	2.1	high	9.2	4.8	0.05	25	8.6
PS	54	M	9	1	20.4	4.5	109	1.29	0.72	105.6	1.8	high	7.9	2.6	3.36		11
PS	77	M	9	1	17.7	4.3	124	1.71	0.86	105.5	1.8	high	8.2	5.5	0.25	51	11.7
PS	62	M	9	1	18.3	4.1	95	1.17	0.76	90.97	2.5	high		7			9.9
PS	66	F	9	1	21.1	4.1	123	1.34	0.82	113.1	2.1	high		4.6	0.09	22	9.9
PS	70	F	8	1	24.3	3.9	174	1.86	0.78	70.58	1.8	high	9.4	8.8	0.2	28	9.8
PS	73	M	8	1	20.7	4	71	1.27	0.9	89.99	1.8	high	8.6	4.8	0.81	24	11.4
PS	67	M	8	1	17.9	4.1	101	1.51	0.9	113.4	1.8	high	8.4	7.4	0.31	26	11.2
PS	57	M	8	1	23.6	4.4	160	1.35	0.91	79.99	1.8	high	9.1	4.8	0.1	25	11.6

PS	46	M	7	1	22.6	4.2	119	1.36	0.89	116	1.8	high	8.6	3.2	0.05	18	10.9
PS	59	M	7	1	25	4.3	200	1.53	0.61	47.17	1.8	high	8.6	3.9			12.2
PS	63	M	6	1	18.4	3.5	96	1.48	0.75	108.5	1.8	high	9	6.3	0.2	25	11.8
PS	74	F	6	1	18.2	4.3	51	1.32	0.75	113.9	1.8	high	9.1	4.1	0.2	21	10.4
PS	47	M	5	1	19.9	3.9	142	1.75	0.81	93.19	2.5	high		5.7			10.8
PS	68	F	5	1	17.6	3.7	176	1.34	0.71	97.89	2.2	high	9.4	5	0.07	28	13.1
PS	63	M	4	1	28.5	3.9	155	1.47	0.89	68.1	1.3	high	9	3.1	0.1	20	10.7
PS	44	M	4	1	19.8	3.5	137	0.96	0.69	90.56	1.5	high	9.2	2.8	3.8	29	11
PS	50	M	4	1	27.5	4.1	139	1.2	0.87	67.59	1.6	high		5.1	0.23	30	10.8
PS	76	M	3	1	21.4	3.9	204	0.9	0.6	64.71	2.1	high	8.8	10.7	0.04	25	8.8
PS	68	F	3	1	20.3	3.7	166	1.35	1.1	110	2.2	high		5.8		28	11.2
PS	55	M	3	1	22.7	3.7	136	1.48	0.73	87.21	1.8	high		4.9	0.08	30	10.1
PS	42	M	2	1	20.1	4.1	268	1.71	0.74	73.56	1.8	high	8.6	3.9	0.5	26	11.2
PS	67	M	2	1	25.1	3.7	154	1.19	0.66	52.28	2.1	high		5.4			9.7
PS	63	F	17	1	17.6	3.7	115	1.45	0.76	89.7	1.3	high		5.6	0.1	23	8.8
PS	59	F	6	1	18.3		80	1.38	1.13	116.8	2.1	high		5.1	0.06	35	7.3
PS	71	F	25	1	20.7		147	1.4	0.98	90.7	1.8	high		4.9		26	10.3
PS	46	M	13	1	21.8		165	1.14	0.85	99.78	1.8	high		6.2		30	9.2
PS	66	M	10	1	21.3	6.1	189	1.19	1.15	126	2.1	high	8.9	7.2	0.27	28	8.9
PS	55	F	5	1	18.5		164	1.52	1	106.5	1.6	high	8.6	6.3	0.09	31	10.2
PS	71	F	9	1	16.3	3.4	181	1.55	0.69	111.6	1.4	high	8.8	4.5	0.83	24	11
PS	29	M	10	1	19.9			1.28	0.95	119	1	high		5.7	2.42	32	12.7
PS	74	F	6	1	26.3		137	2.08	0.83	114.6	1.6	high	9.4	4.2	0.04	31	9.4
PS	67	F	29	1	19.3		179	1.14	1.29	91.41	1.8	high		7		28	10.4
PS	61	F	8	1	18		189	1.46	0.99	89.59	2.1	high	8.8	7.5	0.05	27	11.7
PS	84	F	5	1	18.3		178	2.32	0.81	90.57	1.5	high	8.7	3.6	0.05	19	8.9
PS	70	M	3	1	22.1		95	1.82	0.92	110.3	1.5	high	9.2	5.1	0.05	24	11.8
PS	62	F	2	1	16.5		109	1.34	1.18	119.1	1.6	high	10.5	6.5	0.13		
PS	85	F	3	1	21.9		116	1.72	0.63	120.2	1.6	high	9	6.3	0.04	31	12.8
PS	71	M	9	1	17.2	3.5	168	1.12	0.68	97.22	2.1	high	8.9	6.2	1.78	26	9.4
PS	67	M	35	1	17.3	3.7	110	1.36	0.8	58.87	2.1	high	9.5	6.8	0.89	27	9.7
PS	67	F	21	1	20.1	3.6	109	1.18	1.25	119.7	1.8	high		6.1	0.9	18	10.7
PS	58	F	19	1	19.5	3.9	131	1.43	0.83	100	1.8	high		5.7	0.08		11.5
PS	69	M	18	1	20.2	3.3	127	1.04	0.89	116.8	1.8	high		5.6		34	9.9
PS	48	F	9	1	17.6	3.5	136	1.51	1.24	97.65	1.3	high	9.2	2.5	0.2	26	8.1
PS	59	M	4	1	24.4	4.3	185	1.33	0.93	124.4	1.5	high	8.4	6	2.14	23	10.4
PS	78	M	4	1	25.5	3.2	110	1.4	0.89	125.5	2.5	high	8.6	9.1	0.03	27	12.7
PS	57	F	3	1	23.6	3.6	141	0.94	0.98	142.7	1.5	high		5.2			9.6
PS	47	F	3	1	19.6	4.1	164	1.42	0.97	103.8	1.5	high		6	0.1	27	11.3
PS	56	M	2	1	25.1	4	131	1.63	0.81	104.4	2.1	high		7.8	0.2	25	10.6
PS	62	M	2	1	19.4	4.3		1.77	0.96	86.64	1.8	high	8.4	4.6	0.01	23	8.1
PS	40	M	2	1	23.9	4	189	1.41	0.96	105.1	1.8	high		4.6		33	9.5
PS	66	F	36	1	18	3.6	178	1.88	1	90.46	1.9	high	9.5	4.7		32	9.6
PS	53	M	9	1	22.4	4	148	1.59	0.84	120.1	1.3	high	8.4	5	0.4	30	8.3
PS	48	F	8	1	18.1	4.2	134	1.55	1.2	73.91	1.5	high	9.8	6.4	0	19	10.6
PS	59	F	6	1	20.9	4.2	164	0.98	1.04	121.7	1.8	high	7.6	3	0.05	30	9.5
PS	64	M	6	1	22.5	3.9	201	1.37	0.77	125.6	1.5	high		4.6	0.1	23	11
PS	74	M	2	1	21.5	3.6	231	2.02	0.9	118.4	1.8	high	8.3	4.9	0.1	22	8.4
PS	58	M	2	1	21	3.4	63	1.57	0.85	150.5	1.6	high	7.5	6.9	0.32	29	9.4
PS	57	F	2	1	24	4.2	144	1.46	0.91	97.09	1.1	high	8.8	7.3	1.9	33	10.7
PS	73	F	2	1	19	3.4	112	1.62	0.48	75.49	1.5	high		5.3	0.12	28	10.3
PS	53	F	2	1	29.5	3.8	116	1.11	0.8	88.99	2.2	high		5.7		34	10.2
PS	62	F	2	1	19.5	3.7	197	1.32	0.87	101.9	2.2	high		6.9		34	9.2
PS	43	M	9	1	21.6	4.3	135	1.59	0.85	123.5	1.5	high		6	0.1	22	11
PS	64	M	5	1	18.7	4.1	118	1.65	1.14	107	1.5	high	10.3	6.5		35	9
PS	55	M	2	1	26.2	3.9	193	1.38	0.6	109.7	2.1	high	8.4	7.2			11.5
PS	77	F	7	1	21.4	3.8	146	1.08	1.04	125.2	2.2	high		4.1		27	10.8
PS	55	M	6	1	19.5	4	191	1.42	0.78	111	1.1	high	8.9	5.6	0.35	34	11
PS	60	F	4	1	29	3.9	146	1.63	1.28	73.44	1.5	high	8.7	4.6	0.1	28	11.1

PS	73	M	3	1	20.3	3.6		1.26	0.82	89.61	1.8	high	9.8	4.6		22	
PS	59	M	6	1	22	3.6	143	1.09	0.89	115.7	1.8	high		7.3	0.05	33	10.8
PS	55	F	32	1	18.5	4.7	165	1.19	1.04	65.1	1.3	high	8.4	7.4	1.54	21	9.1
PS	66	M	30	1	21.3	4.2	175	1.57	1.09	105.1	1.8	high	8.6	6.4	0.09		11.6
PS	56	F	30	1	17	4.5	135	1.89	1.03	79.24	1.9	high	9.5	5		26	11
PS	55	M	30	1	23.4	3.7	164	2.05	0.68	99.52	1.9	high	9.8	5.6		29	10.7
PS	59	F	28	1	21.2	4.1		1.53	0.94	90.14	1.4	high	9.4	5.6	0.16	29	10.3
PS	66	M	27	1	18.4	4	163	1.85	1.24	90.82	1.4	high	9.1	7.8	0.16		10.9
PS	73	F	27	1	21.1	4.5	145	1.46	1.04	120.9	2.2	high	9.7	4.8	0.52	35	10.4
PS	43	M	25	1	20.2	4.4	122	2.13	1.23	85.18	1.6	high	8.7	4		32	11.5
PS	57	M	24	1	18.5	3.4	214	1.81	0.82	115.5	1.8	high	11	5.7	0.03	26	10.1
PS	67	M	22	1	22.9	3.6	147	1.03	0.86	113.2	1.8	high		4		22	8.9
PS	67	M	22	1	22.7	4.4	173	1.95	1.04	108.4	1.4	high	9.2	5.4	0.13		10.3
PS	68	M	20	1	21.3	4	110	0.83	1.02	102.6	2.1	high	9.7	9	0.58	35	7.8
PS	69	M	20	1	20.7	3.4	135	1.93	0.8	94.2	1.4	high	9.8	5.5	0.36		10.1
PS	80	F	19	1	17.7	3.6	167	2.6	0.76	120.7	1.6	high	10.2	2.9		31	10.3
PS	58	M	19	1	18.2	4	90	1.46	1.07	92.3	2.1	high	9.7	4.1		24	10.1
PS	69	M	18	1	17.7	3.6	231	1.3	1.11	118	2.2	high		5.9		34	11.1
PS	45	M	18	1	21	4.2	195	0.61	1.31	96.29	2.1	high	9.1	6.6	0.32	15	14.1
PS	58	F	17	1	21.2	3.9	155	1.73	1	89.98	2.1	high	8.6	5.3		37	11.8
PS	59	M	17	1	29.9	4.4	177	1.47	0.89	74.86	1.3	high	10	2.5	0.1	26	10.8
PS	73	M	16	1	19.6	4.2	130	1.05	0.89	113.1	1.8	high	9.1	7.8	0.1	28	12.4
PS	60	M	15	1	21.6	4.5	92	1.42	0.81	113.5	1	high	9.4	4.4	1.14	34	9.1
PS	66	M	15	1	21.9	3.9	146	1.07	0.92	120.8	2.2	high		8.1		32	10.6
PS	52	M	15	1	24.7	4.9	151	1.1	1.18	102.1	1.8	high	9.8	4.6	0.11	22	10.5
PS	53	M	14	1	24.6	4.2	176	1.34	1.11	115	1.5	high		3.1	0.2	15	9.6
PS	44	F	14	1	24.8	3.7	227	2.3	1.04	108.7	1.4	high	8.9	4	0.12	38	9.7
PS	41	M	12	1	21.5	4	206	1.5	0.73	82.09	1.4	high	9.8	5	0.05	29	11.2
PS	78	M	11	1	20.9	6.7	154	1.76	0.99	104.1	1.8	high	9.4	4.9	0.01	31	10.4
PS	60	M	10	1	21	6.8	164	1.48	0.99	91.39	1.4	high	9.1	3.6	0.05	25	11.1
PS	68	F	10	1	24.7		174	1.55	0.85	139	1.4	high	8.5	4.3	0.3	23	11.6
PS	67	M	10	1	20.9	6.8	117	1.6	1.02	124.7	2.2	high		6.8		28	10
PS	68	F	9	1	20.1	4.7	137	1.22	0.94	103.5	1.8	high	9.3	5.7	2.44	32	10
PS	65	M	8	1	23.5	6.3	140	0.78	0.98	124.8	1.8	high		3.4		19	9.7
PS	42	M	8	1	24.9	6.2	146	1.37	0.51	96.63	2.1	high	8.7	4.7	0.15	22	11.7
PS	71	M	7	1	17.6	3.8	202	0.97	0.99	93.15	1.5	high		3.7	5.2	41	6.8
PS	42	M	7	1	27.7	4.3	144	1.13	0.89	125.2	2.2	high		7.2		29	9.7
PS	36	M	6	1	22.3	4.4		1.44	0.85	97.75	1.4	high	12.7	5.1		26	9.5
PS	60	M	6	1	22.6	4.2	195	1.67	0.78	116.7	1.3	high	8.5	2.9	0.12		10.1
PS	47	F	6	1	19.1	4.4	165	1.18	0.66	119.3	1.8	high	10.2	6.3	0.1		12.8
PS	47	M	6	1	24.4	3.4	173	1.57	0.89	105.9	1.8	high	9.6	7.4			10.4
PS	70	M	6	1	22.3	4.2	105	1.26	0.79		2.2	high	7.6	4.9	0.16	31	
PS	61	M	5	1	27.4	4.2	125	1.4	0.82	98.15	1.5	high	7.9	2.6	0	47	8.6
PS	71	M	5	1	25.2	3.7	260	1.58	0.9	88.64	1.8	high	9	5.7		25	11.3
PS	73	M	5	1	20	3.3	88	1.23	0.83	41.9	2.2	high		4.8		34	8.1
PS	50	M	5	1	24	2.4	139	1.44	1.44	55.28	2.1	high	7.6	4.7	1.75	23	8.9
PS	32	M	4	1	22			1.56	1.17	127.3	2.1	high	9.5	6.1	0.05	23	11
PS	80	F	4	1	17		182	1.82	0.98	94.11	2.2	high	9.2	4.8	0.05	20	11
PS	51	M	4	1	20.6	3.9	155	1.31	0.88	108.3	2.1	high	9	7.9	0.23	31	9.2
PS	31	M	4	1	24.4	3.8	177	1.28	0.73	106.9	2.1	high		3.5	0.36	22	9.6
PS	73	M	3	1	23	3.8	152	1.36	0.77	106.7	1.5	high	8.7	4.1	0.1	28	10.1
PS	61	M	3	1	26.7	4.2	167	1.34	0.85	132.4	1.8	high	9.7	6	0.12	22	9.9
PS	57	M	3	1	20.7	6.8	138	1.82	0.89	139.1	1.8	high	7.4	5.4	0.01	34	10.7
PS	47	M	3	1	25.3		150	1.16	0.93	78.29	1.5	high		6.7	0.05		11.1
PS	58	M	2	1	24.5		137	1.28	0.79	93.52	1.8	high	8.7	3.4	0.02	21	10
PS	26	M	2	1	31.7		112	1.33	0.78	101.3	2.1	high		5.8	0.1	29	11.7
PS	64	M	19	1	23.6	3.9	172	1.17	1	122.6	1.5	high	9	6.2	0.06	28	11
PS	67	M	6	1	18.6	3.8	157	1.36	0.97	97.09	1.8	high	8.8	4.7	0.02	29	10
PS	77	M	5	1	19.4	3.8	149	1.25	0.88	105.4	2.2	high	8.9	2.6	0.25	25	10.2

PS	55	F	20	1	23.9	3.4	156	1.39	1.1	101.2	2.2	high	9.8	4.1	0.05	28	8.9
PS	58	F	19	1	20	4	176	1.33	0.81	128.4	2.2	high	9.7	4.1	0.09	29	10.3
PS	78	F	16	1	19.3	3.9	151	2.32	0.93	118.4	2.3	high	9.5	5.3	0.02	28	10.1
PS	64	F	14	1	25.8	3.8	181	1.74	0.78	89.91	2.1	high	9.8	4.3	0.16	25	9.9
PS	68	M	14	1	17.3	4.2	117	1.64	0.93	118.5	2.1	high	9.6	5.1	0	24	10.1
PS	68	M	13	1	23.2	3.9	148	1.39	0.88	148.7	1.8	high	9.5	5.5	0.17		11.1
PS	59	F	11	1	22.9	3.9	139	1.57	1.09	118.6	1.4	high	9	4	0.13	24	10.9
PS	64	M	11	1	19.1	3.8	146	1.35	0.96	100.4	2.2	high	10	7.3	0.1	29	10.9
PS	64	F	9	1	20.6	3.7	125	1.56	1.1	118.6	2.2	high	8.8	5.2	0.05	29	10
PS	58	M	9	1	21.6	4	157	1.35	1.02	131.8	2.2	high		4.6	0.13	30	11.3
PS	71	M	8	1	23.2	3.9		0.91	0.85	99.03	1.3	high		1.1	2.22	38	9.7
PS	61	F	8	1	19.7	4	150	1.32	0.93	128.1	2.2	high	10.3	5.6	0.05	14	10.9
PS	57	F	7	1	20.6	3.8	187	1.12	1.06	104.9	1.1	high		5.1	0.6	32	10.7
PS	84	M	7	1	19.2	3.3	183	1.46	0.9	107.1	1.3	high	9.7	6.1	0		10.6
PS	69	F	7	1	19.6	3.4	178	1.53	1.19	103	1.8	high	10.1	7.7	0.71	23	9.9
PS	73	M	6	1	22.7	4		1.41	0.85	123.6	2.2	high		6			9.8
PS	76	M	6	1	18.9	3.7	226	2.07	1.04	95.83	1.5	high	9.1	4		23	10.9
PS	58	F	6	1	21.6	4.4	176	2.1	1	110.4	2.1	high	10.2	5.6		30	10
PS	67	M	6	1	18.5	3.9	116	1.62	0.8	126.5	2.1	high		5.6	0.1	28	12.3
PS	65	F	5	1	17.8	3.4	158	1.69	1.12	102.9	1.4	high		5.5	0.02	27	10.7
PS	79	M	5	1	19.1	3.3	181	1.59	0.81	110.1	1.8	high	9.4	3.5	0.02	18	10.7
PS	64	F	5	1	18.5	4	184	1.95	0.95	85.35	1.5	high	9.1	4.7		36	8.6
PS	74	F	4	1	19.6	3.8	220	0.98	0.84	83.15	2.1	high	8.6	4.8	0		11.1
PS	78	M	4	1	19.7	3.9	116	1.5	1	116.7	1.5	high	8.5	6.4	1.72	21	9.6
PS	68	M	3	1	19.8	3.6		1.23	1.18	147.4	2.2	high		5.5			9.9
PS	65	M	3	1	24.2	3.8	128	1.3	1.01	92.92	1.5	high		3.7	0.7	7	11.2
PS	68	M	3	1	21	3.9	132	1.68	0.58	55.01	1.8	high	8.4	2	0.14	19	11.1
PS	49	M	2	1	25.2	3.9	209	1.4	1.02	81.46	1.5	high	9.7	4.5	0.05	23	10.4
PS	69	M	2	1	24.4	3.7	273	1.59	1.02	95.99	1.8	high		4.1	0.02	23	11.9
PS	70	M	2	1	19.9	4	159	1.25	0.67	81.48	1.3	high	9.3	4.6	0.5		10.6
PS	73	M	9	1	21	3.4	139	2.02	0.79	109.9	2.1	high	8.8	4.6		30	9.3
PS	69	M	12	1	18.4	3.7	180	1.51	1.1	94.92	1.8	high	9.4	5.1	1.13	18	8.3
PS	57	M	4	1	24	3.9	163	1.33	1.29	134.8	1.3	high	8.2	5.9	0.05	24	11.9
PS	69	F	4	1	22.4	3.6	135	0.95	0.88	96.99	1.3	high	8.1	4.5	0.6	23	7.4
PS	68	F	34	1	19.3	3.8	149	1.19	0.84	85.98	2.1	high	8.3	4.9	0.15		12.3
PS	59	M	14	1	22.1	4	115	1.78	0.94	92.29	1.8	high	9.6	4.7	0	22	8.7
PS	60	F	11	1	17.9	3.8	126	1.35	0.87	91.11	1.8	high	10.4	4.4			9.9
PS	62	F	11	1	24.4	3.7	183	1.84	0.69	90.77	1.5	high	9.7	5.4	0.1	27	11
PS	38	M	8	1	21.2	4.7	180	1.74	1.21	136.5	1.3	high	8.6	3	0.16	33	9.3
PS	63	F	5	1	18.4	4	145	1.59	0.99	125.1	1.8	high	9.3	6.6		34	10.9
PS	86	M	3	1	21.3	3.6	150	1.56	0.82	81.8	1.5	high	8.6	2.9	0.05	33	11.8
PS	37	M	3	1	28.6	3.7	187	1.3	1.38	110.5	1.8	high		4.9	1.64	24	9.7
PS	61	M	2	1	19	3.4	136	1.65	0.59	9.55	1.6	high	8.3	5.3	4.07		8.5
PS	84	M	2	1	20.3	3.7	156	1.49	0.64	64.24	2.1	high	9.4	7.3	0.31	33	11.2
PS	76	F	14	1	17.2	4	93	1.26	0.97	79.45	1.3	high	10.5	6	0.05	29	11.5
PS	65	M	12	1	23.3	3.4	126	1.41	0.99	132.1	1.3	high	10.4	5.6	0.06	30	10.7
PS	51	F	12	1	22.3	3.8	106	1.2	0.8	122.2	1.4	high	9.7	6.2		25	
PS	51	M	10	1	19.4	4.5	198	1.44	0.91	117	2.2	high	8.9	6		33	
PS	58	M	5	1	19.6	3.9	114	2.43	1.64	96.55	1.8	high	8	5.2	0.05	30	9.8
PS	61	M	3	1	21.6	3.9	129	1.14	1.15	111.9	1.8	high	8.8	6.3	0.05	26	10.9
PS	59	M	3	1	23.5	4.3	144	1.43	0.97	131	1.5	high	9.1	5.9	0.44	24	11
PS	60	M	3	1	22.4	3.9	119	1.5	0.89	123	2.1	high	7.7	5.3	0.07	26	9.2
PS	62	M	29	1	23.2	4.8	195	1.54	0.98	109.9	2.2	high	8.8	6.6			12.1
PS	68	F	29	1	22.5	3.8	148	1.47	0.65	105.1	1.8	high		5.4	0.05		10.6
PS	68	F	28	1	25.6	3.9	131	2.1	0.75	93.67	1.9	high	8.7	3.3		32	9.9
PS	67	F	27	1	17.6	4.4	160	1.63	1.17	111.1	1.8	high	9.3	5.1	0.1	31	10.6
PS	40	M	26	1	22.8	4.4	114	1.52	1.18	108.5	1.9	high	10.8	4.3		39	11.1
PS	66	M	25	1	23.5	4	111	1.21	0.84	113.2	1.6	high	9.9	5.8			11
PS	50	M	25	1	18.9	4.5	130	0.74	1.24	125	1.8	high	8.9	5.7	0.05	18	10.2

PS	59	M	24	1	19.9	4.4	147	1.94	0.93	122.7	1.9	high	7.8	4.3		24	10.6
PS	58	M	23	1	23.3	4.3		1.38	0.83	118.8	2.1	high	8.1	4.7	0.14	28	10.4
PS	59	M	23	1	24	4.2		1.11	1	107.8	2.2	high		6			9.7
PS	65	F	22	1	20.2	4.3	151	2.67	0.87	147.9	1.9	high	9	3.9		30	9.1
PS	67	M	22	1	15.3	4.3	130	1.55	0.9	99.67	1.6	high	7.8	4.8			9.6
PS	55	M	22	1	25.3	4.6	286	1.86	1	112	1.5	high	9.2	6.3		32	10
PS	57	F	20	1	23.8	4	136	1.34	0.83	97.23	2.1	high		5.5		26	10.8
PS	61	F	18	1	16.8	3.9	186	1.46	1.28	112.6	1.3	high	8.4	5.4	0.05	24	9.7
PS	60	M	16	1	17.3	4.5		1.35	0.98	107.3	2.2	high		4.8			10.9
PS	64	F	16	1	22.3	4.3	223	1.53	0.84	98.78	1.6	high	9	4.9	0.74	31	11
PS	47	F	15	1	15.3	3.7	146	1.39	1.13	90.36	1.8	high	8.9	7.4	0.05	18	10.4
PS	37	M	15	1	26	4.5		1.46	1	145.6	1.8	high	7.6	2.1		27	10
PS	55	M	14	1	24	4.3	148	0.82	1.03	131.7	2.1	high		4.3	0.01	23	10.1
PS	27	F	14	1	17.8	4.4	170	1.57	1.3	96.94	1.8	high		4.8	0.05	20	11
PS	66	F	14	1	22	4.2	137	1.36	1.02	129.2	1.6	high	7.3	5.3	0.07	25	10
PS	53	F	13	1	13.7	4	137	1.62	0.86	56.21	1.8	high	8.6	6.4	0.03	30	11.7
PS	46	F	12	1	18.5	4.4	181	1.56	0.95	103.2	1.1	high		4.9	0.09		10.2
PS	57	M	12	1	19.7	4.3	163	1.41	1.06	144.6	1.5	high	9.6	6.6	0.33	29	12
PS	54	M	12	1	17.5	4.2	138	1.33	1.17	110.1	1.8	high	8	5.1	0.63	22	10
PS	46	F	11	1	21.3	4.1	129	1.45	1.27	124.8	1.8	high	9.8	4.8	0.32	15	11.6
PS	41	M	11	1	23.6	4.6	122	1.48	0.94	103.5	1.8	high	8.6	5.5		28	10.1
PS	62	F	11	1	20.5	4.5	183	1.55	1.11	130.9	1.1	high	8.4	5.9	0.13	24	11
PS	66	F	10	1	26.2	4.5	118	1.79	1.16	81.3	1.8	high	8.9	4			9.8
PS	64	M	10	1	25.7	4.6	204	1.23	0.67	104.5	2.1	high	8.1	4			10.6
PS	63	F	10	1	26	4.2	134	1.09	1.05	98.33	1.5	high	9.6	3.9	0.01	41	11.4
PS	34	M	9	1	17.3	4.9	115	1.88	1.07	102.4	1.8	high	7.4	5	0.1	24	8.5
PS	36	M	9	1	20.8	4.6	174	1.39	1.01	104.1	1.8	high	8.3	5.4	0.04	26	12.8
PS	75	M	9	1	19.8	4.2	141	1.48	1	123.7	2.1	high	8.2	8.7	0.79	26	11
PS	61	M	8	1	18.6	3.9	98	1.53	1.17	106.8	2.2	high	9.4	8.6		43	10.9
PS	37	M	8	1	19.8	4.3	150	1.39	0.98	108.2	2.2	high	9.1	9.3		32	10.8
PS	59	M	8	1	24.2	4.5	123	1.17	0.92	133	2.1	high	9.3	6.3	0.2	19	10.8
PS	54	F	8	1	23.4	4.6	143	1.4	1.17	101.5	1.3	high		5.7	0.12		11
PS	36	M	8	1	17.4	4.7	136	1.47	0.92	117.7	1.3	high		4.9	0	23	10
PS	49	F	8	1	20	4.7	99	1.38	1.11	120.8	1.5	high	8.5	5.4	0.05	28	11.6
PS	64	F	7	1	19.4	4.4	172	1.24	1.11	110.9	1.3	high	9.4	5.4	0.09	26	10
PS	71	M	7	1	18.7	4.4	225	0.94	0.83	99.91	1.3	high	8.2	5.7	0.21	26	9.4
PS	58	F	7	1	19.9	4.6	151	1.14	1.11	129.6	1.5	high		4	0.12	21	10.3
PS	47	M	7	1	25.2	4.1	141	1.33	0.99	121.6	1.8	high	7.9	6.4		29	11.3
PS	56	M	7	1	22.1	4.7	143	1.25	1.05	127.7	2.1	high	9	5.2	0.06	21	9.9
PS	78	F	5	1	20.9	4.7	147	1.2	0.96	121.8	1.8	high	8	6.8		25	11.4
PS	38	M	5	1	31	4.4	127	1.44	1.16	135	1.5	high	10.2	5.1	0.1	30	8.9
PS	82	M	5	1	19.9	4.5	143	1.52	0.89	106	1.3	high		5.9	0.05	37	12.2
PS	79	M	5	1	31.2	4.3	147	1.58	0.85	105.7	1.5	high		3.9	0.05	23	11.4
PS	58	M	4	1	18.6	4.5	163	1.42	0.71	116.8	1.8	high		4.4	0.05	20	11
PS	77	F	4	1	21.2	4.6	127	1.34	0.81	93.49	2.1	high	9.9	7.5	0.08	29	10.1
PS	80	M	3	1	20.2	3.7	149	1.34	0.48	64.7	1.6	high	9.3	5.6	0.07	25	11.3
PS	68	M	3	1	22.8	4.3	174	1.26	0.86	124.7	1.8	high	9.2	2.5	0.12	25	10.1
PS	37	M	3	1	29.6	4.7	148	1.55	1.09	112.5	1.8	high	9.3	7.6	0.9	33	9.9
PS	64	M	3	1	22.1	4.8	201	1.35	0.71	140.9	1.8	high	9.4	7.9	0.5	29	
PS	59	F	3	1	18.8	4.7	160	1.53	1.11	93.27	1.6	high		4.3	0.1	30	14.4
PS	59	M	3	1	25.7	4.1	132	1.43	1.08	106.7	1.3	high	8.8	5.1	0.06	22	9.2
PS	62	M	2	1	20.5	4	122	1.33	0.76	73.92	1.8	high	9.8	4.4	0.08	27	
PS	56	M	2	1	21.8	4.2	147	1.37	0.97	113.1	2.5	high		7.9		27	10
PS	52	M	2	1	25.5	4.3	106	1.26	1.03	74.95	1.3	high	8.7	4.6	0.12	2	
PS	69	F	2	1	26.7	3.9	145	1.81	0.84	125.1	1.6	high		6	0.19	30	9.2
PS	52	M	2	1	24.5	4.7	124	1.3	1.05	100.6	1.5	high	6.6	4.3	0.07	21	7.6
PS	56	M	5	1	20.3	4.6	127	1.26	0.93	117.2	1.8	high	8.9	5.3		27	10.9
PS	73	M	13	1	22.7		94	1.52	0.93	140.3	2.1	high		5.4	0.05	31	9.9
PS	61	M	10	1	28.7		189	1.16	0.97	115.7	2.1	high		7.3	0.1	27	12.7

PS	77	M	9	1	21		200	2.1	1	118	1.9	high		5	0.15	28	10.1
PS	68	M	2	1	18.6	4	157	1.55	1.28	110.9	2.1	high	9.1	5.7	2.08	23	9.6
PS	55	M	28	1	20.4	4.6	196	1.14	1.08	60.79	2.1	high	9.9	5.9	0.03	13	9.9
PS	60	F	22	1	15.9	4.9	169	1.53	0.67	88.2	2.1	high	9.1	5.5			
PS	69	F	18	1	20.6	4.1	97	1.56	0.79	108.6	2.5	high	9.1	4.3		23	12.8
PS	54	F	9	1	19.4	4.1	126	1.22	1.14	84.8	1.8	high	11.8	5.1	0.1	27	10
PS	59	F	7	1	36.4	4.2	98	1.36	1.01	106.6	1.6	high	8	5.3	0.05	41	12.2
PS	73	F	2	1	19.2	4.5	152	1.6	0.81	116.4	1.6	high		4.2	1.15	29	8.8
PS	58	M	34	1	17.8	3.9	154	2.47	1.19	112.9	1.6	high	10	5.5		28	11.6
PS	68	M	30	1	22	3.5	169	0.89	0.86	109	2.2	high	11	6.2		45	9.5
PS	60	M	29	1	18.4		114	1.07	0.85	99.2	2.5	high	9.5	7.4	0.1	30	12.1
PS	54	M	24	1	21.1	3.9	126	1.39	0.88	113.3	1.9	high	9.9	5.8		34	9.6
PS	69	M	20	1	18.7	3.8	130	1.49	0.87	148.7	1.6	high	10.1	5.1		20	10.5
PS	67	F	18	1	17.4	4	97	1.32	1.21	102.1	1.8	high	10.5	5.7		36	10.3
PS	60	M	14	1	21.5	3.8	133	1.16	0.89	113	1.5	high	9.1	4.7	0.31	26	13.2
PS	68	M	13	1	22.2	3.6	171	1.33	0.96	81.5	1.6	high	9	5.5		22	10.7
PS	64	M	13	1	24.6	3.6	131	1.13	0.95	139.2	1.4	high	8.7	6.8	0.02	21	11
PS	73	F	13	1	26.5	3.9	124	1.77	0.82	157	1.8	high	9.6	6	0.02	26	11
PS	65	F	11	1	19.2	4.2	150	0.96	1.01	113.1	1.4	high	9.1	5.3	0.03	31	10.5
PS	78	F	11	1	21.3	2.3	140	1.19	1.08	129.4	1.8	high		5.5	0.18	26	11
PS	91	F	10	1	16.6	3	103	1.16	1.5	137.9	2.2	high	7.3	4.6	0.05	37	8.6
PS	58	M	8	1	18	3.8		0.97	1.16	128.4	1.5	high		3.1	0.03	28	8.4
PS	75	M	8	1	21.2	4.1	156	1.41	1.05	116.9	2.1	high	8.7	7.2	0.11	28	11.1
PS	73	F	7	1	27.7	3.7	214	1.61	0.88	119.1	1.8	high	9.9	4	0	38	8.4
PS	84	F	7	1	19.8	3.3	143	1.1	0.65	118.1	1.3	high	8.7	4.6	0		11.8
PS	76	F	7	1	18	3.6		1.61	0.76	94.95	1.5	high	8.4	6.6	0.1	25	11
PS	65	F	6	1	24.2	3.8	171	1.51	1.04	109.8	2.2	high	9.7	5	0.1	31	9.5
PS	68	M	6	1	20.3	4.2	175	0.54	0.9	137.3	0.8	high		2		2	13.3
PS	67	F	6	1	25.2	3.5	156	1.63	0.9	100.7	1	high	8.9	7		29	9.6
PS	59	M	6	1	22.5	4.1	135	1.1	1.25	124.7	1	high		5.6		21	11.5
PS	68	F	5	1	22	3.5	140	1.16	1.2	156.8	1	high	6.2	8.4		27	9.4
PS	68	F	5	1	21.1	4	148	1.25	0.72	91.04	1.8	high	9.8	6.7	0.11	23	8
PS	82	M	5	1	20.2	3.3	141	1.7	0.74	98.54	1.1	high		2.7	0.7	30	11.5
PS	57	F	5	1	19.4	4	188	1.81	1.08	75.64	1.5	high	8.5	5.1	0.37	29	11
PS	70	F	5	1	22.1	4.1	133	1.44	0.9	135	1.4	high	8.9	5.4		17	8.9
PS	68	M	4	1	25.6	3.8	201	1.64	0.67	146.5	1.5	high	9.4	5.1	0.01	24	9.7
PS	62	M	4	1	23.4	3.8	121	1.37	0.8	88.84	1.8	high	10.5	4.1		28	10
PS	68	M	4	1	22.2	2.2	120	1.49	0.76	125.1	2.2	high		3.6	0.05	31	10.9
PS	84	F	4	1	20.1	3.7	183	1.81	1.13	170.9	1.8	high		5	0.13	28	10.3
PS	65	F	4	1	18.1	3.9	103	1.22	0.86	145.3	1	high	9.1	5.4	0.08	26	9.3
PS	63	M	3	1	23.3	3.9	114	1.38	0.93	110.3	2.1	high		6	0.1	28	10.6
PS	68	F	3	1	29.3	3.7	120	1.42	1.14	120.6	1.8	high		5	0.1	27	8.8
PS	65	F	3	1	21.2	3.4	151	1.42	0.87	126.4	1.8	high		5.9	0.04	30	11.9
PS	75	F	2	1	22.4	3.9	208	1.36	1.2	80.75	2.1	high		6.2			8.3
PS	81	F	2	1	19.7	3.8	189	1.44	0.99	79.72	2.2	high		4.4	0.45	37	10.6
PS	56	F	2	1	19.1	3.8	177	1.42	1.14	78.64	1.8	high	8.6	5	0.1	38	9.1
PS	76	F	2	1	22.5	3.7	133	1.56	0.87	102.8	1.8	high	8.7	4	0.64	32	8.7
PS	66	M	2	1	19.8	3.8	79	1.46	0.85	86.28	1.5	high	8.4	5.9		22	9.6
PS	60	F	27	1	15.5	4.3	147	1.25	1.08	81.6	1.3	high	10.2	4.9	0.07	26	12.1
PS	60	F	3	1	19.3	4	94	1.46	1.06	81.52	1.6	high	7.7	4.6	0.01	38	9.2
PS	73	M	21	1	20.4	3.7	171	1.46	0.79	74.9	2.2	high	8.5	4.8	0.21	38	7.8
PS	78	M	2	1	27	4.2	124	1.39	1.22	124.7	2.1	high		3.3	0.2	23	10.2
PS	66	F	3	1	21.1	3.6	263	1.56	1.08	78.67	1.8	high		5.2	0.11	23	11
PS	61	F	3	1	25.2	3.9		1.57	0.71	59.45	1.5	high	9.3	5	0	27	11.6
PS	62	F	4	1	24.8	2.1	210	1.59	0.83	46.77	1.5	high		3.4	0.8	18	9.2
PS	56	F	19	1	20	3.7	132	1.77	1.06	128.8	2.2	high		4.9	0.02	26	10.6
PS	59	M	6	1	20.9	4.2	101	1.61	0.98	63.97	1.6	high	8	4.3	0.17	23	9.7
PS	75	M	7	1	18.9	4.1	179	1.74	0.77	136.4	1.8	high		5.3		27	10.4
PS	67	F	26	1	19.3	4.1	198	2.2	1.35	109.3	1.8	high		5.6		34	9

PS	61	M	18	1	21.3	3.9	185	1.36	0.88	126.4	2.1	high	9.9	7.2	2.3	30	11.8
PS	74	F	11	1	21.2	3.8		1.59	0.89	111.5	1.3	high	10.7	5.3	0.8	36	10.9
PS	66	M	11	1	17.8	3.7	171	2	1.05	95.43	1.8	high		6.8		29	10.2
PS	60	F	9	1	20.6	3.8		1.26	1.03	103.2	1.5	high	9.1	5.3	0.25	24	10.6
PS	45	M	9	1	25.5	3.7	175	1.53	0.86	91.76	1.5	high		4.4	3.17	23	12.2
PS	73	M	8	1	15.8		153	2.54	1.25	121.9	1.8	high		6		27	12
PS	69	F	8	1	21.8	3.5	159		1.08	142.9	1.3	high	9.1	4.9	0		11.2
PS	47	M	7	1	20.2	4.1	152	2.39	0.94	108.6	1.8	high		6.5		34	11
PS	56	M	3	1	26.7	1.9	120	1.08	0.87	89.83	1.5	high	8.5	4.7	0.16	37	10.2
PS	44	M	2	1	30.7	4	184	1.28	0.87	99.57	1.6	high		7.6	0.06	27	9.9
PS	69	M	17	1	19.7	4.2	148	1.4	0.83	108.4	2.1	high	8.8	2.7	0.06	29	10.1
PS	82	M	4	1	20.2	3.9	126	1.12	0.77	73.06	2.1	high	8.9	6.4	0	21	9.9
PS	60	M	4	1	27.2	4	152	1.41	0.74	122	2.1	high	9.4	5.6	0.1	30	11.2
PS	61	M	3	1	21	4.2	152	1.34	0.92	93.11	1.8	high	9.4	4.5			10.4
PS	53	M	22	1	18.6	3.6		1.78	0.8	96.13	1.6	high		3.8	0.06	28	9.1
PS	81	M	4	1	29.4	3.6	146	2.05	0.88		2.2	high		5.9		26	11.4
PS	58	M	3	1	19.9	3.1	132	1.06	0.53	59.19	1.5	high	9.9	3.5	0.2	23	10.7
PS	71	F	3	1	19.4	3.6	175	1.33	1.1	97.76	1.6	high	8.8	6.3			
PS	34	F	3	1	24.3	3.5	171	0.96	0.86	74.57	2.1	high	9.3	4.2	0.26	30	10.4
PS	82	F	2	1	21.7	3.5		0.97	1.17	182.7	1.3	high		3.6	0.01	27	10.2
PS	51	F	29	1	17.2	3.9	118	1.46	1.01	132.4	2.5	high	10.3	5	0.15	26	11
PS	67	M	20	1	21.2	3.8	141	1.63	0.8	118.5	2.2	high	9.5	4.6	0	28	11
PS	62	F	18	1	13.6	3.2	125	1.34	0.84	58.25	2.1	high	9.6	7.5		27	11.1
PS	73	M	8	1	25.1	4.2		0.98	0.89	125.2	1.6	high		3.4	0.14	34	10.4
PS	40	M	6	1	22.8	4		1.09	1.19	133.7	2.1	high		5.4	0.13	26	9
PS	60	M	6	1	20	3.9		1.26	1.1	124	1.6	high		2.4	0.04	30	9.5
PS	56	M	3	1	18.6	3.9	111	1.25	0.97	108.8	1.5	high		5.2	1.02	28	11.5
PS	45	M	3	1	19.9	4.1	101	1.22	0.89	92.69	2.1	high		6.1	0	25	11.2
PS	60	F	3	1	19.6	3.9		1.28	0.71	114.2	1.6	high		3.8	1.52	37	9
PS	50	M	2	1	24.3	4.3	169	1.04	0.9	88.52	1.5	high	9.3	6.1	0		11.5
PS	79	F	2	1	26.8	3.7	180	1.14	0.68	83.27	1.5	high	9.8	6.9	0.05	27	9.6
PS	47	M	16	1	22.3	3.9	138	1.24	1.11	109.1	1.5	high	8	7.9	0.08	20	11.4
PS	68	F	13	1	20.8	4.2	96	1.41	1.04	113.4	1.5	high	9.2	6.7			9.9
PS	61	F	10	1	19.7	2.9	179	1.55	1.01	101.7	1.5	high	9.4	4.6	0.2	26	13.1
PS	63	M	9	1	21.9	4	169	1.77	0.95	109.7	1.3	high		4.2	0.17	28	11.6
PS	52	M	9	1	22	4.1	182	1.91	1.05	132.7	1.5	high	10	7.1		32	11
PS	76	M	9	1	19.5	3.3	140	1.45	1.05	131.4	1.3	high		3.5	0.2	32	9.7
PS	73	F	7	1	19.5	3.7	221	1.68	0.9	131.6	1.8	high	9.4	7.9	0.03	30	10.5
PS	51	M	6	1	22.5	2	154	1.41	0.84	105.4	1.8	high	8.8	7	0.3	26	11.3
PS	68	M	4	1	17.9	2.3	140	2.05	0.92	121.4	1.8	high		7.3		32	8.3
PS	77	F	3	1	19.4	2.3	199	2.04	0.79	103.1	1.8	high		6		31	10
PS	67	F	3	1	18.4	1.9	152	1.73	0.85	99	1.6	high	9.6	3.8	0	30	9.9
PS	39	M	2	1	22	4	66	1.28	0.93	114.1	2.1	high	8.6	2.9	0.2	27	12
PS	61	F	36	1	18.8	3.4		1.72	0.96	84.4	1.5	high	8.4	4.2	3.2	32	10
PS	61	M	31	1	15.8	4.5	178	1.66	0.89	107.5	1.6	high	9.7	5.1			10.7
PS	53	M	30	1	19.7	3.8	142	1.41	0.86	92.71	2.1	high	9.1	3.6		23	10.4
PS	60	M	30	1	21.3	3.7	161	1.82	1.2	114.8	1.6	high	6.5	7.3		20	10.6
PS	66	F	29	1	18.7	4.4	135	1.64	1.2	90.09	1.8	high	9.4	5.7			10.6
PS	58	M	28	1	21.6	4.4	128	1.82	0.88	107	1.3	high	8.9	6.3	0.3	30	12.1
PS	65	F	27	1	21.2	3.6	130	1.88	1.1	130.9	2.1	high	8.7	8.7	0.28	32	12.2
PS	55	M	25	1	19.7	3.7	100	1.31	1.12	118.1	2.2	high	9	5			10.4
PS	68	F	25	1	17.4	4.3	151	1.58	1.14	126.2	2.2	high	8.8	4.6	0.34		10.9
PS	58	M	25	1	20.6	3.6	199	1.19	0.88	133.1	1.8	high	9.9	5.2	0.06	17	9.8
PS	45	F	25	1	18.7	3.6	146	1.5	1.05	105.2	2.1	high	9.9	7.6	1.75	39	13
PS	78	F	24	1	18.9	3.8	137	1.48	1.1	135.8	2.1	high	10	5.5	0.06	38	13.6
PS	68	F	24	1	19.6	3.7	149	1.36	1.06	129.5	1.6	high	9.4	7.4			10.6
PS	61	F	22	1	21.9	4.2	159	1.27	1.28	143.9	2.2	high	8.3	5.8			9.2
PS	54	F	22	1	13.3	4	183	1.39	0.83	84.01	1.8	high	8.8	4.1			8.9
PS	58	M	22	1	21.3	4	216	1.8	1.07	137.6	1.4	high	10.2	5.2		25	10.4

PS	74	F	21	1	19.7	3.1	240	1.37	0.83	130.7	2.2	high	8.7	5.8	2.36	28	8.8
PS	56	F	19	1	19.9	4.1	201	1.5	1.15	151.2	1.3	high	9.5	4.9		18	8.9
PS	68	M	19	1	23.4	3.3	150	1.26	0.82	81.62	1.8	high	8.4	3.6	0.08		10.1
PS	73	F	18	1	20.1	4	176	1.75	1.08	124.4	2.1	high	8.9	8.2	0.3	34	11.8
PS	72	F	18	1	16.4	3.9	143	1.33	0.74	111.6	2.1	high	8.9	4.8	0.05	34	11.3
PS	58	F	18	1	22.9	3.6	142	1.28	0.78	131.3	1.4	high	8.5	5.5			12.2
PS	39	M	18	1	23.4	4	172	2.04	0.88	116.8	2.1	high	9.3	5.1		30	8.2
PS	59	F	18	1	21.7	4.2	82	1.21	0.97	135.5	2.1	high	8.3	7	0	39	10.4
PS	81	F	17	1	16	3.7	121	1.62	0.98	117.7	1.8	high	8.2	4.7			10.4
PS	40	M	16	1	23	4.2	155	1.57	0.91	80.44	1.8	high	9.2	5.8	0.1	32	11.6
PS	65	M	16	1	17.8	4.1	127	1.36	0.95	102.2	1.5	high	9.1	6.6	0.2	23	9.6
PS	73	M	16	1	21.1	3.7	195	1.44	0.74	130	2.1	high	8.8	9.4	0.1	33	9.9
PS	68	M	16	1	24.7	4.1	159	1.76	0.92	120.9	2.1	high	9.1	4.5	0.2	24	10.6
PS	48	F	15	1	16.7	3.9	151	1.24	0.91	111.2	1.3	high	10.7	5	0.03	28	12.2
PS	54	F	15	1	27.3	3.9	99	1.51	1.05	121.9	2.1	high	8.2	4.5	1.03	29	8.9
PS	35	M	15	1	22.6	4.5	113	1.45	1.06	117.8	2.1	high	8.9	4.8	0.1	27	9.5
PS	33	F	14	1	17.7	4.2		1.45	1.21	107.6	1.6	high	9.5	5.6	0.75		14.3
PS	64	F	14	1	24.6	3.7	92	1.49	0.82	120.9	2.1	high	8.9	6.2	0	28	10.5
PS	60	F	14	1	18.2	3.9	114	1.63	0.94	127.1	2.1	high	10.1	6.9	0	30	10.9
PS	64	M	13	1	21.9	4.2	147	1.67	0.91	136.9	2.2	high		5.2		32	10.5
PS	59	F	13	1	21.3	4.1	149	1.61	1.16	125.9	2.1	high	9.3	6.7	0.4	25	10.1
PS	47	M	13	1	20.3	4.4	184	1.47	0.88	116.4	1.8	high		5.8		27	11.3
PS	49	F	13	1	18.9	4.7	204	1.86	0.96	93.04	1.1	high	10.1	5.4	0.1	27	10
PS	45	M	13	1	20.6	4.2	132	1.6	0.99	89.95	2.1	high	7.9	3.7	0.1	31	11.2
PS	69	F	13	1	20.2	4	145	1.3	1.19	163.8	2.1	high	9.1	3.1	0.4	27	11.2
PS	78	M	13	1	24.3	3.9	143	1.64	0.79	100.4	1.8	high		5.7		24	10
PS	47	F	12	1	20.9	4.2	160	1.39	1.14	121.3	1.5	high	10.7	3.8	0.16	26	10.7
PS	87	F	12	1	24.3	3.4	100	1.58	0.81	132.2	1.3	high	8.3	4.2	2	21	9.1
PS	41	F	12	1	16.1	4.4	139	1.21	1.08	127.2	2.1	high	8.2	5.5	0.1	24	10.8
PS	57	M	12	1	20.4	3.8	136	1.42	0.98	111.2	1.4	high	9.2	5.5	0.27	33	9.9
PS	63	F	12	1	31.3	4	177	1.37	1	130.1	2.1	high	9.6	5.1	0.1	31	12.5
PS	53	M	12	1	22.9	3.9		1.3	1.11	126.8	1.8	high	8.3	4.3	0.1	26	10.1
PS	78	M	12	1	18.9	4.2	134	1.39	1.04	143.2	1.4	high	10.3	3.7		32	10.8
PS	45	M	12	1	18.5	4.7	204	1.75	0.76	88.02	1.5	high		4.4	0.6	25	10.1
PS	39	M	12	1	17.8	4.3	125	1.14	1.09	135.6	1.8	high	8.9	6	0.26	23	11.8
PS	67	F	11	1	23.9	3.7	177	1.92	1.09	143.1	1.8	high	9	3.5		29	14
PS	59	M	11	1	22	3.8	204	1.52	0.97	128.9	2.1	high	9	5.4	0.09	18	10.1
PS	70	M	11	1	21	3.8	133	1.44	0.71	135.7	2	high	9.8	4.3	0.03	33	10.1
PS	76	M	11	1	25.6	4.3	125	1.91	0.89	154.4	1.5	high	9.2	1.9	0.08	31	8.5
PS	61	M	10	1	20.3	3.8	148	1.43	0.85	74.73	1.5	high	9	6.5	0.3	22	9.4
PS	47	F	10	1	17.6	3.8	178	1.76	0.78	150.2	1.8	high	9.2	4	0.05	24	11
PS	60	F	9	1	20.6	4.4		1.46	1.18	158.1	1.1	high	9.5	6.8	0.1	28	12.7
PS	62	F	9	1	25.5	4.2	138	1.37	1.17	106.8	1.8	high	9.1	7	0.32	50	8.3
PS	47	M	8	1	21.3	4.3	231	1.46	1.08	132.3	2.1	high	10	5.6		24	8.8
PS	51	F	8	1	18.5	4.3	176	1.26	0.94	111.6	1.1	high		4.9	0.2	29	11.5
PS	68	F	8	1	17.5	4.2	141	1.16	0.98	100.3	1.5	high		5.5	0.2	29	10.1
PS	62	F	8	1	30.9	4	53	1.3	0.87	123.8		high		6.7	0.06	27	11.3
PS	56	M	7	1	25.8	4	133	1.76	0.8	98.7	1.3	high		4.2	1.9	35	8.8
PS	71	M	7	1	22.9	3.5	167	1.84	0.96	97.15	1.8	high	10.3	6.4		28	10.5
PS	66	M	7	1	24.9	4	140	1.04	0.99	187.5	1.3	high		4.3	0.1	30	10.4
PS	64	F	7	1	19.9	3.9	150	1.1	0.94	120.9	1.1	high		2.4	0.2	21	10.3
PS	74	M	7	1	24.1	4.4	136	1.4	0.9	168.5	1.3	high		6	0.2	41	10.6
PS	49	M	7	1	26.9	4.5	129	1.76	0.96	115.8	1.1	high	9.4	4.6	4.3	33	10.2
PS	54	M	7	1	19	4	135	0.97	0.91	89.86	1.4	high	9.3	6.1	0.3	31	12.1
PS	63	M	6	1	21.2	3.8	119	1.28	0.94	141.1	1.5	high		6.8	0.1	29	10.7
PS	74	M	6	1	18.3	4.2	204	2.04	1	135.1	1.8	high	9	4.1			10.9
PS	56	M	6	1	20.3	4.4	138	1.21	1.01	139	1.8	high	9.3	4.9	0.06	22	12.5
PS	72	F	5	1	16.8	4.3		1.83	1.07	132.9	1.5	high	8.6	4.8	0.9	27	9.2
PS	60	F	5	1	19.2	4.7	125	1.7	1.14	120.2	1.3	high		4	0	28	10.1

PS	67	M	5	1	24	4.2	146	1.3	0.92	116.1	1.8	high	8.1	5	0.1	25	8.5
PS	73	F	4	1	22.4	3.9	64	1.14	0.73	97.29		high		4.6	5.94		9.5
PS	72	M	4	1	22.3	3.5	214	1.4	0.95	101	1.8	high		4.2	0.1	29	10.7
PS	73	F	4	1	19.5	3.5	227	1.31	0.59	122	1.3	high	9.1	6.3	0.66	30	10.5
PS	38	M	4	1	27.7	4.6	180	1.43	0.71	72.46	1.5	high		4.9	0	26	11.4
PS	74	M	4	1	23.6	3.8	111	1.58	0.87	130.1	1.8	high		6.3	0	39	10.6
PS	29	M	4	1	19.2	5	177	1.81	1.4	107.2	1.8	high	8	4.9	0.1	28	11.4
PS	60	M	3	1	19.4	4.4	142	2.04	1	129.2	1.8	high	9.7	5.2	0.1	29	8.9
PS	54	F	3	1	17.8	3.6	172	1.64	0.7	119.7	1.3	high	9.7	5.8	0.05	23	10.1
PS	68	M	3	1	20.8	3.4	144	1.7	0.81	61.46	1.4	high	9.1	6.9	0.18	25	10.4
PS	56	F	3	1	21.3	3.4	172	2.04	0.99	89.39	1.8	high	10.5	6.2	0.27	23	10.5
PS	51	F	2	1	18.1	4.5	241	1.39	0.84	123.5	1.1	high	9.6	5.6	0.32	17	11.3
PS	74	F	2	1	26.2	3.8	139	1.86	0.72	106.9	1.5	high	8.2	3.7		26	10.2
PS	67	M	2	1	36.3	3.7	149	1.11	0.96	107.5	1.8	high	7.8	3.3	0.07	29	9.9
PS	63	M	2	1	16.6	3.9		1.45	1.04	98.36	1.8	high	7.8	4.3	0.1	24	11
PS	72	M	2	1	21.6	3.7	73	1.18	1.08	76.27	1.6	high	8.8	4.4		28	8.6
PS	58	M	6	1	23.3	4.1	121	1.67	1.1	150.8	1.3	high		6.5	0.2	28	9.9
PS	56	M	20	1	23.1	4.2	134	1.86	0.99	121.2	1.3	high	8.4	6.1	0.3	42	8.8
PS	42	F	10	1	16.5	4.1	163	1.42	1	89.37	1.5	high	8.3	4.7	0	21	11.1
PS	64	F	25	1	17.9	3.4	184	1.61	1.21	137.6	1.1	high		4.5	0.4	39	10.7
PS	74	M	21	1	17.6	3.4	177	1.18	1.2	116.2	1.8	high	9	4.5	4.87	33	11.3
PS	63	F	21	1	24.3	3.5	166	1.54	1.17	123.8	1.3	high		6.5	0	33	10
PS	59	M	21	1	24.9	3.7	142	1.28	1.1	116	1.3	high	8.2	5.2	0.3	25	10
PS	71	M	20	1	22.9	3.7	127	1.09	0.76	133.6	1.3	high	9.8	3	0.3	29	6.8
PS	62	M	18	1	24	3.5	187	1.55	0.83	130.8	1.4	high	8.2	4.7	0.05	14	12
PS	65	F	13	1	22.6	3.6	105	1.59	1	139.1	1.5	high		4.5	0	24	9.9
PS	61	M	9	1	26.6	3.9	175	1.62	1.12	137.2	1.1	high		2.4	0	24	10.5
PS	53	M	8	1	18.7	4	187	1.97	0.98	97.3	1.8	high	10.1	4.3	0	26	9.5
PS	39	M	8	1	19.1	3.7	180	1.19	0.98	125.4	1.3	high		5.5	0.3	39	10.6
PS	81	F	7	1	21.4	3.6	169	1.89	1.14	118.8	1.6	high	8.8	6.2		37	9.4
PS	55	F	7	1	33.8	4	140	2.17	1.05	134.9	1.8	high	9.8	4.1	0.1	30	9.6
PS	59	F	7	1	19.3	3.8	123	1.67	1.21	94.66	1.5	high	8.6	6.2	1.9		10.4
PS	43	F	4	1	19.6	3.8	140	1.54	0.95	43.39	1.6	high	8.9	6.9	0.08	27	11.2
PS	67	M	4	1	23.7	3.8	125	1.09	0.98	90.86	1.5	high		6.2		27	9
PS	79	M	3	1	24	3.5	216	2.36	0.79	156	2.1	high	9.9	8.8	0.1	29	9.2
PS	46	F	3	1	21.6	4	154	1.73	0.78	57.01	2.1	high	8.7	3.9	0.14	19	10
PS	71	F	2	1	25.9	3.4	114	2.1	0.99	101.8	1.4	high	9.7	6	0	22	7.4
PS	75	M	2	1	24.9	3.7		1.31	0.78	108.5	1.5	high	9.4	6.6	0.01	24	10.2
PS	47	F	7	1	17.3	3.4	160	1.99	1.09	125.5	1.3	high	8.4	6.5		27	4.5
PS	69	M	34	1	20.1	2.7	168	1.06	0.93	78.52	1.8	high	8.5	4.6	0.3	31	10.9
PS	54	M	29	1	21.2	4.1	115	1.08	0.86	110.7	1.8	high	9.5	4.9	1	22	10.7
PS	57	M	27	1	16.9	4	156	1.76	1.13	100.7	1.4	high	8.7	5.2		34	11.4
PS	70	M	26	1	19.1	4	200	1.56	0.87	115.8	1.3	high	9	3.6	0.3	18	9.4
PS	58	M	26	1	20	4.1	171	1.64	0.88	107.6	1.6	high	9.2	5.7	0.35	23	11.3
PS	78	M	26	1	17.5	3.5	139	1.16	0.8	66.95	1.8	high	9.4	7.5	0.3	29	10.3
PS	58	M	24	1	21.3	4.4	161	1.45	1.02	106.5	1.6	high	8.5	3.4	1.52	26	9.6
PS	63	M	24	1	18.6	3.7	162	1.26	1.13	92.25	1.6	high	8.9	5.1	0.05	22	10.3
PS	67	M	24	1	15.9	3.9	190	1.38	1.11	89.06	2.1	high	9.1	4.7	0.38		10.1
PS	51	M	24	1	27.6	4.3	148	1.31	0.87	92.62	2.2	high	10.1	5.5	0.4		10.9
PS	72	F	24	1	16.5	3.5		0.95	1.23	91.85	1.6	high		5.6	0.09	26	9.3
PS	60	F	23	1	21	3.8	146	1.4	0.95	86.22	1.6	high	8.8	5.8	0.05	22	10.7
PS	60	M	23	1	20.6	3.6	121	1.18	1.16	119.5	1.9	high		4.2			8.9
PS	55	F	22	1	18.5	3.9	150	1.15	1.29	125.4	1.9	high	8.8	6.1	0.28	26	12.6
PS	58	F	19	1	21.6	4	143	1.36	1.19	162	1.3	high	9.8	7.3	0.08	31	10.7
PS	38	M	19	1	19.5	4	214	1.34	0.93	95.55	1.6	high		5.9	0.59	22	11
PS	64	F	19	1	16.1	3.6	128	1.43	0.74	108.4	1.4	high	9.1	5.5	0.31	27	7.9
PS	73	F	18	1	25.5	3.6	108	1.07	1.07	120	2.2	high	9	10.1			10.6
PS	49	M	18	1	17.1	4.4	143	1.57	1.01	83.34	1.4	high	8.5	5.8		31	9.6
PS	60	M	18	1	22.3	4.1	136	1.79	0.83	123.1	2.1	high		5.7	0.1	42	12.5

PS	68	M	18	1	19	3.7	135	1.66	0.92	130.2	1.4	high	10.1	4.8	0.44	43	11.2
PS	59	F	17	1	19.9	4	132	1.07	1.05	143.8	1.8	high	10.2	2.4	0.16		11.7
PS	69	M	16	1	26.2	3.9	149	1.3	0.88	122.6	1.8	high	7.6	7.3	0.11	32	10.7
PS	66	M	16	1	20.3	4.2	118	1.39	0.87	120.6	1.8	high	8.5	5.2	0.01	31	10.3
PS	40	F	16	1	21.4	3.9	120	1.95	0.88	123.2	1.8	high	8.8	3.3	0.46	43	11.7
PS	61	F	15	1	21.6	3.9	180	1.5	0.87	138.5	1.8	high	9	5	0.02	21	10.5
PS	58	F	15	1	24.1	3.9	135	1.57	1	113.6	2.1	high		4.9	2.5	42	8.2
PS	66	M	14	1	25.3	3.7	146	0.94	0.72	119	2.1	high	8	7.7		21	7.7
PS	52	M	14	1	22.9	3.6	139	1.41	1.12	119.7	1.5	high	8.5	3.7	1.1	22	8.6
PS	72	M	14	1	20.4	3.5		1.16	0.77	103.8	2.1	high	8.8	7		27	10.7
PS	42	M	14	1	22	4.4	159	1.6	0.98	134	1.4	high		4.9	0.27	25	10.5
PS	76	M	13	1	19.3	3.2	107	1.35	0.53	94.25	1.8	high		4.8	0.06	26	9.9
PS	48	M	13	1	19.9	4.2	135	1.41	0.93	125	1.8	high	8.9	6.7	0.13	27	9.7
PS	71	F	13	1	24.4	3.3	155	1.29	0.56	104	1.8	high	9.4	5.5	0.1	27	10.3
PS	67	F	12	1	20.6	3.6	117	1.01	0.99	103.1	1.8	high	8.8	5.5	0.05	29	10.8
PS	64	M	12	1	24.5	3.3	160	1.57	1.08	133.9	2.2	high	10.1	5.3	0.06	24	8.6
PS	47	M	12	1	22.2	4	203	1.56	0.87	98.29	1.8	high	11.3	4.9	0.06	24	10.2
PS	48	M	12	1	22.3	4.2	115	1.35	1.04	109.3	1.8	high	9.2	6		27	9.4
PS	74	M	12	1	20.9	3.7		1.43	0.84	151.6	1.8	high	7.3	5.6	0.18	9	8.2
PS	75	M	11	1	25.6	3.7	161	1.47	0.62	119.3	1.8	high	9.5	5.8		28	9.4
PS	60	F	11	1	19.1	4	200	1.75	0.94	90.79	1.3	high		4.5	0.05		10.6
PS	60	M	11	1	20.3	4.8	190	1.39	1.01	126.1	1.8	high		6.2	0.73	31	11.7
PS	61	M	11	1	22.3	3.9	189	1.66	0.98	84.19	1.8	high	8.3	5	0.4		9.5
PS	46	M	11	1	19	4.2	145	1.13	0.85	128.1	1.4	high		3.6	0.05	24	9
PS	71	F	10	1	21.8	4.4	110	1.52	0.89	97.53	1.8	high	9.2	5.3	0.02	24	10.3
PS	57	M	10	1	20.1	4.1	128	1.71	0.73	123.7	1.4	high	8	5		28	12.6
PS	40	M	10	1	24.1	3.7	173	2.14	0.81	117.4	1.6	high	8.2	5.8	0.1	20	10.8
PS	52	M	10	1	21.2	4.3	113	1.23	0.93	106.1	1.8	high	9.4	5.8	0.1	29	11.5
PS	38	F	10	1	13.6	3.7	153	1.27	0.93	82.3	1.8	high	9.8	7.2	0.08	19	10.2
PS	36	M	10	1	20.3	3.9	178	1.9	0.86	93.83	1.4	high	9.3	5.3	0	19	10.4
PS	46	M	10	1	17.4	3.7	154	1.58	0.84	98.75	1.6	high	8.7	4.6	2.99	47	12
PS	49	F	10	1	21.8	4.4	173	1.29	0.86	89.07	1.8	high	8.6	5.2	0.2	20	10
PS	54	M	10	1	19.1	3.9	168	1.31	1.08	117.4	1.6	high		6.1	0.13	23	7.9
PS	61	F	9	1	17.7	3.8	62	1.31	0.72	115.1	1.5	high		5.8	0.1	36	11.4
PS	76	M	9	1	20	3.8	224	1.91	0.96	119.3	1.8	high	10.7	5.6	0.04	26	10.8
PS	53	M	9	1	20.4	4.4	229	1.23	0.91	129.5	1.5	high	9.9	5.2	0.05	13	10.2
PS	56	F	9	1	23.1	4.5	123	1.78	1.03	105.4	1.8	high	8.5	6.3	0.11	29	10.4
PS	68	F	9	1	21.5	3.6	170	1.08	1.04	116.9	1.8	high	8.9	4.4	0.2	28	10.9
PS	58	M	8	1	17.4	3.6	183	1.3	0.73	101.9	2.2	high	8.1	6.2	0.18	35	11.6
PS	64	M	8	1	20.3	3.7	140	1.47	0.84	99.16	2.2	high	9.7	7.7	0.07	27	11.6
PS	61	M	8	1	25.8	3.9	215	1.42	0.89	101.8	2.2	high	8	7.2	0.02	6	11.2
PS	65	M	8	1	18.2	3.2	139	1.35	0.92	137	2.2	high	8.3	5.7	0.03	34	11.5
PS	60	M	8	1	23	3.5	153	1.26	0.7	101.6	1.5	high		5.4	0.9	26	9.3
PS	64	F	8	1	19.6	3.7	139	1.07	0.95	103.6	2.2	high	8.6	4.5	0.02	12	11.5
PS	45	M	8	1	21.4	4.1	211	1.42	0.9	118.6	1	high	9.3	5.1	0.05	29	11.7
PS	50	M	8	1	18.5	4.5	171	1.58	0.92	123	1	high	9	4.1	0.05	25	9.9
PS	69	M	8	1	23.7	3.7	149	1.37	0.76	122.8	1.6	high	9.2	5.3	0.1	21	11.2
PS	47	F	8	1	17.4	4.1		1	0.8	106.2	2.1	high		5.5	0.05	31	9.8
PS	41	M	8	1	27	4.2		1.53	0.72	120.3	1.3	high		4.3	0.17	40	9.2
PS	57	M	7	1	26	4.1	137	1.89	0.79	107.7	1.8	high	7.6	6.2	0.3		9.3
PS	66	M	7	1	22.3	3.8		1.99	1.01	121.8	1.5	high	10.2	5.6			8.1
PS	71	M	7	1	25.2	4	164	1.46	0.86	117	1.6	high	8.9	5	0.1		11.8
PS	56	M	7	1	23.6	4.2	93	1.18	0.89	127.1	1.5	high	8.7	4.9	0.06	27	9.4
PS	62	M	7	1	21.2	3.7	159	1.6	0.71	73.05	2.1	high	9.5	5.7	0		11.4
PS	79	M	6	1	20.8	3.5	251	1.92	0.9	119.6	1.8	high	8.7	3.4	0.1	31	11.3
PS	44	M	6	1	21.6	4.2	134	0.99	1.07	144.1	1.6	high	9.3	5.7	0.04	28	8.9
PS	59	F	6	1	27.6	3.5	243	1.54	0.79	91.98	1.5	high	9.2	5.8	0.1		11.9
PS	62	M	6	1	24.3	4.1	243	1.75	1.06	123.5	1.8	high	7.8	4.7	0.02	26	13
PS	67	M	5	1	18.4	4	119	1.38	1	85.38	2.1	high		4.5	0	31	12.3

PS	51	M	5	1	28.7	3.6	185	1.12	0.82	105.5	2.1	high	8.4	3.1			10.6
PS	64	M	5	1	20.7	3.2	188	1.58	0.9	84.49	1	high	9.1	6.1	0.05	27	10.9
PS	71	F	5	1	26.1	3.8	106	1.32	0.78	106	2.1	high	8.8	4.4	0.11	23	9.1
PS	57	F	5	1	26.7	3.7	220	2.08	1.13	90.4	1.5	high	8.6	5.2	0.17	30	12.3
PS	45	F	5	1	22.2	3.9	122	1.59	0.76	121.4	1.5	high	8.7	4.6	0.05	34	10.5
PS	55	M	5	1	25.6	3.9	153	1.79	1.09	107	1.8	high	9.7	4.5	0	21	11.7
PS	68	F	5	1	25.2	4.1		1.16	0.71	123	1.3	high	8	6.3	0.22	32	9.9
PS	44	M	5	1	29	4.5		1.49	1.08	163.9	1.3	high		4.4	0.69		11.9
PS	53	M	5	1	18.8	3.8	251	1.18	1.11	93.63	1.8	high	6.8	11	1.28	35	15.3
PS	43	M	5	1	23	4.8	131	1.17	1.03	109.5	1.8	high	9.9	10.2	0.1	34	9.8
PS	67	M	5	1	21.1	3.8	172	1.06	1.17	113.8	1.6	high		4.5	0.67	22	10.3
PS	76	F	4	1	20.1	3.9	177	1.75	1.05	130.7	1.5	high	8.9	6.1	0.05	29	9.7
PS	28	M	4	1	31	3.8	131	1.44	0.79	93.89	2.2	high	9.8	5	0.36	21	9.5
PS	45	M	4	1	27.6	4.2	100	1.24	1.15	85.52	1.1	high	8.4	6.1	0.03	27	10.7
PS	59	M	4	1	21.6	4.1	229	1.81	0.84	83.3	1.8	high	8.7	6.5	0.1		11.4
PS	71	M	4	1	20	3.8	192	1.45	1.03	88.36	2.1	high	5.9	4.2	0.19	24	11.4
PS	71	M	4	1	24.1	3.5	140	1.41	1.13	114.7	1.5	high	9	5.7	0.2	29	9.6
PS	81	F	3	1	16.3	4.3	130	1.26	0.81	82.35	1.8	high	9.1	5	0.7	30	9.6
PS	65	M	3	1	22.2	4.1	164	1.2	0.88	92.18	2.2	high	10.3	5.6	0.04	21	10.4
PS	77	M	3	1	24.9	3.1	168	1.64	0.86	70.38	1.8	high	8.8	5	1.06	28	9.5
PS	82	M	3	1	21.9	3.6	110	1.3	0.86	96.88	1.1	high		7.4		31	9.4
PS	42	M	2	1	16.5	4.2	199	1.24	0.91	108.4	1.6	high	8.6	6.3			
PS	65	M	2	1	23.5	4.2	137	1.13	0.91	101.6	2.1	high	9.3	5.4	0.15	26	11.9
PS	35	M	2	1	22.5	4.6	186	1.26	1.05	116.6	1.4	high	9.9	6.1	0.3	29	10.1
PS	59	M	2	1	27.1	3.6	157	1.78	0.66	77.76	1.5	high	9.5	4.4	0.25	34	9.4
PS	53	M	2	1	24.1	3.8	163	1.08	0.81	56.48	2.1	high	8.8	6.7	8.82	31	9.1
PS	38	M	2	1	24	3.7	166	1.6	0.78	81.38	2.1	high	8.7	5	0.1	26	8.9
PS	53	M	2	1	28.7	3.9	122	1.16	0.98	110.6	1.5	high	9.9	5.8	0.34	23	9.3
PS	76	F	3	1	18.2	3.9	170	1.23	0.81	86.56	1.3	high	8.2	5.3	4.7		12.9
PS	35	F	24	1	22.7		220	1.7	0.94	110.1	1.8	high	10.3	7.9	0.05	24	11
PS	78	M	2	1	19	3.7		1.38	0.91	106	1.4	high	8.8	4.4	0.04	29	11.5
PS	44	M	2	1	33.9	3.9	114	1.01	1.09	103.5	1.3	high	8.4	6	2.56		7.8
PS	76	M	3	1	26.8	3.9		1.27	0.97	89.75	1.8	high	8.2	4.2	0.3	26	11.8
PS	55	M	2	1	19.7	3.9	149	1.36	1.08	88.79	2.1	high		5.1	0.1	33	11.2
PS	71	M	9	1	23.4	3.9	189	0.93	0.63	84.35	1.5	high	10.2	6.9	0.16	14	10.2
PS	60	F	32	1	20.2	3.6	154	1.95	1.03	94.94	1.4	high	9.6	6.3	0.03	18	8.8
PS	58	M	27	1	18.5	3.9	218	1.4	1.14	115.9	1.4	high	10.4	6	0.05	37	9.5
PS	49	M	24	1	21	4.2	93	1.73	0.91	112.3	1.8	high	8.8	6.3		29	10.4
PS	65	F	16	1	23.4	3.5	122	1.29	0.69	140.8	2.1	high	9.4	5.4	0.01	28	10.7
PS	67	M	14	1	20.2	4	183	1.34	0.79	140.8	1.1	high		5.3	0	26	12.4
PS	71	F	14	1	28.3	3.6	135	1.29	1.11	121.8	1.6	high		2.5	1.49		10.1
PS	70	M	12	1	24	3.7	143	1.41	1	120.7	1.5	high		4.7	0.2	29	9.5
PS	47	M	11	1	22.5	3.7	121	1.12	0.76	117.8	1.8	high	8.9	9.9	0.33	22	11.7
PS	54	M	11	1	22.1	4.1	157	1.81	1.01	142.6	2.1	high	8.2	4.1	0.37	34	9
PS	68	M	10	1	16	3.6	143	2.23	0.75	108	2.1	high	8.9	6.1		25	11.5
PS	64	M	10	1	19.6	3.6	139	1.4	1.04	82.06	1.8	high		6.4	0.1	26	9.8
PS	67	M	6	1	23	3.6	234	1.3	1.03	119.5	1.5	high		3.1	0	27	10.7
PS	60	F	6	1	23.4	3.6	160	1.98	0.77	95.4	1.5	high	10	3.7	0.02	27	10.6
PS	43	M	5	1	20.7	4.5	191	1.36	1.06	126.3	1.3	high		4	0.6	26	11.1
PS	73	M	5	1	21.6	3.6	163	1.42	0.84	115.9	2.1	high	10.5	6.1	0.55	27	10.6
PS	49	M	5	1	25.8	4.2	194	1.67	0.63	70.7	1.3	high		4.8	0	28	10.1
PS	76	F	5	1	21.9	3.6	109	1.34	0.87	93.89	1.5	high		3.7	0.1	30	12.7
PS	57	M	4	1	20.2	3.7	184	1.39	0.74	121.9	1.3	high		6	0.1	28	10.1
PS	74	M	4	1	21.7	4	141	1.48	0.67	136	1.8	high	9.4	5.7	1.22	18	10.7
PS	60	M	4	1	24.5	4.2	139	1.43	1.09	122.5	1.5	high		7.2	0.05	26	10.2
PS	38	M	4	1	21.7	4.8	134	1.28	1.08	100.9	2.1	high	7.3	6.3	0.05	26	10.1
PS	68	F	4	1	20.4	3		1.45	0.76	79.54	2.5	high	9.9	6.1	0.2	24	8.6
PS	41	M	4	1	25.1	4	101	1.09	0.94	114.2	1.5	high	8.1	2.8	0.1	20	6.9
PS	64	F	3	1	26.9	3.6	141	1.52	0.85	96.33	1	high	9	6	0.02	30	8.8

PS	80	M	3	1	21.1	3.7	175	1.21	0.73		1.6	high	9	5.1	4.2	17	10.7
PS	74	M	3	1	25.2	3.8	133	1.43	0.66	91.74	1.5	high	9.3	3.7	0.1	25	11
PS	53	M	3	1	19.2	4.1	161	1.29	1.11	126.3	1.5	high	9	4.1	1.72	28	10.4
PS	67	M	2	1	19.4	3.9	138	1.04	0.98	108.9	1.5	high		5.8	0.1	23	10.9
PS	68	M	2	1	25.7	3.8	165	1.26	0.92	102.6	1.5	high		3.9	0.39	25	11.6
PS	56	F	14	1	24.2	4.2	160	1.62	0.86	137.6	2.1	high	10.2	6.2	0.1	18	10.8
PS	67	M	11	1	19.4	3.9	129	0.99	0.93	122.8	1.8	high	8.9	2.6	1.01	25	9.5
PS	70	F	33	1	23.8	4.3	98	1.47	0.79	102.5	2.1	high	9.5	4.1	0.1	18	10.9
PS	64	M	20	1	19.7	3.9	110	1.67	1.04	126.1	1.8	high	8.7	4.9	0.1	27	8.4
PS	43	F	17	1	19.3		232	1.11	1.12	128.9	2.2	high	8.6	7.2	0.08	18	10.6
PS	69	F	15	1	19.2	2.7	153	1.47	0.66	65.44	1.3	high		4.5	0.1	27	10.5
PS	56	F	14	1	18.4	4.2	141	1.36	1.04	101.4	1.5	high		4	0	31	10.1
PS	64	F	14	1	18.6	4.1	155	1.14	0.75	124.9	1.5	high	8.8	4.9	0.52	27	14.1
PS	40	F	14	1	27.9	4.1	165	1.78	1.16	114.2	1.5	high	8.8	4.2	0.01		10
PS	68	F	13	1	26.3	3.9		1.17	1.27	126.8	1.3	high	7.7	7.3	0.7	28	12.6
PS	55	M	13	1	21	4.2	183	1.4	1.12	108.3	1.8	high		4.7	0.1	30	11.4
PS	69	F	12	1	17.6	4.2	175	1.14	1.1	143.4	2.2	high	9	4.4	0.53	23	7.6
PS	62	F	12	1	23.4	3.8	191	1.51	0.99	135.3	1.8	high	9.1	3.3			6.7
PS	73	F	11	1	20	4.3	117	1.29	0.94	114	2.2	high		4.6	0.57	20	9.5
PS	53	F	9	1	18	3.8	94	1.59	1.07	121.6	1.8	high		7.4	0.1	31	10.2
PS	60	F	8	1	19.1	3.9	184	1.13	1.02	136.5	1.3	high		5.6	0.3	26	9.2
PS	72	M	7	1	20.8	3.5	157	1.23	1.02	105.8	1.5	high	8.3	6.9	0.1	30	9.8
PS	55	M	7	1	24.8	4.2	205	1.56	0.9	135.1	1.8	high	9.9	9	0.15	29	13.4
PS	72	M	7	1	18.7	4.2	213	1.46	1.1	125.4	1.3	high	8.8	5	0.4	26	10.9
PS	61	F	7	1	17.3	3.6	115	1.54	1.04	100.4	1.8	high	8.9	5.6	0.1	24	10.2
PS	78	F	7	1	19.9	3.9	128	1.21	0.9	113.7	1.3	high		6.8	0.2	31	9.9
PS	81	F	7	1	19.6	3.9		1.92	0.97	125.5	1	high	8	4.4	0.05		
PS	59	F	6	1	16.9	4.2	166	1.56	1.02	126	1.8	high	10.7	6.4	0.04	25	11.1
PS	63	F	6	1	20.3	4.1	171	1.24	0.91	107.1	2.1	high	9.8	4.7	0.04	27	10.6
PS	65	F	6	1	18.9	4.3	112	1.3	0.9	104.8	1.5	high	8	5.5	0.28	40	8.3
PS	77	F	6	1	19.3	3.9	169	1.1	1.16	116	1.5	high	10	6.4	0		10
PS	60	F	6	1	18.5	3.9	126	1.19	0.98	120.2	1.1	high		3.9	0.1	22	11.8
PS	24	F	6	1	13.4	4	133	1.36	1.07	98.69	2.1	high	9.2	5.7	0.07	26	10.4
PS	65	F	5	1	21.6	3.9	160	2	1.02	125.3	1.5	high	8.7	6.6	0.48	32	10.5
PS	44	M	4	1	20.7	4.4	268	1.36	0.87	69.36	2.2	high		6.2	0	26	9.6
PS	61	M	4	1	29.5	3.9	221	1.25	0.99	114.8	1.5	high		6.5	0.18	26	12.4
PS	83	M	4	1	19.9	3.9	135	1.22	1.07	125.6	1.6	high	9.2	4.1	1.28	26	7.8
PS	67	F	3	1	20.4	3.7	169	1.19	1.21	58.97	1.5	high	6.7	6.1	0.74	34	9.1
PS	63	M	3	1	23	3.8	134	1.12	0.84	79.1	1.6	high	8.9	5.2	0.5	21	9.2
PS	64	F	2	1	17.7	6.7	96	1.56	1	104.2	1.5	high	10.4	3.2	0.02	32	10.2
PS	61	M	2	1	20.1	4.2	145	1.67	0.65	100.9	1.5	high	9.4	6.6	0.65	32	10.3
PS	72	M	2	1	21.4	3.9	112	0.79	0.75	42.83	1.3	high	8.2	5.9	0.08		10.5
PS	74	M	2	1	20.1	4	238	1.7	0.95	55.96	1.6	high	8.8	4.5		38	10
PS	61	M	13	1	22.8	3.7	126	1.04	0.85	131	1.8	high	9.1	4.2		28	11.6
PS	61	F	27	1	20.3	3.9	208	1.46	0.88	99.48	1.5	high		5.4	0	28	11.4
PS	79	F	20	1	18.3	4.1	171	1.49	0.85	103.3	2.2	high	8.4	6.5	0.02	19	9.9
PS	57	M	18	1	22.2	6.8	133	1.35	1.16	115.5	2.5	high	10	4.7	0.34	28	10.3
PS	56	F	18	1	18.8	4.5	201	1.34	1.03	102.1	1.3	high	8.7	5.5	0.76	27	10.7
PS	52	M	17	1	16.5	4.1	165	1.01	0.83	108.9	2.2	high	10	7.8	0.98		10.2
PS	65	M	16	1	23.1	3.7	180	1.56	0.95	129.5	1.3	high		4.5	0.1	23	10.4
PS	54	F	16	1	19.1	3.9	150	1.13	1.21	120.4	1.8	high		7.9	0.1	37	11.6
PS	62	M	13	1	21.9	4	215	2.31	0.89	102.3	1.3	high	9.5	2.5	0.17	28	10.3
PS	59	M	13	1	17.7	4.3	187	2.01	0.91	94.39	1.5	high	10.4	6.8	0.1	28	9.8
PS	71	M	13	1	18.7	3.7	198	1.88	0.84	93.65	2	high	10.4	6.6	0.09	31	11.3
PS	66	F	12	1	19.4	3.6	141	1.54	0.92	125.7	1.4	high	10.2	4.8	2.81	22	13.5
PS	46	F	12	1	22.9	3.8	207	2.07	1.26	119.1	1.5	high	8.9	5.9	0.74	24	10.8
PS	45	M	10	1	25.5	3.6	175	1.9	0.74	70.34	1.3	high	9.3	4.9	0.02		12.1
PS	75	M	10	1	20.5	3.6	183	1.42	0.89		2.1	high	10.2	4.2	0.04	28	11
PS	56	F	10	1	18.3	3.8	199	1.63	0.99	109.5	1.4	high	9.7	5.1	0.11	26	10.9

PS	77	F	9	1	19.1	3.2	105	1.66	0.83	62.87	2.5	high	8.7	2.8			10
PS	51	F	9	1	28.3	3.9	161	1.08	0.53	100.7	1.8	high	8.6	4.7	0.34	31	9.2
PS	61	F	9	1	24.3	4.1	147	1.26	0.66	101.2	2.1	high	10	6	0.1	23	9.1
PS	63	M	7	1	21.2	3.6	136	1.29	0.8	88.25	1.6	high	9	6.6	1.69	33	8.7
PS	52	M	7	1	18.3	3.9	166	1.28	1.03	126.2	2.1	high	8.7	7.2	0.4	24	9.1
PS	46	M	6	1	26.5	3.9	179	1.81	0.88	136.5	2.1	high	10.7	4.6	0.54	31	13.5
PS	38	M	5	1	20.8	4.3	202	1.62	1.38	91.46	1.5	high	8.7	5.7	0.69	33	11.4
PS	68	F	5	1	21	4.1	180	0.99	0.85	141.7	1.5	high	8.8	5.4	0.1		10.7
PS	58	M	5	1	21.8	3.7	136	1.28	0.73	89.21	2.1	high	9.9	4	0.04	24	12.8
PS	69	M	5	1	19.3	4		1.6	1.02	93.67	1.3	high	8.5	5.3	0.1	28	9.2
PS	62	M	4	1	31.4	4.1	153	1.81	1.18	81.55	1.5	high	8.2	5.4	0.02	34	11.3
PS	68	F	4	1	28.6	4	142	1.4	1.15	95.63	2.1	high	9.5	5	0.06	35	14.7
PS	66	M	3	1	19.4	3.9	109	1.02	0.73	59.62	2.1	high	8.2	6.2	0.01	28	10.5
PS	55	M	3	1	27.1	4	192	1.34	0.85	83.78	2.1	high	10.6	5.7	0.04	28	11.5
PS	64	M	2	1	18.7	3.4	137	1.46	0.74	60.73	1.8	high	8.6	5.6	0.03	25	10.8
PS	82	M	4	1	24.8	4	195	1.47	0.65	122.1	2.2	high		6.1	0.01	22	9.4
PS	82	M	2	1	23.5	4	107	1.04	0.79	43.36	2.1	high	8.4	3.5	0.67	20	8.6
PS	56	M	2	1	22.5	4.1	159	1.04	0.72	85.94	2.1	high	8.4	6.8		26	10.3
PS	78	F	2	1	18.5	3.4	200	1.66	0.76	95.19	2.2	high		7.1	0.09	28	8.7
PS	61	F	21	1	17.6	3.6		1.23	1.39	121.7	1.6	high	7	2.1	0.05	24	9.5
PS	30	M	11	1	18.6	3.9	160	1.59	0.87	101.9	1.8	high	8.1	3.5	1.58	24	7.6
PS	54	M	9	1	22.4	3.7	229	1	0.8	110.8	1.1	high		5.4	0.27	18	10.6
PS	75	M	9	1	21.9	3.5	100	1.12	0.86	133.3	1.3	high	9.5	4.4		31	
PS	60	M	9	1	24	4	170	1.77	0.71	97.48	1.6	high	8.4	6	0.24	21	9.5
PS	75	F	8	1	19.6	3.8	70	1.3	1.17	92.43	1.8	high	8.3	4.5	17	34	9.7
PS	60	F	8	1	26.7	3.6	139	1.32	0.88	129.3	1.8	high	8	5.6	0.43	19	11.6
PS	78	F	6	1	19.5	4	156	1.46	0.78	137.5	1	high	8.1	3.6		22	9.3
PS	59	F	3	1	20.5	3.8	134	1.33	1.05	113.7	2.1	high	9.4	6.9	0.08	22	10.2
PS	81	F	3	1	17.8	3.9	171	1.33	0.96	63.67	1.8	high	9.3	6.2	0.13	19	10.6
PS	65	M	2	1	18.8	3.6	187	2.26	0.93	99.31	1.5	high	9.4	5.5	1.13	24	10.8
PS	81	M	5	1	19.9	3.6	96	1.34	0.95	121.1	1.5	high	9.2	2.9	4	32	11.2
PS	71	F	25	1	19.7	3.7	123	1.69	0.94	117.6	1.6	high		4.8	0.06	22	10.6
PS	60	M	19	1	18.5	3.8	126	1.36	1.1	93.3	1.8	high	8.1	5		33	11.3
PS	57	M	10	1	19.2	4.3	184	1.75	0.8	111.8	1.5	high	9.8	6.6	0.05	29	10.8
PS	57	M	3	1	24.7	3.9	142	1.02	0.85	182.7	1.5	high	8.9	4.5	0.06	24	10.3
PS	79	F	3	1	18.7	3.3	217	1.61	1.2	107.9	2.2	high		4.9	0.01	28	10.5
PS	78	M	3	1	21.3	4.1	124	1.29	0.8	84.36	1.3	high	8.3	4.1		30	10.4
PS	60	M	3	1	24.5	4.2	131	1.23	0.93	127.8	1.8	high	9.1	3.2		31	10.9
PS	69	M	2	1	18.7	3.8	144	1.53	0.98	130.5	1.5	high	8.8	3.8		15	10
PS	71	F	2	1	25.6	4	156	1.49	0.77	92.12	1.5	high	9.1	4.8	0.11	35	9.6
PS	78	M	2	1	20.6	4	165	1.43	0.98		1.5	high	7.8	4.9	0.1	27	10.4
PS	83	M	2	1	22.6	3.7	116	0.68	0.87	113.4	0.8	high	7.6	2.9		20	8.5
PS	63	M	2	1	25.2	3.4	198	1.51	0.97	88.83	1	high	9.4	6.4	0.3	25	11.3
PS	80	F	7	1	15.9	4	198	1.54	0.78	104.7	1.8	high	8.9	3.1			
PS	63	F	5	1	24.4	4.5		0.98	1.13	110.9	1.3	high	7.5	5.1	0.1	19	10.4
PS	62	M	2	1	22.3	4.6		1.32	0.73	94.41	1.8	high		4.8			6.1
PS	76	F	13	1	18.2	3.6	115	1.42	0.85	118.6	1.5	high	8.2	2.8	0	30	8.1
PS	43	M	29	1	20.9	4.1	115	1.17	0.93	110.5	1.5	high	8.8	3.5	0.1	27	9.1
PS	35	M	19	1	19.6	4	132	2.16	1.07	109.4	1.5	high	8.9	4.2	0.08	31	9.9
PS	51	M	13	1	22.1	3.5	126	1.32	1	103.6	2.1	high	7.9	5.1	1.06	30	9.2
PS	76	F	13	1	22.8	3.8	160	0.97	0.87	114.8	1	high	8.7	3.8		27	10.6
PS	56	M	12	1	16.4	4	114	1.18	1.15	92.35	2.2	high	9.4	6.6	0.1	34	10.7
PS	66	M	11	1	22.1	3.6	202	1.61	1.03	115.3	1.4	high	9	5.7	0.05	27	11.4
PS	59	M	8	1	22.3	4	126	1.69	0.85	65.21	1.5	high	10	4.9	0.3	40	11.7
PS	67	F	8	1	18	4.1	159	1.53	0.98	91.96	2.5	high	10.4	5.3			8.8
PS	56	F	8	1	18.3	4.4	187	1.27	1.06	102.4	2.2	high	9.1	5.2	0.15	31	9.1
PS	48	M	8	1	21.9	3.8	127	1.26	0.78	99.69	1.8	high	9.1	6.4	0.07	34	10.1
PS	66	F	7	1	22	4	152	1.09	1	120.6	2.2	high	8.4	4.1	0.02	24	9.7
PS	61	M	6	1	21.5	4.5	127	1.47	0.63	81.63	2.1	high	9.7	4.2	0.1		11.9

PS	73	F	5	1	18.3	4.2	146	1.58	1.01	93.43	2.5	high	9	4.7	0		10.6
PS	56	M	3	1	23.5	4	132	1.99	0.8	88.82	1.5	high	9.3	5	0.31	38	10.5
PS	69	M	2	1	21.5	4.1	134	1.88	0.8	51.02	1.8	high	7.9	5.8	0.1		9.3
PS	58	M	13	1	24.2	4	158	1.83	1.1	99.8	1.8	high	9.6	3.4	0.76	27	11
PS	50	F	34	1	16.4	3.7	220	1.06	1.04	108.7	2.2	high	9.2	8.3	0.33	25	11.1
PS	72	M	34	1	16.5	3.6		1.32	0.84	78.16	1.8	high	8.9	5		31	9.3
PS	64	M	33	1	19.9	4		1.42	0.91	102	1.8	high	9.1	7.9	1.55	30	12.7
PS	58	F	32	1	21.4	3.3		1.13	1.03	93.8	1.8	high	8.4	6.2		24	9.2
PS	59	F	32	1	16.6	3.1		1.49	1.09	105.3	1.8	high	8.9	2.8		29	10.1
PS	63	F	32	1	22.9	3.5	135	1.85	1.06	70.79	1.4	high	8.5	5.2	0.05	27	9.6
PS	67	F	32	1	21.9	3.5	109	1.15	0.78	89.55	1.6	high	8.3	3.7	0.27	31	8.4
PS	55	M	30	1	19.4	4.2	120	1.1	0.84	90.5	2.1	high	8.8	4.7	0.05	31	9.7
PS	55	F	29	1	20.2	4.1	120	1.34	1.22	109.9	1.5	high	9.7	7.9	0.24	46	11.6
PS	66	M	27	1	16.8	3.7	175	1.69	0.67	101.2	1.6	high	9.5	4.3	0.05	24	12
PS	72	F	27	1	16.4	3.8	131	1.45	0.9	101.6	2.1	high	9.2	4.4	0.04	24	8.6
PS	77	M	26	1	21	3.6	179	1.51	0.67	102.8	1.4	high	9.6	5.7	0.05	24	10.8
PS	64	M	25	1	20.7	3.3	147	2.02	0.76	108.1	1.4	high	9	5.1	0.05	36	10.1
PS	60	M	25	1	18.2	3.8	209	1.45	1.01	120.3	1.6	high	9.9	4.9	0.21	28	10.8
PS	54	F	24	1	19	3.6	208	1.62	0.9	102.7	1.6	high	9.8	4.7	0.2	26	10
PS	75	M	22	1	26.7	3.3	159	1.3	0.92	110.4	2.1	high	8.7	5.6	0.02	25	9.2
PS	70	F	22	1	20.8	3.7	175	1.39	0.51	104.5	1.6	high	9.9	3.8	0.83	33	10.6
PS	66	F	21	1	19.3	3.5	190	1.76	1.06	123.2	1.4	high	9.7	3.8	0.05	26	12
PS	65	F	20	1	17.2	3.6	144	1.78	0.8	115.4	1.6	high	10.9	4.2	0.05	27	9.3
PS	58	M	19	1	20.7	3.8	162	1.57	1.12	131	1.6	high	9.9	6.1	0.05	25	10.9
PS	60	F	19	1	20.5	3.9	176	1.99	1.14	101.7	1.4	high	9.5	6.2	0.05	27	11.1
PS	66	F	19	1	16.7	3.7	179	1.4	0.98	117.1	2.2	high	9.4	6.9		29	
PS	66	M	18	1	19.1	3.4	222	1.45	0.76	107.3	1.5	high	9.7	4.1	0.08	31	12.4
PS	66	M	18	1	18.2	3.5	159	1.66	0.84	127.3	1.5	high	9.5	3.9	0.05	29	11.7
PS	63	M	15	1	19.6	3.5	117	1.29	0.93	129.3	2.1	high	9.7	4.5	0.01	17	8.2
PS	68	M	13	1	21.9	3.7	164	1.29	0.93	125.1	1.8	high	9.4	3.7		24	11
PS	69	M	13	1	18.3	3.8	172	1.08	1.06	117.8	2.1	high	9.1	5.1	0.02	21	10.1
PS	73	F	13	1	24.9	3.4	121	1.65	0.94	129.9	1.1	high	6.8	5.5	0.22	33	10.5
PS	64	M	12	1	23	3.8	137	1.63	0.77	134.1	1.8	high	8.3	7.4	0.04		9.4
PS	79	F	12	1	17.6	3.4	118	1.52	0.87	95.43	2.1	high	9	4.9	0.45	29	11.8
PS	65	M	11	1	20.7	3.7	165	1.38	1	121.4	2.1	high	7.9	5.9	0.48	25	9.6
PS	58	F	10	1	19.5	3.8	166	1.24	1.2	122.8	2.1	high	9.9	6.2	0.19	15	11.1
PS	62	M	9	1	17.9	4.1	191	1.82	0.95	101.2	1.5	high	9.7	5.7	3.48	35	10.4
PS	40	M	9	1	31.2	3.9	138	1.43	0.89	125.1	1.5	high	9.3	6.2	0.02	36	10.5
PS	58	M	9	1	21.7	3.8		1.59	1.04	128.9	2.1	high	8	5.2	0	25	11.3
PS	47	M	9	1	27.8	3.9	121	2.17	1.33	115.3	1.8	high	8.4	4.3	0.11	31	10.4
PS	64	M	8	1	23.2	4	112	1.87	0.86	104.2	1.8	high	8.9	7.7		38	11.8
PS	70	F	8	1	16	3.7	144	1.26	0.97	98.4	2.1	high	9	4.5	0.03	26	10
PS	73	F	7	1	20.2	3.6	126	1.74	0.91	115.7	1.5	high	9.5	7.1	0.02	27	8.7
PS	63	M	7	1	23.3	4.1	157	1.46	0.83	132.7	1.5	high	7.8	3.5	0.57	22	10.4
PS	71	F	7	1	25.7	3.4	96	1.27	0.74	125.4	2.1	high	8.6	4.5	0.16	22	9
PS	63	F	6	1	20.2	3.7	193	1.65	0.97	111.2	1.8	high	9.8	4.9	11.7	32	12
PS	41	F	6	1	17.9	4	189	1.12	0.93	127.3	2.5	high	8.9	6.6	0.18	25	12.2
PS	59	M	6	1	20.6	3.9	136	1.34	0.8	91.1	2.1	high	9.4	4.6	0.11	23	11.8
PS	54	M	6	1	23.1	4	155	1.17	0.83	87.97	1.8	high		6.4	0.6	27	11.2
PS	59	F	5	1	18.9	3.4	161	1.4	0.73	93.02	1.3	high	9.1	3.9	0.2		7.4
PS	28	M	5	1	19.2	4.5	132	1.06	0.79	87.4	2.1	high		5.5			10.8
PS	26	M	5	1	21.6	4.2	166	1.96	1.34	109.1	1.3	high	9.7	4.2	0.64	30	10.6
PS	65	F	5	1	16.2	4.4	102		0.73	94.41	2.1	high		2.8	0.2	19	8.9
PS	58	F	5	1	19.2	3.7	147	1.62	1.09	127.7	2.1	high	8.9	7.7	0.07	29	10.4
PS	71	M	4	1	25.3	3.3	185	1.75	0.8	110.8	2.1	high	9.6	8.7	0.03	28	14.2
PS	58	M	4	1	28.9	4.1	144	1.25	0.88	90.51	1.6	high	8.9	7.6	0.84	26	8.1
PS	46	M	4	1	28.1	4.1	160	1.14	0.83	78.67	1.8	high	7.8	6	0.3	26	10.8
PS	64	M	4	1	22.1	4.4	190	1.09	0.85	122	1.6	high	9.3	7.7	1.28	30	14.1
PS	66	M	3	1	23.5	3.6	162	1.57	1.06	113.8	1.8	high	8.3	6.2	0.58	29	11.9

PS	26	F	3	1	23.6	4	192	1.44	1.12	105.2	1.1	high	8.3	4.9		26	12.1
PS	68	M	3	1	18	3.8	151	1.21	1.08	87.04	1.6	high	8.9	4.1	4.07	21	11.3
PS	36	M	3	1	17.6	4.4	139	1.9	0.93	80.38	1.5	high	8.5	4.1	0.36	31	10.9
PS	81	M	2	1	17.8	3.1	122	1.07	0.65	103	1.5	high	8.5	4	0.19	26	10.6
PS	44	M	2	1	25	4.2	198	1.54	0.8	115	1.4	high	11.5	4.8	0.66		10.1
PS	78	F	2	1	12.9	3.1	169	1.35	0.77	83.77	1.3	high	9	3.6	0.06	22	10.3
PS	66	M	2	1	19.5	3.5	208	1.48	0.68	89.51	2.1	high		7.3	0.04	32	11.1
PS	72	F	2	1	22.9	3.6	134	1.3	1.03	122.7	1.6	high	8.6	4.3	0.17	23	9.8
PS	73	F	2	1	20.3	3.1	170	1.2	1.08	85.83	1.8	high	10.3	5.6	0	26	10.8
PS	36	F	2	1	17.9	4.1	172	1.21	0.96	93.6	1.6	high	7.9	4.9	0.08	26	10.4
PS	57	F	2	1	18	3.9	154	1.55	1.03	91.34	1.8	high	8.2	3.1	0.21	35	8.1
PS	71	F	6	1	23.6	3.6	142	1.13	0.9	87.2	1.8	high	8.5	3.5	0.36	21	9.3
PS	50	M	18	1	20.6	3.8	137	1.36	0.92	74.33	2.1	high	9.2	6.1	0.03	39	12.4
PS	53	F	33	1	18.1	4.2	192	1.56	0.68	75.95	1.9	high		4.1	0.04	23	13
PS	67	M	21	1	21.8	3.8	240	1.71	0.74	107.1	1.6	high		5.6	0.03	20	10.9
PS	61	F	21	1	20.3	4	211	1.81	0.84	119.6	1.4	high	9.1	8	0.05		10.8
PS	48	M	16	1	20	4.6	209	1.37	0.9	112.1	1.8	high	8.3	5.6	1.34		11.7
PS	51	M	16	1	24.7	4.4	148	1.78	0.93	115.7	1.3	high	9	5.6	0.1	26	11
PS	61	M	16	1	21.4	4.1	139	1.26	0.95	107.3	2.2	high		5.1	0.1	25	10.4
PS	55	F	16	1	19.7	4.2	135	1.47	1.14	140	2.2	high		6.7	0.1	35	10.7
PS	57	M	12	1	20.3	4.1	108	1.32	1.08	169.3	1.4	high	8.3	4.2	0.5	23	10.5
PS	53	M	12	1	20	4.2	177	1.23	0.9	108	2.2	high	10	6.2	0.05	24	10.1
PS	50	M	9	1	22.9	4.4	162	1.23	1.16	113.1	1.8	high	9.3	7	1.31		8.7
PS	65	F	9	1	25.8	4.3	126	1.75	1	137.4	1.5	high	8.4	4	0.04	23	10.9
PS	65	M	8	1	19	4.2	125	1.67	0.61	98.79	2.1	high	9.6	5.1	0.2	22	7.9
PS	35	F	8	1	25.2	3.8	165	1.46	1.04	97.08	1.5	high		6.7	0.78	34	8.9
PS	69	M	8	1	22.1	4.3	117	1.85	0.65	104.1	2.1	high	9.5	5.8	0.1	28	9.4
PS	45	M	7	1	22.9	4.3	221	1.62	0.78	121.3	2.2	high	7.3	5.7	0.21	19	8.6
PS	44	M	6	1	33.4	3.9	217	1.36	0.95	111.2	2.1	high		4.1	0.71	21	9.2
PS	63	F	5	1	17.3	4.2		1.38	1.16	95.03	1.3	high	9.9	5	0.5	20	11.1
PS	53	F	4	1	18.8	4.2	135	1.3	1.09	120.3	2.1	high	9.5	5.2	0.05	20	11.6
PS	74	M	4	1	25.8	4	132	1.49	0.87	136.7	1.5	high	11.1	4.1	0.04		11
PS	29	M	3	1	32.3	4.1	157	1.12	0.65	66.65	1.8	high	9	5.9	0.05	24	10.1
PS	38	M	3	1	20.3	4.2		1.29	0.77	113.6	1.6	high		5.5	0.42	34	8.4
PS	50	M	3	1	20.7	4.5		1.28	0.64	85.54	1.6	high		6.3	0.07	35	9.4
PS	39	F	12	1	22.6	4.4	186	2.03	1.04	136.2	1.8	high	8.6	6.5	0.3	23	9.8
PS	52	F	15	1	24.6	3.8	142	1.58	0.74	77.23	1.4	high	8	4.2	0.02	30	9.7
PS	41	M	15	1	20.7	3.9	229	1.29	0.84	100.9	1.3	high	8.4	5.3	0.12	48	13.5
PS	63	F	11	1	18.9	3.4		1.2	0.51	80.74	1.8	high	9.5	3.1	0.36	30	8.5
PS	70	M	6	1	18.3	4.2	117	1.47	0.96	106.6	1.5	high	8.6	6.7	1.84	40	8.9
PS	55	M	2	1	18.6	4.1	169	1.51	0.75	104	2.1	high	9.4	5.5	0.11	35	11.1
PS	68	M	2	1	20.5	3.9	122	1.32	0.67	74.26	1.8	high	9.2	4.5	0	19	11.8
PS	54	F	2	1	19.3	3.7	117	1.27	0.73	104.1	1.8	high	8.1	6	0.04	23	10.3
PS	57	M	24	1	19.5	4	174	1.04	1.08	120.1	1.3	high		6.3	0.2	30	12.5
PS	61	F	23	1	18.4	3.5	140	1.24	1.06	121.6	1.4	high	8.6	3.5	0.19	20	10
PS	54	F	23	1	19	3.4	143	0.77	0.74	122.4	1.4	high	8.2	3	0.1	28	10
PS	59	F	21	1	23	4.1	136	1.22	0.84	93.92	1.8	high	9.6	6.1	0.05	19	11.4
PS	43	M	20	1	19.6	3.8	141	1.44	0.82	102.6	2.2	high	8.8	4.8		26	10
PS	72	M	19	1	22.8	3.5	141	1.23	1.02	145.3	1.3	high		6	0.1	29	9.5
PS	30	M	19	1	19.5	4.1	132	1.22	1.14	131.9	1.8	high	9.7	5.8		29	10.4
PS	66	F	19	1	21.5	3.6	178	1.55	1.03	106.9	1.8	high	10	4.5	0.78	26	10.2
PS	61	F	18	1	17.7	3.5	144	1.3	1.18	107.5	1.1	high		3.6	0	27	9.8
PS	61	F	17	1	24.6	2.8	198	1.21	1.08	130.6	2.2	high	9.8	6.9	0		11.6
PS	67	F	17	1	18.6	3.5	122	1.63	0.86	119.4	1.8	high	9.4	7	0.8	38	10.9
PS	54	F	17	1	22.8	3.9	149	1.31	0.73	138	1.5	high		4.9	0.1	23	9.9
PS	49	M	16	1	19.2	4.4		1.69	0.86	109.4	1.5	high	8.3	5.2	1.5	30	8.6
PS	62	F	16	1	25.1	3.9	133	1.14	1.21	117.8	1.5	high		5.3	0.1	28	11.4
PS	58	F	15	1	17.3	4	118	1.34	1.06	138.3	2.1	high	8.5	4	0.05	21	10.5
PS	34	M	14	1	23.3	4	132	1.46	0.93	126.2	2.1	high	9.3	5.7	0.05	32	11

PS	45	M	14	1	20.1	4.1	187	1.33	0.98	101.7	1.5	high	10.3	7.5	0.02	28	10.1
PS	69	M	14	1	18.4	3.8	142	1.17	0.92	96.81	1.3	high		6	0.1	28	9
PS	34	M	12	1	22.6	4.4	144	0.98	1.03	103.7	1	high	8.3	6.9		33	9.1
PS	70	M	11	1	19.6	3.9		1.63	0.95	129	1.8	high	9.4	5	2.77		10.9
PS	58	M	10	1	22.4	3.9	154	1.76	1.13	137	1	high	9.5	4.3	0.04	30	6.8
PS	63	F	10	1	17	4.4	94	1.17	1.11	117.3	1.5	high		6.4	0.1	36	11.2
PS	46	F	10	1	16.5	4	187	1.01	0.71	70.18	1.6	high	9.1	8.1			10.2
PS	51	F	9	1	18.3	3.7	135	1.69	0.76	114.7	1.3	high		3.6	0.1	27	10.7
PS	56	M	9	1	25.2	3.7	126	1.45	1.03	119.8	1.5	high	9.1	4.8	0.1	33	10.2
PS	59	M	8	1	23.1	2.8	142	1.47	1.3	121.6	1.5	high	9.3	4.4	0	33	10.7
PS	76	M	8	1	24	3.7	125	1.48	0.85	121.3	1.3	high	6.8	3.9	3.84	34	8.5
PS	59	F	7	1	26.2	3.6	155	1.06	0.92	118.1	1.5	high	10.5	5.1	0	27	11.5
PS	64	M	5	1	28.7	3.8	125	1.23	0.79	55.63	1.8	high	9.6	4.8	0.06	26	10.3
PS	81	M	6	1	21.1	3.9	126	1.35	1.05	135.7	1.1	high	8.9	3.9	0.2	19	10.4
PS	74	M	6	1	24	3.8	159	1.46	0.88	122.7	1.5	high	9.2	4.5	0.66		10.4
PS	65	F	5	1	20.2	4		1.65	0.95	123.8	1.3	high	8.1	8.3			12.8
PS	70	F	5	1	25.4	4		0.89	0.7		1.6	high		5.3	0.13	37	9.2
PS	55	M	4	1	26.1	4.1		1.95	0.84	140.6	1.3	high	10	6.1		33	11.4
PS	53	M	4	1	26.9	4	157	1.42	0.75	100.8	1.5	high		5.5	0.05	29	10.6
PS	61	M	3	1	21.5	4.2	202	1.87	1	77	1.5	high	10.2	5.1	0.33	21	11.5
PS	51	M	3	1	28.7	3.7	157	1.56	1	88.34	1.8	high	8.7	5.7	0.2		10.5
PS	34	M	2	1	20.9	4.4	174	1.71	0.99	137.4	1.3	high		5.4	0.1	36	11.3
PS	70	F	2	1	21.3	4.5		1.1	0.76	95.38	1.3	high		4.9	0.21	28	8.1
PS	72	M	13	1	19.9	3.8	229	1.6	0.88	100.5	2.2	high	9.1	6	0.3	26	10.9
PS	70	M	10	1	22.8	3.6	189	1.72	1.17	111.9	1.8	high	9	4.7	0.3	28	10.9
PS	58	M	2	1	19.8	4	192	1.86	0.99	100.4	1.3	high		5.8	0.3	36	10.8
PS	57	M	11	1	22.9	3.4	179	1.69	0.72	107.7	1.8	high	9	5	0.3	35	11.3
PS	42	M	4	1	25	4.6	94	1.47	0.92	116.6	1.8	high	8.5	4.5	0.1	24	8.9
PS	67	M	4	1	25.4	4.6	226	1.57	0.93	123.5	2.5	high	9.1	3.4	0.3	21	10.8
PS	60	M	10	1	24	3.9	140	1.36	0.8	113.9	1.1	high	9.5	6		21	12.1
PS	58	M	27	1	21.1	3.6	251	1.51	1.42	85.66	1.4	high	9.4	6.4	0.08		9.8
PS	48	F	21	1	19.2	2.4		1.09	1.39	61.4	1.8	high	9.2	9.2		29	11.3
PS	57	M	6	1	28	3.7	115	1.63	0.86	128.9	2.1	high	9.9	4.7	0.58	28	11
PS	77	M	2	1	17.9	3.1	151	1.3	0.64	103.4	1.8	high	8.9	3		41	10.4
PS	57	F	9	1	24.5	3.6	131	1.24	1.1	122.4	1.8	high	8.8	4.8		33	
PS	56	M	5	1	18.6	4.2	261	0.87	0.85	65.32	1.3	high	9.5	6.9	0.2	66	10.1
PS	71	M	5	1	19.9	3.6	151	1.23	0.87	108.4	1.8	high	8.7	5.9	0.13	21	10.4
PS	70	F	21	1	18.7	3.9	132	1.62	0.97	113.6	1.4	high	9.3	4.5	0.05	31	10.9
PS	51	F	20	1	15.9	3.6	175	1.22	0.92	133	1.6	high		5.1		28	8.5
PS	70	F	24	1	16.8	3.5	176	1.35	0.93	83.84	1.6	high		4.8	0.23	26	11
PS	45	M	19	1	18.3	4	138	1.27	0.96	105.8	1.8	high	8	7.4	0.05	30	12.1
PS	67	F	19	1	22.3	3.5	132	1.79	1.04	96.85	2.1	high	8.4	5.3	0.06	27	9
PS	69	F	19	1	17.2	3.8		1.63	1.12	81.54	2.2	high	9	5.7	0.17	27	10.3
PS	74	M	17	1	26.9	3.6	182	2.14	0.63	104.2	2.1	high	9.7	6.1	0.1	22	10.5
PS	66	M	17	1	18	3.9	154	0.93	1.23	121.1	1.8	high	8.5	8	0.1	23	11.3
PS	61	F	15	1	20.8	3.6	139	2.23	1.2	121.4	2.1	high	9.2	4.5	0	32	9.5
PS	80	F	12	1	17.8	4.1	216	1.59	0.88	102.1	1.3	high		4.3			9.1
PS	59	F	12	1	15.7	3.8	150	1.01	1	127.4	2.5	high	8.1	6.2	0.78	28	12.3
PS	73	F	12	1	20.3	3.1	133	1.85	0.92	132.9	1.3	high	8.2	2.4	0.04	30	7.2
PS	76	F	12	1	28.6	3.7	137	1.5	1.11	133.8	1.8	high	7.9	5.1	0	17	10.8
PS	43	M	11	1	23.6	4.2	89	1.15	0.96	112.1	2.2	high	9.2	4.9	0.2	20	9.3
PS	69	F	11	1	16.8	4	135	1.39	1.07	110.4	1.8	high	8.5	6.8	0.1	22	10.4
PS	61	F	11	1	16.5	4	215	1.84	0.94	31.75	2.1	high	9.5	3.8	0.1	27	10.3
PS	47	M	11	1	23.7	4.1	167	1.43	1.03	99.87	1.8	high	8.1	2.7	0.08	30	10
PS	77	F	8	1	19	4.5	143	1.76	0.92	105.2	1.3	high	8.9	4.2	2.1	37	10.4
PS	78	M	8	1	23.4	3.6		1.2	0.66	84.53	1.6	high	8.1	7.2		36	10.7
PS	48	F	7	1	21.3	3.9	214	1.16	0.78	113.4	1.1	high		4.2	0.4	22	11.4
PS	78	F	6	1	25.5	3.6	177	1.88	1.06	108.1	1.8	high	9.3	2.9	0		9.4
PS	64	M	6	1	21.9	4	122	1.27	1	121.4	1.8	high	9.4	4.8	0.3	30	10.8

PS	46	F	6	1	23.8	4.1	137	1.96	0.79	126.4	1.6	high	7.9	3	0.16	29	10.9
PS	64	F	6	1	22.7	4.2	123	1.51	0.8	107.2	1.8	high	9.1	7.9	1.1		11.7
PS	41	F	5	1	20.9	3.8	204	1.06	1.1	124.2	1.5	high	10.6	4.6	0.1	24	12.5
PS	76	M	4	1	20	3.6	96	1.47	0.96	80.61	1.5	high	9.6	3.9	0.78	47	9.6
PS	79	F	3	1	22.1	4.1	213	1.48	0.93	133.5	1.3	high		4.4	0	24	10.9
PS	54	F	3	1	20.2	3.8	211	1.15	1.2	131.8	1.3	high	9.3	2.8	0.23	34	11.7
PS	68	M	3	1	27.5	3.6	194	1.34	0.96	92.65	1.6	high	7.8	6.5	0.44	28	8.2
PS	82	M	3	1	23.8	3.7	142	1.13	0.81	138.3	1.8	high		10.4	0.47	33	11.4
PS	70	F	2	1	19.3	3.9	156	1.38	1.02	80.24	1.8	high	9.1	5.4		27	10
PS	59	M	2	1	22.7	3.8	129	0.97	1.23	112.7	2.1	high		5.6	18	23	9.7
PS	66	M	2	1	21.2	4.4	207	1.49	1.01	100.6	1.8	high	9.4	2.9	1	15	12
PS	72	F	16	1	24.1	3.2		1.07	0.79	134.2	1.6	high		3.7	0.07	29	9.5
PS	76	F	2	1	14.3	3.5	146	1.48	0.85	119	1.6	high	8.8	7	0.22	31	11.5
PS	75	M	3	1	22.8	3.4	89	1.42	0.6	55.84	1.8	high	9.1	3.9	0.54	30	9.9
PS	67	F	13	1	22.6	4.2	154	1.31	1.02	107.5	1.8	high	9.8	4.6	0.01	23	11.9
PS	60	M	11	1	21.7	3.7	128	2.1	1.02	101	1.8	high	9.7	4.8	0.24	32	8.7
PS	47	F	10	1	16.7	4.1	169	1.37	1.18	96.21	1.5	high	8.2	6.8	0.97	30	11.1
PS	54	F	9	1	19.6	4.3	105	1.5	0.31	118.4	1.8	high	9.8	3.5		30	9.7
PS	68	F	9	1	17.2	4	114	1.25	0.98	89.94	2.1	high	9.4	6.5	0.5	25	10.5
PS	53	M	4	1	30.9	4.3	179	1.42	0.93	103.8	1.8	high	9.6	5.9	0.3	29	10.2
PS	55	M	3	1	25.2	3.6	120	1.15	0.78	121.4	2.1	high	10.4	7.1	0.1	25	11.5
PS	78	F	31	1	21.6	3.3	95	1.55	1.08	126.6	1.3	high		4.5	0.5	49	9.6
PS	75	F	28	1	24.2	3.9	136	1.48	0.83	127.9	1.5	high	8.3	4.5	0.37	29	9.7
PS	70	M	23	1	19.5	3.7	159	1.21	0.96	86.74	2.2	high	9	6	0.3	32	11.4
PS	52	F	21	1	24.2	4.1	131	1.36	0.79	112.6	1.8	high	7.9	4.6	0.09	29	7.6
PS	61	M	21	1	20.7	3.2	188	1.56	0.65	87.41	1.8	high	9.1	4.4	0.3	24	9.8
PS	56	F	19	1	17.6	3.5	106	1.4	0.77	77.56	1.8	high	8.6	6.7	0.58	24	9.9
PS	54	M	19	1	17.8	3.7	121	1.44	0.77	116.7	2.5	high	8.9	7.6	1.06	31	10.5
PS	38	M	15	1	23	4	149	1.51	0.8	131.5	1.8	high	10	7.3	0.3	27	9.7
PS	67	M	12	1	16.3	3.1	120	1.42	0.7	53.25	1.8	high	9.5	7.8	1.03	31	11.8
PS	59	F	13	1	18.8	3.6	136	1.3	0.83	97.55	1.6	high	9.1	4.3	0.1		10.1
PS	68	M	13	1	17.7	3.4	124	1.34	0.93	111	1.5	high	9.2	5.4	0.02	25	11.9
PS	60	M	12	1	23.8	3.7	221	1.43	0.82	125.3	1.5	high	8.8	3.9		25	10.6
PS	58	M	11	1	24	3.5	183	1.59	0.77	108.4	1.1	high	12.6	4.5	0	26	10.5
PS	70	M	12	1	21.4	3.3	137	1.42	0.64	113.6	1.3	high	9	6.6	0.05	26	11.3
PS	53	M	10	1	21.5	4.3	172	1.27	0.69	96.97	1.5	high		5.1	0.3	36	9.4
PS	70	F	8	1	19.5	4	115	1.25	1.01	94.93	1.8	high		5.2	0.4	26	9.6
PS	73	F	8	1	26.4	3.8	106	1.22	0.84	176.9	1.1	high		2.4	0.3	24	11.4
PS	77	M	8	1	22.7	3.9	213	1.2	0.7	131.5	1.6	high	9.1	4.3	0.14		12.2
PS	54	M	8	1	22.9	4	127	1.46	0.9	134.8	1.8	high		3.4			10
PS	75	M	7	1	18.1	3.9	187	1.48	0.82	105.2	1.5	high	9.6	2.4	0.55	34	10.4
PS	81	M	6	1	18.7	3.2	110	1.37	0.71	128.9	1.8	high		4.5	0	28	10.7
PS	68	M	6	1	22.6	3.8	179	1.11	1.34	145.4	1.8	high	7.3	5.4	0.12	24	10.3
PS	40	M	5	1	28.7	3.6	180	1.77	0.78	104.3	1.8	high	9.2	5	0.3	21	10.5
PS	66	F	4	1	19.7	4.1	127	0.87	0.98	152.7	1.8	high	8.3	8.1	0.18	19	11.3
PS	74	F	4	1	22.2	2.9	140	1.24	0.7	79.8	2.5	high	9.3	4.8	0.03	27	10.7
PS	52	M	3	1	25.3	3.6	179	1.41	0.76	92.3	1.8	high		7.7	0.05	26	9.4
PS	61	F	3	1	24.3	3.6		1.67	0.73	104.7	1.5	high	8.1	4.3	0.2	29	12.2
PS	61	M	3	1	19	3.7	121	1.29	0.79	89.45	1.3	high		6.1	0.1	23	10.5
PS	63	F	2	1	16.3	4.1	149	1.24	1.11	96.26	1.8	high	9.7	4.7	0.02	30	10.7
PS	67	M	2	1	19.7	3.9	141	1.28	0.68	104.1	1.6	high		5.4	0.05	30	10.1
PS	56	M	2	1	21	3.9	150	1.35	0.79	104.7	1.6	high		4.8	0.06	31	9.4
PS	56	M	2	1	22.7	3.4	212	1.32	1.24	123.5	1.5	high	9.1	7	0.05	25	11.5
PS	60	F	5	1	18.1	4.4	108	1.44	0.73	84.98	1.8	high	10	4.4	1.26	30	9.3
PS	52	M	3	1	23.4	3.9	119	1.28	1	72.9	1.8	high	9.9	5.7	0.05	21	10.4
PS	73	F	16	1	20.6	3.8	132	1.47	0.8	107	1.8	high	8.5	5.4	0.3	25	11.8
PS	60	M	5	1	16.9	3.7	194	2.05	0.56	65.57	1.8	high	8	6	0.3	30	10
PS	41	F	2	1	29.7	3.9	131	2.25	0.78	106.1	1.8	high	9	4.8	0.3	25	9.8
PS	68	F	2	1	24.6	3.6	161	1.58	0.86	137.5	1.8	high	7.8	4.9	0.3	15	10.1

PS	66	M	9	1	18.7	3.5	167	1.04	0.92	106.4	2.1	high	6.9	7.3	0.08	28	13.2
PS	76	M	3	1	27	3.6	122	1.3	0.62	78.68	1.8	high	8.2	7.7	0.3	22	11
PS	65	F	5	1	24.1	3.5	135	1.31	0.74	116.1	1.8	high	7.9	5.3	0.71	32	11.2
PS	64	M	11	1	17.4	3.8	111	1.3	0.83	96.37	1.6	high	8.8	5.1	0.14	26	8.1
PS	79	M	9	1	21.4	3.6	158	1.48	1.01	107.7	1.8	high	8	4.5	0.3	26	11.1
PS	62	M	7	1	20.5	3.8		0.72	0.97	103.2	1.8	high	8.2	4.5	0.2		10.8
PS	61	M	4	1	20.2	4	173	0.89	0.4	19.03	1.3	high	7.9	3	0.4		10.1
PS	62	M	4	1	22.3	3.5	149	1.67	0.67	89.6	2.1	high	7.3	4	0.3		9.3
PS	73	M	3	1	15.2	3.9	237	1.25	1.01	94.7	1.3	high	8.1	1.9	0.64	36	9.7
PS	80	F	3	1	14.8	3.9	174	1.27	0.91	142.4	1.8	high	9.1	5	0.12	31	10.4
PS	70	M	3	1	24.5	3.7	165	1.08	0.86	89.05	1.5	high	8.2	2.6	0.07	29	10.1
PS	86	M	3	1	25.1	3.3	172	1.37	0.83	64.13	1.5	high	8.3	4.6	0.06	26	10.8
PS	56	F	6	1	20.8	3.7	175	1.48	0.83	124.8	1.5	high		6	0.06	21	9.6
PS	65	M	2	1	22.8	3.5	123	1.59	1.07	94.41	1.5	high	8.8	2.8	0.03	22	9.6
PS	58	F	14	1	25.1	3.9		1.82	0.92	95.74	1.3	high	8.8	3.6	0.48	23	11.8
PS	74	F	9	1	20.1	3.5	151	1.94	0.85	134.2	1.8	high	9.9	4.4		26	
PS	65	M	7	1	22.2	3.7	153	1.33	0.69	109.4	1.8	high	8.6	5.6	0.6		13.4
PS	39	F	16	1	18.3	4.2	160	1.27	0.95	108	2.1	high	9.6	7.3	0.49	29	14.6
PS	77	M	11	1	20.7	3.8	115	1.44	0.89	131.7	1.8	high	8.9	7.1	0.67	26	11
PS	67	F	11	1	19.2	3.4	195	1.15	1.03	137.9	1.8	high		4.3	0.2	27	11.5
PS	55	M	10	1	25.1	3.7		1.7	1	131.9	1.5	high		4.5			5
PS	63	F	10	1	15.6	4.3	194	1.89	0.78	152.5	1.8	high	8.9	6.3		42	10.2
PS	60	F	9	1	17.3	4.1	138	1.42	0.83	114.6	1.8	high	11.2	6.4	0.19	50	9.7
PS	58	F	8	1	22.8	3.8	137	1.44	0.69	116.7	2.1	high	8.1	6.7	0.56	17	12.2
PS	69	M	7	1	25.3	3.7	155	1.81	0.98	128.8	1.5	high	10.3	4.7		30	11.7
PS	60	M	3	1	25.6	3.7	161	2.02	1.25	152.8	1.3	high	10	7.1	0.04	27	9.5
PS	41	F	3	1	17.2	4.6	111	1.46	0.75	93.1	2.5	high	8.8	4.4		25	11.7
PS	75	M	2	1	25.2	3.4		0.99	0.82	100.5	1.5	high		6.3			
PS	70	F	3	1	20.2	2.9	138	1.67	0.89	41.48	1.6	high	10.1	5.9	0.02	30	10.9
PS	64	M	3	1	20.3	2	201	1.9	0.52	56.21	1.5	high	9.1	6.9	0.05	25	10
PS	72	M	3	1	23.8	3.6	167	1.53	0.75	144.1	1.3	high	9	5.1	0.1		14.6
PS	53	M	10	1	23.4	4	171	1.86	0.84	121.3	1.8	high	9.6	5.1	0.5	29	10.4
PS	67	F	3	1	22.5	3.3	140	1.51	0.62	108.6	1.5	high	11.6	3.8	0.3	28	11.8
PS	56	F	4	1	21.8	3.7	264	1.98	1.08	67.95	1.8	high	8.8	5.2	0.3	26	10.1
PS	60	M	13	1	23.1	4	192	1.29	0.78	126.1	1.8	high		7.3	0.2	26	10.4
PS	67	M	6	1	22.9		84	0.96	0.86	106.8	1	high		3.2	0.05	49	12.3
PS	64	M	12	1	20.1	3.2	157	2.07	1.23	120.7	1.5	high	9	3.3	0.05	30	11.1
PS	56	M	4	1	21.3	3.3	181	1.57	1.01	68.97	2.1	high	8.8	5.5	0.2		12.6
PS	63	M	3	1	18.7	3.4	161	1.34	0.84	65.43	2.5	high	9.3	7.5	0.04	28	11.3
PS	76	M	7	1	20.4	3.6	162	1.07	0.88	105	1.8	high		6	0.16	40	9.3
PS	74	M	3	1	18.7	2.9	100	1.01	0.74	77.45	0.8	high	7.6	4.8	7.33	47	10
PS	54	F	10	1	16	4.1	152	1.53	1.3	108.3	1.4	high	9.2	3.9	4.15	28	10
PS	70	F	2	1	18.5	3.3	189	1.14	0.88	73.18	1.5	high	8.5	4.9	0.31	27	10.1
PS	63	F	4	1	17.8	3.7	203	1.3	0.93	77.12	1.4	high	10.1	4.3	0.28		10.4
PS	58	M	35	1	23.5	3.6	132	1.52	0.74	96.88	2.2	high	9.6	5.5	0	22	10.3
PS	51	M	9	1	31.8	4.3		1.75	0.99	134.2	1.3	high	10.2	7.2	0	28	11
PS	55	M	24	1	22.7	3.6	174	1.65	0.84	78.61	1.3	high		5.5	0	22	10.5
PS	61	M	15	1	24.8	3.6	116	1.8	0.82	115.3	1.5	high	11.1	6	0.15	30	7.5
PS	50	M	11	1	20.9	4.1	135	1.4	0.84	77.8	1.3	high	8.1	2.4	1.26	29	10.2
PS	68	M	10	1	22.8	3.8	130	1.41	1.13	116	1.5	high	9.1	4.3	0.06	27	10.7
PS	54	F	10	1	18.8	4	110	1.16	1.25	116.9	1.8	high	8.4	5	0.09	32	11
PS	42	M	6	1	19.3	4.3	140	1.47	1.02	97.8	1.5	high	9.7	7.1		28	9.3
PS	60	F	4	1	20.7	3.2	142	1.12	0.79	116.3	1.5	high	9.2	3.6	0.05	35	12.4
PS	45	M	2	1	25.8	4.1	89	1.15	0.81	76.68	1.6	high	10.1	5.1		26	11.7
PS	70	M	17	1	16.8	4.3	119	1.08	0.83	103.6	1.8	high	8.8	4.9	0.03	21	10.3
PS	65	M	11	1	20.4	3.4	151	1.26	0.93	101.4	2.1	high	7.4	4.2		23	7.5
PS	68	F	6	1	19.4	3.8	172	1.62	0.8	127.8	1.6	high	9.9	5	0.05	28	9.9
PS	66	M	5	1	29.5	3.6	129	1.26	0.74	59.96	1.5	high	8.9	6.3	0		9.4
PS	55	F	5	1	24	4	193	1.35	0.82	102	1.5	high	8.2	5.5	0.1	30	10.7

PS	48	F	3	1	29.8	3.7	118	1.23	0.69	86	2.1	high	8.7	8.7	0.1		15.5
PS	75	M	3	1	22	3.9	159	1.54	0.69	96.34	1.5	high		4.7	0.05	17	11.3
PS	52	F	3	1	26	4.1	169	1.05	0.74	65.55	1.1	high	8.4	5.9	0.14	27	10
PS	65	F	3	1	33.9	3.8	126	1.5	0.82	109.9	1.3	high	8.3	5.1	0.44		8.3
PS	75	M	2	1	20.2	3.9	116	1.52	0.68	61.52	1.3	high	8.5	4.5	0.37	37	9.3
PS	67	F	2	1	20.4	3.7		1.28	0.53	93.29	1.6	high		3.6	0.02	26	9
PS	63	F	2	1	19.7	4.3	150	1.17	0.74	65.37	1.5	high	10.2	6.3	0.05	24	11.1
PS	70	M	7	1	21.1	3.7		1.23	1.15	112.8	1.5	high		4.5	6.2	27	9
PS	61	M	3	1	23.9	4.4	139	1.19	0.66	110.1	1.8	high	8.8	3.9			9.2
PS	62	M	14	1	20.1	4.1		1.61	1.07	104.8	1.6	high	8.9	3.1	1.95	43	10.4
PS	78	M	4	1	19.5	3.8	116	1.61	1	96.42	1.4	high	8.3	5.1		23	9.8
PS	65	M	16	1	24.1	4	188	2.91	1.23	117.7	1.8	high	7.8	4.3	0.09	24	11.2
PS	64	M	12	1	22.1	4.2	166	1.58	0.9	113.2	1.5	high	10.2	5.4		43	10.3
PS	31	M	9	1	23	3.8	116	1.3	1.01	110.8	1.8	high	9.9	5.6	0.05	31	10.1
PS	57	F	5	1	17.9	3.7	133	1.28	1.34	97.01	1	high	8.7	4.6	0.1	23	
PS	69	M	4	1	22.9	3.9		1.15	0.82	59.51	2.1	high		11.5	0.12	25	8.2
PS	81	M	5	1	24.3	3.9	118	1.29	0.78	97.82	2.1	high	8.7	5.7	0.26	27	8
PS	51	M	3	1	19.9	4	131	1.21	0.74	25.86	2.2	high	10	4.6	0.4	30	10
PS	43	F	6	1	21.8	3.6	199	2.27	1.21	134.2	1.5	high	9.4	2.6	0.09	21	9.6
PS	78	M	9	1	23.3	3.9	136	1.5	1.14	108.5	1.5	high	9.5	3.5	0.1		8.8
PS	61	M	3	1	23.2	3.1	175	2.07	0.91	98.64	1.8	high	9.2	3.6	0.02	29	12.1
PS	60	M	13	1	21.4	3.8	135	1.3	1.02	131.6	1.8	high	10.7	5	0.19	25	11
PS	76	M	13	1	20.6	4.3	154	1.1	0.95	128.7	2.2	high	9	3.3		28	10.3
PS	28	M	12	1	19.8	4.2	273	1.64	1.22	95.96	2.2	high		5		24	9.8
PS	78	M	7	1	19.4	3.9	154	2.27	1.22	113.7	2.2	high	9.4	3.4	0.1	20	10
PS	53	M	2	1	22.6	4	133	1.62	0.84	44.22	2.1	high	9.3	5	0.09	32	11
PS	66	F	4	1	24.1	4	137	1.21	1.03	110	2.2	high	7.8	7.3	0.47	32	11
PS	58	M	4	1	22.6	4	126	1.72	0.86	134.9	2.5	high	8.5	5.3	0.06	26	10.8
PS	74	M	3	1	22.2	4.3	182	2.32	1	116.8	1.8	high	9.5	5.9	0.15	23	11.4
PS	74	M	2	1	25.4	4	124	1.52	0.78	131.9	2.2	high	8.8	3.9	1.7	23	9.8
PS	44	M	6	1	22.9	4	180	1.85	0.81	106.1	1.8	high	9.1	5.8	0.1	29	10.1
PS	73	M	8	1	22.7	3.8	175	1.65	1.12	116.8	1.5	high	10.4	3.5	0.32	28	11.3
PS	52	M	4	1	17.7	4.5	159	1.24	0.91	81.19	1.4	high	8.3	4.8	0.2		10.8
PS	78	M	3	1	22	3.1	144	1.29	0.7	106.2	2.2	high	10.6	6.9	0.05	26	9.9
PS	75	F	4	1	17.7	3.6	200	1.6	1.17	113.5	1.3	high	10	4.4	0.01	27	9.8
PS	59	M	4	1	22.6	3.8	174	1.21	0.9	13.54	1.5	high	10.3	6.7	0.13	26	12.2
PS	71	M	5	1	20.8	3.3	184	1.07	0.8	68.9	2.1	high	8.3	6	0.5	28	10.5
PS	77	M	6	1	19.1	3.1	199	1.38	0.82	114	2.2	high	9.1	7.7	0.2	25	10.1
PS	68	M	8	1	22.1	3.2		1.38	0.75	90.83	1.6	high					
PS	79	M	9	1	20.3	3.2	168	1.14	1.02	127.8	1.8	high	10.1	10.8	0.21	21	11.6
PS	64	M	9	1	19.7	3	122	1.27	0.64	71.47	1.5	high	5.2	8.5	0.05	25	11.4
PS	57	M	11	1	27.2	3.8	109	1.41	1.19	127.1	1.5	high	8.8	5.6	0.04	33	10.9
PS	48	M	20	1	26.6	3.7	198	1.41	1.12	112.2	1.6	high		5.2	0.06	30	11.1
PS	62	F	23	1	16.4	3.2	194	1.26	0.93	81.04	1.9	high		3.4	0.03	28	13.7
PS	79	M	3	1	20.5	3.2	109	1.36	0.83	83.12	1.8	high	8	5.7		33	8.9
PS	57	F	11	1	27.2	3.6	176	1.38	1.04	140.2	2.1	high	10.3	6.4	0.01	24	10
PS	61	M	9	1	19.8	3.6	134	1.58	0.99	112.2	2.5	high	10.6	5.6	0.1	25	11.1
PS	66	M	24	1	18.4	3.5	215	1.36	1.01	103.6	2.2	high	9.5	4.4	0.16	21	9.5
PS	72	F	5	1	27.6	3.9	213	1.81	0.79	109	1.5	high	8.4	3.4		30	9.8
PS	64	F	4	1	15.9	3.6	132	1.31	1.07	77.8	2.1	high	9.4	8.1	0.2	24	10.5
PS	62	M	5	1	28.9	4.1	180	1.38	1.09	69.45	1.5	high		4.8	0.1	22	10.6
PS	60	M	12	1	23	3.7	175	1.21	1.17	120.4	2.2	high	9.4	9.4	0.07	24	9.6
PS	54	M	26	1	24.8	3.9	140	1.45	1.13	115.6	1.8	high	7.8	3.9	0.15	24	9.6
PS	61	F	7	1	19.5	3.6	203	2.12	1.1	104.3	2.1	high	10.4	4.6	0	29	10.8
PS	50	M	3	1	25.7	4	221	1.34	0.73	121.2	2.1	high	7.9	9.8	0.97	24	9
PS	80	M	7	1	19.9	3.9	134	1.35	1.14	145.7	2.2	high		6.3	1.66	35	8.7
PS	70	F	31	1	16.3	3.9	139	1.37	0.89	108.8	2.5	high		5	0	29	12.1
PS	65	M	20	1	15.8	3.8	133	1.35	0.59	100.6	1.3	high	9.7	5.9	0.29	35	10.7
PS	77	M	14	1	23.5	3.8	139	1.93	0.57	120.9	1.3	high	9	6	0.02	32	10.1

PS	80	F	8	1	24.5		167	1.17	1.15	122.5	2.1	high	9.7	5.8	0.05	24	10.1
PS	83	M	7	1	23	3.5	119	1.89	0.72	107.2	1.1	high	9	0.7		20	10
PS	68	M	5	1	22.6	3.6	104	1.34	0.8		1.5	high	10.5	6	0.01	20	9.9
PS	62	M	4	1	28.8			1.47	0.82	116.6	1.8	high	9.3	3.5	0.1	25	10.1
PS	81	M	2	1	21.3	3.4	100	1.28	0.78	120.8	1.8	high	8.5	3.5	0.08	45	9.7
PS	77	F	2	1	19.5	3.9	140	1.33	1.13	118.3	2.1	high	9.1	5.3	0.01	23	10.1
PS	69	M	4	1	17.5	3.7	184	1.24	0.81	59.23	1.3	high	8.6	6.7	0.12	29	12.2
PS	76	M	4	1	23.3	3.2	98	0.86	0.6	77.56	2.1	high		6.4		29	8.4
PS	35	M	5	1	42.3	3.9	161	1.2	0.88	88.76	1.5	high	8.9	6.8	0.33	29	9.1
PS	73	M	5	1	25.4	3	225	1.18	0.87	103.8	1.6	high		2.8	0.01	12	9
PS	75	F	9	1	17.2	3.3	179	1.63	0.65	111.3	1.5	high	9.3	5.2	0.17	22	10
PS	57	F	10	1	19.5	4	174	1.26	0.91	110.1	1.1	high		5.8	0.05	29	9.8
PS	60	M	24	1	18.4	3.1	141	1.19	0.84	86.35	2.2	high	9	7	0.2		12.1
PS	68	M	14	1	29.7	4	168	1.57	0.9	102.4	1.8	high	9.1	4.7	0.21	25	12.2
PS	71	F	11	1	18.5	2.5	117	1.21	0.89	112.6	1.4	high	9.4	3.7	3.3	20	10.1
PS	72	F	10	1	17.2	3.5	152	1.7	0.71	102.2	1.3	high	10.2	4.9	0.14	27	9
PS	56	F	14	1	17.6	4.1	113	1.27	0.93	129.1	2.1	high	8.5	5.2			9.1
PS	61	M	19	1	19.5	3.7	154	1.07	0.94	128.2	1.8	high		4.6	0.05	33	11.3
PS	58	M	21	1	18.1	3.7	124	1.43	0.91	117.7	1.8	high		5	0.17	23	9.5
PS	59	F	12	1	19.9	3.6	116	1.24	0.93	145.3	2.1	high	8.8	5.4	0.02	21	10.8
PS	76	F	12	1	24.6	3.6	144	1.72	0.91	145.5	1.3	high	8.8	5.6		28	8.8
PS	75	F	3	1	23.7	3.7	115	1.26	0.84	101.4	1.3	high	8.4	6.5	0.01	34	9.4
PS	57	F	11	1	20.3	3.6	177	1.58	0.82	116.8	2.1	high	9.8	3.3	0.03	25	10.5
PS	64	M	3	1	20.1	3.4	159	1.12	0.92	92.33	1.5	high	8.9	4.5	0.86	35	
PS	56	F	14	1	17.4	3.6	183	1.34	0.75	79.88	2.1	high	9.5	7.3		29	10.6
PS	75	M	18	1	22	3.4	151	1.37	1.14	127	1.3	high	8.3	5.9	0.24	23	11.4
PS	59	M	8	1	25.9	4	233	1.32	1.12	116.3	1.8	high	8.7	4.7	0.1	22	9.3
PS	49	M	2	1	25.6	3.8	119	1.29	1.05	128.2	2.1	high	9	6.1	0.09	26	10.1
PS	43	M	8	1	19.4	4	195	1.24	0.86	83.46	1.6	high	9	5.4	0.15	30	11.4
PS	49	M	26	1	21.8	3.7	121	1.2	0.93	95.94	1.5	high	8.7	9.3	0.42	28	11.4
PS	56	M	6	1	27.1	4.3	124	1.41	0.93	143.6	1.1	high	8.7	3.5	0.05	23	9.6
PS	67	F	4	1	16.1	3.6	216	1.03	0.94	112.5	2.1	high	9	4.3			10.5
PS	66	F	11	1	19.9	3.4	143	1.27	0.94	108.1	1.8	high	8.8	4.2	0.05	25	10.4
PS	56	F	14	1	20.8	3.6	130	1.43	0.93	122.7	1.6	high	9	4.8		29	12.1
PS	56	F	26	1	17.2	3.8	141	1.39	0.6	117.2	1.8	high	9.8	5.6	0.14	22	11
PS	86	M	2	1	19.9	3.5		1.9	0.95	97.05	1.6	high	8.8	3.6	0.61	26	9.3
PS	78	M	7	1	17.7	3.8		1.53	0.95	91.53	2.1	high	8.8	4.8	0.05	30	10.5
PS	58	F	22	1	18.4	3.6	122	1.53	1.14	123	2.1	high	11	3.1	0.04	24	10.5
PS	66	M	23	1	16.2	3.3		1.02	0.82	97.48	1.8	high	9.4	3.5	0.08	19	9.4
PS	63	F	4	1	20.1	3.6	180	1.3	0.94	85.85	2.1	high	10.2	7.8	0.22	26	11
PS	77	M	8	1	24.8	3.5	118	1.14	0.87	116.5	1.5	high	10.2	4.4	3.02	22	
PS	51	F	2	1	25.4	3.8	164	1.3	0.86	35.65	1.8	high		8.1	0.1	22	9
PS	70	M	10	1	17.5	3.8	177	1.42	0.69	81.48	1.5	high	10.5	5.4			10.8
PS	80	M	6	1	20.1	3.7	178	1.77	0.94	106.4	1.5	high	7.5	3.3	12.55	28	9.4
PS	69	M	4	1	24.7	3.7	120	1.65	0.84	129.1	2.1	high	8.9	4.8	0.05	18	11
PS	57	F	20	1	18.1	4	194	1.2	1.01	120.6	1.3	high	8.6	6.4	3.09	27	10
PS	74	M	3	1	24.8	3.2	157	1.21	0.63	66.93	2.1	high		3.1	0.07	32	7.9
PS	53	M	2	1	24	3.7	174	1.45	0.86	90.11	1.8	high		6.2	0.2	33	11
PS	81	F	5	1	22.1	3.6	89	1	1.02	128.3	1.8	high		3.6	0.05	16	6.6
PS	61	M	19	1	26.9	3.6	123	1.75	0.83	115.9	1.3	high	9.4	5.2	1.3	22	10.1
PS	49	F	23	1	18.9	4	135	1.83	1.49	112.6	2.2	high	10.2	4.5	0.16	23	13.2
PS	51	M	25	1	20.3	3.5	134	1.43	1.06	114.6	1.3	high		3.6	0.5	33	10.3
PS	65	M	34	1	18.1	3.4	156	1.22	0.96	91.87	2.2	high		7.4	0.7	29	10
PS	55	M	21	1	21.3	4	175	1.43	0.89	96.73	1.3	high		6.1	0	29	10.9
PS	45	M	27	1	21.6	4	138	1.48	0.71	104.9	2.2	high	8.2	6.1	0.05	29	10.5
PS	55	F	25	1	16.6	3.9	160	1.51	0.8	121.7	1.4	high		6.5	0.32	21	10.1
PS	59	F	34	1	16.5	4	185	1.42	0.81	122.7	1.3	high		5.8	0	30	10.1
PS	70	F	25	1	25.7	3.5	197	1.54	0.77	116.6	1.3	high		5.5	0.5		10.7
PS	49	M	27	1	17.7	3.7	150	1.73	0.98	77.16	1	high	9.5	4.3	1.69	28	9.7

PS	54	M	23	1	23.9	4.4	165	1.52	0.92	120.7	1.8	high		6.6	0.1	40	10.6
PS	46	F	25	1	28.1	4.1	137	1.47	0.79	105.1	1.3	high		3.4	0	21	9.8
PS	67	F	24	1	17.6	4.2	156	1.45	0.93	100.2	1.3	high		3.2	0	28	9.8
PS	45	M	12	1	23.4	4.3	132	1.24	0.84	107.8	1.6	high	8.8	5.8	0.2	23	10.2
PS	58	M	17	1	21	3.9	168	1.57	1	112	1.8	high	8.1	4.6	0.3	20	11.1
PS	54	F	16	1	21.7	3.8	196	1.29	1.12	115.8	1.8	high	9.5	4.1			11.8
PS	66	F	15	1	19.6	3.9		1.42	0.95	115.9	1.6	high	8.1	5.1		31	10.6
PS	52	F	17	1	18.3	4.2		1.16	0.86	108.1	1.8	high	8.8	6.1			10.6
PS	41	F	15	1	20.2	3.4		1	0.54	79.72	2.1	high	9	5.4			
PS	61	M	15	1	23.7	4.2	160	1.17	0.79	84.13	1.8	high		3.7	0.17	25	9.4
PS	67	F	17	1	16.8	3.8	125	1.26	0.87	130.3	1.8	high	9	3.9			9.9
PS	64	F	14	1	23.3	4.2	182	1.3	1.18	97.56	1.5	high		5.1	0.3	28	7.7
PS	43	F	14	1	21.3	3.7	184	1.3	0.94	106.4	2.1	high	8.7	5.1			10.8
PS	69	F	14	1	11.2	3.8	118	1.19	1.14	116.5	1.3	high		6.5	0.2	33	11
PS	33	M	10	1	23.9	4	184	1.19	1.06	108.8	2.1	high	8.9	4.9	0.1	0	10.2
PS	62	M	14	1	25.7	3.7		1.35	1.01	136.9	1.3	high		4.6	0.08	27	13.6
PS	56	F	12	1	21.7	3.8	141	1.47	0.87	127.5	2.1	high	9.2	6.7	0.1	27	11.5
PS	47	M	12	1	19.9	3.9	130	1.07	0.78	126.5	1.8	high	8.1	4.2			9.3
PS	75	M	12	1	18.2	3.7	166	1.7	1.11	120.2	1.3	high		6.8	0.23	43	10.5
PS	56	M	11	1	21.2	4	154	1.47	0.87	112.5	2.1	high		7.8	0.1	38	12.8
PS	39	M	10	1	20.1	4	185	1.24	1.03	107.2	2.1	high	8.4	6	0.52	27	11
PS	65	M	10	1	15.8	3.7	127	1.35	0.88	116.5	1.8	high	9	3.8			9.2
PS	49	M	10	1	18.7	4.1	121	1.32	0.78	82.77	2.1	high	9.5	6.8	0.02		10
PS	40	M	9	1	27.6	3.9		1.38	0.77	88.39	1.3	high	9.3	3.1	0.1	18	10.4
PS	36	M	12	1	20.9	3.8	184	1.31	0.74	110.4	1.3	high	7.9	3.8		29	9.3
PS	64	M	8	1	21	3.8		1.77	0.78	120.3	1.3	high	9.4	4.1	0.1	20	10.2
PS	59	M	7	1	22.5	3.6	209	1.81	0.88	107.8	1.8	high		7.5	0.1	27	11.2
PS	56	M	7	1	20.6	4.1	114	1.38	0.87	111	0.8	high	7.5	3.5		24	9.2
PS	56	M	7	1	23.9	3.8		1.52	0.96	119.8	1.8	high	8.6	4.4	6.3		10.2
PS	48	F	5	1	22	4		1.09	1.1	66.3	1.6	high	8.4	4.2	0	18	10
PS	61	M	5	1	14.3	4.4		1.45	0.73	85.14	2.1	high	9	3.5		20	
PS	74	M	5	1	20.1	3.7	143	1.6	0.6	85.62	1.3	high	9.8	5.1	0.1	39	9.1
PS	53	F	5	1	25.2	3.9	140	1.4	1.11	89.15	1.8	high	9.4	5.4			8.3
PS	52	M	5	1	22.4	4.2	140	1.62	1.09	96.18	2.1	high	9.6	5.2			9.5
PS	50	M	4	1	22.4	3.9	196	1.62	1.02	106.9	1.5	high	9.6	4.9			10.4
PS	64	M	4	1	24.7	3.5	107	1.14	1	101.2	1.8	high	8.8	7.2	0.27	30	10.8
PS	47	F	4	1	20.5	3.9		1.44	0.77	87.54	1.6	high	9.8	11.5	0	23	11.9
PS	40	F	3	1	17.1	3.8	140	1.17	0.99	88.22	1.3	high	8	5		22	7
PS	62	M	3	1	24.8	4.1	110	1.55	0.92	101.8	2.1	high	9.8	5.2	0	24	11
PS	56	M	3	1	19.9	4	147	1.49	0.72	79	1.5	high	8.4	4.3	0	19	9.1
PS	77	M	2	1	21.2	3.6	110	1.19	0.78	98.01	1.8	high	8.6	6.6			8.8
PS	65	F	2	1	19	3.9	138	1.4	1.05	72.1	2.2	high	8.4	5.9	0.14	13	11.4
PS	59	M	2	1	20.1	3.8	164	1.36	0.59	58.99	1.5	high	9	6.6			10.1
PS	25	F	2	1	25.6	3.9	123	1.38	1.16	109.7	2	high	9.7	6.1	0.03	43	8.9
PS	82	M	2	1	20.1	3.6	131	1.23	0.71	70.99	1.3	high	9.9	4.7	0.5	37	9.2
PS	62	M	2	1	17.8	3.6	152	1.13	1.26	99.68	1.8	high	8.5	7.1	1	33	12.8
PS	49	F	2	1	25.6	3.7	121	1.12	0.96	89.18	2.2	high	10	4.1	3.14	27	11.2
PS	76	F	8	1	23.6	3.8	212	1.78	1.01	90.85	1.8	high	9.2	2.8	0.02	29	10.9
PS	48	F	4	1	21.3	3.8	118	1.57	0.94	115	1.3	high	9.4	5.3	0.13	26	10.1
PS	44	F	20	1	19.9	3.7	126	1.32	0.91	114.8	1.8	high	9.5	7	0.1	10	
PS	64	M	22	1	20.4	3.9	115	1.63	1.02	110.5	1	high	9	3.4	1.14	30	11
PS	53	M	19	1	18.9	3.8	203	1.42	0.85	86.02	1.4	high	8.4	7.6	1.2	32	10.6
PS	56	F	25	1	17.2	3.8	239	1.88	0.77	106.9	1.4	high	9.8	4	0.15	23	9.7
PS	54	M	18	1	18.8	4	147	1.77	0.67	88.55	1	high	9.9	3.8	0.5	26	12.4
PS	53	M	28	1	18.3	3.8		1.47	0.98	60.12	1.6	high	8.2	3.2	0.2	38	10.9
PS	70	F	35	1	20.4	3.9	157	1.69	0.96	84.45	1.6	high	8.3	3.3		25	10.4
PS	59	F	18	1	17.3	4.3	168	1.42	1.12	120.6	1.8	high		6.6	0.2	30	10.9
PS	55	F	19	1	21.3	3.8	119	1.35	0.85	132.8	1.8	high	8.9	5.9	0.05	27	11.8
PS	64	M	21	1	17.9	3.7	141	1.29	0.75	98.4	1.8	high	10	6.1	0.09	29	10.4

PS	50	M	25	1	17	3.7	166	1.63	0.83	86.38	2.2	high	8.7	3.5	0.01	24	9.9
PS	62	F	34	1	18.2	4		1.44	0.75	92.98	1.4	high	9.7	3.8	0.26	24	12.1
PS	53	F	29	1	16.2	4	199	1.62	0.73	97.7	1.6	high		3.4	0.88	27	9.8
PS	79	M	14	1	24.4	4	181	1.31	0.84	129.6	2.2	high	9.2	6.4	0.08		8.9
PS	48	M	15	1	18.8	3.5		1.48	0.88	113.4	2.2	high		6.2			9.4
PS	66	F	17	1	20.2	3.6	177	1.44	0.97	103	1.5	high		5.9	0.06		11.1
PS	69	M	18	1	19.4	3.8	133	1.77	0.79	87.31	2.1	high	9.2	4.2	0.04	36	10.9
PS	84	F	17	1	16.6	3.8	182	1.51	0.81	109.4	1.5	high	8.9	4.7	0.2	28	10.7
PS	66	F	16	1	26.8	3.8		1.39	0.72	111.2	1.6	high	8.2	6.2	0.29	27	8.7
PS	59	M	16	1	23.3	4.1	135	1.93	0.96	116.7	1.6	high	9.1	5.8		29	10.5
PS	56	F	22	1	15.3	4.4	183	1.23	1.15	84.67	2.2	high	9.2	5	3.28	29	14.6
PS	84	M	15	1	15	3.7	126	1.17	1.23	96.24	1.3	high	9.5	3.7	0.1	25	10.2
PS	60	F	15	1	22.2	3.3	141	1.82	0.89	113.3	2.1	high	7.8	5	0.1	31	12.8
PS	80	F	13	1	15.2	3.7	171	1.75	1.04	93.73	1.8	high	8.7	4.5		30	10.5
PS	53	F	31	1	19.4	4	145	1.76	1.04	120.2	1.5	high		4.7	1.75		9.9
PS	59	F	14	1	17.6	3.8	147	1.56	1.12	106	1.8	high	8.5	6.2	1.68	27	11.2
PS	73	M	13	1	23.7	3.8	218	1.91	1.3	124.1	1.3	high	8.7	3.8	0.05	25	11.7
PS	37	F	13	1	15.2	4.2	163	1.35	1.15	90.2	2.1	high	9.3	5.5	0.1	16	11.6
PS	51	M	12	1	27.5	3.4		1.05	0.65	85.11	1.6	high	10.4	7.1	0.33	17	12.2
PS	41	M	11	1	19.7	3.9		2.07	0.82	101.1	1.6	high	8.9	4	0.46	21	11.6
PS	51	F	9	1	22.7	3.8	196	1.81	0.91	134.9	2.2	high	9.8	7.6	1.31	27	10.3
PS	55	F	10	1	24.6	3.8	106	1.47	0.93	103.5	1.5	high	7.7	3.5	0.09	24	10.7
PS	68	F	9	1	19	3.9	125	1.25	1	97.98	1.5	high	8.3	5.3	0.31	36	8
PS	69	M	12	1	20.3	3.6	219	1.68	0.96	113.5	1.3	high	10.2	7.2		19	9.2
PS	71	M	9	1	21	3.8	126	1.76	0.74	125.1	1.8	high		3.2	1.46	29	10.4
PS	33	M	9	1	22.6	4.4	118	1.17	0.88	89.71	2.1	high		6.9	0.01	32	11.2
PS	58	F	5	1	21	3.6	256	1.68	1.02	122.3	1	high	9.5	4.8	0.1	22	
PS	39	M	8	1	22.6	4.2		1.23	1	87.06	1.6	high	9.7	6.1		24	10.6
PS	55	M	8	1	22.4	3.8	183	0.78	0.91	110.2	2.1	high	8.9	5.5		22	6
PS	60	F	7	1	16.6	3.7	133	1.22	1.06	132.4	1.3	high	8.2	5.2			13.3
PS	62	M	7	1	21.4	3.8	118	1.26	0.9	104.1	1.8	high	9	2.9	0.05	27	10.5
PS	56	M	6	1	20.4	4	125	1.33	0.89	106.6	1.5	high		5.1			10.6
PS	58	F	5	1	19.1	4.1	160	1.49	0.8	114.3	1.4	high	10.7	4.5	0.1	21	
PS	46	F	5	1	14.5	4.1		1.3	0.51	53.31	1.8	high	8.2	5.5	0.97	24	11.1
PS	77	F	5	1	20.5	3.5	148	0.98	0.83	88.52	1.3	high		4.9	1	28	10.2
PS	62	M	5	1	17.2	3.6	130	1.61	1.07	89.95	1.8	high		6.9	0.4	29	9.3
PS	57	M	5	1	24.1	3.8	152	1.46	0.84	102.3	2.2	high		4.4			7.3
PS	64	F	4	1	16.7	4	145	1.7	0.76	120.9	1.5	high	9.2	4.3	1.25	29	7.8
PS	61	F	5	1	25.5	3.9	128	1.68	1.2	118.5	1.8	high	8.4	4.5	0.01	22	10.3
PS	67	F	4	1	20.5	3.5	121	1.19	0.84	89.35	1.5	high	9	6.6	0.01	24	10.5
PS	40	M	4	1	20.7	3.6	260	1.64	1.24	91.52	1.6	high		3.9		17	9.2
PS	63	M	3	1	24.2	3.6	136	1.29	1.05	118.2	1.5	high	7.3	4.3	0.5	34	10.5
PS	49	M	3	1	24.7	4		1.14	0.73	114	2.5	high	9.8	6.3		33	9.5
PS	47	M	3	1	20.9	4.3	138	1.39	0.96	116.3	2.1	high	9.7	5.8	0	28	11.5
PS	58	F	3	1	20.6	3.7	173	1.68	1.09	122.5	1.4	high	9.1	5.7	0.1	23	5.6
PS	59	M	3	1	20.3	3.7		0.95	0.85	107.6	1.5	high	8.1	5.8	0.1	21	9.6
PS	52	M	3	1	18.5	3.8	182	1.34	0.72	72.43	1.5	high	9	5.4	0.1	26	11.2
PS	85	M	2	1	22.8	4.1	154	1.38	0.92	90.8	1.4	high	8.5	4.8	0.6	15	
PS	59	M	2	1	21.7	3.6	132	1.54	0.75	91.73	1.8	high	10.2	5.3	16.4	29	8.7
PS	76	F	2	1	23.4	3.8	166	1.9	0.84	98.08	1.8	high	9.6	8.4			12.3
PS	58	M	2	1	28.1	4.1	149	2.6	0.75	88.06	1.8	high	9.4	2.1	4.87	30	11.5
PS	45	M	2	1	33.8	4.4	167	1.08	0.66	91.35	1.8	high	8	3.1	0	15	10
PS	47	M	2	1	22.1	3.9	198	0.92	0.76	98.57	1.5	high	8	4.7	0.13	21	9.1
PS	61	F	8	1	21.7	3.4	192	1.42	0.62	84.59	2.1	high	9.9	6.7			9.6
PS	36	M	5	1	20.8	3.9	89	1.24	0.97	101.1	1.3	high		6.8	0	17	9.1
PS	51	M	19	1	25.3		140	1.41	0.75	111	1.8	high	8.4	6.4	0	19	10.1
PS	76	M	4	1	29.3	3.8	106	1.42	0.91	109.8	2.1	high	8.3	7.2	0.7	32	10.4
PS	46	M	23	1	20.1	4.1	145	1.3	0.97	93.9	1.8	high	9.9	5	0	21	5.4
PS	70	M	8	1	19.8	4.2	177	1.65	0.79	129.1	1.4	high	8.1	5.2	0.1	21	

PS	70	F	2	1	31.9	3.5	148	1.41	0.82	54.48	2.1	high	8.8	5.4			11.5
PS	43	M	4	1	20.6	4.2	182	1.57	1.01	88.75	1	high	8.7	2.1	0	22	
PS	61	M	17	1	20.3	3.7	198	1.64	0.69	99.03	1.4	high	9.9	5	0.1	26	
PS	81	F	5	1	23.2	3.1	220	1.42	1.02	103.5	2.1	high	8.4	3.7		24	10.1
PS	52	M	13	1	19.9	3.8	171	1.39	0.94	132.9	1	high	11.4	3.5	0.1	43	
PS	60	M	8	1	22.1	3.5	140	1.63	1.11	95.65	1.5	high	8.7	2.8	3.5	33	12
PS	60	M	10	1	19.4	3.9	137	1.25	1.03	99.49	1	high	11.4	3.4	0.2	21	
PS	60	M	6	1	27.6	3.7	211	1.53	0.77	116.9	1.8	high	7.8	7.7	4.3	23	12.4
PS	80	F	12	1	20	3.6	148	1.32	0.76	133.5	1.8	high	10.7	4.3		25	10.3
PS	52	M	19	1	22	3.8	126	1.17	0.85	107.7	2.1	high	9.5	6.3	0.06	41	7.9
PS	59	F	2	1	31.1		137	2.12	0.83	84.83	1.8	high		3.9		19	9.9
PS	63	M	12	1	19.5		179	1.72	1.12	97.49	1.5	high	8.6	6.7	0.24	22	10.6
PS	77	F	4	1	20.7	3.5		1.35	0.95	128.2	2.5	high	8.9	3.6	0.06	35	11.2
PS	58	M	20	1	25.2	3	150	1.23	0.72	121.5	1.8	high	10	8	0.1	25	
PS	70	F	12	1	18.8	3.7	141	1.44	0.85	123.7	1.3	high	9.2	9.1	1.6	24	10.9
PS	65	M	8	1	21.8	4	131	1.7	0.89	121.5	2.1	high	8	4.3	0.1	22	11.8
PS	52	M	6	1	20.4	3.4		0.89	0.8	117.4		high	8	5.3			9.7
PS	77	F	2	1	17.5	4.3	120	1.44	0.88	137.6	1.5	high	7.6	7.3	0.63	25	10.3
PS	68	M	21	1	20	3.5	123	1.52	1.15	95.97	1.8	high	9	6.3		31	10.9
PS	45	M	19	1	17.5	3.9	170	0.56	1.19	69.31	1.8	high	9.2	6.2	0.05		8.8
PS	74	F	12	1	20.1	3.8	178	2.09	0.81	161.6	1.8	high	9.6	5.6	0.11	23	10.9
PS	76	F	5	1	22	3.4	214	0.98	0.91	111.4	1.5	high		5.1		19	12.6
PS	83	F	2	1	19.4	3.6		1.99	0.95	65.54	1.6	high	10.4	5.9	0	19	9.6
PS	75	M	10	1	22.7	3.1	124	1.48	0.66	119	2.1	high	8.8	6.7	0.02	15	10.3
PS	59	M	36	1	20.4	3.5	109	1.14	0.9	39.2	1.6	high		7.8	0.37	43	10.1
PS	62	F	29	1	22.3	3.7	172	1.51	1.01	99.8	1.6	high		6.5		20	10.9
PS	61	F	9	1	20.9	3.7	240	1.14	0.65	91.82	1.8	high	8.2	7.1	0.05	27	11.8
PS	69	M	8	1	27.1	3.9	171	1.53	0.98	116.5	1.3	high	8.7	4.8	0.17	27	11.3
PS	67	F	17	1	17.3	4.7	157	1.53	1.08	106.6	2.1	high	10	5.2	0.2	26	12
PS	74	M	8	1	19.1	3.7	115	1.51	0.82	124.7	2.1	high	9.5	7.8	0	23	10.7
PS	52	F	6	1	18.5	3.6	154	1.24	0.6	42.97	1.8	high	8.6	5	0.52	18	10.8
PS	63	F	2	1	19.4	3.9	224	2.08	0.69	3.46	1.6	high		4.2		20	9.8
PS	74	M	3	1	24.5	3.7	83	1.48	0.73	88.9	1.3	high	8.9	3.4	0.2	30	8.6
PS	67	M	21	1	21.1	3.3	203	1.54	0.81	100.6	1.6	high		4	0.26	30	10
PS	78	M	4	1	20.9	3.5	98	1.47	0.78	118.5	1.6	high	9.1	3.1	0.1	33	12.3
PS	76	F	28	1	21.5	3.5	151	1.18	0.89	142.7	1.8	high	9.2	6.9	0.1	18	10.4
PS	72	F	4	1	17.3	3.5	160	1.39	1.19	91.78	1.8	high	8.8	4.4	0.1	30	11
PS	75	M	3	1	19.6	3.9	154	1.52	0.86	92.64	1.8	high	8.7	4.1	0.07	26	11.3
PS	71	M	2	1	18.8	4	133	1.57	0.76	72.22	1.5	high	10	5.5	0.12	25	9.8
PS	36	M	5	1	26	3.9	170	1	0.69	79.34	1.5	high	8.9	2.6	0.31	27	11
PS	55	M	2	1	29.5	4.2	208	1.16	0.94	63.97	1.5	high	7.9	6.8	0.2	25	11.6
PS	40	F	12	1	18.3	3.1	132	1.14	0.75	65.38	1.5	high		6.4	0	23	10.3
PS	36	F	10	1	20.7	4.1	132	1.29	1	101.9	1.8	high		4.7	0	24	9.9
PS	65	F	5	1	20.2	4	156	1.44	0.93	131.7	2.1	high	8.2	2.6	0.06	22	10.1
PS	65	F	3	1	19.7	3.5	137	1.31	0.92	120.9	1.5	high	9.1	6.3	0.05	31	11.2
PS	76	M	3	1	18.3	3.5	170	1.43	0.78	107.8	1.8	high		5.7	0.2	31	11.8
PS	78	F	3	1	21.2	3.2	182	1.18	1.19	103.5	1.6	high	9.6	6.7	0.1	16	12.5
PS	73	M	3	1	19.4	3.7	151	1.03	0.92	77.22	1.8	high		3	0	30	10.8
PS	81	M	2	1	23	3.2	172	1.32	0.7	39.19	2.1	high	9.7	7.8	0	23	10.2
PS	68	F	3	1	18.3	3.4	140	1.33	0.72	131.5	1.8	high	9.8	3.3	4.75		8.3
PS	64	M	17	1	15.3	3.5	216	1.41	0.82	101.8	2.1	high	9.4	5	0.02	23	10.9
PS	54	M	11	1	21.9	3.7	82	1.38	1.3	85.51	1.8	high	8.1	5.3	7.5	49	7.2
PS	64	F	4	1	21.3	3.4	136	1.57	0.62	78.61	1.8	high	9.3	5.6			10.4
PS	60	F	2	1	24.8	3.8	119	1.23	0.83	82.3	1.3	high	9.1	4.7	0.65	26	12.3
PS	85	F	2	1	31.3	3.3	145	1.36	0.95	129.4	2.1	high		5.8	0.14	36	10.3
PS	61	F	2	1	19.3	4.1	125	1	0.77	88.58	1.6	high	8.3	4.8	0.02	24	12.7
PS	67	F	3	1	20.5	3.8	149	1.69	0.8	59.56	1.6	high	8.9	4.3	0.8	31	8.9
PS	60	M	4	1	22	3.8	138	1.06	0.94	128	1.8	high	9.7	4.8	0.06	32	9.8
PS	55	M	4	1	30.5	4.2	155	1.06	0.91	150.9	1.3	high	8.7	4	0.41	31	10.2

PS	77	F	5	1	22.6	4.1	173	1.7	1.13	107.1	1.3	high		3.5		23	9.1
PS	74	M	6	1	21.1	4.1	154	1.28	0.95	119.1	2.1	high	6.4	6.3	0.51	27	11.7
PS	63	M	6	1	20.5	3.8	145	1.22	0.8	90.27	1.8	high		4.4	0.79		9.8
PS	64	F	7	1	20.8	3.8		1.24	1.05	119.4	2.1	high	9.8	5.1	0.1	22	11.6
PS	58	M	9	1	21.8	3.9	121	1.27	0.86	81.16	1.6	high	9	5.6	0.17	22	9.1
PS	40	M	9	1	22.7	3.6	109	1.29	0.85	107.7	2.2	high	9.2	5.3	0.06	27	8.5
PS	64	M	9	1	25.1	3.8	184	1.54	0.98	166.7	2.1	high	9.3	6.4	0.15	53	10.8
PS	68	M	9	1	24.8	4.1		1.65	0.75	144.8	2.1	high	9.3	6.6	0.05	28	11.2
PS	50	M	10	1	25.6	4.1	119	2.12	0.81	119.6	1.5	high	8.2	2.6	0.08	44	8.8
PS	60	F	10	1	19.7	4	212	1.44	0.88	125.5	1.6	high	10.2	5.7	0.64	26	7.2
PS	66	F	21	1	15.9	3.8	151	1.39	1.14	108.5	1.6	high		5.5	1.2	28	9.8
PS	38	M	10	1	29.1	4.4	151	1.31	1	127.8	2.2	high	9.9	6.5	0	29	10.6
PS	43	M	7	1	21.5	4	157	1.32	0.8	83.49	2.2	high	9.4	5.5	0.3	37	12.2
PS	57	F	7	1	26.3	4.3	228	1.31	0.85	132	1.1	high	8.8	5.5	0.13	24	7.8
PS	53	M	6	1	21.5	4.3	130	1.31	0.95	123.5	1.8	high	10.4	5.4	0.14	34	12.1
PS	43	M	4	1	22.4	4.1	168	1.63	0.94	108.8	1.5	high	9.4	4.9		26	12.3
PS	60	M	6	1	23.5	4	108	1.75	0.8	91.18	2.1	high	7.6	4.2	0.17	27	9
PS	44	F	10	1	25.7	4.2	156	1.1	0.91	89.26	2.1	high	8	8.4	0.54	30	11.5
PS	71	F	6	1	18.8	3.8	197	1.77	0.95	123.5	1.5	high	9	7.2	0.16	36	10.4
PS	77	M	3	1	22.8	3.4	152	1.15	0.85	102.2	1.8	high	8.8	9.2	0.31	21	9.9
PS	57	M	13	1	19.2	3.9	118	1.41	0.78	127	1.8	high	7.8	2.9	0.29	24	11.4
PS	46	M	2	1	21.4	3.2	71	1.13	0.71	55.09	1.6	high	8.4	5.7	0.84	30	8
PS	60	M	26	1	21.4	3.7	153	0.91	0.93	119.8	2.1	high	8.4	7.7	2.39	44	9.7
PS	51	F	2	1	27.9	4.2	105	1.27	0.94	110.8	2.1	high	9.7	7.1	0.01	36	11.4
PS	47	M	2	1	26.3	4.2	168	1.53	0.85	93.06	1.3	high		4		36	9.9
PS	68	F	12	1	17.2	3.8	196	1.86	1.11	111.8	2.1	high	8.9	4	0.29	31	10
PS	59	M	7	1	21.2	4.2	111	1.2	0.79	116.7	1.6	high	9	3.6	0.03	22	10.8
PS	61	F	16	1	20.2	4	147	1.18	0.97	122.8	1.6	high	9.6	4		24	9.7
PS	52	M	29	1	18.3	4.2	203	1.32	1.17	92.73	2.2	high	10.2	5.2	0.14	28	10.7
PS	68	M	6	1	22.8	4.1	157	1.33	0.9	124.6	2.2	high	9.7	7.6	0.05	26	9.4
PS	61	M	3	1	18.4	3.8	105	1.3	0.82	114.2	1.6	high		4.2		21	9.8
PS	61	M	16	1	23.8	3.9	149	1.65	0.69	109.9	1.8	high	8.2	3.3	0.04	28	11.8
PS	54	F	11	1	17.1	4.2	144	1.37	0.77	122.9	2.1	high	8.3	2.2		28	9.5
PS	53	M	3	1	23.3	4.1	99	1.45	0.75	107	2.1	high	8.3	6.2	0.03	31	10.5
PS	85	F	5	1	15.2	4	133	1.37	0.88	99.29	1.5	high	7.9	7.6	0	34	8.9
PS	40	F	13	1	17.4	4	102	1.32	0.83	119.6	2.2	high	8.4	6.7	0.1	25	10.6
PS	69	F	12	1	22.8	3.6	131	1.53	0.86	126.2	1.6	high	9.3	3.6	0.5	25	9.2
PS	60	M	10	1	23.4	3.3	181	1.49	0.88	122.3	1.8	high	10.2	6.4	0.08	23	9.6
PS	69	M	6	1	21.7	4	178	1.54	0.99	84.21	1.5	high	9.2	5.3	0.08	21	11.5
PS	82	F	5	1	16.2	3.7	113	1.41	0.91	130.7	1.1	high	8	2.5	3.42	19	10.5
PS	72	F	4	1	24.7	3.9	143	1.7	0.87	96.17	1.8	high		5.7	0.1	33	10.3
PS	65	F	4	1	21.2	3.9	212	1.45	1.12	106.3	1.8	high	8.6	8.8	0.1	27	11.2
PS	61	M	4	1	21.6	3.3	140	1.13	0.63	102.4	2.1	high	9	6.7	0	22	11.3
PS	66	M	4	1	22.6	3.9	154	1.24	0.84	111.8	1.8	high		5.3	0.2	20	9
PS	75	M	3	1	20	3.3		2.23	0.75	98.61	1.5	high	8.7	4.4	0.38	26	11.2
PS	80	F	3	1	26	3.5	126	1.19	0.66	72.44	2.1	high	9.8	8			10.7
PS	72	M	2	1	21.1	3.9	120	1.46	0.9	120.3	1.3	high	8.6	2.4	0.33	31	9.9
PS	59	M	2	1	32.5	3.7	153	1.18	0.94	109.4	1.3	high	8	4.2	0.05	16	10.7
PS	49	M	2	1	23	3.8	135	1.54	0.83	91.61	1.5	high	8	4.6	0.05	23	10.2
PS	84	M	2	1	23.7	3.6	114	1.7	0.74	5.24	1.5	high	8.3	5	0.16	33	10.5
PS	52	M	20	1	19.2	3.5	212	1.69	0.92	92.78	1.6	high	7.8	6.6	0.29	31	10.8
PS	50	M	8	1	21.5	4.3	213	1	1.03	134.7	2.5	high	9.2	6.1	0.15	21	12.5
PS	44	M	7	1	26.7	3.5		1.44	0.71	88.48	1.5	high	9.8	4.9		31	9.5
PS	42	F	5	1	20.3	3.7	104	1.61	0.87	109.7	1.8	high	9.4	4.7	0.1	27	9.6
PS	69	M	4	1	19.5	3.9	195	1.31	1.01	201.9	1.8	high	9.4	5.7	0.04	20	11.4
PS	73	M	2	1	19.2	3.8	156	1.41	0.65	96.48	1.5	high	8.4	4.5	0.07	25	8.3
PS	47	M	6	1	24.6	4	122	1.63	0.79	123.2	1.8	high	9.7	5.2	0.2	23	9.3
PS	62	F	20	1	24.5	4.6	162	1.2	1.05	119.5	1.5	high		4.3	0.1	20	10.3
PS	59	F	19	1	21.4	3.7	154	1.8	1.11	123.5	1.8	high	9.3	3.6	0.02	24	10.5

PS	57	F	18	1	20.5	4.1	108	1.91	1.18	104.8	1.8	high		5			10.7
PS	61	M	17	1	18.3	3.9	143	1.06	1.03	98.31	1.8	high		4.7	1.1	25	10.7
PS	70	M	13	1	18.9	4	124	1.26	0.98	128.9	1.5	high		9.3	0.1	26	9.8
PS	52	M	13	1	23.8	4	175	1.18	0.99	127.2	1.8	high	8.8	2.6	0.5	63	10.6
PS	49	M	12	1	21.5	4.2	186	1.96	1.06	113.7	1.8	high	9.4	4.6	0.1	24	10.1
PS	62	M	12	1	23.4	4	121	1.12	1.05	93.78	1.1	high	7.6	2.6	0.04	44	10.9
PS	58	F	11	1	18.3	4	151	1.3	1.33	115.6	2.2	high	10.3	5.7	0.05	27	10.9
PS	52	F	9	1	18.8	3.9	114	1.12	1.12	114.6	1.8	high	7.3	7.6	0.04	30	13.1
PS	44	M	7	1	20.6	4.1	183	1.76	1.02	124.2	1.5	high	9.2	5.6	0.98	24	11.8
PS	46	M	7	1	20.8	4	131	1.16	1.26	122.8	1	high	9.4	2.1	0.1	18	4.8
PS	65	M	6	1	18.4	3.6	129	1.05	1.14	90.05	1.5	high	8.9	4.3	0.13	23	9.7
PS	64	F	6	1	18.9	4.3		1.7	1.09	110.9	2.5	high	9.2	5.9	0.1	37	12
PS	53	F	5	1	19.4	4.4	177	1.29	0.88	111.8	2.1	high	8.9	3.3	0	24	10.2
PS	63	M	5	1	22.8	3.4	135	1.59	0.75	112.8	1.3	high	9.7	4.7	0.1	26	10.6
PS	68	F	5	1	19.9	3.9	121	1.4	0.83	107.6	1.5	high		5.2	0	20	10.8
PS	63	F	5	1	17.1	3.9	153	1.71	1.26	110.4	1.1	high	10	5.2	5.3	36	10.6
PS	54	M	5	1	24	4.2	136	1.49	0.81	130.2	1.8	high		5.1	0.28	35	12.4
PS	59	F	5	1	24.6	4.2	144	1.09	1.09	132.7	1.5	high	10.3	5.8	0.05	25	10.3
PS	66	M	5	1	29.1	4	48	1.49	0.97	93.39	1.3	high	9.7	6.9	0.09	25	10.7
PS	49	F	4	1	18.9	4	122	1.3	0.74	87.03	1.5	high	9.4	3.8	0.13	28	10.1
PS	63	F	4	1	24.8	4	151	1.46	1.08	76.25	1.8	high		9.7		22	10.4
PS	59	M	4	1	21.9	4.2	133	1.21	0.91	69.98	2.1	high	8.6	4.7	0.06	26	9.3
PS	53	F	4	1	18.2	3.9	149	1.38	0.95	125.4	2.2	high	8.1	4	0.1	23	9.7
PS	49	M	4	1	21.1		196	1.42	0.95	84.47	1.8	high		5.4		22	10.2
PS	62	M	4	1	27.5	4.2	140	2.01	0.79	146.8	2.1	high	8.4	3.9	0.1		10.6
PS	57	M	3	1	24.8	4	176	1.42	0.71	79.29	1.5	high		6.6	0	21	11.4
PS	67	F	3	1	22.8	3.6	117	1.11	1.09	125.8	2.2	high	8.6	4.7	0.6	24	10.5
PS	50	M	3	1	17.4	4.4	136	1.29	0.98	107.2	2.1	high	9.5	6.8	0.1	27	10.9
PS	57	M	3	1	20.6	3.4	96	1.33	0.78	111.7	2.1	high	9	3.7	0	38	6.1
PS	66	M	3	1	23.1	4.2	155	1.02	0.82	83.74	2.1	high	9.2	5	0.05	31	9.6
PS	61	F	3	1	25	3.9	119	1.15	0.98	103.4	2.1	high	8.7	6.3	1.1	25	10.4
PS	74	M	3	1	23.4	3.9	171	1.4	0.96	139	1.1	high	8.7	4.5	0.08	27	10.2
PS	57	M	3	1	23.3	4.3	150	1.74	1.02	142.7	1.3	high		6.4	26.6	46	12.5
PS	74	F	3	1	27.9	3.7	154	1.26	0.78	89.79	1.5	high		8.5	0.05	28	11.2
PS	60	M	2	1	24	3.8	141	1.46	0.97	131.7	1.8	high	8.5	7.2	0.04	31	9.9
PS	64	F	2	1	21.1	3.8		1.72	0.82	102.7	1.5	high	6.3	5.6		28	9.2
PS	72	M	2	1	16.6	4.3	78	1.18	0.91	142.8	2.1	high	7.7	6.2	2.02	43	11.2
PS	65	M	27	1	22	3.7	158	1.34	1.09	111.1	1	high	8.7	6.4	0.4		11.7
PS	51	M	7	1	18.4	4.4	97	1.6	0.88	65.7	1.3	high	9.5	4.1			8.9
PS	63	M	3	1	21.5	3.7	141	1.29	0.89	98.46	1.5	high	9	3.4	0.1	32	9.6
PS	62	M	7	1	24.3	4.3	178	1.04	1.14	115.8	1.3	high	7	10.2	0.23	28	11
PS	61	M	5	1	24.1	4.4	152	0.85	1.07	147.5	1.8	high	9.5	4.9	0.01	21	10.6
PS	78	M	4	1	23	3.6	125	1.48	1.1	106.7	1.5	high	9.4	5.4	0.02	38	9.9
PS	81	F	3	1	22.1	3.6		1.39	0.92	112.3	1.1	high	9.9	5	0.03		11.5
PS	44	F	3	1	24.2	3.9	131	1.2	0.73	58.15	2	high	9.3	4.7	0.2	21	11.2
PS	66	M	8	1	18.7	4	159	1.38	1.05	126.9	2.1	high	8.4	5.7		25	11.9
PS	69	F	8	1	19.4	3.9	120	1.47	1.16	121.5	2.1	high	9.9	6.4	0.12	22	10
PS	63	M	3	1	15.9	4	146	1.44	1.05	104.3	2.2	high	8.9	5.9	0	19	11.3
PS	65	F	20	1	21		206	1.59	0.8	101.8	1.3	high		3.8	0	29	10.8
PS	35	F	10	1	17.1	4.1	142	1.51	0.85	100.3	2.1	high	8.3	6.3	0	23	11.6
PS	73	F	5	1	20.4	3.6		1.71	0.9	116.6	1.6	high	9	5.3	0.03		10.5
PS	67	M	4	1	25.6	3.6	154	1.3	0.86	71.09	2.1	high	8.8	5.7	0	13	10.7
PS	66	M	3	1	20.2	4.1	160	1.48	1.08	81.31	2	high	10	4.9	0.24	24	10.5
PS	65	M	3	1	19.9	3.9	138	1.6	0.91	90.5	2.1	high	8.1	6.1		30	11
PS	75	F	3	1	17.6	4.1	122	0.9	0.85	116.7	1.8	high	8	4.1			9.5
PS	76	F	3	1	30.4	3.7	151	1.39	0.88	122.1	2.5	high	10	5.1	0.65	22	11.2
PS	44	M	3	1	32.8	4	127	0.5	0.77	63.91	1.1	high		8.2	6.08		8.6
PS	52	M	2	1	22.1	4.1	160	1.87	0.94	80.84	2	high	8.5	5.5	0.09	16	10.8
PS	49	M	2	1	19	3.9	166	1.32	0.56	105.6	1.1	high		5.1	0.29	31	10.2

PS	56	M	2	1	20.6	3.4		1.47	0.65	49.59	1.5	high	9.5	3.6		26	12.8
PS	65	F	30	1	22.8	3.8	167	0.92	1.08	107.2	2.2	high		7.1		22	11.8
PS	68	F	26	1	22.3	3.9	146	0.97	1.12	89.76	1.4	high	8.3	6.4	0.18	22	11.4
PS	57	F	19	1	25	3.6	173	1.79	0.95	92.66	2.1	high	10	5	0.03	27	9.6
PS	53	F	15	1	16.7	3.2	148	1.41	0.99	125.6	1.3	high	8.5	5	11.92	28	9
PS	70	M	14	1	23.7	3.5	228	1.65	0.73	114.8	1.5	high	8.3	7.9	0.05	27	10.7
PS	60	F	11	1	22.4	3.9	164	1.87	0.83	94.17	1.3	high	9.3	5.3	0.3		12.2
PS	51	M	9	1	23.9	4		1.57	1.07	139.4	1.5	high	6.7	4.2		32	11.4
PS	48	M	8	1	18.4	4.2	174	1.83	1.15	110.9	2.1	high	7.3	4.8	0.21	26	9.7
PS	79	F	4	1	19.1	3.2	185	1.81	0.81	148.1	1.3	high	8.3	4.9		31	9.4
PS	89	F	2	1	18.8	3.8	171	1.66	1.04	76.33	1.8	high	8.4	5	0.1		10
PS	85	F	2	1	16.8	2.7	176	1.48	0.4	36.5	2.1	high		5.6			8
PS	36	M	2	1	18.7	4.3	164	1.21	1.04	71.75	2.1	high	9	4.8	0.11	22	8.6
PS	69	M	10	1	19.6	3.6	108	0.56	0.84	152.1	1.3	high	8.3	4.9	0.5	30	10.8
PS	67	F	15	1	22.1	3.6	177	0.98	0.93	117.8	1.8	high	9	7.6	1.5	22	9.9
PS	48	F	10	1	18.2	3.7	106	1.35	0.86	147.9	1.5	high	8	5.3	0.1	31	9.1
PS	58	F	9	1	19.4	4.1	151	1.42	1.12	121	2.1	high	9.9	4.3	0.1	23	10.7
PS	61	M	8	1	18.7	3.5	205	1.48	0.89	133.3	1.8	high	9.4	2.7	0.05	13	13.4
PS	44	M	7	1	22.8	2.4	150	1.18	0.87	63.5	1.8	high	9.2	3.4	0.2	22	6.4
PS	34	F	7	1	17.2	4.2	147	1.28	0.83	110.4	2.5	high	8.6	8.2	0.42	31	11.3
PS	65	M	7	1	21.8	3.6	143	1.17	1.05	114.1	2.5	high	9.1	6.5	0.43	23	10.5
PS	66	M	7	1	20.4	3.7	137	1.01	0.64	71.43	2.2	high	10.7	7.7	0.1	28	10.9
PS	40	M	4	1	23.1	3.9	148	2.44	0.9	103.3	2.1	high	10.6	6.9	0.36	40	9.8
PS	75	M	3	1	19	3.5	121	1.23	1.07	137.5	1.4	high	8	5.4	0.48	38	10.5
PS	60	M	3	1	20.3	3.9	174	1.55	0.93	105.6	1	high		5.3	0.05	28	9.9
PS	61	M	16	1	20.1	4.1	94	1.24	0.89	99	2.5	high	8.3	8.6	0.53	31	12.6
PS	70	M	15	1	18	3.7	113	1.05	1.12	126.5	1.8	high	8.1	6.1	0.1	29	9.2
PS	57	M	14	1	19.3	4	151	1.13	1.1	119.2	1.8	high	8.7	5.4	0.07	25	13
PS	64	M	13	1	16.7	3.7	146	1.68	0.83	138.1	1.8	high	8.4	4.7	0.07	24	10.6
PS	58	M	10	1	21.2	4.3	208	1.44	0.91	156.3	1.5	high	8.8	7.8	0.14	19	10.4
PS	67	M	10	1	22.6	4.1	116	0.98	0.84	107.7	1.5	high	9.2	6.8	5.15	36	8.8
PS	63	M	9	1	22.8	3.3	154	1.88	0.79	110	1.8	high	9.4	6.7	0.1	26	10.1
PS	60	M	8	1	26	4.1	114	0.94	0.73	113.9	1.8	high	9.5	5.3	0.2	21	9.5
PS	63	M	5	1	16.8	3.5	192	1.38	0.84	125.7	1.8	high	9.7	6.7	0.15	32	12
PS	72	F	4	1	18	3.8	140	1.34	0.93	105.3	2.5	high	9.7	4.4	0.05	26	10.8
PS	41	M	2	1	20.5	4.2	162	1.7	0.68	86.13	1.8	high	9.1	5.1	0.24	27	10.2
PS	35	M	2	1	31.3	4.6	116	1.46	1.03	126.4	1.8	high	8.2	8.2	0.2	30	9.3
PS	74	F	15	1	20	4	156	1.46	1.03	126.2	1.5	high	6.2	3.6	0.1	22	11.2
PS	66	F	12	1	21.5	4	202	1.18	1.07	115.3	2.5	high	8.6	4.9	0.33	28	11
PS	61	F	11	1	24.4	3.8	150	2.14	0.94	126.9	1.8	high	9.8	5.9	0.12	28	9.6
PS	44	F	9	1	33.4	3.7	178	1.93	0.89	91.99	1.5	high	8.7	4.2	0.02	22	10.5
PS	60	M	8	1	25.6	3.8	182	1.57	1.02	116.6	2.2	high	8.3	6.3	0.05	12	11
PS	58	F	6	1	20.6	3.6	183	1.63	0.99	86.21	1.8	high	9.3	3.5	0.2	27	9.2
PS	65	F	32	1	19.1	4.1	160	1.02	1.25	93.38	1.8	high	8.8	5.7	0.04		10.7
PS	82	M	4	1	15.3	3.8		1.3	1.39	83.37	2.1	high		4			
PS	59	M	11	1	18.7	3.6		1.45	1.06	125.6	1.5	high	10.6	4.5		27	11.6
PS	66	M	3	1	20.6	3.7	201	1.1	0.67	82.9	1.5	high		4.4	1.03	15	9.7
PS	87	F	2	1	21.9	3.6		1.27	0.85	121.9	1.5	high	9.4	6.2		35	12.5
PS	70	M	3	1	22.1		231	1.59	0.58	93.96	1.5	high	9.8	4.6	0.09	26	10
PS	63	F	8	1	22.1	4.4	135	1.37	0.94	110.9	2.1	high	11	5.7		26	11.3
PS	75	M	4	1	19.4	4.1	139	1.35	1.05	133.2	1.8	high	9.2	4.1	0.05	24	11.4
PS	81	F	7	1	21.1	3.7	144	1.2	1.02	98.52	1.8	high	9	6.5	0.1	25	13.5
PS	80	F	13	1	18.2	3.7	147	1.44	0.75	115.4	1.3	high	8.6	3.8	0	25	11.5
PS	50	M	11	1	31.9		153	1.06	1.17	114.6	2.2	high	8.3	6.1	0.05	25	10.3
PS	62	F	4	1	15.6	4.2	146	1.15	0.97	114.4	1.5	high		9.4	0.06	31	9.4
PS	68	M	9	1	22	3.8	154	1	0.63	73.56	1.1	high	9.6	7.7	1.6	33	12.7
PS	67	M	8	1	22	4.3	145	2.07	0.99	127.6	1.5	high	8.8	4.8	0.64		10.7
PS	56	F	7	1	21.3	4	144	1.43	0.88	81.24	1.8	high	10.7	7.6	0.05	29	10.7
PS	47	M	4	1	17.1	3.9	130	1.53	0.72	74.23	1.3	high	9.1	3.5	0.23	27	10.6

PS	56	M	2	1	22.8	3.8	161	1.73	0.61	94.91	1.6	high	8.2	4.7	0.1	26	9.9
PS	56	M	3	1	22.2	3.7		1.45	0.64	88.82	1.8	high	8.7	4.2	0.34	26	12.1
PS	62	F	5	1	29.7	3.8		1.1	0.95	78.94	1.5	high	8.6	6.2		32	9.5
PS	61	M	7	1	20.5	4.1	132	2.07	0.97	140.7	2.1	high	8.8	3.3	0.1	28	12.4
PS	62	M	2	1	22.4	4.1	161	1.12	0.94	90.77	1.5	high	9	3.1	0.63	27	10.7
PS	67	F	8	1	15.8	4.1	163	1.99	0.89	77.49	1.8	high	9.4	2.7		27	10.5
PS	34	F	7	1	21.6	3.8	140	1.33	0.89	119.1	1.8	high	10.1	6.3	0.2	26	9.2
PS	51	F	6	1	24.4	3.2	181	1.28	0.89	109.3	1.8	high	9	3	0.05	25	9.7
PS	44	F	37	1	13.9	3	119	1.99	0.89	60.57	2.2	high	10.4	3.9	0		12.1
PS	62	M	5	1	16.2	3.7		1.18	0.86	134.8	1.8	high	9.4	7.5		28	10.8
PS	73	F	2	1	25.9	3.5	148	1.6	0.75	127.3	1.5	high	9.1	4.2	0.21	39	10.2
PS	73	F	2	1	26.5	3.4	125	1.37	0.96	119.1	1.5	high	10	8.3	0.06	29	10.1
PS	55	F	3	1	20.4	4	127	1.69	1.16	85.33	1.3	high	8.5	6.1	8.61	41	8.5
PS	71	M	3	1	20.7	3.9	149	1.43	0.59	82.65	1.8	high	10.7	6.5	0.01	30	9.9
PS	70	F	3	1	20.1	3.8		1.57	0.91	94.84	1.5	high		3.5			
PS	62	F	26	1	19.6	3.9	142	1.54	0.92	118.4	2.1	high	8.5	6.3	0.78		11
PS	59	M	3	1	22.6	3.9		1.34	0.82	82.24	1.8	high		3.9			
PS	54	F	2	1	20.9	3.7	226	1.22	0.83	125.5	1.1	high	9.7	5.3	0.37	32	9.9
PS	67	F	5	1	18	3.7	187	1.33	0.82	139.1	1.5	high	9	7.8	0.02	24	9.9
PS	75	F	14	1	17.7	3.3	151	1.36	0.78	148	1.8	high		4.1	0.08	22	10.3
PS	69	M	2	1	18.1	3.6	119	1.56	1.01	93.43	2.1	high	9.4	6.7	0.1	25	9.7
PS	71	F	13	1	19.4	3.4	114	1.44	0.91	121.4	1.6	high	8.4	2.9	0.2	30	10.4
PS	58	M	14	1	24.7	3.9	163	1.62	0.93	101.3	2.2	high	8.5	7.4	0.1	27	9.5
PS	40	M	10	1	17.8	4		1.55	0.34	95.55	1.4	high	9.5	6.9	0.13		12.6
PS	67	M	4	1	22.4	4.1	144	1.71	1.17	113.3	2.1	high	9.9	5.9	0.06	25	11.4
PS	79	F	10	1	19.1	3.7	107	1.72	0.87	121.8	1.5	high		4.7	0.05	20	10.2
PS	73	M	8	1	23.1	2.8	104	1.17	0.52	65.58	2.1	high		4	0.03		13.6
PS	58	F	6	1	13	3.3	180	1.2	0.78	42.73	2.1	high		4.8	0.49	28	10.4
PS	53	F	3	1	16.2	3.8	117	1.14	1.03	90.79	2.1	high		4.6	0.01	32	12.6
PS	51	F	2	1	18.6	4	142	1.35	0.95	91.67	2.1	high		3.3	0.62	31	9.9
PS	50	M	27	1	17.6	3.6	159	1.2	0.93	98.01	2.2	high	6.3	6	0.18	28	10.5
PS	55	F	24	1	22.6	4.2	190	1.54	1.14	120.1	1.8	high		6.1	0.03	20	10.7
PS	44	M	16	1	25	3.8	202	1.07	1.13	113.9	1.4	high	8.9	5.6	1.3		8.9
PS	66	F	12	1	18.9	3.6	92	1.41	1.32	140	1.6	high	8.2	3.8	0.1	24	11.5
PS	75	M	9	1	24.4	3.6	110	1.14	0.66	103.3	1.8	high	9.4	7.2	0.12	27	10.1
PS	55	M	8	1	22.5	3.8	101	1.33	0.84	126.8	2.1	high	9.3	5		22	11.9
PS	59	F	7	1	23.9	3.6	136	1.42	0.65	120.6	1.8	high	9.7	4.5	0.2	16	11.9
PS	61	M	6	1	33	4.1	204	1.23	0.79	127.9	1.8	high	8.8	6.8	0.3	28	10.6
PS	60	M	4	1	20.7	3.5	97	1.3	0.81	107.1	2.1	high	9.6	5.3	0.2	15	9.5
PS	72	M	2	1	21.8	3.9	94	1.5	0.73	52	1	high	8.7	4.5	1.2	34	7.2
PS	44	M	15	1	28.2	3.9	197	2.08	0.83	89.14	2.1	high	9.7	7.9	0.1	23	10.6
PS	34	F	3	1	22.9	4.7	146	1.04	0.85	130	1.5	high	10	6.4	0.05	24	11.2
PS	61	M	2	1	21.6	3.9	198	1.8	0.85	115	1.5	high	10.5	5.5	0	22	10.2
PS	40	F	14	1	23.5	3.7		1.43	0.98	89.18	2.1	high	9.1	4.2	0.4	23	9.9
PS	65	M	10	1	21.3	3.7	134	1.17	1.19	99.35	2.1	high	9.7	7.2	0.7	29	12.6
PS	75	M	7	1	21.5	3.5	153	1.29	0.62	102.5	1.6	high	8.7	6.3	0	28	12.1
PS	71	M	11	1	25.9	3.2	93	1.21	0.64	95.42	2.1	high	9.4	5.5	0.4	31	9.4
PS	66	F	11	1	22.6	4.1	114	1.06	1.15	98.26	2.1	high		7.2	0.23	30	10.9
PS	61	F	33	1	22.4	3.2	134	0.95	0.57	76.26	2.1	high		7.1	0.04	30	10.7
PS	54	M	28	1	19.4	3.5	160	1.4	1.11	142.5	1.4	high	10.9	6.5	0.05	26	11.6
PS	57	M	15	1	19.1	4	137	1.2	0.94	136.9	2.1	high		4.5	0.01	27	11.5
PS	51	M	10	1	24.1	4	132	1.76	0.88	106.8	1.5	high	9.8	5.8	0.01	28	12.4
PS	68	F	21	1	24.3	3.8	167	1.27	1.18	114.7	2.1	high	10.3	4.6			12.3
PS	64	F	5	1	16.7	3.9	144	1.15	1	86.36	2.1	high		9	0.2	22	10.8
PS	45	M	15	1	23.5	3.9	89	1.69	1.08	118.6	1.8	high	8.5	3.5	0.29	30	9.9
PS	62	M	12	1	25.8	4	97	1.44	0.79	143.1	1.6	high	8.4	4.7	0.8	30	10.4
PS	43	M	6	1	19.9	3.9	139	1.43	1.08	109.1	1.5	high	10.3	4.9	0.2	24	11
PS	47	M	2	1	22.7	4	125	1.05	0.86	95.69	1.5	high		3.7	0.04	23	9.7
PS	59	F	4	1	23.4	4		2.05	1.5	114.8	1.6	high	9.8	3.3	0.1		9.4

PS	58	F	15	1	20	3.6	173	1.65	0.91	113.4	1.5	high	8.7	4.8			11.9
PS	61	F	14	1	20.5	4.1	117	1.45	0.95	113.4	2.1	high	9.2	5.1			9.8
PS	68	M	8	1	22.6	3.5	133	1.63	0.82	147.2	1.8	high	8.3	5.2		25	9.9
PS	78	F	19	1	18.6	3.9	117	1.16	0.81	106.7	2.1	high	8.6	5.1	0.13	21	10.9
PS	71	F	5	1	17.8	3.8	111	1.5	0.9	102.2	1.8	high	9.4	4.8	11.4	33	12
PS	53	F	8	1	19.5	3.8		1.57	0.9	89.33	1.3	high	8.5	3.6	0.05	23	10.4
PS	53	M	5	1	20.3	3.8	154	1.13	0.85	80.82	2.5	high	9.7	4.7	0.25	32	11.9
PS	58	F	2	1	22.8	3	157	2.31	0.71	59.79	2.1	high	9	3	1.7	28	11
PS	63	M	6	1	20.7	3.8	139	1.59	0.7	83.4	1.5	high	8.6	4.3	0.29	30	11.3
PS	47	M	4	1	20.1	3.8	138	1.36	0.96	76.9	1.8	high	9.2	4.7	0.01	36	8.7
PS	61	F	6	1	17.4	3.8	174	1.54	0.86	99.26	1.5	high	9.4	3.1	0.01	20	10.7
PS	58	M	8	1	19	3.8	218	1.02	0.7	79.46	2.1	high	9.4	6.7		31	
PS	43	F	11	1	20.6	3.8	100	1.37	0.84	82.96	2.1	high	7.9	8.3	0.08	27	9.4
PS	53	M	13	1	20.9	3.8	199	1.76	0.94	78.61	1.1	high	9.7	4.4	0.03	24	12
PS	34	M	4	1	22.4	3.8	154	1.13	0.89	70.3	2.2	high		15.1			9.4
PS	68	M	2	1	18.4	3.8	134	1.69	1.02	89.29	1.8	high	11.4	5.5	0.2	19	10.6
PS	61	M	11	1	18.9	3.8	160	1.93	0.84	82.88	1.8	high	9.2	5	0.03	20	10.3
PS	74	F	18	1	16.8	3.8	146	1.04	1.05	116	1.5	high		4.1	0.59	24	8.9
PS	59	F	7	1	16.8	3.8	135	1.55	0.73	101.4	1.5	high	9.4	5.6	0.6	34	9.2
PS	56	M	30	1	19.2	3.7	70	1.46	1.27	78.75	1.6	high	9.3	1.3		49	8
PS	67	M	10	1	19.6	3.7	147	1.41	1.02	112.8	1.5	high	8.3	6	0.3	26	12.2
PS	57	M	10	1	23.5	3.6	119	1.72	0.9	99.51	2.2	high	10.1	7.6	0.01	26	9.7
PS	47	M	5	1	19.7	3.8		1.27	1.02	109	2.2	high	9	7.7	0.1	20	11
PS	68	M	4	1	22.8	3.6	154	1.18	1.13	112.9	2.2	high	9.6	4.2	2.9	21	10.3
PS	60	M	4	1	23.3	3.7	217	1.53	0.72	75.11	2.2	high	9.3	6.5	0.1	19	11.5
PS	75	M	2	1	19.3	3.4	168	1.19	0.7	68.09	1.8	high	9.4	5.3	0	29	14.4
PS	59	M	2	1	24.2	3.9	158	1.36	0.82	100.3	1.5	high	8.3	3		26	11.9
PS	78	M	7	1	20	3.9	132	1.25	1.12	124.9	1.4	high		7	0.08	23	9.8
PS	48	M	6	1	19	4.4	171	1.06	0.98	106.6	1.8	high	11	4.7	0.11	23	9.1
PS	83	F	2	1	13.9	4.1	146	2.96	0.87	87.32	2.1	high	8.4	4.6	0.1	19	10.4
PS	46	F	12	1	17.7	4.1	128	1.35	1.14	132.8	2	high	8.2	5.4	0.1	27	11.5
PS	57	M	9	1	19.8	4		1.47	0.83	131.1	2.1	high	11.8	4.4		19	
PS	70	M	8	1	20.7	3.8	166	1.19	0.83	80.57	2.1	high	8.3	6.3	0.7	29	10.4
PS	60	M	6	1	22.7	3.8	247	1.39	0.87	131.8	2.2	high	9.4	7.9	0.1	20	11.7
PS	61	F	2	1	20.2	4.2	160	1.07	0.87	66.29	1.6	high	8.2	4.6	2.51	29	10.5
PS	66	M	6	1	22.5	3.5	179	1.5	0.93	162.1	2.2	high	9.6	5.6	0	19	11.1
PS	68	M	7	1	23.3	3.8	109	2.8	0.64	127.5	2.1	high	8.8	4.9	0.1	26	13.5
PS	61	M	7	1	22.7	3.5	166	1.58	0.64	119.2	2.2	high	9.9	3.2	0.1	12	12.3
PS	69	M	3	1	19.2	4	138	1.26	0.92	145.8	2.2	high	9.3	5.7	0	14	10.5
PS	62	F	3	1	20.2	3.8	134	1.62	0.71	85.63	2.1	high	9.5	6.9	0.05	22	10.7
PS	58	M	7	1	16.9	3.6	130	2.47	0.76	122.1	2.1	high	8.6	4.2	0.1	28	12.4
PS	60	M	5	1	19.5	3.2	146	1.73	0.85	93.06	2.1	high	8.7	6.4	0.2	27	11
PS	62	F	2	1	26	4.2		1.13	0.75	48.51	1.5	high	8.2	4.9	0.2	17	11.6
PS	64	M	2	1	19.9	3.2	121	1.76	0.71	53.82	1.3	high	8.1	3.8	3.47		9.7
PS	64	F	7	1	20.2	3.9	114	1.52	0.54	132.8	2.5	high	8.8	6.2	0.1	33	13.9
PS	75	F	3	1	33.2	3.6	100	1.44	0.64	78.26	1.3	high	8	5.4	0.1	17	9.3
PS	47	M	2	1	34.9	4.2	150	1.34	0.83	76.98	2.2	high	9.5	6.5	0.1	20	10.5
PS	54	M	18	1	18.6	4	166	1.47	0.97	120.3	1.3	high		6.1	0.2	23	10.5
PS	36	M	8	1	28.6	4.2	144	1.74	0.92	134	1.4	high	10	5.4	0.05	36	
PS	61	M	6	1	20.8	3.3		1.79	0.58	91.28	1.6	high	9.5	3.2		37	10.5
PS	54	F	2	1	26.4	3.8	162	1.12	0.71	31.11	1.6	high	8.1	5.1	0.1	23	8.7
PS	56	M	2	1	23.5	4.6	137	1.27	1.07	74	2.1	high	8.9	5.6	0.09	46	11.6
PS	33	M	7	1	21.7	3.9	220	1.55	0.83	109.2	1.5	high	9.1	3.9	0.04	24	10.2
PS	49	F	10	1	20.2	3.7	187	1.67	1.01	103.3	2.1	high	8.3	4.6	0.28	26	12.8
PS	37	M	11	1	20.6	4.4	119	1.06	0.77	116.1	2.5	high	8.8	5.5	0.69	34	9.3
PS	76	F	10	1	22	4		2.02	1.12	118.8	1.5	high	9.9	5.3	0.05	34	10.3
PS	49	M	19	1	21.7	3.7	194	1.33	0.79	118.8	1.8	high		7.5	1.53	29	9.7
PS	81	F	6	1	18.1	3.5	114	0.89	0.92	49.76	2.1	high		5	0.05	22	12.1
PS	55	F	7	1	21.8	4.3	145	2.19	0.89	135	1.9	high		3.7	0.05	28	9.6

PS	38	M	21	1	18.8	4.1	165	1.09	0.96	104.5	1.1	high	7.7	4		17	10
PS	50	F	6	1	24.6	4.1	191	1.05	0.58	83.16	2.2	high	8.2	3.6	0.04	15	9.9
PS	67	M	2	1	22.3	4.1	172	1.18	0.76	121.6	2.1	high	9.4	5.1	0.06	23	9.8
PS	70	F	6	1	24.9	3.4	149	1.54	0.69	65.45	1.5	high	9.6	5.3	0.2	25	11.6
PS	42	M	5	1	24.1	3.7	154	1.49	0.86	117.3	1.3	high	8.6	3.6	0.32	22	11.4
PS	59	M	18	1	21.3	4.3	124	1.04	1.12	109.6	1.5	high	9	4	0.1		8.3
PS	74	F	18	1	19.2	3.3	171	1.4	0.81	70.55	2.1	high	10	4.9	0.01	31	8.9
PS	52	F	7	1	22.3	3.7	195	1.38	0.81	104.2	2.5	high	8.6	7	0.23	30	12
PS	64	M	2	1	19.4	3.7	173	1.25	0.69	25.6	1.5	high	9.6	5.2	0.3	27	6.7
PS	66	F	8	1	23.5	4.1	113	1.58	0.95	139.8	2.1	high	8.5	6.3	0.02	30	10.8
PS	54	M	2	1	17.1	2.3	205	1.43	0.74	58.12	2.2	high	8	5.3		38	10.8
PS	64	F	6	1	21.5	3.9	124	1.65	1.03	128.2	1.8	high	9.8	8			7.8
PS	33	M	12	1	18.4	4.3	237	1.56	0.98	110.6	1.5	high	9	5.2	0.22	30	9.9
PS	72	F	3	1	18.4	4.3	178	0.96	1.03	106.4	2.1	high		6.2	0.07	30	11.9
PS	58	F	10	1	19.1	3.9	201	1.27	0.69	100.1	2.1	high	9.4	4.9	0.05	31	9.8
PS	76	F	4	1	26.1	3.3	174	1.52	0.76	79.58	1.8	high	9	5.6	0.38	30	10.7
PS	64	M	17	1	20.5	3.9	95	1.01	1.01	119.7	2.2	high	8.9	5.4		33	11.7
PS	44	M	13	1	20.8	4.1	178	1.7	1.42	104.5	2.1	high	10	4.8	0.02	23	11.1
PS	71	F	10	1	27.9	3.3	135	0.99	0.68	119.8	2.2	high	10.1	6.4		32	12.4
PS	58	M	10	1	22.4	4.1	150	1.43	0.91	101.2	1.8	high	7.5	4.1	0.14	23	8.1
PS	41	M	7	1	20.4	4.3	228	2.31	0.97	89.98	2.1	high	8.1	4.3	0.1	26	12.7
PS	62	F	7	1	21.5	3.6	194	1.61	0.72	115.3	1.8	high	10.2	7.3		28	9.7
PS	56	M	7	1	20.7	4.3	132	1.22	0.69	103.1	2.1	high	9	5.6	0.1	11	10
PS	55	F	7	1	15.4	3.4	135	1.19	1.15	98.23	1.5	high	8.3	5	0.13		10.7
PS	70	F	6	1	23.9	4	177	1.66	0.99	88.04	1.5	high	10.4	7.2	0.32	43	10.4
PS	43	M	5	1	23.4	3.7	102	1.41	1.12	111	1.8	high	9.8	5.6	0	30	13.1
PS	59	M	5	1	23.8	3.7	141	2.2	0.83	103.9	2.1	high	8.5	4.6	0.2	31	11.8
PS	64	M	4	1	23.1	4	154	1.22	0.88	102.2	1.3	high	9.1	4.5	0.03	25	9.6
PS	63	F	4	1	18.6	3.9	130	1.45	0.76	89.79	1.3	high	8.5	4	0.06	26	8.6
PS	69	M	3	1	17.8	3.9	161	1.44	1	102.5	1.8	high	8.8	4.7	0.08	25	8.7
PS	52	F	3	1	16.8	3.6	147	1.56	0.82	79.25	1.6	high	9.8	5.6	1.11		10.1
PS	63	M	3	1	19	3.4	179	2.06	1.25	84.15	1.3	high	9.3	5.4	0.06	27	9.6
PS	44	M	3	1	19	4.1	197	1.71	1.12	98.79	1.8	high	10.4	6.7	0.07	23	9.5
PS	55	F	2	1	23.5	3.8	180	1.27	0.82	97.92	2.1	high	10.2	5.3	0.36		10.9
PS	39	F	17	1	18.1	3.9	152	1.86	1.03	103.9	1.8	high	9.1	4.9			9.8
PS	71	M	14	1	20.4	3.8	164	1.4	0.88	108.3	1.6	high	9.7	4.9	0	24	9.5
PS	54	F	11	1	19.4	4.2	135	1.29	0.79	94.99	1.5	high		4.8	0.04	21	11.6
PS	56	M	9	1	26	3.6	179	1.2	0.91	71.7	1.5	high	9.9	6.9		27	10.9
PS	42	M	8	1	20.3	4.2	155	1.58	0.94	92.45	2.2	high	8.5	2.9		37	8.9
PS	72	M	6	1	20.9	3.7	94	0.86	1.23	120.8	1.5	high	8.4	5.3	0		11
PS	60	F	4	1	18.9	4.4	166	2.02	0.86	121.4	1.5	high	8.3	4	0.18	23	10
PS	63	M	4	1	14.9	3.9	117	1.52	1.18	130.1	1.5	high	8.2	5.4	0.1	26	10.2
PS	71	M	2	1	21.3	4.2	166	1.18	0.87	87.03	1.3	high	9.5	7.1			10.7
PS	60	M	2	1	20.5	4.4	140	1.68	0.71	78.48	1.8	high	8.6	5.4	0	34	9.3
PS	43	M	4	1	27	3.9	228	1.59	0.8	65.67	1.5	high		6.1	0.05	25	11.3
PS	57	F	2	1	23.4	4.1		1.9	0.86	51.88	1.8	high	10.1	5		19	
PS	57	F	2	1	24.2	3.6	176	1.86	0.9	129.3	1.8	high	9.5	5.6	0.1	26	12
PS	66	F	3	1	21.2	3.5	113	1.22	1.15	127.2	2.1	high	8.6	4.2	0.09	26	9
PS	70	M	13	1	19.9	3.4	160	1.53	0.71	116	2.1	high	8.7	7.3	0.2	26	12.7
PS	77	F	6	1	24.4	3.6	175	1.21	0.71	74.15	2.1	high	8.6	11.1		32	10.7
PS	68	F	2	1	18.4	3.3	253	1.78	0.73	60.44	1.5	high	9.6	3.1	0.1	28	11.9
PS	68	M	30	1	18.7	3.2	131	1.33	0.8	69.27	2.2	high		6.8	0.43	28	10.6
PS	66	F	19	1	17.6	3.8	236	1.5	0.94	125.4	2.2	high	9.4	3.1		42	10.6
PS	73	F	4	1	21.8	4	155	1.77	0.97	108.9	1.8	high		5.1	0.05		10.3
PS	70	F	2	1	19.8	3.9	180	1.5	0.77	79.34	1.8	high		4.7	0.29	23	10
PS	73	M	6	1	21.2	3.3	130	1.36	0.94	69.13	1.5	high	8.1	4.5	0.05	27	10.4
PS	70	M	3	1	20.6	4.3	163	1.29	0.98	121.1	1.5	high	8.4	6.8	0.5	28	10.6
PS	62	F	6	1	24.4	3.3	145	2.26	0.73	97.18	2.5	high	9.3	5.3	0.1	30	12.6
PS	67	F	19	1	24.4	3.3	122	1.6	0.75	127.7	1.8	high	11.1	5.2	0.2		9.9

PS	54	M	18	1	24.4	3.6	152	1.36	1.06	143.4	1.8	high	10.1	5.6	0.04	23	11
PS	52	M	15	1	19.4	4	194	1.13	0.95	136.6	1.8	high	9.4	5.3	0.68	27	10.8
PS	52	M	13	1	20.7	3.9	151	1.36	0.82	81.76	1.8	high	8.8	5.2	0.03	22	9.7
PS	61	F	13	1	21.7	4.2	116	1.36	1.19	94.45	1.5	high		4.8	0.18		10.7
PS	72	F	12	1	22.1	3.6	199	1.79	0.8	33.03	1.5	high	10.7	6.7	0.01	32	11.1
PS	60	F	12	1	22.7	3.7	152	1.55	0.79	112	1.8	high	9.3	5	0.1		9.4
PS	28	M	11	1	21	4	105	1.33	0.89	106.7	1.8	high	8.3	5.3	0.24	25	9.8
PS	51	M	11	1	18.7	3.8	127	1.71	0.92	87.3	1.6	high	9.5	6.1	0.03	32	9.7
PS	41	M	11	1	24.7	3.7	189	1.65	1.06	132.9	1.8	high	9.5	4.4	0.01	24	10.1
PS	58	M	9	1	25.5	3.7	168	1.29	0.97	128.3	2.1	high	7.5	5.5	0.1	26	8.9
PS	39	M	9	1	21	3.8		1.54	0.87	142.3	1.8	high	7.3	4.2	0.2	21	10.7
PS	73	M	9	1	20.2	3.7	122	1.36	1.04	133.2	2.1	high	8.7	4.2	0.1	31	11.3
PS	73	F	8	1	21.4	3.2	167	1.67	1.02	84.71	1.6	high	8.9	5.4	0	29	9.9
PS	61	F	8	1	20.3	3.7	165	1.6	0.88	103.4	1.5	high	9.8	3.2	0.25	29	9.3
PS	57	F	8	1	16.8	3.7		1.39	0.96	135.4	2.1	high	9	5.1	0.1	32	9.4
PS	53	M	7	1	27.9	3.6	156	1.46	0.68	111.8	1.8	high	8.2	7.2	0.03	29	11
PS	80	F	7	1	22.7	3.8	153	0.99	0.65	111.1	2.1	high	8.8	6	0.05	28	10.1
PS	52	M	6	1	20.6	3.8	143	1.2	0.81	87.67	1.6	high		6.2	0.17	55	12.4
PS	84	F	6	1	20	3	149	1.16	0.8	124.3	1.3	high		5.3	4.24	33	11.5
PS	60	M	6	1	28.1	3.6	162	2.34	0.89	105.7	1.3	high	8.4	2.5	0.99	39	7.9
PS	59	F	5	1	21	3.2	150	1.26	0.91	111	1.3	high		3.3	0.07		10.8
PS	54	M	5	1	27.4	3.7	132	1.65	0.62	118.5	1.5	high	8.3	4.2		47	9.7
PS	44	M	5	1	25	3.9	119	1.07	0.96	127.4	1.5	high	8.3	6	1	29	8.4
PS	53	M	5	1	19.3	3.8	153	1.22	1.06	55.64	1.5	high	9.7	3.3	0.08	21	11.4
PS	79	M	5	1	21.3	3.2	176	1.09	0.71	80.28	2.1	high	9.2	6.1	0.93	26	9.8
PS	48	M	4	1	20.2	4.3	178	1.3	0.81	85.72	1.6	high	8.9	5.6	0.05	36	11.6
PS	40	M	4	1	21.9	3.6	105	1.2	0.97	110.7	1.5	high	9.7	4	2.1	34	9.5
PS	83	M	3	1	21.6	3.7	145	1.21	0.82	125.8	1.8	high	9.1	5.2	0.03	17	10.5
PS	51	M	3	1	17.9	3.7	128	1.55	1.01	70.08	1.8	high	8.6	4.5	0.93	27	10.4
PS	69	F	3	1	19.4	3.4	144	1.74	1.09	112.8	2.1	high	9.3	3.7	0.3		
PS	67	M	3	1	27	3.8	177	1.36	0.91	116.9	2.1	high	8	5.6	0.29	32	9.1
PS	64	M	3	1	26	3.5	135	1.85	0.84	120.1	2.1	high	8.7	5.2	0.05	33	9.9
PS	63	F	3	1	22.8	3.4	121	1.38	1.07	112.5	2.1	high	8.7	4.6	0.1		10.4
PS	77	F	2	1	23.6	3.7	237	0.99	0.81	110.3	1.3	high	8.5	5.6	0.1		11.6
PS	47	M	2	1	19.3	4	180	1.37	0.89	88.53	2.1	high	10.2	5.3	0.01	34	7.6
PS	41	M	2	1	37.8	4	93	1.12	0.67	73.46	1.8	high	9.2	3.4	0.62	35	8.1
PS	64	M	2	1	24.6	3.5	149	1.91	0.91	73.39	1.8	high	8.5	6.2	0.01	31	10.2
PS	62	F	9	1	16.7	3.9	119	1.34	0.9	122.5	1.8	high	8.2	4.2	0.44	58	10.3
PS	67	M	8	1	19.7	4.2	127	1.56	0.75	106.3	2.1	high	10.2	3.8	0	24	11.7
PS	56	F	4	1	20.7			1.58	0.92	118.1	2.1	high	10.2	7.1		37	11.1
PS	41	M	6	1	24.2	4.1	171	1.12	0.74	48.24	2.2	high	7.9	6	0.02	33	11.5
PS	42	M	6	1	20.5	3.8	139	1.1	1.26	68.6	2.2	high	8.3	3.9	0.02	36	9.9
PS	72	F	11	1	21.8	3.8	137	1.2	0.9	119	2.2	high	8.8	4.5			8.7
PS	59	M	5	1	19.2	3.9	105	1.26	0.89	121.6	1.5	high	8.9	5.4	0.1	22	12.1
PS	62	M	3	1	22.6	3.7	167	1.56	0.82	98.88	1.4	high	9.3	6.8	0.05	33	10.1
PS	65	M	3	1	22.6	3.7	189	1.36	0.95	104.5	1.4	high	9.2	5.9	0.05	22	10.6
PS	49	F	2	1	15.3	4		1.48	0.74	82.06	2.5	high	9.2	8.4		31	9.8
PS	67	F	2	1	26.9		145	1.94	1.02	86.33	1.3	high	8.9	5.8	0.05	23	9.3
PS	43	F	4	1	23.7	3.9	138	1.69	1.19	90.26	1.5	high		3.7	0.1		11
PS	59	F	32	1	15.6	4.2	126	1.17	1.22	89.58	1.8	high	8.7	5.7	0.05	24	11.5
PS	78	M	29	1	21.2	4.1	212	1.33	0.96	103.3	1.8	high	8	5.7	0.05	28	11.5
PS	68	M	2	1	25.2	4.3	148	1.47	0.96	120	2.1	high	9.7	6.3	0	25	11.7
PS	72	M	2	1	25.7	3.6	175	1.32	0.58	70.36	1.8	high		6.6		30	10.2
PS	55	M	5	1	25.7	4	140	1.46	0.56	104.7	1.5	high	8.3	3.6	0	41	11.3
PS	70	M	9	1	19.2	4.1	159	0.88	1	122.6	1.6	high		4.2	0.12	27	6.2
PS	59	M	10	1	23.9	4.3	258	1.59	0.78	106.3	1.6	high	8.9	5	0.1	22	12.1
PS	58	M	5	1	23.1	4	124	1.89	0.97	118.8	2.1	high	9.5	4.1	0.1	29	10.7
PS	35	M	14	1	16.8	4.2	123	1.37	0.83	91.88	2.1	high	9.8	7.1	0.14	34	12
PS	48	M	15	1	22.9	4.2	130	1.4	0.93	122.5	1.8	high	8.1	6.3	0.11	34	10.9

PS	41	M	3	1	23.7	3.8	136	1.72	0.9	72.21	1.8	high	9.8	6.3	0	22	11.1
PS	58	M	20	1	20.5	3.9	111	1.64	0.96	116.3	1.4	high	8.1	5.3	0.1	24	10.6
PS	59	M	9	1	17.9	4.2	163	1.27	0.69	97.41	2.1	high	9.4	5.8	0	24	10.1
PS	55	M	9	1	21	4.4	176	1.27	0.7	111.3	2.1	high	10.8	6.1	0.01	26	11.4
PS	80	M	7	1	18.2	3.9	159	1.05	0.79	106.5	2.2	high	8.7	6.7	0.68	32	12.9
PS	58	M	5	1	18.8	4.5	115	1.45	0.88	125.4	1.8	high	5	6.6	0	21	10.8
PS	53	M	32	1	18.4	3.9	200	1.64	1.44	93.4	2.2	high	10	7.3	0.03	23	11.6
PS	55	M	27	1	18.9	3.8	172	1.28	1.07	107	2.2	high	10	7.9	0.11	27	9.2
PS	56	F	16	1	18.6	3.8	147	1.39	1.42	93.84	2.2	high	8.9	6.1	0.06	23	10.7
PS	73	M	14	1	19.7	3.5	183	1.75	0.88	126	1.4	high	9.6	6.4	0.11	41	10.2
PS	80	M	12	1	20.9	3.6	99	1.32	0.82	130.1	1	high	8.6	4	4.74	20	10.6
PS	59	M	12	1	19.4	3.9	181	1.61	1.07	110.3	1.8	high	10	6.8	0.6	21	10.6
PS	64	M	9	1	22.8	3.7	202	1.7	0.95	133.9	1.5	high	9.1	6.6	0.63	31	9.7
PS	43	M	8	1	23.4	3.9	182	1.46	1	104.1	2.1	high	8.2	2.9	0.32	30	10.6
PS	65	F	8	1	23.9	3.6	179	1.13	0.96	129.5	2.1	high		9		29	11
PS	65	M	7	1	21.1	3.8	140	1.09	0.98	127	1.8	high	8.3	6.7	1.92	31	12.5
PS	64	M	6	1	22.7	3.4	136	1.2	0.79	132.1	1.4	high	9	3.7	0		8.5
PS	31	M	5	1	18.6	3.6		1.16	1.01	128.2	1.4	high	10	6.8			11.1
PS	38	F	4	1	20.3	3.9	136	1.49	1.03	122.6	1.4	high	9.1	4.5	0.05	37	12.5
PS	63	M	4	1	21.2	3.8		0.94	0.84	89.73	1.4	high	8.9	4.9			10.7
PS	58	M	2	1	24.8	3.5	132	1.72	1.02	71.2	1.5	high	8.8	6	0.1	26	11
PS	38	M	28	1	18.2		116	1.31	1.24	86.62	1.8	high	7.6	4.1	0	27	9.9
PS	44	F	19	1	23.2	3.4	120	1.09	0.85	89.04	1.5	high	10.1	4.3	0.08	32	11
PS	72	F	19	1	17.5	4	126	1.62	1.02	140.5	1.5	high	9.5	3.6			12.5
PS	46	M	6	1	26.2	4	185	1.23	1.15	111.7	2.1	high		6.3		25	8.7
PS	48	M	6	1	28	3.5	114	0.99	0.9	129.7	1.5	high	9	3.9	0.27	23	10.3
PS	73	F	4	1	18.6	3.4	145	1.29	0.93	112.9	1.8	high		3.9	0.17	19	8.3
PS	73	M	2	1	23.9	3.4	130	1.41	0.9	100.6	1.5	high	9.2	5.8		26	10.5
PS	71	M	2	1	20.3	3.9	125	1.37	0.72	74.73	1.5	high	9	3.5	0.76	19	10.5
PS	66	M	2	1	22	3.4		1.29	0.62	60.93	1.1	high	8.2	6		28	9.8
PS	57	F	16	1	20.3	3.8	130	1.56	1.14	93.07	2.2	high	8.9	4.8	0.06	27	11.2
PS	69	M	8	1	20.8	3.8	144	1.98	0.71	101.8	1.5	high	7.8	4.7	0	30	12
PS	60	M	7	1	26.1	4.2	132	1.33	0.93	97.43	1.3	high	8.4	4.3	0.29	32	10.5
PS	63	M	22	1	23.3	3.6	97	1.22	0.86	109.8	1.8	high	8.7	5.4	0.45	22	11.7
PS	69	F	7	1	22.6	3.8	207	1.74	0.91	101.3	1.3	high	8.9	5.3	0.13	26	10.8
PS	61	M	7	1	26	3.9	138	1.25	0.96	130.4	2.1	high	8.9	5.7	0.08	33	8.6
PS	50	M	6	1	25.1	3.9	130	2.39	0.99	110.5	1.5	high	8.7	6.2	0.08	23	9.4
PS	39	M	4	1	26.7	4	111	1.47	0.96	149.9	2.1	high	8.1	5.5	0.02	32	10.1
PS	66	M	18	1	20.8	4	222	1.36	0.95	106.7	2.5	high	8.2	4.3	0.13		11.4
PS	56	M	17	1	23.4	4	147	1.04	0.99	101.7	1.3	high	10.4	7.1	0.12	14	11.6
PS	59	M	12	1	19.1	3.8	121	1.36	0.9	77.74	2.2	high	10.1	3.9	1.05	27	9.5
PS	48	F	8	1	24.2	3.8	161	1.86	1.21	99.66	1.8	high	6.7	5.1	0	28	11.1
PS	54	M	7	1	25.1	3.7	139	1.04	1.25	108.3	1.6	high		5.2	0.12	37	11.3
PS	77	F	7	1	22.6	3.8	172	1.24	1.01	134.9	1	high	8.3	4.7		36	10.9
PS	46	M	4	1	26.1	3.9		1.33	0.93	110.8	1.5	high	9	4.5	0	25	9.9
PS	55	F	22	1	14.7	4	163	1.55	0.86	88.25	2.2	high	9.1	4.2	0.31	25	13.3
PS	45	M	15	1	19.2	4.3	111	1.52	0.9	107.7	2.1	high	10.5	5.7		28	10.2
PS	58	M	9	1	21.4	3.9	134	1.94	0.9	97.14	1.6	high	9.3	4.2	0.5	36	9.7
PS	44	M	7	1	28.9	3.8	183	1.49	0.84	101	1.3	high	10.7	6.6	0.17	20	12.1
PS	55	F	3	1	17	3.9	204	1.2	0.97	97.99	0.8	high	9.4	5.5		22	8.6
PS	82	F	9	1	24.1	3.3	150	1.4	0.86	92.66	1.8	high	11.2	4.2	0.15		
PS	56	F	8	1	22.6	4.1	163	1.56	1	108.9	2.2	high	9.5	4.2	0.06	22	9.3
PS	64	M	4	1	24.1	4.2	99	1.33	0.9	111.9	2.2	high	9.4	3	0.1	28	12.2
PS	64	M	13	1	18	3.8	133	1.89	1.01	133.5	2.1	high	9.4	6.2	0.1	26	12.2
PS	82	F	5	1	21.1	3.9	138	1.69	1.08	121	2.1	high	7.5	5.2	0.33	27	9.9
PS	59	M	2	1	19.2	4.3	126	1.73	0.99	110.8	2.1	high	9.1	4.4	0.1	21	11.9
PS	49	M	21	1	22.5	4.3	181	1.67	0.97	115.6	1.6	high	8.9	3.1		34	8.7
PS	68	F	15	1	21.4	3.5	129	1.37	0.74	92.12	2.1	high	7.7	4.6		14	11.3
PS	68	M	9	1	21.4	4	121	0.79	0.85	113.9	2.2	high	9.4	3.7	0.28	35	11.4

PS	51	F	8	1	21.4	4.2	167	1.69	0.75	89.99	2.2	high	8.6	2.9	0.05	11	10
PS	68	M	4	1	24.4	3.8	144	2.5	0.83	123.6	2.1	high	8.7	3.7	0.1	31	11.1
PS	66	F	3	1	15.2	3.8	201	2.03	0.77	36.98	1.6	high	9.1	5.6	0.1	25	10.5
PS	80	F	5	1	19	3.8	106	1.31	0.87	168.4	1.8	high	8.6	5.6	0.28	31	9.8
PS	48	M	2	1	19.6	3.7	170	1.01	0.76	82.65	1.5	high	8.2	2.4	0.94	13	10.7
PS	71	M	30	1	19.5	3.4	141	1.25	0.95	94.71	2.2	high	8.1	5.2	0.08	28	9.8
PS	60	M	16	1	22.4	4.1	132	1.27	0.8	132	2.1	high	9.2	4.6	0.1	22	11
PS	35	M	4	1	17.9	4.3	169	1.47	0.87	84.62	2.1	high	8.6	4.3	0.1	28	9.1
PS	80	M	2	1	21.5	3.8	182	1.32	0.94	150.9	1.8	high	10.2	4.3		32	11.4
PS	75	M	17	1	19.8	4		1.62	0.98	110.6	1.6	high					
PS	47	F	28	1	16.9	3.9	178	1.78	0.92	107.6	1.8	high	9.6	6.2	1.5	39	9.5
PS	60	F	20	1	22.2	3.8	136	1.82	1.04	86.15	1.6	high	10	5.3			8.8
PS	42	M	12	1	25	4.2	122	1.26	0.71	86.45	1.8	high	8.5	5.4	0.1	30	12.2
PS	83	F	11	1	23.5	4	196	2.47	0.9	127.5	1.3	high	8.6	4.7	1.26	18	11.7
PS	76	F	9	1	23	3.8	212	1.39	0.64	113.9	1.8	high	9.5	5.7		22	11.5
PS	63	M	9	1	20.4	4	197	1.75	1.03	114.7	1.5	high	9.2	6.4	3.49	29	10.5
PS	47	F	6	1	21.3	4	154	1.59	0.99	129.4	2.5	high	9.1	5.8		30	10.6
PS	47	F	4	1	11.5	2.6	220	2.28	0.82	56.98	1.6	high	9.8	6.1	5.1	24	12.2
PS	64	F	2	1	27.1	4	150	1.44	0.93	78.49	1.8	high	10.4	7.2	0.7	33	9.9
PS	59	F	18	1	28.6	4.1	218	1.29	0.93	105.7	0.8	high	9.5	4.7		34	10.8
PS	64	M	16	1	19.2	3.7	169	1.84	1.04	124.3	1.3	high	8.4	5.3	0.05	31	11.3
PS	66	M	3	1	20.3	4	124	1.28	0.84	90.3	1.5	high	8	6.4	0.12	36	9.9
PS	43	F	18	1	18.1	3.8	115	1.54	1.04	125.2	1.5	high		4.6	0.17		10.7
PS	48	M	27	1	21.2	4.1	136	1.38	1.2	120	2.1	high	8.6	6.6	0.13	34	10.5
PS	55	M	15	1	16.7	3.5	102	1.52	0.92	113.9	1.8	high	9.3	2.8	1.77	32	10.1
PS	61	F	15	1	23.1	3.7	180	1.99	0.81	145.7	1.6	high	8.1	5.1	0.8	30	14
PS	52	M	15	1	24.3	2.9	202	1.51	0.95	93.48	2.2	high	8.8	6.9			10.8
PS	43	M	16	1	19.5	4.4	196	1.49	0.77	100.2	1.8	high	9.1	7.2	0.07	21	9
PS	60	F	9	1	19.4	3.8	117	1.34	0.97	111	1.8	high		6.8	0.1	40	12.9
PS	33	F	8	1	22.9	3.9	217	1.99	1.17	115.2	1.3	high	10	3.6		21	9.1
PS	58	F	2	1	26.9	3.9	105	0.55	1.06	127.3	1.8	high	9.7	3.1	0.13	29	8.2
PS	41	F	15	1	21.4	3.9	127	1.42	0.95	143.3	1.4	high	8.1	7.1	0.21	29	11
PS	56	M	19	1	24	3.7	116	1.27	0.94	132.4	2.1	high		6.1	0.1	28	10.6
PS	77	F	14	1	17.7	3.9	192	1.25	0.9	126.7	1.8	high		6.6	0.3	25	10
PS	60	M	8	1	20.9	3.7	176	2.6	1.23	130.1	1.6	high	8.9	5.9	0.1	20	11.2
PS	61	M	8	1	23.9	4	113	1.55	0.94	106.9	1.8	high	9	4.9			10.8
PS	41	M	3	1	19.9	4.3		1.58	1.02	110.4	1.3	high	9.8	4	3.01	34	10.2
PS	46	M	3	1	26	3.9	133	1.1	1.08	116	1.5	high	8.4	4.1	0.36	31	10.1
PS	48	M	16	1	17.7	4	115	1.38	1.1	93.93	2.1	high	9.7	4.2		21	7.7
PS	58	F	15	1	18.1	3.9	121	1.47	1.45	111.4	1.8	high	9.3	5.1	0	31	11.2
PS	55	M	10	1	20.1	4.3	149	1.11	0.97	88.52	1.5	high	7.6	4.5	0.13	29	9.8
PS	72	F	4	1	23.5	3.9		1.31	0.83	128.5	2.5	high	9.2	7.1		23	
PS	71	F	7	1	19.2	3.7	189	1.53	0.9	88.63	1.4	high	8.3	5.2	1.3	26	11.4
PS	63	F	22	1	21.6	4.2	174	1.34	1.2	104.4	1.8	high		5.2	0.1	36	11.3
PS	66	M	9	1	20.7	4.2	137	1.7	0.95	137.5	1.8	high	9.7	5.3	0	32	11
PS	63	M	8	1	25.7	3.8	121	1.6	0.86	131.2	1.4	high	8.8	4.1	0.05	39	8.8
PS	36	F	21	1	25.2	4	160	1.31	1.11	99.41	1.5	high	8.9	4.7	0.05	16	13.3
PS	59	M	16	1	21.5	3.9	146	1.7	0.65	111.8	1.8	high	9.9	9.9	0.2	40	10.2
PS	55	M	11	1	26.7	4.2	163	1.71	1.05	120.1	1.6	high	9.3	5.2	0.1		11.8
PS	65	F	9	1	20.2	4	201	1.23	1.01	111.2	2.2	high	9.4	5.3	0.18		11.4
PS	68	F	3	1	22.5	4.2	197	1.22	1.29	128.8	2.1	high	8.5	6.9		22	11.1
PS	52	M	3	1	20.3	3.6	126	1.18	0.54	72.27	1.8	high	8.6	5.4	0.18	34	11.3
PS	45	M	14	1	19.2	4.1	118	1.62	1.01	102.6	2.2	high	9.6	3.9			10.6
PS	71	M	11	1	20.8	3.7		1.27	1.14	115.5	1.4	high	8.8	7.1	0.09		10.3
PS	44	M	6	1	34.6	3.7		1.29	0.59	106.5	1.5	high	9.6	4.2	1.3	31	11.4
PS	61	M	17	1	19	3.6	137	1.72	0.96	97.84	1.5	high	8.7	5.6	0.05	23	8.8
PS	46	M	4	1	19.8	3.7	199	1.33	0.87	99.47	1.5	high	9.7	7.2	0.1	23	11.5
PS	60	M	3	1	16.9	4	208	1.17	0.89	75.29	1.5	high	9.6	6.9	0.05	18	11.6
PS	52	M	2	1	23.6	3.5	158	1.16	0.88	59.27	2.1	high	9.7	4.4	0.18	18	10.2

PS	66	M	2	1	22.5	3.9	180	1.2	0.79	58.72	1.6	high	8	5.7	0.8	25	12.5
PS	71	F	2	1	21.6	3.7	132	1.13	0.89	103.8	2.1	high	8.6	5.9		22	10
PS	43	F	2	1	17.5	3.7		1.18	0.81	88.72	2.1	high	10.3	6.9		25	8.7
PS	79	F	10	1	22.2	3.8	152	1.43	1.1	83.36	1.5	high	9.5	4.5	0.02	19	8.7
PS	82	F	3	1	24.5	3.2	167	2.29	0.65	103.7	1.6	high	8.8	4.3	0.17	29	10.3
PS	66	M	12	1	22.2	3.6	143	1.76	0.91	133.1	1.5	high	9.7	5	0.07	27	11.5
PS	74	F	3	1	22.3	3.7	193	1.73	0.9	122.5	1.3	high	9	6.9	0.1	25	10.6
PS	48	M	8	1	19.8	3.7	125	1.44	0.85	67.86	2.2	high		4.4	0.04	21	10.2
PS	45	M	5	1	22.2	3.9	178	1.61	1.44	127.8	1.4	high		5.7	0.09	31	9.7
PS	53	M	19	1	18.5	3.4	137	2.45	0.74	82.04	1.8	high	8.7	4.2	0.08	23	9.3
PS	56	M	5	1	25.4	3.8	160	1.83	1.04	102.4	1.6	high	8.8	6.6	0.1	26	9.4
PS	42	M	7	1	18.7	4.2		1.31	0.95	70.98	1.4	high	8.9	5.9	0.05		10.6
PS	76	M	5	1	18.3	3.9	108	2.43	0.85	123.4	2.1	high	8.1	5.3	0.2	24	11
PS	56	F	14	1	19.4	4	154	1.4	1.04	119.9	1.5	high	9	4.4	0.05	30	11.5
PS	54	M	7	1	25.4	3.9		1.36	0.98	140.6	1.4	high	9.5	5.8	0.03		12.4
PS	64	F	11	1	20.4	3.9	141	1.58	0.99	108.4	1.5	high		4.9	0.12	28	11.4
PS	44	M	13	1	23.6	3.7	88	1.4	0.87	90.91	1.5	high	6.9	4.7		43	
PS	57	F	16	1	17.5	3.9	153	1.56	1.15	126.9	1.5	high	9.5	3.6	0.04	30	10.8
PS	44	M	3	1	31.2	4	123	1.43	0.84	87.77	2.1	high	9	6.2	0.09	30	10.9
PS	59	M	3	1	23.6	4.2	218	1.65	0.95	106.7	1.8	high	9.8	6.8		22	10.6
PS	58	M	2	1	19.8	4.1	79	1.25	1.09	105	2.5	high	8.9	7.9	0	24	11.2
PS	44	F	5	1	20.1	3.7	244	1.58	0.69	111.2	1.6	high	7.9	4.8	0.1	31	11.1
PS	48	M	16	1	21.2	3.7	166	1.53	0.91	120.3	1.5	high	9.2	7.6	0.72	29	11.9
PS	71	M	5	1	23.5	3.7	218	2.06	0.86	138.5	1.5	high	9	4.2	0.08	26	10.8
PS	67	M	4	1	33.3	4	225	1.64	1	70.81	1.8	high	10	4.9		27	10.2
PS	73	M	7	1	24.3	3.7	169	1.4	0.74	128.1	1.3	high		4.8	0.2	26	8.2
PS	62	F	2	1	26.2	4.3	186	1.42	0.8	67.52	1.8	high	8.9	6.1		48	11.8
PS	70	M	12	1	16.9	3.2	156	1.23	0.83	90.56	1.3	high		3.9	4	30	11.4
PS	77	F	7	1	23.4	4	122	1.37	0.71	72.99	2.1	high	8.9	5.9	0.09	24	8.4
PS	67	F	7	1	20.9	4	109	1.34	1.09	110.2	1.5	high		5.1	0.21	34	10.5
PS	71	F	5	1	20.3	3.9	125	1.28	0.85	149.3	2.5	high	8.8	4.8	0.02	23	7.4
PS	76	F	5	1	20	3.6		2.18	0.69	118.1	1.4	high	8.8	5.4	0.03		9.8
PS	60	F	18	1	22	4	201	1.71	0.84	82.9	1.5	high	8.7	4.4	0.15	25	11.1
PS	67	M	11	1	20.8	3.8		2.2	1.08	147.9	1.4	high	10	5.2	0.05		11
PS	71	F	5	1	22.1	4.3	112	1.54	0.91	118.1	1.8	high	9.3	6.8	0.15	29	11.6
PS	76	M	3	1	18.6	3.7	132	1.69	0.79	87.64	2.2	high	9.7	5.3	0.08	26	11
PS	42	M	18	1	20.5	4.1	123	0.98	0.89	123.9	2.5	high	8.8	5.6	0.07	13	11.1
PS	65	F	9	1	17.3	4	155	1.34	1.06	129.8	1.4	high	8	5	0.1	29	11.9
PS	65	M	6	1	20.3	3.8	201	1.88	1.01	137.4	1.8	high		6.2	0.05	28	8.2
PS	39	M	2	1	33.4	4.2	171	1.45	0.96	119	1.8	high		5	0.12	28	10.1
PS	70	M	9	1	22.8	4.4	207	1.21	1.04	126.7	1.5	high	10.2	5.9	0.04	25	10.6
PS	49	M	21	1	20.2	4.3	130	1.38	1.06	136.4	2.1	high	8.9	8.1	0.26	27	8.8
PS	52	M	10	1	22.3	4	113	1.68	0.84	97.55	2.1	high	8.5	6.1	0.1	24	7.9
PS	40	M	8	1	21	4.3	136	1.11	0.87	136.2	1.8	high	8.6	6.4	0.3	20	10.9
PS	62	M	3	1	19.2	3.8		1.49	0.79	118.3	1.5	high	9.4	6.3			11.2
PS	48	M	19	1	17.3	4.1	160	1.9	0.94	112	1.8	high		6	0.1	29	9.6
PS	66	M	13	1	25	3.9	156	1.38	0.76	111.2	1.8	high		6.7	0.06	27	9.2
PS	63	M	12	1	24.5	3.5	133	1.15	0.81	90.43	1.5	high	7.4	4.3	0.1	28	10.6
PS	58	M	8	1	19.2	3.9	211	0.8	0.9	122.6	1.3	high	8.3	7.1	0.06	20	10.6
PS	56	F	6	1	19.5	4.2	173	1.1	1.34	123.6	1.8	high	8.5	4.3	0.08	30	11.1
PS	83	M	4	1	20.1	4	118	1.1	0.71	48.61	1.5	high	12	7.2	0	28	11.5
PS	83	M	7	1	18.7	3.8	128	1.27	1.1	93.27	1.3	high	8.8	10.3	0	25	11.6
PS	42	M	15	1	30	3.8	154	1.69	1	133.3	1.8	high	8.4	5.4	0.6	31	11.9
PS	70	F	3	1	19.2	3.6	180	1.37	0.73	94.32	1.8	high	9.2	5	0.09	26	9.4
PS	49	M	22	1	22.2	4.3	159	1.1	1	108.6	1.8	high	8.3	5	0.11	21	10.6
PS	66	M	3	1	22.4	3.8	143	1.07	0.99	111.8	1.3	high	8.5	4.4	1.9	36	9.3
PS	49	M	17	1	20.9	4.2	195	1.91	1	125.7	1.8	high		6	0.07	27	11
PS	53	M	13	1	28.9	4	97	1.43	0.76	147	1.8	high		5.3	0.05	22	10.4
PS	61	M	21	1	21.1	4	163	1.3	0.83	135.7	1.8	high		5.5	0.05	25	8.4

PS	71	M	10	1	23.9	3.6	169	1.46	1.15	131.6	1.8	high		6.3	0.05	19	11.1
PS	70	M	5	1	22.7	3.8	172	1.41	0.99	123.5	1.8	high		6.4	0.05	22	9.3
PS	60	F	12	1	16.5	4	199	1.45	0.9	130.7	1.8	high		6.6	0.59	24	11.2
PS	72	F	10	1	18.5	3.7	174	1.25	0.99	112.3	2.2	high		6	5.46	22	9.5
PS	41	F	9	1	20.6	4.1	116	1.56	0.86	122.3	1.8	high		5.4	0.05	26	10.9
PS	64	M	8	1	19.2	3.6	190	1.18	1.03	120.1	1.8	high		4.5	0.05	21	10.6
PS	42	M	3	1	19	3.8	143	1.89	0.84	104.4	1.8	high		6.3	0.05	29	10.2
PS	67	M	7	1	28.5	4.2	155	1.67	0.99	134.8	1.8	high	8.8	4.8	0.9	27	11.5
PS	57	M	21	1	17.8	3.7	185	1.59	1.16	116.5	1.6	high	9.6	5.1	0.08	27	11.5
PS	57	F	25	1	22.8	4	192	1.48	0.74	108.1	1.8	high		7.2	0.06	25	11.1
PS	44	M	24	1	22.2	3.8	108	1.46	0.95	107.6	1.8	high		8.3	0.25	22	10.7
PS	58	F	17	1	20	4.2	148	1.7	0.81	112.5	1.8	high		6.3	0.05	25	11.5
PS	68	M	2	1	18.4	3.8	197	1.4	0.98	119.1	2.2	high		8.8	0.1	27	10
PS	78	F	14	1	21.6	4	158	1.31	1	107.5	1.8	high		5.4	0.2	31	14.6
PS	55	M	2	1	21.7	3.9	163	1.37	0.81	72.77	1.8	high	8.9	6.2	0.05	28	10.3
PS	72	M	7	1	19.2	3.4	205	1.89	0.77	138	1.8	high		5.2	0.06	23	9.6
PS	81	M	2	1	21.3	4	155	2.2	0.62	98.25	1.5	high		8.2	0.13		9.5
PS	75	F	12	1	19.9	3.9	125	2.02	1.11	111.7	1.3	high	8.5	3.5		24	10.7
PS	50	M	8	1	26.6	4	132	1.53	0.89	142.9	1.8	high		6.4	0.05	39	10.9
PS	58	M	12	1	20	3.8	140	1.07	0.93	91.67	1.5	high	8.5	7.3	0	28	10.7
PS	68	M	7	1	24.4	3.5	152	1.14	0.81	132.7	1.5	high	9.4	5.1	0.09	33	9.7
PS	73	M	2	1	20.9	3.9	213	1.75	0.87	104.6	1.8	high		5.2	0.05	47	9.7
PS	53	M	7	1	21	3.9	163	1.34	1.16	128.9	2.2	high		4.1	0.18	33	10.4
PS	66	M	6	1	30.5	4.1	164	1.46	1.03	88.19	2.2	high		4.7	0.05	22	10.6
PS	79	M	5	1	21.3	4	166	1.14	1.03	90.93	2.1	high	9.2	8.8	0.38	29	11.3
PS	54	M	8	1	20.1	3.9	163	1.7	1.01	129.6	2.1	high	9.7	5.2	0.08	23	9.8
PS	64	M	2	1	25.7	3.9	176	1.32	0.94	110.5	1.8	high		5.4	0.11	29	10
PS	64	F	23	1	19.5	3.9	132	1.4	0.92	95.21	1.8	high		5.5	0.08	27	8.9
PS	67	F	4	1	25.4	4	153	1.66	0.84	112.2		high	8.1	4.6	0	28	8.7
PS	62	F	21	1	18.3	4	209	1.51	1.26	96.68	1.8	high		6	0.17	49	10.9
PS	67	F	20	1	16.6	4	160	1.58	1.03	127.8	1.8	high		7.7	0.68	21	10.9
PS	39	F	19	1	19.6	3.9	172	1.67	1.53	95.14	1.5	high	9.4	6.2		32	9.8
PS	73	F	18	1	18.9	3.8	110	1.15	0.75	108.5	2.2	high		5.5	0.14	26	11
PS	63	F	17	1	22.3	4.2	123	1.27	1.04	102.7	2.2	high		6.7	0.09	32	10.7
PS	51	F	14	1	17.6	4.3	117	1.46	1.02	107.6	1.8	high		5.4	0.05	25	10
PS	69	M	14	1	19.7	3.8	114	2.31	1.21	111.5	2.1	high	9.4	3.2	0.8	26	8.9
PS	65	M	13	1	19.7	4.3	77	1.38	1.02	90.35	1.5	high	8.5	5.5	0.05	30	10.1
PS	77	F	13	1	20	3.9	188	1.14	1.12	109.7	2.2	high		4.9	0.25	33	10.1
PS	40	M	12	1	19.7	4.6	193	1.14	0.93	119.5	2.2	high		7.8	1.25	29	10.7
PS	66	F	12	1	21.5	4.1	124	1.07	0.78	100.1	2.2	high		6.1	0.05	22	10.8
PS	70	M	12	1	21.1	4	289	1.88	1.1	124.5	1.8	high		6.1	0.05	25	10.1
PS	68	M	12	1	22.4	3.7	153	1.62	0.96	142.6	1.8	high		8.4	0.15	23	10.7
PS	71	M	12	1	18.3	3.7	126	1.5	0.65	29.18	2.1	high	8.2	7.1			8.6
PS	67	F	8	1	26.3	4	154	0.8	0.83	105.1	2.2	high		7.2	0.41	22	10.1
PS	79	M	6	1	23.6	3.5	174	1.12	1.22	110.4	2.2	high		6.8	0.05	17	10.1
PS	70	M	6	1	17.8	3.9	200	1.54	0.97	130.2	2.2	high	10.8	4.4	0.06	29	12.2
PS	70	M	5	1	20.6	3.8	118	1.3	1.07	119.9	2.2	high		4.9	0.31	33	10.4
PS	65	M	5	1	17.8	4.1	119	1.39	1.14	108.1	1.8	high	8.8	5		44	11.2
PS	68	M	4	1	22.7	4.1	145	1.53	0.8	101.1	1.5	high	9	4.9		29	12.2
PS	55	M	3	1	16	4.5		1.19	0.8	107.4	1.5	high	8.9	3.7	0.49	30	9.3
PS	57	M	29	1	18.8	4.2	99	1.62	0.97	91.37	2.1	high	7	9.9	0.04	33	8.7
PS	63	F	21	1	21	3.8	182	1.27	1.29	122.4	1.8	high	9.2	5.1	0.19	25	10.6
PS	75	M	20	1	21.6	4	103	1.51	1.15	113.6	1.5	high	8.5	5.6	0.17	28	10.1
PS	64	M	19	1	20.4	3.9	109	1.83	1.04	138.2	1.5	high	10.3	4.6	0.02	32	11.6
PS	59	F	17	1	16.8	4.2	137	1.28	0.79	115.6	1.8	high	8	7.4	0.01	22	9.9
PS	63	F	16	1	17.4	3.9	105	1.1	0.9	119.7	2.1	high	9.1	7.7	0.27	25	12.9
PS	71	F	15	1	20.8	3.9	190	1.3	0.92	109.8	1.8	high	9.4	4.5	0.6	25	10.8
PS	50	M	15	1	15.8	4.2	175	1.46	0.56	139.8	1.5	high	9.5	2.6	6.85	39	11.8
PS	45	M	11	1	20.2	3.5	123	1.62	0.89	97.85	2.2	high	8.2	5.4	0	24	8.7

PS	58	M	11	1	19.2	4.5	115	1.33	1.11	106.3	1.6	high	8.6	4	0.1	21	10.4
PS	58	M	11	1	23.7	4.3	101	1.12	0.84	101.6	2.1	high	9.5	5.8	1.57	30	9.2
PS	49	F	10	1	18.6	4.3	212	1.88	1.15	81.22	1.4	high	8.5	4	0.03	29	10
PS	61	F	10	1	23.3	4.1	126	1.37	1.03	91.88	1.6	high	8.4	5	0	25	10.2
PS	51	F	10	1	23.8	3.9	75	1.56	1.14	125.3	2.1	high	9.4	5.3	0.08	25	10.7
PS	53	M	7	1	22.1	4.3	118	1.43	0.8	110.2	1.6	high	8.7	4.3	0.7	31	10.5
PS	59	M	7	1	23.6	4	149	1.26	1.2	120.3	1.5	high	8	6.3	0.1		10.8
PS	66	F	7	1	21.5	4	181	1.34	0.64	101.8	1.5	high	9.5	6.1			9.8
PS	73	M	6	1	24.6	4.2	183	1.51	0.66	120.2	1.9	high	8.3	6.1	0.18	23	9.9
PS	57	M	6	1	24.6	3.7	151	1.76	0.81	101.5	1.8	high	8.3	4.7	0.03	25	10.4
PS	46	M	6	1	20.4	4.3	53	1.52	0.83	101.6		high		4.9			11
PS	59	M	5	1	23	4.1	224	2.04	1.14	116.9	2.1	high	8.7	3.7	0.1	24	14.2
PS	43	M	5	1	26.1	4.1	237	1.95	1.26	84.14	1.5	high	8.9	4.8		30	10
PS	58	M	5	1	24.1	3.7	141	1.63	0.43	106.4	1.5	high	8.6	3.2	0.16	22	8.4
PS	57	M	5	1	32.9	4.5	164	1.18	0.83	135.3	1.8	high	10.6	5.4	0.05	19	11.9
PS	66	M	4	1	24.1	4.1		1.31	0.83	95.11	2.1	high		4.8	0.1		9.8
PS	66	M	4	1	20.5	3.9	150	1.33	1.12	94.64	2.2	high		3.8	0.3	18	9.2
PS	59	M	4	1	24.8	3.8	127	1.01	1.02	125.3	1.6	high	9.7	5.9	0.1	27	9
PS	57	M	4	1	21.6	3.8	204	1.39	1.07	124	1.5	high	8.6	4.2	14.02	16	11.7
PS	64	F	3	1	22.2	4.3	144	1.31	0.93	107.4	2.1	high	9.2	6.6		32	14.1
PS	42	F	3	1	21.3	4.4	143	2.34	0.69	62.77	1.6	high	8.5	4.6	0.1	27	10.8
PS	71	M	3	1	21.8	4.5	47	1.34	0.88	79.06		high		4.8	0.04	32	8.9
PS	69	M	2	1	20.9	4	111	1.79	1.05	91.8	1.3	high	8.1	4.4	0.2	19	9.3
PS	54	F	2	1	19.7	4.5	160	1.84	1.2	71.05	1.6	high	9.6	6	0	23	10.8
PS	76	M	12	1	16	3.3	118	1.48	0.8	47.19	2.1	high	9.8	6		32	10.9
PS	65	F	5	1	20.3	3.7	154	1.33	0.92	79.33	1.5	high	8.4	6.4		25	9.7
PS	75	F	27	1	15.4	3.3	199	1.86	0.84	93.82	1.9	high		4.6	0.22	25	11.1
PS	64	M	8	1	23.9	4.4	187	1.86	0.94	93.53	1.6	high	9.9	6.2	0.1	24	10.8
PS	68	M	9	1	20	3.5	123	1.48	1.03	79.11	1.5	high	9.4	4.8	0.4	28	10.9
PS	67	M	2	1	24	3.6	200	1.49	1.06	149.3	2.2	high	9.7	5.2		28	10.9
PS	52	F	31	1	14.8	3.6	154	1.1	0.86	67.41	1.8	high	7.8	3.8		25	10.3
PS	53	M	23	1	20.1	3.6	173	1.66	0.95	117.1	1.8	high	9.6	4.5			
PS	45	F	23	1	32.7	3.6	107	1.44	0.94	98.86	2.1	high	9.5	3			10.9
PS	53	M	18	1	18.6	3.5	198	1.75	0.97	97.87	1.5	high	8.7	8.2	0.2	33	10.8
PS	51	M	18	1	22.4	4.1	144	1.67	1.06	105.8	2.1	high	8.6	6.5	2.82	24	9
PS	67	M	15	1	19.7	3.7	128	1.13	1.01	129.4	2.1	high	9.2	4.3	0.29	26	10.9
PS	70	F	12	1	27.9	3.6	162	1.22	0.76	122.5	2.2	high	10.9	7.1	0.1	27	11.2
PS	66	M	11	1	21.8	3.6	147	1.62	0.73	99.28	1.3	high		6.2		28	10.6
PS	57	M	10	1	22.6	4.1	87	1.08	0.97	120.7	1.8	high	9.5	5.4	1.37	30	12.1
PS	71	F	9	1	19.9	4	152	1.26	1.06	86.78	2.1	high	8.6	5.6	0.04	30	10.1
PS	81	F	7	1	16.7	3.7		1.64	1.04	117.7	2.2	high		4.9			
PS	61	M	6	1	23	3.8	134	1.31	0.99	127.2	1.8	high	8.1	4.4	0.11	22	11.7
PS	77	F	3	1	25.7	3.5		1.27	0.89	141	2.2	high		7			
PS	64	M	3	1	23.5	4	146	1.1	1.11	109.3	2.1	high	8.6	6.1	0.05		11.9
PS	61	M	2	1	27.7	3.7	155	1.59	0.97	88.22	1.8	high	8.5	4.6	0.2	26	7.6
PS	70	M	2	1	20	4.2	160	1.71	0.72	109.4	1.5	high		4.4	0.16	29	9.9
PS	65	M	2	1	17.8	3.6	166	1.39	0.9	68.5	1.5	high	10	5.3	0.64	36	10
PS	71	F	27	1	20.6	4		1.36	0.92	117.3	2.2	high		7.6			
PS	61	F	27	1	18.2	4.3		1.52	1.32	93.12	1.8	high		4.2			
PS	68	M	15	1	22.8	3.6		1.37	0.63	139.6	2.2	high		4.5			
PS	66	M	12	1	19	3.4		1.43	0.74	117	2.2	high		4.9			
PS	75	M	3	1	20.2	4.2	177	1.66	0.63	75.27	1.5	high	10.3	8.4	0	19	11.9
PS	52	M	10	1	19.3	3.4		1.55	1.02	103.1	2.2	high		5.6			
PS	68	M	8	1	22.3	3.6	127	1.71	1.18	154.3	1.8	high	8.8	6.1		30	8.6
PS	71	F	8	1	25	3.3	125	1.19	1.21	86.14	1.5	high	9.9	4.6	0.32	39	10.4
PS	54	F	8	1	20.6	3.8		1.16	0.75	119.6	1.8	high		5.6			
PS	72	F	6	1	20.8	3.4	123	1.12	0.63	112.8	1.3	high	8.3	3.5	2.15	9	7.8
PS	51	F	5	1	19.8	3.4	184	1.86	0.98	88.35	1.5	high	8.2	4.9	0.05	26	10.7
PS	67	F	3	1	19.7	4.1	111	1.19	1.1	119.9	1.5	high	8.8	9.1	0.44	26	10.2

PS	71	M	3	1	19.5	3.6	100	1.15	0.72	85.57	1.8	high	8.7	8.1	0.16	27	10.4
PS	76	F	3	1	23.8	3.5	173	1.07	1.04	99.66		high	8.8	7.7	2.04	25	10
PS	46	F	2	1	18.1	3.5		1.45	0.86	62.03	2.2	high		5.4			
PS	45	F	4	1	14.4	4.5	181	1.23	1.03	77.4	1.5	high	9.5	5.7	0.11	30	9.6
PS	62	M	4	1	24.5	3.6	133	1.37	0.69	83.62	1.1	high	8.5	5.2	0.4	32	10.9
PS	46	F	3	1	18.5	3.6	153	1.6	1.16	104.8	2.2	high	8.9	3.4	0.3	30	12.6
PS	65	M	3	1	24.3	3.9		1.19	0.74	85.13	1.8	high	8.7	4.2	0.17	25	11.4
PS	71	F	18	1	21.6	3.9	123	1.42	0.85	119.2	1.5	high	10.4	3.5	0.08	22	11
PS	82	F	3	1	18.9	3.5	137	0.6	0.76	116.9	1.3	high	8.8	6.5		22	12.6
PS	80	M	3	1	20.2	3.9	94	1.29	0.9	131.6	2.1	high	8.4	4.2	0.38	27	10
PS	58	M	7	1	20.1	4	239	1.39	0.93	117.3	1.4	high	9.8	5.1	1.12	32	12.2
PS	46	M	2	1	22.7	3.6	104	1.44	0.76	62.14	1.8	high	8.9	5.8	0.2	30	11.1
PS	59	M	2	1	25.3	3.3	161	1.57	0.53	112.2	1.8	high	10	6	0.08	26	11.1
PS	58	M	2	1	20.9	3.7	153	1.25	0.72	67.44	2.1	high	9.5	5.2	0.05	34	10.8
PS	73	M	13	1	19.1	3.2		1.7	0.85	105.4	2.2	high		6.2			
PS	41	M	8	1	23.1	3.8	162	1.22	1.1	111	1.5	high	8.3	5.4	0.3	28	10.1
PS	64	M	6	1	21.4	4	178	1.19	1.17	82.63	1.5	high	8.8	4.7	0.04	23	9.9
PS	73	M	3	1	31	3.5	197	1.54	0.69	106.5	1.8	high	9.3	5.8	0.71	28	8.4
PS	73	M	3	1	23.6	3.9	114	1.25	1.18	121.8	1.5	high	8.9	5.8	0.07	22	10.5
PS	62	F	2	1	31.6	3.8	117	1.42	0.91	60.98	1.8	high	8.8	8.5	0.2	25	9.5
PS	66	F	2	1	21.7	3.8		1.3	1.03	96.44	2.1	high	10.4	4.5		22	
PS	41	M	2	1	24.3	4.2	152	2.03	0.87	137.1	1.6	high	9.3	4.6	0.1	27	10.4
PS	78	M	9	1	17.9	3.7	142	1.09	0.8	131.8	2.1	high	9.3	6.7	0.05	26	12.7
PS	74	M	7	1	22.4	3.6	115	1.46	0.8	98.57	1.5	high	9.6	6.2	1.05	31	10
PS	62	F	6	1	33.8	3.6	114	1.46	1.09	113.9	1.1	high		2.8	0.85	30	11.1
PS	69	M	4	1	20.9	3.6	155	1.42	0.79	73.43	2.1	high	8.6	3.6	0.18	24	9.2
PS	62	M	16	1	19.3	3.8	155	1.58	0.92	121.4	1.1	high	8.2	5.2	0.02	35	10.8
PS	71	M	16	1	21.7	3.8	100	1.7	0.83	103.1	1.8	high	8.2	3.3	1.04	19	10.4
PS	68	F	15	1	21.1	3.8	110	1.44	1.16	97.35	1.5	high	8.2	3.3	5.61	27	11
PS	74	F	11	1	22.5	3.9	166	1.6	1.22	117.5	2.2	high		4.2	0.3	28	10.1
PS	74	F	9	1	25.1	3.4	208	1.66	0.57	112.5	1.5	high	10.3	5.1			11.3
PS	69	F	9	1	29.1	3.7	164	1.28	1.02	125.4	2.1	high	9.6	7.6	0.01		10.6
PS	55	M	8	1	20	3.7	112	1.1	0.85	105.5	2.1	high	10.6	6.3		29	12.1
PS	73	F	8	1	17.8		103	1.12	0.71	19.56	2.1	high	9.6	8.1		27	11
PS	76	M	8	1	23.8	3.4	108	2.08	0.88	117.4	1.5	high	9	3.9	1.5	33	9.6
PS	59	F	6	1	21.3	3.9		1.25	1.04	94.64	1.4	high	8.6	4.3		17	10.7
PS	73	M	6	1	21.3	4.1	99	1.16	0.87	81.36	1.8	high	9.2	4.5	0.1		10.2
PS	58	F	5	1	23.7	4.3	233	1.07	0.91	105.8	1.3	high	9	5.7	0		11.5
PS	81	M	5	1	21.6	4.1	124	1.8	0.83	78.13	1.4	high	8.6	5.8	0.64	16	11.3
PS	70	M	5	1	23.5	3.3	160	2.03	0.61	77.53	1.5	high	9.8	4.2	0.03	29	9.7
PS	67	M	4	1	23	3.8	158	1.33	0.78	162.2	1.8	high	8.8	6	0.03	21	9.2
PS	69	M	4	1	25.5	4	180	1.85	0.61	51.27	2.1	high	9.4	7.1		24	12.1
PS	91	M	2	1	26.4	3.7	107	1.17	0.54	69.42	1.5	high	9.7	3.4	0.03	29	9.7
PS	72	M	2	1	20.7	3.9	109	0.96	0.87	92.2	1.8	high	9.4	7.3		29	11.6
PS	79	M	17	1	26.1	3.4	146	1.22	0.69	100.6	1.8	high	8.2	4.9	0.07	25	9.3
PS	50	M	17	1	22.2	4.1	128	0.97	1.03	148.4	1.8	high	8.5	6.7	0.06	31	9.8
PS	46	M	17	1	21.8	4.1	177	1.25	1.19	125.1	1.3	high	10	6.6	0.03	29	10.6
PS	71	F	4	1	18.5	3.5	89	1.29	0.83	132.4	1.5	high	9	5.3	0	42	10.3
PS	71	F	3	1	26.1	3.6	151	1.83	1.19	125.7	2.1	high	9.1	2.5	0.02	19	11.6
PS	53	M	12	1	24.6	2.1	170	1.53	0.83	101.6	1.4	high	7.9	4.6	0.04	26	10
PS	64	F	19	1	19.2	3.8	205	1.56	0.94	153.9	1	high	9	5.5	0.09	21	12.7
PS	74	F	12	1	18.3	4		1.25	0.92	124	1.8	high		2.7			
PS	58	M	37	1	20.9	3	116	1.54	0.6	70.34	1.8	high	8.2	6.4	0.07	29	10.6
PS	80	M	11	1	21.6	3.7	111	1.64	0.58	94.24	2.5	high	8.8	5	1.63	23	10.4
PS	70	F	8	1	20	4	163	1.56	0.93	104	1.5	high	9.9	3.9		29	9.1
PS	77	M	7	1	19.1	3.7		1.68	0.92	88.1	1	high	10.1	2.4	0.37		9.6
PS	55	M	6	1	18.8	3.4		1.17	0.7	78.68	1.8	high		5.9			
PS	69	M	6	1	18.1	4.1		1.28	0.88	130.5	2.2	high		4.1			
PS	67	M	4	1	21	2.8	171	1.42	0.71	99.15	1.8	high	9.7	5.2		28	10.8

PS	67	F	4	1	22.1	3.8	120	1.36	1.01	99.66	1.8	high	8.7	4.9			11.8
PS	52	F	3	1	16.7	4.1	114	1.2	1.06	81.44	1.8	high	9.5	5.4			10
PS	73	M	3	1	20.4	3.6	117	1.62	0.89	130.3	1.4	high	9.7	6.3			11.4
PS	69	F	2	1	18.8	4.1	134	1.79	1.02	103.9	1.4	high	8.3	5.6	2.8	26	9
PS	72	M	2	1	21.8	3	220	1.4	0.81	94.92	1.4	high	11.2	4.9	0.1	29	9.2
PS	65	F	31	1	19.6	3.7	126	1.15	0.92	77.75	1.8	high	11.6	4.3	0.2	30	9.4
PS	80	F	30	1	18.9	3.8	169	1.44	0.83	87.91	1.8	high	8.7	8	0.05	22	12
PS	58	M	28	1	18	4.1	172	1.82	1.01	79.07	1.4	high	8.8	7.3	0.08	29	10.8
PS	76	M	25	1	20.3	3.9	117	1.36	1.01	90.71	1.8	high	9.5	4.4	0.01		10.7
PS	44	F	24	1	20.2	4.2	128	0.87	0.98	88.88	2.2	high	7.3	5.7	0.07	29	9.7
PS	46	F	23	1	21	4.3	173	1.67	0.8	85.04	1.4	high	11.2	8	0.15	26	10.3
PS	58	F	22	1	22.1	4	109	1.25	1.14	105.4	1.4	high	7.8	4.8	0.01	28	7
PS	74	F	19	1	16.2	3.7	126	1.32	1.04	106.2	1.4	high	8.7	7.1	0.16	20	11.9
PS	70	F	18	1	16.3	4.2		1.21	1.21	115.5	1.8	high	7.7	6.1	1.1		
PS	58	M	16	1	25.2	3.7		1.47	1.09	151.9	1.8	high	8.8	6.4	0.12		11.1
PS	39	F	16	1	16.9	4.2	139	1.3	0.9	95.57	0.8	high	8.9	4.1	2.83	30	10.8
PS	55	M	15	1	22.1	3.9	138	1.25	1.03	98.56	1.8	high	9.4	4.5	0.06	23	10.5
PS	39	M	6	1	24.7		196	1.34	1.11		1.8	high		4.1		17	10.5
PS	86	F	6	1	14	3.7	125	1.32	1	103.9	1.3	high	8.7	5.3	0.02	31	10
PS	56	M	6	1	23	4.2	174	1.85	0.93	112.4	1.8	high	9.2	6	0.33	32	9.1
PS	68	F	3	1	24.1	3.3	123	1.02	1.05	80.69	2.1	high	7.7	7.2		23	11
PS	62	M	5	1	20.9	3.9	118	1.17	0.74	120	1.5	high	8.2	4.7	0.13	27	11.6
PS	67	F	5	1	20.8	3.8	119	1.2	1.32	114.9	1.8	high	9	4.9	0.08	31	9.6
PS	44	M	3	1	31.7	3.8	154	1.49	0.95	114.9	1.8	high	8.3	6.1	0.65	27	13.5
PS	78	F	3	1	23.8	3.6	195	1.89	0.82	80.45	1.8	high	8.8	5.4	0.3	25	11.3
PS	80	M	3	1	21.9	3.6	167	1.76	0.73	113.7	1.5	high	9.9	5			10.4
PS	64	M	3	1	15.4	3.2	153	1.53	0.95	91.98	1.8	high	8	10.3	0.49	30	10.3
PS	58	F	20	1	18.8	3.8	136	1.64	0.83	87.26	2.2	high	8.6	5.1	0.1	29	11
PS	49	F	17	1	21	3.9	178	1.25	0.76	82.89	1.8	high	8.2	4.6	0	15	11.6
PS	36	M	10	1	20.6	4.1	145	1.75	1.54	127.2	1.8	high	7.8	6.4	0.2	38	7.7
PS	53	M	8	1	21.3	3.6	159	1.31	0.88	129.7	2.1	high	9.9	5.3	0.02	31	10.7
PS	72	M	8	1	16.5	3.8	199	1.47	1.01	104	1.8	high	8.3	7.7	0	24	10.4
PS	51	F	6	1	24.3	3.4	136	1.31	0.9	114.5	1.8	high	8.2	5.1	0	25	10.4
PS	57	M	4	1	21.2	3.6	172	2.17	0.85	64.37	2.1	high	9.4	5.2	0.3	33	13.6
PS	48	M	4	1	20.6	4.1	140	1.54	1.05	109	1.8	high	8	6.7	0.1	25	8.6
PS	81	M	3	1	22.1	3.3	200	1.51	0.9	79.84	1.8	high	8.8	6.9	0	23	10.1
PS	73	M	3	1	24.1	3.9	151	1.86	0.77	108.8	1.8	high	7.8	6.6	1.4	23	9.7
PS	40	M	3	1	23.7	3.5	120	1.47	0.8	103.5	2.1	high	7.7	6.6		27	10.9
PS	50	M	3	1	30.4	3.9	136	1.43	1.02	93.76	1.8	high		5.5	0.06	26	9.5
PS	59	M	2	1	18.5	4	114	1.18	1.2	73.12	2.1	high	10.2	6.8	0.2	32	9.2
PS	63	M	2	1	20.2	3.8	145	0.96	0.79	105.8	1.3	high		4.6		15	11.5
PS	71	M	2	1	18.3	3.8	157	1.57	0.86	117	2.2	high	7.7	5.7	0.8	31	12.6
PS	73	M	2	1	24.7	4	112	1.33	0.76	93.57	1.8	high	8.9	1.8	1.05	36	8.6
PS	65	M	5	1	19.1	3.4	147	1.62	1.01	110.7	2.2	high	7.4	4.2	0	31	10.4
PS	72	F	14	1	16.3	3.9	129	1.33	1.02	106.9	1.8	high	8.6	4.4	0	19	9.9
PS	39	M	21	1	23.7	3.9	103	1.94	0.94	81.33	2.1	high	9.1	4	0.02	26	10.1
PS	59	F	17	1	16.8	3.4	131	2.27	1.22	88.81	1.3	high	9	3.2		28	10.4
PS	35	M	9	1	21	4	194	1.47	0.88	145.6	2.1	high	10.1	5.8	0.14		12.6
PS	73	M	9	1	20	2.4	84	1.12	1.03	108.1	2.1	high	8.5	4.9	1.3		10.1
PS	48	F	8	1	16.3	3.8	137	1.25	0.88	78.54	1.6	high		3.6	0.2	31	11.1
PS	61	M	7	1	24.5	2.5	95	1.08	1.27	106.4	2.1	high	9.2	6.9		33	9.3
PS	78	F	7	1	20.4	3.6	115	1.22	1.18	99.09	1.6	high	9.3	7.6	0.07	28	11.1
PS	68	F	5	1	15.8	1.6	146	1.26	0.99	105.3	1.3	high	7.8	4.8	0.35	19	9.4
PS	74	M	5	1	14.5	3.7	170	1.47	0.82	50.45	1.8	high	7.9	7.9	0.1	28	10.1
PS	29	M	4	1	21.7	4.1	123	0.97	0.84	54.15	1.5	high		5.2	0.1	31	12.3
PS	73	M	4	1	29.4	3.7	163	2.05	0.97	134.5	1.8	high	8.7	4.3	0.3	30	11
PS	55	M	3	1	19.9	4.1	185	1.3	0.94	65.91	1.5	high		4.7	0.05		12
PS	79	F	3	1	23.5	1.5	87	1.42	1.16	93.51	1.8	high	9.2	6.9	0.08	33	10
PS	60	M	3	1	26.3	3.6	158	1.21	0.81	116.3	2.2	high	9	4.4	0	38	9.7

PS	55	M	3	1	30.3	2.7	151	1.8	1.06	78.32	2.1	high	8.9	4.5		30	9.6
PS	74	F	3	1	20.4	3.4	143	2.02	0.79	91.22	1.4	high	8.2	5.7	1.18	38	10.7
PS	59	M	3	1	20.7	3.3	227	2.1	1.25	106.1	1.5	high	9.9	4.1	0.01	25	10.3
PS	59	M	3	1	23.3	2.2	195	1.53	1.26	114.6	1.8	high	8.7	5.3	0	22	10.5
PS	69	F	3	1	26.2	3.5	158		0.37	142.9	1.1	high		4.1	0.03		10.1
PS	66	F	3	1	21.3		185	2.05	0.72	102.9	1.8	high	7.9	7.5	0.1	31	10.8
PS	70	F	2	1	22.3	2.4	113	1.22	1.07	54.61	1.8	high		8	0.3		9.8
PS	83	F	2	1	18.2	1.9	169	2.16	1.37	121.9	1.8	high	9	4	0.05	23	10.5
PS	33	M	2	1	25.6	2.7	150	1.31	0.83	41.11	2.1	high	10.6	5.9	0.14	32	11.1
PS	67	F	2	1	21.3	3.3	92	1.33	1.19	108	1.5	high	8.2	4.3	0.05		12.4
PS	81	F	2	1	29.9	4	189	2.01	0.81	44.11	1.1	high	5.9	5.3	0.32		9.5
PS	55	M	12	1	20.7	4.1	129	1.37	1	122.4	1.8	high	8.3	3		31	10
PS	60	M	16	1	16.1	3.9	143	1.95	0.75	77.82	1.6	high	8.7	4.2	0.02		10.3
PS	62	M	15	1	16.8	3.9	152	1.45	0.74	79.08	2.1	high	9.6	5.8	0.07	25	12.3
PS	58	F	23	1	19	3.9	130	1.17	1.22	90.77	1.5	high		6.4	0.5	20	11.1
PS	68	M	3	1	20.7	4.1	137	1.68	0.58	80.97	1.6	high	9.8	4.8	0.05	27	10.9
PS	35	F	6	1	19.4	4.4	129	1.47	1	124.3	1.5	high	9.9	2.4	0.01	45	10.3
PS	60	M	5	1	19.8	3.9	130	1.37	0.77	117.3	1.8	high	9.4	4.6		13	9.4
PS	76	F	4	1	20.8	2.9	85	1.11	0.97	119.3	1	high		3.2	0.11		8.3
PS	59	F	4	1	19.7	2.2	80	1.69	0.81	88.91	2.1	high	8.7	5.9	0.1	28	10.5
PS	45	M	7	1	24.8	3.9		1.83	0.84	123.7	2	high		4.2			9.9
PS	33	M	6	1	20.9	4.2	103	1.66	1.61	112.1	1.6	high	8.3	5.4	0.08	25	10.1
PS	44	F	7	1	17.7	4.4	122	1.56	1.28	101.5	2.2	high	9.4	6.8	0	29	10.6
PS	77	F	2	1	18.8	2.7	159	1.38	0.89	81.09	2.2	high	8.4	4.7	0.4	36	10.1
PS	56	F	16	1	18.6	4.2	174	1.21	0.99	125.9	1.8	high	8.9	5.2	0.05	32	9.4
PS	59	M	22	1	22.2	4	168	1.86	0.95	89.03	1.4	high	7.9	5.9	0.08	31	10.5
PS	43	M	13	1	21.3	4.2	145	1.47	0.95	98.6	1.5	high		7.1	0.1	18	9.9
PS	49	F	15	1	16.5	4.5	149	1.4	0.89	111.2	1.4	high	9.3	5.3	0.17	27	11.5
PS	59	M	4	1	26.6	4.3	264	1.49	1.04	109.2	1.6	high	9.8	3.7	4.79	23	10.2
PS	71	F	14	1	15.9	4	152	1.04	1.11	65.05	2.2	high	8.5	5.7	0	17	10
PS	71	F	16	1	18.3	4.1	322	1.3	1.28	111	1.6	high	9.7	4.9	0.41	29	11.7
PS	59	M	15	1	21.8	3.9	149	1.57	0.74	112	1.8	high	8.5	5.7	5.44	23	10.4
PS	84	M	6	1	17.1		124	1.64	1.08	66.82	2.2	high	8.2	5.6	0.4	35	10.3
PS	68	M	7	1	20.8	4.3	131	1.22	1.09	98.15	1.5	high	9.3	4.3	1.9	23	10.3
PS	70	M	11	1	19.5	4	137	1.43	1.17	121.6	1.3	high		7.6	0.3	20	11.9
PS	63	M	4	1	22.4	4.6	100	2.12	1.12	111.6	1.6	high	9.6	6.5	0.07	31	11.1
PS	32	M	6	1	21.4	3.9	152	1.17	0.84	130.8	2.1	high	8.5	6.5	0.24	30	9.2
PS	50	F	25	1	18.8	4.1	99	1.44	0.9	107	1.8	high	10	4.4		16	10.5
PS	67	M	17	1	20	3.9	90	1.16	1.03	125	2.5	high	9.5	6		31	10.7
PS	71	M	14	1	20.6	3.5	204	2.39	1.03	132	2.1	high	8.3	5.7	0.1	22	11.7
PS	67	M	7	1	19.8	4.2		1.72	0.79	127.5	2.1	high	8.7	3.7		15	
PS	59	M	14	1	25.7	4.3	88	1.2	0.76	146.9	1.5	high	8.3	4.5	2.1	28	10.1
PS	64	M	7	1	21.4	4.5		1.18	0.82	129	1	high		5.7	0.12	20	9.7
PS	69	M	8	1	24.3	3.9	180	1.52	0.69	94.65	1.5	high	9.3	5.8	0.05	30	13.2
PS	66	F	16	1	22.2	4.1	171	1.4	1.1	117.9	1.8	high	8.5	6	0.1	34	9.7
PS	44	M	22	1	21.5	4.2	195	1.74	1.01	76.86	1.6	high		6.1	0.05	24	10.6
PS	67	F	18	1	20.2	3.6	147	1.64	1.01	96.93	2.1	high	9	4.1	0.1	25	7.7
PS	69	M	16	1	23.7	3.4	201	1.85	0.69	120	1.8	high	7.8	6.8	0	33	12.1
PS	83	M	3	1	26.8	3.4	119	1.55	0.93	89.93	2.1	high	8.8	4.7	0.08	23	9.8
PS	70	F	2	1	21.2	3.8	99	1.23	0.88	101	1.5	high		5.8	1.57		9.6
PS	38	F	20	1	24.7	4	116	1.12	0.92	124.8	1.5	high	8.6	8	0.1	23	7.5
PS	71	F	18	1	18.4	3.6	168	1.4	0.85	102.7	2.5	high	8.4	5.2		25	8.9
PS	64	M	16	1	20.2	3.8	169	1.55	1.02	89.09	1.5	high		3.4	0.37	33	10.8
PS	61	F	13	1	21.9	4.2	121	1.09	0.88	133.7	1.8	high	8.6	5.6	0.2	27	9.6
PS	56	M	11	1	19.4	3.5	196	1.1	0.83	64.69	1.8	high	9.1	7.9	7.47	29	12.4
PS	20	M	8	1	16.6	4.1	160	1.29	0.7	66.94	2.1	high	9.1	4.3	0.05	31	10.9
PS	65	M	8	1	19.6	3.7	78	1.69	0.92	82.49	2	high	9.5	7	0.05	23	16.5
PS	68	M	6	1	26.1	3.9	137	1.29	0.69	111.3	2.1	high	9.3	4.8	0.18	26	9.9
PS	71	M	3	1	21.3	3.8	129	1.11	0.64	66.42	1.6	high	8	4.2	0.23	22	8.9

PS	47	M	3	1	27.6	3.8	140	1.73	0.8	73.56	1.8	high		6.1		35	6.9
PS	70	M	3	1	23.4	3.8	240	1.59	0.76	78.84	1.8	high	8	7.8	0.3	30	10.5
PS	77	M	2	1	22.7	3.9	210	1.66	0.72	96.02	1.5	high	9.5	3.5	0.66	27	9.5
PS	69	M	2	1	15.4	4	235	1.49	0.68	78.69	1.3	high	9.6	4	0.05	32	9.7
PS	50	F	2	1	23.5	3.2	196	1.66	0.89	90.39	1.3	high		4.4		27	8.5
PS	45	F	2	1	20.8	3.7	128	2.15	1.01	55.46	1.8	high	9.8	3.7	0.75	27	7.2
PS	57	F	4	1	20.6	3.8	140	1.09	0.96	102.3	1.8	high	8.3	5.5	0.21	16	11.5
PS	67	M	11	1	20.6	3.9	145	1.58	0.86	124.2	2.1	high	8.6	4.9	0.08	25	10.3
PS	50	M	5	1	22.4	4.3	205	1.64	0.78	35.11	2.1	high	9.7	5	0.1	22	13.4
PS	58	F	4	1	20	4.1	208	1.12	0.82	110.4	1.3	high	8.5	5.7	0.1	26	10.3
PS	70	M	6	1	23.4	3.6	122	1.79	0.95	123.5	2.1	high	8.6	5.5	0.1	25	12.9
PS	59	F	5	1	19.7	3.6	145	1.4	0.84	83.54	1.8	high	9.3	7.6	0.04	21	9.1
PS	45	F	6	1	21.3	4.2	176	1.66	0.81	93.51	2.1	high	8.8	5.2			9.8
PS	70	F	2	1	20.6	3.9	159	1.88	0.87	114.2	1.6	high		6.4	0.15	32	10.6
PS	31	M	3	1	22.9	4	178	1.73	0.99	87.49	1.8	high		6.1		33	9.1
PS	57	M	17	1	16.8	3.7	177	1.61	0.78	91.17	1.6	high	9.4	7	0	26	9
PS	49	M	3	1	17.6	3.9	132	1.53	1.24	100.6	1.5	high	10	5	0.02	25	10
PS	67	M	5	1	20.9	3.8	183	1.96	0.91	117.9	1.8	high	7.9	3.7	0	33	10.1
PS	45	M	8	1	24	3.4	188	1.5	1.06	90.36	1.8	high	9.3	6.1	0.8	26	11.9
PS	58	M	3	1	27.4	4.2	202	1.34	1.18	114.3	2.1	high	8.9	6.5	0.07	26	9.5
PS	77	M	2	1	21.3	3.2	230	2.07	0.61	103.2	1.8	high	8.4	5.2	0	30	11.7
PS	57	F	12	1	26.2	3.9	203	1.22	0.78	168.1	2.1	high	9.5	3.2			10.7
PS	60	F	10	1	19.8	4	179	1.81	1	113.9	1.3	high	9.4	4.5	0.05	27	10.9
PS	78	F	6	1	22.4	3.6	151	1.51	0.87	126.8	1.1	high	8.2	4.2		22	11
PS	42	M	5	1	20.5	4.1	126	0.78	0.93	118.9	1.3	high	8.6	4.4	1.2	21	11.6
PS	46	M	8	1	16.2	3.6	140	1.29	1.29	85.5	1.3	high	10.8	5.9	0.08	29	10.4
PS	80	M	2	1	22.7	3.7	117	1.35	0.45	15.61	1.8	high	8.4	3.7		20	11.2
PS	60	F	5	1	24.7	3.8	165	1.34	0.94	98.6	1.6	high	8.9	3.4	0.1	25	9.8
PS	62	M	14	1	17.3	3.8	186	1.29	0.99	100.7	1.5	high	9.7	4.6		30	10.9
PS	38	M	6	1	23	3.7	139	1.5	0.74	104.9	1.5	high		6.5	0.06	25	11
PS	72	M	3	1	23.6	4	129	1.29	1.05	105.4	1.3	high	8.6	6.2	0	30	10.9
PS	52	M	24	1	20.8	3.5	126	1.63	1	98.8	2.2	high	8.4	5.4	0	28	10.4
PS	78	M	16	1	21.8	3.6	168	1.48	0.84	112.7	2.2	high	9	7.1	0	24	9.5
PS	57	F	2	1	20.9	4.4	173	1.4	1.03	70.33	1.8	high		6.1		27	8.6
PS	66	M	3	1	26.9	4.3	224	1.47	0.91	46.34	1.8	high	8.7	6.6	0.1	31	10.4
PS	31	F	2	1	20.6	3.6	132	0.98	0.93	74.05	1.6	high		4	0.09	19	9.1
PS	57	M	3	1	22.7	4	126	1.53	0.76		2.1	high	9.2	6.1	0.18	38	10.7
PS	41	M	10	1	23	4.3	94	1.55	1.17	90.74	1.8	high	7.8	4.5	0.7	28	10.5
PS	43	F	9	1	17.7	4.3	156	2.05	1.03	140.8	2.2	high	9.3	6.1		29	11.2
PS	63	M	5	1	20.5	3.7	110	1.21	0.67	104.1	2.1	high	9.3	8.3			13
PS	71	M	24	1	23.5	3.8	199	1.64	0.93	115.2	1.5	high		6.7		43	11.4
PS	62	M	16	1	18.8	3.5	128	1.13	1.06	114	1.3	high		5.3		20	11.6
PS	75	M	14	1	28.6	3.5	171	1.51	1.15	135.1	1.8	high	9.7	4.2	0	19	11.1
PS	53	M	2	1	23.3	3.9	367	2.02	0.82	83.26	1.6	high	9.8	4.4	0.82	32	8.6
PS	66	M	26	1	19.2	3.9	165	1.06	0.68	112.8	2.1	high		7.3		30	10.9
PS	64	M	8	1	20.3	3.8	151	1.59	1.19	153.3	2.2	high	8.4	5.8		25	11.7
PS	62	M	5	1	21	3.9	157	1.29	0.98	99.93	1.8	high		4.7		23	10.1
PS	51	M	31	1	22.5	3.7	216	1.12	1.08	103	1.8	high		6		25	10.5
PS	62	M	9	1	22.4	3.4	156	1.59	1.05	96.72	1.5	high	7.6	5.1		28	7.8
PS	78	M	5	1	20.5	3.5	158	0.92	0.71	77.27	1.5	high		5.9		25	8.9
PS	44	M	24	1	17.8	3.8	147	1.59	0.86	86.91	1.5	high	9.6	6.1	0.25	27	12
PS	52	M	8	1	31	3.8	127	1.28	0.76	107.2	1.3	high	10.1	4.6	0.05	33	9
PS	48	M	7	1	28.6	3.5	144	1.77	0.86	120.2	1.8	high	9.1	4.5	0.2	18	9.6
PS	47	M	6	1	21.7	3.2	169	1.27	0.81	90.4	1.5	high		5.8	0.07		9
PS	63	F	18	1	21.4	3.8	122	2.08	1.2	142.5	2.2	high	10.1	4.3		20	10.3
PS	56	M	4	1	21.4	4.1	213	1.26	1.02	101.3	1.8	high	8.8	5.3	1.63	27	10.2
PS	66	F	30	1	18.9	3.3	122	1.37	1.16	114.1	1.3	high		7.3	0.81	41	10.3
PS	63	F	28	1	20.2	3.3	157	1.15	1.04	101.9	1.5	high	8.1	8.2	0.25	34	10.5
PS	40	M	2	1	24.9	4	179	1.51	0.76	127.7	1.5	high	8.5	4.9	0.09	20	10.9

PS	52	M	22	1	22.9	3.2	191	1.57	1.08	119.6	2.2	high	9.5	5.7		26	10.1
PS	62	M	6	1	32.7	3.9	161	1.92	0.87	117	1.4	high	5	7.9	0.03	31	10.5
PS	56	M	5	1	26.6	4.1	152	1.86	1.07	111.7	2.1	high	8.4	3.9	0.1	21	10.3
PS	39	M	17	1	21.2	4	182	1.8	1.26	142	1.3	high	10	4.3	0.05	47	11.7
PS	62	M	14	1	19.7	3.8	233	1.71	0.86	118.9	2.1	high	9.5	5.3		26	12
PS	55	F	3	1	20	4	227	1.05	1.19	117.9	2.1	high	8.2	6.8	0.24	25	9.6
PS	36	M	11	1	23.6	3.9	142	1.21	0.93	85.39	2.1	high	9.4	6.4	0.05		10.5
PS	63	M	10	1	27.8	3.9	135	1.2	0.87	140	1.5	high		5.7	0.11	31	10.3
PS	54	M	31	1	19.5	3.3	196	1.25	0.8	87.43	1.5	high	9.3	5.7	0.16	18	11.8
PS	60	M	27	1	20.5	3.5	159	1.35	1.02	111.8	1.3	high		4.9	3.42	32	13.5
PS	68	M	24	1	21.2	3.5	120	1.29	0.9	146.9	1.3	high	7.2	5.7	0.5	24	10.9
PS	59	M	14	1	26.3	3.7	183	1.52	0.97	154.7	1.3	high	9.4	6.2	0.24	24	11
PS	63	M	11	1	28.9	3.7	171	1.4	1.1	141.7	2.2	high		4.4	0.21	22	10.7
PS	53	M	8	1	25.1	4	130	1.55	0.88	80.27	1.3	high	10.5	3	1.39	33	11
PS	61	F	8	1	18.1	4	149	1.43	1.05	150.9	1.4	high	9.5	3.6	0.33	27	11
PS	57	M	32	1	22.3	4	111	1.1	1.12	94.09	1.3	high	9.8	5.7	0.05	31	12.2
PS	60	M	9	1	25.2	3.7	144	1.52	1.15	119.7	2.1	high	8.2	5.3	0.06	24	10.5
PS	55	M	9	1	24.2	3.9	107	1.4	0.89	116.1	1.5	high	8.5	3	0.17	13	12.5
PS	64	M	9	1	25.6	3.6	167	1.19	0.98	105	1.8	high	10.4	6.1	0.2	29	10.1
PS	64	M	3	1	25.3	3.8	178	1.22	0.75	112.1	1.5	high	8.4	5.4	0.16	19	12.5
PS	66	M	19	1	20.1	3.6	140	1.48	0.81	122.7	1.5	high	8.5	5.9	2.7	29	10.7
PS	60	F	19	1	16.3	4.1	142	0.88	0.98	121.2	1.5	high	8.4	3.7	0.1	27	9.6
PS	57	M	9	1	24.4	3.8	159	1.03	0.74	174.3	1.1	high	8.7	3.2		16	8.4
PS	61	M	4	1	18.5	3.6	151	2.23	1	94.66	1.4	high	8.5	7.3	0.05	25	10.5
PS	73	M	15	1	17.6	3.6	140	1.09	0.92	132	1.3	high	8	4.8	0.96	29	10.5
PS	52	M	15	1	22.2	3.6	85	1.46	0.94	120.7	1.3	high	8.6	5.9	0.15	28	11
PS	62	M	8	1	24.3	3.8		1.8	0.99	128.6	2.1	high					
PS	76	M	4	1	23.3	3.9	151	1.48	0.98	96.88	1.5	high	9	5.6	0.1	24	10.9
PS	44	F	31	1	19.4	3.5	195	1.26	1.02	116.7	1.5	high	9.6	6.9	0.23	21	9.5
PS	69	F	27	1	20.6	3.8	133	1.35	1.32	104.6	1.5	high	9.7	6.3	0.08	25	10.8
PS	60	F	8	1	24.1	3.9	132	1.2	1.17	127.2	2.1	high	8.5	7.4	0.2	30	13
PS	51	M	17	1	18.6	3.6	160	1.35	0.73	98.88	1.8	high	8.3	9.2	0.4		8
PS	62	M	13	1	18.9	3.5		1.39	0.95	117.1	1.5	high	10.2	4.9		31	9.5
PS	55	M	35	1	20.3	3.7		1.45	1.04	94.25	1.5	high	9.2	6		22	8.4
PS	60	F	31	1	16.1	3.8		1.57	0.94	98.58	1.5	high	9.1	3.7		17	10.2
PS	32	M	6	1	19.8	4.3	212	1.27	0.96	104.3	1.3	high	8.2	3.5	0.39	26	10
PS	78	M	18	1	23.9	3.1	193	1.38	0.82	142.8	1.3	high		5.6	0.37	31	7.9
PS	61	M	19	1	20.5	3.8	162	1.48	0.83	111.4	1.5	high	8.8	5.6	4.44	25	13.3
PS	76	F	10	1	25.6	3.6		1.21	0.74	78.63	1.8	high	9.5	5		29	9.8
PS	66	M	5	1	22.4	4	179	2.21	1.17	118.6	1.8	high		5.7	0.2	14	10
PS	65	F	4	1	25.1	3.8	208	1.53	0.87	144.1	1.4	high	8.3	3.1	0.05	30	10.8
PS	75	M	2	1	23.5	3.9	176	1.52	0.65	84.97	2.1	high		5.7	0.05	25	9.8
PS	79	M	4	1	19.5	4	106	1.32	1.1	131.2	1.6	high	7.6	5	0.14	24	11.1
PS	51	M	18	1	19.4	3.8		0.72	1.07	105	1.5	high	9.5	4		8	9.5
PS	74	M	2	1	20.3	3.8	77	1.7	0.99	88.17	1.8	high		5.5	0.05	23	12.6
PS	50	F	27	1	25.1	3.8	125	1.61	0.65	87.67	1.6	high		4.1	3.1	43	10.3
PS	38	M	12	1	19.6	4.3	197	1.69	0.78	86.44	1.5	high	10.8	5.7	0.08	21	10.3
PS	59	F	6	1	14.2	3.4	147	1.37	0.91	70.93	2.2	high	8.3	6.9	0.12	27	11
PS	62	M	27	1	17.8	3.6	180	1.66	1.14	105.7	1.6	high		4.9	0.49	33	9.8
PS	76	M	11	1	21.7	3.9	152	1.87	0.85	123.9	1.5	high	8.5	5.8			8.4
PS	60	F	3	1	18.6	3.9	183	1.54	0.76	72.91	2.5	high	9.3	8.2			11.1
PS	52	M	2	1	18.9	3.8	159	1.91	0.93	60.51	1.5	high	8.9	3.6		23	8.6
PS	51	F	22	1	22.1	3.8	179	0.72	1.29	150.9	1.4	high	10.9	7	0.05	21	11
PS	52	F	22	1	19.6	3.8	154	1.38	0.73	83.5	1.8	high		6.5	0.54	27	10.8
PS	50	F	13	1	16.4	4.1	161	1.6	0.88	73.67	1.5	high	8.7	4.2		25	11.5
PS	62	F	10	1	18.7	3.9	144	1.61	0.95	113.8	1.6	high	8.7	4.5	0	26	11
PS	55	F	5	1	17.7	3.6	179	1.61	0.85	98.98	1.8	high	8.1	4.3	0.2	26	9.2
PS	48	M	16	1	15.8	3	110	1.29	0.59	26.56	1.6	high	8.9	4.3		23	9.2
PS	56	F	14	1	22.4	3.6	161	1.86	0.93	114.8	2.1	high	9	8.3			11

PS	58	M	29	1	19.7	4.1	124	0.88	0.82	77.21	1.6	high		9	0.69	39	8.4
PS	50	M	13	1	23.2	3.8	99	0.98	0.93	124.2	1.8	high	7.9	5.3	0.02	24	11.4
PS	84	F	6	1	20.9	3.6	180	2.29	0.58	77.5	1.8	high		6	0.05	21	12.6
PS	43	M	7	1	24.7	4.5	189	1.93	1.15	103.4	1.5	high	9.7	6.2			10.3
PS	48	F	18	1	18.3	4	245	1.94	0.95	123.5	1.5	high	9.2	6.8			9.1
PS	74	F	34	1	17.4	3.3	153	1.54	1.07	104.4	1.4	high	7.8	3.9			8.6
PS	64	M	31	1	26	3.2	143	1.61	0.84	91.86	1.8	high	8.5	4.7	0.05	28	11.2
PS	62	M	17	1	20.3	3.9	192	1.37	1.42	97.99	1.5	high	9.9	5.8			10.9
PS	83	M	5	1	21.4	3.7	218	1.79	0.81	93.03	1.8	high	9.1	5.5	0.2	20	9.4
PS	77	F	5	1	20.2	4	121	1.01	0.91	126.5	1.8	high	9.5	5.2	0.05	32	10.4
PS	71	F	10	1	23.1	3.9	101	1.45	0.97	121.9	1.4	high	9.6	2.9	0.04	33	9.1
PS	81	F	3	1	18.4	3.5	113	2.29	0.66	55.06	1.8	high		4.1	0.05	23	11.4
PS	60	M	3	1	21.8	4	109	0.94	1.03	118.7	2.1	high	9.5	4.5	1.59	17	9.9
PS	72	M	17	1	22	3.7	189	0.99	0.93	96.64	1.8	high	9.1	5	0.07	20	11
PS	83	F	2	1	23.1	3.8		1.81	1.01	89.81	1.8	high	10.1	6.9		27	
PS	61	F	7	1	18.4	3.5	146	1.47	0.82	80.22	1.8	high	8.8	6.4	0.2	27	9.2
PS	63	M	29	1	22.1	3.8	148	1.38	0.96	92.55	1.9	high		4.6	1.32	31	10.6
PS	44	M	8	1	20.1	3.8	140	1.48	1.24	130.6	2.1	high	8.4	5.8			11.8
PS	63	M	6	1	19.3	3.5	155	1.1	0.81	83.2	2.1	high	9.8	6.1	1.4	24	11.2
PS	75	F	6	1	28.4	3.4	154	1.61	0.66	114.1	1.8	high	9.8	5.3	0.2	29	8.9
PS	66	M	5	1	23.7	4.4	197	1.83	0.92	137.5	1.5	high	8.5	6.9			7.1
PS	61	M	13	1	21.1	3.4	115	1.23	0.84	104.9	1.8	high	10.4	5.4	0.01	32	9.3
PS	62	F	34	1	20	3.8		1.34	1.03	89.57	1.5	high	8.4	4.5			11.3
PS	69	F	13	1	17.4	3.7	109	1.49	1.4	95.71	2.2	high	9.8	3.3		28	9.7
PS	62	M	12	1	17.1	3.3	83	1.54	0.95	103.3	1.5	high	8.4	7.6		41	10.4
PS	67	M	10	1	21.5	4	154	1.59	1.12	116	2.2	high	8.6	4		24	10.8
PS	73	M	7	1	20.4	3.3	163	0.86	0.96	91.37	1.8	high	11	5.3	0.04	18	11
PS	55	M	6	1	23.6	4	145	1.28	1.07	109.9	1.5	high	8.1	2.7	0.49	26	10.1
PS	86	F	5	1	22.5	3.8	170	2.32	0.77	104.2	1.5	high	8.6	5.4	0.33	27	12.6
PS	81	F	5	1	16.4	3.2	239	1.76	1.27	54.3	1.8	high	10.6	6.2			12.3
PS	71	M	3	1	21.2	3.9	207	1.65	0.92	89.17	2.2	high	9.3	3.7	4.3	30	11.3
PS	70	M	2	1	18.1	3.9	168	1.18	0.78	58.19	1.5	high	8	5.6	0.05	34	8.9
PS	66	F	2	1	26.1	3.7	172	1.5	0.97	99.73	2.2	high	8.9	5.3			11.1
PS	80	F	2	1	26.4	3.7	124	1.89	0.94	96.18	2.2	high	9.7	6.2			11.6
PS	59	M	2	1	23.9	3.9	150	1.15	0.74	100.2	2.2	high	8.8	5.2			10.3
PS	42	M	17	1	23.6	3.9	194	1.32	1.14	108.4	1.5	high	8.3	4.5	0.06		10.3
PS	63	F	15	1	18.2	3.7	241	1.75	1.17	116.5	1.6	high	9.5	6	0	29	12.5
PS	60	M	6	1	21.1	3.5	135	1.04	0.97	94.74	1.3	high	8.8	5.6	0.1	18	10.1
PS	56	M	5	1	23.5	4.5	105	1.28	0.88	95.78	2.2	high	10.2	4.7			10.8
PS	79	M	4	1	18.9	4	199	1.07	1.09	109.7	2.1	high	9.6	4.8	4.37	37	10.6
PS	49	F	4	1	29.2	3.7	129	1.29	1.25	92.52	2.1	high	9.7	8.8	0	30	11.2
PS	58	M	4	1	24.9	4	184	1.83	0.88	123.2	1.8	high		6.6	0.11	30	11.5
PS	72	M	2	1	21.5	4.1	116	1.51	1.01	98.49	1.8	high	8.6	5.6	0.3	35	10.5
PS	51	M	28	1	19.5	3.4	120	1.61	0.98	94.3	2.1	high	8.5	6.1	0.1	26	10.7
PS	58	M	16	1	18.1	3.4	147	1.29	1.04	113.4	2.2	high	8.8	6.4			11.1
PS	44	F	17	1	14.7	3.5	139	2.11	1.07	63.72	2.1	high	8.7	6.7	0.8	30	10.9
PS	77	M	12	1	17.1	3.4	158	2.88	0.65	87.6	1.8	high		7.5	0.05	23	10.5
PS	59	F	8	1	23.5	3.4	193	1.59	0.72	119	1.3	high	8.6	4.1	0.4	28	12.4
PS	40	F	18	1	20.1	3.9	121	2.43	1.06	119.7	2.1	high	9.6	3.6	0.1	29	13.3
PS	55	M	9	1	18.5	3.5	171	1.48	0.85	128.9	1.5	high	9.3	3.1		31	11.2
PS	69	M	6	1	24.7	3.3	130	1.35	0.6	96.98	1.5	high	9.4	6.1		25	9.8
PS	74	F	2	1	26.1	3	155	1.22	0.92	112.8	1.8	high	8	4.5	0.1	31	11.3
PS	54	F	2	1	21.6	3.7	155	1.28	0.88	88.9	1.5	high	9.9	3		27	10.8
PS	46	M	18	1	20.8	3.8	142	1.4	0.91	110.5	2.2	high	8.7	4.5			10.9
PS	54	F	18	1	20.9	3.7	124	1.63	1.13	103.8	1.5	high	9	6.1	0.55	45	12.4
PS	66	F	7	1	21.6	3.8	119	1.89	0.92	120.5	1.6	high	8.9	3.9	0.1	18	11.6
PS	59	F	6	1	30.5	3.3	157	1.61	0.68	75.08	1.3	high	9.8	3.5		23	10.4
PS	58	F	15	1	23.4	3.9	215	1.51	0.95	112.8	2.2	high	8.7	4.3		22	10.3
PS	54	M	10	1	20.5	4.2	141	1.3	0.83	165.2	2.1	high	8.8	5.8	0.08	31	11.4

PS	70	M	2	1	19.4	3.5	119	1.7	0.74	43.49	1.8	high		5.9	0.05	27	12
PS	68	M	12	1	22.7	3.2	149	1.1	0.83	120.3	1.8	high	8.5	5.8	0.35	26	9.9
PS	66	M	7	1	19.4	3.6	169	1.65	0.66	115.7	1.8	high	8.4	1.9	0.7	35	10.6
PS	71	M	9	1	23.6	3.3	166	1.22	0.74	123		high		6.9	0.16	40	9.8
PS	73	M	7	1	23	3.5	140	1.09	0.75	139.6	1.6	high	9.6	6.4	0.1	28	11.1
PS	41	M	17	1	20.6	3.8	182	1.51	0.74	127.6	2.2	high	9.2	8.9		33	10.5
PS	70	M	7	1	22.9	3.5	111	1.64	0.74	111.9	2.1	high		5.8	0.05	23	11
PS	61	F	17	1	17.5	3.6	172	1.65	0.91	124.9	2.2	high	10	5.2			10.1
PS	56	F	9	1	23.8	3.5	165	1.29	0.88	111	1.3	high	9	3			11.6
PS	76	M	2	1	16.9	3.6	165	1.31	1.06	68.26	1.8	high	5.9	4.3	1.35		9.5
PS	52	M	14	1	19.4	3.7	161	1.69	1.14	120.1	1.5	high	9.5	6.8	0.05	32	12.4
PS	55	F	24	1	22.7	3.2	127	1.29	0.8	85.16	1.6	high		3.8	10.41	36	9.6
PS	74	M	6	1	21.3	4.1	171	1.53	0.75	99.9	2.1	high	9.5	6.1	0.12	23	12.5
PS	52	F	29	1	19.6	3.6	184	1.36	0.87	98.29	1.6	high		4.5	0.3	31	11.7
PS	29	F	8	1	22	4	167	2.53	1.08	133.3	1.5	high	9.5	5.8	0.1	24	11.2
PS	57	F	9	1	19.1	3.4	137	1.45	0.77	54.28	1.5	high	8.9	4.4		30	12.1
PS	65	F	7	1	20.4	4.3	179	1.5	1.08	140.6	1.6	high	9.1	4.3	0	21	12.9
PS	66	F	11	1	26.7	3.5	173	1.38	0.7	84.39	2.1	high		3.5			8.8
PS	71	F	10	1	16.6	3.2	130	1.34	1.17	98.6	1.5	high	8.9	4.9	0.12		8.2
PS	67	M	6	1	27.8	3.9	150	1.38	1.03	118.9	2.1	high		4.8	0.81	32	11.3
PS	69	F	5	1	20.3	4.1	133	1.27	0.89	122.1	2.2	high	9.7	5.3		21	11.6
PS	72	M	4	1	18.4	3.7	125	1.34	0.99	95.38	1.8	high	9.1	4.7	0.02	53	10.9
PS	50	M	7	1	24	3.5	133	2.41	0.72	84.64	1.8	high		5.5	0.05	19	9.8
PS	61	F	35	1	18.7	3.5		1.71	0.83	97.71	2.2	high	9.2	3.2	6.98	30	9.6
PS	58	M	32	1	19.1	3.6	168	0.86	0.92	101.8	1	high		6.2	0.2	17	9.5
PS	67	F	32	1	22	3.7	113	1.23	0.78	102.1	1.8	high	10.2	5	7.34	37	8.2
PS	58	F	30	1	17.4	3.9	148	1.38	0.95	137.9	1.6	high	8.5	5.5	0.09		9.1
PS	49	M	31	1	17.6	3.7	182	1.49	0.92	89.8	1.4	high	9	4.7	0.08	15	10.1
PS	59	F	29	1	17.1	3.9	164	1.39	1.23	123.1	2.2	high	10.2	3.6			14.2
PS	67	F	28	1	18.9	3.2	185	1.34	0.76	77.77	1.9	high		3.5	0.05	30	11.3
PS	58	F	28	1	17.7	3.6	143	1.52	0.74	95.07	1.5	high	8.9	5.6	0	26	9.3
PS	57	M	28	1	19.4	3.6	181	0.9	0.8	113.7	2.1	high	8.4	6.3	0.15		10.2
PS	61	M	28	1	21	3.2	150	1.51	1.02	93.31	2.1	high	9.8	6.2	0.07	25	10.8
PS	66	F	28	1	18.7	3.9	74	1.34	0.97	116.7	1.8	high	8	4.2	0.09	41	7.5
PS	54	M	25	1	21.9	4	206	1.79	0.88	131.6	1.6	high		5	0.1	32	13.1
PS	58	M	25	1	20.8	3.7	112	1.42	0.96	143.8	1.8	high	8.5	4.9	0.2	26	11.3
PS	60	M	24	1	17.2	3.6	155	1.35	1.11	100.3	1.6	high		4.4	0.31	23	9.8
PS	67	F	21	1	19	4	174	1.21	0.86	114.4	1.8	high	7.9	4.5	0.05	22	10.9
PS	59	M	21	1	21.4	4	223	1.63	1.04	118.7	1	high	9.3	6	0.05	26	10.2
PS	52	M	20	1	18.5	3.8	160	1.37	0.89	113.3	1	high	9	6.1	0.1	20	12
PS	57	F	18	1	17.2	3.5	168	1.07	1.13	76.51	1.1	high		4.8	0.6	18	11.4
PS	52	M	18	1	20.1	3.8	137	1.43	1.07	73.02	2.1	high	9.2	4.1		33	10.1
PS	43	M	18	1	19.3	4	167	1.12	0.81	111.2		high	8.8	6.8	0	26	9.8
PS	50	M	18	1	20.7	4.2	133	1.34	0.91	130.6	2.5	high	9	6.2	0.5	21	12.2
PS	70	M	16	1	21.3	3.6		1.84	0.68	107.7	1.3	high	10.5	5.5		17	
PS	66	M	15	1	20.8	3.6	166	1.46	0.83	90.49	1.1	high	9.1	3.5		30	10.4
PS	54	F	15	1	20.7	3.7	149	1.48	1.22	102.3	2.1	high	8	6.8	0.09	26	8.9
PS	55	F	14	1	15.6	3.8	132	1.3	1.02	100.4	1.8	high	8.3	4.1	1.2	32	10.2
PS	50	F	14	1	18.7	3.9	209	1.31	1.15	140.1	1.6	high	8.6	3.5	0.05	23	10.4
PS	70	F	14	1	23.2	3.5	169	1.68	0.84	121.5	1.8	high	9.8	6.1	0.05	21	9.8
PS	65	M	13	1	18.4	3.6	123	1.58	0.92	117.5	2.1	high	9.1	4.6		31	11.1
PS	71	F	13	1	17.7	3.9	176	1.89	0.81	121	1.5	high	8.5	5	0.01	27	11.1
PS	61	M	12	1	24	3.9	142	1.94	0.65	95.95	1.6	high	9.7	5.9	0	29	10.8
PS	53	M	12	1	19.7	4.2	145	1.49	0.94	118.3	2.2	high	10.3	4.1		28	10.8
PS	69	M	11	1	20.8	3.9	111	1.37	0.87	110.5	1.8	high	10.3	4.6		19	8.1
PS	58	M	11	1	25.4	3.9		0.85	0.93	141.7	1.3	high		3.2	0.08		11.3
PS	76	F	11	1	23	3.9	125	1.88	0.91	128.1	2.1	high		5.6	0.05	31	10.9
PS	69	M	10	1	22.2	3.6	164	1.17	0.74	127	2.2	high	10.4	5.9	0.09		10.5
PS	59	M	10	1	22	3.7	157	0.95	0.96	116.3	2.2	high	8.6	5.6	0	23	11.2

PS	40	M	9	1	18.6	3.9	152	1.8	1.13	125.2	1.1	high	9.2	3.5		31	9.3
PS	58	F	9	1	22.5	3.8	186	1.94	1.01	112.3	1.8	high		5.5	0.02	20	10.6
PS	62	F	9	1	16.8	4	149	1.27	0.76	85.29	1.8	high		6.9			8.3
PS	70	M	9	1	24.3	3.8	165	1.31	0.7	131.3	1.8	high	8.8	4.4	0.02	29	10.3
PS	70	M	8	1	17	3.9	174	1.45	1.21	112.9	2.1	high	8.6	3.5			
PS	77	F	7	1	24.2	3.6	168	1.14	1.03	97.98	1.8	high	8.8	4.7	0.16	19	10.7
PS	73	F	7	1	18.8	3.5	114	0.85	0.83	102.7	1.4	high		4.9	0.09	26	10.4
PS	69	M	7	1	26.4	4	190	1.51	1.12	110.8	1.5	high	9.1	4.4	0.2	31	9.2
PS	46	M	7	1	24.2	3.8		1.61	0.86	114.4	1.5	high	9	4	0.05	24	10.9
PS	50	F	7	1	23	3.9	172	1.78	1.04	115.2	1.8	high	8	7.3	0.1	31	9.9
PS	58	M	6	1	19	3.8	146	1.61	0.86	114.3	2.1	high	9.4	4.5	0.05	28	12
PS	48	F	6	1	29.6	3.7	153	1.55	0.88	99.78	2.1	high	8.3	5.6	0.22	27	10.6
PS	57	F	6	1	24.4	4.1	127	1.33	1.03	88.95	1.5	high	7.8	6.1	0.05	26	9.8
PS	63	M	6	1	23.1	3.4	155	1.38	0.68	100.9	2.1	high	8.2	5.1	0.44	24	10.9
PS	74	M	5	1	27.2	3.5	128	1.17	0.87	62.23	1.3	high		5.7	0.06	31	10.8
PS	65	M	5	1	15.5	4		1.33	0.87	81.32	2.1	high	9.5	2.7	0.03	47	10.3
PS	56	F	5	1	22.5	3.9	124	1.61	0.9	123.6	2.1	high	8.6	5.1	0.03	34	11.6
PS	72	M	5	1	18.5	3.7	111	1.49	0.83	96.05	1.4	high	8.8	4	0.31	35	11
PS	72	M	5	1	22.1	3.8	154	1.23	0.86	106.9	1.8	high	9.8	5	0.54	24	11.2
PS	64	F	5	1	19.2	3.8		0.99	0.85	101.4	1.5	high		7.8			9.9
PS	80	M	5	1	22	3.5	138	1.46	0.77	106.5	1.6	high	8.4	4.1	0.16	34	10.2
PS	44	M	5	1	21.8	3.5	153	1.27	0.76	114.5	2.1	high	8.7	4.1	0.05	19	10.2
PS	59	M	4	1	22.3	3.9		1.33	0.66	130.1	2.1	high		12.1	0.1	37	11.9
PS	70	F	4	1	20.8	3.9	153	1.34	1.04	112.1	2.1	high		4.1	0.5	19	8.2
PS	62	M	3	1	22.9	3.6	193	1.65	0.97	133.9	1.6	high	9.4	4.4	0.1	27	10.8
PS	86	M	3	1	23.8	3.4	157	1.96	0.88	113.8	1.5	high	8.3	4.4	0.05	17	11.6
PS	64	F	3	1	20	4.1	128	1.35	1.02	90.14	1.8	high	9	2.7	0.57	23	8.8
PS	55	M	2	1	20.8	4	102	1.15	0.92	87.79	1.8	high	8.9	7	0.14	29	11.2
PS	63	M	2	1	21.6	3.9		1.26	0.65	135.7	2.1	high		5.2	0.1	29	11.7
PS	42	M	2	1	25.5	4.4	58	1.86	0.97	103.5	1.5	high		5.6	0.08	27	12
PS	51	F	2	1	21.6	3.9	163	1.34	0.86	68.09	1.5	high		5.5	0.1	28	10.5
PS	78	F	2	1	26.1	3.3	117	1.19	0.83	86.9	1.5	high	9.4	5.8	0.05	21	10
PS	69	M	2	1	22.9	3.7	162	1.28	0.64	73.61	2.1	high	7.9	10.3	0.17	29	10.7
PS	81	F	2	1	20.3	3.3	138	1.14	1	129.9	1.3	high	8.3	3.2		22	8.8
PS	67	M	2	1	17.5	3.2	146	1.44	0.68	62.53	1.6	high		4.4	0.3	26	11.3
PS	81	F	2	1	19	3.5	140	1.67	0.95	64.42	1.6	high	8.3	4.6	0.07	19	9.9
PS	86	F	2	1	20.9	3.6	129	2.04	1.05	94.46	1.8	high	7.7	3.9	0.01	24	13.7
PS	58	F	7	1	31.4	4.1	176	1.72	0.62	64.82	1.8	high	9.8	5.1	0.06	25	10.5
PS	64	F	4	1	24.2	3.7	136	1.2	0.91	113.3	1.8	high	8.7	2.8	0.1	43	8.1
PS	66	F	6	1	20.3	3.8	203	1.55	0.98	142.2	2.1	high		8	0.03	31	11.5
PS	65	F	4	1	15.4	4	113	1.49	1.02	93.8	1.8	high		4	0.27		9.8
PS	42	M	15	1	26.5	3.6	139	1.61	0.57	97.99	1.3	high	8.9	5	0.4	39	10.9
PS	67	F	8	1	23.8	3.5	160	1.4	0.88	103.3	1.8	high	10.2	6.2	0.02	22	10.7
PS	71	M	31	1	20.1	3.3	118	1.25	0.89	82.26	1.8	high	8.6	5.1	0.03	26	10.3
PS	64	F	4	1	21.4	3.8	169	1.81	0.81	98.92	2.1	high	8.4	6.4	0.36	28	11.3
PS	59	M	33	1	17.2	4.3	150	1.74	0.65	95.98	2.1	high	9.4	5.5		26	11.1
PS	57	F	21	1	18.5	4.3	161	1.5	1.24	75.68	1.6	high		5.5	0.22	27	10.5
PS	55	M	17	1	25.9	4	118	1.15	0.86	106.2	2.1	high	8.7	8	0	28	8.9
PS	61	F	16	1	24.6	4.4	83	1.22	0.82	139	2.1	high	8.6	5.2			11.9
PS	37	M	15	1	20.5	4.4	154	1.65	1.13	115	2.1	high	9.4	7.6			11.3
PS	42	M	15	1	17.1	4.4	173	2.14	0.88	94.12	1.5	high	8.9	3.9	1.36	19	9.9
PS	58	M	14	1	20.7	4.2	146	1.47	0.81	98.17	1	high	9.6	3.6		34	9.1
PS	60	M	14	1	23.5	4.4	163	1.47	0.88	124.5	1.5	high		3.1	1.27		10.4
PS	74	F	14	1	18.2	4	84	1.19	0.91	111	1.4	high	10.6	2.8		34	9.6
PS	59	M	13	1	20.2	4.3	196	1.66	0.88	108.2	2.1	high	6	4.1		31	10.9
PS	67	M	13	1	23	3.9	150	1.1	0.61	103	1.4	high	9.1	3.5		29	10.5
PS	65	M	12	1	21.5	3.8	186	1.25	0.68	93.12	1.4	high	9.7	3.7		28	8.9
PS	64	F	12	1	25.2	4.4	169	1.84	1.03	100.1	1.6	high	8.8	5.5	0	26	11.1
PS	43	M	11	1	20.6	4.6	175	1.23	0.89	121.9	1.8	high		5.7	3.43	27	12.7

PS	76	F	10	1	20.7	3.9	114	0.96	1.06	96.23	1.8	high	9.5	4.4			10
PS	60	M	9	1	19.6	4.6	253	1.23	0.88	83.98	2.1	high	8.8	2.8		24	9.7
PS	65	M	9	1	21.3	4.4	116	1.16	1.02	116.9	1.6	high		4.2		30	9.8
PS	56	M	8	1	22.7	4.2	138	1.36	0.87	123.4	2.1	high	8.2	4.2			10.2
PS	61	F	7	1	22.1	4.3	152	1.16	1.22	119.8	1.8	high	8.6	7.5		22	10.1
PS	67	M	7	1	23	3.9	134	1.36	1.08	131.4	2.1	high	9.7	6.5		23	9.9
PS	53	F	6	1	19.1	4.1	151	2.02	1.32	141.4	1.8	high	9.5	5.9	0.05	29	10.2
PS	59	M	6	1	20.8	4.6	164	1.33	1.03	86.26	1.8	high	9.6	5		24	8
PS	44	F	6	1	29.5	4.3	81	1.18	0.9	134.5	1.4	high	7.8	4.3	0.39	28	8.1
PS	65	M	5	1	24.9	4.7	188	1.91	1	125.7	1.8	high	9.3	5.9		17	10.1
PS	55	F	4	1	17	4.1	130	1.23	0.89	98.05	2.1	high	8.9	5.1			11
PS	69	M	4	1	23.9	3.9	145	1.32	0.82	108.3	2.1	high	9.8	5.6		26	11.7
PS	49	M	4	1	32.7	3.9	164	1.63	0.91	78.42	1.8	high	9.2	4.1			11
PS	68	M	3	1	28.2	4	150	2	1.08	125.4	1.6	high	8	5.7	0.4	26	12.3
PS	65	M	2	1	20.7	4.3	157	1.58	0.65	89.31	2.1	high	9.8	6.4		40	10.5
PS	64	M	2	1	30.3	3.8	124	1.42	1.16	105.1	1.6	high	8.3	4.5	0	23	10.3
PS	71	M	2	1	26.1	3.9	42	1.31	0.81	71.09	1.8	high		4.8	0.14	25	10.6
PS	31	M	2	1	19.5	4.9	117	1.17	0.93	65.22	1.8	high	9.2	5.2	0.01	20	9.7
PS	47	M	3	1	19.2	3.6		1.26	0.94	57.15	2.1	high	8.5	3.7		36	11
PS	33	M	2	1	17.8	3.9	139	1.53	0.81	69.88	2.1	high	9.8	5.1		32	9.3
PS	76	M	4	1	22.2	3.7	226	1.52	0.82	106	2.1	high	7.3	5.8	0.1	25	10.3
PS	44	F	4	1	19.9	3.7	172	1.52	0.78	51.42	2.1	high	9.3	5.7		21	11.1
PS	72	M	8	1	23	3.9	128	1.49	1.12	130.2	1.6	high	8.6	3.5	0.11	33	9.3
PS	67	M	4	1	21.8	3.8	121	1.65	0.71	126.3	1.8	high	8.4	6.8	0.2	24	9.7
PS	54	M	5	1	24	3.6	136	1.15	0.86	150.4	1.5	high		3.1	0.23	25	10.1
PS	48	M	4	1	23.7	3.8		1.69	0.92	86.19	2.5	high	8.2	5.6	0.05	23	11
PS	46	M	3	1	24.5	4	123	1.48	0.84	96.23	1.8	high		3.8			10.6
PS	61	M	3	1	22.2	3.7	149	1.48	0.82	108.7	1.3	high		4.4			9.3
PS	65	F	9	1	19.9	4		1.55	0.97	131.6	2.5	high	9.7	6.8	0.04	27	10.8
PS	60	M	13	1	19.4	4.2	158	1.87	0.98	121.6	1.5	high	8.5	6.7	0.04	18	8.9
PS	67	F	13	1	21.4	4	139	1.36	0.88	113.4	1.8	high	10.5	5.7	0.03	29	10.1
PS	52	M	7	1	29.1	3.9	106	1.02	1.01	138.1	1.8	high	8.2	5.4	0.1	37	7.6
PS	71	M	2	1	25.7	4.1	117	1.44	0.87	114.2	1.8	high	9.3	6.5	0.27	29	11
PS	69	M	29	1	16.9	3.3		1.75	0.83	21.93	2.5	high	10.6	4.5	0.02	46	9.2
PS	53	M	24	1	16.4	4.1	172	1.41	0.95	91.31	1.8	high	7.7	6.9	0.02		10.5
PS	65	M	8	1	19.6	3.7		1.56	0.76	87.31	2.5	high	8.7	3.5	0.04	50	9.9
PS	71	M	7	1	26.5	3.8	82	1.43	0.74	111.5	1.5	high	7.8	3.8	0.05	35	9.8
PS	18	M	9	1	17.8	3.9	139	1.32	1.22	74.8	2	high	9.2	4.4	0.22	44	10.1
PS	47	F	18	1	20.3	3.9	178	1.53	0.91	116.5	1.3	high		7.6	0.03	29	10.3
PS	63	M	4	1	18.6	3.5		1.49	1.59	80.61	2.5	high	8.6	4.9	0.16	38	10.3
PS	54	M	7	1	19.8	4.1		1.71	1.01	123.1	2.5	high	8.4	7.4	0.09	33	10.6
PS	59	M	4	1	22.5	3.9	143	1.59	1.09	141.7	1.4	high	8.1	3.7		22	7.9
PS	60	M	8	1	23.7	4.2	137	1.37	0.95	96.95	2.1	high	10.2	7.5	0.08	31	10.9
PS	44	M	26	1	29.3	3.3	167	1.3	0.88	90.15	1	high	7.4	4.6		27	8.7
PS	60	F	24	1	17.8	3.9	110	1.68	0.82	92.19	2.1	high	10.3	6.3		22	9.4
PS	42	M	23	1	18.1	3.9	123	1.24	1.07	85.19	1	high		3.4	1.2		8.7
PS	45	F	18	1	18.6	3.9	96	1.09	1.19	108	2.1	high	9.8	5		30	11
PS	55	M	18	1	22.1	3.2	134	1.15	0.96	111.3	1.4	high	8.5	3.8		8	8.2
PS	52	F	17	1	28.5	3.4	144	1.23	1.32	131.6	1.8	high	10.4	2.5		26	8.5
PS	57	F	16	1	21.4	3.3	152	1.42	1.35	109.9	1.4	high	9.8	6.9		27	9.2
PS	63	F	16	1	17.8	3.3	151	1.44	0.89	100.9	1.5	high	10.3	6.4		32	10.6
PS	48	M	14	1	23.2	3.8	144	1.65	1.08	115	1	high	8.3	3.9			9
PS	60	F	14	1	21.3	3.6	154	1.57	0.75	66.82	1.8	high	9	6	0.05	26	12.7
PS	58	M	14	1	22.2	3.8	110	1.21	0.91	96.64	2.1	high		6.3	0.05	24	11.9
PS	66	M	13	1	23.9	4.1	141	1.54	0.89	119.4	1.8	high		6.1	0.33	30	10.8
PS	67	M	13	1	20.5	3.4	207	1.22	0.98	116.3	1.4	high	10.7	8.3		26	11
PS	52	F	12	1	21.6	4	105	0.95	1.2	107.1	1.8	high	8.7	4.1		16	11.3
PS	72	M	11	1	19.6	4	165	1.81	1.01	102.3	1.8	high	9.1	3.9	0.3	48	9.9
PS	55	M	11	1	22.3	3.4	157	2.43	0.99	105.8	1.6	high	8.6	9	0.05	29	12

PS	57	M	10	1	26.9	4	198	1.43	1.07	145	1.8	high	9.1	3.8	0.03	22	11.4
PS	66	F	10	1	16.4	3.5	159	1.75	1.08	103.8	1.8	high		3	0.05	15	11.7
PS	33	M	10	1	21.7	4	161	2.28	1	109.4	2.1	high	8.8	3.8	0.1	24	8.6
PS	53	F	9	1	23.7	3.7	213	1.43	1.03	102.8	1.8	high	8.6	5.7	0.1	9	
PS	79	F	9	1	20.1	3.4	152	1.46	1.02	115.8	1.8	high	8.5	5.3	0.9	23	9.8
PS	44	M	8	1	19.6	3.7	162	1.54	0.98	114.1	1.4	high	8.5	4	0.13	20	9.4
PS	62	M	6	1	20.8	3.7	133	1.37	1.05	107.9	2.1	high	8	5.2	0.06	29	8.4
PS	59	M	6	1	26	3.5	120	1	0.73	109.5	1.1	high		1.9	1.18	39	10.2
PS	64	M	6	1	16.8	3.6	161	1.24	0.66	65.14	2.5	high	11.4	7.4	0.2	22	8.6
PS	60	M	6	1	20.3	4.1	191	2.04	0.92	100.5	1.5	high		5.3	0.05	18	10.1
PS	78	F	5	1	23	3.6	195	1.3	1.25	108.9	1.3	high	8.8	2.2	0.05		11
PS	71	M	5	1	21.3	3.9	121	1.46	0.97	77.83	2.1	high		4.4	0.05	24	9.7
PS	41	F	5	1	16	3.8	118	1.17	0.93	103	2.1	high	9.5	4.4		33	12.3
PS	89	F	4	1	22.2	3.6	120	2.02	1	50.76	1.5	high		5.4	0.05	21	11.7
PS	67	M	4	1	20	3.6	208	1.43	1.07	111.8	1.5	high	8.5	6.5	0		11
PS	63	M	3	1	30.1	3.5	144	1.7	1	69.17	2.1	high		5.5	0.05	16	10.6
PS	68	M	2	1	21	3.6	151	1.79	1.1	100	1.5	high		3.8	0.08	12	11.2
PS	80	M	3	1	22.2	3.5	177	1.55	0.69	95.84	2.1	high	10.4	6.6	0.07	19	10.6
PS	77	M	10	1	21	3.6	158	1.38	0.81	121	1.8	high	9	7.2	0.05	21	9.8
PS	67	M	3	1	27.1	3.5	117	1.64	0.73	86.36	1.5	high		5.7	0.05		12.6
PS	79	M	4	1	20.4	3.2	164	1.8	0.7	98.72	1.8	high	8.4	8.7	0.66	26	9.3
PS	66	F	6	1	26.2	3.8	160	1.74	0.88	112.5	1.5	high	8.4	5.2	0.03	57	11.2
PS	55	M	17	1	20.2	3.7	136	1.32	0.97	104.6	2.1	high		5.9			10.9
PS	44	M	11	1	23.6	4	129	1.55	1.1	91.65	1.5	high	7.7	7.4		24	9.6
PS	51	F	5	1	24.7	3.6	164	1.43	0.82	101.7	1.5	high	8.2	7.5	0.3	33	9.8
PS	73	M	4	1	19.4	4.1	161	1.13	0.88	113.7	1.5	high	9.3	5.3	0	22	11
PS	63	M	14	1	20.8	4.2	144	1.32	0.94	110.2	2.1	high	9.7	5.1	0.1	25	9.9
PS	50	M	15	1	20.6	3.7	167	2.02	0.95	113	1.4	high	9	4.6	0.85	26	8.9
PS	50	M	4	1	32.3	4	188	1.88	0.95	111.8	2.1	high	9.8	4.6	0.1	24	9.2
PS	57	M	13	1	25.7	3.2		1.54	0.8	126.4	2.2	high		6.6			
PS	52	F	11	1	18.2	3.6		1.77	1.18	99.3	2.5	high	9	4.8	0.08	37	10.5
PS	59	F	9	1	19.2	4	300	1.22	1.15	93.51	2.1	high	10.1	3.2	0.13	29	13.1
PS	57	M	4	1	18.3	3.4	150	1.73	0.85	111.5	1.6	high	9.3	4	1.1	33	9.3
PS	61	F	5	1	19.7	3.5	127	1.47	0.92	69.65	2.1	high	9.7	7.5	0.06	25	12
PS	68	M	18	1	18.5	3.6	166	1.51	1.08	100.3	1	high		3.7	0.1	31	10.6
PS	84	F	14	1	33.1	3.2	294	1.77	0.61	118.1	2.2	high	9.7	4.7	0.02	25	10.7
PS	48	M	26	1	21	3.6	160	1.28	1.02	104.8	0.6	high	10	2.5	3.38	61	11.6
PS	70	M	3	1	23.9	3.6	126	2.09	1.02	86.76	1.5	high	7.7	5.8		43	10.6
PS	68	M	6	1	22.1	3.8	151	1.6	0.65	82.62	1.5	high	10.3	7.1		32	9
PS	44	M	14	1	18.3	4.1	184	1.73	0.85	83.22	1	high		3.8		25	9.2
PS	61	M	17	1	21	3.3	249	1.71	0.87	93.68	1.6	high	10.6	5.4	0.04		10.2
PS	58	F	18	1	21.9	3.8	153	1.54	0.64	120.9	2.1	high	9.2	6.4	0.49	30	8.7
PS	43	F	6	1	21.7	3.5	153	1.95	1.18	102	1.8	high	10	3.7	0	25	10.4
PS	50	M	4	1	22.4	4.1	110	1.38	0.75	116.9	2.1	high	8.6	5.3		23	9.7
PS	31	F	9	1	15.2	3.8	147	2	1.14	77.22	1	high		3.8	3.2	27	10.7
PS	73	F	27	1	20.4	2.8	148	1.31	1.06	78.71	2.2	high	10	7.1	0.1	22	9.7
PS	61	F	34	1	14.1	3.7	161	1.16	0.9	95.03	1.9	high		5.4	0.47	28	11.6
PS	57	M	2	1	20.7	4.1	129	1.6	0.74	99.96	2.1	high	8.5	3.9	0.04	26	10.7
PS	55	M	6	1	23.1	4.1	158	1.24	0.99	97.75	2.1	high	9.8	4.8			10.4
PS	61	M	16	1	16.7	4	140	1.59	0.95	111.8	1.4	high	7.5	1.6	0.08	22	10.5
PS	56	M	35	1	19.1	3.2	109	1.6	1	93.24	1.6	high		4	0.65	19	11.2
PS	40	M	14	1	15.3	3.8	111	1.02	0.92	100	1.3	high	8.1	5.6	0.08	39	10.1
PS	74	M	27	1	21.1	3.5		0.97	0.67	96.14	1.4	high	8.7	3.9	0.08	24	11.2
PS	58	M	22	1	22.5	3.8	110	1.35	0.85	105.7	1.8	high		4.8	0.05	22	10.1
PS	82	M	19	1	19.1	4	161	1.62	0.9	109.6	2.1	high	9.4	6.3	0.49	28	10.7
PS	41	M	3	1	22.2	4	205	1.38	0.78	104	2.2	high	9.8	3		20	9.9
PS	58	M	10	1	20.4	3.3	125	1.18	0.71	104.8	1.8	high	8.6	5.8			10
PS	53	M	24	1	23.4	4	139	1.74	1.01	110	1.8	high	9.3	3.1	0.11	20	8.2
PS	76	F	6	1	20	3.7	268	2.2	1	91.91	1.8	high	9	4.1	0.05	23	11.1

PS	74	F	2	1	28.6	3.3	215	1.43	0.74	90.14	2.1	high	9	4.1	0.14	32	10
PS	67	F	27	1	18.9	3	138	1.21	1.41	91.64	1.8	high	7.8	3.1	5.62	33	8.9
PS	61	F	6	1	23.8	3.8	135	1.24	0.86	111	1.5	high		4.8		20	11
PS	58	F	5	1	24.2	3.7	190	1.32	0.87	142	1.3	high	8.9	4.1	0.93	37	10.6
PS	65	M	17	1	17.5	3.7	172	1.99	1.04	111.6	1.8	high	10.2	5.6			10.1
PS	39	F	9	1	21.5	4.1	210	1.8	1.07	130.7	1.4	high	10.3	4.3	0.29	36	9.3
PS	62	M	6	1	23	3.5	183	1.6	1.02	135.9	1.8	high	9	4.8	0.05	14	10.2
PS	52	M	3	1	27.8	3.2	163	1.21	0.72	93.14	2.1	high	10.8	5.4		25	11.7
PS	46	M	2	1	22.1	4.2	210	1.13	0.83	101.7	1.8	high	9.7	5.6	0.05		
PS	71	F	6	1	22.2	3.3	120	1.24	1.15	136.4	1.8	high	11	4.3	0.1	21	9.8
PS	67	F	12	1	18.4	3.4	129	1.46	0.83	127	1.5	high	7.8	5.8	1.83	18	10
PS	59	M	3	1	32.4	3.4	207	2.27	1.05	97.93	1.5	high	10	6.3		27	9.4
PS	73	M	7	1	26.7	3.6	198	0.93	1.04	107.4	1.8	high		6.4		29	8.6
PS	39	M	25	1	23.4	3.7	159	1.62	0.89	89.83	2.5	high	9.6	5.6	0.05	20	10
PS	71	M	4	1	20.6	3.7	107	1.41	0.8	89.44	2.1	high	8.6	5	0.04	29	10
PS	71	F	2	1	22.9	3.4	128	0.91	0.76	76.3	1.5	high	9	5.5	0.15	25	
PS	49	M	6	1	21.1	4	124	1.43	0.75	91.89	1.6	high	11.1	3.8	0.06	29	9.8
PS	61	M	19	1	21.9	3.8	143	1.35	0.72	110.7	1.8	high	8.7	5.1			11.5
PS	56	F	24	1	20.1	3	148	1.43	1	104	1.6	high		4.2	0.03	20	11.3
PS	67	M	26	1	21.2	3.4	191	1.45	0.76	113.4	1.4	high	9.1	6.8	0.7	11	
PS	46	M	29	1	22.1	4.4	191	1.65	0.69	86.71	1.6	high		6.6	1.8	27	11
PS	57	F	10	1	24.5	3.6	240	1.34	0.95	120.8	2.1	high	9.1	4.9			9.7
PS	48	M	6	1	20	3.6	175	1.39	0.86	73.25	1.5	high	8.7	5.7	0.02	21	8.7
PS	84	M	6	1	13.5	3.5	141	1.12	0.83	116.7	1.5	high	9	5.4	0.17	30	9.8
PS	58	F	4	1	17.9	3.9	131	1.36	1.29	105.9	1.5	high	9.3	4.9	0.2	23	13.3
PS	34	F	10	1	24.1	4	147	1.77	0.91	112.5	1.5	high	9.6	7.6	0.1	23	10.4
PS	48	M	18	1	19.4	3.8	191	1.36	0.71	119	1.8	high		7.9	0.09	31	12.5
PS	60	F	21	1	17	3.3	105	1.04	0.73	88.48	2.1	high	9.3	7.1		27	9
PS	50	M	15	1	19.1	3.9	188	1.68	0.94	93.93	1.4	high	7.9	7.8	0.07	31	11.6
PS	78	M	4	1	20	3.8	182	1.71	0.68	80.54	1.5	high	9.7	5.4	0.02	30	10.6
PS	35	M	12	1	21.6	3.9	119	1.32	1.02	91.03	1.5	high		4.6	0.3	22	10.3
PS	69	M	4	1	18.8	3.5		1.73	0.81	89.84	1.5	high	9.2	4.9	1	45	9.3
PS	43	M	23	1	19.2	3.7	156	1.22	0.76	112.8	1.4	high	8.7	4.7	0	24	8.7
PS	61	M	26	1	17.8	3.1	127	1.8	0.78	92.34	1.6	high		4.1	0.1	39	12.4
PS	74	M	4	1	18.6	3.7	159	1.48	0.99	106.8	1.5	high	10.2	8	0.04	56	11.5
PS	59	F	9	1	15.2	3.6	216	1.82	1.11	117.6	1.3	high	9	5.1	0.05	30	10.6
PS	61	M	6	1	19.7	3.9	104	1.26	0.88	88.98		high	8.7	2.9	0.43		
PS	47	F	14	1	21.8	3.6	106	1.25	0.8	132	2.2	high		4.2	0.3	20	11.9
PS	73	F	4	1	21.5	3.9	145	1.15	0.94	125	1.8	high	11.1	4.5			10.2
PS	68	M	11	1	19.7	3.3	175	1.2	0.67	125.4	2.1	high	9.4	5.6	0.05	30	10.1
PS	74	M	6	1	19.7	4	124	1.03	0.9	141.6	2.1	high	9.3	5.8	0.59	27	11.5
PS	56	M	30	1	18.7	3.6	118	1.27	0.77	91.18	1.6	high		6.4	0.1	26	10.2
PS	74	F	3	1	23.3	3.5	121	1.14	0.76	87.35	2.1	high		6.4	0.24	27	11.7
PS	60	M	7	1	18.9	3.6		1.62	1.08	109.9	1.4	high	8.5	2.2			10.4
PS	67	M	10	1	18.3	3.7	86	1.24	0.85	114.7	1.8	high	10.2	5.6	22.1	39	8.4
PS	39	F	8	1	19.4	4	214	1.01	0.93	106.6	2.1	high		6.9	0.1	41	10.3
PS	44	F	5	1	20.8	3.5	154	1.6	1.27	115.1	2.1	high		5.9	0.05	26	12.8
PS	55	M	9	1	16.5	3.7	121	1.25	0.9	119.8	1.5	high	9.8	4.6	1.03		11
PS	60	F	24	1	21.8	3.5	162	1.56	0.89	111.8	1.6	high	8.7	4.3	0.62		12.3
PS	50	M	16	1	25.2	3.6	140	1.67	0.93	86.89	1.1	high	9.3	6		30	11.4
PS	48	M	2	1	19.9	4.2	173	1.05	0.61	70	2.2	high	7.7	17.2	1.13	32	10
PS	63	M	16	1	19	3.9	146	1.22	0.72	93.48	2.2	high	8	5.6	0.05	26	9.7
PS	58	M	16	1	23.9	3.6	160	1.48	0.72	105.5	1.8	high	10.8	9	0.05	28	8.7
PS	31	M	7	1	17.4	4.3	134	1.13	0.97	105.8	2.1	high	8.8	7.1			11.2
PS	40	F	5	1	16.7	3.9	170	1.76	1.11	69.33	1.8	high	8	7.5	0.1	29	9.3
PS	77	M	13	1	17.7	3.9		1.51	0.91	105.9	2.1	high	9.1	4.8	0.03	21	10.4
PS	75	F	11	1	18	3.6	120	1.42	0.84	140.2	1.1	high	8.9	5.8		27	13.2
PS	33	F	13	1	18.9	4.5	135	1.35	1.14	103	1.8	high		4.1	0.1	24	12.1
PS	44	M	26	1	18.6	3.5	119	1.48	1.07	91.24	1.8	high	7.5	4.7	8.8	30	9

PS	54	M	4	1	25.4	3.4	208	1.37	0.79	68.93	2.1	high	8.6	4.9		27	
PS	66	M	2	1	15.3	3.1	206	1.23	0.97	93.81	2.1	high	8.3	5.4		30	12.1
PS	56	F	7	1	21.5	3.6	230	1.94	1.19	102.5	1.9	high	9.1	5.6	0.15	24	9.9
PS	65	M	22	1	24.2	3.5	153	1.36	0.69	135.5	1.8	high	9.2	5.2	0.1	29	12.1
PS	45	M	3	1	19.8	3.4	150	1.74	0.94	101.1	1.5	high	8.5	2.4	0.48	24	10.7
PS	59	F	10	1	21.2	3.4	144	0.84	0.93	104.8	1.6	high	9.4	4.7	0	16	11.2
PS	72	M	25	1	17.4	3.5	300	1.19	0.98	102	1.9	high	10	7.6	0.13	31	10.5
PS	51	M	2	1	24.4	4.1	173	1.32	0.83	60.71	1.5	high		5.5	0.1	30	11.6
PS	56	M	15	1	22.6	4.2	111	1.46	1.11	141.6	1.4	high	9.6	4.2	0.18	34	13.4
PS	53	F	8	1	19.1	3.7	128	1.41	0.97	103.4		high	8.3	6.6	0.28		
PS	73	M	4	1	20.3	4.3	156	0.99	0.9	93.48	2.1	high		4.9	0.2	24	11.6
PS	55	M	14	1	25.6	3.4	118	1.3	0.87	115.7	1.8	high	9.4	5.9	0.18	34	10.5
PS	68	M	11	1	22.4	3.8	98	1.11	0.74	113.1	2.5	high	9.2	5.7	0.17	26	9.8
PS	83	F	15	1	18.2	3.5		1.69	0.78	96.42	2.5	high	7.7	8.8	0.19	24	14.1
PS	69	M	2	1	21.7	3.6	176	1.82	0.82	108.4	1	high	9.1	6	0.02		9.2
PS	63	F	7	1	18.6	3.8	291	1.88	1.05	110.4	1.8	high	8	3.4	0.11	21	10.9
PS	73	M	2	1	22.4	4.2	145	1.52	0.85	72.26	2.1	high	10.6	5.2	3.63	27	10.6
PS	65	M	7	1	24	4	130	1.36	1.23	177.5	1.5	high		4.8	0	24	11.3
PS	73	M	16	1	19	4	147	1.33	0.91	116.8	1.4	high		5	0.02		11
PS	70	M	12	1	21.5	4	101	1.21	1	55.34	2.1	high	9.2	5.2	1.45	26	10.6
PS	72	F	4	1	21.6	3.4	209	2.15	0.99	69.7	1.6	high	9.6	4	0.03	25	9.2
PS	61	M	3	1	27.2	3.6	143	1.75	0.82	116.9	2.1	high	8.1	3.7	0.1		9.9
PS	67	M	2	1	21.7	4.1	331	1.23	1.12	107.2	2.2	high	9.3	4.6	0.04	22	11.8
PS	38	M	16	1	20.4	4.2	126	2.02	1.01	119.1	1.4	high	8.8	6.1	0.39	38	12.3
PS	57	M	15	1	21.8	2.7	174	1.37	1.33	88.91	1.4	high	10.1	5.8	0.1	32	10.1
PS	64	M	11	1	21.7	2.8	194	1.29	0.71	119.5	2.1	high	10	5.5	0.07	27	12.7
PS	62	F	2	1	19.9	3.9	185	1.55	0.8	89.61	1.3	high	6.8	5.4	0.05	32	9.8
PS	54	F	7	1	24.8	3.9	159	1.32	0.86	89.32	1.3	high	9.4	4.6	0.17	26	11.3
PS	71	F	4	1	22.9	3.9	153	1.1	0.82	114.8	2.1	high	7.8	7.6	0.05	21	12.3
PS	67	M	6	1	21.3	3.9	143	1.64	0.83	100.8	1.5	high	8.9	6.2	0.35	31	
PS	55	F	5	1	17.3	3.4	266	1.48	0.94	120.2	1.6	high	9.5	4.2	0.03	26	12.2
PS	57	M	4	1	29.1	3.5	119	1.39	0.67	55.96	2.1	high		10	0.13	38	8.9
PS	55	F	3	1	22.3	4	146	1.04	0.87	132.9	1.8	high	9	5.6	0.05	25	12
PS	76	F	5	1	18.2	4	184	1.41	0.93	97.8	1.8	high	10.2	4.3	0.07	30	11
PS	56	F	5	1	31.6	3.6	140	1.34	0.68	92.87	1.5	high	9.1	5.3			12.3
PS	81	F	4	1	17.9	3.9	202	1.45	0.94	127.4	1.3	high	8.9	4.2	0.33	23	10.8
PS	52	F	3	1	20.7	3.8	269	1.45	0.82	69.59	2.2	high	9.5	7.3	0.21	24	13.1
PS	53	M	20	1	19.5	3.8	165	1.66	0.81	99.49	1.8	high	9.4	7.8	0.08	28	10.3
PS	64	M	3	1	28.8	4	277	1.22	0.87	97.93	1.8	high	9.6	6	0.1	26	10
PS	66	F	2	1	23.4	4.2	204	1.48	0.8	101.6	1.5	high	9.6	2.5	0.14	24	10.3
PS	66	M	6	1	21.6	3.8	218	1.36	1.01	129.7	2.2	high	8.7	4.6	0.05	26	10.2
PS	53	F	6	1	25.2	3.6	170	1.44	0.93	85.47	2.2	high	8.8	6	0.88	39	11.2
PS	56	M	2	1	24.3	3.5		1.56	0.82	55.37	2.1	high	9.4	5.1		21	
PS	60	F	2	1	19.9	3.7	103	1.17	0.78	77.2	1.5	high	7.6	4.4	0.05	26	8.4
PS	59	M	5	1	23	3.9	125	1.07	0.81	84.26	1.3	high		6.6	0.1	29	9.8
PS	36	F	19	1	16.3	3.8	127	1.11	0.87	46.29	2.1	high	11	3.8		27	6.6
PS	43	M	8	1	21.5	3.5	187	1.59	0.78	102.5	1.4	high	8.5	7	0.1	31	11.4
PS	70	M	3	1	17.3	3.7	134	1.47	0.77	73.42	1.3	high	8.8	5.6		27	11.8
PS	64	F	2	1	25.7	3.7	93	1.44	0.8	83.23	1.4	high	8.4	5.1	0.6	30	9.6
PS	42	F	17	1	17.2	4.1	124	1.31	0.85	86.56	2.1	high	8.4	7.2	0.11		9
PS	52	F	24	1	19.5	3.9	91	1.35	0.82	102.6	1.8	high	8.4	5.5	0.18	18	10.6
PS	67	M	15	1	23.8	3.6	203	1.28	0.92	99.99	1.4	high	8.2	5.4	0.7	21	11.1
PS	60	M	2	1	22.5	4	241	1.27	0.75	59.78	1.5	high	9.5	3.8	0.03	28	12.6
PS	60	F	3	1	19.4	3.7		1.08	0.64	66.07	1.5	high	10.1	4.4	0.01	23	11.8
PS	60	M	3	1	23.6	3.5	223	1.68	0.76	76.66	2.1	high	9.7	6.7		33	9.9
PS	56	M	4	1	20.9	4	118	1.49	0.99	83.98	1.8	high		5.3	0.69	24	14
PS	62	M	13	1	19.6	3.4	115	1.27	1.02	94.82	2.2	high	9.7	4.4	4.85	34	7.9
PS	61	F	11	1	21.9	3.8	133	1.19	0.7	96.66	1.5	high		10.3	0.34		11
PS	57	F	6	1	26.4	4.1	139	1.28	0.83	97.28	1.5	high	9.5	3.4	0.77	30	8.6

PS	65	F	2	1	19	3.7	197	1.25	0.76	71.68	2.1	high	8.1	4.4	0.3	15	11.4
PS	63	M	32	1	22.6	3.7	129	1.43	0.92	78.74	1.8	high		3.4	0.05	26	9.6
PS	64	M	5	1	22.1	4.2	159	1.42	0.91	95.8	2.1	high	8.8	5.1	1.5	24	12
PS	72	M	2	1	22.1	4.2	136	1.39	0.93	92.57	1.8	high	9.9	4.6	0.04	23	8.6
PS	43	M	15	1	18.4	4.2	136	1.61	0.86	77.93	1.5	high		3.9	0	19	9.2
PS	30	M	6	1	19.5	4	168	1.48	1.11	95.79	1.8	high	7.9	6.9	0.09	29	11.1
PS	37	F	2	1	19.1	3.7	144	1.21	0.82	75.08	2.1	high	9.2	4.8		27	9.3
PS	39	M	13	1	25.1	3.8	138	1.26	0.67	77.93	2.2	high	9.3	6.9	0.19	28	11.6
PS	55	F	9	1	19.9	4	181	1.58	0.93	84.71	1	high		8.1	0.05	22	11
PS	62	F	5	1	28.8	4	86	1.85	0.75	81.56	1.5	high	8.3	2.3	5.11	42	7.4
PS	72	F	7	1	21.7	4	85	1.19	1.02	104.9	1.6	high	7.9	3.4	7.36	27	10.1
PS	55	M	29	1	18	3.8		1.59	1.01	86.66	2.5	high	8.1	4.2	0.62		10.8
PS	53	M	27	1	18.9	3.7	192	1.23	1.01	90.92	1.5	high	9.7	3.7	1.3	20	9.1
PS	64	M	24	1	17.9	3.8	130	1.42	1.13	104.1	1.3	high		6.2	0.88	30	9.5
PS	52	M	20	1	18.9	4.1	102	1.55	1.04	89.95	2.1	high	9.1	5.5			9.4
PS	41	F	20	1	19.5	3.5		1.73	0.95	91.1	1.6	high	10.3	5.3		27	10.8
PS	53	F	17	1	25.4	3.7	206	1.79	0.79	109.9	1.5	high	10.2	7.5	0.06	30	13.9
PS	69	F	17	1	17.1	4	152	1.19	1.48	101.4	2.2	high		5.3	0.05	29	11.6
PS	65	M	16	1	18.8	3.8	174	1.68	1.01	94.45	1.5	high	8.5	4.9	8.8	25	8.6
PS	72	F	14	1	19.6	3.5	189	1.39	0.89	59.93	1.8	high	9.6	4.8	0.2	16	9.5
PS	55	M	13	1	20.5	3.9	185	1.68	1.41	102.5	1.5	high	8.8	3.3			11.2
PS	64	F	13	1	23	3.6		1.33	0.91	103.4	2.5	high	8.9	4.5	0.1	22	9.2
PS	62	F	11	1	22.3	4	147	1.32	0.94	132.7	2.5	high	9.3	7.4			9.9
PS	54	M	11	1	21.4	3.7		1.24	0.91	107.9	1.8	high	8.7	4.3			10.1
PS	57	F	9	1	19.6	3.9		1.87	0.87	114.8	1	high	9.4	4.2		23	11.3
PS	59	F	8	1	26.4	3.8	133	1.17	0.99	123.3	2.5	high	9.3	5			9.6
PS	51	M	8	1	17.5	3.6	173	1.96	0.94	54.82	2.1	high	8.5	3.3	0.51	27	10.6
PS	65	M	8	1	18.8	3.6		1.28	1.12	121.5	1.8	high	9.5	4.7			10.9
PS	60	M	8	1	18.4	3.3	207	1.74	0.72	103.4	1.8	high	9.3	3.8	0.2	31	10.5
PS	69	F	7	1	19.6	3.9	245	1.18	0.75	86.1	2.2	high	9.4	6	0.04	23	11.3
PS	42	F	7	1	25.2	4.1	114	1.62	0.94	97.52	2.1	high		5.9	0.16		11.1
PS	65	M	6	1	21.1	3.8	161	1.41	1.4	89.15	2.1	high	9.5	8.8			9.8
PS	76	M	5	1	21.7	3.7	185	1.31	1.27	116.7	2.5	high	10.1	8.8			8.7
PS	75	F	5	1	19.6	3.7	187	1.29	0.99	115.2	2.1	high	10.8	8	1.06		10.2
PS	71	F	5	1	20.7	3.8	179	1.03	1.08	62.82	2.1	high	9	4.2			11.3
PS	90	M	4	1	20.8	3.6	122	1.61	0.96	121.2	1.8	high	10.1	3.3	0.03	22	11
PS	58	M	4	1	17.7	3.5	147	1.4	0.95	109.7	2.5	high	9.1	5.3			11.1
PS	67	M	2	1	28.3	3.6	212	1.63	0.71	85.05	2.1	high	8.3	6.7	0.02		10.5
PS	66	M	2	1	17.3	3.9	182	1.48	1.13	61.14	1.1	high	8.7	6			9.6
PS	63	M	2	1	21.1	4.2	181	1.37	1.03	82.04	1.5	high	9.1	4.9	0.05	28	10.6
PS	57	M	2	1	24.2	4.4	178	1.26	1.03	98.29	2.1	high	9.7	5.6	0.8	39	8.7
PS	74	M	4	1	22.1	3.6	133	1.13	0.98	90.16	1.3	high		3.1	0.19	22	10.2
PS	77	F	3	1	22.4	3.7	120	1.61	1.2	108.8	1.8	high	8	4.5	0.06	26	8.8
PS	54	M	25	1	22.4	4.5	116	1.4	1.02	142.2	2.2	high	10.1	4.1	0.1	35	11.8
PS	54	M	25	1	20.6	4.3	195	1.64	1.14	121.8	1.8	high	10.3	8.2	0.04	24	11.2
PS	58	M	17	1	23.9	4.3	132	1.71	0.81	150.2	1.4	high	8.8	7.1	0.12	32	11.3
PS	64	F	17	1	16.9	4.2	143	1.47	1.08	109	1.5	high		5.9	0.04	27	8.6
PS	73	M	15	1	22.4	3.8	162	1.64	0.79	138	2.2	high	8.6	4.1	0.51	26	11.5
PS	44	M	13	1	19.8	4.1	121	1.06	0.92	118	2.2	high	8	3.1	1.54	26	9.2
PS	71	M	11	1	21.6	4	173	1.62	0.86	112.2	1.5	high	8.9	6.7	0.2		10.3
PS	70	M	11	1	15.1	4.4	202	1.45	0.61	99.98	1.4	high	8.3	7.3	0.05	26	10.4
PS	62	F	11	1	17	4.4	155	1.52	1.07	115.6	1.8	high	8	3.3	0.04	26	9.2
PS	65	M	10	1	17.8	4.7	205	1.44	1.07	116.4	2.1	high	8.4	4.2	0.62	28	12.5
PS	69	M	10	1	21.6	4.1	98	1.25	0.88	105	1.8	high	8.6	4.9	0.14	26	12.4
PS	51	F	9	1	21	4.4	139	1.24	1.02	135.5	1.8	high	8.6	5.6	0.12	22	10.8
PS	76	M	8	1	22.4	3.8	153	1.88	0.77	137.8	1.5	high	8.3	5.2	0.01	24	10.4
PS	32	F	8	1	17.4	4.3	170	0.74	0.82	110.7	2.1	high	8.8	3.7	0.34	29	12.1
PS	72	M	6	1	20	4.1	154	2.86	0.95	160.4	1.8	high	9.3	2.8	0.04	26	10.1
PS	62	M	5	1	22.3	4.1	166	1.23	0.81	87.27	2.1	high	10.2	6.4		24	10.6

PS	49	M	5	1	19.1	4.2	123	1.52	0.8	107.2	2.2	high	8.5	4.1	0		10
PS	66	F	3	1	21.7	4.3	167	1.57	0.85	90.82	1.8	high	8.4	6.1	0		10
PS	76	M	2	1	21.4	4.5	125	1.34	0.79	98.36		high	8.8	3.4	0.76		
PS	62	F	2	1	28.4	4.2	135	2.29	1.03	108.5	2.1	high	8.7	3.5		22	11.1
PS	62	M	2	1	19.9	3.8	175	1.63	0.79	87.36	2.1	high	7.9	6	0.05	25	11.7
PS	57	M	2	1	24.6	4.3	148	1.39	0.76	64.85	2.1	high	8.6	6.4	0.05	30	11.6
PS	37	F	8	1	25.4	4.1	196	1.67	0.67	78.3	2	high		4.6	0.03	24	10.3
PS	58	M	3	1	13.8	3.6	221	1.39	0.65	78.01	1.8	high	8.9	6.8	0.46	33	11.3
PS	61	M	24	1	21.4	4.8		1.36	0.77	104	1.5	high		5.6			10.6
PS	36	F	7	1	21	3.8	126	1.89	0.9	104.6	1.5	high		5	0.45	26	8.5
PS	58	F	12	1	22.8	3.9	95	1.41	0.91	123.5	2.2	high	8	7	0.74	30	9.7
PS	59	F	10	1	24.3	3.6	176	1.45	0.79	119.3	2.2	high		3.7		20	10.1
PS	59	F	3	1	21.3	4.1	153	1.1	0.85	78.02	2.1	high	8.5	5.7	1.85	31	12.5
PS	63	F	5	1	21.8	4	130	1.33	0.87	103.1	2.5	high	9.8	7.6	0.1	27	12.2
PS	53	F	10	1	21.7	3.8	141	1.77	0.93	127.8	2.2	high		3.8		25	8.5
PS	66	F	3	1	18.4	3.9	120	1.65	0.96	134.3	2.2	high	10.1	4.5		44	9.9
PS	69	F	4	1	17.4	3.2	143	1.19	0.8	79.51	1.8	high	9.5	4.1	0.1		9.6
PS	60	F	8	1	17.4	3.7	167	1.44	0.82	115.2	1.3	high		4.6	0.02	37	9.4
PS	80	M	28	1	19	3.2	246	1.5	0.89	74.29	1.4	high		9	0.05	27	8.7
PS	64	F	27	1	22.1	3.5	127	1.07	0.93	117.5	2.1	high	9	8.5			9.6
PS	72	M	12	1	20.3	3.3	133	1.38	0.77	106.3	1.5	high	8.5	7		25	9.8
PS	61	M	19	1	19.6	3.3	140	2.46	0.73	88.56	1.4	high	9	2.9	0.02	28	9.7
PS	75	M	13	1	17	3.9	193	1.14	1.04	80.52	2.1	high	10.3	7.4	0.5	24	12.2
PS	59	M	2	1	17	3.9		1.18	0.93	70.73	2.1	high	10.8	7.5	0	20	9.9
PS	43	M	14	1	19.7	4.1	114	1.48	0.97	94.66	2.1	high	8.8	9.9	0.04		8.5
PS	65	M	8	1	24.3	4	201	1.1	1.15	125.7	1.1	high	6.1	5.9	0.03	18	12.4
PS	53	F	2	1	19.3	4.1	110	1.12	0.91	103.6	1.8	high	8.3	6.5	0.05	23	11
PS	60	M	13	1	16.5	3.8	140	2.46	1.06	108.6	1.5	high	10.4	4.2	0.12	28	9.9
PS	60	M	8	1	23.2	3.9	160	1.9	0.74	101.2	1.5	high	9.6	3.8		31	12.3
PS	63	M	9	1	24.1	3.6	178	1.38	0.94	94.34	1.6	high	8.7	6.8	0.2	20	9.7
PS	62	M	18	1	21	3.9	123	1.63	0.97	127.6	2.2	high	8.8	4.8	0.04	26	10.5
PS	30	M	10	1	20.5	4.1	134	1.37	0.92	101.7	1.8	high	10.2	5.4	0.14	21	12.8
PS	64	M	3	1	20.5	3.7	148	1.37	0.85	125.7	2.1	high	9.4	5.5	0.02	25	10.6
PS	59	F	22	1	20.1	3.9	212	1.52	1.11	137.5	1.5	high		4.6	0.25	20	10.4
PS	56	F	16	1	17.3	3.9	174	1.27	0.92	129.1	2.2	high	10.7	5.6		31	12.9
PS	59	M	23	1	23.5	3.7	149	1.44	0.92	126.2	1.6	high		5.6	0.1	24	11.3
PS	48	F	16	1	18.3	4	135	1.35	0.97	123.5	2.2	high	8.3	6.3		25	9.9
PS	65	M	8	1	21.2	3.7		1.68	0.97	121.7	2.1	high	7.4	6.3	5.31		11.2
PS	73	M	11	1	23	4	161	1.31	0.92	113	2.2	high	10.2	5.9		24	11.1
PS	60	M	17	1	24.4	3.8	174	1.01	0.92	125.6	1.8	high	8.4	6.8		18	9.8
PS	60	M	7	1	17.3	4	141	0.91	0.96	134.2	2.1	high	8.1	9.9	0.52	20	10.2
PS	48	M	4	1	18.8	4.4	206	1.32	1.18	103.7	1.3	high	8.8	4.5	0.2	23	10.6
PS	67	M	30	1	20.7	3.5	200	1.81	0.93	114.3	1.8	high	8.6	5.3	0.04	26	11.7
PS	69	F	7	1	17.7	3.3		1.51	1.46		1.8	high	9.6	4.6		19	
PS	69	M	29	1	21.7	4.1	129	1.49	0.96	159	1.8	high		6.5	0.01	24	10.8
PS	73	M	23	1	21.7	3.7	165	1.7	1.02	116.7	1.5	high		3.3	0.05	25	10.9
PS	70	M	16	1	20.9	3.7	185	1.65	0.92	120.7	1.8	high	9.8	6.8	0.03		11.1
PS	60	F	13	1	17.8	3.9	178	1.16	0.86	101.1	2.1	high	8.4	5.5	0.1	16	11.3
PS	56	M	13	1	21.9	4.3	108	1.44	1.08	135	1.5	high		3.9	2.46		9.6
PS	72	F	12	1	17.9	4.1	135	1.48	0.88	144.4	1.1	high	8.9	5.7	0.34	44	9.3
PS	62	F	12	1	19.6	4	155	1.28	1.04	108.4	2.2	high	8	6.6	0.4	34	11.8
PS	61	M	10	1	20.2	4.4	181	1.29	0.92	99.58	2.2	high	9.8	7	1.2	29	10.1
PS	61	M	8	1	18.8	4.2	131	1.74	1.55	130.7	2.2	high	10.8	4.7	0.1	27	9.9
PS	47	M	8	1	23.7	4	125	1.52	0.89	127.4	1.8	high	8.6	4.2	0.08	38	8.5
PS	60	M	7	1	25.8	4.2	121	1.47	0.69	57.72	1.5	high	8.5	5	0.25	31	11.9
PS	64	M	7	1	23.3	4.1	102	1.35	0.9	116.7	1.5	high	8	4.9	0.17		6.8
PS	80	M	6	1	20	3.5	116	1.68	1.08	129.3	1.1	high	9.2	4	0.04	25	10.5
PS	58	F	5	1	26.3	4.1	158	1.76	0.96	131.1	1.8	high	7.6	8.1	1.54	28	9.9
PS	68	F	5	1	17.5	4.4	108	1.54	0.8	123.5	1.3	high	7.5	4.9	0.71	28	10.4

PS	60	M	5	1	21.5	4.1	102	1.72	1.12	133.8	1.3	high	8.6	3.3	0.21	28	10.1
PS	57	M	4	1	21.3	4.5	116	1.57	1.06	97.66	2.1	high	8.2	5.8	0.19	18	10.4
PS	52	M	3	1	24.3	4.7	166	1.73	1.04	140.6	2.2	high	9.4	5.3	0.05	30	8.4
PS	56	F	3	1	16.8	1.9	177	1.65	1.38	118.6	1.6	high		5.1	0.03	30	10.8
PS	76	F	2	1	26.5	3.9	178	1.05	1.12	128.4	2.1	high	10.8	7.8		22	11.5
PS	58	M	7	1	23.2	4	134	1.51	0.95	106.4	2.1	high	8.7	5.3	0.4	44	11.5
PS	61	M	6	1	26.3	3.3	140	1.4	0.86	83.36	1.8	high	9.1	7		28	9.2
PS	80	F	4	1	19.6	3.5	244	1.23	0.76	99.91	1.3	high		4.6	0.17	31	10.6
PS	84	M	2	1	21.6	3.4	194	1.15	0.73	87.6	1.8	high	9.1	3.6			12.1
PS	50	M	29	1	18.2	3.8	204	1.03	0.76	88.42	2.2	high	9.2	5.7	0.06	20	10.5
PS	73	F	2	1	15.9	3.5	136	1.52	0.94	104.4	2.1	high	8.3	5.4	0.2	32	11.3
PS	75	F	5	1	21.5	3.4	206	1.2	0.61	85.74	2.2	high	9.1	5.8	0.09	29	10.8
PS	49	F	14	1	21.8	3.8	143	1.43	0.91	122.9	1.6	high	10.4	6.4	0	24	10
PS	61	F	12	1	17.5	4	209	1.78	1.23	126.5	1.3	high	10.5	6.4	0.05	21	9.9
PS	59	F	12	1	22	3.6	117	1.24	1.16	129.9	1.5	high		5.8	0.1	25	10.6
PS	67	M	11	1	20	4	212	1.51	1.16	110.1	1.6	high	9.2	6.9	0	32	9
PS	81	F	11	1	25.8	3.6	138	1.04	1.19	119.6	1.6	high		6.8	0.01	24	8.3
PS	55	F	11	1	16.2	4.4	189	1.75	1.08	110.8	2.1	high	9.1	5.5	0.08	23	10.9
PS	42	M	10	1	22.3	4.5	171	1.65	0.84	103.1	1.5	high	8.2	4.2	1.19	33	9.3
PS	75	M	10	1	20.3	3.8	170	1.48	0.97	118.3	1.6	high	7.7	7.4	0.54	34	11.1
PS	75	M	5	1	22.9	4	133	1.34	0.9	172.7	1.3	high	8.7	4.3	1.9	27	9.3
PS	63	M	4	1	21	4	125	1.18	0.77	131.1	2.1	high	8.2	5.7	0	18	10.8
PS	69	M	4	1	20.2	4.3	153	1.37	0.95	133.7	1.4	high	9.1	6.7		28	11.9
PS	70	M	3	1	28	3.8	147	1.34	0.9	143	1.8	high	8.6	8.4	0.3	29	11.1
PS	64	M	3	1	23.6	4.2	124	1.3	1.06	112.5	2.1	high	9.3	5.3		25	9.7
PS	74	M	3	1	27.3	3.7	121	1.15	0.73	143	2.1	high	9.3	5.5		29	10
PS	81	F	2	1	26.5	4	165	1.22	0.88	109.6	1.6	high	10.9	6.1	0.1	34	11.8
PS	62	M	2	1	23.6	4.3	147	1.86	0.85	90.97	2.1	high	9	6.4	0.3	20	7.3
PS	60	F	2	1	21.1	4.1	120	1.7	0.9	108.6	1.8	high	9.5	3.2	1.77	27	10.7
PS	85	F	2	1	21.9	3.7	134	1.22	0.72	74.89	2.2	high	8.4	10.9	0.4	44	10.5
PS	68	M	2	1	21.9	3.3		1.37	0.6	86.82	1.8	high	8.5	3.4	0.05	22	10.4
PS	61	F	12	1	22.2	4	152	1.11	1.04	110.5	2.2	high	8.4	3.4	0.11	19	8.3
PS	87	F	7	1	17.3	3.9	177	2.24	0.67	104.6	1.5	high	8	6.1	0.05	29	10
PS	67	M	12	1	20.5	3.4	169	1.95	0.86	74.93	1.3	high	9.4	4.9		26	11.8
PS	70	M	24	1	18.8	3.4	129	1.32	0.73	105.2	1.8	high	9.2	3.6		36	9.5
PS	61	F	7	1	17.2	4.4	134	2.15	1.02	76.85	2.1	high		4.7	0.06	26	10.3
PS	53	M	5	1	27.3	4.5	161	1.67	0.77	78.9	1.5	high	10.5	5.4	0.1	29	10.4
PS	59	F	5	1	24.9	3.9	102	1.52	0.91	133.3	1.5	high	9.5	4.4		33	9.7
PS	58	F	2	1	21.9	3.8	135	1.06	0.8	87.66	2.1	high	8.7	5.8		22	11.5
PS	60	F	5	1	18.7	4.3	83	1.15	0.87	95.89	1.6	high		4.2	0.08	23	9.5
PS	56	M	5	1	22.9	4.3	193	1.36	0.91	41.96	1.5	high		5	0.46	29	10.6
PS	61	F	11	1	21.8	4	148	1.73	0.89	110.9	2.2	high	8.7	5.2	0.2	25	9.7
PS	29	M	3	1	20.6	4.5	203	1.23	0.58	79.85	2.2	high	8.6	4.9	0.06	22	10.2
PS	85	F	5	1	23.2	3.6	174	1.69	0.72	102.6	2.2	high	9.1	7.5	0.49	30	10.1
PS	54	M	9	1	16.3	4.4	146	1.36	1.01	119.7	2.2	high	9.1	5.2	0.05	28	10.7
PS	61	M	8	1	26.9	4.6	114	1.26	1.14	78.57	1.1	high	9.9	4.1	0.1	34	9.1
PS	69	M	5	1	18.8	4.1	111	1.57	1.04	168.3	1.5	high	8.3	4.1	0	24	11.8
PS	87	M	4	1	20	3.9		1.64	0.75	120.8	1.5	high	8	4.7	5.64	35	10.7
PS	67	M	26	1	21.1	4.2	123	1.53	1.14	122.4	2.2	high	9.5	8	0.05	27	9.7
PS	60	M	2	1	20.4	4.3	147	1.26	1.14	93.65	1.6	high	9.4	5.4			8.6
PS	56	F	7	1	30.1	4.4	162	1.44	0.9	106.7	1.3	high		4.7	7.12		11.2
PS	61	F	12	1	23.1	4.3	115	1.24	0.92	113.7	2.2	high		6.9	0.06	25	9.5
PS	72	F	4	1	29.9	4.3	90	1.01	0.75	107.3	1.1	high	7.6	3.3		16	8.9
PS	80	M	2	1	20.1	3.8	165	1.57	1.23	78.74	1.8	high	9.1	8.4	0.08		9.6
PS	63	F	9	1	23.8	3.7	180	1.9	1.06	118.6	1.5	high	8.7	6.5	1.26	27	10
PS	66	F	7	1	14.5	3.2	113	1.14	1.08	93.22	1	high	8.5	6.5	0.05	29	11.4
PS	58	M	5	1	20.1	4	134	0.76	0.69	143.5	1.6	high	11	8.8		30	9.5
PS	59	M	5	1	25	4.3	191	2.01	0.82	134.9	1.3	high	9	5.1	0.09		10.5
PS	61	F	23	1	16		181	1.28	1.19	89	1.6	high	9.7	7.1	0.13		7.3

PS	53	M	23	1	21.9		136	1.64	0.99	94.96	2.2	high	9	4.9	0.05	30	10.6
PS	59	M	19	1	16.8		201	1.29	0.8	93.32	2.2	high	9.7	3.2	0.51	37	9.7
PS	39	M	19	1	19		110	1.69	0.96	105.1	2.2	high	9.4	6.7	0.05	29	10.2
PS	67	M	13	1	17.6		108	1.5	0.99	104.8	1	high	8.3	3.2	0.21	37	10.8
PS	80	F	9	1	18.5		142	1.31	0.94	86.31	1.8	high	9.9	4.3	0	34	7.8
PS	90	F	6	1	16.6		195	2.02	0.87	87.58	2.1	high	9	4	0.1	19	10
PS	52	F	5	1	24.7		172	1.67	1.06	101.7	2.2	high	10	5.4	0.07	22	9.5
PS	76	M	5	1	20.4		179	1.17	1.07	154.3	2.2	high	10	5.2	0.1	25	11.9
PS	60	M	8	1	21	3.7	156	0.87	0.78	126.6	1.3	high	8.6	4		34	10.6
PS	58	M	32	1	22	3.8	150	1.85	0.87	122.9	1.4	high		1.9	0.04	25	10.6
PS	68	F	30	1	20	3.7	182	1.48	1.35	114.3	1.4	high	8.7	4.9	0.04		10.4
PS	67	M	25	1	19.8	4.3	181	1.95	0.96	110.1	1.8	high	8.8	5.9	0.01		9.6
PS	66	F	21	1	19.2	3.4	152	1.53	0.66	104.9	1.8	high	9.5	6.2	0.13	22	10.4
PS	77	M	20	1	21.4	3.5	111	1.28	0.75	84.88	1.5	high	9.1	3.8		33	10.3
PS	61	F	20	1	17.1	3.9	154	1.52	0.91	125	1.6	high		4.3	0.2	25	12.7
PS	64	M	18	1	20.7	4.1	158	1.78	1.06	139.8	1.5	high	8.8	6	0.08	30	10.6
PS	58	M	15	1	18.3	4	163	1.74	1.12	149	2.2	high	9.6	6.7	0.21		8.9
PS	59	M	15	1	17.9	4.3	165	1.69	1.14	112.1	2.1	high	9.4	5.3	0.5	24	10.2
PS	72	M	15	1	23.4	4		1.91	1.03	100.8	1.8	high	9.2	4.6	0.08	38	9.7
PS	76	M	14	1	21.3	4.2	154	1.15	0.97	137.1	1.8	high	7.7	4.3	0.86	19	10
PS	80	M	13	1	25.7	4.2	126	1.18	0.75	111.8	1.5	high	8.3	6.7	0.11	33	8.5
PS	86	F	11	1	21.5	4	150	1.53	0.98	128.9	1.8	high	8.8	5.7	0.42	26	10.4
PS	72	F	10	1	19.6	4	171	0.73	1.21	96.62	2.2	high	9.2	4.7	0.17		9.3
PS	67	M	10	1	22.9	4.1	158	1.47	0.97	148.8	2.2	high	9.5	5.6	0	25	14.4
PS	86	F	8	1	19.4	6.8	119	1.34	0.96	105.4	2.1	high	9.5	3.6		26	12.7
PS	80	M	8	1	20.1	3.3	121	1.21	1.11	111.2	1.4	high	8.8	6.1	1.06		8.8
PS	68	M	8	1	28.1	3.8		1.4	0.85	131	2.1	high	8.9	6.4	0.21	31	10.9
PS	88	F	8	1	21.6	4	141	1.68	0.91	114.6	1.8	high	8.3	4.2	0.28	28	10.6
PS	72	M	7	1	20.8	3.8	153	1.75	0.95	107	1.5	high	8.8	4.8		35	11.7
PS	43	M	6	1	29.9	4.3	138	1.36	1.08	138.7	1.3	high	7.4	4.1	0.17	21	10.4
PS	42	M	6	1	21.7	4	137	1.51	0.67	99.94	1.5	high	9.4	4.5	0.06	26	10.1
PS	45	M	6	1	20.7	4.6		1.25	0.96	109.4	1.5	high		4.6	0.01	32	8.9
PS	63	M	6	1	21	4.1	149	1.7	1.12	93.7	2.1	high	10.2	4.6	0.52	33	9.5
PS	76	F	5	1	25.4	3.4	105	1.66	0.84	113.2	2.1	high	8.5	5	0.3	35	10.3
PS	81	F	5	1	19	3.8		1.51	1.16	90.23	1.8	high	9.8	5	0	23	10.4
PS	72	F	5	1	23.3	3.9	243	1.46	0.98	140.5	1.3	high	10	2.3	1.04	30	11
PS	67	M	4	1	23.4	3.6	196	1.28	1.1	110.6	1.6	high	8.1	8.1		28	10.7
PS	58	M	3	1	17.9	4.2	168	1.58	0.83	99.72	1.3	high		5.7	6	37	9.6
PS	71	M	2	1	23.8	3.9	154	1.69	1.15	128.2	2.1	high	8.7	4.2	0.11	24	9.4
PS	71	F	2	1	21.5	3.7	78	1	0.82	97.06	2.1	high	8.4	5.7	7.36	20	9.5
PS	81	F	2	1	21	3.9	108	1.19	0.88	117.9	2.1	high	7.5	6.2	2.36	31	6.8
PS	62	M	30	1	18.3	3.7	171	1.42	1.16	82.33	1.1	high		2.4	0.39	37	10.2
PS	55	M	27	1	22.7	3.9	127	1.73	0.93	132.3	1.8	high	8.8	7.1	0.91		9.1
PS	68	F	22	1	19.9	3.6	154	1.13	0.99	116.4	1.5	high		5.2	0.12	23	11.4
PS	57	F	21	1	16.8	3.4	210	1.49	0.96	96.18	1.5	high		5	0.02	24	10.6
PS	60	F	20	1	23.4	4.2	174	1.33	1.32	120.4	1.1	high		5.2	0.03	22	10.1
PS	66	M	19	1	19.3	3.9	125	1.7	0.89	119.8	1.5	high		5.9	0.07	31	10.9
PS	52	M	18	1	19.3	3.9	162	1.26	0.61	109.4	2.1	high	8.5	4.1	0.09	26	9.1
PS	57	F	17	1	18.4	4.3	147	1.22	1.31	156.6	2.1	high		6.6	0.03		10.2
PS	71	M	17	1	22.6	4.2	172	1.45	1.4	156.8	2.2	high	9.3	5.5	0.21		6.8
PS	58	F	15	1	16.5			1.25	0.91	96.56	1.5	high		8.9			10.7
PS	58	M	13	1	20	3.5		1.56	0.7	119.7	1.5	high		7.3			10.7
PS	64	M	12	1	20.5	4	162	1	0.61	109.6	2.2	high	10.9	6.3		26	10.8
PS	66	M	12	1	25.1	4	149	1.24	0.83	128.8	2.2	high	10.1	7.8		45	9.4
PS	40	M	12	1	18.1	3.8	143	1.38	1.02	110.8	1.8	high	10.9	4.1		25	14.2
PS	50	M	11	1	23.2	3.9	143	1.12	0.72	102.7	2.2	high	9.6	8.7		28	12.2
PS	55	M	11	1	18.3	3.8		1.23	0.85	124.9	2.1	high		4.7			9.2
PS	64	M	10	1	25.1	3.8	119	1.63	1.1	134	1.4	high	9.4	5.5	0.09	36	11.3
PS	50	M	10	1	21.8	4.3	182	1.36	0.86	126.8	1.4	high	8.6	3.4	0.1	30	9.8

PS	55	F	10	1	20.2	4	179	1.27	0.93	122.9	1.5	high		4		32	11
PS	63	M	10	1	20.5	3.5		1.14	0.89	114.2	2.5	high		5.7			11.2
PS	60	F	10	1	19	3.8		1.79	0.97	136.1	1.8	high		7			12.1
PS	68	F	10	1	20.6	3.7		1.28	1.12	109.7	1.5	high		7.5			11.2
PS	55	M	9	1	21	3.8		1.69	0.71	67.08	1.5	high	8.4	6	0.09	38	10.5
PS	63	M	9	1	23.2	4.6		1.25	0.77	129.9	2.1	high		4.9			10.2
PS	48	M	9	1	24.6	3.9	144	1.75	0.82	135.4	1.5	high	9.7	5.3	0.07	28	11.5
PS	81	F	9	1	19.6	4.1	149	1.44	0.7	115.6	1.5	high	8.6	5.4	0.03	20	12.1
PS	70	M	9	1	18	3.5		1.5	0.89	120	1.8	high		6.2			9.8
PS	79	F	9	1	22.1	3.4	125	0.71	0.95	84.95	1.5	high		3.4	0.05		7.2
PS	52	F	8	1	19.6	3.9	214	1.37	0.9	93.49	1.6	high	9.1	4.4	0.1	17	10.3
PS	54	M	8	1	32.8	4.1	229	1.38	0.99	115.3	2.2	high	10.1	12.1	0.1	17	10.5
PS	58	F	8	1	25.5	4.1		1.7	0.95	143.4	2.2	high	8.1	7.4			11.6
PS	44	M	7	1	19.8	4.5	140	1.18	0.73	125.2	1.5	high	8.8	3	0.1		11.6
PS	58	F	7	1	22.4	4		1.32	0.88	120.8	1.5	high		7.5			11.8
PS	80	F	7	1	28.5	3.8	148	1.1	0.65	110.4	1.6	high	8.4	5.7		30	11.4
PS	27	M	6	1	17.3	4.2	160	1.75	1.25	85.05	1.8	high	9.2	5.3	0.02		10.4
PS	61	F	6	1	24.1	3.5	134	1.16	1.11	95.62	1.5	high		2.9	0.03	19	9.5
PS	26	M	6	1	19.7	4.1	177	1.61	0.81	118.6	1.8	high	8.1	4.4	0.3	36	11.8
PS	63	M	5	1	22.4	3.7	150	1.55	0.85	105	1.8	high	9.5	2.7			
PS	69	M	5	1	22.3	4	161	1.48	1.06	105.7	1.6	high	9.1	3.7	1.9	33	12.8
PS	50	M	5	1	24.7	3.7	175	1.44	0.82	127.2	1.5	high	8.4	4.6	0.19	21	10.9
PS	44	M	4	1	22.5	4.2	116	2.11	0.69	62.51	1.6	high	8	4.5	1.26		10.4
PS	63	M	4	1	23.2	3.8	154	1.67	1.06	121.2	1.8	high	11	5.5			4.8
PS	65	M	4	1	30.8	4.4	149	1.29	0.97	137	1.8	high	10.1	6.1			
PS	60	M	4	1	21.2	4.2	118	1.57	0.83	82.53	1.8	high		7.3	0.12	32	10.2
PS	52	M	4	1	22.6	4	135	1.28	0.74	95.47	2.1	high		5.2	0.21	26	13.6
PS	64	M	4	1	20.3	3.9		1.1	0.86	115.5	1.8	high		9.1			13.4
PS	56	M	4	1	26	4.2	205	1.31	0.56	71.84	2.1	high		5	0.1	27	9.8
PS	64	F	3	1	18.2	3.8	119	1.19	0.98	102.8	2.1	high	9.8	5.3			9.3
PS	54	M	3	1	28.5	3.7	156	1.6	0.72	128.5	1.3	high		5.5	0.1	32	10.9
PS	69	M	3	1	26.9	4.1		0.85	0.81	116.6	1.8	high		7.1			9.8
PS	54	F	3	1	20.4	4.3	130	1.49	0.85	82.42	1.5	high	9.1	4.5	0.27	29	7.8
PS	63	M	3	1	20.1	4.1		1.07	0.77	77.89		high	7.9	4.4			6.5
PS	69	M	3	1	18.1	3.6	149	1.63	0.68	83.8	1.3	high	9	4.6	0.1	28	10
PS	70	M	3	1	23.2	4	193	1.68	0.74	78.65	1.5	high	8.3	2.9	0.39	27	11.9
PS	45	M	3	1	26.3	3.8	147	1.44	0.84	87.92	1.5	high	9	4		26	9.1
PS	36	F	2	1	25.1	4	109	1.3	0.86	105	2.1	high	9.7	4.8	0.03	25	10.6
PS	62	M	2	1	23.3	4.6	146	1.38	0.7	64.95	2.1	high	8.8	5.8	0.04	28	8.1
PS	57	F	2	1	20.8	4	170	1.1	0.81	97.98	2.1	high	9	4.2	0.05	31	10.9
PS	47	F	2	1	23.5	3.3	156	1.06	0.77	86.62	1.8	high	7.5	6.6	0.83	35	9.1
PS	54	M	2	1	24.7	4.4		1.6	0.69	93.23	2.1	high	8.2	6	0.1	32	10
PS	72	F	2	1	24	4	161	1.69	0.76	106.8	2.1	high	8.4	4.1	0.27		9.9
PS	49	F	27	1	23.7	3.8	155	1.82	1.05	122.3	1.4	high	9.2	4.2	1.45	32	
PS	72	M	12	1	21.2	3.5		1.03	1.03	126.1	2.1	high		4.3			9.6
PS	59	M	10	1	20.4	4.1	126	0.69	0.99	85.1	2.1	high	9.2	8.2	0.06	23	10.5
PS	34	M	10	1	23.9	4.2		0.97	1.11	97.42	1.5	high		4.5			11.9
PS	76	F	10	1	20.2	3.6	202	1.57	1.02	106.1	1.5	high	8.8	5.6	0.06	27	7.7
PS	73	M	10	1	19.9	3.6	227	1.38	0.83	134.4	2.1	high	10.1	6.5	0.3	36	5.5
PS	65	F	8	1	22.3	4.1	124	1.06	0.82	126.9	2.1	high	8.4	5.1	0	18	5.1
PS	67	M	6	1	25.3	4.1	112	1.05	0.89	129.6	2.1	high	8.4	4.4	1.4	21	4.9
PS	75	M	4	1	18.2	4.1	148	1.23	0.63	89.5	1.5	high	8.3	7.2		28	11
PS	83	F	3	1	23.7	3.5	160	1.22	0.8	104.3	2.1	high	9.5	4.1	0	21	
PS	75	F	3	1	23.3	3.7	103	1.21	0.96	93.01	2.1	high	8	4.1	0.6	28	5.9
PS	62	M	3	1	21.5	4	153	1.31	0.74	114.1	1.6	high		1.5	0.23	14	9.2
PS	84	F	2	1	16.4	3.8	140	1.86	0.75	10.77	1.8	high	9.6	4.4	0.05	24	10.8
PS	71	M	2	1	21.9	3.6	102	1.37	0.83	127.7	2.1	high		5.8	0.07	27	10.4
PS	52	M	28	1	22.8	3.4	157	1.25	0.91	101.8	2.2	high	9.6	5.7	0.1	32	10.4
PS	52	M	17	1	19.9	3.8	238	1.31	0.93	88.4	1.8	high	8.4	4.9			7.2

PS	56	M	15	1	20.9	3.8	213	1.74	0.81	125.4	1.8	high	10	4.7			
PS	67	M	13	1	21.6	4.1	154	1.05	0.83	136.3	1.3	high	9.3	5.5		23	9.4
PS	65	F	13	1	26.1	4.1	91	1.41	1.4	116	1.8	high	10	4.1			4.7
PS	70	M	11	1	22.6	3.4	159	1.76	1.24	100.7	1.5	high	9.3	5.6	0.02	30	9.5
PS	65	F	9	1	20.5	3.5	185	1.15	1.31	139.3	2.2	high	10.3	6.6			
PS	66	F	5	1	21.3	3.6	178	1.33	0.78	122.1	1.8	high	8.4	6			6.4
PS	73	M	5	1	23.4	4.2	144	2.13	0.78	99.77	1.8	high	9.8	4.4	0.74	25	12.1
PS	45	F	4	1	24.1	4.1	151	1.98	0.6	80.29	2.1	high	8.8	7.2		26	11.8
PS	81	F	4	1	19.3	3.5	171	1.15	0.9	97.52	2.2	high	8.4	4.9			7.3
PS	71	M	3	1	19.7	3.5	116	1.41	1.1	105.3	1.8	high	8.2	4.7			
PS	74	F	2	1	18.7	4.1		1.5	0.91	79.43	1.8	high	9.2	5.6		23	
PS	46	F	2	1	24.2	3.7	177	1.38	0.66	66.34	1.6	high	8.2	3.8	0.1	25	11.4
PS	55	F	2	1	18.5	4.2	170	1.31	0.99	90.85	1.3	high	8.1	5	0.27	29	11.8
PS	68	F	10	1	23.4	4	137	1.9	0.6	87.87	1.3	high	8.5	4.6	0.05	25	11.4
PS	70	F	8	1	26.5	3.7	171	1.13	0.9	123.5	1.8	high	9	5.1	0.04	23	11.9
PS	77	F	6	1	20	3.7	152	1.63	0.82	92.64	1.8	high	10.4	4.1	0.11	31	11.5
PS	86	M	3	1	24.7	3.7	280	1.8	0.78	55.07	1.8	high		9.5	0.89	29	9.5
PS	48	F	2	1	20.2	3.5	101	1.56	1.01	120.2	1.6	high		6	0.05	28	9.8
PS	64	M	26	1	20.5	3	99	1.45	0.9	98.68	1.3	high	9	7.2	0.18	30	10.6
PS	68	F	19	1	15.5	3.2	160	1.76	0.84	68.12	1.5	high	9.5	6.1	0.38	24	10.4
PS	52	M	11	1	16.8	3.4	115	1.09	1.45	127.3	2.2	high	9.4	4.9	0	31	9.4
PS	65	F	10	1	16.4	3.4	105	1.31	1.02	117.1	1.1	high	11.2	5	0.05	28	10.5
PS	76	F	10	1	16.3	3.4	122	1.18	0.83	88.31	2.1	high	9.5	6.6	0.64	30	11.5
PS	44	M	10	1	19.5	3.9	194	1.5	0.92	120.4	1.5	high		6.1	0.16	27	8.6
PS	65	M	5	1	17.6	3.9	192	1.73	0.94	122.6	1.5	high	7.8	4.9	0.22	42	10.2
PS	67	M	4	1	23.3	3.3	107	1.76	0.96	139.4	1.5	high	9.5	2.7			8.9
PS	37	M	3	1	19.6	3.6	155	1.27	0.79	68.04	2.1	high	9.3	8	0.01	23	10.8
PS	45	M	3	1	20.9	3.1	181	2.38	0.8	70.52	1.3	high	8.8	6.5		33	
PS	79	F	2	1	20.8	3.7	206	1.63	0.72	88.83	1.5	high		8.3	0.1	30	11.2
PS	69	M	34	1	19.3	3.6	123	1.47	0.82	103.6	1.8	high	9.6	5.1			
PS	60	M	27	1	23.5	4.1	135	1.55	0.77	92	1	high	9.7	6.1	1.1		9.2
PS	65	M	22	1	14.6	3.9		1.4	1.2	79.52	1.6	high	9.5	3.4	0.28	30	13.3
PS	66	F	22	1	19.7	3.6	168	1.57	1.06	87.84	1.8	high	10.3	8.1		28	10.1
PS	58	M	22	1	22.1	4.1		1.62	0.76	109.3	1.4	high	9.1	4.5	0.81	23	10.9
PS	56	F	20	1	16.5	3.8	157	1.47	0.87	107.1	2.2	high	9.2	5	0.1	32	9.6
PS	39	M	17	1	20.8	4.4	156	1.19	1.06	125.3	1.4	high	9.4	2.1	0.21		11.5
PS	41	F	17	1	17.6	4	109	1.11	0.85	103.8	1.4	high	7.8	5.4		27	10.4
PS	49	M	16	1	16.2	3.5	157	1.09	1.16	115.2	1	high		3.2			10.7
PS	50	M	16	1	21.6	4.1	160	1.52	0.97	143.5	2.2	high	8.9	5	2.6	29	9.1
PS	58	M	16	1	22.1	3.5	119	1.43	0.9	120.6	1.8	high		3.3			11
PS	40	M	16	1	21.3	3.6	139	1.48	0.79	123.2	1.3	high	7.8	4.3	0.34	22	10
PS	35	M	16	1	18	4.3	158	1.08	0.55	100.3	1.5	high	9.7	4.6	0.05	20	11.4
PS	48	M	15	1	21	4.2	160	1.67	1.04	121.7	1.3	high	10.1	5.4	0.03	28	10.7
PS	59	M	14	1	23.9	3.8	166	1.52	0.88	124.6	1.4	high	9.9	6.8	0.11	31	12
PS	68	M	13	1	14.5	4.1	125	1.48	0.78	90.96	1.8	high	9.5	4.5	0.5	32	10.4
PS	49	M	13	1	20.5	3.8	128	1.24	0.77	112.7	1.8	high		7.1	0.05	28	11.2
PS	80	M	12	1	26.8	3.5	178	0.94	0.94	139.1	1.4	high		4.3	0.94		10.9
PS	42	M	10	1	18.8	4.5	116	1.43	0.95	126.7	1	high	8.5	5.9	0.4	35	9.7
PS	50	F	10	1	18.2	4	126	1.39	0.89	138.3	1.5	high	10	7.1	0.3	22	11.9
PS	76	M	10	1	26.1	3.6	117	1.22	0.88	117.8	1	high	8.3	3.1	0.47	25	11.7
PS	59	M	10	1	22.6	4	140	1.61	0.91	136.1	1.8	high	7.2	3.9		31	8.5
PS	83	F	9	1	17.5	3.2	172	1.68	1.18	123	1.4	high	9.8	3.6	0.1		
PS	76	M	9	1	19.4	4	151	1.32	0.86	108.9	1.4	high	9.3	4.1		32	11.8
PS	61	M	9	1	18.3	3.8	192	1.7	1.18	107.5	1.4	high	9.1	8	0.1	45	9.7
PS	65	M	8	1	25.5	4	135	1.75	0.7	119.8	1.4	high	8.4	6.5	0.92	35	11.2
PS	59	F	8	1	27.9	4.1	253	1.64	0.91	143.8	2.2	high	8.7	7.7	0.1	32	9.4
PS	55	M	8	1	20.5	4	147	1.38	0.9	155.1	0.6	high		6.9	0.02	29	9.8
PS	78	M	8	1	24.9	4.1	150	1.15	1.06	142	1.8	high	9.9	7.8	0.23	34	12.2
PS	53	M	8	1	22.8	4.1	140	1.69	0.97	129.4	1.5	high	10.2	6.5		23	9.6

PS	65	F	7	1	22.4	3.7	206	1.51	0.79	92.26	1.8	high	9.6	6.4		21	11.3
PS	54	M	7	1	24	4.1	97	1.01	0.99	160.3	1.5	high	8.9	8.1	0.58	36	10.2
PS	50	M	7	1	19.4	3.7	127	1.1	0.87	109.3	1.4	high	9.2	4.9	0.13	32	10.7
PS	63	F	7	1	17.2	4.3	137	1.25	0.8	100.9	1.8	high	10	5.7	0.08	30	10.6
PS	62	M	6	1	20.1	4.4	92	1.41	1.21	118.2	1.8	high	9	4.7	0.5	25	10.6
PS	40	M	6	1	21.5	3.8	176	1.6	0.77	124.6	1.4	high		5.2	0.38		10.9
PS	55	M	6	1	20	4.1	187	0.75	1.15	142.4	1.8	high		7.5	0.6	25	8.1
PS	36	M	5	1	19.6	4.2	146	1.65	0.85	121.2	1.3	high	8.7	4.2	1.48	26	10.1
PS	73	M	5	1	19.6	4.2	162	1.36	1.06	142.8	1.8	high		5.9	0.14	24	9.6
PS	49	M	4	1	23.4	3.7	154	1.13	1.02	119.8	1.8	high	9.2	6		26	13.4
PS	60	M	4	1	23.4	4	99	1.39	0.86	112.8	1.8	high	8.6	6.3			11.2
PS	69	M	4	1	18.4	3.7	203	1.63	0.83	70.3	1.8	high	10.2	4.6			11.3
PS	60	M	4	1	20.8	4	106	1.58	0.96	154.6	1.1	high	8.5	4.1	0.17	26	12.1
PS	54	F	4	1	30.5	4.2	140	1.26	1.13	114.4	1.5	high	8.6	3.4	0.1	23	9.6
PS	56	M	4	1	21.1	3.5	153	1.75	0.98	88.13	1.8	high	9.3	4.7			11
PS	49	M	3	1	21.1	4.3	132	1.25	0.92	152.6	1	high	9	5.1		30	
PS	42	M	2	1	18.2	4.5	157	1.93	0.83	94.54	1.5	high	9.8	8.4	0.12	37	12.1
PS	50	M	2	1	26.3	4.4	171	1.6	0.93	96.65	1.8	high	9.3	5		20	9.6
PS	53	M	2	1	25.4	4	112	1.14	0.83	87.47	2.1	high		4.5	0.35	28	10.8
PS	39	F	17	1	18.5	4.4		1.54	0.91	119.4	2.1	high	9.2	5.2		26	
PS	36	M	9	1	21.6	3.8	185	1.59	0.63	82.37	2.1	high		5.3			9.7
PS	59	F	6	1	16.3	3.9	172	0.91	1.17	99.71	1.3	high		4.6	0.01	23	10.8
PS	50	M	2	1	29.4	3.5	143	1.92	0.71	84.59	1.8	high	9.3	5.4		23	11.6
PS	39	F	9	1	18.5	3.5	120	1.52	0.9	85.08	1.6	high	9.9	6	0.14	24	10.7
PS	55	F	4	1	20.8	3.5	172	1.67	0.87	118.8	2.1	high	8.9	3.9	0	26	9.6
PS	72	M	9	1	17.8	3.3	151	1.33	1.1	128	1.5	high	8.1	5.8	0.16	26	10.4
PS	53	F	4	1	15	3.8	216	1.57	0.7	81.53	1.5	high	8.4	5.3	0.2	26	10.5
PS	46	M	6	1	21.3	3.9	180	1.15	0.8	94.62	1.8	high	8.7	4.9			10.9
PS	51	F	16	1	20.5	3.5	173	1.58	0.69	127.5	1.4	high		5.6	0.24	34	9.9
PS	58	F	10	1	19.1	3.9	134	1.23	0.98	108.9	1.5	high	9.3	4.6	0.32	30	10.2
PS	48	F	13	1	19.2	4	163	1.6	1.11	132.3	2.2	high	8.7	4.8	0.1	25	9.2
PS	60	M	6	1	16.9	4	179	1.63	0.91	113.1	1.5	high		3.8	0.1	25	11.8
PS	73	F	6	1	26.3	3.4	135	0.95	0.93	113.6	1.3	high		7.7	0.45	33	11.2
PS	64	F	5	1	19.4	3.8	135	1.26	0.8	92.05	1.8	high	9.2	5.8		25	11.7
PS	77	M	8	1	21.3	3.6	148	1.36	0.98	100.9	1.5	high	8.8	3.5	0	19	11.2
PS	65	M	4	1	27.8	3.7	149	1.74	0.68	59.53	1.5	high		6.9	0.1	26	9
PS	52	F	24	1	20.3	3.7	128	1.63	0.9	128.3	1.4	high		5.9	0.05	35	10.4
PS	68	M	5	1	21.4	2.8	143	1.31	0.69	123	1.8	high	8.9	4	0	22	11
PS	64	F	13	1	18.8	4	127	1.2	1.02	121.3	1.6	high	8	3.3			8.6
PS	65	M	3	1	24.5	3.7	167	1.47	0.77	103.7	2.1	high	9.3	6.2	0.01	32	11.2
PS	41	F	24	1	19.2	3.9	175	1.46	0.87	123	2.1	high	10.1	7	0.07	29	10.1
PS	72	M	3	1	21.3	3.8	225	1.09	0.6	65.06	1.6	high	9.7	5.4			10
PS	61	M	10	1	21.6	3.9	184	1.55	0.81	98.4	2.2	high	9.1	4.2	0	26	10.2
PS	70	M	21	1	25.5	3.5	120	1.21	0.71	93.82	2.2	high	9.1	6	0.1	27	10.4
PS	65	M	13	1	21.4	3.9	149	2.29	0.89	150.3	2.2	high	8.9	4.3	0.1	34	9.1
PS	54	F	4	1	19.7		171	2.02	0.94	60.81	1.8	high	9.4	4			10.2
PS	75	M	3	1	24.4	3.6	134	1.56	1.07	124.1	2.2	high	10	5.5	0.2	35	11.1
PS	57	M	4	1	22.2	4	138	1.18	0.75	84.14	2.1	high		6.3	0.1	27	12
PS	67	M	24	1	20.4	3.7	208	1.75	0.78	109.3	2.2	high	10.1	4	0	32	10.1
PS	63	M	23	1	22.6	3.5	131	1.23	0.68	116.5	1.4	high		5.3	0.05	25	9.4
PS	58	F	18	1	26	3.9	156	1.12	0.92	133	2.2	high	8.8	4.6	0	27	9.8
PS	64	F	11	1	25.2	3.7	159	0.99	0.81	90.09	2.1	high		6.4	0.1	24	9.7
PS	57	M	32	1	20.1	3.8	126	1.14	1.06	110.5	1.8	high		6.4	0.06	23	8.7
PS	67	F	13	1	17.4	3.6	123	1.22	0.95	131.3	1.8	high		5.6	0.09	21	9.4
PS	74	M	7	1	25.5	3.5	210	1.29	0.79	151.7	2.2	high	9.1	4	0	22	9.9
PS	72	M	28	1	23.2	3.4	158	1.1	0.77	88.05	1.8	high		7.1	0.05	32	10.9
PS	39	F	2	1	26.3	3.7	212	1.9	0.85	94.51	1.5	high		5.7	0.1	26	9.6
PS	50	F	7	1	19.7	4.2	187	1.49	0.93	106.1	2.2	high	8.9	4.9	0	23	9.8
PS	61	F	7	1	18.5	3.7	135	1.32	0.87	121.1	1.8	high		7	0	21	11

PS	57	F	12	1	18.9	3.6	206	1.34	1.03	110.6	2.2	high	8.5	4.4	0.6	24	10.7
PS	57	F	5	1	23.9	4.1	105	1.59	0.78	101.9	1.8	high	9.2	4.3		27	10.6
PS	65	F	4	1	22	4	184	1.8	0.88	104.8	2.2	high	8.3	7.3	0.1	29	10.7
PS	39	F	9	1	19.8	4	147	1.31	0.83	114.1	1.3	high		6.8	0	23	10.1
PS	58	F	23	1	19.9	3.6	120	1.17	0.92	128.3	1.8	high		5.2	0.05	20	9.9
PS	61	F	32	1	22.8	3.5	170	1.43	1.14	87.25	1.8	high		9	0.1	30	8.8
PS	62	M	2	1	21.4	3.7	177	1.05	0.82	93.67	1.8	high		7.2	1.1	47	9.3
PS	56	F	3	1	26	3.7	197	1.07	0.94	124.2	2.1	high		7.9	0.6	28	9.9
PS	60	M	10	1	24.8	4	188	1.38	0.61	96.36	1.5	high		5.6	0.1	27	10.4
PS	55	M	3	1	22.9	4	202	1.21	0.85	95.74	2.1	high		8.9	0.62	28	10.6
PS	52	F	31	1	23.6	3.5	223	1.58	0.9	115.4	2.2	high	8.2	6.2	0.1	28	11.7
PS	70	F	5	1	19.1	3.4	141	1.18	1.19	86.97	2.2	high	9.4	6.5	0.1	26	10.2
PS	54	M	16	1	24.9	4.4	115	1.05	0.88	100.3	1.8	high	9.6	7.7		24	10.5
PS	75	M	12	1	20.4	3.6	169	1.2	0.8	147.5	2.1	high	9.4	5.2		31	10.7
PS	66	F	11	1	21.3	3.7		1.6	1.21	132.8	1.8	high		4.4	0.81	40	8.2
PS	78	F	11	1	23.1	3.6	155	1.11	1.05	113.6	2.2	high	10	5.7		25	11.1
PS	64	F	9	1	24.1	3.6	160	1.52	0.95	112.7	1.5	high		4.7	0.05		12.2
PS	56	F	9	1	29	3.5	181	0.94	0.86	84.41	2.1	high		6.2	0	38	10.7
PS	70	M	6	1	26.2	2.7	287	1.01	0.95	132.2	2.1	high	9.1	7.2	0.98	23	11
PS	46	M	6	1	28	3.7	114	1.07	0.91	96.81	2.1	high	9.9	6.5			12.8
PS	30	M	6	1	20.3	4.3	150	1.6	0.99	122.2	1.8	high	8.6	4.1	0.2	12	10.5
PS	79	F	4	1	18.6	3.3	126	0.9	1.1	135.4	1.8	high	9.7	4.8			11.6
PS	58	M	3	1	20.3	3.5	95	1.83	0.69	66.7	1.3	high	9	3.3	0.35	44	8.5
PS	67	F	3	1	22.6	3.7	156	1.21	0.98	125.5	2.5	high	8.6	4.4	1.2	24	8.6
PS	70	F	3	1	29.5	3.5	115	1.57	0.65	109.7	1.3	high	7.6	3.6	0.05	22	10.8
PS	62	M	3	1	21.1	3.7	185	1.37	1.24	116.2	1.5	high	8.9	4.5	0.08	48	10.8
PS	71	M	2	1	25.6	3.6		1.1	0.93	132.9	1.3	high		4.3	1.96	29	9.6
PS	61	M	2	1	25.8	3.5	162	1.65	0.87	71.66	1.8	high		3.8	0.28	29	10
PS	66	M	2	1	18.4	3.7	226	1.54	0.89	74.58	1.5	high		5.4	0.1	23	11.3
PS	77	F	2	1	23.5	3.6		1.68	1.02	122.5	1.5	high	8.9	3.7	0.3	22	9.2
PS	74	F	25	1	20.1	3.6		1.82	0.94	143	1.5	high	8.9	4.1	0.05	14	10
PS	76	F	13	1	21.3	3.4	169	1.58	0.81	116.2	1	high	8.4	4.6	0.9	34	7.6
PS	56	M	4	1	22.6	3.6		1.8	0.82	73.08	1.8	high	9	4.3	0.2	25	10.6
PS	60	M	3	1	19.6	4	108	1.48	0.67	73.31	1.3	high	10.1	5.9	0.09	46	11.5
PS	66	F	13	1	19.6	3.7	126	0.98	0.97	129.1	2.1	high		4.8		29	9.1
PS	75	F	16	1	16.2	3.7	164	1.62	1	120.7	2.1	high	8.1	9.6	0.1	32	12.3
PS	50	F	27	1	20.6	3.9	232	1.23	0.69	80.26	1.8	high	7.5	4.8	0.1	27	10.4
PS	44	M	30	1	19.7	3.9	128	1.12	1.27	94.45	1.8	high	10	4.7	0.09	31	12.3
PS	49	M	3	1	27.2	3.9		1.31	0.8	42.73	1.8	high		2.1			9.2
PS	73	F	35	1	19.3	4		2.02	0.75	112.4	2.2	high	9.7	7.6	0.15	32	10.3
PS	51	M	2	1	33.1	3.8	149	0.99	0.9	98.82	2.1	high	8.9	4.7	8.53	30	6.2
PS	59	F	11	1	22.6	3.8	128	0.99	1.07	103.7	2.1	high	8.8	2.9	0.09	25	10.3
PS	82	F	2	1	24.1	3.7	123	1.73	0.93	119.6	1.8	high	8.9	5.5	0.1	24	10.4
PS	58	F	19	1	23.3	4.1	101	1.46	1.28	123.6	1.8	high		5.3	0.11	31	12.1
PS	67	M	14	1	25.1	4.1	128	1.29	0.92	129.1	2.2	high	9.6	6.3	0	27	11.7
PS	59	F	5	1	19.4	4.7	55	1.72	0.83	77.23	1.5	high	9.9	6.1	0.05	33	8.3
PS	71	F	12	1	25.2	3.8	202	1.19	0.8	135.2	2.2	high	9	10.5		19	9.1
PS	52	F	7	1	17.4	4.1	130	1.06	1.13	116.5	2.2	high	8.8	9.7		17	9.9
PS	69	M	6	1	22.7	3.4	155	0.98	0.99	145	2.2	high	7.4	5.6		18	9.4
PS	36	M	8	1	21	4.3	179	1.42	0.76	107.3	1	high	8.8	5.7	0.5	27	11.7
PS	44	M	8	1	20.1	4.6	199	1.67	1.17	83.42	1.5	high		4.6	0.1	28	12.7
PS	59	M	10	1	23	3.8	142	1.12	1.3	106.7	2.1	high	8.8	4.6	0.5	32	10.2
PS	68	M	2	1	21.2	3.3	190	1.21	0.56	84.48	1.5	high	9	4.8	0.09	20	9.9
PS	57	F	19	1	18.1	4	226	1.69	1.36	110.7	1.3	high	9.3	6.2	0.07	26	11.7
PS	61	F	8	1	22.3	4.1	195	1.44	0.88	129.3	1.4	high		6.6	0.05	20	10.7
PS	63	M	5	1	19.8	3.8	170	1.58	0.8	103.9	1.5	high	8.3	5.8	0.5	30	10.1
PS	60	M	4	1	20.4	4	230	1.42	0.72	98.92	1.5	high	9.4	4.4	0		11.1
PS	83	F	13	1	17.5	3.4	111	1.3	0.72	86.09	1.3	high	7.4	2.8	0.05	41	9.3
PS	41	M	13	1	17.1	3.6	161	1.33	0.85	84.73	1.5	high	9.4	4.2	0.08	26	11.9

PS	74	M	7	1	18.8	4.1	113	1.5	1.13	94.64	1.8	high	10.4	5.3	0.15	25	10.6
PS	54	M	5	1	17.1	3.7	188	1.24	0.92	82.25	1.8	high	8.7	5.5	0.09	28	10.3
PS	48	M	5	1	15.9	3.9	141	1.73	1.08	122.1	1.5	high	9.6	5.6	0.12	27	11
PS	52	F	4	1	16.2	3.7	111	1.6	0.89	122.7	1.8	high	9.3	5.2	0.02	60	8.7
PS	71	F	4	1	16.7	3.1	112	1.21	1.04	54.13	1.3	high	9.6	6.4	0.34	28	10.6
PS	69	M	3	1	19.8	4	202	1.56	0.96	114.2	1.5	high	9.1	6	0.06	25	12.3
PS	44	F	2	1	18.5	4.4	231	1.52	0.86	52.35	1.6	high	9.9	4.9	0.3	24	9.6
PS	55	M	2	1	23.6	4.5	152	1.87	1.01	112.2	1.5	high	10	9.3	0.19	34	9.2
PS	85	F	2	1	18.8	3.5	163	1.22	0.98	93.54	1.5	high	9.3	3.6	0.09	38	11.4
PS	60	M	25	1	21	3.4		1.64	1.07	78.16	1.8	high	9.1	4.9		22	
PS	45	F	18	1	17.4	3.1	136	1.4	0.73	88.14	1.8	high	8.9	7.2	0.05	25	11.8
PS	48	F	6	1	26.2	3.5	159	1.68	0.88	71.27	1.8	high	8.3	7.6			10.3
PS	59	M	5	1	20.3	3.7	160	1.54	0.66	106.9	1.5	high	8.5	3.5	0.38	37	11.6
PS	60	F	3	1	20.1	3.8	131	1.05	1.06	95.78	0.6	high	9.6	6.2	1.37	31	8.3
PS	75	M	3	1	18.7	3	155	1.08	0.72	100.9	2.5	high	9.3	7.9	1.1	30	10.4
PS	67	F	7	1	21.4			1.68	0.88	94.05	1.6	high	8.1	3.7	0.2	23	7.2
PS	40	M	22	1	20		146	1.48	1.38	109.4	1.3	high	7.9	4.5	0.3	27	10.5
PS	62	M	3	1	23.4		101	1.31	0.79	85.64	1.8	high		7	0.02	29	11.1
PS	71	M	5	1	20.3		85	1.41	0.97	78.21	2.1	high	10.1	7.1	0.7	35	10.4
PS	61	F	28	1	19.4	3.7	176	0.99	1.19	90.85	2.2	high	9.8	6.4	0.21	20	9.4
PS	51	M	22	1	19.3	4.1	215	1.26	1.21	109.9	1.4	high	9.4	6.6	0.13	31	11.6
PS	66	M	20	1	18.1	3.5	139	1.09	0.69	74.07	1.6	high	9.6	6.9	0.03		7.7
PS	44	M	19	1	21.4	4.4	133	1.26	0.88	105.2	1.6	high	8.1	6.9	0.1		9.6
PS	47	M	19	1	17.5	4	160	1.04	0.92	104.7	2.2	high		5.3	0.1	17	10.6
PS	62	F	18	1	19.5	4.3	166	1.45	0.76	117.2	2.2	high	9.5	7.2	0	28	11.9
PS	48	F	18	1	17.3	4.2	137	1.35	1.03	123.2	2.2	high	10.2	6.8	0.1	32	11
PS	65	M	17	1	26.5	4.1	143	1.11	1.07	104.5	0.8	high	8.4	4.9	1.87	41	8.6
PS	60	F	15	1	26.8	3.7	186	1	0.78	109.7	1.4	high	12.1	6.4	0.67	35	
PS	61	M	15	1	18.7	3.9	149	1.56	0.74	135.2	2.2	high	9.6	4.2		31	10.5
PS	49	M	14	1	21.8	4.4	168	1.29	1.11	84.5	2.2	high	9	6.5	0.05	26	12
PS	59	M	12	1	33.5	4	164	1.71	0.92	99.99	1.5	high	8.8	6.3	0.01	24	11.3
PS	38	M	11	1	18.8	4.3	116	1.78	1.23	115.3	1.5	high	9.2	5.9	0.09	26	10.7
PS	75	M	10	1	22.3	4.3	151	1.58	1.05	119.9	1.5	high		9.6	0	22	10.1
PS	26	M	8	1	17.3	4.2	167	1.44	1.27	97.16	2.1	high	8.9	4.7	0.08	31	10.8
PS	49	M	5	1	18.2	4.4	201	1.57	0.97	129.3	1.8	high	8.8	4.3	0.1	24	11.3
PS	56	F	3	1	18.6	4.2	216	1.29	1.12	132.6	1.5	high		4.8	0.1	19	11
PS	71	M	3	1	20.2	3.7	195	1	1.37	75.32	1.8	high	9.2	5.9	0.2	13	11.2
PS	79	F	10	1	23.6	3.6	163	1.08	0.92	143.6	2.1	high	9.1	4.2	0.16	30	9.6
PS	51	M	8	1	20.9		192	1.48	1.15	111.4	1.5	high	7.7	3.2	0.1	25	8.6
PS	65	M	3	1	19.8	3.8	111	1.41	0.59	53.33	1.8	high		6	0.1	27	9.6
PS	74	M	4	1	21.5	4.1	173	1.5	0.98	90.91	1.8	high		8.6			9.5
PS	55	F	9	1	18.2	4	137	1.23	0.99	112.2	1.8	high	8.5	5.3			11.6
PS	69	F	21	1	22.2	3.6	133	1.35	0.99	106.3	1.8	high	8.7	4.6	0.6	30	9.4
PS	70	F	11	1	21.3	3.6		1.71	0.84	112	2.2	high		5.5			
PS	61	M	8	1	21.3	4	188	1.92	0.88	122.8	2.1	high	8.7	5.5	0.04	26	10.1
PS	45	M	7	1	26.4	3.3	169	1.12	0.97	80.9	1.5	high		5.2		21	10.3
PS	63	F	22	1	21.6	4	187	1.47	0.76	121.3	1.6	high	8.4	5.2			10.8
PS	75	F	23	1	17	3.4	110	1.09	0.91	100.1	1.5	high	9.6	2.4	1.58	27	8.2
PS	62	F	7	1	18.1	3.7	182	1.83	0.88	96.26	1	high	10.9	5.4	0.02	29	10
PS	61	M	13	1	20.6	4	178	0.88	1.09	128.3	1.5	high		4.8	0.99	13	8.7
PS	68	M	4	1	20.2	3.4	84	1.56	0.8	83.72	2.1	high	9.1	4.1	0.04	38	9.1
PS	58	F	7	1	27.4	3.4	132	1.6	0.82	125.1	1.5	high	8.2	4.2	0.6	24	10.6
PS	72	M	6	1	23.5	3.8	127	1.66	1.02	144.3	2.2	high	8.6	3.4	0.08	19	11.3
PS	82	F	2	1	20.4	3.8	139	1.26	0.93	49.22	1.8	high	8.7	3.9			10.2
PS	43	F	8	1	20.3	3.7	175	1.15	1.12	109.9	1.5	high	8.6	6.4	0.08	28	11.5
PS	59	F	13	1	21.3	4.2	184	1.71	1.05	119.1	1.6	high	8.8	3	0.05		10.2
PS	62	M	7	1	26.8	4.2	169	1.08	0.76	106.2	2.1	high		5.4	0.1	13	11
PS	60	F	15	1	18.5	3.7	134	1.67	0.95	138.4	2.2	high	8.7	3.4	0.14		7.8
PS	62	M	4	1	22.2	3.7	154	1.77	0.78	82.5	1.8	high	9.4	5.6			12

PS	72	M	6	1	20	3.6	125	1.57	0.96	117.4	1.5	high	8.7	4.1	1.6	37	9.1
PS	76	F	27	1	20.4	3.5	122	1.3	1.18	116.2	1.4	high	8.2	3.7	0.22		11.9
PS	60	F	2	1	18.3	3.7	131	1.2	0.99	56.2	1.8	high	8.5	8.9	0.05	36	10.6
PS	74	M	7	1	21.9	3.7	129	1.4	0.84	114.2	1.6	high	9.4	6.9	0.08	27	12
PS	61	F	6	1	18.4	3.9	150	1.5	1.09	128.4	1.8	high	9.2	3.9	0.1	32	10.5
PS	53	F	17	1	26.1	4	240	1.2	0.95	103.2	1.8	high	7.8	4.2	0.13	20	11.8
PS	78	F	12	1	15.9	3.6	144	1.27	0.87	127.8	1.5	high	10.9	4.1	0.12	33	11.3
PS	83	F	12	1	16.1	3.7	119	1.2	0.94	67.05	1.3	high		4.1	0.2	40	10.4
PS	57	F	11	1	26.2	3.8	116	1.43	0.72	123.9	1.8	high	9.6	6.1	0.05	19	11.9
PS	67	F	8	1	20.5	4.2	149	1.29	1.12	103.6	1.5	high	10.3	7.3	0.02	26	11
PS	63	F	6	1	20.9	3.8		1.28	0.91	137.1	1.1	high	10.3	8.6			10.9
PS	74	F	6	1	23.6	3.5	155	1.7	1.15	95.84	1.5	high	10.3	6.2	0.02	27	10.9
PS	78	F	5	1	24.3	3.1	135	1.36	0.74	87.29	1.5	high	9.5	5.3	0.1	11	13.2
PS	83	M	3	1	21.9	4	157	1.32	0.93	112.2	1.8	high	8	4.5	0.35	23	13.7
PS	71	M	31	1	23.9	3.3	112	1.34	0.91	115.3	1.6	high		6.4	1.8	30	10.4
PS	42	M	26	1	20.9		119	1.58	1.19	106.6	2.2	high	9.2	3.5	0.11	19	11.1
PS	69	M	17	1	18.2	3.8	124	1.03	1.2	117.4	2.1	high	9.6	8.1	0.1	29	10.2
PS	59	M	13	1	20.4	3.5	138	0.97	0.83	98.26	2.1	high	8.5	8.6	0.1	26	9.5
PS	40	M	11	1	30.6	4.1	202	1.32	0.83	128.7	2	high		5.7	0.05	28	11.5
PS	59	M	19	1	17.6	3.9	166	1.6	0.96	105.6	1.8	high	8.9	4.3	0.14		11.3
PS	64	M	6	1	24.5	4.1	201	1.23	0.99	100.4	1.6	high		4.1	0.11	27	10.2
PS	62	F	6	1	17.5	3.8	130	1.29	0.81	111.4	2.1	high	8.8	4.3	0.1	28	8.9
PS	38	F	5	1	32.6	3.5	118	2.02	0.86	106.6	1.8	high	9.6	5.1		26	11.8
PS	47	M	5	1	24.5		130	1.51	0.78	101.3	2.1	high	7.2	2.1		25	10.6
PS	60	F	4	1	22.2	3.8	133	1.13	0.95	78.83	1.5	high	8.3	2.4	0.55	34	11.8
PS	37	M	4	1	30.8	4.1	151	1.67	0.8	98.38	1.8	high	7.2	9.5	0.16		11.3
PS	59	M	4	1	23.7	4.7	195	1.82	0.8	101.5	2.1	high	9.2	5.1	0.93		10.1
PS	70	M	4	1	28.4	4	178	1.79	0.89	97.99	1.8	high	8.6	4	0.2	26	8.7
PS	74	M	5	1	20.1	3.9	202	1.27	0.99	98.19	1.5	high	9.4	6.2		25	11.5
PS	65	F	16	1	18.3	4.2	195	1.28	1.12	104.1	2.2	high		4	0.07	23	9.7
PS	63	F	12	1	16.6	3.8	191	1.34	0.83	127.8	2.1	high	9.7	6.3	0.1	28	11.2
PS	61	M	21	1	20.4	3.4	132	1.26	0.82	104.9	1.4	high	8.9	8.3	0	13	
PS	58	M	8	1	21.1	3.8	113	1.25	0.96	114.5	2.2	high	7.9	7	0.05	21	10.1
PS	56	F	9	1	18.2	3.6	150	1.47	1	122.7	1.5	high	9.7	4.5	0.08	29	11.2
PS	60	M	9	1	20.9	3.3	181	1.41	0.91	84.12	1.8	high	8.2	2.6	0.05	17	10.2
PS	40	F	19	1	17.2	4.1	219	1.77	0.89	107.7	1.3	high	9.4	5.7	0	28	11.6
PS	72	F	5	1	20.6	3.6	168	1.43	1.22	105.2	1.8	high	9.5	3.3	0.06	20	12.2
PS	36	M	13	1	26.4	3.8	126	2.1	0.88	128.6	1.8	high	8.4	4.7	0.05	29	11.5
PS	72	M	3	1	23.6	3.8	140	1.11	1	101.8	2.1	high	9.7	4.5			9.5
PS	84	M	2	1	23.1	3.8	149	1.36	0.77	68.6	1.1	high	8.8	4.7	0.1	31	10.3
PS	69	F	5	1	20.2	3.9	146	1.01	0.9	117.3	2.1	high	9.7	5.3		29	12
PS	57	M	2	1	14.3	4.2	192	2.02	0.81	108.2	1.8	high	8.9	4.8	0.1	26	10.9
PS	67	F	4	1	19.2	4	169	1.73	1.07	139.4	2	high		5.6	0.34	30	10.4
PS	89	M	4	1	18.4	3.2	181	1.85	0.76	109.2	1.3	high	8.8	4.4	0.03	24	11.2
PS	78	M	2	1	17.9	3.4	227	1.32	0.61	85.8	2.1	high		5.4		15	11.9
PS	80	M	7	1	19.8	4.6	162	1.85	1	124.1	1.3	high	8.9	5.3	0.41	27	10.5
PS	52	F	18	1	20.8	3.9	174	1.09	0.94	104.3	1.3	high		4.1	0.3	29	9.5
PS	59	F	4	1	17.2	3.7	119	1.6	0.65	102	2	high		5.8	0.06	32	10.6
PS	38	F	5	1	20.8	3.9	131	1.4	0.88	72.13	2	high		6.2	0.02	37	10.5
PS	45	M	13	1	20.5	4.8	197	2	0.7	106.3	1.8	high	9.9	6.5	0.2	34	12.1
PS	66	M	8	1	24.2	4.7	169	1.51	0.75	107.7	1.6	high		5.1	0.03	27	10.2
PS	74	M	15	1	17.1	3.5	234	1.22	1.35	106.7	1.5	high	6.5	6.7	1.42	25	7.9
PS	68	F	8	1	26.3	3.6	193	1.4	0.74	97.15	1.1	high	8.8	4.3	0.1	20	10
PS	70	M	23	1	21.4	3.9	167	1.37	0.73	77.77	2.1	high	9.2	3.6	0.06	2	9
PS	72	F	2	1	19	3.5	177	1.88	0.91	121.3	0.8	high	9.5	3.4	0.07	26	9.6
PS	59	M	34	1	20.1	3.8	153	1.67	1.12	118.4	1.4	high	8	2.4	0.29		7.7
PS	59	M	27	1	21.2	3.8	140	1.36	0.87	145.9	1.6	high	10.8	7.3	0.05		7.9
PS	76	F	23	1	20.4	3.8	157	0.96	0.9	110	1.4	high		6.2	0.05	22	8
PS	66	M	18	1	21.1	4	148	1.32	0.93	126.3	1.4	high	9	8		25	9.2

PS	54	F	16	1	24.2	3.7	154	1.01	1.27	97.81	2.1	high	8.1	6.1	0.79		11.2
PS	65	M	16	1	17.1	4.7	245	1.35	0.9	69.44	1.5	high	10.3	7.1	2.45	28	8.5
PS	71	F	13	1	22.1	3.6	179	1.36	0.95	120.5	1.8	high	10.4	5.4			12.4
PS	71	F	11	1	20.3	4	154	1.41	0.91	124.9	1.8	high	8.9	7.1	0.2	35	11
PS	58	F	8	1	19	3.8	159	1.24	0.8	113.7	2.1	high	8.3	7.1	0.71	28	11
PS	53	M	4	1	23.5	4	119	1.47	0.7	74.53	1.8	high	8.2	4.7		24	9.3
PS	73	F	3	1	26.2	4.1	120	1.7	0.9	106.2	1.3	high	8.7	5.1	4.46	36	9.8
PS	56	M	2	1	24.2	3	171	1.74	0.93	96.17	1.5	high	8.8	4.8	0.72	29	10.5
PS	68	M	2	1	19.7	4.2	139	1.29	0.86	149.4	1.5	high	10	6.2	0.03	34	9.9
PS	47	M	2	1	22.9	4.3	143	0.77	0.64	27.48	1.1	high	10.1	2.2	0.1	22	9.1
PS	61	F	2	1	37	3.5	104	1.37	0.78	82.27	1.3	high	7.8	4.8	0.19	23	10.9
PS	70	M	7	1	17.8	3.5	160	1.07	1.35	115.7	1.8	high	10.7	5.1	0.1	31	9.9
PS	50	M	8	1	21.8	3.7	194	1.47	1.04	105.1	1.5	high	8.9	5.9	0.87	19	10.3
PS	57	M	2	1	23.9	3.9	137	1.42	0.82	133.7	1.3	high	9.7	4.7	0.08	36	9.5
PS	64	F	13	1	19.9	3	103	1.51	0.41	70.48	2.2	high	8.7	4.8	0.05	24	12.1
PS	43	M	11	1	25.9	4	131	1.08	1.17	125.3	1.3	high	10.3	4.7	0.24	27	8.5
PS	67	M	8	1	20.4	4	209	1.04	0.93	111.7	1.3	high	9.4	4	0.12	28	8.9
PS	54	F	12	1	18.7	4.1	201	1.74	0.92	135.2	1.3	high	9.9	5.1	0.42	26	11.8
PS	67	M	7	1	20.5	3.7	144	1.45	1.06	91	1.8	high		4.3		16	9
PS	67	F	21	1	25.3	3.6	121	1.05	0.88	111.5	1.5	high	9.5	4.3	0.12	38	9.9
PS	67	M	3	1	20.5	3.9	149	1.14	1.1	100.7	1.3	high	9.1	4.6	0.04	32	8.5
PS	59	M	2	1	22.4	4.5	181	1.12	0.66	83.66	1.8	high	9	4.7		38	10.2
PS	63	F	19	1	18.3	3.7	156	1.64	1.08	128.1	1.8	high		5.1	0.12		10.8
PS	63	M	18	1	22.1	4	138	1.23	0.99	127.3	1.8	high	8	3.8	0.9	29	9.8
PS	62	F	16	1	19	3.9	183	1.42	0.91	132.2	1.8	high		6	0.12		10.5
PS	69	F	16	1	17.5	4.3	155	1.65	0.52	97.65	1.3	high		5.3	0.06		10.2
PS	56	M	14	1	23.1	3.7	176	1.68	1.03	115.2	1.3	high	8.9	3.4	0.1	30	10.2
PS	36	F	7	1	19.8	4.1	104	1.26	1.22	105.4	1.8	high	8.7	6.1	0.07	31	11.4
PS	74	M	7	1	21.8	4	141	1.86	0.84	155.5	1.5	high	10	5.8	0.02	27	10.5
PS	55	F	6	1	18.5	4.2	127	1.38	0.83	92.66	2.1	high		6			8.7
PS	49	M	6	1	24	3.9	134	1.59	0.53	6.56	2.1	high	8.3	5.8	0.22	29	10.7
PS	56	F	6	1	17.4	4.3	187	1.81	0.99	81.79	2.2	high	7.9	3.6	0.05	19	10.8
PS	34	F	6	1	22.7	3.7		1.13	0.83	116	1.8	high	9.5	6.2	0.08	18	11.6
PS	49	F	6	1	17.8	4		1.07	0.66	68.33	1.5	high	8	3.8	0.74	26	11.7
PS	61	M	5	1	20.1	3.4	143	1.46	0.97	81.02	2.1	high	10.2	4.8	0.03	26	10.8
PS	58	F	5	1	15.5	3.9	105	1.03	1.23	98.63	2.2	high		7.3	0.02	23	10.9
PS	58	F	5	1	17.1	4.4	135	1.52	0.72	125.7	1.8	high	9	4.9	0.4	26	8.6
PS	75	F	5	1	23.5	3.8	196	1.83	0.82	109.4	1.8	high	9.4	4.9		29	9
PS	69	M	4	1	19.2	3.6	148	1.27	1.13	125.3	2.2	high		9.2		0	8.6
PS	69	M	4	1	21	3.8	141	1.23	0.82	92.86	1.5	high	8.6	4.1	8.3	21	10.9
PS	32	F	3	1	15.6	4.1	116	0.99	1.14	140.9	1.8	high		6.3	0.05	41	10.7
PS	59	M	3	1	21.3	3.4	147	1.16	0.88	91.18	2.1	high	9.3	4.5	1.38	17	11.5
PS	81	M	3	1	26.8	3.8	163	1.69	1.11	126.6	2.2	high		4.2	0.9	28	11
PS	61	M	3	1	21.8	4.5	158	1.11	0.85	106.8	2.2	high		4.7	4.2	20	9.8
PS	70	M	3	1	24.8	3.9	92	1.34	0.7	61.96	1.8	high	9	6.2	0.06	38	8.2
PS	78	M	2	1	20.7	4	192	1.85	0.95	64.22	2.1	high	8.6	6	0.05		9.6
PS	63	M	2	1	21.2	4	117	1.44	0.81	99.61	2.1	high	8.9	3.5	0.06	28	11
PS	67	M	2	1	22.4	3.8	197	0.59	1.07	133.3	1.5	high	9.9	5.6	0.52	27	12.3
PS	57	M	8	1	24.4	3.8	164	1.48	1.03	81.69	2.2	high		6.2	0.3	16	9.7
PS	53	M	16	1	24.7	3.3	148	1.33	0.97	113.6	2.1	high	8.3	5.1	0.03	32	9.6
PS	67	M	13	1	18.4	3.7	144	1.26	0.89	119	1.8	high	9.2	6.3	0.05	26	9.9
PS	78	M	28	1	17.6	3.1	79	1.38	1.13	110.1	2.2	high		4.5	0.34	23	10.1
PS	78	F	12	1	20.4	3.4	143	0.87	0.91	116.6	1.3	high	7.7	5.3	0.12	20	
PS	68	F	9	1	15.6	4	156	1.62	0.68	86	1.3	high	9.3	5.3	0.05	27	12.5
PS	74	F	19	1	15.6	3.7	151	1.56	0.93	62.61	1.6	high	8.8	3.2	0.19	34	9.8
PS	75	M	2	1	21.6	3.7	148	1.55	0.83	100.3	1.6	high	8.8	4.6	1.8	29	10
PS	83	M	3	1	23.5	4.1	106	1.7	0.66	114.6	2.1	high	9.2	4		24	11.2
PS	74	F	15	1	16.9	3.6	145	1.85	1.15	112.4	1.8	high	7.5	3.6	0.8	18	8.4
PS	66	M	2	1	18	3.9	159	1.39	1.14	132.3	1.5	high	9.4	3.4	0.7	23	10.6

PS	65	F	21	1	17.8	4.2	202	1.03	1.15	106.6	1	high	8.8	5.3	0.03	19	11.8
PS	74	M	2	1	26.4	4.1	131	1.26	0.59	107.1	1.6	high		6.8	1.35	33	9.3
PS	56	F	2	1	21.1	3.8	165	1.81	0.72	88.35	1.8	high	9.1	5.4	0.92		9.8
PS	57	M	2	1	20	4.2	157	1.83	1.1	63.79	2.1	high	8.4	4.8	0.1	33	9.4
PS	56	F	19	1	22.3	3.5	180	1.5	0.64	96.43	1.4	high		6.2	0.11	27	10.4
PS	44	M	6	1	23.6	4.3	159	1.44	1.07	108.2	1.8	high	10.1	3.5			9.4
PS	65	F	2	1	21.1	3.6		1.65	0.99	91.44	1.5	high	8.9	4			10.2
PS	37	M	2	1	33.8	4.5	151	1.3	1.12	95.4	1.6	high	8.9	3.9	0.1	36	11.2
PS	48	M	28	1	20.1	4.2	137	1.53	1.28	97.58	1.4	high	8.5	5.2	0.31	31	11.2
PS	42	M	9	1	19.5	3.9	176	1.64	1.03	106.1	2.1	high	8.9	4.2	0.2		10.4
PS	57	F	5	1	20.2	3.7	210	1.63	0.78	110.1	1.5	high	10	7.1	0.02		9.2
PS	72	F	5	1	25.7	3.8		1.64	0.89	102	1.5	high		9.3	1.35		9
PS	71	F	16	1	18.6	3.8	241	1.47	0.9	115.8	2.2	high	9.2	6.7	0.17	28	9.8
PS	38	M	2	1	22.4	3.8	149	0.97	0.69	89.4	1.8	high		4.2		19	8.3
PS	31	M	7	1	23.9	4.2	148	1.02	0.71	104.4	1.6	high	8.8	3.7			10.1
PS	31	M	14	1	24.1	3.9	118	0.84	0.66	101.5	1.9	high		7	0.15	36	10.2
PS	56	M	12	1	19.9	3.5	208	1.58	0.86	97.29	1.3	high	8.4	5.7	0.08	35	11.7
PS	71	M	4	1	22.2	4.1	142	1.61	0.96	124.1	1.6	high		4.7	0.19	30	8.8
PS	81	M	10	1	16.9	3.6	199	1.37	1.23	140.4	1.6	high		5.8	0.74	29	9.9
PS	55	M	11	1	19	4	117	1.23	0.97	119.5	2.5	high	8.3	5.8	0.06	34	10.5
PS	56	F	10	1	18.1	4.2	176	1.53	1.15	100.3	1.8	high		3.7	0.3	24	8.3
PS	43	M	26	1	19	3.9	103	1.09	1.12	82.02	2.1	high	10.8	6.1	0.16	27	9.8
PS	75	M	4	1	21.4	3.9	139	1.18	0.63	109.4	1.3	high	10	3.2	0.23	21	12.6
PS	75	M	8	1	22.8	3.3	107	1.56	0.82	125.1	1.5	high	11	5.4	3.5	31	7.5
PS	65	M	7	1	21.5	3.8	176	1.66	0.87	138.7	1	high		5	0.27	33	9.1
PS	62	F	4	1	16.1	3.6	176	1.29	0.87	71.34	1.8	high	9.5	2.6	0.3		9.5
PS	69	M	12	1	20.1	4	166	1.8	0.86	132.1	2.2	high	9	9.4	2.25	31	11.4
PS	67	M	3	1	20.4	4.1	140	1.29	0.97	131.9	2.1	high	10.8	4.5	0.13	29	10.8
PS	47	M	24	1	25.4	3.7	116	1.73	1.12	88.02	1.8	high	10	6.5	0.08		9
PS	60	F	8	1	17.1	4	113	1.25	0.79	93.23	2.1	high	8.9	6.4	0.4	21	11.3
PS	45	F	5	1	21.8	3.8	107	1.33	1.02	101.3	1.8	high	8.9	5.6		13	8.5
PS	62	F	23	1	25.8	3.7	121	1.57	0.92	111.6	1.6	high		4.3	0.2	32	13.1
PS	68	F	2	1	17.1	3.8	160	1.81	1.15	67.45	1.5	high	8.3	4.4	0.27	29	10.6
PS	75	F	6	1	18.6	3.7	203	1.32	1.07	110.2	1.8	high	9.4	6.2	0.35		10.4
PS	56	M	13	1	18.4	3.9	156	1.43	1.09	122.6	1.4	high	9.7	4.1	0.03	14	11.8
PS	65	M	3	1	23.3	3.6	141	1.66	1.18	133.4	1.8	high	10.3	6.7	0.15	32	9.8
PS	62	F	5	1	19.2	3.9	119	1.71	1.16	98.43	1.5	high	9.7	4.6	0.15	24	10.5
PS	72	M	3	1	20.2	4.1	124	1.28	0.71	128.2	1.8	high	8.8	4.6			7.1
PS	70	M	10	1	18.7	3.6	125	0.96	0.98	92.7	1.5	high	8.2	5.1	0.05	27	10.8
PS	46	M	13	1	18.5	4.2	153	1.28	1.08	95.84	2.1	high	8.7	5.3	0.04	19	8.6
PS	53	M	15	1	20.5	3.5	96		1.01	110.9	1.4	high	8.1	3.6	6.38	32	9.3
PS	61	M	7	1	21.8	4	157	1.56	1.02	98.77	1.8	high	9.6	3.1	0.03	28	11.1
PS	49	M	3	1	16.4	4.1	145	0.83	0.89	84.75	1.8	high	9.5	6.4	0.2	30	10.6
PS	65	M	9	1	20.2	4.5	151	0.91	0.92	97.58	1.8	high	7.7	3.3	1.3	45	8.3
PS	65	F	29	1	17.4	3.3	198	1.22	1.16	81.11	1	high	8.3	5.9	0.04	26	10.9
PS	60	F	3	1	19.5	3.4	155	1.09	0.99	60.48	1.5	high	7.8	2.6	0.16	24	8.6
PS	73	M	2	1	21.4	4	150	1.16	0.96	101.5	1.5	high		7.2	0.1	21	10.8
PS	54	M	8	1	20.2	3.9	84	1.06	0.9	122.9	2.1	high	8.9	7.1		35	11
PS	63	M	6	1	20.5	3.8	115	1.19	0.97	104.5	2.1	high	8.6	3.6		37	13.7
PS	57	F	35	1	19.3	3.2	134	1.51	0.89	89.37	2.1	high	8.9	5.6	0.21		11.8
PS	64	F	28	1	16.4	3.4	149	1.55	0.76	71.14	1.2	high		4.2	0.1	23	11.4
PS	77	F	9	1	19.5	3.7	132	1.58	1.12	109.1	1.5	high	8.8	4.3	0.19	37	9.2
PS	57	M	3	1	23.5	3.8	159	1.62	1.08	109.6	1.3	high	9.1	5.3	0.05	22	8.9
PS	60	M	2	1	25.5	4.3	183	1.79	0.99	104.3	1.6	high		5.2	0.03	24	9.8
PS	55	M	14	1	23.4	4.1	103	1.71	0.94	160.4	1.5	high	8.9	4	0.05	33	8.8
PS	53	M	13	1	21.1	4.2	141	1.44	0.85	133.9	2.1	high	8	5.3	0.28	24	9.9
PS	75	M	29	1	25.8	3.6	157	1.12	0.8	143.8	1.8	high	8.5	7.3	0.2	34	10.1
PS	56	F	2	1	19.9	3.7	127	1.37	0.99	75.52	1.5	high	9.2	4.9	0.52	31	10
PS	78	F	11	1	17.4	3.5	170	1.62	0.91	129.3	1.8	high	10	5.5		49	10.9

PS	48	M	8	1	29.1	4	150	1.82	1.23	119.9	1.4	high	8.7	3.7	0.24	28	9.7
PS	58	F	14	1	20.4	4.1	156	1.26	1.26	121.6	1.4	high	9.9	8.1		36	9.6
PS	57	M	15	1	16.6	3.5		1.62	0.84	100.1	1.1	high		4.2	0.02	30	10.9
PS	56	F	26	1	19.5	3.8	160	1.69	1.08	113	1	high	9	6	0.4	35	9.7
PS	69	M	3	1	25.1	3.9	153	1.84	1.01	145.2	1.8	high		4.2		16	8.8
PS	53	M	19	1	18	3.6	134	1.26	0.91	110.6	2.1	high	9.1	6	0.02	29	11.3
PS	60	M	10	1	19.7	3.8	107	1.08	0.85	85.26	1.5	high	7.4	6.7	0.27	33	9.2
PS	64	F	11	1	20.5	3.8	191	1.32	0.89	116.7	1.5	high		6.9	0	17	10.4
PS	65	F	13	1	16.7	3.9		1.66	1.1	127.2	1.5	high	8.7	4.1	0.66	33	9
PS	59	F	5	1	20.6	4.3	148	1.2	1.02	91.16	1.8	high	9.1	4.9	0.13	22	9.4
PS	53	M	4	1	21.6	4	161	1.26	0.92	114.5	2.1	high	8.2	5.8	0.03	28	7.6
PS	34	M	5	1	28.1	3.5		1.06	0.97	90.41	1.3	high		5.2	0.07	26	11.8
PS	60	M	3	1	21.1	3.8	118	1.88	0.84	111.8	1.5	high	9.1	5	0.2	24	9.6
PS	66	F	29	1	18.8	3.8	171	1.63	1	130.7	2.1	high	8.5	5	0.08	26	10
PS	36	M	8	1	19	3.8	136	0.79	1.17	111.1	1.8	high		6.7		34	10.1
PS	48	M	2	1	30.2	3.9	151	1.97	0.83	88.33	2.1	high	9.4	6.3	0.11	27	11.6
PS	46	M	9	1	21.9	4.3	118	1.41	0.81	128.2	1.5	high	8	5.9	0.05	42	10
PS	50	F	4	1	20.9	3.9	201	1.85	0.93	91.71	1.5	high	10	5.7	0.82	33	9.4
PS	64	M	9	1	24.8		71	1.08	0.74	119.3	2.1	high		6			11
PS	57	M	6	1	23.7	3.8	148	1.18	0.81	110	1.5	high	10.4	5.2			
PS	76	M	7	1	19.5	3.6	152	1.13	0.91	96.91	2.1	high	9.8	7.4	0.3	30	11.4
PS	86	M	2	1	20.1	3.4	145	1.27	0.84	119	2.1	high		6.3	1.74	34	11.9
PS	75	M	2	1	22.5	3.2	151	1.29	0.9	92.41	1.6	high	9	4.7	0		11
PS	80	M	2	1	18.1	3.7	157	1.28	0.8	109.2	1.5	high	9.8	3.3			7.8
PS	56	M	23	1	24.1	4	124	1	1.02	121.8	1.5	high	7.8	4.6	0.1	29	9.3
PS	49	M	2	1	20.3	4.3	159	1.31	1.08	106.1	1.5	high	10	4.2			
PS	60	F	11	1	23.1	3.4	158	1.56	0.88	103.2	2.2	high	8.7	8.6	0.1	33	8.8
PS	52	M	30	1	22.7	3.7	199	1.28	1.08	113.1	1.2	high	8.9	4.2	0.53		6.8
PS	61	M	8	1	18.9	3.9	151	1.25	0.98	68.53	2.1	high	8.9	6.9	0.03	30	10.3
PS	53	M	4	1	20.3	4.1	166	1.99	0.9	92.55	2.1	high	9.8	5.5	0.15	21	11.5
PS	89	F	3	1	19.9	3.5	101	0.94	0.7	120.2	1.5	high	8.9	4.2			5.4
PS	63	M	3	1	20.7	4.1	223	1.93	0.66	84.62	1.5	high	9.8	4.8	0.06	27	10.2
PS	45	M	2	1	22.8	3.9	120	1.03	0.84	97.63	1.5	high	8.8	4.3			6
PS	80	F	6	1	25.2	3.9	191	1.77	0.55	135.1	1.8	high	8.6	5.2	1.12	22	9
PS	76	F	10	1	26.5	3.5	162	1.73	0.93	120.8	2.2	high	9	4.4	8.58	32	9.7
PS	34	M	4	1	29.4	3.8	162	1.98	0.79	80.01	1.6	high	8.6	4.5	0.04		9.6
PS	55	M	35	1	19.5	3.8	221	1.84	1.08	101.3	1.8	high	9.4	4.8	0.05	26	11.1
PS	60	M	31	1	22	3.5	95	1.3	0.81	126.6	1.5	high	7.7	6.7	1.47	25	9.5
PS	58	F	25	1	19.3	4	143	1.31	1.25	133.6	2.1	high	9.2	4.7	0.56	28	10.7
PS	74	M	22	1	23.4	3.4	156	1.7	0.73	121.7	2.1	high	8.6	5.4	0.37	24	6.7
PS	63	F	22	1	22.3	3.6	137	1.52	1.08	95.25	2.1	high	8.8	7.3	0.02	24	11.5
PS	61	M	20	1	17.5	3.3	141	1.27	0.81	105.6	1.8	high	8.4	7		21	12.1
PS	51	M	20	1	18.3	4	111	1.32	0.8	74.4	1.5	high	9.2	5.6	0.14	15	12.2
PS	51	M	15	1	22.9	3.8	168	1.61	0.87	110.5	1.8	high	9	6.2	0.03		9.8
PS	46	M	12	1	22.8	3.6	128	1.41	1.01	108.7	1.8	high	10.1	7.6	0.02	25	9.4
PS	72	F	12	1	21.9	3.8	152	1.84	1	153.5	1.1	high	9.6	7	0.03	43	11.1
PS	72	M	10	1	20	3.6	175	1.48	0.9	131.1	1.3	high	8.9	6.3	0.19	38	11.2
PS	53	M	7	1	23.5	4.1	163	1.76	0.88	133.8	1.5	high	8	4.7	2.46	37	9.8
PS	76	F	3	1	22.1	3.6	159	1.31	0.94	106.3	2.5	high	8.9	7.5	0.12	18	10.1
PS	64	F	22	1	21.7	3.6	147	1.24	0.75	138.3	2.5	high	9.2	8.1	0.08	19	12.2
PS	59	F	10	1	23.1	3.8	187	1	0.69	146.2	1.5	high	10.9	5.9	0.17	26	
PS	73	F	7	1	18.8		90	1.65	1.08	107.6	1.5	high	10.5	6.4	0.1	25	
PS	59	F	5	1	18.6	3.6	118	1.75	0.89	52.63	1.8	high	10.7	5.3	0.06	28	8.9
PS	55	M	2	1	26.1	4.1		0.83	0.87	114.1	2.1	high		5.4	0.19	23	11
PS	36	M	23	1	21	4.1	174	1.24	0.91	101.7	2.1	high	9.2	3.4	0.23	23	8.8
PS	55	F	20	1	15.8	3.2	193	1.3	0.83	57.72	2.1	high	7.8	7.7	0.3	28	10.6
PS	69	F	13	1	16.4	4.1	159	1.32	0.62	97.1	1.8	high	9.6	5	0.36	27	12.8
PS	46	M	11	1	21.3	3.2	137	1.46	0.81	98.55	1.5	high	9.6	3.3	0.54	52	6.4
PS	84	F	10	1	19.1	3.2	201	1.45	0.75	75.41	1.8	high	8.6	4.2	0.07	24	11.2

PS	68	M	10	1	20.2	3.4		1.51	1.1	84.22	1.5	high		6.2	0.05	27	9.9
PS	75	M	8	1	21.3	3.6	114	1.16	0.83	112.5	2.2	high	9	7.4		17	8.8
PS	65	M	8	1	24.5	3.8	169	1.89	0.82	93.13	1.8	high	8	5.3	0.2	29	10.3
PS	59	M	5	1	20.5	3.8		1.54	0.8	116.2	1.5	high		7.3	2.54	24	7.2
PS	71	F	5	1	13	4.2	195	1.3	1.1	66.77	1.8	high	10.5	5.5		26	
PS	60	M	4	1	20	4.2	181	1.29	0.75	85.61	1.5	high	8.2	4.8	0.05	23	8.4
PS	64	F	4	1	18.4	3.5	167	1.87	0.83	94.06	1	high	7.9	5.1		22	
PS	74	M	3	1	21.2	3.7	133	1.53	0.89	60.45	2.1	high		4.9		29	11
PS	35	M	2	1	20.4		176	1.36	0.92	84.22	2.1	high	8.5	4.3	0.87	31	9.9
PS	67	M	30	1	21.4	3.5	123	1.1	0.72	74.2	1.3	high	10.4	5.8	1.2	27	11.7
PS	40	F	11	1	23.1	3.8	171	1.47	1.31	89.21	1.5	high	8.8	4.3	1	24	10.9
PS	67	M	9	1	17.9	4.8	95	1.16	0.81	109.2	2.2	high	9.4	4.3	0.06	14	10.2
PS	44	M	7	1	25.4	4	161	1.21	0.68	87.27	1.6	high	8.7	4.9	0.68	18	11.7
PS	79	M	8	1	23.4	3.7	126	1.34	0.67	120	1.6	high		7.3	0.03	22	10.8
PS	72	M	7	1	20.1	3.9	105	1.38	1	142.6	1.8	high	10.4	6.4	0.1	35	11
PS	60	F	6	1	21.5	4.3	114	1.21	0.81	111.2	1.8	high	9.3	8.9	0.23	40	10.8
PS	53	M	6	1	17.9	4.2	177	2.25	1.25	111.7	1.3	high	9.3	4.7	0.29	27	7.7
PS	59	F	5	1	19.9	3.6	146	0.93	0.85	103.2	1.6	high		3.4	0.1	1	10.9
PS	60	M	5	1	21.4	4	196	1.3	0.81	138.5	1.8	high	8.8	4.9	0.03	16	10.2
PS	75	M	4	1	21	3.7	136	0.76	0.66	93.66	1.8	high	9	7.4		29	10.3
PS	75	M	4	1	19.6	4.1	129	1.26	0.93	81.78	1.3	high	7.4	4	0.62	21	7.4
PS	65	F	4	1	17.5	3.9	149	1.7	0.84	111.4	1.8	high		5	0.46		9.2
PS	74	M	4	1	17.6	3.9	152	1.4	0.85	141	1.8	high	9.6	3	0.05	27	10.4
PS	80	F	3	1	18.9	4	146	1.72	0.97	118.2	1.8	high	8.7	4.6	1.06	50	8.5
PS	84	F	3	1	15.9	4.2	190	1.19	0.77	128.7	1.5	high	9.2	5.1	0.03	24	11
PS	69	M	3	1	22	4.2	153	1.53	1.19	125.4	1.8	high	9.4	5.3	1.07	23	10.4
PS	72	F	3	1	18.5	4.3	179	1.24	0.46	27.75	2.2	high	8.2	2.8	1.5	20	7.8
PS	64	M	3	1	21	4.3	112	1.51	0.92	102.6	1.8	high	8.8	7.7	0.26	29	11.3
PS	61	F	3	1	18.4	3.8	142	0.98	1.11	131.6	1.8	high	9.4	8	0.1	27	10.5
PS	67	M	3	1	18.1	3.8	156	1.41	0.86	111.5	1.3	high	9.3	4.4	1.54	35	9.8
PS	31	M	2	1	19.9	4.6	162	1.6	0.75	84.78	1.8	high	9.2	5.7	0.79	26	10.1
PS	65	F	2	1	22.3	3.6	126	1.23	0.72	49.5	1.8	high	10.2	5.9	0.05	30	10.8
PS	75	M	2	1	17.8	4	169	1.46	0.77	66.38	2.1	high		6.2	0.14	27	9
PS	74	M	2	1	20.5	3.8	156	0.97	0.86	89.42	1.1	high	7.1	4.7	0.23	23	9.9
PS	66	F	2	1	17.9	4	158	2.1	1.33	54.66	2.1	high	9	5.8	0.02	25	10.2
PS	83	F	2	1	19.2	4.2	108	1.44	0.64	117.4	1.4	high	7.9	4.3	0.3	27	10.3
PS	73	M	2	1	24.8	4.3	171	1.4	0.88	110.4	1.4	high	8.9	5.2	0.05	19	10.4
PS	80	F	2	1	18.9	4	134	1.56	1.02	86.59	1.4	high	9	4.6	0.26	18	9.7
PS	64	F	20	1	22.4	3.9	215	0.85	1	113.2	1.8	high		4.1		18	9.9
PS	52	F	19	1	23.9		219	1.44	0.72	105	1.8	high		6.7	0.17	36	10.7
PS	38	M	5	1	26.4	4.6	158	1.55	0.96	119.6	2.5	high	8.3	8.4	0.04	21	11.1
PS	75	M	4	1	22		142	1.47	0.8	143.8	1.8	high		5		22	10.7
PS	73	M	8	1	21.5	3.9	137	1.43	1	104.1	1.5	high	9.3	5	0.05	23	11.5
PS	63	F	13	1	19.8	3.7	305	1.19	0.88	88.79	2.2	high		8.2		32	10.7
PS	55	M	23	1	20.5	4.1	169	1.33	0.73	110.9	2.2	high	8.8	8.1	0.4	31	12.4
PS	52	F	12	1	16.7	4.6		1.37	1.07	117.3	2.2	high	8.3	4.2	1.92		
PS	79	M	10	1	19.7	4.3	171	1.37	1.02	113.1	1.5	high	9.4	3.8	0.06	25	9.4
PS	59	M	10	1	22.2	4.1	80	1.24	1.02	106.9	1.5	high	8.3	4			
PS	58	M	9	1	17.9	4.4	157	1.27	0.89	116.7	1	high	8.7	4.1	5	30	9.4
PS	46	F	7	1	24.9	4	114	1.65	0.92	129.1	1	high	12.8	6.3	12.6	57	
PS	73	M	6	1	20	3.9	136	1.48	0.87	110.6	1.5	high	9.2	4.3	0.62	28	11.8
PS	59	F	5	1	22.2	3.9	121	1.29	0.75	131.3	1.3	high	10.1	6.7	6.9	30	5.7
PS	77	F	5	1	18.7	4	180	1.4	1.4	107.2	1.8	high	6.8	3.2	0.3	28	10.5
PS	58	F	3	1	23.2	3.3	154	1.25	0.74	125.4	2.1	high	9.6	4.1	0.05	31	11.5
PS	63	M	4	1	20.4	3.8	188	1.5	0.82	110	1.6	high	8.5	5.4	0.05	29	13.3
PS	66	M	5	1	25.4	4.4	134	1.88	0.63	120.1	1.5	high	8.2	5.6	0.05	39	10.3
PS	58	M	14	1	21.6	3.8	161	1.19	0.89	114.5	1.3	high		4.5	0	24	10.4
PS	50	F	7	1	18.4	3.9	130	1.24	0.94	113.1	2.1	high		6.2	1.63	26	11.3
PS	75	M	3	1	23.4	3.8	162	1.45	0.9	106.7	2.1	high	10.4	3.9	0.06	32	11.7

PS	67	F	25	1	19.7	3.7	129	1.02	0.89	109.9	1.6	high	7.8	5.2		17	11.4
PS	62	M	13	1	20.8	3.7	103	1.76	0.79	116.6	1.3	high		4.9	1.3	24	11.1
PS	64	M	10	1	23.4	3.4	158	1.39	0.67	112.8	1.8	high	8.2	3.9	0.75	20	10.1
PS	73	M	4	1	21.7	3.7	129	1.45	1.13	123.3	1.1	high		3.4	1.4	28	10.6
PS	45	M	3	1	18.2	4	102	1.46	0.93	95.32	1.8	high	8.8	3	4.1	40	9.6
PS	57	M	4	1	23.9	3.7	226	1.74	0.78	108.9	1.6	high	9.2	2.8	0.1	22	9.3
PS	65	M	13	1	22.5	3.7	165	1.35	0.98	104.7	1.3	high		5.9	0	13	11.1
PS	58	M	4	1	27.6	3.7	180	1.17	0.75	113.7	1.8	high		6.2	0.29	29	10
PS	80	F	7	1	21.8	3.5	164	1.68	0.76	85.43	1.5	high		4.9	1.67	14	12.2
PS	82	M	3	1	20.9	3.5	121	1.44	0.72	51.14	1.5	high	9	4.7	0.3	29	11
PS	69	M	21	1	19.7	3.9	115	1.32	0.97	147.6	2.2	high	9	4.9	0.1	28	10.1
PS	67	F	10	1	19.3	3.6	155	1.4	0.92	120.9	1.6	high	9	4			
PS	72	F	5	1	18.1	3.6	117	1.58	0.8	119	1.5	high	8.1	5.9	0.97		12.3
PS	64	M	2	1	22.1	3.7	153	1.83	0.89	131.2	1.5	high	8.6	3.2	0.34	24	9.6
PS	59	M	2	1	23.3	3.7	143	1.07	0.72	135.8	1.4	high	8.5	3.9	0.7	33	8.5
PS	65	F	2	1	28.1	3.3	210	1.73	1.01	64.51	1.5	high	9	3.6	0.12	20	11
PS	69	M	2	1	22.2	4.2	135	1.13	0.89	119.4	2.1	high	7.6	4.9	0.06	26	9.2
PS	68	M	2	1	20.9	3.8	142	1.49	1.01	120.6	2.1	high	8.9	5.5	0.1	27	10.4
PS	61	M	15	1	22.9	3.7	99	1.36	0.82	92.39	2.1	high	8.7	3.8			10.9
PS	61	M	9	1	24.6	3.7	137	2.17	0.7	113.9	1.5	high	8.8	6.1	0.07	35	9.6
PS	68	M	23	1	20.7	3.7	151	1.5	0.77	116.1	1.8	high	9.1	7.1	0.06	27	9.8
PS	65	M	6	1	18.8	3.8	174	1.32	0.9	116.6	1.8	high	8.2	8.8			10.7
PS	54	M	10	1	18.7	4.4	112	1.4	0.85	102.2	1.4	high	8.3	4.6	0.2	19	10.4
PS	56	F	13	1	17	3.9	161	2.22	0.96	116.6	1.3	high	10.2	4.4	0.35		10.8
PS	71	M	4	1	23.5	3.4	137	1.43	0.85	89.28	1.5	high	9.3	4.7	0.16	36	11.8
PS	63	M	9	1	23.4	4.1	123	1.44	0.98	125.3	1.8	high		3.2	0.1	25	8.9
PS	74	M	29	1	20.1	3.9	144	1.21	1.06	121.7	1.4	high	9.8	4.7	0.6	27	10.9
PS	74	F	20	1	16.2	3.8	136	0.83	0.81	136.1	1.5	high	7.3	5.8	0.82	28	9.4
PS	58	F	16	1	19.1	3.2	162	1.65	1.41	123.6	1.6	high	8	3.6		27	11.1
PS	77	M	14	1	20.5	3.8		1.46	1.14	114.5	1.3	high	9.1	6.1	0.15	20	11.4
PS	25	F	13	1	18.6	3.7	140	2.27	1.21	94.1	2.1	high	9.4	3.9	0.03	31	11.6
PS	58	M	7	1	21.7	3.8	211	1.62	1	93.02	1.8	high	9.4	4.6	0.2	30	11.1
PS	69	M	3	1	22.3	4.1		1.13	0.97	120.1	1.5	high	7.7	5.9	0.36	21	9.3
PS	72	M	2	1	18.7	4.1	223	1.67	1.08	97	1.5	high	10.3	6.2	0.28	48	8.3
PS	83	M	2	1	19.6	3.6	146	1.47	0.69	85.58	1.8	high	7.3	6.5	0.57	33	8.7
PS	76	M	3	1	26.2	3.5	104	1.12	0.73	125.4	1.5	high	8.3	4.5	0.03	23	10.7
PS	65	F	17	1	18.7	4.1	218	0.84	1.14	113.1	1.5	high	8	5.4	0.58	31	11.5
PS	59	M	15	1	20.5	3.5	175	1.68	0.65	122.4	1.1	high	9.1	6.4	0.02	33	8.9
PS	78	F	3	1	20.1	3.7	182	1.82	0.78	89.62	1.1	high	10.2	4.7	0	30	10.4
PS	59	M	2	1	20.4	3.9	142	2.09	0.7	79.97	2.1	high		5.6	0.05	20	12.1
PS	67	M	9	1	24.4	3.3	116	1.31	0.8	107	2.1	high		5.6	0.04	28	10
PS	62	M	6	1	19.4	3.7	123	1.66	0.85	120.8	1.5	high	9.9	6.8	0.48	27	9.6
PS	71	M	7	1	20.2	3.5	275	1.77	0.9	106	1.8	high	10	5.1	0.03		10.1
PS	59	F	4	1	22.4	3.5		1.29	1.27	76.73	1.3	high	8.5	6.3	0.13	13	11.4
PS	48	F	12	1	17.4	4.5	175	1.5	1.16	130	1.8	high	9.1	4.3	0.11	22	11.6
PS	67	M	9	1	19.8	3.9	122	1.47	1.1	103.6	2.2	high	9.6	5.3	0.13	26	10.7
PS	61	M	7	1	24.2	3.9		1.93	1.06	116.9	1.3	high	9.4	2.7	0.01	17	12.9
PS	70	M	6	1	23.5	3.7	117	0.77	1.05	141.7	2.2	high		4.5			8.6
PS	62	F	5	1	19.8	3.5		2.12	1.31	122.7	1.3	high	10.7	3.8	0.33	22	9.9
PS	58	M	3	1	18	3.6	130	1.33	0.57	90.09	2.1	high	9.4	4	0.27	26	10.4
PS	73	M	2	1	26.2	3.8		1.49	0.88	81.54	1.3	high	9.5	3.9	0.09	16	9.9
PS	53	M	13	1	22.9	3.8		1.49	1.11	124.1	1.5	high	9.7	4.2	0.06	17	10.4
PS	56	M	10	1	23.9	4.2		1.53	1.19	142.5	1.8	high	9	3.2	0.48	22	10.8
PS	28	M	10	1	17.6	4.2	95	0.91	0.89	134.1	1.6	high	8	5.6			11.9
PS	54	M	6	1	26.9	4	131	1.37	0.89	99.24	2.5	high		5.1	0	27	11.7
PS	79	F	8	1	21.5	4.5	206	1.22	0.77	67.55	1.6	high	7.9	6.4			9.5
PS	72	M	4	1	25.3	4	165	1.68	0.64	95.63	1.8	high	10.4	4.2	0.41	26	9.9
PS	63	F	3	1	18.6	4.1	198	1.14	1.1	114.6	1.8	high	10.4	6.8	1.02	29	12
PS	59	F	3	1	18.7	4		1.49	0.61	88.1	1.8	high	10.1	6.6	0.28	18	9.3

PS	60	M	2	1	23.7	4.1		1.86	0.82	77.71	1.3	high	11	4.2	0.79	23	9
PS	67	M	8	1	16.2	4	134	1.33	1.04	126	2.2	high	8.4	7.7	0.52	39	10.5
PS	61	M	4	1	24.5	3.8		1.83	0.84	103.5	1.5	high	9.7	4.7	0.01	24	11.7
PS	64	F	2	1	16.9	3	159	1.47	1.12	69.82	1.1	high	9.2	5.1	0.17	38	6.6
PS	61	M	2	1	23.6	4		1.39	0.79	76.91	1.3	high	9.8	7.2	2.47	23	10.9
PS	69	F	22	1	19.8	3.4	176	2.16	0.68	111.8	1.5	high	9.4	5.4	0.06	31	10.5
PS	56	F	22	1	20.5	4.4	116	1.75	0.93	92.2	1.6	high	9.1	5.7	0	31	11.5
PS	59	F	19	1	21.1	3.8		1.29	0.89	108.5	1.3	high			6	16	9.4
PS	56	M	14	1	22.3	4	162	1.24	1.11	123.3	1.8	high	10.3	6.5	0.1	26	10.3
PS	40	M	12	1	22	3.8	160	1.7	0.83	105.3	1.5	high		6.9	0.6	31	10.6
PS	46	M	12	1	23.2	3.8	73	0.97	1.24	96.4	1.5	high	10.6	4.5	0.48	25	9.2
PS	48	M	11	1	19.8	4.2	185	1.47	0.73	113	1.5	high	9.5	9	0.04	16	11.3
PS	44	F	9	1	18	4.3	146	1.23	0.88	96.42	1.3	high	8.6	6.8	0.06	32	10.3
PS	57	F	9	1	21.2	3.7	201	1.57	0.56	91.28	1.3	high	9.1	3.3	0.17	32	12.3
PS	71	F	8	1	19.9	4	137	0.99	0.93	118.4	2.1	high	7.6	5.4	0.18	17	10.2
PS	51	F	8	1	20.6	3.9	88	1.31	0.75	87.37	1.8	high	9.5	4.1	0.52	44	9.8
PS	60	M	7	1	23.6	3.8	176	1.31	1.22	101.7	2.1	high	8.3	4.1		26	11.3
PS	64	M	7	1	23.8	3.8	122	1.75	0.75	76.79	1.6	high		6	0.14	26	10.7
PS	61	M	7	1	25	3.6	171	1.46	1.01	130.2	1.8	high	8.6	6.6	0.16	33	11.9
PS	76	M	7	1	21.2	3.7	115	1.39	0.81	136.5	2.2	high	8.4	5.6	0.4	34	10.3
PS	70	M	7	1	24.6	3.6		1.63	0.59	109	1.3	high	8.7	4.3	4.18	25	11.5
PS	59	M	6	1	22	3.8	167	1.66	0.73	96.14	1.8	high	8.7	6.5	0.17	20	11
PS	45	F	6	1	32.5	3.7	131	1.57	0.94	120.3	1.5	high		5.5		22	10.9
PS	70	F	6	1	25.5	3.6	112	1.23	1.18	100.7	1.5	high	9.5	5.5	1.22	34	11.9
PS	71	M	5	1	22.4	3.5	117	1.26	0.62	82.81	2.1	high	8.5	4.9	0.79	29	8.4
PS	57	M	5	1	23.1	3.4	186	1.44	0.71	96.24	1.5	high	9.4	8.7	0.21	30	10.2
PS	57	M	3	1	23.9	3.5	112	1.21	0.7	106.1	2.1	high	7.5	6	0.1	32	10.3
PS	65	F	3	1	23.7	3.6	148	0.95	0.95	133	2.1	high	8.2	4.7			10.7
PS	58	F	3	1	26.1	4	159	1.36	0.95	123.7	1.6	high		2.5	0.09	23	9.1
PS	71	F	2	1	19.4	3.9	140	1.38	0.83	108.2	1.8	high	7.9	7.6	4.67	14	10.5
PS	41	F	9	1	17.2	3.9	112	1.21	0.79	82.81	2.1	high	9.5	5.3	0.12	32	10.7
PS	41	M	18	1	17.4	4.5	176	2.18	1.01	105.1	1.3	high		4.1		21	9
PS	71	F	11	1	19	3.6	181	1.3	0.65	109	1.4	high	8.8	3.6	0.09	20	9.7
PS	51	M	4	1	15.6	3.8	162	1.06	0.86	85.87	2.5	high		7.8			10.1
PS	56	M	25	1	24.5	4	151	1.5	0.8	97.31	1.5	high	7.7	7.9	0.05	25	10.9
PS	47	M	20	1	23.4	3.7	127	1.77	0.73	106.8	2.1	high	9.8	6.9		23	10.9
PS	45	M	5	1	20.7	3.8	143	1.42	0.74	97.79	1.1	high	8.4	4.4	3.18	34	9.8
PS	41	M	18	1	19.1	3.4	145	1.78	0.89	76.17	2.1	high	8.9	5.8	0.5	29	12.2
PS	57	F	5	1	18	3.9	150	1.02	0.76	105.2	1.8	high	10.2	6.9	0.05	28	11.9
PS	44	M	5	1	22.9	3.8	174	1.37	0.94	96.9	1.4	high	8.5	5.4	0.81	29	12.1
PS	71	M	3	1	18.3	4.1	130	1.17	0.69	139.2	1.5	high		5.9	0.32	29	13.3
PS	72	F	19	1	14.7	4.1	133	1.22	0.87	83.53	1.9	high	8.5	8.9		34	15.9
PS	66	F	17	1	17.3	3.9		1.14	1.02	100.9	1.8	high	8.7	6.3	0		11.2
PS	55	M	13	1	22.7	3.9	116	1.73	0.86	107.5	1.5	high	9.8	7.1	0.42	25	10.9
PS	30	F	2	1	21.7	4.1	197	1.13	0.91	76.33	1.6	high		4.5	0.16	19	8.8
PS	52	F	19	1	18.6	3.8	100	1.3	0.78	86.62	2.5	high	8	8.2	0.07	21	11.2
PS	38	M	19	1	18	4.3	186	1.58	0.66	101.3	1.6	high	8.6	5.7	0.14	25	9.5
PS	37	M	9	1	27.4	4.1	161	1.28	0.96	94.08	2.5	high	9.2	6.7	0.26	25	12.3
PS	58	M	2	1	23.3	4.2	164	1.49	0.97	104.8	1.4	high	9.1	3.8	0.8		10.1
PS	77	M	18	1	20.2	3.4	184	1.4	0.62	116	1.8	high	10.7	6.5	1.81	38	11.4
PS	43	M	16	1	17.2	4.1	222	1.28	0.95	104.4	2.2	high		6.6	0.86	27	10.2
PS	49	M	8	1	24.2	4.5	171	0.95	0.77	102.2	1.8	high	8.9	5.5		28	10
PS	32	M	3	1	18.5	4.7	159	1.16	0.9	95.06	2.1	high	9	4	0.1	28	11
PS	55	M	30	1	21	4.3	157	1.25	0.95	105.4	2.5	high	8.8	9.5	0.68	21	13.3
PS	74	M	15	1	19.1	4	146	1.29	0.93	111.1	1.8	high	9.6	4.7	0.28	23	10
PS	57	F	7	1	21.9	5	129	1.57	0.9	90.7	2.1	high	9.7	6.2	0.8	25	13.6
PS	73	M	6	1	19.9	3.7	131	1.41	0.87	87.39	1.8	high		6.2	0.05	18	12.6
PS	80	M	3	1	19.8	3.9	128	1.08	0.96	79.21	1.3	high	9.3	3.8	1.26	27	10
PS	58	M	2	1	21.8	4.6	218	1.61	1.17	72.91	1.5	high	9.5	3.5	0.05	37	9.6

PS	68	M	2	1	21.9	3.7	199	1.45	0.96	124.4	1.5	high	8.3	5.3	0.18	44	10.9
PS	61	M	10	1	19.8	4.1	182	1.8	0.92	135.4	1	high	10.6	5.1	0.35	23	9.2
PS	71	F	2	1	19.5	3.7	176	1.12	0.89	82.13	1.6	high	8.1	5.5	0.3	29	10.7
PS	78	M	16	1	21.9	3.7	121	1.13	0.89	100.2	1.5	high		9.1			10.6
PS	59	M	15	1	21.7	4.1	117	1.46	0.98	103.3	1.5	high	8	5.2	0.15	37	7
PS	58	F	14	1	21.6	3.9	182	1.47	1.31	124.5	1.5	high	9.3	5.7	0.04		9.8
PS	68	F	13	1	18.8	4.2	97	1.78	1.08	120.4	1.5	high	8.4	6.3	2.75		9.7
PS	69	F	13	1	17.8	3.9	141	1.52	0.77	100.8	1.1	high	9.5	6.2	0.2		10.4
PS	58	F	12	1	26.5	3.6	161	1.32	0.95	116.5	1.5	high	8.7	5.1	0.04	39	12.7
PS	69	F	12	1	23.1	3.8	165	0.99	0.95	159	1.1	high		6.5	0.59	35	8.9
PS	61	M	12	1	22.8	3.7	158	1.51	0.82	110.5	2.1	high		4.5	0.12	39	9.8
PS	25	M	9	1	16	4.1		1.04	0.99	89.3	2.1	high	9.1	5.4	0.02	23	12.6
PS	42	F	8	1	19.2	4.1	158	1.57	1.3	105.2	2.1	high	8.6	4.1	0.22	28	11.6
PS	59	F	8	1	19	4	139	1.3	1.05	109.8	1.5	high	9.6	7.2	0.02		10.6
PS	44	M	8	1	19.5	4.3		1.62	0.7	90.18	1.5	high	9.8	4.7	0.04	29	7.6
PS	72	M	7	1	19.2	3.4	127	1.14	0.77	133	1.5	high		5.3	0.67	22	10.4
PS	57	F	7	1	24.2	4	118	1.2	0.92	137.7	1.5	high		5.1	0.02	22	10.9
PS	52	F	7	1	15.9	3.8	154	1.53	0.78	99.6	2.1	high	8.9	2	0.47	33	10.4
PS	57	M	6	1	21.6	4.1	212	1.25	0.91	133.8	1.8	high	7.8	8.4	0.08	17	9
PS	31	M	6	1	24.6	4.6	155	1.66	0.97	128.9	1.5	high		4.5	0.3	28	10.2
PS	63	M	5	1	17.6	3.7		2.04	1.11	133.1	1.3	high	9.3	3.7	0.06	23	9.3
PS	61	M	5	1	22.8	3.9		1.7	1.02	101.2	1.3	high	9.4	4.6	0.09	20	12.1
PS	40	F	8	1	18.4	4.3	171	1.37	0.69	121.3	2.1	high	9.7	3.4	0.09		11.3
PS	74	F	4	1	22.9	3.8	141	1.01	0.65	111.5	0.8	high	7.1	3.1	5.98	20	6.7
PS	52	M	4	1	21.8	4.4	143	1.68	0.79	83.55	1.8	high	8.6	3.1	0.12		8.4
PS	66	M	3	1	22.3	4	196	1.28	0.72	87.55	1.6	high		4.4	0.05	22	11.5
PS	59	M	3	1	20.5	3.5	166	1.63	0.91	88.24	1.6	high		3.6	0.06	36	11.5
PS	57	M	2	1	22.4	3.7	168	1.36	0.63	52.68	1.8	high	10.7	9	0.04	25	9.4
PS	51	M	2	1	21.3	3.9	164	1.3	0.75	98.82	2.1	high	8.7	6.6		23	10.6
PS	55	M	2	1	30.2	4.5	175	1.51	0.8	82.44	1.6	high		2.5	0.02	31	11.1
PS	43	F	2	1	17.2	3.9	180	1.37	0.74	73.66	1.3	high		5.2	0.11	21	11.6
PS	65	M	8	1	19.9	4.1	159	1.5	0.98	101.8	2.2	high	9.3	4.8	0.06		13.6
PS	70	M	2	1	21.9	4.2	178	1.86	0.85	59.52	1.5	high	9.8	4.2	0.2	32	8
PS	35	M	3	1	18.9	4.4	145	1.39	0.79	80.46	1.8	high	9.5	4.3	0.05	39	9.9
PS	80	M	3	1	28.8	4	175	1.6	0.56	52.88	1.5	high	8.8	5.5	0.15	22	8.8
PS	72	F	5	1	21.2	4.1	145	0.99	0.66	115.4	2.1	high	8.4	7.7			10.3
PS	68	M	6	1	21.1	3.8	165	1.71	0.92	90.15	1.5	high	9.6	5	0.07	24	10.2
PS	22	M	3	1	17.7	4.2	132	1.35	0.84	83.05	2.1	high	10.4	6	0.21	33	12.3
PS	56	F	20	1	18.4	4.5		1.34	0.78	112.9	2.2	high	9.7	4.8	0.25	24	10.9
PS	78	M	6	1	21.1	4.5	201	1.79	0.76	119.3	1.4	high	9.2	5.4	0.03		11
PS	70	M	6	1	19.3	3.9	192	1.46	1.08	95.81	1.8	high		6		16	10.8
PS	45	F	2	1	27.6	4.3		1.11	1.1	97.4	1.5	high	10.8	5.4	0.1	24	10.3
PS	65	M	3	1	24.7	4.2	189	1.79	1.08	108.8	1.3	high	9.2	5.1	0.04	21	7.7
PS	42	M	3	1	33.1	3.9		1.61	0.9	60.57	1.3	high	9.7	3.9	0.16	25	11.8
PS	56	F	18	1	16	3.6	162	1.37	1.02	112.7	1.5	high	9.6	3.6	0.35	28	10.6
PS	69	M	4	1	27.4	4.2		1.77	0.89	162	1.5	high		5.1	0.19		10
PS	74	F	5	1	16.7	3.7	193	1.29	0.69	115	2.2	high	8.1	6.9	0.9	26	10.9
PS	76	M	6	1	16.9	3.9	228	1.3	0.66	110.2	2.1	high	10	5.2		25	11.3
PS	63	M	2	1	19.3	3.9	190		0.67	56.05	1.3	high	8.4	5.2	1.78	16	10.8
PS	61	F	15	1	20.9	3.9	147	1.39	0.87	106.9	1.8	high	9.9	6.1	0.06	29	11.9
PS	63	F	2	1	23.5	3.5	119	1.72	0.74	79.08	2.1	high	9.6	5.5		22	9.9
PS	64	F	14	1	16.6	4.1	197	1.15	0.77	131.7	1.8	high	10.7	8.3	5.48	30	13.3
PS	48	F	9	1	17.1	3.6	179	1.87	1.02	143.1	1.5	high		5.6	0.1	29	11.6
PS	64	M	8	1	19.3	4.2	115	1.66	0.59	124.5	1.5	high	9.3	5.2	0.07	43	9.6
PS	71	F	18	1	17.9	3.1	93	0.9	0.91	112.6	2.1	high	8.5	5.6		32	10.4
PS	30	F	4	1	21.7	4.3	148	2.02	0.79	96.74	2.1	high	8.3	3.2	0.23	32	11.1
PS	64	M	9	1	24.2	3.9	226	1.69	0.57	136.8	1.8	high	8.5	5.1	0.03	26	10.7
PS	77	M	2	1	18.9	4.3		1.26	0.72	109.4	2.2	high					
PS	78	M	3	1	20.3	4	112	1.42	1.08	94.35	2.1	high	8.7	7.4	0.1	34	10

PS	89	M	4	1	21.8	4.2	126	1.74	0.73	65.73	1.8	high	9	4.3	0.18	21	12
PS	68	F	30	1	17.4	3.5	152	1.25	0.94	100	2.1	high	8.6	5.6	0.03	17	11
PS	73	F	6	1	19.9	4.2	140	1.43	0.93	122.9	1.8	high	9.8	4.5	0.02	31	10.7
PS	57	M	3	1	20.5	3.7	171	1.08	0.93	127.2	1.5	high	8.3	3.7	1.4	14	11.5
PS	42	M	24	1	15.8	4.4	165	1.46	1.09	123.5	1.8	high	9.1	5.1	0.03	29	10.5
PS	64	F	30	1	14.9	4	99	1.71	0.85	89.87	1.8	high	9.8	5.8	0.1	33	11.1
PS	55	F	11	1	27	3.6	99	1	0.79	113.8	1.8	high	8.5	6.2	0.04	27	12.8
PS	73	F	11	1	21	4.1	80	1.47	1.15	118.9	1.8	high	9.7	6.6	0.05	27	10
PS	66	M	17	1	16.3	3.7		1.31	0.97	94.92	2.2	high		5.1			
PS	72	M	4	1	21	3.7	178	1.09	0.86	117	1.5	high	10.4	6.5	0.14	29	12
PS	64	M	2	1	18.6	4	161	1.09	1	94.92	2.1	high	9.4	3.8	0.15	24	6.2
PS	74	M	2	1	20.7	4	204	1.06	0.76	78.07	1.5	high	7.9	5.1	0.17	21	10.4
PS	55	M	2	1	20	3.4	180	1.34	0.94	99.12	1.6	high		4.1	0.1	1	12.1
PS	47	M	16	1	20.9	3.4	116	1.05	0.62	108.8	2.1	high	8.7	5.9	0.03	26	13.5
PS	35	M	4	1	17.7	3.9	139	1.33	0.76	78.37	1.8	high	8.4	5.5		25	10.6
PS	66	M	2	1	22.5	4.2		1.26	1.29	123.5	1	high	10.6	5.3		29	11.6
PS	64	M	11	1	19.9	3.9	130	0.96	1.03	117	1.8	high		5.8			9.6
PS	73	M	6	1	21.8	3.9	150	1.4	0.89	83.56	2.1	high	9	4.9	0.74	15	8.5
PS	52	F	9	1	26	4.2	140	1.69	0.87	105.5	1.4	high	7.3	4.8	0.1	20	8.8
PS	60	M	19	1	18	3.6	209	1.62	0.86	120.7	1.8	high	8.5	9.5	0.09	24	11.9
PS	55	F	19	1	18.8	3.6	136	1.86	1	124.1	1.8	high		5		36	9.9
PS	18	M	10	1	16.2	3.8	156	1.27	1.19	95.07	1.5	high		2.7			10.6
PS	60	M	6	1	19.8	3.4	97	1.42	0.89	126.4	1.5	high		6.8			10.8
PS	69	F	16	1	17.5	3.7	148	1.32	0.98	136.3	1.8	high		6.8	0.2	18	11.6
PS	46	F	20	1	21	3.9	143	1.37	1.15	113.5	1.8	high	9.1	6.4	0.11	30	11.3
PS	51	M	20	1	22.7	3.5	152	1.59	0.98	100	1.4	high	8.2	5.1	0.03	30	9.7
PS	65	F	13	1	22.9	3.8	101	1.45	1.05	114.3	1.8	high	8.3	5.8	0.08	30	9.7
PS	65	F	13	1	22.9	3.7	105	1.37	0.94	129.3	1.5	high	8.6	7.5	4.81	39	10.4
PS	70	M	12	1	20.6	3.7	207	2	0.72	99.09	1.8	high	10.3	6	0.06	31	11.6
PS	66	M	12	1	20.2	3.7	58	1.35	0.99	105.8	1.8	high	9.7	5	0	27	10.3
PS	58	F	11	1	27.9	3.7	144	1.75	1.06	115	1.5	high	9.6	4.5			10.9
PS	55	F	10	1	23	3.5	198	1.52	0.55	87.13	1.6	high		5.7	1.63	28	10.6
PS	65	F	10	1	24.8	3.6	154	1.71	0.89	113.1	1.8	high	8.8	4.5	0.15	29	11.9
PS	62	F	8	1	26.1	3.7	145	1.35	0.8	129.5	1.8	high	10.3	5.3	0.02	25	10.3
PS	54	M	8	1	17.2	3.6	116	1.3	1.05	125.9	1.8	high	9.2	7.1	0.03	28	10.8
PS	69	M	6	1	23.6	4.1	128	2.04	1.01	143.8	1.8	high	9	6.4	0.15	35	9.7
PS	59	M	6	1	24.8	4	173	1.1	1.06	100.3	2.1	high	8.4	5.2	0.02	22	12.5
PS	31	F	5	1	21.3	4.2	175	1.05	0.66	90.49	1.8	high	8.4	5.9		21	10.5
PS	58	F	5	1	27	3.9	202	1.62	1.19	124.4	1.8	high	10.3	3.9	0.68	34	10.2
PS	69	F	5	1	22.4	3.7	104	1.32	0.99	118.8	1.8	high	9.7	6.3	0.03	29	10.4
PS	73	M	5	1	15.5	4	104	1.42	1.09	111.8	1.5	high	9.9	4.9	0.26	26	10.1
PS	54	F	5	1	27.6	3.8	159	1.37	0.93	77.22	1.5	high	9.8	4.5	0.07	32	10.8
PS	62	F	5	1	23.9	3.7	200	1.86	0.81	107.4	1.5	high	10.2	3.2	0.05	25	10.4
PS	37	M	5	1	27.1	3.5	158	1.48	0.59	24.14	2.1	high	9.9	4.8	0.14	30	11.2
PS	47	M	5	1	29.7	3.9	106	1.29	0.95	116.8	1.6	high	8.2	2	1.3	32	7.7
PS	77	M	4	1	22.6	3.5	145	0.89	1.14	88.82	1.8	high	9.5	5	1.93	48	10.1
PS	70	F	4	1	16.8	3	133	1.39	0.8	66.07	1.8	high	9.1	7.1	0.16	35	9.7
PS	63	F	3	1	19.2	4.1	154	1.6	0.76	56.61	1.5	high	10.5	4.3	0.83		12
PS	60	M	3	1	22.1	4.1	175	1.29	0.98	115.7	2.1	high	9	3.7	0.03	23	
PS	66	M	3	1	19.3	4.1	157	1.26	0.85	83.61	1.5	high	8.8	5	0.2	31	10.5
PS	75	M	3	1	23.4	4.1	163	1.37	0.89	131.6	1.8	high	8.8	5.1	0.02	30	11.3
PS	74	F	3	1	15.2	3.3	149	1.31	0.63	64.97	1.8	high	8.8	4.8	0	32	10.3
PS	77	M	2	1	24.2	3.5	98	1.39	0.87	143.3	1.8	high	8	3.8	0.04	22	10.5
PS	72	F	2	1	22.2	3.8	144	1.12	0.95	115.5	1.4	high	8.8	5.9		26	
PS	71	M	2	1	22.3	3.7	138	1.15	1.02	79.7	1.8	high		5.1	3.3	30	10.8
PS	69	F	2	1	18.2	3.7	114	1.19	0.87	127.8	1.8	high	9.7	5.3	0	25	8.8
PS	72	M	2	1	19.2	3.9	196	1.35	0.77	84.69	1.5	high	10.1	5.6	0.3	20	11.3
PS	66	M	2	1	21.1	4	150	1.29	0.71	53.81	1.5	high	8.2	5.9	0.21	29	11.5
PS	63	F	2	1	22.2	3.5	188	1.5	1.16	85.94	1.4	high	8.4	4.1	0.14		8.9

PS	23	M	5	1	19	4.1	135	1.35	0.93	103.5	1.5	high	8.6	1.5		27	9.9
PS	34	M	17	1	19.3	4.2		2.02	0.69	58.81	1.8	high	8.9	5.8	7.94		11.2
PS	43	F	26	1	19.4	4.1	145	1.8	0.98	135	1.6	high	10.9	6.2	0.19	34	10.4
PS	55	M	15	1	22.3	3.5	198	1.47	1.01	101.4	1.8	high	9.4	9.8	1.23	32	11.6
PS	55	M	7	1	21.9	3.5	191	1.47	0.9	113.8	1.3	high	9.1	5.9	0.02	21	12.8
PS	57	F	2	1	25.2	3.5	117	1.44	0.81	105.3	2.1	high	8.7	5	1.01		9.4
PS	56	F	21	1	19.9	3.6	139	2.13	0.85	160.3	1.9	high		5.2	0.05	26	7.7
PS	44	M	8	1	23.8	4	139	1.59	0.91	107.2	1.3	high	8.4	4.9	0.04	17	10.5
PS	56	F	5	1	24.6	3.7	149	1.46	0.66	58.89	2.1	high	10.9	6.2	0.3	19	9.6
PS	61	M	3	1	21.5	3.7	162	1.74	0.91	132.2	2.1	high	9.2	6	0.06	35	9.6
PS	60	F	2	1	28.8	3.5	151	1.84	0.9	102.9	2.1	high	10.8	6.4	0.04	38	10.9
PS	50	M	10	1	25.3	3.7	206	1.27	0.95	101.2	2.2	high	7.9	4.2	3.8	31	8.9
PS	36	M	5	1	17.3	3.9	167	1.27	1.28	94.99	1.8	high		4.7	0.35	23	10.5
PS	68	F	17	1	20	2.8	178	1.77	0.96	85.05	2.1	high	8.4	8.6	0.83		10.2
PS	66	M	16	1	20.2	3.5	189	1.49	1.15	124.8	1.8	high	10.5	3.8	0.49	32	8.4
PS	72	M	16	1	19.7	4.2	204	2.32	0.87	105.1	2.1	high	9.7	7.3	0.04	41	10.4
PS	35	M	7	1	18.9	4.2	126	1.44	1.06	129.3	1.5	high		5.3	0.2	22	14
PS	72	M	2	1	22.5	3.5	147	1.51	0.83	95.52	2.1	high	9.6	4.5	0.05	31	9.7
PS	62	M	9	1	23.4	3.9	183	1.19	0.88	151.9	1.4	high	7.7	6.1	10.3	36	9.5
PS	57	M	4	1	21.9	3.9	140	1.33	1.08	116.7	1.5	high	8.6	3.7	0.04	22	13.1
PS	58	M	3	1	19.1	3.8	104	1.35	1.07	135.2	1.5	high	7.8	5.9	0.09	22	11
PS	60	F	3	1	22.5	3.7	142	1.14	0.75	115.8	1.6	high	8.3	7.2			10.6
PS	51	M	2	1	23	3.5	193	1.85	1.04	70.58	2.1	high	9.5	5.9	0.03	36	9
PS	40	M	10	1	29.8	4.3	200	1.39	0.82	117.8	1.6	high	9.3	7.8			12.3
PS	73	F	6	1	21.3	3.6	170	1.72	0.82	102.1	1.8	high	8.3	5.2	0.39	27	11.4
PS	46	F	10	1	23.5	3.2	132	0.96	1.05	94.35	2.1	high	9.8	5.6			10.9
PS	38	M	14	1	21.1	4.1	225	0.97	0.82	118.9	1.8	high		4	0.05	73	10.6
PS	40	M	7	1	27.9	3.8	137	1.55	0.82	98.83	1	high		5.4		40	10.7
PS	39	M	6	1	24	4.1	113	1.87	0.79	142.8	1.5	high	9.5	4.7	0.1	26	10.7
PS	55	F	25	1	20.1	3.7	101	1.16	1.17	118	1.8	high		6.9	0.05		10.8
PS	59	M	5	1	26.6	3.9	143	1.28	0.87	90.75	2.1	high	8.3	6.3	0.08	35	9.7
PS	71	M	13	1	22.3	3.6	224	1.91	0.98	104.4	2.1	high	10.9	7.4	0.03	31	10.6
PS	55	F	19	1	17.8	3.9	111	1.34	0.71	127.1	2.1	high		4.6		24	11.7
PS	50	M	6	1	30.5	3.7	162	1.56	0.82	116.4	2.1	high	10.3	4.4	0.01	51	8.7
PS	45	M	14	1	16.6	4	100	1.35	1.17	124.3	1.6	high	8.3	4.6			10
PS	74	M	11	1	22.1	2.6	157	1.62	0.75	87.76	2.2	high		4.5	0.03	33	11.4
PS	72	M	5	1	20.5	4	158	1.32	0.94	85.94	2.1	high	8.9	6.1	0.01	27	9.7
PS	68	M	5	1	23.7	3.5	168	1.26	0.72	153.2	2.1	high	9.2	6	0.07	30	10.8
PS	35	M	10	1	18.3	4.1	167	1.41	0.95	139.4	1.8	high	8.5	6.3	0.27	25	9.6
PS	69	M	5	1	24.9	3.4	128	1.39	0.83	72.26	2.1	high	9.8	3.5	0.01	27	9.8
PS	66	M	4	1	21.9	3.7	119	1.02	0.98	103.9	2.1	high	8.6	6.2	0.01	41	9.5
PS	61	M	4	1	20.4	3.7	132	0.97	0.78	138.3	2.2	high	8.9	1.8	1.52	18	6.1
PS	77	M	12	1	26.7	3.8	173	1.39	0.91	96.61	2.1	high	8.5	6.4	0.18	29	10.6
PS	58	F	6	1	17.9	3.8	117	1.52	0.95	74.35	1.8	high	10	5.4	0.35	28	11.4
PS	67	M	11	1	23.1	3.5	176	1.67	0.76	113.2	2.1	high	9.3	6.5	11	31	8.1
PS	58	F	7	1	22	3.2	150	1.3	0.71	88	1.3	high	10	6.8		31	12.9
PS	60	M	2	1	20.5	3.9	119	1.43	0.77	103.4	2.5	high		4.8		25	11.6
PS	80	M	20	1	24.7	3.1	200	1.36	1.34	177.1	1.8	high	9	2.9			4.6
PS	48	F	12	1	16.6		118	1.29	0.71	123.4	1.8	high	8.4	5.6	0.1	22	
PS	38	M	8	1	22.7	3.5	142	2.07	1.21	107.3	1.8	high	10.1	5.9		31	7.1
PS	58	F	7	1	26.3	3.9	167	1.28	0.94	121.2	2.1	high	9.8	6.2	7.2	24	12.6
PS	72	M	7	1	38	3.5	161	1.38	0.74	98.46	1.8	high	9.4	7.9	0.3	28	11.8
PS	31	M	6	1	23	3.9	143	1.57	0.65	105.8	2.1	high	9.4	6.5	0.66	27	11.7
PS	70	F	4	1	27	3.7	159	1.58	0.82	121.2	2.1	high	9.7	5.3	2.52	30	10.8
PS	70	M	7	1	24.8	4.2		1.32	0.99	152.9	1.8	high	9.9	5.1	0.05	26	7.8
PS	58	M	29	1	21.2	3.4	104	1.65	0.83	83.4	1.4	high	8.1	4	0.8	36	10.7
PS	65	M	2	1	28.8	3.5	113	1.09	0.78	88.06	2.1	high	8.2	5.6	0.17	23	10.9
PS	29	M	24	1	22.3	4.1	179	1.6	1.09	116.7	1.8	high		3.7	0.05	26	11.1
PS	60	M	13	1	23.3	3.6	202	1.93	0.85	120.3	1.4	high	9.6	6.3	0.07		11.4

PS	37	M	13	1	23.5	3.6	180	1.6	0.95	95.43	2.1	high		5.7	0.2	24	10.5
PS	76	M	11	1	18.3	3.4	225	0.61	1.19	125.9	1.8	high	8.6	3.6			11.1
PS	47	M	4	1	21	3.4	153	1.63	1.09	106.3	2.1	high	9.4	4.9	1.24	23	10.6
PS	73	F	13	1	17	3.5	144	1.28	1.03	104.2	1.8	high	7.8	6.5	0.1	24	9.1
PS	69	F	10	1	19	3.6	157	1.09	0.84	109	2.2	high	8.3	4.2			8.1
PS	74	M	6	1	20.4	3.5	183	1.21	0.77	106.1	1.6	high	10.2	4.1			10.9
PS	60	F	2	1	19.9	4.3	142	1.3	0.97	85.75	1.8	high	7.6	6.8	0.07	30	10.6
PS	66	F	15	1	19.9	3.4	159	1.58	1.02	131.9	1.8	high	8.9	6.7	1.06		9.2
PS	61	M	15	1	17.8	4	117	1.29	1	120.3	1.4	high	9	3		32	7.3
PS	70	F	12	1	25.6	3.5	173	1.76	0.91	114.9	1.4	high	9.7	4	0.05	24	10.1
PS	79	M	12	1	21.8	3.6	162	1.29	0.84	102.9	1.8	high	8.8	4.7	0.7	29	11.2
PS	68	M	11	1	20.5	3.9	131	1.45	0.88	112.6	2.1	high		5.2	0.36	33	15.2
PS	55	M	11	1	19.3	3.8	190	1.98	1	86.75	2.1	high	8.8	7.5	0.28	23	10.6
PS	63	F	11	1	19.1	3.8	111	1.26	1.28	112.1	2.1	high	8.7	4.1	0.1	25	10.7
PS	63	M	10	1	26.1	3.8	84	0.95	0.88	125	2.2	high	8.3	4	1.43	40	10
PS	49	F	9	1	18.9	3.9	183	1.32	1.16	141.3	1.8	high		5	0.65	32	9.5
PS	61	M	8	1	21.2	3.3	105	1.18	0.56	83.76	2.1	high	8.6	4.8	17.3	37	8.8
PS	45	F	8	1	21.1	4	177	1.62	1.19	97.61	1.8	high	8.5	6.8	0	24	9.6
PS	72	M	7	1	22.4	3.2	122	0.77	0.67	92.19	1.5	high	9	4.3	3.09	27	10.3
PS	56	M	7	1	20.1	3.9	140	0.97	0.85	122.1	1.8	high	7.9	3.6	0.66	34	8.3
PS	75	M	4	1	26	3.9	126	1.78	0.89	107.2	2.1	high	8.6	5.5	0.16		12.4
PS	61	F	3	1	21.9	4	137	1.54	0.95	94.46	1.5	high	10.7	5.9	0.1	16	12.7
PS	57	M	3	1	25.3	4.3	195	1.34	0.94	127.6	2.1	high	8.1	6.1	0.53	23	11
PS	69	M	3	1	21	3.7	249	1.41	0.73	106.3	1.5	high	9.6	1.7	0.86	21	10.1
PS	47	M	5	1	20	3.7	128	1.48	0.92	107.4	2.2	high	9.9	7	0.14	27	10.6
PS	46	F	17	1	18.8	3.8	159	1.67	0.57	72.22	2.1	high		6.6	0.05	29	10.7
PS	74	M	13	1	20.1	3.7	196	1.64	0.88	145.1	1.5	high	8.8	2.7	0.04	27	8.4
PS	64	M	11	1	19	3.8	137	1.1	0.73	111.9	2.1	high	9	6.5	0.15	15	10.4
PS	59	F	10	1	17.4	4.1	143	1.06	1.15	165.8	1.8	high	8.2	5.9	0.17		9.6
PS	65	M	10	1	19.2	4.4	114	1.47	0.59	89.42	2.1	high	9.7	5.9	0	29	10.2
PS	68	F	8	1	22.1	3.9	151	1.49	1.02	136.6	1.1	high	9.6	2.3	0	21	10.4
PS	61	F	6	1	20.6	3.8		2.01	0.83	71.35	2.1	high	9.3	5.8	0.39	28	11.1
PS	66	M	3	1	24.1	3.6		1.74	0.96	93.96	1.1	high	9.4	1.9	0	23	10.2
PS	60	M	13	1	22.6	3.2	127	1.34	0.73	96.34	2.1	high	9.2	5.1	0.05	23	11.7
PS	51	F	8	1	23.3	3.7	163	2.18	0.74	105.4	1.5	high	9.2	4.3	0.1	31	7.5
PS	48	M	5	1	22.4	4	144	1.31	1.22	90.69	1.5	high	11.6	5.1	0.4	32	13.7
PS	76	F	4	1	24.3	3.9	137	1.15	0.93	100.7	2.5	high	8.5	6	0.3	35	15
PS	44	M	4	1	31.8	3.7	112	1.31	0.67	69.16	2.1	high	9.2	5.3	0.2	33	10.6
PS	58	F	3	1	21.3	3.6	154	1.63	0.83	89.7	1.5	high	7	7.1	3.71	24	7.4
PS	58	M	2	1	20.2	3.7	120	1.17	0.89	85.84	2.1	high	8.9	4.4	0.07	25	11.3
PS	62	F	20	1	18.4	3.3	160	1.47	1	106.4	1.5	high		3.7	0.05		11
PS	65	M	5	1	21.3	3.5	150	1.1	0.74	113.4	2.1	high		3.4			9.6
PS	57	M	30	1	18	4.1	161	1.69	0.83	75.61	1.6	high	9.3	4.1	0.1	27	11
PS	43	F	16	1	21.5	3.8	203	2.05	0.71	104.2	1.8	high		0.8		36	11.8
PS	67	M	13	1	20.8	4.3	135	1.55	0.69	110.5	1.8	high	9.5	6.6	0.06	46	10.3
PS	54	M	7	1	22.2	4.5	137	1.5	0.85	90.96	2.1	high	9	3.7	1.93	27	8.4
PS	65	M	5	1	23.9	4	181	1.74	0.99	99.88	1.8	high	9.5	4.4	0.05	28	9.4
PS	63	M	10	1	22.3	4	180	0.95	1.06	97.93	2.1	high	9.2	5.4	1.88	23	11.2
PS	54	M	4	1	17.6	4.2	146	1.58	0.68	81.4	2.1	high	8.3	4.2	3.1	26	10.8
PS	39	F	3	1	17.8	4.2	118	1.28	0.6	97.95	1.6	high	8.6	4.4	0.06	35	11
PS	73	M	10	1	16.9	3.8	169	1.36	0.95	109.6	1.4	high	9.2	4.6		25	8.5
PS	57	F	9	1	22.1	3.6	155	1.76	0.85	94.28	1.8	high	8.1	4.8	0.1	27	9.3
PS	73	M	3	1	20.5	3.9		1.34	0.98	86.33	2.2	high	7.9	4.2			
PS	78	F	6	1	16.3	3.2	139	1.57	0.97	85.56	1.5	high	9.2	4.6		25	10.5
PS	59	F	3	1	20.4	3.8	179	1.38	1.09	73.8	2.2	high		7.7	0.3	27	9.2
PS	73	F	3	1	21.4	3.7	186	1.45	0.91	71.66	2.1	high	7.8	10.8	1.68	26	11.7
PS	66	M	13	1	17.3	3.7	221	1.57	1	108	2.1	high	8.8	6.9	0.17		11.1
PS	75	F	7	1	16.3	3.1	104	1.66	0.92	129	2.2	high		4.4	0.1	22	11.4
PS	78	F	5	1	19.3	3.9	166	1.76	0.83	103	2.1	high	9.7	4	1.17	23	10.8

PS	61	M	2	1	28.3	3.8	136	1.5	0.87	113	2.1	high	9.5	6.3	4.12	29	11.3
PS	69	M	10	1	20.1	4	129	1.29	1.03	113.1	1.6	high		6.6		29	14.4
PS	76	F	8	1	23.2	3.6	185	1.81	0.98	150.8	1.5	high	8.8	5.4		20	10.1
PS	72	F	8	1	18.3	1.6	155	1.07	0.9	125.4	1.3	high		6	2.55	22	10.6
PS	65	M	3	1	23	3.8	180	1.15	0.83	122.1	1.5	high	8	2.1	0.37	16	9.6
PS	42	F	4	1	23.8	3.8	117	1.14	0.98	90.18	1.5	high	10.2	6.8	0.15	31	10.8
PS	27	F	9	1	17.7	3.6	126	1.24	1.24	69.58	1	high	8.6	4.4	2.53	27	10.9
PS	37	F	8	1	19.2	3.5	357	1.65	0.97	91.83	2.1	high	9.8	7.1	0.09	22	11.2
PS	28	F	5	1	19.4	4.1	97	1.25	0.85	114.8	1.5	high	8.5	3.7	0.05	35	9.5
PS	68	F	20	1	17.9	4	116	1.29	1.13	118.3	1.5	high	8.6	6.4	4.08	23	8.5
PS	64	M	16	1	20.3	4.1	140	1.34	0.92	129.1	1.8	high	10.1	4.8	0.35	34	10.3
PS	70	F	2	1	22.9	3.8	212	1.38	0.83	78.08	1.6	high		4.7	1.01	23	11.7
PS	49	M	15	1	21.2	3.6	132	1.29	0.92	91.35	2.3	high	9.6	4.1	0.04	25	11.1
PS	60	M	5	1	22.2	3.9	191	1.71	0.85	112.3	1.4	high	8.7	3.3	0.9	23	10
PS	66	M	18	1	19.5	4	109	1.25	1.17	138.8	1.8	high	9.6	5.1	0.08	17	10.5
PS	72	M	8	1	19.8	3.9	98	1.8	1.19	118.6	2.1	high	8.6	3.3	25.17	25	10.1
PS	42	M	2	1	31.1	4	200	1.66	0.89	130.1	2.1	high	9.9	3.5	3.69	30	12.1
PS	71	F	2	1	15.6	3.4	167	1.75	0.7	30.16	1.1	high		6.2	0.02	30	10.6
PS	84	F	16	1	21.1	3.3	137	1.14	0.68	93.43	1.8	high		7.3		36	11.8
PS	53	M	8	1	24.5	4	180	1.47	1.12	80.15	1.8	high	9.5	5.2	0.05	26	11.7
PS	76	M	5	1	20	4	126	1.36	1.16	81.56	1.5	high	9.1	5.7	0.2	18	8.9
PS	59	F	5	1	21.5	4.2	159	1.22	0.86	91.03	2.1	high		4.1	0.03	23	9.3
PS	75	M	2	1	18.1	3.9	152	1.52	0.93	124.2	2	high		3.8		16	12
PS	62	M	2	1	33.1	4	153	1.16	0.71	69.56	1.8	high		8.2	0	28	11
PS	65	M	28	1	18.9	4.1	168	1.41	1.08	120.7	1.8	high	10.1	7.1	0.14	29	11.8
PS	69	F	3	1	19.9	4.2	110	1.4	1.03	121.4	1.8	high	9.3	4.1	0.25	37	10.4
PS	88	F	7	1	19.4	3.2	125	1.34	1.07	123.4	1.4	high	9.4	9.2	0.22		13.9
PS	58	F	4	1	16.4	2.8	133	1.93	0.53	40.94	1.4	high	7.9	5.7	0.17	20	9
PS	64	F	8	1	21.3	3.7	171	1.15	0.96	130.3	1.5	high		7	0.1	17	8.7
PS	57	M	12	1	22.5	3.2	128	1.24	0.82		1.8	high	9.1	5.4	0.02	19	10.9
PS	67	M	5	1	22.5	3.7	198	1.94	0.82	75.58	1.1	high		3.9		22	8.7
PS	57	F	6	1	17.3	4.2	134	1.3	0.74	98.49	1.3	high	7.8	4.3		26	8.8
PS	64	F	3	1	24.6	3.8	181	1.19	0.92	80.38	1.8	high	8.7	6			10.9
PS	54	M	4	1	22.3	4.1	121	1.16	0.93	106	1.5	high	8.2	9.9	0.09	28	12.8
PS	60	M	3	1	21.3	3.6	172	1.64	0.79	110.7	1.5	high	9	5.8			12.6
PS	67	M	16	1	20.5	3	133	1.36	0.87	107.7	1.8	high	7.7	4.6	0.92	25	11.5
PS	43	F	12	1	21.4	4	139	1.4	0.81	126	1.5	high	8.6	5		21	9.8
PS	68	M	24	1	21.6	3.4	159	1.92	0.96	100.8	1.8	high	8.9	6.1	0	27	10.9
PS	67	M	12	1	23.6	3.7	219	1.2	0.94	120.7	1.8	high	9.5	3.8	0	20	10.5
PS	47	M	5	1	21.4	3.9	192	1.73	1.15	121	1.5	high	9.4	5.9	0.1	26	12.2
PS	40	M	6	1	22.7	4.3	154	1.46	0.78	100.7	2.2	high	8.6	4.7	1.1	28	9.2
PS	65	M	23	1	17	3.7		1.3	0.73	121.6	1.8	high	8.9	3.6	4	31	11.7
PS	69	F	8	1	24.5	3.6		1.34	0.63	88.13	1.3	high		2.9	0.2	41	9.7
PS	80	M	6	1	24	3.6	184	1.46	0.76	56.96	2.2	high	9	2.6	1.92	38	11.4
PS	64	F	7	1	20.7	3.5	221	1.65	0.77	83.37	1.3	high	10.2	4.7	0.04	31	8.8
PS	74	F	4	1	19.4	3.6	199	1.5	0.94	112.9	1.8	high	8.9	5.7	0.76	31	9.7
PS	45	M	9	1	17.6	4	152	0.81	0.83	102.8	2.1	high	8.2	8.7	0.54	22	10.1
PS	39	F	8	1	24.1	3.4	125	1.58	0.56	110.4	2.1	high	8.2	4.9			12.6
PS	77	M	2	1	20.3	3.7	176	1.49	0.65	100.3	1	high	9.4	7.7	1.05		10.8
PS	45	M	3	1	29.2	4.4	136	1.81	0.85	86.31	1.8	high	9.7	4.1	0.05	24	9.5
PS	60	M	9	1	25.2	3.6	99	1.2	1.22	128.7	1.8	high	9.4	5	0.05	30	11
PS	52	M	5	1	27.1	3.7	176	1.5	0.97	150.2	2.1	high	9.3	9.3	0.03	21	9.8
PS	61	M	19	1	17.9	3.5	166	1.24	0.99	107.5	2.1	high	8.8	8	0.17	30	9.9
PS	58	M	27	1	22.4	4		1.12	1.06	124.2	1.6	high	8.2	5.4	0.17	24	9.4
PS	59	F	8	1	22	3.5	172	1.68	0.88	117.3	1.5	high	8.4	6.2	0.08	59	8.7
PS	48	M	3	1	21.7	4	179	1.48	0.9	81.29	2.1	high	9	5.8	0.05	18	9.3
PS	57	M	29	1	20.3	3.2	135	1.31	0.9	114	1.8	high	9.3	5.9	0.26		10.2
PS	68	M	25	1	22.1	3.8	150	1.54	0.95	129	1.8	high	9.5	4.6	0.01		10.3
PS	62	F	21	1	18.1	3.5	202	1.76	1.16	106.5	1.5	high	11.5	4.4	0.04	27	11.6

PS	57	M	28	1	20.3	3.5	214	1.52	0.9	116.4	1.8	high	10.2	6.5	0.08		10.3
PS	59	M	34	1	19.5	4.1	189	1.34	1.17	122.5	1.9	high	9.1	5.4		33	9.7
PS	69	M	10	1	19.5	3.8	141	1.13	0.74	108.3	1.8	high		5.5	0.13	19	8.7
PS	78	M	6	1	20.7	3.4	156	1.83	0.9	133.7	1.4	high	8.9	4.4	2.3	36	9.2
PS	64	M	2	1	20.6	3.1	170	1.36	0.64	75.03	1.5	high	8.7	5.8			11.4
PS	66	M	4	1	23.4		141	1.39	0.9		1.8	high	9.6	7.6	0.2	30	10.3
PS	66	F	9	1	15.6		208	1.47	1.32	121.9	1.1	high		6.8	0.1	2	11.3
PS	61	F	16	1	18.5		130	1.46	1.16		1.4	high	9.3	5.5		26	11.2
PS	46	F	13	1	19		190	1.31	0.98		1.8	high	9	4.4	0.09	33	10.5
PS	55	F	11	1	17.4		158	1.32	1.21		1.5	high	9	5.9	0.31	23	11.3
PS	33	M	4	1	17.5		189	1.25	1.05		1.5	high	9.1	6.1	0.21	30	10.4
PS	55	F	6	1	28.8	3.9	158	1.42	1.03	118.6	1.5	high	8.5	3.3	2.19	16	10
PS	40	M	4	1	17.8		118	1.71	0.79		1.8	high	8.9	7.6	0.08	25	12
PS	68	M	6	1	18.3		182	2.14	1.11		1.5	high	9.3	6.3	0.05	25	10.9
PS	67	F	11	1	15.4		187	1.19	0.72		1.8	high	9.1	4.9	0.16	28	10.6
PS	62	F	24	1	21.4		146	1.6	1	127.8	1.5	high	8.9	5	0.52	28	10.7
PS	59	F	7	1	14.8		87	1.12	1.13		1.8	high	8.4	5.5	0.05	26	9.1
PS	54	F	8	1	18.6		178	1.28	1.48		1.6	high		3.9	0.02	26	12.2
PS	54	F	7	1	20.9		169	1.25	1.23		1.5	high	8.7	6.8	0.01	30	10
PS	75	F	12	1	25.1		152	1.36	1.1		1	high	9.3	6	0.13	26	10
PS	55	F	27	1	17.2		215	1.5	1.03		1.5	high	9	2.9	0.21	36	9.8
PS	75	F	20	1	21.6		112	1.37	1		1.5	high	9.7	7.8	0.01	20	10.4
PS	62	F	10	1	18.8		112	1.43	1.05		2.1	high	9.4	6.7	0.2	48	9.7
PS	60	M	12	1	22.4		249	1.77	1.01		1.5	high		3.8	0.52		12.9
PS	47	M	3	1	22.3		169	1.34	1.21		1.8	high	8.2	7.2	0.2	23	11.7
PS	79	F	21	1	15.8		150	1.54	0.95		1.5	high	9.4	5.8	0.04	27	10.4
PS	65	F	20	1	20.3		164	1.43	0.89		1.4	high	8	5.4		24	12.8
PS	56	M	5	1	21.8		104	1.51	0.99		1.5	high	9.4	3.6	3.95	11	8.9
PS	60	M	2	1	26.6		164	1.17	0.7		1.8	high	9.6	4.8	0.01	26	10.4
PS	35	M	4	1	23.4		119	1.2	0.98		1.6	high	9.4	5.1	0	30	9.9
PS	59	F	3	1	23.3			1.31	0.81		1.1	high	8.9	4.4		21	10
PS	50	M	4	1	24.5		165	1.59	1.12		1.3	high		3.9		26	9.9
PS	63	M	4	1	21		142	1.4	0.99		1.8	high		5.9	0.25	34	12
PS	59	F	9	1	20.6		146	1.72	0.92		1.3	high	8.9	4.5	0.16	15	11
PS	67	M	19	1	23.2		149	1.51	0.99		2.5	high	9.1	4.3	0.6	26	8.8
PS	55	F	10	1	19		114	1.34	1.13		2.1	high	9.6	3.9	0.1	27	10.5
PS	54	M	25	1	20.4		108	1.21	1.36		1.8	high	9.8	4.3	0.23	26	11
PS	52	F	28	1	17.9		168	1.36	1.1	112.7	1.8	high	10.3	6	0.1	21	11.2
PS	57	M	4	1	19.9		147	1.7	0.92		1.8	high	10	6.6	0.01	27	8.5
PS	59	M	14	1	20.5		112	1.2	0.91		1.8	high	10.1	5.7	2.16	37	12.4
PS	71	F	9	1	20		173	1.13	1.4		2.1	high	9.7	5.5	0.14	32	9.7
PS	58	F	19	1	22		171	1.21	0.97		1.8	high	9.6	4.6	0.3	26	8.1
PS	59	F	3	1	16.4		233	2.79	1.36		1.3	high	8.5	4.6		21	8.8
PS	49	M	2	1	28.8		146	1.36	1.16		1.5	high	9.9	3.6	0.2	25	10.8
PS	27	F	14	1	20.8		158	1.64	1.19		1.5	high	8.7	5.2	0.89		8.5
PS	87	F	7	1	20.8	4.1	226	1.45	1.03	114.5	1.6	high		4.2	0.12	25	10.3
PS	41	F	11	1	18.8	4.2	100	1.45	0.93	92.75	1.8	high	9.4	9.4	0.05	35	12.7
PS	59	F	27	1	15.4	4.3	148	1.76	0.72	112.5	1.8	high	8.3	4.5			8.5
PS	66	F	14	1	18	3.6	104	1.57	0.55	69.24	1.8	high	8.8	3.5	5.1	38	8.6
PS	34	F	13	1	18.2	3.7	159	0.85	1.38	128.6	2.1	high		4.3	0.06		10.1
PS	60	F	5	1	18.6	4.5	150	1.45	1.01	107.2	1.5	high	8	4.6	0.2	23	10
PS	50	M	3	1	19	3.7	172	1.81	0.97	59.92	1.8	high	8.6	4.6	0.02	31	11
PS	54	M	3	1	25.6	3.8	167	1.2	0.82	117.6	2.1	high		3.6	0.15	22	10.1
PS	63	M	4	1	18.9	4.1	119	1.23	0.96	101.7	2.2	high	7.9	4.5	0.2	22	10.5
PS	71	M	4	1	21.3	3.6	133	1.99	0.73	87.86	1	high	9.8	5.5	0.07		10.3
PS	74	M	5	1	21.8	3.8	123	1.41	1.11	119.9	2.1	high	9.2	5.6			10.7
PS	72	M	5	1	23	3.3	142	1.32	1.2	112.6	2.1	high	9.6	4.1	0.28	21	11.5
PS	45	F	7	1	25.5	3.9	182	1.22	0.95	116.4	1.3	high		2.1	0.6	30	8.9
PS	69	M	7	1	24.2	4	148	2.04	0.74	132.4	1.5	high	8.9	4.7			10.9

PS	66	M	4	1	22.5	3.7	163	1.38	0.99	98.05	1.3	high	10	4.3	0.1	26	13.1
PS	68	F	10	1	23.7	4	135	1.65	1.11	107.6	2.1	high	8.8	6.3			10.6
PS	60	M	2	1	23.1	3.9	125	1.75	0.75	83.58	1.3	high		5.1	0.03	32	9.8
PS	70	M	5	1	24.3	4.1	132	1.88	1.07	109.1	1.3	high	9	5.4			12.7
PS	60	M	6	1	19.1	3.9	218	1.58	0.73	100.4	1.5	high	10.6	5.6		27	10.6
PS	62	M	4	1	23.3	3.9	221	1.86	0.93	83.2	1.3	high	8.5	5.5			11.8
PS	38	M	20	1	16.5	4.2	157	1.27	1.23	100.4	2.1	high	8.5	5.4	0.1		11.1
PS	42	M	2	1	29.1	4.2	124	1.55	1.22	90.56	1.5	high	8.6	4.5			11
PS	61	F	20	1	17.5	4.2		1.31	1	134.9	1.8	high	7.9	8.8			12.9
PS	44	M	6	1	28.1	3.9		1.64	0.76	128.1	1.5	high	7.7	6	0.4	26	10
PS	46	F	9	1	18.7	3.6	200	1.82	0.95	86.2	2.2	high	9.2	4.8	0.04	26	10.5
PS	41	M	13	1	20.8	3.9	115	1.2	0.89	112.2	2.5	high	10.5	6.2	0.04	17	11.8
PS	43	M	15	1	24.8	3.4	180	1.2	0.78	112.9	1.4	high	9.8	6	0	21	7.1
PS	59	M	3	1	29.5	4.1	139	1.42	0.98	101.6	1.6	high	10.5	5	0.87	24	11.5
PS	58	M	2	1	24.6	3.7	120	1.56	0.73	96.26	1.5	high	8.2	5.4	0.19	28	10.7
PS	53	F	22	1	18.9	4.3	156	1.47	0.91	100.5	1.6	high	8.9	6.3	0.5	29	10.9
PS	71	F	32	1	19.3	3.2	176	1.38	0.79	116.1	1.8	high	8.7	3.3	1.42	25	10.4
PS	52	F	22	1	27.3	3.6	130	1.26	0.95	119.4	1.8	high	8.6	4.4	0.72	33	9.9
PS	61	M	12	1	20.7	3.7	124	1	1.14	105	2.1	high	9.3	8.7	0.3	33	9.9
PS	83	M	8	1	20.7	4	171	1.22	0.87	123	1.3	high	9.6	5	0.09		10.6
PS	57	F	7	1	17.9	4.1		1.43	1.14	89.85	2.1	high	8.7	8.8			10.4
PS	50	M	4	1	28	3.7	161	1.49	0.76	104.3	1.6	high		5.6	0.09	21	9.8
PS	58	F	4	1	15.4	4.4	168	1.62	0.75	75.95	1.8	high	8.3	7.2		21	12
PS	68	M	3	1	20	3.7	122	2.11	1.07	87.39	1.5	high	9.2	4.2	0.1	29	9.7
PS	52	M	3	1	22.8	3.7	165	1.63	0.76	58	1.6	high		6.1	3.38	34	11.6
PS	69	M	21	1	20.8	2.9	145	1.26	0.77	91.29	2.5	high	9.9	7.2	0.03	28	13.5
PS	79	M	11	1	23.5	3.5	117	1.84	0.88	114.1	1.8	high	10.2	5.3	0.31	31	9.3
PS	69	M	10	1	21.4	3.8	146	1.28	0.74	130.7	2.2	high		6.7	0.35		14.1
PS	58	M	9	1	17.3	3.7	238	1.71	1.03	113.4	2.1	high	8.6	6.4			
PS	78	M	6	1	23.5	3	173	1.3	0.89	131.3	2.1	high	8.7	6.2	0.18	30	11.7
PS	61	M	5	1	26.2	3.4	128	1.55	0.58	108.4	1.8	high	8.6	6.1	0.05	27	10.1
PS	67	F	17	1	23.6	3.7	149	1.37	1.05	141.8	1.8	high	8.2	6.7	0.09	30	13.2
PS	53	M	3	1	18.1	3.7	92	1.26	0.78	98.12	1.5	high	8.3	4.9	0.52	27	9.4
PS	68	M	3	1	21.7	3.7	126	1.74	0.99	85.2	1.6	high		3.6	0.11	22	10.9
PS	65	F	3	1	23.3	3.9	158	1.8	0.8	143.8	1.5	high	8.8	3.8	0.11	38	9.8
PS	83	M	3	1	20.3	3.8	163	1.73	0.76	56.92	1.6	high		5.6	0.85	22	10.5
PS	61	F	11	1	16.3	4.2	142	1.66	0.76	102	2.2	high	9.9	7.5		26	9.9
PS	49	F	28	1	20.5	3.9	210	1.59	1.08	93.68	1.8	high	7.8	4.4	1.1	23	8.8
PS	43	M	20	1	21.3	3.5	142	1.43	0.76	116	1.1	high	7.9	7.5	0.2	40	6.5
PS	67	F	20	1	18.2	4.5	157	1.11	0.95	105.9	2.1	high	8	2.6	0.15	30	12.3
PS	59	F	17	1	20.3	3.9	168	0.95	0.91	130.8	1.8	high	9.1	3.8	0.51		8.6
PS	75	M	17	1	18.9	3.7	118	1.5	0.81	151.3	2.2	high	9.2	5.2		24	10.3
PS	46	M	15	1	19.2	4.3	164	2.06	0.72	114.1	1.5	high	9.3	3.4	0.1	26	11.8
PS	74	M	15	1	20.8	4.2	175	1.25	0.92	131.6	1.4	high	9.1	6	4.1	34	6.5
PS	55	F	14	1	30.3	4.1	182	1.72	0.94	116.6	1.8	high	9.5	5.8			
PS	79	F	13	1	18.7	3.6	106	1.33	1.07	114.1	1.8	high	9.2	3.8	0.6	16	10.6
PS	60	M	12	1	20.6	3.8	132	1.54	0.8	124.5	1.8	high	8.8	4.7	0.22	30	10.1
PS	57	M	12	1	24	3.7	165	1.36	0.82	81.31	1.6	high		3.8	0.39		11.6
PS	76	M	11	1	24.7	4	132	1.23	0.92	147.3	2.5	high	9.2	8.1	0.12	18	11
PS	75	F	11	1	21.3	3.7	137	2.06	0.89	137.1	1.8	high	8.7	6.4	2.6	25	11.4
PS	72	F	10	1	22.7	3.7	164	1.28	0.82	149.4	2.2	high	10.1	6.3		29	10.4
PS	71	M	10	1	20.6	3.9	149	1.5	0.78	124.8	1.6	high	9.2	5.6	0.04	21	10.6
PS	64	M	8	1	20.4	4	139	1.77	0.91	108.5	1.3	high	7.2	4.8	0.7	27	13.1
PS	56	F	8	1	19.1	3.4	194	1.61	0.89	109.5	1.5	high	9.8	4.1	0.78	21	12.3
PS	62	F	13	1	22.4	3.5	144	1.11	0.9	133.9	2.1	high	8.3	7.4	3	27	8.7
PS	74	F	7	1	19.5	3.3	113	1.48	0.93	112.3	1.8	high	8.6	6.7	1.16	34	11.2
PS	65	F	7	1	20.7	4.1	97	1.49	0.9	122.4	1.8	high	8.5	4.5	0.4	29	9.3
PS	65	F	7	1	15.5	4.1	209	2.02	1.03	93.47	1.8	high	8.6	4.4			11.7
PS	59	M	6	1	20	4.4	187	1.16	0.8	112.9	1.5	high	9.4	4.2		23	

PS	70	M	6	1	26.7	3.7		1.06	0.8	122.4	1.8	high	9.1	3.9	0.5		12.3
PS	43	M	6	1	14.9	3.7	149	1.23	0.87	97.84	2.5	high	8.8	4	0.03	19	11.5
PS	72	F	5	1	18.7	3.9	170	1.58	0.74	121.8	1.5	high	8	2.8	0.76	23	13.1
PS	41	F	5	1	19.1	4.1	134	1.44	0.82	118	1.8	high	9.6	4.7	0.17	21	10.8
PS	89	M	3	1	25.5	3.8	181	1.23	0.73	98.98	2.1	high	10	4.8	0.05	25	10.9
PS	64	M	5	1	18	4.4	188	1.37	1.16	91.4	1.6	high	8.6	5.8			
PS	76	M	4	1	22.1	3.6		1.23	0.86	89.42	2.1	high	10.9	6.6	0	33	11.9
PS	70	M	3	1	23	3.8	118	1.81	0.95	136.4	1.5	high	9.3	3.7	0.03	37	10.5
PS	72	F	3	1	25.8	3.4	95	1.32	0.81	84.38	1.8	high	8.1	3.5	0.61	41	10.4
PS	66	M	3	1	20.5	4.1	156	1.47	0.78	110	1.3	high		6.5	0.12	33	11.4
PS	58	F	3	1	22.8	3.9	166	1.3	1.13	115.5	1.8	high	8.2	8.2	0.75	33	12.3
PS	68	M	3	1	24.7	4.4	161	1.43	0.69	73.35	1.5	high	9.3	4.4	1.5		8.9
PS	72	M	3	1	24.6	3.4		0.79	0.69	77.59	1.5	high		10.1			11.5
PS	59	M	2	1	32.6	3.9		2	0.93	62.73	2.1	high					
PS	44	M	2	1	22.4	3.9	139	1.69	0.72	89.07	0.8	high	9.3	5.9	1.6	27	11.5
PS	63	F	2	1	17.7	4	116	1.26	1.15	97.61	1.5	high		4.1	0.4		11.1
PS	60	M	2	1	27.9	4.1	152	1.52	0.68	121.5	1.8	high	9.9	4.6	0.04	29	9.1
PS	66	M	2	1	24.1	4	148	1.3	0.91	136	2.5	high		5.7	0.01	16	10.8
PS	79	M	2	1	22.2	3.7		1.77	0.84	99.31	1.5	high	8	9.8		2	11.9
PS	65	M	9	1	23	3.5	131	1.51	0.86	102.9	1.6	high	8.3	5.5	0.03		10.3
PS	59	F	37	1	21.4	4.5	165	1.96	1	107.4	1.5	high		4.6	1.68		8.7
PS	61	M	32	1	18.6	4.4	130	1.52	1.19	121.5	1.8	high	9.1	5.9	0.02	25	10.7
PS	60	M	31	1	19.2	4.3	213	1.1	0.82	101.7	1.8	high	7.5	6	0.3	24	10.7
PS	54	M	31	1	17.9	4.4		1.35	0.92	103.9	2.2	high	7.6	5.1	0.05		
PS	53	M	30	1	15.8	5	198	2.17	1.12	104.3	1.5	high	10.5	4.6		29	9.3
PS	60	M	28	1	17.3	3.5	150	1.83	0.85	89.4	1.4	high	9.7	3			11.9
PS	58	M	28	1	23.2	3.7	108	1.32	1.05	132.5	1.6	high	8	5.9	0.1		11.6
PS	54	M	27	1	20.6	4	103	1.2	0.85	113.3	2.2	high	9	4.7	0.8	21	9.9
PS	58	M	23	1	21.1	3.5	126	1.36	0.89	94.72	1.4	high	8.9	4.1	0.02		10.1
PS	57	M	23	1	19.2	4.7	114	1.33	0.95	109.6	1.5	high	9.8	5.8	0.8	24	11.3
PS	57	M	23	1	21.1	4.4	116	1.63	1.01	122	1.4	high	8.9	4.1	0.11		12.5
PS	60	M	22	1	21.4	4.5		1.46	0.9	127.3	2.5	high	8.7	4.5	0.06	23	11.3
PS	76	M	21	1	19.2	3.9	262	1.71	0.95	111.9	2.2	high	8.2	5.8	0.2	24	11.5
PS	47	M	21	1	20.5	4.3	195	1.68	1.1	104.1	1.8	high	8.6	6.1	0.2	24	10.5
PS	53	F	19	1	18.9	4.5	124	1.58	0.92	153.4	2.2	high	8.9	5.6	0.06	25	8.7
PS	51	F	18	1	20.7	4	178	1.37	1.03	118.8	1.8	high	8.8	5.2	0.02	27	11
PS	63	M	16	1	22.6	4	157	1.58	0.91	138.8	1.8	high		5.3	0.36	32	9.4
PS	44	F	16	1	19.2	4	121	1.28	1.06	128.2	1.8	high	9.3	2.2	2.71	26	8.4
PS	66	F	13	1	18.5	3.6	177	1.22	0.94	95.68	2.2	high	10.1	5.3	0.3	26	10.6
PS	61	M	12	1	19.5	4.4	165	1.24	1.03	114.8	1.3	high		4.6	0.02	24	10.3
PS	57	M	12	1	21.1	4.2	162	2.34	1.04	133.2	2.1	high	9	4.4	1.12	41	9.7
PS	52	M	9	1	20.7	4.1	169	1.07	0.84	96.33	1.4	high	9.2	5.9	0.5		10.7
PS	68	M	9	1	20.8	4.3	216	1.67	1.06	117.3	1.4	high		5.8	0.36	23	10.1
PS	61	M	9	1	22.8	3.7	149	1.31	0.88	120.9	1.5	high	8.6	4.5	0.1	14	11.6
PS	68	F	9	1	22.6	4.2	153	1.59	0.57	94.93	1.5	high		4.7		22	10.3
PS	72	M	9	1	17.9	3.8	175	1.65	0.83	133.6	1.5	high	9.4	5.5	0.1	28	10.8
PS	61	M	8	1	22.1	3.9	170	1.57	0.99	125.2	1.8	high		5.8		33	9.8
PS	58	F	8	1	24.4	3.5	167	1.32	1.19	120.6	1.8	high	9	5.4	0.1	24	10.5
PS	55	F	8	1	15.1	3.9	168	1.25	1.15	68.88	2.1	high	8.3	6.9	0.1	27	12.8
PS	46	F	8	1	25.4	4.7	152	1.4	1.1	109.9	2.2	high	10.5	5.3	0.52	24	11.2
PS	70	M	8	1	23	4.3	134	1.37	0.77	153.6	1.8	high		5.1	0.1	28	11.5
PS	65	M	8	1	21.1	4.3	151	1.02	1.03	116.8	2.1	high	8.7	8.7	0.38		11.6
PS	64	M	7	1	22.4		176	1.24	0.95	103.7	1.8	high	8.7	4.8	0.12	18	10.8
PS	63	F	7	1	22.3	3.9	91	1.79	0.89	116	1.5	high	8.8	4.1	0.1	27	9.7
PS	44	F	7	1	20.6	4.1	157	1.37	0.98	126.8	1.4	high	8.4	5.8	0.02	23	12.7
PS	33	F	7	1	19.5	4.3	140	1.4	1.04	113.6	1.6	high	9.5	6.6	0	22	10.2
PS	61	M	7	1	20.7	4.2	149	1.57	0.94	133.5	1.3	high	9.8	4	0.06	24	9.1
PS	47	M	7	1	18.9	4.1		1.41	0.87	107.8	2.2	high		6.2	1.1	30	11.2
PS	61	M	6	1	17.6	4.2	183	1.18	0.87	90.74	2.1	high	8.9	7.6	0.2	15	12.1

PS	60	F	6	1	25.4	4.2	185	1.41	0.82	90.55	1.8	high	8.1	5.5	0.1	25	9.6
PS	57	F	5	1	33.2	4.4		1.23	0.74	123.8	2.2	high	7.6	5.9	0.06		
PS	46	M	5	1	21.1	4.3	155	1.11	0.96	113.6	1.8	high	9.1	4.2	0.1		10.5
PS	57	M	5	1	24	4	145	1.4	0.95	107.8	2.2	high		5.6	0.85	27	10.7
PS	74	F	5	1	17	4.3	166	1.58	0.86	130.7	1.8	high		3.1	0.07	24	11.4
PS	57	M	5	1	22.3	4.2	127	1.16	0.91	131.1	1.8	high	9.2	5.4	0.05	39	10.5
PS	35	M	4	1	20.9	4.4	232	2.07	0.88	87.73	1.5	high	9.4	6.5	0.1	33	12.4
PS	69	M	4	1	23.3	4	186	1.68	0.94	136.8	1.8	high	8.3	5.7	0.1	30	11.6
PS	68	M	4	1	19.5	4	157	1.27	0.64	108.6	1.8	high	8	5.9	0	43	10.1
PS	62	M	4	1	21.9	3.8	167	1.6	0.74	101.2	1.8	high		4.3	0.04	27	10
PS	78	M	4	1	26.8	3.9	139	1.44	0.92	100.3	1.5	high	9.1	4.1	0.05	24	9.3
PS	55	M	4	1	26.8	3.3	142	1.12	1.06	116.4	2.1	high	9.1	7.3	0.1	22	10.8
PS	51	M	3	1	28.3	4.6	165	1.52	0.96	122.5	1.5	high	9.8	4.5	0.08	30	10.1
PS	41	F	3	1	25.5	3.3	116	1.01	0.64	73.18	1.8	high	8.1	4.2	0	18	9.7
PS	67	M	3	1	24.1	3.9	136	1.21	0.78	147.1	1.3	high	9.9	3.2	0.3	31	10.1
PS	59	M	3	1	19.8	4.3	151	1.63	0.91	108	1.8	high	8.4	6.5	0.1		11.7
PS	39	M	2	1	18.5	4.3	137	1.26	0.73	42.81	1.5	high	10.1	5.9	0.2	31	10.6
PS	79	F	2	1	19.1	4.2	150	1.08	1.05	92.7	1.8	high	8.9	5.3	0.7	33	10.7
PS	66	F	2	1	22.9	3.9	152	1.28	1.03	122.4	2.1	high		4.9	0.2	34	9.3
PS	69	M	2	1	25.3	4.1	179	1.55	0.61	106.5	1.5	high		5.1	0.07	27	10.7
PS	61	M	2	1	20.5	4	162	2.03	0.64	87.91	1.8	high	7.6	5			
PS	58	M	2	1	24.2	4.5	131	1.2	0.76	96.59	1.8	high		3.5	0.12	28	10.5
PS	56	M	2	1	19.8	4.5	140	1.43	0.89	96.53	1.3	high	9.4	5.5	11.6	39	11.6
PS	75	M	2	1	22.5	3.7	208	1.69	0.72	65.04	1.8	high	8.2	2	1.18	33	13.3
PS	52	M	3	1	31.3	3.9		1.7	0.98	96.64	1.1	high	8.4	5.5	1.34		10.2
PS	41	M	3	1	25.4	4.1	196	1.73	1.24	121.2	1.5	high		5.9	0.08	22	11.2
PS	52	M	3	1	24.3	4	118	1.57	0.91	95.68	2.1	high	8.1	4.9	0.1	25	8.1
PS	60	M	4	1	29.2	3.6	181	1.45	0.79	115.8	1.8	high	10.1	6.4	0.06		12.8
PS	45	M	15	1	19.5	4.5	132	1.04	0.96	92.17	2.2	high	8.8	4.6	0	21	10.2
PS	54	M	15	1	18.3	2.9	170	1.34	0.83	68.15	1	high	9.6	3.7	0.1	26	5.6
PS	32	F	15	1	16.3	4.4	122	1.38	0.98	93.99	1.8	high	9.9	5.2	0.1	19	11.2
PS	39	M	9	1	24	4.3	173	1.2	1.29	117	2.5	high	9.5	7.1	0.11	17	12.3
PS	64	M	2	1	28.2	4.5	158	1.05	0.74	103.5	2.1	high	8.7	4.3		22	10.7
PS	69	F	6	1	20.1	3.8		1.45	0.79	45.71	1.8	high	9.5	5	0.02	25	10.5
PS	35	M	3	1	34.5	4.1	145	2.02	0.8	58.71	1.8	high		5.4	9.1	33	10.9
PS	59	M	8	1	25.3	4	197	1.31	0.87	116.1	2.1	high	8.5	6.6	0.36	30	10.6
PS	33	F	20	1	19.7	4	124	1.68	0.69	99.02	1.3	high	8	4.8	2.16	25	9.3
PS	77	M	3	1	26.6	3.7		1.03	0.79	100.5	2.1	high	7.9	4.5		33	11.5
PS	67	F	2	1	20.3	3.7	89	1.4	0.61	56.44	1.5	high	8.5	4	0.02	24	8.2
PS	57	M	4	1	30	3.7	125	1.31	0.92	106.5	1.5	high	8.6	2.9	0.76	33	9.9
PS	65	F	8	1	20.6	3.7	201	1.69	1.01	136.7	1.5	high	9.8	4.8	1.06		11.6
PS	68	M	5	1	17.7	4.1		1.41	0.76	126.6	1.5	high	9.8	4.8	0.01	24	10.8
PS	82	F	3	1	19.4	4.2	196	1.53	0.81	65.63	1.6	high	8.7	3.6	0.1	28	12.4
PS	70	M	5	1	19.3	4	125	1.53	0.99	117.2	1.8	high	7.5	6.7	0.07	24	10.4
PS	50	M	4	1	20.7	4	83	1.42	0.82	76.09	2.1	high	8	5.3	0.1	30	11
PS	33	M	17	1	19.5	4.4	175	1.57	1.09	96.81	1.5	high	9	6.2	0.01	31	10.4
PS	66	M	8	1	22.4	3.8	141	1.38	0.69	122.8	1.8	high	9.2	4.9	0.05	29	11.9
PS	80	F	4	1	22.9	4.1	116	1.51	0.79	96.82	1.8	high	9	4.7	0.14	39	9.1
PS	57	F	21	1	19.2	4.5	106	1.43	1.09	99.44	2.1	high	9.8	5.5	0.07	25	10.6
PS	57	M	16	1	22.3	4	132	1.57	0.71	71.33	1.5	high	9.5	5		23	11.3
PS	65	M	14	1	23	4.2		1.08	0.92	138	1.1	high	8.5	1.7	0.33	32	10.2
PS	51	M	10	1	22.2	4.1	118	1.26	0.91	130	1.5	high	8	5		20	10.6
PS	59	M	9	1	19.9	4.1	215	1.45	0.8	93.89	1.8	high	8.2	3.6	0.08	34	9.2
PS	47	M	5	1	33.4	3.9	159	1.44	0.7	81.64	2.1	high	9	5.9	0.14	21	12.9
PS	64	M	5	1	20.1	4.3	133	1.21	0.79	70.7	2.1	high		6.1	0.04	33	12.2
PS	57	M	4	1	29.7	4.3	128	1.22	0.54	87.8	1.5	high	8.6	4.4	0.08	22	8.5
PS	58	M	30	1	20.9	3.7	104	1.16	0.84	74.46	2.1	high	9.4	5.9	0.09	26	13.3
PS	63	M	27	1	17.9	3.8	142	1.23	0.85	82.08	2.5	high	9.4	5	0.05	21	9.9
PS	59	M	17	1	21.4	3.9	123	1.18	1.01	115.8	2.5	high	8	8.3	0.08	25	10.5

PS	54	M	13	1	24.8	3.6	145	1.35	0.84	100.2	1.8	high	10.1	6	0.11	17	10.1
PS	56	M	12	1	24.7	4	178	1.26	0.76	74.22	2.5	high	6.1	8.8	1.37	20	9.6
PS	63	M	10	1	20.9	3.8	135	1.52	0.61	97.55	1.1	high	8	4.5	0.01	22	9.1
PS	56	M	7	1	21.4	4.1	144	1.36	0.94	117	1.5	high	8.5	3.6	0.07	37	9.3
PS	62	M	4	1	22.7	3.6		1.77	0.92	109.5	1.8	high	8.2	4.9	9.8		12
PS	75	M	4	1	23.2	4	162	1.15	0.81	100.6	1.3	high	8.9	4.8	0.13	26	10.7
PS	42	M	3	1	22.3	3.9	145	1.42	0.93	108.9	1.5	high	9.6	4.6	0.1	22	10.3
PS	59	M	2	1	21.2	4.1	98	1.03	0.68	84.33	2.1	high	9.8	6.1	0.28	35	11.3
PS	73	M	27	1	22.5	3.4	156	1.16	1.03	98.29	1.6	high	8.3	4.5	0.05		9.8
PS	70	M	26	1	20.2	3.5	127	1.22	1.09	74.5	2.2	high	8.9	5.6	0.15	28	11.3
PS	55	M	16	1	20.9	3.8	123	1.32	0.93	120.3	1.8	high		8		37	9.9
PS	78	M	9	1	19.2	3.5	174	1.76	1.09	120.4	1.8	high		3.6	0.03	22	9.3
PS	57	M	9	1	22	4.5	163	1.4	1.01	121	1.8	high	9.3	3.4	0.13	26	9.9
PS	67	F	5	1	24	3.9		1.65	1.04	128.4	2.2	high		6.6			
PS	72	F	8	1	21.4	4.2	126	1.68	1.05	127.6	1.6	high		6	0.18	20	10.1
PS	63	F	11	1	21.8	3.9	164	1.52	1.26	113.7	1.3	high	8.1	4.4	0.05	26	10.3
PS	72	M	32	1	17.6	3.7	160	1.29	0.66	80.61	1.8	high	9.8	5.8	0.59	30	10.6
PS	50	M	4	1	22.4	4.3	104	1.41	0.95	124.4	2.1	high		3.8	7.77	38	9.3
PS	54	F	6	1	27.7	3.6	129	1.22	0.72	140.3	2.2	high		7	0.12	29	10.2
PS	94	M	2	1	22.8	4	134	1.15	0.97	104.7	0.8	high	6.4	4.2	0.08	15	8.8
PS	72	F	5	1	26.7	3.7		0.98	0.81	99.18	1.8	high	7.6	4.7	1.08	31	10.2
PS	57	F	29	1	19.3	3.3		1.92	0.91	84.36	1.8	high	8.4	4.8			10.3
PS	47	M	24	1	20.3	3.4	159	1.89	0.96	103.2	1.4	high		7.4	0.6	30	10.9
PS	48	M	7	1	29.9	3.7		1.15	1.05	127.6	1.5	high		6.9			11.6
PS	57	M	3	1	31.9	4.2	242	1.47	1	139.7	1.8	high	9.9	4.6	0	16	11.1
PS	70	M	28	1	20.4	3.2	160	1.67	1	98.37	1.8	high	8.4	4.5	0.3	34	9.5
PS	59	F	18	1	17.8	3.9	137	1.44	0.95	92.8	2.2	high	9.3	3.8	5.09	31	7.8
PS	76	F	18	1	19.8	3		1.5	0.83	103	2.2	high		4.6			
PS	49	F	14	1	19.2	4.2	162	2.04	0.96	101.8	1.5	high	8.4	5.9	11.75	28	9.3
PS	61	M	7	1	19.8	4.2	183	1.41	0.88	116.7	1.4	high	8.8	6	0.1	25	10.8
PS	54	M	5	1	23	3.2	197	1.34	0.92	131.6	1.6	high	8.9	5.6	0.1		9.6
PS	44	M	4	1	35.7	4.1	150	1.06	0.9	118.2	1.3	high	8.2	6.6	0.8	32	10.8
PS	78	M	3	1	22.4	3.5	165	1.25	1.17	171.2	2.1	high	8.9	4.7	0.05	23	9.7
PS	60	M	28	1	21.2	3.3	132	1.37	0.63	93.59	1.8	high	8.3	6.8	0.18	29	10.6
PS	57	M	26	1	18.4	3.9	137	1.36	0.93	113.6	2.2	high	9.3	5.9	0.23	29	7.7
PS	49	M	11	1	20.4	3.8	141	1.53	1.06	125.7	1.3	high	8.9	4.2	0.21	30	10.3
PS	55	F	17	1	20.5	3.6	135	1.95	0.95	127.8	1.8	high	8.8	3.2	0.01	20	8.9
PS	58	F	27	1	16.2	3.2	127	1.49	0.93	43.5	1.8	high	8.7	5.8	0.57	36	7.4
PS	52	F	13	1	18.4	3.9	130	1.32	1.12	108.4	2.1	high	8.6	4.5	0.1	24	11.3
PS	46	M	17	1	20.7	3.6		1.17	0.77	132.1	2.2	high		4.6			
PS	53	M	10	1	22.1	4.1	135	1.43	0.86	102	1.8	high	8.6	6.9	0.02	36	10.7
PS	49	F	23	1	20.5	4	124	1.68	0.73	86.48	1.5	high	8.9	5.4	5.86	33	11.6
PS	41	F	14	1	21.3	3.8	100	1.57	0.86	95.8	1.6	high		4.4	0.05	27	9
PS	41	F	18	1	18.4	3.6	130	1.77	1.06	91.31	1.8	high		5.6	4.15		9.1
PS	71	M	12	1	21.1	3.6	140	1.36	0.93	135.3	2.1	high		3.3	0.17	31	10.5
PS	68	M	4	1	22.1	3.7	151	1.69	0.97	122.1	1.5	high	8.4	5.8	0.4	23	11.2
PS	66	M	4	1	23.1	4.1	201	1.4	0.75	96.56	2.1	high		6	0.51	25	13.7
PS	72	F	6	1	25.6	4	157	1.7	1	115.5	1.5	high		5.4	0.08		11.3
PS	60	M	12	1	23.6	4		1.35	0.88	138.6	1.8	high	8	3.9	0.65		
PS	32	F	3	1	14.6	4	233	1.87	1.14	108.5	1.5	high	10.3	4.1			11.3
PS	74	F	32	1	20.2	3.6	143	1.43	0.88	107.1	1.8	high	8.7	7.3	1.06		9.3
PS	66	F	11	1	19.3	3.9	164	1.4	0.68	158.6	1.8	high		5	0.03		11.4
PS	65	M	23	1	18.6	3.9	186	1.5	1.02	107.9	1.8	high	8.5	5.3	0.8	25	10.7
PS	57	M	23	1	21.1	3.8	114	2.07	1.12	132.5	1.8	high	9.1	5.1	0.32	24	9.1
PS	49	M	19	1	23.2	4.2	153	1.35	1.13	136.6	1.8	high	9.3	5.8	0.07	38	12.4
PS	42	F	7	1	17.9	4.2	159	1.27	0.52	72.16	1.8	high	9.1	4	5.05	31	11.2
PS	69	M	25	1	21.1	3.6	219	1.96	0.97	159.1	1.8	high		5.2		21	11
PS	64	M	8	1	23.9	3.5		1.79	0.78	172.4	1.8	high	11.2	5.2	0.03	26	11.8
PS	54	M	4	1	20.4	3.5	166	1.25	0.74	112.3	2.1	high		8.6	6.82	22	9.8

PS	61	M	16	1	24.8	3.5	142	1.34	0.81	146.1	2.2	high	9.4	6.9	0.14	26	12.2
PS	78	F	11	1	17	3.6	124	1.72	1.14	143.1	1.8	high		5.4	0.01		12.3
PS	67	F	5	1	17.3	3.2	136	1.5	1.05	104	1.3	high	9.3	7.6		34	
PS	56	M	25	1	22.6	3.7	136	1.53	1.03	117.8	1.8	high	8.6	6.3	0.4	22	12.3
PS	64	F	5	1	22.5	3.8	164	1.75	0.85	85.54	1.4	high	7.9	5.8	0.05	22	10.9
PS	69	M	28	1	20.7	3.6	118	1.52	1.05	114.4	1.4	high	7.3	2			10.9
PS	58	M	5	1	25.5	4.2	164	1.26	0.74	130.4	2.5	high	8.7	9.3	0.28	21	10.3
PS	61	F	11	1	15.8	3.9	156	1.35	0.94	110.8	1.1	high	10.1	4.2	0.1	24	12
PS	63	F	5	1	17.8	4	180	1.55	0.72	91.57	1.5	high	10.1	5.5	0.05	23	9.4
PS	56	M	2	1	22.2	3.9	136	1.2	0.87	107.9	2.2	high	9.3	8.7	0.2	32	10.6
PS	53	F	5	1	16.6	4	186	1.28	0.83	90.53	1.5	high		4.6	1.5	21	10.3
PS	50	M	4	1	24.2	3.8	167	1.86	0.96	127.7	1.8	high	9.3	4.5	0.41	32	11
PS	34	M	3	1	16.4	3.7	131	1.65	1.02	99.46	1.8	high	9.5	3.7		27	9.6
PS	55	F	7	1	22	4.2	192	1.54	0.74	99.26	1.8	high		5.8	0.1	31	10.6
PS	69	M	7	1	20.4	3.8	171	1.61	1.08	109.2	1.8	high	7.8	3.8	0.99	31	10
PS	54	F	7	1	20.6	4.2	175	1.17	0.94	95.7	1.3	high	9.2	5.2	0.01	27	9.9
PS	61	F	4	1	19.3	4.3	99	1.17	0.79	133.1	1.8	high		5.5		30	9.4
PS	74	F	3	1	21	3.6	138	1.19	0.76	84.54	1.8	high	8.5	6.4	0.01		10.1
PS	82	F	11	1	18.4	3.6	150	1.86	0.95	92.3	2.1	high	9.8	4.2	0.02	29	9.3
PS	45	F	16	1	16.9	3.8	125	1.27	1.06	110.1	1.5	high	8.5	5.7		29	9.9
PS	59	M	3	1	17.5	4	167	1.15	1.06	103.6	1.5	high		5.8	0.2	21	10.7
PS	83	M	6	1	21.7	3.2	192	1.68	0.91	133.7	1.6	high		5.6	0.75	26	10.8
PS	72	M	5	1	21.1	3.5	135	2.02	0.95	100.4	1.5	high	8.9	6.2	0.05	23	9.9
PS	80	F	3	1	19.1	3.8	186	0.99	0.69	102.4	1.8	high	10.7	4.7			10.6
PS	55	F	6	1	18.8	4.1	162	1.26	1.23	59.06	1.8	high		4.4	0.1	26	10.6
PS	72	M	3	1	18.2	4	148	1.33	0.63	87.83	2.1	high	8.8	4.7	0.84		12
PS	62	M	11	1	20.3	4	151	1.61	0.95	138	1.8	high	9.3	3.3	0.04	38	10.2
PS	56	F	4	1	28	4.1	174	1.49	0.8	98.51	1.8	high	9.4	5.8	0.05	26	10.7
PS	48	F	5	1	18.1	4.1	158	0.97	0.9	113.5	2.2	high	9.8	6.4		22	7.3
PS	77	F	5	1	24.5	3.4	195	1.61	0.76	93.93	1.8	high	10.4	4.7	0.02		10.2
PS	61	M	5	1	28.4	4	104	1.68	0.64	81.08	2.1	high	9	6.5	0.02		10.7
PS	68	M	5	1	22	4	129	1.55	0.9	124.4	1.8	high	9.4	5.2	4.4	30	8.8
PS	58	M	4	1	20.8	4.6	154	1.63	1.02	116.8	1.8	high	8.5	3.9	0.5	24	11.1
PS	74	M	12	1	18.7	4.2	208	1.45	1.11	99.58	1.8	high		9.2	0.62	30	11.5
PS	55	F	2	1	24	2.1	127	1.84	0.82	83.02	2	high	7.9	5.4	0.11	41	10
PS	72	M	4	1	21.6	3.7	144	1.56	0.82	96.07	1.3	high		6.1	0.05	26	10.3
PS	40	F	7	1	17.5	4.2	144	1.25	0.76	98.24	1.4	high	8	3.9	0.06	25	9.5
PS	67	M	32	1	17.1	3.2	111	0.7	0.83	88.02	1.3	high		7.1	0.1	39	8.6
PS	70	M	30	1	21.8	3.1	158	1.42	0.57	92.64	1.9	high	10.4	6.5	0.19		10.4
PS	62	F	28	1	14.3	3.2	107	1.4	1	86.37	1.9	high	8.7	2.8	5.37	32	10.1
PS	62	M	26	1	21.7	3.3	167	1.33	0.91	97.84	1.9	high		7.7	0.12	32	12.6
PS	60	F	26	1	18.1	3.6	166	1.24	0.99	93.91	2.2	high	8.9	6.1	0.69	24	10.5
PS	76	M	25	1	22.5	2.7	168	1.51	0.64	82.04	1.5	high	9.5	6.1	0.03	25	11
PS	43	M	25	1	25.8	3.4	208	1.89	0.54	78.71	1.9	high	8.3	3.5	0.5		10.2
PS	68	F	24	1	16.7	3.2	149	1.84	0.72	83.03	1.9	high	8.8	4.9	0.03		10.7
PS	48	F	22	1	18.9	3.8	317	1.86	0.98	119.5	1.8	high	8.6	4.6	0.02	28	10.1
PS	55	M	21	1	21.1	4.2	148	1.44	0.91	136.4	1.9	high		3.6	0.01	25	10.3
PS	58	M	21	1	18.2	3	122	1.78	0.77	108	1.6	high		4.8	0.08	30	12.2
PS	57	F	21	1	20.2	2.1	182	1.33	0.86	106	1.8	high		3.6	4.58	30	10.4
PS	57	M	18	1	18.8	3.9	77	1.37	1	98.65	1.5	high	8.3	6.3	9.1	25	9.3
PS	66	M	17	1	18.1	3.5	174	1.27	0.88	99.04	1.5	high	7.9	4.2	0.08	24	10.1
PS	61	F	16	1	17.5	4.3	125	1.18	0.84	127.6	1.5	high	8.7	5	0.1	18	10.4
PS	62	F	16	1	17.1	2.9	153	2.05	1.04	112.5	1.5	high	9	5	1.1	20	10.1
PS	56	F	16	1	29.9	3.7	207	1.9	0.63	112.5	1.8	high	9.3	5.9	0.3	28	11.9
PS	57	F	16	1	18.1	2.9	154	1.45	0.91	111.8	2.1	high		6.3	0.05		11.5
PS	58	M	15	1	16.8	4.3	132	1.3	1.27	98.73	2.1	high	9.6	5.3	0.1	18	10.8
PS	59	F	15	1	18.5	4.2	168	1.79	0.9	122.8	1.3	high	8.4	4	0.8	25	11.5
PS	67	F	15	1	18.4	3.5	137	1.03	0.91	97.48	1.8	high	9	5.6		19	11
PS	72	F	14	1	19.9	4.1	203	0.61	0.95	127	1.1	high	9.9	5.6	0.7	24	9.1

PS	47	M	13	1	19.9	3.8	277	1.45	0.4	77.04	1.8	high	8	10.1	2.3	32	10.4
PS	55	M	13	1	22.2	3.9	156	1.32	0.84	103.6	2.2	high	8.9	3.6			9.8
PS	73	F	12	1	15	1.5	180	1.56	0.96	127.9	1.3	high	9.8	7	0.1	21	11.2
PS	71	M	11	1	19.4	4	133	1.32	0.81	135.1	1.1	high	11	6.8	1.8	17	12.9
PS	74	F	11	1	20.9	3.1	213	1.42	0.9	127.7	1.3	high	10.2	2.9	0.2	19	12.9
PS	59	F	11	1	17.9	4.4	103	1.6	0.81	123	1.5	high	9.7	5.2	0.4	29	12.4
PS	60	M	11	1	19.2	4.1	189	1.62	0.53	91.21	1.6	high	9.6	4.9	0	25	11
PS	64	F	10	1	17.7	4.1	171	1.15	0.78	107	1.3	high	8.7	7.5	0.5	39	11.9
PS	61	F	10	1	22.9	4	135	1.31	0.69	126.8	2.1	high		6.6	0.2	23	10.8
PS	34	M	10	1	23.2	4	99	1.17	0.75	133.7	2.2	high	9.6	4.4			11.3
PS	38	F	10	1	20.7	3.7	149	1.77	0.85	129.8	1.5	high	10.5	6.6	0.21	46	9.5
PS	64	M	10	1	19.9	4	162	1.29	1.19	120.8	2.2	high		5.3	0.09	14	9.6
PS	79	M	10	1	23.6	3.8	145	1.14	0.88	116.5	1.4	high	9.4	3.4	0.6	22	5.8
PS	67	M	10	1	22.7	3.9	157	1.8	0.81	94.68	1.5	high	9.9	6.7	0.05	30	10.4
PS	76	M	10	1	19	3.1	163	1.11	0.73	82.66	1.8	high	9.4	9.9	0.14	27	8.6
PS	70	F	10	1	18.9	3.8	168	1.17	0.69	115.5	1.8	high	8.8	8.3	0.1	16	11.2
PS	60	F	9	1	20.1	4.5	122	1.6	1.04	75.14	1.3	high	7.6	5.4	0.1	19	7.9
PS	49	M	8	1	21.1	3.5	172	1.79	0.85	108.8	1.5	high	9.6	6.1	0.05	34	10.4
PS	68	M	8	1	17.1	3.6	102	1.31	0.67	103.2	1.5	high	9.4	5.5	0.7	26	9.8
PS	56	M	8	1	25.3	4.2	157	1.76	0.78	116.1	1.8	high	9	7.7			8.2
PS	76	F	8	1	19.2	3.9	136	1.55	0.97	75.19	1.1	high	10	5.9	0.3	20	13.9
PS	74	M	8	1	19.7	4.2	143	1.37	0.9	112.9	1.8	high	11.1	5.3	0.45	34	11.2
PS	77	M	8	1	21.7	2.8	144	1.22	0.55	68.57	1.8	high	9.4	4.2	0.2	34	10.7
PS	24	F	7	1	15.2	4		0.76	1.24	116	2.5	high		6.8	0.01	26	10.4
PS	73	M	7	1	22.5	4	147	1.07	0.78	116.3	1.3	high	8.9	4.2	0.2	22	10
PS	66	F	7	1	20.5	3.6	130	1.65	0.78	107	1.6	high		2.4	0.2	20	11
PS	59	F	7	1	23.4	4	145	1.25	0.83	113.8	2.1	high		3.2	0.03	22	11.3
PS	79	M	6	1	22.4	4.1		0.99	0.8	106.6	1.1	high	7.5	5.7	1.52	34	11.6
PS	73	M	6	1	20.5	4		1.78	0.99	90.06	1.1	high	9.3	6			9
PS	68	M	6	1	22.7	3.9	140	1.34	0.78	112.2	1.3	high	9.2	7.2	0.3	23	11.7
PS	62	M	5	1	24.4	3.8	181	1.49	0.73	138	1.4	high	8.8	5.5		20	9.3
PS	71	M	5	1	20.1	4.2	159	1.57	0.91	114.3	1.8	high	9.1	4.4	0.19	25	10.2
PS	66	M	5	1	26	3.8	157	1.59	0.71	71.73	2.1	high	9.9	6.8	0.15	23	10.7
PS	33	F	4	1	23.4	4.2	84	1.44	1.34	114.7	1.3	high		3.9	7.1	37	8
PS	60	M	4	1	19.9	4.2	165	1.33	0.75	116.5	2.1	high	9.4	3.3	0.1	27	10.2
PS	50	F	4	1	21.4	3.6	184	1.84	0.96	97.92	1.5	high	8.2	2.3	0	24	10.9
PS	48	F	4	1	27.2	4.2	113	1.35	0.88	113.2	1.5	high	9.2	6	0.1	20	12.9
PS	56	F	4	1	24.7	4.8		1.64	0.76	80.43	1.4	high	8.9	4.1			11.4
PS	81	F	4	1	16.5	4	105	1.23	0.9	117.9	1.8	high	8.6	5.1			10.4
PS	56	F	3	1	20.4	4.2	111	1.23	0.96	94.82	1.8	high	7.4	6.7	0.2	27	8.9
PS	75	M	3	1	24.8	2.1	121	1.19	0.88	91.1	1.3	high	9	7.2	0.05	27	8.2
PS	80	M	3	1	22.6	3.8	166	1.46	0.63	110.9	1.5	high		3.7		23	10.8
PS	63	M	2	1	19.6	3.7	145	1.63	0.77	113.4	1.5	high		6.1	0.3	29	11.4
PS	71	M	2	1	20	3.8	160	1.38	0.8	94.8	1.3	high	9.3	4			11.2
PS	69	M	2	1	21.2	3.8	186	1.33	0.64	13.81	1.8	high	9.2	5.6	0.98		8.8
PS	64	M	2	1	19.5	4.1	185	1.44	1.03	118.2	1.5	high	9.3	4.3	0.1	23	9.1
PS	58	M	2	1	19.6	3.8	198	1.36	0.72	131.2	1.8	high		2.4	2	17	9.6
PS	66	M	2	1	17.1	3.8	175	1.48	0.93	83.12	2.2	high		3.5	0.05	6	10.7
PS	64	M	2	1	23.4		177	1.51	1.08	106	1.5	high	10.1	5.5	0.2	27	9.6
PS	83	M	13	1	23.5	4.6	190	1.37	0.75	151.4	1.5	high		4.8	0.1	26	11.5
PS	78	M	5	1	24.6	3.9	113	1.27	0.66	98.38	2.1	high	8.7	6.1	0.05	26	11.8
PS	50	M	14	1	21.1	4.5	181	1.71	0.93	148.1	1.5	high	10.1	5.4	0.19	23	10.9
PS	34	M	4	1	27.1	4.6	175	1.59	0.95	138.5	1.5	high	9.1	5.1	0.52	27	9.3
PS	84	F	7	1	17.4	3.8	118	1.16	0.77	139.6	2.1	high	8.8	5.5		20	11
PS	72	M	8	1	20.3	4.3	77	1.39	0.66	115.9	1.5	high	8.4	6.7	0.03	23	12.3
PS	45	M	5	1	18.1	4.2	162	1.28	0.93	150.6	2.2	high	9.8	7.6	0.15	25	12.7
PS	59	M	23	1	18.2	4	202	1.16	0.7	73.27	1.8	high		5.4	0.48	25	10.9
PS	61	M	10	1	22.3	3.9	130	1.46	0.68	99.4	1.8	high		5.7	0.63		11
PS	44	M	6	1	19.9	4.2	146	1.47	0.77	105.3	1.8	high		6.7	0.05		10

PS	45	M	6	1	20.2	4.5	172	1.22	0.91	111.1	1.5	high		5.7	0.2	22	12.6
PS	45	M	4	1	30.2	4.1	141	1.31	0.79	101.8	1.5	high	8.4	5.1	0.12	39	12.3
PS	77	M	10	1	20	3.9	101	1.05	0.64	120.8	1.5	high	8.7	5.2	0.02	37	10.2
PS	83	F	4	1	17.2	3.7	127	1.47	0.97	103	1.5	high	8.4	4.4	0.3	33	10.6
PS	64	M	4	1	19.7	3.8	191	1.1	0.91	102.9	1.5	high	9.4	3.4	0.78		11
PS	68	M	2	1	19.2	3.9	134	1.14	0.84	87.42	1.8	high	8.1	4.8	0	22	9.7
PS	63	M	4	1	27.7	3.6	121	1.32	0.94	94.64	2.1	high	8.5	6.9	1.7	43	10.8
PS	80	M	9	1	20.7	3.6	99	1.49	0.88	144.3	1.8	high	9.5	5		31	10.2
PS	52	F	4	1	23.1	4.2	122	1.35	1.05	124.7	1.6	high	7.6	4.2	0	34	15.1
PS	58	F	8	1	22.2	4.1	141	1.09	1.17	96.4	1.8	high	7.7	4.7	0.03	22	12.4
PS	67	M	4	1	24.1	3.9	162	1.53	0.83	70.56	1.8	high	9.6	5.6	0.05	26	10.7
PS	42	M	8	1	37.7	3.8	130	1.28	1.03	115.7	1.8	high		6.1	0.25	22	10.2
PS	69	F	5	1	21.2	4.1	118	1.58	0.78	95.23	1.5	high	8.7	4.9	0.55	24	11
PS	38	M	9	1	19.9	4.2	120	1.47	0.95	124.5	1.1	high	9.7	5.4	6.27	34	11.1
PS	62	F	5	1	35	4	115	1.97	0.74	100.1	1.5	high	8.7	4.4	0.54	30	10.4
PS	73	F	10	1	17.9	3.7	123	1.19	0.84	125.3	1.4	high		4.8	1.84	45	10.5
PS	79	M	5	1	19.5	3.7	143	1.4	0.89	120	1.8	high	8.7	3.2	0.69	25	10.2
PS	60	F	5	1	22.6	3.8	209	1.18	1.16	139.1	1.8	high		4.5	2.44		13.2
PS	61	M	5	1	19.2	3.2	171	1.65	0.66	118.4	1.3	high	8.7	5.4	0.06	21	10.6
PS	64	F	4	1	19.6	3.7	129	1.36	0.8	112.2	2.1	high	10.2	4	0.1	24	10.3
PS	74	F	3	1	19.6	3.4	125	1.42	0.75	82.8	2.1	high	8.5	6.4	0.1	37	12.3
PS	58	F	24	1	17.1	4.1	145	1.42	0.93	128.7	1.3	high		7	0.05		10.3
PS	73	M	13	1	23.4	3.6	163	1.21	0.99	130		high	10.3	5.6	0.03	27	11
PS	77	M	3	1	22.5	3.5	119	1.62	0.73	84.93	1.8	high	10.6	1.1	1.35	35	8.6
PS	59	M	3	1	20.3	3.8	133	1.72	0.73	112.1	2.1	high	8.6	5.3	0.23	40	11
PS	57	F	5	1	16	4.3	173	1.29	0.59	131.5	1.4	high	10.1	3.5	0.12	27	12.4
PS	84	M	2	1	22	3.8	150	1.41	0.89	92.65	1.5	high	8.2	5.2		17	9.7
PS	58	M	18	1	20.9	4	166	1.13	0.75	105.1	1.8	high	8.7	5.2	1.59	26	9.1
PS	41	M	6	1	17.3	4.1	98	1.63	0.97	102	1.6	high	9.8	5.3	0.6	22	10
PS	72	F	5	1	23.6	4.2	106	1.25	0.83	55.33	1.8	high	9.1	4.2	0.1	26	9.8
PS	61	M	23	1	17.6	3.4	150	1.47	1.07	129.2	1.3	high	10.4	4.7	0.2	32	8.7
PS	69	F	5	1	32.4	3.8	150	1.27	0.94	100.4	1.6	high	9.3	6.7	0.07	20	10.9
PS	79	F	4	1	16.6	3.8	211	1.47	0.9	83.55	1.3	high	8.9	6.3	0.1	31	10.3
PS	69	M	8	1	25.1	3.7	128	1.19	1.04	151.4	1.8	high	9.4	6.3	0.28	32	9.2
PS	55	F	9	1	18.3	4.1	157	1.31	1.17	115.7	1.8	high	8.4	5.1	0.01	25	8.1
PS	39	M	3	1	20.6	3.4	131	1.11	1.18	91.6	1.5	high	10	4.7	0.7	30	10.1
PS	69	M	4	1	23.8	4	178	1.24	0.86	129.1	1.8	high	11.9	7.4	0.6	28	10.4
PS	59	M	15	1	23.7	3.7	135	1.53	0.74	81.25	1.5	high	11.3	7.3	0.2	27	10.5
PS	69	F	2	1	25.9	3.4	176	1.22	0.98	118.2	1.5	high	12	7	0.6	29	11.3
PS	70	M	6	1	21.8	3.5	99	1.38	0.94	157.4	1.5	high	8.2	5	0.2	29	9.9
PS	62	M	6	1	29.9	3.7	113	1	0.91	115.5	2.5	high	7.6	7.5	0.5	38	10.2
PS	57	F	2	1	18.4	4.3	161	1.67	1.07	82.59	1.4	high	9	4.4	0.1	27	10.4
PS	65	M	16	1	22	3.9	103	1.63	0.75	90.72	2.5	high	9.8	7.5	0.1	31	9.9
PS	84	F	13	1	21.2	4.1	159	1.28	0.87	108	2.2	high		6.2	0.07	25	12.2
PS	68	M	12	1	20.7	4	213	1.61	0.9	106	1.3	high	10.3	2.9	0.1	29	11.1
PS	61	M	11	1	21.3	3.7	213	1.19	0.89	97.91	1.8	high		7.2	0.11		9.3
PS	58	M	7	1	26.7	3.5	177	1.15	0.86	81.63	1.6	high	9.3	4.4	0.21	27	11.3
PS	81	M	7	1	18.3	3.1	128	0.88	0.67	91.42	1.4	high	8.9	5.5	0.01	25	10.6
PS	79	F	6	1	18.5	3.8	132	1.37	1.01	93.23	1.8	high	8.5	5.4	1.41		8.8
PS	56	F	6	1	20.7	4.2	168	1.88	0.9	105	1.5	high	8.3	6.2	0.1	21	11.2
PS	70	F	4	1	32.2	3.9	134	1.58	0.71	41.65	2.1	high	8.9	7.1	0.33	29	10.7
PS	72	M	4	1	21.7	3.8	148	1.64	0.82	104.4	1.3	high	7.5	6.7	2.77	29	8.2
PS	67	F	4	1	16	3.4	160	1.29	0.97	92.34	1.8	high		7.3	0.2	24	10
PS	68	M	3	1	24.3	3.9	161	1.79	1.08	70.32	1.8	high	9.2	3.4	0.04	27	8.7
PS	68	M	2	1	19.4	3.6	147	1.55	0.79	101.4	2.1	high	9.2	5.2	0.1	33	11.5
PS	74	F	4	1	29.7	3.7	112	1.4	0.82	105.7	1.3	high		6.4	0.5	32	12.4
PS	60	M	3	1	25.5		161	1.74	0.94	94.67	1.6	high		4.7	0.06	23	10.6
PS	59	F	7	1	25.3	3.8	145	1.38	1.03	112.3	1.8	high		6.6	0.11		11.5
PS	51	M	6	1	21	4.3	161	1.74	1.04	516.8	1.4	high	8.9	5.7	0.15	20	10.4

PS	55	M	10	1	22.4	3.7	109	1.2	0.65	97.49	2.1	high	7.6	5.1	0.06	20	10.5
PS	85	M	14	1	20	3.2	170	1.3	0.74	156.8	2.5	high	9.5	6	0.05	20	10.4
PS	58	F	10	1	21.9	3.6	165	2.1	0.93	82.51	1.5	high	8.8	3.1	0.1	18	9.2
PS	74	M	3	1	21.8	3.2	162	0.9	0.55	115.3	2.1	high	8.1	5.6	0.24	20	10.3
PS	58	F	14	1	20.3	3.5	152	1.52	0.99	127.8	2.2	high	9.8	6.1	1.2		9.4
PS	73	M	7	1	17.6	3.7	122	1.25	0.95	129.7	2.1	high	9.9	7.9	7.56		11.4
PS	65	M	3	1	20.3	3.8	123	1.32	0.77	88.8	1.5	high	8.3	4	1		10.9
PS	60	M	11	1	21.5	3.5	188	1.78	0.72	123.9	1.5	high	9.6	3.6	0.03	29	12.9
PS	66	M	10	1	17.5	3.9	107	1.66	0.8	91.1	2.2	high		4.4	0.07	26	10.2
PS	69	F	18	1	22.6	3.1	144	1.07	0.76	104.2	1.8	high	8.6	3.6	0	30	9.8
PS	65	M	5	1	21.3	3.7	149	1.67	0.87	159.7	1.8	high		4.2		33	11.3
PS	68	M	4	1	21.1	3.2		1.56	0.85	107.9	1.8	high	9.5	6.2	0.26	34	10.7
PS	71	F	26	1	19.6	3.6	118	1.45	0.79	120.4	1.8	high	9.8	4.2	0.03	23	9
PS	70	M	23	1	19.5	3.8	137	1.71	1.16	127.7	1.3	high	9.6	5.6	0.1	20	
PS	67	M	20	1	19.5	3.8	213	1.19	1.15	149.4	2.5	high	8.9	8	0.06	17	11
PS	66	M	17	1	19.6	3.9	138	1.07	0.85	110.8	2.5	high	8.7	5.5	0.49	20	9.9
PS	70	M	15	1	21.8	2.4	168	1.23	0.75	96.96	1.8	high	9.1	6.1	0.08		9.2
PS	61	M	14	1	18.6	3.8	160	1.1	0.92	105.3	2.5	high	6.7	6.5	2	25	12.2
PS	78	M	11	1	17.2	3.9	182	1.2	1.23	129.7	1.8	high	8.9	3.7	1.01		9.6
PS	60	M	10	1	18.7	4.1	192	1.75	1.18	146.2	1.4	high	8.8	3.8	0	21	11.3
PS	70	M	10	1	16.5	4.3	192	1.49	0.8	123.2	2.2	high	9.5	4.5	0.27	22	11
PS	60	M	7	1	23.3	3.7	150	1.13	0.93	137.2	1.5	high	9.5	7	0.03	18	9.2
PS	65	F	7	1	17.9	4		1.48	0.94	98.51	1.8	high	9.3	6.1	0.3	30	11.6
PS	79	M	4	1	22.7	3.4	119	1.61	1.06	83.35	1.4	high	8.2	3.7	0.35	29	10.3
PS	74	M	4	1	23.8	3.8	182	1.53	0.81	99.76	1.8	high	7.5	5.6	0.19	21	11.7
PS	69	F	4	1	23.7	3.9	132	1.37	1.09	88.15	1.3	high	8.9	4.4	0.18	20	10.9
PS	73	F	3	1	19.3	3.8	178	1.22	0.7	137	1.5	high	9.2	5.1	0.58	25	11.4
PS	69	M	2	1	19.4	3.6	185	0.87	0.95	105.9	2.1	high	9.7	5.2	0.09	26	11.1
PS	63	M	7	1	19.4	4	113	1.47	1.05	109.2	1.4	high	8.8	4.4		37	
PS	63	M	4	1	33.1	3.2	147	1.38	0.77	86.73	2.1	high	9.4	5.2	0.05	23	11.1
PS	39	M	4	1	27.1	3.9	162	1.69	0.8	75.15	1.3	high	10.5	6.3	0.04	29	11.8
PS	62	M	2	1	22.5	3.9	93	1.38	0.81	67.9	1.8	high	8.8	4.7			5
PS	43	M	3	1	20.8	4.1	154	1.28	0.88	104.7	2.1	high		4.1	0.31	29	10.7
PS	54	M	6	1	23.5	4.1	186	1.93	1.28	108.2	2.1	high	8.4	5.2	0.4		10.2
PS	60	F	30	1	17.6	3.8	173	1.24	1.15	113	2.2	high	9.4	4.7	0.05	20	11.1
PS	77	M	24	1	20.9	3.4	129	1.6	0.48	110.4	1.6	high	8.4	6.1	0.05	26	11.1
PS	60	M	37	1	20.4	3.4	198	1.42	0.95	95.17	1.8	high	9.6	5	0.1		12.2
PS	62	F	20	1	19.9	3.7	143	1.37	0.79	115.6	1.8	high		3.7	0.3		9.4
PS	62	F	22	1	25.6	3.8	117	1.87	0.91	163.6	1.3	high	8.3	5.3	0.05	27	9.9
PS	48	M	22	1	20.9	3.7	131	1.25	1	87.71	1.6	high		5.1			11.2
PS	60	F	30	1	16.3	3.5	127	1.32	1.18	128	1.1	high	9.3	5.5	0.18	28	9.8
PS	81	M	18	1	23.1	4.1	206	1.2	0.75	148.6	1.6	high		5.3			10.1
PS	49	M	21	1	23	3.9	205	1.16	1.1	130.8	1.6	high	7	5.7	0.86	38	13
PS	66	M	24	1	20.6	3.8	129	1.15	1.12	116.2	2.2	high	8	5.4	0		10.7
PS	51	M	21	1	20.3	4	124	1.2	0.99	130.2	1.9	high	9.6	4.9	0.12	26	8.5
PS	72	M	5	1	24.9	3.6	152	1.76	0.69	112.1	1.6	high	6.5	3.9	0.53	32	10.6
PS	56	M	26	1	17.7	3.9	114	1.84	1.12	88.21	1.8	high	9.9	5.4	2.43	29	10.4
PS	63	M	5	1	23.9	3.7	124	1.31	0.73	131.9	1.3	high	7.8	4	0.05	47	10.3
PS	31	M	16	1	30.2	4.1	149	1.39	1.03	97.96	2.1	high	8.9	6.7	0.05	17	11.2
PS	80	F	11	1	20.8	3.5	118	1.64	0.86	128.6	1.3	high	8.7	5	0.02	21	10.2
PS	67	M	17	1	22.7	4	262	1.99	1	138.1	1.8	high	9	6.7	0.1	32	10.9
PS	72	M	14	1	21.9	3.5	185	1.5	0.82	107.9	1.5	high	8.5	6.1	0.1	30	12
PS	72	F	15	1	15.2	3.8	154	1.22	1.31	90.49	1.5	high	8.2	5.8	0.1	30	14.8
PS	81	F	4	1	18.6	3.9	117	1.19	0.85	98.36	2.1	high	8.7	6	0.33	33	10.5
PS	60	F	10	1	21.8	3.7	91	1.24	1.1	132	1.3	high	9.4	4.9	0.01	32	9.3
PS	79	M	15	1	22.7	3.5	167	1.59	0.84	110.8	1.6	high	8.5	4.1	0.08	20	9
PS	72	F	15	1	16.6	3.9	151	1.53	0.91	149.8	1.3	high		7.4	0.8	32	10.7
PS	66	M	14	1	28.9	3.8	185	1.68	0.74	112.7	1.5	high	9	5.2		23	10.8
PS	72	M	13	1	19.7	3.8	176	1.4	0.81	109	1.5	high	9	5.1		29	9.5

PS	59	M	11	1	19.3	3.9	158	2.18	1	92.19	1.5	high	8.9	5	0.64	30	7.8
PS	51	M	10	1	19.4	3.9	144	1.59	1.04	124.2	1.6	high	10.1	5.2		26	
PS	58	F	10	1	21.4	3.9	116	2.18	0.78	103.7	1.8	high	9.4	5.2	0.05	23	9.6
PS	52	M	20	1	24.1	4	163	1.58	0.92	117.4	1.8	high	7.9	5.1	0.14	30	11.1
PS	41	M	9	1	21.4	4.3	156	1.45	0.87	130	2.1	high		6.6	0.2	25	11.7
PS	52	M	8	1	25.2	3.6	159	1.71	0.84	88.08	1.8	high		5.1	0.05	27	10.9
PS	62	M	8	1	22.4	3.9	122	1.35	0.94	31.1	2.1	high	10	8.7	0.81	2	10.3
PS	35	F	8	1	17	4.1	166	1.68	1.3	81.82	1.3	high	8.9	3.3	0.03	30	10.6
PS	58	F	8	1	22.6	4	187	1.62	0.95	150.2	2	high	9	2.9	0.03	18	10.7
PS	44	M	7	1	23.3	3.9	191	1.4	0.89	114.2	2.1	high		6.8	0.6	24	10.7
PS	61	M	7	1	20.7	3.9	172	1.82	1.02	111.2	1.8	high	9.5	4.9	1.45	32	10.6
PS	72	F	6	1	25.5	3.9	114	1.29	0.86	108.4	2.1	high	9	5.2	0.15	34	10.9
PS	72	F	6	1	21.8	4		1.13	0.75	94.29	1.8	high	8.6	5.4	0	27	8.4
PS	44	M	5	1	23.8	4.3	141	1.29	1.08	107.1	2.1	high		5.4		29	9.7
PS	60	F	5	1	29	3.8	171	1.71	0.71	87.37	1.5	high	9.7	6.2	2.3	31	10
PS	76	F	4	1	20.5	3.7	47	1.53	0.98	118.9	1	high	6.9	3.5	3.7		9.1
PS	66	F	3	1	22.6	3.7	209	1.37	0.76	93.51	1.5	high	8.6	5.2	0.19	28	9.6
PS	78	F	3	1	25	3.6	144	1.29	0.9	140	1.3	high		6.5		35	11.3
PS	87	M	3	1	17.7	3.5	139	1.43	0.84	76.05	1.9	high		5.1		34	10.8
PS	72	M	2	1	22.1	3.7	152	1.45	0.99	91.41	2.1	high	10.8	7.9		22	11.2
PS	60	F	3	1	33.1	3.6	131	1.73	0.88	104.1	1.5	high	9.4	3.8	0.08	25	10
PS	48	F	11	1	20.3	3.4	133	1.45	1	103	2.1	high	10.3	3.3	0.18	22	9.6
PS	34	M	3	1	22	4.1	126	1.25	0.77	107.7	1	high	8	2.8	5.5	32	10
PS	79	F	2	1	18	3.7	153	1.49	0.74	100.9	1.5	high	8	3.9	0.38	23	8.4
PS	39	M	2	1	24.9	4.1		1.37	0.96	102.3	1.8	high	9.3	5.6	0.12	26	11.1
PS	41	M	2	1	18.5	4.1	166	2.12	0.9	77.02	1.5	high	8.6	7.4	0.3	27	10.1
PS	47	M	2	1	27.1	4.4	198	1.68	0.82	89.99	1.5	high	8.8	3.8	10.4	34	9.8
PS	77	F	2	1	19.3	3.8	130	1.74	0.88	115.8	1.8	high	8.6	4.8	0.03	32	12
PS	58	M	2	1	26	4.1	141	1.56	0.86	87.34	2.1	high	9.2	6.1	0.1	24	11.4
PS	77	M	4	1	22.7	3.7	213	1.69	0.95	92.48	1.3	high	8.6	3.8	0.04	28	9.6
PS	69	M	31	1	19.2	3.9	153	1.07	1.23	87.62	1.8	high	9.7	5.9	0.2	24	12.1
PS	71	M	14	1	23.4	4.1	151	1.35	0.77	85.93	1.9	high		6.5		20	10.7
PS	27	M	3	1	22.2	4.3	177	1.85	1.11	120.6	2	high	9.3	2.7	0.28	22	9.1
PS	56	F	27	1	18.9	3.9	188	1.43	1.09	80.78	1.6	high		5.3		19	9.1
PS	39	F	18	1	34.7	3.7	132	1.4	0.93	102.2	1.3	high	9.2	4.8	0.2	33	11.1
PS	65	M	14	1	17.9	3.7	154	1.51	0.96	101.5	1.3	high	10.5	3	0.12	30	9.9
PS	51	F	14	1	18.9	4	167	1.28	1.18	107.2	1.5	high	8.8	4.7	0.1	26	10.4
PS	58	F	13	1	22.9	3.9		1.64	1.09	124.4	1.8	high	9.7	6	0.27	32	11.5
PS	45	F	12	1	17.9	4	130	1.68	1.23	105.8	1.6	high		6.4	0.74	24	11.1
PS	66	M	12	1	19.3	3.8	161	1.74	1.02	109.1	1.5	high		5.5	0.09	31	9.7
PS	72	M	12	1	19.9	3.9	143	1.78	0.98	108.2	1.6	high		4.8	0.05	27	10.2
PS	60	F	11	1	20	4.1	121	1.39	0.98	125.1	1.5	high		5.2		25	9.7
PS	64	F	11	1	19.7	4.1	211	1.52	1.09	110.6	1.3	high		6.3	0.19	22	10.6
PS	56	M	10	1	24.3	4	115	1.33	1.03	131.6	1.6	high		5.1	0.06	31	10.5
PS	55	M	10	1	20.8	3.9	93	1.62	1.11	91.73	1.3	high		7.2	0.05	37	11.2
PS	55	M	10	1	22.4	3.9	183	1.41	0.72	92.45	2.1	high	9.1	5.1	1.5	22	12.2
PS	61	F	9	1	24.5	4.1	167	1.13	0.78	127.5	1.5	high	10.1	6.4	0.08	28	12.1
PS	74	M	9	1	21.3	3.5	266	1.41	0.54	116.4	1.6	high		5.2	0.26	23	9.6
PS	48	M	8	1	23.1	4.2	101	1.41	0.76	101.2	1.3	high		4.7	1.95	47	11
PS	58	M	6	1	19.7	4.1	141	1.79	1.07	113	1.6	high	9.9	5.8	0.32	30	11.8
PS	35	M	6	1	18.6	4.1	153	1.83	0.84	117	1.8	high		2.4	1.2		7.3
PS	59	F	5	1	18	4.1		1.52	1.3	107.5	1.3	high	10.7	4.9	0.08	22	10
PS	65	F	5	1	18.6	3.8	128	1.82	0.77	84.88	1.8	high		3.9	0.17		10.4
PS	75	M	5	1	21.3	4	120	1.62	1	97.7	2.1	high	10.6	6.9			
PS	50	M	5	1	18.4	3.8		1.49	0.89	97.12	1.5	high	8.8	5.3	0.04	17	10.1
PS	49	F	5	1	22.8	3.8	189	1.95	0.92	79.95	2.1	high	8.9	6.4	0.6	27	10.9
PS	48	M	5	1	25.1	4.3	117	1.17	0.73	97.83	1	high	8.7	5.4	1.39		13.3
PS	61	M	4	1	17.6	4.2	102	1.22	0.97	91.76	1.3	high		6.8	1.96	33	10.2
PS	53	M	2	1	27.2	4	179	1.68	0.87	97.26	2.1	high	8.5	3.7	3.15	28	10.5

PS	75	M	2	1	18	4	79	1.27	0.79	42.39	1.6	high		4.7	4.38	50	6.4
PS	36	M	18	1	18.9	3.6	187	1.45	0.81	66.83	1.5	high	11.1	4.7	0.2	27	11.5
PS	63	M	4	1	24.1	3.6	127	1.28	0.85	130.6	1.5	high	9.3	6.4	1.6	30	12.8
PS	63	M	5	1	26.2	3.7	183	1.46	1.03	116.3	1.5	high	11.7	4.4	7.78	24	11
PS	67	M	3	1	21.7	3.8	132	1.63	0.65	67.11	1.6	high		2.9	0.12	24	9.4
PS	70	F	10	1	21.3	3.3	180	1.44	0.89	134.4	2	high	9.1	7.1	0.02	29	11.6
PS	64	M	8	1	23.2	4.1	112	1.24	0.85	144.9	1.8	high	9.5	6	0.08	33	10.3
PS	20	F	4	1	19	4.2	148	1.48	0.86	97.68	1.8	high		4.9	0.07	26	11.1
PS	64	M	11	1	22.4	3.7	98	1.2	0.86	140.5	1.3	high	9.5	5.1		23	10.3
PS	63	F	3	1	16.6	3.4		1.48	0.92	131.1	1.3	high	9	4.5		13	
PS	66	F	16	1	21.3	3.5	155	1.51	1.07	116.9	1.6	high	10.7	8.1	0.32	24	11.1
PS	73	F	10	1	19.7	2.2	245	1.77	0.67	81.22	1.3	high	9.1	5.6	0.2	28	11.2
PS	67	M	2	1	21.8	2.7	199	1.67	1.07	102.3	1.3	high	9	4.9	0.31	26	11.1
PS	42	M	6	1	22.3	3.7	127	1.19	0.92	113.3	1.3	high		3.6	0.05	28	9.3
PS	69	F	29	1	16.9	4	105	1.46	0.91	93.75	1.4	high	9	4.8	0.2		10.9
PS	61	M	36	1	21.6	3.2	117	1.55	0.91	62.01	1.4	high	9.8	5.7	0.07		11
PS	59	F	31	1	18.1	3.9	132	1.52	0.79	82.82	1.8	high	8.4	3.9	0		9.3
PS	60	M	20	1	18.5	4	145	1.31	0.81	108	2.1	high	10.2	4.3	0.27	35	11.5
PS	57	F	19	1	18.4	4	190	1.06	0.8	107.2	2.1	high	8.4	4.3	0.02	27	11
PS	72	M	18	1	20.2	4.1	191	1.91	0.84	117.1	1.5	high	8.8	4	0.21	28	9.3
PS	61	M	17	1	22	3.8	170	1.86	0.98	102.2	1.5	high	9	5.1	0.02	30	9.5
PS	64	M	17	1	21.8	3.9	118	1.83	0.95	126.3	2.1	high	10.1	8.5	0.06	31	10.7
PS	59	F	16	1	17.2	4	144	1.6	0.8	106.8	2.5	high	8.7	8.5	0.18	25	9.5
PS	45	M	15	1	20.1	3.8	153	1.17	0.98	120.9	1.5	high	9	5.9	0.02	24	8.7
PS	62	F	13	1	24.8	3.7	139	1.31	0.91	92.74	2.1	high	9	6.3	0.03	23	9.6
PS	56	M	12	1	21.5	3.9	169	1.43	0.77	121.3	2.1	high	9.5	4.3	0.1	31	10
PS	61	F	11	1	23.2	3.8	202	1.85	0.87	92.54	1.5	high	9.4	4.3	0.59	30	9.8
PS	61	M	8	1	22.7	4	145	1.79	0.91	124.4	1.5	high		4.7	0.07	26	10.9
PS	45	F	8	1	22.9	4.1	186	1.44	0.92	145.1	1.5	high	9.8	6.7	0.1	25	9.2
PS	59	M	6	1	23.7	3.8	152	1.26	1.13	116.9	2.1	high	8.3	4.9	0.56	28	10.7
PS	55	F	6	1	19.6	4	119	1.11	0.98	85.36	2.1	high	8.8	7.2	0.04	25	10.7
PS	36	M	5	1	26.2	3.8	92	1.77	1.14	105.6	2.1	high	7.8	2.8	0.12	21	9
PS	76	M	5	1	22.1	3.8	193	1.99	1.04	124	2.1	high	9.6	4.8	0.04	26	11.9
PS	61	M	5	1	26	3.4	122	1.22	0.58	92.96	2.1	high	9.1	6.1	0.15	20	11.3
PS	28	M	4	1	17.8	4	126	2.08	1.04	106.2	1.5	high	9.7	6	0.9	33	13
PS	75	M	4	1	22	3.9	133	1.48	0.99	101.1	2.1	high	10	5	0.05		13
PS	77	M	3	1	19.7	3.8	188	1.28	0.74	135.9	1.8	high	8.5	5.4	0.58	13	8.5
PS	66	M	2	1	23.8	3.8	119	1.37	0.95	70.35	2.1	high	7.9	3.9	0.13	13	11.1
PS	73	M	3	1	24.2	3.7	124	1.3	0.79	134.7	1.6	high		5.4	0.05	23	10
PS	62	F	11	1	21.4	3.8	158	1.76	0.77	99.88	1.5	high	8.7	4.9	0.03	27	9.6
PS	55	M	10	1	20.3		198	1.32	0.96	129	1.5	high	9.2	6	0.2	24	12.9
PS	68	M	6	1	23.8	4.2	126	0.6	0.87	98.8	2.5	high	8.9	9.7	0.26	20	10.8
PS	37	M	3	1	29.2	4	142	1.23	0.85	115.5	1.5	high	8.2	5.8		45	8.6
PS	59	M	11	1	22.2	3.9	178	1.43	1.37	149.1	1.5	high	7.7	3	0.15	28	10.8
PS	59	M	5	1	21.7	3.3	179	1.46	0.78	86.44	1.8	high		5	0.05		
PS	61	M	6	1	28.1	3.8	163	1.38	0.89	96.77	2.1	high	10.2	5.6	0.1	21	8.6
PS	45	F	12	1	28.6	3.9	141	1.68	1.27	129.9	1.3	high		3.9	0.18	29	
PS	62	M	17	1	22.4	4	133	1.31	1.08	146.4	1.6	high		5.3	0.13	32	9.7
PS	73	F	8	1	21.8	3.7	132	1.39	0.77	109.1	1.3	high		4.4	0.06	23	11.9
PS	60	M	4	1	21.8	3.8	151	1.22	1.22	130.7	1.6	high		5.6	0.05	17	10.7
PS	37	M	7	1	19.1	4.2	151	1.87	0.88	77.85	1.8	high	8.5	7.2	0.13	37	10.8
PS	47	F	10	1	25.5	3.8	188	1.56	1.36	104.3	1	high		6	0.22	26	9.8
PS	53	F	10	1	19.3	4.2	160	1.13	0.94	121.8	1.6	high		5.8	0.11	25	10
PS	72	M	4	1	24.2	3.8	155	1.26	0.86	127.1	1.3	high		2.9	0.62	24	9.8
PS	75	M	7	1	19.9	3.7	116	1.27	0.88	102.7	2.1	high		7.7	1.12	31	9.9
PS	67	M	3	1	20.9	3.8	146	1.52	1.04	126.8	1.6	high		5.5	1.5	30	7.9
PS	42	F	11	1	31.4	3.8		1.35	0.94	130.6	1.6	high		5.9	0.1	30	10.7
PS	41	F	2	1	27.3	3.4	174	1.16	0.89	32.95	1.8	high	6.7	8.1	0	25	10.8
PS	54	F	17	1	17.9	4.2	152	1.43	1.46	114.7	2.5	high		8.8		32	11.7

PS	67	M	10	1	22.9	3.9	202	1.71	0.73	123.8	1.6	high	9.9	4.8	0.08		13.6
PS	58	M	10	1	22.4	4.3	141	0.88	0.71	106.1	1.5	high	9	4.2	0	24	11.6
PS	60	M	10	1	19.6	3.7	176	1.81	0.91	116.1	1.8	high	8.8	6.9	0.02	27	9.8
PS	58	M	7	1	24.6		124	1.33	1.3		2.1	high	8.8	5.5			
PS	63	F	7	1	18	3.9	140	1.09	0.99	145.6	2.1	high	8.3	6.4	5.81		10.2
PS	55	M	4	1	30.8	3.7	120	1.62	0.85	120	1.5	high	8.1	3.6	0.68	29	7.9
PS	34	F	3	1	17.3	3.9	134	1.28	1.37	107.4	1.3	high		1.3	3.39	33	13.5
PS	57	M	3	1	18.5	4.4	166	1.5	0.82	104.8	1.5	high	9.2	5.1	0.09	17	9.4
PS	57	F	2	1	20	3.8		0.86	0.96	121.5	1.8	high	10.3	6.2	0.03	27	11.2
PS	43	M	13	1	22.6	3.7	173	1.29	0.94	128.5	1.8	high	9.2	4.9	0.05	25	9.4
PS	66	M	13	1	20.3	3.5	99	1.04	1.1	141.3	1.8	high	7.7	5.1	1.34	29	8.1
PS	71	F	7	1	20.6	4.3	157	1.53	1.06	128.6	1.5	high	10.1	5.2	0.07	38	10.5
PS	58	M	4	1	20.2	3.7	101	1.56	0.8	99.22	1.5	high	8.2	3.3	0.22	21	8.6
PS	66	F	2	1	33.7	3.6	170	1.22	0.52	90.88	2.2	high		5.1	1.48	19	12.6
PS	68	M	5	1	22.2	3.8	168	1.51	0.98	71.88	1.5	high	9.3	6	1.2	21	10.6
PS	48	M	14	1	22	3.9	137	1.98	0.95	147.3	1.3	high	8.8	5	0.27	37	10
PS	79	F	3	1	17.1	3.9	154	1.58	0.68	42.77	2.1	high		6.7	0.16	27	10.8
PS	61	F	10	1	27	3.8		0.99	0.72	96.73	1.6	high		6.1	0.66	18	9.4
PS	57	M	4	1	26.6	3.8	164	1.67	0.7	92.88	1.1	high	9.4	4.3	0.18	33	8.6
PS	64	M	4	1	20.4	4.3	123	1.52	0.83	81.7	1.5	high		7.6	0.01	20	14.6
PS	68	M	2	1	18.7	4.1	89	1.49	0.72	78.93	1.5	high		4.3		20	10.1
PS	56	M	5	1	34.9	3.5	95	0.89	0.8	112.6	2.1	high		4.3	0.06	25	8.8
PS	65	M	7	1	21.5	3.9	167	1.43	0.74	117.5	1.5	high	8.1	5.1	1.79	26	10.1
PS	52	F	5	1	21.2	3.9	141	1.64	0.93	128.2	1.5	high	9.6	8.7		29	8
PS	36	F	2	1	32.5	4.4	162	1.32	0.75	79.64	2.1	high	8.8	4.3	0.6		11.6
PS	56	M	3	1	24.3	3.6	189	1.33	0.85	100.5	2.1	high	10.7	7.9	0.45	36	13.1
PS	37	M	6	1	25.1	3.8	135	1.08	1.05	141.2	1.8	high	8.2	3.4	15.53	22	9.2
PS	74	F	9	1	18.8	3.8	152	1.1	0.76	124	1.8	high	9	3.3	0.03	14	10.3
PS	60	F	31	1	18.9	3.7	152	1.51	0.83	100.6	1.5	high	9.2	4.4	0.04	20	11.1
PS	50	F	4	1	17.7	4	133	1.3	0.96	139.4	2.5	high	8.3	6.8	0.02	35	10.6
PS	72	F	5	1	17.5	3.7	163	1.19	1.18	118.1	1.6	high	8	4.4	0.01	27	9.4
PS	58	M	7	1	20.4	3.8	178	1.35	0.9	104.9	1.5	high	9	7.1	0.1		13
PS	57	F	6	1	26.5	3.7	152	1.5	0.94	99.24	2.1	high	9.3	4.4	0.1	28	9.3
PS	49	F	8	1	21.8	3.8	138	1.27	1.22	114	1.8	high	8.6	7.6	0.02	21	11.2
PS	61	F	20	1	20.2	4.3	167	1.42	1.07	124.1	1.3	high	9.1	5			11.4
PS	50	M	8	1	20.1	4.4	139	1.37	1.15	118.2	1	high	8.9	5.3	0.15	36	12
PS	57	M	15	1	21.3	4.2	205	1.48	1.03	123	1	high	8.5	3.8			11.4
PS	48	M	16	1	21.8	4.1	146	1.6	1.04	119.1	1.3	high	9.6	5.5			10.6
PS	59	M	23	1	19.6	4.1	145	1.33	0.95	81.87	1.3	high	9	5.5			11.3
PS	60	M	7	1	20.6	4.2	126	1.5	1.07	144.5	1.5	high		5.2	0.83	14	10.8
PS	70	M	7	1	23.8	3.6	148	1.15	0.82	128.8	1.5	high	10.2	6.5	0.1		11.3
PS	72	M	12	1	22.1	3.5	185	1.61	0.89	98.86	1.3	high	9.9	4.9	1.5		9.2
PS	50	F	14	1	26.9	4	159	1.26	0.95	98.12	1.3	high	9.1	4.3			12.2
PS	58	F	7	1	24.5	4	201	1.48	1.16	132.3	1.3	high	8.8	7.4	0.1		11.8
PS	42	M	6	1	33.6	4.3	155	1.49	1.12	127.4	1.6	high		1.7	0.05	21	8.8
PS	51	M	18	1	19.4	3.9	129	1.27	1.08	95.39	1.6	high		4.6	0.05	28	8.3
PS	66	F	20	1	21.7	3.7	142	1.4	1.26	109.6	1	high		3.1			8.4
PS	61	F	34	1	19.4	3.6	105	1.85	0.85	90.12	1.3	high		6.7			9.5
PS	67	M	5	1	23.4	3.6	141	1.77	1.21	154.7	1	high		6			9.8
PS	57	M	31	1	27.5	4	146	1.27	0.97	104.8	1.6	high		5.8			9.2
PS	52	M	27	1	22.7	3.8	147	1.88	0.79	96.45	1	high		3.1			8.2
PS	62	F	18	1	18.5	3.7	130	1.37	0.71	94.8	1.6	high		4.8			10.7
PS	72	M	27	1	18	3.6	155	1.56	0.61	98.2	1.6	high		6.9		28	11.1
PS	77	M	2	1	24.2	3.4	144	1.17	0.85	117.7	1.8	high	9.5	6.6	0.03	27	12
PS	64	M	31	1	16.7	2.3	204	1.26	0.89	95.59		high	9.8	6.3	0.06	33	9.9
PS	54	F	31	1	22.8	3.6	115	1.35	1.2	97.56	1.3	high	8.9	4.8	0	25	8.9
PS	56	F	20	1	17.8	4.2	105	1.58	1.1	100.4	1.6	high	10.8	7.1	0.08	26	12.1
PS	25	M	4	1	14.9	4.2	161	1.65	1.09	90.31	2.5	high	9.1	5.8	0.03	25	10.5
PS	61	F	16	1	21.6	3.2	120	1.13	1.01	103.5	1.5	high	9.9	6.1	2.25	28	12.5

PS	59	F	14	1	16.2	3.8	128	1.15	1.32	99.33	2.1	high	11.6	8.6	0.1	24	13.8
PS	66	M	7	1	23.9	4.1	197	1.61	0.88	85.06	1.8	high		4.9	0.05		
PS	58	M	6	1	21.4	3.7	197	1.64	0.75	97.23	1.8	high		6.1	0.05		
PS	75	M	5	1	21.6	3.2	113	1.46	0.77	86.52	1.8	high	8.7	4.6	0.03	26	10.3
PS	69	M	4	1	27.6	3.5	195	1.49	0.94	87.32	2.1	high		5	0.05		
PS	68	F	4	1	24	3.5	140	1.36	0.77	107.3	1.9	high		4.7		26	9.9
PS	58	F	4	1	20.6	3.7	128	1.21	1.16	116	1.5	high	9.9	8.1	0.1	31	11
PS	36	M	3	1	33.8	3.8	128	1.34	0.71	91.22	1.8	high		6.3	0.08		
PS	57	M	2	1	27.2	3.3	107	1.1	0.93	41.9	1.8	high	8.5	4.5	0.05	32	9.6
PS	55	M	20	1	17.1	4.1	206	1.33	0.86	82.78	1.5	high	8.7	4.8	0.01	20	10.3
PS	56	M	13	1	24.1	4.3	196	1.8	1.18	139.5	1.5	high	10.2	4	0.04	33	10.5
PS	76	F	17	1	18.6	3.9	104	1.31	0.67	95.75	1.5	high	10	4	0.04	26	12
PS	52	M	6	1	25.4	4.5	138	1.67	1.16	80.85	2.1	high	9.6	5.8	0.04		10.4
PS	76	F	5	1	22.4	3.9	126	1.93	0.82	107.2	2.1	high	10.1	5.8	0.05	27	9.4
PS	52	M	2	1	23.4	4.5	120	1.68	0.79	60.12	2.1	high	8.6	4.7	0.05	26	10.6
PS	62	F	12	1	20.3	4.3	166	1.27	0.72	140.5	1.8	high	8.9	5.2	0.65	32	12.4
PS	33	M	20	1	20.7	4.6	180	1.71	0.85	88.41	2.5	high	10.2	6.5		25	12
PS	53	M	5	1	22.7	4.4	169	1.17	0.84	86.99	2.1	high	9.9	5.7	0.18		10.7
PS	47	M	7	1	22	4.2	178	1.38	0.99	93.43	2.2	high	8.9	3.9	0.06	23	12.8
PS	61	M	4	1	22.2	4.3	161	1.51	0.74	151.3	1.1	high		5.2	0.05	28	11.6
PS	65	M	6	1	22.6	4.5	189	1.55	0.68	140.3	1.8	high	9	5.6	0.38		10.3
PS	60	F	9	1	20.6	4.6	129	1.23	0.78	148.8	1.8	high		3.4			10.2
PS	52	F	20	1	16.9	4.1	168	1.55	1.13	115.5	1.3	high	8.4	4.3	2.39	27	9.7
PS	52	F	14	1	17.6	4	137	1.28	0.9	116.7	1.5	high	10	4.4	0.06	28	10.3
PS	62	M	20	1	18.8	4.1	159	2.14	0.76	81.97	1.8	high	8.6	3.6	0.09	34	10
PS	43	M	12	1	20.5	4	167	1	0.9	99.66	2.5	high	10.9	4.8		29	10.2
PS	51	F	3	1	27	3.4	134	1.74	0.84	73.85	1.3	high	9.2	4.4	0.02		9.9
PS	75	F	2	1	18	4.4	125	1.24	0.97	95.07	2.5	high	10.5	7.8		28	10.1
PS	70	M	18	1	19	4.4	174	1.72	0.89	130.1	1.1	high		6.8	0	26	10.7
PS	60	M	3	1	26.5	4.2	93	1.31	0.93	71.43	1.5	high	7.9	2.2	2.46	23	8.6
PS	47	M	19	1	20.4	4	116	1.44	0.69	112.9	1.5	high	8.1	2.5	0.32	33	12.2
PS	69	F	9	1	16.4	4.3	121	1.51	0.87	71.82	2.2	high	9.3	4.4	0.1	35	9.4
PS	72	M	10	1	21.3	4	110	0.91	0.96	137.3	2.5	high	11.1	5.5	0.05		10.2
PS	61	M	7	1	28.4	4.3	158	1.8	1.01	100.6	1.5	high	10.9	4.6	0.04	35	9.9
PS	41	M	15	1	22.6	4.3	102	1.23	0.97	118.1	2.5	high	9.7	5.6	0.14	24	11.9
PS	65	M	4	1	20.5	4.4	155	0.87	0.86	98.7	1.3	high	9.6	3.2			9.2
PS	48	M	8	1	20.5	4.6	177	1.33	1.27	111.1	2.1	high	11.3	6.6	0.07	18	10.1
PS	73	F	9	1	18	4.3	167	1.45	0.79	127.5	1.4	high	8.9	5.5	0.03	21	8.7
PS	65	M	8	1	24.1	4.1		1.83	0.71	104.1	2.1	high	10.2	6.3		27	10.9
PS	63	M	21	1	23.9	4.1	166	1.26	1.16	109.6	1.8	high	10.1	7	0.18		11.7
PS	50	F	10	1	21	4.3		1.41	0.87	113.7	1.8	high	9.4	6.3		29	8.8
PS	53	M	13	1	19.9	4.1		1.92	0.82	99.6	1.8	high	9	4.7		27	10
PS	73	M	2	1	22.7	4.5	139	1.49	0.88	108.5	2.1	high	8	2.3	0.07		11.2
PS	75	F	11	1	17.2	3.8	104	1.57	0.83	91.47	1.5	high	8.2	7.1	0.27	29	10.1
PS	59	M	18	1	17.8	4.6	158	0.75	1.07	86.62	2.1	high	8.9	5.1		31	10.3
PS	58	M	4	1	19	4.5		1.58	0.94	100.6	1.5	high	8.4	6.1		39	10.4
PS	59	M	20	1	21.2	4.1	109	1.14	0.97	126.4	2.1	high	8.6	5.1	0.16	34	10.6
PS	56	M	3	1	22	4.4	196	1.75	0.55	39.77	1.8	high	9.4	4.6	0.02		11.9
PS	74	M	3	1	21.8	4	159	1.2	0.75	111.8	1.6	high	8.4	5.4	0.19		8.7
PS	50	M	14	1	19.8	4		1.46	0.96	96.89	1.5	high	9	4.5		26	11.4
PS	68	F	9	1	16.2	4.5		2.19	0.92	131.8	1.5	high	9.4	5.4		25	10.6
PS	57	M	8	1	21.3	4.4	184	1.53	0.92	94.46	1.8	high	8.7	6.4	0.05		
PS	54	M	8	1	21.6	4.1	100	1.39	0.7	128.1	1.8	high	7.7	6.3	0.05	27	11.4
PS	53	M	16	1	18.7	4.5		2.22	1.02	113.5	2.1	high	10.8	4.8		28	9.1
PS	64	M	15	1	18.7	4.1		1.21	1.02	128.1	2.5	high	10	5.5		32	11.4
PS	49	F	4	1	21.4	4.4	115	1.58	0.75	97.59	1.5	high	9.4	4.8	0.38	36	11.6
PS	61	M	15	1	18.1	4.2		1.37	0.97	121.5	2.5	high	9	6.3		34	11.7
PS	54	M	10	1	23.5	4		2.41	0.77	118.1	1.5	high	9.5	4.3		28	9.7
PS	60	M	7	1	17	4.5	121	1.35	1.44	95.08	2.1	high	8.3	4.6	0.05	24	10.2

PS	48	F	15	1	20	4.2	171	1.48	0.89	118.4	2.1	high	9.2	4.2	0.05	28	11.9
PS	60	M	17	1	20.3	3.5	167	1.21	0.77	88.27	2.1	high	9.1	5	0.02	26	11.7
PS	69	M	8	1	20.6	4.3	242	1.79	0.92	97.72	1.5	high	9.8	5.5		29	10.9
PS	59	M	15	1	17.8	4.4	141	1.62	0.95	120.6	2.1	high	9.5	4.7	0.37		8.8
PS	60	M	6	1	25.3	4.2	166	1.49	0.72	98.23	2.1	high	9.2	6	0.37	27	11.1
PS	79	M	4	1	20.1	4.1	242	1.57	1.02	145.9	1.8	high	9.7	4.6	0.16		10.6
PS	75	F	9	1	15.9	3.6	120	1.4	0.65	114.6	1.5	high	8.7	5	0.24	34	10.2
PS	66	F	4	1	18	3.7	128	1.12	0.92	96.53	1.9	high		4.8		29	9.9
PS	81	F	8	1	24.2	3.4	124	0.9	1.13	117	1.3	high	8.1	3.9	0.6	13	10.7
PS	65	M	8	1	18.9	4.2	146	1.53	0.82	84.9	2.1	high	9.2	7	0.1		10.2
PS	72	F	2	1	20.9	3.7	149	1.34	0.77	129.7	1.9	high		8.8		28	10.9
PS	67	F	7	1	16.5	3.5	104		0.79	50.59	1.5	high	9.5	5	1.4	39	8.4
PS	48	M	4	1	24.6	3.9	115	1.54	1.14	121.9	1.5	high	9.2	6.9	0.37	27	9.2
PS	69	M	13	1	22.1	3.8	138	1.07	0.95	134.1	2.1	high	8.3	7.7	0.2	24	10.9
PS	52	M	4	1	23.3	3.7	216	1.34	0.96	124.3	1.7	high		4.4	0.05	23	12.2
PS	61	M	2	1	22.2	3.9		1.21	0.83	98.55	1.5	high	9.2	5.9			9.8
PS	63	M	9	1	20.1	3.5	140	1.21	0.75	114	1.5	high		5.2	0.1	1	8.7
PS	69	F	3	1	22.5	3.7	117	1.26	0.7	150.8	1.9	high		6		34	10.5
PS	65	F	7	1	19.9	3.8	184	1.58	0.8	120.7	1.5	high		4.5	0.18	22	10.6
PS	77	M	5	1	20.5	3.7	96	1.69	0.99	119.2	1.1	high	9.4	5.2		18	12.5
PS	67	M	8	1	25.1	3.9	116	1.32	0.96	124.6	1.9	high		5.4		39	8.7
PS	58	M	13	1	25.7	3	144	1.45	0.62	100.3	1.5	high	8.5	5	0.07	27	11.3
PS	44	F	6	1	23	3.8	218	1.55	0.66	104.9	1.1	high	9.9	5.4	0.3	31	12.4
PS	48	M	5	1	28.5	3.9	186	1.9	0.75	121.2	1.1	high	9.9	6.4	0	29	10.1
PS	54	M	8	1	30.5	4.1	164	1.36	0.79	105.6	1.8	high	9.8	5.6	0	21	10.1
PS	60	F	11	1	18.1	3.3	127	1.46	0.49	65.36	1.5	high	9	4.2	0.47	23	11.6
PS	62	M	21	1	18.7	4.2	196	1.47	0.7	69.39	1.5	high	8.2	5.8	0.9	29	12.6
PS	65	F	12	1	21.4	4.2	100	1.18	0.95	72.53	2.1	high	8.3	5.5	0.1	21	12.3
PS	71	F	8	1	16.5	3.5	133	1.38	0.52	47.35	1.5	high	8.6	6.5	0.16	25	12.3
PS	68	F	7	1	18.9	3.6	154	1.05	0.74	100.7	1.9	high		5		19	10.3
PS	61	M	9	1	20.9	3.8	125	1.35	0.78	97.75	1.8	high	8.8	5.1	0.03	21	10.9
PS	62	M	3	1	26.3	3.9	111	1.72	0.59	82.32	1.3	high	9.8	5.2	0.26	21	8.7
PS	75	F	4	1	20.2	3.8	218	1.13	0.97	82.03	2.1	high	9.2	7.7	0.2	30	16.4
PS	43	M	5	1	22.7	4.2	227	1.65	0.61	75.48	1.5	high		3.3	0.2	12	10.3
PS	46	M	4	1	35.1	4.1	146	1.75	1.08	97.41	1.3	high	9.4	5.4	0.19	26	10.9
PS	79	M	7	1	21.8	3.9	172	1.12	0.64	130.3	1.5	high		3.8	0.5	5	11.7
PS	69	F	9	1	26.2	3.6	106	1.1	0.82	83.32	1.1	high		6.5	5.1	23	10.7
PS	68	M	6	1	24.7	4	135	1.23	0.95	109	1.5	high		4.8	0.9	2	10.6
PS	73	F	5	1	21.2	3.9	133	1.56	0.82	70.85	1.5	high		4.4	4.96		9.2
PS	65	M	4	1	19	3.7	149	1.18	0.75	121.6	1.3	high	9.5	6.7	0.5	29	15.3
PS	73	M	3	1	26.1	3.6	170	1.35	0.6	53.85	1.8	high		3.5	0.17		
PS	70	M	2	1	22.8	3.8	156	1.36	0.76	60.64	1.8	high	8.3	3.7	0.22	24	11
PS	66	M	2	1	25.5	3.9	143	1.3	0.9	95.65	2.1	high		4	0.05		
PS	62	M	6	1	18.1	3.7		1.5	0.88	99.87	2.1	high	9.5	9.5	1.78		14
PS	58	M	4	1	22.6	4.1	182	1.28	0.83	112.8	2.1	high		6.6	0.12		
PS	62	F	8	1	22.3	3.7		1.29	0.67	77.64	1.6	high	8.2	6.2		24	10.3
PS	59	M	13	1	20	4.1	141	1.58	0.76	101.4	2.1	high	10.1	6	0.05	25	8.2
PS	59	M	4	1	26.7	3.7	159	1.55	0.7	75.94	2.1	high		5	0.05		
PS	82	M	3	1	21.7	4		1.83	0.85	103.2	1.3	high	9.7	7.8	0.1	30	8.1
PS	64	F	3	1	26.8	3.4	98	1.41	0.63	75.96	1.6	high	10.2	5.2	0.6	36	
PS	80	F	2	1	21.1	3.7	165	1.45	0.79	77.46	1.5	high		3.7	0.1	2	11.8
PS	60	M	3	1	18.7	3.7	152	1.31	0.63	25.25	1.6	high	9.1	3.8		22	9.4
PS	59	M	2	1	19.8	4.1	84	1.31	0.75	53.8	1.6	high		5.3	0.77		
PS	68	M	6	1	25.9	4	181	0.99	0.78	101.9	1.8	high	7.4	3.2	0.3	14	12.2
PS	80	M	6	1	19.6	3.5	139	1.21	0.8	130.1	2.1	high	10.4	5.5	0.02	28	9.4
PS	84	M	5	1	19.3	3.8	213	1.86	0.75	122	1.8	high		7.8	0.01		12.6
PS	48	F	10	1	21.6	3.7	130	1.35	1.02	118.4	1.3	high	9.3	4.5		26	11.9
PS	65	M	3	1	22.5	3.6	175	1.42	0.73	57.12	1.8	high	9.8	4		20	10.6
PS	78	F	2	1	16.6	3.9	117	1.04	1.03	66.68	1.8	high		5	0.02	49	11.6

PS	39	F	25	1	19.3	2.9	142	1.1	0.86	91.93	1	high	9.1	5.2	0		11.5
PS	83	F	3	1	18	3.6	144	1.25	1.01	81.8	1.5	high	9.3	5.3	0		10.9
PS	67	M	2	1	23	3.9	115	1.26	0.86	98.72	1.8	high		7.4	0.07	30	8.7
PS	52	F	9	1	18	4.1	193	1.37	0.88	145.1	1.8	high	9.6	5.9	2.1	26	8.9
PS	69	M	7	1	21.4	3.6	125	1.53	0.95	130.5	1.3	high	8.4	5.9	5.2	30	10.8
PS	77	M	2	1	22	3.2	152	1.73	0.69	111.3	1.1	high	9.4	4.1	0.1	23	7.8
PS	75	F	14	1	24.9	3.2	120	1	0.96	109.1	1.8	high	10.5	5.8	0.2	27	12.4
PS	58	F	3	1	22.1	4.1		1.23	0.93	106.8	2.1	high	7.8	10.9	22.01		8.2
PS	55	F	2	1	18.6	3.8	116	1.4	0.95	99.3	1.5	high	11.5	4.2	0.17	23	11.2
PS	57	F	2	1	26.6	3.5	93	1.12	0.98	116	1.5	high	9.6	2.4	0	23	6.1
PS	31	F	8	1	16.8	4	130	1.65	1.23	123.2	1.3	high	9	5.9	2.1	28	12.7
PS	75	M	5	1	20.6	3.7	190	1.67	0.65	67.31	1.6	high	9.3	6		34	10
PS	51	F	2	1	18.8	3.7	168	1.5	0.67	65.8	2.1	high		4	0.16	24	11.4
PS	76	M	7	1	22.5	3.6	223	1.53	1.14	110.9	1.5	high		4.4	0.1	1	10.7
PS	77	M	3	1	25.3	3.6	125	1.43	0.95	65.9	1.8	high	8.7	6	0	20	9.9
PS	59	F	6	1	23.9	3.8	140	1.44	1.18	118.6	1.8	high		5	0.05	23	9.6
PS	64	F	24	1	21.3	3.4	147	0.96	0.75	74.35	1.8	high	9.4	5.8	0.86	19	10.4
PS	41	M	14	1	20.8	3.7		1.77	0.98	135.7	1.8	high	9.2	8.6		29	10.3
PS	58	F	11	1	17	3.7	131	1.38	1.07	71.08	1.3	high		6		23	10.5
PS	47	M	6	1	22.3	4.1	134	1.16	0.8	128.7	1.5	high	8.5	4.9	0.1	27	10.9
PS	54	M	15	1	19.8	3.1	160	1.81	0.74	125.9	1.6	high	9.7	4.8	0.39	21	11.3
PS	55	M	7	1	24.4	3.6	142	1.67	0.96	132.3	1.8	high	8.7	3.8	0.21	27	12.3
PS	57	F	3	1	20.5	3.9	122	1.9	0.8	91.71	1.3	high	9.4	2.5			7.7
PS	56	F	21	1	15.5	3.9	147	1.23	1.16	123.8	2.2	high		4.5	0.1	37	9.4
PS	41	M	15	1	22.8	4.2	124	1.25	1.03	131.2	1.3	high	10.2	8.3	0.1	26	11.9
PS	71	F	4	1	24	3.9	137	1.58	0.81	77.84	1.8	high		4.2	0.28	37	11.6
PS	54	M	20	1	22.5	4.2	117	1.23	0.95	111.3	2.2	high		5.8	0.1	28	9.5
PS	59	F	20	1	15.6	4.1	168	1.63	1.16	118.9	1.8	high	9.2	5.8	0.09	21	11.6
PS	67	M	30	1	19.1	3.8	167	1.52	1.02	70.53	1.8	high	8.9	4.4	0.01	22	11.9
PS	77	F	27	1	16.8	3.8	128	1.4	0.87	100.8	1.8	high	9.1	4	0.08	25	10
PS	67	M	26	1	18.6	4	168	1.36	0.83	109.6	1.6	high	8.8	5.9	0.11	31	11.5
PS	60	M	26	1	19.2	4.1	122	1.69	0.99	99.81	1.8	high	9.6	6.3	0.02	33	11.9
PS	62	F	25	1	15.8	3.8	91	1.31	0.96	121.3	2.2	high	9	5.1		26	11.2
PS	54	M	25	1	24.3	3.9	196	1.27	0.78	66.14	1.8	high	10.2	5.8	0.09	28	10.2
PS	63	M	24	1	22.8	3.9	112	1.19	1.1	119.9	1.8	high	7.8	5	0.1	23	12.6
PS	53	F	23	1	17	4	169	1.49	1.2	129.3	1.8	high	9.5	4.4	0.1	32	10.2
PS	39	F	23	1	16.6	4.4	138	1.58	1.19	139	1.6	high	8.5	3.8	0	23	12.2
PS	64	F	22	1	17.8	3.9	120	1.59	1.02	148.9	1.6	high	10.3	6.2	0.12	22	11
PS	55	F	18	1	17.3	4.3	165	1.52	1.21	109.3	1.9	high	8.6	6			10.8
PS	78	F	18	1	26	3.7	147	1.02	0.99	122.1	1.6	high	9	4.7			11.6
PS	61	F	18	1	18	3.5	137	1.2	1.07	118.2	1.5	high	9.6	4.3	0.3	16	10.4
PS	64	F	18	1	22.8	3.6	208	1.93	0.91	134	1.4	high	8.7	5.8	0.03	26	11.7
PS	52	F	17	1	23.6	4.5	196	1.44	0.92	110.8	0.9	high	9.5	3.6		20	11.8
PS	50	F	17	1	19.3	3.8	174	1.37	0.94	116.4	0.6	high	8.1	5.9		19	10.4
PS	63	M	17	1	19.1	4.1	217	1.26	1.22	124.4	1.8	high	10	8.3	0.94	33	13.7
PS	34	F	17	1	18.4	4.3	187	1.48	1.39	119.9	2.1	high	10.1	5.7	0.1	23	10.6
PS	48	M	17	1	22.7	3.9	159	1.82	0.77	118.6	1.6	high		4.2	0.05	25	9.9
PS	59	M	17	1	18.2	4.3	170	1.19	0.95	122.7	1.5	high	10.7	6.2	0.3	28	13.3
PS	71	F	16	1	21.1	4	137	1.35	1.16	109.3	1.3	high		3.5	0.33	24	8.8
PS	77	M	16	1	18.9	3.6	94	1.48	0.75	101	1.3	high	9.2	4.3	0.31	30	12.2
PS	46	M	16	1	23.5	4.1	136	1.7	0.93	128.6	1.6	high		3.7	0.07	28	10.6
PS	56	M	15	1	21.9	4.3	191	1.56	0.8	94.64	1.4	high	8.3	6.2	0.11	25	12.5
PS	79	F	15	1	21.4	3.5	81	1.24	0.57	71.02	1.5	high		7.4	0.2	40	10.5
PS	53	F	15	1	23	4.4	99	1.48	0.76	135.6	1.6	high		7.4	0.14	19	12.2
PS	60	M	15	1	22	4.1	112	1.37	1.12	134	2.1	high		4.3		22	11.4
PS	38	M	14	1	18.7	4.2	182	1.41	1.15	122	1.6	high	10	6.6	0.21	15	10
PS	64	M	14	1	20.5	3.7	184	1.51	0.83	119.5	1.9	high		4.7	0.1	24	10.4
PS	49	M	13	1	18.6	4.1	138	1.79	0.89	117.7	1.3	high		4.3	0.05		9.5
PS	58	M	13	1	23.2	4	199	2.2	1	139.8	1.3	high	9.2	5.8	0.01	27	9.3

PS	74	M	13	1	19.2	3.5	118	1.02	0.86	108.1	1.5	high	8.7	3.3	1.3	10	12.7
PS	60	M	13	1	20.9	3.8	162	1.22	0.91	136.4	1.8	high	9.3	3.1	0.02	24	11.7
PS	71	M	13	1	19.8	4	121	1.24	0.74	151.5	1.5	high	8.1	7.4	0.1	24	12.7
PS	66	M	13	1	19.2	4.3	121	1.23	1.11	134.2	1.1	high	8.1	4	0.1	24	10
PS	53	M	12	1	19.2	4.6	117	1.65	0.95	99.99	1.3	high		6.3	0.25		9.9
PS	66	M	12	1	22.6	4.2	164	1.11	1.02	102.7	1.8	high	8	4.7	5.15		9.3
PS	67	F	12	1	17.8	3.8	168	1.33	1.14	126.5	1.6	high		5.1	0.07		9.6
PS	70	F	12	1	20.8	4.1	247	1.53	0.99	129.5	1.6	high		6.6	0.05		9.5
PS	66	F	12	1	19.1	3.9	137	1.36	0.92	114.6	1.6	high		4.8	0.05		10.7
PS	49	F	11	1	16.6	4	152	1.2	1.23	127.9	1.8	high	9.4	5.3	0.25	20	11.8
PS	72	M	11	1	17.8	3.9	155	1.13	0.91	111.5	1.5	high		3.3	0.1	4	10
PS	65	M	10	1	19.6	3.7		1.64	0.74	114.9	1.3	high	9.2	7.1	0.1	26	12.1
PS	66	M	10	1	17.2	4	183	1.51	0.92	92.08	1.5	high		5.6	0.1	2	10.8
PS	67	M	10	1	20	3.7	164	1.46	0.85	115.2	2.1	high	8.9	4.2	0.3	26	9.9
PS	58	F	10	1	17.1	4	177	1.37	1.15	115.9	2.1	high	8.7	5	0.1	28	11.9
PS	59	M	10	1	16.6	4.4	112	1.36	1.24	85.63	1.6	high	9.2	4	0.18	27	10.9
PS	83	F	10	1	20.7	4.1	189	1.71	1.18	119.6	1.8	high	9.5	6.6	0.05	27	11.7
PS	54	M	10	1	18	4.1	221	1.62	0.8	123.1	2.1	high	8.9	5.7	0.3	21	9.8
PS	64	F	9	1	21.3	3.7	98	1.33	0.59	109.8	1.3	high		4.2	0.05		9.7
PS	65	M	9	1	23.4	3.9	146	1.81	1.01	139.4	1.4	high			0.18		
PS	65	F	9	1	22.3	4.2	184	1.65	1.02	105.2	1.8	high	8.9	4.5	0.2	30	11.6
PS	68	M	9	1	23.5	3.9	179	2.18	0.86	162.7	1.4	high	9.6	6.1	0.1	34	10.1
PS	80	F	8	1	27.4	3.5	215	1.41	0.9	132.9	1.6	high		4.9	0.08		10.1
PS	84	F	8	1	16.9	3.8	174	1.65	1.13	112.6	1.8	high	9.5	6.1	0.03	24	9.6
PS	78	F	8	1	22.1	3.9	172	1.41	0.84	118	1.5	high		5.3	0.1	24	11.5
PS	66	F	8	1	17.7	4	127	1.31	1.11	106.8	2.1	high	9.3	5.1	0.77	20	9.5
PS	58	F	8	1	21.9	3.7	162	1.26	0.76	127.5	2.1	high	8.8	6.7	0.5	32	11.4
PS	75	M	7	1	20.2	4.2	173	1.05	0.62	100.1	1.5	high		4.9	0.2	2	9.8
PS	40	F	7	1	21.5	4	109	1.12	1.11	101.9	1.9	high		4.8	0.59		10.3
PS	55	M	7	1	23.4	4.2	164	1.19	0.96	126.3	1.8	high	9.4	5	0.2		11.2
PS	50	M	7	1	20.8	4.4	157	1.07	1.14	79.89	2.1	high	11.1	6.1	0.01	24	9.3
PS	54	F	7	1	23.1	4.3	164	1.37	1.08	93.68	1.5	high		3.7	0.04	40	9.8
PS	67	F	7	1	18.8	4	124	1.29	1.07	106.6	1.6	high	9.7	4	0.07	30	10.5
PS	27	F	7	1	23.1	4.1	144	1.68	0.81	134.8	2.1	high		4.5	0.18	31	10.5
PS	63	F	6	1	23.1	4.1	139	0.97	0.88	94.03	1.3	high		4.9	0.05		10.4
PS	56	M	6	1	25.5	4.3	152	1.78	1.1	114	1.6	high	9.2	5.4	0.72		9.8
PS	70	F	6	1	23.6	3.4	123	1.9	0.81	115.8	1.6	high		5	0.1	31	8.8
PS	82	F	6	1	20.7	3.7	193	1.73	0.85	120.4	1.8	high		3.1	0	24	9.8
PS	40	M	6	1	23.2	4.4	143	1.32	0.76	130.7	1.8	high	9.5	5.9	0.08	25	10.4
PS	64	M	6	1	25	4.4	113	1.22	1	116.4	2.1	high	10.3	4.7	0.06	24	11.6
PS	82	F	6	1	19.9	4.2	166	1.24	1.2	105.5	1.5	high		5.8	0.1	2	10.4
PS	74	M	6	1	21.6	3.7	144	0.92	0.61	90.36	1.5	high		5.5	0.1	2	10.5
PS	64	M	6	1	20.3	4.2	140	1.17	0.64	107.3	1.5	high		5.5	0.8	2	7.4
PS	57	M	6	1	27.8	4.3		1.53	0.97	124.3	1.5	high	9.4	6.3	0.16	26	10.4
PS	62	M	5	1	19	3.7	188	1.21	0.95	122.8	1.6	high	9.4	5	0.02	23	9.6
PS	37	M	5	1	23.7	4.1	133	1.53	0.89	100.7	2.1	high	9.6	4.1	1.32	34	9.7
PS	60	M	5	1	19.8	4	160	1.33	0.75	111.6	1.9	high		7	0.1	26	13.9
PS	63	F	5	1	25.6	3.6	127	2.42	0.79	90.58	0.8	high		2.3	0.15	27	7.2
PS	81	M	5	1	16.3	3.5	147	1.25	0.8	94.1	1.3	high	8.9	4.6	0.29	28	10.9
PS	64	M	5	1	21.3	3.8	156	1.3	0.66	76.05	1.5	high		6.2	0.1	2	9.6
PS	81	M	5	1	25.6	3.8	114	1.09	0.67	76.36	1.5	high		4.9	0.1	2	8.3
PS	76	M	5	1	21.7	4	131	1.12	0.98	129.7	1.5	high		6.5	0.3	2	10.3
PS	77	F	4	1	25.2	3.4	218	1.31	0.8	107.6	1.5	high		6.6	0.1	3	11.2
PS	74	F	4	1	22.4	4.4	161	1.16	0.69	127.5	1.9	high		5.4	0.3	28	11.6
PS	51	F	4	1	19.5	4.3	164	1.21	1.18	143	2.1	high	10.2	4.9	0.7		11.3
PS	73	M	4	1	23.3	4.1	128	1.58	0.99	117.7	1.6	high		5.7	0.1	22	9.6
PS	71	M	4	1	29	3.8	150	1.06	0.7	146.6	2.1	high	9.6	3.8	0.01	25	10.6
PS	58	M	4	1	24.7	4.4	138	1.22	1.13	94.71	1.5	high		5.5		2	9.9
PS	49	M	4	1	25	4.4	134	1.41	0.85	70.69	1.5	high	10	4.8		8	10.3

PS	67	F	4	1	21	4.1	148	1.81	0.98	117.5	1.6	high		4.2	0.1	29	12.3
PS	57	M	4	1	24.2	3.8	202	1.5	0.55	88.52	1.5	high	9.9	7.8	0.18	29	9.7
PS	74	M	3	1	24.2	3.6	122	1.25	0.71	116.8	2.1	high	7.7	6.4	0.1		9.4
PS	58	M	3	1	17.8	3	141	1.22	0.76	42.6	1.8	high	8.8	5.4	0.18	33	11.9
PS	60	F	3	1	15.5	4	226	1.89	1.32	100.3	1.8	high	8.6	5	0.12	12	10.3
PS	75	M	3	1	19.6	4	155	1.59	0.92	108.2	1.8	high	8.4	6.2	0.96	31	11
PS	66	M	3	1	16.9	3.4	149	1.52	0.84	82.06	1.5	high	10.3	8.5		28	11
PS	37	M	3	1	17.3	4.3	186	1.53	0.88	106.3	1.5	high	8.6	4	1.5	29	10
PS	68	M	2	1	28.9	3.6	205	2.26	0.62	83.18	1.6	high	8.7	4.7	0.3	30	11
PS	62	F	2	1	20.3	4.4	129	1.34	1.03	131.4	1.1	high	9.8	5	0.1	24	11
PS	82	F	2	1	21.4	3.3	146	1.14	0.57	135.1	1.5	high	8.8	6.2	0.95		12
PS	57	F	2	1	18	4.4	83	1.36	0.95	113.6	1.5	high	9	5.2	0.3	32	9.8
PS	79	M	2	1	18.1	4	105	1.26	0.84	137.1	1.3	high		4.3	0.1	20	9.9
PS	65	M	2	1	22.5	3.8	114	1.3	0.88	80.43	1.5	high	10.7	3.3		28	9.2
PS	76	F	2	1	35	3.8	105	1.64	1.07	93.18	2.1	high	9	5.9	0.63	19	11
PS	56	M	4	1	21.2	4.6	163	1.27	0.73	93.8	2.1	high	8.5	4.7	0.28	30	9.2
PS	62	F	32	1	19.3	4	119	1.44	0.82	110.6	2.1	high	8.5	5.2	0.4		11
PS	76	M	11	1	18.9	4.1	151	1.21	0.86	126.7	2.1	high	7.8	6.3	0.1		11.2
PS	55	M	9	1	23.3	3.9	128	1.46	1.02	110.9	1.8	high	8.1	6.5	0.7		11.4
PS	75	M	8	1	19.3	4.2	163	1.62	1.02	94.26	1.8	high	8.9	5.1			13.7
PS	60	F	6	1	19	4.6	107	1.51	0.83	122.5	1.8	high	9	5.7	0.1		10.9
PS	59	M	4	1	21.2	4.4	158	1.52	1.05	154.2	1.5	high	8.7	5.9	0.18	30	11.5
PS	71	M	6	1	21.4	4	126	1.19	0.87	117.3	1.8	high	9	4.1	4.48	26	10.1
PS	52	F	30	1	16.7	4	223	1.77	1.14	101.8	1.5	high		5	0.02		10.2
PS	58	M	4	1	28.6	3.8		1.53	0.96	99.26	2.1	high	10.2	5.1		30	10
PS	39	M	3	1	26	3.9	197	1.19	0.83	88.49	1.8	high		7.3			10.5
PS	47	F	13	1	21.4	3.4	154	1.18	0.86	119.4	1.5	high	9.2	5.2	0.04	22	9.5
PS	60	F	16	1	17	3.9	202	1.69	1.08	127.6	0.8	high	8.6	4.2	0.3	28	10.3
PS	81	F	2	1	22.3	3.7	142	1.13	0.91	76.78	2.1	high	8.8	7.1		15	12.6
PS	59	F	2	1	23	3.5		2.19	1.16	112.8	1	high	8.2	5.4	0.07	27	10.5
PS	74	M	7	1	18	3.4	53	1.69	0.79	126.9	1.6	high	8.8	5.6	5	22	11.4
PS	73	M	5	1	26.1	3.9	130	1.87	0.91	124.9	1.8	high	9.6	4.4	0.4	31	10.4
PS	49	M	16	1	20	4.3	161	1.61	0.92	91.83	1.5	high		3.6	0.09	25	10.5
PS	66	M	5	1	23.6	3.9	134	1.16	1.04	106	1.6	high		6	0.81	24	11
PS	77	M	7	1	26.5	3.2		1.32	0.77	114.4	1	high	8.4	4.3	0.11		10.5
PS	67	F	19	1	21.6	3.5	185	1.44	0.95		1.6	high		4.6	0.43	22	11.9
PS	58	M	17	1	19	3.5	168	2.1	0.94		1.3	high	8.3	4.5	0.09	23	9.8
PS	38	F	17	1	20.3	4		1.25	1.1		1.5	high	9.3	2.7	0.37	22	10.7
PS	42	F	13	1	18.7	4		1.55	1.06		1.5	high	9	5.2	0.16	29	12.5
PS	72	F	13	1	30.5	3.4	91	1.75	0.77		1.5	high	8.8	5.7	0.03	22	9.9
PS	59	M	11	1	17.8	4.1	248	1.5	1.13		1.6	high		8.1	0.09	22	10.9
PS	68	F	11	1	18.6	4.2	154	1.51	0.84		1.6	high		5.3	0.1	23	10.4
PS	57	M	11	1	21.4	4.5	156	1.07	1.08	128	1.6	high		4.5	0.1	25	11.7
PS	57	M	8	1	18.1	3.8	115	1.64	0.96	92.22	1.5	high	9.7	4.1	0.46	25	11.3
PS	70	M	8	1	21.2	3.7	155	1.4	0.87	124.7	1.6	high	8.7	8.1	0.05	23	8.2
PS	72	F	7	1	21.8	3.6	94	1.12	0.98		2.1	high	8.6	6.9	0.02	20	11.3
PS	27	M	7	1	17.9	4.3		1.86	1.21		2.1	high	8.8	6.6	0.54		12.3
PS	57	M	4	1	26.9	4.1	125	1.45	0.82		2.1	high		5.4	0.17	21	11.2
PS	74	M	3	1	22.8	4	157	1.33	0.94		1.3	high	8.1	3.7	6.34	52	8.4
PS	67	M	19	1	22.7	3.7	121	1.81	1.07	120.1	1.6	high	9.2	5.3	0.04	25	10.9
PS	54	F	18	1	22.2	3.8	124	1.89	1.06	112.3	1.5	high		3.9		30	10.1
PS	58	M	14	1	25.6	3.7	136	1.41	0.99	123.1	1.6	high		5.2	0.1	29	10.8
PS	68	M	9	1	23.9	3.6	158	1.07	0.74	104.4	1.5	high	9.1	4.6	0.08	29	10
PS	53	M	9	1	21.8	3.6	127	1.28	1.35	130.6	1.3	high		4.7	1.56	20	11
PS	81	M	7	1	18.5	3.1	167	1.11	0.92	60.52	2.1	high	8	6.5	0.3	27	10.9
PS	71	M	9	1	30.7	3.6	131	0.92	1.08	129.2	1.6	high	9.1	9.8	1.57	20	8.2
PS	60	M	6	1	21.7	4.6	125	1.33	0.9	132.5	1.9	high		5.1	0.1	33	13.4
PS	72	M	8	1	22	3.9		1.26	1.03	87.7	1.8	high	9.1	5.1	0.1	20	9.9
PS	65	F	8	1	20.9	4.3	211	1.28	0.81	85.93	1.3	high	8.9	4.7	0.55	24	11.9

PS	33	M	3	1	19.1	4.4	145	1.07	0.87	89.22	1.5	high	9.1	4.2	0.05	15	10.6
PS	67	F	9	1	23.2	3.8	135	1.58	0.66	101.1	1.3	high	8.3	5	2.27	29	9.5
PS	76	M	6	1	25.5	4.2	179	1.73	0.72	92.69	1.6	high		4.7	0.05	27	8.3
PS	46	F	5	1	25.5	3.6	175	1.54	1.16	105.2	1.1	high	9.5	5.3		31	10.9
PS	60	M	25	1	18.4	3.9		2.08	1.11	118.4	1.8	high	9.7	4.9		25	8.9
PS	72	F	18	1	20	3.8	127	1.31	0.72	121.9	2.1	high	9.6	5.6	0.14	32	13.7
PS	48	F	17	1	17.7	3.8		1.71	1.11	103.2	2.1	high	10	4.6		26	9.2
PS	61	F	17	1	26.1	3.9	146	1.37	1.02	144.4	1.8	high		8.6	0.21		10.7
PS	46	F	11	1	19.7	4	128	1.21	1.2	112.7	2.1	high	9.3	4.6	0.02	26	11.5
PS	42	M	10	1	20.7	3.9	134	1.59	1.02	103.6	1.5	high		5.2	0.03	36	10.5
PS	67	M	10	1	19.2	3.8	232	1.55	0.95	137.9	1.8	high	9.4	6		31	12.7
PS	71	F	9	1	18.3	4	169	1.79	0.77	109.3	2.1	high	8.9	3.7	0		11.7
PS	47	M	9	1	25	3.8	203	1.4	1.06	104.3	2.1	high	10.2	5.4	0.3		10.1
PS	68	F	8	1	21.6	3.9	110	1.72	1.18	129.6	2.1	high	9.6	4.5		29	9.5
PS	72	M	7	1	21.9	3.8	102	1.18	0.96	112.2	2.5	high	9.6	3	0.05	27	11
PS	69	M	7	1	22.1	3.8	103	1.58	1.05	141.4	1.8	high	9.3	2.3	0.04	20	9
PS	55	M	7	1	20.5	4	132	1.75	0.94	99.33	1.5	high	10.7	9.5	5.53	21	9.6
PS	59	F	7	1	22.9	3.9	228	1.29	1.09	119.4	1.8	high	8.9	4.6	2.02	28	9.9
PS	66	F	7	1	17.8	4	155	1.36	0.79	111.1	2.1	high	8.9	4.5	0.1		11.9
PS	64	M	6	1	19.7	3.8	111	1.09	0.98	121.6	2.5	high	9.7	6.7		28	11.1
PS	40	F	6	1	17.4	4.1	152	1.35	0.96	118.5	2.1	high	9	4.1	0.1	23	10.4
PS	60	M	5	1	19.7	4		2.3	1.12	116.5	1.8	high	9.3	6.4		25	10.5
PS	39	F	5	1	16.4	3.8	153	2.31	0.8	92.03	2.1	high	9.1	5.1	0.2	24	9.6
PS	64	M	5	1	20.8	3.7	135	1.33	1.05	85.01	1.8	high	8.7	7	0.1	30	10.2
PS	44	M	4	1	20.2	4.5	115	0.99	1.07	55.94	1.8	high	9.2	5	1.48	32	10.3
PS	77	F	4	1	21.6	3.9	161	1.75	0.79	129.4	1.6	high		5.3	0.1	26	11.2
PS	62	F	4	1	18.7	3.6	91	0.78	1.08	112.9	1.6	high		3.1	1.77		8.6
PS	44	F	4	1	36.4	4.2	147	1.29	0.71	114.7	1.8	high	8.7	2.6	0.06		8.9
PS	81	M	3	1	20.3	3.8	135	1.54	0.92	131.6	2.1	high	9.4	4.7	0.1	27	10.8
PS	71	M	3	1	20.1	3.9	105	1.41	0.89	125.4	1.6	high	9.4	7.3	0.07	27	10.6
PS	72	F	3	1	26.2	3.6	197	1.47	0.5	9.15	2.1	high	9.1	6.8	0.28	29	10.9
PS	76	M	3	1	21.5	3.9	145	1.7	0.81	113.2	1.5	high	9.2	5.3	0.1	29	10.1
PS	76	M	3	1	20.1	3.5	151	1.31	0.93	99.72	1.5	high	8.1	4.7	0.3	25	8.9
PS	70	F	3	1	23.9	4.2	130	1.32	0.85	76.38	2.1	high	9.7	5.5		25	10.1
PS	73	M	3	1	21.1	3.8	109	1.29	0.79	77.7	2.1	high	9	4.4	0.05	23	8
PS	60	M	3	1	26.5	3.7	95	1.3	1.15	127.2	1.8	high	9.4	4.8	0.07	33	9.1
PS	47	M	2	1	22.2	4.2		1.93	0.75	94.36	1.6	high	8.9	4		31	9.5
PS	58	M	2	1	21.4	4.1	148	1.5	0.67	80.76	1.8	high	8.5	3.8	0.05	26	10.6
PS	58	F	2	1	18.6	3.9	136	1.41	0.88	113.3	1.5	high	9.3	5	0.01	22	11
PS	37	F	2	1	16.3	3.5	145	1.15	1.16	87.46	1.8	high	7.9	6.6	2.1	26	10.2
PS	68	M	2	1	20.4	3.8	160	1.52	0.78	80.69	2.1	high		5.4	0.26	30	10.9
PS	71	M	2	1	21.4	4		1.64	0.86	135.5	1.6	high	7.6	5.5		33	10.3
PS	52	F	22	1	20.3			1.57	1.11	117.4	2.2	high	8.5	5.6			9.6
PS	68	F	22	1	17.6		187	1.75	0.91	98.57	1.4	high	9.3	5.6	0.04		8.8
PS	66	M	13	1	18		63	1.2	0.81	106.1	1.5	high	7.6	5.2	1.62	34	
PS	57	M	10	1	22.3			1.77	1.09	147.7	1.8	high	7.9	5.1		32	9.9
PS	60	M	8	1	22.5			1.41	0.76	128.8	1.8	high	8.8	5.8		20	11.9
PS	45	M	5	1	29.5		128	1.09	1.13	129.4	2.1	high	7.2	6.7	0.23	30	11.7
PS	43	F	5	1	19.4		153	1.7	0.74	110.8	1.8	high	11.5	4.4	0.02	21	10
PS	72	M	4	1	20.1		168	1.44	0.86	94.95	1.5	high		5.8	0.17		9
PS	66	M	4	1	22.6	4		1.58	0.77	104.5	1.6	high		3.7			8.4
PS	67	F	3	1	25.4		111	1.43	0.94	61.2	1.8	high	8.2	4.9	0	24	8.8
PS	48	M	3	1	23		199	1.53	0.93	96.72	1.8	high	9.8	5.1	0.02		10.5
PS	73	F	2	1	16.3		133	1.22	0.92	70.02	2.1	high		6.7	2.38		12.7
PS	68	M	2	1	18.9		123	1.36	0.82	43.88	1.3	high		5.6	0.02	23	10.9
PS	46	M	30	1	23.3	4	89	1.23	0.93	101.8	2.2	high	9.4	5.5	0		11.8
PS	64	F	22	1	23.8	3.8	134	1.41	1.08	112.6	1.8	high	9.3	4.3	0.1		11.7
PS	50	M	3	1	23.3	3.8	159	1.97	0.65	82.36	1.8	high	9.1	6	0.08	25	8.6
PS	29	F	12	1	18.4	4.1	134	1.72	0.96	120.3	2.1	high	9.5	4.8	0.03	27	12.7

PS	44	M	8	1	25.6	3.7		1.31	0.62	107.6	1.8	high	9.3	5.1		23	9.7
PS	38	M	5	1	27	4.2	177	1.39	0.96	134.4	2.1	high	8.3	6.1	0.04	27	11.3
PS	32	M	4	1	22	4.1	180	1.46	0.9	111.4	2.5	high	11.6	7.1		27	13.4
PS	59	M	4	1	21	3.6	139	1.43	0.66	99.27	1.5	high		3.4	0.01	29	12.2
PS	52	M	3	1	22.7	3.8	199	0.96	0.76	80.67	2.1	high		4.9	0.68	18	10.5
PS	18	F	2	1	22	4		1.45	0.77	82.36	1.8	high	9.8	7.6		27	10.8
PS	51	F	10	1	22.2	4.3		1.65	1.11	147.2	1.5	high	8.4	7.1		32	10.1
PS	68	M	9	1	25.9	3.6	204	1.21	0.73	131.4	1.3	high	9.2	4.9	0.05	28	10.9
PS	60	F	19	1	14.5	3.7	146	1.22	0.94	66.48	1.8	high		4	0.06		11.5
PS	54	F	6	1	23.8	3.6	126	1.17	0.92	95.69	1.9	high		5.3	0.16	23	11.6
PS	80	F	9	1	16.1	3.7	113	1.48	0.94	103.9	1.6	high		6.1	0.1	24	10.9
PS	60	M	6	1	25.8	4.8	134	1.64	0.81	127.3	1.9	high		7.3	0.1	25	10.3
PS	56	M	5	1	23	4	206	1.27	0.95	75.04	1.5	high	9.4	5.3	0.26	13	10
PS	72	F	4	1	19.3	3.8		1.29	0.97	61.3	1.8	high	9.2	5.9		33	10
PS	78	F	8	1	23.9	4	144	1.57	0.83	121.2	1.9	high		4.9	0.1	18	10.3
PS	65	M	7	1	20.5	4	92	1.44	0.94	124.8	1.3	high		4.3	0.1	30	11.6
PS	58	M	9	1	25.3	4.4	111	1.1	0.95	136.6	1.6	high		4.9	0.1	20	11.9
PS	69	M	8	1	17.3	4.1	164	1.34	0.87	137.9	1.6	high		4.5	0.1	20	10.7
PS	71	F	3	1	17.9	4.6	197	1.6	1	79.91	1.5	high	8.6	4	0.35	33	10.3
PS	53	F	29	1	19	4	160	1.61	0.95	105.8	2.1	high	8.5	5.7	0.02	23	10.8
PS	74	M	9	1	17.9	3.6	146	1.08	0.97	122.6	1.5	high	8.8	3.7	0.05	25	11.8
PS	38	M	4	1	26.6	4.3	141	1.15	0.74	91.1	1.8	high	9.5	6.2	0.11	21	10.1
PS	60	F	11	1	19.4	4.6	136	1.37	0.72	128.7	1.9	high		4.2	0.1	25	12.8
PS	82	M	10	1	22.7	3.8	140	1.37	0.92	112.8	1.9	high		4.9	0.1	28	11.5
PS	63	M	9	1	21.1	4	128	1.33	0.8	155.3	2.5	high	6.3	7	0.8		7.2
PS	73	F	7	1	25.6	3.8	138	1.37	0.85	106.2	2.1	high	9.7	6.1	0.18	28	10.7
PS	66	F	7	1	17.7	4.4	191	2.27	1	104.5	1.5	high		4.7	0.4		9.6
PS	38	F	17	1	10.9	4.6	141	1.28	1.04	93.05	1.5	high	8.1	3.8	0.02	26	12.9
PS	68	M	12	1	21.1	4	146	1.17	0.91	157	1.3	high	8.6	4.4	0.56		10.3
PS	70	M	12	1	18.9	3.7	199	1.09	0.9	86.06	2.1	high	9.1	5.6	0.12	25	9.7
PS	61	F	31	1	23.2	3.7	127	1.31	0.91	104.9	1.5	high	10.3	4.1	0.03	31	10
PS	65	F	2	1	18.9	3.4	176	1.7	1.06	82.61	1.6	high	8.3	4.6	0.1	28	9.7
PS	73	M	10	1	23.6	4	153	1.43	0.88	120.1	1.3	high		5.6	0.17	25	11.2
PS	67	F	9	1	16.6	3.8	144	1.28	1.03	120.9	1.8	high		3.7		21	11.1
PS	71	F	8	1	20.4	3.8		1.46	0.63	137.5	1.5	high	9.5	6.8	0.2	23	8.4
PS	42	F	10	1	20.7	3.9	86	1.44	1.12	125.9	2.1	high	8.4	5.4		23	11.3
PS	41	F	13	1	21.3	3.5	138	1.68	1.12	96.41	1.8	high	8.5	4.6	0.04	24	10.4
PS	59	M	11	1	20.5	3.8	120	1.55	0.99	123.3	1.6	high	9.2	5.4	0.1	34	11.5
PS	36	F	2	1	33.8	3.8	165	1.34	0.67	56.27	1.3	high		4.2			10.9
PS	54	M	3	1	24.4	4.2	158	1.11	0.97	80.17	1.8	high		7.2	0.21	28	11
PS	59	M	6	1	20.1	3.9	185	1.49	0.8	122.5	1.6	high		8.9			9.1
PS	69	M	4	1	20.5	3.5	116	1.29	1.05	96.76	2.1	high	8.4	6.5		24	10.9
PS	55	M	2	1	29	4.3	340	1.18	0.94	101.2	1.1	high		4	0.46	19	9.2
PS	51	F	7	1	28.6	3.9	158	1.38	0.84	80.14	1.3	high	8.8	6.1	0.7	21	11.7
PS	61	F	11	1	17.7	3.8		1.09	0.68	99.28	1.3	high	7.2	10.1	0.05	21	10
PS	61	F	25	1	18.1	3.5	137	1.58	1.03	126	2.1	high	8.5	5.6	0.1		12.4
PS	57	M	21	1	23.1	3.6	163	1.09	0.71	93.9	2.1	high	8.2	6	0		9.1
PS	71	M	21	1	21.2	3.7	84	1.08	0.94	99.63	2.1	high	8.5	5.3	0.2		10.4
PS	45	F	19	1	16.9	4.1	189	1.75	0.8	83.35	2.1	high	8.6	6.7	0.23		11.5
PS	59	M	19	1	20.4	3	192	1.35	0.79	88.2	2.1	high	9.3	3.7	0.1		10.5
PS	75	F	16	1	18	3.7	187	1.91	0.94	107.3	2.1	high	8.8	7.2	0		10.7
PS	54	F	15	1	20.8	3.8	133	1.67	0.95	134.4	1.8	high	8.8	5.4	0		10.3
PS	59	F	11	1	20.1	3.6	125	1.44	0.93	126.3	1.8	high	9.9	4.3	0.05	23	11.5
PS	83	M	9	1	23.4	3.7	129	1.43	1.01	116.9	1.5	high	9.8	4.8	0.3	32	6.6
PS	60	M	9	1	18.5	3.7	142	1.76	0.82	80.27	1.5	high	9.2	3.7	0.14	27	11.2
PS	66	F	9	1	23	3.4	134	1.48	1.07	93.9	1.5	high	8.9	5.3	0.06	29	11.2
PS	68	M	7	1	26.2	3.9	111	1.32	0.99	103.8	1.5	high	8.4	4.2	3.32		9
PS	50	M	6	1	19.5	2.5	119	2	1.06	104.3	1.8	high	8.4	6.6	0.05	24	9.9
PS	59	M	6	1	23.8	4	195	1.02	1.13	129.2	1.6	high		5.5	0.8	28	14

PS	38	M	5	1	24.1	3.9		1.38	0.66	67.27	2.2	high	9.6	3.9	0.04	28	10.8
PS	59	M	3	1	24.4	3.8	118	1.32	1.03	123.2	1.3	high	7.8	4.6	0	27	10.4
PS	70	F	2	1	23.9	3.4	155	1.13	0.73	110.5	1.5	high	9.3	3.4	0.57	31	8.7
PS	58	F	2	1	17.8	4	94	1.39	1.11	96.91	1.4	high	8.7	3.7		31	
PS	60	M	2	1	20.6	3.1	176	1.06	0.69	32.4	2.1	high	8.3	4.6	0.52	24	11.8
PS	64	M	2	1	25	4.1	124	0.94	0.83	83.73	2.1	high	7.8	6			11.1
PS	68	M	2	1	19.7	3.4		1.24	0.79	45.4	1.5	high	9.9	5		25	13.5
PS	77	F	2	1	19	3.6	174	1.26	0.88	86.34	1.8	high	9.1	6.2	0.05	28	12.9
PS	51	M	2	1	21.3	4.1	211	1.28	1	64.78	1.5	high	8.5	6.3	0.1	23	9.9
PS	71	M	4	1	19.3	3.6	172	1.31	0.77	105.7	1.6	high		5.7			10.4
PS	70	M	16	1	18.6	4.3	178	1.23	1.02	121.5	1.8	high	9.2	6.2		19	10.6
PS	64	M	6	1	18.3	3.9	129	1.49	0.78	71.46	1.3	high	8.3	3.4	0	26	9
PS	77	M	2	1	25.3	3.6	268	1.67	0.9	104	1.5	high	9.8	3.7	0.9	21	11
PS	68	M	3	1	17.4	3.2	161	1.37	1.07	90.09	1.3	high	8	3.7	0.25	34	10
PS	63	M	2	1	26.6	3.7	93	1.26	0.74	104.2	2.1	high	7.8	7	0.06	29	14.1
PS	55	F	27	1	17	3.8	188	1.38	0.99	109.2	1.5	high	9.5	5.4	0.31	32	10.8
PS	51	M	7	1	26.5	4.3	155	1.29	1.07	96.77	1.8	high	8.4	4.6	0.18	28	10.5
PS	60	M	7	1	21.4	3.8		1.26	0.83	109.7	1.5	high	10	5	0.12	24	9.7
PS	69	F	7	1	19.2	3.9	173	1.72	0.82	87.35	1.8	high	9.4	4.8	0.05		11
PS	45	M	5	1	19.2	3.8	97	1.33	1.2	116.7	1.5	high	8.5	4.6	0.03	23	10.2
PS	52	F	15	1	29.9	3.7	159	1.46	1.19	116.7	1.5	high	9.4	6.2	0.54	30	9.8
PS	76	F	31	1	19.8	3.4	167	1.23	0.76	93.14	2.1	high	9.2	7	0.1	23	9.7
PS	58	F	30	1	18.7	3.6	181	1.53	1.26	94.54	1	high	9.3	6.1	0.05	33	10.3
PS	51	F	17	1	19.2	3.9		1.65	0.89	89.64	1.8	high	9.4	6.1			9.3
PS	73	M	17	1	23.2	4	208	1.87	0.8	84.25	1.6	high	10	6.2	0.03	23	8.8
PS	48	M	14	1	20.8	4	192	0.91	1.11	121.1	1.9	high		4.1			10.9
PS	62	M	14	1	18.3	3.4	176	1.77	0.75	108.2	2.1	high	8.7	5.2	0.1	24	9.6
PS	74	F	12	1	20.3	3.7	183	1.29	1	111.8	1.5	high	8.1	5	0.3	30	10.9
PS	59	F	11	1	27.9	3.7	166	1.68	0.68	131.7	1.3	high	9.6	5.5	0	35	11.3
PS	74	M	10	1	18.7	3.3	185	1.53	0.71	106	2.1	high	9.4	3.3	0.06	25	10.1
PS	56	F	9	1	17.8	4.4	184	1.67	1.09	133.2	1.3	high		3.8		36	10
PS	69	M	9	1	21.3	3.9	110	1.4	1.08	124.9	2.1	high	8.3	6	0.05	39	10
PS	59	F	9	1	17.5	4.5	155	0.98	0.99	126.9	1.3	high		3.5			11.7
PS	56	M	8	1	22	3.5	122	1.48	0.58	72.49	2.1	high	9.1	3.1	0.1	27	10.4
PS	58	F	7	1	18.1	4	165	1.61	0.97	126.9	1.8	high	8.2	4	0.34	32	11.6
PS	60	M	7	1	25.8	4	150	1.3	0.78	96.68	1.1	high	9.1	5.2	0.75	32	9.2
PS	55	F	5	1	24.8	3.7	155	1.15	1.05	133.2	2.1	high	8.1	6.4	0.35	21	10.2
PS	60	F	5	1	31.4	4	131	1.12	0.72	81.63	2.1	high	9	6.7	0.02	23	9.9
PS	72	M	4	1	23.4	4.4	112	1.04	0.93	122.3	1.8	high	9.5	6.1	0.25	26	9.2
PS	45	F	4	1	19.6	4.3	186	1.37	1.13	145.4	1.8	high	10.2	4.2		26	13.5
PS	48	F	4	1	20.5	4.2	194	1.41	0.87	111.6	1.5	high	8.3	4.9	0.06	16	9.5
PS	72	M	4	1	18.3	3.9	112	1.91	0.94	104.7	1.9	high		5.6			9.6
PS	68	M	4	1	25.5	3.9	147	1.43	0.92	107.2	2.1	high	7.9	4.2	0.3	31	10.1
PS	41	F	4	1	17.9	4.3	109	1.05	1.1	137.4	2.1	high		6.7	1.17		10.6
PS	71	F	2	1	17.4	4	117	1.15	1.15	92.04	1.6	high		4.8	0.21	30	10.1
PS	50	F	2	1	20.6	4.1		2.09	1	116.2	2.1	high	7.8	4.6	0.11	34	7.9
PS	70	F	2	1	20.4	4		1.96	0.72	118	2.1	high	10.6	4.7	0.07	29	9.5
PS	58	M	2	1	19.6	3.8	119	1.12	0.94	76.82	2.1	high	9.4	6.9			12
PS	57	M	26	1	19.1	3.4	153	1.49	0.84	103.6	1.6	high	10	5.7		30	10.8
PS	63	M	22	1	26.2	3.8	230	1.91	0.81	116.6	1.8	high	10	6.5	0.61	29	8.3
PS	67	F	20	1	18.2	3.4	108	1.44	0.82	133.8	1.6	high		7.2	0.21	29	11.9
PS	61	M	16	1	22.1	3.8		1.65	0.85	102.2	2.5	high	11.6	6		30	11.1
PS	59	F	15	1	24.3	3.8		2.19	1.07	122.1	2.1	high	9.1	3.6	0.92	26	8.1
PS	65	M	14	1	16.4	4.1	105	1.37	1.13	100.4	2.1	high	7.7	5.7	0.71	33	10.9
PS	64	F	12	1	19.3	3.4	172	1.9	1.05	134.7	1.6	high		6.6	0.04	26	9.9
PS	38	M	12	1	20.6	3.9	159	1.59	0.89	99.24	1.6	high	8.5	5.2	0.05	38	8
PS	82	M	11	1	17.5	3.5		1.5	0.56	140.9	2.1	high	9.9	5.8	0.03	25	9.7
PS	56	M	11	1	20.5	3.7	171	1.71	0.79	85.51	2.1	high	9.5	3.5	1.53	29	9.3
PS	85	M	10	1	19.5	3.6		1.78	0.9	119.2	2.1	high	9.4	5.3	0.06	29	11.3

PS	66	M	10	1	25.8	3.7	150	1.19	0.76	118.6	1.5	high		5.8	1.59	34	9.2
PS	60	F	10	1	19.9	4		1.13	0.95	130.4	2.1	high	8.6	7.8	0.03	19	10.7
PS	58	F	9	1	25.2	3.9	136	1.1	1	116	2.1	high	8.6	5.9	0.04	20	11.1
PS	54	F	9	1	23.5	3.9		1.95	0.95	105.5	1.5	high					
PS	63	M	8	1	19.3	4.2		1.46	0.92	112.8	2.1	high	9.2	5.4	0.09	32	10
PS	60	F	8	1	21.3	3.5	180	1.96	0.85	115.3	1.8	high	9.1	3.9	0.1	20	9.5
PS	62	F	7	1	18.9	3.9	102	1.25	0.88	131.8	1.6	high		7.1	0.03	32	10.4
PS	67	M	7	1	23.2	3.8	160	1.43	0.93	109.6	1.6	high		6.9	0.19	29	12.3
PS	57	F	7	1	26.6	3.8		1.79	0.75	115.6	1.8	high	9.7	5.6		27	9.5
PS	63	M	7	1	18.5	4.3	164	1.4	0.95	116.4	1.6	high		4.3	0.07	12	11.6
PS	82	F	7	1	14.9	3.4	130	1.11	0.71	91.6	1.3	high	7.4	6.1	0.1	22	9.4
PS	54	F	6	1	19.1	4		0.95	0.82	117.8	2.1	high	9.5	6.3	0.35	26	9.6
PS	43	M	6	1	25.5	4.3	156	1.13	0.81	85.72	1.5	high	8.7	4.1	0.11	21	9.4
PS	45	M	6	1	24.7	4.2	160	1.13	1.01	109.3	1.8	high	10.5	6.8	7.3	36	9.4
PS	73	M	6	1	23.8	4		1.78	1.13	110.5	2.1	high	9.4	5.1	0.08	27	9.2
PS	76	F	6	1	26.9	3.4		1.63	0.85	111.6	2.1	high	9.7	3.4	0.6	30	9.6
PS	58	M	6	1	20.7	4.4	165	1.29	0.83	108.3	1.8	high	8.7	5.6	0.08	30	10.6
PS	39	F	6	1	18	3.7		2.35	0.95	135.5	1.5	high	7.9	4.4		26	11
PS	49	M	6	1	26.8	4.2		1.37	0.84	94.66	1.8	high	10.2	4.9		26	10.3
PS	63	M	5	1	24.8	4	119	1.58	1.01	126.6	2.1	high	10.5	3.9	0.02		10.6
PS	55	M	5	1	16.6	3.4	114	1.4	0.83	93.48	2.1	high		6.5	0.05	23	10.4
PS	47	M	5	1	21.1	4	139	1.65	1.14	105.9	1.6	high		3.9	0.1		10.5
PS	59	F	5	1	24.7	4.4	162	0.85	1.06	110	2.1	high	8.2	7.5	0.14	22	9.5
PS	51	F	5	1	20.8	3.9	165	1.51	0.91	108.6	1.8	high	10	6.9	0.2	29	9
PS	77	M	4	1	23.9	3.8		1.26	1.14	134	2.1	high	8.9	3.7	0.02	20	10.2
PS	69	M	4	1	20.8	3.5	162	1.14	0.93	118.7	2.1	high	8.5	5.1			12.8
PS	70	M	4	1	25.2	3.7	103	1.43	0.89	100.3	1.5	high	10	4.3	0.1	19	12.3
PS	57	M	4	1	28.7	3.7		1.49	0.97	90.65	2.1	high	8.9	2.7	0.01	23	12.6
PS	65	M	4	1	20.2	4.4	138	1.23	0.91	117.2	1.3	high	9.9	4.3	0.01	35	10.1
PS	81	M	3	1	19.1	3.7	141	1.93	1.05	110.8	1.8	high		4.6	0.03	28	11.4
PS	53	F	3	1	20	3.2	123	1.48	0.76	61.5	1.6	high	8.5	4.4	0.6	33	11.5
PS	61	M	3	1	18.8	3.7	102	1.78	0.71	70.23	1.3	high		3.4	3.2	36	7
PS	77	F	3	1	21.3	3.9		1.46	0.86	102	1.9	high		5.3	0	28	12.7
PS	52	M	3	1	17.9	3.7	198	1.88	1.18	118.5	1.5	high	10.8	6.5		25	9.5
PS	62	M	3	1	19.5	3.8	198	1.22	0.75	82.74	1.3	high		4.3	0.3	28	11.1
PS	53	M	3	1	26.5	4	174	1.28	0.86	120.6	1.6	high	9.1	6.4	0.27		9.3
PS	50	M	2	1	21.5	2.9	160	0.9	0.74	92	1.5	high	9.4	5	0.11	13	10.4
PS	73	M	2	1	24	4.1	124	1.23	0.72	102.3	1.9	high		4.8		23	11.2
PS	53	M	2	1	32.4	4.1	107	1.44	0.96	80.64	0.8	high	8.3	6.1	0.05	24	7.6
PS	67	F	7	1	20.6	4	163	1.55	1.05	119.5	1.9	high		4.1		31	12.3
PS	58	M	8	1	21.6	3.8	131	1.62	1.41	87.77	1.8	high	9.5	4.1	0.3	28	10
PS	59	F	2	1	29.4	4.2	170	1.12	0.91	119.3	1.6	high		4.6		36	11.1
PS	56	F	10	1	21.6	3	207	1.34	0.91	132.6	1.8	high	9.2	6.4	2.69	32	10
PS	59	F	14	1	16.9	4.2	141	1.67	1.09	54.78	1.8	high		4.9	0.05		9.4
PS	55	M	8	1	22.3	3.7	141	1.82	0.8	114.8	2.1	high	9.3	3.9	0.1	26	11.5
PS	56	F	4	1	26.2	3.6	127	1.26	0.55	96.78	1.4	high		5.6	0.05	22	10.8
PS	61	M	2	1	22.3	4	164	1.44	0.71	148.7	1.5	high	9.2	5.2	0.1	27	10.8
PS	56	F	2	1	25.3	3.8		1.21	1.03	107.4	2.1	high	10.1	6.4	0.8	19	9.6
PS	61	M	10	1	18.1	3.9	187	0.93	0.78	96.65	2.1	high	8.5	5.6		24	10.9
PS	79	F	10	1	17.5	3.5	114	1.66	0.87	83.24	1.8	high	8.8	3.6			9.7
PS	64	M	13	1	24.9	3.7	109	1.62	0.73	116.2	1.6	high	8.7	4.3		40	10.2
PS	72	M	5	1	18.5	4.6	178	1.69	0.92	127.4	1	high	8.4	7.3			9.7
PS	88	M	10	1	17.5	3.5	119	0.94	0.87	108	1.5	high		5.6	1.56	38	9.9
PS	61	F	34	1	21.8	3.7	180	1.25	0.99	81.6	1.8	high	9.3	6.6	0.05	28	11.6
PS	65	M	14	1	22.8	3.3	181	1.18	0.85	85.17	1.3	high	8.4	5.2	0.3	20	5.7
PS	76	F	13	1	22	3.5	138	1.39	0.75	99.77	1.6	high	10.5	5.2	1		11.1
PS	66	F	32	1	21.1	3.8	192	1.47	1.1	97.57	1.9	high		4.8		29	12
PS	56	F	2	1	19.4	4	152	1.38	1.03	100.1	1.5	high	8.5	5.7	0.3	25	9.8
PS	48	F	17	1	17.2	3.7	146	1.68	1.12	111.3	2.1	high	8.9	5.1		31	11.7

PS	76	F	2	1	21	3.5	168	2.04	0.75	85.41	1.6	high		5.5		32	10.2
PS	60	F	34	1	21.5	3.1	140	1.51	1.2	93.04	1.8	high	8.8	5.9	0.05		11.1
PS	65	M	30	1	20.8		153	1.3	1.29	109.6	1.4	high	8.7	5.1	0.25	2	10.7
PS	69	M	6	1	27.1	4	117	1.58	0.91	133.5	1.8	high	8.6	4.8	0.04	19	14.4
PS	54	M	31	1	24.1	3.7	140	1.61	0.91	114.2	1.5	high	9.1	4.4	0.05	28	12.1
PS	68	M	8	1	22.4	3		1.68	1.03	101.6	2.5	high	9.3	4.3		20	8.9
PS	64	F	7	1	22.6	4.1		1.97	0.8	148	1.5	high	8	5.3		17	9.7
PS	60	F	28	1	17.3	4.6	133	1.32	1.08	130.6	1.8	high	10.6	5.5	0.28	29	12.5
PS	47	M	24	1	18.6	3.7	158	1.4	1.26	107.3	1.8	high	8.8	5.7	0.06	33	11.9
PS	63	M	19	1	16	3.7	146	2.29	1	75.17	1.4	high	8.4	4.7		30	9.8
PS	61	M	17	1	19.1	3.1		1.79	1.06	80.8	1.8	high	9.1	6.5	0.03	21	11.1
PS	58	M	13	1	20.4	3.8	193	1.54	1.02	103	1.9	high		5.9		28	11
PS	56	M	12	1	23.3	4.5	152	1.36	0.98	80.3	1.9	high		5.5		27	11.5
PS	78	F	9	1	25.1	3.3	173	1.48	1.02	180	1.5	high	9.5	6.9	0.07	27	8.7
PS	45	F	8	1	14.2	3.4	175	1.39	0.99	89.91	1.8	high		4.7	0.01	26	12
PS	70	M	5	1	21.3	3.7	145	0.81	0.94	126.2	1.3	high	8.5	4.9	0.05	19	10.8
PS	78	F	5	1	19.1	2.9	113	0.93	0.93	111.4	1.9	high		4.2		29	9.4
PS	51	M	3	1	23	4		1.03	0.79	116.2	1.5	high	8.8	3.9	0.1	22	10.8
PS	55	M	2	1	23.1	3.3	181	0.88	0.68	99.04	2	high	9.2	2	2.07	31	12.1
PS	59	M	2	1	20.4	3.9	154	1.54	0.54	73.86	1.8	high	8.7	4.5			9.9
PS	36	M	9	1	22	3.3	151	1.15	0.77	103.1	1.6	high		4.7	0.06	32	11.3
PS	60	M	2	1	20.6	3.7	160	1.1	1.36	106.6	1.8	high	9.2	6	0.03	28	10
PS	79	F	2	1	23.6	3.6	166	1.03	0.51	86.94		high	9.6	6.6	0.14	25	10.8
PS	74	M	5	1	22.1	3.9	158	1.94	1.05	98.89	1.5	high		5.4	0.06		11.8
PS	76	M	11	1	22.3	4		1.91	0.85	126.3	1.8	high	8.4	7.8			9
PS	56	M	8	1	27.6	4.3		1.96	0.85	136.9	1.8	high	9.3	7.4		27	8.4
PS	44	F	23	1	17.5	4.5	130	1.18	1.13	89.1	1.9	high		4.7		18	10.9
PS	71	M	3	1	19.6	3.1	123	1.32	0.7	90.63	1.5	high	8.3	2.8			5.1
PS	67	F	4	1	28.5	3.3	123	1.13	0.8	87.06	1.8	high	8.7	7.5	0.13	26	9.8
PS	71	M	2	1	23.3	3.1	170	1.5	1.01	108.2	1.6	high	10.4	5.4	0	23	9.7
PS	75	F	5	1	25	3.6	127	1.87	0.71	106.8	1.6	high	8.6	5.3	0.03	26	9.9
PS	69	M	3	1	18.6	3.4	231	1.99	0.68	98.8	1.9	high		6.2			9.9
PS	69	M	2	1	22.5	3.7	184	1.09	0.7	139.6	2.1	high	9.4	5.4		18	9.2
PS	76	M	4	1	24.1	3		1.31	0.71	84.76	1.9	high		5.2			10
PS	70	F	12	1	21.5	3.6	140	1.17	0.88	135.8	2.1	high	10.9	5.7	0.1	24	11
PS	64	M	7	1	22	3.6	105	1.16	0.87	119.4	1.8	high	9	5.9	0.1	26	10.3
PS	73	F	7	1	19.9	3.7	114	1.53	0.94	73.88	1.8	high	11.3	7.6	0.27		11.5
PS	59	M	2	1	18.8	3.4	135	1.7	0.82	87.16	2.1	high	9.8	4.5	0.06	25	10.4
PS	67	M	2	1	18.4	4.1	175	1.61	0.96	100.4	2.1	high	9.6	9.8		28	10
PS	56	F	9	1	19.1	3.7	142	2.01	0.99	88.68	2.1	high	9.7	4.5	0.05	34	10.8
PS	55	M	7	1	19.7	4.5		1.58	1	113.9	1.9	high		6.5			11.4
PS	65	M	23	1	18.7	3.5		1.75	0.48	123	1.9	high		6.9			10
PS	65	M	3	1	23.3	4.2	116	1.46	0.96	123.1	2.1	high	8.7	6.8	0.1	22	10.2
PS	48	F	3	1	18.1	4.5	238	1.68	1.34	119.4	1.3	high		5.4	0.05		11.9
PS	55	M	3	1	20.5	4.3	128	1.48	0.99	74.04	2.1	high	9.9	6.1	0.1	22	10
PS	66	M	6	1	23	4.1	112	1.38	0.77	100.1	2.1	high	8.8	6.9	0	29	10.4
PS	64	M	35	1	20.7	3.9		1.37	0.88	80.11	1.9	high		5.1			11.1
PS	66	M	25	1	21.2	3.6	112	1.24	0.82	95.71	1.9	high		6.9		6	10.9
PS	55	F	16	1	20.5	4.1	128	1.4	0.89	109.3	1.1	high	8.9	4.4	0.03	27	13.6
PS	43	F	15	1	17.3	4.1	155	1.27	0.87	124.1	2.1	high	8.7	4.8	0.1	27	11.4
PS	35	M	13	1	23.5	4.2	184	2.12	0.98	104	1.5	high	9.4	7.3	0.1	28	12.7
PS	81	M	7	1	17.7	4	179	0.92	0.9	110.9	1	high	8.6	3.6	0.05	20	13.3
PS	65	M	7	1	18.5	3.3	188	1.43	0.64	71.15	2.1	high	8.9	4.1	0.3	25	9.9
PS	74	F	6	1	29.6	4.1	159	1.24	0.77	121.5	2.1	high	9.3	3.4	0	28	10.2
PS	69	M	5	1	20.3	3.6	149	1.13	0.88	100.1	2.1	high		4.3	0.19	30	9.5
PS	62	M	4	1	22.9	3.9	149	1.53	1.19	108.3	1.8	high	10.1	4.7	0.02		10.6
PS	79	M	3	1	18.5	3.4	143	1.44	0.8	68.73	1.5	high	9.7	2.7	0.63	23	11.5
PS	64	M	3	1	21.9	3.6	206	1.1	0.72	107.5	2.1	high		8.3	0.22	28	10.3
PS	72	F	2	1	21.6	3.6	144	1.54	0.73	97.03	1.6	high	9.7	5.7	0		9.6

PS	71	F	2	1	31.2	4	142	1.52	0.58	71.21	2.1	high	7.6	4.8	0.15	25	9.4
PS	34	M	2	1	21.7	4.2	209	1.55	0.71	99.89	1.8	high	8.3	5.4	0.07	28	11.1
PS	58	F	10	1	21.4	3.8	138	1.45	0.8	102.1	2.1	high	8.6	7.4	0.6	33	9.5
PS	63	F	3	1	21.8	4.2	200	1.77	1.07	116	1.5	high	9.3	7	0.04	23	10.3
PS	70	F	2	1	22.3	4	214	1.41	0.88	75.15	1.6	high	8.1	3.7	0		10.7
PS	61	M	11	1	22.3	3.6	154	1.54	0.75	115.7	1.5	high		7.7	0.06	19	9.7
PS	63	M	2	1	21.4	3.8	165	0.98	0.7	77.05	1.8	high	7.6	4.3	0.31	30	11.6
PS	67	M	9	1	23.1	3.7	130	1.68	0.81	120.3	2.1	high	9.4	6.5	0.3	26	11.5
PS	67	M	5	1	25.6	3.5	112	1.65	1.14	111.1	1.8	high	8.6	5.4	0.02	24	9.9
PS	40	F	2	1	33.2	3.2	132	1.28	0.87	94.37	2.1	high	7.8	4.7	0.1	26	9.2
PS	43	F	14	1	18.2	4.1		1.39	1.11	105.6	1.9	high		4			10.2
PS	71	F	13	1	19	3.7	139	1.62	0.91	139	1.9	high		5.8			12.4
PS	46	M	13	1	24.8	4		1.52	0.63	103.5	2.5	high	9.4	7.3		33	11.8
PS	61	F	13	1	21.6	3.7	113	1.33	0.97	148.1	1.9	high	9.1	6.8	0.28	36	12.9
PS	72	M	12	1	21.3	4	152	1.57	0.75	117	1.2	high	10	5.4	0.07	16	9.7
PS	73	M	9	1	21	4	155	1.54	0.84	133.6	2.1	high	9.8	8.1	0.1	37	11.1
PS	72	M	8	1	20.8	3.8	145	1.25	0.88	132.1	1.5	high	9.6	3.1	0.63	24	11.7
PS	69	F	7	1	20.9	3.8	150	1.54	0.85	163.8	0.9	high	10.4	6.4	0.32	27	12.2
PS	21	F	7	1	19.1	4.2	155	1.13	0.9	88.29	1.6	high	9.5	6.4	0.5	20	12
PS	61	M	6	1	20.5	3.8	188	2.07	1.03	119.8	1.1	high		3.4	0.18	37	11.4
PS	42	F	4	1	20	4.3	149	1.44	0.66	85.01	1.3	high	8.6	5.4	0.09	34	10.9
PS	64	M	8	1	23.8	4.1	191	1.25	1.04	102	2.1	high	9	5.6	0	22	11.5
PS	63	M	3	1	17.9	3.4	128	1.51	0.74	48.13	1.3	high		5.6	0.01	25	10.1
PS	32	M	8	1	17.6		127	1.8	0.95	90.98	1.8	high	8.6	5.1	0.53		11.5
PS	66	M	6	1	25.4	4.1		1.5	1.24	132.8	1.8	high	6.3	4.2		48	4.7
PS	55	F	5	1	18.6	4.4	126	1.45	0.62	84.08	1.6	high	9.4	6.9	0.5	30	12
PS	49	M	4	1	21	4	126	1.54	0.69	81.62	2.1	high	8.9	6.9	1.3	28	10.6
PS	65	M	4	1	22.4	3.5	85	1.37	0.9	110.3	2.1	high	7.9	3.7	0	45	11.1
PS	52	F	4	1	24.8	3.3	156	1.29	0.73	51.44	2.1	high	8.7	6.5	0	35	11
PS	54	M	2	1	21.5	3.9	103	1.49	0.98	98.29	1.5	high	7.5	5.2	0.45	26	9.4
PS	74	F	2	1	16.1	4.1	150	1.45	0.81	152.2	1.6	high	9.2	5.3	0.26	28	11.8
PS	71	M	11	1	23.6	3.6		2.02	0.62	111	2.1	high		4	0.05	32	10.7
PS	62	M	7	1	16.7	2.8	112	1.31	0.74	78.97	1.8	high		4.4	0.04		10.5
PS	85	M	10	1	20.1	3.5	213	1.82	1	143.8	1.6	high	8.9	4.6	0.26	32	11
PS	65	F	5	1	24.2	3.9	98	1.57	1.1	83.71	1.5	high		5.9	0.28	28	10.5
PS	88	F	3	1	19.5	2.5	151	1.09	0.72	98.29	1.8	high		7	0.06	27	9.8
PS	61	F	30	1	20	2.8		1.11	1.05	77.47	2.1	high	9.2	5.3	1.11	26	13.4
PS	56	M	23	1	17.9	4.1	106	1.16	0.8	116.5	1.6	high	8.3	7.5	0.35	25	11.5
PS	60	F	22	1	20.6	4.1	146	1.31	1.13	94.09	1	high	10.1	8.3	0.06		11
PS	50	F	21	1	18.9	3.5	84	1	0.97	125.9	1.5	high		6.5		19	7.9
PS	63	M	19	1	19.8	4	196	1.34	1	104.1	1.6	high	9.5	5.8	0.68	31	10.5
PS	74	F	19	1	20.9	3.8	128	1.52	0.83	130	1.6	high	9.2	6.2	0.03	22	10.8
PS	68	F	19	1	20.1	4	134	1.75	0.88	123.2	1.5	high	9.4	6			11.9
PS	70	M	17	1	22.2	3.3	153	1.93	0.57	106.1	2.1	high	8.7	6.1	0.12	26	10
PS	71	M	15	1	20.4	3.7		1.88	0.95	119	1	high	8.1	5.9	0.09	34	8.8
PS	62	M	13	1	21.6	4	162	2.12	1.06	88.9	1.1	high	8.5	4.8	0.1	25	11.8
PS	47	M	11	1	21.3	4.3	135	1.51	0.71	92.61	1.4	high	8.8	4.4	0.2	23	11.4
PS	69	M	11	1	21.6	3.7	244	1.09	0.89	123.1	1.1	high	9.1	3.6	0.56	24	9.9
PS	36	M	10	1	19.5	4.4	170	1.67	0.99	110.8	1.6	high	9	4.8	0.02	27	9.7
PS	39	M	9	1	24.7	4	140	1.29	0.76	134	1.3	high		5.4		23	8.8
PS	65	F	9	1	22.7	3.7	146	1.35	0.85	116.4	1.5	high	8.4	4.1	0.05	24	11.6
PS	68	F	8	1	25	4.5	112	1.02	0.94	94.12	2.1	high	9.4	5.9	0.23	23	12.9
PS	41	M	8	1	19.9	3.9	119	1.6	0.81	96.57	1.5	high	9.3	4.6			11
PS	46	M	8	1	21.1	4		1.25	0.74	92.84	1.5	high	8.9	6.5	0.16	27	15
PS	70	M	8	1	21.2	3.8	129	1.91	0.75	131.8	1.8	high	9.6	5.3	0.1	19	10
PS	45	M	7	1	31.8	3.7	113	1.39	0.62	114.9	1.8	high	9.8	5.1	2.17	27	9.2
PS	58	M	6	1	21.9	3.8	153	1.48	0.93	140.6	1.6	high	9.5	6	0	32	10
PS	44	M	6	1	22.5	3.5	177	2.58	0.89	105.9	1.5	high	8.6	6.2	0.05	21	9.8
PS	38	F	5	1	24.3	3.7		1.59	0.59	73.97	2.1	high	10.5	6.7		29	11.6

PS	61	F	5	1	18	2.8		1.78	0.99	70.67	1.5	high	8.3	5.6	0.05	22	9.5
PS	67	M	3	1	21.3	4.1	152	1.06	0.96	139.1	1.5	high	8.9	5		23	10.5
PS	80	M	3	1	19.5	3.7	199	1.63	0.64	110.8	1.6	high	9.5	6	0.03	25	9.7
PS	70	F	2	1	21.1	3.8	238	0.87	0.67	94.82	2.1	high	9.1	5.2	0		11.4
PS	70	F	2	1	17.5	4	160	1.73	0.85	41.12	1.3	high	8.6	6.7	0.04	31	10.1
PS	61	M	25	1	19	3.5	135	1.57	0.78	108.4	1.6	high	7.8	6.5	0	27	10.9
PS	42	M	21	1	30.3	4	184	1.28	0.8	120.9	1.6	high	10	6.1	0.12	29	12.2
PS	39	F	15	1	17.9	3.9	167	2.08	0.95	101.3	1.8	high	8.8	3.9	0.1	20	9.1
PS	58	M	6	1	22.6	3.7	134	1.25	0.96	117.3	2.1	high	9.7	3.9	0.1	26	9.4
PS	56	M	5	1	27.2	4.2	124	1.16	0.76	116.9	1.8	high	8.9	6	0.17	27	9.8
PS	65	M	4	1	20	4	155	1.49	0.95	105.2	1.8	high	9.3	6.3	0.09	27	8.8
PS	58	M	3	1	34.4	3.9	124	1.8	0.83	113.8	2.1	high	8.2	7	1.44	33	5.8
PS	63	M	2	1	26.8	4		1.68	0.77	88.93	1.3	high	9.3	4.5	3.68	33	10.4
PS	77	F	11	1	17.3	3.6	165	1.28	0.85	128	1.6	high	9.3	5.8	0.23	27	13.7
PS	55	M	15	1	22.8	3.3	137	1.36	0.74	106.4	1.5	high	8.6	5.3	4.5	34	9.6
PS	80	M	5	1	23	3.6	135	1.85	1.12	167.3	1.1	high	9.1	4.1	0.03	24	9.6
PS	61	M	3	1	27.5	3.7	153	1.13	1.01	106	2.1	high	9	5.1	0.23	24	11
PS	56	M	26	1	18.7	3.7	175	1.6	1.07	124.8	1.8	high		3.8	0.02	29	9.3
PS	52	F	22	1	19.7	4.2	108	1.18	1.14	127.4	1.5	high	9	6	0.3		11.2
PS	72	M	17	1	25.7	3.4	157	1.65	0.9	132	1.8	high	9.7	4.7			11.1
PS	47	M	16	1	20.5	3.9	217	0.98	0.91	120.8	1.8	high		4.7	0.04	31	11.7
PS	59	M	14	1	21.7	3.9	160	1.84	1.07	112.4	1.3	high	9.1	5.5	0.03	29	9.7
PS	78	M	13	1	18.8	3.6		0.96	0.7	111.1	0.6	high	8.6	2.7	0.29		11.1
PS	63	M	11	1	15.2	3.8	189	1.44	0.79	118.1	1.8	high		5	0.12	26	13
PS	71	F	10	1	16.9	3.8	179	1.56	1.02	116	1	high	9.2	6.8	0.1	11	
PS	46	F	9	1	20.4	3.9	143	1.14	0.99	105.7	2.1	high	8.8	5.6	0.7	27	11.2
PS	55	M	7	1	20.4	4.4	143	1.26	0.77	119.3	1.5	high	9.8	5.6	0.05	32	10.2
PS	54	F	7	1	21.6	3.6		1.61	0.72	106.1	1.8	high	9.7	5.2	0.05	25	9.5
PS	55	F	4	1	21.9	3.6	98	1.84	0.92	74.44	2.1	high	8.1	5.9	0.04	28	10.8
PS	77	F	4	1	18.6	3.4	116	1.56	0.81	126.2	1.8	high		2.9	0.13	27	8.5
PS	58	F	3	1	19.1	3.8	128	1.33	0.95	109.1	2.2	high	9.3	4.3		31	10.3
PS	61	F	2	1	21.8	3.8	149	1.41	1.12	123.1	1.5	high	8.5	5.8	0.41	28	10.5
PS	41	F	2	1	20.2	3.6	156	1.86	0.79	59	1.8	high	8.4	3.5	0.11	28	11.5
PS	56	M	2	1	21.6	3.6	132	1.58	0.97	75	2.1	high	7.6	4	1.1	28	10.2
PS	71	M	4	1	23.4	3.7	119	1.44	0.83	158.7	1.8	high	11	5.4	0.04	33	10
PS	55	M	2	1	24.8	4.1	89	1.79	0.76	106.2	1.8	high	7.7	5.2	0.1	35	9.9
PS	70	F	21	1	16	3.6		2.56	0.82	99.7	1.5	high	9.3	6.3		27	
PS	39	F	16	1	17.9	3.7		1.32	0.93	128.6	2.5	high	8.8	5.8		24	10.4
PS	68	M	2	1	19.6	2.9	177	1.5	0.5	106.6	2.1	high	7.8	2.9	0		12.1
PS	65	F	7	1	20.5	3.6		1.44	0.77	117.7	2.1	high	9.1	3.2	0.1	27	11.5
PS	70	F	5	1	22.2	3.2	116	1.31	0.77	134.7	2.1	high		3.5			10.1
PS	55	M	8	1	24.6	3.7		1.31	0.93	6.83	2.1	high	9.6	6.4		27	9.6
PS	57	M	4	1	21.6	3.6	106	1.59	0.84	99.6	1.5	high	9.4	4.4	0.12	44	11.2
PS	55	M	13	1	20.5	3.7		1.69	0.86	93.09	2.1	high	8	8.9		41	11.7
PS	49	F	26	1	21.2	4.3		2.07	1.04	74.74	1.8	high	9.6	5.6		24	10.9
PS	41	M	5	1	24.6	4.3	183	1.56	0.57	77.58	2.1	high	7.8	5.3	0.08	14	10.5
PS	67	F	4	1	24.1	3.5	168	1.52	0.7	93.41	2.1	high	9.1	6.1	1.21	25	10.4
PS	65	F	5	1	19.4	4	118	1.92	0.92	103.4	1.1	high	8.5	5.1	0.02	25	12.3
PS	64	M	4	1	19.6		130	1.94	1.05	124	1.8	high	10.6	5.1	0.1	17	11.8
PS	58	M	17	1	19.4		135	1.32	1.15	103.3	1.3	high	7.5	5.2	1.53	27	9.1
PS	63	F	15	1	16.2		164	1.12	0.96	110.6	1.8	high		5.8	0.03	24	10
PS	64	F	12	1	17		188	1.48	1.21	97.66	1.5	high	10.7	4.1	0.12	27	10.7
PS	59	M	11	1	22.2		171	1.53	1.14	116.7	1.8	high	8.3	5.9	1.09	37	12.4
PS	37	M	5	1	20.7		184	1.18	0.99	74.54	1.8	high	10.4	4.8	0.08	22	11.6
PS	55	M	11	1	21.7		130	1.39	1.84	115	1.8	high	8.2	7.4	0.05	30	11.5
PS	47	M	10	1	15.3		135	1.42	0.92	116.1	1.5	high	10.1	5.6	0.05	27	12.5
PS	56	M	10	1	19.5		161	1.25	1.26	114.7	1.5	high	9.8	3.2	0.05	26	11.6
PS	73	M	9	1	20.2		125	1.65	1.2	80.96	2.1	high	9.5	4.5	0.1	29	10.5
PS	70	F	9	1	17.8		156	1.27	1.08	75.72	1.8	high	9.9	5.4	0.06	26	12.3

PS	50	M	9	1	24.1		145	0.94	0.99	120.3	2.1	high		2	0.3	23	11
PS	70	M	9	1	22.4		124	1.5	1.04	86.87	1.3	high		3.7	2.21	23	8.5
PS	69	F	8	1	23.5		116	2.02	1.28	117.6	1.5	high	9.1	5		28	11.8
PS	81	F	5	1	21.5		154	1.05	0.74	70.8	1.8	high	10.3	8.5	0.23	22	10.7
PS	52	M	5	1	18.1		95	1.22	1.27	106.9	1.8	high	8.9	5.6	0.05	53	12.9
PS	54	M	5	1	20.2		114	1.62	1.04	75.35	2.1	high	8.2	5.2	0.07	25	10.4
PS	83	F	4	1	18.1		131	1.65	1.24	92.26	1.5	high	8.9	5.8		24	9.4
PS	56	M	4	1	23	3.9	101	1.85	0.64	56.71	1.5	high	8.8	5.9	0.22	37	10.9
PS	54	M	4	1	27		150	1.48	0.79	92.12	1.5	high	8.5	3.6	0.12	27	9.5
PS	73	M	4	1	25.8		148	1.82	1.1	107.6	1.3	high	9.8	5	0.01	32	9.4
PS	80	F	3	1	28.2	4	91	1.35	1.03	97.06	1.5	high	8.2	4.4			
PS	60	F	3	1	22.2		205	1.25	1.05	103.9	1.5	high	8.8	8	0.05	21	11.1
PS	27	M	3	1	18		172	1.33	0.83	51.63	1.6	high	8.5	6.7	0.1	27	11.7
PS	63	M	3	1	25		85	1.24	1.36	101.2	1.8	high	8.5	5.1	1	31	11.4
PS	75	M	2	1	22		127	1.43	1.01	60.54	1.8	high	9.6	4.4	0.05	23	9.8
PS	83	M	2	1	15.7		155	1.2	0.67	88.04	1.5	high	8.8	6.2	0.05	31	9.3
PS	66	F	2	1	23.3		155	1.34	0.73	64.63	2.1	high	10.9	5.3	0.05	24	12.3
PS	61	M	5	1	20.9	4.1	153	1.05	0.94	95.14	2.1	high	11.6	7.3	0.16	24	10
PS	65	M	3	1	24.4	4.6	116	1.91	1.05	101.3	1.4	high	10.1	2.9	0.12	33	9.7
PS	86	F	5	1	27.8	4	165	1.25	0.68	82.06	2.1	high		5.6	0.19		10.8
PS	63	F	16	1	19.4	3.8	213	0.91	1.09	122.3	1.6	high	7.9	6	0.28	32	12.1
PS	74	M	9	1	18.4	4.1	92	1.05	0.78	95.35	1.6	high	9	5.4			11.3
PS	83	M	9	1	17.7	3.5	144	1.52	1.09	135.5	1.5	high		4.9	0.05		10.5
PS	85	M	9	1	16.9	4.2		1.64	1.05	123.7	1.6	high	8.8	4.4			11
PS	81	M	7	1	18.6	4.2	149	1.48	0.78	89.88	2.1	high	9.6	5.5	1.08	32	10
PS	65	F	7	1	27.5	3.7	161	1.85	0.91	114.2	1.3	high	9.9	5.2	0.02	34	9.6
PS	57	F	6	1	29.7	4.1	222	1.6	0.73	116.5	2.1	high	9.4	5.5	1		8.5
PS	68	M	6	1	22.6	4.1	143	1.7	1.05	112.6	1.6	high	8.9	4	0		10.7
PS	68	F	6	1	17.4	3.9	207	2.06	1.07	110.9	1.8	high	8.7	4.5	0.08	27	11.2
PS	65	F	6	1	16.6	3.8	134	0.92	0.97	119.5	1.5	high	8.6	2.8		29	9
PS	75	M	5	1	21.1	4.1	157	1.66	0.89	91.99	1.3	high	9.3	4.3	1.51	29	9.6
PS	49	F	4	1	24.1	4.1	162	1.55	0.69	75.42	1.3	high	9.6	4	1.07	31	12.6
PS	62	M	4	1	21	4.5	106	1.59	1.21	125.4	1.6	high	10.1	4.7	0		8.6
PS	85	M	4	1	14.6	4	201	1.39	1.2	78.68	1.5	high	8.7	4.3	3.56	19	10.7
PS	68	F	4	1	22.1	3.6	127	1.27	1.09	102.8	1.3	high	8.6	4.7	0		8.8
PS	67	F	4	1	19.7	4	234	1	1.03	107.9	1.8	high	8.7	6.2	0.04	27	10.2
PS	69	M	4	1	20.6	3.8	125	1.4	0.9	99.46	1.5	high	10.2	5	0.15	25	10.9
PS	60	M	3	1	23.5	4.4	116	1.22	0.98	113.2	1.8	high	8.9	5.1	0.7	18	10.7
PS	74	M	3	1	24.5	3.6	135	1.43	1.05	139.5	1.6	high	9	5	0		10.2
PS	58	M	3	1	23.8	2.6	166	1.26	1.2	126.3	1.5	high	9	5.2	0.05	26	10.4
PS	81	F	3	1	18.3	3.7		1.68	1.21	123	2.1	high	8.7	6.1		19	9.9
PS	76	F	3	1	21.4	3.9	130	1.09	0.96	89.56	2.1	high		5.6	0.38	26	10.2
PS	52	F	2	1	15.6	4.3	233	1.62	1.04	114.3	2.1	high	9.1	6.3	0		9.9
PS	77	F	2	1	19.7	4.1	121	2.01	1.08	115.3	2.1	high	9.2	6	0.09	35	10.6
PS	58	M	2	1	25.8	4.4	159	1.82	0.9	108.6	1.3	high	8.8	4.8	0.3	30	11.4
PS	67	M	2	1	28.9	3.6	129	1.29	0.84	101.4	1.6	high	8.8	4.7	0		10.9
PS	59	M	2	1	27.2	4	191	1.37	0.92	119	1.8	high	9.2	5.4	0.11	24	12.9
PS	68	M	5	1	19.2	3.3	179	1.36	0.63	95.41	1.6	high	7.5	2	0.2	28	8.9
PS	67	F	11	1	23.5	3.8	137	1.52	0.71	122.8	2.1	high	9.4	7.7	0.18	25	9.2
PS	70	F	9	1	20.3		218	1.99	0.78	92.05	1.5	high	12.3	6	0.13	31	10
PS	71	M	6	1	20.1	4		1.32	0.79	129.7	1.6	high	8.3	6	0.11	24	14.6
PS	58	F	12	1	20.4	4.1	112	1.51	0.96	103.6	1.6	high	10	4.2	0	25	10
PS	48	M	6	1	25.4	4.1	215	1.03	0.89	138.9	1.3	high	8.4	5	0.2	36	11.6
PS	71	F	4	1	23.1	3.6	163	1.15	0.69	103.2	1.3	high	7.8	4.4	1.9	36	10.2
PS	59	F	5	1	18.6	4	130	1.2	0.91	98.09	1.6	high	8.9	4.2	0.04	26	11.6
PS	71	M	7	1	21.8	4.1	205	1.08	1	116.2	1.6	high	8.1	3.7	0		10.6
PS	56	M	25	1	20.5	3.5	107	1.98	0.94	121.1	1.4	high	10.1	4.2	0.1	24	12.5
PS	65	M	18	1	20.5	3.3	240	1.48	0.66	73.55	1.6	high	8.8	6	0.39	21	10.8
PS	38	M	11	1	21.5	4.1	109	1.4	1.12	113.5	1.6	high	9	6.1	0.05	27	11

PS	67	M	4	1	23.2	3.8	197	1.73	0.95	113.6	0.8	high	9.2	3.9	0.3	24	10.3
PS	61	F	3	1	21.1	4.1		1.47	1.11	110.6	1.5	high	9.3	7.2	0.05	24	9.9
PS	59	M	2	1	24.8	4.2	178	0.86	0.73	64.28	1.5	high	9.2	5.3	0	29	8.7
PS	61	F	2	1	19.8	3.5	117	1.42	1.28	146.5	1.8	high	8.5	6.1	0.4	22	10.4
PS	87	F	5	1	16.4	3.7	169	1.65	0.94	116.8	1.6	high	8.5	6.3	0.24	28	9.5
PS	57	M	34	1	19.7	3.8	149	1.09	0.86	107.9	1.8	high	8.9	7.8	0.2	33	11.8
PS	69	F	32	1	20.3	3.5	168	1.53	0.74	114.3	1.6	high	8.9	5.2	0.05	29	11.7
PS	52	M	28	1	22.2	4.2	181	1.39	0.91	122.3	1.8	high		4.3	0.05	27	9.5
PS	69	M	26	1	15.1	3.6	139	1.75	1.23	52.67	1.5	high	8.2	3.3		25	11.1
PS	61	M	17	1	18.2	3.8	184	1	1.13	120.9	1.8	high	8.4	3.1	0		10.5
PS	71	M	16	1	19.5	3.3	158	0.97	1.05	109.1	1.8	high	9.8	4.9	0.05	13	11.6
PS	44	F	14	1	16.4	4.2	146	1.67	0.73	90.97	1.8	high	9.2	7.3	0.06	26	10.1
PS	55	F	12	1	22.2	4	197	1.5	0.88	104.5	1.6	high	8.3	6.2	0.39	31	10.4
PS	56	M	12	1	19.1	4.2	137	1.64	1.24	105.5	2.1	high	9.4	4.1	0.03	35	10.1
PS	54	F	12	1	19.2	3.8		1.12	1.3	111.5	1	high		5.3	0.05		12.3
PS	68	F	11	1	15.3	4		1.04	1.06	119.7	1	high		5	0.14		10
PS	50	M	9	1	20.2	4.2	154	1.64	0.68	140.4	2.5	high	8.7	4.8			10
PS	54	F	8	1	26.5	4	148	1.52	0.99	127	1.1	high	8.5	5.3	0.2	16	9.9
PS	57	M	8	1	20.7	3.9		1.26	1.29	109.9	1.5	high	9	5.9	2.33	27	9.6
PS	76	F	7	1	18.6	4.1	159	1.63	0.89	115.4	1.5	high	9.6	6.2		25	
PS	38	F	5	1	27.1	3.7	179	1.5	0.96	108.2	1.5	high	9.1	4.2	0.1	35	7.6
PS	73	M	5	1	26.5	3.8	153	1.21	0.79	102.1	1.8	high	9.5	6.9	0.15	27	11.5
PS	62	M	5	1	21.2	4.4	213	1.18	1.22	122.6	2.1	high		5.7	0.11	23	12.7
PS	33	F	4	1	18.4	4.2	140	1.27	1.23	83.77	1.6	high	9.4	4.1	2.5	27	9.3
PS	58	M	4	1	21.4	3.4	195	1.69	0.91	115.8	1.8	high	9.6	7.5	1.69	27	10.8
PS	74	M	4	1	20.6	3.4	184	1.36	0.86	100.9	1.3	high	10.2	5.4	0.54	28	8.1
PS	73	M	3	1	23.7	3.8	234	1.2	0.94	136.5	1.8	high	8.9	8.1	0.1	30	11.3
PS	66	F	2	1	22	3.8	135	0.93	0.91	63.49	1.8	high	8.7	6.3	0.57	32	9.4
PS	64	F	2	1	26.5	3.6	159	1.2	0.92	101.5	2.1	high		5.6	0.13	28	11.3
PS	35	M	2	1	19.8	4	177	1.71	0.89	77.33	1.5	high	10.8	5.2	0.09	35	8.8
PS	73	F	3	1	21.3	3.8	114	1.29	1.03	68.98	1.5	high	10.2	4.2	0.01	24	10.5
PS	80	F	10	1	19	4.2	152	1.49	0.93	118.2	1.8	high	10	4.7	0.01	37	9.6
PS	77	F	4	1	16.5	3.3		1.48	1.07	27.96	1.6	high	9.2	2.5	1.7		10.3
PS	46	M	9	1	24.8	3.5		1.67	1	132.9	2.1	high	8.7	7.8		26	10.7
PS	73	F	32	1	19.1	3.7	103	1.48	0.96	105.7	1.8	high	9.8	4.8		23	11.2
PS	68	F	8	1	17.8	3.5	111	2.14	0.8	92.52	1.8	high	10.1	4.5	0.01	36	10.1
PS	61	M	3	1	22.8	4	132	1.56	0.81	102.4	2.1	high	10	4.6		28	13.4
PS	67	F	21	1	20	3.4	177	2.13	0.92	87.77	1.6	high		5.3	0.05	27	9.8
PS	64	M	8	1	24.7	3.6	179	1.79	1.15	115.2	2.1	high	8.5	5.3	0.08		9.5
PS	56	M	28	1	18.6	3.8	160	0.88	0.59	75.21	1.5	high	8.5	4.5	0.09	22	11
PS	33	M	13	1	22.4	4.2	84	1.48	0.91	120.1	1.3	high	7.7	4.3	0.55	38	9.1
PS	47	F	10	1	27.2	4.1	133	1.71	1.09	101.4	1.6	high	10.3	5.9	0.24	17	9.7
PS	37	F	5	1	31.8	4.2	145	1.15	0.91	111.3	1.8	high	9.5	7.7	0.09	30	11
PS	73	M	3	1	18.8	4.2	172	1.21	1.25	104.5	1.8	high	8.3	7.1	0.35	27	11.4
PS	53	M	22	1	20.3	4	162	0.61	1.26	118.2	2.1	high		7.6	0.01	18	10.9
PS	46	M	22	1	18.9	4.1	170	1.53	0.73	83.85	1.3	high	8.4	5.6	0.02	21	9.4
PS	58	M	15	1	18.1	4.3		2	0.64	113.4	1.3	high	9.1	4		20	8.9
PS	66	M	6	1	20.5	4.2	210	1.13	0.83	130.8	1.3	high	8.6	5.1	0.38	26	10.3
PS	59	F	4	1	19.6	4.1	106	1.6	1.25	139.7	1.8	high	7.6	3.7	0.29	31	10.4
PS	68	M	2	1	22	3.9	148	2.24	0.72	118.7	2.1	high	9.8	5.4		25	10.6
PS	57	F	19	1	14.8	4.2	102	1.39	1.03	97.9	2.1	high	9.8	5.6	0.03	44	9.9
PS	63	M	16	1	13.8	4.1	139	1.06	0.93	118.3	2.1	high	9.2	4.9	0.1	29	9.9
PS	49	F	15	1	17.3	3.7	171	1.38	0.83	107.5	1.3	high	8.8	7.1	0.3	27	10.4
PS	44	F	10	1	16.8	4.3		1.22	0.88	109.7	1.6	high	8.2	5.7		36	9.1
PS	67	M	4	1	23	4	115	2.22	0.96	86.59	1.6	high	9.5	4.7		31	10.8
PS	60	F	15	1	19.2	3.7	164	1.32	0.67	119.5	2.1	high	8.3	5.7	0.64	30	9.1
PS	59	F	14	1	24.9	3.9	132	2.15	1.13	110.9	2.1	high	9.5	6.4		20	12.1
PS	71	M	12	1	22.3	3.5	134	1.39	0.96	148.2	2.1	high	10.1	9	0.21	34	11.7
PS	55	M	11	1	23.4	4.4	150	1.84	0.96	120.4	1.5	high	7.7	3.5	0.09	31	7.5

PS	55	F	5	1	27.1	3.8	131	1.4	0.76	145	1.5	high	7.9	4.6	0.44	26	11.7
PS	54	M	2	1	24	4.1	152	1.4	1.03	121	1.8	high	8.6	6.3	0.4	24	9.9
PS	70	M	14	1	22	3.3	156	1.33	0.73	111.7	1.6	high	8.9	7.2	0.3	33	10.4
PS	74	M	13	1	25.2	4.1	131	1.33	0.85	110.3	2	high	7.9	3.9	0.12	29	8.6
PS	72	F	6	1	16.5	4.4		1.35	0.82	54.74	1.5	high		4.2	1.42	35	10.2
PS	68	M	3	1	24.2	3.4	123	1.25	0.75	90.47	1.8	high		5.3	0.1	26	10.4
PS	54	F	3	1	29.5	4.1	111	1.14	0.86	103.9	1.1	high	8.7	6.3	1.19	36	7.3
PS	65	M	9	1	24.3	3.4	148	1.37	0.84	86.59	1.3	high	9.9	7.4	0.31	26	10.1
PS	46	M	19	1	17.4	4	151	1.3	1.12	114	2.1	high	9	4.9	0.07	33	11.1
PS	44	M	8	1	17.9	4.1	122	1.52	0.73	108.1	1.8	high	8.1	2.3	2.39	18	8.6
PS	53	M	5	1	24.9	4.6	217	1.53	0.75	116.5	2.1	high	8.3	5.7		28	12
PS	60	M	3	1	19.5	3.6	213	1	0.69	107.9	1.1	high	8.9	7.6	0.9	20	11.5
PS	66	M	3	1	20	4		1.75	1.2	124.8	1.6	high	9.9	6.3	0.1	27	10.9
PS	67	F	14	1	22.9	3.6	172	1.42	0.76	96.35	1.8	high	10.6	3.3	0.1	25	9.1
PS	56	M	3	1	19.7	4.5	139	1.34	1.14	90.22	2.1	high	8.5	4.1	0.3	30	11.6
PS	74	M	5	1	22.2	3.8	97	1.32	0.79	143.3	2.1	high	10.3	6.6	0.16	40	10.6
PS	57	F	5	1	23.3	4.2	121	1.29	0.97	106	1.5	high	9.9	6.4	0.06	29	11.3
PS	70	F	18	1	18.3	3.3	154	1.28	0.76	103.4	1.8	high	9.2	4.8	0.23	25	8.9
PS	60	F	10	1	21.1	3.7	174	1.31	1.33	141.8	1.3	high	10.1	4.4	2.88	34	9.2
PS	56	M	6	1	23.4	4.2	149	1.95	0.66	88.04	2.1	high	8.6	5.1		29	10.7
PS	59	M	5	1	28	4	135	1.14	0.85	129.4	2.1	high	9.1	6.4	1.41	24	9.5
PS	60	M	15	1	22.5	3.9	149	1.16	0.76	127.2	1.8	high	8.2	7.1	0.05	23	10.3
PS	56	M	3	1	27.3	4.2	103	1.9	1.15	97.87	2.1	high		4.2			13.4
PS	46	M	5	1	22.5	4.3	119	1.69	1.43	87.73	1.8	high	9.5	3.9	0.18	32	10
PS	75	M	5	1	24.9	4	140	1.12	0.95	145.4	1.3	high	7.5	6.6	0.4		13.8
PS	71	M	3	1	23.8	3.8	155	1.43	0.85	81.12	1.3	high	7.7	4.2	0.05	28	8.1
PS	55	M	33	1	20.5	3.5	189	1.26	0.78	76.11	1	high	9.9	5.7		43	10.8
PS	54	M	17	1	21.6	4	117	1.73	1.12	127.4	1.6	high		4.6	0.05	26	9.8
PS	76	F	2	1	18.8	3.6	202	1.36	0.84	90.16	1.8	high	9.3	7.3	0.07	37	11.2
PS	75	F	13	1	19.3	4	211	1.61	0.94	116.8	1.5	high	8.5	4.8			9.7
PS	62	F	3	1	28.7	3.6	175	2.1	0.73	92.31	2.1	high		7.2	0.03		10.1
PS	63	M	4	1	21.3	4	83	1.1	0.81	86.01	2.1	high	11.1	5.6	0.27	26	11.7
PS	68	F	2	1	17		155	1.8	1	117.7	1.3	high	8.7	3.4	0	42	11
PS	46	M	19	1	20.7		163	1.86	0.96	106.2	1.4	high	8.9	5.4	0		10.2
PS	55	F	3	1	25.9		92	1.34	0.97	83.89	2.1	high	8.7	4.6	0.27	27	10.6
PS	45	F	18	1	17.7		255	1.46	1.03	113	1.5	high	9.1	7.5	0.03	28	12.1
PS	47	M	4	1	22.5		124	1.71	0.78	96.83	2.1	high	8.4	5.1	0.09	22	10.4
PS	68	M	19	1	20.2		142	1.19	0.87	105.8	1.5	high	8.7	6.7	7.79	32	9.6
PS	66	M	15	1	23		144	1.33	1.1	122.8	2.1	high	8.2	4.3	0.05	27	10.1
PS	58	M	26	1	16.4		148	1.78	0.96	125.5	1.9	high		4.8	0.22	29	10.1
PS	65	M	13	1	19.1		142	1.57	0.92	123.9	1.5	high		2.5	0.1	19	9.9
PS	56	M	5	1	21	3.5	182	1.76	0.93	76.76	1.5	high	11.1	6.6			10.9
PS	62	M	23	1	18.7	4.1	139	1.25	0.94	92.46	1.8	high	9.7	5.2		25	10.5
PS	73	F	4	1	18.6	3.7	138	1.36	1.19	132.3	1.9	high		4.8	0.54	34	11.6
PS	57	F	28	1	21.3	3.5	176	0.99	1.03	107.4	1	high	8.3	6.7	0.05	18	11.3
PS	51	F	36	1	18.6	3.4	205	1.61	0.99	80.83	2.1	high	7.7	3.4	0.02	14	9.2
PS	51	F	2	1	22.5	3.9	173	1.46	0.97	87.01	1.5	high	9.1	4.8	0		10.7
PS	55	F	6	1	26.1	3.6	84	1.87	0.65	111.5	1.6	high		7		30	7.9
PS	57	M	8	1	21.8	3.9	147	1.45	0.87	102.6	1.3	high	9.4	8.1	0.3	30	11.1
PS	68	M	6	1	17.1	3.9	131	1.4	1.13	82.68	1.5	high	8.2	6.1	0.9	28	9
PS	54	F	22	1	19.8	3.6	144	1.43	1.02	116.5	1.6	high	9.6	5.6	0.03		11.3
PS	54	F	20	1	19.2	4.1	154	1.28	1.11	95.8	1.8	high	9.1	4.9		26	11.7
PS	58	F	17	1	18.4	3.9	128	1.77	1.34	91.12	1.6	high		6.5	0.3	36	12
PS	59	F	9	1	22.5	3.3	138	1.34	1.34	109.9	1.3	high	9.1	5.3	0.36	30	8.3
PS	59	F	14	1	20.7	3.9	143	1.16	1.09	114.2	1.5	high	7.7	6.4	0.88	26	10.8
PS	57	M	5	1	22	3.5		1.62	0.8	106.5	1.8	high	9.2	6.1	0.02	24	10.3
PS	71	F	5	1	15.5	3.1	123	1.81	1.39	77.54	1.8	high	9.7	4.9	0.01		10
PS	41	M	3	1	25.5	3.4	179	1.17	0.61	82.43	1.3	high	9.8	6.2	0.2	29	11.8
PS	66	F	11	1	25.9	3.6	125	1.12	0.84	97.36	1.1	high	8.4	2.5	1.03	36	7.8

PS	53	F	8	1	18.3	3.7		1.57	0.97	80.6	2.1	high		6.7		23	10
PS	54	F	25	1	25.9	3.7	188	2.13	0.88	115.2	1.3	high		4.3	0.07		9.9
PS	76	M	14	1	21.9	4.3		1.75	0.81	69.79	1.5	high	8.6	4		35	11.5
PS	57	F	10	1	21.9	3.9	165	1.76	1.03	107.3	2.1	high	10.9	4.6	0.07	24	11.1
PS	77	M	26	1	26.8	3.9	224	1.49	0.99	94.18	1.8	high	10	4.8	0.1	26	10.7
PS	57	F	25	1	17.1	3.9		1.26	1.11	113	1.8	high	8	4.3	0.41		10
PS	71	M	2	1	20.1	3.7	167	1.44	1.02	111.7	1.3	high	9.4	5	0.05	33	10.4
PS	61	F	7	1	19.7	3.9	146	1.42	0.89	92.36	1.8	high	9.9	5.5	0.05	24	10.1
PS	25	F	4	1	22	4.4	183	1.45	0.83	74.01	2.1	high	10.3	3.9	0.07	32	10.7
PS	52	M	5	1	34.1	3.9	80	1.53	0.78	113.4	1.9	high		3.6		25	9.3
PS	53	M	2	1	19.3		161	1.74	0.83	89.54	1.6	high		5.6	0.02	30	10.1
PS	47	F	3	1	17.6	4.1		1.47	0.9	103.5	1.5	high	9.6	5.3	0.33	34	10.4
PS	65	M	3	1	23	3.5	187	1.71	0.72	137.8	1.5	high	10.2	5.4	1.2	28	9.4
PS	55	M	3	1	24.5	3.8	169	1.18	0.55	73.36	2.1	high	8.6	4.6	0.1		11.4
PS	55	F	16	1	23.5	3.6	170	1.22	0.83	88.56	1.8	high		4.1	0.03	22	11.9
PS	65	F	9	1	16.8	3.7	137	1.67	1	91.22	2.1	high	8.2	4.2	0.33	32	10.2
PS	78	F	6	1	21.8	4	150	1.5	0.91	101.7	1.4	high	9.8	2.5	0.13	27	9.7
PS	48	F	16	1	24.6	3.5	118	1.8	0.72	109.1	0.6	high	7.9	2.8	0.88	39	9.9
PS	80	F	2	1	22.7	3.5	128	1.64	0.93	99.02	1.8	high		6.1	0.05	24	9.7
PS	49	M	30	1	19.9	4	149	1.47	0.83	95.55	2.1	high	8.7	3.6	0.06	22	9.8
PS	79	F	3	1	26	3.8	146	1.43	0.5	60.38	2.2	high	10.7	7	0.21	26	10.5
PS	80	M	2	1	26.2	3.1	130	1.35	0.46	36.75	2.5	high	9.4	6.5	0.4	48	8.5
PS	40	F	8	1	16.7	3.9	145	1.04	0.98	113	1.8	high	9.1	5.4	0.4	34	11.5
PS	67	M	4	1	25.8	3.8	139	1.44	0.73	92.27	2.1	high	7.6	4.7	0.05	19	10.7
PS	57	M	4	1	27.2	4.1	177	1.7	1.16	117.5	1	high		5.9		29	11.5
PS	70	M	3	1	18.1	3.8	136	1.84	0.94	119.5	1.5	high	10.1	4.6	0.15	37	10.4
PS	67	M	16	1	20.3	3.2	177	1.53	0.79	94.9	1.8	high		3.8	0.01		12.6
PS	53	M	16	1	19.6	3.8	166	1.54	0.77	115.8	1.3	high	9.4	3.8	0.3	31	11.7
PS	37	F	14	1	17	4.1	183	1.41	1.13	106.9	1.6	high	8.1	5.7	0.17	28	10.4
PS	81	M	9	1	19.1	4	97	1.31	1.05	99.52	0.6	high		6.3			11
PS	60	F	8	1	23.6	3.5	165	1.67	1.23	111.6	1.8	high		3.8	0.26		10.3
PS	65	M	8	1	23.2	3.7	143	1.28	0.38	76.85	2.1	high	8.5	4.9		21	13.1
PS	81	F	6	1	18.2	3.3	208	1.54	0.91	73.55	1.8	high		7	0.05	23	10.8
PS	78	F	5	1	20.5	3.5	176	1.38	1.12	108.3	1.8	high		5.2	0.04		11
PS	79	F	4	1	21.8	3.9	121	1.44	0.4	102.5	1.5	high	9.3	4.8	0.05	25	9.4
PS	66	F	3	1	23.4	3.6	149	1.7	0.78	89.62		high	10.8	6.2	0.01		
PS	65	M	10	1	18.4	3.9	121	1.45	0.75	92.47	1.5	high		4	0.55	26	8.5
PS	57	F	6	1	19.8	3.7		1.49	0.89	106.8	1.5	high	9.2	3.4	0.1	35	10.6
PS	54	F	6	1	25.3	3.7	178	1.49	0.71	105.5	1.8	high		5.6	0.18		9.9
PS	66	M	3	1	20.1	3.3	131	1.81	0.98	94.67	1.8	high		12.5	0.26	21	9.4
PS	59	M	3	1	23.3	3.7	116	1.11	0.64	112.8	2.5	high		6.6	0.18	24	10.9
PS	70	M	3	1	22.8	3.6	160	1.53	0.91	133.2	1.5	high	8.7	4.7	0.42	27	10.4
PS	57	F	3	1	20.7	3.8	117	0.96	0.71	98.97	2.1	high	10.7	7.1	0.06	26	11.4
PS	57	M	2	1	26.2	3.5	110	0.89	0.9	91.68	1.8	high	8.6	4	0.2	24	11.3
PS	61	F	2	1	21	3.6	133	1.43	0.83	97.6	1.8	high		4.9	0.9	27	10.3
PS	37	F	4	1	26.9	3.1	107	1.2	1.31	94.75	2.1	high	11.2	6.2	3		9.6
PS	56	M	22	1	18.1	4.2	105	1.4	0.93	111.5	2.2	high	8.9	4.9		51	10
PS	53	F	32	1	18.9	3.5		1.45	1.67	71.54	1.4	high	8.4	6.1		25	11.8
PS	66	F	6	1	18.8		150	1.67	0.92	91.8	1.5	high	9.8	4.6	1.01	30	10.2
PS	70	M	4	1	21.1		118	1.94	0.79	90.29	2.1	high	8.4	4.5	0.02	22	9.6
PS	62	F	2	1	15.8		225	0.78	0.96	119.8	1.6	high	8.3	4.9	0.2		9.8
PS	55	M	2	1	28.2		210	1.89	0.81	110.5	1.3	high	10.2	3.7	1.95	26	10.1
PS	29	M	2	1	16.3		149	1.43	0.98	73.48	1.3	high	9.3	4.4			9.9
PS	54	M	4	1	21.8	4.1	144	1.44	0.83	81.42	1.3	high	8.7	4.9	0.01	14	10.1
PS	79	M	8	1	20.5	3.5	132	0.83	0.9	136.1	2.1	high	8.8	5.6	0.05	12	10.8
PS	71	F	19	1	20	3.6	114	1.31	0.86	122.1	1.6	high	8.5	3.6	0.13	29	9.3
PS	69	M	13	1	23		249	1.93	0.78	104.6	1	high	10.2	6.7	0.11	26	10.2
PS	54	F	9	1	24.7	3.7	137	0.97	1.04	105.9	1.8	high		4.3			9
PS	71	M	8	1	20.2	3.6	133	1.02	0.97	116.4	2.1	high	9.6	3.4	0		10.1

PS	62	M	7	1	21.7	3.8		1.26	0.8	92.62	1.3	high	10.2	6.4	0.05	25	12.2
PS	61	M	6	1	18.4	3.3	94	1.25	0.81	75.31	1.5	high	9.9	4.4	0.88	33	11.6
PS	79	F	6	1	17.8	3.6	133	1.52	0.85	84.78	1.8	high		7	0.04		10.3
PS	69	F	6	1	23.4	3.6	165	1.06	0.83	115.7	2.1	high	7.9	7.6		23	10.2
PS	59	M	5	1	20.6	4.4	119	1.35	0.77	135.9	1.4	high	8.5	5	2.21	42	10.4
PS	59	F	5	1	17.3	4.2	145	0.87	1	79.4	1.8	high		5	0.55	36	10.3
PS	51	M	5	1	22.5	3.9	172		1.29	142.5	1.9	high	9.8	4.5	0.51		10.3
PS	26	M	5	1	20.3	4.3	125	1.64	1.06	99.54	1.8	high	7.2	5.3	0.42		10.2
PS	79	F	5	1	19.9	3.6	164	1.91	0.77	80.35	1.8	high		6.5			11.7
PS	70	M	4	1	28.4	3.5	173	1.9	0.95	109		high	9	3.9	0.09	33	11.5
PS	55	M	4	1	22.8	3.8	116	1.53	1	103.1	1.5	high	10.1	5.1	0.44	25	9.6
PS	38	F	4	1	25.1	3.8	156	1.16	0.81	95.37	1.8	high	8.5	4.8	0.09	23	8.1
PS	65	M	4	1	24.7	3.9		1.57	0.83	103.5	1.6	high	11.7	5.7	0.01	26	11.3
PS	70	F	4	1	17.7	3.8	155	1.7	0.83	68.91	1.3	high	9	4.4	0.1	25	10.2
PS	56	F	3	1	18.9	4	227	2.12	1.15	103.9	1.6	high	9	4.4	0.06	27	10.2
PS	74	F	3	1	25.4	3.7	118	1.22	0.86	114.3	1.3	high	9.8	5.8	2.17	26	9.3
PS	67	M	2	1	24.1	3.8	188	0.98	0.85	108.1	1.6	high	9.3	4.4	0.07	19	9.1
PS	71	F	2	1	26.1	3.9	98	1.35	0.88	92.13	1.8	high	9	5.1	3.06	26	11.1
PS	50	M	2	1	23.2	3.8	121	1.62	0.87	85.15	1.8	high	9.2	4	0	23	10.9
PS	63	M	2	1	23.2	3.5	135	1.3	0.84	99.94	2.1	high	10.7	4.6		12	10.6
PS	76	M	2	1	22.5	4	142	1.01	0.83	66.85	1.6	high	8.4	3.4		19	8.6
PS	62	M	2	1	23.5	3.4	170	1.34	0.7	102	1.5	high	9.1	3.6	0.2	30	9.8
PS	57	M	16	1	22.5	3.7	192	1.15	0.84	82.56	1.8	high	9.1	6	1.4	34	11.6
PS	51	M	2	1	22.6	3.7	133	1.75	0.91	76.31	1.5	high	9.2	5.5	0.05	39	8.7
PS	70	F	15	1	16.2	3.8	155	1.38	0.89	93.54	1.6	high		6.9			9.3
PS	49	M	6	1	22.6	4.1	149	1.59	0.8	78.19	1.3	high	9.8	5.1	0.22	33	10.3
PS	57	M	8	1	22.4	4.2	154	1.4	0.71	131.6	1.5	high	9.2	3.3	0.04	23	10
PS	65	F	5	1	18.3	4.1	211	1.75	1.16	97.21	1.6	high	9.7	6.5	0.1	24	11.5
PS	25	M	2	1	17	3.9	124	1.31	0.91	69.47	1.6	high	9	4.4	0.02	22	10.2
PS	42	M	15	1	23.5	4.4		1.33	0.8	110.9	1.6	high	10.2	6.7		29	11.3
PS	76	F	11	1	17.5	3.5		1.24	0.81	106	1.8	high	8	8.3	0.52	25	9.9
PS	70	M	9	1	22.4	3.7	124	1.63	0.63	105.8	1.5	high	8.6	5.8	0.37	42	10.6
PS	54	M	5	1	23.6	4	141	1.29	0.99	105.6	1.6	high	8.5	4.6	0.29	26	9.3
PS	75	M	4	1	21.4	4.3		0.88	0.87	97.54	2.1	high	8.2	7.4	0.15	28	10.3
PS	73	M	15	1	20.5	3.5	168	1.38	0.82	106.3	1.5	high	9.6	6.8	0.09	35	9.8
PS	75	M	6	1	24.8	3.8		1.17	0.96	116.9	1.6	high	10.1	4.6		38	10.3
PS	47	M	21	1	18.7	4.4		1.03	0.56	89.38	1.6	high	9.5	5		17	11.1
PS	56	F	13	1	19.5	3.9		1.17	1.02	124.9	1.6	high	9.1	4		33	10.7
PS	67	F	8	1	19.2	4		1.77	0.58	137.1	1.6	high	8.5	4.3	0.6		10.2
PS	61	F	11	1	16.9	4		1.77	0.8	93.85	1.9	high	9.8	4.6	0.2	34	10.2
PS	79	M	10	1	22.8	4.1		1.53	0.83	141.6	1.6	high	8	3.9	0		10.9
PS	64	F	10	1	18.6	3.9		1.89	1.17	113.6	1.6	high	7.8	3.6	2.1	34	9.6
PS	58	F	6	1	20.3	3.7		1.43	1.11	87.16	1.9	high	9.7	3.3	0.8		9.1
PS	50	M	6	1	19.5	3.9	182	2.34	0.75	127.6	1.6	high	9.6	6.3	0.7	25	11.5
PS	45	M	3	1	19.6	4.3	89	1.33	0.87	69.98	2.1	high	8.3	5.4	0	34	10.2
PS	76	F	11	1	16.5	3.8	192	1.27	0.72	70.83	1.8	high	8.6	5.6		19	9.3
PS	44	M	3	1	26.7	4.2	102	1.43	0.73	101.8	1.1	high	7.8	3.3		53	10.2
PS	60	F	11	1	24.5	4.1	181	1.58	1.04	139.4	1.5	high	8.2	3.7			8.4
PS	79	M	11	1	18.1	3.6	189	1.36	0.63	81.81	1.5	high	8.4	3.1	1.82	40	10.1
PS	37	M	10	1	22.2	4.6	171	2.13	0.88	118.7	1.6	high	9.8	5.9	0.1	28	9.8
PS	81	M	6	1	20.8	3.7	182	1.92	0.86	130.3	1.6	high	9.4	4.5	0.1	26	10.7
PS	68	M	5	1	21.1	4.1	131	1.9	0.98	99.42	1.8	high	9	4.6	0.34	28	9.7
PS	44	F	5	1	31.6	3.2	130	1.51	0.78	87.3	1.3	high	7.3	3.4	0.3	31	11.7
PS	64	F	34	1	18.1	3.6	144	1.23	1.15	68.93	1.9	high	9.5	5.4	0.2	27	11.1
PS	79	M	7	1	19.9	3.8	144	1.7	0.81	110.1	1.9	high	10	5.5	1.4	28	10.3
PS	65	F	5	1	22.9	3.8	168	0.98	1.07	110.2	0.9	high	8.7	6.1	0.3	21	10
PS	79	F	6	1	23.8	4.2	153	1.78	0.76	112.1	1.6	high	10	4.9	0.1	26	11.1
PS	60	M	14	1	24.8	3.5	110	0.93	1.01	142.8	1.4	high	8.8	5.1	0.1		12.6
PS	57	M	4	1	18.8	3.9	120	2.15	0.95	103.5	1.5	high	6.2	5.5	0.46	32	10.2

PS	79	F	6	1	24.1	3.4		1.66	0.97	108	1.5	high	10.7	6.4	0.27		10.4
PS	76	M	14	1	22.6	3.9	140	1.33	0.88	136.8	1.8	high	8.7	5.6	8.1		10.7
PS	54	M	4	1	21.4	3.8	140	1.84	0.81	106.1	1.9	high	9.3	6.6	0.2	33	10.4
PS	50	M	4	1	27.7	3.8	146	1.56	0.99	102.4	2.1	high	10	6.2	0.1		12.1
PS	69	M	2	1	22.9	3.8	181	1.43	0.9	125.3	1.5	high	10.2	5		26	11.6
PS	42	F	7	1	21.8	3.9	157	1.37	0.53	63.58	1.8	high	8.4	4.2	0.29	26	6.2
PS	65	M	15	1	22.7	3.6	161	1.18	0.72	106.8	1.3	high	9.2	5.5	0.1	15	10.9
PS	69	M	2	1	18.4	3.3	178	2.39	0.72	81.12	1.6	high	9.3	4.2		26	10.4
PS	39	M	12	1	21.7		165	1.19	0.89	101.2	1.6	high	8.5	4.7	0.36	31	9.8
PS	69	M	9	1	18	3.7		1.44	0.87	87.26	1.3	high	9.3	3.5		30	10.7
PS	48	M	4	1	29.9	4	141	1.37	0.96	83.73	1.1	high	9.4	5	0.3	24	10.5
PS	78	M	6	1	21.8	3.5	128	1.75	1.14	143.2	1.1	high	10.5	5.5	8.1	33	8.5
PS	86	M	3	1	17.6	3.9	244	1.73	1.26	95.98	1.9	high	8.7	4.3	0.1	25	11.2
PS	58	M	13	1	20.4	3.8	158	1.19	1.02	80.54	2.1	high	9	5.4	0.05	22	10.8
PS	49	M	13	1	21	3.6	148	1.51	0.73	110.3	1.6	high	8.1	5.3		27	9.7
PS	75	M	30	1	20.1	3.5	215	1.41	0.92	98.23	1.5	high	9.5	5.1	1.17		13.1
PS	40	M	20	1	20.1	3.8	218	2.03	0.92	114	1.3	high	8.9	4.7	0.02	26	10.9
PS	65	F	27	1	22.8	3.7	116	1.27	1.06	109.7	2.1	high	7.9	5.9	0.11	30	12.1
PS	61	M	5	1	21	3.2	138	1.73	0.7	71.25	1.5	high	9.5	5.4	0.12	37	9.8
PS	63	M	4	1	25.7	4	119	1.7	0.63	45.36	1.6	high	9.7	3.9	0.2	24	10.2
PS	60	M	5	1	19.8	3.3	147	1.05	0.66	63.99	1.5	high	9.1	5.9	0.4	25	9.9
PS	73	M	4	1	18.8	3	158	1.48	0.71	111.9	1.5	high	10	5	0.22	20	10
PS	56	M	20	1	21.2	4.2	169	1.62	0.84	122	2.1	high	8.9	5.4	4.99	29	11.1
PS	58	M	5	1	22.5	3.5	162	1.65	0.93	71.48	1.5	high	8.2	5.9		27	11.4
PS	72	M	3	1	21.8	3.6	158	1.74	0.88	96.47	1.6	high		6	0.16	34	9.3
PS	54	M	25	1	18.2	4.1	171	1.76	0.92	116.4	1.2	high	10.3	5.2	0.1	23	11
PS	70	M	7	1	20.4	3.7		1.79	1.15	102.7	2.1	high	8.9	5	0.51		12.4
PS	68	F	14	1	21.3	3.5	147	1.52	1.03	111.2	1.9	high	9.9	6.2	0.1	27	
PS	57	M	4	1	29.8	4	97	1.16	0.93	116.6	1.5	high	7.7	4.5	0.1	28	9.8
PS	64	M	5	1	27	3.8	226	1.37	1.15	96.7	1.9	high	9.4	6.1	0.1	26	12
PS	45	F	2	1	18.2	3.8	88	0.98	1.07	78.01	2.1	high	9.4	5		28	11.3
PS	49	M	28	1	20.7	4.7		1.48	0.93	97.35	2.1	high	8.4	7.2		27	10.4
PS	57	M	18	1	20.3	4.1	136	1.77	0.91	118.7	1.2	high	10.2	6.5	0.1	26	
PS	71	M	8	1	18.6	4.1	162	1.86	0.97	92.66	1	high		4.4	0.54	25	8.9
PS	56	F	22	1	17.8	3.7	137	1.3	0.81	94.37	1.6	high	9.5	3.8	0.4	29	11.7
PS	69	M	12	1	21.6	4.2	143	1.31	0.85	126	1.5	high		4.7	0.05	29	10.2
PS	69	F	16	1	19.9	4.2	184	1.9	0.84	116	1.5	high	9.9	5.8	0.18	25	9.5
PS	62	M	8	1	19.1	3.9	145	1.37	0.73	98.4	1.3	high	9.1	6.2	0	25	11.2
PS	79	F	4	1	18.1	3.4	98	1.88	0.91	104.5	1.8	high		4.7	0.11	27	10.5
PS	58	M	5	1	20.1	3.9	190	1.36	0.91	104.2	1.5	high	8.9	5.7	0.09	23	10.3
PS	62	M	4	1	21.3	3.6	182	1.57	0.98	138.3	1.5	high	10.1	5		26	12.1
PS	66	M	30	1	25.2	4	105	1.35	0.72	92.3	1.8	high	8.9	5.3	1.6	25	11.3
PS	45	M	4	1	23.5	3.8	105	1.27	0.56	124	2.1	high	8.1	8.1	0.68	32	12.3
PS	79	F	5	1	18.8	3.8		1.47	0.94	85.67	1.5	high	8.1	7.4	0.1	29	11.5
PS	69	F	4	1	18.8	4	236	1.62	0.89	83.47	1.6	high	9	6.6	0.07	23	11.7
PS	82	M	5	1	19.8	3.4	89	1.24	0.75	76.2	1.8	high	7.9	5.7	0.22	33	11.9
PS	56	F	13	1	25.3	3.8	189	1.12	0.94	112.7	1.3	high	8.9	5	0.83	21	10.4
PS	61	M	30	1	25.2	3.9	178	1.44	1.01	80.51	1.4	high	9.4	4.8	0.3		9.6
PS	61	F	14	1	23.2	4.1	152	1.15	1.12	115.1	1.9	high	10.4	5.2	0.1	28	10.6
PS	74	F	9	1	23.7	3.5		1.1	0.82	126.8	2.1	high	8.9	2.2	0.1	22	10
PS	51	F	23	1	21.1	4.6	238	1.64	1.27	123.4	1.1	high	8.6	5.5	4.8	28	9.7
PS	41	M	16	1	22.3	4.3	119	1.83	1.11	128.5	1.5	high	8.7	5.3	0.04	30	11
PS	71	M	8	1	20.1	3.8	159	1.19	0.69	141.4	1.5	high		7.3		21	9.6
PS	59	F	28	1	22.6	3.8	185	1.34	0.82	109.3	1.6	high	9.2	8.4	0.1	26	10.3
PS	68	F	29	1	19.4	3.6	208	1.41	1.06	80.9	1.5	high	9.9	5.4	0.41	27	9.4
PS	56	F	31	1	16.5	3.8		1.38	0.86	97.43	1.5	high	9.6	8.4	0.25	32	10.8
PS	74	M	4	1	21.5	3.4		1.44	0.83	87.7	1.8	high	9.2	8.6		25	11.6
PS	69	F	27	1	13.5	3.7	95	1.36	0.91	127.9	1.8	high	8.9	5.8	0.1		10.3
PS	61	F	11	1	28.3	3.7	130	1.3	1.34	151.3	1.6	high	10.4	5	0.1	28	11.2

PS	83	M	2	1	17.6	3	139	1.92	0.55	63.46		high		5.3	0.01	29	12.1
PS	63	F	25	1	27.4	3.4	263	1.45	1.09	114.8	1.6	high	8.9	3.3	0.2	44	11.1
PS	64	M	24	1	19	3.6	181	1.69	0.89	83.91	1.4	high	8.9	4.9	0.06	33	11.2
PS	43	M	7	1	29.5	3.8	152	1.33	0.77	107.8	1.5	high	8.1	5.7	0.2	28	11.1
PS	54	M	5	1	25.6	3.9	152	1.55	0.93	140.9	1.8	high	10.1	5	0.07	22	10.8
PS	57	M	4	1	27.7	3.4	145	1.49	0.96	128.1	1.8	high	8.1	7.5	0.82	32	11.4
PS	47	M	3	1	21.6	3.8		1.62	0.57	61.45	2.1	high	8.8	4.1		23	
PS	43	M	3	1	19.1	4.1	207	1.95	1.04	95.19	1	high	9.1	4.6	0.48	33	10.7
PS	64	M	10	1	19.1	4.4	116	1.35	1.08	113.9	1.5	high	8.4	3.2	1.4	43	13.1
PS	54	M	17	1	23.2	4	206	1.74	0.99	122.9	2.1	high		6.2	0.04		12
PS	65	F	15	1	19	3.5	211	2.09	1.1	130.9	1.4	high	9.3	2.5	0.93	25	11.4
PS	60	M	6	1	23	4.5	151	1.14	0.82	90.06	1.4	high	9.2	3.4	0.08	27	10.5
PS	50	M	4	1	22.8	4.5		1.06	0.86	134.5	1.8	high	9.9	7	0.53	19	10.3
PS	45	F	7	1	19.5	4	102	1.48	0.88	87.48	2.2	high	10.4	5.5	0.02		9.7
PS	82	M	4	1	25.6	3.9	115	1.89	0.88	114.7	1	high	8.1	3	0.07	35	9
PS	67	M	11	1	16.7	3.5		1.72	0.87	108	1.8	high	9.7	8.3			9.5
PS	67	F	7	1	24.8	3.5	153	1.26	0.84	122.3	1.6	high	9.3	5	0.1	22	10.9
PS	73	F	6	1	19.8	3.7		1.37	0.9	99.96	2.1	high	8.2	4.9	0.04	24	9.2
PS	76	M	3	1	22.9	3.8	125	1.44	0.91	145.3	1.8	high	8.1	5.1	0.49	29	9.2
PS	62	F	2	1	22.7	4		1.31	1.3	120.1	1.5	high	9.8	5.5	0.09	25	10.4
PS	64	M	3	1	23.5	3.7		1.71	0.86	131.9	1.5	high	12.8	7.3	0.11	22	12.6
PS	42	M	18	1	21.3	3.8	147	1.39	0.78	121.7	2.1	high		7.3	0.04		10.2
PS	52	F	15	1	24.3	3.7		1.28	1.08	118.2	2.1	high	10	5.7		30	10.8
PS	65	F	3	1	18.4	3.5	146	2	0.68	79.53	1.6	high	9.1	4		31	10.7
PS	61	M	10	1	20.8	3.8		1.26	1.01	129.6	2.1	high	9.3	3.7	0.11	24	10.3
PS	66	F	20	1	24	4		1.18	1.28	108.1	2.1	high	9.7	7.9	0.26	17	8.9
PS	59	F	11	1	22.4	3.9	182	1.36	0.9	112.7	1.8	high		4.5	0.24	32	10.7
PS	41	M	11	1	26.5	4.3	169	1.85	0.74	124.3	1.6	high	9.2	5.7	0.1	20	11.9
PS	61	M	17	1	24	3.7	134	1.34	1.02	92.52	1.6	high	9.6	6.3			10.8
PS	67	F	5	1	22.7	3.7	104	2.69	0.98	58.69	2.1	high	9.6	6		29	10
PS	49	F	12	1	16.1	4.1	162	1.68	0.98	132.8	1.8	high	8.9	7.3	0.08	23	11.6
PS	50	M	2	1	26.2	3.9	196	1.71	1.09	91.47	1.8	high	9.5	5		25	10.8
PS	59	M	4	1	22	4.2	179	1.02	1.1	138.9	1.3	high	8.9	4.1			9.1
PS	61	M	19	1	24.6	4.2	173	1.31	0.86	113.4	1.3	high	9.6	5.8	0.66	28	10.4
PS	48	M	14	1	20.8	3.9	122	1.17	1.02	114.3	1.9	high	10.3	6.6	0.09	30	10.4
PS	69	M	9	1	21.8	4	166	1.21	0.79	92.94	1.9	high		6.3	0.12	19	10.6
PS	60	F	5	1	29.1	3.7	154	1.41	0.8	89.76	2.1	high	9.2	4.5	0.28	33	11.4
PS	67	F	5	1	20.6	3.9	156	1.34	0.87	112.1	2.1	high		7.1	0.14	23	11.1
PS	62	F	12	1	19.4	3.6		1.43	0.78	86.09	1.8	high	8.1	4.2	0	23	10.9
PS	82	M	7	1	19.9	3.6	206	1.29	0.76	109.3	1.6	high	8.7	5.6	0	24	9.8
PS	61	M	7	1	20.5	4	144	1.32	0.79	104	1.6	high	8.1	4.5	0.05		10.1
PS	77	M	7	1	26.1	3.9		1.35	0.72	113.7	2.1	high	9	6.5	0.05	26	10.5
PS	65	F	6	1	18.9	3.5	100	1.1	0.84	77.29	1.8	high	7.9	5.6	1.17		9.6
PS	66	M	6	1	23.3	3.4	158	1.66	0.81	110.5	1.8	high	8.8	3.7	0.13	21	9.4
PS	41	M	14	1	22	4	92	1.33	1.11	110.1	1.5	high	8.7	5.3	0.12	29	10.7
PS	47	M	5	1	29.1	3.8	81	1.08	0.89	88.02	1.8	high	8.4	2.3		29	10.6
PS	58	M	13	1	23.6	3.8	66	1.32	1.29	129.8	1.8	high	8.2	5.4	1.51	23	11
PS	81	F	6	1	18.6	3.6	123	1.26	0.95	125.9	2.1	high	8.3	3.5	0.18	8	10.5
PS	65	F	12	1	22.5	3.7	174	1.13	1.15	89.25	1.5	high	9	5.5	0.1	27	8.7
PS	61	M	6	1	15.1	3.8	159	1.93	0.77	89.57	1.3	high	9.1	4.6	0.03	17	10.1
PS	55	F	8	1	23.5	3.4		1.76	0.94	115.8	1.1	high	9.3	5.1		25	10.7
PS	69	F	12	1	17.4	3.8	96	1.74	0.97	123.5	1.6	high	9.6	5.1	0.06	2	10.8
PS	57	M	5	1	29.6	3.6		1.48	0.77	108.7	2.1	high		5.1			
PS	53	M	9	1	25.9	4.2	126	1.37	1.14	132	1.5	high	9.7	4.3	0.3	18	7.5
PS	52	F	13	1	21.2	4.2	163	2.23	1.01	95.52	1.3	high	9.5	5	0.01	26	8.8
PS	37	F	5	1	18.3	4.1	157	1.68	1.02	104.8	1.8	high		4.7	0.31	37	11.8
PS	52	M	6	1	22.9	4.1	107	1.84	0.96	100.8	1.8	high	9.7	5	1.53	31	10.3
PS	66	M	6	1	27.1	4	146	1.09	0.76	98.85	2.1	high	9.7	4.8	0.17	28	12.2
PS	71	M	8	1	18.2	3.6	164	1.82	0.93	122	1.8	high	8.8	4.7	0.25	36	10.6

PS	58	F	5	1	19.7	3.8	166	1.94	0.82	125.1	2.1	high		8.9	1.6	25	11
PS	65	M	6	1	26.1	4	146	1.37	0.91	109.8	2.1	high	10.6	7.1	0.08	26	9.7
PS	75	M	7	1	22.9	3.6	211	1.75	0.7	128.9	1.3	high	10	4.1	0.07	19	9.8
PS	84	M	2	1	17.9	3.2	140	0.85	0.94	112.4	0.6	high	7.5	4.1	0.61	24	11.5
PS	68	F	5	1	16	4	130	1.23	0.99	85.03	2.1	high		3.9	0.08	22	9.3
PS	43	F	2	1	21.3	4	109	1.16	0.93	105.4	1.5	high		4.7	1.54	31	10
PS	59	M	6	1	22.7	4.2		1.61	0.93	82.24	1.3	high	9.4	6	1.1	24	9.9
PS	71	M	19	1	22.7	3.9	101	1.25	0.98	94.2	1.3	high	9.2	5.8	0.1	34	11.9
PS	66	M	17	1	19.8	3.4	193	1.22	0.91	98.82	1.8	high		4.3	0.1	27	10.2
PS	64	F	5	1	20.6	4	193	2.13	0.96	117.1	1.8	high	11.1	2.4	0.44	25	11.8
PS	59	M	2	1	19.4	4.4	157	0.63	1.25	109.7	1.6	high	9.8	5	0.22	27	11.7
PS	65	F	3	1	14.8	3.8	159	1.4	1.14	106.2	1.8	high		5.7	0.1		13.1
PS	66	M	2	1	20.5	4	196	1.65	0.88	108	1.5	high	8.6	5.1	0.1	28	10.4
PS	58	F	9	1	17.6	3.9	110	1.97	1.18	122.7	1.5	high	9.1	4.6	0.02		10.8
PS	59	M	12	1	20.4	3.7	125	1.73	0.97	152.6	2.1	high	9.2	3.6	0.32		10.6
PS	60	M	6	1	25.9	3.9	107	1.19	1.07	124.7	2.1	high	9.2	4.2		28	8.6
PS	59	F	2	1	17.3	4		1.17	0.97	95.36	1.6	high	8.8	5.9		25	10.3
PS	56	M	4	1	23.2	3.8	159	1.69	0.82	100.9	2.1	high		5.8	0.11	37	9.7
PS	75	M	8	1	20.8	3.7		1.06	1.43	106.9	2.1	high	8.7	5.9	0.08	26	12.3
PS	54	M	12	1	19.1	4	140	1.49	0.91	94.71	1.8	high	8.9	8.3		20	8.7
PS	61	F	17	1	19		91	1.33	1	112.3	1.3	high	8.8	5.3	0.1	25	10.2
PS	69	M	13	1	17.9	3.3	196	1.45	0.98	122.4	1.3	high	8.1	3.5	6.4	10	9.9
PS	56	F	7	1	33.3	2.9		1.21	0.9	122.4	1.5	high	9.6	5.2	0.06	35	11.5
PS	62	F	6	1	19.3	3.7	149	1.4	1.07	110.7	1.3	high	8.7	13.8	0.6	27	10.7
PS	66	M	5	1	32.5	3.1	159	1.33	0.79	136	2.2	high	10.7	5.8	0.99		11.1
PS	80	M	4	1	19.8	3	130	1.42	1.2	118.1	1.8	high	8.4	5.1	0.03		9.9
PS	57	M	3	1	21.7	3.6		1.77	0.83	116.2	1.5	high	8.4	4.1	0.1	20	11.9
PS	54	F	3	1	18	3.3	147	1.27	0.96	83.04	1.8	high	8.4	6.6	1.35	28	12.2
PS	68	F	8	1	20	4.3	173	1.03	0.82	119.9	2.1	high		6.3	0.5	24	11.7
PS	49	M	18	1	20.6	4.3		1.19	1.21	142.4	1.3	high	8.5	7.8		30	10.9
PS	76	F	18	1	18	4.4	115	1.17	1.05	102.4	1.8	high	8.2	5.1		32	11
PS	59	F	4	1	19.7	4.8	204	1.26	0.92	82.67	1.8	high	8.1	4.2		22	9.8
PS	54	M	2	1	23.2	4.5		1.29	0.94	100.9	1.8	high	9.2	7.6	4.1	20	10.6
PS	74	F	2	1	22.4	4.1	111	1.7	0.93	103.1	1.8	high	8.4	4.6		27	11.3
PS	49	F	14	1	19.3	4.6	144	1.16	0.96	97.54	1.8	high		5.4	0.9	20	10.7
PS	41	M	4	1	17.3	4.6	134	1.42	0.92	82.76	1.8	high		5.3	0.18	24	9.8
PS	55	F	24	1	23.4	4.4	160	1.6	1.27	108.7	1.5	high	9.4	4.4	0.57	35	13.9
PS	64	F	13	1	17.7	4.2	189	1.19	0.67	106.8	2.1	high	9.1	6.4		30	13.1
PS	38	F	9	1	19.8	4.5	149	1.46	1.09	86.73	2.1	high	7.4	5.3	0.15	24	12
PS	64	F	13	1	17.3	4.8	165	1.5	0.89	113.8	1.5	high	10	5.3	0.1	25	10.5
PS	43	M	26	1	18.8	4.3	190	1.64	1.13	85.53	1.1	high	8.8	5.3		48	10.8
PS	53	F	21	1	18.5	4.5	109	1.59	0.67	83.48	2.1	high	7.8	4.5		32	9.8
PS	58	F	20	1	18.1	4.5	147	1.26	0.97	88.27	1.5	high	10.2	4.6		37	9.6
PS	60	F	16	1	19.7	4.1	168	1.2	0.98	141.8	1.1	high	8.2	4.3		19	10.6
PS	62	F	11	1	19.8	4.6	176	1.37	1.01	107.2	1.5	high	8.9	5.1		29	10.5
PS	70	M	5	1	20.6	5	83	1.56	1.23	114.1	1.8	high	9.5	5.2	0.18	35	9.7
PS	50	M	2	1	21.6	4.3	230	1.21	1.04	103.1	1.8	high	10.1	6.2		22	11.8
PS	68	F	5	1	19.2	4.3	151	1.3	0.85	62.92	1.8	high	9.4	4.8	0.49	20	9.9
PS	52	M	20	1	18.5	5.1		1.27	0.97	87.75	2.1	high	9.4	5.5	0.1	19	10.3
PS	38	M	12	1	17.1	4.8	119	1.25	1.19	92.69	1.5	high	9.2	6.4		29	11.4
PS	75	M	4	1	20.6	4.5		1.35	0.72	94.32	2.1	high	9.2	4.8	0.66		11.6
PS	44	F	12	1	21.1	4.5	140	1.75	1.1	72.12	1.5	high	8	5			9.5
PS	52	M	18	1	22.2	4.7	173	1.71	1.07	116	1.5	high	8.9	3.9	0.05	31	13.1
PS	50	M	17	1	23.6	4.1	149	1.41	1.14	124.3	1.3	high	8.5	4.8		28	11.7
PS	81	F	2	1	23.2	3.7	156	1.2	0.66	48.82	1.5	high	10.2	5.2		19	11.3
PS	81	M	3	1	29.3	4	217	1.29	0.65	52.8	1.6	high		7	0.2	23	10.6
PS	60	M	3	1	22.9	3.9	156	1.18	1.03	118.5	1.6	high	8.7	4.3	1.59	16	10
PS	74	F	2	1	21.4	3.4	145	1.45	0.77	124	2.1	high		5.1	0.11		10.6
PS	78	F	2	1	16.5	3.7	133	1.4	0.96	95.63	1.9	high	9.2	6.6	0.02	28	11.4

PS	60	F	12	1	20.9	3.7	128	1.75	0.97	97.3	1.8	high	10	5.6		20	11.3
PS	58	F	22	1	20.6	3.5	161	1.81	0.9	82.17	1.8	high	9	3.4		24	10.6
PS	72	F	2	1	22.2	3.8	151	1.17	0.78	101.5	1.6	high	9.1	8.5	0.03	23	10.2
PS	55	M	5	1	21.4	3.8	208	1.25	0.82	65.08	2.1	high		6.6	0.34	30	13.5
PS	62	F	7	1	22.3	3.5	133	0.9	0.52	99.29	2.1	high		5.5	0.02	25	10.4
PS	74	F	5	1	19.9	3.4	155	1.22	0.59	72.63	2.1	high		5	0.06	27	11.4
PS	63	M	8	1	24.4	3.9		1.44	0.76	115.2	1.9	high	8.5	5.9	0.09	23	9.1
PS	72	M	5	1	23.7	3.9	109	1.28	0.74	130.4	1.3	high	7.6	5.9	0.03	34	11.4
PS	59	F	13	1	17.1	3.9	192	1.23	0.95	127.4	1.3	high	8.6	5.4	0.16	31	10.8
PS	81	F	10	1	28.1	3.5	166	1.14	0.82	52.19	2.1	high	9	4	0.03	25	11.5
PS	56	F	4	1	22	3.8	163	1.21	0.92	105	2.1	high		7	2.47		11.3
PS	59	M	3	1	27.1	4	223	2.11	0.68	78.95	1.6	high	8.5	5.6		30	10.6
PS	65	F	17	1	23.1	3.7	137	1.45	1.17	132.8	1.8	high	8.3	4		23	12.3
PS	61	M	5	1	21.8	3.8	219	1.28	0.88	114.8	1.8	high	9.5	6.6	0.13	16	11.6
PS	90	F	3	1	27.2	3.2	122	1.09	0.94	122.6	1.6	high	7.5	5.3	0.11	32	9.4
PS	60	M	3	1	23.5	3.7	129	1.27	0.88	69.26	2.1	high		4.7	0.16	24	9.2
PS	68	F	18	1	23	4	120	1.59	1.02	129.5	1.5	high		6.7	0.13	45	10.7
PS	57	M	12	1	21.3	4.3		1.55	1.06	106.8	1.5	high		3.3			8.3
PS	55	F	9	1	17	4.5		1.08	1.12	90.17	2.5	high	8.5	7.4	0.17	17	10.2
PS	49	M	8	1	24	4.5	120	1.39	1.17	138.5	1.6	high	8.7	5.8	0.08	31	8.6
PS	81	F	6	1	17.3	4	143	1.48	1.03	119.6	1.3	high	9.1	3.5	8.5	30	9.2
PS	60	F	10	1	19.7	4.1		1.22	1.17	126.3	1.8	high	8.6	3.5	0.35	16	9.6
PS	76	F	6	1	16.6	3.6		1.43	0.81	67.05	2.1	high	9.1	4.7		33	8.6
PS	79	F	3	1	21.9	4.1	184	2.14	0.89	52.05	2.1	high	7.5	6.5	0.24		10.3
PS	60	M	2	1	18.4	3.8	119	1.49	0.74	45.51	2.1	high	8.8	5	0.03	27	11.8
PS	62	F	4	1	18.8	3.7	165	1.54	0.93	98.32	1.8	high	9	5.6	0.06	26	10.2
PS	67	M	7	1	22.4	3.9	141	1.57	0.89	109.9	1.3	high	9.4	6.5	0.02	26	11.1
PS	70	M	7	1	20.5	3.7	169	1.75	1.1	123.9	1.8	high	8.3	8.1	0.59		11.8
PS	72	M	7	1	20.2	3.9	178	1.72	1	142	1.6	high	10.4	4.8	0.05	38	13.7
PS	65	M	7	1	18.9	4.1	190	1.83	0.96	115.1	1.3	high	9.2	5.3	0.03	24	9.8
PS	57	F	7	1	16.4	3.6		1.66	1.17	125.8	2.2	high	9.2	4.3	0.06	32	12.2
PS	63	M	6	1	21.1	3.9	147	1.42	1.07	95.09	1	high	9.9	4.3	0.02	27	10.5
PS	74	F	6	1	18	3.7	160	1.34	0.75	125.9	2.1	high	8.1	3			
PS	76	M	6	1	17.8	3.8	135	1.24	1.04	127.2	0.6	high	9.6	3.8	0.57	32	8.7
PS	58	F	5	1	16.3	4.1	132	1.37	0.96	93.51	1.6	high	9.8	5.1	0.23	28	10.7
PS	68	M	4	1	22	4	204	1.8	1.02	128.9	1.3	high	10.3	7.1	0.12	26	11.7
PS	44	M	3	1	26	4	115	1.34	1.05	95	1.8	high	9.5	4.5		47	12.6
PS	72	M	3	1	19.9	4.1	106	1.18	1.13	86.24	1.5	high	10.8	4.7	0.6	24	8.7
PS	63	M	3	1	22.5	3.5		1.86	0.93	80.01	2.2	high	8.8	6.4		29	10.7
PS	92	M	2	1	18.1	3.5	153	1.92	0.65	105.8	2.2	high	9.8	6.2	8.3	29	9.3
PS	32	M	2	1	30	4.4	168	1.53	1.08	113.4	1.8	high	9.9	6.6	0.1	26	9
PS	76	M	2	1	30.9	3.5	139	1.15	0.73	80.02	1.6	high	9.5	4.7	0.77	21	10.2
PS	63	M	4	1	25.3	4	104	1.22	0.94	134.8	2.1	high	8.5	5.2	1.67	19	11.5
PS	77	F	21	1	21.8	3.6	145	1.86	1.08	90.88	1.3	high	9.5	5.5	0.04	29	10.7
PS	84	M	6	1	18.2	4		1.63	0.9	127.1	1.5	high	9.4	5.3	0.22	26	10.4
PS	67	M	4	1	28.3	4		1.01	0.88	108.2	2.5	high	8.5	6.9	0.04	20	11.6
PS	78	M	8	1	18.5	4.4		1.37	1.44	152	1.8	high	11.7	9.2	0.05	22	8.7
PS	51	M	19	1	22	3.8	136	1.72	1	108.2	1.3	high	8.5	2.3	1.01	24	10.5
PS	37	M	9	1	27.4	4	132	1.5	0.98	128.9	1	high	9	2.9	0.12	17	10.5
PS	39	M	8	1	18	4.2	220	1.58	1.05	115	1.3	high	9.5	3.8	0.72	35	8.5
PS	52	M	7	1	22.5	3.2	153	1.88	1.17	128	2.1	high	9.6	4.9	0.01	24	10.8
PS	65	M	4	1	25.2	3.8	193	1.5	0.79	84.14	2.1	high	8.5	4.8	0.15	29	10.7
PS	67	M	3	1	20.6	3.6	101	1.3	0.82	103.1	1.6	high	8.9	5.1	0.01	25	9.1
PS	59	M	2	1	21.2	3.6	211	1.55	0.83	121.4	2.1	high	8.6	4.3	0.46	35	10.4
PS	64	M	35	1	20.1	3.8	162	1.37	1.12	48.86	1.6	high	8.7	5.1	0.04	33	11.4
PS	40	F	16	1	19.8	4		1.18	1.15	95.25	1.5	high	8.8	4.3	0.73	31	9.4
PS	52	M	14	1	18.3	4.5		1.56	0.81	104.7	1.3	high	9.1	4.5	0.07	20	10.6
PS	67	M	14	1	18.2	3.9	160	1.6	1.03	101.8	1.3	high	8.9	7.4	2.44	29	12.4
PS	68	F	13	1	19.9	6.2	169	1.74	0.96	104.1	1.6	high	9.6	5.2	0.06	26	8.7

PS	47	F	11	1	22	3.9	216	1.87	0.98	141.1	1.6	high	9.2	4.1	0.33	27	11
PS	43	F	10	1	21.7	4.1	105	1.56	1.21	136.5	2.1	high	10.1	4.9	0.3	30	10.4
PS	85	F	8	1	19.7	3.8	132	1.7	0.92	99.91	0.6	high	9	3.7	0.12	31	9.8
PS	63	F	8	1	19.7	4.2	176	1.65	0.75	90.67	1.6	high	8.7	7.4		29	11.2
PS	60	M	7	1	23.4	4.3	152	1.48	1.04	106.7	1.6	high	9.3	4.3	0.57	26	11.3
PS	64	M	3	1	20.1	4.2	137	1.86	0.67	84.88	1.1	high	8.9	6.2	0.2	34	9.2
PS	37	M	3	1	21.4	3.6	176	1.75	0.88	60.68	1.8	high	8.7	6.1	0.07		10.4
PS	65	F	3	1	19.9	4.2	88	1.23	0.96	93.21	1.5	high		2.6	10.29	26	8.4
PS	61	F	3	1	22.1	3.9	195	1.56	0.76	106.9	1.1	high	9.7	7.2	0.09		10.8
PS	64	F	3	1	28.4	4	131	1.01	0.86	70.02	1.5	high	8.7	3.2		32	9.5
PS	67	M	2	1	18.2	4.1	159	1.75	0.56	25.8	1.6	high	7.3	6.5	0.11	29	11.4
PS	61	M	2	1	21.5	3.9	152	1.67	0.72	76.1	1.5	high	9	4.2	0.01	38	11.6
PS	58	M	36	1	22.6	3.8	219	1.52	0.75	108.6	2.1	high		5.2	2.26	31	10.8
PS	67	F	16	1	16.4	4.3	187	1.57	0.83	131.3	2.1	high		6.8	0.23		10.2
PS	69	F	16	1	20	3.6	188	1.44	1.07	108	1.6	high	8.9	4.7	0.04	24	10.6
PS	57	F	15	1	18.2	3.8	155	1.75	0.81	104.8	1.6	high	8.5	9.8	1.35	28	10.3
PS	60	M	12	1	22.4	4.2	160	1.87	1.1	124.8	1.6	high	9.9	6.1	0.1	27	11
PS	52	F	11	1	16.6	4.4	156	1.53	0.98	93.13	1.8	high	9.3	4.5	0.1	38	10.1
PS	52	F	10	1	22.1	3.9	136	1.63	1.12	115.7	1.6	high	8.6	5.6	0.07	28	11.7
PS	64	F	8	1	17	4.1	113	1.05	1.05	127.4	2.1	high		5.1	0.08	27	13.1
PS	69	M	7	1	20.6	3.7	175	1.48	0.94	105.7	2.1	high	9.8	6.9	0.11	25	11.4
PS	60	F	6	1	24.1	3.9	125	1.63	0.89	107	1.3	high	9.2	5.4	0.03	30	10.9
PS	54	M	6	1	34.5	3.5	121	1.27	0.67	78.11	2.1	high	9.2	5	0.31	29	7.8
PS	59	M	6	1	17.8	3.4	170	1.5	1.36	88.18	2.1	high	7	8.1	0.2	31	10
PS	84	F	5	1	15.8	3.5	194	2.22	0.75	142.7	1	high	9.1	5.2	0.03	26	10.5
PS	81	M	5	1	20.5	3.7	151	1.75	0.88	136.9	1.3	high	10.1	5.8	0.07	25	11.1
PS	70	M	5	1	25.4	4	160	2.27	0.97	126.5	1.3	high	8	6	0.05	24	10.4
PS	65	F	4	1	16.4	3.8	184	1.57	1.13	118.2	1.3	high	9	5.4	0.11	27	11.5
PS	72	M	4	1	22.9	3.4	129	1.81	0.87	109.7	1.6	high	8.3	4.4	0.05	23	10.8
PS	56	M	4	1	29.4	4	169	1.26	0.94	85.49	2.1	high	9.2	5.2	0.11	25	10.4
PS	65	F	4	1	17.6	3.1	107	1.31	1.01	103.7	1.6	high	8.4	3.3	0.09	31	10.8
PS	61	M	3	1	22.3	3.9	174	1.91	0.96	99.16	2.1	high	8.2	4.8	0.48	37	9.7
PS	74	M	2	1	18.8	3.4	110	1.55	1.12	88.58	1.3	high	7.8	0.6	0.44	25	11.5
PS	66	F	2	1	19.8	4.3	137	1.45	1.03	80.05	2.1	high	8	6.1	0.07	35	10.5
PS	76	M	4	1	18.6	3.5	137	1.6	1.02	102	1.5	high	8.8	4.4	0.95	35	10.5
PS	65	M	8	1	18.9	4		1.08	0.88	117.1	1.5	high	9	3.8	0.09	29	8.5
PS	64	F	17	1	20.9	3.3	219	1.53	1.01	107.3	1.3	high	9.4	7	0	27	11.7
PS	68	M	5	1	22.4	3.6	237	1.39	0.92	123.8	1.6	high	9.1	2.8		29	9.8
PS	38	F	9	1	19	3.5	170	1.73	0.85	114	1.8	high	8.6	3.6	0	31	11
PS	76	F	9	1	20	4	97	1.42	0.97	129.9	0.6	high	9.7	3.9	0.04	27	11
PS	70	F	12	1	26.8	3.9	171	2.2	0.89	130.3	1.5	high	8.9	7.1	0.13	23	9.9
PS	56	F	15	1	18.5	3.8	125	1.36	0.96	125	1.8	high		8.8	0.58	24	14.4
PS	71	F	4	1	24	3.9	149	1.35	0.88	117.3	2.1	high	8.2	4.4	0.63	35	9.2
PS	51	F	17	1	20.2	5.5	163	1.77	1.24	125.7	1.6	high	10.2	4.1	0.07	24	10.5
PS	47	M	10	1	18.3	3.9	122	0.85	0.72	110.4	1.6	high		6.9		34	9
PS	86	M	2	1	21.8	3.9	165	1.56	0.87	94.44	1.6	high	9.2	5.5		25	10.1
PS	35	M	8	1	19.5	3.9	81	1.42	0.74	102	2.1	high	10.3	7.3	0	28	13.2
PS	68	M	13	1	24	3.7	84	1.4	0.91	120.6	1.4	high	9.4	4.9		30	10.5
PS	61	M	5	1	22.5	3.7	175	1.7	0.91	92.98	1.6	high	10	3.3		25	9.8
PS	43	M	4	1	17.1	4	143	1.47	1.13	130.3	1.5	high	10.1	5.8	0.2	33	11.5
PS	43	M	2	1	20.4	4.1	144	1.39	1.07	86.59	1.8	high	8.8	4		30	9.9
PS	59	F	30	1	18.2	3.9	172	1.37	1.14	107.4	1.6	high	9.7	5.1	0.07	31	10.1
PS	69	M	5	1	19.2	3.9	117	1.31	1.17	84.1	1.8	high	9.8	4.8		33	11
PS	72	M	8	1	21.3	3.8	118	1.67	1.02	149.2	1.3	high	8.5	4.6	0.52	25	10.7
PS	49	F	5	1	22.2	3.9	213	2.02	0.92	99.41	1.3	high	10.1	4.8	0.09	27	10.2
PS	60	F	8	1	21	3.9	252	1.85	1.15	104.4	1.5	high	9.8	5.7		36	10.8
PS	43	M	2	1	17.1	4	171	1.26	0.81	70.87	1.8	high	9.6	5.7		35	10.6
PS	65	F	13	1	30.3	4	138	1.61	1.17	131.6	1.5	high	8.8	3.7		32	9.5
PS	67	M	20	1	14.2	3.9	208	1.06	1.01	111.2	1.6	high	8.8	8.8	0.28	25	10.1

PS	58	F	2	1	22.3	4.2	172	1.77	0.88	124.6	1.6	high	9.8	4.3		31	7.8
PS	55	F	11	1	17.8	4	126	1.52	0.94	102.7	1.6	high	10.5	3.6	0.05	33	11.6
PS	36	M	4	1	23.3	4.5	144	1.47	1.14	120.6	1.5	high	11.3	6.6		27	12.2
PS	33	M	5	1	24.5	4.4	126	1.08	1.06	131.3	2.1	high	8.4	5.1		25	11.5
PS	54	M	4	1	22.3	4	202	1.32	0.97	126	1.8	high	9.7	4.5		30	10.4
PS	62	M	11	1	23.1	3.8	141	1.37	1.15	126.7	2.1	high	10.2	7.8		22	10.5
PS	54	M	2	1	25.8	4.2	67	1.32	0.98	126	1.6	high	9.7	5.3	0.29	27	10
PS	59	M	7	1	17.3	3.9	162	1.51	1.13	68.45	2.1	high	8.2	7.2		26	10.7
PS	52	M	8	1	21.3	4.4	167	1.7	1.35	140.4	1.5	high	8.3	5.7		34	11.4
PS	64	F	24	1	16.4	3.8		1.37	0.96	126.1	1	high	8.2	3		31	9.6
PS	68	M	8	1	23	3.7	159	1.13	1.04	106.3	1.8	high	9.1	5.1		21	9.7
PS	63	M	2	1	25.6	4	147	1.42	0.9	74.77	1.8	high	10	7.1		28	9.7
PS	64	F	22	1	23.8	3.7	178	1.5	0.97	114.6	2.1	high	9.5	3.7	0.2		10.7
PS	75	F	15	1	21.3	3.1	160	1.09	1.17	116.9	2.1	high	8.5	6.3	0.61	28	8.7
PS	44	M	7	1	17	3.6	123	1.31	0.89	100.7	1.5	high	8.5	3.7		29	9.6
PS	65	M	5	1	23.3	3.9	174	1.63	0.83	98.46	1.1	high	9.6	4.3	0.39	28	11
PS	66	M	3	1	20.2	3.7	140	1.96	0.86	83.76	2.1	high	9.6	4.2		27	10.5
PS	79	M	3	1	18.4	3.3	102	1.56	0.79	85.95	2.1	high	8.8	5.7	0.05	30	8.7
PS	62	M	2	1	27.8	4.3		1.29	0.77	81.01	2.5	high	10	7.1	0.01	23	11.4
PS	74	M	2	1	23.2	3.4	159	1.5	0.85	68.72	1.8	high	9.4	4.9		25	11.5
PS	67	M	4	1	21.4	4	124	1.66	1.01	98.82	1.5	high	8.6	5.4	0.04	26	10.6
PS	83	F	7	1	20	3.7	115	1.78	0.46	63.52	1.8	high	9.3	6.2	0.3	26	10.2
PS	67	F	5	1	18.6	3.7	106	1.46	1.24	83.19	2.1	high	8.4	4.3	0.63	33	11.2
PS	69	M	4	1	22.2	3.9		1.2	0.94	82.6	1.8	high	8.2	2.4	6.29	23	7.3
PS	62	M	3	1	30.1		205	1.56	0.94	66.61	1.6	high	8.8	6	0.2	23	11.5
PS	34	M	4	1	19.6	4.1	172	1.71	1.24	113.7	1.8	high	9.7	7.2	0.1	38	11
PS	77	M	3	1	21.8	3.7	217	1.51	0.85	62.93	1.8	high	9.2	5.2		18	10.9
PS	35	M	6	1	21.6	4.5	117	1.41	1.12	133	1.6	high		4.8	2.52	28	8.8
PS	60	M	3	1	23.7	3.2		1.62	1.06	90.64	1.8	high	7.9	4.6	0.93	29	10
PS	61	M	34	1	19.5	3.8	167	1.62	0.9	83.3	1.3	high	10.1	6.9	0.03	26	10.9
PS	53	M	30	1	19.7	4	95	1.33	1.07	103.6	1.8	high	8.8	5.5		29	12.7
PS	60	M	27	1	23.7	4.3	174	1.79	0.86	113	1.3	high	9	6	0.04	23	10.6
PS	60	F	27	1	21.9	4.2	109	1.31	1.1	133.1	1.6	high	8.9	4.1	2.15	21	9.4
PS	46	M	25	1	18.6	4.1	122	1.75	1.06	100.7	1.6	high	9.7	4	0.12	27	10.3
PS	55	F	21	1	18.4	4	120	1.04	0.88	90.56	1.9	high	8.2	5.6	0.08	25	10
PS	55	F	16	1	23.3	4	122	1.09	0.94	127.3	1.9	high	7.3	4.1	0.2	2	8.1
PS	60	M	13	1	21.8	3.7	159	1.35	0.85	120.7	1.6	high	8.8	4.5	0.63	36	10.7
PS	62	M	10	1	27.9	3.7	133	0.99	0.95	119.8	2.1	high	8	7.4		32	11.1
PS	74	M	9	1	25.1	3.8	117	1.44	0.81	107.8	1.6	high	8.9	5.8	0.03	32	11.2
PS	69	M	8	1	22	2.7		1.1	1.01	121.6	2.1	high	8.4	7.5	0.01	23	9.6
PS	66	F	7	1	19	3.6	153	1.5	1.03	117.5	1.8	high		4.9	0.04		7.7
PS	56	F	6	1	23.8	4.2	220	1.51	0.89	113.9	2.1	high	8.8	5.5	0.1	29	10.4
PS	76	F	5	1	18.7	3.8	157	1.41	0.92	97.84	2.1	high	8.8	5.1	0.09	20	10.8
PS	61	M	5	1	24.2	4.3	115	1.35	0.71	96.41	1.6	high	8	5.7	0.79	29	10
PS	53	M	4	1	24.6	4.2	101	1.26	0.79	75.46	1.5	high	9.2	5.5	0.1	30	12.5
PS	40	M	4	1	29.1	3.7	170	1.51	0.91	109	1.6	high	8.2	5.4	0.06	23	9.7
PS	75	F	4	1	21.7	3.6	103	1.08	0.7	119.6	1.6	high	9	4.1	0.07	18	10.2
PS	66	M	5	1	21.1	3.2	139	1.32	0.85	90.28	1.4	high	8.6	3.3		36	10.5
PS	73	M	4	1	20.8	4.1	120	1.52	0.82	99.33	2.1	high	10.8	6.2	0.12	28	10.5
PS	47	M	13	1	19.8	4.1	131	1.26	0.96	96.45	1.8	high	9.5	5.3		23	12.2
PS	58	M	11	1	21.8	3.9	269	1.74	1.12	119.4	1.5	high		2.2	0.03	29	9.8
PS	66	M	10	1	20.7	3.4	135	1.55	0.86	143.5	2	high	10.4	4.9	0.05	15	10.7
PS	57	M	9	1	21	4.2	105	1.6	0.67	96.48	1.4	high	8	6.8	0.12		10.2
PS	73	M	3	1	19.6	4.1	147	1.47	0.98	121.5	1.5	high	6.7	9	1.43	33	10.6
PS	80	M	3	1	24.9	3.7	136	1.51	0.91	124	1.6	high	9.7	4.7	2.04	23	10.1
PS	47	M	14	1	19.9	4.1	173	1.18	1.01	132	2.1	high	9.5	3.8		31	12.1
PS	51	F	27	1	17.1	3.6	120	1.47	0.99	112	1.8	high	10.1	4.7		22	10.2
PS	57	M	11	1	19.9	3.9	100	1.37	0.82	119.2	1.5	high	8.6	3.5		27	10.7
PS	79	M	6	1	23.7	3.8	163	1.21	0.78	124	2.1	high	10.6	7.7	0.45	24	10.7

PS	70	M	6	1	22.3	3.4	178	1.43	1.2	127.4	2.1	high	8.7	4.4	0.18	24	10.8
PS	63	M	10	1	18.2	3.8	142	1.17	0.94	137.9	2.1	high	10.4	6.3		27	10.9
PS	66	F	4	1	18.1	3.6	118	1.35	0.83	115.1	1.8	high	9.4	5.2	0.06	25	10
PS	31	M	16	1	20	4	147	1.64	0.66	94.49	2.1	high	9.8	7.3	0.1	38	10.5
PS	59	M	12	1	21.2	3.6	160	1.55	0.88	121.9	1.5	high	8.2	3.9		44	11
PS	49	F	2	1	16.6	3.5	209	1.94	0.67	75.11	1.3	high	8.8	5.2	0.05	26	10.7
PS	71	M	14	1	17.2	3.6	147	1.21	0.93	119.2	1.8	high	10.6	6.3		25	11
PS	61	M	5	1	18.6	3.9	186	1.81	0.81	94.94	1.3	high	9.5	4.9	0.22	26	10.3
PS	64	F	7	1	20.3	3.8	112	1.34	0.94	113.4	1.5	high	9.1	3.6	0.6	26	9.6
PS	64	M	13	1	19.5	4	85	0.98	0.86	145.6	1.8	high	9.9	3.5	0.24	26	9.6
PS	38	M	14	1	19.4	3.7	125	1.39	0.9	146.7	2	high	8.5	4.3			8.3
PS	77	M	7	1	19.9	3.9	141	1.09	1.16	118.7	1.6	high		3.9	0.05	19	11.9
PS	69	M	4	1	22.9	3.2	175	1.21	0.67	121.9	1.6	high	9.7	5.4		30	11
PS	70	M	4	1	21.2	3.8	161	1.41	1.07	94.73	1.6	high	8.7	8.3	2.26	27	9.6
PS	48	F	23	1	22.5	3.8	202	2.07	1.04	136.2	1.5	high	10.7	6.8	0.2	32	11.6
PS	54	F	7	1	23.1	4.1	185	1.12	0.82	80.21	1.5	high		6.4	0.02	15	9.5
PS	75	F	7	1	20.7	4	144	1.68	1.03	96.49	1.3	high		6	0.2	17	11.5
PS	56	M	3	1	23	4	180	1.05	1.09	132.5	1.8	high	10.8	6.9	4.41	26	11.5
PS	60	M	8	1	25.6	3.7	101	1.58	0.71	111.4	2.1	high	9.9	5.7	0.1		10.1
PS	55	F	7	1	22.9	4		1.42	1	127.9	2.1	high	8.6	6.5	0.01	22	11.5
PS	69	M	3	1	21.8	3.7	119	0.95	0.89	132.7	2.1	high	8.1	6.2	0	55	6.8
PS	60	M	5	1	22.2	3.8	99	1.24	0.84	127.3	2.1	high	8.9	4.8	0.1		10.8
PS	58	M	6	1	19.5	3.5	125	1.02	1.02	138.9	2.1	high	8.5	9.3	0.1		12.3
PS	74	F	11	1	21.5	3.5	204	1.2	0.82	116.2	2.1	high	9.6	6	0.05	15	10.4
PS	76	M	5	1	18.9	3.8	167	1.47	1.06	116.9	2.1	high	9.2	4.5	0.2	25	11.2
PS	55	F	2	1	17.8	3.7	141	1.3	0.9	96.95	1.3	high	7.1	4.5	1.8	34	7.3
PS	50	M	12	1	25.3	4.2	165	1.52	1.21	122.3	1.6	high	8.9	5.3	0.12	27	11.4
PS	62	F	9	1	17.4	3.6	184	2.27	0.93	66.36	2.1	high	8.4	5.9	0.2	26	10.8
PS	65	F	19	1	22.8	3.5	184	1.43	0.97	130.4	1.4	high	8	4.5			9.9
PS	38	M	3	1	26.5	4.1	154	1.31	0.91	87.72	1.5	high		5.3	1.3	39	12.1
PS	55	F	8	1	21.2	3.7	131	1.24	0.95	83.08	1.5	high	6.3	4.2	0.11	30	12.1
PS	36	F	12	1	16.9	3.9		0.96	0.82	99.29	2.5	high	11.2	4.8	1.54	25	9.1
PS	56	F	10	1	18.7	4.1		1.28	1.21	105.8	2.1	high	10.5	5.2	0.19	23	10.8
PS	72	F	27	1	21.3	4	192	1.76	0.8	90.45	1.5	high	9.4	6.6	0.1	43	11.6
PS	45	M	18	1	22.5	4	126	1.44	0.98	131.3	2.1	high	10.7	4.7	0.12	38	12.8
PS	70	M	3	1	24.8	3.6	155	1.15	1.06	82.34	2.1	high	9.2	8.5	1.35		10.3
PS	42	M	2	1	23.8	4	146	1.82	1.25	127.1	1.5	high	8.3	5.7	0.1		10
PS	62	F	4	1	21.4	3.9	118	1.23	1.09	94.78	1.3	high	7.5	5.9	0.05	30	9.7
PS	52	M	17	1	16.1	3.8	186	1.12	1.02	115.1	1.3	high	8.9	5.6	0.05	19	10.6
PS	74	M	2	1	21.9	4.1	210	1.58	1.3	110.2	1.3	high	9.6	3.8		23	10.8
PS	50	M	14	1	20.4	3.5	118	1.65	1.17	85.39	1.4	high	7.8	5.3		33	10.5
PS	81	F	7	1	19	4.1	173	2.04	0.85	98.26	1.1	high	9.4	2.8	0.2	27	10.4
PS	77	M	4	1	23.6	3.2	108	1.39	0.86	72.14	1.4	high	7.6	4.9	0.57	23	9.3
PS	78	M	2	1	24.4	4.1	110	1.37	0.86	86.78	2.1	high	9.6	4.2	0.17	27	9.1
PS	63	M	2	1	23.4	3.5	165	0.98	1.16	97.2	2.1	high	8.5	4.8	0.52	24	8.8
PS	60	M	6	1	19.3	3.7	205	2.36	0.92	140.5	2.1	high	9.5	4.6	0.1	28	12.7
PS	64	M	22	1	15.6	4.2	104	1.23	1.2	126	1.6	high	9.1	8.3		35	11.4
PS	80	F	7	1	27.2	3.6	206	1.31	0.95	145.8	1.3	high	9.5	6.6	0.31	27	9.6
PS	75	M	10	1	19.2	3.8	160	1.72	0.99	147.3	2.1	high	8	6.6	0.06	25	8.7
PS	52	M	6	1	16.8	4.1	101	1.3	0.53	49.55	1.8	high		5.5	0.59	27	10.5
PS	58	M	3	1	21.4	3.6	124	1.21	0.93	110.7	2.1	high	10.1	6.3	0.11	23	11.2
PS	64	M	10	1	20.4	3.8	158	1.65	1.08	124.4	1.3	high	8.8	6.4	1	24	10.6
PS	77	M	5	1	24	3.7	130	0.6	0.9	123.7	1.6	high	9.3	6.9	0.03	26	9.3
PS	60	M	10	1	24	3.6	151	1.83	0.67	146.1	1.6	high	9	5.2	0.05	27	10
PS	65	M	9	1	27.2	3.8	137	1.21	0.85	77.46	1.8	high	9	6.1		34	10.5
PS	53	F	4	1	29.5	4.1	104	1.63	0.83	104.9	1.5	high	9.4	5			10.3
PS	63	M	6	1	25.1	4.4	199	1.21	1.19	93.9	2.1	high	10.9	8.1	0.02	26	12.9
PS	65	M	6	1	22.2	4.3	127	1.4	0.64	81.88	2.1	high		3.8	0.18		10.3
PS	58	M	8	1	25.6	3.9	153	1.45	0.97	129.7	1.5	high	8.8	6.6	0.97	22	9.6

PS	59	M	7	1	22.8	3.8	166	1.44	0.93	93.03	1.5	high	9	4.1	0.1	22	12.2
PS	35	M	4	1	21.5	4.2	136	1.37	1.07	114.9	2.1	high	8.4	3.5		25	12.2
PS	52	M	7	1	19	3.8		1.03	0.67	56.44	2.1	high	9.7	6.8		23	10.9
PS	70	M	4	1	21.8	3.5	149	0.84	0.87	105.3	1.5	high	8.8	3.1	11.69	22	8.4
PS	74	F	16	1	19.3	3.6	145	2.17	1.12	129.2	1.1	high	9.3	3.3	0.05	29	10.5
PS	67	M	14	1	18.7	3.7	154	1.16	1.23	100.9	1.6	high	8.2	3.9	1.86	25	7.8
PS	72	M	13	1	20.1	3.3	158	1.97	0.85	140.7	1.8	high	9.4	6.6	0.02	25	10.6
PS	64	F	8	1	24.8	3.7	154	1.3	0.94	108.2	1.5	high	9.1	7.2	0.6	25	10.7
PS	70	M	8	1	22.8	3.8	167	1.53	0.75	99.48	1.5	high	8.3	5.9	0.24	37	8.4
PS	78	F	5	1	19.3	3.7	115	1.76	0.76	48.33	1.8	high	8.5	3	0.05	27	10.2
PS	57	M	4	1	21.2	4	102	1.5	1.03	86.03	2.1	high	10.2	6.3	0.08	26	10.5
PS	74	M	3	1	19.8	3.6	139	1.31	0.98	108.9	1.3	high	10.5	5.8	0.21	20	10.4
PS	55	M	2	1	25.7	3.9	134	2.25	1.19	108.2	1.6	high	9.5	6.9	0.05	26	10.9
PS	71	M	2	1	16.2	4	179	1.08	1.03	76.97	1.3	high		3.4	0.3	23	9.3
PS	72	M	2	1	22.5	3.5	135	1.1	0.9	77.88	2.1	high	9.8	5.4	0.41	30	11.9
PS	64	F	7	1	17.1	3.4	119	1.26	1.08	101.7	2.1	high	9.5	6.4	0.33	25	11.1
PS	59	F	5	1	19.1	4.4	110	1.18	1.04	85.27	1.5	high	9.3	6	0.3	37	10.1
PS	46	F	4	1	22.4	4.3	218	1.76	1.04	96.31	1.3	high	8.5	5.7	4.72	29	8.7
PS	65	M	2	1	16.8	3.9	120	1.57	0.89	98.57	1.9	high	8.8	4.8	0.4	59	10.4
PS	82	M	14	1	16.6	3.7	137	1.52	0.78	86.68	1	high	9.3	4.6	0.2	16	10.3
PS	68	M	3	1	22.7	3.7		1.51	1.06	134.1	1.5	high	9.3	5.5		28	9.4
PS	57	M	11	1	27.8	4.1	122	1.22	0.82	94.99	2.1	high	8.6	4.4	0.23	25	10.7
PS	66	M	4	1	22.1	4.2	143	1.23	1.26	130.6	2.1	high	8.3	7.6		31	11.1
PS	59	M	2	1	24.1	3.9	190	1.46	0.85	83.32	1.5	high	10.4	5.4	0.56	27	11.8
PS	79	F	3	1	18.4	4.2	219	1.77	0.94	97.41	1.6	high	8.5	7.4	0.1	28	10.8
PS	70	F	2	1	19.8	4	146	1.17	0.71	100	2.1	high	9.4	3.5	0.02	25	10.6
PS	61	M	3	1	20.9	4.2	103	1.56	1.1	83.23	1.9	high	10	4.6	0.4	18	10.3
PS	71	M	14	1	18	3.9	155	1.23	0.76	105.2	1.6	high	9.9	6.5	0.05	24	9.5
PS	59	F	10	1	16.6	3.9	188	1.78	0.98	94.43	1.9	high	9.6	5	0.4	32	11.5
PS	58	F	3	1	20.5	4.5	168	1.45	0.76	68.26	1.5	high	10	4.3		31	11.1
PS	62	M	9	1	20.2	4.1	101	1.4	0.86	86.31	1.5	high		5.1	0.53	39	11.8
PS	67	M	2	1	22.6	4.3	160	1.02	0.9	67.89	2.1	high	10.7	5.6	0.35	25	16
PS	61	M	2	1	21.9	4	170	1.3	1.07	96.55	2.1	high	8.3	4.4	0.07	27	11.8
PS	66	M	6	1	17.1	4	120	1.32	1.14	90.89	1.9	high	9.9	4.5	0.1	30	11
PS	37	M	3	1	27.4	4.3	188	1.84	1.06	107	1.5	high	8.7	9.8	0.02	30	10.3
PS	72	M	2	1	16.2	4.6	122	1.42	1.22	107.1	1.9	high	9.3	3.5	0.1	25	12
PS	57	M	3	1	28.3	4.1	139	1.36	1.1	83.5	1.8	high	8.2	2.5	0.37	38	10.6
PS	59	F	30	1	19.9	3.3	198	1.22	1.22	87.3	2.1	high	8.8	5.4	0.4	22	9
PS	77	M	16	1	19.5	4.3	144	1.61	0.81	122.8	1.6	high	10.1	6.4	0.1	27	13.2
PS	79	F	11	1	17.1	3.6	146	2.03	1.02	134.3	1.6	high	9.9	6.4	0.1	27	11.2
PS	57	M	10	1	22.2	3.3	103	1.35	1.7	150.4	1.8	high	10.2	3.2	0.23	35	7.3
PS	61	M	5	1	22.4	3.4	170	1.13	0.61	105.6	1.6	high	9.2	4.6		26	9
PS	73	M	5	1	23.1	3.5	114	1.22	0.68	94.64	2.1	high	8.6	4.6		28	10.8
PS	63	F	20	1	22.7	3.5	112	1.13	0.94	137.7	1.8	high	9.1	4		25	
PS	45	F	3	1	23.5	2.8	162	1.45	0.72	114.4	1.8	high	8.5	8.5	0.2	37	13.2
PS	56	M	13	1	21.5	3.8	122	1.63	0.96	134.1	1.8	high	7.6	4.5	0.55	29	8
PS	75	M	10	1	21.3	3.8	123	1.36	0.94	106.2	1.8	high	9.8	7.7	0.5	30	10.1
PS	67	M	16	1	21	3.8	147	1.45	1.09	127.8	1.4	high	10.6	5.5	0.74	20	9.4
PS	74	M	7	1	23.8	3.7	119	0.83	0.84	122.6	1.3	high	8.7	3	0.27	25	9.4
PS	67	F	6	1	19.4	3.4	175	1.39	1.12	142.2	2.1	high		10.4	0.27	30	11
PS	31	F	3	1	23.9	2.8	165	1.87	1.09	126.5	1.5	high	7.8	6.1	0.1	29	10.1
PS	61	F	2	1	18.6	3.6	116	1.33	1.03	123	1.5	high		6.2	0.63	39	9.3
PS	51	F	5	1	21.4	3.7	214	1.08	1.05	130.8	1.8	high	9.5	4.6	0.51	24	9.8
PS	66	M	7	1	25.5	3.8	199	1.69	0.85	130.3	1.8	high	11.8	8.8	0.05	25	10.6
PS	60	M	9	1	24	3.9	94	1.11	1.14	135.7	1	high	9.2	4.2	1.9	36	9.3
PS	52	F	2	1	21.3	4		1.78	1.06	109.6	1.3	high	8.4	1.5	2.63	33	10.5
PS	60	M	2	1	25.6	3.4	134	1.48	0.94	105.7	1.5	high	8.5	5.3		34	10.8
PS	58	M	3	1	22	3.4		1.23	0.71	119.8	2.1	high	9.3	9.3	0.05	26	9.9
PS	67	M	5	1	21.3	3.8	159	2.02	0.81	117.9	1.6	high	9	6.3	0.1	43	11.8

PS	55	F	15	1	19.7	3.5	143	1.24	1.39	146.6	1.8	high	8.9	4.6	0.02	15	11.5
PS	73	M	5	1	20.7	3.6	136	1.44	0.88	59.92	2.1	high	9.3	5.3	0.07	21	9
PS	60	M	10	1	22.6	3.9	124	1.99	1.13	173.9	1.5	high	8.3	3.5	1.67	38	8.1
PS	64	M	6	1	17.1	3.1		1.58	0.87	98.62	1.8	high	10.3	7.9		24	11.2
PS	42	F	6	1	18.1	3.2		0.92	0.52	123.8	1.5	high	8.7	6.9	0.19	33	11.2
PS	68	M	15	1	23.4	3.9	179	2.03	1.32	117.1	1.8	high	7.1	6.7	0.01	51	10
PS	60	M	12	1	23.3	3.7	191	1.02	0.89	151.2	1.5	high	9	5.1	0.05	24	11.1
PS	71	F	18	1	18	3.4	130	1.23	1.1	144.8	2.1	high	9.1	4.2	0.31	28	12.8
PS	52	M	11	1	16.3	4.1	172	1.75	0.82	92.73	1.6	high	8.9	6.1	0.1	7	11.6
PS	62	F	10	1	23.5	3.7		1.13	0.8	85.22	1.6	high	7.8	4.9	0.25	25	10.8
PS	61	M	6	1	24.7	4.2		1.18	1.04	104.7	2.2	high	9	4	0.05	27	10.2
PS	64	F	5	1	22.9	4.2	135	1.19	0.98	86.39	1.5	high	8.6	5.4	0.08	30	10.1
PS	77	M	3	1	20.6	4.2		1.35	0.83	93.2	2.1	high	8.8	5.6	0.07	24	9.5
PS	63	M	3	1	23.6	4.1		1.4	0.88	78.5	1.5	high	9.3	5.9	0.18	25	9.7
PS	67	M	2	1	18.7	4.1		1.3	0.94	106.1	1.5	high	8.6	3.7	0.19	27	10
PS	67	M	4	1	19.6	3.6	158	2.31	0.92		1.8	high	9.8	8.3	1.3		10.9
PS	70	F	20	1	20.2	3.9	108	0.99	1.01	114.9	1.6	high	8.3	5	0.32		10.4
PS	38	M	15	1	20.2	4.3	157	1.02	0.61	63.22	1.3	high	9.6	4.7	1.47		11
PS	60	M	11	1	18.4	3.5	109	1.53	0.73	81.05	2.1	high	8.7	4.5	0.04	23	9.8
PS	67	M	7	1	18.8	3.9	115	1.62	0.95	105.6	1.6	high	8.7	5.3	0.12	46	10.9
PS	66	M	5	1	19.9	4.1	128	1.5	1.32	117	2.1	high	11.7	7.3	0.17	46	9.8
PS	66	F	5	1	22.5	3.9	134	0.97	0.71	80.34	2.1	high	10.7	7	0.08	27	9.9
PS	66	M	4	1	21.5	3.8	164	1.33	0.65	81.21	1.1	high	9.2	6.8	0	26	11
PS	47	M	3	1	24.2	3.6	126	0.99	0.98	85.4	2.1	high	8.9	6.1	0.05	18	11.6
PS	79	M	2	1	21.5	3.8	160	1.94	0.75	122.9	2.1	high	8.5	5.3	0.08	28	10.9
PS	64	F	19	1	18.4	3.6	189	1.79	1.06	119.4	2	high	9.8	3.6	2.67	37	9.2
PS	60	M	27	1	19.2	4.3	111	1.82	0.85	88.23	2.5	high	9	6.2	0.02	25	10.2
PS	72	F	15	1	15.3	3.8	121	1.36	1.01	110.7	2.1	high	9.1	5.1	0.05	24	11.1
PS	66	M	15	1	22.2	3.9	130	1.61	0.88	120.3	1.6	high	8.4	4	0.1	25	9.7
PS	71	M	13	1	23.4	3.6	138	1.42	1.18	119.7	1.5	high		5.8	0.05	24	10.5
PS	34	F	13	1	12.6	4.5	107	1.18	1.18	119	1.9	high	9.8	7			11.9
PS	59	M	12	1	19.4	3.9	159	1.24	1	111.1	1.9	high	10.5	3.9			10.6
PS	33	M	11	1	21	4	176	1.16	1	84.35	1.1	high	9	5.2	0.02	25	10.9
PS	68	M	11	1	20.3	3.7	110	1.4	0.82	112.3	1.5	high	8.3	4.6	1.41	21	10.3
PS	61	M	10	1	22.6	4	180	1.63	0.95	128.8	1.1	high	9.4	5.9	0.12		11.1
PS	73	F	9	1	20.6	4	147	1.61	0.83	128.1	1.3	high	9.1	5.6	0	18	9.9
PS	47	M	9	1	17.4	4.3	86	1.37	0.92	94.46	1.9	high	10.5	5.2			10.6
PS	58	M	8	1	22.9	4.3	178	1.29	1.01	111.9	2.1	high	8.9	4.6	0.09	18	10.5
PS	27	F	8	1	19.5	3.8	175	1.64	1.19	94.52	1.6	high	8.6	7		17	10.3
PS	31	M	7	1	19.4	4.6	138	1.29	0.75	103.5	2.5	high	7.6	2.7	0.06	25	11.5
PS	63	F	7	1	24.8	4	171	1.07	0.85	98.73	2.1	high	10.8	6.1	0.17	29	10.8
PS	51	M	7	1	19.2	4.4	130	1.9	0.78	92.88	1.5	high	10.8	5.6	0.01	25	10.2
PS	70	M	6	1	25.5	4.2	175	1.54	0.77	111.4	1.5	high	10	6.2	0.3	28	10.8
PS	37	F	6	1	18.8	3.5	141	0.99	0.82	121.3	1.5	high	9	5.2	0.03	13	9.1
PS	55	F	6	1	31.2	3.6	149	1.32	1	101.5	1.3	high	8.5	5.3	0.03	31	9.2
PS	59	F	5	1	26.7	3.7	126	1.57	0.82	142.2	2.1	high	10.5	6.8	0.04	25	9.9
PS	61	F	5	1	21.8	4	126	2.11	0.93	132	1.8	high	8.9	2	0.48	26	11.2
PS	52	F	5	1	20.9	3.8	100	1.32	0.73	108.1	1.3	high	9.8	4.2	0.1	30	10.1
PS	88	F	4	1	23.8	3.8	177	1.49	0.94	112	1.4	high	9.7	2.7	4.38		7.4
PS	72	M	3	1	27.9	3.5	163	1.11	0.73	104.4	1.5	high	7.2	5.7		27	8.7
PS	49	F	3	1	17.6	4.1	237	1.36	1.09	88.07	1.3	high	10.1	4	0.1	24	9.8
PS	66	M	3	1	25.1	3.8		1.48	0.65	75.89	1.5	high	8.6	5.4	0.56	26	9.6
PS	48	M	3	1	21	4.3	116	1.23	0.79	49.56	1.9	high	8.1	8.7			10.5
PS	72	M	8	1	27	4	116	1.49	0.97	117.2	1.9	high	9.1	6			9.5
PS	61	F	8	1	21.5	4	135	1.28	0.94	97.81	2.1	high	7.9	6.8	0.26	24	12.2
PS	71	F	3	1	15.8	3.8	226	1.37	1.17	90.95	1.8	high	10.1	8.4	0.05	22	12.1
PS	55	M	19	1	21	3.6	94	1.35	0.87	95.33	1.5	high	10.1	5	1.1	35	8.5
PS	70	M	18	1	20.5	3.7	190	1.9	0.95	105.5	1.8	high		5.3	2.86		10.7
PS	73	M	5	1	22.5	4.1	117	1.77	0.84	96.43	1.5	high		5.2	0.07		10

PS	66	F	17	1	17.6	1.5	108	1.04	1.04	100.4	1.9	high	7.8	7.1			10.7
PS	77	M	9	1	21.7	3.3	93	1.34	0.92	107.9	1.5	high	8.3	3.9	0.96	41	
PS	73	M	2	1	27.6	3.9	204	0.98	0.87	110.1	1.6	high	8.7	5.1	0.05	25	10
PS	59	F	22	1	17.8	3.9		1.26	0.97	117.1	2.2	high	9.7	4.8		26	12
PS	42	F	3	1	19.5	4	181	1.98	0.99	71.85	1.6	high	10.3	4	0.05	19	11
PS	65	F	10	1	25.8	3.3	143	1.8	1.34	122.4	1.3	high	8.7	5.5	0.06	28	12.5
PS	72	M	23	1	22.4	3.7	164	2.01	0.82	110.5	1.6	high	10.7	5	0.05		10.9
PS	60	M	35	1	22.4	3.8	128	1.4	0.9	93.71	2.5	high	8.9	5.9		37	9.1
PS	65	F	31	1	22.7	3.6	141	1.19	1.13	115.7	2.1	high	8.3	4.4	0.3		11.2
PS	64	F	29	1	18.7	3.8	152	1.35	0.93	110.2	1.9	high	9.3	5.8		36	9.4
PS	61	M	21	1	27.1	4	215	1.21	0.88	109.3	1.8	high		5.3	18.31		12.5
PS	61	F	19	1	16.6	4		1.7	1.12	107.1	2.2	high	9.1	4.1		25	11.5
PS	55	M	16	1	20.9	3.6	188	1.61	0.85	118.4	1.8	high		7.4	0.16		11.3
PS	53	F	15	1	17.8	4.1	263	1.87	0.87	114.8	1.1	high	9.8	5.4	0.11	21	9.7
PS	72	M	13	1	22.2	3.1	126	1.6	0.81	82.4	2.1	high	9.1	4.5			
PS	61	F	10	1	24.1	4.1	172	1.55	0.8	114.5	1.8	high	7.5	3	1.06	13	9.5
PS	55	M	9	1	16.5	3.8	143	1.91	1.01	131.2	1.9	high	7.9	5.1	0.05	40	11.1
PS	76	M	8	1	23.5	3.7	200	1.13	0.81	116.1	2.1	high		8.2	1.44		11.6
PS	73	F	8	1	21.3	3.3	180	1.74	1.1	105.1	1.9	high	8.1	3.5	0.06	26	11.1
PS	71	F	7	1	22.2	4.1	115	1.66	0.86	108.3	1.9	high	8.6	4.7	0.05	23	10.4
PS	55	F	6	1	23.1	3.6	181	1.03	0.72	102.6	1.1	high		6.5	0.3	36	8.8
PS	65	M	6	1	22.1	4	164	2.11	0.88	136.8	1.6	high	9.2	4.8	0.05	26	11.6
PS	57	F	5	1	16.8	4.3	167	1.2	0.72	67.1	2.1	high	8.8	4.2	0.3	32	7.7
PS	58	M	5	1	21.1	3.3	117	1.61	0.63	92.32	2.1	high	9.7	6.1	0.06	25	10.6
PS	60	F	4	1	25.5	3.7	115	1.34	1.06	95.16	1.8	high	9.4	7	0.1	34	11.5
PS	59	M	4	1	25	4.1	194	1.65	1.19	121.4	1.8	high		4.5	0.05	22	10.8
PS	64	M	3	1	28.3	3.7	149	1.6	0.79	74.64	1.9	high	9.5	3.1	0.16	29	11.6
PS	59	M	3	1	23.5	3.7	138	1.58	1.14	113.1	1.6	high	7.9	3.1	0.08	14	11
PS	57	M	2	1	24.8	3.5	113	1.39	0.68	64.56	1.5	high	8.3	4.8	0.1	21	10.9
PS	37	M	24	1	22.7	3.8	136	1.7	0.84	84.09	1.6	high	8.7	6.6	0.07	29	9.8
PS	43	M	19	1	20.5	3.9		1.15	0.86	110	1.5	high	8.5	7.5	0.52	28	11.2
PS	33	F	5	1	18.6	4	160	1.72	1.19	96.45	2.1	high	9.1	3.6	0.1	21	12.2
PS	64	M	30	1	22.4	3.6	138	1.45	0.85	105.6	2.2	high	9.9	4.1	0		10.1
PS	61	M	23	1	20.2	3.4		1.14	0.79	112.6	2.1	high	9.5	7.7	0.55	14	10.5
PS	72	M	15	1	24.2	3.8	128	1.38	0.95	118	1.5	high	8.8	6	1.4	32	12
PS	71	F	9	1	21.1	3.5	153	1.49	0.83	134	1.6	high	9.1	5.6	0.05	28	11.1
PS	65	M	8	1	22.7	3.7	142	0.95	0.65	98.28	2.1	high	9.3	5.1	1.36	20	11.3
PS	58	M	7	1	23	3.6	169	1.97	0.85	111.5	2.1	high	8.9	5.5		33	9.5
PS	71	M	6	1	18.6	4	189	1.31	0.95	116.2	1.3	high	8.5	5.5	0.05	25	10.5
PS	61	M	3	1	21	4.2	97	1.27	0.75	107.1	2.1	high	10.1	2.6	0	30	10.1
PS	71	F	3	1	15.5	3.9	132	1.97	1.09	118.4	1.8	high	8.3	4.4	0.02	31	11
PS	71	M	3	1	23	4	134	1.4	0.69	95.61	1.5	high	7.8	6.8	0.14	23	10.3
PS	50	F	31	1	23.8	4		1.6	1	102.8	1.4	high	8.4	5.2	0.06	25	10.7
PS	68	M	5	1	19.2	4.1	30	1.71	1	67.99	1.3	high	8.9	6	0.1	27	11.2
PS	55	F	24	1	19.2	4		1.39	1.14	107.3	2.1	high	9	7.2		28	10.9
PS	58	M	15	1	22.9	4.1	130	1.8	0.98	125.3	1.9	high	9.4	4.5	0.07	29	10.6
PS	52	F	13	1	15.6	4.4	197	1.38	0.76	96.88	1.6	high	9.2	3	0.1		8.7
PS	46	M	12	1	27.6	3.8	134	1.55	1.03	111.7	1.9	high	9.1	4.5	0.3		10.3
PS	59	M	9	1	24.1	3.7	91	1.75	0.83	109.4	2.1	high	8.7	5.8	1.36		9
PS	62	M	9	1	23.8	4.1	164	1.31	0.94	117.7	1.8	high	8.3	5	0	30	11.5
PS	56	M	7	1	23	3.8	108	0.92	0.69	96.39	1.5	high	8.7	3	0.09	23	10.6
PS	67	M	4	1	20.9	3.6	156	1.38	0.99	112.8	1.8	high	8.8	7.4	0.43	30	9.7
PS	68	M	3	1	19.3	3.8		1.27	0.91	99.23	2.2	high	10.2	4.9	0.1	27	13.5
PS	72	M	4	1	23.2	3.9	199	0.93	0.92	125	1.8	high	9.1	8.1	0.05	23	11
PS	83	M	4	1	21.2	3.6	157	1.3	1.1	123.7	1.5	high	8.1	7.1	1.64	34	12.4
PS	74	M	4	1	24	3.8	170	1.28	0.86	70.67	1.6	high	8.5	4.7		34	12.1
PS	74	F	4	1	25	3.1	167	1.53	0.91	127.6	1.5	high	8.5	6.5	0.19	40	10.8
PS	73	F	8	1	21	3.7		2.02	0.97	70.08	1.5	high	7.9	7.2	0.2		9.6
PS	73	M	2	1	22.9	3.7	101	1.56	0.82	76.02	1.8	high	7.5	6.1			7.9

PS	73	M	3	1	19	3.7	205	1.72	0.94	114.3	1.5	high	8.9	5.9	4.29		12.3
PS	73	M	5	1	18.8	3.4	150	1.31	0.78	110.6	1.5	high		6.1	0.1	18	10.8
PS	73	M	7	1	20.7	3.6	135	1.31	0.86	122.7	2.1	high	9.6	4.7	0.14	27	11.2
PS	72	M	12	1	22.7	3.7	145	0.92	0.97	114.7	1.6	high	8.2	5	0.11	26	10.4
PS	71	M	6	1	20.9	3.6		1.23	0.98	90.62	1.1	high	8.6	3.2			6.8
PS	71	M	3	1	26.5	3.6	172	1.48	0.88	93.75	1.8	high	9.6	3.9			11.8
PS	71	F	16	1	17	3.5	207	2.11	1.05	113	1.8	high	9.7	5.1	0.12	31	10.5
PS	71	F	21	1	23.2	3.5	203	1.34	0.82	130.2	1.2	high	9.1	8.3		32	9.2
PS	70	M	7	1	22.3	3.9	102	1.62	1.34	116.6	1.5	high		5	0.2	29	11.7
PS	70	M	3	1	21.5	3.1		1.89	0.68	87.79	1.3	high	8.8	3.4	1.18	41	9.1
PS	70	F	3	1	20.7	3.6	144	1.47	0.93	69.18	1.8	high	8	4.1			10.3
PS	70	F	6	1	20.8	2.8	44	1.46	0.76	114	1.8	high	6.1	7.2	0.08	29	10.9
PS	69	F	8	1	17.4	4.1	136	1.52	0.95	121.3	1.9	high	11.2	5.7	0		9.3
PS	69	M	5	1	21.9	3.5		1.87	0.72	114.4	1.8	high	9.5	4.2	0.15	17	10.3
PS	69	F	13	1	25.1	3.4	146	1.38	0.77	123.6	1.5	high	10.2	6.2	0.05		
PS	69	M	11	1	18.3	3.5	178	1.29	0.94	85.91	1.9	high	8.9	5.3	0.2		11
PS	69	M	13	1	20.4	3.3	165	1.45	0.81	101.7	1.2	high	8.8	5.6	0		12.8
PS	69	F	8	1	27.3	3.7	132	1.12	0.86	91.51	1.3	high		7.5	0.1		14
PS	69	F	10	1	18.6	3.5	110	1.55	0.91	115.5	1.8	high	9.5	4.5	0.69	25	10.2
PS	68	M	33	1	19.5	3.3	154	1.37	0.82	102	1.6	high	7.6	4.1	0.2		9.9
PS	68	M	15	1	24.9	3.4	158	1.38	0.71	131.3	1.9	high	8.6	4.9	0		9.6
PS	68	F	27	1	20.7	3.4	99	2.23	0.96	66.65	1.6	high	9.3	4.5	0.09	46	9.1
PS	68	F	4	1	23.1	3.5	193	1.45	0.99	125.5	1.8	high		5	0.05	26	10.4
PS	68	F	21	1	19.1	3.6	104	1.29	1.01	108.8	1.9	high	10.7	5.4	1.3		7.9
PS	67	F	4	1	19.5	3.8	142	1.68	1.19	110.8	1.9	high	7.9	4.7	2.3		7.6
PS	67	M	3	1	20.1	4.1	105	1.3	0.72	95.49	2.1	high	10.3	2.3	2.9		9
PS	67	F	3	1	23.2	3.4	143	1.21	1.05	74.19	2.1	high	8.3	6.5	0.3	21	11.4
PS	66	M	3	1	21.3	3.4	162	1.34	0.56	115.8	2.1	high	10.1	6.1		30	9.8
PS	66	F	18	1	19.6	3.5	144	1.36	0.83	72.9	1.5	high	9.2	4.8	0.1	26	10.9
PS	66	M	9	1	20.1	3.5	140	2.02	1.14	122	1.3	high	9.1	4.7	0.4	20	11.1
PS	66	F	4	1	20.4	3.5	179	1.42	0.88	103.7	1.6	high	9.4	4.4		26	9.2
PS	65	M	21	1	23.1	3.7	177	2.92	1.35	128.4	1.6	high	10.2	7.6	0.17	35	12.8
PS	65	M	5	1	22	3.7	181	1.64	0.85	110.5	1.3	high	9.4	3.7	0.1		10.6
PS	65	F	3	1	16.7	3.9	133	1.29	0.9	103.1	1.5	high		6.4	0.1	19	10.6
PS	64	F	32	1	19.7	3.3	156	1.26	1.21	108.3	2.2	high	9.5	5.8	1.6	35	11.4
PS	64	F	3	1	35	3.7	112	0.93	1.07	107.6	1.5	high	9.3	5.5	0	27	11.3
PS	64	M	22	1	21.3	3.5	167	1.4	1.01	116.1	1	high	8.7	5.2		27	10.1
PS	64	M	2	1	21.5	3.9	121	1.28	1.21	105.6	1.8	high	10.2	4.8	0.05	16	10.5
PS	64	M	30	1	20.9	3.2	172	2.17	0.97	105.5	1	high	11.3	6.5	0.1	23	11.4
PS	64	M	22	1	19.3	4.2	194	1.74	0.92	113.8	1.3	high	10	7.8	0.09	28	12
PS	64	M	16	1	19.1	4.1	155	1.65	0.89	123.3	1.9	high	8.5	5.8	0.5		10.7
PS	63	M	36	1	18.6	3.2	202	1.69	0.75	73.67	1.6	high	9	7.4	0.29	24	10.1
PS	63	F	11	1	19.6	3.8	183	1.81	0.76	134.5	2.1	high	10	5.4	0.63	23	10.6
PS	62	F	12	1	16	3.8	100	1.22	0.93	104.5	2.2	high	8.3	6	0.05	28	10
PS	61	F	6	1	23.4	3.5	185	1.95	0.98	129.8	1.5	high	10.7	6.2	0.17	25	10.5
PS	61	F	7	1	26.8	3.4	101	1.2	0.88	139.2	2.1	high	10	5.1	0.07	26	10.5
PS	61	M	4	1	26.2	3.9	164	0.87	1.16	101.2	1.6	high	8.8	4.9	0.09	26	10.3
PS	61	M	5	1	23.4	3.5	133	1.58	0.94	100.5	1.5	high	9.1	6	0.55	22	10.4
PS	61	M	16	1	23.6	3.5	37	2.01	1.02	111.1	1.5	high	9.2	5.8	0.4	24	11.9
PS	61	M	8	1	22.3	3.2	152	1.42	0.95	130.7	1.6	high	8.4	4.5	0.12	26	10.9
PS	60	M	4	1	20.1	4	189	1.59	1.03	117.1	1.8	high	10.8	4.9	0.03	26	11.9
PS	60	M	11	1	23	3.3	78	1.26	0.9	90.31	1.6	high	7.3	6.5	0.06	25	10.1
PS	60	M	32	1	21.6	2.7	159	1.73	0.59	63.73	1.6	high	9.9	5.6	0.04	22	12.1
PS	60	F	2	1	23.4	3.7	153	1.23	1.1	135.9	2.1	high	9.9	6.2	0	25	10.8
PS	59	M	4	1	23.2	3.7	170	1.37	0.79	129.5	1.3	high	9.1	5.8	0.2	44	10.1
PS	59	M	14	1	17.4	3.5	136	1.44	0.88	104.3	1.9	high	9.4	5.2	0.2		10
PS	59	M	16	1	19	3.7	189	1.76	1.17	104.6	1.9	high	8.9	4.7	0.2		8.6
PS	58	F	6	1	24.8	3.8	258	1.34	1.18	106.5	1.3	high	9.8	5.8	0.02	21	9.9
PS	58	M	2	1	22.1	3.5	199	1.18	0.69	83.41	1	high	9.5	2.9	0.83	26	11.7

PS	58	M	27	1	20.2	3.5	187	1.97	1.21	64.05	1	high	9.3	6	0.04	27	10.6
PS	57	F	2	1	27.5	4	121	1.31	1.11	89.92	1.5	high	8.8	5.5	0.1	23	11.9
PS	57	M	25	1	23.2	3.4	128	1.23	0.76	97.36	1.8	high	11	4.4		31	12.7
PS	57	M	11	1	21.8	3.8	192	1.64	0.98	127.3	1.9	high	10.6	7.9	0.1		10.8
PS	57	M	7	1	22.7	3.9	160	2.08	1.12	124.4	1.5	high	8.4	4.1	0.62	25	11.4
PS	56	M	3	1	20.3	3.4	42	1.51	0.77	74.03	2.1	high	9.2	9.1	0.2	22	12.3
PS	56	M	8	1	21.8	3.8	152	1.28	1.14	118.2	1.6	high	9	4.4	0		11.8
PS	55	F	5	1	34	3.9	131	1.9	1.38	111.9	1.8	high	9.1	5.3	0.05	29	10.2
PS	54	M	9	1	24.9	3.9	157	1.58	0.98	90.56	1.3	high	9.9	5.5	1.8	30	12.1
PS	54	F	25	1	21.5	3.9	183	1.9	1.14	106.4	1.3	high	9.4	5.4	0.2	21	11.1
PS	53	F	25	1	19.7	3.5	133	1.19	0.8	94.38	1.2	high	9	8.5		35	9
PS	53	F	4	1	30.2	3.8	206	1.9	0.62	63	1.5	high	9.1	5.6	0.08	22	11.1
PS	53	M	5	1	23.9	3.9	134	1.55	0.79	105.5	1.8	high	9.6	4.7	0.02	29	9.7
PS	52	M	7	1	19.7	3.8	179	1.34	0.88	89.86	1.9	high	8.2	5.3	0.1		8.3
PS	52	M	14	1	23.5	3.9		1.16	0.96	109.7	1.4	high	9.6	6.6		35	9.5
PS	51	M	5	1	26.8	3.9	149	1.48	1.06	106.5	2.1	high	9.2	5.6		28	9.2
PS	51	F	2	1	34.3	4.1	197	1.43	0.72	77.88	1.3	high		6.6			10.3
PS	48	M	3	1	29.8	3.6	141	1.36	0.65	82.45	2.1	high	7.9	6.3	0.08	27	11.1
PS	47	M	20	1	23.9	3.8	136	1.22	0.77	107.8	1.2	high	9.2	8.6		28	8.4
PS	47	M	6	1	30.1	3.7		2.26	0.91	127	2.1	high	8.9	3.6	0.2	33	11
PS	47	F	12	1	23.6	3.5	130	1.21	0.89	114.3	2.1	high	9.6	7.7	0.12	32	11.7
PS	46	M	4	1	36.2	4.2	128	1.88	0.87	86.51	2.1	high	8.6	5		26	9.2
PS	46	M	4	1	25.2	4.1	113	1.24	1.04	107.3	2.1	high		5.6	0.06	29	11.5
PS	44	F	17	1	18.3	3.9	104	1.68	0.8	122.9	1.9	high	7.9	4.5	3.5		10.3
PS	44	M	8	1	24	3.8	149	1.37	1	142.5	1.5	high	10.6	3.3	1.57	28	11.2
PS	42	M	2	1	31.1	3.4	103	1.71	0.71	82.39	1.8	high	8	5.2	0.14	27	11.1
PS	41	M	7	1	23.8	3.7	133	0.82	0.85	125.1	1.5	high	8.7	5.2	0.1	27	10.9
PS	32	M	4	1	22.3	3.9	158	1.42	0.92	89.84	2.1	high	9.4	5.9	0.13	20	11.5
PS	53	M	15	1	22.5	4.3	113	1.53	0.96	123.8	1.8	high	8.6	5	0.07	26	
PS	57	F	30	1	19.7	3.4	128	1.58	0.96	83.13	1.6	high	9.1	4.2	0.2		10
PS	65	F	11	1	18.1	3.7	180	1.56	0.7	102.2	1.3	high	9	5.4	0.02	20	10.9
PS	59	F	14	1	23.1	3.5	193	1.17	1.04	130.3	1.3	high	10.6	5.5	0.47	25	9.2
PS	65	M	13	1	20.7	3.5	125	1.03	0.73	68.65	1.5	high	9.2	7.1	0.1	28	9
PS	69	M	12	1	23.9	3.8		1.46	0.81	103.9	1.5	high	8.5	4.6	2.24	34	8
PS	64	M	11	1	22.5	3.4	173	1.49	1.29	144.8	1.3	high	8.7	5.1	0	20	10.8
PS	57	F	9	1	17.8	3.7	171	1.3	1.28	127	1.6	high	9.4	5.7	0.2		10.8
PS	48	M	9	1	22.3	3.9	189	1.58	1.13	107.6	1.6	high	8.6	5.8	0.1		9.4
PS	69	M	7	1	25	3.4	144	1.31	0.92	121.4	2.1	high	8.6	6.8		31	10.4
PS	80	M	6	1	18.2	3.4	143	1.39	0.93	93.81	1.3	high	9.4	3.5	0.1	31	11.7
PS	70	M	6	1	23.9	3.5	156	1.5	0.93	143.9	1.9	high	7.5	3.6	0		10
PS	66	M	5	1	24.4	3.8	127	1.12	0.88	133.5	1.6	high	8.9	6.2		29	10.1
PS	71	M	5	1	22.5	3.7	187	1.81	0.81	118.6	1.8	high	10.2	7.7	0.01	33	9.4
PS	58	M	5	1	23.7	3.4	182	1.01	0.89	116.9	1.8	high	9.3	6.1	0.1	27	12.3
PS	58	M	5	1	21.8	3.9	182	1.24	1.03	91.08	1.8	high	10.2	7.5	0.29	28	9.5
PS	70	M	4	1	21.1	3.6	171	1.64	1.06	117.3	1.6	high	9.8	5.8		16	9.7
PS	70	M	4	1	19.1	3.5	118	1.11	0.92	127.4	2.1	high	8.4	6.1	0.07	27	13.6
PS	87	M	4	1	16.1	3.6	164	1.31	0.9	118	1.5	high	9.3	3.9		35	10.3
PS	40	M	3	1	31.3	3.5	143	1.27	0.45	95.49	1.8	high	8.4	4.3	0.27	31	14.3
PS	68	M	2	1	25.3	3.5	107	1.45	1.16	105.9	1.8	high	9.2	5.7	0.01	33	8.5
PS	69	M	2	1	20	3.8	235	1.51	0.87	83.89	1.5	high	7.9	3.7		17	
PS	44	M	2	1	30.4	3.9	182	1.17	0.69	112.2	1.9	high	10	5.3	0.1		10.2
PS	67	M	2	1	25.7	3.8	148	1.53	0.82	77.03	2.1	high		4.9	0.14	24	11.7
PS	71	F	33	1	24.5	3.8	174	1.92	1.01	128.9	1.9	high	8.2	7.9	0		10
PS	75	M	16	1	25.4	3.5	166	1.57	1.03	121.8	1.6	high	9.2	4.6	0		11
PS	64	F	12	1	23.8	3.7	123	1.42	1.11	121.2	1.6	high	9	5.6	0		10.3
PS	65	F	12	1	17.7	4.5	161	1.27	1.22	117.2	1.9	high	9.1	4.9	0.7		11.3
PS	62	F	19	1	19.7	3.9	132	1.4	1.03	134.9	1	high	8.1	6.3	0.16	27	8
PS	63	F	21	1	20	3.9	163	2.19	0.96	131.8	1	high	9.3	3.6	0.2	26	11.5
PS	55	F	14	1	17.9	4.3	160	1.32	1.02	139.3	1.3	high	8.6	5.4	0.26	27	9.3

PS	59	F	14	1	21.8	3.9	143	1.18	0.78	147.6	1.8	high	9.2	5		29	11.6
PS	72	M	25	1	21.8	3.5	127	1.99	0.89	121	1.5	high	9.8	4.4	0.1		10.3
PS	72	F	15	1	18.7	4.3	138	1.98	1.17	105.1	1.6	high	9.7	5	0.2	26	9.5
PS	59	F	12	1	21.3	3.8	143	1.3	1.02	118.2	2.1	high	8.4	5.9		25	10
PS	61	M	11	1	21.8	3.8	162	1.09	1	126.1	1.3	high	8.3	4.5	0.1	16	11.6
PS	46	F	11	1	32	3.9	171	2.32	0.92	155.3	1.6	high	9.6	4.7	0.2	24	8.5
PS	63	M	11	1	21.1	4.7	128	1.88	0.75	154.2	1.6	high	10.7	5.4	0.2	30	8.9
PS	58	F	11	1	23.7	3.7	175	1.09	1.02	94.56	1.1	high	8.6	5.2	0.1	16	11.8
PS	48	F	10	1	23.5	3.9	163	1.03	1.13	130.8	0.6	high	9.5	6	0.2	35	10.7
PS	43	M	9	1	22.6	4.5	152	1.8	1.19	111.4	1.3	high	9	11.4	1.9	28	9
PS	54	M	9	1	17.9	4.2	188	1.83	1.03	138.4	1.4	high	9.1	4.7	0.14	33	10.3
PS	43	M	9	1	24.1	4.2	174	1.4	0.89	126	1.6	high		6.4		15	10.5
PS	66	F	11	1	26.9	3.8	118	1.27	0.63	148.7	1.3	high		4.8	1.23	24	11.2
PS	72	F	8	1	18.2	4	210	0.96	0.87	122.1	1.3	high		7.7		16	12.4
PS	59	F	16	1	16	4.2	186	1.71	1.22	147.2	1.9	high		6.2		32	10
PS	47	M	6	1	18.4	3.2	211	1.3	0.79	79.96	1.3	high	9.4	5.6	0.09	27	9
PS	72	F	8	1	19.1	3.9	122	1.46	1.1	124.4	1.6	high		5.5	2.01	32	9.8
PS	67	M	14	1	22.1	3.7	89	1.69	1.06	132.4	1.8	high	8.3	4	0.41	21	8.8
PS	63	M	5	1	22.1	3.8	136	1.27	0.8	99.59	1.8	high	8.8	5.6	0.33	27	9.5
PS	61	M	14	1	18.8	4.4	166	1.22	0.87	117.9	1.2	high	9	5.3	0.35	36	12.8
PS	61	M	3	1	23.3	4	127	1.64	0.9	121.1	1.3	high	8.2	7.9	1.71	21	10
PS	72	M	2	1	27.2	3.6	142	1.15	0.79	108	1.6	high		4.5	0.1	20	10.5
PS	45	M	18	1	17.8	3.8	114	1.13	0.97	112.3	1.9	high		5.7	0.04	25	10.4
PS	74	M	2	1	21.2	3.4	258	1.49	0.9	111.5	1.6	high	8.7	9.3		29	11.2
PS	77	M	16	1	19	3.2	155	1.65	0.69	86.18	1.5	high	10.2	7.2	0.18		11.9
PS	57	F	15	1	16.9	4.3	119	1.36	0.79	100.5	1.8	high	8.4	6.3	0.07	35	11
PS	55	F	5	1	20.2	3.8	110	1.93	1.15	81	1.5	high	8.4	5.5	5.52	31	9.2
PS	72	F	3	1	23.7	3.9	151	1.75	1.06	107.4	1	high	9.2	5.1	0.05	28	10.6
PS	49	F	14	1	18.7	3.7	137	1.5	1.07	119.6	1.3	high	9	4.3	0.18	26	11
PS	47	M	27	1	23.1	4.2	155	1.76	1.09	126.4	1.5	high	8.7	4.3	0.11	21	9.4
PS	58	F	9	1	29.4	3.5	141	1.63	0.67	133.4	1.6	high		6.3	0.4	30	9
PS	58	F	6	1	20.8	4.1	128	1.72	1.11	88.47	1.5	high	9	5.5	0.3	28	11.6
PS	32	M	2	1	21.4	4.3	202	2.04	1.27	73.91	1.5	high	11	4.2	0.3	23	10.1
PS	68	M	26	1	18	3.8	222	1.75	1.04	108.2	1.6	high	5.2	5.2		21	10.3
PS	49	F	15	1	16.3	3.7	176	1.51	0.69	117.9	1.5	high	10.4	4	0.1		9.7
PS	31	M	3	1	17.6	4.2	105	1.36	0.74	63.22	1.8	high	9.1	7.1	0.07	26	11.3
PS	69	F	3	1	17.9	3.9	111	2.11	1.34	64.89	1.6	high		7		36	9.3
PS	54	M	6	1	24.8	4.3	124	1.69	0.82	104.8	2.5	high	8.4	7.5	0.1	25	10
PS	62	M	6	1	21.9	3.9	116	1.3	1.48	135.6	1.9	high		6.3		29	9.8
PS	63	M	16	1	20.9	4	151	1.65	1	101.1	1.3	high	10.1	6.1	0.08	26	9.9
PS	57	M	3	1	22.8	4.2		1.59	1.09	119.4	2.1	high	10.5	7.3	0.08		11.3
PS	69	F	2	1	23.1	3.8	155	1.17	0.53	73.51	1.9	high		6.5		26	9.7
PS	75	M	14	1	13.8	3.6	158	1.69	0.69	82.18	1.5	high	9.9	6.4		28	10.6
PS	42	M	4	1	22.2	3.1	189	1.48	1.02	94.46	1.8	high	8.2	3.6	4.81	27	10
PS	56	M	2	1	22.1	3.9	105	1.17	0.95	147.1	1.9	high		5.4	0.3	27	11.6
PS	68	M	6	1	19.8	3.6	171	1.37	1.17	133	1.5	high		3.2	0.12	25	11.6
PS	62	M	4	1	23.4	3.7	198	1.29	0.91	129.7	1.5	high	9.6	10	0	28	11.1
PS	68	F	8	1	22	4.3	107	1.6	1.2	120.6	1.8	high	8.1	3.9	0.16	26	9.6
PS	51	F	2	1	22.7	4.2	158	1.75	1.14	84.6	1.5	high		6.1	0.19	23	6.6
PS	60	M	3	1	18.9	3.7	138	1.25	0.69	82.28	1.5	high	9.4	7.1	0	27	10.4
PS	67	M	2	1	22.1	3.6	167	1.17	1.14	119.4	1.5	high	8.1	4	0.3	26	10
PS	63	F	3	1	21.8	4	139	1.1	0.85	74.84	1.5	high	9.4	4.6	0	29	10.1
PS	55	F	34	1	20	3.6	178	1.79	0.8	114.1	1.8	high	9.8	7	0.66	24	12.5
PS	65	M	21	1	25.4	3.8	223	1.3	0.68	115.5	1.8	high	8.5	6.4	0.3		11.6
PS	66	F	19	1	18.8	3.9	152	1.48	1.12	89.5	1.8	high	10.3	5.5	0.3	27	9.5
PS	70	M	19	1	22.5	3.6	127	1.39	1.14	138.5	1.9	high		5.7		28	11.6
PS	54	M	17	1	23.8	3.5	139	1.54	0.85	93.25	2.1	high	8.6	3.6	0.13		11.4
PS	71	M	16	1	18	3.8	151	0.9	1.54	160.3	1.6	high		6.7	0.8	32	13.5
PS	44	M	13	1	21.9	4	149	1.57	0.89	101.6	1.6	high		6	1.3	30	9.2

PS	68	M	12	1	17.8	3.3	160	1.86	1.08	110.7	1.3	high	8.8	4.7	0	25	11.6
PS	60	M	12	1	20.5	3.7	122	1.58	0.89	108.1	1.8	high	8.8	9.3	0.08	22	11.2
PS	72	M	12	1	23.6	3	134	1.47	0.56	85.15	2.1	high	9.1	4.8	0.05	28	10.6
PS	64	M	11	1	18.6	3.9	89	1.35	0.75	108.1	1.3	high	8.8	6.8	0.08	23	10.3
PS	68	M	11	1	18.8	3.9	154	1.18	0.95	103.6	2.1	high		6.2	0.07	23	10
PS	64	M	9	1	18.4	3.8	150	1.43	0.9	129.1	1.8	high	8.8	5.5	0.11	25	9.4
PS	65	M	9	1	23.8	3.6	142	1.33	0.86	103.1	1	high	8.5	6.1	1.9		9.7
PS	60	M	9	1	19.9	3.9	153	1.15	1.04	122.8	1.6	high		7.8		26	10.4
PS	73	M	9	1	22.4	3.6	110	1.3	1.07	114.2	1.6	high		4		23	7.9
PS	79	F	8	1	18	3.4	90	1.37	0.7	79.68	1.8	high	7.6	4.5	0.15	40	10.6
PS	66	M	8	1	19.9	3.8	110	1.08	0.77	93.11	1.5	high	7.5	5.1	0.14	30	8.1
PS	66	F	8	1	21.9	3.7	141	1.48	0.9	115.4	1.5	high	9.8	5.3	0.05		11.9
PS	60	M	12	1	23.7	4.2	182	1.57	0.63	112.9	1.5	high		5.2	0.14		10.3
PS	71	M	8	1	21.7	4.1	92	1.21	0.88	117.6	1.9	high		6.7		30	10.7
PS	67	M	6	1	19.4	3.7	215	1.05	0.76	90.47	2.1	high		6.4	0.05	23	10.7
PS	73	F	6	1	19.8	4.1	184	1.62	0.81	97.59	1.6	high		5.3	0.04	28	10.8
PS	81	F	6	1	21.7	3.5	119	1.04	0.72	47.5	1.6	high		4.7	0.2	26	10.1
PS	61	M	6	1	22.3	3.9	106	1.13	0.73	95.29	1.6	high		5.5	0.06	28	9.5
PS	57	M	6	1	22.1	4.1	159	1.45	1.12	135.4	2.1	high	9.9	5		23	10
PS	78	M	5	1	22.6	3.4	193	1.61	0.99	96.16	1.5	high	8.4	6.4	0.09	30	11.7
PS	70	F	5	1	22.6	3.8	161	1.64	0.87	109.1	1.6	high	9.4	7.9	0	29	9.5
PS	50	F	5	1	31.2	3.9	154	1.4	0.86	122.6	1.8	high	9.4	5.3	0.2	18	11.6
PS	60	M	4	1	27.9	3.6	149	1.38	0.86	140.3	0.6	high		5.1	0.2	33	9.1
PS	50	M	4	1	19.2	4.2	127	1.22	0.85	139.9	1.6	high		4.8		29	10.1
PS	64	M	4	1	22.5	4	176	1.31	0.91	150.1	2.1	high		5.9		30	11.5
PS	66	M	4	1	21.3	3.5	140	1.51	0.85	123.6	1.8	high	8.7	5.1	0.4	32	12.1
PS	68	F	3	1	26.1	3.5	168	1.25	1.46	143.3	2.1	high		4.3		28	10.9
PS	71	M	3	1	20.3	4	110	1.45	0.87	106.9	1.6	high		5.1		33	12.8
PS	66	M	3	1	27.4	3.9	170	1.37	0.77	88.65	2.1	high		5.8	0.03	30	11.9
PS	74	F	3	1	17.5	4	168	1.41	0.87	77.87	1.8	high		7.2	0.33	32	10.8
PS	62	M	2	1	25.1	4	130	0.77	0.69		1.1	high	8.7	5.7	0.4	24	10.8
PS	61	M	2	1	24.6	3.7	184	1.12	0.53	64.24	1.3	high	9.8	4.2	0.6	27	9.6
PS	79	M	2	1	20.8	3.8	170	1.92	0.8	102.5	1.6	high	8.5	5.6	0.81	30	11.2
PS	46	F	2	1	21.3	4	196	1.08	0.85	85.07	1.8	high	9.7	4.4	0.09	29	10.7
PS	65	M	2	1	19.5	3.7	175	0.76	0.87	82.79	1.3	high	8.4	4.7	0.09	32	8.7
PS	55	M	2	1	26.4	3.9		1.59	0.96	116.7	2.1	high	9.1	4.8	0	24	10.2
PS	60	M	8	1	22.1	4	173	1.15	0.66	114	2.1	high		6	0.05	23	9.6
PS	77	M	29	1	18.4	4.4	175	1.1	0.76	123.2	1.2	high	9.1	8.1		24	11
PS	52	M	20	1	21.7	4.4	159	1.42	0.95	129.9	1.8	high	9.6	6.2	0.1		11
PS	61	M	11	1	21	4.1	143	1.91	0.82	103.7	1.8	high	10.5	7.3	0.7	40	10.5
PS	57	F	11	1	17.7	4.2	139	1.22	1.26	111.1	1.6	high		5.1		31	12.1
PS	71	F	11	1	21.5	3.6	98	1.24	1.14	107.6	1	high	9.4	3.7	0.96	30	9.9
PS	58	F	10	1	21	4.4	138	1.24	1.29	119.1	1.5	high		5.5	0.2	17	10.8
PS	32	M	9	1	18.6	4.2	92	1.21	1.11	119	1.8	high	8.5	5.4	0.26		
PS	71	F	8	1	21.4	4	206	1.44	0.77	123.7	1	high	9	4.7	0.05	24	9.4
PS	54	F	7	1	19.8	4	168	1.76	0.95	117	1.3	high	9.5	4.4	0.04	24	9.3
PS	51	M	7	1	24.9	3.9	145	1.26	0.78	101.6	1.8	high	9	4.9		23	10.6
PS	77	F	22	1	19.2	3.8	182	2.11	1.03	109.5	1.5	high	8.2	4.9	0.07	26	8.3
PS	48	M	9	1	25.2	4.4	139	1.13	1.17	120.4	1.8	high	12.4	7.7	0.06	34	10.9
PS	66	M	6	1	20.3	3.7	161	1.18	1.08	96.74	1	high	9.6	3.6	0.07	27	10
PS	78	F	6	1	28.3	3.7	106	1.09	1.04	158.4	1.6	high		7.6		30	10.7
PS	72	M	6	1	21	3.9	142	1.58	0.78	77.7	1.5	high	9	6.7	0.5	25	10
PS	54	M	6	1	21.9	3.9	149	1.45	1.33	110.5	2.2	high	9.3	6.8	0.02	29	10.1
PS	60	F	6	1	15.8	4.2	171	1.4	1.36	53.56	1.3	high	7.4	8.7	0.55	28	11.2
PS	52	F	24	1	22.5	4.1	161	1.28	1.21	97.79	1.2	high	8.8	5.1		22	9.2
PS	70	F	13	1	21.8	3.9	179	1.15	0.98	75.43	2.2	high	9.8	7.3	0.05	19	10.9
PS	34	F	5	1	27.3	4.4	97	1.05	1.11	80.76	2.2	high		7.6	0.05	51	12.5
PS	58	M	5	1	19.3	4.1	134	1.82	1.1	106.2	1.5	high	8.9	5.2	0.07	33	12.1
PS	54	M	5	1	24	4.2	137	1.22	0.83	69.81	1.6	high		5.9		32	9.4

PS	74	F	5	1	18.9	3.7	121	1.62	0.92	103.4	2.1	high	10.5	3.9	0.02	20	10.4
PS	61	F	4	1	26.3	3.8	154	1.34	0.98	120.2	1.5	high	8.9	2	8.61	25	10
PS	60	F	28	1	19.4	4	163	1.36	1.06	83.42	1.8	high	8.4	4.6	0.05	23	11.2
PS	54	M	4	1	17.4	4	108	1.1	1.14	114.2	1.6	high	8.9	3.2	1.36	42	9.6
PS	64	M	4	1	19.5	4.2	149	1.27	0.86	133.4	1.3	high	9.2	5.3	0.04	21	9.3
PS	46	M	25	1	16.2	4.2	113	1.19	0.88	108.7	1.4	high	8.3	5.2	0	22	8.5
PS	73	F	3	1	16.8	3.9	136	1.11	1.09	101.4	2.1	high	9.4	9.1	0.12	32	10.4
PS	73	F	3	1	20.4	3.9	149	1.52	1.26	108.5	0.6	high	8	6	0	34	9.5
PS	57	M	3	1	18.6	4.1	175	1.07	1.1	126.4	1.4	high	8.6	5.9	0.1	20	9.8
PS	53	M	3	1	22.6	4	174	1.61	0.9	86.53	2.1	high	7.6	4.5	0.16	27	10.1
PS	57	M	23	1	18.9	4.4	134	1.44	0.92	101.9	1.4	high	9.1	5.8	0		10.2
PS	69	F	3	1	19	4.1	115	1.27	1.07	117.8	2.2	high	8.6	7.6	0.1	26	9.5
PS	70	M	2	1	24.1	4.3	166	1.43	1.04	102.4	2.1	high	10.1	6.6	0.74	30	8.6
PS	81	M	2	1	16.6	4	171	1.11	0.66	95.98	1.8	high	7.9	4.4	0.2	16	9.5
PS	67	M	5	1	23	4.1	190	1.38	0.76	137.8	1.1	high	10.8	4.9		33	11.5
PS	58	F	11	1	19	4.1	118	1.24	1.09	114.9	2.2	high	8.9	3.9	0.1	21	9.4
PS	60	F	21	1	23	4	150	1.32	0.82	127	1.8	high	8.6	6	0.25	28	11
PS	32	M	13	1	19.9	4.4	143	0.94	0.8	50.1	1.8	high	9.6	4.5	0.1		11.4
PS	64	F	23	1	17.4	4	167	1.34	1.26	122.2	1.8	high	8.7	7.4	0.41	24	12.4
PS	68	M	19	1	21.7	4.7	130	1.64	0.77	110.8	2.1	high	8.2	8.1	1.29	30	10.9
PS	68	F	10	1	17.6	4.1	167	1.68	0.83	125	2.5	high	10.5	5.4	0.37	37	13.1
PS	60	M	21	1	20	3.9	191	1.37	1.32	99	1.6	high	8.5	4.9		19	9.8
PS	67	F	30	1	20.5	4.1		1.73	1.08	113.6	1.8	high	9.4	4.9			11
PS	63	F	17	1	21.3	4.2	128	1.23	0.89	127.6	2.2	high	9.3	4.8	0.1	23	11.1
PS	62	M	19	1	17.4	4.4	138	1.09	0.93	117.9	1.6	high	9.9	10		38	8.9
PS	58	M	16	1	15.9	4.4	106	1.04	1.34	95.33	2.1	high	10.3	4.5	0.14	35	9.8
PS	55	M	4	1	24.1	3.4		0.87	0.69	126.9	1.8	high	7.4	5.1	0.15	19	9.1
PS	73	F	6	1	17.7	4	111	1.36	1.05	112.3	1.9	high		6.8		30	10.3
PS	58	M	9	1	17.7	4	156	1.26	0.92	123.2	1.3	high		6.1		29	9.7
PS	55	F	11	1	18	4.4	134	1.23	1.02	122.5	1.6	high		6.1		26	10.2
PS	64	M	12	1	19.3	3.8	148	1	1.02	128.3	2.2	high	8.1	5.4	0.1	20	10.8
PS	64	M	3	1	28.5	3.5	222	1.21	0.89	53.03	1.5	high		6.7	0.19	27	9.9
PS	71	M	14	1	17.4	4	131	1.24	1.05	120.1	1.9	high		6.2		29	9.8
PS	28	F	16	1	18.4	4.4	130	0.9	0.97		1.8	high		4.8	0.13	14	11.1
PS	55	M	8	1	22.2	4.1	180	1.42	1.06		2.1	high	10.2	7	0.06		12.4
PS	80	F	4	1	15.4	4.1	125	2.03	1.08		1.5	high	10.3	4.3	18.4	35	9.3
PS	43	F	11	1	18.9	3.9	175	1.49	1.09		1	high		7	0.27	19	9.8
PS	50	M	4	1	25.6	3.6	140	1.24	0.78		1.8	high	8.7	7.9	0.25	21	9.6
PS	56	M	2	1	34.8	3.9		1.86	0.9		1.5	high	7.1	5.2	0.52	25	7.8
PS	70	M	11	1	16.2	3.7	112	1.82	0.69		1.8	high	11.2	7.3	0.03	29	9.6
PS	43	M	3	1	33.3	4.2	151	1.81	1.05		1.8	high		5.9	0.11	31	10.6
PS	66	F	4	1	18.1	3.7	196	1.24	1.14		1.5	high	8.6	3.6	0		9.7
PS	59	F	12	1	16	4.1	165	1.29	1.18		1.6	high		6.5		25	10.3
PS	77	F	8	1	18.1	4.1	99	1.2	0.73		1.9	high		5.3	4.8	40	8.6
PS	61	M	8	1	18.9	3.7	117	1.26	1.05		2.1	high	9.1	6.2	0.03		12.1
PS	68	M	4	1	24.7	3.8	143	1.34	0.75		1.6	high	8.1	5.1		36	10.3
PS	43	M	2	1	26.9	4.1	119	1.34	0.91		1.5	high		4.4	0.21	35	9
PS	56	F	3	1	20.5	4		1.63	1.03		1.5	high	9.9	6.6	0.06	19	11.9
PS	60	M	11	1	20.8	3.9	207	2.14	0.74		1.8	high		5.8	0.05	25	10.8
PS	53	F	2	1	24.1	3.7	133	0.97	0.93		1.6	high	8.3	6.3		19	11
PS	74	F	13	1	22.3	3.6	140	1.5	0.7	96.37	1.8	high		7.1	0.1		11
PS	75	F	8	1	18.2		209	1.18	1.08	101.2	1.3	high	8.4	6.9	0.24		11.9
PS	58	F	3	1	17.3	3.9	134	1.16	0.93		1.5	high	9.1	5.9	0.19	22	10.5
PS	80	M	5	1	20.8	3.3	158	1.44	0.44	104.3	1.6	high	9.5	8.7	0.06	24	10.6
PS	60	M	5	1	22.1	4.1	89	1.49	0.85		1.5	high	8.5	2.8	0.15	29	12.7
PS	68	F	4	1	18.7	4.1	187	1.29	1.16		2.1	high		5.4	0.2	24	9.8
PS	74	F	29	1	16.5	2.6	191	1.6	0.83	78.54	1.8	high	9.6	5.8	0.05	23	10.7
PS	58	M	10	1	18.4	4.3	134	1.34	0.8		1.9	high		5.9	0	28	9.9
PS	59	F	27	1	16.8	3.5	126	1.46	0.92	55.44	2.1	high	8.5	5.5	0.45	38	

PS	68	M	13	1	19.3	2.9	210	2.11	0.84	89.73	1.8	high	10.8	6.3			11
PS	64	M	13	1	20.3	3.7	122	1.81	0.4	127.3	1.5	high	7.9	3.5		36	9.1
PS	73	F	12	1	20.4	3.5	110	1.54	0.73	115.2	1.3	high		8.9		23	9.8
PS	65	M	8	1	22.9	3.4	157	1.3	0.66	78.46	1.5	high	9.5	5.1	0.03	28	10.4
PS	77	F	5	1	25.8	3.3	121	1.46	0.89	109.1	1.8	high	9.1	4.9	0.3		9.6
PS	74	M	2	1	21.5	2.9	154	1.33	0.63	82.12	1.6	high		5.9	0.46	35	10.6
PS	84	M	9	1	21.9	3.4		0.79	0.74	129.6	1.4	high	9.2	6.1	0.01	20	10.8
PS	77	F	2	1	27.4	4	137	1.09	1.02	94.45	1.8	high	10.2	4.2	0.05	24	10.7
PS	70	M	17	1	20.4	3.2	162	1.74	0.69	69.26	1.8	high	8.7	3.9	0.1	17	10
PS	67	F	19	1	16.4	3.4	98	1.48	1.27	58.98	1.6	high	9.3	4		31	9.6
PS	78	F	3	1	18.7	3.8	133	1.11	0.76	85.03	1.9	high		5.7		28	10.2
PS	79	M	6	1	24.6	3.8	110	1.19	1.03	115	1.9	high		5.8		32	8.6
PS	85	F	2	1	17.4	3.5	146	1.05	1.25	134.7	1.6	high		6.8		21	10.4
PS	60	M	7	1	17	3.6	171	1.17	0.9	107.8	1.6	high		5.6		34	9.5
PS	55	M	9	1	22.1	3.8	136	1.33	0.97	108.6	1.8	high	10.2	7	0.06	26	8.6
PS	79	F	21	1	17.8	3.5	145	1.16	0.91	75.53	2.1	high	8.4	5.9	0.41	21	10.9
PS	63	M	7	1	20.2	3.9	90	1.16	0.82	113.7	1.6	high		6.4	0.65	39	9.5
PS	68	M	7	1	19.5	3.8	169	1.69	1.07	78.02	2.1	high	8.3	7	0.31	41	10.1
PS	70	M	4	1	22.5	4	108	1.38	0.62	101.9	1.8	high	9.4	9.9	0.1	30	9.6
PS	60	F	2	1	28.3	4	133	1.55	0.8	77.3	1.1	high	5.7	5.7	0.5	45	9.7
PS	59	M	2	1	18.6	3.9	165	1.46	0.68	83.8	1.5	high	9	5.6	0.05		11.7
PS	72	M	8	1	18.7	3.9	153	1.15	0.81	98.85	2.1	high		6.1	0.15	30	11
PS	67	M	34	1	18.9	3.5	196	1.36	1.1	73.3	1.8	high	8.9	5.3	1.53	18	9.2
PS	60	M	31	1	19.6	3.7	168	1.19	0.99	98.68	1.9	high		5.8	0.05	29	11.4
PS	56	M	30	1	18.9	4	126	1.06	1	75.11	1.6	high		8	0.3	31	11.1
PS	65	F	22	1	21.5	3.3	165	1.43	0.74	89.92	2.1	high		4	0.32	23	11.4
PS	57	F	20	1	16.1	3.7	116	1.14	0.98	96.2	1.4	high	8	6.5	2.2	26	8.8
PS	69	M	20	1	22.8	3.7	192	1.64	0.83	106.9	1.8	high	9.2	7.2	0.65	28	10.9
PS	67	M	15	1	25.2	3.4		1.91	0.85	149.1	1.4	high	9	3.8	0.03	18	11.4
PS	68	F	15	1	20.6	3.9	140	1.19	1.04	123.5	1.8	high		7.2		20	9.3
PS	75	M	13	1	22.3	3.6		1.84	0.9	123	1.4	high	9.6	4.7	0	23	12.2
PS	59	M	12	1	18.6	3.1	105	0.66	1.04	102.6	1.6	high		7.4		28	9.4
PS	74	F	12	1	17.5	4	132	1.07	1.05	115.1	1.8	high	8.4	8	0		10.8
PS	65	F	12	1	21.5	3.5	176	0.97	0.68	113.7	1.4	high	8.9	5.9	0.58	21	12.5
PS	62	M	12	1	23.1	3.5		1.58	0.99	116.9	1	high	8.8	3.2	0	21	11.1
PS	48	M	10	1	29.8	4	146	1.41	1.25	109.7	2.1	high		3.9	0.07	22	10.2
PS	50	M	10	1	22.6	3.9	125	1.32	1.06	123.4	1.6	high		5.8		30	11.1
PS	56	F	6	1	20	4	148	1.09	0.98	132.2	1.4	high	8	5.3	0.05	30	8.8
PS	66	M	9	1	22.4	4		2.12	0.75	140.6	1.8	high	9	4.5	0.51	31	10
PS	50	M	9	1	26.4	3.9	204	1.98	1.2	116.4	1.5	high	9.2	2.3	0.3	30	10
PS	75	M	9	1	22.9	3.7	123	1.36	0.79	133.2	2.1	high	9.5	6	0.31	46	10.8
PS	69	F	8	1	25.3	3.5	205	1.38	0.96	148.5	1.8	high	9.1	4.3	0.7	22	10.7
PS	68	M	8	1	22.3	3.8	124	1.33	0.9	112.2	1.5	high	8.3	4.8	0.03	21	15.1
PS	71	M	8	1	21.1	3.5	116	1.32	0.61	111.4	2.1	high	9	4.8	0.04	28	9.9
PS	60	M	7	1	21.2	4.1	108	1.42	0.91	161.8	1.8	high	9.6	4.7	1.2	32	10.3
PS	71	M	7	1	20.7	3.8		1.15	1.09	132.6	1.8	high	9.2	5	0.06	30	11.2
PS	59	M	7	1	19.5	3.8		1.76	1	134.3	1.8	high	8.7	4.7	0	28	11.1
PS	59	F	7	1	28.6	3.8	144	1.11	1.06	85.39	1.5	high		6.4	0.09	27	11.7
PS	29	F	6	1	20	4.2	121	0.98	0.99	131.7	1.8	high	9.7	3	0.46	31	12.4
PS	64	M	6	1	19.9	3.4	105	1.76	0.94	150.4	2.1	high	9.7	3.8	0.2	40	9.3
PS	68	M	6	1	24.1	3.7		1.68	0.9	112.2	1.8	high	9	5.6	0.03	30	11
PS	64	F	5	1	14.8	3.2	149	1.52	0.64	94.73	1.5	high	11	3.6	0.1	27	10.8
PS	52	F	4	1	20.7	4.3	109	1.45	0.81	128.8	1.5	high		7	2.96		12.1
PS	64	F	3	1	26.4	3.7	163	1.36	0.78	86.59	1.5	high	9.1	7	0.05	25	11.9
PS	55	M	3	1	38.6	4.3	112	1.31	0.79	86.17	2.1	high		5	0.05	25	9.8
PS	66	M	2	1	22.4	3.8	214	1.5	1.04	124.9	1.3	high		5	0.1	25	11.3
PS	73	M	2	1	25	3.7	143	1.76	0.84	101.5	0.8	high	9.5	5.2	0.22	29	9.6
PS	43	M	2	1	21.3	3.6	163	1.09	1.02	95.5	1.9	high		6.9		39	10.6
PS	58	M	2	1	21.2	3.1	199	2.01	0.7	76.43	2.1	high		6.3	0.19	21	11.8

PS	50	M	6	1	20.8	3.8	128	1.22	1.03	116.2	1.5	high		7	0.4	25	12.5
PS	57	M	28	1	23.1	3.7	88	1.17	1.03	112.3	1.6	high	9	4.7		30	11.1
PS	60	F	24	1	19.1	3.4	130	1.62	1.01	129.7	1.3	high	8.3	4.2	0.08	35	11.3
PS	52	M	21	1	24.7	4		1.78	0.97	136.2	1.6	high	8.1	3.6		22	10
PS	59	F	17	1	17.4	4	134	0.99	0.84	103.3	1.6	high	8.7	5.2	0.86	36	10.7
PS	68	M	16	1	22.7	3.8	175	1.08	0.91	121.8	1.6	high	9.5	5.7	0.05	42	11.9
PS	73	F	15	1	17.6	3.3	190	0.97	1.04	112.6	1	high	8.9	5.6	0.05	31	11.4
PS	59	F	14	1	17.4	3.7	152	1.35	0.95	98.65	1.6	high	8.8	5.8	0.32	31	12.2
PS	46	M	9	1	25.9	4	162	1.35	0.84	122.7	2.1	high	8.5	5.8	0.2		11.7
PS	65	M	7	1	19.1	3.2	173	1.3	0.81	96.13	1.5	high		6.8	0.3	21	11.5
PS	37	M	6	1	20.4	4.2	156	1.3	0.79	116.3	1.6	high	10.1	6.9	0.05	28	11.5
PS	69	M	5	1	26.7	3.7	143	1.82	0.84	107	2.1	high	9	7.1	0.05		10.3
PS	59	F	5	1	18	3.6	190	1.49	0.92	120.8	1.6	high	9.4	5.9	0.05	30	11.4
PS	54	M	5	1	18.7	4.6		1.41	1.29	119.3	1.3	high	8.8	5.2			
PS	57	F	3	1	25.6	3.5	145	1.3	0.92	113.6	1.5	high	9.1	6	0.04	21	10
PS	57	M	3	1	20.2	4	186	1.43	1.01	81.61	1.8	high		5.3	0.26	30	8.4
PS	61	M	3	1	21.5	4.1	198	1.11	0.99	86.12	1.6	high	8.8	7.5	0.05	27	10.7
PS	76	M	3	1	20.6	3.6	144	1.02	0.92	100.6	1.9	high	12.7	6.6	0.33	22	8.8
PS	61	M	2	1	24.7	3.6	184	2.1	0.81	100.6	1.6	high	8.3	4.6	0.05	25	8.9
PS	66	M	2	1	19.1	4.1	135	0.99	1.04	99.97	1.3	high	9	5.6	1.48	24	10.8
PS	50	M	2	1	25.5	4.2	116	1.31	0.88	117	1.6	high	9	3.9	0.05	26	10.8
PS	66	M	2	1	19.5	3.7	193	1.89	0.65	125.2	2	high	7	3.9	0.19	24	12.9
PS	79	M	4	1	19.9	3.7	141	1.48	1.14	96.18	1.9	high	8.5	4.4	0.05	14	12.3
PS	90	M	2	1	23	3.3	108	1.17	0.69	87.94	2.1	high	8.9	5		24	10.9
PS	71	M	4	1	22.5	3.8	147	1.45	0.65	56.71	2.1	high	8.3	6.4			13.1
PS	66	M	6	1	20.4	3.2	158	1.84	0.81	132.3	1.6	high	9.8	2.9	0.08	20	10.5
PS	57	F	5	1	16.7	3.7	141	1.33	0.7	106.3	1.6	high	8.7	5.4	0.15	25	11.5
PS	72	M	5	1	21.7	3.4	241	1.65	0.6	60.59	1.8	high		4.6	0.35	29	9.9
PS	46	M	15	1	20.4	3.8	221	1.38	0.83	117.8	1.6	high	8.5	7.2		26	11
PS	77	M	8	1	23	3.7	186	1.67	0.66	97.27	1.5	high		6.9	0.22	31	10.1
PS	61	M	5	1	20.7	3.7	128	1.68	0.99	125.2	1.5	high	7.8	3.8		24	11.1
PS	52	M	15	1	22.1	3.9		1.98	1.01	152.4	1.8	high	9.5	5.1	0.01	32	13.1
PS	52	M	3	1	25.6	3.9	187	1.17	0.82	147.6	2.1	high	9.8	5.5			
PS	57	F	4	1	22.2	3.9	244	1.92	0.69	55.89	1.5	high		6.9	0.28	25	11.1
PS	59	F	24	1	21.7	3.5	163	1.46	1.02	92.72	1.5	high	9.8	5.7	0.05		10.3
PS	39	F	15	1	21.8	3.5	203	1.61	0.89	105.4	1.5	high	9.4	6.2	0.34		12.1
PS	67	M	14	1	13.4	3.3	129	1.16	0.71	86.19	2.1	high	9.5	5.4	0.02		10.9
PS	60	F	12	1	18.4	3.7	179	1.16	1.08	126.7	1.5	high	9.4	5.1	0.04		10.5
PS	41	F	12	1	11.7	4	178	2.09	0.84	73.79	1.8	high	8.9	2.9		29	11.4
PS	64	F	7	1	18.3	3.2	172	1.31	0.94	123.3	1.8	high	9.4	6.9	0	31	11.3
PS	59	M	7	1	26.9	4.4	198	1.43	0.69	94.6	1.6	high	8.6	4.4		32	10.7
PS	63	M	7	1	23.4	4.4	157	1.65	0.77	95.24	2.1	high	10.2	4.2		29	11
PS	76	M	6	1	17.5	4.2	101	1.46	0.84	114.5	1.6	high	10.3	7.1	0.04	23	11.2
PS	74	M	4	1	17.1	3.2	170	2.06	0.84	67.02	1.8	high	9.5	4.9	0.08	30	9.2
PS	58	F	4	1	19.6	3.9	130	1.7	1.05	116.4	1.5	high	9.3	5.3			10.5
PS	84	F	5	1	23.8	3.3		1.33	0.62	149.5	0.8	high	9.8	4.3	0.05	15	10.4
PS	56	F	13	1	18	4.2	199	1.49	0.83	132.1	1.5	high		5.3	0.05		11
PS	78	M	2	1	23.4	3.7		1.88	0.98	123	1.3	high	9.3	4.7	0.09	26	9.5
PS	69	M	4	1	24.6	4	179	1.75	0.83	107.1	1.5	high		5.4	0.75	32	11
PS	60	M	17	1	19.3	3.8	190	1.83	1.02	100.2	1.6	high	8.1	6	0.05	25	9.2
PS	56	M	10	1	21.8	3.5	126	1.22	0.97	85.54	1.8	high		6.9		28	8.8
PS	70	M	8	1	23	4	214	1.91	0.92	102.3	1.6	high	9.4	6.5	0.05	22	9.9
PS	61	M	8	1	23.7	4	167	1	0.77	99.49	2.1	high	7.7	5.9	0.16	24	10.1
PS	66	M	6	1	18.9	3.6	164	1.57	0.63	95.63	1.9	high	9.6	4.7	0.05	21	11.2
PS	70	M	4	0	20.8	3.3	118	1.19	0.44	40.26	1.8	high	9.3	5	0.99	29	10.5
PS	58	M	2	1	23.1	4.2	145	1.37	0.59	32.59	2.1	high		5.6	0.13	28	9.7
PS	85	F	5	1	23.3	3.8	152	1.97	0.72	130.3	1.8	high		4.4	0.1	26	10.3
PS	48	M	16	1	17.2	4.2	170	1.82	1.18	101.6	1.3	high		8.2	0.11	34	11
PS	62	F	23	1	20.9	3.9	142	1.38	0.95	118	1.8	high		5	0.16		10.6

PS	73	M	19	1	20.8	3.9		1.22	1.16	118.1	1.8	high	9.3	5.1	8.44	35	10.4
PS	68	M	7	1	21.8	4	121	1.19	0.89	120.9	1.5	high	9.6	6.3	0.12	24	10.8
PS	67	F	7	1	19.9	4	122	1.25	0.87	112.8	1.3	high	8.4	3.4	0.4	24	12.1
PS	73	M	14	1	18.1	3.8	185	1.08	1.02	160	1.9	high	8.9	10.2	4.79		10.1
PS	81	F	8	1	14.7	4.1	166	1.48	0.93	107.5	1.6	high	8.6	4.6		33	10.5
PS	42	M	15	1	15.6	4.5	114	1.58	0.86	86.64	1.5	high	9.7	6.8	0.14	28	11.2
PS	61	F	3	1	26.6	3.6	209	1.36	0.73	81.47	1.1	high	8.8	7.6	0.1	54	9.7
PS	57	F	2	1	16.2	3.7	189	1.18	1.15	92.49	1.6	high	9.5	7.8	0.05	22	9.6
PS	59	M	22	1	18	3.6	207	1.42	0.94	97.29	2.1	high	9.9	8.9	0.05	27	10.7
PS	55	F	24	1	21.6	4.3	121	1.68	1.03	114.2	1.6	high	10.9	5.4		39	10.9
PS	75	F	16	1	19.5	3.8		1.48	1.32	91.63	1.8	high	8.5	7	0.03	26	11.5
PS	59	M	8	1	24.4	4	124	1.19	0.8	136.6	1.8	high	8.2	5.7		27	12.1
PS	64	M	22	1	18.7	4.3	145	1.33	1.01	115	1.9	high	9	6.7	0.5		7.9
PS	57	F	18	1	16.8	3.9	108	1.59	1.21	123.8	1.8	high	7.8	4.4	0.12	33	9.7
PS	72	F	16	1	19.4	4.2	131	1.33	0.82	107	2.1	high	8.5	4	0.18	22	10
PS	70	F	2	1	22.6	3.4	178	1.58	0.87	91.67	1.6	high	10.8	6	0.08		8.8
PS	51	F	18	1	21.4	4.3	164	1.45	1.44	83.42	1.6	high	8.7	6.9	0.35	28	9.3
PS	44	M	12	1	26.4	4.1		1.12	1.01	99.89	2.1	high	9.1	11	0.4	26	10.9
PS	61	M	11	1	23.7	4	96	1.46	1.05	109.2	1.3	high	7.9	6	0.06	19	9.6
PS	77	F	7	1	17.7	3.7	140	1.53	1.09	84.99	1.6	high	9.6	5.7	0.08	26	9.6
PS	81	F	4	1	17.6	3.9	265	1.39	0.83	73.93	1.8	high	7.9	9.1	0.1	32	10.6
PS	57	M	2	1	22.5	4.2		1.29	1.21	104.4	1.5	high	8.3	3.4		24	12.1
PS	64	M	33	1	20.2	3.6	239	2.21	0.68	74.16	1.8	high	8.8	4.6	0.83	20	10
PS	72	M	12	1	23.7	3.6	175	1.1	1.03	143.4	1.5	high	9.5	7.7	0.4	25	10.7
PS	69	F	7	1	23.6	3.8	171	1.97	1.17	125.4	1.5	high	8.9	3.6	0.52		10.1
PS	66	F	17	1	19	3.9	135	1.22	1.44	71.11	2.1	high	8.3	6.4		22	11.7
PS	50	M	18	1	21.3	4.2	153	1.79	0.99	121.7	1	high	8.3	6.3	0.1	29	10.3
PS	44	M	14	1	20.2	3.9	165	1.29	0.97	123.2	2.1	high	10	7.9		28	12.1
PS	84	M	4	1	21	3.9	171	1.21	1.12	132.6	1	high	9.9	6.1	0.14	20	9.4
PS	51	M	25	1	23.5	4.1	136	1.88	0.96	133.8	1.8	high	7.8	4.1	0.29	25	11.4
PS	68	F	23	1	22.7	3.6	140	1.48	0.89	99.63	2.1	high	8.7	4.9	0.1	23	10.5
PS	60	F	18	1	20.3	4.3	143	1.41	1.14	100.9	1.8	high	8.9	6		32	9.8
PS	58	F	22	1	20.8	3.8		1.5	0.96	113.9	1	high	9.1	4.9	0.1	22	12.2
PS	43	M	18	1	23.6	4.3		1.42	1.04	131.4	1.8	high	8.8	4.8	0.08	31	11.1
PS	68	F	4	1	20.6	4.2	151	1.44	0.66	94.82	1.5	high		6.3	0.07	30	11.2
PS	44	F	24	1	23.8	3.5		1.11	1.2	92.5	1.4	high	7.5	3.5	0.17	12	11.8
PS	57	M	24	1	18.7	4	133	1.37	0.79	88.03	1.5	high		4.7		21	7.4
PS	55	M	20	1	21.8	3.9	21	1.38	0.94	108.8	1.8	high	9.3	2.3		24	9.9
PS	68	F	18	1	16.2	3.7		1.6	1.03	85.85	1.3	high	9.9	5.2	0.08		11.2
PS	66	F	16	1	16.7	3.8	146	1.44	1.08	113.1	2.1	high	9.7	6.7		25	11.1
PS	73	M	9	1	22.8	4		1.55	0.95	105.8	1.8	high	9	3.3	0.48	27	10.9
PS	40	F	8	1	22.4	3.6	184	2.16	0.76	61.72	1.1	high	9.4	3.6	0.02	31	10.9
PS	57	F	7	1	32.5	4.1	125	1.4	1.01	109.7	1.8	high		4.6	0.09		8.4
PS	57	F	7	1	18.1	3.7	159	1.3	1.07	96.55	1.6	high	8.4	4.2		23	11.5
PS	52	M	4	1	23.4	3.1		2.11	1	104.2	1.8	high	8.2	5.4	0.06	32	11.9
PS	56	M	17	1	20.3	3.8	142	1.35	0.72	115.9	1.6	high	9.7	6.1	0.25	27	9.2
PS	81	M	13	1	19.1	3.6	209	1.26	0.7	113.6	2.1	high	9.3	6.4	0.16		11.6
PS	57	M	3	1	21.5	3.9	143	1.38	1.07	104.1	1.5	high	9.6	5.2	0.07	26	10.9
PS	76	F	2	1	19.2	3.9	61	1.19	1.07	135.1	2.5	high	9.4	7.6	0.2	22	11
PS	73	M	2	1	17.7	3.8		1.99	0.82	51.96	1	high	8.5	5.6	0.15	27	10.2
PS	48	M	18	1	19.8	4.3	172	1.49	1.08	136.5	1.3	high	8.5	2.8	0.54	25	11
PS	47	F	14	1	19.9	4	169	1.17	0.94	129.4	2.1	high	10.3	7.8	0.17		11.1
PS	39	F	11	1	22.5	4	187	1.35	1.17	110.3	1.6	high	8.6	5.1	0.07	14	8.9
PS	76	M	8	1	18.4	3.5	171	1.34	0.89	98.32	1.8	high	8.7	5.5	0.07	24	10.6
PS	81	F	6	1	17.4	4	151	1.14	1.03	102	2.5	high	8.9	4.7			10.3
PS	53	M	4	1	17.9	4	239	1.45	0.76	101.9	1.1	high	8	6.3	0.05	26	11.5
PS	62	M	3	1	18.5	3.5	176	1.34	0.86	143.2	2.1	high		5.5	0.04	30	11.6
PS	57	M	14	1	22.2	3.9	154	1.11	1.25	90.05	1.8	high	8.9	8.3	0.09	27	12
PS	60	M	4	1	23.3	3.7	134	1.56	1.4	146.5	2.1	high		8.1	0.01	28	12

PS	36	F	14	1	18.9	4.4	179	1.39	1.1	105.9	2.1	high	7.9	4.8	0.11	32	10.7
PS	71	F	22	1	20.8	4.4	199	1.29	1.08	106	2.1	high	8.6	4.8	0.49	27	9.9
PS	62	F	19	1	25.6	3.7		1.27	0.96	92.17	2.1	high	9.2	10.2	0.05	27	13.6
PS	76	M	11	1	20	3.2		1.6	0.91	114.5	1.6	high	9.3	5.4	4.4	18	10.7
PS	22	M	7	1	18.8	4.3	177	1.63	1.14	106.1	2.1	high	8.8	5	0.8	19	10.4
PS	55	F	4	1	19.6	4.1	122	1.02	0.7	85.04	2.1	high	9.2	6.4		25	10.7
PS	59	F	22	1	18.7	3.6	134	1.7	0.86	91.64	1.5	high	8.5	5.2	0.13	24	11.2
PS	44	M	4	1	24.7	3.4	133	1.63	0.54	82.31	1.3	high		9.7	0.02	32	8.6
PS	83	F	4	1	19.2	3.3	68	1.38	0.76	81.24	1.3	high	9.6	6.9	0.02	30	8.1
PS	58	F	22	1	20.8	3.8	170	1.12	1.23	124.8	2.1	high	8.4	4.2	0.55	24	12.4
PS	68	M	18	1	23	3.9	171	1.31	1.03	115.6	2.1	high	8.4	5.1	0.06	31	10.3
PS	43	M	16	1	19	4	132	1.29	0.73	115.3	1.3	high	8.7	3.7	0.29	22	9.8
PS	59	F	16	1	22.6	3.7	114	1.3	0.95	137.7	1.6	high	9.3	3.7	0.15	29	9.4
PS	58	M	12	1	17.9	4.2	177	1.49	1.21	111.1	1.6	high	8.8	4.7	1.45	27	10.7
PS	57	M	23	1	20.3	4.2	118	1.38	0.63	91.93	2.1	high	8.7	4.3		20	11.1
PS	62	F	10	1	18.7	3.9		1.46	0.93	116	1.8	high	9.3	4.4	0.22	25	11.4
PS	81	F	5	1	15.9	3.8	164	1.37	0.99	96.29	1.5	high	9.5	5.4	0.05	26	11
PS	45	F	26	1	19.5	3.9		1.99	0.93	97.94	1.4	high	8	3.6	0	26	9.8
PS	55	M	8	1	24.1	4	147	1.11	0.93	128.1	2.1	high	8.5	4.4	0.04	23	11.1
PS	69	M	3	1	21.9	3.8	137	1.2	0.87	92.98	1.6	high	7.9	5.7	0.09	26	10.7
PS	48	F	26	1	22.3	3.8	120	1.39	0.98	113.7	1.8	high	8.9	7.3	0.27	33	11.1
PS	55	M	22	1	19.5	3.7	109	1.76	0.84	109.4	1.8	high	8.6	5.2	0.72	20	8.6
PS	53	M	21	1	22.6	4.1	200	1.59	1.06	90.1	1.6	high	8.7	5.7	0.19	34	11.5
PS	66	M	21	1	19.2	3.9	177	1.91	0.88	96.7	1.1	high	8.8	4.7	0.6	27	10.4
PS	53	M	21	1	20.9	3.9		1.78	0.73	134.5	1.4	high	8.3	5.1	0.13	26	11.5
PS	49	F	17	1	20.2	4.2	115	1.37	0.96	113.2	2.1	high	10.4	4.6	0.26	25	8.9
PS	42	M	17	1	18.3	4.1		1.57	1.02	98.87	1.4	high	9.3	5.8			10.7
PS	52	M	12	1	21.3	4.6	118	1.65	0.95	122.4	1.5	high	10.1	2.9	0.07	28	10.5
PS	85	F	8	1	19.2	3.6	118	0.93	0.96	102.2	2.1	high		4.8	0	31	10.5
PS	59	F	4	1	28.7	4		1.29	0.86	125.5	1.8	high	9.2	6.6	0.33	36	12.2
PS	47	M	5	1	27.3	4	113	0.95	1.12	115.5	1.4	high	8.5	4	0.2	30	10.8
PS	56	F	3	1	23.5	4.2	150	1.61	0.9	116.8	1.5	high	10.2	6.3	0.81	29	9
PS	61	F	3	1	17.9	3.7	245	1.11	1.17	74.21	1.5	high	11.2	4.8	4.09	37	13.9
PS	68	M	2	1	23.5	4	166	1.67	1.05	109.8	1.8	high		5.8	0.44		11.3
PS	66	F	5	1	19.7	3.2	122	1.02	1.05	97.7	1.6	high	9.5	7.9	0.3	28	10.9
PS	62	F	3	1	25.8	3.5	165	1.35	0.79	89.21	1.3	high	8.9	5.7	0.05	21	9.9
PS	69	M	14	1	22.4	4.1	95	1.04	1.16	155.2	1	high	8.4	4.7	1.55	24	7.1
PS	35	M	11	1	16	3.9		1.74	0.69	114.1	1.6	high	10.1	5.6	0.03	31	10.6
PS	58	M	11	1	16.1	3.7	138	1.3	1.28	123.2	1.3	high	9.1	5.5	0.09	23	10.2
PS	59	F	11	1	23.3	3.8	194	1.66	0.87	139.2	1.6	high	7.5	5.4	0.11	38	10.9
PS	42	M	9	1	20.5	4.1	141	1.22	1.16	135.2	1.6	high	9	5.4	0.06	17	10.7
PS	56	F	6	1	22.7	3.8	134	1.19	0.98	83.59	1.6	high	8.6	5.7	0.02		12.9
PS	51	F	6	1	18.3	4		2.33	1.3	101.6	1.4	high	8.9	5.2	0.03	26	11.7
PS	42	F	6	1	24.4	3.8		1.6	0.86	94.02	1.8	high	7.7	3.2	4.8	30	11.4
PS	49	M	6	1	20.2	4	163	1.22	0.8	64.56	2.1	high	8.4	4.1		29	10.6
PS	69	M	6	1	23.1	3.6	112	1.24	1	117.4	1.5	high	10.2	6		34	11.2
PS	39	F	6	1	21.6	3.1	151	1.26	1.26	109.7	2.1	high	8.3	6.5	0.05		11.3
PS	74	F	6	1	20.7	3.4	135	1.32	1.13	136.2	1.8	high	8.9	3.9	0.12	30	9.9
PS	78	M	6	1	20.6	3.6	183	1.56	0.77	142.3	1.8	high		4.1	0.78	31	11.4
PS	45	F	5	1	16.2	4.6	159	1.37	1.28	131.9	1.8	high	9.2	4.6	0.05	33	9.5
PS	70	M	5	1	23.1	3.7	143	1.42	0.86	110.6	2.1	high	9.7	4.1	0.11	26	11.6
PS	78	F	4	1	21.9	3.9	241	1.27	1.29	145.4	2.1	high	9.2	4.3	0.05	27	12.8
PS	59	M	4	1	21.6	3.7	206	1.76	0.93	102.2	1.8	high	9.5	6.4	0.8	26	10.8
PS	53	M	3	1	20.4	3.2	168	1.94	0.85	120.3	1.8	high	8.6	4.3	0.3	26	9.1
PS	48	M	3	1	18.1	4.1	172	1.28	0.62	82.93	2.1	high		5.5	0.01	31	11.1
PS	57	F	2	1	22.8	3.4	142	1.1	0.52	136.3	1.5	high		4.8	0.48	25	10.9
PS	67	F	2	1	22.2	4	238	1.36	0.94	99.97	2.1	high	9.8	7.8	1.13	24	11.7
PS	39	F	2	1	22.2	3.7	115	0.92	0.73	100.8	2.1	high		5.7	0.85		10.5
PS	89	F	2	1	20.1	3.5	160	1.5	0.86	105.1	1.6	high	10	4.9	0.07	27	9.8

PS	36	M	23	1	18.8	3.2	197	0.82	0.8	128	1.6	high		7.1	0.1	24	13.4
PS	62	F	16	1	16.6	4.4	133	1.38	0.9	74.59	1.1	high	8.4	5.9	0.3	26	10.5
PS	61	M	11	1	21.6	4.5	102	1.2	0.76	114.8	1.5	high	10.8	6	0.5		10.9
PS	52	F	11	1	23.1	3.9	134	1.17	0.83	118.7	1	high	9	2.9	0.1	24	9.5
PS	63	M	11	1	18	4	137	0.83	0.88	115.6	1.5	high	9.3	5.7		28	9.9
PS	72	M	23	1	19.3	3.7	123	1.26	1.05	136.6	1.5	high		5.8	0.1	24	9.8
PS	61	F	5	1	22.7	3.8	131	1.18	1.22	95.39	1.8	high	9.6	5.2		28	9.7
PS	40	M	22	1	19	4.2	137	1.51	0.96	96.01	1.5	high	10	6.7	0.98	36	10.3
PS	58	M	8	1	23.7	3.6	198	1.4	0.99	95.07	1.8	high	8.5	4.7		14	11.9
PS	49	F	8	1	19.9	3.8		0.51	0.86	119.7	1.3	high	8.1	4.6	0.75		12.7
PS	58	M	5	1	25.3	4		1.44	0.78	102.1	1.8	high	8.7	8.4	0.21	25	11
PS	63	F	5	1	32.6	3.9	94	1	0.94	132.6	2.1	high		4.2	0.05	20	10.5
PS	64	M	5	1	19.9	4.1	142	1.07	0.72	93.82	1.5	high	8.3	6.9	0.12	31	11
PS	82	F	5	1	21.4	4	150	1.3	0.83	71.08	1.8	high	9.6	5.9	2	27	10.2
PS	47	M	5	1	18.6	4.4	111	1.35	0.94	96.56	1.5	high		4	1.89	28	10.8
PS	66	F	4	1	22.9	4.1	172	1.3	1.04	102.3	2.1	high	8.8	6.6	0.13		10.6
PS	83	M	3	1	23.8	3.8	101	1.45	0.86	116.2	1.8	high	8.8	4.2	0.06	35	10
PS	47	M	2	1	22	4.1	154	1.35	1.14	114	2.1	high	9.4	4.5	0.12	27	11.9
PS	65	F	3	1	15.5	3.7	144	1.49	0.92	58.33	2.1	high	9.1	6.1	0.01	25	10.6
PS	38	M	5	1	23.9	4.1	91	1.46	0.72	93.36	1.5	high	11	6.2	0.01	31	13.6
PS	64	M	4	1	24.8	3.4	120	2.15	0.57	62.01	1.3	high	8.2	3.6		16	11.1
PS	51	F	3	1	18.2	3.6	166	1.27	1.16	96.06	2.1	high	9.4	5	0.08	30	12
PS	57	F	2	1	17.7	3.4	162	1.64	1.08	102	1.8	high		5.2	0.03	19	11.2
PS	64	F	3	1	20.4	3.7	183	1.38	0.98	131.5	1.3	high	8.5	6.4	0.07	30	10.3
PS	55	M	2	1	21.1	4.1		0.98	1	113.8	1.8	high	9.5	6.4	0.1	29	12.5
PS	65	F	5	1	17.2	3.3		1.44	1.04	124.1	2.1	high	8.7	5	0.1	28	10.2
PS	72	M	6	1	22.3	3.7	157	1.52	0.83	141	1.5	high		7.2	0.81		10.2
PS	72	F	7	1	16.3	3.5	199	1.57	0.97	96.78	1.3	high		5	0.08	34	11.5
PS	66	M	7	1	22	3.6	216	1.48	0.83	108.5	2.1	high	9.6	6.5	0	28	10.1
PS	77	M	4	1	19.4	3.6	177	1.29	0.62	60.67	1.3	high		5.7	9.04	27	11.1
PS	65	F	7	1	20.5	3.6	104	1.4	0.9	65.5	2.1	high	8.9	3.5			10.7
PS	56	F	4	1	18.5	3.7	197	1.36	0.84	101.4	1.1	high	8.8	4.8		41	9.8
PS	60	F	3	1	21.1	3.4	119	1.63	1.03	101.2	2.1	high	8.9	4.6	0.2	27	8.8
PS	65	M	3	1	23.2	3.8	131	1.35	0.78	65.18	1.5	high	9.4	6.1	0.4	32	11.5
PS	47	F	26	1	18.8	3.5	138	0.98	1.65	100.4	1.5	high	8.4	6.6	0.05	30	10.2
PS	65	M	3	1	21.4	4	108	1.39	1	111.4	1.3	high	9.1	3.7	0.03	34	10.5
PS	40	M	5	1	32.5	4.3	143	1.09	0.71	102.8	1.5	high	9.4	4.1	0		9.6
PS	54	M	12	1	28.8	3.7	148	1.17	1	89.93	2.1	high		5.3		41	8.8
PS	67	M	2	1	22.5	4.2	134	1.4	0.85	98.17	1.5	high	8.4	6.6	0.29	42	11
PS	64	M	3	1	22.5	4.1	140	1.52	0.84	147.3	1.3	high	8.8	4.4	0.04	34	10.9
PS	55	F	29	1	19.1	3.7	227	1.01	1.17	130.8	1.9	high	8.9	5.1	0.16	28	9.9
PS	68	M	28	1	16	3.3	246	1.07	1.29	96.4	1.3	high	9.2	3.8	0.06	28	10.1
PS	66	M	24	1	22.2	3.6	129	1.1	1.18	124.4	1.9	high	9.5	6.1	0.01	22	11.2
PS	67	M	23	1	23.5	3.4	119	1.17	0.88	140.4	1.6	high	8.9	4.2	0.04	35	10.8
PS	82	F	19	1	16.5	3.5	316	0.71	0.78	99.47	1	high	8.4	5.1	0.08	14	9.8
PS	55	F	15	1	23.4	3.6		1.13	1.12	135.3	1.5	high	9.5	6.5	0.05	34	10.2
PS	65	F	10	1	21.2	3.9	152	1.31	1.04	125.4	2.1	high	9.2	5.6	0.17	26	7.2
PS	75	M	8	1	20.6	3.7	130	1.51	0.95	102.4	2.1	high		4.6	1.81	26	10
PS	76	M	7	1	23.1	3.9	154	1.1	1.18	112.1	1.6	high	9.5	5.8	0.41	21	12.1
PS	74	F	6	1	21.6	3.3	133	1.03	0.92	95.78	1.5	high		4.6	0.04	26	10.5
PS	64	M	6	1	17.8	3.7	175	1.12	1	109	2.1	high		5	0.57	28	10.3
PS	56	M	6	1	27.4	4	139	1.44	0.91	136.2	2.1	high		5.1	0.38	29	8.3
PS	61	F	6	1	29.8	3.4	144	1.8	0.91	128.1	1.3	high	8.9	6.7	0.2	22	10.4
PS	74	M	5	1	17.7	3.6	166	1.11	1.19	97.44	2.1	high		5.2	0.35	32	10.6
PS	64	M	4	1	23.6	3.6		2.27	1.03	104.4	1.8	high	9.7	5.8	0	28	10.5
PS	64	M	3	1	23.2	3.5	107	1.14	0.84	131.1	2.1	high	7	4.8	0.05		10.2
PS	52	F	6	1	21.7	3.5	123	1.88	0.85	114.3	1.1	high	8.1	6.4	0.1		8.6
PS	76	M	17	1	19.8	3.1	124	1.53	0.51	102	2.1	high		6.4	0.82	33	10.9
PS	58	F	19	1	25.8	3.9	116	1.64	0.83		1.6	high		4.9	0	27	9.5

PS	66	F	12	1	27.3	3.7	92	1.29	0.81		1.8	high	11	3.7		26	10.4
PS	69	M	12	1	24	3.6	188	1.31	0.68		2.1	high		5.6	0.02	30	11.7
PS	62	M	4	1	19.3	3.2	185	1.37	0.71		2.1	high	8.2	7.2	0.37	29	10.5
PS	67	M	26	1	20.9	3.7	137	1.12	1.11	120.8	2.2	high	10.6	5.6		27	10
PS	68	F	20	1	20.8		154	1.48	0.76	106.8	2	high	9.1	6.7	0.03	29	9.5
PS	78	M	4	1	19.3	3.7	217	0.91	0.83	122.7	1.3	high	8.3	4.9		24	9.8
PS	76	M	4	1	21.5	4	162	2.07	0.77	100.6	1	high	8.9	4.5		24	11.1
PS	44	M	7	1	25.5	4		1.19	0.99	108.7	2.5	high	9.7	5.6	0.3	27	10.7
PS	78	F	4	1	21	4	169	1.56	1.01	133.9	1.5	high	9.6	5.8	0.1	15	12
PS	59	M	10	1	21	4.1	97	0.96	0.73	90.97	2.1	high	9	6.7	0.1	29	12.9
PS	47	M	6	1	23.6	4.1	126	2.07	0.69	87.1	1.8	high	9.6	4.1	0.08	24	10.2
PS	75	M	5	1	17.4	4	149	2.18	0.9	121.4	1.8	high	9.9	5.4	0.08	26	9.1
PS	67	F	15	1	19.7	3.6	178	1.43	1.17	142.6	1.5	high	8.5	5.3	3.7	41	10.1
PS	69	F	4	1	19	3.6	154	1.14	1.05	123	2.1	high	9.3	4.7	0.02	27	11.5
PS	81	F	7	1	24.2	4	179	2.46	0.9	109.1	1.8	high	9.1	4.1	0.1	29	10.6
PS	72	F	2	1	23.8	3.7	134	1.38	0.72	117.2	1.6	high	8.7	5.7		29	10.3
PS	73	M	23	1	16.8	3.2	187	1.62	0.54	82.98	1.8	high	8.9	6.4	0.13	24	9.7
PS	67	F	23	1	20.6	3.8	179	1.25	1.12	99.79	1.8	high	8.8	7.9	1.3		10
PS	67	F	10	1	16.1	3.2	66	1.12	0.79	102.9	1.5	high	8.4	2.9		25	9.1
PS	57	M	4	1	27.9	3.8	235	1.6	1.01	123.4	1.5	high	9.8	5.9	0.07	22	10
PS	57	F	13	1	24.1	3.9	140	2.03	0.88	128.7	1.5	high	8.2	5.3	0.05	26	10.8
PS	77	F	9	1	24.6	3.8	121	1.07	0.97	135.5	1.3	high		5.5	0.3	29	10.8
PS	47	M	11	1	22.8	4	153	1.31	0.75	93.95	1.8	high		7.2	0.02	24	11.1
PS	60	M	6	1	19.2	3.7	124	1.12	1.02	91.59	2.1	high	7.7	3.8	0.18		10.7
PS	60	M	17	1	22.2	3.8	124	1.51	0.97	115.6	1.8	high	11.1	4.4	0.3		10.7
PS	50	F	12	1	24.6	3.8	197	1.3	0.84	122.2	2.1	high	8.8	4.8	0.18	31	11.8
PS	72	F	15	1	21.4	3.5	202	1.59	0.82	83.04	1	high	8.5	5.4		18	10.2
PS	45	F	12	1	19.5	1.3	178	1.76	0.94	131.5	1.6	high		4.2	1.1	31	11.3
PS	70	M	11	1	21.8	3.8	226	1.32	1.22	130.9	1.6	high	8.2	6.4		32	10.8
PS	42	M	8	1	22.9	4.1	146	1.45	0.91	89.25	1.8	high	7.7	6.6		30	12.1
PS	73	M	7	1	24.5	3.9	154	1.1	0.75	127.6	2.1	high	9.9	8.9	0.2	22	11.7
PS	57	M	4	1	23.9	4.1	113	1.3	1.07	107.6	2.1	high		5.5	1.27	42	10.5
PS	50	M	14	1	25.4	3.6	120	1.24	0.88	106.6	2.1	high	8.8	7.2	0.31	21	10.8
PS	47	F	15	1	16.3	4	164	1.36	0.91	98.43	1.2	high	9.6	4.9	0.1		11.4
PS	42	F	13	1	21.8	3.8	97	1.63	1.34	130.8	1.5	high		5.3	0.35	32	10.6
PS	57	M	13	1	19.5	4.1	193	1.07	1.02	111	1.9	high	7.5	6		28	10.4
PS	70	F	14	1	19.6	3.7	126	1.33	0.66	108.7	1	high	8.4	6		33	11.4
PS	78	F	9	1	15.9	3.7	226	1.67	1.15	92.87	1.5	high	8.5	4.4	0.05	17	9.8
PS	65	F	11	1	21.2	3.9	150	1.41	0.66	114.5	1.5	high	9.1	5.4	0.05	32	10.1
PS	79	F	7	1	20.5	3.7	151	1.85	1.03	126.5	1.8	high	8.8	6.2	0.1	40	10.5
PS	82	M	6	1	21.9	3.8	146	1.12	1.06	131.2	2.1	high	8.2	5.8	0.12	20	9.9
PS	63	M	6	1	16.3	4.2	147	1.59	1.07	109.7	1.5	high	8.8	1.9		25	9.8
PS	69	F	4	1	25.8	3.6	203	0.95	0.74	71.64	1.9	high	8.6	9.6		25	11.1
PS	68	F	4	1	18	3.8	173	1.79	0.85	149.5	1.5	high	8.4	4	0.08	22	8.3
PS	62	F	4	1	17.4	3.9	178	1.36	0.98	65.71	1.9	high	8.5	6.6		28	10.3
PS	73	F	8	1	18.4	3.9	186	1.39	0.92	121.9	2.1	high	7.7	7.5	0.15	27	10.8
PS	52	F	2	1	25.5	4.1	196	1.37	0.87	133.9	1.3	high	8.1	5.3		25	10
PS	44	F	2	1	22.4	4	121	1.39	0.85	97.05	1.8	high	10	7.9		30	9.1
PS	70	M	2	1	24.3	4	127	1.65	0.82	93.92	1.5	high	8.8	6.4	0.06	29	9.2
PS	70	M	2	1	18.6	4.2	136	1.87	1.04	84.08	1.5	high	9.3	5.6	0.04	29	10.6
PS	61	F	2	1	23.3	4	155	1.5	0.85	72.07	1.3	high	9.6	5.7		23	11.4
PS	60	M	4	1	20.8	4.4	117	1.38	1.15	133.4	1.8	high	8.2	4.4	2.26	27	13.1
PS	56	F	14	1	26.6	3.9	149	1.11	0.95	97.46	2.1	high	9.3	6.7		35	11.2
PS	69	M	15	1	22.1	4.2	149	1.6	0.82	99.15	1.9	high	9.2	5.4	5.1	34	9.8
PS	63	M	3	1	18.6	3.8	126	1.38	1.03	102.5	2.1	high	9	4.7	0.04	27	10.4
PS	62	F	7	1	27.8	3.7	179	1.81	1.07	105	1.8	high	8.8	6.5	0.1	25	9.3
PS	68	M	8	1	16.4	3.9	231	1.36	0.74	93.38	1.8	high	8.8	6.3	0.05	26	10.8
PS	36	M	14	1	19.2	4.1	155	1.21	0.74	96.17	1.5	high	10.1	8.6	0.13	24	11
PS	63	F	2	1	27.5	4.3	171	1.23	0.83	113.5	2.1	high	8.8	5.9		37	11.7

PS	72	M	2	1	22.1	3.8	158	0.98	0.84	47.49	2.1	high	9.5	8.2		32	9.3
PS	66	M	12	1	24	3.9	143	1.5	0.84	144	2.1	high	8.7	4.6	0.2	22	11.1
PS	65	M	28	1	20.8	3.6	145	1.23	0.76	93.87	1.9	high	10.3	4	0.03	31	12.1
PS	64	F	16	1	18.4	4.1	167	2.29	0.75	142.3	1.9	high	10	4.1		28	11.6
PS	59	F	3	1	23.4	3.8	113	1.23	1.06	125.3	1.5	high		3.4	0.04	48	9.8
PS	57	M	2	1	22.1	4.1	130	1.58	0.85	88.08	1.6	high	10.5	4.4	0.15	29	9.6
PS	61	M	8	1	20.9	2.2	172	1.41	0.66	120.8	1.5	high	8.3	4.2		18	7.4
PS	77	M	20	1	23.4	3.2	163	1.06	0.83	127.4	1.6	high	9.7	4.7	0.06	25	10.5
PS	78	M	5	1	22.5	3.5		1.15	0.98	95.76	2.1	high		5.7	0.08		9.5
PS	79	M	4	1	24.4	3.7	165	1.15	0.65	80.63	1.5	high		5.1	0.03	18	12.2
PS	70	M	2	1	23.6	3.7	125	1.3	0.86	69.63	1.6	high		4.5		22	10.1
PS	77	F	3	1	20.9	3.9	129	1.41	0.78	99.6	1.6	high	9.1	5.2	0.05	29	11.3
PS	60	M	5	1	21	3.7	179	1.52	0.68	83.32	2.1	high	8.3	7.1	9.3	21	11.4
PS	64	M	12	1	24.9	3.6	167	2.1	0.91	137.4	1.5	high	8.9	3.9	0.02	27	10.4
PS	56	F	21	1	18.3	4	169	1.26	0.81	97.44	1.9	high	9.8	7.1			11
PS	54	M	20	1	17.9	4.3	145	1.52	1.35	111.5	1.9	high	9.3	6.5			10.9
PS	59	F	19	1	19.4	3.7	142	1.2	1.12	127.1	1.8	high	9.2	7	0.3	26	10.5
PS	70	F	18	1	19.8	4	125	1.37	0.82	104.1	1.8	high		7.4	0.24	26	7.1
PS	45	M	16	1	20.4	3.6	100	1.23	0.74	121.9	1.5	high		1.7	0.16	27	10.7
PS	54	F	15	1	19	3.7	150	1.45	1.19	127.1	1.3	high		5.3	0.06	22	10.9
PS	57	F	13	1	19.8	4	225	1.68	0.98	136.2	1	high		7	0.05	31	9.3
PS	72	F	13	1	20.3	3.6	131	0.99	0.93	120.9	1	high	9.6	5	0.03	23	9.7
PS	54	F	12	1	18.4	4.2	177	1.31	1.02	108.8	1.8	high	8	5.9	0.6	38	11.3
PS	74	M	10	1	20.1	3.1	158	1.23	0.76	102	1	high	9.4	3.7	0.1	25	10.9
PS	39	M	10	1	18.4	4.3		1.11	0.83	94.23	1.1	high	8.3	7.3		24	8.5
PS	34	M	9	1	18.5	4.5	195	1.75	0.94	104	1.5	high	9.8	5.9	0.06	29	13.4
PS	76	M	9	1	22.6	3.7	148	1.37	1.11	133.9	1.6	high	9.6	5.7	0.03	28	8.7
PS	75	M	9	1	13.7	3.9	171	0.89	0.98	75.4	1.8	high	9.8	4.9	0.1	16	11.2
PS	56	M	8	1	20.4	3.3	170	1.21	0.94	125	1.9	high	11.2	4.9	0.02	28	11.7
PS	52	F	8	1	19.7	3.6	109	1.6	0.81	120.7	1.3	high		5.5	0.13	25	10.8
PS	49	M	8	1	25.8	3.8	132	1.14	1.09	143.1	2.1	high	7.6	6.9	0.59	29	9.2
PS	66	M	8	1	17.7	3.5	146	1.79	1	106.7	2.1	high		3.3	0.09	27	12
PS	45	F	8	1	17.6	4.5	129	1.65	1.07	134.5	1.5	high		5.9	0.04	26	9.8
PS	49	F	8	1	19.5	3.9	130	1	0.96	90.7	1.9	high	6.8	9	0.78	29	9.4
PS	55	M	8	1	23.7	3.8	193	2.21	0.88	132.8	1.6	high	9.6	4.3	0.02	21	9.8
PS	50	F	8	1	16.5	4.5		1.47	0.96	124.5	1.3	high	9	5.2	0.02	28	9.7
PS	35	M	8	1	20	4.3	102	1.72	0.87	98.13	1.8	high	9	6.4	0.3	26	8.9
PS	56	F	8	1	21.6	3.2		0.95	0.71	122.1	1.3	high	8.4	5.9	0.31	27	10.3
PS	61	F	7	1	17.3	3.8	187	1.11	1.08	122.4	1.8	high	8.6	4.5	0.1	10	11.3
PS	32	M	7	1	17.4	4.8	127	1.95	0.82	102.1	1.5	high	7	4.4	0.06		10.4
PS	69	M	7	1	23	4.2	187	1.27	0.95	114	0.6	high	9.2	4.9	0.2	24	10.5
PS	51	M	7	1	19.7	3.5	186	1.43	0.83	108.6	2.1	high	9.6	7.7	0.05		11.4
PS	57	M	6	1	24.1	3.6	138	1.16	0.98	126.6	2.1	high	8.9	4.1	0.53	30	9.6
PS	47	M	6	1	22.1	3.8	96	1.37	0.74	107.9	2.1	high		6.4	0.01	28	10.1
PS	38	M	5	1	20.6	4.1	180	1.19	0.92	97.93	2.1	high	10.1	7.7	0.08		11.8
PS	47	F	5	1	20.1	4	136	1.39	0.95	149.9	1.6	high	9.2	6.3	0.2	29	12.2
PS	45	F	5	1	26	3.6	173	1.65	0.86	66.42	1.8	high	8.8	5.8	0.06	24	8.3
PS	40	M	5	1	21.8	4.3	181	1.72	0.85	86.61	1	high	9	4.3	0.2	21	9.8
PS	62	M	4	1	20.7	4.1	145	1.46	0.77	109.6	1.5	high	8.3	2.3	8.62	28	6.4
PS	70	F	4	1	22.7	4.1	81	1.26	1.04	118	1.8	high	10.6	3.1	5.3	18	8.1
PS	55	M	4	1	20.8	4	198	1.16	0.92	104.5	1.8	high		6.5	0.1	34	11.3
PS	69	M	4	1	18.9	3.7	188	1.8	1.14	85.57	2.1	high		1.9	0.1	17	9.4
PS	62	F	4	1	16.8	3.8	140	1.53	0.94	76.01	1.8	high		6.2	0.16	24	10.2
PS	58	M	4	1	23.7	4	135	1.53	1.18	120.9	1.5	high	6.7	3.9	1.31	29	10
PS	72	M	4	1	25.4	3.8	183	1.22	1.31	125.8	1.8	high		6.4	0.46	24	10.2
PS	54	M	4	1	21.8	4.3	103	1.51	1.2	134.4	1.5	high	8.4	4.8	0.55	26	10.7
PS	78	M	3	1	16	3.8	173	1.59	0.92	109.9	2.1	high	8.4	5.4	1.54	28	9.6
PS	67	M	3	1	22.5	4	212	1.23	0.88	135	1.4	high	9.7	3.7	0.03	18	9.1
PS	58	M	3	1	21.2	3.6	151	1.62	0.8	106.5	2.1	high	9.6	6.1	1.02		11.6

PS	74	M	2	1	20.2	4.1	195	1.23	1.01	119.2	1.5	high	10.5	5.6	0.17	27	11.7
PS	49	M	2	1	25.2	4.6	218	1.59	0.69	55.45	1	high	10.5	5.8	0.2	28	10.4
PS	59	M	2	1	22.5	3.8	161	1.31	0.9	108.7	1.5	high	10.6	6.7	0.37	56	15.4
PS	54	M	2	1	20.8	4.1	192	1.27	0.87	97.55	1.1	high		4.2	0.42	37	10.8
PS	60	M	2	1	22.2	4.1	183	1.51	0.99	146.1	1.4	high		6.2	0.3	26	10.4
PS	41	M	2	1	28.8	4.2	147	1.62	1.04	103	1.5	high	9.2	4	0.1	19	9.5
PS	60	M	7	1	21	3.8	148	1.7	0.97	136.8	1.3	high	9.7	5.4	0.3	30	10.3
PS	70	M	2	1	19	3.7	140	1.64	1.02	105.7	1.5	high	9.3	3.7	0.73	20	10.8
PS	77	F	18	1	16.4	4	103	1.74	1.36	105.2	1.5	high	8.3	6.9	0.07	29	8.7
PS	60	F	18	1	21.8	3.9	178	1.77	1.19	77.13	1	high	9.5	4.6	0.1	22	11.6
PS	42	F	18	1	19.2	4.1	92	1.01	0.82	115.4	1.8	high	7.7	4.9	0.05	27	12.3
PS	63	F	15	1	17.5	4.2	135	0.87	0.97	101.2	1.9	high	7.4	10.1	0.47	23	9.5
PS	65	M	14	1	16.8	3.5	158	1.47	0.59	83.88	1.3	high		2.8	0.79	25	10.6
PS	58	M	13	1	18.6	4.3	175	1.56	0.89	112.6	1.5	high	8.8	7.8	0.05	25	11.2
PS	35	F	10	1	22.2	4.2	223	1.61	0.85	85.07	2.1	high	10.2	6	0.08	19	9.6
PS	78	M	10	1	23.3	4	183	1.16	0.96	121.4	1.8	high	7.7	4.5	0.11	32	12.9
PS	49	M	9	1	23.1	4.2	78	1.22	0.89	98.42	1.1	high	6.9	7.3	0.2	45	7.6
PS	65	F	9	1	18.7	4.1		1.01	0.99	114.3	1.5	high		5.7	0.01	35	9.5
PS	61	M	9	1	22.6	4.2	134	0.98	1.09	106.6	1.5	high	7.9	5.9	0.09	18	11.3
PS	76	M	9	1	21	3.9	220	1.42	0.65	99.65	2.1	high	8.6	3.6		28	11.3
PS	46	M	8	1	21.5	4.3	214	1.19	0.88	125.2	1.3	high	9.8	5.4	0.04	21	11.5
PS	44	M	8	1	21.8	4.4	192	1.36	1.18	99.17	2.1	high	9.3	4	2.04	32	11.3
PS	59	F	8	1	16.5	3.9	149	1.35	1.04	105.2	1.5	high	10.6	4.1	0.04	24	9.1
PS	59	M	7	1	22.8	3.9	104	1.73	0.79	97.76	1.8	high	9.7	5.3	0.04	25	12.3
PS	59	M	7	1	22.6	4	199	1.96	1.01	111.3	1.8	high	9.2	5.4	0.02	20	11.2
PS	43	M	6	1	15.4	4.4	212	1.88	0.95	103.4	2	high	9.1	7.3	0.09	31	10.4
PS	59	M	5	1	21.2	4.4	141	1.42	0.75	32.43	1.8	high	9.2	3.8	0.1	25	11.4
PS	56	M	5	1	21.5	4.4	127	1.14	1.18	121.1	2.1	high	9.6	3.1	0.28	25	10.7
PS	61	M	4	1	18.3	3.6	217	1.51	0.94	69.33	1.6	high	10.2	4.7	0.07	22	10.5
PS	61	M	13	1	20.1	4.1	200	1.15	0.88	126.8	1.9	high	9.1	5.3			14.7
PS	63	M	5	1	19.5	4.4	207	1.62	1.03	103.2	1.3	high	9.9	7	0.1	29	10.6
PS	58	M	3	1	19	3.6	146	1.31	0.82	32.14	1.5	high	7.5	3.3	1.7	33	9.8
PS	71	M	3	1	25.8	4.1	116	1.32	0.9	194	1.6	high	8.9	3.4	0.92	19	9.2
PS	66	F	19	1	19.3	3.5		1.06	0.92	107.5	1.3	high	9.8	4.3	0.12	23	10
PS	78	M	7	1	24.6	3.7	150	1.05	0.86	99.3	1.8	high	9.6	6.8	0.24	33	9.3
PS	73	M	5	1	19.9	3.3	132	1.34	0.81	138.7	1.8	high	8.3	5.6	2.99		9.6
PS	79	M	3	1	24.7	3.4	125	0.7	0.74	105.2	2.1	high	10.5	6.3	0.05	24	9.7
PS	81	M	3	1	18.9	3.5	137	1.35	0.82	60.87	1.6	high	7.8	3.9	0.37	25	10.6
PS	75	F	3	1	18.7	3.4	165	1.44	0.8	102.9	1.8	high	9.5	3.1	0.05	19	12.2
PS	75	M	2	1	19.9	3.6	156	1.32	0.69	128.2	1.8	high	7.2	5.9	0.07	28	13.1
PS	73	F	31	1	19.9	3.5	133	1.22	1	100.9	1.1	high	9.2	6.7	2.1		9
PS	71	M	8	1	20.9	3	161	1.19	0.84	106.6	2.1	high	10.1	4.9	0.12		9.1
PS	70	F	3	1	25.1	3.4	169	1.49	0.76	84.31	2.1	high	8.6	3.5	0.18	27	10.6
PS	69	F	13	1	18.2	3.1	152	1.02	0.62	100.4	2.1	high	7.6	4.2		26	9.9
PS	69	M	8	1	22.4	3.7	105	1.3	0.76	108.7	1.6	high		7.5	0.11	25	10.8
PS	68	F	33	1	19.5	3.3	161	1.48	1.11	112.7	1.5	high	10	5	0	25	11.9
PS	66	M	2	1	15.9	2.6	164	1.43	0.77	56.14	2.1	high	10.9	5.6	0.24	30	11.9
PS	64	M	34	1	21.8	3.2	150	1.22	0.98	87.24	2.1	high	10	5	4.5	35	11
PS	63	F	13	1	21.2	3.4	193	1.77	1.11	130.8	1.5	high	9.4	5		28	10.3
PS	62	F	19	1	19.4	3.3	198	1.71	1.01	116.5	1.8	high	8.8	4.3	1.84	42	12.8
PS	61	F	23	1	21.5	3.6		1.41	1.05	143.1	1.6	high	9.1	8.4		25	10.5
PS	60	M	29	1	17.5	2.8	141	1.27	0.64	61.76	1.6	high	8.1	5.4	0.03		10.2
PS	59	M	3	1	23.4	3.8	172	1.72	0.86	89.86	1.6	high	10.2	4.9	0.07	24	11.6
PS	58	M	5	1	22.1	4.3	133	1.5	1.23	104.5	2.1	high	10.7	7.6	0	25	11.9
PS	57	F	7	1	25.3	3.5	134	1.06	0.91	131.6	1.8	high	10	6.8		32	10.2
PS	55	F	19	1	20.4	3.2	126	1.12	0.85	110.2	1.4	high	9	5.7	3.62	31	8.5
PS	53	F	7	1	31.8	3.3	137	1.39	1	114	1.5	high	7.1	6.2	0.08	32	10.8
PS	51	F	16	1	18.1	3.6	87	1.45	0.94	61.81	1.3	high	9.4	3.2	0.04	22	10.5
PS	48	F	3	1	19.6	4.1		1.53	0.81	98.76	2.1	high	9.5	5	0.09		12.2

PS	45	F	5	1	18.7	3.8	148	1.27	0.89	120.5	1.9	high		7.2		36	10.7
PS	41	M	4	1	30.1	4	173	1.52	0.99	95.29	1.8	high	8.2	3.6	0.9	15	9.4
PS	34	F	5	1	22.9	3.7	170	1.71	0.86	135.3	2.1	high	9.6	6.5		24	10
PS	66	F	20	1	18.1	1.8	206	1.47	0.85	112.6	1.6	high	10.5	8.6	1.95		10.4
PS	55	M	15	1	18.5	3.2	143	1.68	0.72	63.79	1.6	high		4.1	0.11	25	10.8
PS	67	M	14	1	22.7	4.1	105	1.34	0.67	124.9	1.5	high	9.5	3.7		32	10.8
PS	52	M	11	1	19.9	4.5	185	1.53	0.85	102.2	2.1	high	9.5	3.7	0.06	25	11.2
PS	48	M	11	1	19.3	4.1		1.75	1.07	102.8	1.6	high		5.1	0.04	26	10.8
PS	53	F	10	1	16.6	3.7	204	1.59	0.97	95.44	1.8	high	8.3	7.5	0.12	31	10.8
PS	57	F	10	1	19.7	4		1.57	1.01	121.6	1.6	high		4.8	0.39	25	10.8
PS	66	M	10	1	18.4	3.3	148	1.53	0.62	106.9	1.6	high	9	5.2		24	10
PS	74	F	10	1	19.4	4	135	1.36	1.01	146.1	1.8	high	7.3	5	0.1	21	9.4
PS	56	M	9	1	23.5	4.3		0.85	0.81	109.7	1.6	high		5	0.02	16	10
PS	68	M	9	1	20.6	4.2	119	1.58	0.9	112.1	1.4	high	9.1	4.8	0.13	28	9.2
PS	51	M	8	1	23.2	3.6	196	1.54	0.85	108.4	1.5	high	9.6	5.3			11.1
PS	62	F	8	1	21.7	3.9	228	1.8	1.15	106.6	1.5	high	9.6	5	0.05		10.4
PS	67	M	7	1	20	3.2	113	1.37	1.03	113.9	1.6	high	8.3	4	0.84	29	9.4
PS	65	F	6	1	20.7	3.8	146	1.57	0.95	129.9	1.5	high		3.4	0.05	19	10.4
PS	71	M	6	1	21.2	3.8	122	1.31	0.64	110.9	2.1	high		4.7	0.27	22	11.5
PS	55	F	6	1	24.2	4	172	1.75	0.86	114.2	1.8	high	10.1	4.6	0.3	25	10
PS	61	M	5	1	23.9	3.9	179	1.25	0.86	121.6	1.6	high		4.4	0.23	38	10.2
PS	50	F	5	1	21.1	4.1	144	1.32	0.52	62.15	1.5	high	8.2	4.2	2.08	23	8.6
PS	61	F	5	1	20.4	3.9		1.75	0.92	108.8	1.6	high		2.8	0.53	29	10.3
PS	54	F	4	1	20.2	4.3	243	1.54	1.01	122.6	2.1	high	7.6	3.9	7.73		8.7
PS	47	M	3	1	30.1	4.1	159	1.13	0.9	92.82	1.8	high		7	0.01	20	11
PS	71	F	2	1	21.5	3.8	166	1.83	0.91	161.1	1.3	high	8.4	4.9	0.05	18	10.4
PS	58	F	2	1	21.2	3.7	128	1.43	0.96	116.4	1.5	high	8.2	5.2	0.25		10.3
PS	61	M	2	1	25.1	4	203	1.39	0.69	119.1	2.1	high		3.6	0.1	24	9.7
PS	80	M	8	1	17.8	3.1	116	1.74	0.84	88.33	1.8	high	9.1	6.1			9.2
PS	63	M	4	1	26.8	4.2	138	1.51	1.13	112.4	1.5	high	6.8	4.7	1.97	29	10.5
PS	75	F	14	1	16.2	3.5	148	1.7	1.22	122.7	2	high	9.5	5.3		25	8.7
PS	82	M	3	1	21	3.9		1.44	0.72	110.6	1.6	high		3.9	0.03	28	10.5
PS	74	M	18	1	22.6	4		0.96	0.78	119.4	1.6	high		4.3	0.06	23	10.6
PS	75	F	7	1	20.8	3.6	116	1.37		42.86	2.1	high	10.2	5.3	0.1		7.8
PS	70	F	7	1	17	3.6	177	1.11	0.86	100.4	1.5	high	8.6	4.6	0.05	21	10.3
PS	73	M	7	1	23.2	4	138	1.66	0.83	124.1	1.3	high	8.7	6.8	0.05	27	9.9
PS	75	M	4	1	24.5	3.9	128	1.13	0.85	133	1.3	high	8.1	3.6	0.05	29	11.7
PS	83	F	4	1	22.7	3.9	152	1.24	0.73	106.1	1.6	high	8.9	3.9		26	10.7
PS	55	F	27	1	22.9	3.9	187	1.24	1.28	101.3	1.9	high	8.1	6.4	0.08	27	11.2
PS	48	F	19	1	25	3.9	131	1.2	0.94	114.3	1.9	high	8.5	2.9	0.15	27	8.7
PS	72	M	4	1	17.8	3.1	191	1.6	0.61	68.5	1.5	high	9.7	4.2		35	8.6
PS	65	M	3	1	23	4.1	173	1.97	1.2	108.1	1.5	high		6.1	0.1	27	9.8
PS	60	M	2	1	23.3	3.6	112	1.52	0.75	111.5	2.1	high	11	6.2	0.04	34	9
PS	54	F	2	1	27.1	3.9		1.56	0.71	79.39	1.8	high	9.8	4.7		30	9.2
PS	71	M	2	1	20.4	3.8	225	1.57	0.97	119.2	1.8	high	8.1	5.3	0.2	25	8.5
PS	80	F	4	1	25	3.5	143	1.68	0.92	90.02	1.8	high		4.7		25	11.2
PS	59	F	3	1	32.8	3.4	113	1.16	1.17	75.69	1.6	high	9.1	6.8	2.8	34	11.5
PS	58	M	32	1	20.1	3.6	140	1.22	1.01	90.86	2.1	high	8.8	8.2	0.1	23	11.7
PS	78	M	10	1	21.1	3.5	148	1.43	0.87	105.1	1.5	high		6.4	0.21	37	14.9
PS	56	F	7	1	17.4	3.9	122	1.21	0.81	118.9	1.6	high	10.1	8.3	0.1	27	11
PS	61	F	4	1	25.5	4	155	1.24	0.74	96.68	1.3	high	10.2	4.2		25	11.7
PS	65	M	7	1	18.7	4.1	109	1.32	0.83	110.7	1.8	high		5.4	5.2	36	10.8
PS	79	M	10	1	18.8	3.7		1.3	0.64	91.42	2.1	high	9.7	6.4		32	11.5
PS	65	F	14	1	15.9	3.2	179	1.6	1.05	102.2	1.8	high	8.8	2.9	0.1	25	11.6
PS	81	M	10	1	17.9	3.4	180	2.4	0.92	110	1.3	high	9.5	3.9	0.05	22	9.6
PS	50	M	7	1	28.9	4.4	101	1.07	0.78	105.5	2.1	high	8.9	4.7	0.01	32	7.8
PS	50	M	2	1	24.6	3.5	154	0.82	0.81	104.1	2.1	high	9.6	5.3	0.21	15	11.6
PS	52	M	2	1	23.2	4.2	203	1.68	0.86	94.48	1.8	high	9.9	7.1	0.23	26	10.7
PS	45	M	26	1	20.1	3.8	173	1.42	0.78	107.8	1.8	high	9	4.8		37	

PS	71	M	18	1	17.3	3.2	127	1.22	1	131.6	1.2	high	8.8	4.3	0.2		12.4
PS	59	F	17	1	19.3	3.4	182	1.52	1.02	113.4	2.1	high		4.1	0.01		9.9
PS	77	M	15	1	18.5	3.1	145	1.54	0.76	81.28	1.8	high	9.8	3.1	0.07	18	9.9
PS	59	M	15	1	18.3	3.3	232	1.53	0.86	109.5	1.8	high	9	3.9	0.11	24	9.9
PS	44	F	14	1	19.8	3.6	156	1.44	0.61	98.97	1.8	high		6	1.64		11.3
PS	60	F	12	1	18.3	3.5	183	1.53	0.86	133	1.8	high		5.3	0.67		10.5
PS	56	F	11	1	20.8	3.4	178	1.43	1	150.8	2.1	high		6.1	1.34		11.5
PS	70	F	10	1	21.5	3.4	147	1.52	0.7	102.9	1.5	high		5.3	0.17	26	11
PS	70	F	10	1	23.9	3.5	101	0.83	0.91	89.67	2.1	high	8.6	6	0.25	29	10.8
PS	39	F	10	1	16.5	4	126	1.53	0.84	68.73	1.6	high	8.9	5.3	0.05	31	11
PS	75	F	8	1	16	4	174	1.49	1.09	118.6	2.1	high		5	0.04		10.8
PS	70	F	8	1	21.5	3.8	148	1.35	1	113.7	1.5	high	8.4	8.8	0.05	27	11.3
PS	70	F	7	1	23.6	3.6	129	1.2	1.02	98.61	2.1	high		7.1	0.02		10.7
PS	79	M	7	1	21	3.3	138	1.34	0.81	106.9	2.1	high		6.8	0.11		10.7
PS	61	M	7	1	23.6	3.5	163	1.72	0.84	118	1.2	high	8.9	4.1	0.3		9.7
PS	80	M	6	1	20.1	3.7	132	1.14	0.99	100.4	2.1	high		4.6	0.76		12.1
PS	67	M	5	1	20	2.9	138	1.79	1.13	86.55	1.5	high	9	5.3	0	35	11.8
PS	55	M	5	1	15.8	3.8	157	1.34	1.04	85.21	1.3	high	9	7.2	0.36	29	10.9
PS	65	M	5	1	25.6	3.3	128	1.63	0.58	84.04	1.5	high		6.3	0	49	10.3
PS	89	M	5	1	20.8	3	126	1.34	0.62	94.94	1.5	high		2.9	0.2	19	12.5
PS	72	M	4	1	19.4	3.5	146	1.19	0.93	107	1.5	high		5.6	0.19	33	11.2
PS	70	M	4	1	26.6	3.4	126	1.29	0.71	121.6	1.2	high	8.5	3.9	0.1		10.8
PS	65	M	4	1	20.2	3.5		1.28	0.84	100.8	1.3	high	8.7	3.9	0.6	37	10.5
PS	71	M	4	1	21.3	4.3	155	1.23	0.7	93.56	1.8	high	8.3	8.1	5.38	20	9.3
PS	60	F	4	1	23	3.6	165	1.63	0.93	87.76	2.1	high	9.3	6.7	0.14		9.4
PS	59	M	3	1	21.8	3.4	123	0.86	0.98	130.1	1.3	high	7.3	3.8	8.12		10.6
PS	65	M	3	1	25.1	3.9	143	1.03	0.86	111.1	1.8	high	9.3	5.8	0.16	28	10.9
PS	65	M	3	1	22.6	3.3	123	1.58	0.65	80.39	1.1	high	9.6	5.5	5.56	32	7.9
PS	52	M	3	1	29.5	3.6	139	1.39	0.64	65.92	1.6	high	9.7	7	0.2	25	10
PS	57	M	3	1	20.8	3.5	189	1.65	0.78	80.33	2.1	high		4.8	0.06		10.1
PS	70	M	2	1	26.1	3.4	116	1.82	0.65	100.2	1.1	high		8.5	0.03	29	11.9
PS	50	M	9	1	20.4	3.8	116	1.46	1.09	112.5	2.1	high	9.1	5.4	0.12		11.1
PS	53	M	8	1	16.1	3.7	189	1.75	0.95	76.66	1.6	high	9.5	5.4	0.2	28	7.4
PS	37	M	22	1	17.5	3.8	120	1.04	1.03	127.9	1.4	high	8.9	6		44	
PS	62	M	17	1	17.1	3.5	137	1.41	1.06	119.2	2.1	high		6.7	0.04	23	10.7
PS	78	M	16	1	22.6	3.6	131	1.55	1	130	2.1	high	9.3	3.9	0.11	30	9.2
PS	65	M	7	1	19.9	3.8	180	1.61	0.91	127.3	2.1	high	9.4	4.8	0.53	26	11.7
PS	58	F	4	1	11.8	2.7	172	1.67	0.98	45.91	2.1	high	9.6	4.4		23	10
PS	54	M	26	1	21.8	3.4	163	1.58	1.11	82.72	1.3	high	10.6	3.8	0	28	11.7
PS	74	M	9	1	24.4	3.9	198	1.72	0.75	165	1.3	high	9.7	4.4	0.2	26	8.4
PS	75	F	6	1	19.5	4.1		1.4	1.07	87.96	1.8	high	8.6	3.9		29	9
PS	86	M	5	1	22.9	4.1	142	1.2	1.13	124.7	1.8	high	10.2	5.9	0.05	30	12.1
PS	67	M	4	1	26.4	4.5	164	1.49	0.83	84.13	1.1	high	10.7	8.2	0.1	27	12.2
PS	59	M	3	1	21.5	4.1	106	1.17	0.99	91.42	1.8	high	8.7	4.6	1.4	33	10.2
PS	74	M	2	1	19.5	4	133	1.61	0.73	73.48	2.1	high	12.2	6.4	0.17		10.6
PS	69	M	2	1	30.1	3.5	197	1.92	0.73	93.77	1.5	high	6.4	3			9.6
PS	51	M	13	1	18.1	3.9	140	0.99	0.78	120.2	1.3	high	8.9	9.3	0.05	19	12.4
PS	55	M	12	1	20.4	4.1	139	1.46	0.98	114.6	1.6	high	8.9	7	0.2	25	10
PS	62	M	10	1	17.3	4	151	1.8	0.96	126.2	2.1	high	9	7.8	0.01	27	12.3
PS	68	F	8	1	20.7	3.8		1.49	1.37	107.2	1.8	high		5.9	0.35	36	10
PS	40	F	6	1	19.4	4.2	157	1	0.82	126.2	1	high	10.5	3.8	0.1	30	8.3
PS	70	M	5	1	22	4.1	198	1.21	0.8	36.77	1	high	10	6.1	0.2	27	10.3
PS	50	F	4	1	21.5	4.6	138	1.07	0.87	115.6	1.6	high	9.2	6.7	0.1	26	9
PS	68	F	16	1	19.6	3.3	213	1.19	1.05	130.8	1.5	high	8.7	4	0.2	25	11.1
PS	75	M	8	1	21.8	4.1		1.31	1	97.94	1.8	high	8.6	5.7	0.14	28	10.4
PS	60	F	8	1	23.5	3.7	179	1.19	0.96	131.6	1.5	high	9.9	5.3	0.2	40	12.7
PS	57	M	6	1	25.1	3.8	161	1.41	1.02	138.1	2.1	high	8.6	4.8	0.08	32	12.4
PS	62	M	4	1	24.8	3.7	101	1.11	1.02	80.85	1.5	high	8.9	3.2	0.58	38	9.4
PS	90	F	4	1	16.8	3.7	157	2.08	0.94	69.93	1.6	high	9.3	5.1	0.05	21	11

PS	70	F	11	1	19.6	4.1	124	1.32	1.09	126.7	2.1	high	9.9	6.1	0.5	27	12.3
PS	54	M	9	1	21.8	3.8	135	1.24	0.95	129.2	1.6	high	8.4	3.7	3.04	32	9.1
PS	64	M	7	1	23.4	3.9	144	1.08	1	125.8	1.5	high		5.7	0	17	10.2
PS	76	M	3	1	21.3	4.2	149	1.74	0.78	117.8	1.1	high	8.6	5.3	0.18	27	10
PS	44	M	12	1	22.9	4.5	211	1.81	0.91	92.99	2.1	high	9.7	4.2		17	9
PS	84	F	10	1	19	3.8	107	1.49	1.08	106.7	1.4	high	9.1	4.1	0.1	30	9.4
PS	55	M	13	1	25.4	4.2		2.11	0.99	109.7	1.8	high	9.7	7.2		23	10.1
PS	58	M	8	1	22.8	3.9	111	1.99	0.95	135.1	1.3	high	9.2	5.2		22	10.5
PS	55	F	2	1	21.7	3.9	168	1.33	0.74	94.12	2.1	high	9.6	6.8		26	9.2
PS	49	M	27	1	21.6	4.1	178	1.58	1.02	106.2	2.1	high	8.9	4.2	0.09	25	10.5
PS	72	F	2	1	25.7	3.8	197	1.29	0.75	86.61	2.1	high	9.6	6	2.86	33	10
PS	45	F	4	1	26	3.7	191	1.56	0.88	109	1.3	high	9.1	1.7	8.48	29	8.5
PS	54	M	3	1	15.9	4.2	113	1.34	0.61	43.73	1.5	high		5.9	1.1		12.4
PS	55	F	32	1	17.5	3.7	154	1.8	1.24	109.3	2.1	high	9.3	6.7	0.1	36	14.6
PS	49	F	32	1	21.1	3.7	161	1.59	1.09	96.78	1.8	high	10.2	3.9	0.1	31	9.7
PS	67	F	31	1	23.4	3.9	189	1.41	0.98	102	1.3	high	8.5	3.3	0.36	28	9.4
PS	70	F	24	1	23.5	3	170	1.56	1.05	95.61	1.9	high	9.8	7.2	0.05	29	10.8
PS	60	M	16	1	21.3	3.7		1.29	1.11	127.9	2.1	high		9.6	0.13	26	12.1
PS	53	F	15	1	15.2	4.2	192	1.4	1.09	108.3	1.5	high	8.8	6.1	0.3	24	10.8
PS	50	M	4	1	18.3	3.4		1.31	0.6	70.98	1.3	high		6	0.04	26	10.9
PS	68	F	14	1	18.2	3.8		1.05	1.26	91.52	1.5	high		5.2	0.02	18	9.1
PS	65	M	14	1	21.8	3.4	101	1.29	0.72	129	1.8	high	9.9	6.5	0.3	38	13.9
PS	55	M	24	1	18.5	3.6	137	1.53	1.01	94.61	2.1	high	9	5.7	0.1	18	11.5
PS	56	F	13	1	20	4	117	1.07	1.16	104.5	1.6	high	7.9	3.9	0.03	30	11
PS	64	F	13	1	19.6	3.8	163	1.36	1.06	101.4	1.5	high	9.2	7.5	0		9.3
PS	74	M	13	1	20.9	3.9	186	1.64	0.79	133.1	1.6	high	8.4	5.2	0.03	30	10.6
PS	63	M	12	1	21.7	3.8	237	1.34	1.13	123.1	1.5	high	9.5	5.5	0.05	29	9.7
PS	43	M	16	1	27.2	4.4	111	1.03	1	136.4	1.6	high	7.9	5.9	0.05	23	9.6
PS	60	M	10	1	24.1	3.8		1.23	0.98	115.8	2.1	high	9.8	7.5		31	10.4
PS	61	F	8	1	19.6	3.5	192	1.71	0.99	119.5	1.5	high	8.6	4.4	0.71	29	10.2
PS	69	M	8	1	26.2	3.8	88	1.68	1.19	105.2	1.8	high	7.7	4.7		30	5.8
PS	53	M	8	1	22.7	3.9		1.78	1.12	88.26	1.8	high	9.9	7.3		28	10
PS	64	F	7	1	20.9	3.6	137	1.62	1.01	121.7	1.8	high	9.2	5.8	0.63	30	10.3
PS	65	F	7	1	18.8	3.7	123	1.27	1.42	96.83	1.8	high	7.9	6	0.3	31	8
PS	41	M	7	1	25.4	4	197	1.24	0.94	102.4	1.3	high	9.3	5.6		35	9.9
PS	51	M	6	1	21.5	4	193	1.56	1.01	108.7	1.3	high		6	0.34	28	10.1
PS	59	F	6	1	19.5	4		1.32	1.13	61.56	2.1	high	9.3	3.6		26	8.9
PS	65	M	5	1	22.4	3.7	98	1.35	0.79	101.9	2.1	high	9.4	8.8	0.02	52	10.8
PS	42	M	5	1	18.8	4.4		1.51	0.62	72.23	2.1	high		3.8	0.01	21	12.3
PS	63	M	5	1	22	3.7		1.11	0.85	97.02	2.1	high	9.1	5.9		25	9
PS	60	F	5	1	22.7	3.9		1.62	1.28	104.5	1.6	high	10.3	5.3	0.1	24	11.1
PS	67	M	5	1	20.8	3.7	170		0.72	110.2	1.6	high	9.5	10.3	1.6	26	8
PS	43	M	4	1	25.7	3.9	136	1.07	0.8	72.24	1.3	high	10	6.5	0.11	30	12.3
PS	71	M	4	1	30.3	3.4	78	1.24	0.8	101.9	2.1	high	11.4	6	0.1	30	10.2
PS	62	F	4	1	18.4	3.5	189	1.3	0.84	114.5	2.1	high		4.4	0.47		10.8
PS	54	M	3	1	23.7	4		1.48	0.85	103.6	1.8	high	8.6	3.3	0.18	33	11
PS	56	M	2	1	21.4	3.8	172	1.85	0.8	113.1	2.1	high		4.5		27	19.6
PS	70	F	2	1	20.1	3.5	191	1.24	0.86	140.7	1.8	high		4.1	0.04	28	12.3
PS	34	M	2	1	20.1	3.6	174	1.61	1.26	93.65	2.1	high	10.4	5.4	0.06	22	9.5
PS	60	M	2	1	24.2	3.6	135	1.44	0.69	68.43	1.3	high	12	5.6	0.02	27	10.6
PS	43	M	11	1	19.7	4.1	123	1.57	1.25	108.8	1.8	high	8.1	3.3	0.1	27	9.7
PS	84	M	2	1	19.1	3.5		1.1	0.68	82.07	2.1	high	8.5	4.4		26	9.9
PS	75	F	17	1	18.3	3.8	123	1.21	0.98	117.7	1.5	high	10	6.3			10
PS	63	M	16	1	19.5	4	226	1.25	0.84	122.6	1.3	high	8.6	3.1	0.69	13	10.9
PS	58	F	27	1	17.9	3.8	153	0.68	1.05	123.8	1.3	high	8.5	4.8	2.55	18	9.5
PS	61	F	14	1	15.5	4	150	1.26	1.27	109.4	1.6	high	9.4	6.3	0.08	28	11
PS	60	M	14	1	26.7	4		1.53	0.88	99.95	1.8	high	8.8	4.9		29	11.9
PS	38	F	13	1	19	4		1.48	0.73	123.7	1.3	high	9.1	4.2	0.3	15	11.1
PS	75	F	13	1	16.2	3.5	120	1.53	0.93	125.2	1.3	high	9	5.1	0.3		10.8

PS	62	M	12	1	20.8	3.9	94	1.44	1.25	145.8	1.6	high		7.1	0.24		10.4
PS	57	F	12	1	16.1	4.1	175	1.96	1.03	130.9	1	high	5.8	5.7	0.2	23	9.5
PS	72	F	12	1	21.6	3.5	188	1.48	0.97	124.7	2.1	high	10.3	5.9	0.05		12.2
PS	70	M	11	1	19.7	3.7		1.27	0.76	110.5		high	8	5.8			8
PS	57	F	11	1	15.8	4	157	1.72	1.01	112.4	1.5	high	9	6.4	0.33	38	12
PS	63	F	11	1	22.6	3.3	264	1.39	0.78	116.7	1.6	high	11.1	6.2	0.02	36	12.3
PS	60	M	11	1	19.8	4.3		1.03	0.84	121.4	1.8	high	8	5.1		36	9.7
PS	62	M	10	1	20.3	3.9	121	1.42	0.69	114.2	2.1	high	9.1	5.8			10.8
PS	63	F	10	1	24.7	4.1	214	1.41	1.07	127	1.8	high	8.5	7	0.72	27	9
PS	67	M	8	1	16.6	3.8	100	1.44	0.9	74.01	1.9	high	10.4	5		28	12.2
PS	73	M	8	1	21.1	3.7	148	1.44	0.72	104.2	2.1	high	7.9	5.6	0.25		13.5
PS	52	F	12	1	22	3.7	136	1.74	0.83	102.5	1.9	high	9.4	3.1	0.18	24	9.4
PS	72	F	8	1	20.9	3.8		1.4	0.91	96.46	1.8	high	8.8	5.9		32	11.5
PS	51	M	7	1	19.3	3.8	110	1.31	0.71	16.01	1.8	high		6.1			9.3
PS	61	F	12	1	18.7	4	166	1.56	0.81	125.8	1.3	high	8.5	5.7	0	33	10.4
PS	78	M	7	1	20.6	3.7		1.03	1.09	111.2	2.1	high	8.1	6.3		26	11.8
PS	80	M	7	1	22.4	3.6	155	1.55	0.68	102.8	2.1	high	8	5.4	0.2	36	10.1
PS	55	F	7	1	19.1	3.5	187	1.47	0.82	127.9	1.6	high	9.6	2.6	0	25	11.1
PS	78	F	8	1	17.6	3.4	99	1.51	0.74	112.9	1.1	high	7.4	5		35	9.6
PS	60	M	7	1	20.1	3.9		1.47	0.97	79.85	1.8	high	8.3	6		29	8.7
PS	75	F	6	1	18.9	3.6		1.31	0.8	129.2	1.3	high	11.1	5.4	0.18	29	8.9
PS	74	F	6	1	21.5	3.8		1.43	0.79	117.4	1.8	high	10.1	5.3		37	9.6
PS	54	F	6	1	18.4	3.1	142	1.26	0.93	107.7	1.8	high	9.7	5.5	1.2	49	10.5
PS	77	M	6	1	18.9	4	104	1.86	1	137.4	1.8	high	9.1	5.5	0.1	27	8.5
PS	61	F	5	1	18.8	4.1	126	1.2	0.7	105.9	1.5	high	8.9	6.3	0.1		10.4
PS	81	M	5	1	20.2	3.8	152	1.53	0.8	100.8	1.3	high	10	6.8	0.06		
PS	74	M	4	1	24.8	3.5	135	1.47	1.05	148.2	1.5	high	9.1	4.2	0.06	23	10.1
PS	71	M	4	1	26.2	3.9	190	1.15	0.95	87.43	2.1	high		5.8	1.74	35	9.1
PS	62	M	4	1	22.4	4	137	1.51	0.84	92.53	1.5	high	8.3	6.4	0.4	24	10.4
PS	77	F	3	1	17.4	4.2		1.13	1.05	115.1	1.9	high	8.2	7.2	0.02	27	11.9
PS	55	M	3	1	21.9	3.7	158	1.51	1.05	92.13	1.3	high	9.1	3.9	0	19	10.8
PS	82	F	3	1	19.2	4		1.04	0.73	122.1	1.9	high	10.2	4.1	0.02	30	9.6
PS	69	M	3	1	23.9	3.8		0.91	0.82	117.5	1.3	high	7.6	2.1		25	8
PS	56	F	3	1	16.6	4.1	183	1.37	0.82	95.77	1.6	high	8.5	6.7	0.03		11
PS	56	M	2	1	19.1	3.9	168	1.8	0.83	103.1	1.6	high	8.7	3.8	0.49	17	10.2
PS	49	F	3	1	17.6	4.2	140	0.98	1.01	86.24	2.1	high	8.5	7.3	0.2	33	11.2
PS	61	F	2	1	21.3	3.7		1.12	0.77	101.9	1.6	high	9.2	5.8	0.06	22	11.1
PS	53	M	2	1	24	3.7	174	1.62	0.58	78.64	1.8	high	8.7	4	0	30	10.6
PS	81	M	2	1	18.3	3.4	165	1.6	0.99	122.4	1.6	high	8.6	5	0.05	19	11.8
PS	72	M	2	1	25.4	4	178	1.54	0.95	129.8	1.8	high	8.6	4.7	0.29	20	11.7
PS	50	F	13	1	17	4.6	163	1.2	1.07	130.7	1.8	high	8.6	7.6	0.17	18	13.3
PS	88	M	11	1	18.5	3.7	144	1.87	0.99	119	1.3	high	8.2	3.6	0.05	30	8.4
PS	66	M	5	1	20	4.1	70	1.35	0.71	94.4	1.6	high	8.8	5.6	0.2	19	10.8
PS	47	M	4	1	24.1	4	142	1.55	0.86	85.79	1.8	high	8.7	5.2	0.1	27	11.4
PS	61	M	2	1	18.7	4	212	1.16	1.07	119.9	1.8	high	8	6.6	0	21	11.8
PS	68	M	9	1	18.5	4.7	200	1.36	1.1	99.16	1.5	high	7.6	6.9		28	11
PS	59	M	7	1	19.4	4.3	211	0.87	1.27	93.68	2.1	high	9.9	5.7	3.54		11.3
PS	70	M	16	1	22.5	4.9	201	1.19	1.08	120.5	1.8	high	9.7	4.3	0.49	30	10.9
PS	75	M	2	1	17.6	4.7	145	1.33	1.52	102.3	2.1	high	9.3	3.8	0.3	35	11.6
PS	87	F	6	1	17.7	3.5	142	1.08	0.86	109.7	2.1	high	7.5	4.3	0.1	27	11.9
PS	53	M	9	1	28.4	4.5	130	1.26	1.31	108.4	1.8	high	8.8	2.7	0.12	26	8
PS	49	M	2	1	26.5	4.5	149	1.53	0.74	97.52	1.5	high	7.8	4.2	1.89	37	10.5
PS	60	M	18	1	21.1	4.7	170	1.29	0.93	103.1	1.8	high	9.9	6	1.61	52	11.6
PS	66	F	10	1	20.1	3.8	126	2.28	0.81	100.8	1.8	high	8	6	10.6	35	8.2
PS	54	M	2	1	22.1	3.9	138	1.96	0.86	94.55	2	high	9	4.2	0.3	42	11.6
PS	65	M	7	1	21.8	3.7	139	1.47	0.71	107.9	1.8	high	10.8	4.8	0.09	28	11.2
PS	70	F	30	1	14.6	3.6	94	1.16	0.77	65.66	1.8	high	8	6.7	0.36	36	11
PS	83	F	5	1	17.2	3.8	172	1.09	0.75	118.4	1.8	high	7.8	3.4	0.05	19	9.4
PS	65	F	5	1	20.2	3.6	181	1.61	0.89	100.8	1.3	high	8.5	4.9	0.28	24	9.3

PS	70	M	14	1	21.2	2.1	137	1.27	0.94	127.7	2.1	high		7.7	1.36		10.7
PS	69	M	6	1	19.2	3.7	106	1.26	0.8	107.3	1.5	high		2.9		25	9.4
PS	62	M	3	1	25.8	4	189	0.98	0.81	92.49	1.5	high		6	0.08		11.9
PS	40	M	6	1	23	2.3	224	1.91	0.53	49.83	1.5	high	8.4	5.5	0.34	29	12.1
PS	48	M	20	1	23.1	3.9	86	1.16	1.06	119.8	1.6	high	9.6	4.4	0.7	37	11.7
PS	57	M	19	1	24.7	3.9	96	1.65	1.05	126.6	1.6	high	9.3	5.6	1.22	28	10.9
PS	57	M	16	1	21.9	4	90	1.76	0.83	111.4	1.6	high	8.8	7.8	0.2	28	10.8
PS	48	M	19	1	22.7	4.3	128	1.32	0.65	98.22	2.1	high	9.7	5.4	0.04	26	9.7
PS	64	M	12	1	21.1	3.7		1.53	0.87	86.93	2.1	high	9.3	6.7	0.05	26	10.7
PS	42	M	16	1	25.2	4.1	118	1.31	1.14	132.6	1.8	high	7.9	3.1	1.65	31	10.8
PS	65	M	8	1	20.9	4.3	149	2.05	1.05	110.3	1.6	high	9.4	5.3	0.23	28	11.9
PS	68	M	3	1	21.9	3.7	160	1.33	0.85	81.95	1.8	high	9.4	5.5	0	29	10.4
PS	49	M	2	1	22.7	4.2	72	1.88	1.02	81.83	1.6	high	9.6	5.5	0.35	29	9
PS	67	M	10	1	23.8	4.2	175	1.46	0.76	122.9	1.6	high	9.3	7.5	0.01	50	11.9
PS	63	F	23	1	20.9	3.3	171	1.44	0.75	129.3	1.6	high	11.4	5.4	0.1	28	11.1
PS	53	M	26	1	22.2	4	118	1.47	0.74	115.7	1.6	high	11.2	5.6	0.07	30	12
PS	58	F	5	1	21.2	4.3	164	1.35	1.08	113.6	2.1	high	7.4	4.9	0.32		12.6
PS	72	M	3	1	22.3	3.6	218	1.61	0.75	81.01	1.5	high	8.5	3.6	0.6	31	11.3
PS	61	F	10	1	23.3	3.9	233	1.57	1.05	89.32	1.8	high	9	6	0.1	32	11.9
PS	67	M	10	1	21.2	3.7	204	1.34	1.02	95.26	1.6	high	10	4.7	1.5	29	10.8
PS	68	F	9	1	19.6	3.3	155	1.3	0.67	89.8	1.6	high	8.6	6.3	0.03	27	10.4
PS	78	M	18	1	18.2	3.4	133	1.33	0.77	103.3	1.8	high	9.3	6.5	0.05	29	10.2
PS	73	F	4	1	25.5	3.8	137	1.42	0.83	64.51	1.5	high	9.6	8.7	0	17	12.2
PS	67	M	3	1	23.2	4.1	145	0.95	1.03	157.2	1.5	high	8.5	5.1	0.1	21	11.5
PS	73	M	2	1	23.1	3.7	136	1.69	0.82	52.99	2.1	high	7.9	5.4		22	8.7
PS	82	M	9	1	20.2	3.3	146	1.19	0.83	77.98	1.8	high	9.1	5.8	0	27	9.5
PS	78	M	12	1	24.5	4.2	168	1.54	0.67	109.9	1.6	high	9.1	6.7	0.01	25	10.9
PS	83	M	2	1	19.5	3.9	176	1.31	0.93	104.4	1.5	high	8	4.4	0.1	28	9.7
PS	76	M	10	1	19.9	3.7	157	1.23	0.93	110.3	2.1	high	7.9	6	0.01	27	12.7
PS	54	M	8	1	19.2	4.1	103	1.54	0.77	86.13	1.1	high	8.8	4.9	0.06	35	9.9
PS	48	F	8	1	17.1	3.9	154	1.71	0.93	102.2	1.5	high	9.1	4.1		28	9.5
PS	76	M	7	1	18.7	3.6	195	1.23	0.68	131.6	1.3	high	9.2	3.2	22.97	25	7.1
PS	61	F	6	1	20.4	4.2	131	1.38	1.13	129.1	1.5	high	9.9	5.3	0.9	33	9.9
PS	57	F	5	1	19.7	3.8	118	1.2	1.06	100.4	1.6	high	9	5.1	0.07	17	10.4
PS	62	F	3	1	15.6	3.6	175	1.59	0.96	95.27	1.6	high	8.9	5.1	0.15	29	10.5
PS	59	F	3	1	20.5	4	156	1.37	0.92	107.9	2.1	high	8	4.5	0.04		11
PS	68	M	2	1	23.4	3.8	135	1.36	0.92	144.3	1.6	high	10	4.2	0.25	24	10.8
PS	57	M	18	1	19.5	4		0.99	0.84	117.6	1.1	high	11.3	4.6		22	10.7
PS	40	M	18	1	18.1	3.7	184	1.2	0.9	104.5	1.9	high	9.1	8.3		33	11.2
PS	65	F	17	1	18	3.7	163	1.01	0.88	103.9	2.1	high	9.3	4.4	1.26	13	11.3
PS	65	F	16	1	16.6	4.2	128	1.16	0.96	102	1.3	high	7.5	2.7		28	10.8
PS	65	F	16	1	20.9	3.6	114	1.94	0.77	110.7	1.5	high	8.7	5.7	0.28	36	8.7
PS	61	F	24	1	21.1	3.9	138	1.62	0.93	151.1	1.6	high	8.7	2.9	2.2	28	10.4
PS	64	M	14	1	18	4	145	1.18	0.9	126.2	2.1	high	8.8	4.6	0.07	22	10.3
PS	70	F	24	1	21.1	3.5	171	1.35	1.01	71.94	1.9	high	9.1	5	0.03	28	10.2
PS	76	M	14	1	24.5	3.5	126	1.67	0.68	88.85	1.8	high		8.3		56	9.7
PS	65	M	13	1	23.7	3.6		1.56	0.94	119.4	1.6	high	8.4	4.8		27	10.5
PS	62	F	33	1	17.1	3.5	169	1.2	1.12	68.65	1.9	high		8			11.1
PS	83	F	12	1	15	3.7	152	1.44	1.13	122.9	1.8	high	9	2.7	0.82	23	10.3
PS	63	F	11	1	15.9	4.1		1.43	0.88	98.41	1.4	high	8.5	3.5		36	10.7
PS	73	M	11	1	18.5	3.6		1.62	0.78	114.4	2.1	high	8.4	5.5	0.1	22	10.5
PS	59	F	10	1	20	4.3	111	1.4	1.13	111	2.2	high		4		24	10.9
PS	68	F	8	1	19.6	3.8	97	1.19	0.84	118.2	1.5	high		4.9	0.3	35	9.6
PS	64	M	10	1	17.8	3.8	166	1.61	0.94	114.6	1.6	high		3.8			10.2
PS	59	F	21	1	16.9	3.7	108	1.23	1.18	115.7	1.9	high		5.1			11.1
PS	48	F	7	1	19.6	4.3	109	1.19	0.77	88.69	1.9	high		9.6			10.9
PS	66	M	8	1	17.2	3.8	194	1.47	0.81	95.91	1.5	high	9.3	4.1	0.3	23	10.5
PS	32	M	10	1	17.6	4.1	153	1.09	1.1	96.41	2.1	high		6.4	0.14	26	13.6
PS	73	M	6	1	20.1	4.1	226	1.31	1.01	107.4	1.8	high	9	5.2	0.01	18	10.4

PS	38	M	8	1	20.6	4.2	103	1.56	0.57	92.4	1.6	high	9.2	3.1	0.08	22	10.7
PS	62	M	6	1	22.8	3.9	229	1.18	0.9	131.6	1.8	high	8.9	4.3	1.08	21	10.8
PS	68	F	12	1	19.2	3.3	156	1.29	1.18	71.52	2.1	high		2.9		31	9
PS	71	F	4	1	18.4	3	123	1.31	1.06	99.83	2.1	high	8.6	4.5	0.08	18	10.6
PS	80	M	3	1	21.8	3.7	234	1.57	0.82	116.1	1.5	high	8.7	4.8	0.11	17	10.4
PS	71	M	2	1	24.8	4.5	94	1.73	0.8	83.8	1.3	high	8.4	4.9	0.05	27	11.4
PS	57	F	12	1	18.6	3	203	1.28	0.75	90.7	1.1	high	7.8	2.7	0.01	20	9.5
PS	59	M	19	1	19.8	4.7	148	1.12	0.87	100.6	1.9	high		5.7		29	9.5
PS	69	F	17	1	20	4	111	1.1	0.88	101.7	1.3	high	10.3	7.8			10.5
PS	67	F	13	1	20.1	4.1		1.26	1.11	109.9	1.6	high	9.4	4.7	0.47	29	10.9
PS	50	F	13	1	16.2	4.3	189	2.18	0.86	81.78	1.5	high	9.4	5.3	0.07	36	12.1
PS	69	F	11	1	18.6	4.1		1.13	1.02	115.5	1.9	high	8.5	4.7	0.13	33	9.6
PS	55	F	11	1	17.6	3.9	141	1.64	1.17	93.78	1.8	high		4.8	0.05		10.9
PS	70	F	10	1	16.2	4.1	114	1.29	0.98	102.6	2.2	high		6.6	0.3	38	12.7
PS	61	F	10	1	18.6	3.7		1.08	1	131.1	1.9	high	8.9	4.4	0.05	22	10.3
PS	56	M	8	1	22.1	3.7	101	2.29	0.85	137	2.5	high	8.3	6.6	0	24	11
PS	74	F	7	1	15.4	4.2	218	1.28	1.15	115.2	2.2	high		7.5	0.08	27	10.2
PS	79	F	6	1	17.2	4	144	1.31	0.77	113.4	2.1	high	9.4	6.7	0.31	29	11
PS	77	F	6	1	16.5	3.7	188	1.64	1.11	132.1	1.5	high	10.4	4	0.14	27	11.4
PS	56	F	6	1	20.4	4.5	116	1.55	1.17	71.7	2.5	high	9	5.7	0.07	26	9.5
PS	81	M	2	1	19	4.3	174	1.51	0.91	66.42	2.1	high	8.8	3.9	0.17	37	12.8
PS	62	M	7	1	19.8	3.9	133	1.31	0.75	103.5	2.1	high	7.3	3.4	1.1	36	7.8
PS	57	M	7	1	21.2	3.6	64	1.45	0.93	107.4	1.3	high	9.4	4.9	0.01	30	10.9
PS	77	F	5	1	17.4	3			1.45	63.02	1.5	high	8.6	5.3			8.9
PS	79	F	3	1	18.8	3.7		1.44	0.69	97.89	1.6	high	9.9	5.3	0.07	17	10.3
PS	49	F	3	1	22	3.6	153	1.18	1.02	76.36	1.5	high	9.1	3.5	0.2	40	11.5
PS	71	F	20	1	17.2	3.9		1.63	0.97	101.2	1.6	high	9.3	5	0.33	35	11.7
PS	74	M	17	1	20.4	3.8	105	1.62	0.92	125.3	1.3	high	8.6	3.3	26.9		10
PS	69	F	13	1	17	3.8	158	1.06	0.82	76.41	1.8	high	9.3	4.9	0.1	29	11.2
PS	60	M	12	1	18	3.5		1.74	0.68	88.71	1.6	high	9.2	6	0.05	23	11.7
PS	78	M	10	1	20.9	3.6		1.58	0.9	109.3	1.6	high	10.1	5.5	0.17	16	12.9
PS	76	M	9	1	16.1	3.8	185	1.23	1.19	133.1	1.9	high	9.6	6.6	0.1		9.9
PS	80	F	9	1	18.5	3.8	150	1.82	0.93	101.7	1.5	high	9.7	4.9	0.53	24	10.1
PS	42	F	7	1	19	3.8	109	1.47	0.9	98.5	1.6	high	9.2	4.1	0		9.7
PS	26	M	3	1	19	4.2	177	1.58	1	108.2	1.1	high	9.3	4.4	0.07	24	10.1
PS	80	M	2	1	25.8	2.2	217	1.7	0.8	90.28	0.9	high	8	5.3	0.3		11.9
PS	57	F	2	1	16.5	4.2	133	1.15	1.05	67.32	1.3	high	8.7	4.6	0.02	26	11.2
PS	49	F	2	1	18.5	3.8	184	1.53	0.93	110.2	1.9	high	9.3	6	0		10.3
PS	61	F	4	1	19.7	4	220	1.72	0.78	74.91	1.8	high		5.8	0.34		11.4
PS	68	M	4	1	19.3	3.7	146	1.57	0.77	52.03	1.9	high	9.1	5.5	0.1		10.9
PS	67	F	17	1	19.6	4.3	210	1.57	1.18	112.1	1.6	high	9.5	6.3	0.02	28	11
PS	67	F	27	1	17.4	4	119	1.1	1.17	104.5	2.2	high		7.9		27	11.4
PS	68	M	10	1	20.2	3.8	174	1.37	0.98	140.8	1.9	high	9.7	5.6	1.88	31	10.7
PS	67	M	4	1	21.9	4.1	146	1.48	0.86	126.9	1.6	high	9.2	4.7	0.41	38	12.3
PS	72	M	3	1	24.5	3.9	155	1.56	0.65	66.87	1.6	high	10	4	0.55	24	10.1
PS	57	M	30	1	16.2	3.9	120	1.38	0.93	83.75	1.9	high	9.2	5.5	0.04	26	10
PS	66	M	2	1	22.8	3.8	134	1.4	0.97	76.6	1.9	high	8.9	5.9	0.04	29	10
PS	58	M	4	1	20	4.3	174	1.68	0.98	108.5	1.5	high	9.6	3.7			12.2
PS	62	F	9	1	20.5	3.7	98	1.4	1.48	112.2	1.3	high	8.7	3.7	0.6	54	9.9
PS	41	M	7	1	19.7	4.4	168	1.56	0.99	111.4	1.9	high	9.2	5.2	0.31	14	9.9
PS	68	M	5	1	20.6	4.1	192	0.96	1.03	76.95	1.8	high	9	6.2	0	26	11.6
PS	66	M	3	1	23.6	6	131	1.33	0.89	130.5	1.9	high	9.9	5.7	0.24	24	10.5
PS	76	M	11	1	22.2	3.8	157	1.02	0.96	112.5	2.1	high	8.7	5.6	0.6	29	10.7
PS	63	F	17	1	20.1	4.3	216	1.74	0.98	113.8	1.6	high	9.2	4.4	0.05	24	11.8
PS	54	F	32	1	18	4.1	135	1.73	1.35	100.4	1.5	high		6.7	0.2	29	9.2
PS	58	M	28	1	23.6	4.1	166	1.51	1.33	112.5	1.9	high	10.1	4.4	0.09	24	11
PS	72	F	14	1	27.9	3.9	190	1.72	1.1	239.5	1.5	high	8.3	4.9	0.02	30	12
PS	51	F	8	1	23.6	4	116	1.64	0.84	117.8	1.1	high	10.7	3.9	0	16	10.2
PS	69	F	7	1	20.9	3.9	158	1.67	0.83	134.4	1.6	high	10	5.9	0.05	31	10.6

PS	82	F	6	1	23.2	4		1.29	0.89	109.2	2.1	high	7.5	4.7	0.54	27	11.7
PS	50	M	5	1	28	4	112	1.75	0.77	103.6	1.5	high	9.7	5.5	1		9
PS	84	M	2	1	18.4	4.1	129	1.51	0.9	87.68	1.6	high	9.8	4.7	0.06	30	12.3
PS	79	M	4	1	22.4	4.1	159	1.05	1.32	109.8	1.6	high	10.7	4.4	0.1	29	10.2
PS	72	M	8	1	20.3	3.6	137	1.48	1.06	117.2	1.8	high	9.3	6.7	0.07	34	10.8
PS	54	M	3	1	26.5	3.8	173	2.46	1.16	95.41	2.1	high	9	3.8	0.12	38	9.4
PS	81	M	2	1	18.3	3.9	162	1.48	0.73	76.62	1.9	high	9.4	6.8	0.05	24	9.9
PS	61	F	20	1	19.6	3.8	145	1.4	0.85	117.1	2.1	high	8.9	5.3	0.08		10.3
PS	65	M	18	1	22.2	3.9	176	1.51	0.82	118.1	1.6	high	9.9	6.6	0.37	31	10.9
PS	84	M	13	1	22.7	3.3	145	1.21	0.85	116.6	2.1	high	9.8	5.1	0.1	33	12.4
PS	63	F	13	1	19.9	3.7	147	1.48	0.79	137.3	1.6	high	8.8	4.9	0.11	35	10.2
PS	48	M	11	1	21.1	3.8	84	0.9	0.86	106	2.5	high		5.1	0	35	8.9
PS	75	M	8	1	22	3.9	176	1.42	0.73	107.4	1.9	high	9.3	3.8	0.05	31	10.8
PS	64	F	8	1	23	3.7	126	1.59	0.97	86.06	1.8	high	8.2	5.5	0.05	21	10
PS	46	F	6	1	23.7	3.8	81	1.44	0.83	103.4	2.1	high	8.7	2.9	0.35	21	8.3
PS	70	M	5	1	22.5	3.9		1.35	0.94	105.1	1.8	high	7.6	6.3	0.18		9.7
PS	62	M	9	1	23.4	4.1	164	1.1	0.9	131.4	1.5	high	9.2	3.9	0.17	21	9.9
PS	68	F	3	1	18.9	3.7	184	1.25	0.96	99.73	1.9	high	10.9	4.7	0.05	30	12.1
PS	69	M	2	1	27	3.8	106	1.63	0.83	85.17	2.1	high	8.6	6.5	0.02	26	12.5
PS	57	F	2	1	30	4.1	154	1.54	0.78	78.3	1.5	high	8.7	4.8	0.59	27	11
PS	61	F	10	1	19.4	3.8	188	0.99	1.11	119.9	2.1	high	9.4	5.6	1.2		12.3
PS	74	F	3	1	26.3	4	184	2.18	0.95	121	1.6	high	9.4	5.1	0.05	16	10.3
PS	48	F	21	1	24.9	4.1	114	1.27	0.9	115	1.5	high		4.4	0.1	34	7.4
PS	67	M	4	1	21.3	4	157	1.59	0.92	79.11	1.5	high	10	5.2	0	30	10.2
PS	62	M	29	1	19.3	4.1	143	1.65	0.87	103.3	1.9	high	10.3	5.3	0.21	21	9.8
PS	45	F	22	1	18.9	3.5	283	1.46	1.06	107.4	1.8	high	10.6	4.4	0.05	22	11.8
PS	52	F	21	1	18.6	3.9	126	1.3	0.79	128.7	1.9	high	9.1	5.3	0.05	22	10.5
PS	54	M	21	1	25.2	4.1	146	1.36	1	114.9	1.9	high	8.9	3.5	0.84	17	8.8
PS	65	M	21	1	19.4	3.3	117	1.12	0.85	121.2	2.2	high		4.4	0.09	54	9.9
PS	78	M	18	1	23	3.5	280	1.49	0.91	130.2	1.3	high	9.4	4.7	0.06	25	11.8
PS	63	M	17	1	16.3	3.6	139	1.05	0.91	67.92	2.1	high		4.6	1.7	26	10.6
PS	48	M	14	1	22.3	3.8	109	1.51	0.97	111	1.8	high	8.2	2.9		34	9.9
PS	46	M	10	1	19.2	4.2	117	1.36	0.88	95.11	1.6	high	8.7	3.9	0.07	26	11.2
PS	60	F	7	1	16.7	3.9	159	1.06	0.92	117.2	2.2	high		6.4	0.27	29	10.5
PS	68	F	5	1	24.9	3.8	145	1.3	0.84	110.4	1.6	high		5.9		35	8.3
PS	69	F	5	1	19.7	3.1	129	1.39	0.87	86.19	1.6	high	7.9	6.3	0.19	21	10.3
PS	51	M	5	1	24.6	4	131	1.11	0.88	109.3	1.5	high	8.4	7.6	0.3	30	10.5
PS	53	F	4	1	24.1	4	96	1.29	0.89	108.3	1.9	high	9.6	3.7	0.11	26	10.2
PS	71	M	4	1	18.8	3.4	142	1.52	0.75	120	2.1	high	8.8	7.8	2.4		12.5
PS	59	F	3	1	23.6	3.8	173	1.21	1.23	105.9	1.5	high	9.7	6.6	0.42	30	11.6
PS	61	M	3	1	22.8	3.8	139	1.4	0.85	89.69	1.8	high	10	9.9	0.15	31	9.6
PS	64	F	2	1	26.6	4.2	198	1.46	0.78	79.24	1.5	high	8.4	6			11.9
PS	59	M	11	1	19.4	3.6	150	1.37	0.98	107.7	1.5	high		3.6			9.6
PS	74	F	10	1	15.4	4	177	1.37	0.98	87.07	1.8	high		5		29	10.3
PS	62	M	34	1	17.9	4.3	118	1.22	1.08	83.9	1.9	high	9.2	7.8		35	11.5
PS	86	F	2	1	22.5	3.5	98	1.67	0.96	117	1.5	high	8.6	4.6	0.02	28	11.3
PS	81	F	6	1	17.3	3.6	127	1.78	1.08	98.19	1.3	high	9.8	3.4	0.05	21	11.6
PS	86	F	2	1	17.7	3.3	184	1.28	1.03	133.5	2.1	high	8.4	5.7	0.09	17	10.3
PS	79	F	33	1	18.5	3.3	132	1.11	1.01	76.65	1.6	high	10.6	5.8		25	7.9
PS	32	F	3	1	31.9	3.8	216	1.65	0.77	94.1	1.6	high	9.5	4.7		31	9.5
PS	55	F	30	1	17	3.5	134	1.69	0.86	75.5	1.6	high		6.7	0.45	33	9.4
PS	64	F	24	1	16	4	148	1.19	1.36	100.4	1.9	high		6.7	1.53	36	9.7
PS	50	F	18	1	19.3	4.5	217	1.38	0.9	106.7	1.9	high		5.2	0.35	32	9.3
PS	61	M	15	1	18.7	3.7	128	1.46	1.27	123.4	1.5	high	9.4	3.9	0.05	30	9.4
PS	68	F	15	1	24	3.8	156	1.66	0.58	134.6	1.6	high	8.8	4.4	0.19	26	10
PS	61	F	15	1	20.3	3.8	156	1.16	0.95	146.6	1.9	high	8.6	1.9		16	11.4
PS	63	F	14	1	19.9	3.9	143	2.06	0.91	103.7	1	high	10.2	4.9	0.2	43	9.6
PS	58	F	11	1	21.4	3.9	147	1.77	1.09	117.6	1.3	high	9.5	7.9	0.1	33	10.5
PS	59	F	11	1	18.1	2.3	175	1.79	1	138.5	1.3	high	9.1	4.8	0.1	30	9.9

PS	64	M	10	1	18	4.2	132	1.91	0.87	148.9	1.3	high	9.8	5.8	12	34	6.3
PS	61	M	9	1	23.7	4	185	1.56	0.9	83.67	2.1	high	8	6.9	0.15		11.8
PS	72	M	9	1	20.5	4.2	112	1.26	1	105.8	2.1	high	10.6	6.1	0.07	41	9
PS	67	F	9	1	22.3	3.7	147	1.69	1.14	115.1	0.8	high	9.4	4	0.03	31	12.1
PS	68	M	9	1	22.2	3.9	121	1.21	1.28	144.8	1.8	high	9.6	7.4	0.3	33	11.1
PS	47	F	8	1	25.8	4.1	110	1.25	0.79	74.7	2.1	high	9	5.9	0.01	20	11.4
PS	45	F	8	1	19.2	4	177	1.61	1.03	129	1.9	high	9.7	4.7	0.12	32	12.1
PS	60	M	8	1	27	4	152	1.7	1.13	130.3	1.6	high	9.6	6.1	0.05		10.3
PS	62	M	8	1	17.8	3.9	161	1.47	1.2	108.3	1.5	high	9.3	2.9	1.07	25	8.9
PS	44	M	8	1	28.6	3.8	191	1.8	0.91	74.7	1.5	high	8.2	4.6	0.8	25	9.4
PS	78	M	7	1	21	4.3	216	1.15	0.94	102	1.6	high	9.2	6.9	0.84		9.7
PS	60	M	7	1	23.9	3.6	107	1.77	1.02	112.5	1.5	high	10.6	6.6		27	9
PS	49	F	6	1	18.8	4.1	101	1.11	0.8	141.4	1.6	high	10	5.4	0.06		11.6
PS	54	F	6	1	25.9	4.4	113	1.25	0.84	132	1.9	high	9.1	6.4	0.05		10
PS	77	F	6	1	15.9	3.8	151	1.24	0.92	94.58	1.3	high	10	4.6	0.03	28	10.4
PS	50	F	6	1	14.8	4.6	189	0.88	0.92	100	2.1	high		8.3		30	12.3
PS	74	F	6	1	23.4	4.2	181	1.5	0.83	85.38	1.8	high	9.4	5	0.03	29	9.1
PS	81	M	5	1	19.6	3.4	157	1.7	1.11	122.9	1.6	high	9	3.5	0.06		11.1
PS	71	M	5	1	19.9	3.9		1.41	0.97	77.77	2.1	high	8.7	4.3		22	8.5
PS	64	M	4	1	20.6	4	199	1.52	1.12	85.53	1.6	high	9.6	3.8	0.05		10
PS	80	M	4	1	18	3.8	177	1.54	1.01	109.5	1.8	high	10.3	4.3	0.37	21	9.7
PS	66	F	3	1	21.5	4.5	80	1.22	1.01	95	2.1	high	7.9	4.9	0.1	23	8.3
PS	68	M	3	1	20.9	4.3	186	1.08	0.74	106	1.3	high	8.8	6.1	0.21	30	10.2
PS	81	F	2	1	22.4	4.1	145	1.42	0.97	89.95	1.8	high	9.6	3.9	4.42	24	10.1
PS	65	M	2	1	19.3	3.9	165	1.05	1.04	81.28	1.5	high	10.1	2.8	0.12	25	8.2
PS	50	M	3	1	23.1	4.1	233	1.58	1.11	128.8	1.8	high		5.2	0.1	23	9.6
PS	82	F	2	1	19.7	3.7	111	1.61	0.86	54.96	1.5	high	8.3	3.2	0.06	28	10.7
PS	66	M	2	1	18.8	4.1	158	2.2	1.04	76.02	1.6	high	10.2	7.3	0.45		11.8
PS	65	M	4	1	22.4		145	1.89	0.88	106.4	1.3	high	8.1	3.5	0.01	23	8.3
PS	52	F	5	1	19.1		143	1.72	1.2	78.72	1.3	high	9.7	6.7	0.08	22	11
PS	39	M	4	1	35.5		145	0.65	0.85	103	1.8	high		6.7		31	10.5
PS	54	M	9	1	23.5		163	1.67	0.76	124.4	1.5	high		5.1		29	11.3
PS	77	F	6	1	19.5			1.43	1	115.9	2.1	high	8.9	8.4	7.3	25	12.2
PS	55	F	5	1	22.6		131	0.99	0.94	97.65	2.1	high	8.9	6.2	0.06	22	10.8
PS	64	M	15	1	19.1		179	1.45	0.86	103.5	1.8	high	9.4	4.4	0.05	22	11
PS	60	F	9	1	18.5			1.08	0.78	98.95	2.1	high	8.7	6.5	0.3	36	10.3
PS	71	M	11	1	19.5	3.8	161	2.14	0.91	115	1.9	high	9.1	6.5	0.05		10.5
PS	61	M	9	1	20.4	3.4	204	1.41	0.76	132.5	1.8	high	9.6	6.8	0.32	32	12.4
PS	74	M	7	1	22.3	3.3	141	1.41	0.95	152.8	1.5	high	8.9	3.2		22	11.1
PS	46	F	4	1	17.7	3.9	127	1.12	1.12	120.2	1.3	high	8.6	5.9	0.08	23	8
PS	43	M	3	1	23.7	4.3	161	1.4	1.04	119.4	1.1	high	9.1	5	0.11	22	11.2
PS	53	F	2	1	19.6	4.1	190	1.94	0.95	87.09	1.5	high	8.2	4.1			9
PS	72	F	2	1	18.4	5.9	199	1.58	0.7	74.87	1.6	high	10.6	5	0.12		10.2
PS	72	F	5	1	16.8	3.5	205	1.27	0.69	91.75	1.5	high		3.7			11.6
PS	68	M	5	1	21.4	1.8	154	1.2	0.76	48.78	2.1	high		6	0.01	23	10.4
PS	75	F	5	1	19.2	3.3	103	1.93	0.53	31.87	1.8	high	8.3	5.2	0.1	26	8.6
PS	69	M	6	1	20.1	3.6	186	1.75	0.9	102.4	2.1	high	9.1	5.5	0.19	44	11.1
PS	40	F	7	1	17.4	3.5	152	1.77	1.01	64.8	1.5	high	10.5	2.3	0.1	38	8.6
PS	51	M	7	1	20.9	3.9	114	1.38	0.53	77.06	1.8	high	8.4	4.3	0.14	31	9.3
PS	67	M	8	1	21.7	3.9	155	2.37	0.89	102.3	1.3	high	8.7	4	0.07		10.4
PS	57	M	8	1	20.5	2.2	172	1.26	0.78	44.97	1.8	high		4.6	0.1	27	11.6
PS	69	F	8	1	17.9	3.9	201	2.05	0.96	98.93	1.6	high	9.4	4.9	0.05		10.8
PS	83	F	10	1	24.7	3.6	124	1.72	0.68	91.6	1.1	high		6	0.1	22	10.5
PS	63	F	13	1	17.8	3.6	176	1.5	0.71	91.05	1.8	high		8.6	0.1	26	10.5
PS	48	F	14	1	20.3	3.5		1.18	0.93	86.46	1.8	high	10.9	5.3	0.03	20	9.9
PS	51	F	15	1	21.5	3.5	157	0.66	0.74	101.2	1.5	high	9.2	2.7	0.1	25	10.6
PS	31	M	17	1	15.3	2.5	171	1.41	0.54	38.84	1.6	high	9.6	4.8	0.05	23	10.5
PS	55	M	23	1	21.5	3.9	205	1.47	1.01	88.16	1.6	high	9.1	4	0.06		10.5
PS	61	F	28	1	24.7	3.4	150	1.1	0.93	64.39	1.6	high	9.8	4.8	0.37		11.9

PS	63	M	33	1	20.3	2.9	119	1.68	0.86	47.98	1	high	8.8	4.5	0.2	30	10.2
PS	56	M	33	1	18	3.7	137	1	0.97	64.13	1.8	high	8.1	7	0.05	31	9.9
PS	75	F	2	1	19.4	4	167	1.45	0.85	117.1	1.6	high	8.9	4.3	0.05	19	10.1
PS	62	F	3	1	20.5	3.4		1.14	0.95	91.93	1.9	high	9.3	4.1	0.19	24	11.6
PS	76	F	5	1	16.7	2.8	164	1.69	0.53	54.58	1.8	high	9.1	3.2	0.07	19	10.5
PS	63	M	34	1	18.2	3.3	270	1.2	1.09	75.22	2.1	high	8.3	5	0.02	17	12.1
PS	51	M	33	1	21.5	3.6	216	1.58	1.39	86.98	1.8	high		5.8	0.02	28	10.3
PS	44	M	32	1	18.2	3.8	141	1.24	1.13	104	1.9	high	8.8	5	0.02	27	10
PS	45	M	30	1	23.4	3.5	149	1.47	0.94	90.22	1.6	high	8.2	2.3	0.02		10.4
PS	57	M	29	1	19.8	3.4	176	1.72	0.89	88.93	1.6	high	8.6	3.9	0.05	28	10.1
PS	59	M	26	1	23.8	3.9	198	1.29	0.92	93.5	1.6	high	7.8	8.1	0.1	29	13.3
PS	49	M	25	1	20.3	3.9	107	1.68	1.08	86.72	1.4	high	9	5.3	1.8		8
PS	57	F	24	1	23.8	3.4	145	1.27	1.27	105.8	2.2	high	8.5	5.9	0.2		11.6
PS	64	M	20	1	19.8	3.8	165	1.45	0.88	116.7	1.4	high	9.5	6.1	0.1		10.4
PS	65	M	17	1	20.4	4	142	1.27	1.06	101.3	1.2	high	9.8	6.2	0.06	24	11.8
PS	59	M	16	1	22.6	3	133	1.12	1.03	113.8	1.2	high	8.4	5.8	2.08	22	10.7
PS	60	M	16	1	19.8	4	179	1.25	1.17	105.7	1.3	high	9.6	9	0.04	19	9.5
PS	71	M	14	1	20.4	3.7	160	1.33	0.96	125.9	1.6	high	8.8	6.4	0.07	25	12.5
PS	41	F	13	1	18.3	4.1	138	1.47	1.01	121.4	1.1	high	8.2	4.6	0.17	22	9.6
PS	59	F	12	1	24.8	3.9	216	1.28	1.35	138.6	1.2	high	9.7	7	0.03	24	10.6
PS	60	F	12	1	25.8	3.7	150	1.34	1.15	122.8	1.5	high	9.1	6.2	0.03	29	9.5
PS	72	M	11	1	19.4	3.6	155	1.48	1.26	83.36	1.5	high	8.4	4.5	0.3	30	10.7
PS	64	M	10	1	22.7	3.6	149	1.43	0.94	150.6	1.5	high	9.1	3.7	0.01	30	10.6
PS	61	M	7	1	20.5	3.7	147	1.4	0.77	105.2	1.5	high	9.4	4.3	0.1	26	11.7
PS	59	F	7	1	17.9	4.3	211	0.86	1.09	121.4	1.6	high	7.5	5.5	0.29	21	9.7
PS	61	F	7	1	16.4	3.9	198	1.86	0.76	99.75	2.1	high	9.8	5.3	0.07	29	10.5
PS	75	M	6	1	20.1	3.6	164	1.43	0.92	114.9	2.1	high	8	5.4	2.02		8.4
PS	62	M	5	1	19.5	4.1	148	1.3	0.95	93.16	1.3	high	9.4	6.4	0.36	36	10.8
PS	66	M	5	1	20.2	4.2	126	1.19	0.79	90.8	1.2	high	7.2	7.6	2.1	23	9.5
PS	75	M	4	1	19.9	3.8	162	1.17	0.8	77.09	1.2	high	9.7	3.3	0.04	23	8.2
PS	49	F	4	1	21.5	3.6	152	2.23	0.83	108	1.5	high	8.7	6.4	0	30	10.7
PS	53	F	3	1	21.2	3.8	98	1.22	0.9	99.39	1.5	high	7	3.8		30	11.3
PS	75	F	2	1	24.5	3.8	258	1.22	0.94	55.9	2.1	high	9.5	4.2	0.18	30	
PS	54	M	2	1	21.2	3.8	141	1.41	0.83	77.75	1.2	high	9.2	5.7	0.1	33	10.5
PS	39	M	6	1	20.7	3.6	129	1.35	0.93	103.7	2.1	high		6.3	0.1	28	9.9
PS	68	F	2	1	21.5	3.6	155	1.65	0.85	79.3	1.6	high	9.6	5.1	0.05	15	10
PS	62	M	2	1	20.2	3.9	207	1.25	0.73	96.65	2.1	high		4.3		20	10.6
PS	74	F	26	1	17.9	4.1	143	1.62	1.13	119.3	1.3	high	10.2	6.2	0.08	19	11.2
PS	53	M	21	1	22.9	3.6	154	1.62	0.63	79.17	1.5	high	8.8	6.8	0.08	17	10.9
PS	60	M	19	1	21.7	4.3	161	1.62	1.1	109.4	1.5	high	9.1	5.6	0.3	27	10
PS	47	M	13	1	23.5	4.5	188	1.44	0.71	141.7	0.9	high	12.1	7.4	0.08	21	7.4
PS	57	F	11	1	16.9	3.9	138	1.17	1.18	120.5	2.1	high	9.1	5.2	1.54	28	10.9
PS	72	M	10	1	21.6	4	124	1.12	0.9	118.9	2.1	high	9.3	4.2	0.3	30	11.1
PS	54	M	8	1	23.9	4.5	116	0.98	0.74	102.7	1.3	high	7.8	6	0	49	10.7
PS	78	F	4	1	19.8	3.9	186	1.73	0.79	100.3	1.3	high	8.8	5.8	0.13	20	10
PS	72	F	5	0	17.7	3.8	208	1.22	0.9	87.61	1.5	high	9.1	3.7	0.47	16	10.1
PS	57	M	2	1	23.2	4.4		1.47	0.85	118.4	2.1	high	9.2	6.3	0.3	23	10.7
PS	60	M	6	1	23.6	4.2	140	1.45	0.78	128.6	1.8	high	8.9	5.5	0.34	34	10.6
PS	68	M	29	1	19.5	3.7	202	1.46	0.71	97.91	1.2	high	10.4	7.3	0.37	18	9.2
PS	68	M	6	1	22.7	4	98	1.57	0.69	77.46	1.8	high		3.9	0.46	38	10.4
PS	86	M	10	1	23.8	3.6	121	1.44	0.74	78.43	1.6	high	9.1	5.9	0.17	17	10.7
PS	65	F	2	1	19.4	4	155	2.13	0.8	73.52	1	high	9.2	5.9	1.2	25	8
PS	87	F	2	1	15.8	2.9		1.2	0.88	115.8	1.3	high		7.1			10.8
PS	67	F	2	1	19.1	3.7	185	1.72	0.78	95.87	1.8	high	9.6	5.7	0.16	29	11.5
PS	63	M	6	1	19.8	3.3	111	1.63	0.59	106.8	0.8	high		5.7	0.07		10.5
PS	71	M	19	1	20.6	4.1	161	1.05	1.08	124.7	1.8	high	8.7	4.8	0.05	24	10.5
PS	59	F	17	1	20.8	4.2	172	1.69	1.23	95.25	1.5	high	9.9	7	0.01	26	12.9
PS	59	M	9	1	20.3	3.9	158	1.45	0.97	77.52	2.1	high	8.1	4.4	0.16	30	10.8
PS	50	F	9	1	17.5	4.4	151	1.17	1.04	93.11	1.3	high		6.6	0.3	22	10.3

PS	75	F	5	1	20.4	3.5	158	1.29	0.98	114.4	1.8	high	8.3	5.4	0.04	23	10.1
PS	76	F	4	1	24.5	4	88	0.99	0.83	106.7	1.5	high	9.2	4.5			
PS	70	F	4	1	28.3	3.8	179	1.29	1.08	112.9	1.3	high	8.8	6	0.3	28	12
PS	74	M	3	1	20.6	3.9	147	1.43	1.18	89.94	1.1	high	9.4	5.2	0.08	29	9.3
PS	47	M	13	1	22.1	3.8	147	1.49	0.91	114.4	2.5	high	8.4	4.2		22	10.2
PS	56	F	8	1	16.3	3.2	143	1.95	1.22	103.4	1.6	high		4.2		26	8.4
PS	81	F	6	1	25.2	3.6	173	1.49	0.67	103.5	2.1	high	10.3	7	0.37	26	10.7
PS	40	F	14	1	20.4	4.2	213	2.77	0.66	99.41	2	high		2.2			13.2
PS	82	M	13	1	20.4	4.1	157	1.25	1.05	106.2	1.5	high	8.2	4.4	2.89	22	9.9
PS	62	F	11	1	19.9	4.1	142	1.09	0.79	109.1	2.1	high	9.4	6.4			10
PS	72	M	5	1	16.1	3.1	155	1.1	1.26	44.97	1.8	high	9	4.5	0.59	26	9.3
PS	58	F	5	1	17.2	3.5	145	1.06	1.06	118.6	2.1	high	10.2	5.6	0.1	27	11
PS	67	M	5	1	20.5	4.1	120	1.24	0.7	107	1.5	high	10.4	7	0	29	10.9
PS	74	M	3	1	24.7	4.1	198	1.05	1.07	105	1.8	high	8.8	5.6			9.7
PS	58	F	5	1	19.4	4.8	126	1.11	1	119.5	1.5	high	8.6	4	2.09	30	9.4
PS	72	F	14	1	15.7	3.9	149	1.67	0.88	102.5	1.5	high	8.7	4.4	8	38	8.8
PS	66	M	19	1	17.6	3.8	159	1.45	0.99	107.7	1.6	high	10.2	8.7	0.06	17	12.1
PS	61	M	13	1	19.3		175	1.46	0.92	109.3	1.8	high	8.9	4.8	0.9		9.6
PS	63	M	11	1	21.1		100	1.34	0.7	123.2	1.1	high		2		16	8
PS	46	M	9	1	21.1		189	1.38	0.89	118.3	1.5	high	9.5	4.5	0.04	12	11.1
PS	70	F	8	1	21.8		149	1.03	0.75	111.1	1.3	high		6.2	0.3	38	8.9
PS	78	F	8	1	25		222	1.5	0.68	124.6	1.2	high	9	5.6	0.09	27	9.5
PS	70	M	7	1	20.5		167	1.11	0.88	87.88	2.1	high	8.5	8.9			10.9
PS	49	M	6	1	25.9		219	1.61	0.61	99.73	2.1	high	8.5	4.6			11.5
PS	73	F	6	1	20.6		251	1.92	0.77	98.9	1.8	high		4.8	1.09	30	11.6
PS	58	F	6	1	44.4		166	1.47	0.74	106.2	2.1	high	8.5	4.9	1.72	22	10.9
PS	69	M	4	1	25.8		175	1.33	0.95	116.3	1.5	high	9.6	6.1		20	10.6
PS	71	M	3	1	22.6		163	1.42	0.61	66.48	1.8	high	9.3	4.5	0.02	32	11
PS	72	M	2	1	19.2		150	1.66	0.78	139.9	2.1	high	8.8	6.2	0.08	26	10.1
PS	75	M	3	1	19.9		120	1.18	0.62	91.3	2.1	high	8.7	2.7	0.21	36	8.4
PS	52	M	15	1	19.9		154	1.53	0.94	143.9	1.3	high	9.4	2.6	0.2	31	4.5
PS	64	F	30	1	16.8	3.8	111	1.29	1.24	74.57	1.6	high	9.2	7.5	0.16	49	10.8
PS	59	M	28	1	17.9	3.8	150	1.58	0.47	84.95	1.8	high	8.6	5.8	0.4	24	9.3
PS	60	F	26	1	16.7	3.3	172	1.28	0.76	109	1.2	high	9.3	7.4	0.06	40	12.4
PS	72	F	25	1	17.7	3.6	164	1.07	0.97	93.63	1.2	high	7.8	7.7	0.2	30	13.3
PS	70	M	10	1	16.4	3.7	197	1.54	0.98	110.8	1.5	high	9.4	5.3	0.1	30	8.9
PS	57	M	7	1	20.2	3.7	137	1.3	0.84	119.2	1.5	high	7.9	5.1	0.07	36	8.3
PS	58	M	5	1	22.1	3.8	147	1.26	0.71	104.6	1.6	high	9	5.5	0.67	26	9.6
PS	60	F	4	1	19.1	4.3	186	1.49	0.77	66.71	0.9	high	9.6	9	0.04	24	12.9
PS	53	M	4	1	16.4	3.7	105	1.13	0.8	98.29	1.6	high		8.6	0.12		9
PS	75	M	4	1	22.3	3.7	149	1.24	0.98	113.9	1.8	high	9	4.9	0.73	32	13.2
PS	68	F	3	1	16.3	3.8	186	1.43	0.86	84.84	1	high	10	5.7	1.35	26	12.1
PS	61	M	3	1	21.3	3.9		1.19	0.91	96.22	1.6	high		3.7	1.54		10.8
PS	54	F	3	1	19.6	3.6	119	1.16	1.07	106.4	2.1	high	8.8	6.2	0.2		9.6
PS	53	F	3	1	19.3	3.8	100	1.38	0.66	79.77	2.1	high	8	4	0.44	39	9.4
PS	81	M	5	1	25.3	3.4	129	1.59	0.9	132.1	1.8	high	9.5	4.6	0.7		11.2
PS	31	M	11	1	19.2	4.8	118	1.47	1	122.3	2	high		3.9	0.18	22	10.6
PS	21	M	9	1	20.3	4.1	179	1.68	0.86	60.05	1.6	high	9.4	4.5	0.07	32	11.1
PS	71	F	3	1	19.3	3.9	146	0.91	1.06	123.6	2.1	high		6.8	0.47		10.8
PS	56	M	5	1	20.1	3.9	124	1.34	1.16	138.7	1.8	high	8.2	3.4	0.18	29	11.4
PS	68	M	7	1	22		159	1.47	0.86	127	1.2	high	7.7	5.2	0.06	19	11.3
PS	54	F	3	1	21.1		142	1.48	1.03	119.7	1.6	high	8.5	4.5	0.07	19	9.3
PS	88	F	8	1	18.2		167	1.81	0.65	108.1	2.1	high	9	4.2	0.3	26	9.5
PS	65	M	4	1	26.4		150	1.26	1.03	138.9	1.6	high	8.6	4.5	0.26	20	10.9
PS	68	M	4	1	21.3		83	1.03	0.72	74.57	1.5	high	8.4	2.8	1.28		7.4
PS	65	F	4	1	19.1		140	1.49	1.09	105.3	1.6	high		5	0.13	25	11.3
PS	60	M	3	1	23.1		123	1.05	1.24	126.2	1.8	high	9.5	4.3		34	9.7
PS	66	F	14	1	18.6		128	1.06	0.83	72.36	1.3	high	8.6	4.5	0.1	19	10
PS	61	M	22	1	18.6	4	112	1.21	1	83.72	0.6	high	7.4	5	0.02	16	11.3

PS	53	M	11	1	22.5		114	2.68	0.89	134.9	1.8	high		6	1.17	26	10.4
PS	69	F	2	1	23.7		173	1.51	0.76	62.83	1.8	high		6.9	0.14	21	9.1
PS	79	F	26	1	16.1	3.7	141	1.55	1.22	104.2	1.7	high	7.3	6.4		35	9.4
PS	70	F	27	1	19.5	3.6	159	1.16	0.81	86.68	1.7	high	9.3	6.7		27	5.7
PS	75	F	15	1	24.8	3.4	133	1.37	0.71	115.5	1.6	high		5.6	0.06	23	11.9
PS	64	F	35	1	19.6	3.7	146	1.24	1.2	84.37	1.8	high	8.8	5	0.11	31	9.4
PS	61	M	6	1	22.5	3.6	58	1.08	0.82	156.6	2.1	high	8.8	5.6		27	10.3
PS	51	M	28	1	20.6	4.2	194	1.28	1.06	117.2	1.8	high	10.3	4.2	0.05	32	11
PS	71	F	4	1	21.4	3.7	195	1.46	0.74	66.04	1.6	high	10.3	5.8	0.18	32	11.3
PS	61	M	4	1	24.1	4.2	159	2.02	0.88	70.98	1.5	high	9.4	5.9	0	25	10.3
PS	71	F	3	1	20.6	4.1	128	0.95	0.83	119.7	2.1	high	8.7	5	0.13	31	11
PS	51	F	2	1	21.4	4.4	134	1.24	1.02	99.29	2.1	high	9.5	6.5	0.1	28	9.8
PS	55	M	26	1	18	4.1	114	1.35	0.64	98.72	1.8	high	10	7.1	0.05	31	10
PS	73	F	14	1	20.2	3.8	209	1.55	1.11	144.8	1.8	high	9.8	7.9	0.05	33	9.3
PS	83	F	10	1	21.4	3.9	144	1.47	1.16	114	1.8	high	9.3	7	0.05	27	9.4
PS	73	M	8	1	25	4.3	133	1.83	0.82	129.3	2.1	high	10.9	6.2	0	24	10.7
PS	70	F	3	1	20.7	3.7	109	1.8	0.99	94.66	1.3	high	10.9	5.6	0.1	41	11.1
PS	62	F	2	1	19.5	3.4		1.51	0.76	75.54	1.6	high	9.3	5.6	0	22	11.5
PS	78	M	5	1	27.9	4.1	141	1.44	0.51	126	2.1	high	9	8.7	0.1	26	9.7
PS	60	M	8	1	22.9	3.9	72	1.24	0.69	149.2	1.2	high	8.4	3.5	0.02		10.4
PS	53	M	26	1	15.7	3.2	191	1.66	0.59	72.53	1.4	high	9.5	5.8	0		9.5
PS	73	M	14	1	18.1	3.3	242	1.29	0.71	105.1	2.1	high		6.7	0.1	27	8.9
PS	70	M	10	1	20.2	3.5	205	1.86	0.91	102.4	2.1	high	10.3	5.2	0.59		11.7
PS	67	F	5	1	20.3	3.8	180	0.95	0.71	114.6	1.6	high	7.8	3.3	0.08	11	12.4
PS	57	F	12	1	22.3	4.2	119	1.4	0.85	133.8	1.8	high	9.4	5.5	0.05	29	10.3
PS	72	F	3	1	16.4	3.8	164	1.46	0.84	76.55	1.6	high		3.7	0.19	28	9.8
PS	70	M	3	1	24	3.6	99	1.64	0.54	100.3	2.1	high	9.1	4.4	0.17	23	9.3
PS	67	F	9	1	19.6	4.2	138	1.64	1.19	100.1	2.1	high	9.8	7.3	0.77	22	10.8
PS	69	M	9	1	21.5	3.8	189	1.48	0.62	99.34	1.3	high	10.5	5.6	0.05	22	10.1
PS	55	M	3	1	17.7	3.8	169	1.37	0.58	66.89	2.1	high	9.6	6.1	0	24	10.7
PS	74	M	12	1	24.2	4.1	92	1.61	0.76	123.9	1.9	high	8.1	4.2		33	9.5
PS	56	F	7	1	22.3	3.7	137	1.46	0.91	137.2	1.6	high		3.4	0.05	23	9.4
PS	60	F	12	1	18	3.8	86	1.18	1.02	130.2	1.8	high	10.2	7	0.1	32	10.3
PS	61	F	10	1	23.8	3.9	104	1.36	1.16	134.5	1.3	high	9	5.6	0.02	28	10.8
PS	52	M	25	1	17.1	4.1	154	1.89	1.15	130.6	1.5	high	9.3	6.1	0.4	36	10.6
PS	36	M	13	1	27	4.1	140	1.58	1.04	155.3	1.5	high	9.9	7.2	0.1	29	11
PS	79	M	8	1	24.8	3.8	129	1.23	0.77	122	2.1	high		6.8	0.1		11
PS	74	M	11	1	20.4	3.3	164	1.23	0.73	121	1.9	high		6.5			12.7
PS	60	M	12	1	21.4	3.8	122	1.43	0.72	114.4	2.1	high	7.9	3.1	0.1	29	13.3
PS	71	F	4	1	18.6	3.9	149	1.41	0.71	100.8	1.8	high	9.7	5.9	0.05	21	10.4
PS	56	F	20	1	22.2	3.1	179	1.34	0.89	92.07	1.9	high	7.9	4.9	0.1		10.4
PS	62	M	10	1	20	4.2	188	1.48	0.94	94.56	1.9	high	9.1	5.7	0		10.4
PS	57	F	4	1	26.3	3.7	168	1.25	0.96	130.3	2.1	high		5.1	0.3		9.7
PS	59	F	10	1	20.1	3.8	162	1.53	0.9	131.1	1.5	high	10.1	8.2	0.73	29	11.4
PS	82	M	7	1	25.7	3.5	138	2.17	0.42	23.2	1.5	high	9.5	5		31	9.3
PS	59	F	6	1	22.2	3.1	126	1.24	0.47	87.04	1.5	high	7.6	6.6	2.1	26	8.6
PS	69	M	17	1	20.8	3.8	69	1.22	0.9	118.4	1.3	high	8.8	5.7	0.05	23	16
PS	24	M	4	1	25.8	4.1	181	1.38	0.85	100.3	1.5	high	9.5	6.1	0.8	31	12.5
PS	69	M	5	1	33.2	4.1	162	1.32	0.59	114.4	1.5	high	9.4	5.4	0.07	28	12
PS	57	F	22	1	17.1	3.9	154	1.3	1.04	121.1	1.4	high		4.9	2.17	34	9.4
PS	55	M	2	1	26.1	4	171	1.32	0.85	117.9	1.4	high	10.5	5.8	0	33	10.4
PS	59	M	2	1	20.9	4.1	164	1.55	0.81	99.35	1.8	high	9.2	4.7		21	10.9
PS	43	F	21	1	18.3	3.6	151	1.37	1.02	91.03	2.1	high	10.4	5.1	0	31	11.9
PS	63	M	3	1	25.6	3.8	176	1.45	1.21	152	1.8	high	9.8	5.6			10.7
PS	82	M	8	1	19.6	3.5	118	1.52	1.02	132.8	1.8	high	9.6	6.9		30	
PS	33	M	13	1	22.5	4.5	214	1.38	0.86	123.2	1.9	high	8.3	6.7	0.3		12.8
PS	36	M	2	1	27.6	4.1	115	1.21	0.95	123.8	2.1	high	10.6	4.8		28	9.3
PS	39	M	9	1	22.5	3.7	139	1.99	1.01	92.4	1.1	high	9.1	5.4	0.02	28	11.2
PS	60	M	6	1	22.4	4	149	1.21	1.17	162.4	1.6	high	10	7	0.61	21	8.6

PS	77	M	6	1	21.3	4.3	119	1.92	0.96	133.1	1.8	high	9.3	3.1	0.2	2	11.6
PS	43	M	3	1	25.9	4.3	162	1.58	1.1	126.5	1.6	high	8.6	5.9	0.1		11.5
PS	45	F	12	1	20.9	4.2	105	1.75	1.1	118.2	1.6	high	10.1	3.6	0.6		11.4
PS	43	F	11	1	19.7	3.9	96	1.79	1.22	147.7	1.8	high	8.7	4.1	0.1	29	12
PS	35	F	4	1	20.1	3.7	200	1.96	1.17	133.8	1.5	high		4.7	0.13		10.7
PS	55	F	30	1	20.4	3.8	182	1.36	0.87	119.1	1.8	high		3.9	0.25	24	10.2
PS	58	M	31	1	16.1	3.9	109	1.05	1.38	90.08	1.6	high	9	5.1	0.1		10.4
PS	58	F	24	1	23.6	3.7	137	1.34	0.8	133.3	1.8	high	9.2	5	1.9	29	10.8
PS	71	F	2	1	18.4	3.7	139	2.19	1.16	125.4	1.5	high	8.9	2.8	0.47	33	10.1
PS	46	F	2	1	15.8	4.2	194	1.62	0.88	107.6	1.1	high	9.1	4.8	0.77	2	12.4
PS	72	M	4	1	20.3	4.3	141	1.58	0.7	97.41	1.8	high	7.4	4.7			11.6
PS	77	M	6	1	23	3.9	181	1.22	1.04	125.6	1.6	high	8	5.1	0		9.8
PS	43	F	4	1	21.2	4	67	1.1	0.99	111.5	1.3	high	8.5	2.5	0.06	20	10.8
PS	69	F	10	1	23.2	4.1	176	1.78	1.13	130.4	1.5	high	9.4	4.5	0.2	31	10.1
PS	51	M	11	1	17.9	4		1.13	0.95	89.55	1.8	high	9.8	1.7	3.27	26	8.6
PS	51	F	22	1	18.3	3.8	159	1.66	1.24	87.86	1.3	high		4.3	0.01	25	11.3
PS	49	M	21	1	17.8	4.4	150	1.33	1.02	101.1	1.6	high		4.5	0.03	23	10.1
PS	52	F	17	1	18.7	4.3	211	1.34	0.89	115.2	2.1	high		5.4	0.05		10.2
PS	59	F	11	1	21.1	3.6	166	1.67	0.88	107.8	1.6	high	9.8	3.9	0.05	28	9.4
PS	79	M	10	1	18.6	3.8	194	1.02	0.74	80.51	2.5	high	9	4.9	0.4	28	11.6
PS	74	F	9	1	18.6	4.1	200	1.31	0.89	123.6	1.6	high	9.4	6.3	0.3		10.9
PS	61	F	7	1	21.7	3.7	210	1.42	0.88	122.5	1.5	high	9	4.4	0.42	31	11.4
PS	64	F	7	1	20.1	4.1	148	1.45	0.76	80.24	1.5	high	9	6.2	0.65	22	9.7
PS	64	F	7	1	19.6	4	151	0.96	0.87	118.7	1.5	high	9.5	7.2		30	10.4
PS	68	M	7	1	17.3	4.5	169	1.32	0.87	36.55	1.3	high	9.5	5.8	0.3	27	9.4
PS	35	F	6	1	24.2	3.7	94	1.18	0.74	102.3	1.6	high	7.3	5.2	0.09	28	9.6
PS	59	F	6	1	20.6	3.9	210	1.57	0.87	91.34	1.6	high	9.2	4.4	0		10.8
PS	68	M	5	1	20.5	3.9	195	1.54	1.02	107.1	1.8	high	8.2	3.6	1.66	28	7.8
PS	66	M	2	1	21.4	3.8	110	1.38	1.1	79.2	2.1	high		4.9	0.22	32	10.4
PS	69	F	2	1	18.2	4.2	160	1.06	0.98	109.1	2.1	high	7.8	3.4	0.11	27	10.2
PS	60	F	8	1	21.1	3.9	152	1.64	1.2	109.9	1.8	high	8.8	9.2	0	30	12.2
PS	66	M	35	1	19.7	4.2	127	1.48	1.1	71.11	1.3	high	8.1	5.7	0.04	28	10.1
PS	74	M	20	1	20.8	4.2	96	1.28	0.94	74.36	1.5	high	7.7	2.2	0.8	27	10.8
PS	44	M	16	1	16.9	3.8	125	0.96	0.85	97.27	1.6	high	10.9	8.5		47	11.6
PS	55	F	12	1	27.6	3.7	109	1.37	1.28	80.13	2.1	high	10	6			
PS	61	F	11	1	26.5	4.1	90	1.42	0.8	111.1	1.8	high	9.6	4.7	0.05	24	10.9
PS	81	F	9	1	18.3	4.1	154	1.63	0.93	91.85	2.1	high	8.6	8.3	0.02	28	10.4
PS	83	M	6	1	23.4	3.9	144	1.85	0.75	107.4	1.6	high	9.7	6.4	2.14	32	10.7
PS	61	M	6	1	22.8	4.2	112	1.84	0.85	93.8	1.8	high	9.4	3.7	0.05	31	10.7
PS	66	F	5	1	18.1	3.8	207	1.76	1.06	100.3	1.6	high	8.4	5.3	0.11	26	11
PS	65	M	5	1	19.6	3.9		1.34	1.32	95.23	2.1	high	7.3	1.9			11
PS	72	M	5	1	19.3	3.9	110	1.33	0.82	61.95	1.3	high	8.8	5.4	0.07	27	11.1
PS	72	M	5	1	17.4	3.7	142	1.31	0.83	55.54	1.8	high	8.5	4.2	0.9	28	8.6
PS	80	M	4	1	18.2	3.4	153	1.44	0.89	90.56	2.1	high	8.2	5.1	0.13	32	10.4
PS	82	F	4	1	19.5	4		1.32	0.85	102.2	1.5	high	9	3.7	4.54		10.5
PS	51	M	2	1	24.4	4.2	152	1.57	0.93	99.97	1.3	high		4		19	10
PS	69	M	2	1	28.4	3.9	107	1.01	1.22	120.3	2.1	high		3	2.5	22	7.1
PS	59	M	9	1	23.6	4.1		1.51	1.01	117.3	1.5	high	9.3	6.8		30	9.8
PS	77	M	6	1	21.2	3.6	147	1.23	0.7	77.47	1.8	high		4.5	2.99	22	7.3
PS	56	M	4	1	25.4	3.8	135	1.41	0.94	128.7	2.1	high	8	3.1	0.16		12.4
PS	66	M	3	1	21.8	3.8	133	1.27	1.06	80.34	1.6	high		3.4	0.15	14	10.6
PS	66	F	17	1	18.3	2.8	132	1.65	0.47	55.37	1.5	high	7.9	2.2	0.3		10.1
PS	72	F	6	1	17.6	3.5	140	1.46	0.86	103.8	1.3	high	9	4	0.05	33	10.8
PS	42	M	9	1	23.9	4.1		1.65	0.88	104.9	1.6	high		4.6	0.1	29	10.3
PS	68	M	4	1	22.5	3.7	187	1.46	0.91	83.17	1.6	high		3.5	0.18	18	10.6
PS	75	M	2	1	20.8	3.3	104	1.33	0.67	29.82	2.1	high	9.1	5.4	0.1	21	10.7
PS	61	M	2	1	20.2	3.7	163	2.08	1.19	79.69	1.5	high	9.4	5.7	0.3	28	9.4
PS	31	F	6	1	20.5	4.1		1.44	0.53	94.3	1.5	high	7.6	3.6	20.88		9.8
PS	64	M	4	1	25.4	3.9	117	1.46	0.95	118.9	2.1	high	8.5	5	3.9	33	10.4

PS	77	M	5	1	18.6	3.7	182	1.7	0.98	95.49	2.1	high	10.7	6.6	0.08	36	11.3
PS	57	F	14	1	21.1	3.5		1.48	0.7	92.73	1.9	high		6	0.1	30	9.2
PS	43	F	4	1	22	4.1	170	1.6	1	80.51	1.6	high	9.1	3.7	0.1	21	10.3
PS	61	F	4	1	22.6	4	131	1.86	0.61	113.6	1.6	high	8.5	6.9	0.1	23	10.1
PS	37	F	3	1	31.7	4.1		2.06	0.91	92.76	1.5	high	9.5	4.2	0.03	29	7.7
PS	61	M	6	1	24	3.8	169	1.74	0.78	95.04	1.8	high		4.8	0	17	10.3
PS	68	M	5	1	16.6	3.5	98	1	0.91	95.37	1.8	high		6.8	0	24	10.6
PS	50	M	7	1	19.6	4	103	1.4	0.85	107.1	1.8	high		6.4	0.11	29	11
PS	71	F	10	1	20.1	3.8		1.54	0.7	93.78	1.6	high		7	0.1	31	9.2
PS	76	F	7	1	20.9	3.5	119	1.41	1.19	94.24	1.6	high		5.2	0.09	31	9.5
PS	68	F	6	1	19.4	3.7	121	1.17	0.66	93.73	1.5	high	9	6.1	0.2	16	12.2
PS	68	F	5	1	21.5	3.8	184	1.61	0.8	108	2.1	high		5.8	0.03	23	10.3
PS	60	M	2	1	27.4	3.9	127	1.17	1.03	105	2.1	high		5.3	0.01	25	11.6
PS	55	F	16	1	17.1	3.7	106	2.17	0.84	116.6	2.1	high	9.6	7	0.3	19	10.4
PS	73	F	3	1	18.9	3.6	141	1.66	0.91	73.17	1.8	high		4.4			4.9
PS	57	M	4	1	24.3	3.8	137	1.87	1.21	121.6	2.1	high		6.2			8.6
PS	34	M	3	1	26.7	3.9	173	1.38	1.14	81.39	2.1	high		5.5			6.9
PS	44	M	9	1	22.9	3.6	187	1.79	0.93	96.85	1.8	high		8.3			8.7
PS	79	F	4	1	18.2	3.6	154	1.52	0.93	122.2	2.1	high		7.7			9.7
PS	57	M	3	1	24.3	4.2	135	1.54	1.38	104	1.8	high		6.7			11.3
PS	52	M	2	1	19.9	3.9	180	1.49	0.81	98.24	2.1	high		7.6			8.8
PS	67	F	26	1	20.8	3.5		1.1	0.95	106.5	1.6	high		7.7	0.3	34	12.9
PS	68	M	5	1	20.4	3.7		1.78	1.1	118.7	1.9	high		5	1.5	30	10.9
PS	33	M	4	1	18.3	4	115	1.37	0.85	92.33	1.8	high		5.9			9.3
PS	56	F	3	1	32.4	4	145	1.26	1	82.47	1.8	high		5.7			7.2
PS	67	F	13	1	22.8	3.4	154	1.47	1.07	96.69	1.8	high		5			11.1
PS	66	M	12	1	16.2	4.3		0.73	0.74	110.2	1.1	high		5.3	0.02	24	8.9
PS	75	M	12	1	19.9	3.7	135	1.48	0.97	132.9	2.1	high		5.1			10.1
PS	58	F	11	1	20	3.8	148	1.36	0.87	108.5	2.1	high	8.9	5.6	0.28	36	11.1
PS	48	M	10	1	17.8	3.9	134	1.31	1.14	106.2	2.1	high	8.2	3.5	0.01		11.2
PS	72	M	9	1	23.1	3.8	179	1.5	0.76	139.2	1.5	high	9	7	0.18	26	12.4
PS	78	F	7	1	19	3.6	151	1.63	0.72	103.7	2.1	high		5			9.2
PS	46	F	4	1	24.1	4.3	108	1.51	1.06	86.85	1.5	high	8.9	5.7	0.2		10.8
PS	76	M	3	1	20	3.9		2	0.87	134.3	1.6	high		5	0.1	33	10.4
PS	53	F	2	1	19.6	3.6	181	1.68	0.92	112.7	1.5	high		5.4	0.05	25	11.5
PS	41	M	2	1	29.6	3.5	149	1.09	0.72	79.77	1.1	high	8.9	6.3	0.44	35	9.9
PS	56	F	14	1	15.9	4		1.16	1.14	121.2	1.6	high		9.1	0.1	24	10.4
PS	73	M	9	1	22.7	3.9	152	0.72	1.08	142.8	1.6	high	9.6	4.8	0.2	21	8.7
PS	57	M	2	1	20.3	3.8		1.79	0.64	31.2	2.1	high	7.9	6.3	4.53		12.3
PS	63	M	10	1	24.2	3.8	168	1.2	1.24	132.7	2.1	high	8.5	5.9	0.08		10.9
PS	39	M	8	1	40.4	3.7		1.57	0.96	93.89	2.1	high	9.2	5	12.36		11.8
PS	74	M	2	1	18.7	3.1		1.46	0.83	101.2	1.8	high	9.2	4.8		26	10
PS	57	M	11	1	22.4	3.9		1.64	1.03	132.8	1.6	high		4.1	0.1	33	8.8
PS	73	M	8	1	20.9	3.6	170	1.56	0.99	129.5	1.5	high	9.1	6.4	0.33	28	10.4
PS	50	F	11	1	18.1	3.8	120	1.79	0.89	109.5	2.1	high	9	2.1	3.6	20	9.8
PS	64	F	8	1	19.1	3.9	129	1.56	1	100.6	1.5	high	8.5	5.2	0.8	26	10.5
PS	58	M	2	1	21.2	4	142	1.41	1.07	108.8	1.8	high	8.9	10.1	0.05	25	10.5
PS	27	F	7	1	19.2	4.2		1.6	0.96	82.1	2.1	high	9.1	5.4	14.17		10.7
PS	67	M	4	1	19.4	4	213	1.87	1.06	86.74	1.5	high	10.1	5.5	0.1	35	10.4
PS	78	F	3	1	20.2	3.8	126	0.87	0.88	108.3	2.2	high	13.4	4.1	0.08	18	8.3
PS	78	M	9	1	24.4	3		1.01	0.97	123.9	1.6	high		4.7	0.4	30	10
PS	82	F	5	1	15.8	3.3		1.12	1.09	81.89	1.6	high		4	0.1	19	14.8
PS	69	F	12	1	16.8	4	141	1.08	0.96		1.8	high		7.7		22	11.1
PS	72	F	2	1	20.1	3.4		1.28	0.96	89.75	1.6	high		7.3	0.1	31	16.6
PS	73	F	3	1	20.8	2.9	93	1.72	0.71	57.25	2.1	high	9.2	5.8	0.01	28	11.1
PS	56	M	3	1	19.3	3.8	120	1.1	0.93	117.2	1.8	high	8.3	7.4	0.03	22	10.8
PS	75	M	2	1	18.2	4	139	1.76	0.76	102.8	1.5	high	9.7	5.2	0.09	41	10.4
PS	59	M	5	1	22.5	4.1	123	1.5	0.77	103	2.5	high	10.2	4.6	11.87	30	12.9
PS	47	M	30	1	20.2	3.7	170	1.83	0.81	95.29	1.5	high	10.1	6.3	0.4	33	11.2

PS	71	F	10	1	25	3.8	106	1.77	0.6	107	1.5	high	8.8	4	0.05	15	10.9
PS	72	M	2	1	25.4	4.3	138	2.07	0.77	86.64	1.3	high	10.5	6.3		28	12.1
PS	82	F	2	1	16.5	3.5	179	1.46	0.75	127.3	2.1	high	8	5.6	0.26	30	9.7
PS	79	M	2	1	20	4	161	1.1	0.8	117.7	2.1	high		3.9	0.01		10.5
PS	63	M	3	1	21.9	3.9	182	1.24	0.63	118	2.1	high		6.1	0.03		10
PS	78	M	4	1	16.6	3.9	151	1.41	0.69	104.5	1.8	high	9.7	5.4		23	11.9
PS	68	M	6	1	24.9	3.9	218	1.16	0.99	136.5	2.1	high	9	5.5	0.39	26	12.7
PS	63	M	9	1	20.2	4.1	175	1.33	1.05	113	2.1	high	9	7.8	0.05		10.8
PS	60	M	13	1	19.1	4.1	201	1.63	1.23	92.03	1.8	high	9.1	6.1	0.2	28	10.9
PS	63	F	15	1	15.9	3.9	126	1.48	0.9	117.6	2.1	high	9.4	4.3	0.1		10
PS	38	M	12	1	27.3	3.9		1.28	1.02	101.2	1.6	high		4	0.1	26	9.2
PS	51	M	6	1	19.1	4	135	1.11	0.82	94	2.1	high	10.4	5.4	0.05	25	8.7
PS	69	F	34	1	21.8	3.9	154	0.95	1.01	150.4	1.8	high		3.9	0.01	23	9.6
PS	47	M	21	1	20.6	4.1	172	2.14	1.07	109.2	1.3	high	8.8	4.4		22	10.2
PS	52	M	15	1	24.4	3.8	138	0.88	0.63	91.69	2	high	10.1	5.1			10
PS	60	F	27	1	21.1	3.4	199	1.38	0.93	107.3	1.8	high	9.1	6.6	0.05		9.6
PS	62	M	19	1	19.6	4.2	187	1.4	0.94	99.02	1.5	high	9.2	8.4	0.4	36	10.2
PS	37	M	12	1	22.1	3.9	153	1.17	0.98	128.9	1.5	high	9.1	5.1	0.11	26	11.6
PS	73	F	5	1	20	3.8	168	1.49	0.97	111.1	1.8	high		5.6	0.11	25	11.1
PS	64	F	20	1	18.2	3.8	137	1.86	1.13	104.1	1.8	high	8.9	4.4			10.1
PS	77	M	4	1	24.3	3.3	142	1.06	1.03	97.12	1.6	high	9.5	5.9	0.13	29	9.1
PS	67	F	21	1	18	3.7	107	1.49	0.95	103.7	1	high	9.5	3.6	0	21	8.3
PS	60	M	9	1	16.9	3.8	129	1.14	0.89	108.3	1.3	high		3.2	0.1	17	10.1
PS	71	M	5	1	17.3	4	112	1.49	1.18	104.8	1.5	high	8.7	3.2	0.04	27	10
PS	63	F	34	1	17.3	3.9	130	1.53	0.97	73.93	1.6	high	8.9	4.6	0.18	33	10.2
PS	55	M	33	1	16.2	3.5		1.29	1.11	91.86	1.9	high		5.3	0.1	28	10.9
PS	58	M	32	1	20	3.7	144	1.13	0.76	104.9	1.9	high	9.4	5	0.2	37	10.5
PS	77	M	3	1	20.3	3.3	144	1.42	1.25	124	1.5	high		5.8	0.3	32	10.6
PS	58	F	4	1	21.8	3.8	158	1.84	0.78	115.1	1.8	high	9.9	4.6	0.1	32	11.3
PS	66	M	6	1	20.1	4.1	114	2.37	1.09	73.81	1.6	high	8.5	4.4	0.05	28	9.7
PS	62	M	12	1	17.8	3.6	132	1.08	0.77	106.5	2.1	high		5.7	0.11		8.4
PS	73	M	9	1	20.8	3.7	126	1.57	0.71	93.33	1.8	high	9.2	2.4			10
PS	67	M	15	1	22.8	3.4	111	1.27	0.8	86.85	1.6	high		4.9			9.4
PS	53	M	12	1	18.7		159	1.67	0.99	93.13	1.9	high	9	6.1	0.64	28	10.8
PS	35	M	5	1	20.6	4.2	136	1.3	1.19	111.8	1.6	high	9.5	6.8	0.25	28	9.6
PS	56	M	8	1	24.8	3.4	146	2.07	0.6	111.5	1.3	high	9.2	2.6	0.05	23	9.9
PS	59	M	27	1	16.9	4.6	116	1.23	0.95	115.6	1.8	high		3.5	0.16	22	8.2
PS	65	M	13	1	20.4	4.1	126	1.32	0.78	135.1	2.5	high	9	5	0.92	32	10.8
PS	56	M	8	1	19.7	4.6	148	1.56	0.78	125.2	1.5	high	9.8	6.3	0	22	9.6
PS	79	F	20	1	16.5		169	1.64	0.98	89.06	1.8	high	8.9	4.5	0	23	9.9
PS	61	M	2	1	21.3	2.6	143	1.06	0.92	118.9	1.8	high	9.4	4.1	0.57	27	11.4
PS	78	M	2	1	24.6	3.8	153	1.24	1.25	105.3	1.5	high	9.6	6.2	0.8	31	9.9
PS	60	M	18	1	25.3	4.3	176	2.03	0.84	117.5	2.1	high	9.3	4		28	10
PS	39	F	5	1	15.4	4.3	136	1.51	0.98	78.76	1.3	high	9.2	4.6	0.07	26	11.4
PS	46	M	7	1	23	3.5	143	1.56	0.95	111.8	2.1	high	8.8	5.3	0.78		10
PS	43	F	18	1	20.2	3.9		1.35	0.9	107.8	2.1	high	8.4	5.1	0.1	23	11.6
PS	76	F	4	1	17.6	3.8	200	1.55	0.97	107.9	1.8	high	8.7	4.1	0.1	28	10.8
PS	66	F	27	1	17.7	3.8	142	1.32	0.91	86.64	2.1	high		4.6	0.52	78	13.3
PS	41	M	2	1	31.9	4.1	132	1.56	0.93	88.84	1.5	high	8.9	5.6	0.6	29	10.9
PS	60	F	5	1	21.9	3.9	149	1.87	0.9	106.2	1.6	high	10.1	5.8	0.05	27	12.1
PS	67	M	2	1	21.8	3.6	167	1.39	0.84	69.79	2.1	high	8.7	5.9	0.05	25	10.2
PS	47	M	8	1	21.9	4.3	150	2.18	0.77	94.55	1.6	high	9.5	4.2	0.05	29	10.4
PS	36	M	5	1	34.6		157	1.38	1.14	109.7	1.6	high	9.3	4.3	0.07	23	9.5
PS	54	F	3	1	17.7	3.3	109	1.78	0.92	94.81	1.6	high	7.6	5.8	0.69	32	9.9
PS	64	M	2	1	20.7	4.1	154	1.58	1.19	100.7	1.8	high	9.7	5.5	0.1	28	10.6
PS	70	F	10	1	19.6	3.8	207	1.58	0.72	123.5	1.8	high	9.6	4.8	0	29	11.1
PS	66	F	16	1	26.3	3.5	149	1.76	0.92	129.9	2.1	high	9.8	6.6	0	24	10.5
PS	66	M	7	1	23.6	3.9	222	1.43	1.01	90.07	1.5	high	8.3	4.9	0.1	24	9.8
PS	71	F	18	1	18.7	3.7	115	1.37	1	125.2	2.5	high	9.9	5.7	0	33	10.8

PS	59	F	20	1	17.6	3.5	165	1.44	1.01	124.5	1.5	high	10.6	6.1	0	25	10.9
PS	61	F	12	1	20.3	3.5	197	1.75	0.96	151.1	1.5	high	9.7	2.8			12
PS	56	F	5	1	20.9	3.6	189	1.62	0.8	84.54	1.5	high	9.8	5	3.1	32	8.4
PS	60	F	9	1	21.4	3.2	128	1.21	0.99	138.7	2.1	high	8.9	6.5	0	18	10.5
PS	42	F	8	1	18.6	3.9	214	1.24	0.94	125.2	1.5	high	10.2	5.6	0.4	31	10.4
PS	54	F	18	1	20.9	3.6	128	1.3	1.32	103.3	1.5	high	9.2	7.3			11.1
PS	66	M	4	1	30.5	3.9	122	1.73	1.05	93.52	1	high	8.9	2.6	0.16	31	8.9
PS	59	M	4	1	26.3	3.8	158	1.51	0.74	108.7	2.1	high		6.1	0.15	32	9.8
PS	67	F	2	1	22.2	4.3	166	1.36	0.81	80.47	2.1	high		6	2.51	27	10.3
PS	35	M	11	1	18.6	4.4	159	1.69	1.05	95.84	1.5	high	9.5	5.7	0	32	10.1
PS	60	M	9	1	22.8	3.9	213	1.86	1	144	2.1	high		6	0.02	22	10.9
PS	68	F	9	1	18.7	3.6	143	1.6	1.19	97.32	1.5	high	9.1	5.8	0.2	20	11.7
PS	67	M	4	1	20.2	3.9	147	1.45	1.08	77.3	1.5	high	9.4	6.2	0.18	27	12.3
PS	66	M	5	1	25.8	3.5	157	1.76	0.96	112.5	1.9	high	9.1	5.1	0.13	18	11
PS	54	F	17	1	19.6	3.7	119	1.49	1.06	124.5	1.3	high	8	4.9	0.08	28	10.5
PS	75	M	3	1	20.8	3.9	181	1.64	0.92	91.39	2.1	high		8.1	0.11	29	10
PS	38	M	13	1	19.5	4.1	168	1.93	0.97	106.5	1.6	high	10.4	6.6	0.05	28	11.7
PS	32	F	9	1	17.4	4.6	172	1.33	0.87	65.43	1.9	high	9.1	4.9	1.69	23	8.9
PS	69	F	23	1	16.6	4.2	131	1.64	1.12	97.41	1.9	high		3.8	0.28	17	8.2
PS	70	F	18	1	20.5	3.8	201	1.86	1	108.7	2.1	high		3.2	0.05	28	10.7
PS	38	M	10	1	18.9	4	116	1.21	1.34	108.9	2.1	high	9.2	5.9	0.1	32	10.5
PS	51	F	8	1	23.9	4.1	152	1.41	0.8	120.3	1.8	high	10.5	4.4	0.09	25	9.8
PS	31	M	3	1	20.3	4.1		1.85	1.33	105.4	1.3	high	8.7	6.4			10.9
PS	60	F	16	1	26.4	3.9		2.11	1.13	115.8	1.3	high	9.7	3.7			12.6
PS	60	F	3	1	20.8	3.9	198	1.36	0.74	71.96	2.1	high	8.6	6.6	0		9.7
PS	55	M	7	1	35.3	3.9		1.16	0.89	82.39	1.8	high	10.2	4.8		23	10.2
PS	41	M	16	1	21.8	4.4		1.7	0.83	135.7	1.3	high	9	3.4			10.9
PS	74	M	13	1	21	3.5	127	1.4	0.84	99.09	1.6	high	9.7	5.8	0.23	29	10.1
PS	54	M	9	1	21.5	4	168	1.61	1.33	109.9	1.5	high	9.5	5.7		36	9.9
PS	55	M	29	1	16.6	3.7	167	1.31	0.99	98.82	1	high	7.8	4	0.31	27	9.9
PS	72	M	4	1	25.3	3.8	143	1.05	0.76	76.44	1.5	high	8.1	9	0.18	30	7.6
PS	65	F	14	1	30.3	4	100	1.51	1.01	84.68	1.8	high	9.2	7.1	0.01	29	13.6
PS	55	M	4	1	20.1	4.1		1.36	1.22	104.3	1.5	high		5.6	0.07		10.1
PS	67	F	4	1	15.2	3.8	191	1.38	0.9	62.14	2.1	high	9.9	6.4	0.06	30	11.1
PS	58	M	34	1	18.8	3.5	171	1.14	0.57	91.04	1.8	high	9.4	6	0.16	26	13.2
PS	57	M	25	1	23.1	4.1	152	1.11	1.22	107.3	1.8	high	9.6	4.9		25	8.1
PS	44	F	10	1	18.5	4	190	1.32	1.27	118	1.3	high	8.2	4.5	0.1	37	9.9
PS	80	M	7	1	16.5	3.8	175	1.49	0.92	93.43	1.8	high	9.9	7.2	0.2	27	11.6
PS	48	M	6	1	25	4.2	142	1.6	0.75	86.68	1.8	high	8.7	6.8	0.2	29	10.6
PS	59	F	6	1	20.3	3.5	170	1.5	0.88	58.41	1.5	high		3.9	0.09	43	10.1
PS	87	F	4	1	15.9	3.9	161	1.13	0.75	71.95	1.3	high	9	5.2	0.06	26	10
PS	73	F	8	1	19.8	3.4		1.3	0.8	88.01	1.1	high		4.2	0.65	43	11.5
PS	69	M	6	1	18.8	3.9	138	2.18	0.69	80.37	1.5	high	10.7	4.9	0.1	32	8.9
PS	60	M	27	1	16.8	4.1	126	1.27	1	69.32	1.6	high		4.6	0.04	31	8.8
PS	68	F	25	1	17.2	4.1	117	1.42	0.92	103.4	1	high	10.9	6.3	1.43		9.8
PS	42	F	21	1	23	3.6	209	1.34	0.94	107	1.5	high	8.9	6.7		29	10.1
PS	62	M	22	1	19.4	3.8	143	1.37	0.67	77.27	1.4	high	8.8	4.1	0.18	22	11.3
PS	77	F	5	1	17.1	3.4	174	1.43	1.19	98.62	2.1	high	8.5	5.6	0.08		
PS	72	M	4	1	16.8	3.6	192	0.9	0.92	118.2	1.8	high		8.6	0.62		10.7
PS	57	M	36	1	17.6	4.1	143	1.36	1.04	72.3	1.5	high	9.1	3.8	0.13	28	9.6
PS	61	M	28	1	18.1	4		1.4	1.5	78.35	1.4	high	8.5	2.3	0.08	31	11.6
PS	85	F	2	1	21	3.9	158	1.55	1.11	94.05	1.5	high	9	5.9	0.1	32	9.7
PS	38	F	2	1	17.2	4.3	165	1.12	0.89	110.7	1.8	high	8.2	4.7	0.6	30	10.3
PS	49	F	3	1	19.1	3.9	134	1.41	1.15	53.46	1.5	high	8.8	4.8		30	8.7
PS	41	F	6	1	24.1	4	167	1.23	0.87	97.45	1.8	high		6.3	0.1	19	10
PS	57	M	9	1	23.2	4.5	139	1.47	1.18	115.9	1.8	high		4.5		30	10.8
PS	57	M	35	1	17.8	4.1	160	1.12	1.32	95.28	2.2	high		3.6	0.05	27	9.4
PS	56	F	27	1	17.2	4	136	1.18	1.37	90.79	2.2	high	8.4	6.6	0.03	17	10.6
PS	57	M	30	1	19.6	4.2	174	1.25	1.01	118.2	1.8	high	10.4	4.1	0.78	20	10.4

PS	83	F	3	1	17.9	3.6	131	1.01	1.21	24.42	1.8	high	9.2	5.3	1.49	27	9.5
PS	71	M	7	1	18.6	3.4	225	2.27	0.78	92.54	1.8	high	9.4	5.3		33	11.2
PS	46	M	2	1	24	4	229	1.09	0.63	30.58	1.8	high	7	7.1	0.29	38	8.9
PS	56	M	30	1	21.3	4	167	0.98	1.13	69.26	2.2	high	9	7.1	0.3		11.7
PS	65	M	22	1	17.1	4	150	1.52	1.09	85.49	1.8	high	10	4.6			11.8
PS	67	M	20	1	18.2	3.7	141	0.97	1.03	102.2	1.8	high	9.8	8.5	0.13		10.9
PS	68	F	30	1	24.6	3.9	180	1.73	0.82	83.88	1.5	high	9.2	3.9	0.3	31	10.6
PS	59	M	13	1	20.9	4.1	163	1.41	0.85	109.6	1.9	high	8.4	4.6		19	9.2
PS	45	F	10	1	23	3.8	150	1.37	0.93	116.7	1.8	high	8.7	4.7	0.05	13	10
PS	73	M	5	1	21	4.2	125	1.44	0.9	124.8	2.1	high		5.8			9.7
PS	73	F	4	1	26	4.2	132	1.32	0.8	76.9	1.8	high	9.3	4.9	0.2	29	10.2
PS	51	M	3	1	25.1	3.9	174	1.35	0.66	89.27	2.1	high	9.6	7	0.96	35	9.9
PS	48	F	22	1	17.6	3.9	189	1.07	1.12	110.1	1.1	high		5.1	0.47	18	10.3
PS	54	F	14	1	17.6	3.9		1.57	0.81	104.8	1.8	high		3.9	0.05	25	9.6
PS	59	F	11	1	22.8	4.7	148	1.33	0.96	131.7	1.5	high	9.3	5.7	0.03	26	10.7
PS	73	F	10	1	26.2	4.1	178	1.28	1.25	155.1	2.1	high		4.7	0.44		11.4
PS	71	M	7	1	20.9	4.2	100	1.79	0.97	141	1.8	high	7.3	3.5			10.5
PS	61	M	26	1	22.6	3.9	163	1.2	0.92	106.8	2.1	high	9.1	5.6	1.08	33	10.9
PS	66	M	3	1	25.7	3.8	184	2.12	0.95	92.85	1.3	high	8.9	6.9	0.04	26	10.8
PS	64	F	2	1	26.8	4.5	148	2.18	1.27	106.9	2.1	high	8.6	5	0.03	24	9.5
PS	52	M	17	1	17.9	4.2	155	1.83	1.2	116.6	1.3	high	8.6	5.4	0.27	29	11
PS	74	F	12	1	17.7	4.2	159	1.71	1.35	93.76	1.3	high	9.4	5.9	0.4	22	10.4
PS	52	M	5	1	23.2	4.1	167	1.55	0.91	103.1	1.5	high	9.2	5.3	0.1	19	11.8
PS	78	M	4	1	20.7	4	147	1.62	0.82	104.5	1.8	high	8.1	4.1	0.03	29	
PS	67	F	4	1	25.4	4.4	184	2.18	1.02	112	1.3	high		6.4	0.77	23	11.8
PS	67	M	4	1	26.7	3.6	154	0.95	1.11	123.9	1.9	high	7.2	4.3		29	9.9
PS	62	F	25	1	19.5	4.2	156	1.34	1.08	120	2.1	high	9.3	3.9	0.05	32	9.5
PS	67	M	2	1	22.1	4.1		1.17	0.77	93.66	1.8	high	7.6	7.2			9.7
PS	63	M	4	1	18.8	4	129	1.4	0.85	123.3	1.8	high	9.2	4.3	0.12	24	13.9
PS	68	M	4	1	22.5	4.5	161	1.72	0.84	127.5	1.5	high	8.7	7	0.2	40	12.9
PS	78	M	4	1	22.2	3.9	180	1.46	0.64	122.2	1.3	high	10.5	9.2	0.55	16	10
PS	49	M	3	1	28.4	4.2	131	1.15	0.83	119.4	1.8	high	8.2	3.9	0.53	12	12.6
PS	56	F	2	1	23.6	3.9	153	1.51	1.31	99.41	1.5	high	9.5	4.5	0.03	16	10.1
PS	68	F	14	1	21.9	3.9	122	1.95	0.9	89.01	1.3	high	9.2	1.1	1.11	31	8.4
PS	58	F	9	1	18.6	4.3	154	1.27	0.65	106.4	1.8	high	12.2	7.1		27	12.8
PS	72	M	5	1	18.8	4.1	240	1	0.89	108.1	1.5	high		5.3	3.16	22	9.8
PS	68	M	11	1	21.3	4.4	151	1.48	1.07	130.8	1.3	high	7.9	6.5	0.2	20	10.8
PS	66	M	3	1	22	4.1	168	1.16	0.75	74.04	2.1	high		3.3	0.12	26	11.7
PS	71	F	2	1	18.3	4.1	138	1.45	0.8	110.6	1.6	high	8.9	4.9	0.02	21	9.1
PS	59	M	10	1	21.9	3.6	158	1.27	0.95	113.2	2.1	high	9.4	6.1	0.25	28	11.8
PS	48	M	15	1	18.7	4	112	1.27	1.04	121.5	1.5	high	9.2	7	0.05	23	9.7
PS	60	F	10	1	17.8	4.1	168	1.52	1.06	115.8	2.1	high	8.1	4.2	2.4	28	7.5
PS	57	F	4	1	22.8	4.5	190	1.75	0.99	113.9	1.3	high	8.1	4.6	0.06	28	10.3
PS	53	M	4	1	21.3	4.1	122	1.71	1.16	106.8	1.8	high	10.1	5.2	0.06	24	11.6
PS	65	M	12	1	21.2	3.8	151	1.27	1.25	109.9	1.3	high		3.3	0.1	32	10.6
PS	73	F	6	1	23.1	4	119	1.31	1.3	113.3	1.9	high	9.1	9.5	0.26	25	10.6
PS	65	M	7	1	21.9	4.1	185	1.62	1.1	96.69	1.6	high	9.7	9.5	0.23	27	12.5
PS	84	M	5	1	22.6	3.7	226	2.24	0.97	116.1	1.8	high	8.4	5.1	0.1	24	9.4
PS	69	M	4	1	22.3	3.7	104	1.35	0.87	146.4	1.6	high	9.6	3.7	1.44	27	8.3
PS	51	F	8	1	19.6	3.8	101	1.22	0.99	131.1	1.5	high	10.9	4.2		20	10.2
PS	61	M	6	1	19.6	4	170	1.3	0.94	96.91	1.5	high	10	6.6		23	9.7
PS	73	M	8	1	24.5	4.4	112	1.12	0.89	111.9	1.6	high	8.3	5	0.03	23	10.5
PS	65	F	6	1	16	4.4	187	1.95	1.06	117.8	1.3	high	10	3.9	0.22	47	14.7
PS	68	M	4	1	18	4	128	1.29	0.96	93.69	1.5	high	8.9	4.5	0.23		7.7
PS	67	M	9	1	19.5	4.2	133	1.86	1.26	133.1	1.8	high	8.3	3.8	1.93	25	5.6
PS	74	M	7	1	21.2	3.9	134	1.29	0.75	112.1	2.1	high	8.2	5.4	0.3	21	9.2
PS	64	M	4	1	22.1	4.4	132	1.15	0.95	107.8	1.3	high	8.3	5.1	0.1	15	10.9
PS	70	M	2	1	19.6	4.3	175	1.2	0.88	107.4	1.5	high	8.6	6.7	0.3	16	10.9
PS	68	M	7	1	23.6	3.9	131	1.32	0.95	127	1.5	high	8.9	6.4	0.53	30	11.7

PS	72	F	26	1	21.7	3.9	155	1.23	0.95	103.9	2.2	high		4.7	0.02	27	11
PS	50	F	26	1	20	4.1	145	1.5	1.22	113.8	2.1	high	9.2	6.8	0.1	28	11.3
PS	64	F	25	1	26.9	3.9	173	1.85	1.16	103.6	1.3	high	9.2	5.8		25	9.9
PS	64	F	22	1	23.8	3.9	206	1.4	0.93	101.9	2.1	high	9.7	5.6	0.1	27	11.6
PS	59	F	22	1	18.4	3.9	128	1.17	1.03	119.3	1.8	high		6.4	0	24	11.5
PS	36	F	17	1	20	4.2	150	1.24	0.66	102	2.2	high	9.4	2.4	0.97	29	10.3
PS	60	M	16	1	24.8	3.8	117	1.2	0.81	124	1.8	high	8.4	6.9	0.1	37	11
PS	65	F	16	1	26.4	3.7	105	1.36	0.95	124.5	1.5	high		4.3	0	18	8.2
PS	30	M	15	1	20.4	4.2	131	1.35	1.04	118.8	2.1	high	10.9	5.3	0.2	30	10.8
PS	58	F	14	1	30.1	3.8	214	1.65	1.03	124.1	1.8	high	9	5.3	0.16	35	11
PS	74	F	13	1	17.1	3.5	155	1.97	0.96	114.9	1.4	high	9.1	5.2	3.09	18	9.7
PS	72	M	13	1	21.2	3.7	114	1.55	0.6	112.5	1.8	high	8.2	6.7	0.1	28	10.6
PS	80	M	13	1	19.7	3.3	142	1.3	0.8	117.9	2.1	high	9.8	5.9	0	41	11.8
PS	73	M	12	1	22.5	3.3	142	1.49	0.97	141.3	1.5	high	8.1	4	0.2	31	8.2
PS	60	F	12	1	21.8	3.7	147	1.25	0.79	120.3	1.8	high	10	7.1	0.39	25	11.5
PS	56	F	11	1	21.8	3.4	163	1.35	0.83	134.6	1.8	high	10.2	7.1	0.7	35	12.3
PS	35	F	10	1	18.3	3.8	144	1.29	0.8	107.9	1.5	high	9.1	5.2	0.3	30	13.3
PS	72	F	10	1	23.1	3.5	121	1.1	0.87	114.5	2.1	high	8.9	5.4	0.37	31	10.4
PS	61	F	10	1	17.1	4	117	1.39	1.2	118	1.8	high	8.6	4.3	0.1	28	10.9
PS	54	F	9	1	20.6	3.6	160	1.63	0.88	125.4	1.6	high	7.9	4.8	0.06	31	8.9
PS	61	M	9	1	20.5	3.4	158	1.44	0.81	107.6	1.3	high	7.7	4.9	0.05	24	11
PS	63	M	7	1	25.5	3.7	156	1.75	0.81	88.81	1.5	high	8.2	5.5	0.1	29	11.4
PS	52	F	7	1	17.8	3.4	173	1.62	0.9	119.4	1.8	high	8.6	4.2	0.1	25	10.1
PS	61	F	6	1	22	3.8	90	1.45	0.92	100.8	1.3	high	9.3	7.2	0.1	29	11.4
PS	57	F	6	1	19.5	3.7	167	1.29	1.08	90.14	2.1	high	8.7	5.1		29	8.9
PS	68	M	6	1	23.7	3.8	139	1.33	0.99	105.5	1.1	high	9.1	4.6		23	10.6
PS	84	M	6	1	19.1	3.9	164	1.2	0.68	73.47	1.6	high	7.7	5.6		25	8.7
PS	53	M	5	1	23.7	3.7	155	1.35	0.82	102.2	1.3	high	8.4	4.7	0.1	25	10.3
PS	52	F	5	1	20.2	4.1	180	1.72	1.56	132.3	1.8	high	9.7	3.1	0.33	42	11.3
PS	69	M	4	1	21.9	3.5	130	1.35	0.65	99.47	1.5	high	10.2	1.9	1.3	34	9.9
PS	51	F	4	1	15.9	3.7	159	1.25	0.48	106.1	1.8	high	8.2	5	0.1	25	12.2
PS	57	M	4	1	30.1	3.3	191	2.09	0.64	98.66	1.8	high	10	4.3			11.3
PS	73	M	4	1	30.1	3.8	230	1.24	0.83	121.5	1.6	high		5.2	0.1	19	10
PS	57	M	4	1	24.7	4	140	1.3	0.95	104.1	1.3	high	8.9	5.1	0.04	25	10.2
PS	64	M	4	1	22.1	3.9	139	1.93	0.83	81.16	1.5	high	9.7	5.9	0	22	9.8
PS	39	F	3	1	19.1	3.7	167	1.44	0.83	134.3	2.5	high	9.8	7.1	0.21	28	12.7
PS	66	M	3	1	22.4	3.5	201	1.69	0.89	104.9	1.8	high	10.3	4.8	0.06	34	10.9
PS	76	M	3	1	23.9	3.6	127	1.66	0.66	85.83	1.8	high	8	4.4	0.3	25	11.2
PS	55	M	3	1	22	3.9	138	1.51	0.89	95.12	2.1	high	9.7	7.3	0.17	32	8.1
PS	66	M	2	1	26.6	3.6	117	1.43	0.78	97.29	1.8	high	7.9	5	1.7	25	9.8
PS	69	M	2	1	22.1	3.8	212	1.58	1.06	99.36	1.8	high	8.9	6.8	0.25	28	10.9
PS	64	M	2	1	22.3	4	111	1.54	0.83	101.1	1.8	high	10	5.5	0.24	24	10.7
PS	72	M	2	1	23.1	3.8	144	1.18	1	126.3	2.1	high	8.9	4.7	0.17	30	10.2
PS	50	F	2	1	18.4	4.1	116	1.51	1.06	90.11	1.8	high	9.4	6.4	0.07	29	9.4
PS	61	F	2	1	32.1	3.8	134	1.5	0.85	99.36	2.1	high	9	5.1	0.1		10.8
PS	70	M	2	1	29.1	4.2	157	1.22	0.74	139.5	1.5	high	7.1	5.8	0.1	24	8.8
PS	70	F	23	1	15.8	3	120	1.37	0.97	64.46	2.1	high	10.7	7.2	0.28	31	11.1
PS	66	M	22	1	19.6	3	156	1.18	0.96	125.9	1.8	high	8.7	6.2	0.2	18	10.9
PS	55	M	17	1	19.1	3.8	141	1.23	0.95	121.4	2.5	high	9.3	4.4	0.1	27	9.5
PS	60	M	17	1	22.5	3.6	136	1.51	0.93	129.7	1.5	high	8.5	5.4	0.1	34	10.9
PS	55	F	16	1	15.9	3.5	146	1.39	0.67	96.66	1.5	high	10	6.1	0	23	10.2
PS	62	M	16	1	20.2	3.7	119	1.38	1.04	122.3	2.1	high	9.8	6.4	0.1	28	11.6
PS	35	M	14	1	18.8	4.2	111	1.21	0.99	103.2	1.5	high	8.6	6	0.2	29	9.5
PS	61	F	13	1	24	2.4	151	1.27	0.77	104.1	1.5	high	9.2	4	0.2	30	10.1
PS	71	M	12	1	22.2	3.6	169	2.04	0.82	121	1.5	high	9.6	6.8	0.1	29	8.7
PS	24	M	9	1	20.6	4.5	133	1.48	1.01	117.7	1.5	high	9	6	0.2	24	12.5
PS	67	M	9	1	22.8	3.4	176	1.73	0.62	129.8	1.5	high	8.4	6.1	0.1	33	9.4
PS	52	M	8	1	19.5	3.5	147	1.31	0.72	124.3	2.1	high	8.6	7.3	0.3	26	10.4
PS	76	F	8	1	19.6	3.3	210	1.35	1.13	122.8	1.5	high	8.8	4	0	20	10.5

PS	22	F	8	1	25.7	4.3	208	1.52	0.81	122.1	2.1	high	9.3	8.3	0	23	10.5
PS	64	M	7	1	17.7	3.4	196	1.49	1.06	123.5	1.1	high	8.3	6.9	0	25	9.6
PS	32	M	7	1	19.9	3.8	116	1.09	0.96	100.3	2.5	high	8.6	9.1	0	44	10.5
PS	64	M	6	1	17	3.9	149	1.49	1.18	110.2	1.5	high	8.9	6.9	0	31	11.5
PS	47	M	6	1	18.8	3.6	145	1.39	0.78	91.55	2.1	high	8.2	6.5	0.1	20	9.4
PS	33	M	5	1	25.7	4.7	150	1.23	0.67	81.45	2.1	high	9.3	4.1	0.27	23	11.2
PS	62	M	4	1	23.1	3.5	145	1.48	0.99	107.5	2.1	high	9	8.4	0	28	9.6
PS	41	F	3	1	22.9	3.7	206	1.19	1.07	130	2.1	high	7.9	9.8		29	7.4
PS	58	M	3	1	21.4	3.5	142	1.42	0.76	71.24	2.1	high	8.7	6.4	0.1	26	11.5
PS	55	M	3	1	20.8	3.4	128	1.68	1.13	127.6	1.6	high		4.2		19	10.5
PS	61	M	3	1	20.2	3.5	187	1.43	0.61	78.86	1.5	high	10.1	4.5	0.02	31	10.5
PS	47	M	3	1	24.6	3.6	95	1.21	0.8	118.6	1.5	high	8.5	5.1	0	27	10.4
PS	69	F	3	1	19.3	2.3	173	1.24	0.52	55.28	1.6	high		5.7	0.1	22	10.7
PS	46	F	2	1	29.6	3.4	133	1.24	0.75	89.46	2.1	high		4.6	4.21	24	9.8
PS	66	M	2	1	16.6	3.5	144	1.53	0.89	61.09	2.1	high	9	5.8	0.1	26	9
PS	36	M	2	1	18.2	3.8		1.12	0.79	97.51	1.6	high	9.3	4.2		15	8.2
PS	76	M	2	1	23.1	3.1	152	1.27	1.01	125.5	1.6	high	9.4	7.1	0.06	29	11.6
PS	69	F	18	1	16.5	4.2	275	0.79	0.8	115.9	1.3	high	8.1	6.8		40	9.9
PS	64	F	17	1	18.6	4.1	169	1.26	1.02	155.6	1.5	high	8.7	4.3	0.4	16	11.7
PS	67	M	15	1	17.2	4.3	165	1.22	0.95	93.24	1.8	high	9	7	0.2	24	11
PS	66	F	15	1	17.6	3.6	177	1.08	1.07	116.7	1.8	high	8.8	4.5	0.1	24	10.4
PS	75	M	13	1	20	3.5	107	1.85	0.68	89.06	1.5	high	8.1	9	0	42	12.4
PS	72	F	12	1	21.9	3.4	165	1.46	0.83	131.3	1.5	high		6	0.05	20	11.4
PS	74	F	12	1	20.4	3.7	190	1.36	0.76	106	1.3	high	8.3	6.2	0.03	23	10.2
PS	67	F	12	1	13.1	3.8	180	1.51	0.75	67.52	1.5	high	8.7	8.7	0.07	25	7.3
PS	66	F	11	1	21.4	3.4	187	1.59	0.82	98.8	1.5	high	9.6	4.7	0.17	26	9.6
PS	60	F	11	1	21.5	4.1	171	1.7	0.86	107.1	1.6	high		4.9	0.31	29	10
PS	66	F	11	1	16.7	3.7		1.82	0.73	110.1	1.8	high	9.8	4.8	2.52	34	10.5
PS	68	M	10	1	18.7	3.4	164	1.71	0.79	77.15	1.8	high	7.6	4.7	0.23	30	10.1
PS	61	F	7	1	21.8	3.8	116	0.96	1.07	116.8	1.8	high	9.5	4.8	1.3	28	9.6
PS	56	F	7	1	20.8	3.7	120	1.86	1.26	111	1.5	high	8.3	5.4	4.17	26	10.3
PS	68	M	7	1	18.1	3.9	110	1.25	0.85	81.95	1.8	high	9.2	4.5	0.33	29	11.3
PS	68	M	6	1	24.7	3.7	143	1.33	0.72	101.1	1.5	high	8.6	5	0.63	28	11.1
PS	33	F	5	1	20.4	3.7	133	1.64	0.79	94.22	2.1	high	8.6	3.9	0.23	34	11.9
PS	53	M	5	1	19.3	3.8	80	1.23	0.81	123	2.1	high		6.2	0.01	31	10.5
PS	83	F	5	1	19.6	3.8	142	1.32	0.8	126.9	2.1	high		4.8	0.02	26	11.6
PS	74	F	5	1	15.7	3.9	146	1.22	0.91	135.3	2.1	high		4.5	0.07	11	12.2
PS	53	F	5	1	21.6	3.8	155	2.35	0.83	102.1	1.8	high	9.6	4.4	0.62	23	9.3
PS	78	F	5	1	15.3	3.4	142	1.13	0.68	92.03	2.1	high		4.4	0.02	30	10.4
PS	72	M	4	1	20.7	3.4	184	1.56	0.9	92.31	1.8	high	9	5.4	0.05	22	12.3
PS	55	F	4	1	19.2	3.9	115	1.46	0.46	67.11	1.9	high		2.8		30	9.1
PS	86	F	4	1	16.4	4.2	117	1.39	0.7	76.97	2.1	high		4.4	0.01	33	11.3
PS	67	F	4	1	25.6	3.8	157	2	1.04	92.95	1.8	high	8.3	6.1	0.05	23	11.3
PS	70	M	3	1	20.6	3.8	119	1.78	0.73	112.6	2.1	high		3.7	0.34	31	10.2
PS	66	F	3	1	19.8	4.1	125	1.15	0.98	73.14	2.1	high		5.1	0.03	28	10.3
PS	55	M	3	1	20.4	4	138	1.07	0.95	99.33	2.1	high		5.7	0.09		9.7
PS	77	F	2	1	23.9	3.1	126	1.34	0.94	77.08	2.1	high		6.7	0.08	35	11.3
PS	68	M	2	1	19.1	3.9	117	1.31	0.85	100.7	2.1	high		6.2	0.26	29	10
PS	58	M	6	1	25.2	4.2	175	1.9	1.31	151	1.8	high	7.9	7.7		20	9.6
PS	36	M	2	1	16.9	4.3	136	1	1	104.4	2.1	high	8.3	6			
PS	73	M	5	1	26	3.8		1.35	1.05	141.5	2.1	high		4.9			5.4
PS	64	F	21	1	17.8	3.9	79	1.47	0.71	73.31	2.1	high		3.8	0.01	31	10.1
PS	75	F	4	1	26.2	3.8	77	1.51	1.22	130.1	2.1	high		4.7	0.17	25	10.5
PS	58	M	2	1	24.6	3.8	131	1.12	1.01	68.29	1.3	high	9.7	6	3.31	24	10.4
PS	68	F	12	1	18.9	3.9	125	1.43	0.91	114.7	2.1	high		5	0.03	23	9.5
PS	67	M	12	1	17.6	3.5	122	1.04	0.91	49.7	2.1	high		3.4	0.02	22	9.8
PS	60	F	10	1	27	3.7		1.45	0.82	91.7	1.5	high		4			5.1
PS	67	F	10	1	21	4.3	100	1.48	0.83	85.18	2.1	high		4.1	0.16	25	9.4
PS	55	F	3	1	27.1	3.5	127	1.67	0.94	41.06	2.1	high		4.2	0.03	26	10.8

PS	66	F	8	1	23.8	4.1	118	1.32	0.77	89.83	2.1	high		2.8	0.04	23	10
PS	51	F	7	1	19.4	4.5	153	1.35	1.1	94.37	2.1	high		3.1	5.62	32	10.4
PS	65	M	8	1	20.7	3.9	181	1.36	1.04	94.4	1.1	high	8.9	4.7	0	24	9.9
PS	63	M	14	1	24.2	3.8	182	1.29	0.88	109.8	2.1	high	9.6	5.5	0	26	10.9
PS	56	M	10	1	25	4	201	1.94	1.17	123.3	1.3	high	9.5	5.3	0.05	24	11.1
PS	50	M	7	1	27.6	4	151	1.13	1.24	95.38	2.1	high	9.2	5.5	0.26	25	11.9
PS	76	F	11	1	23.3	3.7	111	1.14	0.84	112.3	2.1	high		3	0.01	19	10.4
PS	49	F	6	1	28.3	3.5	197	1.85	1.08	95.76	1.8	high	8.6	3.7	0.03	30	11.5
PS	72	M	3	1	18.4	2.1	124	1.16	1.28	86.94	1.8	high	9.1	4.6	0.06	22	12
PS	48	F	10	1	17.4	4.5	131	1.26	1.05	133.1	1.8	high	8.7	6.6		40	12.4
PS	67	F	21	1	22.8	3.2	134	1.55	0.82	129.4	1.8	high	8.2	5.6	0.58	30	10.9
PS	66	M	8	1	21.4	3.3	196	1.77	1.05	138.3	1.8	high	9.8	3.3	0.06	25	10.9
PS	69	F	12	1	20	3.6	181	1.24	0.86	137.3	1.6	high	8.1	6.5	0.12	21	10.4
PS	60	F	9	1	20.3	4	135	1.26	1.17	147.1	2.1	high		3.9	0.11	26	9.8
PS	59	M	9	1	24.6	4.1	84	1.24	1.33	156.3	2.1	high		4.2	0.03	32	10.6
PS	41	M	10	1	17.4	3.9	134	1.57	0.95	73.62	1	high	8.9	5.1		29	9
PS	43	F	4	1	22.1	3.9	177	1.83	1.71	102.8	1.8	high		6.3			9.9
PS	56	F	2	1	18.7	3.6		1.28	1.06	109.5	2.5	high	7.5	5	0.06	22	11
PS	81	M	10	1	17.8	3.6	136	1.28	0.91	122	2.1	high		4	0.09	29	10
PS	71	M	2	1	28.9	3.9	181	1.34	0.76	48.2	1.8	high	9.1	3	0.6	29	9
PS	60	M	2	1	24.1	3.4	234	1.46	0.96	115.5	1.3	high		3.5	0.1	22	9.3
PS	60	F	8	1	16.9	4.4	143	1.69	0.74	145.3	1.3	high	10.2	5.8	0.06	41	10.2
PS	53	M	10	1	26.1	3.7	116	1.44	1.04	139.4	1.5	high	9.4	5	0.02	22	11
PS	60	F	24	1	26.4	3.6	165	1.54	0.9	121.6	1.5	high		6.5	0.03	24	11.4
PS	51	F	11	1	17.3	4.2		1.49	1.05	96.67	1.3	high	8.2	4.7			10.7
PS	59	M	4	1	24.6	4.2	148	1.8	0.7	98.91	1.6	high	9	5.2		36	11.6
PS	45	M	16	1	19.6	4.1	172	1.58	0.78	101.2	1.5	high	10.4	7.9	0.05	29	11.1
PS	67	F	15	1	20	3.5	215	0.96	0.6	112.8	1.3	high	9.2	5.2	0.4	28	11.7
PS	72	M	2	1	22.8	4	113	1.53	0.87	102.4	1.8	high	9.4	5.1		30	10.2
PS	58	F	15	1	18.3	4.2	124	1.32	0.98	87.93	1.3	high		5.4	5.13	32	9
PS	84	M	2	1	19.6	3.6	179	1.52	0.74	78.59	2.1	high	8.7	7		30	11.3
PS	47	M	13	1	20.1	3.8	160	1.56	0.98	119.4	1.3	high	8.1	6.5	0.24	27	10
PS	59	F	10	1	18.5	4.2	134	1.26	1.54	110.7	1.1	high	8.4	6.5	0.4	25	12.6
PS	77	F	11	1	19.8	3.4	127	1.59	0.92	88.17	1.8	high	9.4	3	0	11	
PS	63	M	6	1	22	3.6	161	1.44	0.74	122.4	1.6	high	10.2	8.5	0.15	39	10.8
PS	71	F	25	1	15.7	3.5		1.31	1.02	82.97	1.3	high		5.7			9.8
PS	70	M	5	1	22.9	3.6	183	1.6	0.87	103.1	2.1	high		3.7	0.24	31	11.1
PS	62	M	12	1	24	4.1	208	1.62	0.99	128.7	2.1	high	10	5.3	0.05		12.4
PS	36	M	4	1	18.7	4.3	179	1.39	1.39	109.3	2.1	high	9.2	4.1	0.2	32	9.6
PS	57	M	14	1	24.8	4.2	194	1.12	0.98	107.2	2.1	high		6.7			10
PS	47	M	4	1	18.1	3.9	173	1.65	1.14	74.93	1	high		5.2	0.26	27	8.5
PS	53	M	9	1	19.2	3.8	93	1	0.8	98.13	1.6	high		6.2	0.1	28	11.2
PS	34	F	5	1	24.8	3.7	147	1.44	1.05	97.11	2.1	high	9.8	4.9	0.09	29	8.8
PS	65	M	8	1	21.5	4	182	1.38	0.84	150.7	1.6	high	9.9	3.8	0.07	20	10.2
PS	32	M	3	1	20.4	3.9	194	1.14	0.86	89.32	1.6	high	9.3	3.6		25	9.4
PS	43	M	11	1	19.2	3.9	206	1.64	1.06	84.02	1.3	high	9.8	6	0.05	34	10.6
PS	24	M	4	1	21.3	4.6	110	1.25	1.06	113.2	2.1	high		5	0.01	42	10.8
PS	48	F	19	1	24.4	3.8	135	1.63	0.94	111.2	1.3	high		5.7	0.01	27	10.3
PS	57	M	8	1	21.6	3.9	152	1.56	0.87	126.9	1.5	high	8.7	5.3	0.1	27	11.6
PS	33	M	2	1	20.2	3.9	182	1.7	0.89	77.3	1.6	high	8.8	6.1		36	
PS	37	M	15	1	14.5	4.3	165	1.09	0.89	86.36	1.6	high	7.3	4.1			10.4
PS	66	F	11	1	16.5	4.1		1.59	0.99	107.7	1.3	high	8.7	4.1			10.6
PS	66	M	10	1	20.1	4.4	140	1	0.93	94.15	1.8	high		5.9	0.82	24	10.8
PS	63	F	11	1	21.7	3.9	262	1.41	0.81	107.2	1.5	high	8.3	6.2	0.1	25	10.7
PS	61	M	23	1	18.6	3.5	159	2.09	1.18	106.1	1.8	high		4.9	0.03	2	10.9
PS	55	F	4	1	19.1	3.8	201	1.61	1.18	77.96	1	high	10	5.1		32	7.6
PS	72	F	18	1	16.7	3.4	172	1.33	0.84	74.46	2.1	high	9.6	5.4	0.04		11.9
PS	88	M	9	1	22.4	3.9	160	1.16	0.83	110	1.6	high	9	1.9		27	10.3
PS	74	F	9	1	18	3.2	107	1.71	1.01	137.6	1.8	high		4.5	0.05	34	13.9

PS	66	F	7	1	19.2	3.7	99	1.51	0.91	90.95	1.8	high	10	5.1	6.86	38	12.4
PS	73	F	14	1	18.3	3.6		1.73	1.13	122.9	1.3	high	9.2	2.6			10.8
PS	55	M	9	1	21.3	3.9	167	1.61	0.91	121.4	1.5	high	8.9	6.5	0.83	35	9.8
PS	69	M	3	1	16.9	4.2	110	1.73	1.03	117.5	2.1	high	9.4	4.9		22	12.1
PS	74	F	10	1	17.2	4.1		1.56	1.11	121.7	1.6	high	9.8	5.3		25	9.6
PS	74	F	6	1	26.4	3.4	93	1.42	0.69	94.37	1.5	high	10.8	7.7		20	16.5
PS	67	F	9	1	18.6	3.8	112	1.13	1.05	99.7	1.6	high	8.4	4.3	0.28	37	11
PS	72	M	11	1	18.5	4	196	1.98	0.88	106.6	1.8	high		5.5	0.06	26	10.9
PS	48	F	5	1	21	4	96	0.98	0.99	83.16	2.1	high	9.8	5.9	1.98		6.8
PS	75	F	11	1	20.7	3.7	167	1.22	1.1	123.9	2.1	high	8.9	4.5		24	11.6
PS	67	M	8	1	18.5	3.3		1.87	0.86	102.6	1.3	high	8.5	3.2			9.8
PS	62	M	14	1	16.9	3.8	136	1.08	0.92	109.1	2.1	high		5.5	0.06	26	9.5
PS	67	M	5	1	20.6	3.9	201	1.12	0.84	111.2	2.1	high	9.2	8	0.06	32	13.1
PS	62	F	11	1	20.5	3.9		1.85	0.94	138.3	1.3	high	8.3	7.3			9.9
PS	63	F	16	1	17.2	3.8	150	1.53	0.98	135	2.1	high	7.9	3.3	0.61	30	12.1
PS	63	M	10	1	23.1	3.7	140	1.16	0.86	116.2	2.1	high	7.1	6.1		26	
PS	53	F	24	1	19.5	4	174	1.58	1.09	101.2	1.3	high	7.5	4.7	1.6	32	10.4
PS	58	M	6	1	25.2	4.3	156	1.08	0.8	121.1	1.8	high	8.6	5.3		27	
PS	56	M	15	1	19.1	4.3	152	1.84	0.98	126.8	1.3	high	9.3	3.1	0.05	31	10.1
PS	52	M	29	1	18.4	4.1	149	1.55	0.79	124.1	1.5	high	9.8	4.6	0.04	28	6.8
PS	58	M	26	1	19.2	3.7	98	1.21	0.8	146.3	1.9	high	9.8	5.5	0.15	27	10.1
PS	60	F	24	1	16.3	4.1	135	1.06	1.01	138.1	2.2	high		8.2	0.05	26	9.8
PS	66	F	21	1	16.9	4.4	147	1.19	1.05	128.8	1.9	high	8.2	5.8	1.17	33	10.3
PS	75	F	19	1	16.8	3.8	128	0.92	0.9	123.1	1	high	7.7	6.9	0.02	29	9.4
PS	69	M	19	1	17.3	4.3	114	1.44	0.9	107.4	2.1	high	8	5.4	0.03	29	9.6
PS	77	F	17	1	25	3.7	149	1.58	0.52	95.05	2.1	high		5.1	0.12	20	9.7
PS	66	F	17	1	17.9	4.2	130	1.73	0.87	114.5	1.3	high	8.9	4.2	0.05	32	10.7
PS	60	F	15	1	24.3	4.4	186	1.28	0.95	130.8	1.9	high	10.2	5.4	0.16	30	10.8
PS	42	M	14	1	28.7	4.2	158	1.58	0.64	100	2.1	high	9.3	6	0.07	28	11.1
PS	71	F	14	1	19	4.1	106	1.25	1.08	120.5	2.1	high	8.4	4	0.03		14.1
PS	64	M	13	1	20.2	4.6	115	1.24	1	130.8	1.6	high	8.1	3.9	0.05	27	11.2
PS	67	M	12	1	18.7	3.7	182	1.59	0.96	133.2	1.9	high	10.6	6.7	0.29	25	10.4
PS	60	F	12	1	23.7	4.1		1.57	0.88	107.5	1.8	high		3.6			
PS	61	F	12	1	19.6	4.4	136	1.97	0.98	139.2	1.6	high	8.3	6	0.05	23	10.6
PS	35	M	11	1	19.1	4		1.52	0.93	105.3	2.1	high		3.9	0.07		10.2
PS	65	M	10	1	20	4	156	1.27	0.97	127.8	2.5	high		7	0.17	29	11
PS	51	M	9	1	28.6	4.1	157	2.02	1.02	99.17	1.9	high	8.3	4.2	0.05	28	10.6
PS	66	F	9	1	23.7	4.5		1.44	0.72	116.1	1.5	high		3.6			
PS	46	M	8	1	23.2	4.3	140	1.48	0.99	111.2	1.3	high	7.3	6.6	0.73	38	12.9
PS	64	F	8	1	17.1	3.5	169	1.43	1.14	126.6	1.3	high		6.8		22	9.8
PS	58	M	7	1	23.7	3.5	138	1.43	0.71	89.25	2.1	high		4.1	0.46	28	9.8
PS	60	M	7	1	26.1	3.9	163	1.51	0.9	117.8	1.5	high	9.4	4.3		30	10.3
PS	78	M	7	1	19.8	3.4	132	1.39	0.93	102.5	1.8	high		5.2	0.03	22	11.3
PS	82	M	7	1	21.6	3.9	115	1.07	0.52	91.15	2.1	high		6.2	0.11		9.9
PS	79	M	6	1	22.8	4.4	154	1.32	1.09	175.3	1.5	high	10.7	3.5	0.77	24	11.1
PS	62	F	6	1	23.8	3.6	139	1.65	0.92	117.2	1.3	high	9.8	3.6	0.05	26	10.6
PS	82	F	6	1	19.5	3.7	138	1.21	1.36	81.63	2.1	high	8.7	4.9	0.04	24	11.6
PS	34	F	6	1	32.1	4	149	1.96	0.95	131.5	1.3	high	9.2	5.3	0.05	29	11.5
PS	85	M	5	1	22.3	3.8	127	1.18	0.97	125.2	1.6	high		7.5	0	26	9.9
PS	74	M	5	1	19.9	4.5	129	1.61	0.65	97.46	1.3	high	9.1	7	0.22	30	11
PS	78	M	5	1	18.6	4.3		1.57	1.04	123	1.5	high		4.3			4.9
PS	83	M	4	1	22	3.8	111	1.22	0.77	131.6	1.3	high	9.4	5.5			10
PS	62	M	4	1	19.9	4	88	1.29	0.98	123.3	1.3	high	8.6	7.2		34	12.2
PS	75	M	3	1	20	4.2	169	1.2	0.97	161	1.6	high	8.8	4		21	11.5
PS	62	M	3	1	24.1	3.9	127	1.34	0.69	122.1	1.6	high	8.3	4.2	0.24	31	11.1
PS	75	M	3	1	22.1	4.5	109	1.83	0.75	115.2	1.5	high	9.2	3.8	0.02	31	9.8
PS	78	M	3	1	22.5	3.8	171	1.33	0.87	133.1	1.6	high		5.8	0.15	27	10.7
PS	62	F	3	1	18.9	4.5	137	1.06	0.81	137.7	2.1	high	8.3	4.6	1	21	10.4
PS	36	M	2	1	20.4	4.3	171	1.55	1.01	106.1	1.6	high		6.1	0.02	25	10

PS	82	F	2	1	22.8	3.6	132	1.76	0.69	73.92	1.5	high		5.4	0.3	26	7.2
PS	68	M	2	1	24.8	3.9	116	1.81	0.62	62.88	1.8	high	9	6.5	1.37	29	13
PS	75	M	2	1	28.9	3.9	174	1.46	0.96	90.27	1.5	high	9.2	4.8	0.1	26	11.3
PS	68	M	2	1	26.8	4.3	189	1.93	0.74	64.24	1.3	high		3.7	1.56	44	10.8
PS	37	M	12	1	18.7	4.3	147	1.22	0.97	124.7	1.3	high	9.2	4.7	0.16	24	11.4
PS	55	F	27	1	18.9	3.7	150	1.47	1	94.45	1.8	high		4.9	0.45	35	9.5
PS	68	F	23	1	19.4	3.6	158	1.37	1.1	120.8	2.1	high		4.9	0.2	23	11.3
PS	58	M	17	1	18.8	3.6	126	1.31	0.77	97.12	2.1	high		6.1	0.09	26	11.3
PS	75	F	16	1	19.9	3.6	81	1.28	1.03	102	1.5	high		3.7	0.14	29	11
PS	57	F	15	1	17.9	3.8	138	1.31	1.24	133.5	1.8	high	9.1	4.8	0.35	24	10.9
PS	55	M	10	1	22.1	4	144	1.19	1.34	110.2	2.1	high	8.4	4.1	0.96	35	10.1
PS	33	F	10	1	21.1	3.8	175	0.83	0.94	104.9	2.1	high		4.9	0.03	26	12.5
PS	69	F	9	1	20.3	3.3	124	1.19	0.65	57.73	2.1	high		4.8	0.09	25	12
PS	70	F	8	1	20	3.7	130	1.3	0.76	138.8	1.8	high	8.6	5.8	0.5	48	9.5
PS	71	M	7	1	24.3	3	180	1.62	0.71	113.1	1.5	high	9.5	3.6	0.35	36	11
PS	44	M	5	1	24.3	3.4	138	1.24	0.93	97.17	2.1	high		5.7	0.03	25	9.9
PS	73	M	3	1	22.8	3.6	121	1.26	1.08	115.5	1.6	high	8.6	5.3		39	11.1
PS	49	M	12	1	25.4	4.3	134	1.98	0.91	111.3	2.1	high		4.9		48	12.4
PS	60	M	5	1	22	4	218	1.93	0.83	115.8	1.3	high		3.2	0.05	28	10.9
PS	88	F	6	1	31.2	3.5	221	1.03	1.23	140.2	1.5	high	8.4	6.2	0.1	36	11.4
PS	88	F	6	1	21.9	2.4	163	1.98	0.76	131.5	2.1	high	8.7	3.7	0.79	33	11.1
PS	55	M	9	1	25.6		149	2.09	0.93	114.3	1.8	high	8.2	3.9	0.19	25	9.3
PS	72	F	8	1	27.9	4	148	1.86	0.62	129.7	1.3	high		3.2	0.04	28	10.9
PS	63	M	10	1	25.4		128	2.09	1.26	143.6	2.5	high	9.3	6.4	0.44	35	10.6
PS	55	M	32	1	20.2	3.8	109	1.19	1.04	107.8	1.3	high		1.9	0.28	25	9.1
PS	63	M	30	1	20.1	3.8	154	1.83	0.86	97	1.8	high		3.5	0.6		10.7
PS	65	M	26	1	19.5	3.8	165	1.55	0.95	115.7	1.8	high		4	0.41		9.9
PS	54	M	25	1	21.9	4	130	1.66	1.01	135.9	1.8	high		4.4	1.41		10.5
PS	66	M	12	1	21.2	4.1	192	1.71	1.02	122.2	1.3	high		4.1	0.07	24	11.2
PS	59	F	11	1	18.9	4.2	216	1.06	1.01	114.6	2.1	high		5.7	1.13		11.7
PS	79	M	11	1	17.8	3.8	125	1.75	0.87	101.4	2.1	high		7	0.34		7.6
PS	57	F	10	1	21.7	4.2	120	1.15	1.11	115.5	1.5	high	8.8	7.1	0.08	37	9.7
PS	62	F	10	1	22.8	3.8	105	1.49	0.99	109.6	1.8	high	8.6	4.9			10
PS	64	F	10	1	21	4	153	1.57	1.06	127.8	2.5	high	8.7	3.5	0.79	29	9.1
PS	58	M	9	1	20.2	4	173	1.54	1.2	114.5	2.1	high		2.8	0.01		10.7
PS	60	F	9	1	24.3	4	79	1.28	1.19	126.7	2.1	high		5.6	0.04		10.6
PS	63	F	8	1	23.2	4.1	171	1.02	0.66	111.2	2.1	high	8.3	5.1	0.07		10.1
PS	66	F	8	1	25.6	3.9	105	1.56	1.04	123.2	1.5	high		5.1	0.13	21	9
PS	69	F	7	1	28.8	3.9	151	1.08	0.74	108.2	2.1	high		4.3	0.19		12.1
PS	52	M	6	1	20.5	3.7	126	1.29	0.96	100.3	2.1	high		6.3	0.01		11
PS	64	M	6	1	21.6	4	126	1.44	0.89	100	1.8	high		2.2	0.02		10.3
PS	75	M	6	1	22.5	4.1	167	1.27	1.25	134.3	2.1	high		5.1	0.72		12
PS	84	F	6	1	18.5	3.8	254	1.79	1.13	111.3	1.8	high		6.4	0.91		11.4
PS	60	M	5	1	21	3.8	191	2.37	1.28	110.8	1.1	high		3.9		33	10.6
PS	79	M	4	1	22.3	4.1	132	1.3	1.05	96.4	1.5	high	8.2	1.9			11.6
PS	56	M	3	1	28.7	4.1	158	1.19	1.05	105.6	1.8	high	9.5	8.9	0.06	23	13.3
PS	69	M	3	1	22.4	4	79	1.81	1.17	102	2.1	high	8.1	7	0.12	27	9.8
PS	49	M	3	1	24	3.7	122	1.86	1.13	97.4	1.8	high	10	3.7		39	8.7
PS	54	M	3	1	21.9	4.1	134	1.56	1.18	139.5	1.5	high	9.4	5.5	0.24	28	9.9
PS	61	F	2	1	26.1	4	175	1.34	0.95	97.18	1.8	high	10.1	5.3		25	12.9
PS	57	M	2	1	24.9	3.9	155	1.54	1.01	90.75	2.1	high		5.2	0.09		11.2
PS	55	M	20	1	24	3.7	134	1.89	0.97	119.7	1	high	8.8	5.8	0.1		11.4
PS	66	F	19	1	16.2	3.9	159	1.48	1	77.17	1.4	high	9.3	6.2	0.77		8.9
PS	52	M	13	1	22.5	4.3	169	1.29	0.78	107	1.8	high	10.1	3.8	1.12	22	9.9
PS	68	F	12	1	23.7	3.9	168	1.29	0.92	101.5	1.5	high	8.7	5.7	0.1	26	10.2
PS	60	M	10	1	22.1	4.1		1.35	0.82	133.5	2.2	high	8.5	3.5	0.09	35	10
PS	61	M	10	1	19.1	3.7		0.91	0.64	93	1.4	high	9.9	4.6			8.7
PS	78	M	7	1	21.1	3.9	171	1.72	0.88	69.75	1.8	high		5.9	0.58	29	9.7
PS	49	M	6	1	24.1	3.8	187	1.47	0.81	104.8	1.5	high	8.3	3.7			9.8

PS	68	F	5	1	18.4	3.9	156	1.87	0.66	96.12	1.8	high	8	4.9			9.5
PS	72	F	2	1	18.3	3.9	187	1.57	0.94	85.1	1.4	high	8.5	5.9	0.1	16	9.9
PS	73	M	18	1	16.7	3.3	139	1.19	1.39	93.68	1.3	high		3.3	0.05	28	9.5
PS	63	M	26	1	22.2	4	148	1.4	0.85	104	1.3	high		3.9	0.13	32	10.3
PS	47	M	26	1	19.4	4.4	173	1.5	0.87	94.68	1.9	high		4.6	0.09	24	11.2
PS	71	F	23	1	19.6	4.2	243	1.64	1.02	118.9	1.6	high		5.7	0.1	26	11.5
PS	58	M	11	1	19.3		175	1.35	0.97	107.8	1.5	high	8.8	5.6	0	20	10.8
PS	59	M	8	1	18.8	4.5	186	1.3	1.31	115.6	1.6	high	9.5	2.5	0.77	25	7.3
PS	63	M	8	1	21.8	4.1	209	1.56	0.82	98.93	1.5	high	9.1	4.2	0		9.5
PS	62	F	8	1	17.8	4.4	151	1.28	0.73	125.8	2.1	high		3.1	0.05	28	10.9
PS	65	M	8	1	18.1	3.4	137	1.47	0.84	113.5	2.1	high	8.9	4.2	0.09		11.1
PS	58	F	7	1	33.9	3.8	172	1.71	0.84	106.5	1.8	high	8.5	4.8	0.05		10.9
PS	54	M	6	1	20.8	4.2	259	0.54	0.81	94.73	1.8	high		2.6	1.09	25	9.1
PS	58	F	5	1	18.5	4.1	144	1.46	1.04	146.4	1.9	high		5.9	0.05	24	8.4
PS	62	M	4	1	21.4	4.1	243	1.72	0.87	98.35	1.6	high		6.7	0.05	25	10.3
PS	46	M	4	1	32.8	4.4	212	1.26	1.07	114	2.1	high	8.2	6.5	1.38	50	11.2
PS	85	F	4	1	17.4	4	137	1.6	0.59	121.1	1	high	7.7	3.7	0.18	36	9.2
PS	75	M	4	1	20.4	4.1	169	1.96	0.99	94.96	1.5	high	8.4	3.8	0.23	28	10.8
PS	82	F	4	1	18.5	4	179	2.15	0.94	110.2	1.6	high	8.7	4.5		28	11.4
PS	43	M	3	1	24.2	3.8	159	1.17	0.77	111.1	2.1	high		7	0.84	33	7.9
PS	66	M	3	1	18.6	3.9	166	1.32	0.82	81.62	1.9	high		5		25	11.1
PS	73	F	3	1	24.8	3.8	104	1.91	0.94	105.9	1.3	high	7.8	3		33	10
PS	68	M	3	1	21.2	3.8	142	1.34	0.8	81.04	2.1	high	9.1	5.4	1.07		10.3
PS	54	M	3	1	22.2	4.1	142	1.48	0.83	82.71	1.5	high		3.7	0.06	27	12.6
PS	72	M	2	1	25.2	3.9	190	1.7	1.12	120.2	1.9	high		5.8		23	9.7
PS	58	F	2	1	20	4.4	171	1.49	0.94	102.4	1.5	high	8.5	4.1	0.03		9.3
PS	59	F	18	1	24	3.7	173	1.5	0.86	131.1	1.8	high		4.6	0.05		9.5
PS	50	M	14	1	30.7	3.9	180	1.08	0.85	108.7	1.3	high		4.8	0.05	18	12.1
PS	37	F	13	1	25.2	4	162	1.19	1.4	108.8	1.6	high		5.1	3.57	30	9.7
PS	59	M	12	1	23.7	3.7	173	1.39	0.91	127.7	1.9	high		5.8	0.05	26	10.6
PS	70	M	12	1	19.2	3.8	146	1.95	1.03	163.9	1.3	high		5.1	0.1	22	10.2
PS	58	M	12	1	20.2	3.7	205	1.93	1.2	125.3	1.9	high	9	5	0.75	28	9.5
PS	71	F	10	1	23.7	3.5	179	2.09	0.98	92.4	1.9	high	8.5	5.5	0.05	25	9.1
PS	61	M	10	1	31	3.2	133	1.36	1.04	137.4	1.9	high	9.1	2.8	0.05	27	9
PS	61	M	9	1	24.5	3.4	150	1.53	0.88	132.4	2.1	high	8.2	4.9	0.14	36	13.5
PS	55	F	8	1	20.9	3.8	147	1.74	1.34	117.2	1.9	high	9.4	6.3	0.05	29	10.5
PS	49	F	8	1	23	3.9	129	1.45	0.94	100	1.8	high	9	7.2	0.12		10.9
PS	66	M	8	1	17.3	3.6	170	1.2	1.01	114.3	2.1	high	8.3	4.1		27	
PS	45	M	7	1	24.3	3.9	144	1.8	0.87	118.9	1.6	high	8.8	5	0.1	27	10.9
PS	36	M	7	1	19.2	3.9	182	1.25	0.98	92.32	2.1	high		5.1	0.03		10.3
PS	65	M	7	1	16.6	3.5		1.87	1	87.13	1.8	high	7.8	4.9	1.05		
PS	51	F	6	1	14.8	3.5	122	1.46	1.11	84.1	1.3	high	10.5	5.5	0.06	29	10.7
PS	64	M	6	1	21.5	3.5	124	2.04	0.8	97.38	1	high	8.6	3	0.04	35	7.7
PS	40	M	6	1	19.1	3.7	179	2.33	0.78	107.7	1.3	high	8.9	5	0.02	33	11
PS	58	M	5	1	20.6	3.6	111	1.61	1.14	102.8	1.6	high	8.8	7.7	0.1	32	12.9
PS	56	F	5	1	16.6	3.6	141	1.81	1.21	128.6	1.3	high	9.1	6.5	0.1	42	10.6
PS	63	F	4	1	16	3.4	105	1.39	1.08	96.19	1.6	high	9.3	6	0	39	12
PS	59	F	3	1	23.6	3.9	148	1.61	0.76	55.51	1.3	high	9.3	4.7	0	24	9.9
PS	73	M	3	1	19.7	3.9	217	1.84	1.18	144.8	1.3	high	9.1	6.5	0.2		9.4
PS	71	M	3	1	17.7	4.1	148	1.99	1.08	118.1	1.5	high	8.9	5.5	0.03	25	9.9
PS	57	F	3	1	28.8	3.7	172	1.44	1.22	114.4	1.3	high	9.6	5	0	29	9.3
PS	64	F	2	1	21.5	3.8	121	1.62	0.8	85.43	1.6	high	9	3.4	0.11	23	10.4
PS	62	F	2	1	23	3.8		1.55	1.14	96.03	1.8	high		4.8	0.79	25	12.2
PS	55	M	4	1	25.8	3.7	133	1.29	0.98	118.4	1.5	high		5.1	0.05		13.6
PS	73	F	28	1	19.3	3.1	136	1.66	0.82	98.56	1.6	high	10.4	5.8	0	33	12
PS	62	M	18	1	23.4	3.8	133	1.41	0.99	132.4	1.3	high	9.7	6.5	0.1	37	12.7
PS	67	M	14	1	20.4	3.8	168	0.93	0.78	128	1.6	high	8.9	6.8	0.05		9.4
PS	43	F	11	1	18.8	3.8	214	1.82	0.83	119	1.3	high	8.9	5.5	0.03		11.5
PS	60	F	9	1	19	4.2	147	1.26	0.94	128.2	2.1	high		5	0.04	29	10.7

PS	64	F	8	1	20.5	3.8	171	2.01	1.07	104.7	1.5	high		3.6	0.03	23	11.3
PS	61	F	7	1	31.2	3.5	138	1.78	0.94	87.72	1.8	high	9.7	4.9	0.56	28	8.6
PS	70	M	5	1	26.3	4.3	138	1	1.18	112.7	2.1	high		7.8	0.01	25	10.7
PS	39	M	2	1	18.4	4.2	104	1.09	1.19	64.76	2.1	high	8.9	5.6	0.18	21	10
PS	66	M	2	1	19.8	3.7	169	1.71	0.85	67.43	2.1	high	8.3	7.3	0.1	26	11.6
PS	46	F	17	1	18.9	3.7	116	1.17	0.83	78.83	2.1	high	10	6.7	0.06	31	12.1
PS	60	M	10	1	18.4	3.7	175	1.72	0.75	87.08	1	high	9.5	3.5	1.2	33	10.8
PS	40	M	8	1	32.5	4.1	140	1.89	0.87	86.51	1.5	high	9	6.3	0.1	18	10.5
PS	49	M	7	1	17.4	3.6	159	0.95	1.02	92.06	1.3	high	10.1	4	0.9		8
PS	74	F	5	1	22.8	3.8	174	2.02	0.76	79.93	1.3	high	9.4	4.2	0.47	30	11.1
PS	52	M	4	1	24.6	3.4	136	1.35	0.62	74.28	1.8	high	8.9	6.4	0.12	22	8.1
PS	65	M	2	1	22.7	3.4	147	1.95	0.75	53.16	1.1	high	10.5	4.2	0.2		9.8
PS	52	F	34	1	20.4	4	104	1.08	0.87	109.9	1.4	high	9.5	7		25	
PS	62	M	17	1	20.5	3.7	136	1.33	0.88	117.3	1.5	high	8.7	5.9	0.1	34	11
PS	67	F	7	1	21.2	4.2		1.44	0.95	135.2	1.8	high		6.8	0.05	25	11.8
PS	75	M	8	1	20.7	3.4	157	2.8	0.88	119.1	1.6	high	9.2	2.8		26	11.4
PS	74	M	6	1	19.7	4.1	134	1.6	1.22	105	1.8	high	9.1	4.9	0.26	25	11.1
PS	73	M	4	1	20.6	3.4	160	1.8	0.85	102.7	1.8	high	9.3	6.6	1.27	26	10.6
PS	75	M	2	1	21.6	3.8	62	1.1	0.88	110.1	1.8	high		3.3	0.68	29	14.8
PS	62	F	14	1	19	4	127	1.4	1.22	117.7	1.5	high	8.5	4.6	0.05	18	10.3
PS	78	F	24	1	17.3	4.1	133	1.46	0.77	108.3	1.3	high	9	5.1	0.03	18	11.1
PS	67	F	22	1	17.8	3.8		1.68	0.99	102.1	1.8	high	9.3	6.2		35	11.1
PS	63	M	31	1	18.4	3.9	150	1.24	0.95	103.3	1.3	high	9.4	3.3	0	26	11.6
PS	54	M	25	1	16.8	4.2	128	1.38	0.97	111.9	1.6	high	8.7	6.2	0.1	28	10.3
PS	60	M	29	1	20.1	4	116	1.47	0.93	82.14	1.3	high	8.4	4.7	0.1	26	11.1
PS	61	M	21	1	18.3	4.1	124	1.31	0.99	99.18	1.6	high	8.6	4	0.2	35	10.7
PS	66	M	17	1	20.4	4	158	1.95	0.8	135.3	1.3	high	9	3.9	1.1	34	8.9
PS	63	F	18	1	17.6	4.4	128	1.57	1.15	113.9	1.3	high	8.9	3.6	0.2	24	11.7
PS	61	M	30	1	21.3	4.3	138	1.24	1.04	92.74	1.3	high	9.5	6.8	0	31	10.2
PS	59	M	35	1	21.1	3.9	126	1.78	0.76	88.43	1.6	high	9.1	3.5	0.1	23	11.6
PS	59	M	31	1	17.1	4.1	139	1.23	0.86	94.89	1.3	high	10.3	6.1	0.1	19	12.6
PS	68	M	30	1	18.6	4.8	115	1.35	0.93	109.6	1.9	high	10.9	7.3	0.1	27	12.7
PS	72	F	21	1	20.6	3.7	168	1.44	0.84	109.5	2.1	high	11.6	3	6.47	35	9.7
PS	66	F	17	1	17.2		167	1.2	1.23	97.89	1.6	high	10.2	5.2	0.1	22	12.6
PS	56	F	16	1	25.2	4	175	1.13	0.9	103	2.1	high	7.6	11.7	0.04	26	12.9
PS	72	M	23	1	22.6	3.6	194	1.38	0.83	105.3	1.6	high	8.4	7.7	0.32	27	10.4
PS	57	F	33	1	20.9	4	152	1.14	1.11	102.1	1.6	high	10.6	9.8	0.39	27	13.1
PS	72	F	18	1	23.3	4	121	1.21	0.84	109.1	1.6	high	10.1	6.5	0.1	23	10.9
PS	45	M	24	1	24.5	3.9	177	1.6	1.1	121.3	1.6	high	9	6.1	0.1	21	10.2
PS	33	F	10	1	20	3.8	155	1.54	0.88	95.34	1.6	high	8.7	4.5	0.1	32	11.7
PS	63	M	8	1	29.2	4.1	192	2.02	0.81	122.4	1.3	high	9.8	5.6	0.1	28	8.5
PS	54	M	15	1	23.2	4.6	205	1.72	1.13	128.9	1.9	high	8.7	5.8	0.55		7.6
PS	67	F	8	1	18.4	3.5		1.37	0.9	101.5	1.6	high	8.8	3.3	0	25	10.5
PS	47	M	14	1	21.8	3.9	210	1.17	1.35	121.3	1.9	high	8.4	7.1	1.2	22	8.9
PS	58	M	16	1	21.7	4.6	109	1.46	0.95	109.3	1.3	high	8.8	6.6	0.01	28	10.8
PS	43	M	8	1	18	4.3	190	1.73	1.03	98.32	1.5	high	11.1	3.9	13.99	32	11.6
PS	60	F	12	1	25.2	3.9	155	1.33	1.19	108.8	1.6	high		3.9	0.01	19	9.5
PS	75	M	6	1	21.7	3.6	129	1.22	0.9	128.4	1.5	high	9	3.9	0.14	30	10.9
PS	60	F	18	1	19.8	4.2		1.44	1	129.4	2.1	high	8.3	5.4	7	28	9.4
PS	69	M	6	1	23.3	3.4	124	1.29	1	106.3	1.5	high	8.6	6.1	0.01	32	10.6
PS	74	M	3	1	22.3	3.8	196	2.14	0.94	97.03	1.8	high	8.8	4.5	0.05	28	11.3
PS	69	M	3	1	19.1	4.1	126	1.33	0.98	96.98	1.6	high	10.9	4.1	0.05	13	10.3
PS	66	M	4	1	20.2	4.3	123	1.39	0.96	127.4	1.6	high	8.6	3.7	0.05	22	9.2
PS	43	M	2	1	19.9	4.5	93	1.18	0.88	79.02	1.5	high		6.6	0.8	45	9.2
PS	73	M	2	1	19.4	4.1	143	1.12	1.21	103.7	1.3	high	10.5	5.7		33	11.6
PS	59	F	20	1	18	3.7		1.57	0.98	132.3	2.1	high	8.4				11.6
PS	56	F	2	1	18.5	4.4	142	1.45	1.15	71.28	1.6	high		3.5	0.08	24	10.6
PS	75	M	2	1	22.2	3.9	93	1.24	0.98	101.7	1.3	high	9.2	6.1	4.57	28	9.1
PS	70	M	2	1	19.9	3.8	176	1.43	1.19	98.38	1.3	high	10.9	4.7	0.05	29	10.6

PS	61	M	3	1	18.3	3.8	140	1.52	1.01	102.9	1.8	high	8.2	5.4	0.78		9.9
PS	61	M	8	1	17.5	4.2	153	1.49	1.18	121.1	1.5	high	9.4	5.7	0.21	30	10.6
PS	66	F	17	1	19.7	4.4	155	1.53	0.91	89.44	1.8	high	10.3	7.7	1.31	0	7.9
PS	71	F	19	1	20	3.5	140	1.54	0.92	96.31	2.1	high	8.6	4.6	0.06		9.4
PS	69	M	5	1	28.7	4	205	1.81	0.9	93.24	1	high	8.7	6		23	10.3
PS	76	M	12	1	20.5	3.5	112	0.92	0.85	107.8	1.5	high	9.3	7		45	8.2
PS	59	F	8	1	21.6	4	98	1.34	1.1	110.8	1.3	high	7.9	5.8			9.1
PS	52	M	8	1	21.2	3.7	139	1.39	0.79	84.23	1.6	high	8.9	7	0.2	25	9.1
PS	58	M	21	1	18.4	3.9	151	1.36	1.34	144.6	2.1	high	9	6.8	2.62	20	11.9
PS	61	M	21	1	16.3	4.4	195	1.64	1.04	118.8	2.1	high	9.4	3.2	0.05	23	10
PS	68	M	19	1	23.7	3.5	174	1.49	0.91	94.6	1.4	high	8.5	6.9	0.02	16	9.5
PS	58	M	5	1	23.1	4.2	182	1.28	0.53	52.68	1.5	high	9.4	4.8	0.13	28	9.8
PS	58	M	4	1	19.7	4	180	1.8	0.94	110.2	1.6	high	10.2	6.8	0.05	28	11.5
PS	69	M	34	1	19.3		122	1.09	0.87	92.45	2.5	high	8.8	5.6	0.18	32	11.5
PS	66	M	29	1	21.2		178	1.56	0.94	120.8	1.5	high	8.5	5.4	0.05	21	10.3
PS	58	F	24	1	18.9		176	2.24	0.97	106.6	2.2	high	9.5	7	0		11.1
PS	51	F	23	1	20.8		102	1.74	1.2	117.9	2.2	high	8.6	3.5			9.1
PS	60	M	23	1	18.1		108	1.29	0.95	110.7	2.2	high	9.5	5.8	0.11	30	11.1
PS	58	M	23	1	20.3		190	1.64	0.94	82.73	2.2	high	9.1	4.8	0.13	25	10.5
PS	54	F	19	1	22.3		156	1.51	1.16	99.18	1.5	high	8.3	4.1	0.55	23	9.7
PS	54	M	18	1	16.4		188	2.07	1.12	70.79	2	high		3.9	0.06	27	9.5
PS	52	F	18	1	15.6		128	1.48	0.96	111.7	1.1	high		4.6	0.11	25	10.2
PS	39	M	17	1	20.9		151	1.63	0.95	104.8	1.8	high		4.1	0.04	25	10.8
PS	68	M	17	1	18.5		134	1.27	1.04	106.9	1.8	high		5.3	0.09	28	11.5
PS	55	M	15	1	27		159	1.74	1.41	130.6	1.3	high	9.5	6	0.1	24	11.1
PS	55	F	15	1	23.3		182	1.56	0.9	90.17	1.5	high	9	5.4	0.05	31	10
PS	61	M	13	1	24.3		179	1.21	0.99	101.1	1.3	high		6.3	0.43	25	9
PS	49	F	13	1	24.5		159	1.44	0.54	79.61	1.3	high		5.1	0.12	29	11.1
PS	57	M	12	1	22.2		144	1.84	1.12	109.6	2.1	high	8.7	5.8	0.9	25	12.7
PS	62	F	12	1	20.2		157	1.1	1.09	132.3	1.5	high		5.1	0.37	29	10.8
PS	66	M	11	1	19		148	1.36	1.18	128.6	1.5	high		3.1	0.09	25	8.1
PS	67	M	10	1	19.3		147	1.08	1.08	118.1	1.3	high		2.4	0.47	28	9.8
PS	78	M	10	1	19.8		162	1.61	0.82	111.7	2.1	high		2.3	0.09	22	10.6
PS	75	M	9	1	18.6		81	1.48	0.77	131.5	1.8	high	8.7	5.7	0.09	38	9.5
PS	46	M	9	1	22.1	3.9	108	1.41	1.42	102.3	1.8	high	8.9	5.2	0.02	26	9.5
PS	66	M	7	1	21		118	1.19	1.02	133	1.8	high		4.5	0.05		10.5
PS	51	M	6	1	21.2			1.69	0.79	92.55	1.5	high		7.8			7.6
PS	53	F	6	1	25.3		146	1.55	0.84	104.9	2.1	high		4.5	0.12	27	10.1
PS	52	F	5	1	26		179	1.92	0.85	106.1	1.5	high	9.8	5.1	0.1	27	10.8
PS	64	M	5	1	22.8		136	1.19	1.09	83.9	1.8	high	6.3	10.3		30	14.6
PS	43	M	5	1	23.5		140	1.27	0.84	111.7	2.1	high	9.7	6.9	0.2	51	11.2
PS	66	F	4	1	19.3		109	1.27	0.9	66.48	1.1	high	8.7	4.8	0.3	20	10.7
PS	45	M	3	1	30.5		128	1.35	0.7	37.57	2.1	high	8.7	3.8	1.21	29	10.8
PS	55	M	3	1	27.2		110	1.46	0.74	92.32	1.6	high	9.1	9.1		30	11.5
PS	69	F	2	1	26.7		180	1.92	0.9	87.79	1.3	high	7.6	5.9	0.03	22	9.7
PS	66	M	2	1	23.5		153	1.64	1.21	93.85	1.3	high	8.5	6	0.1		10.3
PS	69	M	15	1	17.9	3.2	153	1.98	0.67	105.6	1.5	high	8.4	6.4	0.1	28	10.1
PS	62	M	5	1	18.8	4	113	1.36	0.73	83.18	1.5	high	8.2	5.1		28	8.7
PS	83	F	4	1	17.6	4	135	1.36	0.91	101.2	1.5	high	8.7	4.3	0.05	54	10.7
PS	58	M	3	1	22.1	4	199	1.51	0.82	70.29	1.5	high	8.9	4.6	0.33		8
PS	60	M	2	1	25.7	3.9	146	1.45	0.63	100.1	1.6	high		6	0.1	32	9.8
PS	80	F	6	1	25.7	3.5	172	1.78	0.83	113.5	1.8	high		3.5	0.07	21	11.3
PS	77	F	2	1	25.8	3.5	117	1.24	0.75	81.49	1.8	high	9.2	4.7	0.1	24	11.1
PS	75	F	6	1	20.9	3.5	138	1.23	1	126	2.1	high	8.7	6.8	0.05	24	10.2
PS	85	F	4	1	16.9	3.7	166	1.77	0.92	118.4	1.5	high		4.2	0.76	33	9.2
PS	45	M	16	1	20.4	3.8	141	1.54	0.96	97.88	2.1	high	8.9	5.6		31	13
PS	49	M	10	1	23.2	4.5	119	1.15	0.9	113.1	1.3	high	9.6	5.5	0.16	25	10.4
PS	56	M	5	1	21.8	4	128	1.74	1.02	143.9	1.1	high		2.3		31	10
PS	68	F	4	1	18.4	3.4		1.34	0.9	134.3	1.1	high	10.2	4.3		25	10.4

PS	55	M	28	1	19.7	3.6	149	1.51	0.84	80.87	1.4	high		4.9	0.1		9
PS	82	M	4	1	22	3.4	85	1.61	0.74		1.5	high		5.3	0.07	30	9.5
PS	62	M	3	1	23.3	3.7		1.13	0.95		2.1	high	9.2	3.6	0.5	31	8.8
PS	81	M	2	1	22.4	3.4	245	1.47	0.9		1.6	high		5.8	0.1	37	8.8
PS	59	F	9	1	18.9	4.4	131	1.8	0.92	130.1	1.5	high	9	4.3		29	9.2
PS	53	F	5	1	21.8	3.8	191	1.38	1.4	89.51	1.8	high		4.8	0.05		13.2
PS	64	F	3	1	26.5	3.8	106	1.19	1.11	125	1.3	high		5	0.3	35	11.2
PS	73	M	3	1	22.4	4.1	151	1.73	0.77	98.71	1.5	high	8.3	5.3	0.03		11.5
PS	72	F	2	1	16.8	4.1	136	1.99	1.05	64.18	2.1	high	9.7	5.5	0.05	21	8
PS	73	M	6	1	19.1	4	161	1.11	0.97	162.9	2.1	high	9.3	6.2	0.38	27	14.3
PS	67	F	15	1	19.8	3.6	109	1.48	1.21	122	1.6	high	7.9	3.8	0.2	43	9.2
PS	66	F	14	1	20.3	3.4	145	1.34	1.04	103.4	1.8	high	10.3	5.5		30	9
PS	49	M	9	1	25.3	4.1	216	1.27	0.86	111.5	2.1	high	9.4	4.9	0.2	22	10.8
PS	60	F	9	1	23.7	3.7		1.48	0.78	122.6	1.5	high	12.2	4.4	0.68	36	9.7
PS	38	M	9	1	23.9	4	147	2.07	1.06	116.9	2.1	high	9.2	2.9		23	9.7
PS	63	M	8	1	23.5	4	135	1.39	1.03	101.1	1.3	high		3.4	0.31	44	14.5
PS	49	M	8	1	23.3	3.6	190	1.55	0.71	100.1	1.6	high	8.2	5.7	0.04	25	11.1
PS	67	M	7	1	26.2	3.7	116	1.22	0.94	132.7	1.5	high	8.5	6.4	0.4	31	10.8
PS	84	F	7	1	16.2	3.8	201	1.4	0.93	71.53	1.5	high	9.5	6.5		25	12.6
PS	66	M	7	1	24.9	4	140	1.37	0.7	82.52	1.1	high	8.9	5.4	0	19	9.6
PS	68	F	7	1	23.2	3.8	148	1.55	1.05	171.4	1.8	high	10.1	5.1	0.1	32	12
PS	57	M	7	1	24.5	4		1.13	1	122.3	2.1	high	9.9	10.4	1.16	27	11.7
PS	47	M	7	1	23.8	4.2		1.4	1.02	124.2	1.5	high	10.1	2.7			
PS	59	M	6	1	18.4	4	165	1.34	0.9	64.7	1.8	high	10	6.1	0.05	26	10.1
PS	60	F	6	1	21.8	3.4	135	1.76	1.03	117	1.8	high	7.8	4.9	0.17	42	8.6
PS	73	M	5	1	20.1	3.8		1.38	1.03	131.1	2.1	high	8.6	5.7		26	8.9
PS	47	M	5	1	23.5	3.5	139	1.22	1.11	108.3	2.1	high	8.6	5.5	0.05	23	9.3
PS	75	M	4	1	26.9	3.6	156	1.06	0.95	121.7	2.1	high	8.1	5.7	3.5	30	10.3
PS	65	M	4	1	16.7	3.9	180	1.41	0.92	131.7	1.1	high		6.4	0.28	27	
PS	62	M	4	1	20.6	3.5	174	1.64	1.13	71.24	2.1	high	8.7	4.8	0.1	19	11.3
PS	69	F	3	1	27.2	3.8	149	1.7	0.64	80.97	2.1	high	8.7	7.7	1.6	32	11.8
PS	57	F	3	1	19.8	4	171	1.22	0.84	87.74	1.5	high	8.3	6.3	0.3	30	12.8
PS	50	M	3	1	27.5	3.9	158	1.61	0.83	115.8	1.8	high		4.4		23	10.2
PS	58	M	2	1	16.7	3.8		1.46	0.87	86.86	1.3	high	8.6	2.7		24	9.9
PS	65	M	2	1	23.8	3.7	207	1.61	0.77	64.64	1.3	high	9.2	5.6	0.21	26	10.6
PS	49	M	2	1	23.8	3.7	247	1.56	0.86	81.97	1.3	high	8.3	5.7	1.52	26	10.6
PS	60	F	27	1	20.2	4.4	142	2.41	0.87	127	1.3	high	8.2	4.4	0.01	35	8.9
PS	63	M	18	1	20.7	4.2	100	0.87	1.3	133.4	1.6	high	8.5	5.1	0.01	28	10.7
PS	72	M	13	1	22.8	3.8	128	1.3	1.01	135.8	1.8	high		5.9	0.51	23	7.3
PS	43	M	13	1	20.3	4	145	1.2	0.97	97.21	2.1	high	9	5.5	0.36	26	9.3
PS	52	M	12	1	18	4.1	148	1.31	1.13	113.4	1.8	high		5	0.07		9
PS	54	F	12	1	23	3.8	127	1.76	1.19	105.9	2.5	high	10.4	6.2		26	11.2
PS	60	M	11	1	24.7	3.8	143	1.43	0.88	100.7	1.6	high	9.1	5.5		20	11
PS	56	F	10	1	20.6	4.3	195	1.59	0.9	82.6	1.9	high		5.2	0.55	28	10.8
PS	56	F	10	1	19.2	4.4	176	1.45	0.9	120.4	1.6	high		3.1	2.69		8.9
PS	60	F	9	1	18.8	3.9	135	1.41	1.17	119	1.3	high		4.3	0.18	27	10.7
PS	55	M	7	1	23.4	4	143	1.53	0.65	83.13	1.3	high		6.9	0.09	13	10.3
PS	42	M	6	1	23.4	3.7	117	0.91	1.12	140.5	2.1	high	9.4	7	0.06	34	11.7
PS	54	M	6	1	20.2	3.8	151	1.42	0.98	110.4	2.1	high		6.6		21	10.3
PS	60	M	4	1	24.2	4.1	151	1.8	0.81	134.6	2.5	high	10.4	8.1	0.45	19	11.3
PS	53	M	3	1	20	3.5	106	1.61	0.66	63.24	1.6	high		5.2	0.09	20	11
PS	66	M	3	1	20.5	3.8	196	1.04	0.83	147.8	1.3	high	10.2	5.3	0.2	24	9.2
PS	64	F	10	1	18.5		129	1.81	0.6	118.2	1.5	high		5		26	10.4
PS	58	M	7	1	20.1		108	1.62	0.67	58.75	1.8	high	8.5	3.1	0.7	31	12.2
PS	23	F	11	1	19.5	4.1	133	1.27	0.91	125.5	1.6	high	8.8	5.5	0.52	24	11
PS	55	F	22	1	21.1	3.9	186	1.44	1.08	125.7	2.1	high	9.4	5.1	0.5	27	9.8
PS	44	M	16	1	22	3.9	138	1.24	0.71	113.1	1.6	high		8.6			8.6
PS	25	M	2	1	34.1	4.2	113	1.61	0.73	59.28	1.6	high	8.3	4.1	0.04	24	9.7
PS	51	F	17	1	19.9	4.3	137	0.94	1.19	113.8	1.6	high	7.8	7	0.18	28	11.2

PS	65	M	3	1	21.3	3.5	114	1.21	0.99	116.9	2.1	high	9.3	7.3	0.04	32	11.1
PS	51	F	19	1	19.6	3.9	189	1.78	1.03	117.9	1.6	high	10.9	4.1	0.09	29	11.8
PS	65	F	9	1	22.6	3.8	106	1.89	0.96	133.6	1.8	high	8.2	4.4	0.06	40	10.4
PS	64	M	19	1	21.2	3.7	159	1.5	0.93	99.57	1.6	high	9.4	5.6	0.22	23	10.4
PS	60	M	10	1	20	3.7	137	0.73	0.74	126.8	1.8	high	9	7.2	0	30	9.4
PS	29	M	9	1	20.1	4.1	163	0.96	0.76	126	2.1	high		6.1	0.05	43	11.4
PS	65	M	13	1	17.9	4.1	166	1.39	1.13	99.19	1.6	high	8.1	5.9	0.28	26	10.7
PS	78	M	2	1	20	3.5	109	1.36	1.41	122.8	1.1	high	8.3	5.5	0.07	27	11.3
PS	64	F	21	1	14.9	4	247	1.57	0.86	92.59	1.1	high	9.9	6.7	0	34	11.9
PS	61	F	14	1	16.8	4	214	1.35	0.91	100	1.8	high	8.6	5.9	0.03	24	10.8
PS	58	F	10	1	20.6	3.8	146	1.35	1.22	115.1	2.1	high		4.1	0.05	24	10.3
PS	40	F	8	1	14.8	3.6	144	1.54	0.71	72.59	2.1	high	10	4	0.99	29	8.7
PS	39	F	8	1	17.9	4.4	205	1.32	0.82	117.5	1.5	high		4.7	0.07		9.6
PS	68	F	4	1	26.7	3.8	139	1.14	0.76	92.61	2.1	high		7.2	0.04	25	8.8
PS	71	M	3	1	19.4	3.6	170	1.17	0.79	105.2	2.1	high		5.3	0.19	27	10.9
PS	67	F	2	1	26.5	3.7	120	1.28	1	118.2	2.1	high	8.8	8.7	0.17		12.2
PS	69	M	14	1	23	3.7	139	1.8	1.14	118.7	1.3	high	7.8	4.7	2	32	14.4
PS	59	M	13	1	21.8	3.6	146	1.45	0.85	98.11	1.6	high		5.3	0.79	24	11.9
PS	86	M	7	1	21.1	3.3	186	1.6	0.8	131.1	1.5	high		5.7	0.1	22	10.9
PS	48	M	5	1	21.4	3.6	154	1.52	0.94	81.29	1.5	high	9.1	5.8	0.8	26	10
PS	76	M	16	1	18.7	3.5	122	1.57	1	124.1	1.5	high	10.1	5.3	0.02	26	11.6
PS	26	M	5	1	20.8	4	131	2.66	0.76	87.91	1.8	high	9.1	5.1	0.11	29	10.6
PS	72	F	2	1	21.4	4	108	1.45	0.81	68.39	1.5	high	8.1	3.4	0.05	22	9.7
PS	71	F	16	1	18.4	3.8	139	1.54	0.86		1.8	high		3.9	0.07	23	11.4
PS	68	F	12	1	23.4	3.6	120	1.41	0.79		1.8	high	9.5	4	0	30	10.3
PS	67	F	8	1	21.7	3.9	123	1.67	1.2	113.8	1.5	high	8.5	5.1	0	61	11.4
PS	72	M	7	1	23.9	3.7	162	1.14	0.76		2.1	high	8.8	5.9	0	26	10.5
PS	77	F	3	1	20.8	3.4	161	1.56	0.74		2.1	high		4.8	0.12	25	12.6
PS	48	M	31	1	20.2	3.6	135	1.36	0.83	81.19	1.6	high	8	5	0.01	23	11
PS	60	F	28	1	24.9	3.7	205	1.37	0.95	91.44	2.1	high		4.9	0.39	23	10.6
PS	60	M	25	1	21.3	4.4	226	1.65	0.83	88.07	1	high	9.8	5.9	0.19	28	10
PS	66	F	25	1	27.3	4.1	100	1.2	0.75	83.34	2.1	high		3.9	0.05	25	9.4
PS	48	M	22	1	26.6	4.1	154	1.23	0.73	74.7	0.8	high	9.2	4.2	0.08	30	10.6
PS	70	M	21	1	25.5	3.5	182	1.73	0.69	89.75	1.6	high	7.7	4.9	0.1	29	10.4
PS	43	F	19	1	22.8	4.2	146	1.37	0.75	89.31	1.6	high	8.5	4.3	0.11	23	13
PS	44	F	15	1	20	3.9	165	1.39	0.93	88.05	1.6	high	8.7	3.3		26	10.9
PS	59	F	7	1	21.6	3.8	83	1.08	0.73	117.1	2.1	high		4.7	0.16	23	9.4
PS	59	F	6	1	27.7	3.8	128	1.27	0.73	111.1	1.5	high		5.4	0.05	18	12
PS	51	M	5	1	32.6	4.1	147	1.86	1.03	104.5	1.6	high	9.3	5.8	0.18	21	10.9
PS	61	F	2	1	19	4	165	1.71	0.97	90.2	1.6	high	7.6	5.6	0.8	18	10.8
PS	77	M	10	1	21.6	3.6	149	1.85	0.7	75.55	1.5	high	9.1	5.1		16	9.5
PS	86	F	7	1	17.6	3.9	124	1.4	0.66	86.96	1.8	high	8	5.8	0.1	25	9.6
PS	69	M	6	1	20.2	4	200	1.69	1.19	121.8	2.1	high	9.3	7	0.53	29	12.8
PS	78	M	4	1	18.8	4.2	145	1.54	0.79	122.1	1.5	high		4.8			9
PS	76	F	3	1	24.4	4	144	1.78	0.88	111.8	1.8	high	8.9	7.7	0.1	18	11.7
PS	59	M	3	1	25.6	3.9	123	1.35	1.04	104.5	1.8	high	8.7	8	0.08	25	9.5
PS	65	M	7	1	15.6	4.2	142	1.64	0.95	76.47	1.5	high	9	4.8	0.1	30	12.6
PS	48	F	14	1	23.3	3.7	92	1.4	0.51	61.53	1.5	high	7.4	1.4	0.3	40	7
PS	42	M	5	1	23.8	4.2	131	0.96	0.93	102.3	2.1	high	9.2	3.1			
PS	84	F	8	1	15.7	3.4	126	1.42	0.78	82.17	2.2	high	8.8	5.6		26	
PS	68	M	21	1	17.8	3.8	235	1.49	0.75	88	1.1	high	7.9	3.6	2.79	15	10.4
PS	72	M	8	1	18.9	4	101	1.63	0.71	94.45	2.2	high	9.5	4	0.13	35	11.1
PS	66	M	7	1	18.7	3.5	141	1.62	1.01	84.93	1.8	high	9.4	5.7	0.13	28	11.2
PS	71	F	4	1	19.7	3.7	200	1.81	0.63	54.57	1.4	high	9.3	4.3	0.08	25	10.6
PS	66	M	24	1	18.1	3.7	128	1.27	0.89	127.9	2.1	high	9.2	7	0.1	26	11
PS	52	M	23	1	20.4	3.5	195	1.57	0.6	85.15	1.6	high	9.1	4.7	0.05	19	10.2
PS	49	F	19	1	16.5	3.8	235	1.6	0.83	98.69	1.6	high	6.9	6.5	0.39	20	10.6
PS	77	M	18	1	20.2	3.7	139	1.33	0.92	123.1	2.1	high	7.9	2.8	0.24	35	9.9
PS	62	F	14	1	19	3.3	153	2.19	0.64	93.66	1.6	high	10.1	4.6	11.2	24	9.2

PS	68	M	6	1	22.6	4	211	1.7	0.95	98.2	1.6	high	8	5.1	0.46	26	10.8
PS	79	M	4	1	16.6	3.7	157	1.22	0.65	76.36	2.1	high	9.2	8.2	0.38	26	10.8
PS	65	F	36	1	17.6	3.9	114	1.57	1.06	81.53	1.8	high		5	0.46	24	9.7
PS	65	M	26	1	21.1	6.6	144	1.78	0.94	36.9	1.6	high	7.7	7.7	0.1	21	9.8
PS	61	F	23	1	21.4	4.1	182	1.39	1.15	134.5	1.6	high	7.6	4.7	1.6	23	10.2
PS	65	M	19	1	17.8	4.1	164	1.56	1.18	125.4	1.5	high		5.1	0.05	24	10.4
PS	63	M	19	1	19.9	3.7	158	1.93	0.98	86.97	1.6	high	8.4	7.6	3.43	26	8.2
PS	64	M	17	1	18.9	4.6	103	1.29	0.99	103	1.6	high	9.5	3.9	0.1	35	11.6
PS	73	M	16	1	23.4	4	171	1.55	0.82	71.29	1.3	high		4.8	0.31	24	10.1
PS	61	M	15	1	17	3.9	165	1.78	1.08	81.2	1.9	high	10.1	4.5	0.25	25	10.6
PS	60	F	13	1	21.4	4		1.19	1.01	102.1	2.5	high	9.7	5.5	2.96	36	10.3
PS	71	F	13	1	23.6	3.6	141	1.42	0.73	132.8	1	high	8.9	7.9	0	29	11
PS	59	F	11	1	24.6	4	175	1.53	1	128.5	1.8	high		4.9	0.09	26	10.4
PS	46	F	11	1	17.8	4.2	192	1.59	1.02	126.3	1.1	high		4.4	0.42	13	10.6
PS	69	F	10	1	20	3.6	156	1.35	1.2	115.8	1.9	high	9.1	5.5		25	11.6
PS	79	F	9	1	22.4	3.8	214	1.58	1.2	111.5	2.1	high		6.7			11
PS	40	F	9	1	19.9	4.1	154	1.61	0.99	100.8	1.6	high		6.1			11.1
PS	68	F	8	1	22.4	4.4	168	1.42	1.16	100.5	1.8	high	10.6	8.1	0.6	31	9.4
PS	72	M	8	1	24.3	6.7	192	1.49	0.94	87.83	1.5	high	8.8	5.5	0.08	20	10.1
PS	61	M	8	1	20	4.4	142	1.34	1	124.8	1.3	high	9.6	5.2	0	25	9.9
PS	41	F	8	1	31.7	3.8	181	1.49	1.1	121.4	1.3	high	10.9	3.8	0.1	26	9.5
PS	62	F	7	1	17	4.2	164	1.18	1.01	126.9	1.6	high	9.4	5.3	0	33	11.5
PS	65	F	7	1	19.9	3.9	131	1.41	0.69	151.7	1.6	high	11	7.3	0.3	28	10.5
PS	62	M	7	1	27.1	4.1	140	1.28	0.73	108.6	1.6	high	10.8	6.9	0	28	11
PS	47	F	7	1	22.4	3.7	142	1.72	0.84	117.5	1.8	high	9	4.3	0.03	25	9.6
PS	65	M	7	1	23.3	3.6	140	1.29	0.78	123.5	1.3	high	9.8	6.2	0.14		11.2
PS	59	M	6	1	20.3	4	126	1.64	0.8	113	2.1	high	9.7	5.5	0.3	33	11.9
PS	51	M	6	1	22.5	3.7		1.27	0.69	62.92	0.8	high	8.6	4.5	0.1	29	8.4
PS	59	F	6	1	15.7	4.1	142	1.64	1.09	134	1.5	high	8.6	7.4	0.4		9
PS	62	F	6	1	18.9	3.9	133	1.34	0.98	139.5	1.3	high	9	5.5	0	25	10.9
PS	51	F	6	1	17.3	4.2	154	1.34	1.05	104.3	1.3	high	9.4	6.2	0	25	10.8
PS	65	F	4	1	21.1	4.2	200	1.84	1.06	122.5	1.5	high	9.6	3.9	0.14	33	12.7
PS	65	M	2	1	23.8	4.3	206	1.43	0.49	72.6	1.5	high	8.7	5.3	0.05	17	10.7
PS	59	F	21	1	19	4		1.66	0.87	106.7	1.4	high	8.7	4.7			10.3
PS	78	F	15	1	20.8	4.1	194	1.41	0.76	117.4	1.5	high	9.5	5.7	3.61	27	10.9
PS	51	F	14	1	21.3	4.2	192	1.15	1.09	107.3	1.5	high	9.5	5.6	0.05	27	11.9
PS	59	F	13	1	22.6	3.7	100	1.35	1.05	107.2	1.4	high	8.5	5.4	0.2		10.2
PS	37	F	13	1	18.7	3.9	134	1	1.16	106.4	1.6	high	7.4	6.5	0.02	36	10.8
PS	66	F	10	1	18.8	4.2	147	1.51	1.13	118.6	2.2	high	8.9	5.1	0.21	30	11.9
PS	46	M	9	1	20.5	3.5	123	1.46	0.83	76.8	2.1	high	10	6	0		11.7
PS	70	F	9	1	18.8	3.7	118	1.35	0.68	109.3	1.5	high	10	4.2	0.53	27	11.4
PS	57	M	9	1	29.7	4.1	214	1.59	1.04	96.65	1.8	high	8.2	5			10.6
PS	55	M	9	1	18.3	3.8	199	1.43	1.21	105.3	1	high	8.9	5.6	0.1	36	8.7
PS	72	F	8	1	16.4	3.5	272	1.98	1.11	112.3	1.5	high	9.6	6.8	1.98	27	9.4
PS	68	M	8	1	29.5	4.4	218	1.96	0.95	119.1	1.8	high	8.4	3.9		29	12.2
PS	71	F	7	1	19.3	3.4	211	1.28	0.73	89.83	1.3	high	8.9	4.4	0.18	27	10
PS	76	F	4	1	14.3	4	217	1.18	0.97	50.27	1.8	high	10.2	4.1		19	10.3
PS	56	M	3	1	23.2	4	162	1.64	1.19	97.82	1.5	high	8.7	4	0.15	23	10.3
PS	85	F	3	1	22	3.9	145	1.41	1.06	92.38	1.6	high		4.1	1.33	27	11.1
PS	66	M	3	1	26.7	3.7	146	2.09	0.98	101.4	1.4	high	8.6	4.7	0.17	22	10.2
PS	64	M	3	1	37.6	3.9	177	1.67	0.83	61.76	1.8	high	9.4	5.7			11.1
PS	55	F	2	1	20.3	4.2	113	1.3	1.01	122	1.4	high	8.1	4.9	0.9	30	10.9
PS	67	M	2	1	19.6	3.8	186	1.39	0.89	66.01	1.8	high	9.8	4.5			11.1
PS	71	M	2	1	23.3	4.4	144	1.96	1.02	80.33	1	high	9.8	3.7	0.78	19	7.1
PS	59	F	9	1	22.6	4	152	1.59	0.93	112.6	1.5	high	8.1	3.8		29	10
PS	56	M	23	1	19.8	4	179	1.16	1.42	133.9	1.4	high	8.7	4.4		33	10.5
PS	63	F	6	1	25.2	3.6	231	1.42	1.15	116.9	1.3	high	8.3	5.1	0.05	18	8.3
PS	62	F	4	1	19.1	3.7	179	1.97	1.01	119	1.3	high	8.7	5.5	0.96	27	10.1
PS	59	M	37	1	19.6	3.8	164	1.65	0.89	112	1.8	high	10.5	5.9	0.31	25	8.4

PS	73	M	7	1	20	3.9	122	1.17	0.96	126.2	2.2	high	10	7.1	0	20	9.3
PS	73	M	3	1	19.7	3.5	180	1.78	0.69	75.34	1.5	high	8.9	5	0.09	29	10
PS	66	M	22	1	20.1	3.6	165	1.27	0.8	136.6	1.4	high		5.4	0.73	40	11.2
PS	82	F	7	1	18.5	3.9	130	1.33	1.01	129.9	1.5	high	8.7	2.7	1.41	23	10.7
PS	68	M	2	1	25.6	3.7	143	1.19	0.94	65.67	1.8	high	9.1	6.8			10.3
PS	60	M	7	1	22.3	3.9	207	1.3	1.04	139.9	1.5	high	6.9	4.2	0.11	25	10.9
PS	64	F	23	1	18.2	3.5	191	1.25	1.02	120.7	1.4	high	8.2	3.2		51	10.6
PS	59	M	33	1	17.1	3.8	154	1.48	0.93	101.5	1	high	8.9	2.9	0.7	29	10.6
PS	56	F	15	1	19.8	4	136	1.54	1.06	127.2	1.8	high	9	7.8	0.22	19	9.7
PS	51	M	2	1	21.3	3.9	145	1.58	0.99	117.2	2.1	high	8.9	6.5	0.01		11.5
PS	62	M	23	1	26.5	4	126	1.17	0.95	107.6	1.3	high	8.7	5.5	1.71	39	11.3
PS	72	M	2	1	24	3.9	131	0.97	0.96	100.2	1.3	high	8.7	5.5	0.11	20	10.9
PS	65	F	21	1	16.6	4.1	131	1.36	1.2	115.8	1.4	high	8.8	7.2	0		10.2
PS	53	M	29	1	18.5	3.1	123	1.15	0.84	120.3	1.6	high	9.7	4.5	0.02	26	9.4
PS	35	M	2	1	21.8	4.4	180	1.12	0.92	90.63	2.1	high	7.5	6.1	0.12	23	8.2
PS	29	M	6	1	17	4	102	0.95	1.36	106.5	1.5	high	8.8	4.9	0.03	30	11.8
PS	51	M	6	1	24.3	2.8	125	1.02	0.44	40.57	2.1	high	8.9	6.3		29	9.8
PS	61	M	12	1	24.9	4	144	0.99	1.05	129.3	2.2	high	10.9	6.8	0.5	24	10.8
PS	72	F	2	1	29.1	3.7	145	0.85	0.96	116.3	1.4	high	9.5	5.2	0.3	27	6.6
PS	69	F	3	1	21.8	4.1	116	1.68	0.9	64.64	1.6	high	9.5	3	0	24	8.6
PS	87	F	2	1	18.4	4	204	1.2	0.76	86.83	1.8	high	9.3	4.6		20	11.5
PS	72	F	2	1	22.6	3.1	122	1.5	0.72	93.16	1.5	high	8.7	6.4	0.03	25	11.1
PS	66	M	3	1	23.2	3.4		1	0.73	102.8	2.5	high		4.7	0.06	27	9.5
PS	47	F	28	1	20.4	4.1	174	1.12	1.74	119.6	1.5	high	8.7	3.9	0.11	28	11
PS	64	F	29	1	14.4	3.8	118	1.05	1.19	112.6	1.6	high	7.7	6.4	6.9	49	9.2
PS	73	M	7	1	24.4	3.9	202	1.48	0.64	133.1	1.6	high	8.6	4.9	0.06		9.9
PS	67	F	6	1	27.2	3.7	131	1.69	0.98	110.7	1.5	high	10.1	6	0.2	31	10.3
PS	77	M	6	1	20.4	3	121	1.42	1.02	138.6	1.6	high	8.3	5.9	0.44		10.2
PS	71	F	3	1	26	4	161	1.84	0.81	104.9	1.3	high	8.9	3.9		25	10.8
PS	67	F	2	1	26.8	3.8	174	1.18	1.05	100.3	1.5	high	7.3	3	1.51	17	7.7
PS	66	M	2	1	20.4	3.7	134	1.31	0.69	82.93	1.8	high	10.3	5.3	1.33	32	8
PS	69	F	5	1	21.2	3.9		1.45	1.07	168.5	2.2	high	8.6	8			
PS	49	M	2	1	20.6	3.9	184	1.98	0.79	88.34	1.3	high	8.6	3.9	0.35	28	11
PS	77	F	14	1	18.7	3.8	125	1.43	0.8	141.5	1.6	high	7.7	3	0.28	38	9
PS	83	F	5	1	19.5	3.6	144	1.62	0.85	128.7	1.5	high	9.3	4.1			11.2
PS	72	M	11	1	21	3.7	71	1.02	0.71	87.36	1.3	high	7.5	5.1	1.57	31	9.6
PS	57	M	14	1	23.8	4	185	1.26	0.84	128.9	1.8	high		7.4		30	12
PS	75	F	18	1	17.8	3.5	139	1.78	0.81	94.46	1.5	high		5.2	0.11	24	11
PS	63	M	18	1	19	3.6	164	1.56	0.95	103.9	1.6	high		6	0.15	27	10.2
PS	66	M	24	1	18.7	3.8	161	1.29	1.11	141.9	1.8	high	9.3	5.1	0.08	28	10.4
PS	70	M	14	1	19.1	3.5	156	1.44	0.93	148.2	1.9	high	9.5	6.3	2.01		8.3
PS	61	F	13	1	19.8	3.6	142	1.33	0.69	85.63	1.9	high	9.2	6	0.05		10.5
PS	74	M	9	1	22.4	3.7	158	1.19	0.73	93.15	1.8	high	9.5	4.3		21	12
PS	70	F	7	1	28.6	3.8	95	1.38	0.94	130.9	1.5	high		4.3	0.45	18	10.8
PS	76	M	4	1	27.3	3.9		1.33	0.75	130.6	1.1	high	9.2	4.9	0.13	20	10.2
PS	61	M	2	1	26.8	3.9	99	1.08	0.65	95.47	1.5	high	6.5	2.5	0.86	28	5
PS	74	M	2	1	22	4.1	197	1.66	1.03	110.3	1.8	high	9.4	5.4		30	9.8
PS	47	M	20	1	19.1	4	170	0.95	0.99	132.2	1.6	high		6	0.1	14	11.5
PS	53	F	19	1	20.9	3.9	155	1.55	0.95	121	1.6	high	8.4	4.8	0.12		10
PS	63	M	17	1	22.4	3.6	147	1.6	0.88	102.1	1.9	high	9.3	5.3	0.1		9.2
PS	56	M	11	1	25.2	3.6	176	1.21	0.75	105.3	1.5	high		6	0.22	25	14.7
PS	65	F	9	1	21.8	3.5	204	1.38	1	111	1.9	high	7.6	4.8	0.06		10.7
PS	48	F	7	1	24.2	3.9	189	1.43	0.84	116.3	1.6	high	8.9	7.1	0.56		10.8
PS	57	M	6	1	29.3	3.5	147	1.93	0.88	165.7	1.4	high	9.6	6	0.64	28	8.9
PS	64	M	6	1	21.1	4.1	172	1.29	1	128.5	1.3	high	9	5.6	0.16	25	12
PS	76	F	5	1	21.7	3.4	164	1.6	0.89	140.5	1.9	high	8.3	7.1	0.16		10.6
PS	73	F	3	1	21.3	3.6	193	1.45	0.86	79.3	1.6	high	8.8	5	0.12		10.1
PS	87	M	2	1	17.3	3.6	229	1.76	0.61	124.2	1.6	high	8.3	4.2	0.56		10.6
PS	80	F	5	1	19.6	3.6	185	1.56	0.69	117.8	2.1	high	9.1	6	1.79		8.6

PS	75	M	8	1	14.5	3.8	119	1.21	0.99	93.96	1	high	9.3	4.7	0.1	41	9.4
PS	69	M	2	1	19.3	4.1	130	1.38	0.96	61.31	1.8	high	6.9	5	0.08	30	12.4
PS	59	M	10	1	22.1	3.5	137	1.61	0.96	117	1.5	high	9.8	4.7	0.26	26	9.7
PS	65	M	4	1	20	4.1	120	0.96	0.68	95.44	1.3	high	7.7	2.8	0.3	28	6.1
PS	62	M	15	1	18.6	4.1	183	1.2	0.73	134	1.8	high	9.5	7.6	5.4	24	8.8
PS	60	F	26	1	19.2	3.7	168	1.23	1.11	105.3	2.1	high		4.9	0.14		13.5
PS	56	F	7	1	26.4	3.9	90	1.63	0.79	109.5	1.8	high	9.7	6.1	0.3	23	11
PS	71	M	8	1	25.1	3.7	164	1.19	1.08	134.9	1.5	high		9	0.28	23	9.7
PS	49	F	11	1	20.3	3.8	211	1.56	0.95	98.48	1.9	high	8.2	10.4	0.05		12.7
PS	61	F	11	1	19.8	3.9	203	1.61	0.68	117.3	1.6	high	8.5	4.5	0.07		10.5
PS	55	M	27	1	22.5	3.7	145	1.21	0.77	73.44	1.9	high	8.3	5.4	0.22		8.9
PS	71	F	2	1	20	3.5		1.57	0.81	70.24	1.5	high	10.5	4.6	0.1	25	10.8
PS	65	F	22	1	19.3	3.7	165	1.7	0.87	106	1.3	high		4.5		17	8
PS	75	M	3	1	18.7	3.6	180	1.76	0.67	91.77	1.9	high	7.7	4.2	3.3		
PS	68	F	16	1	20.9	3.6	167	1.3	0.8	112.8	2.1	high	9.7	7.1	0.02	26	10.8
PS	61	M	24	1	24.5	3.7	117	1.41	0.97	94.38	1.9	high	9.4	6.6	0.08		11.7
PS	66	F	11	1	16.1	2.2	199	1.58	1.07	88.96	1.6	high	9.5	5.9	0.18		9.7
PS	53	M	25	1	15.7	3.6	202	1.37	0.83	101.4	1.6	high	8.9	5.5	0.05		10.6
PS	61	F	7	1	16.1	3.6	144	1.69	0.8	126.3	1.3	high	8.5	7	0.08	21	10.3
PS	78	M	3	1	21	4.3	178	1.81	0.82	86.03	1.5	high	8.8	4.1	0.17		10.6
PS	56	F	23	1	18.2	4	143	1.51	1.02	108.6	0.6	high	8.1	6.5	0.1	23	10
PS	74	M	15	1	15.5	3.5	92	1.46	1.03	97.43	1.3	high	10.1	4.4	0.1	21	12.4
PS	77	M	5	1	23.1	3.9	108	2.15	1.04	117	1	high	8.8	3.4	0.5	33	8.7
PS	78	M	5	1	18.7	3.4	151	1.43	0.8	117.2	1.5	high	7.3	4.6	0.1		10
PS	72	F	5	1	22.3	4.1	136	0.92	1.14	112.7	1.3	high	8.5	3.5	0.2	4	11.9
PS	64	M	4	1	22.8	3.7	190	2.18	0.79	116.6	1.5	high	9.6	6.6	0.13	29	10.6
PS	50	F	11	1	18.2	4	108	1.22	0.8	94.43	1.8	high	8.2	4.3	0.02	32	10.4
PS	65	M	16	1	17.4	4.2	132	1.07	0.96	98.87	1.4	high	9	3.3	0	12	9.7
PS	79	M	2	1	25	4	144	1.43	0.71	149.1	1.3	high	9.7	6.2	0.14	27	12.6
PS	77	M	13	1	17.3	4.2	198	1.48	0.97	116	1.4	high	8.9	5.6		15	10.9
PS	56	M	3	1	21.2	4	184	1.61	0.88	72.52	1.8	high	6.9	6		23	11
PS	80	M	3	1	20.5	4	87	1.34	1.13	101.7	1.8	high	7.6	4.9	0.58	21	
PS	60	F	2	1	25.4	4.3	209	1.07	0.91	38.14	1.3	high		7.9		31	10.3
PS	73	M	8	1	21	4	174	1.44	1.01	139	2.5	high	8.1	6.1	0.18	35	10.6
PS	77	M	3	1	19.9	3.5	229	1.41	1.06	101.4	1.8	high	9.2	4.4	0.2	29	11.2
PS	53	F	11	1	19.9	3.8	113	1.38	0.84	93.39	1.8	high	8.9	3.8		17	11.7
PS	65	F	2	1	23.9	4.3	151	1.58	1.06	101.9	1.8	high	8.2	4	0.1	27	9
PS	81	M	3	1	18.6	4.2	95	1.55	1.06	97.65	2.1	high	10.3	6.3	0.13	42	8.8
PS	60	M	4	1	23.5	3.7	153	1.11	0.81	105.4	1.3	high		3.4	0.9		9.2
PS	61	M	2	1	30.1	3.8	191	1.11	0.86	77.62	2.1	high	9.1	6.1	0.1	22	10.4
PS	58	M	3	1	21.1	4.4	178	1.83	1.08	122.1	1.4	high	8.2	4.9		23	11.1
PS	61	F	21	1	24.4	3.6	192	1.71	1.08	115.3	1.5	high	8.8	5.6	0.01	18	8.7
PS	73	M	3	1	19.8	3	135	1.58	0.71	48.38	1.5	high	9	4.2	1.05	21	11.1
PS	60	M	24	1	19.9	3.8	164	1.11	1	104.9	1	high		5	0.05		8.3
PS	63	F	3	1	20.4	4		1.59	0.98	99.62	2.1	high		4.7	0.04		12.3
PS	62	M	6	1	16	3.7	144	1.18	0.96	100.8	1.8	high	9.8	5.3	0.21	23	9.9
PS	49	F	15	1	18.2	4	154	1.06	1.06	85.88	2.1	high	8.3	4.5	0.2	32	12.1
PS	82	M	12	1	18.6	3.2	193	1.52	1.02	123.5	2.1	high		4.3	0.03		10.5
PS	66	M	4	1	21.4	3.7	149	1.42	0.87	102.6	2.1	high	8.5	6.1	0.1	23	9
PS	81	M	7	1	19.5	3.7	90	0.91	0.9	132.9	1.5	high	9.5	7.1	0.02	43	10.9
PS	77	M	3	1	20.5	3.7	183	1.92	1.03	135.8	1.4	high	9.8	5.9		19	11
PS	70	M	8	1	21.9	3.2	191	1.13	0.67	78.33	1.5	high	9.1	5.1	0.02	20	11.8
PS	67	M	10	1	31.5	3.7	229	1.47	0.8	121.8	2.1	high	9	9.3	0.43	35	9.9
PS	64	F	16	1	22	3.7	168	2.24	1.07	114.4	2.1	high	7.5	4.8	0.68	29	9.4
PS	68	M	21	1	25.3	3.7	142	1.4	0.88	135.9	1.4	high	9.3	5.1	0.2		11
PS	33	F	18	1	19.4	4	167	1.66	0.82	123.5	2	high	8.6	2.5	0.4		13.7
PS	43	F	16	1	18.2	4.4	105	1.37	0.87	112.2	1.8	high	8.9	5	0.65	27	10
PS	69	M	15	1	21.1	4.2	195	1.01	1.16	169.2	2.1	high	9.6	9.8	0.4		13.8
PS	66	M	12	1	19.5	3.8	217	1.65	0.93	130.4	1.3	high	10.4	6.6	0.27	28	11

PS	66	F	11	1	23.2	3.7	238	1.44	1.01	146	1.5	high	10.2	5.4	0.04	24	10.5
PS	77	M	11	1	23.3	3.2		1.39	0.74	102	2.1	high	8.5	6.8	0.1	26	11.2
PS	55	M	7	1	27.9	4.2	162	1.33	0.87	122.5	1.6	high		3.4	0.05	31	10.1
PS	69	F	6	1	19.8	3.7	148	1.95	0.76	118.4	1.8	high	10	6.4	0.1	21	10.5
PS	67	M	6	1	21.3	4.4	134	1.28	0.99	98.86	1.3	high		7	0.13	27	13.1
PS	49	M	6	1	25.5	4	120	1.34	0.87	162.6	2.1	high	9.6	7.7	0.91	26	11.3
PS	61	F	6	1	18.6	4	175	1.64	1.03	113.5	1.3	high	9.7	4.9	2.6	31	9.6
PS	73	M	5	1	21.6	3.9	121	1.63	0.74	102	1.5	high	8.3	3.9	0.76	32	10
PS	84	F	4	1	16.8	4.1	153	1.67	0.92	107.6	1.6	high	9.7	4.6	0.1	28	10.5
PS	61	M	2	1	23.4	4		1.3	1.16	108.4	1.6	high	8.7	6.6	0.3	25	11.3
PS	70	F	13	1	16.8	4.2	187	1.75	0.98	128.8	1.3	high	9.8	4.2	0.1	31	10.9
PS	68	M	3	1	21.7	3.9	213	1.49	1.01		1	high	9.3	3.8	0.1	21	10.1
PS	63	F	36	1	18.4	3.6	154	1.24	1.09	108.8	2.1	high	8.4	10	0.1	34	11.6
PS	58	F	25	1	16.7	3.7	154	1.47	0.87	104.1	1.5	high	8.7	5.7	0.06	26	9.4
PS	54	M	25	1	19.2	3.8	131	1.82	0.92	111.9	1.5	high	8.4	4.3	0.07	21	10.7
PS	74	F	24	1	19.9	3.8	161	1.68	1.03	110.4	1.8	high	9.1	8.9	0.1	22	12.7
PS	54	F	21	1	20.5	3.7	129	1.44	1.01	123	1.3	high	9.1	7.6	1.3	27	10.8
PS	60	M	19	1	20.6	3.9	103	1.55	0.93	97.28	1.6	high	7.8	5.4	0.3	29	11.1
PS	73	F	18	1	22.2	3.4	179	1.05	0.92	130.2	1.3	high	8.8	5.8	0.1		9.8
PS	37	F	18	1	18.1	4.1		1.76	0.9	118.6	1.5	high	9.8	5.1	0.1	23	10.9
PS	38	F	17	1	18.5	3.7	168	1.4	0.91	116.8	1.5	high	8.8	6.6	0.18		10.4
PS	73	M	17	1	21.3	3.2	189	1.33	0.85	120.5	2.2	high	7.8	4.4		19	10
PS	59	F	17	1	19.4	3.7	152	1.16	1.2	129.9	1.5	high	9.6	6.2	0.02	17	11
PS	68	F	17	1	17.7	3.4	120	1.6	1.07	131.1	1.6	high	9.9	6	0.1	40	10.4
PS	61	F	16	1	17.9	3.4	156	1.29	0.82	128.4	1.8	high	10.2	5.4	0	27	11.3
PS	52	M	16	1	22.2	3.7	170	1.91	0.85	105.4	1.6	high	8.7	4.6	0.05	23	11.1
PS	71	F	16	1	19.5	3.8	120	1.08	1.17	121	1.5	high	8.6	5.9	0.3	29	11.4
PS	66	F	16	1	21.5	3.8	228	1.42	1.01	123.8	1.3	high	9.3	6.9	0.2	21	10.8
PS	52	F	15	1	18.1	3.5	106	1.88	1.12	135	1.5	high	9	7	0.01	29	10.4
PS	60	F	15	1	23.6	3.4	116	1.01	0.9	100.1	1.8	high	10	7	0.04	40	11.7
PS	67	F	14	1	18	3.1	119	1.64	0.96	136.8	1.8	high	8.6	6	0.07	24	9.5
PS	68	F	14	1	21.8	3.7	182	1.95	0.75	101.5	1.3	high	8.2	4.4	1	15	9.6
PS	59	M	13	1	16.8	3.6	168	1.5	1.03	78.11	1.3	high	9.1	5.3	0.37	28	8.6
PS	64	F	12	1	21.8	4	170	2.05	1	99.72	1.9	high	8.9	5.3	0.4		11.3
PS	33	M	12	1	20.4	3.9	189	1.48	0.93	96.31	1.5	high	9.5	5.1	0.39	14	11.7
PS	51	M	12	1	24.7	3.8	155	1.25	1.11	115.8	1.3	high	8.2	4.7	0.08	27	10.4
PS	59	F	12	1	18	3.4	145	1.56	1.39	70.17	1.5	high	8.3	4	0.07	37	9.3
PS	60	M	12	1	16.4	3.8	107	1.45	1.26	87.96	1.9	high	9.1	4.5	0.02		10.9
PS	54	M	12	1	20.7	3.3	116	1.29	0.98	98.39	2.5	high	10.3	4.8	0.06	27	10.3
PS	35	F	11	1	32.4	4.1	166	1.8	0.95	99.61	1.5	high	8.8	4.1	0.01	28	11.4
PS	71	M	11	1	17.4	3.5		1.31	1.15	106.8	1.8	high		4.6	0.29	28	10
PS	71	M	10	1	17.9	3.6	100	1.71	1.05	135.2	1.9	high	10	3.8	1.04		10.8
PS	54	M	10	1	33.5	3.2	170	1.61	0.78	99.69	1.9	high	9.7	4.8	0.03		8.2
PS	48	M	9	1	16.6	4	186	1.5	0.73	96.17	1.3	high	9.9	6.9	0.44		12.1
PS	62	M	9	1	18.3	3.3	104	1.37	1.02	113.8	1.5	high	8.4	3.6	0.74	45	7.5
PS	71	M	9	1	23.1	3.3	117	1.2	0.88	111.6	2.1	high		6	0.02	34	11
PS	62	M	8	1	31.4	3.6	148	1.43	0.89	111.4	1.8	high	8.3	3.5		26	12.8
PS	61	M	7	1	21.8	3.6	105	1.28	0.92	113.4	1.9	high	10.2	4.2	0.08		10.7
PS	58	M	7	1	20.9	3.9	125	1.41	0.93	131.3	1.9	high	10.3	3.3	0.6		8.9
PS	44	M	7	1	25.2	4	118	0.99	1.19	130.7	1.9	high	10	4.7	0.13		11.3
PS	72	M	7	1	21.9	2.5	131	1.23	0.77	97.23	1.8	high	8.8	4.5	0.38	28	11.5
PS	43	M	7	1	20.9	3.8	137	1.51	0.76	91.07	1.5	high		4.3	0.16	39	12.4
PS	71	M	6	1	21.2	3.6	137	1.36	1.11	116.2	2.1	high	8.3	8.3	0.09	33	9.9
PS	51	M	5	1	23.3	3.8	163	1.32	0.96	101.6	1.6	high		5.8	0.28	27	11.7
PS	54	M	5	1	29.1	3.9		1.22	0.98	76.02	2.1	high	9	4.8		28	10.1
PS	48	F	4	1	37.1	3.4	119	1.28	0.57	73.22	2.1	high	10.3	4.7	0.24	30	10.5
PS	57	M	4	1	18.4	3.5	152	1.82	1.59	72.75	2.1	high	7.3	8			9.3
PS	67	M	4	1	19.2	3.8	146	1.36	1.3	96.76	2.1	high	9.1	4.7	0.1	31	11.4
PS	66	M	3	1	23.4	3.7		2.1	0.81	111.8	2.1	high	8.5	6	0.1	39	11.3

PS	54	M	3	1	21.5	3.7	150	1.74	0.89	120.6	1.9	high	8.5	4.8	0.08		10.9
PS	58	M	3	1	22	3.3	188	1.38	0.93	74.72	1.5	high	8	5.8	0.03	33	11
PS	77	M	3	1	20	3.8	132	1.39	0.91	86.38	1.6	high		6	0.08	28	12.1
PS	59	M	3	1	22.5	4	207	1.06	0.97	108.5	1.5	high	8.1	7.2	0.1	23	9.3
PS	50	M	3	1	21.3	3.9	138	1.29	0.78	97.06	2.1	high	9.1	5.1			9.8
PS	70	F	2	1	22.6	3.7	156	1.18	0.88	97.12	1.3	high	9.6	4.9	0.1	26	6.9
PS	81	F	2	1	22.9	3.4	137	1.33	0.81	86.83	1.8	high	9.1	6	0.14	21	8.1
PS	28	M	2	1	28.2	4.4	97	1.42	0.94	103	2.1	high	9.7	6.1	0.18	26	9.4
PS	54	M	2	1	22	3.5	152	1.56	0.74	81.78	1.5	high	9.6	4.9	0.02	26	10.5
PS	71	M	2	1	20.5	3.2	171	1.39	0.89	112.7	1.3	high		2.8	0.23	28	10.7
PS	60	F	10	1	18.8	4	176	1.34	0.91	116	1.8	high	10.2	6.2	0.06	37	11.3
PS	56	M	4	1	23.7	3.8	170	1.07	0.94	120	1.5	high	8.3	6.1	0.26	27	10.7
PS	60	M	31	1	18.8	3.4	175	1.54	0.86	84.63	1.6	high	9.2	4.6	0.02	25	10.4
PS	79	M	16	1	18.5	3.6	137	0.91	0.99	78.68	1	high		4.1	4.9	27	10
PS	53	F	16	1	21.5	3.9	149	1.42	1.17	145.4	1.5	high	8.9	3.9		34	10.9
PS	46	F	14	1	17.8	3.6	216	2.1	1.39	136.2	1.3	high	8.3	4.5	5.71	24	9.5
PS	72	M	14	1	18.7	3.3		1.42	0.89	113.9	1.6	high		8.4			11.7
PS	68	F	13	1	25.4	4	131	1.13	0.79	125.5	1.5	high		4.3	0.66	48	9.6
PS	77	M	12	1	20.2	3.7	93	1.36	0.89	108.7	1.8	high	8.4	6	0.07	26	9.4
PS	60	F	10	1	16.7	3.8	118	1.01	0.94	92.58	1.6	high		4.6	0.06	38	10.6
PS	28	F	9	1	20.8	3.7	163	1.02	1.25	93.43	2.1	high		3.2		32	9
PS	53	M	8	1	19.3	3	169	1.42	0.92	128.2	1.3	high		3.5	0.1	24	10.8
PS	68	M	7	1	25.5	3.8	133	2.09	0.6	118.3	1.5	high	9.9	5.7	0	33	9
PS	70	M	7	1	22.1	4.1	169	1.7	0.82	132.2	1.5	high	8.6	4.2	0.5	27	10.5
PS	63	F	6	1	26.4	4	132	1.72	0.95	112.4	1.5	high	10.1	3.6	1.5	25	10.2
PS	59	F	6	1	26	3.4	144	1.32	0.81	71.53	1.6	high		6.3	0.12	32	11
PS	72	M	6	1	17.6	4.1	122	1.19	0.86	127.3	1.6	high	7.9	6.5	0.09	39	11.2
PS	35	M	6	1	18.9	4.1	137	1.7	1.09	106.2	1.5	high		6.5			14.9
PS	65	F	6	1	19.5	3.9	143	1.07	1.12	109.4	1.8	high	9.1	6.3	0.06	22	11.3
PS	69	F	6	1	23	3.9	128	1.29	0.99	89.98	1.8	high	10.8	5.7	0.03	23	11.5
PS	73	M	6	1	22.4	3.6	102	1.38	0.8	132.5	2.2	high	8.5	5.5		26	10
PS	57	M	6	1	20.8	3.9	105	1.06	0.6	112.3	1.8	high	8.9	4.5	3.01	44	8.6
PS	66	M	5	1	17.7	3.5	132	1.06	0.81	122.3	2	high	9.1	7.5		46	9
PS	70	M	5	1	19.4	3.8	213	1.41	0.85	116.6	1.3	high	10.6	7.8	0.02	25	11.2
PS	62	F	5	1	21.6	3.3	117	1.54	0.86	91.48	2.5	high	8.2	5.6	0.24	48	12.9
PS	66	M	4	1	15.4	3.4	166	1.41	1.01	87.01	1.8	high	9.8	5.3	0.2	28	9.8
PS	71	F	4	1	25.6	2.9	136	1.72	1.02	117.9	2.1	high	10.2	4.3	0.01	28	11.1
PS	68	F	4	1	34.8	3.5	133	1	0.83	103.1	1.6	high	7.5	8.4		29	10.4
PS	79	M	3	1	20.5	3.7	150	2.3	1.1	111.3	1.3	high	8.5	2.3	0.1	27	9.5
PS	57	M	3	1	24.1	3.7	134	1.34	0.68	109.4	1.8	high	9.1	3.5	0.9	21	10.4
PS	59	M	3	1	18.6	4.3	192	2.45	0.78	75.57	1.5	high	9.2	3.5	0.07	18	10.6
PS	74	M	3	1	22	3.7	160	1.14	0.79	118	1.1	high		3.3	1.86		10
PS	64	M	3	1	22.5	3.4		1.5	1.08	129.8	1.6	high	8.6	4.1	0.25	21	9.8
PS	70	F	2	1	16.1	4.3	113	2.25	0.91	77.43	1.3	high	9.6	3.4	1.02	20	10.2
PS	63	M	2	1	20.7	3.9	161	1.87	0.72	94.65	1.5	high	9.3	4.6	0.99	28	11
PS	59	F	2	1	20.4	4.1	166	1.22	0.76	96.26	1.5	high	8.6	9.8	0.6		9.8
PS	77	F	2	1	16.6	3.6	125	1.34	1.12	63.71	2.1	high	8.7	6.2		32	10.9
PS	69	M	7	1	19.3	3.7	148	1.22	0.98	95.62	1.5	high	9.1	4.1	0.1	23	10
PS	59	M	30	1	19.2	4.6	165	1.03	1.01	91.72	1	high		5.1	0.05		10.3
PS	57	M	26	1	18.8	4	159	1.74	0.72	118.9	1.6	high	9.2	4.8	0.02	25	9.5
PS	76	F	25	1	17.3	3.7	117	1.05	0.9	105.8	2.1	high		5.5	0.08	33	12.6
PS	69	M	24	1	20.5	4	177	1.88	0.93	95.55	2.1	high	9	4.7	0.1	28	11.2
PS	65	M	23	1	19.8	4.6	127	1.32	0.85	112.4	2.2	high	9.2	6.4	0.1		10.8
PS	68	F	8	1	24.3	3.5	185	1.77	0.91	98.96	2.1	high	10.1	8	0.15	36	10.3
PS	65	M	15	1	24.7	4	187	1.79	0.75	83.06	1.8	high	7.8	4.7	0.09	32	11.2
PS	81	F	15	1	20.3	4	190	1.25	0.98	125.1	2.1	high		5	0.2	19	12.3
PS	58	M	6	1	22.8	3.9	155	1.45	0.91	89.38	1.6	high	8	2.3	0.09		11
PS	61	M	12	1	21	3.9	143	1.23	0.91	94.2	2.1	high	8	6.5		29	10.3
PS	42	F	7	1	22.1	4.5	166	1.38	0.85	82	1.5	high	10.5	6.7			9.2

PS	45	F	7	1	28.5	3.6	150	1.18	0.78	115.2	1.6	high	9.5	5.5			12.8
PS	48	F	6	1	21.2	3.7	136	1.22	0.92	88.34	2.1	high	8.7	5	0.7	30	9.2
PS	59	M	4	1	21.8	4.6	147	1.2	0.97	103.9	1.8	high	9.9	5.4	0.06	27	12.3
PS	42	M	3	1	22.8	4.9	129	1.31	0.79	93.18	1.3	high	8.4	7.6	0.3	37	11.1
PS	60	M	2	1	24	4.1	149	1.19	0.69	67.2	2.1	high	10.1	4		35	12
PS	73	M	2	1	23.1	3.5	142	2.26	0.78	82.19	1.3	high	9.6	5.1	0.11	29	9.9
PS	71	M	14	1	21.5	3.9		1.65	0.88	143.8	1.8	high	7.6	5.4	0.2		11.6
PS	76	M	9	1	18.8	3.7	202	1.69	0.78	124.8	1.6	high	9.8	4.6	0.05	31	10.3
PS	57	M	8	1	22.2	4.4	120	1.68	0.9	90.68	1.3	high	8.8	3.8	0.22	32	9.1
PS	46	M	12	1	18.2	3.7	141	1.36	1	121	1.9	high	8.5	4.4	0.04		10.4
PS	61	F	11	1	22.5	4.2	138	1.55	0.87	142.1	2.1	high	9.9	5.4	0.05	28	10.7
PS	73	F	4	1	23.6	3.7	156	1.56	0.69	125.6	2	high	9.8	6.2	0.63	21	9.6
PS	44	M	23	1	23.7	4.1	157	1.41	1.16	129.4	2.1	high	9.5	4.6	0.1	24	10.1
PS	86	F	6	1	14.3	2.7	188	1.62	0.54	54.76	1.4	high	9	3.9		19	11.3
PS	60	M	5	1	18.5	4.8	173	1.41	1.02	71.4	1.5	high	9.7	6.2	0.4	38	8.1
PS	49	F	3	1	18.2	4.1		1.31	0.81	84.25	1	high	10.3	5.4	0	24	13.6
PS	65	F	18	1	16.2	4.5	100	1.53	1.11	98.92		high	7.6	7.5	0.58		
PS	72	M	17	1	19.5	3.8	194	1.17	1.02	126	1.6	high	8.7	2.4	0.02		10.2
PS	59	F	23	1	19.4	3.8	94	1.37	1.1	136.1	2.1	high	9.1	4.5	0.94	47	10.1
PS	65	F	21	1	19.3	3.6	137	1.33	1.28	131.1	2.1	high	8.4	5.6	0.08	25	11.9
PS	59	M	19	1	19.2	3.9	263	1.13	0.95	134.3	1.9	high	8.4	4.5	0.13		12.3
PS	65	F	18	1	21.6	4	181	1.59	0.93	123.2	1.3	high	7.5	4.5	0.03		9.9
PS	62	F	9	1	19.7	3.9	249	1.27	0.88	110.5	2.1	high		5.6	1.85		9.8
PS	87	F	8	1	21	4.2	189	1.7	0.78	75.88	1.8	high	8.5	3.9	0.1	25	11.1
PS	70	M	5	1	18.7	4	162	1.68	0.92	130.8	1.9	high	9.6	5.8	0.41		11.4
PS	58	F	27	1	16.3	3.9	132	1.48	0.92	153.9	1.3	high	10.1	6.4	0.13	35	10.1
PS	82	F	11	1	16.7	3.5	148	1.64	0.82	117.2	1.8	high	7.6	3.9		13	10
PS	76	M	2	1	23.8	4	174	1.3	0.72	130.3	1.3	high	8.1	3.9	0.16	11	10.2
PS	48	F	2	1	18.9	4.2	118	1.16	1.03	56.5	2.1	high	9.1	5.6		31	9.6
PS	72	M	2	1	19.4	3.8	167	1.73	0.97	126.5	1.6	high	9.1	3.2		23	10.9
PS	72	M	10	1	26.3	4.2	180	1.16	0.78	121.8	1	high	8	2.2		9	10.9
PS	37	F	5	1	19.7	4.1	160	1.41	1.02	87.63	2	high	7.8	3.9	0.05	24	10
PS	58	M	2	1	22.4	3.8	136	1.64	0.75	53.31	1.5	high	8.5	6	0.1	25	9.2
PS	49	M	2	1	33.9	3.4	148	1.24	0.65	79.33	2.1	high	9.6	5.7	0.21		9.7
PS	58	M	2	1	23.7	4.1	164	1.52	0.87	97.59	1.6	high		6.3	0.15	37	8.5
PS	68	F	29	1	20.4	3.7	155	1.15	1.02	123.7	1.6	high	8.7	5		19	9.2
PS	59	F	8	1	19.4	4.6	141	1.09	0.94	82.11	1.3	high		5.6	0.21	23	9.6
PS	61	F	18	1	17.4	3.9	157	1.35	1	110.4	1.8	high		4.9	0.03		10
PS	93	F	10	1	17.4	3.4	110	1.52	0.81	76.39	1.8	high	7.9	5.3		31	11.2
PS	72	F	9	1	18.9	3.9	147	1.52	0.67	89.59	2.1	high		5.2	0.05	26	10.2
PS	72	F	7	1	15.4	3.7	141	1.52	0.89	94.08	2.1	high	10.7	3.9	0.15	33	10.5
PS	66	M	2	1	19.5	4.1	146	1.45	0.91	97.52	1.6	high	9.7	6		30	13
PS	55	F	14	1	16.8	3.8	164	1.39	1.05	114.3	1.6	high	9.7	3.6	1.35		11.3
PS	69	F	17	1	15.9	4.1	166	1.55	1.1	134.4	1.9	high	9.6	6.5	0.11	29	
PS	75	F	12	1	17.3	3.9	134	1.61	0.93	113.9	1.9	high	8.3	5.7	0.33	32	9.5
PS	69	M	7	1	21.4	3.5	120	1.21	1.07	105.2	2.1	high	9.7	3.9	2.8		8.6
PS	54	M	2	1	23	4.2	225	1.01	0.83	103.6	1.5	high	9.9	5.1	0.13	18	12
PS	49	M	3	1	25.6	3.9	92	1.31	0.77	127	1.5	high	8.2	3.5	0	30	10.6
PS	47	F	17	1	19.6	3.9	163	1.61	1.07	126.3	1.9	high	9	4.6	0.21	26	10.8
PS	63	M	28	1	20.9	3.6		1.4	1.12	113.6	1.6	high	8.8	3.5		23	9.7
PS	69	M	10	1	22.1	3.4	102	1.45	0.94	156.1	1.5	high	9.4	5.7	3.4	34	8.7
PS	64	M	16	1	23.2	3.7	138	1.26	0.92	122.9	1.6	high		4.2		31	10.2
PS	60	F	2	1	34.7	3.6	98	1.5	1.04	107.7	1.6	high	9	3.8	0.05	22	11.4
PS	63	M	15	1	21.6	4.1	133	1.27	0.82	112.2	1.8	high	9.1	4.5	0.17	32	11.7
PS	79	F	21	1	23.5	3.8	126	1.29	0.88	136.3	1.9	high	9.7	5.6	0.05	25	11.4
PS	58	F	20	1	24.6	4.1	165	1.11	0.8	139.2	2.1	high	10	5.1	0.06	23	11.8
PS	73	F	11	1	16.6	4.2	195	1.95	0.94	88.6	1.6	high	8.2	5.2	0.16	29	11.4
PS	73	M	2	1	23.2	3	152	1.5	0.89	125.1	2.1	high		5	0.07	27	10.5
PS	68	F	2	1	21.7	3.5	131	0.93	0.83	106.5	1.5	high	6.4	2.6		33	7.9

PS	67	M	27	1	19.1	3.8	190	1.37	0.71	87.27	1.9	high	9.5	4.3	0.05	27	11.8
PS	66	F	19	1	17.4	3.9	152	1.58	0.64	107.5	1.5	high	9.3	6	0.02	26	11.6
PS	53	F	13	1	21.7	3.6	161	1.6	0.98	99.43	1.9	high	9.4	5.1	0.05	28	11.8
PS	59	F	11	1	21.8	4.3	116	1.27	0.82	130.3	2.1	high	9.1	5.4	0.02	27	11.4
PS	58	M	4	1	25.9	3.9		0.77	0.74	104.6	2.1	high	8.6	8.1	0.02	29	13.7
PS	56	M	18	1	17.3	4.5	187	1.32	0.8	84.03	1.5	high	10.2	3.8	0.16	22	12.1
PS	53	M	14	1	17.6	4.3	137	0.95	0.83	114.7	2.5	high	9	7.8	0	22	11.2
PS	67	F	6	1	24.2	3.5	138	1.48	1.18	168.5	1.6	high	9.4	4.8		27	9.7
PS	66	F	33	1	18.2	3.5	128	1.36	0.84	70.15	1.2	high	9.1	4.8	0.05	37	9.1
PS	59	M	5	1	24.2	4.2	187	1.6	0.88	108.8	1.1	high	9.7	5.8	0.01	27	11.5
PS	63	F	13	1	25.4	3.9	158	1.17	0.75	109.9	2.1	high	8.3	5.2	0.02	25	10.4
PS	54	M	7	1	27.3	3.9	104	1.43	0.8	122.6	2.1	high	5.8	3.2	0.94	22	8.7
PS	61	M	2	1	17.3	3.9	168	1.63	0.93	97.06	2.1	high		4.3	1.21	25	9.3
PS	60	M	6	1	23.1	3.9	173	1.28	0.84	133.2	2.1	high	10.3	6.8	0.18	25	10.7
PS	84	F	2	1	25	3.9	136	1.29	0.61	38.26	1.8	high	10.7	5.2	0.05	36	8.8
PS	43	F	24	1	22.8	4.2	130	1.5	1.18	100.7	1.8	high		6	0.08		10
PS	65	M	21	1	17.7	4.1	96	1.39	0.89	105.1	1.9	high	9.3	7.5	1.1		11.1
PS	49	M	20	1	22.6	3.8	246	1.29	0.76	125.5	1.5	high	8.9	5.6	0.51	29	11.3
PS	63	M	19	1	18.9	4	159	1.1	0.76	82.22	2.1	high	9	7.6	0.07	36	11.5
PS	42	M	19	1	23.5	4.7	139	1.08	0.94	134.4	2.1	high	8.2	5	0.1	30	10
PS	67	M	16	1	20.7	4.3	184	2.13	1.07	83.23	1.6	high	9.4	9	0.18	32	10.7
PS	39	M	16	1	19.5	4.4	167	1.41	1.01	119.8	1.5	high	8.8	7	0.02	24	11.3
PS	40	M	16	1	21.2	4	207	1.79	0.91	116.9	1.1	high	9.1	7.1	0	22	9.3
PS	49	M	16	1	19.5	4.2	184	1.51	1.03	109.4	1.6	high		4.8	0.04	23	11.2
PS	60	F	15	1	28	3.9	94	1.15	1.21	129.6	1.6	high	7.5	7.6	0.1	27	11.6
PS	45	M	15	1	18.7	4.1	116	1.32	0.81	114	2.1	high	8.7	4.9		17	10.5
PS	38	M	20	1	18	4.3	141	1.45	0.83	93.67	1.9	high	9.2	5.3	0.02	18	10.5
PS	39	M	13	1	21.7	4.1	182	1.46	1.27	121.7	1.6	high	9.4	4.7	0.41	28	12.1
PS	45	M	13	1	19.8	4	200	1.42	0.91	128.1	2.1	high	8.8	10.9	0.88	26	10.9
PS	59	M	12	1	17.6	4	116	1.3	0.97	130.8	1.8	high	7.9	5.6	0.2	28	10.3
PS	53	F	20	1	29	3.5	139	1.27	0.77	138.8	1.5	high	9.3	6	0.3	22	13.4
PS	66	M	10	1	18.5	3.4	125	1.59	0.93	106.7	1.9	high	9.2	8.2	0.59	32	10.7
PS	42	M	7	1	16.2	3.3	123	1.15	0.99	86.46	2.1	high	9.5	3.7	0.36	25	10.8
PS	52	M	6	1	23.8	4.3	196	1.21	0.93	80.28	1.1	high	8.1	4	0.3	30	9.4
PS	20	F	3	1	18.4	3.1	169	1.49	0.94	105.6	1.3	high	8.2	7.1	0.05	29	11.6
PS	82	F	17	1	15.1	3	178	1.44	0.78	77.59	1.6	high	9.6	4.6	0.01	16	11
PS	76	M	16	1	19.6	3.4	241	1.94	0.81	100.1	1.6	high	8.6	5.5	0.02	19	10.6
PS	75	F	7	1	19	3.9	145	1.24	0.83	123.8	2.5	high	9	5.5	0.2	28	10.7
PS	72	M	5	1	22.9	3.4	167	1.51	0.8	106.1	2.1	high	8.9	5.1	0.1	24	10
PS	73	F	5	1	22.6	3.1	111	1.66	0.95	75.36	1.8	high	8.3	3.9		30	10.5
PS	80	F	3	1	21.3	3.9	144	1.73	0.98	88.26	1.3	high	8.1	5.6		34	13
PS	55	M	3	1	17.7	4.3	144	1.74	0.95	94.92	1.8	high	9	6.3	3.4	33	10.5
PS	84	F	6	1	19.4	3.4	134	1.26	1.28	110.5	2.1	high		6.9	1.08	24	9.3
PS	78	M	2	1	20.3	3.5	148	1.5	0.84	106.9	1.6	high	10.1	7.5	0.1	30	11.4
PS	57	F	22	1	21.5	3.4	136	1.08	1.14	99.7	2.1	high	9.4	6.3		34	11
PS	49	F	2	1	26	4	186	1.5	0.8	56.94	1.5	high		4.8	0.69	19	10.3
PS	68	F	21	1	25.6	3.2	163	1.43	0.98	97.92	1.3	high		7	0.06	21	10.6
PS	67	M	9	1	21.8	3.2	245	1.58	1.06	110.8	1.3	high		9.3	0.94	38	10
PS	60	M	4	1	18.5	3.9	140	1.27	1.01	138.4	1.9	high	9	9	0.02	24	10.7
PS	73	M	18	1	20.7	4	169	1.49	0.91	129.2	1.9	high	9.5	7.7	0.63		9.8
PS	71	M	9	1	23.4	3.3	156	1.32	0.94	96.54	1.8	high	8.2	7.7	0.76	24	8.3
PS	75	M	9	1	21.7	3.9	129	1.62	0.7	88.77	1.3	high	8.4	6.9	0.8	24	10.8
PS	64	M	19	1	21.8	3.9	143	1.44	0.91	100.7	1.9	high	9.2	6	0	36	11.9
PS	44	M	7	1	22.3	3.7		0.97	0.75	90.41	2.1	high		0.5	0.09	49	9.4
PS	71	F	4	1	16	3.8	158	1.71	0.93	88.79	1.3	high	8.8	5.8	0.14	29	9.7
PS	88	F	2	1	20.3		165	0.99	0.89	129.4	2.1	high	8.4	5.2	0.02	32	11.4
PS	67	M	27	1	19.3	3.4	161	1.6	0.93	126.4	1.3	high	8.3	5.3	0.06	24	9.6
PS	41	M	7	1	29.7	4.7	186	1.34	0.76	121.6	1.5	high		6.6	0.1	29	10.1
PS	56	F	2	1	19.1	3.5	135	1.4	1.04	156.8	1.9	high	8.3	4.1	0.1	36	10.3

PS	52	F	2	1	19	3.7	155		0.86	92.23	1.5	high		3		23	9.1
PS	41	M	4	1	27.4	3.8	106	1.49	0.75	161.2	1.5	high	11.6	4.4	0.05	25	9.8
PS	61	M	4	1	21.3	4.3	144	1.04	1.07	104.8	2.1	high	9.5	4.5		24	10.8
PS	67	F	3	1	23.8	3.4	125	1.56	0.8	89.87	2.1	high	9.3	4.5	0.05		10.9
PS	58	M	3	1	28.1	3.9	134	1.18	0.74	94.43	1.3	high	8.7	4.7	0.32	16	11.4
PS	69	F	4	1	22.8	3.6	122	1.61	0.82	80.72	2.1	high	8.7	5.7	0.04	32	12.2
PS	78	F	7	1	20	3.2	134	1.23	1.07	109.9	1.5	high	9	5.5		22	8
PS	74	M	4	1	31.4	3.9	159	1.22	1.03	113.3	1.1	high	9.7	5.3	0.02	24	8.9
PS	59	M	33	1	17.4	3.7	175	2.21	1.02	73.36	1.4	high	7.8	6.1	0.09		10.1
PS	72	M	12	1	20.1	3.8	124	1.26	0.78	73.63	2	high	8	4.1		25	10
PS	47	F	11	1	19.9	4.2	111	1.16	0.72	90.15	2.1	high	8.2	5.1	0.29	27	9.7
PS	55	F	3	1	34.8	3.8	114	1.18	0.58	48.91	1.6	high	8.8	5.6	0.1	27	10.9
PS	64	F	26	1	16.8	3.6	192	1.22	0.83	95.96	1.2	high	8.2	4	0	16	11.1
PS	44	F	15	1	19.8	3.7	194	1.65	0.83	56.79	1.1	high	8.5	3.8	0	25	10.4
PS	60	F	15	1	18.3	4.2	98	1.43	0.91	85.89	1.6	high	8.7	6	0.1	35	13.2
PS	68	F	15	1	18	3.6	185	1.18	0.58	78.68	2.1	high	8.3	4.6	11.13	20	12.2
PS	63	F	12	1	18.3	4	144	1.05	0.74	88.96	2.1	high	8.8	6.3	0.1	20	11.7
PS	57	M	12	1	19.9	4	138	1.48	1.29	91.39	1.5	high	9.1	4.9	0.18	37	10
PS	45	M	11	1	23.5	3.9	146	1.63	0.64	118	1.6	high	8.4	3.4	1.1	31	10.6
PS	50	F	11	1	22.5	4.2	113	1.67	1.09	96.06	1.3	high	7.9	4.9	0	25	11.7
PS	59	M	10	1	18.4	3.8	167	1.21	0.64	86.89	1.6	high	9.9	5.8	0.4	30	10.6
PS	66	M	10	1	21.3	3.8	155	1.33	0.59	85.41	1.5	high	8.7	6.8	0.06	39	9.3
PS	47	F	10	1	17.6	3.9	138	1.32	0.86	84.31	2.1	high	8.1	5.9		23	11.4
PS	77	F	10	1	24.9	3.9	155	1.43	1.01	83.21	1.6	high		6.1	0.29	27	9
PS	55	M	9	1	18.4	4.2	147	2	1.23	106.8	1.3	high	8.9	5.8		31	10.7
PS	50	M	9	1	19.5	4.1	140	1.82	0.97	114.4	1.5	high	9.8	6.3	0.07	22	9
PS	74	F	9	1	19.4	4	154	1.38	1.01	98.52	1.3	high	8.7	5.5	0.05	29	11.3
PS	67	M	8	1	24.3	3.8		1.06	0.6	108.8	1.8	high	9.4	3.3	1.37	25	11
PS	29	F	7	1	18.2	4.1	174	1.35	1.11	75.94	1.8	high	8.8	5.4	1.52	49	10
PS	53	M	7	1	24.5	4.6	98	1.5	0.65	93.18	1.8	high	10.2	4.3	2.86	25	9.8
PS	51	F	7	1	22.1	4.1	230	1.09	1	99.25	1.5	high	10.1	4.5	0.01	19	12
PS	54	M	6	1	27.3	4.4		1.95	0.84	84.32	2.1	high	9.1	3.6	0.9	30	11.6
PS	64	F	7	1	19.4	3.8	95	1.17	0.76	97.21	1.5	high	9.7	6.8		38	9.7
PS	33	M	6	1	15.1	4.6	142	1.25	0.71	100.5	2.1	high	9.6	3.3		33	11.1
PS	64	M	5	1	17.7	4.3		1.54	0.94	91.46	1.5	high	9.4	4.9	1.84	26	9.7
PS	66	F	5	1	18.1	3.9	204	1.49	0.81	91.38	1.3	high	9.8	6.2	0.05	23	10.7
PS	59	F	5	1	22.8	3.9	156	1.3	0.83	96.07	2.1	high	9.2	5.3	0.18		10
PS	54	F	4	1	25.7	4	204	1.45	0.87	82.21	1.5	high	10.9	3.2	0.6		10.2
PS	72	M	4	1	20.9	4.2	159	1.94	1.07	102.6	1.5	high	9.7	6	0.2	29	9.9
PS	63	M	4	1	27.7	6.7	87	1.51	0.77	100.1	1.5	high	8.3	4.7	4.48	31	9.3
PS	63	F	4	1	21.8	3.7		1.32	0.7	79.42	2.1	high	10	4.9	1.81	26	9
PS	55	F	3	1	26.4	4	108	1.15	0.84	111.5	2.1	high	10.3	3.4	0.11	36	9.9
PS	67	M	3	1	21.5	4.3	212	1.37	0.72	87.19	1.3	high	8.7	8.4	0.07	24	10.7
PS	56	M	3	1	22.9	3.6		1.39	0.6	93.68	1.8	high	8.2	4.3	0.27	34	10.4
PS	60	M	3	1	23.8	4.2	152	1.26	0.63	117.2	2.1	high	9.5	5.3	0.98	0	9.6
PS	57	F	2	1	19.6	3.9	183	1.65	0.75	60.2	1.6	high	11	4.5		25	9.8
PS	61	F	2	1	20.9	4	134	1.45	0.84	88.48	1.6	high	8.4	8	0.02	27	10.9
PS	57	M	2	1	18.5	3.5	147	1.26	0.85	106.2	2.1	high	8.5	4.5		33	10.2
PS	79	M	2	1	17.6	3.8	164	1.32	0.85	103.2	1.6	high	10.6	7.8	0.04	20	11.2
PS	45	F	2	1	16	4.1	176	1.39	0.72	98.97	1	high	9.4	5.4	0.12	29	9.3
PS	59	F	12	1	19.4	4	182	1.38	0.82	113.3	1.5	high	8.4	3.9	0	22	10.5
PS	55	M	8	1	23.2	2.7	192	1.62	0.58	91.24	1.3	high	10.5	6.9	0.05		9.8
PS	53	M	31	1	21	3.8	209	1.3	1.19	81.16	1.6	high	9.3	5.7	0.46	24	10.1
PS	54	F	29	1	18.1	3.6	136	1.39	0.99	95.84	1.5	high		5.7	0.05		11.6
PS	77	F	17	1	21	3.7	163	1.23	0.76	98.92	1.9	high	8.7	6.3	0.11	21	9.3
PS	65	M	10	1	19.6	4	115	1.3	0.68	104.5	2.1	high		7.1			9.2
PS	67	F	10	1	19.9	3.9	148	1.84	0.83	93.52	1.3	high	9	3.7	0	26	12
PS	73	M	7	1	26.5	3.8	158	1.32	0.93	113.2	1.3	high	8.8	8.1	0.53		
PS	69	M	6	1	21.3	4.4	147	1.17	1.23	111.2	1.5	high		3.9	0.17	24	11.6

PS	65	M	6	1	19.3	4	159	1.77	0.9	77.1	1.8	high	8.9	6.9	0.15	18	10.3
PS	76	M	5	1	18.7	3.8	119	1.53	0.79	113.3	1.8	high	7.4	4.8	0.8	29	10.4
PS	79	F	5	1	22.3	3.6		0.98	0.79	102.1	2.1	high	9.1	8.1			11.4
PS	71	F	5	1	24.9	3.6	139	1.05	0.74	84.58	2.1	high	9	6	0.19	32	14.2
PS	57	M	4	1	20.5	4.2	179	1.59	0.92	99.57	2.1	high	9.1	5.3	0.05		12.2
PS	65	F	4	1	20.2	4.1	144	1.2	0.81	110.5	1.6	high	8.5	2	0.2	25	11.6
PS	82	M	4	1	18.3	3.5	123	1.37	0.9	93.56	1.1	high	9.4	3.8	0.18	36	10.3
PS	75	F	3	1	21.4	3.7	90	1.32	0.87	104.8	1.5	high	9.3	4.7	0.06	27	9.2
PS	66	F	3	1	21.6	3.9	171	1.3	0.79	108.4	1.6	high	10.4	4.8		29	10.3
PS	49	F	3	1	21.3	4	118	1.03	0.75	78.09	1.6	high	8.2	2.4	0.34	33	9.4
PS	51	F	3	1	19.1	4	166	1.1	0.73	87.99	1.6	high	8.4	3.3	0.15	33	9.4
PS	81	M	3	1	21.4	3.7	97	0.96	0.97	125.6	1.5	high		5.4	9.57	29	9.6
PS	54	F	2	1	26.1	3.7	176	1.76	0.96	65.09	1.9	high	9.5	3.4	0.05	34	10
PS	59	F	2	1	24.8	3.8	234	1.62	0.67	76.9	2.1	high	11.6	2.2	0.06	23	9.8
PS	74	F	2	1	30.2	3.9	86	1.53	0.8	90.44	1.8	high	8.7	4.8			11.8
PS	59	F	9	1	19.7	4.3	122	1.12	1.03	116.1	2	high		5.8	0.06	28	9.9
PS	63	F	33	1	15.7	3.8	194	1.5	0.75	93.34	1.6	high	8.8	5.6		33	11.1
PS	59	F	12	1	23.9	3.7	139	1.53	0.79	105.6	1.8	high	9.5	4.9	1.14	37	12.5
PS	70	M	10	1	17.8	3.8	135	1.44	1.04	92.15	1.9	high	10.3	5.6	0.29	26	10.3
PS	65	F	10	1	23.1	3.7	141	1.2	0.9	73.91	2.1	high	8.7	3.4	0.39	32	11.7
PS	42	M	10	1	21.8	3.8	157	1.19	0.78	93.03	1.5	high	7.8	5.3		25	10
PS	72	M	9	1	23.1	3.6		1.27	1.06	112.8	2.1	high	8.6	4.8	0.36	21	10.1
PS	67	M	8	1	20.8	3.6	151	1.5	0.62	93.47	1.8	high		6.4	0.05	34	12.4
PS	66	M	8	1	21.5	4.3		1.75	0.81	119.4	1.5	high	8.4	3.9	0.24		10.6
PS	58	F	8	1	19.1	3.7		1.24	0.94	93.24	1.8	high	10.1	3	0.43	36	12.3
PS	84	F	7	1	22.6	3.8	136	1.25	0.99	92.9	1.5	high	8.6	5.1	0.16	29	7.7
PS	63	F	7	1	17.9	3.5	140	2.01	0.89	95.11	2.1	high	8.2	3.5	0.01	26	9.4
PS	54	M	6	1	24.3	3.7	134	1.16	0.95	120.5	2.1	high	9.2	4.3	0.05	22	11.3
PS	56	M	6	1	20.7	3.6	220	1.54	0.94	81.41	1.8	high		4.1	9.5	17	8.8
PS	60	M	6	1	23.9	4	190	1.49	0.88	132.5	1.9	high	8.5	4.7	0.07	25	9.4
PS	75	M	5	1	24.5	3.5	206	2.25	0.83	95.41	1.3	high	9.3	3.9		31	10.9
PS	55	F	5	1	28.3	3.7	114	1.16	0.98	107.4	1.6	high		6.2			11.7
PS	65	M	5	1	22.6	4	181	1.58	0.75	96.63	1.5	high	10.1	4.3	0.6	29	10.8
PS	66	F	4	1	23.2	3.7	145	1.72	0.74	122.2	1.5	high		4.6	2.2	21	9.7
PS	61	F	4	1	19.5	3.7	146	1.57	1.1	86.73	1.4	high	8.9	4.5	1.66	24	9.7
PS	73	M	3	1	17.9	3.7	184	1.8	0.84	97.23	1.8	high		2.6	0.07	28	13.4
PS	71	F	2	1	29.7	3.7	177	1.71	0.51	85.38	1.6	high		4.5			9.3
PS	60	F	2	1	17.8	3.9	142	1.37	0.92	85.97	2.1	high		6.8	2.49	32	12.4
PS	60	M	2	1	22.1	4	177	1.54	0.87	99.19	0.8	high	9.5	6.3	0.35	32	14.2
PS	62	M	2	1	22.4	4.1	145	1.26	0.79	78.67	2.1	high		6.7	0.63	26	10.4
PS	90	F	2	1	24.2	3.8	194	1.34	0.74	147.5	1.8	high	8.4	4			10.6
PS	63	M	2	1	19.9	4	158	1.35	0.86	82.72	2.1	high	8.8	5	0.17		11
PS	75	M	2	1	22.4	4.1		1.24	1.2	126.4	2.1	high	7.9	6.7	0.8	31	8.5
PS	73	M	25	1	21.4	3.8		1.3	0.81	144.4	1.6	high	7.8	4.2			
PS	49	F	24	1	17.4	4.3	197	1.37	0.85	113.6	1.9	high	9.2	2.9	1.16	29	10.3
PS	75	M	22	1	18.7	3.6	169	1.7	0.74	111.2	1.6	high		7	0.07	28	10
PS	69	F	18	1	25.8	4.2	155	1.87	1.04	125.8	1.8	high	9.3	5	1.06	37	11.2
PS	67	M	14	1	22.2	4.4	136	1.88	1.01	142.7	1.5	high	8.1	4	0	40	10.9
PS	44	M	14	1	20.8	2.6	162	1.43	0.59	112.9	1.6	high		7	0.03	27	10.3
PS	67	M	10	1	17.1	4.1	152	1.35	0.84	141.5	2.1	high	8.9	8		27	10.8
PS	59	M	4	1	30	4.1	111	1.19	1	159.8	1.5	high	9.1	4.6		24	10.1
PS	71	F	4	1	24.7	3.7	109	1.36	0.62	88.01	1.5	high	8.3	4.7	4.67	27	10.1
PS	66	M	3	1	21	3.9	183	1.61	0.98	135.1	2.1	high	10.2	4.3	0.02		10.5
PS	61	M	3	1	23.8	5.4	156	1.35	0.72	107.5	1.5	high	9.8	7.3	1.2	38	14.3
PS	79	M	2	1	17.1	4.2	183	1.5	0.64	121.9	1.3	high	11	4.5		29	10.9
PS	61	M	16	1	17.5	3.4	116	1.37	0.55	80.12	2.1	high	10.1	7.9	0	32	11.1
PS	81	M	4	1	17	3.7	169	1.5	0.79	97.37	1.5	high	9.3	6.3		17	11.2
PS	37	F	9	1	18.7	4.6	144	1.23	0.87	118.8	1.5	high	9.7	5		20	10.7
PS	71	M	3	1	22.2	3.9	205	1.7	0.89	85.85	1.1	high	8.4	2.9		15	9.3

PS	39	M	12	1	19.3	4.6	99	0.97	0.85	71.18	1.3	high		6.3	4.37		7.2
PS	81	M	6	1	26.1	3.5	122	1.55	1.01	136	1.5	high	10.5	5.3		20	12.1
PS	60	M	3	1	21.4	4.3	147	1.28	0.82	115	1.8	high		5.7	0.05	20	11
PS	63	M	34	1	20.9	3.7	150	1.77	0.85	76.17	1.3	high	8.8	5.9	0.08	28	10.5
PS	47	M	25	1	18.1	3.4	170	1.67	1.05	65.04	2	high	8.9	5	0.32	27	10.5
PS	58	M	23	1	19.7	3.6	113	1.26	1.01	87.82	1.6	high	8.9	7.1	0.69	44	10
PS	59	F	21	1	19.1	3.7	188	1.53	1.38	132.7	1.6	high		5	0.09	18	9.9
PS	72	F	21	1	18.8	3.5	152	1.27	0.71	86.75	1	high	9	4.7	0.02	26	9.2
PS	73	F	21	1	22	3.6	173	1.8	1.02	89.67	1.5	high	9.7	4.7	0.05	22	10.6
PS	64	M	20	1	21.3	3.7	137	1.45	0.98	109.2	1.5	high	10.7	5	0.36	28	9.9
PS	68	M	18	1	18.7	3.8	177	1.33	0.9	115.5	1.6	high	9.8	2.4			9.6
PS	66	M	18	1	20.4	3.7	211	1.73	0.89	117.8	1.6	high	9	4.9	0.07	24	9.9
PS	61	F	17	1	17.9	3.8	141	0.99	1.04	149.6	2.1	high		7.6	6.09	28	10.4
PS	58	F	16	1	17.8	3.8	217	1.54	1.02	115.2	1.6	high	9	4.4	0.15	25	9.9
PS	78	F	15	1	23.2	3.9	175	1.51	0.96	139	1.3	high	9	5.4	0.05	25	9.9
PS	29	F	14	1	17.1	3.7	159	1.32	0.81	107.8	2.1	high	9.8	5.6	1.21	27	10
PS	43	M	14	1	19.4	3.8	162	1.82	1.27	101.9	1.3	high	9.4	6.8	3.41	29	9.3
PS	43	F	13	1	18.9	3.5		1.1	1.05	140.7	1.8	high	9	3.9	4.9		10
PS	67	M	13	1	18.2	3.4	140	1.55	0.76	71.52	1.5	high	9	5.1	0.08	29	10.7
PS	70	F	13	1	23	3.9	144	1.54	0.91	105.5	2.1	high	8.5	5.8	0.1	23	10.4
PS	69	M	13	1	20.9	3.6	147	0.96	0.96	146.4	1.3	high		3.6	0.39	26	11.6
PS	61	F	12	1	23.1	4	98	1.54	0.9	124.9	1.8	high	9.1	5.4	0.05	30	10
PS	76	M	12	1	24.1	3.4	137	1.34	0.88	86.58	1.3	high	8.4	5	0.07	27	9.3
PS	60	F	12	1	25.1	3.7	118	0.92	1.11	130.5	2.1	high	9.7	7.2	0.1		9.3
PS	70	M	11	1	23.5	3.6	166	1.07	0.91	131.1	1.5	high		6.5	0.25	30	10.9
PS	63	F	11	1	23	3.4	117	0.91	1.15	113.8	1.3	high		5.6	0.11	27	12.8
PS	41	M	11	1	21.8	3.9	175	1.47	0.48	120	1.5	high	8.5	6	0.06	21	9.6
PS	56	M	10	1	21.5	3.5	100	1.16	0.88	124.7	2.1	high		5.9	0.05	24	12
PS	58	F	9	1	42.2	3.9	184	0.88	1.2	112.9	2.1	high		5	0.11	13	11.9
PS	65	F	9	1	22.8	3.5		2.3	0.85	115.3	1.5	high	8.7	4.3	0.08		10.6
PS	74	F	9	1	17.3	3.9	185	1.55	0.94	115.6	1.5	high	8.7	4.5	0.08	25	10.6
PS	71	F	9	1	18.6	3.6		1.34	1.01	126.5	2	high		6.9			9.1
PS	61	F	8	1	23.9	3.9	199	0.91	0.84	147.4	1.5	high	8	4.4	0.03	15	8.6
PS	80	M	8	1	19.3	3.6	211	1.32	0.97	108.1	2.1	high	8.8	5.4	0.53	17	11
PS	79	M	7	1	20.3	3.4		1.35	0.72	113.8	2.1	high	8.3	3.3			8.8
PS	60	M	7	1	19.5	3.7	163	1.57	1.11	102.9	1.5	high	9.2	4.1	0.04	23	11.9
PS	51	M	6	1	23.9	3.5		1.64	1.31	103.9	1.3	high	10.7	6.9			11.2
PS	52	M	6	1	28	4.1	190	1.75	0.97	109.8	1.3	high		5.2	0.1	20	10.4
PS	78	M	6	1	18.6	3.6	163	1.9	0.94	80.6	2.1	high	9.3	5.5	0.2	20	10.6
PS	80	M	6	1	18.9	3.4		1.64	0.83	107.3	1.8	high	8.9	5.1			9.3
PS	64	F	6	1	18.9	3.2	123	1.68	0.73	61.61	1.6	high	10	7	0.06	10	9.6
PS	76	F	5	1	28.6	3.7	115	1.47	1.14	160.7	2.1	high	9.4	3.4	0.79		10.3
PS	78	F	5	1	18.8	3.5	213	2.07	0.73	91.38	1.3	high	8.8	3	0.07	23	10
PS	49	F	5	1	19.9	4.1	109	1.46	1.1	123	2.1	high	9.5	4.7	0.1		9.5
PS	62	M	5	1	24.9	4.1	112	1.84	1.1	137.6	1	high	8.4	5.3	0.41	34	8.6
PS	78	F	5	1	23.6	3.5		1.22	0.83	138.6	1.5	high	9.3	6.2	0.1	13	9.3
PS	73	F	5	1	20.4	3.5	190	1.14	0.78	141.9	2.5	high	8.3	3.6		30	11.3
PS	78	F	4	1	30.9	3.3		1.54	0.67	118.3	1.8	high	8.9	1.9			9.4
PS	71	F	4	1	22.5	3.9		1.49	1.05	109.7	1.8	high	8.4	6.1			12.7
PS	65	F	4	1	22	4	161	2.45	0.9	121.8	1.5	high	9	3.5		26	9.4
PS	69	F	4	1	20.9	3.8	86	0.94	0.87	110.2	2	high	8.7	5.6	0.1	24	9.8
PS	78	F	4	1	18.6	3.8	165	1.6	0.81	141.7	1.6	high	9.3	5.3	0.05	27	9.9
PS	63	F	4	1	21.6		159	1.65	0.87	135	1.6	high	10.2	6.3	0.27	27	10.6
PS	59	M	4	1	22	3.9	120	1.32	0.71	120.4	1.6	high	10.1	7.2	0.06	8	9
PS	78	M	4	1	19.4	3.5		1.64	0.94	121.4	2.1	high	8.5	4.2			9.8
PS	70	F	3	1	22.5	3.5		1.52	0.85	96.37	1.5	high	7.7	5.8			9.3
PS	80	F	3	1	18.3	3.8	139	1.32	0.99	135.4	1.6	high	9.6	2.5		25	11.8
PS	42	M	3	1	23	3.6		1.76	0.89	134.2	1.3	high	8.1	5			10.7
PS	71	F	3	1	20.8	3.8	146	1.81	1.12	122.8	1.5	high	9.3	3.2	0.02	31	10.6

PS	64	M	2	1	21.7	3.7	169	1.35	0.82	98.87	1.3	high	9.8	5.2	0.06	23	11.6
PS	69	M	2	1	20.5	3.8	149	1.48	0.66	105.3	1.3	high	8.5	6.3	6.37	28	8.1
PS	71	M	2	1	25.4	3.8	109	1.36	0.68	77.09	1.5	high	8.4	3.6	0.18	35	10.2
PS	69	M	2	1	26.1	3.9	130	1.83	1.07	127.4	1.6	high	8.7	2.3	0.02	26	8.8
PS	73	F	2	1	16.1	3.5	180	1.73	1.03	62.55	1.8	high	9	5.3	0.2	31	9.6
PS	61	M	2	1	25.5	3.8		1.58	0.73	62.67	1.6	high	7.6	4.1	1.03	26	9.3
PS	66	M	2	1	20	3.5	174	1.18	0.87	106.3	1.8	high	9	3.9	0.64	42	10.7
PS	55	M	10	1	16.7	3.5	117	1.21	0.83	106	1.4	high		6.4		36	11.5
PS	61	M	3	1	18.6	4.2		1.29	0.99	103.3	1.8	high	9.7	6.5		27	
PS	69	F	9	1	19.6		142	1.26	0.91	98.35	2.5	high	8	4.8	0.16		11.2
PS	56	M	17	1	19.6	3.9	139	1.55	1	114.6	1.5	high	8.1	6.4	0.2	24	10.1
PS	66	M	2	1	20.4	3.8		1.74	1.2	131.1	1.5	high	9.9	5.2	0.02	25	10.8
PS	69	M	2	1	21.2	3.9	147	1.62	0.6	26.92	1.8	high	8.8	6.5	0.05	30	11.9
PS	62	M	8	1	24.6	3.7	138	1.75	0.94	104.5	1.6	high	9.2	6	0.05	27	10.9
PS	70	F	17	1	23.4	3		1.32	0.78	112.1	2.1	high		5.8			9.3
PS	77	F	7	1	24.8	4	156	1.67	0.93	106.8	1.5	high		4.4	0.09	29	11.1
PS	57	M	12	1	17.5	3.6	129	1.19	0.85	51.95	1.8	high	8.6	3.8	0	29	10.2
PS	61	M	9	1	18.6	3.8	213	1.6	1.15	87.44	2.1	high	11.3	6.2	0	20	10.5
PS	35	F	8	1	20.2	4.2		1.34	1.01	114.5	2.1	high	7.6	6.5			10.5
PS	44	M	7	1	18.8	4		1.17	0.92	101.1	1.5	high	9.7	5.5			10
PS	43	F	7	1	16.1	4.1	134	1.31	0.92	116.7	1.5	high	11.5	3.7		22	10.1
PS	45	M	2	1	26.7	4.1	135	1.5	0.81	127	1.8	high	9.6	5.9	0.3	28	9.8
PS	51	M	8	1	22.2	4.3	173	1.61	0.9	113	1.6	high	9.1	4.9	0.05	24	10.2
PS	52	M	17	1	20.8	4.2		1.34	1.08	112.7	1.3	high	9.4	9.6	0.74	27	11.2
PS	66	M	9	1	21.9	3.5	104	1.05	1.05	116.4	1.9	high	8.7	6.2		26	10.2
PS	69	F	9	1	23.5	3.7		0.98	0.86	103.2	1.5	high	8.8	4.1		25	8.7
PS	72	M	7	1	22.4	3.8	118	1.25	0.92	104.5	1.5	high	8.8	7.6	0.1		13.5
PS	64	M	6	1	17.8	4	203	1.53	0.84	80.65	1.8	high		6.3		27	8.6
PS	68	F	5	1	23.1	4.1	119	1.02	0.81	135.4	2.1	high	9.7	6.6	0.58	29	11
PS	64	M	4	1	23.7	3.8	148	1.72	0.77	95.5	1.8	high	9.3	4.6		26	10.7
PS	44	M	4	1	23.4	4.3	122	1.64	0.58	91.28	1.3	high		5	0.01	30	9.8
PS	74	M	4	1	18.4	4.2	154	1.12	0.93	71.88	1.5	high		5.5	1.56	29	9.4
PS	50	F	4	1	20.7	4.1	103	1.33	0.98	122.6	2.1	high	9.3	5.9	0.53	23	11.5
PS	50	F	3	1	23.1	4.1	165	1.83	0.88	83.48	1.6	high	8	5.4	0	26	9.7
PS	63	M	2	1	22.3	3.7	195	1.53	0.88	85.57	1.5	high	8.7	7.6		20	8.7
PS	55	M	2	1	28.1	3.8	162	1.49	0.79	83.63	1.5	high	8.4	3.3		22	9.8
PS	76	M	9	1	24.7	4	150	1.29	0.74	120.5	1.1	high		7.4		30	11.6
PS	34	M	5	1	22.1	4		1.37	0.75	114.4	1.6	high	9.2	5.5	0.1	49	10.8
PS	52	M	6	1	22.8	4	182	0.93	0.91	101.4	1.3	high	9.2	5.9	0.01	21	10.9
PS	45	F	2	1	17.9	3.7	146	1.14	1.17	88.54	1.4	high	9.3	5		21	
PS	72	F	12	1	22	3.7	148	1.57	0.89	133.1	1.5	high		5.3	0.48	26	12.7
PS	76	F	12	1	17.9	3.6	178	1.42	0.96	76.75	1	high	9.4	6.7		36	10.9
PS	72	F	11	1	15.8	3.6	103	1.54	1.04	101.5	1.6	high	8.1	3.2		38	9.3
PS	64	M	11	1	20.2	3.3	140	1.16	0.79	98.91	1.8	high		4.7	0.11	31	10.4
PS	72	M	10	1	21.9	3.6	149	1.49	0.93	138.5	1.6	high	9.7	7.7	0.3	37	10.2
PS	42	M	10	1	20.8	3.5	184	1.29	0.61	87.58	1.3	high	8.5	5.8	0.1	21	9.8
PS	47	M	8	1	22.1	4	184	1.24	1.07	118.4	1.5	high		4.6	0.32		11.4
PS	62	M	7	1	21.5	3.9	171	1.54	0.99	105.8	2.1	high	10	4.9	0.05		10
PS	65	M	7	1	23.8	3.6	89	1.22	0.7	93.07	1.6	high	10.2	5.7	0.03	29	9.3
PS	86	F	6	1	17.7	3.2	198	1.33	0.8	104.3	1.3	high	8.7	3.4	0.04	21	8.1
PS	64	F	5	1	18.8	3.6	127		0.88	92.45	1.3	high	10.6	4.6	0.67	32	10.2
PS	48	M	4	1	23.7	4	161	1.07	0.94	100	1.6	high	9.8	5.8	0.11	29	9.4
PS	71	F	4	1	18.2	3.2	131	1.1	0.97	80.54	1.6	high	7.6	5.7	1.34	27	9.5
PS	70	M	4	1	23.4	3.8	147	1.82	1.15	118.1	1.5	high	9.9	6.1	0.06	30	10.2
PS	75	M	3	1	18.9	3.3	160	1.21	0.92	112.1	1.6	high	9.2	7.3		28	10.3
PS	64	F	3	1	24.9	3.3	155	0.98	0.83	89.46	1.5	high	8.6	4.3	0	28	10.5
PS	65	M	2	1	20.9	3.6	96	1.38	0.91	116.3	1.6	high	8.9	6.9		32	10.5
PS	71	M	2	1	20.2	3.8	132	1.09	0.81	88.04	2.1	high	9.3	7.4	0.11	21	9.9
PS	69	M	2	1	17.9	3.7	195	1.06	0.96	152.6	2.1	high		4.5			10.7

PS	76	M	12	1	21		183	1.16	0.61	85.09	1.5	high	7.9	3.4	0.16	19	10
PS	46	M	5	1	18.1	3.7	139	1.33	1.16	101.8	1.8	high		7.6	0.5	50	11.3
PS	65	F	3	1	19.8	4	141	1.27	0.93	77.18	1.5	high		5.1	3.9	24	9.7
PS	70	M	13	1	21.7	3.7	229	1.55	0.72	86.19	2.1	high	8.5	5	0.23	32	9.8
PS	62	F	16	1	23.3	3.9	173	1.42	1.18		1.9	high	9.8	5.6	0.05		11
PS	45	M	17	1	22.7	4	172	1.45	1.07		1.3	high	8.8	5.1	0		9.7
PS	39	M	3	1	34.7	4	127	1.18	0.65	115.4	1.5	high	8.1	5.8	0.3		10.5
PS	59	M	11	1	24.4	4	108	1.24	0.79	81.41	1.3	high	9	8.4	0.2	34	11.7
PS	54	M	6	1	16.9	4.1	139	1.1	0.84	78.14	1.6	high	9	9.9	0.1	43	10.7
PS	59	M	4	1	20.6	3.8	142	1.12	0.77	108.3	1.8	high	9.5	7.5	0.02	28	12.2
PS	78	F	3	1	25.4	4.2	162	1.49	0.89	134.2	1.6	high	9.1	8.7	0.17	33	11.1
PS	59	F	4	1	23	3	193	1.61	0.8	58.75	1.8	high	10.6	6.5	0.03	31	8.2
PS	46	F	5	1	22.9	4	122	1.15	0.83	142.9	1.5	high	8.8	6.7	0.06	27	11.1
PS	56	M	4	1	20.7	4.3	166	1.09	1.15	94.13	1.5	high		6.4	0.31	46	10.8
PS	65	F	3	1	17.5	4.3	139	1.22	1.04	108.8	1.5	high	10.6	5.2	0.09	22	10.1
PS	63	M	6	1	26.6	4.2	182	1.23	1.05	122.9	2.2	high	10.4	8.2	1.07	28	10.5
PS	40	M	8	1	35.2	3.7	165	1.24	1.05	107.8	1.5	high	9	5.3	1.19		
PS	48	M	5	1	24.9	4.2	241	1.75	1.34	101.8	1.5	high	8.9	4.9			9.1
PS	52	M	6	1	21.3	4.1	131	1.18	0.73	121.8	2.1	high	7.8	5.9	0.15	26	11.3
PS	67	F	5	1	23.5	4.1	200	1.27	0.95	93.46	2.1	high	9	5.3	0.1	24	9.8
PS	50	M	11	1	21.4	3.8	189	1.66	1.19	122.1	2.1	high	9.5	5.2	0.09	27	11.3
PS	52	F	4	1	16.6	3.2	128	1.11	0.75	79.65	2.1	high	9.4	2.6	0.04		10.3
PS	56	F	14	1	19.5	3.9	97	1.77	1	70.14	2.1	high	9	8.8	0.03	23	9.8
PS	57	M	7	1	21.4	4.2		1.68	0.73	72.57	1.3	high	8.3	4.7			10.3
PS	21	F	8	1	20.7	3.5	239	1.81	1.42	101.9	1.5	high	9	5	1.04	31	10.7
PS	65	F	28	1	20.1	3	124	1.15	1.11	85.21	1.8	high	8.8	5		38	10.8
PS	61	F	25	1	22.6	2.8	140	0.98	1.12	107.4	1.8	high	8.9	6.1		25	10.8
PS	55	M	23	1	20.6	3.9	169	1.2	0.87	117.1	1.8	high	9	5.8		24	10.9
PS	58	M	20	1	18.9	3.2	155	1.27	1.11	108.5	2.1	high	10.4	6.9		26	10.5
PS	55	F	19	1	20.1	3.7	129	1.26	1	118.7	2.1	high	8.9	6.5			11.8
PS	57	M	18	1	26.2	3.5	189	1.31	0.79	115	1.6	high	9	4.5	0.17	27	9.3
PS	50	M	17	1	21.1	3.4	119	1.28	1	112.1	2.1	high	9.4	7.3	0.2	33	12.3
PS	52	M	16	1	18.8	3.5	161	1.6	0.89	92.5	2.1	high	8.8	5.5	0.5	25	8.3
PS	31	M	16	1	21	4.2	214	1.33	1.22	89.67	2.5	high	9.5	6.9	0.1	23	10.8
PS	70	F	15	1	18.8	3.9	154	1.33	0.85	92.91	2.1	high	9	5.7	0.05	20	11.1
PS	73	M	15	1	17.9	3.2	99	2.17	0.81	61.85	1.8	high	9.3	4.3		26	12.1
PS	64	F	15	1	18.7	3.4	151	1.37	1.11	107.8	1.5	high	8.8	5.6	0.1	28	10.9
PS	46	M	13	1	24	3.6	156	1.37	0.92	104.8	2.1	high	9.2	6.8	0	25	12.1
PS	71	F	13	1	18	3.6	132	1.38	0.84	107.6	2.1	high	9.6	7.2	0.1	30	12.1
PS	55	M	12	1	24.6	3.5	161	2.67	1.1	82.67	1.8	high	9.4	5.2	0	21	10.4
PS	41	F	11	1	14.5	2.4	213	1.47	0.96	86.01	1.5	high	9.3	7.1	0.2	27	11
PS	39	M	11	1	20.1	3.9	172	1.55	0.8	126.1	1.8	high	8.3	5.7	0.1	31	11.7
PS	60	M	11	1	17.8	4	134	1.61	1.3	120.3	1.5	high	8.8	6.3	0.06	32	10.7
PS	83	M	9	1	19.2	3.6	212	1.51	0.74	98.03	1.5	high	9.9	4.4	0	27	9.3
PS	41	M	8	1	17.9	4.1	192	1.26	1.12	125.3	1.8	high	8.4	5.1	0	28	10.4
PS	59	F	7	1	17.7	3.7	148	1.78	0.75	73.74	2.1	high	9.3	3	0.1	28	9.1
PS	30	M	6	1	21.3	4.2	158	1.51	0.95	109.5	1.1	high	9.2	7.9		31	9
PS	74	M	6	1	19.3	3.2	132	1.73	0.83	108.6	1.3	high	8.9	5.8	0.16	24	10.5
PS	81	M	6	1	21.4		157	1.74	0.8	132.2	1.6	high	9.7	5.2		29	10.4
PS	58	M	6	1	22.3	3.4	135	1.78	1.24	108	1.6	high	9.2	5.8		26	9.6
PS	77	F	5	1	19.6	3.2	148	1.09	0.93	72.51	1.5	high	8.8	6	0.16	26	11.7
PS	69	F	5	1	21	2.9	194	1.41	0.95	80.55	1.1	high	8.1	1.9	0.02	21	10.5
PS	56	M	5	1	20.5	3.9	115	1.21	0.99	108.4	2.1	high	9.1	5.1	0	42	8.7
PS	67	M	4	1	21.5	3.5	166	1.18	0.93	111.1	2.1	high	9.9	5	0.2	30	10.1
PS	76	M	4	1	20.1	3.6	170	1.49	0.97	92.72	1.8	high	7.7	2.9	0.57	27	8.4
PS	84	M	4	1	18.5	3.2	177	1.63	1.1	140.9	2.1	high	9	3.9			9.5
PS	58	M	4	1	27.3	3.4	179	1.45	0.66	72.68	1.5	high		3.4		29	9.4
PS	48	M	3	1	23		159	1.27	0.79	91.86	2.1	high		7.6		21	10.1
PS	77	F	3	1	20.9	3.6	162	1.08	0.56	86.09	2.1	high		7.9		23	14.3

PS	69	M	2	1	25	3.9	147	1.17	0.99	94.13	1.6	high	9.1	4.8		39	10.1
PS	67	M	2	1	24.8	4.2	161	0.95	0.71	51.31	1.3	high	9	4.3	0.62	23	9.6
PS	59	F	23	1	15	4.2	215	1.47	0.94	109.7	1.8	high		5.7	0.37		14
PS	60	F	20	1	22.9	3.9		1.51	0.89	116.2	2.1	high		7.4			7.6
PS	36	M	20	1	18.4	4.1	170	1.76	0.91	114	1.8	high	8.4	5.6	0.02	23	11.8
PS	62	F	19	1	15.5	4.3	149	1.66	1.04	116.3	2.1	high	8.8	4.8		26	11.7
PS	49	F	18	1	19.8	4.1	130	1.05	0.94	115.4	2.1	high	7.6	4.7		27	9.9
PS	78	F	16	1	23.5	3.7	252	1.53	0.85	108.2	1.3	high	8.5	5	12.7	30	9.8
PS	68	M	12	1	22.5	3.9	160	1.27	0.88	101.6	1.6	high	8.4	4.1	0	12	11.3
PS	64	F	10	1	15.3	3.7	198	1.67	0.9	64.73	1.3	high		4.6		33	11
PS	45	F	10	1	16.4	4.2		1.68	0.97	115.1	2.5	high	9.6	5.6	0.35		11.7
PS	60	F	9	1	25.1	4	192	1.29	0.97	106.7	1.5	high	8.8	5	0.1	25	10.3
PS	27	M	7	1	19.2	4.2		1.79	0.95	127.3	1.8	high	9.7	5.9	0.7		12
PS	89	M	6	1	21.1	3.1	182	1.14	0.92	120.3	1.1	high		4.2	0.35	13	8.9
PS	58	F	6	1	22.7	3.9	221	1.48	0.84	111.1	1.6	high	9.4	5.2	0.08	30	11.4
PS	47	M	6	1	18.9	4.2	121	2.18	1.06	103.1	2.1	high	8.8	5.8	0.1	25	10.7
PS	73	F	5	1	24	3.6	174	1.74	0.8	139.7	0.8	high	9.8	5.5	0.5	34	9.7
PS	30	M	4	1	16.3	4.6	154	1.02	0.95	64.93	1.8	high	9.1	6.1	0.12	20	8.4
PS	59	M	4	1	18.9	3.4	141	1.42	0.65	81.44	2.1	high	9.8	5.7	0.08		11.4
PS	76	M	3	1	20.1	3.9	254	1.01	0.7	110.8	1.5	high	10.2	5.2	0.8	33	12.4
PS	80	F	3	1	20.5	2.9	122	1.43	0.55	67.65	1.6	high		5.2		16	9.4
PS	77	M	2	1	19.6	3.2	121	0.99	0.87	122.2	1.6	high	9.2	7	0.13	21	11.1
PS	65	F	24	1	23.7	3.5	189	1.81	0.86	115.2	1.3	high	7.6	4.7	0.05	36	11.3
PS	72	F	11	1	16.3	3.6	200	1.8	0.86	119.9	1.6	high		4.7	0.04	28	9
PS	61	M	2	1	22.4	3.8	223	1.12	1.52	63.24	2.1	high	8.1	4.4	0.13	22	10.7
PS	75	F	5	1	18.6	3.9		1.46	1.07	97.02	2.1	high	10.1	4.6		42	10
PS	77	M	27	1	21.6	3.7	154	1.32	0.95	129.4	1.8	high	9.1	6.5	0.41	22	11.6
PS	65	M	7	1	17.8	3.9	155	1.51	0.91	122.9	2.1	high	10	3.9	0.07	28	10.6
PS	77	M	6	1	18.6	3.9	112	1.46	0.89	104.2	1.5	high	8.3	3.7	0.68	28	7.6
PS	69	F	3	1	20.8	3.8	190	0.96	0.78	96.27	1.3	high	9.1	5.4	0.07	17	8.6
PS	45	M	8	1	24.5	4.6		1.72	0.77	101.1	1.5	high	8.8	3.1		28	10.7
PS	34	F	4	1	29.8	4.1	112	1.33	1.08	119.4	2.1	high	7.3	5	0.38	29	9
PS	64	M	16	1	19.5	3.8	161	1.65	0.8	112.8	1	high	8.9	3.6	0.05	33	9
PS	65	M	10	1	21.4	3.6	119	1.41	0.99	116.2	2.1	high	9	6.4	0.1		11.2
PS	77	F	5	1	19.6	4.2	150	1.49	0.8	124.3	1.8	high	8.4	4.2	0.03	32	12
PS	65	F	4	1	28.5	3.6	120	1.41	0.97	81.72	2.1	high	8.4	6.5	0.06		11.5
PS	54	F	3	1	19.2	3.7	144	1.37	1.31	101.1	2.1	high		3.9	0	30	10.1
PS	38	M	6	1	26.6	4.4	126	1.3	0.77	113.7	1.5	high	9.7	4.3	0	23	9.9
PS	78	M	3	1	27.8	4	178	1.57	0.75	60.4	1.8	high	8.1	8.2	0.1	25	12.1
PS	59	F	25	1	17.7	4.2	120	1.47	1.46	100.5	1.6	high	8.5	4.4	0.19	33	9.8
PS	67	M	20	1	16.7	3.9	161	1.98	1.18	97.33	1.8	high	9.3	4.3	0.06		9.8
PS	67	M	18	1	20.2	3.7	135	1.76	0.81	98.37	1.6	high	9.4	5	0.23	33	11.2
PS	39	M	17	1	21.9	3.9	162	1.24	1.2	138.9	2.1	high	8.9	5.2	0.07	19	11.4
PS	55	M	17	1	21.7	4	127	1.46	1.23	119.5	2.1	high	8.6	6.6	0.32	27	9.9
PS	57	F	16	1	20.2	4.2	247	1.68	1.13	142.1	1.6	high		5.6	0.39	38	11.3
PS	64	M	16	1	22.7	3.7		1.59	0.98	151.1	1.5	high		4.7			11
PS	60	F	15	1	17	3.7	140	1.09	1.18	133.2	1.9	high	10.7	7.5	0.26	26	11.5
PS	70	F	13	1	20.2	3.1	98	1.32	0.92	125	2.1	high	9.4	4.9		29	9.6
PS	43	F	13	1	16.9	4.2	145	1.55	1.24	119.1	1.6	high	9.9	4.2	1.02	20	9.8
PS	60	M	12	1	22.6	3.9	172	1.11	1.05	144.4	2	high	9	3.4	0.2	27	10.2
PS	61	M	12	1	23.7	3.8		1.95	0.96	134.6	1.5	high	8.1	3.9	0.1	35	9.4
PS	75	M	12	1	22.8	3.8	162	0.86	1.06	171.5	1.8	high	9.8	4.3		14	11.3
PS	44	M	11	1	21.2	3.6	150	1.18	1.08	113.9	2	high	9.3	6.2	0.1	26	10.3
PS	71	F	11	1	18.7	3.6	133	1.8	1.14	96.87	1.8	high	9.2	6.4	0.2	20	10.1
PS	74	F	11	1	20.6	3.5		1.1	0.74	96.72	2.1	high	8.6	6.3		27	9.3
PS	50	F	11	1	15.6	3.7	190	2.14	0.94	114.5	1.5	high	9	5.3	0.1	27	10.2
PS	49	M	10	1	21.9	3.9	100	1.01	0.78	123	2.1	high	8.9	4.4	0.1	25	10
PS	68	M	10	1	18.7	3.8		1.29	1.15	115.7	2.1	high	9.1	5.3		24	9.3
PS	54	M	10	1	24.2	3.9	210	1.29	1.02	126.3	1.8	high	9.3	4.6		19	12.5

PS	69	F	8	1	18.2	4.2	178	1.57	0.88	148.8	1.6	high	8	4.3	0.08	18	7.1
PS	77	F	8	1	19.6	3.9		1.88	0.99	129.6	1.6	high	8.8	4.9		26	7.4
PS	70	F	7	1	21	4	115	1.93	1.11	106.4	1.5	high	9.9	5.3	0.05	30	10.9
PS	88	M	7	1	20.4	3.5	144	1	1.04	161.5	1.6	high	8.5	5.4		24	8.4
PS	52	M	7	1	19.2	4	140	2.01	0.73	53.44	1.3	high	8.7	3.1	0.1	24	9.2
PS	62	F	5	1	23.9	3.9	150	1.32	0.89	107.5	1.5	high	10.8	6.3		23	11.8
PS	62	M	5	1	25.7	2.9		1.7	0.87	125.1	1.6	high	9.8	3.2		27	9.6
PS	58	F	4	1	17.2	4.3		1.4	1	128.8	1.8	high	8.7	3.7	0.2	30	9
PS	59	F	4	1	18.4	4		1.36	1.24	111.5	1.6	high	10.2	5.7		26	11.1
PS	68	M	3	1	20.6	4		1.57	0.7	117.8	1.9	high	8.8	8.6		27	10.3
PS	69	F	3	1	17.6	3.5	196	1.24	0.88	87.55	1.8	high	9.4	4.9	0.2	33	10.9
PS	51	M	2	1	25.9	3.8	109	1.78	0.84	98.08	2.1	high	7.9	6.2	0.04	46	10.6
PS	78	M	2	1	21.6	3.7		1.35	0.82	71.17	1.6	high	9.7	5.6		30	11.7
PS	80	M	2	1	23.6	4.1	187	1.38	0.89	108.4	2.1	high	8.4	7.3	0.16	32	13.9
PS	55	M	2	1	17	4.3	154	1.18	0.69	74.08	1.6	high	8.8	5	0.41	28	10.5
PS	64	M	2	1	29.9	4	175	1.12	0.86	90.16	2.1	high		7.1			10.1
PS	73	F	3	1	15.7	3.3	151	1.09	0.88	71.73	1.5	high	8.5	4.8	0.2	30	10.4
PS	64	M	20	1	19.9	4.2	194	2.03	0.98	142.9	1.6	high	9.7	5	0.13	26	10.3
PS	59	M	20	1	18	4.3	143	1.56	1.11	118.1	1.8	high	10.9	3.9	0.03		11
PS	71	M	18	1	17.4	4.3	229	2.01	0.73	124.5	1.6	high	10.7	5.5	0.05	29	11.2
PS	83	F	2	1	26.1	3.9	131	1.28	0.99	156.8	1.5	high	10.8	7.7	0.5	34	9.1
PS	79	M	11	1	18	4.3	185	1.62	1.28	150.1	1.6	high	9.7	6.7	0.01	24	10.6
PS	73	M	9	1	21.9	4	174	1.86	1.05	135	1.9	high	8.8	5.4	0.1	31	9.2
PS	60	M	7	1	23.9	4.4	177	1.73	0.9	120.4	2.1	high	7.8	3.9			9.5
PS	56	M	4	1	19.6	4.2	184	1.77	1.02	117.2	1.6	high	9.5	5.1	0.03	24	10.8
PS	63	M	5	1	17.5	3.7	163	1.07	1.34	128.8	2.1	high	8.2	6.6	0.81		10
PS	70	M	10	1	21.5	3.4	120	1.32	0.91	139	1.3	high	9.6	4.2			8.3
PS	75	M	10	1	20.8	3.7	125	1.16	0.8	111.9	1.3	high	8.8	5.2			9.8
PS	61	F	9	1	18.2	4.1	148	2.05	0.94	76	1.5	high	8.4	3.9	0.41	27	10.6
PS	59	M	9	1	20.5	3.6	184	1.44	1.06	119.5	1.5	high	7.6	8.2	0	21	11.2
PS	65	M	8	1	18	3.9	168	1.06	0.9	123.7	2.1	high	7.5	5.4	0	18	10.3
PS	61	F	7	1	19.9	3.3	145	1.1	0.86	109.5	2.1	high	9.4	6	0.05		9.2
PS	66	F	7	1	22.8	3.6	134	1.99	0.83	133.3	2.1	high	10	4.8		24	12.9
PS	68	F	7	1	23.4	4.1	127	1.32	0.82	96.89	1.5	high	8.6	5.7	0.4	34	9.1
PS	79	F	6	1	19.6	3.4	182	1.55	0.73	110.7	1.5	high	9	4.9	0.1	26	11.5
PS	60	M	5	1	24.1	3.9	123	1.3	0.7	78.11	1.5	high	10.2	6.3		27	8.5
PS	73	F	4	1	21.8	3.2	146	1.33	0.72	108	2.1	high	8.8	6.1	0.1	27	10.8
PS	64	F	4	1	20.4	3.7	122	1.25	0.89	90.22	2.1	high	7.9	5.9	0	41	11.1
PS	57	M	4	1	23.7	3.3	135	1.19	0.92	119.9	1.6	high	9.2	5.6		34	11
PS	56	M	4	1	23.8	3.7	152	1.43	1	104	1.5	high	10	9.4		28	12.8
PS	52	F	4	1	23.4	4	224	1.83	0.6	77.59	1.8	high	9.2	6.1	0.12	21	9.6
PS	43	M	5	1	19.5	4.2	133	1.24	0.93	128.7	1.3	high	8.1	4.1	3.9	21	9.3
PS	71	M	4	1	22.2	4.2	172	1.62	0.8	141.8	1.3	high	9.6	5.3	0	30	11.2
PS	67	F	4	1	21.6	3.7	136	1.14	0.81	101.1	1.3	high	7.7	5.9	0.05	27	9.9
PS	50	M	3	1	21.6	3.7	101	1.31	1.03	104.7	2.1	high	9	4.9			10.6
PS	74	F	4	1	17.6	3.6	148	1.66	0.85	111	1.5	high	8.8	3.3	1.1	42	8.5
PS	77	M	3	1	23	3.8	170	1.33	1.17	111.8	2.1	high	9.3	5.1	0.2	33	10.3
PS	69	M	3	1	19.2	3.6	121	0.71	0.91	132.7	1.3	high		5.2	0.17	13	10.4
PS	78	F	4	1	18.7	3.5	133	1.3	0.8	92.57	2.1	high	8.9	5	0.3	29	11.3
PS	68	M	2	1	22.3	3.5	194	0.82	0.71	70.98	2.1	high	9	4.1	0.2	26	10.8
PS	68	M	2	1	24.4	3.5	150	1.54	0.66	59.68	1.5	high		6.2	0.2	27	9.5
PS	59	F	2	1	21.1	3.7	158	1.54	0.68	115	1.5	high	9.2	3.9	0.1	27	8.6
PS	81	F	2	1	29	3.3	199	1.32	0.93	64.74	1.5	high	8.7	6.3	0.18	22	10.7
PS	73	M	8	1	19.4	4		1.24	0.76	132.3	1.4	high	8.6	5.1		33	12.4
PS	72	F	9	1	20.2	3.4	178	1.02	0.85	107.6	2.1	high	10.1	8.5	0.39	25	9.5
PS	51	F	34	1	22.8	3.8	163	1.9	1.15	82.48	1.3	high		4.6	2.33	26	10.1
PS	64	F	3	1	25.9	3.8	119	1.46	0.74	71.21	1.5	high	9	5.9	0.4	18	11.6
PS	67	M	3	1	24.2	3.6	161	1.31	0.72	130.2	1.3	high		5.3	0	17	10.2
PS	44	F	3	1	24.7	4	164	1.14	0.97	93.93	1.5	high		6.5	0	24	10.3

PS	57	M	3	1	20.4	4.1	172	1.31	0.82	127.2	1.8	high		6.1	11.61	29	7.9
PS	65	F	2	1	20	3.6	189	1.16	0.91	77.87	1.8	high	8.5	6	0.2		11.3
PS	79	M	2	1	22.9	4	121	0.97	0.88	89.21	1.5	high	9.5	6.3		18	13.4
PS	58	F	2	1	20.1	3.7	165	1.36	0.74	86.53	1.5	high	8.4	4.5	0.76	26	9.9
PS	61	M	2	1	22.5	3.8		1.34	0.77	56.71	1.8	high	9.8	4.6	0.49	27	11
PS	71	M	2	1	21.1	4	120	1.72	0.83	87.69	1.6	high	9.7	5	0.09	20	10.2
PS	53	M	2	1	25.5	3.2	124	1.71	0.71	69.11	1.1	high	8.8	4.5		29	10.2
PS	64	M	13	1	19.1	3.8	140	1.62	1.16	121	1.8	high	10.2	4.2	1.02	51	10.5
PS	59	M	4	1	21	3.8	156	1.54	0.81	99.98	1.5	high	8.8	4.8	0.27	21	10.6
PS	52	F	5	1	35.7	3.8	94	1.6	1	101.1	2.1	high	8.5	3.8	0.07	31	10.3
PS	58	M	5	1	20.6	3.6	134	1.53	0.57	96.46	2.1	high	10	5.8	0.06		12.8
PS	63	M	6	1	19.6	4.2	147	1.76	0.89	122.7	1.5	high	9.3	4.5	0.49	22	9.3
PS	67	M	4	1	20.4	3.5	174	1.34	0.98	127.1	2.1	high	9.6	7	0.96	35	9.9
PS	53	F	2	1	21.4	4		1.77	0.78	78.11	1.5	high	9.8	4		38	11.5
PS	72	M	9	1	22.1	3.5	129	1.03	0.67	125.7	1.5	high		4.2	0.08	18	11.2
PS	61	F	9	1	18.7	4.1	202	1.46	1.11	145.4	1.6	high		7.1	0.4	23	8.6
PS	56	M	19	1	23.1	4.2	118	1.49	0.99	122	1.8	high	8.9	3.5	0.04	21	11.5
PS	42	F	7	1	19.8	3.7	148	1.76	1.06	109	2.1	high	10.9	4.4	0	31	7.5
PS	73	F	3	1	19.1	3.7	186	1.2	0.84	128.4	1.1	high		8.7	0.14	32	9.5
PS	67	F	27	1	22.1	3.5	170	1.47	0.99	114	1.3	high	8.6	3.7	0.08	22	10.2
PS	46	F	11	1	22.9	3.7	164	1.16	1.07	114.4	1.8	high	9	2.1	0.29	22	10.4
PS	65	F	3	1	24	3.7	92	1.6	0.71	140.7	1.6	high	8.5	4			10.6
PS	54	M	2	1	23.9	3.8	161	1.26	1.22	114.1	1.6	high	8.8	5.7	0.01	22	11.3
PS	57	F	2	1	23.4	4	124	1.03	0.72	82.68	1.8	high	9.7	5.3	0.19	25	10.6
PS	57	F	7	1	24.5	3.8	185	1.32	0.79	120.4	1.5	high	8.7	4.5		28	8.1
PS	54	M	3	1	21.9	3.9	98	1.29	1.05	116.4	1.9	high	9.8	4.9	0.05	28	11.7
PS	77	M	3	1	23.4	3.8	198	1.31	0.65	84.38	1.5	high	8	6.3	0.06	22	11.3
PS	45	F	3	1	27.8	3.4	168	1.07	0.9	125.1	2.5	high	8.7	7	0.04	24	9.3
PS	49	F	32	1	24.1	3.9	140	1.21	1.5	86.99	2.1	high	10.3	5.2	0.07		10.3
PS	65	F	25	1	20.6	3.8	204	1.68	0.87	103.4	1.3	high	8.7	3.9	0.21	38	11.2
PS	36	M	5	1	18.6	4		1.36	0.76	130.1	2.1	high	9.5	6.4		26	10.9
PS	71	M	4	1	16.3	3.7	221	1.48	0.93	132.3	1.6	high		7.1	0.3	24	10.5
PS	69	M	3	1	22.3	4.2		1.28	0.71	69.92	1.8	high	8.5	6.7	0.36	27	10.7
PS	59	M	5	1	32.7	3.7	133	1.72	0.84	115.5	1.8	high	9	4.8	0.85	33	10.9
PS	56	F	33	1	20.6	3.7	122	0.94	1.11	111.2	2.1	high	11.8	7	0.09	29	9.6
PS	56	F	31	1	16.2	4	138	1.06	1.11	103.9	1	high	8.3	5.5	0.11	25	10
PS	57	F	23	1	25.9	3.8	122	1.38	1	143.3	1.8	high	8.8	3.6	0.72	30	9.8
PS	55	M	20	1	19.1	3.8	185	1.92	1.06	107.2	1.4	high		5.7	0.38	23	8.2
PS	66	M	23	1	18.1	3.9	147	1.2	1.01	114.8	2.2	high	10.1	7.4	0.3	30	11.9
PS	64	M	19	1	17.9	3.5	119	0.89	0.84	111.5	1.8	high	7.6	3	0.13	15	9.1
PS	64	M	14	1	22.1	3.7	215	1.24	1.11	128.2	1.1	high	8.4	3.2	0.2	32	11
PS	61	F	11	1	20.2	3.8	163	1.45	1.1	130.1	1.8	high	10.7	3.4			13.1
PS	70	M	10	1	20.6	3.7	148	0.89	0.95	122.4	1.8	high	8.7	4.4	3.55	25	10.2
PS	75	M	8	1	22.8	3.8	108	1.71	1.17	101.1	2.1	high	9.1	6.8	0.18	32	10
PS	72	F	5	1	19.9	4.1	139	1.44	0.87	103.5	2.1	high	9.8	6.8	1.4	29	10.8
PS	39	M	2	1	19.8	4.2		1.13	1.08	111.2	1.6	high	10	6.8	0.03	14	8.9
PS	49	F	2	1	20.4	4	182	1.23	0.92	104.2	1.8	high	9.9	6.4	0.8	28	11.1
PS	66	M	2	1	20.2	4.1	184	1.43	1.05	117.1	2.1	high	9.8	4.7	0.43	35	13.9
PS	52	M	27	1	22.4		100	1.78	0.8	60.33	1.9	high	9.8	5.3	0.04	23	9.5
PS	54	M	25	1	17.5		181	0.9	0.89	117.8	2.1	high	9.1	6.9	1.55	29	11.9
PS	65	F	19	1	15.6		184	1.63	0.87	155.1	1.6	high	9.7	6.3	0.26	33	10.7
PS	51	M	11	1	20.3		155	1.61	0.94	114.5	1.5	high	9	2	0.15	24	9.7
PS	42	F	11	1	18.8		209	1.57	0.73	87.83	1.5	high	9.2	5		29	
PS	45	F	9	1	18.6		191	1.1	1.07	103.7	2.1	high	9.2	5.1			10.8
PS	58	F	8	1	18.4		193	1.19	1.03	109.5	1.9	high		5.9	0.1	24	10.7
PS	79	M	6	1	19		186	1.2	0.85	102.1	1.6	high		4.1	0.1	14	11.4
PS	67	M	6	1	24.5		175	1.39	0.86	104.3	1.3	high		3.6	0.2	28	9.6
PS	74	F	5	1	20.4		162	1.78	0.89	104.7	1.6	high		5.4	0.1	26	9.3
PS	65	F	5	1	20.1		172	1.57	0.69	109.7	1.8	high		5.4	0.05	30	10.3

PS	62	F	5	1	21.8		88	1.49	0.9	98.13	1.8	high				43	9.6
PS	43	M	5	1	28.4		139	1.07	0.74	76.01	1.9	high		4.3	0.1	27	10.6
PS	64	F	4	1	19.1		111	1.41	0.9	81.02	2.1	high	9.6	8.4	0.05		10.1
PS	56	F	3	1	19.6		135	1.6	0.91	92.19	1.3	high	8.2	5.9	0.77	22	7.8
PS	57	F	2	1	18.4		125	1.16	0.97	110.2	2.1	high	9.5	4.6	0.07	24	10.8
PS	72	M	2	1	21		158	1.3	0.84	58.68	1.8	high	9.7	4.9	0.05	24	9.8
PS	34	M	2	1	30.4		145	1	0.78	119.4	2.1	high	10.3	6.3	0.09	26	10.9
PS	34	M	8	1	19.2	4.3	179	1.7	1.16	89.46	1.8	high	9	5.1	0.04	26	9.6
PS	68	F	5	1	22.6	3.7	104	1.25	0.67	76.37	2.1	high	8.4	2.9	0.07	37	10.3
PS	74	F	12	1	20.1	3.3	133	1.25	0.85	107.8	2.1	high	9.1	5.8	0.05		9.6
PS	59	F	5	1	21.2	3.9	107	1.53	1.14	112.5	1.3	high		2.3	0.28	23	10.5
PS	55	M	3	1	22.9	4		1.39	0.67	75.48	2.1	high	9	5.1		43	12.7
PS	47	F	17	1	20.1	4	199	1.1	0.79	116	1.4	high	8.6	6.6	0.25	17	12.4
PS	59	M	19	1	14.8	4.1		1.7	1.07	94.78	1.6	high	8.9	4.7	0.06	30	11.6
PS	67	M	2	1	21.5	3.8	182	1.14	1.19	129	2.1	high	9.6	6.4	0.16	22	10
PS	75	F	5	1	18.4	3.8	191	1.08	0.86	119.2	1.8	high	10.1	4.1		28	10.5
PS	78	F	11	1	22	3.3	126	1.36	0.79	100.8	1.9	high		5.6	0.1	28	10.7
PS	33	F	2	1	21.1	3.7	88	1.2	1.14	107.5	1.8	high	9.4	10.4	0.31	28	11.4
PS	62	M	9	1	21.4	4.9	217	1.58	1.11	103.8	1.6	high		5.5	0.4	21	9.2
PS	47	M	16	1	18.9	3.9	161	1.76	1.14	101.8	1.3	high		5.2	0.1	28	8.7
PS	58	M	4	1	21	3.7	105	1.82	0.99	95.16	1.5	high	8.7	3.2	2.36	26	8.6
PS	42	M	35	1	21.7	4.1	111	1.76	0.77	91.99	1.5	high	9.2	5.6	0.46	24	9.5
PS	64	F	22	1	19.7	3.5	116	1.62	1.09	115.9	1	high	8.7	3.4	0	22	9.6
PS	66	M	21	1	19.4	4	180	1.87	0.86	106.3	1.8	high	9.4	5.1	0.01	22	9.2
PS	64	M	20	1	20.5	4.1	130	2.72	1.08	150.6	1.6	high	7.3	6.3	0.04	22	10.4
PS	65	F	20	1	19	3.9	209	1.42	0.87	138.8	1.6	high		6.8	0.3	25	9.6
PS	70	M	19	1	18.7	3.6	178	1.56	1.09	133.5	1.4	high	9.6	4	0.2	26	11
PS	53	M	18	1	21.2	4.4	144	1.48	0.8	117.7	1.3	high	9.1	5.5	0	27	9.8
PS	76	M	17	1	20.3	3.9	110	1.38	0.94	128.5	2.2	high	9.3	5.2	0.1	23	9.2
PS	68	M	16	1	21.3	3.9		1.81	0.88	116	1.5	high	9.6	7.4	0.01	36	9.8
PS	63	M	16	1	20.6	4.1	118	1.29	0.94	100.2	2.2	high	9.7	8		34	6.9
PS	58	F	14	1	19.3	4.5	166	1.35	1	128.4	1.8	high	10.4	5.9		28	12
PS	67	M	13	1	21.7	3.8	163	1.38	1.01	131.1	1.6	high	9.9	5.9	0.07	27	10.8
PS	70	M	11	1	21.2	3.9	128	0.96	1.06	103	1.5	high	9.6	3.2	0.76		11.1
PS	52	M	11	1	27.4	4.3	137	1.42	0.77	107.6	1.6	high	9.8	4.8	0.05	26	10.6
PS	73	M	9	1	19.9	4.2	115	0.82	0.94	132.1	1.8	high	7.9	8.1	4.9	29	8.4
PS	72	M	7	1	22	4.1	110	1.23	0.92	139.2	1.3	high		3.2	0.28	37	8.8
PS	81	M	7	1	25.9	3.4	105	1.33	0.96	155	1.5	high	7.4	6.6	0.1	25	10.5
PS	67	F	6	1	19.5	3.8	155	2.03	0.89	130.7	1.5	high	8.5	8.3	0.1	28	8.7
PS	59	M	5	1	24.5	4.3	175	1.67	0.77	97.51	1.5	high	8.8	5	0.7	41	9.3
PS	67	M	4	1	21.5	3	133	1.38	0.75	113.9	1.9	high		5.3	0.2	32	11.4
PS	61	M	4	1	24.7	4.3	135	1.63	0.87	110.4	1.5	high	6.7	3.4			9.7
PS	62	M	3	1	21.7	4.2		1.21	0.96	113.3	1.3	high	7.3	3.9	8.7	18	9.9
PS	66	M	3	1	18.2	4.1	187	0.92	0.74	38.35	1.3	high	9.8	3.3		23	10.9
PS	51	F	3	1	18.4	4.2	120	1.57	1.05	116.1	1.8	high	9.1	5.9	0.05	25	10.2
PS	77	F	2	1	17.5	4.2	176	1.61	0.89	128.6	1.5	high	8.7	3.9	0.33	25	10.6
PS	73	F	2	1	21.2	3.9	157	1.23	0.86	148.8	1.6	high		9.1	0.5	57	10.5
PS	78	M	2	1	23	3.9	192	2.36	0.65	100.6	1.3	high	9.2	4.4		22	7.8
PS	79	F	2	1	25.4	3.4	158	0.92	0.88	129.1	1.9	high		4.6	0.1	17	10
PS	69	M	15	1	21.8	3.8	176	1.61	1.12	114	1.5	high	9.5	3.9	0.02	23	9.4
PS	67	F	8	1	22.2	4	153	1.5	1.04	128.1	1.8	high	8.4	5.1	0.06		10.7
PS	60	M	14	1	24.1	4.3	128	1.99	0.98	114	1.8	high	9.2	4.4	0.06	37	11.3
PS	75	M	12	1	20	4.1	132	1.29	0.77	123.5	1.4	high	7.4	8.3	0.53	37	9.2
PS	62	M	8	1	21.9	4.4	149	1.36	1.04	92.8	2.1	high	9	7.3		31	11.5
PS	84	M	7	1	20.5	4.2	174	1.21	0.84	103.1	2.2	high		5	0	34	8.6
PS	76	F	7	1	20.6	3.6	147	1.66	0.92	137.9	1.8	high		5.2	0.86	22	10.1
PS	66	F	7	1	26.2	3.9	205	1.31	0.93	90.87	1.8	high	10.1	5	0.05	27	10
PS	50	F	6	1	24.7	3.9	154	1.65	1.12	135.3	1.8	high		6.1	0.06		11.9
PS	49	M	5	1	27	3.8		1.43	1.05	118.1	1.5	high	8.2	6	1.13	44	7.2

PS	66	F	4	1	26.1	3.9	212	1.46	0.91	124.7	1.3	high		5.5	2.5	29	9.4
PS	65	M	4	1	24.4	4	106	1.75	1.42	101.6	1.6	high		5.2	0.1	22	9.1
PS	68	F	3	1	20.5	3	227	1.43	0.89	119.8	1.5	high	8.8	6.8	12.03	25	8.8
PS	76	M	2	1	21.3	3.3	168	1.76	0.8	104.7	1.8	high	9.8	7.1	0.08	24	10
PS	71	M	2	1	19.9	3.7		1.63	0.97	106.5	1.8	high		5.9			9.9
PS	79	M	2	1	19.5	3.5	144	1.35	0.79	87.61	1.8	high		4.6	0.19	29	9.6
PS	83	M	12	1	21	3.1	203	1.33	1.06	101.5	1.6	high		6	0.2	19	10.1
PS	64	F	2	1	20.6	3.3	189	1.55	0.98	51.9	1.8	high	10.2	5		34	10.6
PS	47	M	5	1	24.6	3.8	101	1.55	0.94	115.3	1.3	high	9.4	6.1		19	11
PS	67	F	2	1	25.4	3.9	174	1.22	1.05	135.6	1.8	high	8.8	5.5	0.2	32	9.9
PS	74	M	9	1	23.3	4.1	159	1.22	0.96	141.9	1.6	high		5.6	0.1	26	11.3
PS	67	F	2	1	27.8	4.1	104	1.48	0.92	106.2	1.8	high	9.2	5.3	0.75	33	9.7
PS	64	M	14	1	22	3.5	122	1.72	0.77	95.5	1.5	high	9.2	5.7	0.07	25	9.4
PS	52	M	15	1	23.1	3.6	149	1.48	1.05	105.7	1.3	high		5.9	0.1	40	8.5
PS	82	F	10	1	16.3	3.9	150	1.5	0.94	90.65	1	high	9.3	5	0.1	30	11.4
PS	77	M	4	1	23.2	3.9	91	1.1	0.83	107.7	1.3	high	8	4.2	4.64	34	10.8
PS	67	F	21	1	17.5	4.2	150	1.4	0.88	114.5	1.6	high		4.7	0.1	26	9.2
PS	61	M	20	1	24.1	3.8	103	1.26	0.93	124.2	1.6	high		4.9	0.4	23	8.9
PS	45	M	17	1	22.4	4.2	102	1.53	0.89	144.1	1.9	high		6.2	0.1	25	9.1
PS	53	F	16	1	15.2	3.7	112	1.1	1.03	111.4	1.3	high		5.1	0.1	20	10.8
PS	51	F	14	1	18.3	4.2	120	1.38	0.79	133.9	1.6	high		8.7	0.1	22	9.7
PS	60	F	13	1	19.1	4.3	158	1.04	1.16	95.69	2.1	high	9.5	5.6	0.1	21	10.9
PS	60	F	12	1	22.6	4.2	98	1.17	0.66	110.6	1	high	10.3	4.9	0.1	30	10.6
PS	60	F	9	1	21.9	3.9	164	1.62	1.03	148	1.6	high	9.1	5.3	0.07	15	12
PS	53	F	8	1	16.8	4.2	155	2.16	0.67	99.71	1.6	high	9.1	4.6	0.09	36	11
PS	59	M	3	1	19.3	4.2	154	1.5	1.05	97.37	1.6	high	9.3	5.3	0.64	21	10.5
PS	50	M	3	1	24.6	3.9	158	1.19	0.82	68.2	1.5	high		4	0.73	40	9.9
PS	64	F	3	1	23.8	4.1	138	1.82	0.94	123.8	1.9	high	9.1	4.8	0.09	27	10.7
PS	61	M	2	1	22	4.2	194	1.17	1.13	95.6	1.5	high	9.1	6.4	0.81	24	8.7
PS	47	M	2	1	25.2	4.4	122	1.63	1.13	98.67	1.9	high	8.8	4.8	0.05	38	10.9
PS	60	F	2	1	35.8	3.8	154	1.2	1.05	99.81	1.6	high	9.4	6.7	0.24	28	11.2
PS	78	M	2	1	17.9	3.9	155	1.69	0.89	89.37	1.6	high	10.7	5.7	0.27	27	10.9
PS	63	F	2	1	19.9	3.9	151	1.48	1.03	84.34	1.6	high	9.3	4.1	0.27	27	9.9
PS	59	M	2	1	19.4	4.1	125	1.31	0.88	92.24	1.8	high	9.1	4.9	0.07	28	11.8
PS	50	M	2	1	17.5	4.3	129	1.21	0.99	84.85	1.8	high	9.5	9.5	0.3		10.7
PS	72	M	7	1	23.2	4	144	1.35	0.9	91.6	2.1	high	9.5	5	0.05	25	11
PS	60	F	23	1	15.7	3.6	185	1.8	1.02	120.7	1	high	9.6	6.4	0	22	10.9
PS	55	M	22	1	22.6	3.9	124	1.49	0.99	124.5	1.6	high	8.3	4.7	0.1	26	12.2
PS	52	F	16	1	22.4	4	219	1.01	1.14	138.5	1.5	high	8.6	4.5	1.6	16	9.7
PS	69	M	12	1	23.4	3.8	146	1.62	0.82	159.1	1.8	high	8.7	4.4	0.47	30	11.2
PS	48	M	10	1	21.6	4.3	169	1.73	0.84	142.6	1.1	high	9.5	3.3	0.16	28	8.8
PS	59	F	4	1	18.8	3.9	148	1.27	0.77	93.16	1.6	high	8.9	4.8		24	
PS	80	M	4	1	20.1	3.3	244	1.3	0.71	89.46	1.8	high	8.1	6.2	1.7		9
PS	71	F	2	1	23.3	3.6	224	1.94	1.3	134.3	1.8	high	8.5	4.7		15	9.8
PS	44	F	2	1	19.8	4.1	146	1.78	1.02	138.2	1.4	high	8.1	6.6	0.51	31	8.2
PS	65	M	12	1	24.9	3.9	156	1.77	1.07	125.3	1.5	high	9.6	4.1	2.1	26	10.7
PS	61	M	5	1	22.5	4	235	1.06	0.74	107.3	1.5	high	9.5	3.5		18	10.4
PS	73	F	2	1	19.7	4.1	123	1.4	0.8	73.94	1.9	high	9.3	3.4	0.1	32	10.5
PS	78	M	6	1	19	3.6	119	1.48	0.83	71.37	1.5	high	9.3	4.4	0.22	26	9.1
PS	73	M	2	1	18	3.4	117	1.7	0.72		1.8	high	9.1	6.3	0.44	51	9.6
PS	85	F	3	1	15.1	3.2	147	1.18	0.8	90.45	2.1	high	10.2	8.8	1.4	32	11.2
PS	75	F	29	1	20.9	2.8	134	1.66	0.66	84.92	1.6	high	9.2	4.5	0.05	25	10.6
PS	72	F	24	1	22.2	2.9	202	1.63	0.96	97.75	1.6	high	10	5.8	0.05	29	10.6
PS	59	M	24	1	17.6	3.5	207	2.07	1.01	113.4	1.6	high	9.1	6	0.05	23	11.4
PS	66	F	12	1	18.4	3.3	138	1.02	1.15	131.2	2.1	high	8.7	5	0.06		10.5
PS	61	M	8	1	25.8	3.9	145	1.55	1.05	128.3	1.5	high	9	4.9	0.04	30	8.9
PS	51	M	7	1	18.9	4.1	213	1.61	1.04	132.7	1.8	high	8.2	5.4	0.08	22	11.1
PS	57	M	6	1	21.7	3.6	147	1.35	0.63	99.5	2.1	high	8.4	4.8	0.03	26	11.7
PS	64	M	6	1	19.4	4	78	1.82	0.88	89.21	2.1	high	8.9	4.3	0.04	26	11.7

PS	64	M	6	1	19.2	4	176	1.64	0.91	82.49	1.8	high	9.4	5.4	0.31	24	9.6
PS	67	M	5	1	22.6	3.5	170	1.7	0.8	165.2	1.6	high	10.2	6.2	0.11	21	11.1
PS	65	F	4	1	24.6	3.6	161	1.26	0.98	118.1	2.1	high	8.9	5.8	0.1	25	11
PS	63	M	4	1	22.9	3.6	167	1.09	0.3	79.72	1.8	high	8.4	6.9	0.05		10.7
PS	73	F	4	1	25.4	3.7	146	1.72	1.05	133.8	1.3	high	8.6	4.7	0.28	37	10.1
PS	68	F	4	1	18.5	3.2	65	1.21	0.89	72.43	2.1	high	10.4	9.2	0.13	24	12.7
PS	81	F	4	1	23.6	3.5	81	0.67	0.92	154.9	2.1	high	7.9	3.4	7.5	24	7.1
PS	71	M	3	1	20.2	3.9	109	1.55	0.85	88.54	2.1	high	8.4	6	0.13	22	11.3
PS	80	M	3	1	15.4	3.7	137	1.31	0.97	137.4	2.5	high	10.4	4.6	0.05	20	10.8
PS	80	F	2	1	15.7	3.2	149	1.49	0.89	118.5	1.8	high	10.4	7.3	0.11	21	9.9
PS	62	M	2	1	20.1	3.8	237	1.38	0.73	83.46	1.8	high	8.7	5.9	0.28	20	10.2
PS	74	M	2	1	22.2	4	163	1.45	0.97	112.7	2.1	high	8.3	4.9			10.9
PS	52	F	9	1	22.8	3.8		1.44	1.25	115.8	1.3	high	9.1	8.1		59	9.1
PS	46	M	9	1	25.8	3.9		1.64	1.14	115.3	1.3	high	8.7	4.7		29	10.8
PS	59	M	3	1	27.1	4.3	211	1.43	0.96	111.3	1.5	high	9.1	5.7	0.05	23	10.4
PS	55	M	12	1	23.2	3.8	141	1.8	1.12	129.8	1.8	high	8.6	5.2	0.1	28	10
PS	42	M	3	1	27.1	4.5	98	1.15	1.01	102.7	1.3	high	8.7	5.1	0.03	26	10.6
PS	44	M	3	1	21.4	4.5	196	1.95	1.23	128.8	1.5	high	10	2.2	0.81	35	10.5
PS	60	M	2	1	23.4	3.7		1.71	1.03	77.7	1.8	high		5.5			10.9
PS	57	F	4	1	33.5	3.4	159	1.42	0.68	121	1.1	high	8.8	5.1	0.1	26	10.8
PS	37	F	14	1	19.9	4.4	122	1.51	0.8	125.8	1.1	high	9.2	3.7	2.03	51	9.4
PS	50	F	18	1	13.6	4.1	78	1.31	0.98	107	1.4	high	8	5.6		35	12
PS	62	F	27	1	17.1	4.3	122	1.3	1.37	103.6	1.3	high		6.4		27	11.3
PS	82	M	4	1	24.7	3.7	161	1.56	0.73	166	1.1	high	9.1	5.9	0.56	36	10.5
PS	68	M	3	1	22.6	3.7	201	1.63	0.72	84.25	1.5	high	9.3	6	0	27	10.2
PS	71	M	11	1	20.7	3.7	148	1.48	0.96	133.6	1.3	high	8.5	4	0.15	33	9.6
PS	55	M	2	1	23.1	4	153	1.72	0.9	68.59	1.5	high	8.2	3.3	1.6		10.3
PS	66	F	6	1	18.4	3.9	119	1.36	1.04	127.1	1.6	high	8.5	5.2	0.02	34	11.6
PS	69	M	15	1	21.1	3.4	219	1.11	0.75	103.4	1.1	high		4	1.7	34	10.8
PS	48	M	3	1	24.5	4	172	1.36	0.89	97.16	1.3	high	9.3	6.2	0.68	29	10.2
PS	59	M	9	1	22.2	3.8	120	1.4	0.95	134.9	1.3	high	7.8	6.7	0.15	32	9.5
PS	61	F	23	1	17.9	3.5	171	1.21	0.97	106	1.6	high	8.5	6.6		22	8.9
PS	29	M	2	1	19.1	4.1	110	0.81	0.96	90.5	1.8	high	9.3	8.8	0.51	26	9.6
PS	55	F	2	1	30.1	3.4	145	0.85	0.89	80.05	2.1	high		5.6	6.92	26	10.1
PS	75	F	5	1	30.7	3.3	126	1.71	0.67	79.52	1.5	high		5.9	6.59	35	9.7
PS	56	F	5	1	19.5	3.5	158	1.58	0.92	130.1	2.1	high	8.8	4.9	0.09	26	9.7
PS	41	M	8	1	23.7	3.7	160	1.12	1.02	93.37	1.5	high	8.2	4.9	0.1		10.3
PS	73	F	10	1	21.1	3.3	115	1.45	1.31	133.4	2.1	high	9.9	7	0.01	27	11.3
PS	70	M	13	1	19.7	3.1	116	1.18	0.72	89.52	2.1	high	8.3	6.5	0.05		11.2
PS	64	M	15	1	23.9	3.2	150	1.32	0.95	125.1	1.9	high	9.6	6.4	0.12	26	11.8
PS	54	M	7	1	19.7	3.6	132	1.61	1.18	138.1	1	high	9.2	5.5	0.28	23	10.1
PS	57	M	7	1	25.4	3.8	159	1.71	0.96	130.7	1	high	9.4	5.5	0.5	22	9.7
PS	58	F	9	1	17.1	3.8	229	1.45	1.02	112.7	1.3	high	8.9	4.1	0.1	14	10.8
PS	65	F	15	1	25.7	3.8	134	1.48	1.32	106.5	1.3	high		4.6	0.1	25	9.7
PS	65	M	4	1	22.3	3.5	198	1.78	1.15	120.9	1.3	high	9.6	4	0.1	23	10.2
PS	67	M	8	1	22.2	3.4	152	1.27	0.95	83.53	1.8	high	9.2	4.1	5.51	33	11
PS	61	F	25	1	20.6	3.2	140	1.18	0.94	104.9	1.6	high	10.4	5.4		17	10.6
PS	64	M	2	1	22.7	3.7	128	1.2	1	76.06	1.5	high	8.3	6.6		42	9.5
PS	57	M	7	1	20.1	3.5	146	1.44	0.89	93.92	1.5	high		7	0.1	34	11.8
PS	53	F	12	1	18.5	3.3	164	1.71	1.11	97.86	1.9	high	9.2	4.3	0.1	24	11.1
PS	62	M	19	1	17	4	92	1.84	0.93	69.29	1.5	high	8.8	6.7	0.3	31	5.8
PS	55	M	24	1	19	3.5	198	1.15	1.37	96.82	1.6	high	8.9	3.4		14	12.3
PS	56	F	2	1	23.2	3.6	209	1.36	0.94	128.7	2.1	high	8	7.9	0.2	37	9.6
PS	54	F	4	1	21.2	3.9	178	1.39	0.99	105.7	2.1	high	10	6.7	0.18	27	10.4
PS	55	M	9	1	21.4	3.5	121	1.75	1.04	107.6	1.6	high	9.2	3.3	0.2	25	10.5
PS	71	M	6	1	26.1	3.6	146	1.25	0.96	130.1	1	high	8	3.5	6.63	23	10.9
PS	58	F	10	1	17.9	3.6	156	2.01	0.8	79.66	1.3	high	8.5	5.5	0.54	36	9.2
PS	42	F	15	1	16.3	3.3	144	1.88	0.83	127.6	1.6	high	9.1	3.9	0.2	25	10.3
PS	65	F	6	1	19.2	3.6	116	1.16	1.09	95.93	1.5	high	7.5	3.9	2.24	21	8.7

PS	73	F	13	1	18.1	3.7	102	2.07	1.38	107.8	1.3	high	8.2	5.5	1.8	26	10.7
PS	60	F	13	1	18	3.7	153	1.78	1	127.3	1.6	high	8.9	3.2	0.5	28	10.1
PS	89	M	15	1	22.8	3.1	137	1.04	0.72	113	1.9	high	9.8	4.3	0.1	19	10.6
PS	59	M	18	1	26.5	3.2	130	1.32	1.01	118.1	1.6	high	9.4	5.6	0.1	23	10.7
PS	67	F	8	1	21.3	3.5		1.77	1.06	132.7	1.3	high	8.8	3.9		31	9.7
PS	41	M	14	1	19.8	3.6	102	1.46	0.98	121.9	1.6	high	9.3	6	0.1	27	11.3
PS	72	F	18	1	24.2	3.3	158	1.81	0.92	124.9	1.3	high	9.6	4.2	0.1	26	9.9
PS	52	M	24	1	22.9	3.9		0.78	0.96	95.85	1.9	high		8.2	0.39	24	8.3
PS	30	M	8	1	24.2	4.2	148	1.56	0.97	129.1	2.1	high	8.9	6	0	29	9.5
PS	60	M	2	1	27.7	3.6	112	2.22	0.86	96.76	1	high	8.3	6	0.2	30	10.4
PS	35	F	3	1	28.6	3.7	175	1.27	0.78	120.4	2.1	high	9	5	0.1	26	10.4
PS	60	M	10	1	19.3	3.5	130	1.57	0.91	128.1	1.3	high	8.5	5.4	0.1	23	11.9
PS	56	M	14	1	20.1	3.2	152	1.71	0.86	84.71	1.3	high	9.8	4.5	2	35	11.5
PS	62	F	9	1	22.3	3.6	119	1.59	1	132.5	1.5	high	9.3	4.5	0.19	20	11.1
PS	55	F	5	1	16.2	3.9	134	1.72	1.06	109	1.3	high	9.7	5	0.2	27	10.4
PS	69	M	21	1	23.1	3.3	160	1.39	0.82	96.49	1.9	high	8.9	4.2	0.05	28	9.2
PS	41	M	3	1	25	3.4	190	1.75	0.74	91.47	1	high	8.5	4.3	0.1	24	10.9
PS	41	F	13	1	22.2	3.7	117	2.11	0.76	104.4	1	high	10	6.2	0.2	25	12
PS	77	F	3	1	20.2	3.6	159	2.29	0.86	80.3	1.3	high	9.1	4.2	0.2	29	11.2
PS	70	F	12	1	18.1	3.6	178	2.03	1.02	112.9	1	high	9.5	4.5	0.1	18	10.2
PS	74	M	5	1	26.5	3.2	224	1.2	1.21	101.5	1.8	high	10	7.4	0.1	27	9.4
PS	56	F	2	1	23.7	3.8	168	1.75	0.76	73.4	1.3	high		4.4	0.1	20	11.1
PS	53	F	7	1	19.6	3.5	207	2.06	0.86	111.3	1	high	8.9	3.9	0.2	25	9.7
PS	67	F	7	1	21.7	3.5	145	1.74	0.8	106.6	1.5	high	9.5	6.7	1.31	23	7.2
PS	57	M	8	1	21.5	3	103	1.72	0.68	89.54	2.5	high	8.7	4.2		56	11.2
PS	48	F	17	1	24.3	3.5	125	1.59	0.99	94.82	1.3	high	8.4	4	2.5	20	10.9
PS	62	F	2	1	16.3	3.5	130	2.26	1.01	76.16	1.6	high	9.4	4.2	0.1	20	10.9
PS	52	M	2	1	25.4	3.8	136	1.68	1.06	113.6	1.5	high		4.7	0.4	24	8.4
PS	66	M	3	1	18	3.8		1.37	0.69	62.57	1.5	high	9.6	6.1	0	22	11.3
PS	60	M	4	1	24	3.8		0.7	0.94	103.3	1.9	high	8.5	5.9	0.2		7.6
PS	52	F	10	1	22.1	3.3		1.61	0.91	84.03	1.9	high	8.2	5.4	0.1		9.4
PS	65	M	13	1	20.4	3.7		1.12	0.97	110.2	1.6	high	8.9	6.7	0.1		7.3
PS	79	F	17	1	25	3.3	107	2.1	0.95	98.53	1.9	high	9.5	4.4		37	10.9
PS	62	F	18	1	17.5	3.6	140	1.69	1.23	113.1	1.5	high	9.2	4.4	0.1	30	10.1
PS	36	F	14	1	20.1	3.7		1.64	0.91	106.2	1.6	high	8.6	4.2	0.1		10.8
PS	63	F	9	1	21.2	3.3	153	1.37	0.94	105.9	2.1	high	9.9	4.4	0.1	27	9.4
PS	62	M	18	1	19.7	3.1		1.32	1.16	100.1	1.6	high	10.4	6.2	0.1		9
PS	60	F	20	1	20.3	3.3	168	1.45	1.01	87.08	1.6	high	9.1	5.4	0.05	33	8.5
PS	69	M	2	1	22.3	3.4	171	1.61	0.8	56.58	1.8	high		3.9	0.2	30	9.8
PS	60	F	11	1	23.6	2.4	167	1.14	0.58	72.73	1.6	high	8.8	4.9	0.09	25	10.8
PS	63	M	11	1	19.2	3.8	116	1.36	1	128.6	2.1	high	9.7	4.8	0.13	25	10.6
PS	77	F	5	1	22.1	3.3	103	1.36	1.27	88.5	1.8	high		4.9	1.1	30	10.6
PS	55	F	6	1	28.4	4.2	174	1.57	1.08	141.8	1.3	high		5.7	0.1	29	9.1
PS	61	F	10	1	25	3.7	144	1.57	0.88	107.2	1.9	high	8.6	6.2	0.07	25	10.8
PS	54	M	11	1	21.4	3.6	187	1.72	0.78	122.5	1.6	high	10	5.7	0.09	20	11.8
PS	37	F	17	1	23.1	3.5	179	1.82	1.09	119.2	1.3	high	8.6	5.1	0.26	26	10.3
PS	67	F	23	1	24.1	3.5	118	1.02	1.39	88.01	1.6	high	8.3	5	0.4	17	9.4
PS	29	M	7	1	16.8	4	140	1.59	1.26	122	1.8	high	8.9	7.4	0.1	27	10.1
PS	36	M	7	1	18.9	2.9	190	1.35	0.67	88.34	1.6	high	8.7	3.5	0.05	9	10.4
PS	62	M	10	1	24.3	3.4	112	1.37	0.76	122.3	2	high	10.6	7.1	0.03	25	11
PS	55	M	16	1	20.9	4	133	1.48	0.97	111.2	1.9	high	8.2	7.4	0.05	28	12.6
PS	67	M	13	1	21.5	3.3	130	1.61	0.91	141.8	1.6	high	9	6	0.28	34	11.5
PS	59	M	9	1	20	4.1	179	1.48	0.95	151.9	1.9	high	8.2	4.3	0.05	27	10.8
PS	57	F	2	1	16	4	127	1.54	1.06	101.4	2.1	high	9.2	6.3			10.2
PS	59	F	13	1	31	3.9	116	1.32	1.19	126.5	1.5	high	9.9	2.8		19	9.9
PS	76	M	8	1	21.4	3.8	155	1.47	0.82	127.5	2.1	high		5.2	1.35	35	10.2
PS	53	F	13	1	19.9	3.4	140	1.68	1.03	99.75	2.1	high	7.6	5	0.11	25	10.1
PS	69	M	4	1	25.4	4.1	154	1.78	0.84	234.2	2.1	high	9.6	6.2	0.19	23	
PS	51	F	3	1	14.1	4.1	123	1.25	0.5	87.51	1.8	high	8.9	5.8	0.97	25	10.6

PS	68	M	8	1	20.2	4	233	1.87	0.74	96.32	1.5	high	8.3	4	2.49	26	9.3
PS	54	M	4	1	28.4	3.9	147	0.92	0.48	100.2	1.3	high	8.7	6.8		23	7.5
PS	75	M	5	1	20.5	3.9	126	1.43	0.86	137.6	1.5	high	8.3	5.1	0.16	27	9.6
PS	82	F	3	1	19.1	4	125	1.16	0.71	105.5	2.5	high	10.4	11.8	0.5	31	11.6
PS	64	M	5	1	21.6	4.3	119	1.4	1.12	145.8	1.5	high	8.4	2.3	0.04	26	9.8
PS	37	F	3	1	20.7	3.4	147	2.61	0.72	105.3	1.5	high	10.9	4.9	0.1	32	9.2
PS	71	M	5	1	22.4	3.4	92	1.12	0.94	75.42	1.8	high	9.3	3	0.07	25	8.8
PS	72	F	4	1	22.6	3.5	154	1.39	0.92	96.13	1.5	high	9.7	6.1	0.06	28	11.5
PS	73	F	8	1	17.8	3.5	182	1.12	1.26	99.32	2.1	high	8	5.5	0.06		11.1
PS	42	M	7	1	21.2	4.1	149	1.23	1.01	90.11	1.9	high	9.4	5.2	0.06	27	9.8
PS	66	F	4	1	20.7	3.8		1.33	1.09	160.5	2.5	high	9.5	5.2	0.88		10.7
PS	35	M	12	1	17.8	3.7	136	1.57	0.99	94.61	2.1	high	10.1	5.3			9.8
PS	60	M	8	1	20.4	4.5	104	0.85	1.03	113.6	2.1	high	8.9	5.2	0.07	31	10.1
PS	66	M	3	1	19.1	3.6		1.74	0.91	99.22	1.3	high	8.8	5	1.2	23	10.4
PS	83	F	2	1	17.1	3.7	125	1.99	0.99	79.8	1.5	high	9.8	4.5	1.39	21	10.9
PS	45	F	2	1	21.9	3.9	142	1.46	0.82	90.99	1.3	high	8.7	4.2	3.74	18	9.5
PS	66	F	22	1	17.2	4	153	1.77	1.26	109.2	1.9	high	8.9	5.3	0.09	33	10.6
PS	64	F	9	1	18.6	3.9	195	1.55	1.25	127.1	1.6	high	8.9	5.1	0.05	23	10.9
PS	60	M	11	1	20.3	4.7		1.77	0.92	130.2	1.6	high	8.7	6.9		36	10.6
PS	60	M	2	1	20.1	4	106	1.45	0.84	86.18	1.5	high	9.5	4.4		32	9.3
PS	69	F	14	1	20.2	4	153	1.2	0.84	130.8	1.8	high	9.8	5	0.02	21	10.4
PS	47	M	9	1	18.1	4.1	159	1.87	1.08	117.5	1.5	high	10.2	4.8	0.11	28	9.3
PS	65	M	12	1	21.9		123	1.81	1.06	123.4	1.8	high	9.2	3.3	0.09	29	11.7
PS	69	M	6	1	22.8	3.4	107	1.31	0.96	99.68	1.3	high		6	0.09	21	11.3
PS	68	F	12	1	16.9			1.32	0.72	31.41	1.6	high	8	2.8		35	9.6
PS	68	M	10	1	18.7		204	1.77	0.76	92.6	1.5	high	9.3	4.6	0.09	27	10.9
PS	81	M	4	1	24		202	1.42	0.84	110.8	1.9	high	8.8	4.4	0.33	24	12.1
PS	47	M	30	1	21.6	3.9	150	1.56	0.92	127	1.8	high	9.1	4.5	0.02	30	11.9
PS	46	M	25	1	21.7	3.8	114	1.28	1.05	118.6	1.3	high	8.4	6.4	0.05	21	11.1
PS	67	M	19	1	19.7	3.9	116	1.67	0.82	119	2.1	high	9.3	6.3	0	25	9.8
PS	51	M	19	1	23.7	3.9	105	1.9	1.19	145	1.5	high	8.3	6	0.16	29	11
PS	53	F	18	1	17.5	4.3		0.98	0.81	92.11	1.6	high	8.7	5.5		23	9.6
PS	31	M	18	1	19.6	4.7	112	1.35	0.86	84.02	2.5	high	8.9	6.9	0.07	34	10.7
PS	46	M	17	1	21.2	4.1	147	0.98	0.83	91.09	2.1	high	10.2	4	0.1		11.2
PS	63	M	15	1	18.8	4	127	1.5	1.21	125.9	1.8	high	9.8	10.3	0.22	33	13.9
PS	68	M	14	1	19	4.2	222	1.5	0.98	121.5	1.8	high	9.3	5.1	0.09	27	10.7
PS	58	F	14	1	19.9	3.9	129	1.22	1.2	70.31	1.3	high	9.2	7.5	0.07	25	10.5
PS	49	F	14	1	24.2	4	118	1.28	0.73	101.6	1.5	high	9.1	3.6	0.2		9.8
PS	60	F	14	1	16.1	4.1		1.07	1.26	119.3	1.5	high	9.7	5.3		26	9.5
PS	48	M	13	1	19.2	4.3	105	1.31	0.98	118.3	1.5	high	10.8	5	1.01	29	12.4
PS	66	F	13	1	18.9		210	0.89	0.97	111	2.1	high	8.5	7			8.7
PS	46	F	13	1	17.9	4.2	138	1.4	1.36	105.1	1.8	high	8.7	2.5	1.8	27	8.6
PS	50	M	13	1	19.8	3.9	152	1.42	1.18	120	1.8	high	8.4	6.4	0.46	26	11.9
PS	65	M	13	1	20.3	4.3	167	1.24	1.51	122.8	2.1	high	6.6	6.5	0.1	34	11.7
PS	66	F	12	1	18.3	3.8	103	1.31	1	135.9	2.1	high	8.6	3.3	1.4	21	9.9
PS	53	M	12	1	23.7	4.1	148	1.18	0.87	107.7	1	high		4	0.44	31	9.5
PS	41	M	11	1	27	4.3	151	1.27	0.91	90.36	1.8	high	9.7	7	0.26	25	12.3
PS	61	F	11	1	19	3.9	136	1.5	1.02	117.4	1.8	high		1.8	0.06		
PS	47	M	11	1	25.9	3.9	186	1.55	0.77	126.8	1.6	high	9.4	3	0.71	19	11.1
PS	51	M	9	1	17.5	3.8		1.05	1.03	118.9	1.8	high	10.1	7.1	0.32	31	10.7
PS	70	M	9	1	27.3		163	1.58	0.53	125.6	1.8	high	9.6	3.8	0.31	31	10.4
PS	50	M	8	1	21.2	4.1	137	1.58	1.49	112.4	1.5	high	7.9	7.5	0.01	24	10.8
PS	68	M	4	1	22.3	3.6	151	1.53	0.74	95.79	1.8	high	9.2	6.2			11.4
PS	60	M	7	1	24.1	4.3	179	1.46	1.06	120.1	1.5	high	8.7	6.3	0	29	13
PS	49	M	5	1	30	4.4	189	1.31	0.8	77.33	1.5	high	9.4	4.8	0.01	26	9.6
PS	74	F	5	1	21.8	3.6	212	1.72	1.1	107.2	1.5	high	9.8	5.1	0.05	34	10.5
PS	51	F	4	1	20.7	4.1	202	1.54	1.15	121.9	1.5	high	10.5	9	0.28	27	11.1
PS	59	F	4	1	28.4	3.9	167	1.19	0.87	127.2	1.3	high	10.4	2.8		23	8.1
PS	47	M	2	1	27.5	3.8		1.16	1.15	69.17	2.1	high	10.3	5.7	0.1		10.1

PS	62	M	11	1	18.3	3.6	133	1.55	0.8	134.9	2.1	high	7.8	4	0.77	53	10.8
PS	50	M	4	1	21.4	4.5	145	1.58	0.81	141.7	1.8	high	9.3	5.1	0.05	21	9.7
PS	65	M	2	1	22.2	3.7	140	1.06	1.07	78.98	1.8	high		5.8	0.4	30	11.2
PS	55	M	21	1	18.9	3.8	141	2.07	1.07	122	1	high	8.8	4.8	0	26	10.5
PS	57	M	2	1	21.4	4.2	89	1.27	0.92	67.03	1.8	high		6.1	0	26	11.4
PS	64	F	5	1	21.2	2.3	124	1.24	0.63	55.89	1.8	high	8	4.5	0.13	28	10.7
PS	58	M	2	1	27.5	2.3	126	1.43	1.39	96.17	1.5	high	8.9	5.5	0.06	41	11.2
PS	60	M	4	1	30.4	3.8	188	1.87	0.85	110.9	2.1	high	9.3	5.2	0.05		10.8
PS	56	F	12	1	23	4.2	161	1.87	0.73	117	1.6	high	9.2	4.7	1.06	35	8
PS	28	M	9	1	19.8	4.2	123	1.59	0.87	96.57	1.8	high	9.8	3.8	0	25	9.5
PS	76	M	8	1	25.7		190	1.58	0.97	99.03	1.9	high	9.8	5.4	0.05	15	10.2
PS	38	M	6	1	17.3		144	1.53	0.77	87.71	1.6	high	8.6	4.2	2.49	30	10.6
PS	76	F	6	1	19.1		169	1.5	0.79	96.38	1.6	high	9.7	4.8	0.05	19	10.7
PS	56	M	6	1	25.7		191	1.53	0.73	126	1.3	high	9	5.7	0.06	27	11.1
PS	50	M	6	1	19.1		156	1.78	0.85	116.1	1.6	high	9.6	4.5	0.05	28	11.2
PS	70	M	6	1	25.3		136	1.87	0.9	173.3	1.8	high		5.8	0.22	31	10.9
PS	59	F	5	1	22.3			1.74	0.72	96.2	2.1	high	7.7	3.2	1.13	38	7.7
PS	69	M	5	1	23.8		143	1.34	0.84	136.3	1.9	high	9.1	5.2	0.38	17	10.4
PS	57	M	5	1	26.6		200	1.94	0.8	52.09	2.1	high	8.7	5.1	0.01	28	9.7
PS	68	M	4	1	19.9		118	1.28	0.82	114.2	1.3	high	9.3	3	1.48	27	10
PS	55	M	4	1	16.2		219	1.27	0.97	111	2.1	high	9.8	5.1	0.08	20	10.1
PS	31	F	4	1	17.3		175	1.32	0.99	108	1.5	high	9.3	8	0.04	28	11
PS	59	F	29	1	18	3.8	118	1.78	0.84	95.82	1.5	high	9	3.9			11.7
PS	60	M	26	1	17.8	3.2	159	0.79	1.1	110.5	1.5	high		6.2		17	8.5
PS	49	F	19	1	21.2	4.2	165	1.81	0.98	90.4	2.1	high	9	5.3	0.01	26	10.4
PS	39	F	18	1	18.8	3.9	107	1.37	0.82	108.3	1.6	high	8.1	4	4.7	37	10.9
PS	65	F	14	1	26	3.2	168	1.66	1	118.7	1.8	high		4.3	0.16		
PS	54	F	14	1	20.4	3.6	111	1.87	0.82	115.3	1.5	high	8.8	4.7			11.5
PS	64	F	11	1	20.5	3.6	138	1.17	0.87	94.47	1.6	high	10.1	5.3	0.1	25	11.5
PS	71	F	8	1	17.8	4	148	1.04	0.99	119.9	1.5	high		6.7		27	9.9
PS	69	F	7	1	20.2	4.1	180	1.35	1.01	113	2.1	high	9.8	4.9	8.63	28	10
PS	70	M	7	1	20.7	4	146	1.24	0.79	106.4	1.5	high	8.6	5			9.4
PS	61	F	6	1	23.3	3.8	129	1.44	1.16	90.57	2.1	high	9.6	5.4	0.46		8.5
PS	69	F	4	1	22.5	3.5	175	1.48	0.75	141.5	1.6	high	9.3	10.3	0.15	23	10.1
PS	40	M	6	1	18.3	3.7	136	1.61	0.94	119.2	1.6	high		5.4	0.05	23	9.4
PS	85	F	5	1	19.7	3.6	178	1.16	0.9	119.7	2	high	8.5	5.9	2.7	25	9.3
PS	36	M	5	1	31.8	4.1	168	1.61	0.86	100.9	1.4	high	8.8	4.9	0.28	26	8.7
PS	73	F	5	1	15.5	4		1.83	0.9	79.46	1.3	high	9.1	5.1		30	10.1
PS	38	M	4	1	28.6	3.7	176	0.81	0.84	124	2.1	high	8.1	5.7	0.36	18	11.7
PS	44	M	3	1	18.2	3.5	180	1.47	1.02	56.93	2.1	high	8.6	6.1	0.1		11.1
PS	79	M	3	1	22.1	3.6	142	1.76	0.7	85.61	2.1	high	9.1	5.9	0.02		10.3
PS	68	M	3	1	23.3	3.5	196	0.86	0.69	82.6	2.1	high	7.9	5.7	1.9		11
PS	43	M	2	1	23	3.3	150	1.37	0.83	70.53	2.1	high	9.2	5.1	0.17		11.4
PS	66	M	2	1	18.8	3.7	165	1.45	0.65	31.69	2.1	high	9.3	2.9	0.02		10.8
PS	68	M	2	1	27.4	3.4	181	1.45	0.79	85.03	2.1	high	9.1	4.1	0.05		11.4
PS	84	F	2	1	21.4	4.5	151	1.62	1.08	102.8	1.5	high		7.3	0.61	27	10.2
PS	65	M	2	1	25.6	3.7	103	1.49	1.09	88.86	2.1	high	8	7.3	0.07	40	8.2
PS	59	F	5	1	21.4	3.7	177	1.7	0.87	103.9	1.9	high	9.3	5.5	0.05	23	11.4
PS	44	M	2	1	20.8	4.6	176	1.32	0.74	89.02	2.1	high		6.8	0	27	9.7
PS	58	M	31	1	22	3.9	188	1.39	0.94	69.08	1.5	high	8.5	4.5	0.07		10.9
PS	41	M	26	1	18.8	3.8	119	0.91	1.11		1.8	high	8	6.4	0.06	25	11.6
PS	63	M	24	1	19.1	4	163	1.74	1.29	107.2	1.6	high	7.8	5.7	0.4	29	9.3
PS	54	F	23	1	22.4	3.8	130	1.84	0.98	121.9	1.9	high	10.6	6.6	0.1	43	10.5
PS	53	M	21	1	24.1	4.2	150	1.33	0.94	106.2	1.9	high	9.5	3.9	0.1	28	9.6
PS	43	M	18	1	30.8	4.1	175	1.26	0.89	124.7	1.9	high	8.2	4.8	0	22	9.7
PS	60	M	15	1	24.8	3.8	110	1.37	0.8	125.6	1.9	high	9.2	4.7	0.1	29	7.6
PS	56	M	15	1	19.3	3.9	182	1.54	1.2	106.6	2	high	9.3	5.4	1.2		11.1
PS	58	M	14	1	19.3	4.3	119	0.97	0.95	117.7	1.8	high	7.7	4.7	0.06	24	12
PS	61	M	13	1	22.3	3.9	189	1.54	1	150.5	1.9	high	8.8	5.2	0.2	17	10.2

PS	64	M	13	1	20.6	3.5	132	1.54	1.55	114.8	1.3	high	9.9	5.7	0.05	29	8.9
PS	66	M	12	1	21	3		1.72	1.02	93.97	1.3	high	10.4	5.7		36	9.9
PS	66	F	12	1	21.1	4	182	1.68	1.26	127.2	1.5	high	7.7	3.8	0.06	39	10.4
PS	46	M	11	1	29.7	4.2	121	1.42	1.12	141.2	2.1	high	9.3	8	0.6	33	10.3
PS	58	M	11	1	19.8	4.2	163	1.65	0.98	105.8	1.2	high	8.3	7.3	0	28	11.8
PS	38	M	10	1	16.9	4.6	145	1.44	1.17	124.9	1.9	high	7.7	5.8	0.1	29	10.5
PS	70	F	9	1	21.6	4.1		1.44	0.93	97.95	1.6	high	9.7	4.6		30	9.9
PS	61	M	9	1	22.4	3.9	164	1.14	0.75	114.2	1.8	high	8.7	4.2	0.05		10.8
PS	54	M	7	1	22.1	3.5	168	1.79	0.75	104.3	1.8	high	9	4.8			10.3
PS	71	M	7	1	22.3	4	140	1.33	1.04	109.9	2.1	high	8.5	2.7	0.56		9.9
PS	43	M	6	1	21.8	4.2	132	1.58	0.75	87.68	1.5	high		3.9	0.55	35	10.5
PS	45	M	6	1	20.7	4.9		1.19	1.03	111.6	1.6	high	8.1	7.3	0.57	27	9.1
PS	87	F	5	1	21.4	3.6		1.8	0.99	106	1	high	9.9	3.8	0.74		8
PS	43	M	5	1	21	3.8	173	1.58	1.12	123.6	1.6	high	9.7	5.3	0.17	25	9.5
PS	71	M	5	1	19.9	4.1		1.25	0.75	65.78	1.6	high	9	5.4		35	12
PS	56	F	5	1	27	4.1		1.52	0.84	82.02	1.6	high	8.3	5.7		38	10.2
PS	55	F	4	1	22.4	3.8	79	1.38	0.88	72.18	1.8	high		5.9	0.05		12.3
PS	79	M	3	1	21.1	4.4	100	1.07	0.68	115.4	1.9	high	8	7.4	0	24	11.7
PS	60	F	2	1	21.2	3.7	138	0.94	0.93	98.93	1.2	high	8.2	5.5	0.1	20	11.1
PS	53	M	18	1	19.3		163	1.52	0.85	95.05	1.6	high	7.5	5.8	0.1	30	10.7
PS	78	M	11	1	22.3			1.76	0.74	126.3	1.3	high	9.6	4.4		25	9
PS	70	F	11	1	23.9		179	1.87	1.06	104.7	1.2	high	9.2	5.7	0.1	25	10.3
PS	28	M	10	1	18.7			1.85	1.11	113.6	1.6	high	8.7	4.5	0.72		10.2
PS	56	F	9	1	18.3		100	1.52	1.18	135.2	1.6	high	8.2	7.4	0.3	58	10
PS	58	M	9	1	19.4		165	1.7	0.87	122.6	1.5	high	8.6	7.2	0.2	26	11.2
PS	66	F	8	1	18.3		211	1.26	1	79.52	1.6	high	7.7	2.8	0.2	12	10.1
PS	60	F	8	1	20.4		158	1.84	0.72	107.9	1.5	high	9.9	5.8	0	26	9.4
PS	76	M	8	1	21.1		138	1.38	1.03	89.14	2.1	high	9.1	4.6	0.09		10.6
PS	57	F	7	1	17.6		182	0.81	1.16	116.2	1.5	high	9.9	6.3	0.07	22	12.5
PS	60	M	7	1	23.1	3.9		0.94	0.78	111.5	1.8	high		5.9	0.09	32	9.8
PS	35	M	7	1	20.7		130	1.31	0.89	123.6	2.1	high	8.6	5.7		20	10.9
PS	75	M	6	1	22.6		120	1.82	0.7	131.3	1.8	high		4.7	2.8	51	8.9
PS	61	M	6	1	21.6		156	1.44	1.05	103.4	2.1	high	9	5.4	0.05		10.7
PS	68	F	6	1	26.9		237	1.34	0.83	114.2	1.6	high	8.3	6.9	0	30	9.5
PS	40	F	6	1	19.2		148	1.79	1.03	97.34	2.5	high	10.2	5.3		29	11.1
PS	61	M	6	1	20.2		185	1.95	0.73	68.57	1.5	high	11.1	7.5	0.77	26	12.6
PS	69	F	5	1	17.5		148	1.31	0.96	111	1.5	high		3.3	0.86	33	9.9
PS	71	F	5	1	16.6		184	1.35	1.26	124.3	1.9	high	8.3	3.9	0.1	30	10.2
PS	82	F	4	1	24.3		117	1.35	0.64	110	2.1	high	8.9	5.6	8.4	30	11.4
PS	39	M	2	1	25.1			1.59	0.56	108.8	1.5	high	10.4	3.6	0.23	27	9.4
PS	57	M	2	1	24.8		131	1.36	0.98	115.4	1.8	high	9.5	5.8	0.11	28	11.1
PS	62	F	27	1	19.3	3.6	150	1.24	0.76	122	1.4	high	9.3	5		22	9.6
PS	63	F	25	1	18.2	4.2	117	1.71	1.1	106.3	2.2	high		4.3	0.24	34	10
PS	47	F	23	1	16.7	4.2	248	2.06	0.97	114.1	1.4	high		4.8	0.05	26	12.1
PS	68	F	20	1	18	3.5	140	1.64	0.62	91.08	1.6	high	8.5	5.6	0.07	20	8.8
PS	56	F	8	1	21.4	3.7	100	1.29	0.98	102.9	1.6	high	8.4	4.8	0.04	25	10.2
PS	74	M	7	1	22.8	3.9	121	1.77	0.7	119.1	2.5	high	8.7	4.3		33	9.9
PS	37	F	5	1	19.5	4.1	117	1.12	0.8	105.3	1.8	high	8.7	6.1	0.74	29	10.1
PS	71	M	5	1	27	3.8	142	1.53	0.93	109.3	2.1	high	8.8	4.1		37	10
PS	65	F	5	1	19.8	3.5	159	1.31	0.66	63.16	2.1	high	9.2	5.6	0.16	22	8.9
PS	54	F	5	1	22.2	3.7	123	1.58	0.8	129.3	1.5	high	9.7	4.5	0.25	23	10.8
PS	54	M	5	1	22.6	3.8	164	1.17	0.88	99.94	1.5	high	9.9	5.2	0.09	17	11.3
PS	61	M	4	1	25.2	3.7	137	1.74	0.81	109.2	1.5	high		5.2	0.2	24	10.9
PS	38	M	4	1	20.2	4.2	85	1.69	0.71	57.68	1.5	high	8.3	4.7	0.66	47	8.9
PS	79	M	3	1	15.9	3.1	213	1.99	0.87	61.14	1.8	high	9.8	4.9			9.3
PS	67	M	3	1	23.1	3.6	201	1.45	0.74	91.57	2.1	high	7.9	3.3	0.04	29	9.6
PS	48	M	3	1	22.9	4	130	1.64	0.81	95.94	2.1	high	9.2	6.4			10.8
PS	76	F	2	1	14.1	3.3		1.68	0.81	107.7	2	high	8.2	3.4			10
PS	62	F	2	1	21	3.7	150	1.92	0.7	69.19	2.1	high	8.6	6.9		27	12

PS	72	M	2	1	19.2	3.7	109	1.72	0.95	107	1.5	high	9.8	4.3	4	29	
PS	62	M	2	1	22.3	3.3	168	1.34	0.74	81.56	2.1	high	8.8	5.2			10.8
PS	81	F	2	1	22.9	4	248	2.03	0.68	101.7	1.5	high	9.2	3.8	0.12	29	9.4
PS	56	M	7	1	27.3	3.9	154	1.9	0.89	130.9	2.1	high	9.9	4.4	0.07		11.3
PS	71	M	4	1	18.9	3.5	180	0.94	1.14	84.84	1.3	high	8.6	7.1	0.55	35	11.5
PS	59	M	30	1	22.2	3.3	233	1.45	1.1	122.4	1.8	high	10.3	3.4	1.3		10.6
PS	51	M	30	1	20.9	3.9	148	2.89	0.85	103.1	2	high	9.7	5.9			10.5
PS	60	F	30	1	19.3	3.4	209	1.39	0.8	102.4	1.3	high		6.1	0.05		10.1
PS	74	M	23	1	22.4	3.7	121	1.24	1.12	125.6	1.9	high	8.3	4.1	0.1	28	9.5
PS	68	F	20	1	19.7	3.9	155	1.55	0.88	107.9	1.5	high		3.9	0.04	30	8.7
PS	71	M	19	1	19.8	3.8	139	1.24	0.97	84.63	1.8	high		4.7	1.82	26	
PS	75	M	18	1	18.7	2.7		1.39	1.03	123.1	1.6	high	9.1	5.6	0.04	29	10.4
PS	58	F	16	1	19.9	3.6	197	1.34	1.01	123.1	1.9	high	9.4	6.3	0.1	24	10.3
PS	50	M	13	1	22	3.8	148	1.41	1.06	113.8	1.5	high	8.7	5.9	3.75		12.6
PS	58	M	10	1	20.1	3.9	138	1.18	0.91	116	1.8	high	8.1	6.3	0.1		9.7
PS	60	M	9	1	22.3	3.6	124	1.35	1.05	132.8	1.6	high	8.9	6.8	0.01	23	12
PS	65	M	7	1	23.6	3.8	120	1.62	1.05	136.5	2.1	high	9.4	5.9		32	9.9
PS	61	M	6	1	25.4	3.8	170	1.58	0.81	121.3	1.8	high		6.2	0.1	30	10.8
PS	37	M	5	1	25.8	3.9	122	1.56	1.15	113.2	2	high	9	7.2		24	11.3
PS	28	F	5	1	24.3	3.6	144	1.15	1.54	102	1.3	high	8.8	5.2	0.1		10.3
PS	70	M	4	1	21.1	3.5	123	1.24	0.68	77.49	2.1	high		5.5	0.14	33	12.4
PS	58	F	4	1	26.1	3.8	153	1.47	0.68	78.56	1.5	high	8.1	5	0.05	34	10.2
PS	56	M	4	1	20.3	3.5	190	1.98	0.8	97.97	1.1	high	8.7	4.4	1.87	26	9
PS	73	F	2	1	21.4	3.5		1.34	1.32	150.5	1.6	high	8.7	5		24	9.9
PS	79	M	4	1	21.2	3.9	174	1.25	0.98	139.2	2.1	high	8.7	6	0	21	10.8
PS	72	M	5	1	21.7	3.5	120	1.25	1.02	113.6	2.1	high		6.5	0.05	25	9.9
PS	75	M	6	1	25.4	3.7	168	1.49	0.96	127.5	1.1	high	8.5	5.6	0.28	31	11.3
PS	56	F	10	1	20.6	4		1.51	0.84	113.9	1.6	high	11.3	4.2		33	9.9
PS	69	M	22	1	18.4	3.4	113	1.53	0.88	97.01	1.8	high	10	6.3	0.07	40	11
PS	65	F	3	1	17.9	3.5	199	0.93	0.8	77.31	1.6	high	8.9	6.6	0.05	22	9.9
PS	66	F	12	1	19	3.4	122	1.32	0.75	58.63	1.8	high	8.4	6.8		22	11
PS	70	F	9	1	24.3	3.6	113	1.24	0.94	116.1	1.8	high	9.8	9.1	0.09		11
PS	53	M	6	1	23.8	3.8	104	1.44	0.9	86.98	1.1	high	9.7	6.4	0.43	24	9.5
PS	63	M	5	1	22.9	4.2	139	1.35	0.89	113.4	2.1	high	9.7	4.8	0.13		9.3
PS	78	F	3	1	18.2	3.6	167	1.19	0.84	79.5	1.5	high	8.8	5.1	0.05	28	10
PS	59	M	4	1	18.3	4.5	68	1.34	0.91	76.82	2.1	high	9	6.2	0.1		10.5
PS	40	F	10	1	20	3.6	121	1.43	1.07	138.9	1.5	high	9.8	5.8	0.11	28	10.8
PS	58	F	8	1	22.5	4.6	165	1.23	0.91	108.8	1.8	high		7.4	0.1	31	12.2
PS	71	M	14	1	21.3	2.8		1.1	0.65	77.79	2.1	high	8.2	5.2	3.08	41	11.4
PS	51	M	5	1	23.3	3.7	146	1.06	0.73	142.4	1.8	high	9.8	5.1	0.55	28	10.7
PS	66	M	11	1	20	3.7	117	1.68	0.81	90.63	1.8	high		5.2	0	31	9.8
PS	48	F	7	1	25.6	3.2	130	1.42	0.79	102.7	1.8	high	9.6	4.3	0.28		9.9
PS	72	F	3	1	22.7	3.8	147	1.41	0.84	129.6	2.5	high	8.2	6		25	13.1
PS	44	M	3	1	22.8	4.5	194	1.47	1.2	102.4	2.1	high	9.6	5.2	0.02	26	10.2
PS	77	F	2	1	23.3	3.7		1.32	0.61	66.01	1.3	high	8.7	4.2		22	11.3
PS	52	M	24	1	20.5	4	134	1.07	1.14	93.06	1.1	high	8.3	6.4	0.06	24	10.9
PS	48	M	4	1	24.4	3.4	161	1.53	0.96	121.5	1.6	high	8.5	4.1	0.1	33	10.6
PS	51	F	28	1	22.1	3	183	1.68	0.98	118.1	1.4	high	8.8	5.3	0.2		9.6
PS	61	M	10	1	16.3	4.1	139	1.21	0.68	80.73	1.6	high	9.3	7.2	0.3	45	11.2
PS	61	M	38	1	20.2	3.9	149	1.26	0.96	121.2	1.6	high	10	7.2	0.01	28	11
PS	55	M	35	1	17.2	2.8	156	1.61	1.08	74.46	2.1	high	9.7	5.7		27	9.7
PS	65	M	31	1	22.5	3.7	152	1.66	0.96	76.44	1.6	high	8	3.9	0.02	22	10.9
PS	54	F	30	1	20.2	3.7	149	2.17	0.96	98.02	1.2	high	7	3.9	0	31	10.5
PS	71	M	30	1	21.3	4.2	135	1.49	1.22	109.1	1.6	high	9.1	4.8	0.05	30	11.5
PS	49	M	28	1	22.3	4.2	162	1.53	0.92	91.21	2.1	high	9	7.1		31	9.7
PS	65	M	35	1	18.2		124	1.37	1.16	102.1	1.6	high	7.6	6.3	0.1	30	10
PS	60	M	23	1	18.6	3.8	119	1.34	1.05	118.4	1.5	high	9.2	3	1.04		8.9
PS	60	M	23	1	18.5	4	118	1.71	0.86	91.16	1.6	high	9.6	7.1	0.07	24	11.2
PS	56	M	22	1	22.2	3.6	131	1.79	0.82	102.1	1.6	high	9.2	8.3	0.1	39	10.9

PS	48	M	21	1	19	4.3	146	1.57	1.14	113.2	2.1	high	8.6	6.6		26	9.4
PS	51	M	20	1	22.7	4	125	0.95	1.18	114.1	1	high	9.7	4.7		32	11
PS	46	F	19	1	18.9	4.1	132	1.13	0.84	90.52	1.8	high	8.2	4.1	1.42	16	10.9
PS	58	F	16	1	22.5	4	205	1.88	1.28	143.4	1.6	high	9.2	6.9	0	30	9.5
PS	40	M	13	1	21.2	4.1	201	1.76	1.01	109.1	1.8	high	9	5.4	0	25	9.7
PS	50	M	13	1	19.9	4.8	169	1.27	0.97	102	1.5	high	8	5.5	0.2	30	10.1
PS	70	M	12	1	25	3.5	158	1.92	0.89	123.8	1.9	high	9.6	4.2	0.05	19	12
PS	53	F	12	1	19	4	163	1.74	1.23	120.2	1.8	high	8.3	7.3	0.1	34	7.4
PS	60	F	11	1	19.9	3.9	222	1.74	1.01	110.4	1.8	high	9.5	4.5	0.04		12.3
PS	43	M	10	1	21.9	4	116	0.86	0.93	116.5	1.6	high	8.3	6.1	0.04	30	10.4
PS	59	M	10	1	22.7	4.1	114	1.69	0.94	117.5	1.5	high	9	5.6	0	26	12.8
PS	64	M	10	1	18.6	3.9	131	1.27	0.96	111.9	1.8	high		4.6	0	29	10.2
PS	53	M	9	1	18.1	4	176	1.72	0.95	105.2	1.8	high	8.8	7	0.28		11.1
PS	66	M	8	1	20.3	4.3	138	1.83	0.96	121.1	1.9	high	8.9	4.5	0.05	35	10
PS	71	F	8	1	20.5	3.7	188	1.63	1.24	127.8	1.5	high	9.3	7.4	0.2	28	10.4
PS	41	F	8	1	19.3	4	197	2.01	1.14	134.5	1.5	high	7.7	4	0.06	35	10.8
PS	67	M	7	1	25	3.3	148	1.6	0.67	91.06	1.8	high	8.3	2.9			9.9
PS	61	F	7	1	28.7	3.7	97	1.43	1.2	86.53	1.3	high	8.9	5.3		23	10.6
PS	44	M	5	1	25.3	4.1	146	2.05	0.79	107.2	2.1	high	9.9	7.1	0.1	19	10.9
PS	66	F	5	1	27.6	3.4		0.98	0.62	51.35	1.8	high	9.5	5.4		14	10.1
PS	43	M	5	1	24.9	3.7	94	1.34	0.96	96.36	2.1	high	10.4	4.8	1.37	23	9.9
PS	67	M	5	1	22.5	3.5	184	1.27	0.89	74.04	1	high	8.8	0.5	0	17	10.4
PS	58	M	4	1	27.8	4	187	1.42	0.86	105	1.8	high		6.1	0	25	12.1
PS	22	M	3	1	18.3	4.8	161	1.2	1.07	110.5	1.8	high	11.7	4.7	0.04		11.2
PS	33	M	2	1	27.5	4.3	205	1.39	1.28	92.76	1.8	high	8	4.9	0.04		9.8
PS	42	M	2	1	19.9	4.1	182	1.64	0.78	119.9	1.8	high	9.6	2.7	0.07		10.8
PS	59	M	2	1	25.9	4.3	128	1.35	1.14	73.27	1.8	high	9.4	6.1	0.11		11.9
PS	64	M	2	1	23.3	4	144	1.44	0.94	131.3	1.9	high	8.8	4.7	0.07	29	11.6
PS	69	F	30	1	19.2	4		1.25	1.18	130.8	1.6	high	9.9	5.9		23	9.1
PS	44	M	12	1	25.1	4.1	155	1.38	1.06	129.6	1.6	high	7.5	10	0.47	16	10.9
PS	38	M	12	1	20.1	4	151	1.49	0.75	103.6	1.8	high	9.2	4.9	0.09	22	11.1
PS	45	M	10	1	25.4	3.9	141	1.31	0.9	128.6	2.1	high	8.5	5.7	0.25	28	12
PS	64	M	9	1	18.7	4	145	1.76	1.03	128.4	1.8	high		3.3	0.06	24	9
PS	55	M	8	1	23.8	3.4	130	1.15	0.91	108.5	1.9	high	8.4	6.5	0.72	26	10.4
PS	58	F	7	1	18.7	3.7	128	1.12	0.74	115.3	1.3	high	9.2	5.7	0.72	30	10.1
PS	85	M	6	1	23.3	3.9		1.3	0.77	151.6	1.5	high	8.4	3.7	0.22	20	10.5
PS	45	M	4	1	32.1	3.8	141	1.48	0.96	99.84	1.5	high	9	7.4	0.6	24	7.9
PS	82	F	3	1	15.2	3.4	148	0.95	0.71	68.89	2.1	high	8.9	7.1	0.05	30	11.1
PS	53	F	11	1	19.4	4.4	117	1.22	0.97	120.7	1.6	high	8.6	6.2	0.26	23	9.9
PS	66	M	11	1	21.8	3.6	116	1.3	1	101.8	1.8	high		5.8	0.6	28	11.2
PS	53	M	6	1	20.9	4.6	152	1.26	0.96	113.4	1.9	high	7.7	7.8	0.14	37	10.6
PS	52	M	3	1	22.4	4.3	106	1.22	0.9	89.55	1.8	high	8.9	5	0.12		10.5
PS	43	M	2	1	23.2	4	162	1.55	0.93	100.6	1.3	high	8.8	4.3	0.04	28	9.2
PS	65	F	19	1	16.6	3.9	182	1.34	1.2	126.5	1.6	high	9.4	5.6	0.02	33	9.3
PS	55	F	9	1	18.2	4.1	114	1.14	1.33	89.3	1.5	high	9.5	5.5	0.29	20	12.8
PS	42	M	4	1	26.7	4.3	130	1.69	0.77	136.3	1.6	high	8.5	6.4		22	9.7
PS	62	F	2	1	31.4	4.2	166	1.43	0.6	77.69	1.3	high	7.7	1.6	0.08	22	7.7
PS	47	M	5	1	19.8	4.1	113	1.06	0.86	99.3	2.1	high	7.7	4.4	0.91	25	10.9
PS	80	M	2	1	20.3	3.6	161	1.45	0.93	107.8	1.8	high	9.7	4.6	0.3	29	10.9
PS	66	M	6	1	21.7	3.9	169	0.97	0.69	130.5	1.3	high	9.6	6.1	0.05	19	9.7
PS	62	M	7	1	23.5	3.9	189	1.73	1.45	64.19	2.1	high	9.3	4.7	0.74	21	10
PS	44	F	12	1	24.8	3	139	1.49	0.75	81.43	1.8	high	8.9	4.1	0.18	34	11.5
PS	74	M	11	1	16.9	4.1	175	1.68	1.2	120.1	2.1	high	9.1	5.4		23	9.5
PS	71	F	10	1	15.8	3.5		0.89	0.91	105.5		high	8.3	7.8			10.2
PS	56	M	9	1	19.5	3	116	1.47	1.13	73.42	2.1	high	9.9	5.3		27	10.7
PS	57	M	8	1	17.4	4	225	0.98	1.1	104.3	1.8	high	9.1	7	0.16		12.4
PS	67	M	8	1	16.8	3.1	124	1.78	0.7	63.8	1.5	high		4.3	4.81		8.3
PS	60	F	8	1	29	3.4	108	0.98	0.92	120.4	1.5	high	10	8.1		26	10.9
PS	72	F	4	1	19.1	3.8	154	1.31	1.1	147.4	1.8	high	8.8	5.3	5.04	46	11.1

PS	72	M	2	1	22.7	3.8	102	1.07	0.89	94.93	1	high	8.2	3	0.49	30	11.2
PS	61	M	2	1	16	3.5	146	1.58	0.92	83.81	1.6	high	8.9	4.6	2.3	33	9.3
PS	67	F	5	1	21.3	3.8		1.38	1	121.8	1.8	high	8.6	4	1.8	27	10.5
PS	69	M	2	1	21.6	3.7		1.35	0.87	87.89	1.5	high	10.3	7		31	9.2
PS	71	M	3	1	22.4	3.7	190	1.07	0.93	92.95	1.8	high	9.2	5.5			10.1
PS	70	M	14	1	16.5	3.4	158	1.26	0.86	111.9	1	high	9.3	5.2		34	10.9
PS	56	M	5	1	18.5	4.3	149	1.14	0.95	86.68	2.1	high	9.9	7.5	0.12		11.6
PS	52	M	4	1	29.5	4	179	1.61	0.71	73.03	1.8	high	8.9	6.1	0.12	27	10.9
PS	66	M	5	1	21.3	4	107	1.61	1.09	137.4	1.4	high	9.6	4.6	0.11	34	9.5
PS	84	M	8	1	18.1	3.4	158	2.03	1.21	91.87	1.4	high		6.1	0.32	33	11.2
PS	57	F	4	1	20.8	4.1	194	1.52	1.14	140.4	1.5	high	8.7	5.1	3.62	32	13.5
PS	71	F	3	1	20.8	3.7	149	1.37	1.05	94.65	2.1	high	9.4	4.2			11.1
PS	69	M	8	1	19.6	4.1	209	1.1	0.86	98.07	1.5	high		7.5	0.4	27	10
PS	55	F	3	1	19.4	4.3	136	1.87	0.63	74.26	1.6	high	9.6	6.1	0.05	27	9.7
PS	73	M	25	1	17	3.7	184	1.35	0.9	85.07	1.6	high	9.9	4.4	0.8	25	9.8
PS	52	M	24	1	16.3	3.8	120	1.07	1.1	105.6	2.1	high	9.3	6.2		19	10.4
PS	73	M	11	1	21.6	3.7	136	1.28	0.91	99.27	2.5	high	8.7	4.3		38	8.9
PS	51	M	10	1	23.6	4.3	126	1.24	1.12	104	2.1	high	8.9	4.6	0.21	25	10.2
PS	65	M	8	1	20.3	3.8	131	1.24	1.3	120.4	1.8	high		5.9	0.02	29	8.9
PS	50	F	8	1	19.1	4.1	199	1.77	1.42	105.4	2.1	high	9.7	4.3		29	9.3
PS	58	M	8	1	35.1	4.1	143	1.35	0.65	102.2	2.1	high	8.8	6.2	0.02		11.5
PS	73	F	7	1	18	3.7	151	1.42	0.88	129.8	2.2	high	9	5.2	0	21	9.7
PS	46	M	6	1	21	4	128	1.41	0.93	132.6	2.1	high	7.4	5.9	0.28	32	10.2
PS	66	M	6	1	29.5	3.9	113	0.89	0.93	87.59	2.1	high	8.3	5.7	0.11		10.1
PS	66	M	5	1	19.1	4		1.55	0.72		1.1	high	8.7	6		26	10.5
PS	75	M	4	1	21.9	3.9	133	1.87	0.71	115.4	1.1	high	8.9	4	0.49	25	10.2
PS	53	M	2	1	23.4	3.9	127	1.35	0.92	99.75	1.8	high	8.7	4.3			12.4
PS	80	F	2	1	24.2	3.9	130	1.48	0.75	48.46	1.8	high	8.9	4.1	0.02	24	11.3
PS	49	M	2	1	18.8	3.9	103	1.47	0.79	79.27	1.8	high	9.2	4.6			10.2
PS	63	M	2	1	18.4	6.6	190	1.21	0.7	52.23	2.1	high	7.3	6.6	0.1	30	11.9
PS	56	M	21	1	19.4	4.1	192	1.69	1.18	123.5	1.5	high	9.5	4.2	0	20	10
PS	40	M	13	1	22.5	4.2		1.39	0.96	87.65	1.6	high	8.7	5.1	0.08	3	9.4
PS	61	F	15	1	26.6	3.9	216	1.81	0.57	99.1	1.5	high	8.6	4.3	0.05	28	9.5
PS	63	M	8	1	17.8	4.1	172	1.86	0.98	127.3	1.5	high	9.9	5.6	0.09	40	11.3
PS	58	M	3	1	22	4.2		1.54	0.97	103.3	2.1	high	8.3	4.8	0.1	24	12.8
PS	55	M	26	1	23.5	2.8	140	1.26	0.67	70.99	2.1	high	8.3	7.8	0.05	35	13.4
PS	48	M	26	1	17	2.8	117	1.26	0.88	94.17	1.6	high	8.6	8	0.08	25	10.1
PS	59	F	20	1	28.1	4.2	175	1.54	1.01	124.5	1.5	high	8.8	7		28	10.6
PS	53	M	15	1	23.6	4.1	152	2.02	1.08	5.12	1.6	high	9.8	3.7		27	10
PS	29	F	7	1	15.6	4	201	1.63	0.92	101.7	1.8	high	8.4	5.7	0	26	10.7
PS	42	M	5	1	28.9	3.6	114	1.44	1.03	98.01	1.6	high	7.8	1.8	1.25		8.4
PS	33	F	3	1	16.1	3.4	104	1.45	1.04	49.58	2.1	high	9.3	4.5	0.13	25	9.4
PS	69	M	2	1	19.6	3.5	198	0.88	0.82	59.28	1.3	high	9.9	5.6	1.3	29	10
PS	58	M	7	1	21.1	3.8	117	0.85	1.29	136.5	1.5	high	10.3	6		19	11.7
PS	63	M	11	1	25.6	4.4	194	1.91	0.96	125.1	1.3	high	8.6	3.9	0.05	14	11.8
PS	34	F	9	1	19.1	4	235	1.25	1.13	101	2.2	high	8	7.4	0.09	33	11.3
PS	75	M	8	1	20.9	3.6	144	1.63	0.82	99.78	1.8	high	9.8	6.5	0.08	32	11
PS	61	F	6	1	22.9	4	134	1.04	1.04	105.4	1.3	high	8	6.2	0.72	45	10.4
PS	47	F	6	1	19.2	2.9	136	1.41	0.77	105.5	1.5	high	8.2	5.1	0.02	27	10.9
PS	41	M	4	1	22.4	3.6	158	1.26	0.78	64.1	2.1	high	9.3	3.9	0.14	28	10
PS	71	M	3	1	24.2	3.8	143	1.38	0.76	83.87	1.9	high	9.4	2.8		25	11.5
PS	58	F	2	1	19.2	4.1		1.59	1.01	100.1	2.1	high	9.7	5.5	3.54	20	10.1
PS	63	M	7	1	22.1	3.6	162	0.98	0.89	93.18	1.3	high		9.8	0.19		12.1
PS	59	M	4	1	23.8	2.6	183	1.61	0.89	72.06	1.8	high	7.2	4.6			8.3
PS	65	M	3	1	16.8	3.7	133	1.54	0.99	89.24	2.1	high	10.4	6.2		23	9.4
PS	68	M	27	1	19.3	3.7	183	1.22	0.91	109.1	1.6	high	9.1	7.8		33	11.3
PS	63	M	24	1	17.4	3.6	117	1.6	1.22	103.1	1	high	10.7	5.3	0.05	27	11.8
PS	66	M	23	1	21.9	3.5	180	1.02	0.85	107.6	1.9	high	9.3	4.5		14	12.2
PS	43	M	21	1	21.4	4.3		1.57	0.94	84.55	2.1	high	9	5	1.29		12.1

PS	47	F	17	1	19.9	4.1	138	1.24	1.12	119.5	2.1	high	8	5.2		35	10.8
PS	53	F	17	1	15.3	4.5	216	0.85	1.14	104.5	1.3	high	10	4.2	0.12	12	11.7
PS	59	M	15	1	18.6	3.8	91	1.54	1.07	104.3	2.1	high	10	5.7	13		9.5
PS	60	F	14	1	21.2	3.4	94	1.29	0.67	104.5	2.1	high	9.2	4.1		30	12.1
PS	59	M	14	1	20	4.1	135	1.2	1.12	132.6	1.8	high	9.3	6.3	0.01	27	9.5
PS	44	M	13	1	21.5	3.8	175	1.14	0.9	116.7	1.8	high	8.2	6.3	0.39	21	11
PS	62	M	13	1	26.2	3.9	152	1.37	0.94	117.6	1.8	high	9.7	6.2	0.08		13.2
PS	74	M	12	1	24.2	3.5	164	1.23	0.88	82.39	1.8	high	9.7	5.3	0.61	33	11.7
PS	63	F	12	1	21.4	4.2	154	1.54	1.13	116.5	1.5	high	8.6	3.7	0.01	33	10.9
PS	77	M	12	1	20.7	3.5	148	1.15	0.55	95.57	2.1	high	9.4	5.4	0.15	27	9.6
PS	72	F	12	1	24.7	4.2	125	1.08	1.25	124.5	1.9	high	8.9	3.2		31	9.5
PS	67	M	11	1	17.3	4.3	122	1.15	1.53	98.27	1.5	high	8.3	4.8	0.41	25	11.8
PS	63	M	11	1	21	3.9	199	1.5	0.97	111.8	1.1	high	9.4	4.2	0.05	28	11
PS	42	M	10	1	23.7	4.1	155	1.63	0.94	89.74	1.8	high	9.1	2.2	0.09	29	10.8
PS	42	M	10	1	17.7	4.2	249	1.4	0.81	107.4	1.5	high	10.3	6.1	0.23	30	10.2
PS	58	F	10	1	19.1	3.9	186	1.25	0.92	112.8	1.6	high	9.9	5.1		29	12.2
PS	45	M	9	1	22.1	3.9	236	1.23	0.98	126.6	1.5	high	8.8	3	0.15	27	10.8
PS	67	F	9	1	25.2	3.8	134	1.13	0.97	120.3	1.8	high	9.3	9.9	0.08	36	9.8
PS	50	M	9	1	29	4	145	1.28	0.93	121.9	1.8	high	9	6.3	0.09	34	10.2
PS	65	M	9	1	23	3.8	109	1.39	1.37	129.4	1.8	high	8.4	5.6	0.12	35	10.4
PS	58	F	9	1	18.8	3.8		0.66	1.02	111.1	2.1	high		4.8	0.6		10.5
PS	61	M	8	1	23.8	3.8	200	0.96	0.96	125.1	1.8	high	8.3	4.4	0.04	21	10.5
PS	74	M	8	1	16.3	4	155	1.21	0.84	84.09	2.1	high	8.8	7.2	0.2	29	12
PS	54	M	8	1	20	4.1	115	1.35	0.96	103.3	2.1	high	8.8	4	0.48	27	10.4
PS	51	M	7	1	21.5	4	170	1.44	0.88		1.9	high		8.3		17	12.2
PS	70	F	7	1	23.8	3.9	132	1.58	1	131.1	1.5	high	8.8	4.4	1.01	35	10
PS	78	F	7	1	22.4	4	191	1.65	0.75	126.3	1.9	high	9.1	7.6		36	11.3
PS	58	M	6	1	38.9	4	114	1.07	0.8	79.52	2.1	high	8.1	6	0.29	25	10.4
PS	64	M	6	1	21.2	3.9	149	1.23	0.95	121.2	1.6	high	8.2	4.2	2.51	47	9.8
PS	59	M	6	1	22.5	4	178	1.55	1.08	125.6	1.5	high	9	6.3		31	11.9
PS	43	M	6	1	27.8	4	98	1.58	1.07	146.2	1.6	high	10.3	5.1		30	11.4
PS	41	M	6	1	23.6	3.9	170	1.5	0.83	96.64	1.8	high	10	5.4	0.39	28	10
PS	50	M	6	1	19.2	3.6		1.43	0.98	116	2.5	high	9.1	4.5		28	9.1
PS	65	M	5	1	25.1	4.4		1.5	0.97	122.5	1.5	high	7.5	4.5		12	12.7
PS	78	M	4	1	26.3	3.7	106	1.04	0.85	90.3	2.1	high	8.4	6.2	0.03	56	8.5
PS	42	M	4	1	24.9	4.1	144	1.49	1	96.05	2.5	high	8.2	6.2		45	11.3
PS	74	M	4	1	25.8	4.1	144	1.19	0.77	75.52	1.8	high		8.1	0.1	22	10.4
PS	55	M	4	1	25.8	3.7	97	1.19	0.71	90.55	2.1	high	9.9	5.2	0.1	24	11.9
PS	66	M	3	1	20.3	3.7	156	1.46	1.01	80.74	1.6	high	8.5	4.2		27	10
PS	80	M	3	1	20.8	4.1	113	1.2	1.11		1.8	high	9.1	4.2	1.56	35	9.9
PS	67	M	3	1	20.4	3.7	87	1.61	0.73	69.54	2	high	8.5	3.1	1.09	39	5.4
PS	62	M	3	1	26.7	4	176	1.7	0.74	79.13	1.6	high	8.3	4.8	0.06	30	10.7
PS	77	F	3	1	18.6	3.7	141	1.44	0.73	112.7	2.1	high	9	6.2	0.1	16	11.8
PS	36	M	3	1	23.1	4.4	148	1.47	0.92	96.71	1.8	high		6.2	0.03	28	12.9
PS	65	M	3	1	24	3.9	159	1.63	0.88	78.41	1.3	high	8.3	3.9	0.19	28	10.4
PS	84	M	3	1	25.1	3.5	137	1.41	0.89	101.7	1.8	high	9.4	7.3			
PS	38	M	2	1	21.7	4.3	159	1.45	0.76	85.65	2.1	high	8.6	5.4	0.1	29	10.9
PS	56	M	2	1	19.5	3.9	179	2.06	0.96	103.5	2.1	high	9.2	4.1	1.8	60	9.1
PS	65	M	2	1	23.2	4	126	0.97	0.86	62.31	2.1	high	9.3	6.8	1.23		10.4
PS	42	F	2	1	23.5	4	79	1.33	0.78	141.9	2.1	high	8.1	3		21	9.4
PS	76	F	2	1	22.8	3.9	115	1.34	0.71	46.37	2.5	high	8.4	4.6		30	9.3
PS	94	M	2	1	21	3.8	178	1.6	0.7	66.02	1.5	high		4.6	1.2	27	12
PS	40	M	2	1	37.9	4	171	1.53	0.76	37.5	2.1	high	9.4	5.2	0.03	32	12
PS	64	M	2	1	20.7	3.5	186	2.47	1.4	105.8	2.1	high	9.1	3.7		18	11.6
PS	70	M	7	1	21.2	3.6	99	1.87	0.79	105.3	1.6	high	9.1	4.2	0.01	30	10.1
PS	67	M	18	1	18.9	3.7	98	1.35	0.9	88.9	1.8	high	8.3	5.3	0.22	30	10.4
PS	58	F	21	1	17.7	4.1	220	1.81	1.17	101.1	1.3	high	9.4	5.4	0.03	32	9.8
PS	72	M	4	1	26.7	4	111	1.27	0.87	132.8	2.1	high	9.5	5.9		21	10.6
PS	70	M	3	1	25.6	4.3	150	0.95	0.85	97.32	2.1	high	8.7	6.4	0.25	25	9.2

PS	55	F	10	1	19	4.1	157	0.97	1.37	118.9	2.1	high	9.3	7.1	0.24	36	13
PS	59	M	19	1	23.1	4	161	1.22	0.87	127	1.8	high	10	4.5	0.29	30	10.4
PS	65	M	2	1	23.6	4.2	186	1.11	0.99	100.6	1.3	high	8.6	7.2		27	11.5
PS	61	M	6	1	26.9	3.5	185	1.44	0.83	91.05	1.3	high	8.4	4.8	0.33	25	10.1
PS	60	F	9	1	20.7	3.7	208	1.35	0.72	87.55	1.5	high		9.3	0.1	25	10.4
PS	66	M	2	1	22	4.1	197	1.66	0.97	99.39	2.1	high	9.6	3.7	0.08	35	9.7
PS	59	M	2	1	22.7	4.2	185	1.88	0.83	71.29	1.5	high	8.4	6.1	0.5		10.5
PS	61	F	2	1	22.7	4	222	1.11	0.87	114.8	1.5	high	9.4	5.8	0.18	26	10.3
PS	58	F	7	1	16.8	4.1	143	1.7	1.21	102.1	1.5	high	9.7	4.8	0.11	24	8.4
PS	64	M	6	1	19.8	4	124	1.58	0.97	67.91	2.1	high	8.1	4.6	0.37	25	11.2
PS	73	M	10	1	22.9	3.4	207	2.12	1.13	170.9	2	high	9.5	4.6		22	10.9
PS	72	M	6	1	21.9	3.8	107	1.23	1.11	101.9	1.5	high	7.4	5.4	0.81	43	11.2
PS	75	M	12	1	26.8	3.9	144	1.72	1.21	116.7	2.1	high	9	5.6		28	10.3
PS	36	M	8	1	28.1	4	128	1.56	1.04	130.8	1.6	high	9.2	5.2		49	10.5
PS	59	M	9	1	21.9	4	181	1.22	0.82	132.1	2.1	high	9.8	10.6	1.6		9.9
PS	70	M	3	1	22.3	3.7	112	1.23		59.16	2.5	high		6.8	3.85	20	10.9
PS	58	F	8	1	20.2	3.9	186	1.2	0.98	115.5	2.1	high	9.5	5.7	0.44	50	
PS	64	M	12	1	23.9	4.3	118	1.56	0.91	139.6	2.1	high	9.2	5.7		33	10.6
PS	47	F	10	1	28.3	4.1	148	1.75	1.18	147.5	1.1	high	10.1	3.5	0.03	24	9.6
PS	64	M	9	1	26.1	4.1	120	1.6	0.94	127.7	1.5	high	8.9	5.3	0.5		12.1
PS	59	M	6	1	22.4	4	190	1.38	1.09	130.5	1.6	high	8.7	3.5	0.57	26	11
PS	83	M	6	1	16.1	4	88	1.81	0.8	87.56	1.6	high	9	7	0.06	23	10.4
PS	36	M	4	1	21.7	3.5	183	1.71	0.98	63.34	1.5	high	9.2	3.4	0.06	24	12.3
PS	62	F	4	1	19.8	4.3	144	1.88	1.23	123.5	1.5	high	7.9	2.9	0.09		8.3
PS	48	F	4	1	19.2	4.2	179	1.46	0.95	109.7	1.1	high		5.8	0.1	22	11.4
PS	29	F	2	1	22.3	4.2	99	0.84	0.94	92.25	1.5	high		5.4	0.3	35	8.7
PS	78	M	14	1	20.1	4.2	67	1.57	0.88	130.1	1.6	high	10	6.9	0.02	27	11.1
PS	60	F	12	1	24	3.4	104	1.43	0.65	91.55	2.1	high	9.5	5.8	0	35	9.8
PS	70	M	30	1	17.4	3.6	138	1.65	0.68	77.31	0.8	high	9	3.1	0.05	35	12.5
PS	46	F	21	1	20.3	3.5	111	1.22	1.08	101	1.3	high	9.1	6.3	0.17	29	8.6
PS	63	M	8	1	20.5	3.9	126	1.22	0.9	109	1.9	high	10	4.9		23	11.5
PS	53	M	8	1	23.1	4.3	156	1.37	0.8	101.7	2.1	high	9.8	7.9	0.78	29	13
PS	71	M	6	1	23	3.9		1.72	0.88	121.9	1.5	high	8	5.1	0.3	26	11.6
PS	74	F	5	1	14.3	3.4	151	1.65	0.82	78.66	1.9	high	8.5	7.2		25	10.7
PS	65	M	5	1	22.8	3.6	195	1.25	0.78	132.9	2.1	high	9.7	6.8	0.02	29	11.1
PS	89	F	5	1	21.5	4.1	154	1.55	0.91	111.4	1.6	high	8.6	3.2		30	9.7
PS	43	F	4	1	18.2	3.8		1.28	0.84	98.72	1.8	high	9		0.89		9.5
PS	82	F	4	1	23.8	4.3	147	1.16	0.61	111	1.3	high	9.2	4.4	0.2	26	10
PS	43	F	4	1	20.7	3.7	146	1.85	0.69	90.56	1.6	high	8.8	4.1		31	11.1
PS	72	M	4	1	24.2	4.2	122	1.35	0.82	142.9	1.9	high	9.7	5.5		28	10.1
PS	78	F	4	1	23.1	3.9	187	1.73	0.96	121.2	2.1	high	10.1	5.9	0.27	28	13.2
PS	58	M	3	1	20.3	3.9	149	1.49	1.02	86.43	2.1	high	9.3	4.9	0.23	30	10.5
PS	64	M	3	1	20.7	3.9	172	0.96	0.96	97.93	1.8	high	8.1	8.3	0.7	26	7.9
PS	53	F	30	1	17.7	3.9	196	1.41	1.24	104.6	1.5	high	8	5.1	0.59	29	10.2
PS	59	M	24	1	19.4	3.4		1.77	0.84	99.82	2.2	high	9.9	2.7	0.03	32	10.6
PS	47	M	23	1	18.9	4.1		2.14	0.85	120.1	1.3	high	9.3	5.5	0.08	27	9.1
PS	74	F	14	1	17.9	4.1	133	1.18	1.09	116.3	1.6	high	9.4	4.6	0.03	37	10.3
PS	48	M	10	1	19.5	4.1	111	1.13	1.13	136.9	1.8	high	10.2	8.5			10
PS	46	F	9	1	16.8	4.1	147	1.26	0.97	96.59	2.5	high	9.8	6.7	0.09	28	11.5
PS	62	M	6	1	29.1	3.5	176	1.71	0.9	119.9	2.1	high	9.1	6.5	0.11	21	9.8
PS	32	F	5	1	20.2	4.2	177	1.31	0.89	95.67	1.5	high		7.2	0	23	14.5
PS	54	M	15	1	21.1	4.2	171	1.34	1	86.6	1.8	high	8.7	5			11
PS	63	M	9	1	21.7	2.8	106	1.25	0.89	101.3	1.5	high	10.5	4.1	2.18	29	9.8
PS	69	F	8	1	22.4	4	158	1.49	0.97	117.4	1.8	high	10.2	6.1	1.13	26	8.9
PS	53	F	6	1	22	3.9	230	1.56	1.19	126	1.5	high	8.9	2.9		23	10.6
PS	70	M	5	1	22.2	2.3	119	2.15	0.8	110.9	1.6	high	9.8	5	0.1	19	10.2
PS	76	M	5	1	21.1	1.9	181	1.85	0.88	91.32	1.8	high	9.5	4.7			9.8
PS	56	M	3	1	19.2	4.1	114	0.99	0.57	81.69	1.6	high	8.6	3.7		22	10.1
PS	61	F	2	1	21.9	2.5	137	1.39	0.83	79.33	1.5	high	8.9	5.5	9.5	30	8.9

PS	77	F	2	1	19	4.1	42	1.17	0.78	69.12	1.6	high	9.8	4.9	0.05	22	8.4
PS	52	F	3	1	18.7	4.3	110	1.45	1.11	150.2	2.1	high	9.3	5	2.76	28	9.9
PS	67	F	5	1	31.5	3.6	146	1.45	1.09	99.07	2.1	high		5.5	0.03	19	12.6
PS	74	M	3	1	22.2	3.9	131	1.4	0.8	108.3	1.6	high	9.5	3.5		21	9.6
PS	48	F	10	1	16.3	4.3	192	1.13	1.06	125.2	2.1	high	9.1	6	0.12	28	10.2
PS	66	M	12	1	18.4	3.4	159	1.58	0.59	80.65	1.3	high		5.7	0.1	21	11.4
PS	60	M	16	1	24.6	3.9	145	1.37	0.86	130.9	1.3	high	9.9	4.3	0.02	35	10.8
PS	51	M	8	1	28.1	3.6	119	1.52	0.87	139.8	1.5	high	9.8	4.6	0.16	50	9.3
PS	67	M	7	1	20.5	3.3	191	1.27	1.01	138.4	1.6	high		7.7	0.56		12.6
PS	71	M	5	1	22.3	3.9	171	1.6	1.07	113.8	1.3	high	8.2	5.9	0.39	44	10.7
PS	68	F	4	1	23.8	3.9	182	1.03	1.22	120.8	1.6	high	9.1	3	0.12	35	10.4
PS	57	F	2	1	25	4.1	188	1.4	0.81	68.25	1.8	high	9.9	5.8	0.3	30	10.1
PS	52	F	4	1	25.6	3.8	183	1.47	0.73	108.6	1.8	high		5.1	0.14		11.7
PS	63	F	4	1	16.3	3.9	152	1.48	1.18	127.8	1.8	high		5.3	0.2	10	9.8
PS	50	M	11	1	23.4	4.1	194	1.49	0.97	129.7	2.1	high	10	6.7	0.64	38	10
PS	54	M	24	1	19.3	3.7	106	1.91	1.44	123.8	1.9	high	9.2	6.3		29	9.2
PS	55	M	20	1	23.2	3.2	143	1.46	1.03	124.3	1.6	high	9	3.8	0.1	25	11
PS	53	F	16	1	17.1	3.5	168	1.6	1.1	89.75	1.6	high	8.9	5.1		32	11.5
PS	64	F	12	1	19.7	3.6	186	1.32	1.07	138	1.8	high		5.4	0.05		10.9
PS	78	F	10	1	17.4	3.6	147	1.32	1.34	110	2.1	high	8.8	5.6	1.83	29	12
PS	79	M	9	1	17	3.7	104	0.99	1.42	97.48	1.6	high		6.9	0	20	12
PS	39	M	8	1	29.7	3.9	193	1.13	1.15	109.8	1.5	high	9.8	6.2	0.16	28	10.8
PS	61	F	7	1	19.3	3.9	170	1.15	0.79	150	2.1	high	9.9	7	0.04	24	8.6
PS	56	F	6	1	23.2	3.6	132	1.11	1.18	149.6	1.8	high	7.5	5.7	0.6	28	11
PS	66	M	4	1	21.3	3.5	175	1.02	1.06	108.6	1.6	high		4.6	0.1	40	11.1
PS	75	F	3	1	21.4	3.4	225	1.48	0.96	147.1	1.5	high	9.4	5	0.1	25	10.1
PS	75	M	3	1	23.1	3.9	93	1.51	0.86	91.61	1.6	high	7.7	3.1	1.83	24	13.3
PS	58	M	2	1	18.1	3.9	179	1	1.15	87.74	1.3	high	9.3	5.9	0	22	
PS	57	M	2	1	25.2	3.9		1.36	1	155.1	1.5	high	8.6	4.1		39	12.2
PS	61	M	2	1	28.7	4	166	1.43	1.03	71.52	1.9	high	8.3	6.4	0	13	11.3
PS	69	F	20	1	22.3	4	213	2.09	0.84	121.4	1.6	high	9.2	5.6	0.04	29	10.5
PS	61	F	14	1	17.6	4	142	1.7	0.77	132.1	1.5	high	9.6	5.1	0.13	18	10.2
PS	60	M	12	1	23.7	3.4	231	1.32	0.62	97	1.3	high	9.6	6.6	0.22	40	10.7
PS	48	F	11	1	20.9	3.8	185	0.92	0.76	104.9	1.6	high		5	0.7	16	12
PS	66	M	11	1	20	2.9	126	1.07	0.78	120.3	2.1	high		4	0.09	30	10.3
PS	76	M	8	1	17.6	3.7	73	0.74	1.06	82.15	1.5	high		5.2	0.01	27	8.9
PS	47	F	6	1	24	3.9	134	1.21	0.92	122.3	1.5	high		6.8	0.02	23	10.8
PS	62	F	5	1	20.5	3.9	143	1.03	1.05	115.5	1.3	high	7.1	4.7	6.88	37	11.1
PS	72	M	3	1	23	3.5	154	1.26	0.58	68.37	1.8	high	9.7	5.5	0.15		11.5
PS	75	M	4	1	25.1	3.7	138	1.73	0.92	95.41	1.5	high		3.3	0.13	22	9.1
PS	65	M	2	1	22.6	3.8	221	1.7	0.74	104.2	1.5	high	7.9	4.4	0.04	26	11
PS	78	F	9	1	24.4	3.4	82	1.54	1	83.37	2.1	high	7.5	4.4	1.29	24	
PS	72	F	9	1	19.5	4.5	136	1.5	1.16	101	1.9	high	10.2	7.6	0	30	11.9
PS	75	M	11	1	19.3	3.8	117	1.6	0.93	140.9	1.6	high	8.5	8.1	0.2	19	9.9
PS	47	F	13	1	21.2	3.6	178	1.2	0.91		2.1	high		5.1	1.06		11.2
PS	61	F	10	1	18.3	3.9	179	1.52	1.13	99.15	2.1	high	8.5	3.7	0.44	47	9
PS	68	F	17	1	18.9	4.3	205	1.22	1.16	112.4	1.6	high	10.1	4.3	0	19	10
PS	59	M	7	1	17.3	3.1	140	1.54	1.1	88.11	1.6	high	10	3.2	0.8	27	10.2
PS	51	M	5	1	20.3	3.8	141	1.34	0.95	112.9	1.8	high	10.4	7	0.54	36	9.7
PS	73	F	3	1	25.2	3.5	95	1.43	0.85	83.01	1.5	high	10	3.8	2.15	23	10.3
PS	66	F	14	1	28.8	3.4	140	1.32	0.8	115.4	1.8	high	7.9	9.1	0.48	32	9.9
PS	27	F	8	1	22.3	4.6	129	1.23	1.13	90.44	1.8	high	8.3	4.7	0.67	46	10.2
PS	76	M	7	1	25.5	3.6	154	1.27	0.78	122	1.5	high		5.4	0.1	26	10.7
PS	70	M	7	1	40.4	3.5	90	1.41	0.76	101.7	1.5	high	9.9	4.2	0.44	32	9.6
PS	80	M	7	1	18.7	1.9	92	1.45	0.94	120.2	1.6	high	8.4	4.5	0.1	30	11.3
PS	64	M	7	1	22.1	3.7	190	1.27	0.79	119.4	1.8	high	9.4	6.7	0.03	27	11.6
PS	82	F	6	1	19.4	3.6	102	1.45	0.93	120.2	1.6	high	10.2	5.4	0.1	30	10.5
PS	55	M	5	1	27.3	4.1		1.56	0.82	102.6	1.6	high	8.9	2.6	0.37	31	6
PS	80	M	5	1	17.8	4	118	1.46	0.75	76.47	2.1	high	8.8	5.7			7.8

PS	51	F	4	1	19.2	4.1	112	1.62	0.89	103.8	1.8	high	8.3	5.5	0.06	34	9.2
PS	92	F	4	1	15.9	3.4	222	1.6	0.63	139.1	1.5	high	8.3	5.1	0.09	31	10.2
PS	67	F	4	1	27.7	4.2	154	1.76	0.9	93.21	1.3	high	9	4.8	0.02	24	9.9
PS	65	F	4	1	24.8	3.4	106	1.27	0.86	64.57	1.3	high	9.1	4.2	0.16	25	9.4
PS	66	M	4	1	21.9	3.8	109	1.1	0.99	121.6	1.6	high	8.9	3.8	0.01	29	10.6
PS	61	M	4	1	22.9	3.9	127	1.55	1.06	116.5	1.3	high	9.2	3.2	0.03	29	11.2
PS	46	M	4	1	22.3	4.2	119	1.56	0.92	106.9	1.3	high	9	4.1	0.93	32	9
PS	59	M	4	1	20.9	3.7	163	1.39	1.09	94.59	1.3	high	9.2	5.6	0.1	33	10.9
PS	62	M	4	1	18.4	3.5	127	1.41	0.99	63.2	2.1	high	9.4	4.2	0.33	37	10.4
PS	49	M	4	1	20.9	3.7	148	1.63	0.97	54.62	1.3	high	8.8	3.5	0.05	33	10.5
PS	32	M	4	1	27.4	4.3	158	1.73	0.9	111.7	2.1	high	6.7	7.3	0.05	34	11.5
PS	77	M	3	1	21.9	3.8	109	1.25	0.54	104.5	1.3	high	10	4.3	0.1	29	10.9
PS	79	M	3	1	19.4	1.4	171	1.78	1.19	81.49	1.5	high	9.9	5.5	0.13	29	10.5
PS	76	M	3	1	21.4	3.4	124	1.17	0.77	123.5	1.8	high	8.6	4.2	0.14	31	9.6
PS	88	M	3	1	18.5	3.4	169	1.41	1.07	115	1.5	high	8.4	6.8	0.24	39	9
PS	48	F	3	1	17.7	3.6	155	1.07	1.38	91.73	2.1	high	8.9	6.5	0.38	35	10.3
PS	58	F	3	1	17.9	3.7	171	1.56	0.74	74.44	1.5	high	9.2	6.4	0.45	31	10.9
PS	64	F	3	1	24.7	3.8	213	1.44	0.84	83.68	1.8	high	9.2	4.9	2.16	28	11.1
PS	77	F	2	1	21.9	3.3	152	1.66	0.81	123.9	1.5	high	7.7	5.9	0.04	35	11.2
PS	83	M	2	1	21.1	3.7	183	1.44	0.92	119.8	2.1	high	9	6.6	0.03	35	10.4
PS	73	F	2	1	19.6	3.4	197	1.28	1.09	118.4	2.1	high	9.8	4	0.12	31	11.6
PS	45	F	2	1	15.7	3.9	140	1.19	0.77	103.7	1.8	high	9.6	5.5	0.2		10.9
PS	57	F	2	1	16.7	3.9	167	1.45	1.07	110.8	1.5	high	7.5	4.9	0.12	45	9.4
PS	90	F	2	1	20.6	3.5	118	1.42	0.92	144.5	1.5	high	8.2	4.8	0.03	30	11.6
PS	33	M	2	1	30.5	3.8	193	1.23	0.87	79.72	2.1	high	9.5	4.8	0.17	26	10.5
PS	59	M	33	1	19.7	3.8	136	1.62	0.91	69.05	2.1	high	9	4.5	0	27	11.3
PS	62	F	32	1	21.5	3.2	151	1.47	0.92	66.07	1.8	high	9.3	6	0.25	26	9.6
PS	73	F	27	1	18.8	3.1	108	1.39	0.85	80.6	0.6	high	9.8	4.8	0		11
PS	64	F	26	1	18.3		120	2.52	1.27	111.6	2.1	high	8.8	5.6	0	28	10.3
PS	72	M	24	1	15.9	4.2	149	1.62	0.99	72.22	1.4	high	9.1	5.5	0		10.5
PS	64	M	24	1	17.8	3.8	173	1.13	0.96	104	1.6	high	8.1	6.6	2.1	32	10.4
PS	61	F	23	1	16.1	3.8	151	1.33	1.14	122.1	2.1	high	9.5	6.6	0.04	32	10.1
PS	48	F	20	1	26.2	3.6	139	1.29	0.91	103.4	1.5	high	7.8	3.1	0	23	9.4
PS	71	F	22	1	20.4	3.3	159	1.95	0.82	123.5	1.6	high	10.5	7.6	0.09	27	11
PS	60	F	21	1	28.8	4	182	1.5	1.07	112.1	1.3	high	9.4	6	0.7		10.3
PS	61	F	21	1	14.4	4.2	140	1.47	0.96	92.34	2.1	high	10.2	7.9	0.06	23	10
PS	49	M	20	1	25.8	4.2	112	0.94	0.85	105.8	1.9	high	8.7	6.2	0.1	32	9.7
PS	60	F	18	1	26.1	3.7	111	1.22	1.29	130.3	1.6	high	9.9	3.8	0	23	9.3
PS	68	M	18	1	20.1	3.7	202	1.36	0.87	130.9	1.6	high		5.3	0.99		14.8
PS	53	F	15	1	17.1	3.9	120	2.14	0.9	110.1	1.8	high	9.6	4.6	0.05	25	10.8
PS	48	M	14	1	22.9	4	153	1.67	1.02	99.29	1.9	high	8.9	5.7	0.8	21	10.3
PS	62	M	14	1	21.4	3.8	149	1.61	1.08	124.8	1.9	high	9.2	5.4	0.1	32	9.7
PS	56	M	14	1	20.6	3.5	183	1.12	0.86	125.7	1.9	high	10.3	6.8	0.04	34	11
PS	64	M	14	1	21.9	4.1	151	1.14	1.02	128.4	1.6	high	8.6	4.5	0.44	38	9.2
PS	58	M	13	1	22.3	4.3	157	1.94	0.8	115.1	1.4	high	8.2	2.7	0.1	35	11.7
PS	69	F	13	1	24.4	3.7	101	1.17	0.84	126.7	2.1	high	8.5	6.1	0	29	10.4
PS	78	F	13	1	25.7	3.4	147	1.26	0.8	113.1	2.5	high	8.4	4.9	2.23		10.3
PS	39	F	13	1	18.6	4	160	1.62	1.28	117.5	1.8	high	9.5	5.9	0.03		11.5
PS	60	M	12	1	19.1	4	154	1.1	0.95	112.8	2.1	high		5.5	0.47	32	8.9
PS	56	F	11	1	23.1	3.9	177	1.88	0.89	120.2	1.6	high	9.5	5.9	0.06	34	9.5
PS	45	M	10	1	20.2	3.9	109	1.24	0.95	115.6	2.1	high	9.2	6.7	0.21	30	11.4
PS	68	F	10	1	19.8	4.3	130	1.42	1.15	135.9	1.8	high	9.8	5	0.05	26	9.2
PS	48	F	10	1	22.7	4.1	169	1.34	1.06	149.5	2.2	high	9.3	4.7	0.05	26	9.9
PS	73	M	10	1	20.7	4.1	116	1.28	0.9	102.7	1.6	high	9	4.6	0.1	30	10.4
PS	73	M	10	1	21.5	3.4	149	1.1	1	163.5	1.9	high	8.8	6.8	0.03	24	10.3
PS	50	M	9	1	17	3.7	249	1.79	0.96	122.1	2.2	high	9.1	7.9	0.24	38	8.5
PS	80	M	9	1	25.3	3.8	156	1.51	0.71	105.5	2.2	high	10	7.8	0.64	33	10.1
PS	73	M	9	1	19.3	3	113	1.83	0.92	83.11	2.1	high	9.6	6.8	0.05	31	12
PS	64	M	8	1	22.4	3.8	153	1.3	1.04	127.1	2.1	high	8.7	5.4	0.11	23	12.1

PS	56	M	18	1	19.7	4.3	150	1.45	1.07	123	1.8	high	8.8	3.9	0.05	29	9.6
PS	64	F	8	1	21	4.3	142	1.39	1.24	96.3	1.5	high	8.2	3.5	0.1	27	10.8
PS	56	F	7	1	20.4	3.8	148	1.27	0.96	138.7	1.8	high	10	6		24	10.2
PS	60	F	7	1	22.4	3.8	179	1.49	0.84	149	1.8	high	8.9	4.9	0.07	29	9.9
PS	40	F	6	1	23.6	3.9	161	0.66	1.04	106	1.5	high	8.8	5.9	4.55	32	7.1
PS	49	M	6	1	21.1	3.2	140	1.37	0.75	79.24	2.1	high	8.5	5.5	0.2	22	13.1
PS	77	M	5	1	24.2	3.5	183	1.61	0.66	134.5	1.6	high	8.8	3.7	0.04	25	9.8
PS	73	M	5	1	22.1	3.8	129	1.47	0.94	139.8	2.1	high	8.4	7.7	0.05	56	11.4
PS	60	M	5	1	24.4	3.3		1.8	1.11	19.41	1.5	high	9.5	5.8		23	11.1
PS	68	M	4	1	21.4	4.1	198	1.04	0.85	79.61	1.8	high	10	6.7	0.73	28	9.5
PS	57	M	4	1	23	4.5	162	1.15	0.84	113.5	2.1	high	9.3	5.2	0.42	30	10.4
PS	59	M	4	1	20.5	4.6	178	1.99	1.09	76.35	2.1	high	8.4	4.4	0.04	26	10.2
PS	56	M	13	1	27.4	3.5	120	1.46	0.7	96.54	1.5	high		4.1	1.48	24	9.6
PS	73	F	3	1	28.7	3.3	120	0.98	0.75	137.4	1.8	high	8.2	7.1	0.05	48	9.6
PS	59	F	3	1	25.4	3.5	103	1.54	0.93	78.8	2.1	high	9.9	5.4	0.05	32	11.9
PS	69	F	3	1	19.8	3.6	129	1.65	0.97	94.95	1	high	8.7	3.7		25	10.2
PS	73	F	2	1	30.3	4.1	208	1.51	0.64	97.94	1.8	high	9.7	4.3			10.2
PS	53	F	2	1	22.4	3.6	198	2.1	0.81	76.92	1.8	high	9.6	5.8		36	11.1
PS	74	M	2	1	20.4	3	152	1.24	0.85	82.23	1.5	high	9.6	5.6	0.22	37	13.1
PS	55	M	2	1	17	3.9	173	1.13	0.87	82.41	2.2	high	8.4	3.7	0.39	9	13.8
PS	41	M	8	1	27.4	3.7	189	1.66	0.86	91.73	1.3	high	9.1	5.5	0.04	25	12
PS	58	M	7	1	25.1	3.9	165	0.9	1	142.5	1.3	high	8.5	6.3	0.46	26	9.5
PS	55	M	13	1	21.9	3.8	124	1.05	0.86	104.2	1.3	high	8.7	4.2	0.3	28	10.8
PS	58	F	11	1	20.1	3.9		0.95	0.98	131.4	1.9	high		8.6			8.3
PS	71	M	11	1	23.2	3.8	203	1.65	0.92	99.75	1.4	high	8.9	3.9	0.26	32	11.1
PS	51	F	9	1	18.2	4	166	1.05	1.09	97.12	1.4	high	8.9	4.3	0.66	12	10.4
PS	57	M	4	1	18.2	4	143	1.53	0.69	120.2	1.8	high	8.7	4.7	0.37	29	10.6
PS	79	F	4	1	21.4	3.5	160	1.42	0.75	98.75	1.4	high	8.7	5.1	0.05	20	10.1
PS	72	F	2	1	21.3	3.9	139	1.57	0.78	87.17	1.5	high	8.3	3.9	0.24	26	10.9
PS	46	M	20	1	20.1	3.9	132	1.36	1.06	104.5	1.8	high	9.7	7.2	0.54	15	8.6
PS	57	M	19	1	20	4	123	1.54	0.8	114.8	1.3	high	9.2	5.2	0.2	32	11.8
PS	76	M	17	1	20.4	3.6	245	1.62	0.8	102.8	1.5	high	9.2	5.6	0.02	23	9.9
PS	43	F	17	1	21.2	3.6	180	1.15	1.18	96.36	1.5	high	8.8	6.3	1.65	20	9.1
PS	59	M	12	1	20.9	4.2	188	1.59	0.84	101.1	2.1	high	8.5	3.6	0	35	10.4
PS	61	M	12	1	20.5	3.5	123	1.52	0.75	79.19	1.5	high	8.2	4	0.05	29	9.4
PS	66	M	11	1	21	3.7	177	2.36	0.79	99.19	2.1	high	8.1	6.6	0	30	9.4
PS	50	M	10	1	19.3	3.8	186	1	0.79	114.6	1.3	high		4.9	0.54	35	11.9
PS	38	M	10	1	20.4	4.3	149	1.77	0.68	112.3	1.8	high	10	5.4	5.1	35	10.9
PS	50	M	10	1	16.4	4.2	158	1.69	0.66	101.1	2.1	high		4.7	0.09	39	11.4
PS	46	M	10	1	19.6	3.6	119	1.64	0.93	86.87	2.1	high	9.1	6	0.05	28	9.9
PS	55	M	10	1	16.5	3.7	224	1.48	1.15	101.9	1.5	high	8.7	4.7	0.03	26	9.8
PS	68	F	10	1	21.1	3.7	149	1.03	0.71	112.1	1.5	high	9	4.8	0.01	28	10.3
PS	61	M	9	1	18.4	3.8	140	1.68	0.82	78.73	1.5	high	8.3	4	0	24	9.8
PS	58	M	9	1	21.2	4.1	123	1.44	0.89	66.21	1.5	high	9.2	3.2	0.36	25	9.7
PS	51	M	8	1	15.8	4.3	99	1.26	0.73	64.07	2.1	high	9	4.6	0	27	9.9
PS	52	F	6	1	20.1	4.4	122	1.24	0.63	67.08	1.8	high	9.6	3.1	0.27	29	11.6
PS	80	F	6	1	19.4	3.7	143	1.11	0.55	83.02	2.1	high	10.8	4.4	0.02	24	9.8
PS	71	M	4	1	22.8	3.5	150	1	0.76	115.2	2.1	high	9.8	5	0.01	29	10.3
PS	51	F	4	1	26.4	3.9	188	1.22	0.85	138.5	1.5	high	7.2	5.2	0.64	31	10.8
PS	63	F	2	1	17.3	3.7	122	1.56	0.53	49.13	1.8	high	8.6	5.7	0.16	34	12.1
PS	71	M	2	1	17.6	3.4	110	1.24	0.69	89.93	1.8	high	8	4.7	0.05	22	9.7
PS	68	M	2	1	20.8	3.7	122	1.39	0.74	48.49	1.8	high	9.8	5.1	0.18	17	10
PS	50	M	2	1	21.1	3.7	123	1.57	0.89	65.12	2.1	high	8.2	5.4			11.5
PS	84	F	2	1	22.3	3.1	115	1.39	0.49	46.99	1.3	high	8.5	5.2		32	10.4
PS	69	F	6	1	20.7	3.4	193	1.33	0.71	119.9	1.9	high	8.7	4.8	0.06	31	9.1
PS	47	M	6	1	20.5	4.1		1.41	1.22	75.78	1.8	high	9.3	5.2	0.12	29	9.4
PS	60	F	28	1	24.9	4	220	1.15	1.05	109.2	1.1	high	9.4	1	0.13	25	10.1
PS	57	M	21	1	23.5	3.9	101	1.23	0.96	112.3	1.6	high	8.6	4.3	0.06	29	9.4
PS	55	M	19	1	17.3	4	174	1.54	0.92	153.2	1.3	high		5.2	0.45	33	9.6

PS	56	F	18	1	24.1	4.1	182	1.25	1.04	89.77	1.9	high	9.7	6.5	0.18	25	10.3
PS	52	F	17	1	24.1	4	125	1.06	0.74	108.3	1.9	high	8.9	4.4	0.63	33	10.4
PS	57	F	14	1	20.1	4.2	106	0.99	1.18	119.7	1.9	high	9.1	6	0.04	18	8.5
PS	47	F	13	1	17.8	4.3	138	1.67	1.29	116.5	1.9	high	8.8	6.5	0.06	24	10.2
PS	66	M	12	1	21.2	4.1	177	1.17	1.11	126.3	2.1	high	8.7	3.7	0.62		10.6
PS	71	M	12	1	23.5	3.7	186	1.58	0.83	137.8	1	high	9.7	6.9		32	11.6
PS	67	F	10	1	22.4	3.9	176	1.7	0.88	98.15	1.5	high	8	3.7	0.4	46	7.1
PS	85	M	9	1	24.9	4		0.85	1.01	106.3	2.5	high		4.6	0.05	31	11.7
PS	67	F	9	1	20.6	4	130	1.56	1.1	131.5	1.6	high		5.2		21	11.5
PS	58	M	9	1	23.5	4	108	1.53	1.1	120.8	1.8	high	8.1	5.1	2.76	24	6.3
PS	53	F	8	1	22.2	4.1	118	1.73	1.04	118.6	1.8	high	9.4	5.4	0.1	23	10.5
PS	47	F	8	1	23.3	3.9	140	1.8	1.07	118.2	1.6	high	7.9	2.9	0.08	20	
PS	85	F	8	1	18.1	4.1		1.4	1.22	87.47	1.5	high		3.8	0.2	17	9.7
PS	57	M	8	1	22	4.2	132	1.55	1.07	150.5	1.6	high	8.3	5.5			9.8
PS	52	F	8	1	22.1	4		1.04	0.86	115.9	1.3	high	7.6	3.7		36	7.9
PS	71	F	6	1	24	3.9	127	1.28	0.85	98.28	1.5	high	8.1	6.3	0.12	29	11.2
PS	43	M	6	1	20	4.1	149	1.55	1.21	103.3	1.9	high	8.7	6.5			11.7
PS	41	M	6	1	26.8	4.2	159	1.34	0.91	88.03	1.5	high	9.6	6.9	0.2	32	11
PS	31	M	5	1	20.4	4.2	152	1.16	0.85	123.9	1.5	high	9.5	5.8	1.02	24	10
PS	85	F	5	1	23.1	4	105	1.28	1.06	89.11	1.5	high	9.2	3.7	0.1	24	11.4
PS	57	F	4	1	26.3	4	121	1.55	0.98	119.5	1.9	high	8.5	6.5			10.2
PS	52	M	4	1	25.9	4.2	139	1.4	0.93	113.8	1.5	high	7.4	1.9	2.31	41	8
PS	51	F	3	1	24.2	4.3	156	1.2	1.13	107.2	1.3	high	9	6.2	0.03	30	11.5
PS	75	F	3	1	25.3	4.1	124	1.23	1.05	116.9	1.5	high	7.9	5.3	0.07	27	10
PS	72	M	3	1	21.8	4	222	0.99	1.03	139.7	1.5	high	9.2	3.2	0.94	45	7.8
PS	57	M	3	1	31.1	4.3	203	1.05	1.24	92.64	1.8	high	8.9	7.3	0.08	23	9.4
PS	56	F	2	1	18.8	4.1	165	1.41	1.3	109	1.9	high	8.2	4.5			11.4
PS	61	M	2	1	22.1	4.3	148	0.97	0.77	78.78	1.8	high	7.3	4.2	0.2	15	10.6
PS	68	F	16	1	21.1	3.8	150	1.29	0.71	95.6	1.3	high	8.6	6.8	1.8	35	10.6
PS	70	F	16	1	17	3.9	218	1.5	0.94	86.05	1.6	high	8.7	7.6			11.3
PS	51	M	31	1	22.1	3.5	151	2.37	0.86	109.1	1.6	high	9.9	4	0.03	24	10.8
PS	56	M	24	1	16.8	3.3		1.77	0.99	86.54	1.6	high	9.3	5.9	0.29	29	8.6
PS	58	M	20	1	21.5	4.2		1.68	1.19	130.9	1.6	high	9.6	5.2	0.18	18	10.3
PS	60	M	18	1	18.6	3.6	126	1.03	1.3	111.9	2.2	high	10.1	6.9		23	
PS	54	M	16	1	18.7	3.4	77	1.31	0.84	107.5	1.6	high		5.8	0.1	27	11.3
PS	45	M	9	1	22.3	4	146	1.54	0.82	100.1	1.9	high	8.5	2.3			8.5
PS	35	M	2	1	25.9	4	181	1.55	1.26	111.1	1.3	high	9.2	6	0.1	25	9.9
PS	58	M	2	1	23.1	3.5	189	1.84	0.71	109	1.3	high	9	7.4	0.11	38	11.6
PS	73	F	27	1	15.6	3.7	121	1.5	1	84.64	2.1	high		4.2	0.26	27	9.3
PS	74	F	11	1	21.2	3.8	124	1.43	1.11	125.2	2.2	high	9.9	5.4	0.15		10.5
PS	57	F	5	1	25	3.9	155	1.69	0.48	96.76	1.9	high	8.5	6.4			10
PS	64	M	32	1	23.8	3.9	191	1.72	1.15	100.8	2.1	high	8.5	4.1	0	23	8.9
PS	56	F	19	1	21.4	4.3	126	1.51	1.14	140.9	1.8	high	10	6.5			
PS	63	F	18	1	20.3	3.9	126	1.54	0.85	116.8	2.1	high	8.7	5	0	31	10.3
PS	53	M	16	1	19.9	3.9	143	1.68	0.85	114.9	1.5	high	9	4.6	0.07	28	10.1
PS	65	M	15	1	21.4	4.1	161	1.57	0.84	124.2	1.6	high	7.8	6.9	0.16	27	10.5
PS	61	M	14	1	22.5	3.8	100	1.14	0.88	121.7	2.1	high	8.9	3.9	0	35	10.3
PS	67	F	14	1	20.5	4.2	200	1.23	1.28	128.1	1.6	high	9.1	4.4	0.17	30	10.4
PS	69	M	14	1	20.3	3.8		1.43	0.96	112.6	2.1	high					
PS	59	M	14	1	22.8	4.1	140	1.48	0.88	148.3	1.5	high	9.3	4.4	0.16	40	10.1
PS	53	F	13	1	23.3	4.1	195	1.37	1.06	111.7	2.1	high	10.3	5.9	0.01	27	10.4
PS	56	M	13	1	26.4	4.1	145	1.33	0.79	109.7	1.8	high	8.9	5.8	0.05		11.3
PS	48	F	13	1	21.1	3.9	134	1.68	0.88	128	2.1	high	11.8	7.8	0.13	29	11.5
PS	70	M	12	1	21.9	3.9	130	1.71	0.73	129.4	1.5	high	9.2	7.1	0	26	9.9
PS	59	F	11	1	19.7	3.9	168	1.35	0.9	117.1	1.5	high	9.4	5.8	0.19	23	10.1
PS	44	F	11	1	17.5	4.1	154	1.69	1.13	115.5	1.6	high	8.1	5.5			8.9
PS	73	F	11	1	21.8	3.9	131	1.82	1.24	101.8	1.5	high	10.7	4.2	0	24	10.3
PS	59	M	11	1	19.6	4.1	176	1.93	1.05	114.6	1.5	high	8.9	3.8	0.1	22	9.6
PS	63	M	10	1	22.6	3.9	141	1.65	0.92	119.7	1.5	high	9	3.5	0.3	30	9.3

PS	60	M	10	1	25.3	4.4	77	1.54	0.99	111.6	2.1	high	9.7	6.4			10.3
PS	61	M	9	1	16.1	4.2	172	1.22	0.77	104	2.1	high	9.5	7.7	0.03	18	9.7
PS	51	F	9	1	23.8	4.4	100	1.08	0.78	95.59	2.1	high	10.5	8			10.5
PS	64	M	9	1	23.9	4.1	162	1.47	0.95	138.3	1.5	high	8.8	3.3	0.08	26	10.5
PS	68	F	9	1	16	3.8	152	1.91	0.84	126.2	1.5	high	8.6	4.3	0.01	25	9.3
PS	36	M	9	1	21.3	4.7	161	1.21	1.26	128.6	1.8	high	10	6		26	10.9
PS	63	F	9	1	19.7	4.3	104	1.39	0.97	83.77	1.6	high		4.7	0.93	36	9.8
PS	67	F	9	1	18.8	4.1	215	1.75	0.77	83.26	1.5	high	8.9	3.5	0.03	30	9.4
PS	47	F	8	1	23.5	4.3	125	1.89	0.95	136.7	1.5	high	8.4	4.2	0	34	10.4
PS	74	M	8	1	22.3	4.1	178	1.63	1.02	124.2	1.5	high	8.6	4	0.04	21	11.1
PS	52	F	8	1	14.1	3.8	111	1.49	0.55	103.2	2.1	high	8.1	5.1	0	31	10.2
PS	54	F	8	1	18.7	4.4	102	1.27	1.1	90.21	1.8	high	9.7	6.2	0.07	50	10.3
PS	56	F	8	1	20.5	4.4	151	1.36	1.17	93.45	1.8	high	9	4.8		30	11.4
PS	59	F	8	1	23.8	4	131	1.81	0.75	144.4	1.5	high	9.4	6	0	26	11.1
PS	62	M	8	1	22.8	3.7	123	1.45	0.98	139.1	1.5	high	8.6	4.7	0	24	9.6
PS	66	M	8	1	19.5	4.4	156	1.34	1.12	115.8	2.1	high	9.5	5.9	0.1	21	12.4
PS	64	M	7	1	22.3	3.8	105	1.46	0.83	108.7	2.5	high	10.1	5.1	0.14	23	11.7
PS	51	M	7	1	23.4	4.4	176	1.86	1.11	121.7	1.9	high	8.2	4.4			9.9
PS	36	M	7	1	16.4	4.4	219	1.27	1.04	109	1.5	high	9.7	4.1	0.09	23	9.2
PS	52	M	7	1	21	4.4	172	1.84	0.78	100	1.3	high		6.2	0.11	26	10.4
PS	60	M	7	1	19.4	3.9	166	1.51	0.85	107.2	1.5	high	8.9	3.9	0	27	10.5
PS	54	M	7	1	26.1	4.4	192	1.41	1.04	105.9	1.6	high	9.2	5.7	0.24		10.9
PS	63	M	7	1	19.9	3.9	131	1.78	1.17	118.9	2.1	high	9.5	4.7	0	24	10.3
PS	54	F	7	1	18.8	4.2	135	1.62	0.89	126.8	1.5	high	8.9	4.9	0	23	9.9
PS	60	F	7	1	19.3	4	171	2.02	1.08	109.8	1.8	high	8.9	6.7	0.08	26	9.8
PS	45	M	6	1	19.9	3.3	120	1.16	1.02	89.37	2.1	high		6	0.08	31	10.6
PS	67	M	6	1	25.2	3.6	147	1.11	1.07	106.6	1.5	high	8.2	4.5	0	25	10.5
PS	60	F	6	1	18.9	2.3	209	1.14	1.16	118.1	1.5	high	9.2	5.1	0.06	17	10.3
PS	79	F	5	1	20.1	3.6	173	1.22	0.6	107	1.5	high	9.3	5	0	22	9.5
PS	39	M	5	1	20.8	3.9	102	1.25	0.84	85.54	1.5	high	9.6	8.1		30	10.2
PS	70	M	5	1	25.5	3.9	125	1.45	0.97	140.9	1.6	high	9.6	7.6	0.07	31	10.3
PS	60	M	5	1	21.6	3.9	125	1.58	1.01	120.5	1.6	high	9.8	5.1	0.35	32	8.9
PS	56	F	5	1	24.7	4.3	115	1.4	0.72	60.79	1.5	high	7.8	4.1	0.18	26	11
PS	76	M	5	1	22.1	4	196	1.35	0.95	144.2	1.8	high	9.2	5.1			9.6
PS	50	F	5	1	29.8	3.8	155	1.11	0.84	79.62	2.1	high	8.6	6.2	0.21	18	11.8
PS	71	M	4	1	22.7	3.5	204	1.82	0.8	126.3	1.6	high	9.6	5.8	0.01	27	10.9
PS	49	M	4	1	23.7	4.1	133	1.86	0.8	89.32	1.8	high	10.6	6.6	0.05	22	10.9
PS	48	M	4	1	21.9	4.2	143	1.36	1.01	119.4	1.5	high	9.5	5.5	0.2	18	11.6
PS	39	M	4	1	31.6	4.2	175	1.65	0.72	20.35	1.8	high		4.2	0.2	14	10.1
PS	64	F	3	1	16.4	4.2	119	1.75	1.04	99.98	2.5	high	8.4	4.7	0.09	22	11.1
PS	65	M	2	1	23.2	4.2	122	1.33	0.98	52.64	2.2	high	7.4	4.5	11.23	11	7.6
PS	60	M	2	1	22.5	3.6	88	1.63	0.84	84.4	2.1	high	9.1	2.1	0.86	25	8.6
PS	66	F	2	1	20.7	3.9	69	1.49	1.07	112.3	1.8	high	10	6.4	0.1	42	7.5
PS	54	F	2	1	32.9	4.2		1.17	0.8	136.3	1.8	high	10	4.2	1.3	22	8.6
PS	66	F	2	1	22.9	3.9	214	2.19	0.93	91.57	1.6	high	10.8	4.7	0.02	34	12.2
PS	45	M	2	1	27.9	4.1		1.44	0.95	127.3	1.1	high		4.4	0.05	31	10.5
PS	58	M	2	1	17.7	4	141	1.08	0.96	103.6	1.8	high	9.2	6.9	1.1	35	8.9
PS	52	M	2	1	21.5	4.3	114	1.26	0.95	117.8	1.3	high	9	4.8	0.1	26	10.3
PS	69	M	11	1	19.4	4.1	162	0.89	0.76	79.98	1.5	high	9	4.2	0.04	25	11.4
PS	59	M	10	1	22.9	3.7	156	1.66	1.17	111.3	1.3	high	8	4.5	0.37	24	9.2
PS	63	F	6	1	24.2	4	154	1.34	1.02	136.2	2.2	high	9.1	5.2	0.35		10.9
PS	62	M	4	1	20.9	4.4	144	0.98	0.9	96.87	2.2	high	9.1	6.6	0.23		11.6
PS	70	M	3	1	22.4	4.3	172	1.47	0.93	77.08	1.5	high	9.4	8.2	0.89	36	11
PS	69	F	3	1	18.8	3.9	138	1.56	1.1	103.8	1.1	high	9.7	5.3	12.37	52	9.4
PS	70	M	2	1	25	3.5	121	1.25	1.03	79.86	1.3	high	9.1	2.6	0.07	29	10.4
PS	63	M	31	1	22.7	3.6	130	1.31	0.94	115.4	1.8	high	8.6	5.6		26	10.6
PS	65	F	25	1	20.7	4	109	1.44	1.27	97.75	2.1	high	9.7	6.5	0.11	37	10.1
PS	43	M	22	1	22.6	3.8	149	1.52	1.18	136.7	1.5	high	8.6	7.7	0.03	33	11.1
PS	69	F	21	1	17	3.6	99	1.25	1.04	121.1	2.1	high	8.8	1.9	23.09	28	10.2

PS	41	F	20	1	19	3.7		1.97	1.41	106.6	1.5	high		4.8	0.02	24	10.3
PS	65	F	16	1	18.8	4.1	163	1.36	1.3	110.3	1.9	high	8.2	3.3			10.4
PS	70	F	14	1	18.9	3.9	156	1.76	0.85	122.5	1.9	high	11.4	4.3			9.7
PS	68	F	13	1	17.1	3.6	209	1.67	1.06	107	1.6	high	8.7	6.1			10.4
PS	43	M	13	1	25.3	3.9	160	1.27	0.78	71.09	1.3	high	8.6	4.6	0.25	28	11.1
PS	65	F	11	1	20.3	3.7	101	1.4	0.77	147.9	1.8	high	8.7	5.6	0	37	9
PS	81	F	10	1	18.5	3.5	117	1.4	1.08	116.4	2.1	high	9.1	5	2.29	30	10.3
PS	65	F	9	1	18.3	3.5	112	1.62	0.68	55.3	1.8	high	9.5	6.2	4.12	27	6.6
PS	69	F	9	1	17.8	3.8		1.46	0.99	111.8	1.5	high		4.9	0.04	24	11.7
PS	71	M	9	1	17.7	3.8		2.29	0.7	88.34	1.5	high	10	5.6	0	36	11.7
PS	66	F	8	1	17.8	3.8	127	1.2	0.84	127.8	1.5	high	9.2	5.5	0.2	29	11.4
PS	60	F	8	1	22.6	3.7	144	1.6	0.92	139.4	1.5	high	9.4	4.2	0.7	29	8.3
PS	67	F	7	1	16.6	3.5		1.51	0.85	110.5	2.1	high	10.1	6.3			12.4
PS	70	F	7	1	24.7	3.9	148	1.49	0.66	137.8	1.5	high	8.1	5.9	0.94	26	6.7
PS	70	M	7	1	22.7	3.6	161	1.44	0.76	111.2	1.5	high	9.3	5.4	0.02		12.2
PS	65	M	7	1	23.6	4.1	134	1.64	1.13	151.3	2.5	high	8.2	7.1	0.05	24	9.6
PS	66	F	7	1	19.8	3.7	167	1.61	0.88	117.4	1.6	high	8.2	4.4			10
PS	70	M	6	1	17.5	3.8	142	1.84	0.73	89.42	1.6	high	8.9	4	0.04	26	9
PS	69	M	5	1	19.5	4.1	107	1.66	0.97	128.1	1.8	high	9	6.3	0	23	9.5
PS	68	M	5	1	21.8	4.2	142	1.28	0.96	114	2.1	high		6.5			12.1
PS	58	M	5	1	24.7	3.7	179	1.45	0.98	135.5	1.8	high	9.6	5.7	0.1	24	11.9
PS	67	M	4	1	16.6	4	180	1.7	0.86	109.4	1.5	high	8.2	6	0	29	10
PS	77	M	4	1	22.6	3.7	286	1.84	0.98	97.53	1.6	high	9.4	3.9	0.13	29	9.1
PS	53	F	4	1	18.1	3.8	163	1.91	0.88	94.71	1.8	high	9.3	6.2	2.6	26	14.1
PS	53	M	4	1	18.7	3.8	127	1.25	0.72	57.57	1.8	high	9.8	6.5	0.11		
PS	68	M	4	1	21	4.2	186	1.69	0.93	135.1	1.8	high	9.5	5	0.1	23	11.7
PS	64	M	3	1	19	3.7	108	1.57	0.65	90.89	1.5	high		4.2			10.1
PS	65	M	3	1	18.3	3.6	175	1.47	0.98	76.14	1.5	high	9.1	4.7		32	10.3
PS	39	M	2	1	30.2	4.2	129	2.13	0.98	86.79	1.5	high	8.9	6.6	0	24	9.9
PS	64	F	2	1	18	4	102	1.3	0.79	82.8	1.8	high	9.8	5.9	0.2	36	8.8
PS	66	F	2	1	16.4	4.2	104	1.81	1.05	120.8	1.8	high	8.6	5.3	0	33	11.3
PS	43	F	2	1	28	4.1		1.36	0.68	46.09	1.5	high	8.4	5.5		32	11.4
PS	58	F	12	1	21.5	3.6	151	1.27	0.59	78.98	2.1	high		4.5		29	10.3
PS	69	F	21	1	20.2	4.1	158	1.37	0.68	91.01	2.2	high	8.9	4.9	0.06		12.1
PS	44	M	20	1	21	4	176	1.5	1.15	118.4	1.6	high	9.2	5.4	0.03	21	11.4
PS	75	F	2	1	29.3	4.3	186	1.25	0.71	55.72	1.8	high	8.8	6.1	0.21	30	10.2
PS	66	M	6	1	19.6	3.6	182	1.36	0.55	47.69	1.5	high	9.6	4.2	0.02	20	10.7
PS	40	M	6	1	17.3	3.6	189	1.26	1.25	141.7	1.5	high	9.4	5.4	0.03	23	9.8
PS	52	F	8	1	21.4	4	165	1.49	1.14	94.88	1.6	high	7.5	5.2	0.04	26	9
PS	68	M	12	1	21.3	3.9	173	1.45	1.01	130.7	1.6	high	9.3	4.4	1.85	25	7.1
PS	47	F	9	1	24.5	3.8	118	1.04	1.04	98.8	1.8	high	9.1	7.7	0.33	26	9
PS	51	M	8	1	21.7	3.5	203	1.76	0.8	99.81	1.6	high	8.2	4.8	0.05	32	10.7
PS	71	F	8	1	21.2	3.3	141	1.2	1.22	120.6	1.8	high	8.6	4	0.16	36	12.4
PS	59	F	26	1	19.5		164	1.34	1.17	127.1	1.6	high	8.2	3.6	0.14	20	10.3
PS	56	F	25	1	18.8		140	1.8	0.86	99.32	1.8	high	9.1	5	0.05	43	10.1
PS	65	F	22	1	16.9		146	1.42	0.88	111.7	1.8	high	9.2	6	0.03	33	8.6
PS	61	M	21	1	18.5		170	1.33	1.05	93.34	1.8	high		5.2	0.06	30	11.2
PS	65	F	20	1	16.8		182	1.63	1.12	86.09	1.9	high	10.1	6	0.05	25	9.8
PS	79	F	19	1	16.2			1.48	0.83	101	1.6	high	9.2	3.4	2.24		9.3
PS	60	F	18	1	23		130	1.29	0.87	104.9	2.5	high	10	7.2	0.1	24	11.4
PS	66	F	11	1	17.9		207	1.73	0.9	129.4	1.1	high	9.2	3.9	0.06	28	10.6
PS	66	F	10	1	24.2		189	1.35	0.99	102.4	1.9	high	9	6.2	0.06	19	9.9
PS	67	M	9	1	18.4		146	0.83	1.27	108.3	1.8	high	9.3	6.1		23	11.6
PS	56	M	7	1	21.2	4.6	155	1.7	0.86	108.5	1.9	high	9.2	4.5	0.06	27	9.8
PS	77	M	6	1	18.6		131	1.34	1.01	124.8	1.8	high	9.8	6.6			11.8
PS	44	M	4	1	17		185	2.65	0.86	93.18	1.5	high	9.9	3.8		24	11.4
PS	76	M	4	1	23		145	1.11	0.64	105.2	1.3	high	9.6	4.5			11.6
PS	63	M	3	1	22.4		202	1.51	0.72	40.93	1.9	high	10	5.5	0.1	23	10.1
PS	62	M	3	1	23.6		165	1.95	1.17	92.82	1.9	high	8.5	6.6	0.14	25	10.3

PS	61	M	2	1	23.6		134	1.25	1.03	30.74	1.8	high	8.6	5	0.14	21	11.2
PS	41	M	2	1	20.9		223	1.51	0.82	88.61	1.8	high		3.2	0.04		9
PS	64	M	2	1	24.8		209	1.43	1.02	132.7	1.9	high	9.3	3.5	0.05	29	8.5
PS	74	F	2	1	32.3		204	1.66	0.69	113.1	1.9	high	8.3	3.7	0.08	26	9.4
PS	60	M	31	1	26.2	3.3		1.23	0.85	77.17	1.6	high	8.5	6.4	0.18	24	10
PS	55	M	30	1	19.1	4.4		1.61	1.12	109.4	1.6	high	9.3	5.2	0.01	29	9.9
PS	52	M	26	1	21.1	3.7		1.68	1.02	98.81	1.6	high	9	5.3	0.14	30	10.8
PS	60	M	23	1	18	3.7	174	1.54	0.99	101.6	1.9	high	10	4.5	4.34	27	10.1
PS	52	M	19	1	21.3	3.7	117	1.34	0.98	103.6	1.5	high	9.1	2.7	0	25	9.7
PS	48	M	14	1	19.7	4.3	177	1.46	0.94	87.16	1.9	high	8.8	4.7	0.15	29	11.2
PS	69	F	11	1	21.4	2	149	1.83	0.96	129.8	1.9	high	9.7	8.6	0.06	44	10.7
PS	61	F	11	1	16.1	3.7	194	1.41	0.92	96.53	1.9	high	8.7	4.9	0.05	27	11
PS	74	M	9	1	25.6	3.8	130	1.59	0.63	119	1.9	high	9.6	6.2	2.23	33	10.2
PS	51	M	7	1	29.3	3.9	177	1.1	0.86	116.6	2.1	high	8.5	6.2	0.01	24	11.9
PS	58	F	7	1	20.5	4.3	172	1.4	1.16	85.44	1.9	high	9.2	7.3	0.11	30	10.4
PS	58	F	6	1	23.2	4.3	168	1.25	0.78	98.49	1.5	high	9.2	4.3	3.5	44	10
PS	64	F	6	1	22.8	4.3	174	1.67	0.8	57.21	1.3	high	8.4	6.6	2.88	24	10.2
PS	43	F	5	1	20.1	4.5	137	1.83	0.93	111.1	1.5	high		4.3	0.05		10.1
PS	56	F	4	1	28.2	4.1	159	1.71	0.88	145.4	2.1	high	10.3	4.5	0.05		15.2
PS	64	F	4	1	19.3	3.2	126	1.38	0.92	82.17	1.5	high		4.5	0.33	33	10.7
PS	76	M	4	1	21.5	3.8	178	1.75	0.88	98.32	1.3	high	9.1	5.8	0.04	13	10
PS	68	M	3	1	25.3	1.9	143	2.22	1.1	148.4	1.3	high	9.6	4.8	0.04	22	10
PS	61	M	3	1	28.6	4.2	111	1.24	0.93	129.3	1.3	high	8.8	7.2	0.24	31	11.6
PS	66	F	18	1	21.3	3.8	101	1.91	0.78	98.42	1.3	high	9.7	4.2	0.06	92	9.2
PS	43	M	5	1	40.6	3.9	153	1.62	0.85	80.31	2.1	high	10.1	6	0.01	28	11.2
PS	65	M	2	1	23	3.5	132	1.12	0.99	63.92	2.1	high	8.9	5.8	0.07	31	10.1
PS	73	F	6	1	18.6	3.5	131	1.13	1.05	103	1.4	high	9.1	5.3	0.09	30	10
PS	69	F	8	1	19.4	4.5	239	2.28	1.02	131.3	1	high	8.9	4.9	0.05	30	11
PS	76	F	7	1	17.6	4	147	1.83	1.05	113.3	1	high	8.5	4.9	0.06	28	11.1
PS	48	F	20	1	18.2	3.4		1.67	0.98	112.5	1.8	high	9.8				9.3
PS	43	F	8	1	21.3	3.5	91	0.79	1.4	109.2	1	high		3.5	0.13	29	9.9
PS	70	F	3	1	27.6	3.7	108	1.04	0.61	63.17	2.1	high	10.4	9.3	0.56	34	12.6
PS	84	F	7	1	18.4	4	189	1.91	1	100.8	1.5	high	9.5	3.8	1.01	28	10.1
PS	58	M	8	1	19.6	4.1	114	1.53	0.93	102.3	1.6	high	9.1	5.1	0.21	26	11.2
PS	67	M	5	1	16.1	3.6	217	1.09	0.88	85.16	1.5	high	7.6	7.3	0.02	33	10.5
PS	70	F	5	1	20	3.6	157	1.64	1.16	122.3	1.5	high	10	5.1		20	8.9
PS	60	M	3	1	23.6	4.3	134	1.56	0.82	97.29	2.1	high	10.2	3.9	0	29	9.8
PS	79	F	10	1	25.6	3.1	204	1.72	0.86	146.1	1.3	high	9.2	4.8	0.05	32	10.2
PS	55	F	33	1	16.3	3.7	139	2.12	1.47	88.6	1	high	9.1	4.5	0.03	31	9.3
PS	68	F	23	1	19.6	3.9	113	1.33	1.01	129.4	1.8	high		5	0.05		8
PS	77	F	10	1	25.9	3.7	105	1.28	1.05	111.3	1.8	high	8	6.4		31	9.8
PS	75	M	8	1	25.9	4	150	1.58	0.65	116.7	1.3	high	6.3	5.2		23	10.8
PS	81	M	8	1	21.3	3.2	170	0.7	0.73	134.9	2.5	high		6.3		30	10.3
PS	69	M	7	1	27	4	168	2.1	0.7	104	1.3	high	10.2	5.4	0.05	25	10.9
PS	73	M	7	1	21.1	3.5	116	1.55	0.84	127.3	0.8	high	9.1	4.4		22	10.4
PS	58	F	4	1	25.6	4.4	150	1.17	0.97	87.39	1.5	high	8.8	6	0.05	26	10.4
PS	68	M	3	1	22.1	4.1		1.58	0.93	121	1.8	high	8.9	4.8		20	12
PS	79	F	2	1	20.3	3.5		1.47	0.79	110.2	1.5	high	9.8	3.9	0.1	22	11.9
PS	73	M	2	1	22	3.7	99	1.24	0.89	117.1	2.1	high	9.2	4.4	0.81	29	10.5
PS	68	M	19	1	19.8	4.1	184	1.98	1.08	107.7	1	high	9.4	4.5	0.02	27	11.1
PS	40	M	18	1	21.3	3.7	98	1.4	0.8	107.6	1.8	high	9	5.9	0.8	25	10.3
PS	63	M	12	1	21.8	4	189	1.95	1.07	110.2	1	high	9.2	3.7	0.02	28	9
PS	64	M	10	1	22.1	3.8	135	1.71	0.81	130.6	1.3	high	9.6	5.2	0.11	28	10.3
PS	70	F	9	1	19.3	3.6	180	1.18	1.02	138.9	1.5	high	8.7	6.4	0.04	29	9.2
PS	65	F	9	1	22.2	3.5	188	1.83	1.03	85.16	1	high	9.5	5	0.06	24	10.3
PS	57	F	8	1	21.5	3.8	154	1.57	0.71	113.1	1.8	high		4		21	9
PS	61	F	8	1	20.9	3.8	174	1.25	0.81	137.5	1.3	high	7.6	4	0.8	35	10.3
PS	33	F	8	1	23.8	3.8	166	1.62	0.93	111.2	1.1	high	9	4.6	0.07	25	10.8
PS	54	M	7	1	22.1	4.2	155	1.49	1.1	106.8	1.8	high	9.3	5.8	0	23	11.7

PS	64	F	7	1	16.5	3.4	191	1.63	0.83	100.7	1	high	9.3	5.1	0.02	24	11.5
PS	65	M	6	1	19.8	3.9	162	1.49	0.92	79.78	0.8	high	8.5	3.3	0.47	39	10.7
PS	60	F	5	1	19.9	3.8	161	1.28	1.01	79.97	1.5	high	10	5.6		25	10.1
PS	36	M	5	1	23.6	4.2	115	1.78	0.8	101.4	1.3	high	9.1	3.1	0.03	34	10.7
PS	61	M	5	1	25.9	3.4	156	1.2	0.7	108	1.3	high	9.7	5.5	0.03	18	9.8
PS	47	M	4	1	20.1	3.7	175	1.36	0.88	89.11	2.1	high	10.4	7.7		16	9.7
PS	54	F	4	1	29.4	3.6	225	1.23	0.68	80.69	1.8	high	10	5.9	0.1	25	
PS	64	M	3	1	21	4.2	183	1.42	0.93	123.9	1.8	high	8.9	4.2	0.26	31	11.7
PS	71	M	2	1	22.6	3.6	130	1.32	0.76	59.34	2.1	high		5.5		27	10.3
PS	54	M	2	1	24.5	4.1	150	1.67	0.6	58.36	1.7	high	9.4	5.1		50	10.5
PS	69	M	5	1	21.5	3.9		1.2	0.74	101.9	1.1	high	9.5	4.5			10
PS	80	F	2	1	21.1	3.3	254	2	0.76	110.7	1.6	high	9.5	4	0.04	23	10.3
PS	53	M	6	1	30.1	3.6	121	1.67	1	116.2	1.6	high	9.8	5.7	0.1	33	10.8
PS	76	F	21	1	18	3.6	120	1.91	1.13	82.07	2.1	high	9.1	3.8	0.04	21	10.3
PS	66	F	2	1	17.9	3.7	195	2.25	0.64	70.1	1.3	high	10.5	3.9	0.05	35	10.7
PS	69	M	7	1	18.4	4.3	174	1.99	1.07	120.1	1.8	high	8.6	4.9	2.77	34	8.8
PS	62	F	27	1	22.9	4	140	1.04	0.91	96.61	2.1	high		6.9	0.05		10.6
PS	61	M	17	1	19.6	4.2	128	1.63	0.99	102.6	2.1	high	9.5	3.1	0.39	27	10
PS	48	M	12	1	19.7	4.3	167	1.89	0.89	93.89	1.6	high	9.7	4.7	0.04	26	10.5
PS	65	M	12	1	21.5	4.2	228	1.39	0.85	130.6	1.8	high	9.2	4.5	0.28	22	11.6
PS	57	M	8	1	20.3	4	153	1.49	0.77	103.7	1	high	9.1	5.7	0.02	29	10.7
PS	42	M	3	1	25	4.4	180	1.71	1.14	98.9	1.6	high	9.7	4	0.1	17	10.6
PS	54	F	3	1	20.3	3.9	166	1.35	0.9	111.7	1	high	8.3	4.3	0.02	21	11
PS	73	M	2	1	21	4.1	200	1.8	1.02	129.2	1.3	high	9.8	4.3	0.04	31	10.8
PS	53	M	3	1	38	4	148	1.35	0.98	114	1.5	high	8	3.2		40	9.7
PS	61	M	26	1	19.1	3	138	1.53	0.93	60.93	0.6	high	8.8	4.8	0.34	36	11.4
PS	57	M	18	1	18.1	3.9	110	1.63	0.84	105.4	1	high	7.9	4.8	0.24	35	10.7
PS	62	M	13	1	23.5	4	201	1.9	0.73	108.6	1.6	high	7.9	3.2		35	10.8
PS	59	M	11	1	21.3	3.9	168	1.21	0.87	121.7	2.1	high	9.6	6	0.02	24	10.4
PS	67	F	8	1	16.8	4.3	209	1.79	1.09	99.01	1	high	9.1	6.8	0.06	26	10.5
PS	66	M	6	1	25.8	2.9	165	1.27	0.85	117.7	1.8	high	8.4	8.4	0.29	33	10.5
PS	65	M	5	1	19.9	4.4	155	1.99	0.97	92.08	1.3	high	8.8	3.3	0.11	29	9.7
PS	68	M	4	1	21.4	4.2	190	1.42	1.28	108.5	2.1	high	9.2	6.2	3.58		15.2
PS	71	F	3	1	18.4	1.7	138	1.28	1.08	95.98	1.5	high		6.5	0.16		11.2
PS	73	M	2	1	22.5	3.9	150	1.12	0.73	121.7	1.8	high		3.8	0.11	13	12
PS	64	M	22	1	21.6	3.9	268	1.53	1.01	111	1	high	9.8	5.1	0.2	28	10.2
PS	74	M	2	1	20.8	3.6	149	1.16	0.78	95.75	1.8	high	8.5	8	0.1	30	8.5
PS	68	M	6	1	22	3.4	146	1.23	0.91	111.7	0.6	high	7.8	4.9	0.18	20	10.9
PS	52	M	35	1	17.9	4.5	201	1.27	0.8	92.34	1.8	high	8.8	5.8	0.6	18	11.1
PS	58	M	35	1	21.9	4	158	2.02	1.1	97	1.8	high	8.6	8	0	24	10.3
PS	58	M	33	1	19.8	4.2	131	1.32	1.12	86.11	1.5	high	8.3	5.4	0.8	31	11.7
PS	55	M	33	1	23.4	3.9	117	1.72	0.86	109.7	1.8	high	8.9	4.6	0	27	10.8
PS	59	M	33	1	19.9	4.1	112	1.23	0.97	105.4	1.8	high	9.7	3.5	0.1	24	9.9
PS	51	F	32	1	20.1	3.6	191	1.23	1.44	115.1	2.2	high	9.2	7.5	0.05	29	12.3
PS	53	M	32	1	20.1	3.6	128	1.48	0.75	74.73	1.3	high	9.4	5	0.03	23	11.1
PS	57	M	31	1	22.1	4.1	127	1.27	1.18	95.97	2.2	high	10.3	7.7		26	12.6
PS	80	F	31	1	18.3	3.8		1.48	0.99	99.98	1.8	high	9.2	4.8	0.04	20	9.5
PS	67	M	31	1	17.5	3.7	160	1.29	0.84	82.9	1.9	high	8	4.5	0.15	26	8.9
PS	57	F	30	1	19.9	3.8	137	1.25	0.88	111.2	1.8	high		6.9	0.14	28	10
PS	52	M	29	1	20.5	3.9	166	1.39	1.1	92.8	1.8	high		6.8	0.09	28	9.9
PS	59	F	29	1	20.6	4	195	1.5	1.18	105.4	1.9	high	9.1	5.8	1.71	26	8.2
PS	56	M	29	1	17.3	4	135	1.07	1.49	95.32	1.8	high	10.7	6	0.11	31	10
PS	56	F	32	1	19.7	4.7		1.57	0.89	91.52	1.8	high		5.5	0.06	23	12.2
PS	57	M	29	1	17.6	3.6	150	1.49	1.15	85.94	1.5	high	9.7	4.4	0.2	30	11.9
PS	56	M	28	1	16.7	3.8	129	1.27	0.93	87.39	1	high	9.6	6		31	9.6
PS	61	F	28	1	21.3	3.6	94	1.05	0.9	98.4	2.2	high	10.6	6.2		35	10.2
PS	59	M	28	1	19.7	3.7	144	1.23	0.93	96.34	1	high	9.2	4.9		26	9.9
PS	52	F	27	1	18.3	3.7	140	1.22	1.4	111	2.1	high	8.4	6.1	1.75	26	11.2
PS	60	M	27	1	28.7	3.7	114	1.4	1.16	115.7	1.5	high	9.3	6	1.1	24	9.5

PS	72	M	26	1	18.2	3.7	190	1.81	0.97	77	1.9	high	10.5	7.2	0.05	26	8.6
PS	56	M	25	1	22.2	4	125	1.18	1.15	86.44	1	high	8.1	4.4		28	10
PS	53	M	25	1	18.2	4.3	111	1.53	1.31	92.45	1.9	high	8.5	3.4			
PS	50	M	25	1	19	3.8	139	1.47	0.97	111.1	1.9	high	9.1	5.2	0.05	29	9.5
PS	50	F	24	1	20.9	3.8	188	1.52	1.22	104.6	1.6	high		6.2	0.06	29	10.9
PS	87	F	24	1	15.6	3.7	166	1.38	0.85	116.9	1.9	high	8.7	5.8	0.12	26	12.1
PS	69	F	24	1	15.4	3.9	138	0.86	1.08	102.8	1.9	high	8.9	3.8	0.05	17	9.3
PS	79	F	23	1	20.4	3.9		1.62	0.76	75.92	1.3	high	9.3	7.5	1.79	22	11.2
PS	72	F	23	1	21.2	3.4	134	1.59	0.98	82.63	1.5	high	8.8	5.8	0.6	23	8.9
PS	55	M	23	1	18	4	167	1.02	0.98	130.4	1.4	high		6.7	0.82	28	7.8
PS	74	M	22	1	19.5	3.6	184	1.61	0.99	106.1	2.1	high	9.8	7	0.3	28	11.1
PS	42	M	21	1	22.4	3.5	120	1.31	0.56	78.42	1.8	high	10.1	8.9	0.29		9.5
PS	50	F	21	1	20.6	3.8	169	1.38	1.18	100.7	1.3	high	9	5.2	0.21	23	11.2
PS	62	F	19	1	20.1	3.9	89	0.97	0.83	106.1	1.9	high	7.7	5.3	0.05	20	9.4
PS	65	F	19	1	17.7	3.7		1.23	1.13	114	1.8	high	9.4	5.7	0.37	32	11.6
PS	54	M	18	1	17.5	3.9	160	1.24	0.98	119.3	1.5	high	10	4.2	0.05	21	11.1
PS	40	F	17	1	21.6	4.1	137	1.55	1.14	114.9	1	high	8.8	4.9	0.08		9
PS	47	F	17	1	29.3	4.1	146	1.67	0.99	133.8	1.8	high	9.2	6.7	0.1	24	12.8
PS	50	M	17	1	24	4	190	1.51	1.02	122.5	0.6	high	8.6	3.2	0.07	28	9.9
PS	59	M	15	1	22	4.2	147	1.55	1	117.1	1.3	high	9.3	3.9	0.04	20	11.5
PS	41	M	14	1	27.1	3.9	111	0.82	1.08	100.4	2.2	high	6	5.9	0	19	11.4
PS	43	F	13	1	23.4	3.6	195	1.61	0.77	83.23	1.5	high	9.5	4.9	0.1	25	13
PS	58	M	11	1	19.2	3.5	172	1.51	1.03	106.3	1.3	high	8.4	5	1.21	22	11.9
PS	72	M	10	1	23.5	3.9	112	1.81	1.13	123	2.1	high	10.6	4.1	0	26	8.6
PS	41	M	10	1	19.7	4.1	121	1.58	1.11	84.8	1.3	high	8.9	5.7	0.56	28	11.3
PS	67	M	8	1	19.7	4	163	1.49	1.15	131.3	2.2	high	9.3	7.5	0.05	27	10.9
PS	67	M	8	1	20.9	3.9	147	1.43	0.89	120.1	1.8	high	9.2	7.8	0.28	19	10
PS	54	F	7	1	17.9	3.8	182	1.93	1.11	108.3	1.3	high	8.9	5	0.47	16	11
PS	86	M	7	1	23	3.8	117	1.4	0.95	115.1	1	high	8.6	4.8	0.03		8.7
PS	81	F	7	1	18.6	3.9		1.4	1.08	117.1	0.9	high	9.2	5.9			11.2
PS	25	M	6	1	26.2	3.9	167	1.56	1.17	69.9	1	high	8.9	5.9	0.09		10.4
PS	52	M	6	1	23	4.2	219	1.69	0.92	117.7	1.3	high	8.8	5.2		21	8.4
PS	29	F	6	1	30.8	3.8	107	1.51	1.06	107.5	1.6	high	8.8	1.5	3.52	27	5.9
PS	77	M	6	1	22.8	4	106	2.24	0.74	105.9	2.1	high	9.7	3.1		20	10
PS	27	M	5	1	18.4	4.3	121	1.58	1.23	105	1.3	high	9.3	3.4	0.02	24	10.2
PS	63	F	5	1	21.8	3.4	135	1.38	0.81	67.09	1.3	high	9.8	3.7	0		9.7
PS	67	M	5	1	23	3.9	189	1.82	1.04	93.85	1.8	high	9	5.6	0.03	31	10.8
PS	51	M	5	1	16.4	4	203	1.36	1.2	71.33	1	high	12.4	4.1	1.15		10.1
PS	43	F	5	1	25.7	3.8	271	1.05	0.75	118.4	1	high	10	4.7	0		7
PS	41	M	5	1	19.2	4.5	108	1.86	0.85	92.23	1.5	high	9	4	0.1	21	11.3
PS	52	M	4	1	28	4	134	2.25	0.78	135.6	1.3	high	9	4.9	0.04	23	9.8
PS	60	M	4	1	21.5	3.8	128	1.17	0.96	81.51	2.1	high	9.5	5		22	9.2
PS	74	F	3	1	21.9	3.8	138	0.77	0.79	97.98	1	high	9.2	6.7	0.08		8.6
PS	83	F	3	1	16.3	3.9	151	1.71	1	38.14	1.8	high	9.2	5.7	0.06	25	14.9
PS	66	M	3	1	26.6	4.1	241	1.96	0.91	91.91	1.5	high	8.4	5.5	1.5	27	11.7
PS	76	M	2	1	24.7	3.9	126	1.57	0.79	95.26	1.3	high	8.6	4.5	0.11	24	9.9
PS	62	M	2	1	23.6	4.1	194	1.89	0.85	83.66	1.5	high	9.7	6	0.4	22	10.9
PS	42	M	2	1	19.2	3.9	138	0.92	0.89	91.28	1.5	high		4.3	0.3	33	10.5
PS	69	M	17	1	23.1	3.7	126	1.45	1.05	101.6	2.5	high	10.7	5.3	0.03	29	10.8
PS	71	M	12	1	19.3	3.5	114	1.43	1.07	98.65	1.5	high	10	5.1	0.27	20	9.1
PS	63	M	10	1	18.2	4	171	1.38	0.8	103.4	2.1	high	10.1	6.2	0.3	23	9.6
PS	71	F	7	1	20.4	3.5	198	1.97	0.84	142.2	1.5	high	9	3.5		26	8.8
PS	72	F	7	1	20.6	4.1	159	1.52	1.04	125.1	1.5	high		5.1	0.01	26	11.2
PS	65	F	7	1	22.1	3.1	225	1.77	0.93	111.1	1.5	high	8.8	5.3	0.14	31	9.3
PS	59	M	7	1	23.5	3.5	166	1.18	0.73	112.8	1.3	high	9.7	4.8	0.21	31	12
PS	61	M	6	1	19.6	3.9	153	1.48	0.74	118.8	1.8	high	8.5	3.1	1.6	23	8.9
PS	80	M	5	1	22.9	3.8	142	0.98	0.83	53.78	1.5	high		6.5	2.97	31	11
PS	77	F	4	1	22.1	3.5	224	1.98	1.05	124.4	1.5	high	9.4	7.7	0	28	10.8
PS	65	F	4	1	26.6	3.8	202	1.87	1.02	113.1	1.5	high	8.8	5.1	0.26	29	10.6

PS	71	M	4	1	21.4	3.4	99	1.46	0.87	97.41	1.5	high		4	0.02	32	8.9
PS	69	M	4	1	25.9	3.7	121	0.99	0.79	138.6	2.1	high	8.3	7.6		29	8.1
PS	66	M	4	1	23	3.3	141	1.16	0.53	65.93	1.6	high	9.1	2.7	0.05	30	9.8
PS	54	M	3	1	22.2	3.9	170	1.28	0.66	93.92	1.3	high	9	5.2	0.2	26	11.1
PS	55	M	3	1	22.3	3.7	151	1.36	0.87	133.1	1.5	high	8	5.6	0.2		9.8
PS	66	M	2	1	21.3	3.9	136	1.61	0.6	104.7	1.8	high	10.3	4	0.52	26	8.2
PS	36	F	2	1	18	3.8	165	2.08	0.92	97.39	1.5	high	10	5.1	0.2		9.7
PS	69	F	2	1	23	3.5	135	0.69	1.05	97.09	1.5	high	8.6	11.1	3.32	23	8.9
PS	54	M	2	1	22.7	4	182	0.89	0.78	68.65	2.1	high	9	4.1	0.4		9.8
PS	74	F	11	1	26.1	3.4	119	1.42	0.87	122.8	2.1	high	10.6	3	0.06	20	12.7
PS	73	M	18	1	24.2	3.7	148	1.25	0.6	100.4	2.2	high		5.2	1.29	27	11.6
PS	68	M	16	1	17.6	3.9	116	1.49	0.96	128.2	1.3	high	7.9	5.6	0.21	42	8.1
PS	52	M	15	1	21	3.7	145	1.72	1.24	115.9	1.3	high	9	3.9	0.12	24	9.4
PS	53	M	15	1	21.9	3.6	128	1.38	1.11	115.3	1.3	high	8.5	4.8	0.05	22	10.5
PS	72	M	12	1	22.6	3.7	176	1.42	0.95	129.6	1.3	high	8.7	6.2	0.12	22	10.1
PS	63	M	10	1	26.7	3.6	135	1.75	0.97	102	1.3	high	8.9	4.8	0.04	25	10.2
PS	52	F	9	1	19.7	3.8	99	1.57	1.11	108.7	1.3	high	9	2.6	0.03	29	10.2
PS	49	M	9	1	23.1	3.5	125	1.3	0.94	102.1	1.3	high	10.3	7.3	0.13		10.3
PS	67	F	8	1	19.2	3.5	123	1.04	1.08	77.34	1.3	high	7.8	6.7	1	36	9.4
PS	66	M	6	1	24	3.9	152	1.42	1.17	123.5	1.3	high	8	2.8	0.11	18	10.4
PS	70	F	5	1	19.6	3.5	143	1.86	1.18	130.3	1.1	high	9.5	6	2.9	18	12.1
PS	54	M	5	1	20.9	4	197	1.34	0.95	61.94	1.8	high	8.3	6.6	0.05	19	10.6
PS	61	M	5	1	23.8	3.8	145	1.31	0.89	127.4	2.1	high	8	7.7	0.66	34	9.1
PS	40	F	4	1	19.3	4.1	141	1.55	1.27	128.6	1.3	high	8.7	4	0.02	23	9.7
PS	81	M	4	1	24.2	3.2	107	1.55	0.93	89.17	1.8	high	9.6	4.9	0.7	25	12.3
PS	61	F	4	1	20.8	3.7	175	1.33	0.94	110.6	2.1	high	8.2	6.3		27	12
PS	71	M	4	1	19.7	3.6	157	1.39	1.02	122.8	1.6	high	8.8	5.6	0.2	16	7.1
PS	66	M	3	1	25	3.6	102	1.39	0.92	117.1	1.9	high	9.2	5.1	0	46	10
PS	58	M	3	1	23.3	4.1	134	1.3	0.72	97.96	2.1	high		7.1	0.9	19	8.8
PS	60	M	3	1	19.3	3.8	128	0.94	1.03	116.3	1.3	high	8.1	4.6	0	22	8.8
PS	58	F	7	1	22.4	4	139	1.42	1.08	105.2	1.8	high	8.7	6.2	0	23	10.5
PS	54	M	14	1	20.6	4	121	1.13	0.69	125.9	1.8	high		5.1	0.6	34	10.9
PS	81	M	13	1	21.5	3.8	199	1.1	1.29	122.3	1.5	high	10.4	5	0	32	10.6
PS	28	M	12	1	20.6	4.6	152	1.62	1.08	89.82	2.1	high	9.6	5.4	0.02	31	9.7
PS	52	F	11	1	18.2	4		1.78	0.74	78.43	1.8	high	9.2	5.3	0.05	29	10.3
PS	66	M	11	1	25.5	3.7	133	0.99	0.7	101.3	1	high	8.8	5.1	0.05	13	
PS	56	M	10	1	21.2	3.5	199	1.63	1.36	118.6	1.8	high		4.7	0.15	26	9.7
PS	61	F	10	1	15.4	4	111	1.61	1.17	102.2	2.2	high		6.1	0.03	24	11
PS	52	F	4	1	25.8	3.6	104	1.19	0.71	96.96	2.1	high	9.8	4.8	0.22	26	11
PS	46	F	3	1	23.7	4.2	129	1.58	0.95	110.4	1.8	high	9.6	3.4	0.31	22	9.2
PS	79	M	2	1	19.1	4.3	189	1.52	0.9	104.8	1	high	9.5	5	0.51	28	9.6
PS	59	F	23	1	26.5	3.2	95	1.83	0.96	114.8	1.8	high	8.8	5.3	0.01	37	10.3
PS	61	M	2	1	28.8	3.9	222	1.28	0.73	95.68	1.8	high	9	3.9	0.1	29	10.3
PS	50	F	25	1	17.7	3.6	172	1.14	0.86	91.76	1	high	8.7	4.7		32	10.2
PS	54	F	4	1	20.9	3.5	158	1.83	0.99	98.15	1.3	high	9.9	5.7		42	12.3
PS	64	M	2	1	19.7	3.4		1.25	0.98	124.5	1.8	high		6.2	0.22	24	11.5
PS	79	F	3	1	17.2	3.4	155	1.14	0.75	102	1.5	high	8.4	5	0.06	19	8.9
PS	55	F	30	1	23.4	3.6	110	1.28	0.99	108.3	1.9	high	8.1	4.7	0.08	27	8.2
PS	62	F	8	1	19.1	3.5	152	1.13	0.97	93.41	1.6	high		5.2	0.67	25	9.5
PS	72	M	7	1	22.1	4	195	1.9	0.81	95.89	1.6	high	9.8	8.7	0.02	26	9.5
PS	51	F	29	1	23	3.4	217	2.05	0.71	99.91	2.2	high	9.3	6.4			10.1
PS	82	M	14	1	23	3.7	143	0.99	0.8	115.3	1.5	high	8.6	5.7	0		11.1
PS	68	F	8	1	24.4	2.8	133	1.24	1.03	121	2.1	high	9	1	0.33		11.6
PS	32	M	2	1	34.5	3.6	163	1.6	0.93	119.4	2.1	high	9.6	4.3	0.3	31	7.6
PS	58	M	6	1	29.5	3.7	152	1.87	0.97	93.83	2.1	high	8.4	4.3	0.03		11.3
PS	71	F	15	1	17.3	3.2	152	1.5	0.74	78.99	1.3	high	9.9	5.5	0	23	10.2
PS	70	M	14	1	22.7	3	155	1.55	0.91	130.1	1.8	high	8.9	4.3	0.34	28	9.7
PS	56	M	10	1	18.7	3.7	210	1.24	0.79	78.93	2.1	high	8.6	6.5			9.9
PS	69	M	18	1	19.6	3.6	120	1.59	0.66	89.95	1.6	high	9.6	4.5	0.2	21	11.5

PS	46	F	17	1	22.9	3.7	162	1.43	1.04	114.5	1.5	high	8.5	4.8			8.9
PS	63	M	2	1	22.6	3.4	104	1.07	1.2	136	1.5	high	8.3	4.7		22	10.7
PS	64	F	26	1	19	3.4	112	1.27	1.31	103.2	2.2	high	8.5	6			
PS	60	M	8	1	20.4	3.1	79	1.46	0.69	110.3	1.3	high		7.1	0.06	42	9.6
PS	59	M	6	1	23.7	3.6	97	1.9	0.99	114.7	1.8	high	8.6	3	1.41	26	8.2
PS	63	F	5	1	21.9	3.6	170	1.6	0.64	97.59	1.6	high		5.9		40	10.3
PS	60	F	18	1	21.7	3.8	121	1.6	1.12	120.3	1.5	high	8.4	5.3			11.4
PS	45	F	14	1	17.4	3.5	149	1.44	0.83	98.86	2.1	high	9.4	5.1	0.02	25	10.9
PS	73	M	6	1	20.6	3.5	161	1.51	0.71	93.48	1.5	high	8.9	5.8	0.19	21	9.6
PS	57	F	4	1	28.8	4	131	1.27	0.72	98.93	2.1	high	8.3	6.8	0.16	38	10.5
PS	52	M	3	1	27.9	3.4	126	1.25	0.99	83.55	1.5	high	9.6	4.3	0.01	24	11.6
PS	70	M	2	1	21.1	3.4	141	0.95	0.98	44.4	2.1	high	9.1	6.8	0.03	22	11.5
PS	60	F	2	1	14.8	3.4	152	1.87	0.75	45.96	1.3	high	8.7	5.8	0.03	25	9.6
PS	76	F	6	1	20.1	3.2	130	1.56	0.69	111.7	2.1	high	11.2	8.3	0.26		12.1
PS	70	M	10	1	28.4	3.1	141	1.48	0.67	111	1	high	8.4	4.9		31	10.7
PS	67	M	7	1	25.9	3.3	122	1.41	0.76	113.8	1.5	high		3.6		27	9.6
PS	84	F	5	1	23.3	3.3	145	1.01	0.82	137.7	1.6	high	8.7	4.3	0.87		10.4
PS	55	F	12	1	20.3	3.7	146	1.36	1.19	122	1.5	high	9.1	5.4		24	12.1
PS	61	F	8	1	21.6	3	161	1.28	0.73	82.19	1.5	high	9.1	6.1	1.99	26	10.5
PS	57	F	5	1	21.9	3.6	212	1.65	0.74	84.79	1.5	high		4.2	0.18	30	11
PS	71	M	3	1	20.5	3.6	168	1.21	1.05	92.45	1.5	high	8.7	5.1	0.23	30	15.2
PS	82	F	5	1	20.6	3.1	166	1.56	0.99	152.1	1.9	high	8.8	4.2	0	27	10.3
PS	50	M	3	1	16.6	3.9	167	1.89	0.98	122	2.1	high	9.6	5.8	0.07		11.1
PS	89	M	3	1	22.7	3.5	145	0.86	1.06	120.5	1.5	high	8.8	3.3	0.22	26	10.1
PS	54	F	10	1	18.7	3.4	126	1.17	0.87	121.6	1.3	high	9.5	5.9	0.9	22	10.4
PS	58	F	3	1	23.3	3.9	135	1.43	0.92	106.3	2.1	high	9.5	4.6	1.06	25	9.5
PS	64	F	3	1	21.3	4	137	1.46	1.03	138.9	1.9	high	9.9	3.9	0.1	14	9.4
PS	47	M	11	1	16.9	3.5	169	1.94	0.69	62.55	1.8	high	9.8	4.7		27	12.1
PS	66	F	4	1	19.7	3.8	168	1.58	1.03	94.19	1.8	high		7.1	0.12	33	10.3
PS	64	F	9	1	25	3.9	215	1.41	0.96	93.24	1.3	high	8.4	6.1	0.22	29	10.4
PS	73	M	20	1	23.4	3.2	147	1.12	0.83	90.08	1.4	high	8.5	4.5	0.1	26	9.1
PS	55	F	15	1	15.5	3.5	112	1.12	0.6	94.04	2.5	high	8.9	5.6	0.03	27	11.7
PS	48	M	9	1	21.5	3.5	155	1.97	0.97	108.1	1.3	high		4.1	0	16	9.7
PS	55	M	9	1	23.7	3.9	158	1.38	0.83	115.3	1.5	high		6.5	0.04	29	11.8
PS	52	F	8	1	14	2.8	159	1.56	1.07	64.55	1.8	high	10.2	7.1	0.1	28	11.1
PS	72	M	7	1	20.3	4	120	1.29	1.04	134.8	2.1	high	9	5.1	0.08	28	10.6
PS	39	M	7	1	16.4	3.5	74	1.26	0.92	76.01	1.8	high	8.9	4.4	3.21	30	5.6
PS	69	F	6	1	20.4	3.3	116	1.5	0.7	91.96	1.5	high		3.5	0.5	31	8.5
PS	74	F	6	1	16.7	3.9	172	1.34	1.03	123.2	1.3	high	9.6	4.7		19	10.5
PS	72	M	5	1	21.8	4	176	1.07	0.89	138	2.2	high		5.1	0.04	22	11
PS	84	F	5	1	20.4	3.4	171	1.25	0.82	104.2	1.5	high	8.7	4.1		29	11.3
PS	71	M	4	1	17.7	3.5	176	1.57	0.73	67.47	1.5	high	8.4	5.8		32	11.3
PS	47	M	4	1	31.1	3.4	133	1.3	0.74	77.67	2.1	high		6.2	0.12	33	12
PS	66	F	3	1	21.5	3.7	102	1.01	0.86	82.26	1.8	high		6.2		23	11
PS	91	F	3	1	22.6	3.6	199	1.45	0.77	91.54	1.5	high	9.8	7	0.12	20	10.7
PS	72	M	3	1	20.4	3.1	124	1.75	0.84	90.86	1.5	high	9.6	4.7	0.05	17	10.8
PS	71	M	2	1	21.8	3.7	155	1.5	1.01	76.12	1.8	high		5.1		23	9.7
PS	58	M	2	1	23.8	3.9	133	1.2	0.87	75.51	1.6	high	9.9	4.1			10.4
PS	54	F	2	1	22.4	3.6	243	1.58	1.01	105.8	1.3	high	9.2	5.2		26	11.1
PS	54	M	2	1	22.7	4.1	203	1.75	0.94	130.7	1.5	high	9.2	5.5	0.09	23	9.6
PS	80	F	8	1	19.4	3.9	162	1.72	1.29	100.8	1.5	high	8	4.6	0	25	10.1
PS	48	M	16	1	22.8	3.8	174	1.16	0.89	100.2	2.5	high		4.2	0.29	25	9
PS	50	F	19	1	21	4.1	138	1.27	0.95	102.8	1.5	high		8.6	0.23	31	9.9
PS	78	M	17	1	18.4	4.1	111	1.95	0.83	120	1.5	high	9.6	6	0.03	24	9.9
PS	63	F	6	1	18.9	3.2	134	1.61	1.19	78.15	1.5	high		5.2			8.8
PS	27	F	5	1	16	4		1.08	1.1	102.4	2.1	high	9.2	5.3	0.1	21	10
PS	46	F	5	1	21.8	3.6	179	1.66	1.06	105	1.5	high	8.7	4.6	0	32	10.4
PS	61	M	22	1	23.4	3.8	150	1.39	0.77	102.7	1.5	high	9.5	4.5	0.19	27	8.7
PS	57	M	24	1	20.5	3.4	167	1.59	1.03	107.6	1.8	high	9.3	5.9	0.05	32	9.1

PS	54	M	28	1	20.9	3.6	176	1.54	0.91	101.7	1	high	9.5	6.2	0.1	28	9.2
PS	51	M	26	1	20.9	3.7	144	1.41	1.38	91.42	1.8	high	10.4	6.2		29	10.8
PS	61	M	16	1	18.9	3.5	137	1.7	0.96	121.5	2.1	high	9.4	5.7	0	23	9.5
PS	43	M	9	1	20.2	3.6		1.58	1.08	106	2.1	high	9.6	6.4		33	8.1
PS	54	M	2	1	17.6	4.3	135	1.72	0.75	79.09	1.8	high	9.5	5.5	0.07	28	10.9
PS	55	M	14	1	20.8	3.9	139	1.71	1.19	114	2.1	high	8.8	6.6	0.21	28	11
PS	76	M	9	1	19.2	3.5		2.05	1.03	139.3	1.5	high	9.3	4.2	0.12	29	10.3
PS	71	F	16	1	22.4	3.3	131	1.14	1.09	131.3	1.4	high	8.5	5.2		42	10.7
PS	64	M	6	1	24.2	3.8	117	1.18	1.18	117.8	2.1	high	10.7	4.5	0.05	25	10.9
PS	43	M	4	1	17.1	4.2		1.59	1.06	112.9	1.5	high	9.4	6.7			10
PS	64	F	7	1	22	3.7	168	1.71	0.85	116.1	1.5	high	10.1	4.8	0	31	9.6
PS	64	M	2	1	21.2	4	142	1.9	0.83	93.81	1.3	high	9.4	6.1	0.09	30	12.4
PS	68	F	15	1	22	3.5	157	1.48	1.19	128.3	1.8	high	8.5	4.2	0.69	30	11.4
PS	32	M	6	1	22.9	3.8		1.51	0.98	123.5	1.5	high	8.6	8.7	0.32	44	11.6
PS	36	F	19	1	16.8	3.9	118	1.75	1.07	114.6	2.1	high	8.8	5.1	0.11	24	11
PS	61	F	23	1	18.6	3.6	167	1.74	0.95	111.7	1	high	9.4	5.9	0.04	25	8.8
PS	61	M	19	1	24	3.7	100	1.4	1.02	111.5	2.2	high		3.9	0.22	32	11
PS	58	M	12	1	21.9	3.2	136	1.79	0.84	111.4	2.1	high	10	6.1	0.03	32	10.6
PS	73	M	10	1	18.9	3.4	116	1.54	1.06	123.5	2.1	high	9.3	2.3	0.1	34	9.1
PS	56	M	8	1	20.9	3.8	116	1.88	1.28	130.3	2.1	high	10	4.4	0	28	9.6
PS	48	M	30	1	24.2	3.4	150	1.7	1.03	133.3	1.8	high	8.9	3	0.03	25	8.5
PS	69	M	12	1	19.9	3.9	182	1.11	0.78	121.5	1.3	high	10.6	3.4	0.6	29	10.4
PS	45	M	7	1	17.4	4.3	141	1.57	0.73	85.28	1.5	high	9.4	5.9	0.06	26	9.4
PS	55	M	19	1	22	3.7	135	1.31	0.93	146.2	2.1	high	9.2	4.9	0.32	23	9.9
PS	72	M	32	1	20.6	3.6		1.45	0.82	82.67	1.8	high	10	3.5		23	10.4
PS	51	M	22	1	18.7	3.9	164	1.43	0.9	103	1.8	high	8.5	5.2		25	11.3
PS	50	M	36	1	19.6	3.7	165	1.41	0.97	92.39	1	high	10.5	4.1	0.4	23	12.9
PS	39	M	9	1	19.6	4.3	151	0.89	1.11	117.7	2.1	high	9.3	4.6	0.04	18	9.8
PS	57	M	3	1	27.1	3.8	151	1.79	0.77	75.75	1.5	high	9.9	5.1	0.25	26	9.5
PS	52	F	6	1	25.4	3.8	134	1.07	1.15	98.35	1.5	high	8.5	8.7	0.18	27	11.2
PS	46	F	19	1	16.9		120	1.11	0.86	106.8	2.5	high	10	7.8	0.06	19	13.5
PS	40	M	6	1	21.8		132	1.22	0.88	101.8	1.6	high	8.5	5.7	3.28	36	8.2
PS	52	M	12	1	21.9	3.6	132	1.45	0.85	111.6	1.3	high	9	7.4	5.4		9.8
PS	68	F	6	1	17.8	3.6	152	1.18	1.24	52.56	1.8	high	8.4	5	0.3	30	13.6
PS	77	F	2	1	14.5	3.9	185	1.72	0.92	62.11	1.8	high	8.7	6.2	0		10.3
PS	72	F	2	1	24.6	3.6	192	1.8	1.04	95.44	1.3	high		4.9	0.07		11
PS	59	M	10	1	19.4	3.8		1.49	0.95	74.93	1.5	high	9.7	5.4	0.13	33	10.7
PS	53	M	3	1	30.1	3.8	204	1.36	0.89	107.2	2.1	high	9.1	4.9	0.09	33	11.2
PS	57	M	4	1	20.7	4	190	1.76	0.74	92.54	1.6	high	10.5	4.7	0.01	20	10.8
PS	57	M	15	1	23.6	3.8	121	1.1	0.84	126.3	2.5	high	9.6	3.9	0.22	28	11.3
PS	61	M	29	1	18.4	3.4	141	1.35	0.95	90	2.2	high	8.2	5.8		18	12.2
PS	60	F	24	1	19.4	3.6	219	1.05	0.97	116.9	1.8	high	9.1	5.6	0.14	29	11
PS	67	M	20	1	20.5	3.2	163	1.54	1.16	128.8	2.2	high	9.3	6	0.2		10.7
PS	71	F	20	1	16.9	3.6	128	1.24	0.72	99.69	1.6	high	7.5	3.4			8.6
PS	59	F	16	1	19.6	3.1	131	1.02	1.07	124.4	2.1	high	9.2	6.9	0.12	27	10.4
PS	71	M	14	1	21	3.2	153	1.38	0.9	146.5	2.1	high	9.5	8	0.07	3	8.9
PS	81	M	14	1	15.6	3.4	172	1.23	1.03	128.1	1.4	high	11.7	4.7		26	9.7
PS	62	F	12	1	18.2	3.6	153	1.05	1.11	124.1	1.4	high	8.4	2.6	1.2	26	9.6
PS	65	M	5	1	20.5	3.7	153	1.38	0.95	135.7	1.8	high	8.8	5.5	0.29	20	10.2
PS	77	M	3	1	24.3	3.3	173	1.61	0.89	146.8	1.1	high	7.7	3.1	0.2	25	12.5
PS	64	M	2	1	20.5	3.6	166	1.69	0.87	128	1.5	high	10.7	4.8	0.03	28	9.8
PS	49	M	26	1	18.7	3.6	104	0.96	1.05	115.2	2.5	high	9.4	5.2	0.11	26	8.8
PS	51	F	21	1	15.5	3	112	1.19	0.89	83.27	2.1	high		6.1	0.01	23	12.3
PS	37	F	20	1	17.2	4.4	159	1.61	0.98	110.4	1.5	high	10.3	3.8		20	10.3
PS	47	M	13	1	19.2	4.6	188	1.6	0.98	125.3	1.5	high	9.5	10.2		33	9.5
PS	44	F	10	1	18.1	3.6	163	1.38	0.99	85.88	2.1	high	9.4	4.5			10.6
PS	55	F	10	1	21.2	3.7	95	1.44	1.22	104.8	1.1	high		4.5	0.34	27	9.6
PS	41	M	10	1	17.8	4.2		1.45	0.88	100	1.5	high	10.2	4.5	0.4	24	11.5
PS	83	F	8	1	28.8	3.6	181	1.09	0.79	92.69	0.8	high	9	2.1	1.1	24	9.4

PS	46	F	7	1	17.7	3.5	115	2.15	0.71	55.24	2	high	8.5	4		22	8.6
PS	58	F	7	1	22.2	4.1	173	1.62	0.85	120.9	1.8	high		3.8	0.05	27	9.9
PS	56	M	5	1	23	4	175	1.29	0.87	112.1	1.6	high	9.6	5.8	0.74	31	9.2
PS	52	M	5	1	21.6	4.2	161	1.7	0.88	103.3	1.5	high	9.5	3.6	0.3	18	9.7
PS	44	F	5	1	22.4	4.1	198	1.04	0.96	137.4	1.6	high	10.1	7.7	1.08	31	8.3
PS	70	M	5	1	22.9	4	76	1.1	0.79	143.2	1.8	high	10.3	6.9	0.15	28	11
PS	85	F	5	1	20.8	3.3	132	1.4	1	111.5	2.1	high	9.4	5.4		27	11.3
PS	70	F	5	1	22.2	3.7	197	1.37	0.55	116.8	1.6	high	9.3	6.8		31	11.5
PS	57	F	5	1	22.7	3.7	99	1.59	0.9	140.8	1.6	high	10.6	7		30	10.8
PS	65	M	5	1	22.3	3.8	151	1.35	0.89	123.7	1.6	high	9.7	5.2		35	12.7
PS	55	M	4	1	23.4	3.8	144	2.05	0.72	126.7	1.3	high	9	3.3	0.13	31	9.4
PS	65	M	4	1	17.5	3.6	127	1.53	0.62	107.6	1.5	high	9	4.9			6
PS	86	F	4	1	18.1	3.5	257	1.62	0.89	113	1.6	high	10.4	6.6		33	10.5
PS	74	M	3	1	23.2	3.8	115	1.11	0.84	97.25	2.1	high	8.1	5.8	0.91	30	10
PS	69	M	3	1	20.1	3.4	131	1.61	0.99	94.38	2.5	high	9.3	6.3	0.04	25	10.2
PS	78	F	3	1	21.9	4.1	140	1.65	0.96	106.2	1.6	high	9.8	4.5		25	11.3
PS	42	F	3	1	22.5	3.8	155	1.36	1.01	119.8	2.1	high	10	6.4	0.06	30	11.6
PS	69	M	3	1	25.4	4.7	242	1.63	0.69	130.5	1.1	high	9.6	4.6	0.06		7.9
PS	60	F	8	1	25.1	4.2	167	1.39	0.98	84.52	1.8	high	11	6.3	0	30	10.9
PS	53	F	13	1	18.9	4	111	1.19	0.86	86.46	1.1	high		4.5	0.06		11.5
PS	48	F	16	1	18.2	4.6	122	1.02	0.99	83.67	2.2	high		8.9	0.12	20	10.9
PS	61	M	16	1	24.3	3.6		1.39	0.86	85.6	2.1	high	7.6	5.8	0.3	28	7.1
PS	53	M	14	1	17.9	3.8	134	0.76	0.78	89.6	2.1	high	8.6	6.9	0.18	23	11.2
PS	75	M	12	1	22	3.5	122	1.28	0.99	108.7	1.6	high		3.7	0.04		7.6
PS	75	M	12	1	18.6	3.7	149	1.8	0.77	98.72	2.1	high	9.4	5.8		29	11
PS	54	F	12	1	19.3	4.8	178	1	0.78	107.6	2.1	high	9.4	2.6		20	9.4
PS	36	M	11	1	22.4	3.8	157	1.43	0.85	111.4	1	high		6.8	0.06		8.5
PS	70	F	11	1	20.1	3.8	166	1.25	0.82	133.7	1.8	high		4.7	1.97	27	9.2
PS	49	M	11	1	17.5	4.5	204	1.65	0.91	115.1	1	high		6.8	0.08		7.7
PS	78	M	11	1	17.4	3.7	134	1.51	0.89	104.9	2.1	high	7.1	6.2	0.2	32	10
PS	75	M	10	1	19.5	4.1		1.63	0.67	110.6	1.5	high	9.6	5.5	0.07	24	11.3
PS	60	F	10	1	21.9	4.4	90	1.94	0.96	74.88	1.1	high	9.9	4.2	0.1		9.1
PS	63	M	10	1	17.9	4.5	130	1.75	1.04	116.8	1.5	high	9.2	3.7	0.09		9.6
PS	55	F	10	1	25	4.4	193	1.74	1.08	122.7	1.5	high	9.5	6.7		28	11.2
PS	65	M	10	1	18.8	4	145	1.52	0.96	113.9	2.1	high	8.6	4.9	0.08	25	10.3
PS	49	F	10	1	23	3.4	164	1.97	0.76	124.9	2.1	high	8.4	3.5	0.1	24	11.1
PS	58	M	9	1	22.7	4.3	112	1.59	0.88	132.5	1.5	high	8.4	4.6	2.6	25	9.9
PS	70	M	9	1	25.7	4.3	142	1.79	0.91	102.3	1.5	high	10.3	7.4	0.08		8.7
PS	63	M	8	1	26.5	4.3	158	1.43	1.04	105	1.3	high		6.5	0.08		7.6
PS	76	M	8	1	20.1	4.7	170	1.43	0.94	132.5	1.3	high		4.9	0.27		8.2
PS	54	F	7	1	20.6	4.3	96	1.54	1.07	139.9	1.5	high	7.5	4.7	0.06	21	7.4
PS	61	F	7	1	30.3	3.7	103	1.51	0.93	104.9	1.8	high	9.2	5.6		34	10
PS	66	M	8	1	19.9	3.6	65	1.14	0.66	101.7	2.1	high	9	5.6	0.05	33	10.1
PS	34	M	7	1	20.7	4.1	171	1.48	0.79	83.02	1.3	high		6.9	0.57		7.4
PS	94	M	5	1	18.4	3.6	156	1.33	0.9	104.3	1.5	high	9	5.6		22	12.4
PS	63	M	4	1	23.7	4.5	163	1.5	1.15	74.47	1.3	high		4	0.67		8.7
PS	65	M	3	1	23.8	4	123	1.48	0.8	107.9	1.5	high	7.8	6.3	0.03	27	11
PS	58	M	3	1	23.1	3.9	110	1.2	0.93	133	1.6	high		2	0.08		9.2
PS	61	M	2	1	23.1	4.2	109	1.52	0.85	71.75	1.5	high	8.8	4.2	0.62	41	10.8
PS	72	M	2	1	22	3.8	119	1.03	0.93	101.1	1.8	high	9.2	4.4		48	10
PS	61	M	2	1	26.2	4.4	131	1.48	0.74	84.49	2.1	high	8.1	2.8	0.4	16	10.2
PS	67	F	2	1	25.5	3.7	154	1.32	0.78	112.6	2.1	high	9.5	9	0.05	29	11
PS	19	F	11	1	21	3.9	178	1	0.98	109.1	1	high	10.1	5.8		31	11.9
PS	69	M	12	1	21.5	4.1	186	1.41	0.9	102.1	1.5	high	9.6	4.2	0	23	10.4
PS	65	F	12	1	21.2	3.8		1.17	0.83	105	1.8	high	8.2	6.7	0.5	17	13.1
PS	64	F	13	1	20.9	3.9	172	1.64	1.11	137.2	1.5	high	7.9	5.5	0.1	22	9
PS	74	M	23	1	19.7	4.1	112	1.45	0.82	86.31	1.5	high		9.9	0.4	32	10.7
PS	62	F	14	1	21.2	3.7	110	1.12	0.79	96.83	1.6	high		3.1	0.02		11.4
PS	28	M	7	1	19.7	4.3	129	1.81	0.9	113.2	2.1	high	8.6	4.6	0	31	9

PS	57	M	10	1	22.4	3.6	141	1.7	0.75	115.6	1.8	high	8.3	3.2	0.28	39	9.2
PS	55	F	7	1	20.8	3.7	130	1.67	0.84	110.7	1.8	high	9.1	2.9	3.23		9.8
PS	60	F	12	1	22.9	3.5	163	1.21	0.87	51.68	1.6	high		4.8	0.03		9.1
PS	72	M	14	1	18.5	4.1	159	1.51	0.68	110.5	1.5	high	8.8	3.8	0.04	18	11.6
PS	68	F	3	1	19.5	4.3	147	1.32	1.03	64.14	2.1	high	9.7	8.4	0.04	29	10
PS	73	F	15	1	28	3.5	67	0.88	0.77	125.1	1.1	high	8.4	3.2	5.54	34	10.3
PS	67	M	4	1	24.6	3.6	140	1.3	0.81	138.5	2.1	high	9	6	0.2	24	9.9
PS	72	F	6	1	22.1	3.5	138	1.37	0.76	158.6	2.1	high	7.9	4.2	8.78	19	11.7
PS	60	F	6	1	16.1	3.6	127	1.32	0.59	77.68	2.1	high	10.9	5.8	0.4		10.7
PS	49	F	12	1	17.5	4.1		1.67	0.94	120.3	1.5	high		5.3	0.09	27	11.6
PS	72	F	7	1	20.8	4	108	1.09	0.69	115.3	1.6	high		8.2	0.06		9.5
PS	57	M	15	1	22.7	3.5	167	1.91	0.87	121.9	1.5	high	8.2	5.7	0.4	22	11.7
PS	50	M	16	1	22.5	3.8	147	1.28	1.01	109.7	1.6	high		8.2	0.03		7.8
PS	60	F	10	1	19.9	3.6	119	1.51	1	131.6	1.8	high	10.6	6.2	0.1	22	9.8
PS	63	F	13	1	15.8	3.9	211	1.19	0.64	120.4	1.3	high		5.3	0.17		11
PS	49	M	11	1	27	4.3	141	1.21	0.72	111.6	1.6	high		2		13	9.8
PS	62	F	8	1	24.9	3.7	114	1.54	0.99	129.1	1.5	high	8.4	3.8		30	10.2
PS	54	F	35	1	16.2		164	1.69	0.9	83.48	1.5	high	8.5	7.5	0	25	11.2
PS	62	M	18	1	17.3	4.1	151	1.56	0.91	97.7	1.8	high	9.1	5.3	0.07	25	11.9
PS	68	M	13	1	20.2	3.8	113	1.68	1.19	115.3	1.3	high	9.9	7.2		44	10.3
PS	58	M	4	1	24.6	4.5	166	1.43	0.95	130.1	2.1	high	11.4	8.8		30	12.9
PS	44	F	4	1	23.8	4	195	1.47	1.01	100.1	2.1	high	8.8	4.7	0.15	35	12.5
PS	56	M	4	1	26	4.1	114	1.16	1.08	99.35	1.4	high	8.9	2.8		27	9.5
PS	62	M	3	1	20.9	4.3	172	1.3	0.97	93.51	2.1	high	8.6	6.9	0.08	29	11.8
PS	65	F	3	1	18.4	3.8	139	1.41	1.23	84.51	1.8	high	8.9	4.3	3.7		9.3
PS	65	F	6	1	20.7	3.6		1.51	0.78	98.79	2.2	high	8.9	4.9			
PS	45	M	7	1	24.6	4.3	157	1.56	1.12	130.3	1.8	high		7.5	0.4	27	8.9
PS	59	M	29	1	23.2	3.2	137	1.99	0.75	90.25	1.5	high	9.2	5.9	0.26	31	9.9
PS	70	F	25	1	19.9	3.5	132	1.35	1.17	110.4	1.5	high	8	7		21	10.5
PS	50	M	16	1	18.4	4	147	1.91	0.75	84.52	1.1	high	8	3.5		19	10.3
PS	74	F	17	1	18.4	3.6	142	1.49	0.76	92.66	1.5	high	8.5	3.8	4.03	25	9.9
PS	58	M	16	1	33	3.5	162	1.65	0.81	103.6	1.1	high		4.9	0.05		10.4
PS	49	M	10	1	23.1	3.7	97	1.27	1.06	123.5	1.8	high		4.7	0.09	47	11.4
PS	72	M	21	1	22.6	3.6	179	1.53	0.9	102	1.3	high	8.7	4.3	0.17	24	10.9
PS	38	M	14	1	22.4	3.8	158	1.92	1.16	102.6	1.5	high	8.1	5.4		46	9.6
PS	60	M	5	1	22.4	3.5	165	1.54	0.74	71.25	1.1	high	8.5	4	0.6	18	7.8
PS	70	F	17	1	24.8	3.7	150	1.49	1.4	107.3	1.3	high	8.8	4.9		28	10.1
PS	69	M	16	1	19	3.5	135	1.4	1.14	95.15	2.1	high	8.2	7	0.12	25	10.7
PS	62	M	13	1	18.2	3.7	169	1.98	0.9	100.8	1.5	high	9.1	4.8	0.1	27	13.1
PS	45	M	26	1	25.9	3.4	140	1.36	1.28	114.2	1.9	high	8.9	4.6	0.08	20	9.5
PS	57	M	15	1	19.4	3.4	128	1.9	1.17	101.4	1.6	high	8.8	5.6	0	30	8.2
PS	69	M	7	1	23.7	3.5	106	1.28	0.94	133.2	1.6	high	8.6	5.6	0.4	20	9.8
PS	60	M	28	1	19.8	3.7	148	1.14	1.09	81.37	1.8	high	9.9	4.4	0.1		10.8
PS	49	M	21	1	21.3	3.7		1.59	0.88	107.2	1	high	9	5.4	0.05	29	10.4
PS	57	M	11	1	29.6	4.1	163	1.51	0.88	101.5	1.3	high	8.6	7.1		29	7.6
PS	35	F	6	1	22.6	3.9	110	1.94	1.02	98.23	1.1	high	8.7	2.7		19	8.8
PS	60	M	5	1	23.8	3.7	225	1.76	0.89	134.8	1.3	high	8.5	4.9		26	10.9
PS	56	M	20	1	23.9	3.7	150	1.2	0.97	86.92	1.5	high	8.3	11		14	13.3
PS	70	M	8	1	19.4	3.2		1.31	1.1	110.6	1.5	high	9.3	5.7	0.06	21	11
PS	70	M	15	1	24.5	3.8	171	1.54	0.84	99.35	1.5	high	10.9	5.5		22	8.7
PS	53	M	11	1	22.1	3.6	165		0.83	117.1	1.3	high	9.3	7.1	0.93	25	12
PS	62	M	24	1	20.9	3.8	130	1.46	1.01	76.76	2.2	high	9.3	4.6	0		10
PS	53	F	20	1	16	3.6	161	1.61	0.92	90.23	1.5	high	8.6	4		29	11.7
PS	62	M	12	1	23.8	3.5	148	1.8	1	98.91	1.1	high	9.1	1.9		22	11.5
PS	61	M	7	1	22.1	3.5	132	1.4	0.87	59.76	1.8	high	8.1	7		30	9.8
PS	45	M	19	1	22.9	3.8	190	1.83	1.06	105.4	1.8	high	8.8	6	0.16	24	9.1
PS	51	M	9	1	23.8	4.2	144	1.63	0.86	100.2	2.1	high	9.5	4.6	0.05	24	9.4
PS	60	F	21	1	25.5	3.4	191	1.41	1	93.9	1.8	high		5.8	0.05	44	11.2
PS	46	M	8	1	22.9	3.3	201	1.43	1.32	107.1	1.3	high	11.1	5.2	0.56	27	9.2

PS	60	M	4	1	24.6	3.7	155	1.43	0.89	104.6	1.5	high	8.1	3.7	0.9	23	9.4
PS	64	M	16	1	23.3	3.5	112	1.25	0.88	112.6	1.3	high	9.2	5.6		23	10.8
PS	72	F	30	1	21.7	3.9	152	1.21	0.72	60.55	1.8	high	9.1	5.6	0.1		9.2
PS	55	M	8	1	23	3.4	155	1.67	0.99	121.4	1.5	high	9.2	5.7	2.53	22	9.9
PS	80	F	6	1	22.1	3.2	130	1.73	0.94	112.7	1.3	high	9.1	4.6	0.1	35	9.2
PS	58	M	11	1	22	3.5		1.32	0.98	93.97	2.1	high	9	4.9		45	11.3
PS	50	M	7	1	25.7	3.2	201	1.48	1.05	111.7	1.3	high	10.7	4.9		21	10.7
PS	72	F	6	1	20.4	3.4	144	1.4	0.79	93.02	1.1	high	9.2	4.7	1.22		12.4
PS	67	F	11	1	19.3	3.7	117	1.44	1.16	104.4	1.5	high	6.7	5.1		16	10.7
PS	56	F	4	1	19.6	3.9	135	1.51	0.69	116.5	1.8	high	10.2	5	0.06	33	9.9
PS	54	M	33	1	16.6	4	91	1.61	0.94	93.14	1.5	high	10.2	4.4	0.05	22	5
PS	60	M	20	1	20.8	3.8	121	1.29	0.85	100.7	1.8	high	8.6	4.8	0.05	29	11.2
PS	47	M	17	1	19.2	4.3	112	1.61	0.95	104.8	1.8	high	9.6	4.7	0.05	30	9.8
PS	55	M	18	1	19.4	3.8	123	1.09	0.59	96.47	1.5	high	8	4.4	0.08	21	10.6
PS	67	F	18	1	20.7	3.7	112	1.15	0.87	106.2	1.3	high	9.4	7.6		31	13.3
PS	63	F	4	1	14.6	4	166	1.38	0.9	112.5	0.8	high	8.8	5		26	10.6
PS	54	M	4	1	21.3	3.6	103	1.59	0.66	121.6	1.5	high	9	6.1	0.1	18	10.8
PS	72	M	16	1	17.4	3.7	135	1.83	0.54	87.49	1.8	high	9.3	7.6	0.04	26	7.6
PS	74	F	19	1	16.5	3.8	125	1.52	0.95	137.9	1.6	high	9.3	5.4	0.21	30	10.5
PS	71	M	12	1	21.8	3.7	145	1.66	0.92	135.2	1.5	high	9.6	7.3	0.1	23	11.7
PS	70	F	5	1	20.5	3.7	132	1.45	0.63	64.67	1.8	high	10.1	4.5	0	16	10.8
PS	65	F	4	1	17	3.7	116	1.46	1.41	107.7	1.8	high	9.8	5.6	0.1	33	9.7
PS	55	M	12	1	18.4	3.5	164	0.97	0.94	134.7	1.8	high	9.8	5.5	0.78	31	10.6
PS	54	M	3	1	19.9	3.8	161	1.86	0.84	114.9	1.8	high	9.9	5.7	0.02	34	10.8
PS	50	M	3	1	21.3	4.3	128	1.45	0.79	75.27	1.8	high	9.1	6.6	0	39	11.3
PS	72	M	3	1	22.6	3.5		1.23	0.64	88.31	1.6	high	7.1	1.6	0.2	25	9.9
PS	38	M	3	1	27.3	4.3	193	1.4	0.86	122.6	1.6	high		5.9		24	10.6
PS	58	M	2	1	23.5	3.5	113	1.53	0.83	71.34	1.5	high	8	4.3	0.2	24	9.2
PS	55	F	26	1	19.3	3.6		1.19	1.07	113.3	1.3	high	8.9	5.2	11.71	28	10.8
PS	63	M	9	1	23.9		200	1.32	1.09	101.5	1.8	high	9.7	3.7	0.04	26	10.4
PS	81	M	6	1	18.2	4.2		1.51	0.68	106.8	2.1	high	7.9	6.2	0.14	25	10.2
PS	85	F	3	1	29.6	3.4		1.15	0.76	98.91	1.8	high	8.4	4.9	0.03	27	9.4
PS	60	M	4	1	23	4.6	152	1.35	0.79	123	2.1	high	9.3	7.2	0.09	28	9.8
PS	70	M	17	1	22	3.8	117	1.5	0.98	123.2	1.5	high	10.2	4.6	0	28	10.7
PS	71	F	3	1	25.5	3.4	136	1.32	0.84	87.33	1.5	high	10.2	5.9	0.09	28	9.6
PS	49	M	4	1	29.1	3.9		1.23	0.91	96.76	1.6	high	10.1	4.4		20	9
PS	46	M	5	1	24.4	3.9	164	1.38	0.89	117.5	1.5	high	8.9	6.7	0.23	30	9.5
PS	35	M	10	1	19.9	3.7	153	1.25	0.8	95.62	1.8	high	9.4	3.9	0.05	27	10.8
PS	64	M	5	1	17.9	3.8	70	1.19	0.81	59.17	1.8	high	8.3	1	19.91	34	7.8
PS	46	M	25	1	25.3	4.1	120	1.4	0.96	102.8	1.5	high	8	4.4	0.1	26	9.4
PS	68	F	13	1	20.4	4.3	161	1.26	0.88	74.15	1.5	high	8.5	5.7	2.5	23	10.7
PS	61	M	12	1	19.2	3.6		1.62	1.05	105.4	2.1	high	9.1	5.6	0.04	22	11.3
PS	67	M	10	1	21.8	3.6	208	1.42	0.67	81.95	1.8	high	8.7	4.9	0.24		10.7
PS	65	M	9	1	24.8	3.9	126	1.39	1.03	116.7	2.1	high	8.5	5.4	0.14		11.7
PS	83	F	6	1	22.5	3.5	159	1.53	0.92	129.4	1.5	high		5	0.1	30	11.7
PS	57	M	5	1	19.8	3.7	191	1.62	1.06	96.93	1.8	high	9.9	6.4	0.05	21	12
PS	47	M	5	1	18.3	4.2	178	1.35	0.9	62.26	2.1	high	9	6.9	0.05	30	9.9
PS	71	M	4	1	17.7	4.4		1.77	1.02	135	1.8	high	9.3	2.8	0	23	10.5
PS	61	M	4	1	24.8	3.7		1.75	1.08	119.3	1.8	high	8.7	7.1	0.1	8	9.6
PS	66	M	3	1	22.4	4.2		1.54	0.77	28.67	1	high	7.5	7.6	0.08	13	9.6
PS	66	M	3	1	21.1	3.7	194	1.41	0.8	68.53	2.1	high	9.8	5.8	0.05	24	11.5
PS	64	M	3	1	21.1	3.6		1.52	0.93	76.35	1	high	8.5	6	0.06	27	11.1
PS	68	M	2	1	21.6	3.1	138	1.46	0.79	57.23	2.1	high	9.3	4.8	0.05	24	9.6
PS	73	M	3	1	18.5	3.2	198	1.13	0.78	82.1	1.5	high		6.9	0.1	18	10
PS	66	M	19	1	22.8	3.5	130	1.37	0.97	141.9	1.9	high	10.2	6.1	0.15	36	9.1
PS	63	M	2	1	17.3	3.9	176	1.91	0.77	61.23	1.5	high	10.2	5.1	0.02	21	10.8
PS	69	F	8	1	18.8	3.9	99	1.78	0.91	86.51	1.5	high	9.3	3.4	1.7		10.1
PS	61	M	5	1	22.7	4	129	1.4	0.88	121.2	2.5	high	8.4	5.9	0.28	38	7.6
PS	71	F	7	1	20.2	4.6	216	2.37	0.8	77.56	1.8	high	8.9	7.9		23	11.4

PS	60	F	17	1	17.3	3.7	178	2.07	0.77	116.1	1.8	high	10.1	5.3	0.01	26	9.8
PS	69	M	14	1	22.1	3.8	139	1.18	0.85	108.4	1.9	high	8.4	3	0.05	24	11.1
PS	68	M	2	1	23.2	4.4	148	1.88	0.97	97.54	1.5	high	10	4	0.71		9.1
PS	78	M	9	1	20.8	4.3	129	0.95	0.73	91.18	1.1	high	8.5	4.5	0.48	35	9.2
PS	88	F	7	1	19.3	3.2	183	1.65	0.74	111	1.6	high	10	7.1	0.44	31	10.7
PS	61	F	3	1	17.1	4.6	110	1.3	0.74	104.3	1.3	high	9.7	5.1	0.06	47	9.6
PS	56	M	4	1	22.4	4.5	147	2.21	0.7	81.27	2.1	high	9.1	5.4	0	30	9.7
PS	60	M	4	1	19.8	3.6	187	1.54	0.85	114.7	1.5	high	11.1	4.7	1.64	26	11
PS	57	F	3	1	17.1	3.9	185	1.41	0.82	48.51	1.3	high	10.1	5	0.01	33	10.5
PS	75	M	3	1	19.2	3.8	168	1.54	1.07	109.9	1.5	high	8.5	5.6		16	9.6
PS	58	M	27	1	21	3.4	136	1.37	0.69	81.14	1.9	high	9.8	5.4	0.08	27	10.2
PS	61	M	8	1	20.8	4.4	138	1.62	0.86	129.8	1.8	high	9.3	3.8	0.21	22	9.4
PS	48	F	10	1	18.3	4.4	132	1.7	0.85	104	1.9	high	8.3	4.1	0.1	49	10.4
PS	33	M	4	1	22.1	4.2	146	2.32	1.08	83.11	2.1	high	8.3	6.2	0.1	29	11.6
PS	62	M	6	1	21.8	3.8	138	1.77	0.77	73.61	1.5	high	9.7	6.6	0.94	30	10.9
PS	62	M	8	1	23.2	3.9		1.74	0.66	88.97	1.6	high	9.4	4.6		31	10.4
PS	65	M	8	1	23.2	3.8		1.35	1.05	131.9	1.9	high	8.6	5.3		37	13.2
PS	89	F	4	1	20.2	4		1.29	0.78	123.4	1.6	high	8.1	6		18	9.8
PS	59	M	8	1	20.7	4.1	198	1.12	0.93	123.7	2.1	high		7.3	0.06	32	9.6
PS	71	M	10	1	27.3	3.8	109	1.28	0.68	91.64	2.1	high	8.3	6.9	2.83		9
PS	54	M	10	1	18.7	4.5	87	1.22	0.98	96.27	2.1	high	9	6.5	0.85	28	12.6
PS	62	M	2	1	19.4	4.2	111	1.64	0.88	77.53	1.8	high		6.1		37	11.2
PS	76	M	24	1	19.3	3.6	181	1.15	0.78	107	2.1	high		6.5	0.19	29	11.8
PS	32	F	7	1	18.3	4.4	191	1.27	0.85	94.89	2.1	high	8.9	4.8	0.06	21	10.9
PS	42	F	6	1	16.6	4.3		1.41	0.71	94.24	2.1	high	8.9	9.3	0.15	28	12.1
PS	70	F	11	1	21.2	3.9	70	0.92	0.93	118.3	2.1	high	8.4	7.5	0.05	23	10.9
PS	43	F	6	1	19.6	5.2	97	1.29	0.96	106.7	1.5	high		5	0.05	25	11.4
PS	45	F	29	1	18.1	4.5	144	2.05	0.93	107	1.5	high		4.6	0.08	27	10.9
PS	43	F	6	1	17.6	4		1.34	1.14	159.9	1.3	high	8.2	4.7	4.6	29	11.1
PS	57	M	4	1	23	4.1	146	1.42	0.62	90.9	1.5	high	9.1	4	0.1		9.9
PS	48	M	4	1	19.5	4.3	121	1.82	1.14	92.99	2.1	high	8.1	4.8	0.2	27	10.8
PS	50	F	21	1	20.5	4.1	122	1.8	0.95	117.1	2.1	high	9.2	5.7	0.5	24	10.1
PS	67	M	2	1	18	4.1	142	1.18	0.71	96.19	1.3	high	9.4	4	0.9		10.8
PS	54	M	3	1	19.1	4.4	153	1.13	0.9	70.83	1.8	high	9.1	5.9	0.7	25	9.7
PS	72	M	7	1	22.7	3.9	135	1.3	0.64	103.6	1.8	high	9.1	4.7	5.26	23	9.4
PS	51	M	18	1	20.1	4.2		1.5	0.75	103.4	1.6	high	9.6	3.8		32	11.9
PS	61	F	19	1	18.2	4	122	1.57	0.95	115	2.1	high	10.1	5.5	0.26	18	9.3
PS	69	F	18	1	16.5	4.2		1.25	0.94	103.8	1.9	high	10.3	6.1	0	26	11
PS	68	F	17	1	16	3.8	257	1.23	0.85	111.9	1.5	high			0.56		12.4
PS	61	F	14	1	22.1	3.9	138	1.39	0.8	115.5	2.1	high		5.1	0.71		9.4
PS	72	M	16	1	20.3	3.7	194	1.42	1.15	116.9	1.8	high	8.8	5.1	0.3	38	10.4
PS	54	F	16	1	17.2	4.4	179	1.36	1.1	62.68	1.5	high		7	1.92	35	11.7
PS	66	F	14	1	20.7	3.9	207	1.52	0.75	113	1.8	high	9.6	5.1	0.04	18	8.3
PS	65	M	11	1	18.6	3.8	93	1.48	1.15	79.65	1.8	high	8.1	6.5	0.15	45	10.6
PS	64	M	8	1	22.4	4	156	1.48	1.3	120.2	1.8	high	9.5	4.7	0.17	33	11.7
PS	64	F	7	1	25	4	193	1.67	1.02	107.2	1	high	8	6.3	0.09	29	12.6
PS	66	M	6	1	19.5	3.6	130	1.51	1	112.8	1.5	high		4.5	0.14	30	10.2
PS	58	M	6	1	21.4	4		1.34	0.99	95.79	1.8	high	8.7	5.5	0.5	12	13.2
PS	68	M	3	1	19.3	3.6		1.26	0.99	77.98	2.1	high	10.4	7.2		33	11.9
PS	50	F	3	1	27.2	3.8	101	1.36	0.9	83.22	2.1	high	8.3	5.5	0.04	31	9.5
PS	40	M	3	1	18.1	3.9	338	1.33	1.14	94.25	1.6	high	10	4.7			12.8
PS	65	M	2	1	19.7	4	217	1.37	1.31	105.1	1.8	high	9.8	6.6	0.08	26	12.7
PS	57	F	2	1	19.2	3.8	125	1.6	1.01	117.7	1.8	high	10.9	7.7	0.04	39	12.5
PS	41	F	2	1	18.1	4.3	207	1.39	1.21	122.2	1	high	8.6	5.4	0.24	21	9.6
PS	60	F	11	1	18.6	3.8	131	1.29	1.36	85.33	2.1	high		7.5	0.54	31	7.8
PS	62	M	2	1	20.8	4.2	173	1.18	0.77	103.2	1.4	high	9.5	5	0.42	11	13.1
PS	60	F	13	1	14.7	3.9	135	1.97	1.28	106.9	1.3	high	9.2	7.5	1.38	21	8.7
PS	71	F	21	1	23.9	3.7	119	1.19	0.91	126.8	2.2	high	7.4	4.8	0.07	24	10
PS	63	M	30	1	21.1	3.6	135	1.08	0.96	112.2	1.9	high	9	4.2	0.11	17	11

PS	57	F	23	1	21.3	3.8	123	0.72	0.91	134.6	1.4	high	8	4.1	0.66	26	7.8
PS	63	M	18	1	20.6	3.7	31	1.67	0.89	134.8	1.4	high	10.6	5.9	3.1		10.3
PS	55	M	21	1	21.2	4.1	151	1.57	0.87	123.9	1.8	high	9.1	6.6	0.05	26	12.3
PS	61	F	15	1	23.2		131	1.23	0.8	138.6	1.8	high	9.4	5.4			9.4
PS	60	M	5	1	22.8		126	1.68	0.85	99.19	1.8	high		5.2	0.1	30	11.5
PS	57	M	2	1	17.1		169	1.71	1	89.82	1.6	high	10.5	5.6	0.03	29	9.7
PS	56	F	2	1	23.8		139	1.55	0.74	98.44	1.8	high	8.1	6.2	0.2		10.5
PS	82	M	4	1	21.3	4	176	1.55	0.82	104.1	1.9	high	10.7	3.3	0.14	29	11.7
PS	64	F	5	1	21.2	3.6	187	1.93	0.94	120.3	1.6	high	7.6	5.4	0.04	27	11.9
PS	50	F	3	1	21.5	3.5	248	1.22	1.05	120.3	1.6	high	8.7	3.7	0.05	19	8.2
PS	60	M	5	1	25.9	4	105	1.4	0.8	108.8	1.8	high	10.1	6.1	0.06	26	9.3
PS	38	F	2	1	23.4	3.7	163	1.62	1.21	84.2	1.8	high		4.1	0.02		11.6
PS	85	F	23	1	17.7	4	177	1.88	0.67	93.69	1.3	high	7.9	4.2	0.45	20	11
PS	67	F	22	1	18.5	3.5	144	1.49	0.8	81.41	1.5	high	10.5	4	0.01	28	10.6
PS	69	F	33	1	26.9	4	128	1.95	0.92	97.65	1.5	high	10.3	3.9	0.74		11.2
PS	58	M	6	1	19.2	3.4	86	1.36	0.71	84.71	1.3	high		5.2	0.06	26	10.2
PS	64	F	10	1	16.1	3.5	150	1.7	1.14	126.1	1.3	high	8.8	4.9	0.02	25	9.7
PS	58	M	5	1	29.3	3.8	113	1.22	0.99	131.9	2.1	high	8.6	5.8		36	10.7
PS	61	F	2	1	17.8	3.8	89	1.33	0.63	88.48	1.6	high	9.5	4.1	0.07	26	10.3
PS	66	M	11	1	24.5	3.9	60	1.45	1.13	167.3	1.3	high	8.4	1.6	5.5		6.8
PS	46	F	3	1	18.4	3.6	165	1.72	1.08	132.6	1.5	high	9.2	4.8		21	10.2
PS	59	M	2	1	24	3.9	124	1.51	0.69	81.52	2.1	high		6.2	0.43	32	10
PS	72	M	10	1	23.3	4.1	137	1.49	0.74	97.44	1.6	high	9.6	5.7	0.02	48	11.7
PS	49	M	4	1	28.6	4.2	210	1.55	0.93	90.29	1.5	high	7.8	6.5	0.38	29	11.3
PS	64	F	4	1	32.8	3.8	128	1.19	0.96	90.61	1.8	high	8.5	4.5	0.19	31	10.7
PS	72	F	7	1	23.5	4	181	1.66	1.05	138.3	1.5	high	10.5	3.4	0.1	29	10.8
PS	67	M	6	1		3.9	130	1.09	0.91	71.66	2.1	high	8.9	3.9	0.1	27	11.1
PS	69	M	2	1	23.1	3.6	126	0.98	0.68	106.6	2.1	high	8.6	6.1		48	8.7
PS	74	M	24	1	18.7	3.3	121	1.46	0.73	96.2	2.1	high	8.4	5.1	0.09	24	10.3
PS	46	M	10	1	25.2	3.9	188	1.76	0.97	110.1	1.6	high	8.8	9.4	0.04	27	11
PS	62	M	26	1	20.7	4	172	1.58	0.86	111	1.3	high	9.3	3.3	0.56	11	10.9
PS	73	F	7	1	23.2	3.6		0.9	1.19	118.4	1.8	high	8.8	7.8	0	28	11.4
PS	71	F	15	1	22.2	3.2	140	1.39	0.82	83.86	1.6	high	9.7	6.7	0.12	30	9.2
PS	64	F	7	1	25.9	3.7	146	1.08	1.13	138.6	2.1	high		7	0.22	36	11.6
PS	46	M	2	1	27.6	3.9	92	1.57	1.02	100.6	1.8	high	8.6	5.7	0.4	32	8
PS	53	M	7	1	26.1	3.8	166	1.35	1.26	134.8	2.1	high	8.7	4.4	0.43	25	11.8
PS	78	F	3	1	21.7	3.9	150	1.3	0.73	72.81	1.3	high	10	6.8		31	12.9
PS	62	F	18	1	20.9	4	151	1.2	1	120.4	1.3	high	9.1	3.7		26	11.2
PS	74	F	7	1	19	3.5	175	1.45	0.86	87.55	2.1	high	9.4	3.3	0.3	27	10.2
PS	70	F	3	1	18.8	3.8	154	0.89	0.9	114.3	1.5	high	9	3.4	1.04	10	6.4
PS	73	M	24	1	17.6	3.5	163	1.85	0.88	97.08	1.9	high	9.3	5.1	0.01	25	9.6
PS	56	M	6	1	26.7	3.6	217	1.73	0.69	93.35	2.1	high	8.2	5.4	0.26		10
PS	66	F	14	1	20.1	3.8	123	1.44	0.98	131.3	1.5	high		4.3	0.03	29	10.5
PS	67	F	15	1	16.6	3.1	140	1.65	0.63	66.2	1.1	high	8.2	6.1		29	10.4
PS	67	M	6	1	22.4	4	144	1.68	1.07	84.5	1.5	high	9.1	4.3	0.74		9.5
PS	72	F	31	1	24.4	3.2	119	1.56	0.79	77.97	1.5	high	8.4	5.4	0.05		9.8
PS	68	M	16	1	19.4	3.9	112	1.27	0.97	130.8	1.6	high	8.6	7.3	0.25	30	9.9
PS	73	F	29	1	16.5	3.6	102	2.17	0.89	85.92	1.3	high	10	6.5	0.02	31	10.4
PS	65	F	4	1	20.7	3.3	120	1.85	1.23	107.7	1.9	high	8.9	3.8	0.07	26	9
PS	69	M	15	1	19.4	3.7	137	1.35	0.86	135.8	1.6	high	7.1	4	0.07	25	12.6
PS	70	F	16	1	20.6	3.5	149	1.29	0.84	101.2	1.8	high	10	8.3	0.71	37	11
PS	84	M	3	1	21.7	3.7	227	1.75	0.96	121.5	1.6	high	9	3.7	0.03	25	9.7
PS	66	F	19	1	17.8	3.5	145	1.31	0.91	117.9	1.3	high	9.2	3.9		23	10.6
PS	60	M	5	1	21.2	4.1	165	1.57	1.12	120.2	1.6	high	9.6	4.7	0.35	28	9.8
PS	62	F	31	1	21.8	3.6	131	1.25	0.96	94.74	2.1	high		7.9	0.01	30	10.4
PS	72	F	15	1	19.1	3.3		1.39	0.7	77.4	1.6	high	7.9	7	0.2	19	11.4
PS	60	M	2	1	22.3	4.1	135	1.58	0.96	87.79	1.3	high	10	4.3	0.04	22	10
PS	64	M	6	1	20.2	3.8	149	1.58	0.75	122	1.1	high	8.9	3.9	0.97	30	7.3
PS	71	F	4	1	21.1	3.7	170	1.82	0.71	112.5	1.5	high	9.9	6	0.2	32	10.1

PS	69	M	14	1	19.1	4	100	1.48	0.93	106.5	1.5	high		4.9	0.06	38	11.3
PS	69	F	5	1	16.4	4	114	1.43	0.91	94.31	1.5	high		9.7	0.02	32	11.2
PS	21	M	6	1	17.3	4.2	195	1.51	0.72	78.72	1.5	high	9.8	5	0.71	25	9.3
PS	52	F	14	1	16	3.8	142	1.98	0.91	99.41	1.5	high	8.8	4.6	0.07	28	8.9
PS	67	M	6	1	18.8	3.8		1.16	0.76	91.06	1.5	high	9.3	5.5	0.1	10	11.2
PS	62	M	7	1	16.3	3.3			0.98	122.7	1.5	high	8.3	3.2	0.19		14.6
PS	46	M	5	1	21.6	3.9	162	1.54	0.97	62.99	1.6	high	9.6	5.1			
PS	73	M	8	1	20.5	3.4	98	1.49	0.74	83.65	2.1	high	8.4	6.9	0.02	27	12.1
PS	40	F	8	1	18.1	4.1	161	1.22	0.97	109.4	2.1	high	9.7	10.9	0.55	27	10.7
PS	50	M	11	1	25.2	4.5	193	1.4	1.09	124.6	1.5	high	10.2	3.8	0.39	30	9.2
PS	54	F	8	1	19	4	87	0.93	1.07	106.2	2.1	high		8.2	10.29	32	9.4
PS	60	F	7	1	17.5	4.2	101	1.05	0.69	24.75	2.1	high	9.3	7.1	1.48	31	10.6
PS	65	F	17	1	21.5	4.1	201	1.94	0.92	118.2	1.8	high	8.3	4.5	0.09	28	10.6
PS	69	F	13	1	16.4	3.6	115	1.04	1.02	122.6	1.5	high		4	0.06	28	11.6
PS	68	M	19	1	21.6	3.5	96	1.65	0.78	98.41	1.5	high	8.6	5.5			8.3
PS	63	F	24	1	21.9	4	135	1.39	0.72	84.76	1.3	high	8.7	5.8	0.3	24	10.2
PS	75	F	4	1	18.4	3.4	102	1.44	0.89	105.3	2	high	9.3	5.2	0.02		8.7
PS	55	M	31	1	19.6	3.8	124	1.33	0.85	86.44	1.9	high	8.7	5.3	0	18	11
PS	60	F	21	1	19.5	3.9		1.66	1.03	117	2.2	high		5.2	0.2	28	9.9
PS	66	M	21	1	21.2	3.4	153	1.75	1.02	124	1.6	high	8.8	4.4	0.01	28	10.7
PS	49	M	20	1	20.3	4	121	1.11	0.71	113.2	1.4	high	7.4	4.6	0.1	17	9.1
PS	79	M	20	1	22.1	3.6	160	1.77	0.68	111.8	1.6	high	10.1	5.5	0.04	34	10.5
PS	52	F	20	1	20.8	3.6		1.76	0.84	128.2	1	high	8.1	3.7	0.03	35	8.2
PS	52	M	19	1	20.5	4.4	177	1.49	0.99	132.1	1.8	high	9.1	8.7	1	28	10.3
PS	45	F	19	1	16	3.7	161	1.56	1.07	84.14	2.1	high	7.8	2.5	0.55	18	10.7
PS	61	M	18	1	17	3.9	169	1.53	1.06	132.9	1.5	high	7.9	4.9	0.11	24	11.8
PS	74	F	17	1	21.2	3.6	166	0.93	0.91	91.02	2.1	high		6.2	1.04	25	11.5
PS	51	F	28	1	17.3	3.5	226	1.56	1.06	92.69	1.8	high	8.1	7.4	0.41	27	10.7
PS	70	M	16	1	22.3	3.5	194	1.98	1.24	129.6	1.6	high	9.8	4.9	0.02	25	10
PS	73	M	16	1	19.6	3.6	111	1.97	0.98	126.2	1.5	high	8.3	4.6	0.8	27	9.7
PS	71	M	16	1	17.9	3.1	174	2.02	0.72	82.9	1.5	high	7.7	4.8	2.7	29	8
PS	44	F	16	1	21.9	3.5	110	0.98	0.72	103	1.9	high	10.1	5.4	0.28	28	11.7
PS	41	F	16	1	18.8	4	132	1.31	1.02	145.3	2.1	high		7.8	0.03	32	10.2
PS	80	M	15	1	18.3	3.7	236	1.82	0.77	114.9	1.6	high	9.5	5.1	0.02	26	10.7
PS	61	F	15	1	22.7	3.8	124	1.24	1.05	135.7	1.8	high	8.6	3	0.05	40	8.8
PS	49	M	15	1	19.1	3.9	142	1.78	0.84	107.6	1.3	high	8.6	4.4	0.07	21	10.6
PS	61	M	13	1	22	3.4	169	1.7	0.84	94.29	1.6	high	9.3	4.2	0.01	32	10.1
PS	68	M	12	1	22.2	3.5	159	1.65	0.9	113.6	1.5	high	9.6	5.9	0.1	26	11.4
PS	73	M	11	1	18.2	4	153	1.78	0.84	99.72	1.5	high	8.6	4	0.7	28	9.5
PS	76	M	11	1	25.6	3	159	1.33	0.72	59.84	2.1	high	8.8	6.8			
PS	62	M	11	1	25.7	3.9	158	1.26	0.85	109	2.1	high	8.9	4.1		23	10.5
PS	41	M	10	1	32.4	3.9	124	1.75	0.91	82.11	1.5	high	9.6	4.8	0.2	27	11
PS	79	F	10	1	22.2	3.4	144	1.66	0.96	107	1.3	high	8.7	4.3	0.03	31	9.5
PS	61	F	27	1	22.8	2.8	122	1.25	0.8	93.86	1.8	high	8.9	3.7	0.73	27	7
PS	61	M	10	1	16.2	3.8		1.41	0.77	131	1.8	high	8.7	4.4	0	31	12.6
PS	64	F	10	1	27.3	3.2	142	1.34	0.7	116.4	2.1	high		4.1	0.83	32	10.7
PS	56	F	10	1	21.6	3.8	173	1.73	0.88	113.4	1.6	high	9.3	7			
PS	38	F	10	1	18.4	3.6	153	1.93	1.11	110.8	2.1	high	8.6	4.8	0.1	22	12.2
PS	76	M	9	1	19.1	3.4	145	1.01	0.75	114.2	1.5	high		4.1	0.02	28	8.8
PS	56	M	9	1	22.6	4	136	1.31	0.94	133.7	2.5	high	9.5	5.4	0.3	30	12.5
PS	60	M	9	1	20.2	3.9	139	1.46	0.85	94.36	2.1	high	10.3	6.7	0.02	28	10.6
PS	70	F	26	1	21	3.5	128	1.37	0.72	136.4	1.8	high	8.4	6.4	0.2	32	9.6
PS	73	F	9	1	18.8	3.1	187	1.64	0.65	94.83	1.3	high	10.2	6.3	0.07	26	9.3
PS	39	F	8	1	20.9	3.5	141	1.73	1.13	135.6	1.5	high	7.5	4.4	0.26	29	9.9
PS	35	M	7	1	37	3.9	150	1.48	1.09	124	1.5	high	8.9	5.5	0.02	24	10
PS	76	M	7	1	21.9	3.4	103	1.51	0.81	114	1.5	high		2.4			8
PS	32	F	6	1	18.9	4.5		1.22	0.99	109.5	1.5	high	9.4	2.7	0	29	11.6
PS	51	F	6	1	26.1	3.9	215	1.52	0.84	103.6	1.6	high	8.4	3.9	1.11	22	9.7
PS	61	M	6	1	25.1	3.9	150	1.35	0.85	116	1.5	high	9.1	5.2	0.09	34	10.2

PS	59	M	5	1	23.5	3.3	100	1.29	1	91.29	1.8	high	8.4	4.1	0.1	29	8.9
PS	40	F	26	1	15.5	4	94	1.3	0.87	78.42	1.4	high	9.2	4.5	0.32	29	9.5
PS	64	M	5	1	20.9	4	107	0.99	1.08	145.9	2.1	high		6.9	0.1	25	10
PS	60	M	5	1	21.1	4	238	1.39	1.18	138.8	1.5	high	9.6	6.4		19	9.9
PS	64	F	5	1	20.4	3.8	122	1.49	0.84	144.9	1.6	high	9.3	3.7	0.06	27	9.1
PS	61	M	5	1	23.1	3.8	146	1.31	0.75	80.79	2.1	high	8.1	4.8	0.13	20	10.7
PS	51	F	4	1	17.1	3.6	146	2.07	0.96	87.62	1.5	high	9.8	5.7	0.03	31	11.4
PS	61	M	4	1	21.8	3.6	133	1.54	1.01	115.1	1.5	high		4.9	1.34	21	10
PS	60	M	4	1	23.1	3.7	184	1.61	0.92	132.5	1.8	high	9.7	5.5	0.1	21	10.5
PS	43	M	4	1	21.4	4	159	1.62	1.02	126.6	1.8	high	8.7	6.6	0.07	27	11
PS	76	M	4	1	20.9	4.4	149	1.87	0.94	126.9	1.6	high	10.2	5	0.05	25	10.9
PS	83	M	4	1	23.2	3.7	106	1.12	0.99	156.9	1.5	high	8.8	6.3	0.11	28	14.2
PS	68	M	3	1	22.4	4	100	1.27	0.94	96.13	1.3	high	8.4	4.2	0	24	9.9
PS	57	M	3	1	18.8	3.9	116	1.2	0.88	119.5	2.1	high	8.4	6.6	0.17	24	10.9
PS	67	M	3	1	19.4	3.4	150	1.31	0.88	134.8	2.1	high	9.9	8.6	0.06	25	12.6
PS	56	M	3	1	24.8	4	116	1.23	0.75	97.39	1.5	high	9	3.9	0.12	26	10.2
PS	59	F	3	1	24.7	3.7	126	1.74	1.07	81.7	1.6	high	8.3	4.9	3.84	28	12.9
PS	69	M	23	1	21.5	3.5	114	1.47	1.14	108.9	1.9	high	9.3	5	0.2	20	9.8
PS	66	M	2	1	23.8	3.3	160	1.34	0.99	119.8	2.1	high	9.5	6.2	0.3	28	13.7
PS	69	F	2	1	24.4	3.6	168	1.25	0.85	107.5	1.6	high	9	4.1	2.24	51	9.7
PS	60	M	2	1	26.3	3.5	180	1.28	0.88	73.96	2.1	high	9.1	4.4	0.24	29	9.7
PS	72	M	2	1	26.4	3.7	127	1.58	0.73	114.4	2.1	high	8.4	3.8	1.33	29	9.9
PS	54	M	22	1	20	3.8	119	1.37	0.87	105.2	1.9	high	9.9	6	0	31	10.7
PS	38	M	2	1	21.3	4.7	203	2.01	0.81	132.5	1.3	high	8.6	4.6	0.15	30	11
PS	57	F	2	1	24.1	4.2	179	1.85	0.63	78.9	1.8	high	10.8	5.7		25	11
PS	63	F	2	1	21.6	3.8		1.26	1.36	134.7	2.1	high	10.5	4.8	0	24	10.9
PS	56	F	2	1	26.1	3.5		1.51	0.77	123.3	2.1	high	8.1	6	4.91	26	8.7
PS	56	M	2	1	20.2	3.7	96	1.59	0.78	134.2	2.5	high	9.7	8	0	29	11.1
PS	54	F	2	1	18.8	3.6	145	1.54	0.91	91.57	1.3	high	8.8	2.1	0	31	9.6
PS	59	F	24	1	23.3	3.9	138	1.27	0.77	109.1	1.4	high	8.1	6	1.7	26	10.5
PS	61	F	19	1	21.7	3.8	165	1.5	0.99	118.2	1.3	high	9.3	5.3	0.04	21	10.6
PS	60	M	19	1	15.9	3.8	159	1.63	0.85	90.71	1.3	high	8.5	4.5	0.03	27	9.5
PS	63	M	18	1	22.6	3.7	186	2.01	0.95	86.37	1.5	high	9.2	4.9	0.12	21	10.4
PS	59	M	11	1	21.8	3.8	187	1.34	1.17	104	2.2	high	8.4	7.3	0.15	22	10
PS	58	F	7	1	24.8	3.9	179	1.82	0.96	112.7	1.5	high	11.1	4.8	0.05	21	10.3
PS	60	M	6	1	23.3	3.9	126	1.43	0.78	95.28	1.8	high		6.7		26	9.4
PS	73	M	6	1	15.9	3.7	151	1.08	0.44	99.59	1.8	high	9.2	5.6	0.02	22	11
PS	33	M	5	1	18.5	3.6	158	2.27	0.7	91.94	1.5	high	8.5	4	0.05	15	11.5
PS	74	M	4	1	18.5	4.1	96	1.63	0.76	96.43	1.8	high	8	6.1	0.26	29	10.2
PS	41	F	2	1	26.1	3.7	191	1.59	0.89	104.3	1	high	8.7	4.9	0.31	28	11.3
PS	24	M	2	1	18.7	3.7	172	1.47	0.82	89.24	1.5	high	9.6	3.9	0.17	27	9.7
PS	81	M	4	1	19	3	95	1.68	1.12	100.7	1.3	high		4.4	5.57	32	11.8
PS	72	F	20	1	21.1	3.4	175	1.44	0.74	109.5	1.8	high		7.8	0.05		12.5
PS	68	M	2	1	20.8	3.7	153	1.2	0.9	152.3	1.8	high	9.1	8.7	0.2	30	11.5
PS	74	M	3	1	27	3.8	122	0.88	0.75	88.66	2.1	high	8.7	6	0.1	33	13.1
PS	71	M	6	1	19.6	3.9	185	1.41	0.97	96.22	2.1	high	9.1	4.8	0.05	31	11.3
PS	60	M	21	1	17.3	3.3	121	1.51	0.94	84.42	1.8	high	9.2	3.5	0.06	25	8.8
PS	76	M	7	1	22.8	3.6	161	1.74	0.91	139.9	1.3	high	8.7	7.8	0.06	32	9
PS	69	M	6	1	18.1	4.1	165	1.25	1.2	95.45	1.5	high	8.9	3.3	1.18	21	10.4
PS	69	F	4	1	21	3.6	171	1.32	0.95	111.4	2.1	high	7.3	3.1	0.1	32	12.4
PS	58	M	3	1	22.2	4	105	1.77	0.53	54.25	1.8	high	8.3	6	0	37	10
PS	67	M	20	1	19.9	3.9	175	1.45	0.78	108.4	1.3	high	8.9	4.9	0.06	26	10
PS	66	M	13	1	22.1	3.9	128	1.43	0.72	68.18	2.1	high	8.7	4.9	0.2	26	9.1
PS	68	M	11	1	18.8	3.7	141	1.39	1.02	118.8	2	high	8.4	7.3	0.1	27	9.4
PS	77	M	6	1	24.8	3.9	145	1.51	0.94	119.2	2.1	high	10.4	6.2	0.34	24	9.5
PS	75	M	5	1	22.3	3.8	109	1.05	1.22	98.38	2.1	high	8.4	5.6	0.05	24	10.6
PS	49	M	5	1	17.5	4.6	123	1.43	1.03	64.34	1.8	high	8.1	5.3	0	19	9.8
PS	80	F	4	1	17.6	3.9		2.05	0.81	115	1.1	high	9	3.5	0.4	29	8.8
PS	67	M	3	1	24.6	4.4	118	1.71	0.78	107.9	1.3	high	8	3.4	0.04	28	10.5

PS	85	F	2	1	20.6	3.6	176	1.54	1.21	89.87	2.1	high	10.6	6.2	1.09	26	10.4
PS	66	M	2	1	22.6	4	159	1.49	0.87	75.12	1.3	high	8.3	5.6	0.07	28	10
PS	44	M	2	1	35.3	3.4	96	1.17	0.71	88.82	1.8	high	10.7	4.9			11.5
PS	83	F	2	1	18.4	3.6	116	1.18	0.83	70	1.8	high	9.3	6	0.03		8.8
PS	79	F	2	1	15	3.7	110	1.25	1.21	75.67	2.1	high	8.7	4.2	0.05		11
PS	72	F	2	1	23.8	3.7	157	1.46	0.81	51.3	1.8	high	8.8	4.7	0.27		8.8
PS	82	M	8	1	20.1	3.3	122	1.17	0.89	130.2	2.2	high	9	4.2	0.22	33	13.1
PS	80	M	2	1	20.2	3.3	116	1.47	0.97	97.46	1.5	high	8.9	6.2	0.14	33	9.6
PS	73	F	11	1	18.2	3.3	155	1.56	1	114.1	1.6	high	9.5	6.4	0.07	32	9.7
PS	71	F	17	1	18.5	3.4	150	1.48	0.84	93.01	1.1	high	10.3	4.8	0.07	26	11
PS	64	F	20	1	17	4.1	164	1.35	0.9	113.3	2.2	high	8.6	7.5	0.05		10.8
PS	54	F	5	1	21.7	4.1	145	1.69	0.71	87.49	1.8	high	8.9	4.9		28	10.8
PS	62	M	2	1	23.9	3.1	149	1.04	0.78	102.3	1.8	high	9.4	5.6	0.11	29	7.1
PS	69	F	5	1	20	3.7	166	0.71	0.98	81.74	1.6	high	8.2	5.4	0.1	20	10
PS	77	M	4	1	19.9	4	139	1.33	1.1	94.68	1.3	high	8.7	5.8	0	23	9.1
PS	72	M	3	1	19.8	4.1	187	1.59	1.02	108.8	1.6	high		5.8	0.05	27	9.3
PS	71	F	2	1	18	3.9	179	0.95	0.71	74.65	1.5	high		5.6	0.19	27	9.7
PS	53	M	33	1	16.6	4.3	124	0.99	0.77	64.6	1	high		3.2	0.11	44	11
PS	75	M	4	1	19.1	3.1	80	1.02	0.77	99.7	1.6	high	7.9	8.6	0.3	33	7.5
PS	74	F	16	1	20.5	3.5	177	1.3	0.69	104	1.8	high	10.1	7.3	0.5		10.7
PS	76	M	2	1	19.5	3.8	133	0.96	0.83	97.97	1.3	high	8.6	4.3	0.3	25	10.6
PS	60	M	7	1	21.7	3.7	157	1.19	0.89	109.2	1.1	high	9.7	4.9	0.45	25	10.8
PS	48	M	5	1	25.6	4.6	131	1.26	0.67	130.5	1.8	high	8.9	4.3	0.05	40	10.4
PS	41	M	6	1	24.6	3.9	134	1.5	0.94	109.8	2.1	high	10.2	6.2	0.06	25	10.5
PS	60	M	12	1	23.8	4	115	1.6	1.02	127.9	1.9	high	8.9	4.9	0.06	31	10.4
PS	56	M	5	1	19.7	3.8	217	1.1	0.9	93.52	1.1	high	7.8	4.5		19	9.8
PS	66	M	4	1	22.6	3.6	88	1.36	0.96	128.2	1.5	high	7.9	5.6	0.3	25	8.9
PS	31	F	4	1	16	4.7	133	2.03	0.94	81.19	2.1	high	9.1	4.9	0.1	27	12.9
PS	82	F	12	1	20.3	3.8	105	1.63	0.77	88.21	2.1	high	9.4	5.6			12.6
PS	58	F	2	1	17.8	3.8	175	1.25	1.17	99.04	2.1	high	9.4	6.4	0.88	26	10
PS	58	M	2	1	19.9	4.2	177	1.35	0.96	71.73	1.5	high	9	4.5	0.68		8.9
PS	80	M	5	1	20.2	3.3	172	1.18	0.87	101.9	1.8	high		5.9		26	9.4
PS	63	M	6	1	22.5	4.4	157	1.16	1.08	124.4	1.9	high	10.8	6.7	0.16	28	10
PS	34	F	3	1	23	4.3	151	1.63	0.93	81.67	1.5	high	9.4	4.3	0.19	23	11.5
PS	66	F	3	1	20.9	3.8	98	1.88	1.01	71.04	1.5	high	9.4	5.2	0.07	25	9.5
PS	88	F	5	1	25.3	3.5	101	1.25	0.84	103.2	1.5	high	10.7	7.5	0.05	33	11
PS	75	M	3	1	18.7	3.4	98	1.08	0.85	121.7	1.5	high	8.6	3.1	0.24	27	11.1
PS	66	M	3	1	16.6	3.4	296	1.33	0.88	92.43	2.1	high		7.7	0.29	23	9.7
PS	66	F	13	1	19.4	3.8	144	2	1.03	115.2	1.6	high	8.7	4.7	0.05	30	10.1
PS	59	M	7	1	20.6	4.2	119	1.24	1.04	126	2.1	high	9.5	7.2	0.4	26	11.2
PS	38	M	15	1	18.2	3.4	178	1.63	0.6	73.09	1.5	high	8.7	4.1	0.03	29	8.2
PS	35	M	2	1	29.3	4.4	124	1.32	0.87	55.09	1.5	high		2.1	0.11	24	9.6
PS	55	F	2	1	20.2	4	110	1.19	1.08	102.3	1.5	high		4.3	3.69	26	7.2
PS	69	M	7	1	17.9	3.6	172	0.86	0.68	88.31	1.1	high		5.9	0.08	27	10
PS	50	F	16	1	26	4	139	1.58	0.86	80.98	1.6	high		6.7	0.4	31	10
PS	58	M	3	1	23.1	3.8	210	1.61	1	129.4	1.3	high		5.9	0.1	11	9.8
PS	67	F	18	1	16.9	4	115	1.19	1.15	81.51	2.1	high	7.8	6.4	0.6		8.8
PS	76	M	26	1	23.8	3.8	165	1.51	0.84	105.7	2.1	high	10	6.7	0.1	28	8.1
PS	64	F	18	1	17.2	3.4	156	1.45	1.09	92.96	2.1	high	9.1	6.3	0.1	34	12
PS	72	F	14	1	19.7	4	109	1.22	1.19	91.45	1.5	high	7.4	8.3	0.1	42	8.7
PS	60	M	12	1	24.2	4		1.8	0.94	125.1	1.5	high	8.6	5.5		22	10.9
PS	65	M	11	1	21.5	3.9	233	1.3	1.08	125.3	1.5	high	9.6	3.3	0.03	19	10.6
PS	77	F	10	1	18.8	3.9		1.25	1.3	108.6	1.5	high	8.6	5.4		15	10.2
PS	65	F	9	1	20.5	3.8	168	2.37	0.94	104.8	1.5	high	9.6	5.7	0.26	25	9.2
PS	68	M	8	1	24.2	3.7	156	1.84	0.96	106.3	1.6	high	10	6.1	0.09	25	7.2
PS	56	F	7	1	20.4	3.8	115	1.32	0.98	70.56	1.5	high	8.8	5.5	0.06	26	10.5
PS	54	F	6	1	20.2	4	126	1.74	0.96	77.16	1.8	high	9.5	7.5	0.66	25	9.7
PS	57	F	4	1	22	4.1	156	1.52	0.87	111.3	2.5	high	8.1	5.1	0.06	27	8.9
PS	71	F	4	1	19.4	4	114	1.32	0.81	72.42	1.5	high	9.2	4.3	0	29	10

PS	78	M	3	1	23.4	4	167	1.61	0.94	72.16	1.5	high	8.8	4.8	0.2		11.6
PS	60	M	4	1	23.6	4	134	1.45	0.63	86.03	1.6	high	8.4	5.1	0.88	25	9.2
PS	59	M	26	1	20.7	3.2	142	1.12	0.92	87.78	1.9	high	8.3	3.8	0.06	17	10.3
PS	58	M	22	1	19.1	3.4	170	1.57	0.87	102.3	1.6	high	9.4	5.3	0.1	30	10.8
PS	52	F	13	1	21.1	3.6	122	1.03	0.91	112.6	1.9	high	10.6	4.5	0.4	29	10.4
PS	69	M	11	1	19.9	4	123	1.02	0.8	116.2	1.9	high	8.5	5	0.11	26	10.6
PS	66	F	6	1	18.8	3.8	162	1.34	0.61	100.1	1.5	high	10.6	4.4	0.06	19	11.2
PS	38	M	4	1	24.1	3.8	51	1.48	0.56	110.9	1.6	high	9.5	3.6	0.4		11.3
PS	78	M	4	1	20.5	3.5	107	1.63	0.68	100.2	2.1	high	10	4	0.19	22	10.9
PS	82	M	2	1	21.2	2.9	143	1.53	0.72	68.92	1.5	high	9.4	6.4	0.7	28	9.7
PS	71	M	2	1	23.5	4	137	1.38	0.87	65.91	1.5	high	8.5	5.3	0.1	31	12
PS	66	M	5	1	20.5	3.8	154	1.23	1.18	119.1	2.1	high	10.3	5.8	0.11	22	11.2
PS	53	F	2	1	22.6	3.9	108	1.46	0.64	58.79	1.3	high	7.9	7	0.29	42	9.7
PS	64	F	22	1	16.3	3.7	168	1.82	1.04	121.2	1.8	high		6	0.03	36	12.2
PS	33	M	17	1	21.8	3.6	157	1.71	0.91	74.21	1.3	high	8.9	3.9	0.34	27	8.4
PS	50	M	16	1	19	4	146	1.25	0.8	95.53	1.8	high	9.2	5.1	0.79	23	8
PS	75	M	15	1	19.6	3.8	123	1.07	0.89	121	1.5	high	9.4	3.9	0	24	9.6
PS	53	M	15	1	19.6	4.3	229	1.25	1.02	106.7	1.5	high	10.8	4.4	0.02	31	9.8
PS	64	F	13	1	20.3	3.7	134	1.14	1.2	118.6	1.8	high	8	3.1	2.78	25	10
PS	37	M	12	1	21.5	4.4		1.27	1.04	100.8	1.6	high		4.4	1.43		7.6
PS	59	M	12	1	20.7	3.3	142	1.25	0.86	103.9	1.5	high		5	0.1	2	11.4
PS	58	M	12	1	19.1	4	123	1.07	0.95	115.3	1.6	high	9.2	4.7	4.3	33	9.8
PS	58	M	12	1	22.2	4.3	119	1.53	1.03	110.3	2.1	high	8.5	2.4	0.44	29	9.8
PS	41	M	11	1	22.5	4.5	161	1.5	1.35	142.7	1.3	high	8.6	5.3	0.01	15	11.5
PS	62	M	11	1	20.1	4.4	186	1.53	0.87	116.9	1.8	high	8.5	7	0.07	28	9
PS	36	M	9	1	22.5	4.6	157	1.74	1.13	129.2	1.3	high	8.9	3.7	0.02	34	10.5
PS	33	M	9	1	19.7	4.1	129	1.64	1.04	111.1	1.6	high	12	5.7	6.39		9.5
PS	60	M	9	1	23.1	4.2	217	1.24	1.19	118.7	1.6	high	10	5.9	0	28	8.8
PS	58	F	9	1	19.6	3.9	108	1.29	1.15	128.4	1.6	high		4.9	0.61	37	9.9
PS	66	M	9	1	23.4	3.8	116	1.72	0.91	117.9	1.9	high	9.3	6	0.05	19	8.7
PS	65	F	4	1	22.3	4	191	1.84	0.97	114.5	1.3	high	9.8	5.9	0.04	30	12.3
PS	78	F	4	1	16.6	4.3	168	1.86	0.9	93.36	1.5	high	8	5.7	1.1	35	8.5
PS	73	M	3	1	24.9	3.6	152	1.32	0.78	78.73	1.5	high	9.8	5.5	0.17	25	10.4
PS	61	M	3	1	18.3	3.7	139	0.95	1.02	72.99	2.1	high	9.5	4.1	0.2		10.4
PS	72	M	14	1	27.8		106	1.22	1.06	163.9	2.1	high	9	7.3	5.1	32	10.7
PS	62	F	10	1	17.7		179	1.49	1.07	123.3	2.1	high	11	4.2			9
PS	51	M	10	1	19.9		110	1.65	0.79	72.93	2.1	high	9.1	8.5	0.52	26	16
PS	59	M	9	1	20.6		163	1.15	0.88	99.6	1.3	high	8.1	4.4		30	8.4
PS	68	M	9	1	21.3		152	1.79	1.2	118	1.5	high	7.7	4.1	0.32	34	11
PS	58	F	8	1	23		130	1.71	1.07	133.7	1.5	high	7.5	7.2	0.09	33	8.6
PS	56	M	8	1	25.1		207	1.39	0.98	96.89	1.8	high	9.6	6.7	0.05	21	9.1
PS	55	F	7	1	17.5		170	1.24	0.98	104.8	1.5	high	8.3	4.7	0.1	25	9.1
PS	30	M	7	1	16.9		140	1.41	0.83	97.02	1.8	high		5.5	0.01	25	11.7
PS	67	M	7	1	22.8		90	1.55	0.89	89.28	1.8	high	7.5	5.4	0.1	34	9.2
PS	67	M	7	1	23.5			1.4	0.89	111.1	1.5	high		7.9	0.35	33	11.2
PS	49	M	4	1	26.7		187	1.41	0.72	68.03	1.8	high	9.6	4.5	0.12	19	9.7
PS	66	M	4	1	19.6		129	1.5	0.99	125.8	1.8	high	8.1	6.1			11.3
PS	43	M	4	1	23.7		142	1.71	1.18	94.23	1.8	high	8.7	3.1			9
PS	68	F	3	1	27.9		175	1.04	0.83	119.9	1.3	high	8.4	5.7			9.8
PS	41	M	3	1	20		202	1.19	1.11	128.7	1.6	high	9.1	2.1	0.07	29	8.6
PS	49	M	3	1	23.5		118	1.39	0.73	97.08	1.6	high	9	4	2.6	26	11.8
PS	61	M	14	1	25.7	3.4	99	1.36	0.94	126.1	1.6	high	9.2	5.8	0.07	31	9.7
PS	51	M	3	1	20.4	4.4	154	1.22	0.87	96.7	2.1	high	7.8	3.3	2.8	32	12
PS	57	M	6	1	20.5	4.3		1.45	1.12	94.96	2.1	high	6.4	6.8		24	10.6
PS	60	M	7	1	19.9	3.3	142	2.06	0.92	117	1.5	high	8.5	5	0.77	25	10.3
PS	49	F	12	1	15.7	3.9	192	2.11	1.02	78.03	1.3	high	8.7	4	0.2	49	12.4
PS	77	F	13	1	19.3	3.8	141	1.28	0.73	123.2	1.5	high	8	7.9		28	8.9
PS	62	M	12	1	23	3.6	255	1.75	0.95	109.7	1.5	high	9.6	8.8	0.01	38	8.7
PS	76	F	11	1	19.6	3.5	106	1.06	0.82	125.2	1.5	high	9.9	4.9	8.38	31	9.2

PS	70	F	8	1	17.3	4.1	147	1.27	0.89	106.9	1.3	high	8.3	3.5	0.98	34	12.3
PS	44	M	8	1	22.6	3.8	153	1.07	0.78	102.1	1.8	high	8.4	4.1	0.13	15	10.1
PS	84	M	7	1	21.5	3.8	173	1.33	0.8	118.9	1.5	high		6.2			8.8
PS	72	M	7	1	22.7	4.4	140	2.17	1.08	128.2	1.3	high		5.5			12.5
PS	66	F	7	1	28.4	3.8	118	1.51	0.97	114.7	1.8	high		6.2	0.53	22	10.3
PS	62	M	7	1	20.8	4	124	1.45	1.09	109.2	1.8	high	8.6	4.1			9.9
PS	74	F	5	1	23.4	3.9	180	1.87	0.59	65.91	1.8	high	8.8	4.9	0.29	30	7.9
PS	80	M	5	1	23.5	3.9	98	0.96	0.78	99.38	1.3	high	8.6	3	0.13	6	9.9
PS	76	M	5	1	22.9	3.8		1.37	0.84	140.8	1.5	high	9.3	5.4	0.09	29	10.2
PS	73	M	5	1	18.3	3.6	145	1.45	0.71	84.72	1.8	high	9.6	8.1	0	27	11.3
PS	43	M	4	1	19.5	3.7	192	1.32	1.06	90.91	1.8	high		4.1	0.48	30	9.8
PS	69	F	4	1	22.8	3.9	179	1.9	1.1	124.5	1.8	high	11.1	5.1	0.05	30	11.4
PS	56	F	4	1	18.3	4.1	196	1.08	0.91	118.3	1.9	high		5.5	0.06		10
PS	58	M	3	1	22.5	3.8	106	1.2	0.93	74.8	1.8	high	7.6	4.8	0.7		9.1
PS	79	F	2	1	16.8	3.4	151	1.48	1.01	108.7	0.8	high	7.9	7.2	0.2	27	10.9
PS	78	F	2	1	25.9	3.9	116	1.49	0.77	91.54	2.1	high	9.9	5.1	0.14	27	10.1
PS	62	M	2	1	20.6	3.8	176	0.98	0.62	99.55	1.8	high	7.9	5	0.22	14	9.4
PS	75	F	2	1	22.7	4	175	1.83	0.72	61.92	1.6	high	10.2	5.4	0.03	21	11
PS	72	F	2	1	23.6	3.5	168	1.45	1.03		1.9	high	8.9	5.4		33	10.6
PS	63	M	24	1	20.1	3.9	154	0.98	1.25	112.7	1.6	high		7.1	0.07	20	9.9
PS	78	F	5	1	17.2	3.9		1.32	0.75	97.33	2.1	high	8.8	6.4		23	11.6
PS	72	M	4	1	22.9	4.2	82	1.06	0.84	98.24	2.1	high	8.9	4	0.7	40	11
PS	61	M	3	1	22	4.3	112	1.41	0.67	78	1.8	high	9.3	5.6		20	10.3
PS	53	F	3	1	29.9	4		1.55	1.48	92.78	1.6	high		6.3	0.37	26	11.6
PS	64	F	3	1	20	3.8	149	1.41	0.9	98.26	2.1	high	8.7	5.1	0.01	33	10.6
PS	80	M	2	1	19.2	4.5		1.37	0.9	127.3	2.1	high	7.7	3.1	0.1	23	10.1
PS	77	M	2	1	17.9	4.8	120	1.33	0.81	75.51	1.3	high	9.3	5.7	3.25		10.4
PS	56	F	5	1	17.6	4.4	139	1.64	0.96	90.34	1.9	high	8.7	3.4		30	12.5
PS	70	M	10	1	17.9	4.2	156	1.91	0.98	96.32	1.3	high		6.4			10.8
PS	68	M	7	1	23.8	4.1	210	1.09	0.66	88.56	1.8	high	8.3	5.1		27	
PS	66	M	4	1	22.6	3.9	169	1.31	0.73	109.8	1.6	high		4.6			10.5
PS	60	F	19	1	20.1	3.6	165	1.72	0.9	117.4	1.5	high	8.6	3.8	0		9.9
PS	71	M	25	1	16.6	4.3	105	1.42	1.1	75.86	2.1	high	7.9	4.8			10.7
PS	59	M	12	1	21.8	4.1	186	1.62	0.87	132	2.1	high	8.7	5			11.3
PS	60	M	22	1	22	4.5	166	1.49	0.92	142.5	2.1	high	9.4	5.6			10.3
PS	50	M	23	1	19.7	4	123	1.14	0.75	110.3	1	high		3.5			9.4
PS	67	M	24	1	20.8	3.6	126	1.42	0.96	124.6	2.1	high	9.6	5.9			11.4
PS	63	F	26	1	22.5	3.8	137	1.52	0.7	115.8	2.1	high	8.2	5.3			11.1
PS	61	M	11	1	24.6	4.1	196	1.39	0.83	132.8	1.6	high	8.7	5.3	0.02	23	10.1
PS	58	M	4	1	23	3.9	154	1.65	1.15	129	2.1	high	9.4	4.1			10.2
PS	57	M	4	1	25.8	3.8	139	1.87	0.94	114	2.1	high	8.6	3.5			9.5
PS	43	M	3	1	21.4	4.2	196	1.35	0.61	65.47	1.8	high	9.8	5.6	0	27	10
PS	75	M	3	1	22.3	3.8	220	1.48	0.9	95.27	1.5	high	9.3	5.1	0.1		11.5
PS	64	M	2	1	20.9	4	142	1.4	0.8	151.4	2.1	high	9.5	4.5			8.7
PS	76	M	2	1	27.3	3.5	90	1.62	0.86	101.4	2.1	high	9.3	6.7			11.6
PS	65	M	2	1	22.1	4.3	136	1.09	0.79	122.7	1.6	high	9.1	5.7	0.6	27	10.3
PS	70	M	9	1	17.5	4.3	140	1.2	1.09	150.7	2.1	high	8.6	4.6		46	10.9
PS	52	M	11	1	22	4.4	137	1.61	0.73	123.6	1.8	high	9.3	4.9	0.03	32	11.1
PS	60	M	6	1	22.1	4	197	1.33	0.99	90.91	2.1	high	9.7	9.1	0.1	33	12.4
PS	68	M	14	1	18.9	3.7	154	1.66	0.71	110.4	1.8	high	8.7	7.3	0.05		10.3
PS	86	M	5	1	17	3.8	167	1.85	0.7	78.58	1.3	high	9.2	6.8		26	15
PS	58	M	31	1	22.9	3.4	170	1.47	0.88	121.9	1.3	high	7.6	3.8	0.17	14	8
PS	61	F	22	1	18.5	3.4	191	1.92	0.85	115.4	1.5	high	8.7	7.5	0.06	29	10.8
PS	45	M	6	1	21.2	4	110	1.25	1.04	108.1	2.1	high		6.2	0.03	27	9.5
PS	60	M	14	1	18.1	4	95	1.24	0.88	83.13	1.8	high	9	6.6	0.58	29	9.2
PS	60	F	27	1	20.2	3.7	138	1.31	1.37	93.57	1.8	high	10.4	8.5	0.26	32	10
PS	86	F	8	1	22.2	4	136	1.24	0.96	134	1	high	9.4	4.1	1.38	38	7.4
PS	57	M	2	1	20.5	3.9	175	1.61	0.85	104.9	2.2	high	9.2	5.6	0.05		11.1
PS	45	M	19	1	20.3	3.7	158	1.35	1.03	115.4	2.2	high	11	7.3		26	9.2

PS	42	M	5	1	21.5	3	169	0.85	0.75	95.18	1.6	high	8.2	4.1	0.7	32	11.2
PS	64	M	2	1	24.7	3.6	171	1.34	0.79	95.14	1.8	high	9	6.4	0.15	25	10.5
PS	54	M	8	1	20.4	3.9	171	1.54	0.83	65.75	2.1	high	7.8	4.2	0.95	29	12.8
PS	77	F	8	1	16.7	4.2	128	1.7	0.82	123.9	1.9	high	8.6	4.8		22	10.8
PS	67	M	7	1	23.5	3.9	147	1.73	0.82	114.3	1.9	high	8.1	5.4		20	11.1
PS	61	M	2	1	20.1	3.1	237	1.65	0.87	65.35	1.5	high	8	3.8	0.11	29	10
PS	69	F	2	1	21.4	3.5	127	1.61	0.63	91.49	1.9	high	7.7	4.9		28	9.3
PS	86	M	2	1	19.2	3.2	155	1.39	0.72	43.05	1.9	high	7.8	5.9		29	10.3
PS	77	F	10	1	18.8	4.1	130	1.57	0.98	133.1	1.8	high	8.9	3.8	0.01	32	9.9
PS	54	M	10	1	19.8	3.5		1.4	0.83	68.97	1.6	high	8.5	4.1	0	25	12.4
PS	55	M	15	1	16.6	3.5	182	1.72	0.56	59.94	2.1	high	10.3	3.8	0.05	32	8.5
PS	63	F	11	1	26	4	213	1.85	1.04	120.9	1.1	high	9.4	3.7	0.01	28	9.6
PS	68	F	11	1	17.3	4	134	1.16	1.01	95.32	1.5	high	8.7	5.5	0.2	28	12.4
PS	60	M	10	1	18.1	3.9		1.7	0.92	113.7	1.8	high	9.5	4.4	0.05	17	11
PS	82	M	8	1	19.5	3.2	210	1.22	0.9	118.8	1.3	high	9.8	6.6	0.23	32	11.3
PS	79	M	8	1	22.4	3.7	143	2.06	1.06	123.9	1.8	high		6.9	0.07	33	10.7
PS	64	F	6	1	24.4	3.6	100	1.26	0.74	101.4	2.1	high	8.9	5.4	0.14	27	9.4
PS	60	M	5	1	20.3	4.4	186	1.23	1	116.1	2.1	high	9.8	5.8	0.97	28	12.7
PS	70	M	4	1	23.7	4.3	182	1.53	0.77	142.3	2.1	high	8.7	5	0.46	25	12.4
PS	79	M	3	1	20.1	3.7	147	1.25	0.83	67.71	1.8	high	8.4	8.3	0.18	28	10.4
PS	80	M	3	1	26.3	4.1	200	1.17	0.95	107.1	1.6	high	10.4	6.2			9.6
PS	60	F	2	1	29.5	4.2	233	1.7	0.8	86.19	1.8	high		5.2			10.1
PS	72	M	2	1	19.2	4.1	137	1.53	0.72	122.4	1.6	high	8.8	4	0.5	31	7.2
PS	61	M	2	1	26.7	4.5	152	1.24	0.84	121	2.1	high	9.4	7.2	0.13	27	10.6
PS	48	M	15	1	17.8	4		1.44	0.69	100.5	2.1	high	9	3.1	0.47		11.7
PS	60	F	12	1	20.5	3	145	1.26	1.3	127.5	1.8	high	7.3	5.1	0.06	28	9.3
PS	38	F	9	1	20.2	4	162	1.54	0.79	101.7	1.5	high	9.7	3.2	0.2	31	9.6
PS	56	F	8	1	17.2	3.4	190	1.36	0.91	93.97	1.1	high	8.5	4.9	1.9	22	10
PS	56	F	8	1	20.3	3.4	166	1.33	0.84	80.26	2.1	high	10.5	6.5	1.4	28	11.4
PS	61	M	8	1	21.5	3.3	171	2.02	1.12	111.3	1.5	high	9.2	4.3	2.2	29	12.2
PS	61	M	7	1	28.6	3.1	138	1.17	0.84	73.02	1.8	high	9.7	5.4	0.24	36	11.3
PS	57	F	5	1	18.4	3.5	158	1.24	0.8	109.7	1.6	high	8.2	3.7	0.24	38	10.3
PS	64	F	4	1	29.5	3.4	100	1.37	1.13	93.6	2.1	high	8	8.7		55	9.1
PS	61	M	3	1	18.6	2.8	119	1.35	0.74	90.91	2.1	high	9.8	5.2	0.1	26	10.9
PS	63	M	2	1	24.5	3.3	187	1.66	0.53	84.52	1.6	high	10.3	4.5	0.02	31	9.3
PS	69	M	2	1	23.5	2.6	160	1.22	0.67	109.4	1.9	high	10	4.4		28	11
PS	81	F	6	1	18.1	3.9	191	1.13	1.24	104	2.1	high	7.8	4.4	0.2		9.2
PS	74	M	2	1	18.9	3.4	179	2.7	0.63	59.98	2.1	high	8.4	4.5	0.45	29	9.9
PS	39	M	15	1	23	4.2	157	1.57	0.87	133.2	1.8	high	8.9	4.6	0.05	26	11.3
PS	59	F	7	1	18.9	4	211	1.29	0.99	122.5	1.8	high		7	0.08		10.4
PS	59	F	13	1	15.6	4.2	171	1.81	0.81	109.6	1.9	high	8.9	3.8		27	10.4
PS	57	M	28	1	21.9	4.5	114	1.55	0.71	99.03	1.4	high	7.8	4.5	1.24	28	9.7
PS	83	F	12	1	19.3	3.6	93	1.6	0.8	119.7	1.1	high	7.7	5.3			9.3
PS	72	F	6	1	16.8	3.9	162	1.45	1.2	81.4	1.8	high	10	5.2	0.07	24	11.4
PS	44	M	10	1	21.8	3.7	111	1.63	1.1	89.19	1.3	high	9.2	5.9	0.23	26	11.5
PS	68	F	24	1	16.8	3.6	176	2.25	1.03	129.1	1.9	high	8.9	4.3	0.01	35	9.5
PS	39	M	26	1	13.9	4.1	178	1.02	1.23	71.13	1.6	high	8.1	4.1	0.1	30	10.5
PS	64	M	22	1	20.1	3.7	172	1.55	0.96	122.5	1.9	high	8.1	4.7		31	10.1
PS	70	F	19	1	23.6	3.8	206	1.55	1.05	158.3	1.3	high		7.4		27	9.3
PS	72	M	16	1	18.6	4.2	143	1.35	0.95	88.44	2.1	high	9.2	5	0.06	25	10.8
PS	80	M	13	1	23.3	3.5	173	1.67	0.73	105	1.6	high	9.4	3.9	0.04	26	10.1
PS	69	F	11	1	21.5	3.5	181	2.03	0.74	128.8	1.3	high	7.9	4.9	0.17	14	11.3
PS	55	F	10	1	15.8	4	162	1.63	0.83	102.3	2.1	high	9.4	4.2	0.06	18	9.3
PS	80	F	9	1	19.2	3.7	88	1.32	0.79	108	1.5	high	8.8	4	0.07	25	10.1
PS	49	F	9	1	16.9	3.8		1.09	0.93	109.3	1.3	high	9.9	5.5		32	11.3
PS	65	M	7	1	23.7	4	143	1.34	0.98	163.4	1.6	high	9.1	3.5	0.72	32	10.6
PS	67	F	7	1	18.8	3.8	140	0.98	1.31	94.32	1.6	high		5.3	0.05	26	9.8
PS	68	M	6	1	21.2	3.6	183	1.81	0.75	121	1.6	high	8.7	3.1		39	11.2
PS	58	M	4	1	17.3	4	172	1.69	1	98	1.8	high	10.6	8.4	0.03	29	11.2

PS	45	F	3	1	18.2	4	145	1.25	0.97	95.33	2.2	high	10	6	0.5	29	12.1
PS	41	F	3	1	25.6	4	211	1.69	1.03	114.3	1.6	high	9.2	6.8		28	11
PS	78	M	3	1	19.2	3.7	249	1.51	0.91	99.99	1.1	high	9.3	3.1		16	8.5
PS	50	M	3	1	23.8	4.1		1.5	1.18	83.39	1.5	high	9	3.4	0.1		9.2
PS	64	M	2	1	20.2	3.8		1.51	1.04	134.6	1.6	high	8.6	4.1	0.09	29	10.5
PS	74	F	2	1	17.7	3.9	136	1.31	0.96	113.9	2.1	high		6.2			10.4
PS	59	M	2	1	23.5	4.5	174	1.49	0.78	131.4	1.5	high	9.8	6.3	0.64	27	10.4
PS	68	F	16	1	21.6	4.2	119	1.47	0.82	130.8	2.1	high	8.4	4.5	0.09	29	11
PS	72	M	12	1	21.2	3.9	112	1.34	0.92	110.9	2.1	high	8.9	4	0.06	26	9.9
PS	71	F	21	1	17.7	4.1	143	1.45	1.12	143.9	2.1	high	8.8	5.5	0.06	25	9.5
PS	67	M	4	1	24.9	4	145	1.08	0.88	119	2.1	high	9.9	4.7	2.05		8.5
PS	63	M	5	1	19.5		193	1.91	0.66	82.4	1.6	high	9.4	4.9	4.49	42	11.3
PS	57	F	15	1	24.8	4	179	1.1	0.88	121.9	1.8	high	9	7.6	0.44	29	11.6
PS	61	F	12	1	22.3	3.6	198	1.75	1.02	113.1	1.8	high	9.2	5.6		20	11.1
PS	59	M	2	1	22.8	4.4	176	1.42	1.02	104.1	2.1	high	9.3	7	0.28	29	10.5
PS	77	M	6	1	22.5	3.8	188	1.5	0.87	82.2	1.2	high	9.2	5.7		20	10.7
PS	53	M	19	1	21.5	4.2	155	1.67	1	79.92	1.9	high	9.2	4.8		26	11
PS	68	M	8	1	19.4	3.4	156	1.68	1.05	110.6	1.9	high	9.2	4.9		24	9.5
PS	56	F	2	1	31.4	3.7	157	1.6	0.81	90.54	1.6	high	10.1	6.7	0.05	38	12.3
PS	69	F	3	1	23.7	4.2	194	1.42	0.81	74.22	2.1	high	8.7	6.1	0.6	37	10.1
PS	59	F	3	1	21.5	4.1	163	1.24	0.7	94.44	1.3	high	8.1	5.9	0.8	18	10.6
PS	55	M	7	1	18.7	3.9		1.48	0.96	108	1.6	high	8.7	4.7	1.24	22	7.7
PS	62	F	8	1	20.6	3.7	191	2.35	0.81	123.9	1.6	high	8.2	6.1		28	11.7
PS	60	M	12	1	15.2	3.9	158	1.15	1	116.1	1.9	high	8.3	6.3		26	11.2
PS	80	M	5	1	21.7	3.4	164	1.86	0.88	141.2	1.8	high	9.9	6.2	0.01	25	11.5
PS	62	M	15	1	20.5	3.4	133	1.24	1	110.1	2.1	high	9.5	4.4	1.86	27	10.4
PS	43	M	28	1	27.7	4	126	1.62	1.16	112.3	1.9	high	8.9	4.5		26	10.3
PS	49	M	25	1	19.6	4	183	1.42	0.89	63.6	1.8	high		5.3	0.55	28	10.9
PS	57	F	24	1	18	3.9	194	1.42	1.06	98.75	1.8	high		4.5	0.02	26	12.5
PS	61	M	24	1	23	3.3	171	1.38	0.81	130.3	1.8	high		3.5	0.48	27	11.1
PS	56	M	22	1	19.5	3.4	130	1.68	0.93	82.45	1.6	high	9.3	4.2	0.03		7.5
PS	54	M	17	1	19.4	4.1	141	1.1	0.95	116.1	2.1	high	9.4	5.3	0.06	33	11
PS	51	M	2	1	27	4.1	148	1.32	0.69	103.1	2.1	high	10.6	6.5	1.12	32	11.8
PS	50	M	15	1	18.1	4.2	127	1.29	1.23	109	1.9	high	8.8	3.4		29	9.9
PS	69	M	14	1	21.9	3.6	153	1.34	0.92	154.7	1.6	high	9.3	4		20	10.1
PS	73	M	14	1	22.2	4	193	1.26	0.9	148.2	2.5	high	11.1	6.9	0.2	16	11.1
PS	57	M	14	1	17.7	3.6	157	1.64	0.8	121.6	2.1	high	9.1	3.5	0.05		10.3
PS	72	F	11	1	27	3.7	166	1.31	0.98	85.55	1.5	high	10	3.7			11.6
PS	73	F	9	1	19.7	3.7	174	1.67	1.02	98.76	1.1	high	8.2	6.6	0.1	27	11.6
PS	75	F	9	1	16.1	3.6	110	1.17	0.83	109.3	1.6	high	19	6.5		34	6.8
PS	62	F	8	1	15.9	3.8	212	1.89	1.12	109.2	1.9	high	10.6	5.2		26	9.9
PS	72	M	8	1	27.1	3	88	1.24	0.54	107.8	2.1	high	7.9	5.4	1.36	25	9.4
PS	67	F	6	1	22.1	3.8	150	1.45	0.98	92.13	1.5	high	9.7	4.5	0.7	29	10.8
PS	71	F	5	1	29.9	3.5	158	1.49	0.79	105.7	1.6	high		3.8	0.05	24	9.9
PS	44	M	5	1	19.5	4.1	172	1.58	0.84	97.79	1.6	high	8.2	7.4	0.23	27	9.8
PS	56	F	2	1	21.1	4.1		1.41	0.78	80.29	1.8	high	7.3	6.9		37	13.2
PS	70	M	2	1	21	4.1	116	1.4	0.78	85.76	2.1	high	9	6.8	0.03	20	11.3
PS	38	M	2	1	33.1	3.5	178	1.71	0.97	121.7	1.8	high		4.6	2.37	14	8.1
PS	48	M	2	1	35.9	3.7	149	1.95	1.12	64.96	1.5	high		5.7	0.1	29	10.3
PS	58	F	20	1	16.9	3	220	1.87	0.81	44.37	1.9	high	8.6	3.9		27	11.4
PS	62	M	6	1	22.1	4.1	158	1.4	1	125.9	1.8	high	10	4.9	0.18	24	11.3
PS	68	F	8	1	20.8	3.6	141	1.64	0.54	67.69	1.5	high	10.3	5.4	0	22	10.7
PS	84	F	5	1	23.4		31	1.3	1.13	104.5	1.6	high	9.1	5.5			10.8
PS	76	M	2	1	20.6	4.5	188	1.48	0.99	100.4	1.8	high	7	6.1	0.11	29	10.9
PS	62	M	5	1	21.6	4	123	1.45	0.8	115.9	2.1	high		6.2	0.15	23	11.5
PS	79	F	3	1	18.7	4	161	1.02	0.99	36.26	1.8	high	9.9	4.8		28	10.3
PS	65	M	20	1	21.8	3.5	159	1.56	0.71	77.93	1.8	high	8.1	4.5			11.4
PS	72	F	12	1	16.5	3.9	129	1	0.94	111.8	1.5	high	8.8	3.1	0.48	31	11.9
PS	76	M	12	1	18.6	4	122	1.26	0.47	41.55	1.8	high		6.8	0.04	25	9.8

PS	42	M	4	1	20.3	3.6	159	1.88	0.82	86.97	1.5	high	9.9	5.4			9.6
PS	61	M	3	1	20.5	3.8	219	2.04	1.11	102.3	1.8	high	8.3	5.5			10.6
PS	54	M	33	1	17.1	4.3	158	1.45	0.96	78.86	2.1	high	10	4.2			12.1
PS	57	F	30	1	17.8	4.1	149	1.36	1.01	127.9	1.5	high	8.6	6.1	0.04	23	12.5
PS	46	M	28	1	20.3	4.4	164	1.19	0.98	108.6	1.3	high	8.7	4.6	0.02	25	11
PS	70	F	25	1	22.8	3.8	131	1.66	1.09	106.1	1.4	high	11.1	4.7	0.32		9.8
PS	50	F	22	1	21	3.9	125	1.45	0.91	111	1.5	high	9.5	5.1			10.7
PS	63	M	12	1	22.4	3.7	170	1.45	0.93	121.8	1.5	high	10.4	8	0.55	28	9.4
PS	65	M	5	1	20.2	3.5	133	2.11	0.91	85.88	2.1	high	8.7	4.8	0.05	24	8.7
PS	78	F	3	1	19.2	3.9	128	1.27	0.78	48.76	1.8	high	9.2	4.6		29	11.4
PS	54	M	14	1	18.5	3.9	180	1.53	0.82	88.79	1.5	high	9.1	5.4		27	10.1
PS	50	M	21	1	21.3	4.4	149	1.34	0.93	123.2	1.5	high	8.2	4.4			10.3
PS	66	M	18	1	19.7	4.1		0.84	0.87	114.4	1	high	8.9	5.1		38	8.2
PS	62	M	15	1	18.4	3.6	166	1.8	0.58	64.63	1.8	high		5	0.06	25	9.9
PS	39	M	12	1	20.7	4.6	157	1.31	0.96	115.1	2.1	high	8.9	6.1			11.5
PS	74	F	3	1	21.4	3.7	144	1.44	0.87	111.5	1.5	high	8.1	5.2	0.13	35	12.2
PS	62	M	12	1	22	4.3	195	1.59	1.42	96.11	2.1	high	8.7	4.3		28	11.4
PS	54	M	16	1	19.9	3.9	185	1.25	0.95	102.5	1.8	high	8.8	7.3	0.13	27	10.4
PS	47	M	15	1	20.9	4	93	1.37	0.67	80.34	2.1	high	10.1	3			10.5
PS	61	M	15	1	23	3.4	113	1.25	0.68	92.93	1.8	high	8.7	4.4	5.42	37	11.4
PS	68	F	9	1	16.8	3.7	139	1.61	0.93	81.84	2.1	high	9.1	4.5	0.25	28	14.4
PS	49	M	2	1	21.6	4.5	128	1.39	1.06	114.5	1.5	high	9	5.9	0.01	24	11
PS	68	M	11	1	20.9	3.8	125	1.06	1.11	99.82	2.1	high	9.6	5.7	0.05	30	12.3
PS	71	M	23	1	19.1	3.3	136	1.71	0.83	79.88	1.8	high	9.6	4.1	0.92	25	10.5
PS	67	M	13	1	18.1	3.9	147	1.77	1.07	127.9	1.3	high	9	4.7	1.87	28	11.2
PS	50	F	21	1	16.3	3.7	124	1.47	0.61	76.04	2.1	high	8.9	5.6	0.06	40	10.8
PS	65	M	3	1	20.6	3.5	164	1.21	0.79	79.34	1.5	high	8.7	5.3	3.25	36	8.8
PS	44	F	6	1	27.9	3.8	127	1.13	0.65	106.3	2.1	high	7.9	6.5	0.37	25	11.1
PS	77	F	2	1	23.5	3.4	126	1.36	0.58	49.6	1.5	high	9.5	5.4	0.19	20	12.2
PS	67	F	6	1	23.4		149	1.27	0.79	84.23	1.5	high	9.9	6.3	0	30	12.3
PS	72	F	9	1	19.8		149	1.79	0.94	102.3	1.1	high	8.9	6.2	0.1	29	9.4
PS	71	M	2	1	18	3.7	187	1.45	0.47	68.2	2.1	high	8.3	4.6	0.01	23	10.2
PS	54	F	3	1	30.6	3.8	149	1.56	0.59	85.75	1.6	high	9	5.8	0.07	25	10.2
PS	58	M	2	1	18.4	4	149	1.3	0.71	80.52	1.5	high	8.4	5.8	0.61	20	9.8
PS	61	F	2	1	18.9	3.9	132	1.45	0.96	72.02	1.3	high		6.3	0.66	24	11.3
PS	63	F	2	1	14.5	3	155	1.21	0.75	65.27	1.3	high	8.3	5.6	0.19	23	10.4
PS	90	F	2	1	20.8	3.7	184	1.57	0.9	61.24	1.8	high	9.7	6.3	0.29	36	11.5
PS	51	F	18	1	16	3.4	163	2.05	0.89	64.53	1.3	high	8.7	3.1	3.4	24	9.2
PS	61	F	18	1	20	3.6		1.52	1.01	83.79	1.1	high	10.1	6.2			13
PS	58	F	16	1	18.3	4.3	156	1.61	0.94	120.9	1.6	high	7.4	4.5		25	10.6
PS	67	F	13	1	17.1	3.8	197	1.64	1.1	135.8	1.9	high	8.8	3.8		25	9.9
PS	67	M	2	1	22	4	160	1.29	1.1	129.2	1.9	high	8.5	5.9		28	10.6
PS	63	M	8	1	21.1	3.9	117	1.32	0.95	122.3	2.2	high	9.6	5.8	0.45	31	12.3
PS	62	M	3	1	18.4	3.8	127	1.47	1.01	81.42	0.6	high	9.7	6.3	0.2	29	8.5
PS	57	M	5	1	22.4	3.8	79	1.32	0.99	120.3	2.1	high	8.5	3.7	0.12	26	9.6
PS	60	M	6	1	21.1	3.8	165	1.29	0.52	99.44	2.1	high	9.6	5.6		32	12.2
PS	51	M	6	1	25.1	4.3	172	1.25	0.97	127.7	2.1	high		5.2	0.2	33	10.7
PS	39	F	8	1	24	4	120	0.97	0.62	124	2.1	high	8.6	6.3			
PS	61	F	6	1	29.2	4	188	1.61	0.69	115.9	1.8	high	10.6	5.5	0.05	29	11.1
PS	78	M	10	1	21.9	3.6	121	1.45	1.17	130	2.1	high		3.6	0.05	24	10.7
PS	60	M	24	1	19.3	3.8	217	1.79	0.66	91.95	1.8	high	9.6	4.2		29	9.8
PS	69	M	8	1	20.5	4	169	1.63	0.74	107.7	1.4	high	9.1	4.2	0.05	33	10.4
PS	58	M	17	1	24.9	4	122	1.2	0.81	131.3	2.2	high	9.2	4.5	0.12	29	11.3
PS	71	M	3	1	21.3	3.9	156	1.29	1.21	102.8	1.5	high	8.3	8.7	0.27	32	9.1
PS	73	F	24	1	22.2	3.7		1.59	0.84	100.3	1.4	high	9.3	7.1		20	11.7
PS	80	M	7	1	21	3.5	115	1.28	0.82	71.63	2.1	high	9.1	5.9		21	11.4
PS	68	M	4	1	21.4	3.9	171	1.31	0.86	95.32	2.1	high	10.4	5.5	0.24	33	12.4
PS	62	M	8	1	22.5	4.1	134	1.57	0.92	153.2	1.5	high	10.8	4.6			10.8
PS	77	F	4	1	20.9	3.7	190	1.26	0.84	105.1	1.1	high	8.6	3.9	0.32	24	8.7

PS	74	M	2	1	18.6	3.7	188	1.66	0.84	61.7	1.6	high		6.3	0.05	23	10.8
PS	80	M	4	1	20.5	3.5		1.17	0.8	146.5	1.5	high	9.9	6.3		27	8.8
PS	68	M	3	1	22.2	3.7		1.29	0.84	96.8	1.8	high	9.3	4.3	1.11	35	9.8
PS	79	F	8	1	20.2	4	198	1.63	0.84	141.9	1.8	high	9	7	0.05	27	11.3
PS	62	M	14	1	25	3.5	126	1.3	0.87	96.54	2.1	high	9.8	4.2	0.01		9.8
PS	72	M	9	1	22.4	3.7	144	1.37	0.7	135	2.1	high	9.2	6		20	9.6
PS	54	M	4	1	18.1	4	198	1.85	0.59	45.09	2.1	high		4.8	0.16		
PS	52	F	7	1	19.5	4.3	148	1.3	1	125.1	2.1	high		5.7	0.05		
PS	77	M	7	1	19.8	3.6	179	1.76	0.64	112.6	2.1	high		6.4	1.17		
PS	70	F	6	1	18.3	3.6	181	1.6	0.87	96.37	2.1	high		5.1	0.05		
PS	55	F	7	1	14.1	3.8	251	1.84	0.57	86.86	2.1	high		5.2	0.05		
PS	67	M	3	1	20.6	3.8	156	1.69	0.7	86.39	2.1	high	8.6	4.7	0.08		11.2
PS	58	F	19	1	19.4	3.9	137	1.3	0.96	124.8	2.1	high		4.5	0.05		
PS	49	F	4	1	21.1	4.3	131	1.1	0.93	145.7	1.8	high	8.1	3.7	0.05	28	10.4
PS	51	F	16	1	22.5	3.3	126	2.32	1.08	114.9	2.1	high		5	0.06		
PS	63	M	16	1	17.5	3.8	161	1.55	0.82	125.7	0.8	high	8.7	4	0.24	30	8.8
PS	62	F	14	1	15.3	4	134	1.16	1.01	136.6	1.4	high	9	5.1	0.4	33	9.8
PS	59	F	14	1	15.3	4	187	1.13	0.88	135.2	2.1	high	8.8	4.8			8.9
PS	64	F	12	1	21.4	3.7	97	1.15	1.04	124.3	2.1	high	9.1	4.5	0.65	22	10.3
PS	59	M	4	1	20.9	3.5	161	1.27	0.86	37.56	1.8	high	10.7	5.7	0.1	32	12.4
PS	66	F	9	1	14.2	3.8	107	1.36	1.04	147.8	1.5	high	9.4	7.9	0.12	39	7.6
PS	50	F	8	1	22.8	4.1	135	1.54	1.09	127.5	2.1	high		5.5	0.5		
PS	38	M	4	1	26	4.1	164	2.17	1.39	103	2.1	high	9.6	4.6	1.12	31	12.2
PS	60	F	7	1	20.1	3.3	108	1.7	1.05	72.76	1.8	high	8.4	4.8	0.42	25	11
PS	43	F	3	1	17	3.9	176	1.79	0.95	74.1	2	high	9.3	9.7	0.04		11.5
PS	44	F	10	1	23.5	3.7	172	1.36	0.7	128.4	2.1	high	9.9	4.7	0.11	25	10.2
PS	66	F	10	1	18	3.8	122	1.86	0.68	91.67	1.4	high	8.2	6.6	0.35		12.2
PS	62	F	2	1	23.6	4.1	146	1.28	0.95	136.9	1.6	high	8.9	5	0.2		11.3
PS	45	F	2	1	17.5	3.8	86	1.59	1.1	124.1	1.3	high	11	4.6	4.92	34	10.3
PS	49	M	2	1	18.5		166	2	0.77	96.97	1.6	high	9.9	4.7	0.02	23	9.7
PS	56	M	23	1	23.5	3.8	166	1.71	0.81	110.5	1	high	10	5.1	0.56	33	10.3
PS	48	M	25	1	19	4.2	152	1.3	0.9	93.31	1.8	high	10.6	2.7			
PS	38	M	5	1	23.1	4.7	160	1.77	0.91	80.24	1.8	high	8.4	6.3		66	10.2
PS	58	M	28	1	15.9	3.5		1.72	1.03	107.9	1.6	high	9.9	6		17	7.1
PS	68	M	14	1	18.2	3.8	172	1.23	0.93	63.1	1.8	high	9.6	5.7	1.14	27	11.2
PS	59	M	13	1	23.4	3.8	166	1.37	0.88	135.5	1.5	high	9.2	3.8	0.12	22	11
PS	50	M	9	1	21.2	3.7	107	1.42	0.98	104.8	1.8	high		6.1	0.2	35	12.2
PS	63	M	7	1	20.7	3.5	187	1.84	1.17	72.36	1	high	9.2	3.4		26	8.6
PS	57	M	6	1	22.5	3.9	136	1.36	1	92.2	1.5	high	7	9.3		29	11.1
PS	74	F	5	1	19.1	3.1	118	1.41	0.99	67.11	1.4	high	8.3	4.8	0.18	26	9.7
PS	68	M	2	1	27.2	3.8	188	1.31	0.85	85.7	1.5	high	8.9	4.6	0.3		11.6
PS	74	F	8	1	20.5		192	1.43	0.92	68.74	1.1	high	8.9	3.8	0.3	27	11.8
PS	68	F	9	1	21.7	3.5	144	1.16	0.75	100.2	1.5	high		3.7		27	9.6
PS	63	F	28	1	14.7	3.8	220	1.23	1.14	90.37	0.8	high	8.5	3.8	0.3	14	10.5
PS	58	M	17	1	19.2	4.8	147	1.64	0.63	76.57	1.3	high	8.2	6.4	0.14	27	9.4
PS	56	F	16	1	18.2	4.4	157	1.84	0.88	109.4	1.1	high	9.4	4.7	0	33	11.1
PS	61	F	14	1	26.4	4.2	80	1.01	0.9	96.88	2.1	high	9.6	4.4	2.34	43	9.8
PS	37	M	13	1	23.7	4.3	39	1.28	1.21	111.6	2.1	high	10	6.4	0.05	23	11.6
PS	63	F	12	1	17	3.7	149	2.26	0.86	81.01	1.5	high	8.7	4.3	0.1	26	11
PS	49	M	12	1	23.8	3.9	153	1.42	0.75	111.2	1.5	high	8.9	4.6	0.66	27	9.9
PS	48	F	10	1	19.3	4.2	159	2.33	0.91	117.3	1.8	high	9.2	5.1	0.05	40	10.8
PS	42	F	10	1	20.5	4.2	123	1.74	0.77	98.42	2.1	high	8	5.5	1.1	26	11.6
PS	72	F	9	1	24.3	3	134	1.55	0.7	72.88	2.1	high	8.2	3.9		26	10.1
PS	55	M	8	1	21.4	4.3	124	1.26	1.33	118.9	1.8	high	8.1	6.4		22	8
PS	61	F	8	1	21.1	4.1	143	1.79	1.27	127.5	1.8	high	10.1	4.2	0.2	22	11.9
PS	43	M	7	1	20.1	4.2	177	1.35	0.89	95.54	1.5	high	9	5.9	0.02	21	11.8
PS	49	M	7	1	23.5	4	103	1.25	0.6	100.1	2.1	high	9.2	7.2	0.05	25	12
PS	62	M	7	1	16.7	4.7	127	1.49	0.96	83.68	1.3	high	9.8	5.4	0.2	19	11.8
PS	46	F	6	1	24.8	4.1	113	1.57	1.09	110.3	2	high	8.5	3.9	0.45	38	

PS	68	M	6	1	17.7	4	262	1.6	0.84	134.2	1.6	high	9	6	0.09	28	10.7
PS	49	F	5	1	26.5	4.4	156	0.95	0.76	124.8	1.8	high		9.2		21	8.2
PS	60	M	5	1	20.9	4.4	168	1.38	0.74	106.7	2.1	high	10.3	6	0.03	23	12.3
PS	55	M	5	1	26.7	4.4	169	1.59	0.99	114.3	1.8	high		4.1	0.31		
PS	38	F	5	1	17.4	3.9	248	1.2	0.87	98.32	2.5	high	10.4	8.3	0.2	30	11.8
PS	35	F	5	1	22.1	4	122	1.8	1.15	121.1	2.5	high	8.9	5.6	0.4	29	9.1
PS	53	F	4	1	20.3	4.3	125	1.77	0.8	118	1.8	high		2.8	2.97	33	9.8
PS	86	F	4	1	21.4	3.9	161	1.55	0.87	118.6	1.5	high	8.5	6.5	0.52	29	9.5
PS	77	F	4	1	24.4	3.7	134	1.53	0.65	62.94	2.1	high	9.3	3.9	0.1		13
PS	65	F	4	1	30.7	4	131	1.4	0.8	120.8	2.1	high	9.7	4	1.3	26	9.2
PS	62	M	4	1	21	4.4	146	1.25	0.7	106.6	2.1	high	9.6	9.2	0.2	24	10
PS	49	M	4	1	29	3.9	24	1.54	0.62	45.08	1.6	high	9.8	6.2	0.2	25	9.4
PS	63	M	4	1	26.4	4	110	1.56	0.83	100.4	1.6	high	9.5	6.9	0.04	21	9.3
PS	70	F	4	1	25.1	4	119	1.42	0.9	97.41	1.3	high	9.5	3.8		34	9.7
PS	56	M	3	1	21	4.6	117	0.72	0.81	81.62	2.1	high	8.9	5.6	0.81	25	10.2
PS	87	F	3	1	21.3	3.8	109	0.76	1.22	112	2.1	high	9.3	7	0.07	21	10.4
PS	72	M	3	1	21.3	3.9	101	1.16	0.75	114	2.1	high	8.9	7.4	0	24	12.2
PS	72	F	3	1	27.1	4		1.2	0.92	120.2	1.8	high	9	7	0.1	27	10.1
PS	75	F	3	1	27.8	3.6	138	1.3	0.85	110.5	1.5	high	9.8	3.4		38	10.3
PS	66	M	3	1	20.1	4.4	129	1.37	0.69	83.14	2.1	high	9.8	5.8	0.1	26	11.5
PS	60	F	3	1	19.6	4.1	121	1.32	0.69	83.86	2.1	high	8.8	3.8	0.21	23	12
PS	49	M	2	1	23.1	4.1	177	1.23	0.81	116.6	1.5	high	9	4.2	0.13	29	10.8
PS	64	M	3	1	17.4	4.1	137	1.35	0.98	76.66	1.8	high	8.9	4.8	0.25	17	9.8
PS	59	F	27	1	18.9	3.6	124	1.79	1.15	83.31	1.6	high	7.4	5.5	0.99	32	8.3
PS	58	M	8	1	18.2	4	136	1.65	0.69	88.97	1.5	high	10.4	6.3	0.03	29	7.7
PS	74	M	22	1	22.7	3.2	166	1.75	0.77	100.2	1.4	high		2.4	2	20	9
PS	79	M	9	1	18.6	3.1	216	1.7	0.72	132.8	1.8	high	8.6	5.4	0.11	26	11.9
PS	79	F	4	1	21.3	3.8	151	1.13	0.81	151.5	2.2	high		8.3	0.2	27	9.5
PS	60	M	4	1	24.5	3.6	158	1.21	0.84	139.8	2.2	high	9.2	5.3	0.07	36	
PS	67	M	13	1	18.4	3.6	165	1.28	0.8	106.9	2.2	high		5	0.3	38	11.5
PS	83	F	6	1	13.7	3.5	129	1.19	0.83	65.92	1	high	9.5	2.9		27	9.9
PS	57	F	23	1	16.6	3.4		1.41	0.7	82.63	1.9	high	8.7	4.1		27	12
PS	59	M	18	1	18.5	3.6	182	1.57	1	106.8	2.1	high	8.1	6.9			
PS	54	F	2	1	26	3.6		1.98	0.85	95.41	1.5	high	8.3	5.2	1.34	31	10.5
PS	44	F	21	1	18	3.7	163	1.78	0.68	95.11	1.4	high	8.4	5.7	0.05	26	11.2
PS	68	M	10	1	14.5	4	147	1.57	1.18	113.7	1.4	high	9.6	3.7	0.05		9.3
PS	46	M	12	1	29	3.7	172	1.2	0.71	92.89	1.5	high	11	6			9.3
PS	28	M	5	1	17.3	4.3	139	1.8	0.55	83.05	1.6	high		6.7			10.4
PS	79	F	8	1	20.3	3.3	181	1.13	0.51	99.33	1.5	high		9.4		23	9.2
PS	74	M	2	1	26.3	3.5	150	1.66	0.76	86.3	1.4	high	8.6	4.4	0.05	34	10.6
PS	55	M	7	1	25.8	4.3	141	1.43	0.68	88.45	1.3	high	9.1	5.6	0.06	22	9.6
PS	64	M	4	1	21.6	3.8	41	0.69	1.01	152.4	2.1	high	9.8	2.7	17.3	34	8.1
PS	57	M	7	1	21	4.2	187	1.71	1.07	119.4	1.6	high	9	4.5	0.1	25	10.7
PS	57	M	15	1	22.7	4	203	1.16	0.9	105	1.6	high		7	0.38	22	7.6
PS	69	F	15	1	16.8	3.7	201	1.59	1.02	96.18	1.6	high		6.7		17	9.5
PS	64	M	8	1	18.6	3.8	176	1.8	1.1	84.13	1.5	high	9.3	4.6	0.45	32	10.8
PS	52	F	20	1	27		185	1.53	1.19	113.7	1.6	high		4.9		28	9.7
PS	62	M	9	1	22		188	1.95	0.97	67.07	1.1	high	9.1	3.9	0.37	31	10.4
PS	64	M	7	1	22.1		175	1.4	0.88	74.41	1.6	high		6.5		28	11.6
PS	39	M	4	1	20.2		105	0.98	1.16	111.9	2.1	high		5.7	0.1	17	11.4
PS	29	M	13	1	25.9		168	1.63	1.16	106.4	1.8	high	9.1	3.9		27	9.9
PS	61	F	12	1	16.4		187	1.43	0.83	76.53	1.5	high		3.2	0.1		8.8
PS	52	M	7	1	21.7		237	1.14	0.64	65.12	1.6	high		4.8		26	10.4
PS	54	M	12	1	28.8		158	1.22	1.11	112.4	1.5	high		4.5	0.17	27	8.5
PS	55	F	7	1	16.7		137	1.38	0.64	103.3	1.8	high	9.7	8.2		24	12.2
PS	55	M	24	1	19.6	3.7	107	1.59	0.96	109.8	1.6	high	9	1.9	2.7		9.4
PS	57	M	14	1	18	3.7	152	1.26	1.1	72.05	1.5	high	9.4	5.9	0.11	30	11.9
PS	67	M	12	1	20.1	4	100	1.2	1.03	113.8	1.6	high		5.8		31	10.3
PS	71	M	10	1	23.6	3.9	133	1.64	0.8	135.1	1.5	high	8.4	3.4	0.01	27	9.3

PS	32	M	10	1	19.5	4	158	1.19	0.85	124.3	1.5	high	8	3.5	0.2	24	12.8
PS	46	M	10	1	19.9	3.8	129	1.2	0.82	121.4	1.9	high		4.9		30	9.2
PS	64	M	7	1	19.6	4.2	130	1.21	1.06	146.1	1.5	high		3.5		28	10.9
PS	52	F	7	1	21.4	4.6	137	1.6	1	111.5	2.1	high	7.8	4.8	0.09	24	9.7
PS	67	M	6	1	21.5	3.9	141	1.38	0.93	130.6	1.6	high	8.7	4.7		19	10.2
PS	66	M	6	1	28.4	4	143	1.69	0.71	107	1.8	high	8.2	5.7	0.06	28	10.7
PS	64	F	5	1	19.6	3.4	156	1.3	0.96	108.4	1.6	high	10.3	4.2	1.42	37	8.8
PS	66	M	5	1	22.2	4.3	171	1.73	0.94	158.8	1.3	high	9.5	6.9		25	10.4
PS	61	F	5	1	24.8	4	122	1.91	0.94	87.96	2.1	high	9.1	4.8	0.1	30	11.5
PS	79	F	5	1	22.4	3.8	111	2.1	1.06	123.7	1.8	high	9.7	5.6	0.2	31	9.5
PS	64	M	5	1	20.7	4.3	130	1.12	0.87	134.7	1.6	high		3.4		28	11.3
PS	63	M	3	1	20.5	4	157	1.88	0.83	158.7	1.8	high	9.5	7.2	0.1	28	10
PS	57	M	3	1	21.4	4	150	1.17	0.8	99.26	1.3	high	9.4	5.4	3.18	21	8.9
PS	60	M	3	1	24.3		132	1.4	0.76	103.2	1.3	high	8.7	7.2	0.58	26	10.6
PS	48	M	2	1	20.8	3.9	178	1.48	1.08	124.2	1.8	high	8.3	4.2		8	10.9
PS	51	M	20	1	20.7	3.7		1.45	0.92	121.2	1.6	high	8.8	5.7		21	10.9
PS	58	M	33	1	18.3	3.5	149	1.54	1.13	106.2	1.4	high	8.5	4.4	0		9.8
PS	56	M	31	1	19.8	3.5		1.57	1.32	53.56	1.9	high	9.8	5.3		21	8.1
PS	58	F	27	1	23.6	4	198	1.38	0.85	115.7	1.8	high	9.2	6.5			10.5
PS	60	M	27	1	21.4	4		1.71	1.03	98.06	1.6	high	9.8	7.1		32	9.5
PS	59	M	4	1	20.5	4.3	120	1.32	0.92	91.13	1.5	high	8.5	6.2		28	10.3
PS	59	M	2	1	22.3		140	1.52	1.27	83.1	1.1	high	8.2	2.8	0.3	22	9.6
PS	52	M	8	1	25.4	3.7	151	1.26	0.54	92.61	2.1	high	9.3	6.7	0.96		12.4
PS	69	F	27	1	16.2	4.1		1.46	0.98	59.32	1.4	high	9.5	5	0.05	25	9.7
PS	79	F	4	1	21.9	4.2	118	1.52	1	123.9	1.6	high	8.4	7.2	0.08	35	13
PS	65	M	4	1	25		100	1.08	0.96	101.5	2.1	high	8.9	3.4	0.05	30	9.5
PS	59	M	2	1	24.4	4.2	100	1.69	0.78	93.01	1.6	high	8.4	4.7	0.09	27	11.9
PS	55	M	32	1	18.7	3.8	112	1.36	1.11	90.56	1.3	high	9.8	3.3	0.07	30	8.8
PS	45	M	17	1	20.3	4.6	142	1.18	1.07	101.4	1.3	high		2.4	0.2	21	10
PS	64	F	20	1	19.7	3.9	89	1.13	0.75	93.11	1.8	high	10.8	5.6			7.9
PS	47	F	5	1	29.6	4.2	176	2.21	0.71	57.27	1.5	high	7.9	4.5	0.05	16	10.3
PS	68	F	30	1	22.6	3.5	163	1.74	1.03	125.6	2.1	high		4.9	0.1		
PS	39	M	2	1	22.2	4.3	184	1.36	0.61	78.04	1.3	high	9.1	2.5	0.47	37	12.4
PS	46	M	11	1	19.8	4.1	120	1.37	1.1	133	1.6	high		4.5	0.07	33	9.9
PS	69	F	11	1	21.2	4.5	138	1.9	1.07	111.4	1.6	high		6.7		25	11.1
PS	80	M	3	1	23	4		1.12	0.79	110.6	1.1	high		6.3	0.09		10.8
PS	60	M	2	1	16.6	3.8	159	1.78	0.86	40.15	1.5	high	9.1	3.9	0.37	24	10.8
PS	71	F	6	1	19.8	3.7	259	1.34	1.04	103.9	1.6	high	9.1	6.8	0.1	21	10.6
PS	70	F	3	1	17.3	4.5	174	1.8	1.02	114.3	1.6	high	8.1	5.7	0.17	27	10.9
PS	44	M	13	1	25.4	4.1	189	1.76	0.88	109.6	2.1	high		4.4	0.05		
PS	54	F	13	1	13.1	3.7	127	1.28	1.21	83.02	1.6	high	8.2	5	0.05	28	10.2
PS	53	M	13	1	26.8	4.3	172	1.93	0.75	104.9	2.1	high		5.5	0.45		
PS	51	M	16	1	25.2	3.7	161	1.36	0.92	130.9	1.6	high	9.8	6.1	0.1	31	11
PS	58	M	11	1	25.7	3.7	122	1.23	0.73	102.8	1.1	high	10.3	7.9	0.1	30	9.6
PS	63	M	10	1	20.2	3.7	121	1.48	0.96	127.1	1.6	high	8.8	6	0.05	27	11.1
PS	72	M	10	1	21	3.7	230	1.44	0.88	104.6	1	high	8.4	9.1	0.74	27	10.8
PS	63	F	9	1	19.7	3.9	149	1.13	0.73	119.7	1.6	high	8.3	5.9	3.31	25	12.3
PS	58	M	9	1	24	3.9	179	1.1	0.91	88.45	1.5	high		5.5	0.13	59	7.6
PS	60	M	9	1	19	3.8	103	1.28	0.7	96.42	1.5	high	10.3	5.7	0.32	24	10.4
PS	68	M	8	1	18.8	4.1	184	1.35	0.78	125.5	1.6	high	9.3	7.1	0.11	32	11
PS	67	F	17	1	19	4.1	129	1.29	1.02	111	1.9	high	10.8	4.4	0.5	27	10.7
PS	28	F	8	1	27.5	4.3	130	1.52	0.78	104.1	1.5	high	10.2	6.7		31	10.5
PS	42	M	8	1	26.9	4.1		0.74	0.77	108.3	1.8	high		5.8	0.02	30	10.2
PS	55	M	7	1	22.2	3.9	101	1.22	1	119.2	1.5	high	9	2.5	4.3	29	8.9
PS	76	M	7	1	23.9	3.5	159	1.15	1.06	106.4	1.5	high		4.8	1.43	26	7.9
PS	73	M	6	1	23.1	3.4	93	1.27	0.76	93.96	1.5	high	9.9	5.3	0	40	14.7
PS	56	F	6	1	17	3.2	141	1.42	0.99	87.31	1.8	high	9.4	4.7		19	9.9
PS	56	M	5	1	17.4	4.3	202	1.6	0.77	108.8	1.9	high	9.4	5.4	0.1	28	12
PS	64	F	5	1	28.6	3.4	118	1.55	1.1	110.7	1.5	high	8.9	7.5	0.28	30	10.3

PS	69	M	5	1	18.5	4	140	1.24	0.72	105.2	1.8	high	8.8	9.1	0.3	37	9.7
PS	26	M	4	1	18.4	4.1	159	1.74	0.98	92.98	2.1	high		6	1.77		
PS	52	M	4	1	22.4	3.9	161	1.41	0.69	114.3	1.9	high	10.8	4.8	0.1	31	11.3
PS	56	F	4	1	20.1	3.9	154	1.89	0.9	97.42	1	high	9.7	4.2	0.23	29	9.7
PS	48	M	3	1	27.8	3.6	177	1.47	0.72	22.05	1.8	high	8.6	4.5		19	11.9
PS	48	F	3	1	29.5	3.4	185	1.56	0.67	98.16	1.8	high	10	4.1	2.16	29	9.7
PS	71	M	3	1	24.1	3.6	207	1.53	0.92	85.64	1.9	high	9.7	5.6	0.1	27	9.1
PS	64	F	3	1	22.8	3.4		1.16	0.69	111.7	1.5	high	11.4	0.4	2.28	16	7.4
PS	68	M	3	1	21.6	4.1	171	1.4	1.18	127.6	1.1	high	9.2	4.8	0.13	19	9.7
PS	68	M	3	1	28	4.1	142	1.8	0.65	67.62	1.9	high	9.3	3.8	0.1	30	10.2
PS	76	F	3	1	23.2	3.8	103	1.28	0.66	47.85	1.9	high	9.9	4.2	0.1	32	11.9
PS	76	F	3	1	20.4	3.8	171	1.37	0.75	64.45	1.6	high	8.6	3.7	0.1	25	11.1
PS	80	M	3	1	22.7	3.9	99	1.12	0.65	80.28	1.8	high	8	5.9	0.32	24	12.6
PS	68	F	3	1	31	3.8	147	1.34	0.64	71.66	1.3	high	9.1	4.7	0.03	24	10.5
PS	60	M	2	1	22.4	4.2	118	1.31	0.9	98.6	1.5	high	8.9	6.3		40	11.3
PS	40	M	2	1	21.1	4	118	0.98	1.02	84.19	2.1	high		3.3	0.05	29	10
PS	75	F	2	1	23.3	3.7	188	1.65	0.68	88.32	1.5	high	6.8	6.5	0.1	23	9
PS	43	F	2	1	18.9	4.3	223	2.22	1.04	80.21	1.5	high	9.3	4.2	0.13	24	10.7
PS	78	M	2	1	22.9	3.9	100	1.75	0.72	61.32	1.8	high	9.3	7.4	0.2	24	11.5
PS	78	M	2	1	18.7	3.6	143	1.47	0.85	129.5	1.9	high	7.7	5.2	0.1	39	10.8
PS	72	M	2	1	24.2	3.5	124	1.73	0.99	147.3	1.3	high	8.5	4.3	0.11	24	9.5
PS	52	M	5	1	22.7	3.8	140	1.21	1.12	88.45	1.8	high	9.9	3.4	0.3	29	10.2
PS	42	F	18	1	28.6	3.8	92	1.41	0.92	124.7	1.9	high	8.5	4.7	0.1	21	12.8
PS	44	M	33	1	22.7	4.1	160	1.59	0.82	98.57	1.9	high	9.6	8.4	0.1	27	10.9
PS	64	M	18	1	19	3.6	87	1.53	0.93	99.75	1.9	high	8.8	4.4	0.1	27	12
PS	42	F	25	1	20	3.5	121	1.11	0.58	110.7	1.5	high	9.8	4	0.09	23	9.2
PS	56	F	27	1	18.6	4	157	1.22	0.74	124.7	1.8	high	8.9	4.6	0.28	31	9.6
PS	54	F	24	1	17.7	3.9	154	1.46	1.09	108.7	1.9	high	8.8	4.1	1.63	26	11.2
PS	69	F	14	1	18.1	3.7		1.71	0.76	62.07	1.3	high	8.7	4.8	0.18	36	11.3
PS	61	F	15	1	17.5	3.9	80	1.47	0.89	120	1.4	high	9.5	2.4	6		
PS	38	M	14	1	29.5	4.2	121	1.6	1.04	107.9	1.3	high	7.9	6.7	0.4	23	9.9
PS	68	M	27	1	19.6	3.7	101	1.09	1.17	120.7	1.4	high	8.4	3.6		27	10.1
PS	62	F	14	1	26	3.3	190	1.8	0.97	102.3	1.8	high		6.3	0.05	20	10.5
PS	39	F	10	1	17.8	3.8	193	1.49	1.32	114.9	1.8	high	9.6	5.5	0.03	31	10
PS	85	F	9	1	17.3	4	123	1.18	0.87	124.5	1.6	high	8.8	6.2	0.4	19	10.3
PS	67	M	20	1	23.3	3.7	193	1.67	0.93	121.2	1.5	high	9.9	4.7	0.04	30	10.4
PS	55	M	6	1	21.7	3.6	126	1.49	0.98	109.1	1.8	high	9.9	4.8	0.1		11.6
PS	46	F	2	1	23.4	4		1.83	0.63	93.2	1.8	high	8.3	4.9	2.77	33	10.2
PS	63	F	17	1	20	3.7	168	1.43	1.05	103	1.6	high	8.5	5.8	0.09	28	10.2
PS	68	M	8	1	20	3.2	195	1.59	0.9	70.98	2.1	high		3	0.05		
PS	70	M	6	1	21.4	3.5	117	1.39	0.73	80.21	1.6	high	7.4	3.7	0.1	27	10.9
PS	83	F	5	1	19.2	3		1.42	0.6	88.39	1.5	high		3.7	0.68	28	10.5
PS	82	M	6	1	29.1	3.5	122	1.55	0.9	124.1	1.6	high	8.6	3.5	0.02	24	11.5
PS	81	F	4	1	16.6	3.6	106	1.09	1.06	59.17	1.8	high		5.6	0.05	28	10.2
PS	79	M	5	1	19.8	4.1	168	1.29	0.83	97.26	2.1	high		6.4	3.51		
PS	64	M	4	1	16.7	3.8	207	1.35	0.8	90.44	1.8	high	10.4	4.5		29	10.2
PS	62	F	33	1	18.3	3.6	178	1.37	1.05	115.7	1.8	high		4.6	0.05		10.6
PS	58	M	9	1	29.7	3.7	181	1.68	1.03	124.2	2.1	high	8.5	5.3	0.11		11
PS	73	F	2	1	14.8	2.5	90	0.54	1.04	75.48	1.6	high	8.3	4.9	0.06	25	8.3
PS	68	M	3	1	26	3.8	138	1.49	0.73	76.84	1.3	high	8.6	4.3	0.8	20	10
PS	68	F	22	1	20.4	3.6	145	1.83	0.89	85.52	1.5	high	9	5.6	1.69	29	11.7
PS	47	M	13	1	23.2	3.6	139	1.14	0.82	105	2.1	high	9.5	7.5	0.12	31	10.5
PS	84	F	6	1	17.3	3.1	163	1.76	0.84	109.5	1.3	high	9.4	5.5	0.1	26	10.2
PS	56	M	26	1	22.2	3.3	173	1.74	0.71	101	2.1	high		6.1	0.74		
PS	49	M	13	1	26.7	4.1	126	1.38	0.84	126.8	1	high	8.4	6.9	0.05		10.8
PS	69	M	9	1	22.3	3.1	114	1.76	0.82	91.93	1.8	high	8.5	4.7	0.2	34	11.5
PS	70	M	3	1	23.5		214	1.16	0.8	128.5	1.5	high	8	2.9	0.04	26	10.5
PS	85	F	3	1	23.6	3.6	191	1.67	1.02	110	1.3	high	9.8	5.5	0.33	23	10.3
PS	56	M	26	1	20.2	3.5	124	1.47	1.1	89.85	2.1	high	8.5	5	0.1	26	9.3

PS	40	M	26	1	19.4	3.9	162	1.22	0.94	58.74	2.2	high		6.3	0.2	31	11.3
PS	77	F	23	1	17.5	3.3		1.3	0.86	122.2	1.9	high	9.2	3.5		31	10.7
PS	53	M	22	1	19.7	4.3	127	1.3	0.77	102.5	1.6	high	8.2	5.4	0.1	20	10.8
PS	52	F	22	1	17.8	3.7	175	1.17	0.97	110.7	2.1	high	9.4	5.2	0.14	26	11.4
PS	69	F	21	1	22.2	3.7	148	1.25	0.74	104.5	1.8	high	10.5	5.4	0.34	25	12.1
PS	59	F	20	1	20.2	3.8	140	1.24	1.13	111.3	1.3	high	9.1	3.9	0	11	10
PS	54	M	18	1	20.5	4	147	1.77	0.7	120.8	1.5	high	8.8	4.4	5.8	26	10.5
PS	49	M	17	1	18.3	3.3	121	1.57	0.8	119.6	1.6	high		3.5			9.7
PS	78	F	16	1	20.5	3.7	112	1.22	0.99	121.6	1.9	high	9.1	6.3	0	30	10
PS	58	M	16	1	19.3	3.9	172	1.35	1.06	122.3	1.9	high	9.6	3.1	0.07	18	10.8
PS	69	F	16	1	19.5	3.7		1.29	0.93	139.2	1.8	high	9.6	6	0.15	31	10.4
PS	73	M	16	1	21.6	3.8	165	1.3	0.9	121.6	1.6	high	9.1	3.7	0.7	27	10.4
PS	58	M	16	1	22.5	3.9	161	1.26	0.98	107.6	2.1	high	8.8	5.1	0.03	33	11.1
PS	71	F	16	1	20.7	4		0.96	0.99	108.6	1.8	high	8	4.4		32	8.6
PS	60	M	15	1	17.4	3.8	20	1.35	0.97	107.7	1.5	high	9.4	5.1	1.1	1	11.3
PS	54	M	15	1	21	3.7	193	1.79	0.8	116	1.5	high	9.8	4.8	0.01	34	13
PS	72	M	14	1	21	3.8	79	1.19	0.75	112.6	1.5	high	7.1	6.5	4.29		12.2
PS	62	F	14	1	19.3	3.3	158	1.35	1.03	123.7	2.1	high	10.5	5	0.24	30	10.8
PS	44	M	14	1	20.4	4.2	176	1.11	0.87	91.86	2.2	high		6.1	0.17	24	10.5
PS	56	M	13	1	21.3	4	130	1.13	0.69	117.2	1.8	high	10.2	6.2	0.09	28	13.4
PS	47	F	13	1	18.1	4	111	1.43	1	123.7	1.8	high	9.9	6.1	0.04	25	12.5
PS	65	F	12	1	16.8	3.7	130	1.35	0.96	106.6	2.5	high		4.7	0.9	23	9.2
PS	58	M	12	1	21.8	3.7	143	0.88	0.94	117.5	1.8	high	9.3	4.1	0.7	21	11.1
PS	51	M	12	1	23.9	3.7	119	0.73	1.1	103	1.5	high	9.9	6.2	20.05	27	9.3
PS	62	F	12	1	19.3	3.3		1.4	0.89	132.4	1.5	high	8.1	3.3			12
PS	80	F	11	1	16.4	3.5	141	1.29	0.73	93.24	1.5	high	8.1	4.1	0.19	26	9.4
PS	59	F	10	1	18.6	4.1		1.64	1.26	148.7	1.8	high	8.9	4.2	4.69		11
PS	58	M	10	1	23.1	3.6	138	1.59	0.82	92.6	1.3	high		5.9	0.29	25	10
PS	58	M	10	1	23.2	3.9	270	1.32	1.02	92.76	1.5	high	9.4	7.6	0.21	30	10.8
PS	31	F	10	1	15.6	4		1.26	0.79	77.82	2.1	high	8.9	6.5	0.49	38	10.8
PS	66	M	9	1	20.3	3.8		1.03	0.94	102.2	1.6	high	8.5	4.5			9.9
PS	46	F	9	1	18.8	3.7	127	1.28	0.99	111.3	2.1	high	9	11	0.05	31	12.3
PS	36	M	9	1	35.2	4.1	173	1.27	0.98	46.62	2.1	high	9.3	4.1	0.05	31	10.8
PS	57	F	9	1	23.7	3.6	133	1.07	1.17	132.4	1.1	high	7	7.1	0.25	27	10.2
PS	67	M	9	1	24.8	3.9	164	1.26	1.08	113.3	1.3	high	7.6	4.2	0.05	36	12.5
PS	63	F	8	1	17.9	3.7	233	1.5	1.04	84.91	1.8	high	9	3.9	0.15	19	10.6
PS	54	F	8	1	20.3	4.2	134	1.44	0.99	127.1	2.1	high		5.1	0.06	28	12.4
PS	70	M	8	1	17.6	2.9		1.42	0.6	88.99	1.6	high	9.9	3.5			11.1
PS	77	M	8	1	22	3.7	129	1.71	0.72	123.6	1.8	high	7.4	8.6	0.5	28	13
PS	82	F	8	1	21.9	3.7	92	1.42	0.78	132.9	1.8	high	10.9	4.2	0.05	31	9.8
PS	68	F	8	1	19.1	3.6	151	1.69	0.66	113.6	2.5	high		8.4	0.2	21	13.6
PS	62	M	7	1	16.6	3.5	86	1.35	0.81	81.47	2.1	high	10	5.9	1.2	23	12.6
PS	46	M	7	1	22.3	3.9	135	1.39	1.17	117.7	1.5	high		3.1	0.33	27	9.5
PS	77	F	7	1	25.5	3.8		1.55	0.96	83.07	1.5	high	8.5	5.3	0.65	39	9.8
PS	57	F	6	1	21.3	3.6	130	1.27	1.03	92.6	1.1	high		3.6	0.05	31	11.1
PS	59	M	6	1	21.6	3.7		1.02	0.92	102.5	2.1	high		4.3	0.35	28	11.5
PS	61	M	6	1	19.4	3.7	117	1.51	1	117.4	1.8	high	8.2	2.9		27	12.6
PS	60	M	6	1	20.7	3.8	159	0.91	0.8	113	1.5	high	9.7	6.6		16	9.1
PS	44	M	5	1	23.3	4	117	1.62	1.44	83.97	1.4	high	8.5	5.4		36	9.3
PS	59	F	5	1	18.5	3.5	111	1.3	0.92	93.79	2.1	high	8.4	5.4	0	26	10.6
PS	56	M	5	1	21.5	4.5		1.32	0.74	76.93	1.8	high	8	4.1	0.02	22	10.8
PS	78	F	4	1	21.1	3.2	122	1.37	0.63	74.75	2.1	high	9.9	4.4	0.11	24	11.1
PS	68	M	4	1	19	3	185	1.41	0.55	100.2	1.1	high	9.5	9.2	2.39	39	10.5
PS	63	F	4	1	21.2	3.7	209	1.14	0.85	60.27	1.5	high		4.9	0.73	28	10.8
PS	61	M	4	1	24.3	3.9	189	1.67	0.81	109.6	1.8	high	10.6	6.2		24	11.2
PS	55	M	4	1	27.1	3.6	121	1.4	0.89	127	2.1	high	9.4	6.5	0.31	22	10.9
PS	61	M	4	1	27.6	3.7	194	1.47	0.88	104.1	2.1	high	8.5	7.3	0.05	23	10.6
PS	57	M	4	1	21.6	3.6		1.53	0.94	62.08	1.6	high	9.4	5.7			10
PS	32	M	3	1	16.9	4.2	143	1.04	0.82	89.47	2.1	high	9.2	6.9	0.61	32	9.6

PS	61	M	3	1	19.7	3.8		1.15	1.07	125.9	1.6	high	8	3.6			11.4
PS	76	F	3	1	22.6	3.5	123	1.02	0.6	78.39	1.6	high		7.4	0.12	26	10.4
PS	71	M	3	1	22.5	4		1.32	0.87	109.9	1.3	high	8.6	3.4			10.4
PS	47	M	3	1	22	3.8		1.31	0.86	153.2	1.6	high	8.4	4.1			12.4
PS	69	F	3	1	21.4	3.6	130	2	0.73	115.7	1.1	high	9.3	4.7	0.5		9
PS	60	F	3	1	24.1	3.7		1.54	1.02	104.4	1.3	high	8.4	3.3			10.6
PS	81	M	3	1	24.6	3.5	135	1.12	0.75	66.09	1.8	high		5.4	0.1	30	10.1
PS	66	M	3	1	20.8	3.5	132	1.6	0.76	84.63	1.5	high	8.8	5.5			8.7
PS	73	M	2	1	22.5	3.5	106	1.61	0.82	116.6	1.6	high		6	0.2	29	11.4
PS	72	F	2	1	19.1	3.8		1.38	0.88	82.96	1.3	high	9.1	3.4			10.4
PS	58	M	2	1	20.7	3.6	157	1.43	0.93	100.4	1.8	high	8.1	3.4		22	10.6
PS	50	M	12	1	22.9	4.4		1.09	1.04	102.9	1.5	high	10.1	2.9	0.47	18	10
PS	62	M	9	1	28	4.3	125	1.66	0.97	95.88	2.1	high	9.8	5.6			
PS	44	F	10	1	14	3.7	162	1.49	1.04	53.56	2.5	high	9.6	7.7	0.1	30	10.2
PS	81	F	5	1	18.2	3.8	127	1.23	0.77	99.22	1.8	high	8.5	6.4	0.4	26	10.8
PS	67	F	21	1	21		130	1.23	1.21	103	1.5	high		6	0.1	37	10.6
PS	74	F	4	1	22.1	3.3	93	1.29	1.03	106.2	2.1	high	7.8	5.3	0.02	36	11
PS	61	M	10	1	17.5	3.4	91	1.46	1.27	130.6	1.6	high		5.4	0.2	26	10.6
PS	71	F	4	1	22.7	3.6	147	1.54	0.76	100.9	1.6	high		5.3	0.2	28	10.5
PS	70	F	22	1	15.4	3.1	153	1.35	0.92	115.8	1.6	high	9.8	4.4	0.12	15	8.3
PS	70	M	8	1	15.1	3.5	127	1.19	1	106.5	2.1	high	9.3	4.6			
PS	56	M	3	1	19.2	3.9	216	2.21	0.95	59.34	2.1	high	9.5	5.8	0.04		10.3
PS	60	F	16	1	17.5	3	189	1.48	0.75	3.12	1.8	high	9	5.8		30	10.8
PS	71	M	4	1	18.6	3.5	176	1.77	0.79	119.9	1.6	high	8.9	5.2	0.01	29	9.1
PS	74	M	11	1	23	3.9	176	1.56	0.72	110.5	1.3	high	9	1.9	0.26	29	11.1
PS	62	M	21	1	24.3	3.9	214	2	0.8	144.6	1.5	high	9.4	5.7	0.06	29	10.6
PS	76	M	2	1	25.1	3.9	132	1.33	0.79	69.48	1.6	high	8.5	4.4	0.1	17	10.6
PS	69	F	8	1	18.5	3.9	147	1.58	0.86	93.16	2.1	high	9	4.5	0.14	23	11.1
PS	71	M	7	1	23.6	3.6	168	1.6	0.9	127.1	1.8	high	8.3	4.8	0.6	31	12.7
PS	76	F	4	1	23.6	4.2	151	0.63	0.64	77.44	1	high	10	6.1	0.32	19	9.3
PS	69	M	9	1	22.3	3.9	217	1.69	0.71	106.5	1.5	high	9.9	5.6	0.05		10.1
PS	58	F	7	1	20.3	3.9	222	1.9	0.84	106	1.5	high	10.4	3.2	1.24	24	10.7
PS	74	F	3	1	19.2	3.6	129	1.75	1.31	114.3	1.5	high	9.6	3.6	0.1	36	10.2
PS	58	F	16	1	24.2	3.7	154	1.78	1	120.6	1.8	high	9.7	5.1	0.1	22	9.1
PS	81	F	6	1	19.7	3.8	107	1.36	1.51	108.4	1.5	high	9.1	5.4	0.11	33	9.4
PS	64	F	18	1	20.8	3.5	140	1.71	0.94	128.7	2.5	high	10.3	6.4		45	9.6
PS	71	M	14	1	18.9	4.1	149	1.01	0.86	132.5	2.1	high		5.6	0.1	27	9.7
PS	69	M	5	1	24	3.6	112	0.87	1.16	114.2	1.8	high		7	0.05	25	10.2
PS	58	M	6	1	21.6	4	206	1.78	0.92	87.57	2.1	high		3.4	0.08		13.5
PS	78	M	5	1	19.8	3.6	126	1.32	0.62	114.6	1.3	high	8.9	4.4	0.05	27	10.8
PS	69	F	7	1	29.8	3.8	173	1.57	0.89	104.1	1.8	high	8.6	6.6	0.1	12	10.6
PS	74	M	24	1	25.2	3.6	141	1.28	0.91	94.14	2.2	high	9.3	8	0.8		11
PS	67	M	7	1	19.3	3.1	204	1.57	0.47	76.48	1.8	high	10.2	4.9	0.1	19	9.9
PS	39	F	18	1	19.9	4.1	147	1.41	1.14	119.8	1.4	high	8.9	4.4	0.08	31	10.4
PS	59	M	32	1	17.7	4	133	1.76	1.21	106.5	2.1	high	9.3	5.4	0.09	31	12
PS	68	M	16	1	21.5	3.3	167	1.23	0.84	100.5	2.1	high	9.8	6.1	0	31	10.7
PS	62	F	14	1	15.4	3.4	147	1.64	1.13	59.18	1.6	high		6.1	0.4	27	13.3
PS	56	F	4	1	25.2	3.7	139	1.56	1.12	106.5	1.5	high	9.7	8	0.04		9.2
PS	51	M	20	1	18.6		156	1.44	1.16	117	2.1	high	9.4	4.8	0.05	18	11.9
PS	50	M	3	1	33.6	4	161	1.49	0.68	93.88	2.1	high	9.3	7.4	0.09	16	10.2
PS	51	F	2	1	21.7			1.3	0.82	45.81	1.6	high	9.5	4.5	0.05	41	10.8
PS	86	M	4	1	21.9	3.7		1.28	1.19	88.23	2.1	high	8.7	4.6	0.85	23	13.5
PS	62	M	14	1	18.5	3.6	172	2.18	0.92	106.9	1.8	high	8.9	2.4	0.27	25	11.3
PS	57	F	11	1	19.3	3.8	166	1.89	0.42	132.6	1.5	high	8.3	4.8	0.05	29	10.7
PS	71	M	11	1	16.9	3.5	79	1.29	1.1	114.5	2.1	high	7.9	5.3	0.13	36	9.4
PS	76	M	9	1	23.3	3.8	151	1.06	0.88	115.5	1.8	high	9.3	3.8	0.05	19	10.6
PS	39	M	8	1	19.6	4.1	156	2.18	1.07	115.9	1.3	high	8.4	4.9	0.04	26	10.9
PS	72	M	4	1	25.2	3.5		1.33	0.81	94.59	1.8	high	8.9	3.5	0.02	23	11.1
PS	65	M	2	1	21.5	3.8	162	1.19	0.8	69.61	1.6	high		5.3	0.02	23	10.3

PS	45	M	2	1	22.7	3.9	139	1.1	0.74	86.03	2.1	high		3.4	0.05	33	9.2
PS	67	M	22	1	16.1	4.1	126	1.38	0.97	96.43	1.6	high		3.6	1.8	29	8.6
PS	53	M	21	1	17.4	3.7	101	1.98	1.05	131.9	1.3	high		4	0.1	30	13.2
PS	60	F	10	1	24.1	4.1	158	1.84	0.73	117.8	1	high	9	5	4.7		9.9
PS	66	F	8	1	18.7	3.8		1.23	0.93	54.42	1.6	high	10.2	5.1	0.02	26	10.3
PS	64	M	6	1	18.6	4.1	88	1.65	1	117.4	1.5	high	9	7.4	0.23	30	10.9
PS	66	M	2	1	32	4	148	1.29	0.89	122.4	1.5	high	9.1	7.4	0.02	22	11.3
PS	73	F	2	1	26.3	3.5	127	1.33	0.82	96.84	1.5	high	9.2	5.4	2.5	32	8.7
PS	55	M	2	1	21.7	3.9	172	1.85	0.73	78.85	1.3	high	8.9	4.2	0.27	17	10.8
PS	50	M	18	1	25.9	3.8	121	1.36	0.64	152.2	2.1	high	10.1	4.3	0.05	29	11.6
PS	50	F	3	1	15.7	4.3	173	1.25	1.28	96.1	2.5	high	8.6	5.1	0.03	21	10.7
PS	71	M	24	1	19.2	4.2	144	1.54	1.2	132.8	1.4	high	10.1	2.8		27	9.7
PS	86	M	4	1	21.8	3.3	147	0.83	0.8	94.69	1.5	high	10.2	4.9	10	46	10.4
PS	70	M	11	1	22.1	3.7	151	1.29	1.02	95.35	1.3	high	8.7	5.7	0.2	45	8.8
PS	65	M	6	1	20.8	3.9	126	1.2	0.99	119.8	2.2	high	9	7	0.4	23	10.3
PS	74	F	5	1	17.3	3.5	148	1.45	0.89	98.12	1.3	high	8.6	3.4	0.2		8.6
PS	59	M	4	1	19.7	4.2	168	1.31	0.98	92.09	1.3	high		5.2	3.22	25	10.9
PS	57	M	19	1	18.5	3.8		1.35	1.04	80.08	1.6	high		5.9	0	21	7.9
PS	62	M	12	1	26.3	3.9	178	1.61	0.88	120.8	1.8	high	10.2	5.3	0	14	11.6
PS	67	M	19	1	26.6	3.8	101	1.25	0.9	115.3	1.6	high		6.9	0	35	14.1
PS	73	F	7	1	18	3.5	169	1.22	0.86	122.1	1.4	high	8.9	6.8	0.05	21	10.9
PS	75	M	6	1	24.4	3.9	123	1.29	0.81	115	1.5	high	8.8	3.8		28	8.9
PS	67	M	33	1	20.8	3.7	143	1.2	1.03	92.63	1.6	high	8.1	5.9	0.08	25	9.9
PS	37	F	8	1	25.1	3.9	210	1.06	0.85	78.95	2.1	high	8	3.7	3		9.9
PS	66	M	7	1	22.6	3.7	136	1.76	0.99	135.2	1.5	high	9.5	4.5	0.07	33	9.7
PS	64	M	5	1	26.2	4.3	146	1.5	1.17	103.7	2.1	high	8.7	3.6	0.5	22	12.2
PS	60	M	8	1	21.6	3.6	206	1.31	1.01	94.23	1	high	10.2	6.1	0.26	27	10.6
PS	59	M	15	1	19.4	3.8		1.14	1.13	137.4	1	high	10	4.4	1.7		8.8
PS	46	M	8	1	21.8	4.3	85	1.16	1.22	120.8	1.8	high	9.4	6.1	0.76	30	8.3
PS	84	M	3	1	24.3	4	177	2.06	1.19	117	1.6	high	9.1	2.3		26	12.1
PS	46	M	4	1	25	4.1	165	1.32	0.88	112.7	1.4	high	8.5	5.7	0.41	19	10.1
PS	65	F	4	1	20.5	4	126	1.24	1.09	79.42	2.1	high	8.5	3.1	0.06	25	10.7
PS	68	F	2	1	25.4	4	157	1.97	0.67	81.75	2.1	high	9.9	4.5	0.2	25	10.9
PS	71	M	6	1	23.7	4.4	155	1.02	0.73	153.3	1.8	high	9.1	3.6	0.94	28	6.1
PS	67	M	8	1	23	3.9	134	1.55	0.77	111.5	2.1	high	9.6	5.8	0.03		10.2
PS	47	M	4	1	16.5	4	169	1.32	1.04	94.76	1.5	high	8.4	4	0.5	29	10.4
PS	71	F	6	1	16.5	3.8	158	1.29	0.98	115.2	1	high	8.3	3.6	0.1	32	9.9
PS	75	M	2	1	29	4.1	132	1.55	0.89	106.9	2.1	high	9	5.4	0.26	27	10
PS	60	M	33	1	22	3.5	163	1.27	0.76	90.05	0.6	high	8.9	7.9	0.3	27	9.4
PS	77	M	20	1	19.4	4.1	124	1.66	1.12	117.4	2.1	high	9.2	6.3		29	11.8
PS	62	F	10	1	22.9	3.3	130	1.46	1.09	99.42	1.6	high	8.6	5.9		22	11.3
PS	62	M	7	1	21.1	3.9	122	1.38	0.89	139.8	1.8	high	8.7	7.1		25	10.3
PS	59	M	2	1	24	4.3	150	1.51	0.71	75.94	1.6	high	8.9	5.4	0.4	22	10.9
PS	50	M	9	1	20.8	4	165	1.69	0.97	111.9	1.8	high		5.1			8.4
PS	57	M	25	1	20.4	3.7	223	1.56	0.7	109.5	1.4	high	8.8	5.8		32	11.1
PS	59	F	8	1	19.6	3.5	100	1.97	1.08	78.65	1.5	high	7.6	3.2	1.3	40	7.5
PS	59	F	2	1	20.4	4		1.63	0.81	104.4	1.3	high		4.1	0.3	25	9.6
PS	55	M	7	1	21.8	3.9	192	1.79	0.81	125.3	2	high	9	3.3	0.06	31	9.7
PS	47	F	4	1	26.2	3.9	115	1.1	1.08	67.1	1.3	high	10	8.9	2.4	33	10.2
PS	75	F	16	1	18.1	4	189	2	0.89	144.7	1	high	8.5	3.5	0.5	26	8.8
PS	52	M	4	1	29.5	3.7	131	1.06	1.19	115.2	1.5	high		3.5	0.63	22	10.5
PS	54	M	25	1	20.7	3.8	229	1.52	1.14	113.6	1.6	high	8.8	5.5	0.4	24	9.7
PS	58	F	17	1	20.1	3.5	145	1.66	1.04	109.9	1.6	high	9	5.1	0.1	17	11.3
PS	43	M	16	1	23.7	3.6	122	1.12	0.94	124.2	2.1	high	8.8	6.1	0.05	26	10.6
PS	75	F	14	1	18.8	3.4	194	1.75	1.13	109.8	1.6	high	9.8	5.8		31	9.8
PS	56	M	10	1	26	4		1.16	0.98	112.6	2.1	high	8.4	2.9			8.5
PS	57	M	8	1	23.6	3.8	127	1.5	1	129.9	2.1	high	8.2	5.2	0.3	29	9.1
PS	59	F	7	1	21.3	3.7	165	1.53	0.62	77.79	1.3	high		6.6	0.17	33	10
PS	73	F	7	1	25.7	3.5	127	1.28	0.87	96.1	1.2	high	8.5	4.8	1.2	27	9.8

PS	32	F	5	1	17	4.1	125	1.28	0.8	35.07	1.5	high	8.4	3.7	0.03	24	11.6
PS	56	M	4	1	18.1	3.6	151	1.61	1	99.97	1.6	high	8.3	3.8	0.1	23	10.4
PS	60	F	4	1	18.9	3.5		0.92	1.09	104.3	1	high		6.9	0.65	29	10
PS	29	M	2	1	20.9	4.2		1.28	1.01	104.5	1.3	high		14.8	0.2	18	10.6
PS	68	F	2	1	20.5	3.9	81		1.08	111.4	1.6	high	7.6	4.8	1.05	23	10.3
PS	60	M	16	1	19.3	3.7	159	1.65	0.93	117.3	2.1	high	8.1	5.6	0.39	25	8.4
PS	70	F	8	1	23.2	3.9	229	1.31	0.88	93.72	1.5	high	9.3	5.7	0.3	21	10.7
PS	47	M	7	1	20.6	3.3	157	1.69	0.9	107.7	1.1	high	10.9	3.6		35	9.8
PS	71	M	18	1	17.1	3.5		1.48	1	107.2	2.1	high	8.1	1.6	9.37	23	10.2
PS	76	F	3	1	21.5	3.4	161	1.36	0.9	107	1.8	high	9.5	4.7	1.13	41	10.7
PS	56	M	30	1	20.7	3.6	108	0.95	0.99	122.5	1.9	high		6.5	0.1	9	7.8
PS	52	M	14	1	17.7	3.9	222	1.87	0.82	101.4	1.6	high	9.5	4.8	0.05	26	10.9
PS	75	M	7	1	21.6	3.9	175	1.17	0.63	80.59	1.5	high	9.7	4.1	0.13	26	10.6
PS	56	M	7	1	20.6	3.9		1.69	0.87	114.7	1.5	high	8.4	4.9		20	8.2
PS	62	M	20	1	20.6	3.4		1.45	0.78	107.3	1.3	high		2.9	0.26	35	8.3
PS	50	M	4	1	24.4	4		1.39	1.13	107.6	1.5	high	9.9	4.3	2.3	28	7
PS	66	M	2	1	20.6	3.7	71	0.68	0.88	77.77	2.1	high	8.3	3.4	4.88	25	7.4
PS	56	M	35	1	14.5	3.5	139	1.22	0.85	67.11	1.9	high	8.7	4.2	0.12	27	11
PS	52	F	12	1	25.5	4.1	87	0.5	0.8	103.7	1.8	high	7.4	1.4			11.1
PS	42	F	20	1	19.2	3.5	161	1.39	0.64	90.12	1.8	high	10	3.9	1.14		9.6
PS	88	M	3	1	21.1	3.5	112	1.74	0.86	110.6	2.1	high	8.7	4	0.26	29	7.6
PS	60	F	23	1	19	3.9	134	1.21	1.01	108.6	2.1	high		6.2	0.05		
PS	60	M	12	1	17.3	4.1	160	1.5	0.79	106.4	1.5	high	8.8	4.5	0.08	26	10.1
PS	58	M	13	1	16.8	3.7	160	1.28	0.96	125.5	1.6	high	8.7	4	0.05	28	11.1
PS	58	F	12	1	19.6	3.9	130	1.48	0.74	123.7	1.3	high	9.8	5.7	0.45	25	10.3
PS	58	F	8	1	22.6	3.7	199	1.44	0.91	133.2	1.9	high	9.4	4.2	0.07	25	9.7
PS	63	F	8	1	34.4	4	110	1.17	0.74	60.9	2.1	high	9.9	6.8	0.11	28	11.3
PS	58	F	7	1	20.2	3.9	183	1.42	1.02	62.3	1.8	high	10.1	6.7		22	9.8
PS	61	M	6	1	23.5	3.5	147	1.55	0.89	70.37	2.1	high	10.8	6.5	0.71		8.1
PS	56	F	6	1	18.9	3.7	168	1.43	0.82	96.46	1.5	high	8.8	6.4		23	10.7
PS	73	F	24	1	18.2	3.7	113	1.75	0.76	95.7	1.8	high	8.4	3.9	1.3	28	9.5
PS	60	M	23	1	19.6	3.5	131	1.51	0.88	107	1.5	high	8.1	5.4	0.23	31	10.8
PS	43	F	22	1	23.8	3.7	150	1.7	1.05	121.7	1.9	high	8.8	7	0.05	30	10.6
PS	60	F	20	1	17.9	3.5	175	1.7	0.9	141.4	1.5	high	9.5	6.2	0.05		10
PS	71	M	20	1	28	3.6	200	0.6	0.66	128	2.1	high	10.2	5.4	0.26	29	10.9
PS	58	M	7	1	23.9	3.6	159	1.77	0.95	111.2	1.3	high	7.9	2.9	0.2	42	10.3
PS	57	M	6	1	20.1	4	158	1.07	1	119.3	2.5	high	10.5	9.3	0.2	25	11.9
PS	61	M	2	1	23.4	3.8	175	1.62	0.8	63.17	1.1	high	10	2.7	0.25	33	9.9
PS	53	M	2	1	21.7	3.7	127	1.26	0.93	127.4	2.1	high		9.6			10.2
PS	69	M	8	1	21.8	3.4	178	1.88	0.97	45.21	1.3	high	9.2	4.1	0.33	28	10.9
PS	36	M	20	1	22.1	3.6	105	1.19	1.05	123.6	1.6	high	8.7	4.5	0.05	22	9.8
PS	60	M	9	1	22.8	3.8	166	1.68	0.97	135.8	1.8	high	8.9	6.5	0.05	22	10.4
PS	77	F	8	1	18.9	3.9	149	1.18	1	116.6	2.5	high	9.1	6.8	0.11	19	9.3
PS	68	M	8	1	24.1	3.9	187	1.09	1	95.93	1.8	high	10.7	6.7	0.07	26	13
PS	63	F	6	1	24.7	3.6	204	1.15	0.97	116.6	2.1	high		5.6			9.7
PS	67	M	3	1	18.4	3.6	108	1.45	1.17	78.1	1.6	high	10	3.4	0.05	23	10
PS	61	M	2	1	19	3.7	112	1.2	0.87	85.86	1.9	high	10	6.6	0.11	32	11.3
PS	66	M	15	1	17.1	3.5		1.32	0.87	102.3	1.5	high	8.9	6.7	1.3	35	9.8
PS	71	M	3	1	22.9	4	122	1.65	0.69	110.6	1.5	high		4.6		27	10.6
PS	64	M	3	1	20.9	3.9	128	1.46	1.16	106.4	1.8	high	8.2	4.5			10.6
PS	58	F	34	1	22.8	3.7	167	1.74	0.9	100.5	1.5	high	9.8	5.2	0.53	32	11.4
PS	78	M	12	1	19	3.6	139	1.69	0.65	138.9	1.3	high	10	6.3	0.03	21	10.5
PS	54	M	7	1	22.4	4	128	1.29	0.7	85.28	1.8	high	9	6.6	0.17		12.9
PS	66	M	6	1	24.5	3.7	142	1.37	0.9	113.9	1.5	high	8.4	4.1	0.12	20	10.7
PS	20	M	10	1	17.5	4.2		1.22	0.83	84.83	1.3	high		4.4	0.12	40	15.4
PS	60	F	22	1	25.3	4.1	265	1.29	1.08	152	1.3	high		5.7	0.18		10.3
PS	59	M	15	1	21.5	3.6		1.35	0.72	125.3	1.3	high	8.5	6	24.55		6.6
PS	71	F	3	1	27.3	3.6	142	2.39	0.79	77.55	1.3	high	9	5.2	0.01	36	9.1
PS	48	M	2	1	23.5	4.3	144	1.83	0.83	107.9	2.1	high	8.7	3.1	0.15	17	10.5

PS	91	M	7	1	19.5	3.2	223	1.21	0.92	77.43	1.5	high	9.4	4.5		33	8.9
PS	49	F	30	1	17.3	3.7	109	1.43	0.88	87.22	1.5	high	9.1	4.6	0.08	44	9.9
PS	46	M	7	1	17.6	3.7	171	1.2	0.72	84.06	1.8	high		7.3	0.19	24	10.8
PS	72	M	13	1	22.3	4.1	128	1.38	0.86	128.8	1.8	high	9.3	8	0.31	26	11.9
PS	74	M	7	1	17.7	3.6	110	1.79	0.81	91.57	1.5	high	8.4	2.4	0	21	8.9
PS	59	M	30	1	17.1	4.2	162	1.4	1.18	108.4	1.8	high	9.6	6.1	0.2	25	10.9
PS	80	F	7	1	15.8	4	167	1.2	0.94	133.2	1.4	high		5.5	0.08	17	4.8
PS	78	F	6	1	18.4	3.7	181	1.35	0.82	124.4	1.4	high	10.9	5.2	0		
PS	62	M	6	1	21.5	3.2	152	1.42	0.59	93.43	2.1	high	9.7	6.3	0	25	9.4
PS	59	M	5	1	21.9	3.9	93	1.13	0.79	64.86	1.8	high	8.9	4.7	0	38	10.6
PS	47	M	4	1	22.6	3.8	115	2.27	0.76	83.06	1.3	high	10.4	2.6	0.63	29	11.3
PS	81	F	3	1	20.1	4	104	1.1	0.84	109.8	1.4	high	9	3.9	0.3	33	10.7
PS	61	M	2	1	29.6	4	174	1.69	0.76	68.63	1.3	high	9.6	6	0	30	11.1
PS	76	M	2	1	17.7	3.5	117	1.17	0.91	107.2	1.4	high	10.9	5.6	0.2		
PS	65	F	2	1	17.6	3.9	146	1.21	0.65	63.14	1.8	high	8.9	6.3	0.04	20	9.7
PS	52	F	2	1	18.1	4.2	112	1.44	0.96	108.5	1.8	high		5.4	0.14		11.2
PS	46	M	6	1	21.9	3.9	132	1.49	0.65	88.95	2.1	high	9.7	6.1		26	11.2
PS	49	F	18	1	20.2	4	209	1.77	0.88	98.95	1.5	high	8.5	6.1	0.45	23	9.7
PS	61	M	14	1	20.3	3.7		1.28	0.71	72.94	1.5	high		6.1	0.05		11.4
PS	61	M	3	1	21.3	4	160	1.4	1.03	104.4	2.1	high	8.8	5.6	0.6	15	12.5
PS	61	M	36	1	20.7	3.5	162	1.78	0.77	54.9	1.3	high	9.9	3.9	0.04	49	10.5
PS	62	M	11	1	23.9	3.8	136	1.53	0.51	94.77	2.1	high	7.6	3.2	0.31	25	11.5
PS	79	F	10	1	17.5	4.1	175	1.4	0.92	130.4	1.6	high	9	4.4	0.07	25	10.8
PS	59	M	10	1	22.5	3.3	245	1.37	1.32	90.1	1.8	high	9.7	6.9	0.94	31	9.8
PS	55	F	9	1	17.7	3.5	213	1.67	0.72	81.92	1.3	high		5.9		30	10
PS	77	F	7	1	21.3	3.5	138	1.36	0.75	96.05	1.6	high	8.1	5.5	0.08	31	11.1
PS	77	F	7	1	23.3	3.7	137	1.2	0.78	135.4	1.6	high	9.2	3.6	0.2	31	9.4
PS	59	M	5	1	20	3.9	177	1.69	0.97	98.59	1.6	high		5.1	0.2	35	10.6
PS	81	F	2	1	17.6	3.4	188	1.18	0.6	59.54	2.1	high		4.5	0.26	20	10
PS	80	M	2	1	21.3	3.4	183	1.55	0.69	54.77	1.5	high	8.2	5.2	0.1	27	9.3
PS	82	F	14	1	20.2	3.5	81	1.33	0.65	98.9	1.6	high	8.7	5.3	0.05	69	11.7
PS	66	M	13	1	16.5	3.6	175	1.32	0.86	114.2	1.6	high	7.8	4.5		25	8.3
PS	75	M	6	1	17.7	3.9	161	1.5	0.96	99.34	1.8	high	9.5	7.4	0.02	31	10.4
PS	50	M	9	1	18.2	4	141	1.85	0.85	75.08	1.5	high	8.8	8.5	0.93	32	9.9
PS	83	F	4	1	22.7	3.6	234	1.67	1.04	103.1	1.1	high	9.4	5.5	0.6	26	9.9
PS	74	F	4	1	20.6	3.8		0.95	0.86	106	1.6	high	8.9	4.3	0.1	19	10.3
PS	69	F	4	1	22.9	3.6		1.91	0.98	78.37	1.3	high		3.6	1.55	32	8.8
PS	60	F	4	1	22.8	3.5	130	1.83	0.8	112.6	1.5	high		3.2	0.15	29	9.8
PS	68	M	2	1	21.7	3.8	66	0.96	1.14	98.83	2.1	high	8.8	6.1	0.24	26	10.7
PS	75	M	21	1	19.4	3.9	211	1.79	0.86	117	1.5	high	9.8	4.5	0.05		9.3
PS	59	F	20	1	20.6	3.9	109	1.48	1.03	105.5	1.5	high	9.8	5.9	8.61	31	8.1
PS	65	F	17	1	23.7	3.8	123	1.35	1.04	129.6	1.6	high	8.6	5.8	0.1	25	10.2
PS	64	F	17	1	20	3.6	147	1.28	1.03	152.5	1.5	high	9.7	3.5	0.12		10.5
PS	65	M	17	1	20.1	3.3	145	1.89	1.07	83.63	1.6	high	11.4	7.1	0.02	24	10.6
PS	59	M	15	1	17.5	4.1	127	1.5	1.06	109.6	1.5	high	9.8	5.9	0.02	28	12.2
PS	58	M	15	1	19.2	4	163	1.15	1.03	127.3	1.9	high	8.8	7.9	1.06	28	10.2
PS	45	F	15	1	26.1	4.2	129	1.43	0.95	90.06	2.1	high	10.8	5.1	0.3	26	10.2
PS	63	M	15	1	23.9	3.9	129	1.15	0.96	120.2	1.6	high	9.5	7.1	0.02	31	11.6
PS	55	F	14	1	26.4	3.8	149	1.46	0.91	79.37	1.3	high	9.2	3.4	0.25		10.5
PS	72	M	14	1	18	3.5	142	1.58	0.65	86.95	1.6	high	9.9	6.6	0.02	24	8.8
PS	63	M	13	1	17.3	4	193	1.2	0.98	68.01	1	high		6.7		31	9.3
PS	56	M	12	1	20.4	4.2	148	1.18	0.81	128.3	2.1	high	9	6.1	0.05		11.8
PS	73	M	12	1	19	3.5	133	1.25	1.04	84.81	1.5	high	8.8	8.8	2.23	26	12.7
PS	63	M	12	1	20.3	3.6	131	1.1	0.62	114.9	1.9	high	9.3	6.2	0.03	23	10.9
PS	61	F	11	1	19.2	3.6	104	1.19	1.16	124.2	1.8	high	9	4.8	0.05	48	12.1
PS	62	M	11	1	20.4	3.8	122	1.98	0.93	57.23	2.1	high	9.3	4.9	5.07	35	8.9
PS	73	M	11	1	24	3.5	161	1.26	0.8	121.9	2.1	high	9.6	4	0.07		9.9
PS	54	M	11	1	27.8	3.6	109	0.95	0.79	75.02	1.3	high	8.6	4.3	0.04	24	10.5
PS	72	M	10	1	15	3.9	120	1.16	0.88	80.49	1.6	high	10.4	8	0.39	35	10.6

PS	50	M	10	1	30.7	4.1	135	1.24	0.67	82.92	2.1	high	7.8	7	3.7	24	11.7
PS	60	F	10	1	24.7	3.9	130	1.06	1.06	127.3	1.9	high	9.3	9.1	0.17	24	10.8
PS	66	M	10	1	22	4		1.71	1.12	132.4	1.5	high	9.4	7.1	0.5	26	10.5
PS	61	M	9	1	17.7	4	140	1.26	0.95	121.4	2.1	high	8.7	3.8	0.1	22	10.4
PS	74	F	9	1	13.9	3.2	169	1.07	0.43	94.17	1.6	high	9.3	3.6	0.01	22	11.7
PS	74	F	9	1	20.3	3.5	142	0.98	0.8	116.4	1.6	high	9.3	6.6	0.09	25	10.7
PS	58	M	9	1	28.3	3.9	125	1.52	0.59	96.74	2.1	high	8	5.2	0.61	23	10.7
PS	71	M	9	1	23.8	3.8		1.63	0.69	99.28	1.5	high	9.7	4.4		38	9.6
PS	55	M	9	1	24.2	4	179	1.06	0.9	121.8	1.8	high	10	8.7	0.08	26	11.9
PS	60	F	9	1	25	3.8	167	1.65	0.95	110.3	1.8	high	8.8	6.1	1.37	28	11
PS	73	F	9	1	21.4	3.8	107	1.17	1.14	102	1.9	high	8.4	5.6	0.45	38	11.3
PS	73	F	8	1	20.9	3.5		1.68	0.76	99.68	1.8	high	7.5	3.7	0.81	29	10.2
PS	62	M	8	1	20.8	4.2	180	1.02	0.72	121.7	1.3	high		5.1	4.6	26	6.8
PS	70	F	8	1	20.6	3.6		1.44	0.76	116.7	1.1	high	8.1	5.3			9.5
PS	77	M	8	1	23.7	2.8	140	1.25	0.51	77.16	1.3	high		3.1	0.03	30	10.8
PS	81	F	8	1	20.2	3.7	128	1.49	0.91	143.3	2.1	high		7.5	0.24	27	14.8
PS	78	F	8	1	22	3.6	108	1.16	0.82	129.1	1.8	high	9	6.6	3.75	42	8
PS	62	M	7	1	23.4	3.6	149	1.15	0.68	109.7	1.9	high	8.9	4	0.02	16	10.3
PS	40	M	7	1	24.8	3.8	166	1.42	0.84	133.8	1.6	high	9.7	5.5	0.07		7.6
PS	52	M	7	1	24.8	3.8	126	1.39	0.91	108.2	1.8	high	9.4	4.8		26	9.8
PS	44	M	7	1	20.2	4	135	1.46	0.9	88.97	1.8	high	10.2	5	0.06	24	11.2
PS	66	M	6	1	24.2	4.2	137	1.5	0.86	127.5	1.6	high	8.7	5	1.1	43	8.1
PS	91	F	6	1	16	3.4	138	0.86	0.85	123.2	1.5	high		5.1	0.2	37	11.5
PS	66	M	6	1	22.1	3.6	124	1.52	1	107.5	1.8	high		3.4	0.56		9.8
PS	75	M	5	1	25.4	3.4	171	1.9	0.56	99.49	1.8	high	8.4	3.7	1.61		9.1
PS	56	F	4	1	19.6	3.9	154	1.38	0.85	91.13	1.5	high	9.5	4.4	0.86	30	11.4
PS	50	M	4	1	26.3	3.9	136	1.45	0.7	107.6	1.5	high	9.2	4.1		25	8.8
PS	63	M	4	1	22.2	3.9		1.12	0.91	107.8	2.1	high	8.3	3.6	2.41	34	10.3
PS	75	M	4	1	18	3.5	154	1.2	0.68	98.55	1.6	high		3.6	8.71		9.7
PS	75	F	4	1	20.7	4.3	159	1.12	1.05	110.7	1.6	high		7.3	0.71	23	7.3
PS	61	M	3	1	21.9	3.6		1.33	0.82	94.01	1.8	high		4.6			8.1
PS	77	M	3	1	20.8	3.8	131	1.59	0.61	97.95	1	high	9	5.6		25	12.3
PS	72	M	3	1	22.9	3.9	116	1.5	0.85	89.11	1.8	high		5.2	0.1	31	11.9
PS	61	M	3	1	21	4.1	142	1.16	0.78	112	1.8	high	8.6	4.3	0.27	19	11.1
PS	78	M	3	1	18.7	3.8	133	1.61	0.98	77.29	1.6	high	8.5	4.7	0.07	24	10.8
PS	53	M	3	1	20.3	4	162	1.21	1.06	66.3	1.8	high	8.9	2.6			8.6
PS	57	M	3	1	27.6	3.8	116	1.29	0.61	89.64	1.5	high	10.7	6.1	0.02	27	8.4
PS	64	F	3	1	25.6	3.6	167	1.18	1.05	104.6	2.1	high	9	5.4	5.91		10.1
PS	71	M	2	1	22.1	3.8	151	1.83	0.86	74.98	2.1	high	10.1	5.6	0.04	30	10.9
PS	63	M	2	1	24.1	3.7	162	1.25	0.97	128.7	1.6	high	7.8	4.7		27	9.7
PS	62	F	5	1	17.9	3.3	222	1.7	0.66	155.9	1.9	high	8.9	4.9	0.06	35	9.9
PS	58	M	5	1	20.3	3.1	145	1.47	0.47	80.08	1.8	high		5.2	0.12	30	12.3
PS	64	F	2	1	17.8	3.8	156	1.1	0.84	82.68	1.6	high	9	5.4	0.47		12.5
PS	75	F	23	1	16.6	3.7	138	1.23	0.83	66.21	2.1	high	10.2	5.4	0.05	31	11.5
PS	53	M	13	1	22.4	4.7	102	1.54	1.11	120.8	1.5	high	8.4	4.7			11.2
PS	58	M	10	1	23.6	4.1		1.29	0.81	86.1	1.6	high	8.4	5	0.74	34	10.3
PS	49	F	5	1	18	4.3	144	1.54	0.92	82.51	1.6	high	8.6	7.9	0.29	29	14.3
PS	76	M	4	1	30.8	3.8	108	1.49	0.49	26.87	2.1	high	9.7	7.3	0.07	29	14.4
PS	69	F	3	1	24.1	3.4	149	1.44	0.84		2.1	high	8.5	6.8	0.3	29	12.1
PS	35	M	15	1	21.8	4.5	98	1.17	0.72	89.2	2.1	high	8.7	9.5	0.67	13	10.5
PS	68	F	14	1	24.7	3.9	103	1.85	0.87	100.7	1.8	high	8.4	4.2	0.05	33	10.5
PS	57	M	7	1	19.8	4.3	137	1.39	0.87	104.6	1.8	high	10.2	6.7	0.06		9.7
PS	55	F	29	1	16.9	3.7	136	1.58	0.8	72.89	1.5	high	9.2	5.8	0.19	31	10.1
PS	74	M	15	1	20.3	3.6	181	1.49	0.63	69.96	1.3	high	9	5	0.99	34	11.2
PS	44	M	13	1	23.1	4.2	159	1.07	0.95	74.01	1.6	high	9.3	5.1	0.09		12.1
PS	66	F	27	1	20.4	4	144	1.86	1.1	89.8	1.5	high	9.6	5.4	0.09	26	9.9
PS	52	F	3	1	20.4	4.3	119	1.46	1.26	114.3	1.8	high	9.4	5.2	0.01		10.3
PS	47	M	18	1	18.4	4	264	1.49	0.78	96.61	2.1	high	9.2	4.8	0.05	26	10.1
PS	62	F	21	1	21.5	4.1	57	1.37	0.9	94.97	1.8	high	10.7	5.5	0.05	39	10.8

PS	63	F	11	1	23.7	4.2	205	1.71	1.05	97.01	2.1	high	8.5	4.2	0.06	27	9.9
PS	51	M	10	1	22.2	4.3	143	1.37	0.92	121.8	1.5	high	8.8	6.3	0.05	31	10.7
PS	82	F	5	1	19.7	3.7	219	1.85	0.91	84.39	2.1	high	8.6	4.2	0.42	25	9.3
PS	62	M	5	1	23.5	4.3	50	1.55	0.5	12.08	2.1	high	9.1	4.8	0.04	24	9.8
PS	55	M	35	1	23.8	4.2	183	1.37	0.69	68.11	1.8	high	9.2	7.8	0.05	30	11.2
PS	66	F	3	1	22.4	4.3	145	1.69	1.14	84.95	2.1	high	8.4	4.5	0.22	25	10.5
PS	51	F	11	1	20.7	4.2	110	1.14	1.03	103.9	1.8	high		6.8	0.09	20	12.8
PS	55	M	6	1	25.2	4	144	1.5	0.85	85.25	1.6	high		6.5	0.67	31	9.3
PS	61	M	14	1	19.2	4	130	1.22	0.92	112.8	2.1	high	8.9	4.1	0.17	28	10.8
PS	59	F	15	1	17.2	4.1	166	1.55	0.8	95.88	1.5	high	10.2	4.9	0.06	26	10.1
PS	54	F	20	1	23.4	4	159	0.83	0.87	108.8	2.2	high	9.7	8.8		28	10.9
PS	62	M	2	1	23.6	4.2	136	1.46	0.69	94.37	1.5	high	9	5.5	0.05	25	11.1
PS	49	M	30	1	21.1	4.2	182	1.61	0.92	85.89	1.8	high	9.8	5.2	0.05	21	10.2
PS	69	M	15	1	21.1	3.5	166	1.24	0.68	87.62	1.5	high	8.9	6.7	0.21	35	10.6
PS	74	F	2	1	29.5	3.6	133	0.97	0.57	108.7	1.5	high	6.9	4		36	10.7
PS	55	M	25	1	20.3	4.6	154	1.17	0.91	120.8	1.8	high	8.2	6.7	0.1	25	10.7
PS	64	M	24	1	18.8	4	165	1.08	0.81	90.08	1.8	high	8.4	5.4	0.05	21	10.4
PS	49	M	22	1	24.2	4.2	151	1.22	0.6	96.03	1.5	high	8.1	5.7	0.17	22	10.8
PS	60	F	14	1	24.2	4	161	1.55	0.87	106.7	1.6	high		2.9	0.21	18	9
PS	74	M	13	1	20.4	4	147	1.84	0.96	123.5	1	high		3.3	0.71	33	9.1
PS	69	M	12	1	21.8	4.1	172	1.13	0.72	126.4	2.1	high	8.8	3.6	0.1	6	10.4
PS	55	M	12	1	18.1	3.9	129	1.75	0.95	96.38	1.5	high	9.2	5.3	0.14	32	10.3
PS	65	F	11	1	16.5	3.9	151	1.37	0.75	105.2	2.1	high	9.2	5.1	0.08	30	12.5
PS	50	F	10	1	29	4.2	109	1.36	0.9	135.1	1.8	high	9.5	4.6	0.07	32	7.4
PS	41	F	10	1	37.7	4.5	208	1.34	0.86	99.53	1.8	high	9.8	4.3		23	10.5
PS	71	M	9	1	24.6	4.1	165	1.58	0.67	125.8	1.9	high		4.6	0.24	21	8.8
PS	40	F	9	1	22.2	4.7	88	1.1	1.13	130.6	2.1	high	8.4	4.6	0.75	29	9.2
PS	21	M	9	1	22.2	4.6	115	1.37	1.32	93.82	1.8	high	9	7.7	0.81	33	8.1
PS	47	F	9	1	17.3	3.9	153	1.38	1.16	82.12	2.1	high	8.9	6.8	0.08	26	10.4
PS	38	M	9	1	21.5	4.2	138	1.39	0.88	105.4	1.8	high		4	0.35		
PS	38	F	9	1	24	4.1	165	1.19	0.97	116.3	2.1	high	8.8	7.2	0.3	27	12.2
PS	27	M	8	1	22.2	4.4	156	1.51	1.11	89.68	1.5	high		5.6	0	17	11.6
PS	55	M	8	1	21.1	4.1	133	1.27	0.86	107.2	2.1	high	9.3	6.7	0.1	21	11.7
PS	55	F	8	1	20.7	4.1	126	0.78	1.14	121.4	1.8	high	8.6	5.2	0.09	22	10.2
PS	67	M	7	1	22.4	4.3	183	0.92	1.18	152.4	1.8	high	8	5.6	0.22	23	10.8
PS	72	F	7	1	18.3	3.8	114	0.97	1	128.8	1.8	high	9	7.5	0.22	22	11.7
PS	45	M	6	1	22	3.9	169	1.2	0.57	105	2.1	high	8.5	6.3	0.06	26	10.3
PS	69	M	6	1	22.3	4.3	135	1.19	0.61	111.5	1.9	high	9.5	5.5		30	11.1
PS	75	F	6	1	22	4.3	109	1.53	0.92	121.4	2.1	high	9.5	7.4	0.23	33	11.6
PS	34	M	6	1	19.2	4.2	201	1.46	1.1	115.4	1.5	high	8.5	5.4	0.5	21	9.9
PS	54	M	6	1	20	3.9	133	1.25	1	77.09	2.1	high	8.9	4.2	0.08	24	10.7
PS	78	M	6	1	20.4	4.1	78	1.21	0.79	132.1	1.8	high		7.5	0.15	33	9.6
PS	70	M	5	1	18.5	4.3	219	1.51	0.9	132	2.1	high	9.9	5.6	0.2	30	10.2
PS	65	M	5	1	19.7	4.3	168	1.58	0.8	104.1	1.5	high	9.1	5.2	8.33	25	8.2
PS	58	M	4	1	24.8	4.4	248	1.57	0.81	121.4	1.5	high	8.7	4.1	0.15	28	11.7
PS	55	M	4	1	28.2	4.6	145	1.65	0.81	91.88	1.1	high	8.2	2.8	0.1	25	10.3
PS	60	M	4	1	21.2	3.7	196	1.54	1.11	123.3	1.8	high	9.3	6.2	1.79	32	11.6
PS	76	M	4	1	17.4	3.6	123	1.31	0.84	93.54	1.8	high	8.9	5.2	0.96	28	9.4
PS	78	M	4	1	21.5	4	192	1.85	1.02	99.01	1.3	high	9.2	5.6	0.15	27	11.9
PS	52	M	4	1	23	4.5	145	1.11	0.89	102.5	1.8	high	9	7	1.09	25	10.9
PS	57	M	4	1	21.8	4.5	129	1.5	0.75	123.4	1.9	high	7.9	6	0.17	40	11.8
PS	75	M	4	1	26.3	4	175	1.78	0.83	106.1	2.1	high	8.6	6.7	3.69	37	9.8
PS	68	F	4	1	23.3	3.9	167	1.57	0.79	106.4	1.5	high	7.9	8.3	1.41	38	9.4
PS	58	M	3	1	24.1	3.7	130	1.12	1	100.8	2.1	high		4.2	0.05		
PS	54	F	3	1	32.8	4	251	1.73	0.84	76.14	1.9	high	8.4	5.3	0.01	25	11.2
PS	70	M	3	1	22.2	4.1	176	2.06	1.21	83.73	1	high	8.9	5.1	0.29	33	9.2
PS	56	M	3	1	30.3	4.3	197	1.85	0.98	113	1.3	high	9.6	3.8	0.15	20	9.1
PS	49	F	3	1	31	4.6	127	1.08	1.06	136.7	1.6	high	8.6	4.9	0.2	17	8.5
PS	66	M	3	1	20.9	3.6	151	1.73	0.76	121	1.5	high	8.2	7.4	0.07	33	13

PS	87	F	3	1	14.7	3.4	223	1.47	1.02	69.01	1.3	high	9.7	5	0.14	25	10.2
PS	71	M	3	1	25	3.7	138	1.71	0.76	96.2	1.6	high	10.3	5.5	0.03	34	10.2
PS	76	M	2	1	19.8	4.1	134	1.08	1	101.1	1.8	high	9.8	5.2	0.08	28	12.4
PS	66	F	2	1	20	3.8	112	1.35	0.69	80.86	2.1	high		6.5			10.5
PS	85	F	2	1	19.7	3.9	168	1.41	0.57	59.52	2.1	high	9.7	5.1	0.02	25	11.7
PS	68	M	2	1	26.6	4	106	1.12	0.77	86.36	1.5	high		4.8	0.96	29	11.4
PS	77	F	2	1	20.8	3.6	160	1.57	0.65	76.52	1.5	high	8.6	5.9	0.01	28	8.7
PS	59	M	2	1	23.1	4.2	169	1.66	0.81	86.15	2.1	high	7.7	7.9	0.04	26	10.5
PS	35	M	2	1	21	4.1	218	1.78	0.81	92.34	1.8	high		7.6	0.15	26	10.1
PS	59	F	2	1	25	4.1	153	1.76	0.95	74.98	1.5	high	9	5.6	0.58	32	8.6
PS	59	M	8	1	21.7	3.7		0.98	0.63	81.42	1.3	high		3.5			10.6
PS	62	F	17	1	19.6	3.9	161	1.66	0.87	120.1	1.4	high	9.5	6.5	0.06	25	10.4
PS	70	M	3	1	19.7	3.3	138	1.13	0.96	138.9	2.1	high		6.3	0.01	28	9.7
PS	79	F	5	1	29.3	3.7	152	1.29	1.06	104.5	1.5	high		4.9	0.06	30	10.3
PS	67	F	11	1	17.8	3.9	148	1.23	1.08	116.9	1.5	high		3	0.42	19	11
PS	77	F	6	1	22.6	3.6	167	1.17	0.83		1.3	high	8.9	5.2	0.05	25	10.8
PS	57	M	8	1	22.1	3.9	164	1.57	1.02	117.8	1.5	high	8.4	4.9		29	11.6
PS	81	F	7	1	19.1	3.6	162	1.43	0.85	119.4	1.8	high	8.6	6.8	3.43	25	11
PS	58	M	17	1	22.9	3.7	138	1.8	0.9	109	1.1	high	9.6	4.5	0.06	32	11.7
PS	72	M	3	1	22.9	3.9	151	1.73	1.11	84.41	2.1	high	9.3	6.3		35	10.6
PS	70	M	4	1	21.5	3.9	117	1.57	1.04	105.6	1.6	high	9	6.9	0.03	23	11.4
PS	53	F	4	1	25.3	4.1	151	1.49	0.8	63.11	2	high	9.9	6.1		18	9.5
PS	43	F	19	1	20.3	3.3	153	1.42	0.85	119.4	2.2	high	8.6	7.9		28	7.7
PS	72	F	7	1	25.7	3.4	135	1.55	0.85	99.97	1.6	high	8.5	3.7	0.05	28	10.5
PS	53	F	7	1	15.4	4.1	185	1.06	1.2	104.3	2.1	high		5.7		20	9.6
PS	68	F	6	1	23.4	3.9	105	1.66	0.92	102.7	1.6	high	8.8	6.6	1.1	23	8.4
PS	64	M	13	1	21.4	4.2	79	1.85	1.16	100.5	1.5	high	8.7	4.4	0.22	39	10
PS	58	F	7	1	21.2	3.8	129	1.28	0.95	105.1	1.5	high	10.2	4.9	0.05	24	9.5
PS	76	F	24	1	17.5	3.5	113	1.21	1.07	124.5	2.2	high	9.2	7.8		31	9
PS	35	M	14	1	20.8	3.7	103	1.24	0.99	117.8	1.8	high		4.9	0.85		
PS	66	M	12	1	20.5	3.6	147	1.28	0.84	104.8	1.6	high	9.1	5.2		29	10.5
PS	68	F	8	1	22.9	3.5	152	1.43	1.33	124.7	1.5	high	9	4.9	0.05	31	10.6
PS	80	M	3	1	22.7	3.6	150	1.28	0.94	61.52	1.5	high	9.8	4.8	0.23	27	12.3
PS	60	M	14	1	21.1	4	194	1.93	0.99	95.91	1.5	high	9.1	5.3			9.8
PS	55	F	7	1	14.5	3.5	122	1.1	1.02	88.84	2.1	high	10.3	7.8		35	12.6
PS	68	F	3	1	20.3	3.9	212	1.38	0.96	103.7	1.3	high	9.5	6.4	0.05	25	12.9
PS	63	F	2	1	21.7	3.5	197	1.44	1.15	106.1	2.1	high	8.3	6.4	0.89		10.5
PS	46	F	8	1	27.2	3.5	158	1.16	0.81	97.34	2.1	high	8.8	5.6	0.03		10.9
PS	77	F	8	1	17.4	3.4		1.56	1.32	113	2.1	high	9.3	3.8	0.1	26	10.8
PS	40	F	27	1	25.8	4.1	120	1.68	0.91	32.91	1.3	high	9.8	4.3	0.04	35	10.5
PS	70	F	12	1	23.9	3.7	160	1.43	0.75	127.7	1.3	high	7.6	4.3	2.41	34	10.2
PS	65	F	12	1	24.9	3.4	130	1.18	1.03	158.8	2.1	high	8.6	6.4	0.05	33	13.5
PS	51	M	8	1	21.3	4.4	156	1.54	0.86	128.2	1.5	high	10.1	5.6	0.3		
PS	59	F	7	1	22.3	3.6	205	1.24	0.95	118.2	1.5	high	8.8	5.6	0.3	20	10.4
PS	65	M	16	1	18.9	3	133	1.37	0.72	97.11	1.8	high		6.8	0.28	23	10.2
PS	46	F	13	1	17.8	3	195	1.28	0.48	94.27	1.6	high		5.1		35	10.5
PS	58	F	10	1	17.2	3.6	136	1.38	0.98	100.9	2.1	high		3	0.13	34	9.2
PS	60	F	6	1	22	3.8	136	1.57	0.92	77.66	1.8	high	9.6	4.3	0.04	34	11.5
PS	56	F	12	1	21.4	3.7	205	1.89	0.79	119.8	1.3	high	8.6	4.1		15	10
PS	59	F	6	1	24.8	3.7	123	1.16	1.02	91.75	2.1	high	11	8.4	0.07	30	11.5
PS	52	F	10	1	29.6	3.9	221	1.3	0.85	106.4	2.1	high	8.1	5.9	0.05	24	9.4
PS	53	M	8	1	22.1	3.3	99	1.26	0.72	108.9	1.3	high	8.2	6.6	0.4	34	11.3
PS	40	M	8	1	18.2	4.5	199	1.02	1.02	94.71	2.5	high		9.1		17	10.2
PS	70	M	2	1	24	3.7	174	1.26	1.08	133.8	1.5	high	8.4	4.3	0.11	22	10.3
PS	52	M	7	1	25.1	3.6	146	2.04	1.08	137	1.3	high	9.6	6	0.12	29	9.5
PS	74	F	5	1	21.9	3.6	114	1.17	0.66	101.1	1.5	high		2.4	0.02		9.1
PS	78	F	4	1	20	3.6	196	1.28	0.75	139.5	1.8	high		4.6	0.3		10.2
PS	71	F	4	1	20.8	4.1	164	1.5	1.25	108.2	1.4	high	9.6	5.5		31	
PS	41	F	3	1	28.8	3.9	207	2.27	1.07	104.5	1.8	high	8.8	3.4	0.07	23	11

PS	68	M	3	1	21.8	3.8	154	1.25	0.8	7.76	1.5	high	8.7	4.8	0.45		10.1
PS	66	F	30	1	18.3			1.62	1.12	81.15	1.8	high	9	5.1			10.9
PS	42	M	28	1	22.4		134	2.35	1.09	95.33	1.4	high	7.7	4.6	0.05	26	10.7
PS	51	F	25	1	19.1		195	1.62	1.02	118.6	2.2	high	9.2	5.9	0.1	26	11.5
PS	40	M	25	1	17.3		177	1.41	1.18	100.3	1	high		5.7		29	10.4
PS	61	F	24	1	15.5		147	1.23	1	118.7	1.4	high		2.9	0.41	14	10.6
PS	41	F	23	1	20.6		150	1.18	0.9	123.1	2.1	high	8.6	3.2	0.16	26	12
PS	58	M	23	1	20.1		131	1.59	0.83	101.4	1.8	high	8.2	5.9		27	10.6
PS	43	F	23	1	21.9		177	1.11	0.92	107.5	2.1	high	9.5	9.3	0.16	29	11.1
PS	67	M	22	1	16.3		137	1.45	0.74	76.9	1.8	high	8.5	4.3	0.1	31	11.4
PS	55	F	21	1	17.7		195	2.09	0.92	113.8	1	high	10	3	0.93	27	8.2
PS	59	F	21	1	18.3		187	1.89	1.19	104.9	1.5	high	8.9	3	0.02	30	9
PS	58	F	21	1	25.2		173	1.57	0.9	128.9	1.5	high	8.6	4.8	0.03	36	6.7
PS	39	M	20	1	23.7		194	1.15	0.99	105.1	1.8	high	8.7	5.4	0.13	23	9.8
PS	69	F	18	1	20.3		173	1.16	0.83	78.68	1.8	high	9.9	3.8	0.08		9.3
PS	46	F	17	1	18.8			1.23	1.08	125.1	1.3	high	9.1	7.8	0.01	30	10.2
PS	29	F	17	1	24.8			1.79	0.82	103.4	2.1	high	9.1	5.9	6.04		12.8
PS	42	M	16	1	19.8			1.89	0.85	101.9	1.3	high	9.6	6.4		25	11.9
PS	64	M	15	1	21.7		148	1.49	0.85	86.48	0.8	high	10	2.9	0.91	23	10
PS	68	F	13	1	19.1		148	1.51	0.67	105.4	1.3	high	9.8	6.2	0.06	30	9.9
PS	69	F	13	1	20		150	1.4	0.87	110	1	high	7.5	4.1	0.31	22	10.5
PS	45	M	13	1	21.1		193	1.94	0.71	98.75	1.6	high	9.4	5.2	2.85		8.1
PS	62	M	11	1	22.4		135	1.26	0.76	124.5	1.6	high	10.1	4.3		28	8.3
PS	60	F	11	1	21		231	1.18	0.86	116	1.8	high	9.2	5.4	0.44	36	11.9
PS	63	M	11	1	20.9		118	1.65	0.93	103.2	2.1	high	10	7.9	0.3	38	11.4
PS	71	M	10	1	22.4		150	1.09	0.89	137.4	1.8	high	8.1	3	1.46	30	10.6
PS	65	M	9	1	19.9		150	2.15	1.06	105.5	1.8	high	9	4.2	0.03	18	10.1
PS	63	F	9	1	18.4			1.44	1.06	134.2	1.8	high	8.5	3.5	0.2	15	12.2
PS	53	M	7	1	25		170	1.66	1.03	104.1	1.5	high	8.8	3.5	0.62	32	10.8
PS	76	F	7	1	25.7		155	1.57	0.69	163.1	1.4	high	8.3	5.1	0	23	11.7
PS	80	M	6	1	20.7		138	1.46	0.84	117	1.3	high	9.1	5.5	0.05	43	10.9
PS	73	M	5	1	26.1		144	1.04	0.82	113.2	1.8	high	9.7	8.6	0		10.4
PS	32	M	4	1	15.1		163	1.31	0.84	81.07	1.8	high	11.1	8.3	0.07		8
PS	74	M	4	1	20.9		157	1.33	0.54	117.5	1.8	high	8.7	5.1			10.1
PS	76	M	4	1	24.6		249	2.15	0.79	94.58	1.3	high	8.9	5	0.04	28	9.4
PS	60	F	4	1	25.4		137	1.2	0.92	114.2	1.8	high	10.4	4	0.07	26	11.3
PS	72	M	4	1	21.8		132	1.41	0.69	63.87	0.8	high	8.4	3.1	5.23	31	7.8
PS	72	F	4	1	27.6		117	1.34	1	95.47	1.3	high	8.9	6.8	0.14	24	10.2
PS	63	M	3	1	28.9		119	1.36	0.85	44.87	1.5	high	8.9	4.7	0.33	26	9.3
PS	39	F	3	1	28.7		135	1.55	0.71	107.4	1.9	high	7.8	6.9	0.38	25	8.9
PS	87	F	3	1	20.6			1.55	0.89	87.21	1.6	high	8.7	4.2		19	
PS	72	M	3	1	19.9		180	1.3	0.65	95.67	1.8	high		7.1		26	10.6
PS	60	F	3	1	17.5		115	1.27	0.8	112.1	1.6	high	8.9	1.7		38	9.4
PS	68	F	3	1	21.4		126	1.27	0.81	128	1.5	high	7.8	4.2		37	10.2
PS	85	F	3	1	19.5		94	1.11	0.61	122.2	1.5	high		6	0.4	37	9.6
PS	72	M	3	1	21.3		103	1.71	0.75	114	2.1	high	9.2	6.4		40	9.1
PS	52	M	2	1	23.5		189	1.42	0.94	59.99	1.6	high	9.8	5.7	0.6	27	11.2
PS	69	M	2	1	20.6		129	1.34	0.75	74.31	1.8	high	7.9	4.4		30	10
PS	74	M	2	1	21.7		125	1.57	0.56	58.62	1.8	high	9.5	4.8			11.5
PS	75	M	2	1	23.4		159	1.65	0.76	106.4	1.8	high	8.5	6.2	1.9	36	9.8
PS	79	M	2	1	22.6		139	1.55	0.72	107.4	2.1	high	8	4.9		31	10.6
PS	51	F	2	1	19.8		98	1.27	0.81	79.06	1.6	high	9.1	3.7	0.12	30	9.6
PS	56	M	2	1	22.3		167	1.78	0.84	91.61	1.8	high	9.2	4.4			11.6
PS	53	F	2	1	24.6		203	1.06	0.76	76.83	2.1	high	8.9	2.5	0.64	33	10.1
PS	49	F	2	1	18.9		127	1.44	0.87	128.4	1.8	high	8.9	5	0.1	8	11.2
PS	78	F	2	1	25.1		128	1.56	0.83	91.02	2.1	high		4		28	11.3
PS	50	M	2	1	19.6		108	1.39	0.79	106.1	1.8	high	9.1	4.4	0.16	16	10.8
PS	71	M	2	1	22.5		220	1.36	0.82	95.14	2.1	high		5.4			10.7
PS	71	M	2	1	20		74	0.74	0.7	98.29	1.6	high	7.6	4.1	7.3		

PS	59	F	2	1	23.4		111	1.23	0.93	86.26	1.5	high		4.7	0.43		8.2
PS	58	M	2	1	20.6		103	1.02	0.75	85.16	1.3	high	13.2	7.7	10.24	25	8.2
PS	35	M	3	1	16.2	4.3	114	1.67	1.36	96.56	1.5	high	8.6	5.6	0.2	26	10.4
PS	67	M	3	1	19.3	4.1	178	1.81	1.19	95.7	1.8	high	9.5	5	1.7	32	9
PS	73	M	12	1	18.3	4	182	1.1	0.87	106.9	1.6	high		5	0.24	25	9.8
PS	59	M	2	1	16.6	3.7	243	2	0.55	63.15	1.5	high	10.9	5.4	0.85	27	10.1
PS	70	F	12	1	21	3.9	155	1.48	0.96	120.6	1.4	high	8.9	3.2	0.96	31	8
PS	62	M	2	1	25.1	3.7	177	1.56	1.03	94.71	1.5	high	10.4	3.2	0.04	29	9.4
PS	48	M	17	1	21.3	4.2	170	1.41	0.79	84.74	1.5	high	9.7	4.5		32	9.3
PS	58	F	2	1	24.8	4.2	135	1.3	0.97	59.85	2.1	high	8.1	6.7	0.05	28	10.9
PS	66	F	16	1	18.6	3.9	133	1.24	0.9	120.2	1.3	high	8.2	2	0.3	7	11
PS	63	F	6	1	21.7	4	127	0.64	0.88	94.59	1.5	high	8.4	5.5	3.7		8.4
PS	69	M	6	1	23.2	3.7	72	0.65	1.11	155.3	2.1	high	8.3	5.7	2.14	30	10.4
PS	63	M	5	1	16.2	3.7	139	1.29	0.86	112.2	1.5	high	9.8	4.2	0.11	29	10.4
PS	72	M	5	1	19.7	3.3	173	1.46	0.91	131.3	1.8	high	8.6	4.6	0.05	26	10.9
PS	46	F	2	1	32.2	3.8	165	1.63	0.85	113.2	1.5	high	9	6.8	0.49	44	9.6
PS	47	M	10	1	23.3	3.5	93	0.88	0.82	100.3	1.5	high	8.5	4.9	0.15	33	10.2
PS	80	F	6	1	21.6	3.9	176	1.8	0.75	109.3	1.5	high	8.7	5.7		24	11
PS	65	F	3	1	30.8	3.8	133	1.1	1.03	101	1.5	high	9	4.5	0.27	27	10.3
PS	55	F	2	1	18.4	3.4	142	2.45	0.99	98.68	1.5	high	9.8	3.9		16	10
PS	60	F	30	1	19.9	4.1	195	1.78	0.95	119.5	1.5	high	10.2	9	0.42	38	8.7
PS	64	M	8	1	19.7	3.8		1.19	0.96	100.6	1.1	high	9.1	2.2	0	21	11
PS	48	F	7	1	17.5	4.2	155	1.62	0.93	110.6	1.8	high	9.2	5.7	0.28		10.1
PS	34	F	4	1	16.4	4.2	153	1.42	1.09	114.9	1.8	high	9.1	4.6			12.8
PS	57	F	16	1	17.5	3.8	151	1.47	0.94	100.8	1.5	high	9	5.1		24	10.3
PS	72	M	2	1	23.1	4.2	180	2.24	0.89	81.97	2.1	high	10.3	4.5		24	10.2
PS	58	M	2	1	21.9	4.1	165	1.34	1.07	118.7	1.6	high	10.5	7.6	0.36	29	9.7
PS	57	M	2	1	22.1	3.8	185	1.71	0.86	75.49	1.5	high		3.5	1.81	35	9.9
PS	59	M	2	1	20.2	3.8	146	1.63	0.72	82.51	2.1	high		4.5	0.05	6	11.1
PS	46	F	12	1	14.9	4.2	179	1.87	0.99	89	1.3	high	8	6	0.18	21	9.9
PS	69	F	9	1	22.3	3.7	91	1.48	1.03	131.8	1.8	high	9.1	5.1		26	11.6
PS	70	M	9	1	18.3	3.6	71	1.4	0.77	113.6	1.3	high		1	4.1	29	9.4
PS	65	F	5	1	19.1	3.6	129	1.86	1.21	116.3	1.5	high	8.8	5.6			10.8
PS	73	M	3	1	21.3	4.2	136	1.19	0.82	130.5	1.8	high	8.8	5	0.3	29	10.3
PS	65	M	7	1	18.8	3.7	169	1.47	0.92	83.59	2.1	high	8.8	5.9	0.05	27	10.8
PS	73	F	3	1	19	3.7	139	1.65	1.03	133.8	2.1	high	9.6	1.9	0.16		11.6
PS	66	M	24	1	22	3.6	170	1.62	1.02	101.8	1.8	high	8.9	5.7	0.04	23	10.8
PS	60	M	19	1	20.4	4.1	144	1.45	1.14	128.1	1.8	high	9.7	6.6	0.06	27	9.9
PS	58	M	15	1	21.1	3.9	130	1.34	0.75	102.7	1.8	high	10.3	5.6	0	29	10.1
PS	72	M	9	1	18.2	4	163	1.57	0.66	90.89	1.8	high	9	5.9	0.03	20	10.7
PS	68	F	11	1	15.1	3.9	196	1.42	1.05	128.7	2.1	high	9.1	6.2	0.33	28	9.4
PS	69	M	7	1	23.7	3.7	129	1.43	1.03	114.2	1.8	high	9.1	5.6	0.11	29	10.9
PS	61	F	5	1	20.1	4.1	134	1.38	1	101.1	2.1	high		5.5	0.05	8	12.4
PS	68	M	4	1	19.6	3.8	133	1.4	0.81	110.7	1.6	high	9.1	5.7	0.36	29	11
PS	60	M	2	1	21.2	3.6	146	1.59	0.94	72.72	2.1	high	8.8	7.1	0.21	33	11.5
PS	57	M	9	1	18.2	3.7	133	2.09	1.01	83	1.5	high	9.4	4.4	0.02	30	10.6
PS	66	M	7	1	16.7	4	174	2.17	0.92	108.5	1.5	high	8	3.4	0.07	22	10.6
PS	71	F	5	1	27.9	4.2	172	1.33	0.79	96.46	1.8	high	8.2	5.5	0.27	25	9.9
PS	59	M	8	1	22	3.6	151	1.26	0.99	89.89	2.1	high	9.2	5	0.05	21	11.1
PS	66	F	10	1	21.2	3.8	105	1.42	0.83	133.2	1.6	high	7.4	2.9			
PS	53	M	2	1	27.9	4	71	1.4	0.66	84.16	1.3	high	7.8	5.8	0.13	34	10.1
PS	66	F	7	1	19.9	3.7	146	1.47	0.72	103	2.1	high	8.8	4.4	0.25	33	10.2
PS	60	F	10	1	19	4	179	1.54	1.35	140	2.1	high	8.4	4.9	0.05	32	12.2
PS	66	M	2	1	20.8	3.8	141	1.41	1.07	103	2.1	high	8.1	5.6	0.05	28	10.3
PS	62	F	6	1	18.9	3.9	183	1.46	0.81	101.3	1.6	high	8.7	6	0.09	19	9.7
PS	40	F	7	1	20.4	4.1	166	1.62	1.44	135.2	1.8	high	9	9.6	0.03	32	8.9
PS	64	M	7	1	21.7	3.5	145	1.31	0.81	103.3	2.5	high	9.6	5.3	0.2	28	9.8
PS	51	M	20	1	16.6	3.5	172	1.61	0.92	60.14	1.6	high	9.6	5.7	0.57	41	11
PS	75	M	23	1	19.4	3.2	136	1.6	0.99	110.2	2.1	high	7.6	5.3	0.46	31	10.7

PS	54	F	12	1	20.9	3.9	91	1.38	0.93	127.6	2.1	high	9.2	5.3	2.16		10.7
PS	58	M	5	1	22.2	3.4	147	1.18	1.06	105.2	2.1	high		7	0.05	28	11.1
PS	57	M	25	1	22.2	4	135	1.36	0.84	94.27	2.1	high	9.7	7.2		20	11
PS	74	F	7	1	23.4	3.7	115	1.29	0.9	134.7	2.1	high	8.4	4.5	0.04	27	9.3
PS	62	M	27	1	19.3	3.3	129	1.56	0.83	87.25	1.5	high	8.4	4.6	0.11	20	9.4
PS	72	M	2	1	16.5	3.5	199	1.41	0.63	20.77	1.5	high	10.4	5.6	0.05	26	12.3
PS	60	M	4	1	26.3	4	169	2.08	0.78	75.79	1.6	high	10.7	5.6	0	29	9.6
PS	65	M	10	1	20.8	3.6	137	1.26	0.9	111.6	1.8	high	9.1	4.8	0.05	21	9.8
PS	50	M	31	1	17.7	3.4	153	1.42	0.72	92.65	2.1	high	7.7	5.7	0.12	25	10.8
PS	61	M	16	1	21.6	3.7	119	1.25	0.93	104	1.6	high	9.6	5.6	0.04	23	10.3
PS	70	M	4	1	24.5	3.8	146	1.13	1.09	118.6	1.1	high	8	5.9	0.1	31	10.5
PS	68	M	20	1	19.5	3.8	206	1.53	1.13	112.5	1.4	high	10.1	5.2	0.06	21	11
PS	50	M	17	1	21.1	3.8	117	1.32	0.91	133.7	2.1	high	9.6	6.3	0.09	28	10.9
PS	53	F	14	1	34.6	3.7	201	2.4	0.83	93.03	2.1	high	8	3.4	0.03	23	10.3
PS	59	F	14	1	23.6	4.1	105	1.75	1.09	127.2	1.9	high	8.5	5.4	0.05	25	12
PS	42	F	10	1	25.2	4.1	217	2.07	0.84	124.1	1.6	high	9	4.4	0.09	22	10.9
PS	57	F	12	1	17.6	3.9	103	1.36	1.11	154.3	2.1	high	8.8	5.6	0.02	25	10.5
PS	49	F	2	1	17.1	4.1	202	2.39	0.7	90.16	1.6	high	9	3.7	0.05	21	12.3
PS	71	M	15	1	18.7	3.7	222	1.36	0.81	108.6	1.8	high	8.7	4		30	10.4
PS	58	F	9	1	17.1	4.1	136	1.11	1.61	128.7	1.1	high	8.7	4.5	0.01		10.4
PS	63	M	2	1	23.8	4.2	196	2.01	1.1	86.1	1.8	high	8.2	5	0.04	27	9.8
PS	60	M	30	1	16.6	3.7		1.68	0.97	78.41	1.6	high	9.3	3.4	0.16	35	10.6
PS	48	M	5	1	26.2	3.9	190	1.78	0.85	116.4	1.8	high	9.8	5.9	0.08	29	10.4
PS	52	M	3	1	21.6	4.1	155	1.57	0.92	98.03	2.1	high	8.8	4.4	0.11	31	10.7
PS	58	M	11	1	24.6	3.7	163	1.74	0.94	126.8	1.3	high	8.3	3.2	0.26	32	11.5
PS	45	M	12	1	23	3.7	169	1.5	0.94	134.5	2.1	high	9.4	6.2	0.27	23	9.7
PS	53	F	6	1	19.9	3.7	133	1.34	1.06	131.8	2.1	high	9.9	5.5	0.07	28	11.2
PS	46	F	10	1	23.2	3.6	133	1.16	1.06	144.1	2.1	high	9.1	5	0.26	29	8.1
PS	53	M	12	1	20.1	3.7	206	1.49	1.46	109	2.1	high	9.6	4.7	0.02	22	10
PS	33	M	9	1	21	3.9	114	0.93	1.11	105.9	1.5	high		4.8	0.07		8.1
PS	57	M	18	1	21.1	3.9	146	1.2	0.92	97.56	2.1	high	9.3	5.6	0.12	33	11
PS	71	F	4	1	24.2	3.7	175	1.13	1.18	76.57	2.2	high	9	5.8		42	9.6
PS	69	F	15	1	21.5	3.8	222	1.35	0.91	130.3	2.1	high	8.7	6.2	0.08	30	9.7
PS	67	F	33	1	17.3	4	128	1.36	1.1	116.5	1.4	high	9	4.1		18	10
PS	68	M	7	1	20.8	4	113	1.33	0.9	83.28	2.1	high	8.7	5.7	0.14	16	11
PS	69	F	3	1	22.2	3.9	254	1.02	1.08	100.2	1.8	high	9.2	4.6		19	13.5
PS	60	M	10	1	19.5	3.7	173	1.44	0.89	108.3	1.9	high	8.7	3.7	0.05	21	13.5
PS	76	F	4	1	21.3	3.8	140	1.26	0.68	57.46	2.1	high	8.7	2.6	0.24	30	10.3
PS	38	F	8	1	16.2	4.1	157	1.08	0.99	134.8	1.6	high	9.2	5.5	0.06	21	10.2
PS	71	F	11	1	22	3.6	123	0.95	0.94	119.2	1.3	high		5.7		22	11.9
PS	70	M	10	1	22.4	4.1	202	1.36	1.3	141	1.8	high	10	5.9		19	10.1
PS	59	M	8	1	18.8	4.1	119	1.95	1.05	106.9	1.8	high	9.1	4.3	0.02	25	9.8
PS	41	M	5	1	26.9	3.8	133	1.44	0.74	81.16	2.1	high	8.5	5.4	0.01	32	13.3
PS	67	M	7	1	16.9	3.7	190	1.49	1.39	96.42	1.3	high		4	0.05	24	11.3
PS	67	M	17	1	17.4	3.9	112	1.49	0.89	110.2	1.4	high	11.3	5.4		33	11.5
PS	65	M	24	1	24.3	3.7	152	1.27	1	113.6	2.1	high	9.4	4.4	0.37	29	10.1
PS	66	F	19	1	24.1	4	129	1.37	1.17	111.9	1.4	high	10.7	6.4		27	11.1
PS	61	M	36	1	19.9	3.7	135	1.37	0.75	81.21	1.4	high	8.8	5.6		23	11.8
PS	59	M	2	1	25.2	3.7	119	1.54	0.79	84.34	2.1	high		4.3	1.38	19	8
PS	46	M	12	1	25.2	3.7	139	1.09	0.76	116.7	2.1	high	8.9	6.1	0.05	27	10.6
PS	55	M	10	1	19.2	3.3	150	1.41	0.78	126.9	2.1	high	8.4	6.8	0.05	21	10.2
PS	76	M	12	1	23.5	3.5	165	1.27	0.94	152.8	1.8	high	8.9	3.9		30	11.2
PS	52	F	11	1	19.3	4.1	29	1.69	0.98	102.1	1.6	high	9.6	4			10.5
PS	58	F	10	1	22	3.9	169	1.67	1	113.4	1.8	high	9.1	5.2	0.05	25	9
PS	50	F	10	1	19.1	4.1	142	2.54	0.94	131.1	1.8	high	9.5	5.5	0.23	27	10.8
PS	38	M	7	1	35.3	4.1	113	1.05	1.05	109.3	0.8	high	8.6	5.2	0.27	30	9.1
PS	64	F	6	1	19.6	3.9	275	1.85	1.07	120.9	1.5	high	9.6	6.4	0.21	23	10
PS	67	M	6	1	19.3	3.8	211	1.36	0.77	121.5	1.8	high	9.2	5	0.05	24	12.8
PS	61	F	5	1	16	3.6	140	1.13	1.04	100.4	1.8	high	9.5	5.3	0.05	25	10.9

PS	70	M	4	1	23.3	4		1.09	0.79	124.7	2.1	high	9.3	6		36	9.2
PS	54	M	4	1	28.1	3.9	153	1.22	0.75	96.07	1.8	high	9.4	4.3	0.14	25	9.9
PS	73	M	3	1	23.5	3.6	193	1.01	0.51	71.92	1.8	high	9.2	7.1	0.33	21	10.4
PS	73	M	3	1	19.1	3.7	177	1.26	0.79	67.14	1.8	high	8.3	5.9	1.4	27	10.5
PS	65	M	2	1	22.6	3.5	136	1.69	0.96	62.04	2.1	high	8.3	8.3	0	44	12.4
PS	55	M	28	1	19.7	4.3	168	0.5	1.14	104.1	1.5	high	7.3	6.5	0.07	26	9.9
PS	51	M	25	1	23.4	4	129	1.56	0.86	113.2	1.9	high	8	4.3	0.6	27	12.9
PS	63	F	21	1	18.2	3.7	163	1.73	1.03	101	1.6	high	8.9	4.5	0.07	27	9.5
PS	64	F	16	1	20.8	3.9	138	1.58	0.98	108.1	1.6	high	8.2	4.1	0.05	28	10.4
PS	66	F	15	1	16	4.2	292	2.13	0.94	102.5	1.9	high	9.9	3.8	0.07	30	10.8
PS	60	M	14	1	19.9	3.8	172	2.34	1.08	116.7	1.3	high	9.6	4.6	0.11	21	11.2
PS	32	F	12	1	25.8	3.7	157	1.22	0.87	121.9	2.1	high		5.3	0.05		11.5
PS	51	M	11	1	19.1	3.9	173	2.11	0.95	105.1	1.6	high	7.7	5.7	0.05	29	10.8
PS	59	F	10	1	15.5	3.7	162	2.1	1.24	103.8	1.6	high	8.4	5	0.09	26	11.7
PS	60	F	10	1	18.2	4.3	97	1.32	1.09	86.82	1.6	high	8.3	4.5	0.08	19	10.8
PS	60	M	10	1	20	4	154	1.43	0.91	128	1.3	high	8.4	4.2	0.01	27	10.9
PS	51	M	10	1	18.8	3.6	153	1.73	0.69	105.8	1.9	high	8.2	3.9	1.08	23	11.8
PS	65	F	9	1	17.1	3.4	185	1.83	0.94	98.84	1.9	high	8.4	6.4	0.11	23	11.4
PS	54	F	9	1	20	4.2	135	1.38	1.07	91.06	2.1	high		5.6	0.32	34	11.1
PS	31	M	8	1	20.9	4.3	111	1.67	0.97	100.7	1.6	high	9.8	4.9	0.23	29	11.1
PS	53	M	6	1	15.6	3.7	197	1.66	0.83	62.25	1.6	high	9	4.3	0.36	24	11.8
PS	61	M	6	1	21.6	4.2	99	1.56	0.88	119.6	1.9	high	8.3	3.6	0.05	24	9.8
PS	54	M	6	1	17.9	4.3	124	1.49	0.76	79.34	1.8	high	9.4	4.3	0.08	29	9.3
PS	44	F	5	1	20.9	4.2	169	1.4	0.89	124.1	1.8	high		4.6	1.98		10.4
PS	65	M	5	1	23.3	4.1	219	1.71	0.87	124.9	1.3	high	8.9	5.2		30	11
PS	64	M	5	1	22.6	3.9	120	1.06	0.9	85.08	2.1	high	9	4.4	0.1	24	10.2
PS	69	F	3	1	18.9	3.4	97	1.18	1.13	108.7	1.6	high	7.2	6.3	0.64	32	6.6
PS	76	F	4	1	19.1	4	165	1.88	0.75	111.9	1.6	high	9.2	3.4	0.4	32	12.2
PS	50	M	3	1	22.2	4.3	175	1.98	1.03	97.53	1.6	high	8.3	4.1	0.05	41	12.6
PS	54	F	2	1	17.8	3.9	151	1.57	0.69	68.09	1.6	high	8.4	3.7	0.37	26	10.1
PS	63	F	2	1	18.6	4	116	1.36	0.57	90.34	1.1	high		5.1	0.06		10.7
PS	63	M	2	1	28.5	3.6	126	1.74	0.99	105.6	2.1	high	9.6	4.7	0.1	29	11.4
PS	63	F	34	1	21	3.7	149	1.61	0.88	93.44	1.3	high	9	4.1	0.01	22	11.9
PS	67	F	4	1	26.5	3.9	192	0.97	1.14	127	1.6	high	10.7	4.4	0.21	31	11.4
PS	84	M	3	1	20	3.8	173	1.65	0.86	58.16	1.5	high	8.7	3.7	0.14	27	8.6
PS	66	F	9	1	20.2	3.8	201	1.29	1.02	103.3	1.9	high	9.2	5.2	0.1	26	11
PS	65	F	11	1	21.8	3.6	115	1.05	0.95	92.36	1.8	high	9.4	6.4	0	32	9.4
PS	70	F	8	1	19.1	4.1	142	1.21	0.79	132.7	1.8	high	8.5	7.7	1.6	42	10.3
PS	58	M	4	1	29	4.6	206	1.18	1.03	115.4	2.1	high	8	4.8		20	9.4
PS	64	F	4	1	36.7	3.5	181	1.2	0.95	115.3	1.8	high	8.5	6.4	0.91	34	15.4
PS	64	M	4	1	24.6	4	143	1.35	0.86	61.11	1.6	high	9.3	6.5	0.31	34	9.6
PS	61	F	2	1	22.6	3.7	145	1.29	0.77	77.82	1.6	high	8.1	4.6	0.03	26	11.3
PS	71	F	15	1	22.1	3.6	102	1.69	0.65	123.8	1.3	high	8.2	6	0.07	34	12.1
PS	53	M	3	1	20	3.3	161	1.05	0.71	55.78	1.6	high		6.9	0.69		9.1
PS	55	F	2	1	19.8	3.7	117	1.78	0.98	126.9	1.5	high	9.3	4.5	0.04	18	10.9
PS	62	M	6	1	19.9	3.7	129	1.11	1.05	123.8	1.5	high	8.6	4.1	0.13	27	10
PS	24	M	4	1	16.4	4.2	131	1.22	0.86	90.64	2.5	high	8.1	7.7	0.18	27	10.3
PS	56	F	5	1	18.3	3.4	218	1.52	0.85	82.53	1.8	high	9.8	4.5	0.13	27	9.2
PS	69	M	2	1	26.5	3.4	142	1.68	0.76	101.4	2.1	high	9	6.1	0.05	29	10.5
PS	22	M	4	1	23.6	4.2	131	1.08	0.85	88.31	1.5	high	9.6	5.9	2.98	23	10.2
PS	56	M	3	1	22.5	3.5	169	1.38	1.03	91.81	2.1	high	8.9	4.6	0.15	23	10.8
PS	71	M	3	1	21.6	4	162	2.01	0.55	60.91	1.5	high		5.4	0.25	36	10.4
PS	66	F	17	1	20.1	3.5	115	1.48	0.87	120.9	1.3	high	8.9	2.8	0.04	27	10.4
PS	60	M	12	1	21.9	3.9	98	1.12	0.89	89.54	1.3	high	9.3	4.6	0.66	23	12.4
PS	56	M	9	1	21.5	3.4	226	1.44	0.86	108	2.1	high	9	2.9	0.23	19	10.7
PS	68	M	4	1	16	3.6	159	1.74	0.81	31.57	1.3	high	10.4	5.4	0.1	30	8.7
PS	53	M	23	1	28.5	4.1	206	1.37	0.65	69	1.2	high	7.1	6.5	0.05	19	9.9
PS	63	M	10	1	31.5	3.8	219	1.3	0.65	66.28	1.8	high		3.4	0.1	25	13.3
PS	66	M	3	1	20.6	4.5	169	1.23	1.03	112.8	1.5	high	9.5	6.8	0.27	23	11.5

PS	65	M	13	1	22.2	3.2	125	1.7	0.87	128.1	1.5	high	9.8	5.6	0.21	39	8.9
PS	53	F	8	1	18.3	4.2	172	2.03	0.94	122.7	1.5	high	8.6	5.9	0.07	28	10.4
PS	68	M	27	1	19	3.3	187	1.84	0.93	85.82	1.5	high	7.6	4.6	0.15	27	9.3
PS	72	F	16	1	21.4	3.3	210	1.46	1.09	98.06	2.1	high	8.7	3.5	0.05	12	11.1
PS	50	M	11	1	19.3	3.9	141	1.06	0.76	114.7	1.6	high	9.1	4.6	0.86		11.6
PS	71	M	11	1	24.2	3.8	188	1.97	0.77	130	1.3	high		5.2	2.2	19	12.1
PS	84	F	10	1	16.8	3.6	195	1.43	0.83	115	1.6	high	8.7	6.9	0.7	30	9.8
PS	85	F	5	1	27.3	3.3	171	1.34	0.78	60.46	1.5	high		4.5		42	13.1
PS	61	F	5	1	19.6	3.4	124	1.27	1.09	62.27	1.8	high		5	0.17	39	10.3
PS	78	F	3	1	23.6	3.8	146	1.1	1.01	103.5	1.8	high	10.1	6	0.1	24	10.7
PS	68	F	2	1	30.2	3.7	195	1.74	0.98	73.32	1	high	8.9	5.5		31	11.7
PS	79	M	24	1	26.1	3.4	148	0.86	1.02	124.8	1.8	high	8.7	6.4	5.45	34	9.5
PS	77	F	13	1	30.7	3.5		1.56	0.75	89.15	1.1	high	10.2	6.3			10.3
PS	83	M	2	1	18.7	3.9	151	1.38	0.63	97.04	1.9	high	9	6.9	0	28	14.1
PS	76	M	10	1	28.5	3.6	133	1.5	0.82	121.3	1.5	high		7.5		28	10.4
PS	71	F	36	1	19.8	2.6	111	1.53	0.72	75.42	1.5	high	8.3	2.5	0.01	25	10.6
PS	73	F	27	1	20	3.9	97	1.39	1.2	108.8	1.6	high	8.5	1.9	0	37	11.2
PS	55	M	23	1	20	3.8	128	1.62	1.01	101.6	1.8	high	10	6.6	2.31	38	10
PS	62	M	20	1	19.1	3.6	189	1.76	1.02	108.8	1.9	high	8.4	6.1	0.4	29	11.3
PS	50	F	19	1	18.9	3.7	198	1.25	0.78	105.2	1.3	high	9	5.3	0.3	24	9.5
PS	71	F	18	1	13.3	4.3	160	0.94	0.86	102.6	1.6	high	8.5	5.5	0	32	10.4
PS	52	M	17	1	18.7	3.9	151	1.33	1.09	112.1	1.8	high	8.5	5.5	0.14	31	12.1
PS	34	M	16	1	19	4.5	137	1.55	1.17	101.6	2	high		5.7	0.21		10.4
PS	62	M	14	1	19.7	3.7	189	1.92	0.91	88.39	1.5	high	8.8	5.3	0.93	26	11.2
PS	36	F	13	1	18.4	4.1	133	1.48	0.97	115.7	1.3	high	8.7	6.6	0.06	23	10.6
PS	52	M	13	1	14.4	4.2	196	1.77	0.94	98.6	1.8	high	8.8	5.5			11.1
PS	42	M	11	1	21.9	3.9	174	1.47	0.99	118.3	1.8	high	10	4.6			12.4
PS	62	M	11	1	20.6	3.8	109	0.91	1.2	103.6	1.9	high	8.9	6.4	0.1	23	10.8
PS	32	F	10	1	16.7	3.8	177	1.38	1.1	103.6	1.5	high	7.9	2.3			9.5
PS	48	M	9	1	21.9	4	125	0.84	0.99	118	1.5	high	9.9	2.6	0.5	39	8.8
PS	57	M	9	1	20.3	3.7	148	1.29	0.98	110.3	1.5	high	9.1	3.5	0.05	26	10.3
PS	52	F	8	1	16.9	4.2	157	1.28	1.07	93.44	1.5	high	10.1	4.7	0.05	35	10.8
PS	80	F	8	1	23.5	3.9		0.96	0.88	107.6	1.5	high	9.8	6.4	0.67	13	11.2
PS	67	M	6	1	20	4.2	125	1.23	1.27	103.5	2.1	high	9.1	5.9	0.05	25	10.6
PS	38	M	5	1	21	4.5	164	1.53	0.81	110.6	1.8	high	10.7	7.4			11
PS	62	M	4	1	29	3.9	199	1.05	1	103.9	1.9	high	8.9	4.4	0	16	10.9
PS	55	M	4	1	25.1	4.2	166	1.34	1.1	128.1	1.5	high	10.3	4.4		26	11.1
PS	36	M	3	1	21.8	4.2	197	1.52	0.99		1.8	high		5.6	0.2	22	10
PS	73	F	3	1	18.6	3.4	216	1.35	0.83	90.54	1.3	high		4.7	0.12		8.4
PS	58	M	2	1	25.9	3.9	173	1.92	0.89	84.79	1.5	high	7.6	3.6			10.2
PS	57	F	7	1	23.1		170	1.33	1.27	126.1	1.5	high	8.8	5.7	0.1	30	10.2
PS	54	M	4	1	19.8	4.3	170	1.25	1.13	115.6	1.8	high	8.6	4.6	0.12	22	10.6
PS	67	F	8	1	17.3	4.2	192	1.42	1.12	101.9	2.1	high	9.9	6.1			11.1
PS	64	F	2	1	24.3	4.2	162	1.33	0.83	79.47	1.5	high	8.5	5.6	0	19	11.5
PS	32	M	13	1	17	3.7	197	1.22	0.86	108	2.1	high	9.3	5.1	0.1	29	12
PS	54	M	2	1	21.6	4	120	1.46	0.81	82.16	1.5	high	8.9	4.9			10.8
PS	74	M	8	1	20.3	3.8	183	1.66	0.99	89.16	1.1	high	8.7	7.3	0.98	30	9.6
PS	64	M	4	1	23.2	4.4	123	1.71	0.77	129.2	2.1	high	10.3	4.4	0.01		10.3
PS	74	F	3	1	24.4	4	147	1.32	0.64	74.6	2.1	high	8.3	5.7	1.01	37	11.5
PS	80	M	2	1	21.3	3.9	147	1.25	0.79	76.19	1.8	high	9.3	7.3	0.07	27	9
PS	64	M	27	1	18.2	4.1	135	1.35	1.17	108	1.8	high	8.3	4.9			7.2
PS	72	F	25	1	16	3.8	96	0.99	0.74	106.2	1.8	high	7.7	3.8	0.06	30	8.1
PS	54	M	17	1	23.4	3.9	178	1.61	1.18	128.9	1.6	high	7.7	3.2	0.1	22	9.9
PS	38	M	13	1	20.4	4.2		1.11	0.83	133.6	2.5	high	9.8	6.6	0.2	40	11.7
PS	48	M	11	1	25.6	3.6	107	1.31	0.94	152.3	1.5	high	7.8	4.9		34	8.7
PS	69	M	8	1	22.6	4.2	151	1.97	0.97	130.5	1.5	high	8.3	4.2	0.8	21	10
PS	53	M	4	1	22.4	3.8	132	1.64	0.9	128.7	1.3	high	8.8	5.8	0.07	35	10.1
PS	51	M	2	1	24	4.2	107	1.38	0.77	83.43	2.1	high	10.3	5		26	9
PS	63	M	9	1	21.4	4	125	1.49	0.73	117.2	2.1	high	8.6	5.3		29	11.6

PS	55	F	9	1	18.2	4	186	2.33	0.99	121.9	1.9	high	10.2	5.6	0.1	28	10.1
PS	76	M	6	1	21	3.5	204	1.07	1.11	132.2	2.2	high	8.7	6.3	0.03	22	
PS	77	F	6	1	18.5	3.6	144	1.53	1.02	96.95	1.9	high	10.8	4.7	0.5	36	12.9
PS	79	M	3	1	21.5	3.8	195	1	0.85	110.4	1.6	high	9.2	5.4	0.1	26	10.5
PS	80	F	2	1	21.6	3.6	148	1.64	0.82	83.64	1.5	high		4.1		28	10.9
PS	57	M	2	1	19.5	4.2	119	1.35	1.04	81.3	1.5	high	9.1	4.9	0.03	20	11.5
PS	65	F	35	1	14.8	3.8	173	1.26	0.96	118.9	1.4	high		5.2		31	9.6
PS	54	M	22	1	18.6	4.1	267	1.37	1.16	106.6	1.6	high	9.9	5.8	1.1	24	11.9
PS	76	F	13	1	26.6	3.9	129	1.12	0.96	88.22	1.8	high	8.9	3.8	1.7	20	9
PS	78	F	12	1	19		176	1.72	1.21	146.2	1.4	high	9.4	4.6		36	10.3
PS	61	M	6	1	19.3	3.5	181	1.7	1.07	139.1	1.6	high	9.6	4.6	0	26	10.2
PS	54	F	2	1	19.1	4.4	193	1.14	1.01	111.8	0.6	high	8.8	4.6		28	
PS	59	M	10	1	20	2.9	168	1.29	0.95	85.83	1.8	high		5.3	0.6	30	10.7
PS	70	F	29	1	16.3	3.7	41	1.1	1.12	114.8	1.6	high	8.2	4.7	8.81	23	
PS	71	F	25	1	17.8	3.7	176	1.48	0.71	97.22	1.6	high		4	0.1	31	7.4
PS	67	F	24	1	18.8	3.7	103	1.4	0.93	111	1.9	high	9.6	4.4	0.1	26	9.4
PS	58	F	22	1	16.1	4.2	128	1.46	0.83	101.7	1.8	high	8.6	5.6	1.58	32	9.1
PS	67	F	21	1	18.9	3.7	171	1.66	0.85	104.8	1.3	high		3	0.34	31	9.6
PS	44	F	19	0	19.8	3.7	148	1.33	1.04	126.2	2.1	high	9.2	4.9	0.2	28	8.8
PS	58	F	15	1	20.4	4.1	106	0.92	0.94	130.3	1.6	high	7.6	1.9	1.42	28	9.2
PS	43	M	15	1	17.9	4.2	122	0.97	0.79	98.67	1.6	high	8.5	5	5.77	30	9.5
PS	66	F	12	1	16.6	3.9	158	1	1.2	126	1.9	high		6.3	0.06	26	9.8
PS	67	F	12	1	18.7	3.9	57	1.13	0.97	102.9	1.6	high	7.2	2.9	0.18	21	7.9
PS	48	F	8	1	23.6	3.8		1.52	0.9	78.2	1.5	high	9.6	4.2		30	10.1
PS	54	M	8	1	20.8	3.8	165	1.1	1.03	108	1.6	high		4.9	0.16	24	10.1
PS	74	F	7	1	23.6	3.6	143	1.27	0.86	113.6	1.6	high		4.8	0.06	33	8.6
PS	71	M	6	1	25.8	3.8	138	1.51	0.69	105.1	1.6	high	9.7	3.9	0.55	29	9.6
PS	74	F	6	1	22	3.5		1.58	0.74	142.1	1.8	high	8.1	4.4	8.64	35	9.6
PS	78	F	6	1	20.6	3.5	186	1.28	0.67	95.9	1.6	high		4.8	0.05	28	9.4
PS	62	M	6	1	22.3	3.7	135	1.3	0.87	139.6	1.8	high	9.9	5.5			10.1
PS	68	F	5	1	20.5	3.7	119	1.24	0.91	109.4	1.8	high	8.1	4.7	0.1		10.1
PS	79	M	5	1	21.9	3.5	121	1.57	0.86	121.3	1.5	high	9.3	3.8	4.85	34	10.1
PS	68	M	4	1	24.2	2.7	145	1.3	0.63	87.88	2.1	high	8	5.7	0.52	26	8.5
PS	71	F	3	1	18.4	4	133	1.44	0.78	108.9	2.1	high	8.3	4.8	0.09	39	9.4
PS	43	F	3	1	18.2	4	127	1.29	0.75	110.2	1.6	high	7.7	5.2	0.05	18	11.4
PS	49	F	7	1	22.7	3.6	140	1.24	1.06	127	1.3	high		4	0.12	18	10.4
PS	57	F	6	1	20.7	3.9	211	1.54	0.99	124.9	2.1	high	9.7	7.3	0.02		11.2
PS	36	F	7	1	32.1	3.5	141	1.32	0.86	124.9	2.1	high		5	0.1	25	10.9
PS	64	M	3	1	20.3	3.8	187	1.4	0.84	89.9	1.8	high	8.8	4.1		24	10.9
PS	64	F	8	1	21.2	3.5	134	1.25	0.94	99.87	1.5	high		4.7		23	10.3
PS	45	M	7	1	20.3	3.9	161	1.34	0.83	90.99	2.1	high	9.2	3.6	0.05	26	10.4
PS	65	M	4	1	29.4	3.9	156	1.99	0.82	101.3	1.8	high	8.6	4.8			10.1
PS	54	F	7	1	22.7	3.6	160	1.04	1.2	98.06	2.1	high	9.9	5.8	0.14	27	11.8
PS	72	M	21	1	17.6	3.7	137	1.33	1.12	111.5	1.3	high	8.6	4.3			6
PS	68	M	5	1	23.6	3.6	116	1.43	0.94	120.5	2.1	high	8.7	6.6	0.11		10.2
PS	61	M	16	1	21.2	3.8	110	0.94	0.96	111.9	2.5	high	8	8.5	0.4	39	6.6
PS	54	M	14	1	21.9	3.9	129	1.55	0.78	80.2	2.1	high	7.7	4.8	0.02		11.8
PS	28	M	9	1	20.3	4.1	132	1.27	0.54	120.9	2.1	high	9.3	4.7			11.1
PS	62	M	3	1	23.3	3.8	157	1.11	0.82	94.51	2.1	high	8.1	6.7	0.1	20	11.5
PS	67	M	3	1	18.9	3.8	128	1.27	0.91	115.5	1.8	high	8.3	4.3	5.37	26	10
PS	48	M	2	1	17.2	3.3	89	1.27	0.64	68.71	1.6	high	10	5.3	0.3	27	10.8
PS	74	M	10	1	18.7	3.2	154	1.49	1.06	131	1.5	high	9.6	6.2	0.12	31	10.2
PS	52	M	8	1	20.1	3.2	168	1.39	1.23	102.7	1.6	high		5.4	0.05	28	10
PS	51	F	7	1	19.8	3.1	182	1.23	0.81	106.5	1.5	high	10.3	5.3	0.1	24	10.8
PS	61	F	7	1	21	3.1	178	1.1	1.06	131.3	2.1	high	9.6	5.7	0.44	24	9.7
PS	26	F	5	1	17.9	3.4	150	1.1	1.34	113.6	1.8	high	7.9	3.9	0.04	12	10.8
PS	53	F	4	1	17.8	3.4	121	1.25	0.59	87.41	2.1	high	10.6	7	0	31	11.1
PS	36	M	2	1	18.6	3.7	154	1.11	1.02	120.6	2.1	high	8.5	4.7	0.2	15	10.2
PS	53	F	8	1	18.6	4.7	171	1.33	1.04	115.1	1.5	high		5.7	0.06	23	10.4

PS	80	M	2	1	21.8	3.5	177	1.67	0.9	62.92	1.5	high	9.4	3.7	0	27	12
PS	54	F	9	1	21.7	3.9	136	1.05	1.09	69.19	1.1	high		7.5	0	28	10.4
PS	60	M	3	1	22.1	3.6	130	1.37	0.97	104.5	1.8	high	9.2	8.3		33	10.3
PS	52	F	24	1	18.6	3.7	180	1.68	0.91	117.6	1.6	high		6.7	0.14	26	9.6
PS	39	M	3	1	19.2	4	149	1.8	0.87	94.91	1.8	high		7.1	0.01	28	10.3
PS	73	M	19	1	23.1	3.8	144	1.33	0.95	106.1	1.8	high	8.2	4.5	0.12		10.9
PS	64	M	7	1	20.5	4	244	1.45	0.86	123.9	1.4	high	9	4.1		29	10.8
PS	56	M	3	1	25.6	4.1	181	1.55	0.53	43.67	1.3	high	9.1	4.2	0.87		9.2
PS	56	F	28	1	24.1	4	112	1.09	1.03	106.8	1.3	high	9.7	7.8	0.21		8.6
PS	54	M	23	1	19.7	4	185	1.55	1.17	110.7	1.4	high	9.8	5		28	10.7
PS	62	M	18	1	19.7	3.8	128	1.48	0.9	95.93	2.5	high	9.4	3.9			9.4
PS	65	M	5	1	19.3	3.9	188	1.52	0.89	93.82	1.5	high		4.6		23	10.6
PS	50	F	11	1	18.9	4.1	183	1.46	1	119.9	1.5	high		5.1	0.05		9.3
PS	49	F	31	1	21.1	4.4	110	2.26	0.6	89.01	1.8	high	10	4.8			10.1
PS	58	F	34	1	20.9	3.7	111	1.43	0.9	103.8	1.4	high	8	5.3		26	11.4
PS	36	M	21	1	20.5	4.2	160	1.47	0.78	87.85	1.8	high	10.2	5.7			9.9
PS	79	M	8	1	17.3	3.6	173	1.5	1.11	125.3	1.8	high	8.9	5.9		28	11.1
PS	59	M	21	1	21	4.3	118	1.34	0.9	87.85	1.6	high	10	5.2	0.03		10.9
PS	49	M	6	1	25.5	3.9	164	1.12	0.79	79.19	1.5	high	8.5	3.4		34	10.1
PS	63	M	3	1	21.7	4	160	1.15	0.69	80.98	1.8	high	7.7	7.7	0.09	25	9.7
PS	67	M	18	1	25.7	3.9	241	1.58	0.95	137.6	1.6	high	9.4	3.6	0.07		10.7
PS	39	F	12	1	15.9	4.1	130	1.22	0.89	102.2	1.5	high		4.3	0.08	18	10.4
PS	59	M	9	1	20.8	3.6	133	1.41	0.98	123.3	1.6	high	9.4	3.8	0.08		10.7
PS	42	F	8	1	19	3.9	125	1.46	1.17	88.64	1.3	high	9.4	4.9	0.1	29	11.1
PS	72	F	30	1	21.6	3.9	186	1.48	1.14	99.82	1.9	high	9.8	3.9	0.1	29	10.8
PS	66	F	12	1	20.6	3.7	129	1.49	0.88	127.2	2.1	high	8.6	2.9	0.05		9
PS	54	M	5	1	20.9	3.5	97	0.72	0.71	120.5	1.6	high	7.9	5.6			11.4
PS	67	M	4	1	21.5	4.2	263	1.81	0.65	114.4	1.5	high	8.2	6.2	2.28	30	10.4
PS	61	M	3	1	19	4	212	1.59	0.69	87.71	1.5	high	8	5.7	0.76	37	10.6
PS	50	M	2	1	27.2	3.5	172	1.53	0.82	87.19	1.3	high		6.4	0.03	29	11
PS	66	F	19	1	19.1	3.9	199	1.72	0.91	123.8	1.3	high	8.4	6.1	0.07	25	12.1
PS	62	F	11	1	17.8	4.1	179	1.81	1.03	145.3	1.6	high	10.2	4.2	0.35	23	10.4
PS	65	M	10	1	24.7	4.1	179	1.12	1.16	117.7	1.6	high		4.4	0.05		7.5
PS	64	F	9	1	17.4	3.6	190	1.75	0.84	135.6	1.8	high	9.2	8.5	0.2	32	6.8
PS	66	M	8	1	19.2	3.8	162	1.35	0.96	129.4	1.3	high	9.7	5.2	0.26	34	10.5
PS	69	M	6	1	22.3	3.9	89	1.4	0.89	87.58	2.1	high	8.8	5	0.1	38	11.5
PS	52	F	4	1	19.3	3.6	93	1.47	0.83	99.46	0.8	high		2.9	0.03		9
PS	33	F	3	1	16.6	4.2	176	1.67	0.99	99.85	2.1	high	8.6	7.6	0.31	28	9.7
PS	66	F	4	1	30.6	3.9	181	1.85	0.81	82.3	1.5	high	9.6	5	2.07	25	9.3
PS	69	M	2	1	26.2	4		1.33	0.68	85.8	1.9	high	8.6	6.8		31	12
PS	73	M	3	1	22.7	3.5	127	1.55	0.68	78.1	1.8	high		5.9	0.85	32	10
PS	75	M	2	1	21.5	3.6	122	1.85	0.69	86.31	1.4	high	8.8	5.1	1.2	39	10.5
PS	41	F	7	1	18.7	3.8	187	1.56	0.57	116.3	1.6	high	9.4	5.3	0.02	28	9.6
PS	60	M	7	1	25	4.2		1.26	1	124.2	1.9	high	9.3	6.1		25	11.4
PS	48	F	2	1	23	3.8	153	0.94	0.78	98.47	1.5	high	9.2	8.1	0	23	10.2
PS	66	F	2	1	18.8	3.9	110	0.72	0.97	102.3	2.1	high	8.6	3.6	0.1	26	10.8
PS	38	M	3	1	19.9	4.3	172	1.59	0.96	109.9	1.4	high	8.6	5.1	0.06	26	12.4
PS	71	F	4	1	19	3.7	122	1.4	0.59	80.93	1.5	high	8.5	6.4	3.71	26	10.2
PS	48	M	4	1	22.7	3.7	140	1.46	0.68	79.45	1.5	high	8.3	7.3	0.32	25	11.6
PS	75	M	4	1	22	3.8	129	1.54	0.65	87.88	1	high	10.1	7		47	10
PS	81	M	2	1	21.6	3.4	195	1.44	0.76	77.05	2.1	high	8.9	6.4	0.2	36	10.5
PS	58	M	8	1	22.2	3.6	137	1.56	0.96	112	1.8	high	9	4			7.7
PS	38	M	13	1	24.9	3.8	136	1.81	1.03	134.2	1.5	high	10.1	5.5			11.6
PS	68	F	6	1	20.9	3.8	244	1.05	0.91	148.8	2.1	high	7.4	7.5	0.1	29	9.4
PS	63	M	3	1	25.2	4	107	1.43	0.82	93.33	2.1	high	9.2	5.3	0.31	36	12.4
PS	71	F	5	1	20.2	4	107	1.99	1.17	67.2	1.5	high		6.5	11	39	9.5
PS	53	M	18	1	26.2	4.2	148	1.51	0.7	126.2	1.8	high	10.9	4.8	0.04	25	10.7
PS	71	M	8	1	20.9	4	125	1.16	0.57	48.52	2.1	high		6.9	0.06	21	9.5
PS	71	M	16	1	22.5	3.6		1.31	0.74	76.49	1.9	high	9.4	5.6		26	10.6

PS	78	F	13	1	19	3	162	1.08	0.74	67.24	1.8	high	9	5.4	0.1	43	9.7
PS	60	F	9	1	20.8	4.2	146	1.49	0.96	137.9	1.3	high	9.3	7.6	0.05	29	10.4
PS	53	M	5	1	23.6	3.7	96	1.59	1.05	139.5	1.6	high	8.2	4.6	0.08	24	10.5
PS	54	F	26	1	20.8	3.5		1.51	1.22	112.2	2.1	high	8.9	3.5	0.2	32	10.9
PS	68	F	5	1	25.4	3.4	127	1.19	1.13	102.2	2.1	high	8.9	7.8			10.8
PS	68	M	2	1	25.6	3.7	145	1.69	0.97	130	2.1	high	9.4	5.8		34	12.2
PS	54	M	16	1	18.8	4.2		1.22	0.86	100.5	1.9	high		8			
PS	59	M	2	1	24.3	4.1	194	1.63	0.81	83.71	1.3	high	8.8	4.1	0.11	31	10.4
PS	53	M	3	1	20.4	3.4	73	1.18	0.99	106.9	1.6	high	8.2	4.4	0.28	27	11.2
PS	61	M	11	1	19.4	3.8	146	1.45	1.09	93.03	1.3	high	9.1	5.1	1.08	51	10.5
PS	56	M	13	1	18.5	3.9	145	1.15	0.89	85.61	1.5	high		3.2	2.68		6.9
PS	69	F	7	1	19.4	3.4		1.36	0.83	83.49	2.1	high	9.6	5.7			10
PS	45	M	4	1	27.6	3.7	165	1.32	0.78	91.4	1.9	high	10	7.9	0.02	27	13.1
PS	59	M	6	1	19.8	3.6	135	1.32	0.94	96.96	2.1	high	8.2	5.7	0.07	26	12
PS	45	M	9	1	23.9	3.7	201	1.33	1.17	119.8	1.9	high	8.8	9.3	0.27	28	14.1
PS	69	M	4	1	25.9	3.8		1.03	0.86	92.28	1.3	high		3.8			8.2
PS	72	M	2	1	14.7	3.1	140	1.2	0.98	112.8	1.8	high		3.1	0	23	9.4
PS	66	M	24	1	22.4	4.2	114	1.32	0.94	102.3	1.8	high	9.2	5.9	0.39	32	12.6
PS	67	M	4	1	22.9	4.4	132	0.87	0.82	112.6	1.1	high		4	0	20	11.4
PS	45	M	6	1	23.7	4.2	141	1.64	0.72	38.21	1.6	high	9.1	4.9	0.08	30	11
PS	68	F	5	1	15.6		183	1.51	0.87	87.68	1.8	high	9.8	3.6		27	11.9
PS	82	M	5	1	20	3.3	123	1.59	0.78	112.9	2	high	8.5	4.1	0.51	33	10.2
PS	66	F	32	1	18.8	3.7	88	1.3	0.71	116.7	1.8	high	9.2	5.9	0.1	30	10.7
PS	57	M	29	1	23.2	3.9	146	1.44	0.89	109.3	1.6	high	8.8	5	0.35	26	12.4
PS	71	F	26	1	18.1	3.4	218	1.94	0.76	82.18	1	high	9.7	4.6	0.2	22	9.8
PS	46	M	23	1	19.7	3.7	111	1.27	0.9	116.8	1.6	high	9.7	6.1	0.13	33	11
PS	49	M	22	1	17.4	4	155	1.62	0.77	91.5	1.8	high	9.7	4.9	0.05	27	10.6
PS	69	M	21	1	19.1	3.5	168	1.99	0.78	125.6	1.8	high	9.7	11.5	0.08	26	10.6
PS	58	M	19	1	18.4	3.9	165	1.55	0.99	127.7	1.6	high		5.1	0.29	29	11.9
PS	44	M	19	1	19.6	4	153	1.35	1.2	115.3	1.8	high	8.4	4.3	0.19	26	10.7
PS	57	M	18	1	17.6	3.8	152	1.58	1.12	109.8	1.9	high	9.6	3.2		26	10
PS	63	M	18	1	17.6	3.9	116	1.7	0.89	119.5	1.9	high	9.9	6.3		26	9.8
PS	65	F	15	1	22.9	3.7	220	1.46	0.85	131.5	1.3	high	9.4	5.6	0.05	26	11
PS	72	M	14	1	17.2	3.7	164	1.51	1	136.1	1.6	high	8.6	5		26	10.1
PS	65	F	14	1	19	4.2	169	1.44	1.06	136.6	1.6	high	9.1	5.3		29	8.9
PS	70	M	13	1	18.9	3.6	115	1.51	0.98	110.8	1.6	high	9.3	4.6		30	9.3
PS	61	M	13	1	18.8	3.6		1.88	0.71	121.1	1.5	high	9.7	7.7	0.22	31	12.3
PS	77	M	13	1	18.5	3.8	205	1.5	0.84	129.3	1.5	high	9.7	5.3	4.33	29	8.9
PS	46	M	11	1	20.1	3.7	162	1.62	0.91	103.1	1	high	7.2	7.4		19	11
PS	37	M	10	1	17.1	4.1	153	1.59	0.81	98.72	2	high	9.5	5	1.41	35	8.9
PS	58	M	10	1	18.8	3.9	118	1.49	1.16	109	1	high	8.5	5.4	0.1	35	11.3
PS	52	M	10	1	19	4.2	123	1.23	1.02	88.94	1.3	high	8.8	4.7	0.1	26	11.4
PS	69	M	9	1	21.3	3.6	168	1.12	1.12	106.8	1.5	high	8.9	7.2	1.2	33	12.3
PS	58	M	9	1	18	4.1	132	1.73	1.03	141.4	1	high	8.6	4.3	0.2	25	8.9
PS	56	M	8	1	19	3.7	113	1.33	1.18	135.3	2.1	high	9.8	7	0.06	34	7.4
PS	57	M	8	1	20.5	3.9	159	1.61	0.8	119.2	1	high		6.3	0.21	23	10.6
PS	56	M	8	1	23.3	4.1		1.74	0.78	111.7	2.1	high	9.2	4.7	0.2	37	11.8
PS	73	M	7	1	23.8	3.6	118	1.5	0.9	114.6	1.5	high	9.2	5		18	10.6
PS	79	M	7	1	19.7	3.4	111	1.1	1	125.2	0.9	high	8.1	4.2	3.9	27	10.1
PS	64	F	5	1	21.5	2.6	231	2.03	0.53	86.83	1.5	high	8.6	7	0.18	27	13.5
PS	54	F	5	1	19	3.7	156	1.92	0.72	94.78	1.2	high	10.1	4.4	0.1	30	11.3
PS	31	M	4	1	23.6	3.3	169	2.15	0.85	75.97	2.5	high	9	3.8	0.01	29	11
PS	66	M	4	1	16.8	3.7	105	1.06	0.73	89.9	2.1	high	9.8	3.9	0.4	32	12.7
PS	52	M	3	1	19.3	3.8	186	1.7	0.97	121.1	1.6	high	9.7	7.4	0.4	24	10.7
PS	83	M	2	1	22.1	3.8	132	2.3	0.71	94.81	1.9	high	10	6.1	0.15		15
PS	57	M	31	1	16.5		161	1.01	1.03	24.96	1.2	high	8.9	8.3		20	10.7
PS	76	M	5	1	20.5	3.5	147	1.72	0.92	112.3	1.8	high	9.5	5.4			9.4
PS	83	M	4	1	23.3	3.9	163	1.04	0.73	87.86	0.8	high	8.1	5.1	0.77	24	11.3
PS	72	M	3	1	19.4	3.4		1.42	0.97	73.44	1.5	high	9.4	5.8	2.62	30	11.6

PS	69	M	2	1	16.4	3.8	221	0.92	0.91	75.85	1.8	high	9.2	5.4	0.26	23	11
PS	65	M	5	1	19.8	3.8	161	1.63	0.97	78.61	1.8	high	9.2	3.3	0.37		10
PS	58	M	4	1	24	3.7	175	1.13	0.59	78.76	1.6	high	8.4	3.2	0.41	26	9.7
PS	53	F	13	1	20	3.6	142	1.12	1.08	119.9	2.1	high		4.9	0.03	28	9.8
PS	47	F	7	1	29.3	3.5		1.26	0.98		1.5	high	9.1	5.6	0.12	22	10.5
PS	71	F	10	1	24.9	3.9	128	1.36	0.72	127.8	1.5	high	9.3	4.9	0.05	26	8.8
PS	52	M	18	1	15.9	3.3	112	0.84	0.77	111.5	1	high		2.9	2.09		8
PS	46	M	6	1	31.3	3.8	171	1.14	0.78	105.1	1.6	high		8.4	0.06		9.1
PS	75	F	5	1	23	3.6		1.09	0.84	102.2	1.8	high	8.8	6.6		19	10.8
PS	73	F	4	1	22	3.6	191	1.59	1.04	156.2	1	high	9.6	4.8		25	10.2
PS	58	M	4	1	20.5	4	118	1.5	1.27	102.6	2.1	high	8	3	0.36	28	7.3
PS	62	F	3	1	21.4	4.1	132	0.98	0.97	124.4	1.6	high		4.6	0.05		9.3
PS	76	M	3	1	20.1	4	219	1.51	0.92	95.85	1.8	high	8.8	3.7			9.7
PS	33	M	2	1	29.3	4.9	189	0.89	1.21	104.6	1.8	high	9	7.6	0.7	35	10.4
PS	61	M	2	1	16.2	4.1	138	1.61	0.78	55.84	1.8	high	9.5	2.8	0.11	24	11
PS	78	F	4	1	21.9	3.3	173	1.46	0.89	79.22	1.8	high		6.8			9.4
PS	68	M	4	1	21.5	3.8	112	1.29	0.68	84.75	1.6	high		4.4	0.02		8.2
PS	54	M	19	1	16.8	3.5	203	1.83	0.77	88.13	1.6	high		4.6	0.25	28	10.8
PS	56	F	20	1	24	4	108	1.26	1.19	126.1	2.2	high		5.8	0.11	25	10.9
PS	47	F	21	1	13.4	3.7	200	1.26	0.78	43.5	2.2	high	9	4.1			
PS	34	M	6	1	23.7	3.9	141	1.86	1.11	37.76	1.9	high	8.9	4	0.25		9.8
PS	65	M	2	1	19.7	4	159	2.06	0.9	92.23	1.6	high	9.5	3.6	0.16		9.9
PS	75	M	5	1	19.9	3.3	130	1.39	0.7	101.5	1.8	high	8.9	6	0.16	28	11.3
PS	74	M	14	1	17.2	3.9	131	1.25	0.94	102.2	1.6	high	9	4.2	1.05		9.5
PS	60	M	4	1	21.3	3.8	96	1.65	0.84	115.8	2.1	high		4.7	0.65	28	10.5
PS	74	F	3	1	17	3.7	160	1.34	0.69	85.97	1.8	high	9.9	6.8	0.06	36	10.7
PS	82	F	2	1	17.7	3.5	96	1.47	0.84	87.63	1.8	high	9.3	3.8	0.01	29	11.6
PS	64	M	4	1	22.7	3.4	137	0.99	1	99.9	2.1	high	9.1	7	0.71	31	12.6
PS	53	F	6	1	16.9	4.1	185	1.46	1.26	91.55	1.3	high	10.1	4.7	0.08	27	13.1
PS	75	M	30	1	19.6	3.4	130	1.06	1	126	2.1	high	9.9	6.2	0.4		10.7
PS	49	F	17	1	16	4.2	103	1.48	0.89	111.1	1.5	high	8	4.2	2.6		10.3
PS	73	F	2	1	15.8	2.8	132	1.5	0.81	46.06	2.1	high	9.4	6.1	0.7	26	11.4
PS	64	F	20	1	20.2	3.7	187	1.38	0.9	112.7	1.6	high	10.1	5		10	10.2
PS	69	M	13	1	23.8	3.6	170	1.1	1	89.05	2.1	high		6.6	0.72		9.5
PS	39	M	5	1	22.2	4.1	151	1.45	0.94	118.2	1.9	high	8.8	7.2	0.1	32	12.1
PS	62	F	4	1	18	4	110	1.71	0.98	124.1	1.9	high	9.5	3.6	0.2		8.6
PS	59	F	15	1	23	3.6	125	1.39	0.77	101.3	1.3	high	9.5	6.6	4.9	31	10.3
PS	53	M	4	1	23.3	4		1.27	0.98	97.18	1.8	high	8.4	5.3	0.55	46	9.4
PS	69	M	5	1	18.4	3.4	146	1.3	1.01	123.6	1.9	high	9.7	6.1	0.56		11.6
PS	58	F	25	1	20.5	4.2	131	1.86	0.89	94.43	1.6	high	9.8	5.6		35	10.5
PS	67	F	30	1	13.9	3.7	139	1.78	0.99	98.66	1.6	high	9.8	5.2	0.05		10.6
PS	71	M	2	1	24.7	3.8	143	1.03	0.57	30.23	1.5	high		5	0.2	23	10.8
PS	68	M	5	1	24.7	3.9		1.15	0.65	90.21	1.5	high	9.9	5.1	0	25	10.1
PS	61	M	11	1	18.9	4.3	152	1.45	0.93	115.8	1.4	high	8.5	5.4		26	11.7
PS	53	M	12	1	17.8	4.4	145	0.81	0.98	103.7	2.1	high	9.8	6.2	0.45	31	12.4
PS	70	M	2	1	19.6	3.7	144	1.26	0.83	70.71	1.5	high		2.9	0.05	27	10.1
PS	65	M	2	1	24.8	4.3		1.17	0.9		1.5	high	7.1	6.6		32	13.6
PS	59	M	4	1	19.8	4.1	153	1.57	0.76	101.1	1.5	high	9.6	5.2	0.3	33	13.2
PS	64	F	14	1	20.9	3.8	249	1.82	1.02	114.1	0.8	high	9.3	4.9	0.06	32	10.1
PS	74	F	8	1	16.9	4.2	123	1.8	0.91	123.2	1.3	high	9.9	4	0.1	18	10.5
PS	69	M	3	1	23.5	4.2	118	1.62	0.74	71.3	2.1	high	10.1	4.9	0.4	26	10.5
PS	58	M	3	1	17.4	4.2	99	1.57	0.85	72.52	1.5	high	7.7	4.8		26	10.1
PS	64	M	9	1	21.8	3.9	95	1.07	0.85	89.55	1.5	high		3.6	0.1	25	10.2
PS	74	M	11	1	17.2	3.6	184	2.12	1.04	89.85	1.6	high	9.7	4.5	0.63		10.9
PS	59	F	7	1	18.1	4	146	1.42	0.99	114.9	1.6	high	8.3	4.7	0.05		10.7
PS	59	F	16	1	19.9	3.9	145	1.55	1.07	137.3	2.1	high	9.5	5.5	0.09	22	10.2
PS	39	M	2	1	19.6	4.1	120	1.35	0.8	66.8	1.5	high	9	6.4	0.05	28	11.5
PS	59	M	25	1	18.2	3.7	211	1.33	0.84	82.67	1.9	high	9.6	4	0.21		12
PS	58	M	2	1	18.4	4.2		1.69	0.82	64.06	1.3	high	8.8	4.9	0.48	30	10.4

PS	78	M	10	1	18.7	4.2	88	1.43	0.83	93.12	1.6	high		5.8	0.16	22	7.9
PS	72	F	2	1	25.5	3.5	118	1.53	0.87	82.56	2.1	high	9.4	2.9	1.4	45	10.1
PS	65	F	7	1	19.7	4	114	1.78	0.96	90.29	2.1	high	9.1	6.8			10.8
PS	53	M	6	1	25.2	3.8	122	1.27	0.99	111.4	2.1	high	8.9	6	0.04	28	6.2
PS	60	M	4	1	23	3.9	158	1.13	0.89	126.7	2.1	high	8.1	5		29	10.8
PS	63	M	11	1	16.2	4.5	217	1.25	1.26	109	1.5	high	8	4.9	0.12	23	11.2
PS	62	M	14	1	24.4	4	148	2.34	0.85	113.9	1.3	high	9.5	3.4	0.01	26	10.4
PS	60	M	2	1	27.8	4.1	176	1.61	0.84	75.08	1.8	high	9.5	4.5	0.3		10.5
PS	50	F	9	1	16.8	4.2	172	1.17	1.08	112.4	1.5	high	10.1	5	0.31	17	10.8
PS	64	M	6	1	21.6	3.9	201	1.54	0.87	97.23	1.5	high	9.3	5.8	0.02	28	10.4
PS	63	F	14	1	19.6	4.1	118	1.88	0.95	133.4	1.8	high	8.9	2.1	1.42	28	9.6
PS	47	F	8	1	18.1	4.2	171	1.73	1.5	109.7	1.8	high	9.4	5.5	0.02	27	9.8
PS	64	M	7	1	22.5	4.3	145	1.19	0.97	120.8	2.1	high	9	7.1	0.2	28	11.8
PS	53	M	2	1	26.9	4.2	166	2.16	1	89.13	1.5	high		6.1	0.4	28	8.7
PS	61	M	3	1	25.4	4	168	1.26	0.81	53.3	1.8	high	9	4.5		37	10.1
PS	54	M	16	1	22.9	4.2	176	1.39	0.92	120.6	2.1	high	9.7	5.6	0.05	24	10.3
PS	56	M	14	1	17	4.3	205	1.45	1.34	127	1.5	high	8.8	6.9	0.02	24	10.6
PS	51	F	12	1	19.6	4.2	204	1.46	1.24	115	1.5	high	8.6	6.6	0.07	30	10.7
PS	59	M	3	1	18.4	3.8		1.75	1.08	62.05	1.5	high	10	6.8		25	12
PS	63	M	34	1	18.3	3.4	146	1.48	1.21	98.27	1.8	high	9.9	5.1	0.82	31	10.9
PS	63	F	10	1	23.5	4	110	1.59	1.07	110.5	1.5	high	8.4	5			9.4
PS	68	F	5	1	22.9	3.9	170	1.74	0.88	113.4	1.8	high	8.7	4.4	0.22	19	9.5
PS	54	M	15	1	18.8	3.2	144	1.23	0.65	49.79	1.5	high	8.8	2.7	2.09	39	10.9
PS	65	F	22	1	17.9	3.5	111	1.65	1.21	119.4	1.8	high	9.6	5.3	2.5	24	8
PS	56	F	13	1	22.1	4.1	168	1.52	1.02	148.1	2.5	high	9.5	6		23	11.1
PS	56	F	2	1	34.6	3.7	132	1.21	0.6	72.38	2.5	high	9.6	5.2	0.05	27	11.3
PS	42	F	2	1	22.8	3.8	180	1.28	0.89	94.99	1.6	high	11.9	8.6	0.2	31	12.7
PS	63	M	2	1	21.4	4	169	1.8	0.77	102.1	1.1	high	9.6	4.3			8.8
PS	71	F	14	1	20.2	3.5	127	1.51	0.49	112.9	1.5	high	9	4.8			9.6
PS	54	M	2	1	20.6	3.6	129	1.13	0.91	98.23	2.1	high	10.2	4.2	0.2		10.7
PS	52	F	4	1	23	3.9	161	1.27	0.92	83.6	2.1	high	8.8	5.1	0.47	30	9.7
PS	71	F	7	1	25.4	4	134	1.4	0.73	93.47	1.8	high	10	7.2	0.17	29	10.3
PS	59	M	4	1	25	3.7	131	1.05	0.95	110.8	2.1	high	7.5	6	0.05	34	8.8
PS	56	M	7	1	22.1	4	175	1.48	0.72	118.3	1.8	high	9.3	5.9			10.5
PS	50	F	4	1	17.9	4.3	158	1.39	0.85	86.6	1.8	high	8.9	5.5			9.2
PS	79	M	2	1	20.8	3.5	167	1.25	0.77	73.36	2.1	high		7.8	0.1	30	11
PS	74	F	6	1	23.4	3.8	105	1.56	0.71	133	1.4	high	9	4.2		42	10.1
PS	22	M	5	1	22.1	4.2	157	1.68	1.01	98.87	2.1	high	8.4	4.6			10.7
PS	80	M	4	1	20.3	3.7	167	1.39	0.86	114.9	2.1	high	8.5	4.7	2.03	27	9.6
PS	57	M	3	1	20.4	4	96	1.45	0.83	91.19	1.8	high	9.6	7.1	0.18	30	10.2
PS	60	M	11	1	19.9	3.3	207	1.68	0.72	117.8	1.3	high		6.2	0.04	30	10.5
PS	43	F	10	1	19.6	3.9	182	1.84	0.95	103.7	1.6	high	9.5	6.5	1.88		9.8
PS	35	M	9	1	22.1	4.1	142	1.08	0.86	99.19	2.1	high	8.7	5.3		16	12.2
PS	54	F	4	1	22.9	4.1	145	1.03	0.97	39.83	2.1	high	8.2	6.2	0.2	21	10.3
PS	77	F	3	1	25.5	3.5	166	1.88	0.75	85.35	1.6	high	9	5.7	0.05		10.6
PS	67	M	2	1	22.3	3.9	131	1.35	0.83	109.1	1.8	high		5.3	0.04	26	9.7
PS	69	M	12	1	22	4.1	150	2.15	1.12	120.8	1.6	high	10.1	4.4	0.05		9.6
PS	56	M	12	1	17.8	4.6	140	1.32	1.03	84.09	1.9	high	9.6	4.6	0.53		9.9
PS	82	F	9	1	19.1	3.5	153	2.14	0.79		1.5	high	7.9	5.2	0.47	29	9.2
PS	77	M	8	1	18.6	4	129	1.67	0.91	126.2	1.1	high	7.3	4.8		28	8.7
PS	77	M	4	1	22	3.7	137	1.55	0.88	91.61	2.1	high	8.4	6	0.09	27	11.3
PS	80	F	2	1	18.3	4.3	133	1.56	1.03	107.1	1.9	high	9.7	8.2	0.05		10.5
PS	67	M	3	1	20.5	3.9	225	1.76	0.98	99.24	1.6	high		2.9		18	10.2
PS	64	F	3	1	20.1	4	119	1.17	0.95	77.36	1.3	high	8	4.4	0.18	24	11.1
PS	71	M	3	1	22.7	4	187	1.1	1.28	115.2	1.6	high		3.8		24	8.9
PS	81	M	3	1	25.7	3.5	147	1.67	1.02	145.5	1.8	high		6		27	8.9
PS	76	M	4	1	18.9	3.6		1.74	1.02	88.29	1.5	high	7.8	6.6	1.3	29	11.5
PS	73	M	3	1	18.7	4.1	164	1.06	1.01	111.7	1.5	high	9.1	4.6	0.1	31	9.8
PS	57	M	4	1	23.4	3.7	151	1.25	0.73	107.1	1.8	high		4.5		28	10.5

PS	56	M	5	1	29.3	4.1	139	1.67	0.84	84.97	1.6	high		4		31	10.1
PS	73	M	4	1	22.2	4	165	0.99	0.83	95.77	1.8	high	9.1	3.7	0.39	27	8.9
PS	34	M	3	1	23.2	4	159	1.26	0.78	113.6	1.6	high	8.8	6	0.07		11
PS	62	M	3	1	26.6	4.1	186	1.5	0.79	83.24	1.3	high	9.5	7.2	0.3	29	9.8
PS	68	F	9	1	18.9	3.4	188	1.58	0.99	117.6	1.5	high	9.4	7.1	0.09	27	10.4
PS	49	M	2	1	21.5	4.2	164	1.15	0.97	39.43	2.1	high	9.6	5.8	0.08	23	13.2
PS	62	M	2	1	20	3.8	139	1.08	0.87	95.52	1.1	high	9.2	3.9	1.04		8.7
PS	44	F	7	1	19.9	4	132	1.3	0.85	124.2	1.5	high	10.7	6.3		18	10.1
PS	66	M	2	1	19.3	4.2	156	1.02	0.92	107.6	2.1	high	6.5				8.3
PS	72	M	2	1	26	3.8	153	1.62	0.76	118.1	1.8	high	9.4	3.3	0.05	34	10.2
PS	73	M	2	1	20.1	3.8		1.41	0.86	100.7	1.8	high	9.4	4.3	0.89		8.4
PS	75	M	2	1	20.3	3.8	181	1.21	0.68	99.24	1.8	high		4.1		22	11.9
PS	65	M	6	1	20.7	3.8	86	1.3	0.87	134.2	2.1	high	8.3	4.4	4.16	41	9.2
PS	52	F	21	1	17.6	3.6	158	1.5	0.93	146.8	1.6	high	9.5	4.7	0.07		10.8
PS	54	M	2	1	19.6	4.1	164	1.48	0.86	77.89	1.8	high	8.3	3			9.8
PS	57	F	7	1	23.3	3.8	131	1.46	0.91	106.2	1.9	high	8.8	4.9	0.05		11.5
PS	58	M	14	1	22.1	4.1	150	1.45	1	96.8	1.8	high	9.1	6.3	0.1	32	10.1
PS	65	M	34	1	17.5	3.7	175	1.21	1.11	109.4	1.6	high	9.6	5.2	0.3		11.8
PS	50	F	32	1	15.3	4.3	179	1.66	0.88	63.54	1.6	high	9.6	5.7	0.76		11.7
PS	64	M	28	1	19.1	3.6	183	1.41	0.84	123.4	1.4	high	9	6.1		20	10.9
PS	59	F	23	1	17	3.9	131	1.91	0.94	86.99	1.6	high	10	4.7	0.43		10.9
PS	42	F	14	1	25.4	3.9	224	1.4	0.92	111.8	2.1	high	10	6.6	0.48	37	10.6
PS	40	M	13	1	17.1	3.8	180	1.64	1.09	84.51	1.6	high	8.5	4.1	0	24	10.2
PS	60	F	11	1	28.8	4.1	169	1.73	0.68	67.92	1.9	high	10.5	5.1	0.18		11.4
PS	66	F	10	1	19.3	4.2	175	1.15	0.88	121.2	2.1	high	9.2	6.4	0.06	22	10
PS	71	M	8	1	18.7	4.1	141	1.49	0.91	117.8	1	high	10.7	3.8		23	10
PS	64	M	7	1	25.2	4	178	1.89	0.94	116.9	1.6	high	10.1	5.8	0.05		10.3
PS	59	M	7	1	15	4.4	143	1.54	0.88	102.8	1	high	8.7	4.1		43	10.4
PS	64	M	6	1	20.1	3.9	288	1.4	1.12	126.8	1.6	high	10.1	6.7	1.01		9.8
PS	58	M	5	1	19.5	3.9	146	1.79	0.75	108.6	1.3	high	10.2	5.6	1.1	16	10.8
PS	34	F	4	1	16.8	3.7	214	1.72	0.98	111	1.5	high	9.8	6.6			9.9
PS	48	M	4	1	30.4	4.1	168	1.87	1	80.86	2.1	high	8.7	4.3		26	11.2
PS	65	M	4	1	19.5	3.5	117	1.48	0.98	45.29	1.6	high	9.6	4.6	0.41		10.1
PS	69	F	4	1	22.7	3.8	172	1.59	0.84	89.18	1.5	high	8.6	3.9	0.14	28	11.8
PS	29	M	3	1	26.6	4.1	127	1.74	0.82	60.57	1.9	high	8.7	5.3	0.05		10.3
PS	56	F	2	1	17.2	3.1	123	1.53	0.62	54.04	1.6	high		4.1	0.22	27	10.4
PS	57	M	2	1	29.4	4	117	1.67	0.78	30.75	2.1	high	9.1	6.4	0.1		9.8
PS	71	F	2	1	20.2	4	136	1.41	0.87	99.23	2.1	high	7.4	4.1	0.21	34	8.7
PS	79	M	2	1	21.2	2.9	108	1.03	0.78	93.66	1.3	high	10	3.6	14.1		10.9
PS	67	M	14	1	22		161	1.27	1	110.5	2.1	high		5.8	0.1	22	12
PS	35	F	2	1	21.9	4	134	1.61	1.1	95.97	1.9	high	9.6	4.8	0.14		12.5
PS	75	F	15	1	16.7	3.2	126	1.25	0.55	86.83	1.8	high	10.1	4	0.42	33	8.6
PS	66	M	9	1	20.5	4.1	180	1.64	0.76	77.36	0.8	high	8.5	4.1	0.1	30	10
PS	80	M	9	1	22.9	3.7	131	1.61	0.74	46.02	2.5	high	8.9	6.9	0.05	33	8.7
PS	82	F	9	1	18.2	3.6	163	1.4	0.75	60.2	1.8	high	9.1	4	0.1	19	
PS	83	F	5	1	23.4	3.5	234	1.04	0.92	122.8	1.8	high	8.8	7.6		25	10.2
PS	84	F	4	1	17.5	3	148	1.16	0.6	61.65	2.1	high	10.4	5.8	0.1		10.3
PS	61	M	29	1	22.8	3.1	157	1.19	0.75	84.05	1	high	9	4.3		29	10.7
PS	67	F	21	1	21.1	3.5	129	1.72	0.98	135.8	1.4	high	11.5	4.4		32	9.9
PS	62	M	20	1	19.2	3.7	90	1.17	0.96	86.97	1.4	high	10.3	4.7		43	10.4
PS	71	M	15	1	17	3	146	1.45	0.67	48.37	1.4	high	9.7	4.8	0.37	30	12
PS	72	F	14	1	17.3	4	115	1.36	0.97	115	1.6	high	8.6	3.4	0.12		11.6
PS	66	M	13	1	21.8	3.4	111	1.58	0.72	80.67	1.8	high	8.2	4.5	0.06	27	11.5
PS	69	M	12	1	19.3	3.5	133	1.54	0.64	95.57	1.5	high	9	5.8	0.1	33	11.4
PS	77	F	12	1	27.1	3.3		1.34	0.58	88.32	2.2	high	10.4	4.8	0.1		10.2
PS	62	F	11	1	19.4	3.4		1.4	0.76	54.07	2.2	high	10.2	4	0.1	28	9.8
PS	65	M	10	1	23.4	3.6	139	1.42	1.18	103.1	1.6	high	9.5	3.9			10.2
PS	69	M	10	1	20.5	4	232	1.53	1.08	122.1	1.3	high	8.9	6.1	0.08	28	10.7
PS	72	M	9	1	22.3	3.6		1.38	0.76	160.3	2.2	high	8.7	5	0.1	25	10.4

PS	56	F	9	1	23.3	4.2	154	1.52	0.92	135.1	2.1	high		7.1	0.17	23	10
PS	40	F	9	1	19.5	4.1	130	1.13	0.68	109.4	1.6	high		3.4		16	10.6
PS	45	M	9	1	20.1	4.4	153	1.58	0.87	94.3	2.1	high		4.2	0.03	25	10.1
PS	67	F	8	1	27	3.4	114	1.32	0.58	101.6	1.3	high	8.2	4.7	1.24	31	9
PS	82	F	7	1	14.1	3.8	126	1.24	1.46	125.5	1.6	high		6.7		32	11.2
PS	48	M	6	1	24.4	4.2	121	1.67	0.8	114.3	1.8	high	9.1	4	0.2	32	9
PS	88	F	6	1	16.1	2.1	108	1.36	1.13	43.77	1.5	high	9.4	4.5		27	10.6
PS	42	M	6	1	22.9	4.1		1.23	0.9	108.6	2.1	high	8.8	6.1	0.4	19	12.6
PS	55	M	6	1	20.9	4.1	129	1.38	1.03	76.81	1.5	high		6.9	4	29	9.9
PS	68	F	5	1	21.6	3.4	160	1.36	0.64	60.48	2.2	high	9.5	7.8			13
PS	62	M	5	1	19.1	3.9	127	1.25	1.01	130.3	1.4	high	9.8	6			10.7
PS	67	M	5	1	16.4	4.1	133	1.49	0.81	45.15	2.1	high	10.1	5.7	0.03	42	11.8
PS	63	F	4	1	20.4	3.9	131	1.61	0.8	82.57	1.8	high	7.6	1.5	0.69	38	11.6
PS	69	M	4	1	20	3.9	144	1.42	0.95	116.2	1.3	high	9.6	5.2	0.9		9.3
PS	65	M	4	1	21.1	4.1	135	1.46	0.79	101.8	1.3	high	8.6	5.8	0.06	27	11.5
PS	60	F	3	1	14.2	3.9	152	1.45	1.25	76.15	1.8	high	9.9	5.6			9.6
PS	67	M	3	1	22.1	2.6	141	1.63	0.93	149.5	1.4	high	9.6	3.7			12.1
PS	86	F	3	1	19.2	2.3	171	1.54	0.4	40.88	2.1	high	7.5	7.3	3.28	32	10.5
PS	52	M	2	1	29.5	3.6	137	1.67	0.59	101.9	1.5	high	7.9	6.3	1.12	25	10.7
PS	24	M	2	1	16.9	4.2	129	1.2	1	92.48	1.4	high	10.1	5.7			10.1
PS	78	M	8	1	18.5	3.6	120	1.64	0.78	81.38	1.6	high	8.4	4	0.69	41	11.4
PS	69	M	2	1	23.2	4.2	164	1.34	0.72	86.21	1.5	high	9.3	4.5	0.05	25	10.7
PS	64	F	11	1	16.1	3.6	160	1.04	0.86	77.65	2.1	high		4.6	0.43	29	10.2
PS	73	M	18	1	17	3.9	144	1.4	0.88	111.4	1.9	high	10.1	5	0.45		9.7
PS	80	M	5	1	17.7	3.5	169	1.7	0.78	106.8	1.6	high	9.3	4.3	0.05		9.7
PS	81	F	11	1	19.1	3.9	111	1.51	1.11	112.4	1.3	high	8.6	4.4	0.05		10.4
PS	62	M	5	1	25.3	4	148	1.69	1.1	69.24	1.5	high	8.6	6.2		40	11.9
PS	49	F	35	1	19.7	3.5	156	1.64	1.41	97.75	1.6	high	10.6	6.8			10.6
PS	59	M	7	1	18.5	4.1	216	1.11	0.84	110	1.8	high		6.3	0.58	24	9.6
PS	62	F	6	1	21.3	3.6	166	1.82	0.87	82.88	1.8	high	9.1	8.7	1.8	34	10.3
PS	65	F	5	1	20.4	3.8	163	1.06	0.91	78.11	1.6	high	9	4.7	0.05		10.3
PS	57	M	4	1	19.1	4.2	97	1.62	0.74	119.8	2.1	high	9	6		25	10.1
PS	65	M	5	1	22.1	3.9	177	1.62	0.77	99.14	1.8	high	10.4	4.7	1.12	28	9.3
PS	37	M	12	1	22.3	4.3		1.69	0.8	97.38	1.5	high	8.8	8.1	1.99	27	9.4
PS	53	F	6	1	19.9	4.1	136	1.71	0.88	120.7	1.8	high	8.3	6.8	1.41	31	10.5
PS	68	M	5	1	20.4	4	155	1.17	1.06	121.4	1.1	high	7.3	4.9	0.61	33	13
PS	64	F	3	1	19.4	3.7	107	1.12	1.04	122.8	1.6	high	8	5	1.1	24	9
PS	75	M	7	1	19	3.3	122	1.71	0.86	86.5	1.1	high	9	3.9	0.6	25	10.8
PS	49	M	10	1	17.8	4.1		1.08	0.99	86.83	2.1	high	8.5	5.9	0.1		10.1
PS	68	M	11	1	17.4	4	218	1.24	0.95	103	1.4	high	9.1	7.5			13.8
PS	66	M	13	1	22.9	4.2	132	1.3	0.9	114.3	1.5	high	9.2	6.8	0.03	34	9.7
PS	65	M	6	1	19.2	3.9	126	1.43	0.92	127	1.8	high	7.9	5.9	0.13	38	9.1
PS	77	M	2	1	19.8	3.6	188	1.61	0.57	31.2	1.6	high	10.3	3.9	0.03	27	11.2
PS	79	M	2	1	19.9	3.8	167	1.44	1.23	85.38	1.6	high	10.1	5.2	0.05		11.9
PS	54	M	3	1	26.6	4.1	159	1.3	0.57	125.6	1.5	high	8.5	6.2	0.04	29	13
PS	82	F	4	1	19.8	3.6	119	1.68	1.12	132.5	1.5	high	10.2	4.9	0.07		10.2
PS	76	M	4	1	25.4	3.5	151	1.44	1	113.4	1.9	high	9	4.9	0.05		11.3
PS	76	M	2	1	27.1	3.9	132	1.33	0.83	88.82	1.8	high	7.9	3.8	0.1		10.6
PS	49	F	5	1	22.4	3.8	145	1.31	0.77	80.17	1.6	high	8.7	6.3	0.41		8.5
PS	69	M	3	1	20.5	4.2	169	1.13	0.93	99.22	1.6	high	9.8	4.7	0.1	29	10.1
PS	67	F	15	1	16.9	3.7	164	1.36	0.81	121.5	1.8	high	9.3	5.8			7.7
PS	60	F	12	1	21.9	4.2	191	1.54	0.82	126.9	1.6	high	9.6	5.8	0.05		10.6
PS	77	M	9	1	22.9	3.9	143	1.43	0.88	114	1.6	high	9.1	4.7	0.08		10.7
PS	72	F	9	1	16.9	4.1	241	1.57	0.97	125.1	1.5	high	9.3	4.1		25	9.6
PS	72	M	9	1	21.1	4.1	207	1.67	0.91	109.9	1.6	high	9.2	5	0.08		10.6
PS	68	M	8	1	21.3	4.4	146	1.61	1.11	116.2	2.1	high	10.3	4.7	0.39	21	9.9
PS	69	M	9	1	17.6	4	157	1.85	0.79	107.1	1.5	high	9.2	5.7	1.42	27	10.6
PS	72	M	8	1	23	3.9	161	1.29	0.85	135.7	1.3	high	10	2.3	0.05	30	9.1
PS	66	M	7	1	21	4.2	195	1.15	0.8	125.6	2.5	high	9.3	10.9	0.49	27	10.6

PS	66	M	7	1	20.4	3.4		1.5	0.96	108.6	1.1	high	9.6	1.7		30	8.4
PS	76	F	6	1	16.8	4.3	119	1.32	0.95	108.4	1.6	high	8.7	5.8	0.07		10.1
PS	70	M	3	1	20.4	3.7	186	1.48	0.96	132.4	1.6	high	9.8	4	0.05		9.9
PS	50	M	7	1	20.6	4.3	132	1.37	0.77	93.73	1.5	high	8.5	5.3			9.7
PS	82	M	5	1	22.5	3.9	166	1.43	0.85	123.3	1.9	high	9.1	5.9	0.05		9.9
PS	65	M	4	1	23.6	3.7	174	1.94	0.93	92.32	0.8	high		2.2			10.2
PS	73	M	4	1	19.9	3.7	157	1.26	0.8	146.1	1.5	high	10	5.4			9.9
PS	73	F	4	1	30	3.7	178	1.4	1	105.1	2.1	high		4.8	0.13	25	9.8
PS	67	F	20	1	22.4	3.6	182	1.19	0.84	86.39	1.3	high	9.3	3.1			9
PS	53	M	3	1	24.3	4	135	0.81	0.92	155.1	1.5	high	9.8	5.3	0.7	24	9
PS	65	M	8	1	23.6	3.9	147	1.69	0.77	143.6	1.6	high	10.2	4.7	0.05		11
PS	63	F	21	1	18.7	3.8	167	1.34	1.1	112.4	2.1	high	9.7	7.1			12.7
PS	40	M	15	1	17	4.5	137	1.43	1.16	99.42	2.5	high	8.8	6.6			11.8
PS	60	M	13	1	21.1	4	172	1.52	0.68	115.6	1.8	high		3.2		21	7.8
PS	64	M	12	1	20.9	4.1	194	1.47	0.78	117.4	2.1	high	9.2	6.9		30	12.5
PS	71	M	7	1	18	3.8	147	1.61	0.94	105.3	2	high	8.5	6		20	9.7
PS	74	M	13	1	21.8	4	224	1.23	0.64	128.2	2.1	high	9.3	6	0.17	27	11.3
PS	39	M	5	1	28.8	4.2	165	1.47	0.79	96.81	1.8	high	9.2	8.1	0.3	31	11.3
PS	73	M	3	1	18.4	3.6	164	1.88	0.85	116.9	1.8	high	8.2	3.6	0.05		11.3
PS	52	M	5	1	29.8	3.9	144	1.58	0.84	102.8	2.1	high	9.2	6.3	0.06	23	9.8
PS	53	F	22	1	20.9	4.1		1.12	1.04	111.4	2.1	high	8.8	5.9		25	9.2
PS	61	F	4	1	19.2	3.8	192	1.49	1	110.1	1.5	high	9.1	5.6			10
PS	62	F	4	1	25.6	3.7	188	1.33	0.92	125.1	1.8	high	9.6	5.5	1.01	18	9.8
PS	40	F	8	1	24	4.5	107	1.45	0.82	103.1	2.1	high	8.8	6			10.4
PS	62	M	6	1	23.4	3.7		1.13	0.87	110.9	2.1	high	9.5	6.5	0	26	12.2
PS	65	M	4	1	23.4	4	151	1.23	0.8	124	1.5	high	8.4	4.7	0.67	23	11
PS	50	M	5	1	27.6	4	120	1.38	0.85	105.4	1.3	high	8	4.5	0.43	26	9.5
PS	57	F	11	1	23.4	4.1	179	1.57	1.05	113.6	1.6	high	9.1	5.2	0.15		10.3
PS	67	M	3	1	19.1	3.2		0.92	0.8		1.6	high		2.8			7
PS	50	M	24	1	21.2	3.9		1.14	0.81		2.1	high	8.2	4.6	0.3	23	7.7
PS	62	M	4	1	26.8	3.7	158	1.48	0.8		1.6	high	10.7	5.4	0.04	27	8.5
PS	58	F	18	1	21.3	4	188	1.4	0.68		1.6	high	8.9	5.3	0.08	25	10.9
PS	51	F	31	1	16.6	3.9	122	1.49	1.06		2.1	high	9.6	5.9	0.3	32	7.5
PS	61	M	2	1	24.1	3.8	151	1.58	0.87		1.5	high	7.8	3.9	0.25	20	9.8
PS	64	M	5	1	24.4	3.8	157	1.17	1.06		1.8	high	9	5.1	0.04	20	11.7
PS	70	M	5	1	21.9	3.8	135	1.03	0.69		2.1	high		5.2			11
PS	33	F	2	1	25	4.2		1.73	0.8		1.6	high		4.2	11.1	48	8.9
PS	57	M	4	1	29.7	3.7	124	1.53	0.74		1.3	high	9.3	5	0.08	36	10.6
PS	59	M	4	1	21.1	4	146	1.63	0.68		1.8	high	9.3	6	0	19	10.7
PS	70	M	2	1	22.5	3.9	70	1.76	0.79			high		8.3	0.26		11.6
PS	51	M	3	1	19.4	4.4	107	1.63	1.08		1.8	high	9.1	7.1	0.2	25	9.6
PS	58	M	24	1	25.1	3.6	184	1.4	0.76	117	1.8	high		3.7	0.12		10
PS	58	F	14	1	18.7		135	1.22	1.26	96.1	1.6	high	9	5.7	0.49		10.9
PS	67	F	14	1	19.5	3.5	124	1.61	1.2	82.06	2.5	high	9.4	5.7	0	37	9.9
PS	69	M	11	1	18.8	3.7	126	1.47	1.12	129	1.6	high	9	5.6	0.09		9.4
PS	67	M	5	1	22.9	4.1	115	1.28	0.78	122.4	1.8	high	7.7	7.9		38	11.2
PS	60	M	5	1	19.5	4	119	1.21	0.94	130.7	1.9	high	8.4	3.7	0.05		10.5
PS	59	M	33	1	22.9	3.7	182	1.79	1.17	93.83	1.4	high	8.5	4.6	0	24	10.4
PS	49	M	28	1	16.3	2.9	101	1.4	0.73	77.91	1.8	high	9.6	7.5	0		11.4
PS	58	F	25	1	16.1	3.3	122	2.85	0.8	72.99	1.6	high	9.7	5.1		16	9.8
PS	66	F	24	1	26.5	4.2	243	1.8	0.87	112.7	1.6	high	12.2	5.8		21	11.6
PS	63	F	24	1	15.9	3.4	152	1.32	0.73	97.28	1.6	high	9.3	4.4	0.18		9.3
PS	59	F	19	1	20.9	4		2.41	1.3	91.61	1.6	high	8.6	4.8	0.04	25	10.4
PS	63	F	19	1	17.1	3.2	174	1.81	1.05	104.6	2	high	8.4	6.5	0.02	21	
PS	62	F	18	1	20.1	3.9	216	1.15	1.25	119.5	1.5	high		6.5	0.01	35	10.4
PS	62	M	17	1	23.2	3.7	131	1.36	0.83	112.9	1	high	9.3	4.8	1.3		7
PS	69	F	16	1	21.3	2.9	84	1.42	0.97	92.51	1.8	high	9.8	4.8	0.26	33	10.9
PS	58	M	15	1	25.3	4	136	1.29	0.94	123.8	1.9	high	10	5	0.02	17	10.2
PS	71	F	15	1	19.2	3.5	135	1.65	1.41	136.6	1.9	high	9.7	5.7	0.02	24	10.7

PS	72	F	14	1	20.7	3.4	129	1.05	1.12	107.8	1.9	high	9.9	4.8	0.22	25	11.3
PS	66	M	12	1	19.1	4.1	127	1.3	0.85	125.9	2.1	high	9.2	6.6	0.18	29	10.7
PS	59	M	12	1	23.5	3.6		1.92	0.92	89.39	1.5	high	8	7		29	10
PS	72	M	10	1	20.2	3.6	156	1.23	0.66	100.9	2.1	high	9.1	8.4		32	13.2
PS	77	M	9	1	26	3.7	154	2.14	0.88	110.9	1.5	high	9.1	3.9	1.36	49	9.8
PS	71	M	9	1	19.2	3.7	203	1.6	0.9	56.05	1.8	high	9.1	6.5	19.4	23	11.5
PS	83	M	9	1	20.3	3.4	191	1.63	0.82	114.1	2.2	high	9.3	4.9	0.15	23	10.2
PS	45	M	9	1	23.4	4.3	113	1.03	1.02	137.4	2.2	high	10.1	9.3	0.28	29	8.7
PS	69	M	7	1	21.8	3.3	136	2.94	1.1	137.2	1.9	high	9.4	3.9		45	10.6
PS	75	F	7	1	27.8	3.5	146	1.52	0.86	152.5	2.1	high	9.2	4.6	0.16	28	10.6
PS	66	M	6	1	20.2	3.5	145	1.59	1.16	124	1.9	high	9	3.5		24	9.1
PS	49	F	6	1	23	3.6	112	1.62	1.12	116.1	2.1	high	9.2	5.2	0.37	30	10.8
PS	61	F	6	1	20	3.2		1.46	1.14	103.8	2.1	high	9.7	5.6	0.19	35	9.5
PS	73	M	5	1	18.9	3.5	193	1.52	0.72	60.71	1.5	high	8.8	3.5	0.38		9.6
PS	61	M	4	1	19.8	4.3	252	2.01	0.83	87.99	1.5	high	9.5	3.2	0.11	18	9.6
PS	75	F	4	1	15.3	3.3	168	1.55	1.05	101.7	1.8	high	8.1	5.4	0.1		10.7
PS	66	M	4	1	19	3.4	144	2.11	1.2	119.6	1.9	high	10.2	5		31	10.4
PS	74	M	4	1	19.8	3.2	138	2.75	0.7	91.6	1.9	high	9.3	4.3		11	9.5
PS	73	F	4	1	20.3	4.1	183	1.25	0.95	107	2.1	high	9.1	6.3	0.14	28	11.2
PS	73	M	3	1	21.3	3.5	190	1.68	1.24	92.72	1.4	high		4.6		32	11.7
PS	83	M	3	1	19.9	3.3	103	1.55	0.94	84.66	2.1	high	9	5.7	0.7	22	10.4
PS	65	M	3	1	20.8	3.6	146	1.49	0.92	93.92	2.1	high	8.1	6.4	0.37	32	11
PS	49	M	3	1	21.3	3.7	179	1.5	0.82	100	2.1	high	8.4	4.6	0.97	25	9.1
PS	83	M	2	1	21	3.7	164	2.82	0.98	112.1	1.9	high	9.9	4.9		22	12
PS	50	F	2	1	19.9	3.3	173	1.3	0.72	55.52	1.1	high	10.7	3.7	4.48	23	9.2
PS	54	F	34	1	17.7	3.7	195	1.94	0.81	100.7	1.9	high	9.4	4.6		15	12.5
PS	66	F	16	1	21.2	4.1		1.25	0.78	117.3	1.3	high					
PS	47	F	14	1	15.3	3.7	84	1.24	0.63	102	1.9	high	8.8	5.5	0.13	28	10.1
PS	70	M	12	1	26.6	4.2	186	1.44	0.55	95.69	1.8	high	10.2	3	1.55	30	7.9
PS	66	M	7	1	21.3	3.7	207	2.22	0.8	112	1.5	high	9.5	4		25	9.6
PS	60	F	7	1	22.2	4.1	150	1.45	1.1	108.8	1.5	high		6.7	0.43		11.2
PS	60	M	6	1	22.5	4.5	135	1.13	0.79	105.1	1.5	high	9.6	5.8	0	33	10.2
PS	53	M	5	1	21.1	4.4	149	1.32	0.62	97.84	2.1	high	9.1	4.7	0.03	28	11.6
PS	60	M	5	1	26.3	3.8	106	1.2	0.81	103.4	1.8	high		7	0.2	44	11.1
PS	72	M	5	1	19.4	4.1	106	1.39	0.91	113.6	2.1	high	9.8	4.6	0.24	36	10.1
PS	47	F	4	1	18.1	4.1		1.42	0.63	73.52	2.1	high	8.5	5.5	0.09	44	10.3
PS	63	M	3	1	23.6	4.1	90	1.3	0.73	96.84	2.1	high		5.9	8.09		8.2
PS	58	M	2	1	24	3.8	134	1.36	0.91	100.5	2.1	high	9.8	5.8			4.8
PS	34	M	2	1	19	4.2		1.2	1.05	108.6	1.6	high	8.9	4.5	0.05	19	11.5
PS	61	M	6	1	22.8	3.6		1.29	0.84	112.8	2.1	high	8.4	4.2	0.06	28	11.2
PS	49	M	19	1	17.5	3.6		1.63	0.81	75.71	2.1	high	8.2	8.6	0.17	27	10.9
PS	66	F	7	1	25.7	3.5	128	1.55	0.73	71.46	2.1	high	10.6	6.2	0.05	42	10.8
PS	59	M	11	1	26.2	4	119	1.37	0.91	124.7	1.6	high	8.5	3.2	0.04	32	10.8
PS	67	F	23	1	16.5	4.1	157	0.92	1.04	85.95	0.6	high	8.9	4.6	0.33	41	9.1
PS	67	M	20	1	18.6	3.9	139	1.37	0.98	98.51	1.9	high	8	2.9	1.7	22	10.9
PS	63	M	8	1	25.3	3.8	127	1.43	0.87	116.2	2.1	high		7.5	0.14	25	10
PS	69	F	4	1	19.2	3.6	89	1.42	1.12	113.1	1.8	high	9.3	4.8	0.1	27	11
PS	62	F	9	1	22.7	3.8		1.39	0.88	136.9	1.6	high	9.3	4		30	9.6
PS	74	F	20	1	21.6	3.5	140	1.06	1.02	102.2	1.8	high	10.7	6.3	0.04	20	9.9
PS	80	M	4	1	17.5	3.7	174	1.25	0.87	117.9	2.2	high	8	10.9			13.5
PS	59	F	14	1	16.2	4	201	1.43	1.08	116.4	1.4	high	9.8	7.8			9.9
PS	72	F	12	1	19.7	4.1	108	1.42	1	136	1.4	high	9.2	5			9.8
PS	64	F	9	1	17.5	4.1		1.28	1.12	108.9	1.6	high	8.5	5.1		31	10.3
PS	75	M	4	1	21.4	4.2	217	1.57	1	128.7	1.5	high	9.3	4.2		29	10.6
PS	27	M	6	1	17.6	4.3	185	1.5	0.92	86.77	1.5	high	9.7	3.2	0.1	35	10.3
PS	77	F	3	1	18.4	4	144	1.68	0.83	89.36	1.1	high	8.9	7.1	0.05	20	9.7
PS	59	F	6	1	18.9	4.2	215	1.39	1.12	125.6	1.4	high	7.8	6.5			10.6
PS	39	M	2	1	22.2	4.3	142	1.13	0.74	99.76	1	high	8.7	4.5	0	18	12.1
PS	72	M	5	1	20.5	4.3	184	1.57	0.79	99.51	1.8	high	9.2	3.3	0.3	29	13

PS	19	F	5	1	17	3.1	219	2	0.65	85.01	2	high	10.7	6.3		16	8.8
PS	65	F	9	1	18.5	4.2	127	1.52	1.02	126.7	1	high	9	4.1	0.4	27	8.9
PS	31	F	4	1	17.3	4.3	178	0.92	0.82	95.74	1.5	high	8.8	7.4	0.65	34	8.6
PS	70	F	6	1	17	3.9	116	1.25	1.08	84.19	1.5	high	8.7	7.4	0.05	23	10.2
PS	68	M	9	1	21.3	3.7	151	1.35	0.79	104.6	1.8	high	9.5	3.8	1.79	28	9.6
PS	49	M	5	1	23.5	3.8	125	1	1.4	127.6	1	high		3.9	0.2	24	8
PS	58	M	15	1	23	3.6	163	1.08	0.66	88.5	2.1	high		6.3		30	11.7
PS	43	M	5	1	20.1	4.1	165	1.57	1	93.72	1.6	high	9.1	5.5		33	9.8
PS	81	M	8	1	23	3.6	190	1.95	0.86	93.32	1.6	high	7.7	5.8		32	10.1
PS	33	M	2	1	22.5	4.5	149	1.36	1.06	133.5	1.8	high	9.6	5	0.07	27	11.4
PS	70	M	4	1	23.4	3.5	225	1.56	0.65	92.1	1.3	high	9.2	5	0.27	32	9.7
PS	53	F	2	1	22.3	3.8	160	1.31	1.08	79.71	1.6	high		7.2	0.32	35	10.3
PS	59	M	20	1	18	3.9		1.09	1.45	102.7	1.3	high	8.7	4.1		17	10.1
PS	57	F	4	1	24	3.4	130	1.63	0.78	118.2	1.8	high	8.2	4.8	0.02	17	9.7
PS	37	M	2	1	21.3	3.9	318	1.5	1.35	118.9	1.6	high	11.3	4.8		27	12.3
PS	71	M	20	1	19.6	3.7	174	1.83	1.16	116.5	2.1	high	9.1	5.2	3.46	28	10.5
PS	64	M	2	1	21.1	3.5	204	1.36	0.91	101.5	1.3	high	9	5.5	0.12	17	10.4
PS	39	M	9	1	20	4.1	156	1.23	0.63	74.79	2.1	high	12	7.4	0.09	24	11.4
PS	54	F	26	1	17.2	4.2		1.12	1	88.11	1.6	high	9.7	5.4		31	11.2
PS	73	M	6	1	22.8	3.6		0.89	0.91	110.8	1.6	high	8.4	5		19	9.6
PS	70	M	16	1	22.9	3.9		1.38	0.78	127.9	1.3	high	8	4.5		46	10
PS	51	F	16	1	28	3.9	141	1.7	0.98	93.57	1.5	high	9.1	4.3	0.38	30	11.4
PS	45	F	15	1	24.7	4.4	169	1.49	1.17	123.1	1.5	high		4.8	0.03	19	11.6
PS	50	M	10	1	27.7	3.7	109	1.48	0.75	101	1.8	high	9.8	2.5	0.34	25	9.4
PS	34	M	7	1	19.4	4.4	144	1.02	1.14	110.1	1.6	high	8.3	2.7	0.14	26	9.2
PS	64	M	7	1	23.3	3.5		1.45	0.93	138.3	1.5	high	10.2	5.7		30	9.6
PS	56	F	6	1	20.4	3.9	124	0.63	0.94	110.2	1.3	high	8.3	3.2	1.97	22	8.2
PS	43	F	4	1	23.6	3.6	104	1.5	0.94	69.33	1.8	high	7.6	4.5	0.05	28	10.7
PS	66	M	13	1	19.8	4.4	203	1.4	0.77	90.34	1.8	high	9.2	5.2	0.5	24	9.5
PS	53	M	2	1	18.4	3.8		1.54	0.83	69.63	1.8	high	8.9	8.9	0.24	28	8.9
PS	55	F	2	1	17.7	4	155	1.7	1.12	111.5	1.6	high	8.6	5.2	1.1	24	8
PS	56	F	16	1	19.1	3.7	96	1.13	1	93.39	1.8	high	9	6.3	0.21	27	8
PS	54	M	3	1	18.2	3.9	168	1.39	0.68	96.51	1.3	high	8.5	5.9	0.34	29	11
PS	53	F	3	1	26.5	3.5	124	1.22	1.06	80.25	1.6	high		7.5	0.3	30	11.5
PS	60	F	20	1	20.4	3.7	174	1.81	0.75	127.1	1.8	high	8.8	6.9	0.05	25	12.3
PS	50	F	14	1	15.4	4		1.27	1.13	87.86	1.6	high	9.6	6		25	10.5
PS	47	F	2	1	23.9	3	110	1.07	1.08	44.15	1.6	high	9.5	6.6	0.4	34	10.8
PS	78	F	4	1	20.4	3.8	275	1.93	1.11	91	1.8	high	9.1	5.4	0.05	27	9.4
PS	60	F	10	1	19.9	4.4	120	1.57	0.93	121.6	1.8	high	9.9	8.1	0.1	24	9.7
PS	41	M	10	1	21.4	4.1	224	2.25	0.82	138.7	1.5	high	9.3	5.6	0.05	25	10.4
PS	75	F	3	1	17.8	3.3		0.69	0.97	89.65	1.6	high	6.2	4.1	0.07	20	9.3
PS	66	F	2	1	25.1	3.7	141	1.53	0.84	86.49	1.6	high	8.9	2.6	0.03	19	9.6
PS	60	F	2	1	22.4	3.5	136	1.52	0.65	73.02	2.1	high	9.5	4.4	0.09	26	11.2
PS	50	F	18	1	19.9	3.8	164	1.48	0.65	89.98	2.1	high	7.2	4.6	0.19	30	10.2
PS	45	M	6	1	16.5	4.2	113	1.28	1.15	78.39	1.3	high	10.2	4.3		20	10.9
PS	55	M	3	1	19.7	3.9	218	1.31	1.01	84.92	1.8	high		5.7	1.21	28	10.1
PS	52	M	3	1	24.8	3.8	159	1.43	1.07	127.5	1.5	high	10.1	6.5		28	10.1
PS	44	M	20	1	18.9	3.7	111	1.52	1.09	104.6	1.6	high	8.6	2.8		18	9.7
PS	65	F	5	1	18.2	3.7	124		1.29	105.6	1	high		9.3	1.61	34	9.2
PS	61	F	6	1	24	3.7	184	1.63	0.98	103.6	1.3	high	9	3.2	0.13	25	8.2
PS	71	F	13	1	17.1	3.6	135	1.71	0.91	125.2	1.6	high	9.4	2.2		34	12.5
PS	59	M	9	1	18.7	4.3	119	0.99	0.99	87.04	1.8	high		6.3	1.68	28	10.6
PS	41	M	5	1	26.6	4.1	186	1.52	0.97	89.09	1.6	high	9.5	4.3		24	13.4
PS	72	M	2	1	20	3.2	203	1.69	0.75	16.08	1.6	high	9.6	5.7		35	10.4
PS	57	M	4	1	22.5	4.4	130	1.29	0.75	98.17	1.8	high		6.3	0.3	32	11.4
PS	78	F	9	1	22.2	3.7	139	1.67	1.17	88.05	1.5	high	8.5	5.2		27	6.7
PS	60	F	3	1	31.6	4.2		1.74	1.29	104.9	2.1	high	8.9	3.7	0.12	26	7.3
PS	69	M	7	1	21.4	3.9	158	1.68	0.96	119.4	1.8	high	10	7.2	0.12	25	9.2
PS	68	F	7	1	18.3	3.8	146	1.34	0.97	100.8	1.6	high	8.9	5.2	0.1	24	10

PS	58	M	2	1	18.8	4.1	199	1.69	1.28	102.8	1.6	high	9.8	3.1		27	9.5
PS	55	F	28	1	17.6	3.6	157	1.67	1.14	98.08	1.8	high	10.3	4.7	0.04	30	10.8
PS	46	M	16	1	21.2	4		1.14	0.93	96.47	1.6	high	7.9	5.9		30	13.1
PS	57	M	2	1	27.2	3.8	197	2.35	1.08	103.6	1.3	high		3	0.01	17	11.6
PS	66	F	3	1	18.4	3.8	69	1.35	0.64	96.54	2.1	high	9.6	3.3	0.9		10.7
PS	64	M	14	1	19.7	3.8		1.21	0.96	91.06	1.6	high	9.1	4.5			11.9
PS	68	F	2	1	24.6	4	107	1.48	1.01	68.17	2.1	high	8	6.3	0.44	29	9
PS	63	F	10	1	20	4	160	1.22	0.92	109.1	1.8	high	8.8	3.3	0.2	22	9.9
PS	66	M	3	1	24.2	3.7		1.46	0.81	124.4	1.8	high	8.7	4.8	0.06	28	8.2
PS	51	M	12	1	24.7	4.1	131	1.52	1.18	103.2	1.5	high	8.6	4	0.07	29	8.7
PS	67	F	25	1	18.4	3.4	154	1.48	0.98	80.01	1.8	high	8.4	4.7	0.02	28	
PS	69	F	2	1	20.7	4	168	1.76	0.98	55.45	1.6	high	9.5	5.1		27	10.2
PS	50	M	11	1	22.9	3.6	179	1.96	1	138.8	1.5	high	9.1	5.3		20	10
PS	66	M	4	1	21.1	3.9	158	2.21	1.36	104.9	1.8	high	9.7	5.3	3.3	34	9.2
PS	63	M	3	1	28.7	4.2	145	1.18	0.94	127.6	1.5	high	9.8	7.4	0.08		9.1
PS	60	M	5	1	27.1	3.9	127	1.7	1.24	98.25	1.6	high	9	4.1		41	11.5
PS	23	M	5	1	18.7	3.6	150	0.99	1.22	69.41	1.5	high		5.8	2.29	25	11.2
PS	58	M	3	1	20.3	3.5	124	1.43	0.96	89.55	1.6	high	9.1	4.1		43	9.7
PS	66	F	6	1	16.7	3.4	121	1.25	0.91	96.72	2.1	high	10.1	4.9	0.05	27	11.1
PS	55	F	5	1	20.7	3.6	89	1	1.06	111.1	2.1	high	8.3	6.4	0.88	33	11.4
PS	49	F	5	1	21.8	4.2	141	1.79	0.71	86.08	1.3	high		2			11.4
PS	61	M	3	1	21.5	4.3	233	1.74	1.22	96.71	2.1	high	9.7	5.3		19	12.4
PS	46	M	23	1	25.9	3.8	190	1.59	0.82	96.45	1.6	high		5.6	0.37	30	10
PS	61	M	7	1	20.9	4		1.97	0.92	102.9	1.6	high	8.3	3.6			12.3
PS	46	M	19	1	19.1	4.3	145	1.39	0.95	91.02	1.3	high	9.4	4.5	0.1	31	11.6
PS	68	F	8	1	31.4	3.8	194	1.14	0.97	119.8	1.5	high	9.1	9.1		29	12.3
PS	63	M	9	1	21.2	3.7		1.72	1.18	150.5	1.9	high	10	4.4			11.9
PS	38	F	5	1	20.7	4	160	2.09	1.12	101.9	1.6	high	8.9	5.6	0.1	19	8.6
PS	29	M	5	1	19.1	4.2		1.6	1.54	160.7	1.8	high	7.6	2.6	5.9	31	7.4
PS	56	M	3	1	19.1	3.4	153	1.55	0.8	66.31	2.1	high	8.4	5.6	0.09	36	11.3
PS	61	F	25	1	17.1	4.2	139	1.43	1.26	69.48	1.9	high	9.7	5.5	0.14		12.7
PS	38	F	12	1	20.9	3.9	140	1.7	1.08	109.1	1.6	high		6.4		35	11.2
PS	55	M	10	1	21.1	3.7	154	1.69	1.03	101	1.8	high	10.4	5.8		22	9.2
PS	62	F	8	1	21.1	3.6	97	1.3	1.2	95.25	1.5	high		2	3.86	32	8.3
PS	71	F	2	1	20.6	3.8		1.32	1.04	81.48	1.6	high		4.5			
PS	74	M	5	1	23.1	3.3	184	1.68	0.73	59.93	1.5	high	8.5	6.9	0.07	33	9.6
PS	61	M	27	1	25.3	3.7	157	1.32	1.11	86.15	1	high	9.4	3.1	0	25	8.1
PS	57	F	24	1	20	3.4	164	1.54	1.12	106.5	1.6	high	11.4	5.9		17	13.3
PS	76	F	19	1	24	3.7	123	1.95	1.06	103.6	1.6	high	8.6	4.2		19	13
PS	57	F	19	1	14.8	3.6	133	1.58	1.08	85.97	2.1	high	10	6.8	0.25	27	13.3
PS	62	F	18	1	21.1	4.1	122	1.41	1.27	100	1.5	high	8.3	6.8	0.03	41	10.9
PS	60	F	17	1	20.6	4.3	175	1.6	1.08	124.3	1.5	high	8.7	5.1	0.75	30	11.8
PS	71	M	16	1	19.6	4	167	1.29	0.99	106.9	2.1	high	9.7	7	0.75	28	11.8
PS	56	M	15	1	18.5	4	175	1.55	1.04	95.08	1.8	high	9.4	4	0.82	30	11.2
PS	34	M	14	1	20	3.9	205	1.23	1.28	111.6	1	high	9.1	5.3	0.1	13	11.3
PS	60	M	12	1	19	4.2	145	1.46	1.13	114.4	1.6	high	9.2	3.5	0.1	25	9.7
PS	62	M	7	1	19.5	3.7	128	1.01	1.06	81.64	1.8	high	8.6	7.9	0.1	26	9.9
PS	48	F	4	1	19	1.6	133	1.33	0.82	60.78	2.1	high		8.3	10.1	27	9.6
PS	61	F	3	1	19.1	4.1	161	1.18	0.86	85.55	1.8	high	9.1	6.3	0.14	29	12.5
PS	41	F	2	1	24.7	3.9	181	1.46	1.08	116.5	1.3	high	9	1.3	0.1	27	10
PS	64	F	13	1	16	3.5	218	1.65	1.07	84.72	2.1	high	11.3	4.1	0.02	23	11.8
PS	45	F	9	1	26.3	4.4	170	1.24	1.07	106.2	2.1	high	10.6	6.6	0.08	30	10.4
PS	66	M	5	1	23.5	3.3	213	1.38	1.24	100	1.3	high	8.4	5.7	0.31	27	10.8
PS	67	F	4	1	19.4	4	125	1.49	0.92	140.8	1.5	high	9.4	3.4	0.1	27	11.3
PS	71	M	4	1	18.4	3.4	152	1.52	0.76	90.75	1.8	high	9.4	5.5	0.47	35	9.4
PS	49	M	3	1	19.2	3.8	184	1.36	0.99	88.84	1.5	high	8.4	5.2	0.34	31	10.7
PS	84	M	2	1	24.5	3.3	137	1.1	0.66	82.54	1.3	high		4.6	0.26	31	11.2
PS	63	F	20	1	17.3	4	168	1.31	0.87	85.46	1.5	high		6.5	0.16		11.8
PS	80	F	13	1	18.4	3.9	186	1.49	0.88	103.4	1.6	high		5.7		25	10.6

PS	52	F	10	1	25.5	4.1	192	1.56	0.67	90.41	1.5	high	8.5	3.8	0.3	29	12
PS	66	M	7	1	29.3	3.8	142	1.19	1.09	118.7	2.1	high		3.5	3.56	23	9.3
PS	60	M	2	1	23.3	3.9	153	1.45	0.93	61.77	1.5	high	9.1	4.9	0.12	30	11.7
PS	48	F	21	1	18.7		180	1.87	1.07	118.4	1.5	high	9.7	6.3	0	30	10.5
PS	53	M	19	1	19.3	3.6	198	1.22	1.03	122.4	2.1	high	9	5.8		26	10
PS	68	M	3	1	15.7	3.9	159	1.2	0.9	129.9	1.3	high	9.3	6.2	0.06	25	10.6
PS	53	M	4	1	23.3	3.9		1.05	0.98	113.6	1.8	high	8.3	6.1	0.27	21	10.7
PS	72	M	3	1	16.7	3.2	181	1.89	0.96	93.42	1.5	high	10.3	5.5	0.05	25	11.1
PS	52	M	14	1	25.2	3.6	117	1.61	0.67	114	2.1	high	9.6	5.1	0.88	25	9.1
PS	49	M	4	1	28.2	4.2	178	0.97	1.09	138	1.6	high	9.2	4.9	0.01	15	8.9
PS	63	F	4	1	21.4	4.4	152	1.67	0.84	130.3	1.8	high	9.1	6.2	0.06	29	10
PS	73	F	6	1	21.4	3.5	163	1.07	1.07	141.3	1.8	high	8.5	7.2		21	11.4
PS	39	M	6	1	24	4	109	1.52	0.83	117.5	1	high		7.1			10.1
PS	53	M	10	1	23.7	3.8	149	1.2	1.24	128.9	2.1	high	9.6	6.1	0.06	23	10.5
PS	49	M	12	1	29.1	3.8	124	1.29	0.98	106.5	1.3	high	8.9	7.4	0.15	26	14.1
PS	59	M	7	1	24.3	3.2	150	1.23	1.08	90.04	1.1	high	9.1	4.7	1.17	28	10.6
PS	67	F	24	1	19			1.85	0.87	90.62	1.5	high	9	3.2	0.38	26	10
PS	49	M	2	1	21.6	3.8	177	2.57	0.96	81.96	1.8	high	10.1	3.5		19	8.2
PS	81	M	8	1	18.6	3.7	147	1.43	0.6	89.14	1.9	high		6.3		33	12.4
PS	74	F	5	1	19.3	3.7	182	1.51	0.98	80.45	1.5	high	9.7	6.2		19	11.4
PS	77	M	4	1	22.5	4	120	1.33	0.68	132.1	1.6	high	8.9	3.7	0.05	20	12.7
PS	51	M	4	1	26.9	3.8	122	1.09	0.88	84.37	2.1	high	8.9	7.8	0.17	27	8.5
PS	71	M	2	1	18.9	1.8	190	1.6	0.77	81.8	1.5	high	9.3	5	0.05	36	11.1
PS	76	M	4	1	20	3.4	149	1.56	0.83	115.4	1.8	high	8.5	5.5	0.2	26	10.3
PS	60	F	3	1	33.5	3.4	128	1.5	0.72	98.88	1.8	high	9.1	5.3	0.15		9.3
PS	41	M	2	1	29.8	4.1	188	1.09	0.81	94.7	1.5	high	8.4	6.4	0.6		8.7
PS	66	M	2	1	23.8	3.2	145	1.18	0.73	74.18	1.5	high	8	4.6	0.3	33	
PS	63	M	8	1	21.3	3.4	156	1.29	0.9	129.6	1.8	high	9.3	5.8	1.17	24	9.4
PS	60	M	3	1	22.4	4.1	178	1.93	0.98	133.8	1.8	high	8.9	6.3	0.05	29	10.4
PS	52	F	6	1	18.9	3.5	146	1.54	0.98	100.4	2.1	high	9.5	6.6	0.1	27	9.9
PS	58	F	30	1	17.7	3.9	227	1.07	1.25	71.77	1.5	high	9.8	4.9	0.05	18	11.3
PS	57	M	10	1	17.5	3.8	138	1.37	0.91	99.04	1.1	high	9.2	5.2	0.07	27	9.6
PS	75	F	6	1	27.4	3.6	154	1.36	0.65	94.08	1.3	high		5.1			10.5
PS	56	M	6	1	30.6	2.6	142	1.49	0.64	53.98	2.1	high	9.9	7.3	0.02	29	9.1
PS	50	F	5	1	31.1	3.7		1.53	0.81	125.6	1.6	high	9.2	5.7	1.31		12.4
PS	55	F	5	1	24.1	3.3	106	1.6	0.95	107.2	0.8	high		3.7			9.2
PS	61	M	3	1	18.9	4.3		0.87	0.67	66.54	1.9	high	9.7	4.9	0.24	29	9.6
PS	44	F	3	1	22.4	3.8	136	1.18	1.15	111.8	2.1	high		4.7	0.05	32	9.8
PS	44	M	16	1	21.2	4	106	1.44	0.94	122.5	1.5	high	8.4	4.9	0.03	41	10.2
PS	41	F	13	1	24.4	3.9	172	1.52	1.05	131.9	1.8	high	10.3	8.8	0.38	33	10.4
PS	71	F	4	1	19.2	3.6	156	1.3	0.62	94.07	1.5	high	8.7	5.4	0.4	16	8.6
PS	59	M	15	1	20.3	3.8	129	1.2	1.06	114.7	2.1	high		5.1	0.04	23	11.3
PS	69	M	6	1	26.3	3.5	126	1.4	1.13	116	2.5	high	8.1	5.9	1.21	33	9.8
PS	69	M	3	1	25.7	5.6	174	1.46	0.7	126.2	1.3	high		5.3			9.8
PS	50	M	3	1	27	3.7	189	1.36	1	116.6	1.1	high	9.6	5.1	0.1	21	10.7
PS	33	F	10	1	30.1	3.8	219	1.5	1.06	103.3	1.5	high	9	9.1	0.05	38	10.2
PS	40	F	5	1	20.2	4		1.29	1.09	113	1.8	high		4.2		33	10.4
PS	58	F	7	1	21.6	4.2	187	2.01	0.57	85.86	1.8	high	10	5.5	0.08	23	8.8
PS	71	M	13	1	16.7	3.8	161	1.46	0.81	93.1	1.8	high	8.5	4.2		27	11.2
PS	72	F	9	1	23.1	3.4	139	1.34	0.96	119.9	1.5	high	8.8	4	0.3	28	9.9
PS	71	M	2	1	23.4	3.8	142	1.17	0.77	89.13	2.1	high	9.6	5.8	0.48	35	11.5
PS	48	M	3	1	23.8	4.4	183	0.93	1.05		1.5	high	9.9	6.1		31	11
PS	64	M	4	1	25.6	4.1	151	1.38	0.82		1.3	high	7.8	5.3	0.01	31	10.9
PS	37	F	2	1	25.3	4	113	1.26	0.91	97.76	2.1	high	9.5	6.1	0.2	29	11
PS	54	M	2	1	22.6	3.9	99	1.28	0.71	84.53	2.1	high	8.5	4.5		35	10.4
PS	74	M	6	1	23.5	3.8	200	1.72	1.03	104.9	0.8	high	9.6	5.9	0.1	43	10
PS	72	M	6	1	20	3.9	123	1.2	0.66	101.1	1.5	high	8.5	4.4	0.6	35	11.4
PS	63	M	11	1	19.3	3.7	115	1.25	0.91	108.5	1.6	high	7.9	3.9			
PS	63	F	29	1	20.7	3.3	150	1.16	1.05	127.8	2.5	high	9.2	8.2	0.44	28	10.7

PS	77	M	7	1	19.9	3.9	174	1.76	1.02	111.3	0.8	high	8.1	3.4	0.2	42	10.9
PS	60	M	17	1	17.7	3.7	98	1.27	0.99	87.91	2.1	high	9.3	4.9	9.53	33	6.7
PS	76	F	4	1	19	3.4	133	1.47	1.05	106.6	1.3	high	9.1	6.1	0.07	24	10.6
PS	60	M	14	1	21.6	4.5	131	2.08	0.97	122.2	2	high		5.1	0.05	22	9.8
PS	78	M	14	1	24.2	4.1	116	1.3	0.72	60.17	2.1	high		4	0.44	31	11
PS	66	M	10	1	23.7	3.9	201	1.47	0.89	116.7	1.8	high	9.6	6.5	0.21	32	11.3
PS	70	M	4	1	24.5	4	165	1.29	0.86	115.3	2.1	high		6.8	0.34	18	9.5
PS	61	M	5	1	22.5	3.8	130	1.2	1.01	131.8	2.1	high		3.4	0.79	28	10.9
PS	72	M	5	1	21.8	3.8	172	1.45	0.73	109.9	1.8	high	9.5	4.6	0		10.8
PS	46	F	31	1	13.9	3.8	123	1.9	0.89	87.51	2.1	high	9.3	4.6	0.1	24	11.1
PS	64	M	9	1	20.3	4.4	81	1.35	0.84	136.5	2.1	high	9.9	5.9	0.07	32	10.9
PS	49	M	6	1	24	4		1.78	0.7	112	1.9	high	7.8	5.6			
PS	74	M	4	1	18.8	3.8	256	1.56	0.73	67.42	1.3	high	10.8	4.8	0.24	32	8.2
PS	59	M	2	1	28.3		138	1.88	0.64	50.81	1.5	high	8.4	6			11.4
PS	63	M	2	1	23.4	3.8	192	1.27	1.06	119.3	1.8	high		6.5	0.05	19	10
PS	69	F	13	1	15.8	3.5		1.93	0.83	85.78	1.3	high	9.7	5.4		28	11.1
PS	59	M	9	1	21.8	3.8	103	1.36	0.81	114.3	1.8	high		5.5	0.06	27	11.7
PS	64	F	17	1	20.3	4.4	152	1.34	1.27	117	1.1	high	8.1	4.6	0.56	21	9.2
PS	62	F	10	1	22.1	3.3	156	1.33	0.79	127.5	1.5	high	8.3	8.8	0.1	22	9.8
PS	61	M	3	1	31	3.6	158	1.35	0.89	77.11	2.1	high	8.2	6.2		28	
PS	53	M	2	1	29.3	3.6	88	1.18	0.79	75.92	1.6	high	8.5	4.2	0.17	16	10.4
PS	58	F	5	1	28.6	4.1	99	1.44	0.62	118.9	1.6	high	9.6	6	0.12	20	10.3
PS	64	F	5	1	20.1	4.2	151	0.98	0.83	112.2	2.1	high	9.9	4.9	4.12	25	10.3
PS	55	F	2	1	30.4	3.9	141	1.2	0.93	88.62	1.8	high	8.5	5.8	0.05	20	10.5
PS	41	F	5	1	29.8	3.9	141	1.41	1.03	125.5	1.3	high	8.3	4.4	0.1	29	11.5
PS	75	M	9	1	18.5	3.6	143	1.7	0.6	105.7	1.8	high		7.3	0.35		13.1
PS	69	M	11	1	21.6	3.4	93	1.11	0.83	115.5	1.1	high	8.4	5.9	3.2	46	11.1
PS	53	F	37	1	15.3	3.5	114	1.32	0.99	67.21	1.4	high	8.8	5.2	0.1	32	10.1
PS	62	F	29	1	18	3.6		1.64	0.95	107.7	1.6	high	9.8	7.4	0.15	28	11.4
PS	53	F	24	1	19.2	3.8	117	1.17	1.07	94.47	1.8	high	8.7	6.6	0.05	22	11.7
PS	62	F	17	1	15.5	3.7	160	1.72	1.35	96.56	2.1	high	8.9	6.3	0.02	61	10.8
PS	65	F	15	1	18.6	4.1		1.72	1.01	118.2	1.6	high	8.4	4.7			
PS	69	F	15	1	18	3.7		1.66	0.92	105.3	1.9	high	8.2	6.6			
PS	83	M	11	1	19	4.1	159	1.32	0.82	92.02	2.1	high	9.6	3.8		22	9.5
PS	62	F	11	1	18.3	3.7	138	1.42	0.89	124.2	2.1	high		5.6		22	8.6
PS	62	M	10	1	24.6	4.3	90	1.61	0.93	106.1	1.5	high	9.7	6.3	0.09	32	10.4
PS	53	F	9	1	16.3	4.3	176	1.34	1.1	120.2	1.5	high		4.8	0.05		10.8
PS	64	M	5	1	23.9	3.8	148	1.53	0.87	91.92	2.1	high	9.7	4.6	0.11	26	11.8
PS	70	F	4	1	17.6	3.7	124	1.58	0.85	122.1	2.1	high	8.3	4.3	1.61	34	8.7
PS	86	M	2	1	16.6	3.7	111	1.24	0.93	96.11	1.3	high	8.2	6.6		27	9.4
PS	39	M	12	1	20.2	4		1.2	1.38	120.8	1.9	high	9.3	5.5			
PS	59	M	9	1	21.5	3.7	175	1.46	0.84	129.2	1.5	high	10.5	5.6	0.2		10.7
PS	35	M	3	1	16.9	4.3	43	1.19	0.95	73.88	1.3	high		4.3	0.24		9.8
PS	51	M	16	1	25.3	3.5	244	2.35	0.84	115.2	1.5	high	9.2	4.9	3.8	18	11.1
PS	76	M	12	1	18.9	3.5	135	1.69	0.84	103.6	1.4	high	8.7	3.4	0.05	31	9
PS	71	M	5	1	19.3	3.5	165	1.59	0.79	134.7	1.8	high		6.8			13.8
PS	73	M	4	1	24.2	3.6	123	1.16	0.66	109.5	1.8	high		4.8	0.05	32	13.2
PS	59	M	23	1	19.1	3.7	166	1.33	0.88	117.3	1.6	high	9	2.4	0.4	37	8.8
PS	79	M	4	1	19.5	4.1	193	1.22	0.77	80.1	2.1	high		4.3	0.06	79	12.3
PS	53	M	30	1	19.9	3.1	152	0.85	0.99	90.34	2.2	high	7.4	4.6	0.1	25	
PS	60	M	6	1	25.4	3.8	137	1.35	0.82	137.7	1.3	high	9.7	4.5	0	29	11
PS	66	M	4	1	21.2	4.3	185	1.93	0.97	100.3	1.1	high	9.9	5.9	0.1	36	9.9
PS	46	M	2	1	18.6	4	157	1.32	0.98	90.71	1.6	high	9	5.4	0.14		10.5
PS	52	M	13	1	23.7	3.8	158	1.25	0.7	106.6	1.5	high	9.5	4.3	0.3		10.5
PS	87	F	6	1	18	3.6	183	1.41	0.92	117.4	1.5	high	8.3	4.2	0.06	29	8.9
PS	70	F	3	1	24.4	3.6	210	1.69	1.41	96.02	1.6	high		5.2	0.05	28	10.7
PS	62	M	4	1	23.3	3.7	148	1.42	0.67	67.23	1.6	high		5.3	13.3	41	8.3
PS	61	M	3	1	22.2	4.2	101	1.55	1.03	101.6	1.8	high	9.2	4.6		30	10.6
PS	79	F	4	1	31	3.3	150	1.63	0.68	37.45	1.5	high	9	3.7	3.3	29	7.7

PS	46	M	5	1	24.2	4.1	159	1.36	0.85	117.4	0.8	high	9.6	4.2	0.1	27	9.6
PS	74	M	5	1	26.2	3.4	133	1.75	0.93	98.18	1.8	high	9.6	2.6	0.7	20	10.6
PS	71	M	12	1	19.7	3.5	176	1.32	0.79	127.2	0.8	high	10.3	7.7			8.6
PS	74	F	12	1	18.2	3.8	186	1.56	1.07	86.79	1.5	high	6.2	4.9	0.04	31	7.5
PS	75	M	8	1	16.8	3.9	138	1.72	1.29	110.4	1.3	high	10.5	6.2	0	22	11
PS	69	M	2	1	19.6	3.7	156	1.12	0.9	128.3	1.5	high	9.1	7.4	0.06	25	10.5
PS	67	M	2	1	21.5	3.4	121	0.98	0.83	82.95	1.8	high		5.6			10.9
PS	61	M	4	1	20.9	3.7		2.15	0.98	161.3	1.5	high	8.4	4.1	0.65		9.5
PS	50	M	16	1	19.7	4.3	132	1.42	0.9	99.94	1.5	high	9.7	6.5	0	37	10.4
PS	38	M	3	1	20.9	4	137	1.12	0.81	83.04	1.3	high		6.9	0.05	28	9.4
PS	65	F	2	1	18	3.7	134	1.23	1.07	121.1	1.1	high	8.1	4	0.1	31	8.6
PS	61	M	6	1	24	3.6	162	1.53	1.1	121.7	1.8	high	9.4	7.7			6.7
PS	59	F	11	1	23.6	3.7	172	1.71	0.94	93.66	1.5	high	9.2	4.8		30	10.7
PS	59	M	8	1	24.8	4.8	182	1.38	0.91	158.3	1.5	high	7.9	5.8	0.3	22	10.1
PS	83	M	3	1	17.1	3.1		1.02	0.76	103.1	1.6	high	9.1	5.3			
PS	54	M	30	1	15.9	3.5	149	1.27	1.1	71.97	1.6	high	10.5	6.3		21	10.7
PS	56	M	29	1	23	3.5		1.76	0.82	86.79	1.6	high	9.7	7.3			
PS	54	M	21	1	20.5	3.9	149	1.83	1.01	112.6	1.6	high	9.8	3.7			11.5
PS	63	M	21	1	19	3.6		1.26	0.91	105.5	1.6	high	8.3	6			
PS	58	F	20	1	15.9	3.7	197	1.68	0.81	99.2	1.6	high	8.6	7.5		20	10.2
PS	67	F	19	1	16.3	3.8		1.29	1.44	111.4	1.3	high	9.9	4.3			
PS	60	F	16	1	20.7	3.8	137	1.75	0.72	81.14	1.6	high	8.8	5.9	0.12	34	11.2
PS	69	F	14	1	21	3.5	158	1.59	0.98	111.1	1	high	8.8	4.7		32	11
PS	72	M	14	1	20.5	3.7	146	1.26	0.99	111.8	1.3	high		4.9	1.16	26	12
PS	41	F	14	1	22.5	3.8	140	1.49	1.05	94.08	1.9	high	8.5	5.2	0.1	40	11
PS	56	M	12	1	19.6	3.8	189	1.6	0.78	121	1.1	high	9.4	5.1	1.48	24	10.2
PS	38	M	11	1	14.5	3.7	195	0.99	0.81	73.39	1.9	high	9.2	5.4	0.1	26	10.5
PS	41	M	11	1	26	4	95	1.39	1.07	110.6	1	high	9.1	4.6	0.03	34	9.4
PS	55	M	10	1	21.8	3.6	203	1.82	0.86	111.5	2.1	high	9.2	4.6	0.04	28	9.8
PS	59	M	9	1	20.9	3.6	186	1.4	1.02	107.9	1.5	high	8.2	4	1.11	31	11
PS	73	M	9	1	19.3	3.8	147	1.24	0.96	125.1	1.5	high	8.9	5.8	0.02	32	10.2
PS	59	M	8	1	21.9	3.4	179	2.21	0.79	82.47	1.6	high	8.8	4.6	0.06	30	9.4
PS	68	F	7	1	18.2	3.5	163	1.41	1.04	96.18	1.9	high	8.6	3.4	3.26	20	6.2
PS	89	F	7	1	19.3	3.5	128	1.05	1.06	109.4	1.9	high	8.2	3	0.12	24	8.5
PS	49	M	7	1	21.3	3.6	127	1.69	1.05	82.08	1.5	high	8.7	5.7	0	44	10.8
PS	60	M	7	1	17.1	4	156	1.31	1.01	85.66	1.5	high		2.6	0.1	11	9.5
PS	62	M	7	1	23.6	4	185	1.68	1	114.4	1.5	high	8.2	8.6	3.7	45	7.7
PS	76	M	5	1	25.7	3.3	148	1.44	0.85	131.7	1.3	high		7.8	0.1	29	11.9
PS	55	M	4	1	16.3	3.6	175	1.26	0.79	64.83	2.1	high	10.1	6.6		28	12.7
PS	77	F	3	1	19.9	3.5	190	1.69	1	110	1.5	high		3.2	0.04	21	10.3
PS	70	F	3	1	23.3	3.6	118	1.54	0.82	112.6	1.3	high	8.4	3.6	0.05	35	11.5
PS	48	M	3	1	26.7	4.1	135	1.01	0.76	46.54	1.8	high		5.2	0.05		10.5
PS	64	M	2	1	17.5	3.7	110	1.52	0.87	118.6	1.3	high	9.3	6.3	4.8	28	11
PS	59	M	2	1	25.8	3.6	149	1.44	0.89	55.77	1.5	high	9	3.3	0.4	40	11.5
PS	83	F	2	1	24.3	3.4	175	1.41	0.92	72.61	1.5	high	9	6.6	0.05	27	11.5
PS	89	F	2	1	24.9	3.5		1.52	0.89	108.3	2.1	high	8.3	2.8			10.8
PS	56	F	18	1	19.1	4.3	170	1.64	0.81	121.5	1.5	high	10.2	6.5	0.01	22	11.5
PS	72	F	4	1	22.6	3.8	160	1.35	0.75	70.19	1.5	high		4.5	0.12		11.4
PS	78	F	2	1	20.6	4	179	1.6	0.76	61.13	2.1	high	9.2	3	0.1	20	10.4
PS	61	M	10	1	21.5	3.6	202	1.39	0.99	94.1	1.8	high	10.2	4.4		27	10.2
PS	63	M	8	1	17.8	3.5		0.98	0.84	107.3	1.5	high	9	3.2	4.98	25	10.9
PS	61	M	13	1	26.3	4		1.21	1.02	119.8	2.5	high	9.6	5.7	0.3		10.1
PS	65	M	2	1	23.5	3.6	129	1.18	0.97	71.28	1.6	high	8.7	6.4	0.05	23	10.3
PS	62	M	17	1	23.2	3.6	96	1.58	1.17	124.7	1.3	high	9.2	4.6		34	10.1
PS	57	F	12	1	17.6	3.9	167	1.04	1.04	123	1.6	high	7.4	3.6	0.92	12	10.7
PS	57	M	26	1	25.5	4	109	1.85	0.73	128.1	1.4	high	8.1	3.5	1.24	29	10.4
PS	45	F	14	1	27.7	4.1	109	1.26	0.81	92.96	1.5	high	9.3	7.2	0.01	20	11.1
PS	68	F	3	1	26.1	4.4	148	1.47	0.98	139.3	1.5	high	9.8	4.4	0.43	25	9.8
PS	64	F	2	1	26.9	5.8	185	0.81	0.78	104	2.1	high	8.2	9.1		25	10.4

PS	83	F	3	1	23.2	3	178	1.2	0.66	84.59	1.9	high	8.8	5.1	0.54	28	9.3
PS	59	M	16	1	19.4	4	185	2.05	0.99	107.6	1.5	high	9.1	5.4	0.1	25	9.6
PS	86	F	5	1	17.9	3.8	138	1.21	0.94	105.1	2.5	high	9.4	6	0.52	25	10.1
PS	57	M	7	1	19.8	3.7	126	1.77	1.23	110.8	2.1	high	8.5	5.4			10.4
PS	60	M	5	1	27.8	3.8	180	1.63	0.99	107.9	1.5	high	9.8	4.6	0.44	34	9.3
PS	55	M	10	1	23.3	4.1	176	1.55	0.88	115.1	1.8	high	9.5	4.4	0.4	28	9.6
PS	55	F	20	1	22.3	4.1	165	1.51	0.86	106.6	1.3	high	8.7	5.9	0.3	22	11.1
PS	40	M	17	1	18.6	4.1	215	1.2	0.8	90.81	1.8	high	7.9	6.1		21	10.6
PS	73	M	8	1	20.3	3.5	127	1.51	1.03	120.9	1.8	high	7.6	6.4			8.8
PS	42	M	17	1	21.2	3.5	185	1.32	0.75	81.56	1.6	high	9.7	4	0.02	32	11.6
PS	47	F	11	1	20.1	3.7	119	1.51	1.04	100.5	1.9	high	10	8.4	0.25	25	12.5
PS	47	M	5	1	32.6	3.8	236	1.26	0.69	100.8	1.3	high	9.8	3.7	0.16	6	12.2
PS	70	M	8	1	21.9	4.3	138	2.24	1.08	114.1	1.3	high	9.4	2.9		41	9.8
PS	62	F	27	1	16.5	4	192	1.7	1	117.3	2.2	high	10.7	4.8	0.16	25	10.2
PS	73	F	22	1	22.2	3.6	225	1.21	0.88	146.8	1.3	high			0.27		10.9
PS	70	F	18	1	19.8	3.8	150	1.55	1.01	106.4	1.5	high	9.3	6.2	0.15	24	12.4
PS	50	M	7	1	21.5	4	106	1.41	0.98	104.1	1.3	high	9.2	5.1	0.18	31	12.2
PS	61	M	8	1	23.7	4.3	188	1.34	0.49	114.1	1	high		5.8			9.8
PS	68	M	10	1	20.6	3.4	130	1.03	0.9	111.7	2.1	high	8.9	7.6	0.13	27	9.4
PS	44	M	5	1	22.4	3.9	147	1.41	0.89	78.72	1.8	high	8.6	4.3		29	11.2
PS	52	F	16	1	23.2	4.5	108	1.36	1.41	135.5	1.8	high		3	0.1	32	11.5
PS	69	M	5	1	23.9	4	194	1.22	1.01	103.5	2.1	high	9.5	7	0.1	22	11
PS	52	F	29	1	16	4	156	1.84	1.17	95.25	1.6	high	9.3	5.4		17	10.6
PS	61	M	17	1	22.5	3.7	129	1.46	0.86	116.3	1.9	high	9.9	4.9	0.36	20	9.2
PS	71	F	13	1	19	3	121	1.16	0.81	102	1.5	high		2.7	0.02		9.5
PS	81	F	11	1	18.2	3.6	88	1.63	0.74	113.5	1.6	high	7.7	6.4	0.56	24	9.6
PS	67	M	3	1	21	4.1	183	1.41	1.06	110.8	1.8	high	9.5	5.2	0.57	36	13
PS	72	F	2	1	19.2	3.9	150	1.44	0.94	95.13	1.5	high	9	3.8	16.57	17	9.4
PS	55	M	4	1	21.7	4	186	1.81	1.21	107.6	1.4	high	10.2	3.9	0.13	28	10.6
PS	54	M	4	1	23.1	4.1	92	1.41	0.91	103.9	1.8	high	8.5	3.3	0	37	8.5
PS	69	F	25	1	16.3	3.6	156	1.75	1.24	117.4	1.6	high	9.2	3.5	0.02	16	8.8
PS	52	F	29	1	21.2	4	126	1.3	1.17	111.2	1.8	high	8.9	6.1	0.02	34	10.4
PS	38	M	4	1	31.7	3.9	185	1.18	0.96	95.63	2.1	high	8	8	0.1	23	8.1
PS	51	M	17	1	18.9	3.9	100	1.3	0.77	119.6	1.5	high	9.1	2.3	0.2	27	10.6
PS	48	M	15	1	18.4	4.3	117	1.22	0.82	112	1.6	high	8.3	6.2		32	11
PS	59	M	30	1	18	4.1	144	0.97	1.04	102.8	1.6	high	9.2	5.6	0.19	24	10.9
PS	59	F	20	1	24.8	4.3	178	1.19	0.85	95.09	1.9	high	9.5	4.9	0.49	50	10.8
PS	62	F	20	1	23.1	3.8	132	1.16	0.85	134.6	1.6	high	7.2	5.9	0.04		12
PS	33	F	18	1	18.3	4.3	166	1.4	1.04	110.2	0.8	high		2.7	0.04		13.1
PS	66	M	13	1	26.1	4.3	97	1.81	0.79	155.6	1.3	high	10.5	5.3	0.02		9.9
PS	36	F	10	1	19.1	3.9	172	1.37	0.89	107.6	1.3	high		4.8	0.34		10.9
PS	63	F	10	1	22.1	4.1	192	1.19	1.15	87.45	1	high		4.9	0.43	30	10.7
PS	33	M	9	1	21.8	4.6	177	1.29	0.99	133.9	1.5	high	9.9	7.9	0.03	26	9.6
PS	68	F	8	1	21	3.8	171	1.27	0.98	88.92	1.6	high	10.2	7.2	0.03		9.9
PS	47	F	7	1	25.7	3.5	135	1.26	1.46	112.4	1.6	high	9.4	5.2	0.03		11.1
PS	76	M	7	1	28.8	4	150	1.29	0.87	97.74	1.3	high	10	5.9	0.23	24	10
PS	61	M	7	1	19.4	4.1	186	1.26	0.83	96.02	1.1	high		5.5	0		11
PS	54	F	6	1	24.5	4.2	116	1.27	0.99	139.2	1.6	high	8.9	4.8		34	11.4
PS	57	F	6	1	29.7	4	157	1.36	1.35	104.1	1.8	high	9.8	5.5	0.39	26	12.4
PS	63	F	6	1	21.9	4.2		1.63	1.03	132.4	1.8	high	9.1	5.1	12.88		8.9
PS	71	F	5	1	21.9	3.8	174	1.32	1.15	106.8	1.1	high		5.1	0.02		9.9
PS	65	M	4	1	23.5	3.6	124	1.55	0.75	100.6	1.5	high	10.3	6.3	0.5	19	11.2
PS	65	M	4	1	28.1	3.8	107	1.18	0.75	97.59	1.3	high	7.8	5.6	0.13	33	11.6
PS	73	M	3	1	20.6	4.4	106	1.77	0.85	80.76	1.5	high	8.6	4.3	0		
PS	71	F	6	1	23.6	3.8	181	1.64	0.79	110	2.1	high	8.9	6.2	0.13	26	11.2
PS	68	F	9	1	24.7	3.7	190	1.21	0.98	94.52	1.6	high	8.9	6	0.1		9.3
PS	65	M	3	1	23.5	3.8	124	1.75	0.83	110.8	1.6	high	8	4.7	3.2	24	8.9
PS	76	M	16	1	19.3	3.3	159	0.91	0.91	96.6	1.6	high	7.9	6.8		32	11.8
PS	59	M	11	1	19.9	4.2	146	1.27	1.07	101.9	1.3	high	10.7	5.6		33	10.6

PS	53	F	32	1	17.9	4.3	169	1.12	0.9	118.6	1.5	high	10	6.8		31	13.5
PS	52	F	16	1	18.7	4.4	117	1.39	0.73	111.8	1.4	high	8	4.7	0.05	27	9.9
PS	54	F	15	1	18.4	4	142	1.56	0.93	130.3	1.4	high	8.8	4.7	0.06	16	13.4
PS	69	F	14	1	18.1	3.9	136	1.2	0.93	92.53	1.3	high	7.2	2.2		39	11.4
PS	80	M	8	1	21	4.3	146	1.26	0.85	115.5	1.8	high	8	5.5	0.03	32	9.3
PS	71	F	8	1	22.4	4	149	1.22	0.89	133.1	1.4	high	8.7	4.6	0.28	26	11
PS	60	M	7	1	22.6	4		1.81	0.97	111.2	1.8	high		2.5	0.03	21	10.2
PS	79	M	6	1	18.8	4.2	173	1.32	1.11	116.3	1.5	high	10.3	5.8	0.17	27	11.2
PS	40	M	6	1	21.4	4.2	175	1.65	0.8	123	2.1	high		5.5	1.5	15	11.8
PS	59	M	6	1	19.3	4	158	1.08	1.01	104.5	1.8	high	8.6	6.2			11
PS	53	F	5	1	24.7	4.3	176	1.78	1.02	103.3	2.1	high	8.9	6.3	0.02	25	7.2
PS	78	M	5	1	23.8	4.4	125	1.72	1.11	143.4	2.1	high	9.1	3.4	0.03	24	9
PS	61	F	3	1	25.8	3.5		0.65	0.82	94.28	0.8	high	8.5	5	3.2		9.2
PS	72	M	3	1	20.5	4.2	145	1.12	0.95	84.69	1.5	high		6	0.06		10
PS	61	F	2	1	26.1	4	138	1.76	0.72	95.04	1.8	high	9.8	8.8	0.03	25	12
PS	71	F	13	1	17.3	4	118	1.89	0.8	110.3	1.8	high	8.7	4.8	0.29	39	10.6
PS	56	F	12	1	25	3.8	138	1.34	1.02	135.3	1.4	high	9	4.2	0.32	25	9.5
PS	66	M	7	1	22.2	4.2		1.64	0.93	112.9	1	high	9.2	6.8	0.05		10.3
PS	72	M	5	1	24.3	3.9	119	1.34	0.99	119.1	1.6	high	7.2	1.9	0.12		8.8
PS	66	M	3	1	21.6	3.8	201	1.43	1.02	82.98	1.6	high	10.5	6.9	0.05	25	9.2
PS	69	F	3	1	19.6	4	110	1.06	0.93	59.21	2.2	high	10.2	7.2	0.09	30	9.5
PS	63	M	7	1	21.4	4.2		1.41	0.9	99.16	2.1	high	9	5.7	0.2	28	9.8
PS	63	M	12	1	20.8	4.4	154	1.65	1.14	139.8	1.8	high	7.7	4	1.61	31	10.8
PS	73	F	16	1	14.9	3.4		0.83	0.98	101.7	1	high	9	6.5	0.2	32	9.6
PS	47	F	12	1	19.1	3.7	185	1.68	1.11	111.6	1.3	high		3.2	0.48	31	9.5
PS	45	F	7	1	18.5	4	169	1.29	0.89	111.3	1.3	high	11.8	3.6	0.6	29	10.2
PS	61	M	4	1	19.7	3.6		1.24	0.99	105.4	1.5	high	8.7	4.3	0.65	34	11.6
PS	59	M	11	1	22.3		123	1.61	1.03	93.27	1.5	high	8.4	4.5			12.4
PS	61	M	30	1	19.8	3.4	152	1.69	0.67	92.53	1.8	high	8.1	4.3	0.02	27	10.2
PS	61	M	26	1	19.5	3.7	109	1.66	0.92	121.7	1.4	high	8.7	5	0.05	28	9.9
PS	83	M	19	1	15.1	3.4	198	1.39	0.9	87.4	1.6	high	8.2	9.6	0.08		11.6
PS	63	M	16	1	17.2	3.7	175	1.68	0.88	119.2	1.6	high	8.2	6.9		35	11.2
PS	74	M	14	1	23.5	3.7	148	1.73	0.83	75.28	1.5	high	9.8	4	0.3	41	10.6
PS	67	M	14	1	16	3.2	151	1.98	1.04	78.62	1.8	high	9.1	2.3	0.1	30	10.9
PS	60	M	13	1	23.4	3.5	165	1.43	1.09	161.7	1.9	high	10.1	8.4	0.05	35	9.8
PS	74	M	13	1	25.3	3.2	111	1.71	0.9	116.6	1.5	high	9.7	6.4	0.1	29	11.5
PS	60	F	13	1	18	4	144	1.88	1.08	108.1	1.8	high	8.2	4.4	0.19	39	10
PS	61	M	13	1	18.3	3.2	155	1.19	0.76	98.86	1.4	high	8	6.1	0.05	36	10.9
PS	62	F	10	1	18.8	4	97	1.89	1.07	128.3	1.5	high	7.5	3.8	3.53	28	9.2
PS	82	F	10	1	21.2	3.2	118	1.16	0.83	98.78	1.1	high		5.6	0.97	28	11.6
PS	66	M	10	1	18.9	3.9	156	1.46	1.13	108.6	2.1	high	8.9	5.9	0	28	11.7
PS	79	F	8	1	19.2	3.8	205	1.34	0.85	112.3	1.1	high		5	0.64		12.7
PS	59	M	8	1	29.7	3.7	118	0.99	0.87	98.68	1.5	high		3.4	0.2	20	9.8
PS	61	M	8	1	19.7	3.5	155	0.85	1	125.1	1.6	high	9.4	6.3	0.06	11	10.9
PS	71	F	7	1	30.7	3.2	162	1.43	0.65	75.47	1.3	high	8.8	3.6		26	9.8
PS	74	M	7	1	25.4	3.5	195	2.12	0.84	139.8	1.4	high	8.9	5.1	0.05	24	9.6
PS	66	F	7	1	20.3	3.5	124	1.03	0.79	94	1.3	high	7.7	2.2			11.4
PS	68	F	6	1	21.7	3.5	132	1.22	1.03	128.3	2.1	high	8.4	7.2	0.1	20	11.1
PS	34	M	6	1	22.2	3.7	130	1.61	1.14	123.1	1.6	high	9.7	6.1	0.02	25	11.2
PS	69	M	6	1	21.9	3.2	153	1.2	1.05	91.36	1.1	high	8.2	4.2	3.7	18	10
PS	51	F	5	1	20.8	3.7	155	1.62	1	82.6	1.3	high	8.1	6.9	0.1	28	10.9
PS	68	F	4	1	29.9	3.5	150	1.45	0.85	81.04	1.5	high	9.1	6.3	0.02	25	12.5
PS	80	M	4	1	25.3	4	226	1.75	1.04	90.31	1.5	high	8.8	5.1	0.07	31	10.3
PS	47	M	3	1	34.1	3.9	184	1.46	0.91	123	1.3	high	9.3	1.2	9.1	40	8.8
PS	70	M	3	1	18.4	4	113	1.06	0.72	99.3	1.5	high		8.4	0.1		11.1
PS	70	F	3	1	18.5	3.4	152	1.54	1.01	92.94	1.5	high		4.9	0		10.6
PS	77	F	3	1	22.7	3.5	178	1.94	0.76	113.2	1.1	high		7.2	0		10.5
PS	61	F	3	1	25.1	3.6	180	2.12	0.88	68.21	1.5	high	9.3	5.2		22	10.6
PS	69	F	3	1	24.9	4	192	1.4	0.89	89.66	1.5	high	9.1	3.8		20	10

PS	44	M	2	1	45.7	3.7	151	1.15	0.66	50.05	1.6	high	9.6	6.3	0.3	41	10.2
PS	55	M	2	1	23.8	3.9	147	1.03	1	84.77	2.1	high	8.4	5.7	0.09	52	11.4
PS	68	M	10	1	24.8	3.4	124	1.81	1.16	117.9	1.5	high	9.8	8.3		42	8.8
PS	59	F	2	1	37.9	3.9		2.02	0.69	58.05	1.3	high	8	5.1	0.03	28	10.8
PS	52	M	11	1	26.4	4	147	2.04	1.02	104.5	1.3	high	7.8	5.2	0.82	37	8.2
PS	60	M	34	1	18.9	4	83	1.3	1.13	105.8	1.6	high		3.2	0.1	28	10.3
PS	54	M	34	1	24.4	3.3		1.94	0.75	72.39	1.6	high	9.4	6.8	0.1		11.6
PS	44	M	30	1	17.2	3.9		1.78	1.13	96.37	1.6	high	9.6	4	0.2		7.9
PS	48	F	23	1	18.5	3.6	148	1.63	1.15	99.37	1.9	high	9.3	3.3	0.14	21	10.9
PS	68	F	22	1	18.9	3.7	174	1.52	1.25	114.4	1.5	high		7.3	0.05	34	10.3
PS	56	F	21	1	26.6	3.8	170	1.34	0.94	124.2	1	high	8.6	5.1		20	11
PS	54	M	19	1	20.2	4.2	146	1.32	0.48	68.81	1.9	high	9.3	4.2	4.27	25	8.5
PS	57	F	17	1	17.4	3.7	190	1.39	0.9	115.5	1.6	high		6.1		32	9.6
PS	57	F	15	1	20.4	3.5	119	1.6	0.92	119.1	1.9	high	10.2	5.7		28	11.6
PS	61	M	14	1	18.5	4	149	1.47	0.96	92.58	1.8	high	8.6	4.3			11.1
PS	58	M	14	1	22.4	3.8		1.52	0.97	113.2	1.9	high	7.8	5	0.1	21	10.8
PS	40	M	12	1	19.5	4	136	1.39	0.88	110.9	1.5	high	9.1	4.3	0.04	26	10.1
PS	47	F	11	1	21.5	3.7		1.49	1.05	112.1	1.9	high	9.4	6.5	0.1	26	11.4
PS	69	F	11	1	21.7	4		1.19	0.97	115.8	1.6	high	8.8	5.7	0.1	27	10.7
PS	73	F	10	1	16.5	3.8	129	1.46	0.93	103.7	1.5	high		5.1	0.17	31	11.3
PS	83	F	10	1	14.2	3.3		1.56	0.82	106.7	1.6	high	8.9	3.6	0.1	29	10.7
PS	51	F	10	1	24.7	3.5	159	1.66	1.15	109.7	1.3	high	8	4.2	0.7	28	10.1
PS	57	F	9	1	22.7	3.7		1.77	0.88	113.8	1.6	high	9	6.9	0.1	22	11.3
PS	65	M	9	1	23.2	3.7	157	1.19	1.02	104.4	1.8	high	7.9	4.5	0.23	28	10
PS	49	F	8	1	23.5	3.8		1.38	0.85	86.09	1.6	high	7.9	4.6	0.1	25	10.8
PS	76	M	8	1	20.7	3.6	163	1.15	0.91	101.4	1.8	high	8.3	5.2	0.18	22	10.3
PS	66	F	7	1	32.9	4.1	109	1.26	0.55	84.05	1.8	high	8.1	5.4		25	10.6
PS	61	F	7	1	22	3.8	182	1.26	0.81	74.17	2.1	high	9	5.9	0.7	25	9.7
PS	44	M	6	1	21.1	2.8	113	1.66	1.2	46.15	1.6	high	9.4	7.1	0.02	29	11.2
PS	65	F	5	1	17.3	3.2	146	1.62	0.93	89.91	1.6	high	8.8	4.6	0.29	30	10.4
PS	79	M	5	1	22.8	3.3		1.24	0.94	96.4	1.9	high	8.9	5.9	0.4	29	10
PS	55	F	5	1	20.4	3.8		1.43	0.92	91.99	1.6	high	9.4	5.2	0.1	24	9.6
PS	43	F	4	1	18	4.4		1.35	0.87	106.5	1.9	high	8.6	4.9	0.1	25	10.5
PS	73	F	3	1	20.6	4	99	1.11	0.81	104.6	1.5	high		5.5	0.6	16	9.4
PS	39	F	3	1	28.6	4.1	193	1.58	0.66	29.97	1.5	high	8.8	6.3	0.2		9.9
PS	82	F	3	1	20.1	3.7	124	1.97	0.88	111.7	1.5	high	10.3	2.6	7.88	25	10.4
PS	75	F	3	1	21.2	3.5	130	1.32	0.99	106.9	1.5	high	9.2	6.1		30	9.6
PS	56	M	2	1	22.6	4.2		1.39	0.7	71.97	1.6	high	9	3.7	0.1	25	10.7
PS	39	M	2	1	22.3	4.2	193	1.63	0.8	107.2	2.1	high	9.6	7.8	0.1	24	11.1
PS	56	F	2	1	21.2	3.8	250	2.33	0.94	86.97	2	high	9	5	1.1		9.4
PS	59	F	2	1	28.8	3.6	113	1.24	0.98	116.1	1.3	high	8.4	3.2	0.29	24	8.4
PS	75	M	21	1	20.5	3.7	176	1.8	1.08	101	1.1	high	8.3	7.4			7.5
PS	64	F	9	1	27.1	3.4	137	1.13	0.7	81.23	1.3	high	8.5	5.6		29	9.7
PS	63	F	7	1	22.2	4.1	129	1.18	0.82	123.8	1.5	high		6.7	0		11.1
PS	72	F	7	1	25.4	3.7	114	1.73	0.88	119.8	2.1	high	7.8	6.1		17	11.9
PS	44	F	6	1	20.8	3.9	189	1.69	0.92	101.6	1.5	high	9.3	6.1	0.05	37	11.4
PS	59	M	5	1	18.7	4.2		1.37	0.76	96.67	1.6	high	8.4	4.5	0.1	19	10.6
PS	54	M	2	1	22.9	3.9	102	1.57	1.49	100	2.1	high	10.2	5.6	1.42	35	10.5
PS	68	F	2	1	20.8	4.2	173	1.58	0.89	114.8	2.1	high	9.5	6.7	9.2	26	11.8
PS	57	M	2	1	23.5	3.7		1.29	0.79	77.22	1.5	high	8.8	4.6	0.04	30	9.6
PS	80	F	2	1	25.1	3.5	196	1.61	0.66	71.6	1.8	high	10.9	5.8		52	10.8
PS	78	F	15	1	21.2	3.5	81	1.31	0.73	117.7	1.5	high		4.6	0.13		9.6
PS	68	M	7	1	16.6	3.9	175	1.91	0.98	75.45	1.3	high	9.6	5.1	0.2		11.4
PS	71	F	4	1	21	3.9	136	1.7	1.16	93.61	1.5	high	9.6	5.8	0.22		10.7
PS	53	M	10	1	18.1	3.9	200	1.95	0.79	141.2	1.5	high	9.1	5.7	0.15		8.9
PS	81	M	5	1	28.7	3.7	143	1.66	0.71	123.3	1.5	high	9.3	3	0.37	26	10.6
PS	59	F	11	1	17.9	4.1	164	1.39	1.01	109.9	1.3	high	10.6	5.2	0.1		11.9
PS	73	M	3	1	20.4	3.4	137	1.08	0.8	88.32	2.1	high	8.6	7.3	0.07	26	9.5
PS	31	M	3	1	20.2	4.1	137	1.18	0.89	85.99	2.1	high		5.3	0.05	34	10.1

PS	35	F	2	1	23.2	3.9	128	1.41	0.82	65.2	2.1	high		3.8	0.05	24	7.4
PS	79	M	7	1	20	3.3	149	1.53	0.7	107.8	1.6	high	9.1	5.8			
PS	60	M	31	1	17.6	4.1	177	1.52	0.89	67.02	1.6	high	10.1	3.5		20	11.1
PS	87	M	4	1	21.4	3.6	157	1.36	0.71	90.13	1.5	high	10.8	5.3	0.1		10.5
PS	70	F	6	1	19.1	4	138	1.6	1.03	87.57	2.1	high	8.8	5.1	0	36	11
PS	74	F	4	1	19	3.3	142	1.58	0.93	95.86	1.5	high	8.1	3.6	0.22	31	9.8
PS	64	F	20	1	18.8	4		1.36	0.82	112.8	1.6	high	8.1	5.8	0.2	26	10.5
PS	77	F	10	1	20	3.5	131	1.53	0.79	91.26	1.3	high		5.3	0.09	31	10.3
PS	82	F	2	1	21	3.8	181	2.36	0.63	54.68	1.6	high	9.4	5.6	0.46	32	9.9
PS	71	F	9	1	18.3	3.3	108	1.14	0.94	135	1.8	high	8.6	5.1	1.51	15	14.3
PS	53	F	3	1	31.6	4	190	1.11	0.59	102	2.1	high		3.4	0.11	32	10.1
PS	82	F	4	1	19.7	3.8	189	1.88	0.85	126.3	2.1	high	8.8	4.8			11.8
PS	64	M	8	1	18.1	3.4	131	1.6	0.94	77.17	1.5	high		5.4	1.4	22	10
PS	60	F	15	1	14.5	3.4		1.58	0.81	59.33	1.6	high	9.1	4.1	1.4	24	10.3
PS	69	F	15	1	18.1	4	108	1.55	0.79	117.3	2.2	high	9.4	6.1	0.19	26	10.1
PS	46	M	24	1	17.8	3.8	170	2.12	0.74	97.15	1.3	high	10.4	5	0.01		11.2
PS	63	F	7	1	19	4.3	147	1.58	0.98	102.1	1.1	high	8.4	4.7	0.21	24	
PS	53	M	7	1	25.1	3.3	146	1.34	0.72	102.6	1.3	high		5.1	0.3	32	9.6
PS	71	F	10	1	19.2	3.9	183	1.13	0.93	94.21	1.3	high	10.3	7.1	0.4	29	7.3
PS	55	M	2	1	19.6	4	157	1.56	0.8	88.41	2.1	high	8.7	6.5	1.11	30	5.5
PS	67	F	2	1	29.2	3.9	154	1.13	0.67	104.6	1.8	high	10	6.8	0		10.9
PS	65	F	6	1	19.5	4	103	1.4	1.01	131.9	1.6	high	8.9	3.5	0.03	22	11.5
PS	55	M	30	1	17.9	3.6	165	1.48	0.87	107.5	1.8	high	8.9	5.2	0.5		8.6
PS	49	M	28	1	16.3	3.6		1.39	0.83	82.36	1.1	high	8.8	5.3	6.9	29	9
PS	70	M	25	1	20.5	4	102	1.28	0.91	94.75	0.8	high	9.4	5.3	0.2	38	11.3
PS	50	M	25	1	19.7	4.1	81	1.21	0.78	105.8	2	high	9.1	4.7	5.2		10.1
PS	43	F	23	1	19.1	3.8	148	1.08	1.29	118.3	2.5	high	9.5	4.4	0.05	25	10.7
PS	33	F	20	1	19.4	4.1	160	1.38	0.99	134.7	1.3	high	7.9	4.2	0.78	50	10.9
PS	43	F	18	1	20.9	4	225	1.36	1.06	125.6	2	high	8.8	3.9	0.31	25	11.1
PS	59	M	16	1	18.5	4		2.04	1.08	116.7	1.6	high	10.1	7	0.2	23	11.3
PS	68	M	15	1	18.4	4.1	113	1.84	0.82	119.2	1.8	high	8.7	5.4			11.1
PS	66	M	15	1	17	3.8	151	1.84	1.06	111.5	1.5	high	8.6	4.4			9.8
PS	72	M	15	1	22.3	3.9	165	1.69	0.91	113.7	1.8	high	8.2	5			9.2
PS	67	M	13	1	20.5	3.8	152	1.92	1.19	137	1.5	high	8.6	4.6			10.2
PS	56	M	13	1	17.8	4.1	234	1.43	1.15	99.45	1.3	high		3.8		23	9.5
PS	64	M	13	1	20.3	3.8	153	1.85	0.63	101.3	1.5	high	10.3	5.8	0.1	36	11.1
PS	53	F	11	1	19.4	4	180	1.23	1.03	92.4	1.3	high	8.9	4.5	0.2	23	11.4
PS	62	M	11	1	20.6	3.4	163	1.47	0.75	78.99	2.1	high	8	9.1	0.93	25	9.6
PS	76	M	10	1	17.2	3.7	181	1.53	0.93	98.91	1.8	high	8.9	5.5	0.1	26	9.1
PS	68	M	9	1	20.3	3.9	155	1.69	0.96	69.44	1.5	high	8.2	3.4		24	
PS	58	M	8	1	27.3	3.8	95	1.02	0.77	109.8	0.8	high	9	5.7	1.6	32	9.7
PS	57	F	8	1	18.4	3.4	196	1.22	0.71	152.9	1.8	high	9.9	3.6	0.07	22	10.5
PS	67	M	7	1	23.7	3.9	128	1.16	0.86	135.2	1.5	high	8.8	10	0.02	27	11.7
PS	60	M	6	1	27	4.2	161	1.16	0.98	120.9	2.5	high	8.4	8.5	0.22	28	9.4
PS	59	M	5	1	24.9	3.7		1.23	0.69	87.97	1.5	high	9.1	4.5	0.03		9.6
PS	72	M	2	1	21.1	3.7	134	1.41	0.61	58.38	1.5	high	9.1	3.9	0.1	22	10.4
PS	75	M	14	1	23.6	3.7	181	1.44	0.64	100.7	1.8	high	8.8	7.4	0.3	31	11.8
PS	54	M	11	1	24.5	3.6		1.59	0.96	121.2	1.5	high		5.2	0.34	28	8.7
PS	44	M	9	1	24.6	4.1	105	1.25	0.54	95	2.1	high	8.1	5.4		45	9.7
PS	69	M	8	1	19	3.6	148	1.22	1.14	139.3	1.5	high	7.8	8.8	0.06		10.2
PS	65	M	7	1	29.7	4.2	159	1.67	0.87	102.6	1.8	high	9.5	6.6	7.63	35	7
PS	47	M	4	1	24.5	4.2	124	1.78	0.72	120.6	1.5	high	8.8	4.2			9
PS	44	M	16	1	20.6	4.4		1.11	0.85	135.6	1.6	high	9.4	3.8	0.1	27	9.7
PS	52	M	11	1	17.4	3.9	149	1.5	0.86	111.3	1.5	high	8.3	5.7			13.5
PS	52	M	10	1	19.9	4	165	1.44	0.85	106.2	1.8	high		6.5		21	10.7
PS	60	M	9	1	19.8	4.3	74	1.44	0.71	142	1.5	high	8.8	4.3			9.7
PS	45	M	3	1	21	4	152	1.58	1.05	107.9	2.1	high	8.7	5.7			11.3
PS	60	M	11	1	18.4	3.7		1.42	1.02	108.4	1.6	high	8.7	5	0.1	13	10.8
PS	52	M	3	1	27.8	4.4		1.36	1.14	130.4	1.9	high	9.6	6.7	0.1	25	11.2

PS	48	M	2	1	24.8	4.2	118	1.13	0.88	110.4	1.5	high	10	4.4	1.33	24	8
PS	67	F	7	1	22.4	3.7	142	1.2	1.03	112.6	1.8	high	8.9	6.3	0.13	27	11.1
PS	73	M	4	1	16.2	3.4	192	1.47	0.69	104.1	1.6	high	8.8	2.6	0.6	29	10.1
PS	60	M	16	1	19.5	4	93	1.36	1.04	92.3	1.3	high	9.2	2.9	1.72	18	8.9
PS	70	M	19	1	21.2	3.9		1.36	0.98	120.3	1.9	high	8.3	4.7	0.2	30	11
PS	77	M	13	1	20	4		1.28	0.75	110.8	1.6	high	9.6	1.6	0.1	26	10
PS	82	F	4	1	21.5	3.8		1.59	0.7	94.57	1.9	high	10.9	6.8	0.2	24	10.1
PS	72	M	15	1	19.1	4.3	105	1.07	1.06	132.4	1.4	high	8.5	4	0.07	31	10.5
PS	54	M	5	1	30.4	4.2	115	0.64	0.91	131.5	1.1	high	8.1	4.6	2.7		7.7
PS	60	F	3	1	17.2	3.8	208	1.85	0.94	87.6	2.1	high	9.6	6.8	0.1	29	9.9
PS	81	M	3	1	20.4	4		1.46	1.03	81.06	1.9	high	9.9	4.5	0.2		12.3
PS	57	M	12	1	24.5	3.7		1.41	0.88	119.9	1.6	high	9	4.3	0.1	23	12
PS	55	F	9	1	22	3.9		1.33	0.46	87.34	1.6	high	9.2	4.6	0.2	32	11.6
PS	65	M	4	1	30.7	4.2	195	1.17	1	111.8	1.5	high		7.3	5.87	30	10.6
PS	74	M	5	1	23.8	4		1.48	0.74	122.8	1.9	high	9	5.1	0.1	22	9.8
PS	74	M	2	1	24.9	4	203	1.87	0.81	99.03	1.3	high	8.4	3.4	0.08	21	8.7
PS	58	M	4	1	24	4.1	105	1.24	0.9	66.3	2.1	high		4.8	0.15	34	9.4
PS	44	F	32	1	19.8	4.2	194	0.89	0.66	131.4	2.1	high	9.8	7.4	0.04	24	10.5
PS	36	F	3	1	24.5	4.2		1.49	1.08	111.8	1.6	high	8.1	4.3	0.6	30	8.6
PS	46	M	10	1	19.8	3	175	1.59	0.79	81.96	1.6	high		4.7	0.43	29	9.2
PS	33	F	12	1	22.4	3.7	193	1.82	0.83	117.1	1.8	high	9.4	6.2	0.1	23	11.2
PS	29	M	11	1	21	4.6		1.33	0.84	101.9	1.9	high	10.3	5.6	0.1	26	11.1
PS	45	M	9	1	18.8	4.3	105	1.11	0.85	116.5	1.9	high	9	4.8	0.1	30	11.2
PS	75	F	8	1	17.4	3.8	106	1.41	0.89	67.15	1.5	high	7.9	8.3	0.1	21	9.6
PS	78	M	7	1	16.7	4.1	177	1.15	0.75	87.52	1.8	high		6.7	0	21	12
PS	69	M	7	1	24.7	4.2	176	1.17	0.93	113.7	1.9	high	9.5	4.9	0.01	24	10
PS	75	F	3	1	26.5	4.2		1.28	0.84	116.3	1.5	high	8.5	4.9	0.05		10.7
PS	69	M	27	1	20.3	3.7	173	1.2	1.01	122.7	1.9	high	9.6	5.6	0.02	26	11.2
PS	71	F	26	1	19.5	3.9	131	1.39	1.06	108.9	1	high	8.4	5.6	0.57	33	10.8
PS	60	M	8	1	23.6	4.3	162	1.32	0.85	114.5	1.5	high	8.2	4.4	0.16	35	9
PS	66	M	3	1	22.1	4.1	187	1.51	0.68	60.1	1.5	high		5.5			9
PS	61	M	6	1	25.1	4.2	163	1.18	0.84	121.3	1.5	high	8.7	5.2	3.39		8.7
PS	59	M	2	1	22.3	4.2	164	2.54	0.9	99.82	1.5	high	8.6	5.7	0.1	25	10.6
PS	81	M	13	1	17.6	3.9	138	1.41	0.75	135.1	2.2	high	9.9	6			10.1
PS	55	M	9	1	18.5	4.2	99	1.62	0.91	111.3	1.5	high	9.3	5.4	0.05	26	9.8
PS	77	M	10	1	20.6	4.3	105	1.23	0.76	89.3	2.1	high	8.6	6.3	0.1	29	9.8
PS	84	M	7	1	19.3	4	123	1.25	0.79	106.4	1.9	high	9	4.2	0.07	38	12.6
PS	63	M	17	1	19.4	4	130	1.31	1.09	101.5	1.9	high	9.4	4.5	0.23	21	12.3
PS	51	M	10	1	23.8	4.1	219	1.23	0.78	108.5	1.8	high	8.7	3.7	0.04	26	10
PS	50	F	12	1	20.3	4.5	230	0.9	0.89	99.23	1.6	high		6.5			9.6
PS	81	M	10	1	21.8	3.3	118	0.94	0.65	67.03	2.2	high	8.7	7.5	0.16	24	11.4
PS	63	M	6	1	20.7	3.8		1.3	0.91	107.4	2.1	high	9.2	5.3	0.08	25	9.6
PS	65	M	5	1	19.9	4.6	128	1.37	0.83	96.41	1.9	high	10.4	4.6	0.04	28	11.3
PS	63	M	6	1	20.1	3.9		2.08	0.76	112.4	1.3	high	9.6	4.9	0.02	24	10.6
PS	67	F	4	1	17.4	3.7	179	1.04	0.94	85.9	1.3	high	9.5	4.7	0.4	28	11.9
PS	74	M	35	1	21.3	3.4	159	1.47	0.77	57.18	1.8	high	10.1	7.1	0.1		12.2
PS	64	M	26	1	17.9	3.9	134	1.49	0.82		1.9	high	8.9	3.8	0.08	31	11.2
PS	68	F	16	1	20.2	4.6	119	1.51	0.71	93.35	2.1	high	9.3	4.3	0.21	20	10
PS	68	M	13	1	23.5	3.9	151	1.31	0.91	124.4	1.9	high	8.8	4	0.03	25	11.5
PS	42	M	9	1	17.4	4.1	78	0.66	1.12	118	2.2	high	9	7.8	0.05	30	9.9
PS	61	M	5	1	24.8	4.6	222	1.21	0.78	122.9	1.9	high	9.8	5.6	0.11	28	12
PS	52	F	26	1	27.3	3.8	183	1.15	0.97	103.9	1.9	high	9.7	5.6	0.05	28	11.2
PS	68	M	11	1	25.6	4.5	139	1.71	0.76	118	1.3	high	7.7	2.7	0.08	22	10.6
PS	69	F	16	1	20.1	3.6		1.13	0.81	130.4	1.4	high	8	4	1.59	27	9.1
PS	69	F	20	1	21	3.8	83	1.33	0.75	107.9	1.5	high		6.5	0.16	37	9.3
PS	63	M	16	1	22.8	4.1	181	1.62	0.62	93.06	1	high	8	6.1	1.25	29	10.7
PS	61	M	10	1	19.8	3.7		1.65	1.08	106.9	1.5	high	8.2	4.5		34	8.4
PS	81	M	8	1	24.9	3.7	136	1.23	0.97	124.3	2.1	high		6.4		35	9.8
PS	49	M	7	1	19	3.7	140	1.7	1.01	102.9	1.8	high	8.6	4.9			9.9

PS	44	M	6	1	26.7	4.2	168	1.62	0.76	108.8	2.1	high	11.1	3.9	0.07	24	7.9
PS	35	M	5	1	27	4.4	132	1.39	0.85	101.6	1.9	high	9.5	3	0.05	21	10.4
PS	74	M	3	1	18.7	4.2	182	1.65	1.02	100.5	1.6	high		5.5	0.05	22	9.5
PS	60	M	3	1	25.9	4	141	1.34	0.88	102.7	1.5	high	8.9	5.8	0.46	28	10.6
PS	78	M	7	1	16.8	3.4	152	1.48	0.56	92.54	2.1	high		6.1	1.07	35	10.5
PS	55	F	18	1	24.4	3.4	195	1.78	0.53	91.74	1.5	high		3.3		25	10.7
PS	51	M	17	1	21.3	3.7	161	1.25	0.96	110.3	1.1	high	8.6	3.5	1.98	36	10.6
PS	57	M	15	1	24.7	3.8		1.41	1.01	118.2	2.1	high	8.5	6.2	0.18	26	10.5
PS	62	F	15	1	22	4	115	1.49	1.06	100.3	1.3	high	8.6	5.3	0.3		10.9
PS	53	F	14	1	19.1	4.2	126	1.27	1.02	89.97	1.9	high	9.4	6.1	0.14	23	12
PS	59	F	13	1	17.7	3.6	148	1.27	1.36	100.4	1.9	high	8.7	6.2	0.05	29	
PS	76	F	12	1	22.7	3.7	157	1.48	0.74	130.9	1.5	high	10.3	4.2	0.2	24	12.9
PS	48	M	11	1	21.8	3.9	135	1.6	0.82	110.9	2.1	high	8.9	6.2	1	32	11.7
PS	31	M	8	1	28.2	3.9	206	1.57	1.01	114.1	2.1	high	9.5	5.2	0.1	14	8.3
PS	71	F	8	1	37.6	4.1	203	1.39	0.95	86.63	1.9	high	9.3	5	6.81	25	10.7
PS	79	F	8	1	24.8	4.1	94	1.21	0.92	121.3	1.9	high	9.6	5.6	0.34	33	10.1
PS	41	M	8	1	35.8	4.1	131	1.53	0.73	87.11	1.8	high	7.1	6.8	0.14	26	11.3
PS	82	M	8	1	19.5	4.2	119	1.05	0.81	139.8	1.9	high	8.5	5.9	0.04	48	9.7
PS	38	M	8	1	25.3	4.4	106	1.5	0.86	133.8	2.5	high	8.8	9.6	0.1	26	11
PS	65	M	8	1	21.3	3.7	133	1.34	0.73	115.5	2.1	high	9	5.2	0.19	28	10.3
PS	80	F	7	1	27.8	3.8	115	1.92	0.78	128	2.1	high	10.1	5.2	1.44	33	8.6
PS	72	M	7	1	23	3.9	129	1.69	0.75	117.3		high	8.7	5.3	0.26		
PS	42	F	7	1	29.1	3.8		1.63	0.87	116.6	1.5	high	8.5	4.9	0.02	19	11.6
PS	51	F	5	1	26.5	4	144	1.67	0.98	77.91	1.8	high	8	6.6	0.04	33	10.2
PS	70	M	5	1	18.4	3.8	234	1.7	1.2	102.6	1.9	high	9.2	4.4	0.09	29	10.7
PS	61	M	4	1	24.8	4.1		1.79	0.82	115.1	1.5	high	9.8	4.7	0.01	24	11.9
PS	71	F	3	1	25.4	3.8		2	0.75	100.4	1.5	high	8.6	5.5	0.1	25	11.3
PS	56	M	2	1	18.9	4.4	108	1.07	0.49	28.25	2.1	high	9.9	3.3			8.9
PS	61	F	2	1	22.2	4.3	189	0.97	0.63	36.97	1.9	high	9.6	4.1	0.1	22	11.5
PS	48	M	29	1	20	4	155	1.66	0.76	68.37	1.3	high	9.4	4.5	0.03		9.8
PS	62	M	21	1	20.7	3.9		2.37	0.92	76.12	1.3	high		5.1	0.04	34	11.1
PS	70	M	20	1	19.4	4.4	147	2.15	0.94	93.66	1.3	high	9.8	4.2	0.02		10.6
PS	64	M	20	1	21.4	3.7	131	1.28	0.68	110.1	1.9	high	8.7	6.4	0.11	27	11.7
PS	73	M	18	1	23.8	4.1	128	1.39	1.02	88.03	2.1	high	7.8	5.4	1.1	20	11.2
PS	46	M	18	1	17.5	4.1	120	1.04	1.09	107.9	1.5	high		8.6		26	8
PS	60	M	17	1	20.4	4		1.81	1.2	105.2	1.5	high		2.3	1.12	27	9.9
PS	30	M	16	1	22.2	4.4	145	1.33	1	98.8	1.9	high	8.9	4.8	0.02	21	11.2
PS	65	F	15	1	16.6	4.2	148	1.82	1.11	116.8	2.1	high	9.7	6.6	0.1	24	10.2
PS	72	F	12	1	17.5	3.7	126	1.56	1.28	83.97	2.1	high	8.7	5.9			10.5
PS	56	F	9	1	19.4	4.1	108	1.26	1.21	109.9	1.5	high	9.1	4.1	4.3	31	9
PS	51	M	9	1	25.7	4.1	177	1.65	0.91	99.02	1.5	high	9	4.7	0.05	26	10.2
PS	74	F	9	1	17.3	3.9	147	1.57	1.06	90.33	2.1	high	10.3	4.6	0.1	26	11
PS	61	M	7	1	17.3	4	182	1.32	0.99	84.76	1.4	high		5	0	19	8.5
PS	62	F	5	1	20.9	4.5	156	1.28	0.74	103.4	2.1	high	8.6	5.8	0.1	34	11.4
PS	40	F	4	1	21.6	4	155	1.15	1.03	115.5	1.9	high	8.6	6.4	0.05	31	10.1
PS	77	F	4	1	20	3.7		1.27	0.99	87.82	2.1	high	8.7	5.3	0.23	30	11.2
PS	52	M	4	1	24	4.4	122	0.95	1.02	130	1.3	high	8.9	4.7			10.5
PS	57	M	4	1	24.4	4		1.53	0.87	84.24	1.5	high		6.6	0.1	26	11.6
PS	80	M	4	1	21.4	4.5	162	1.34	1.23	109.6	2.1	high	8.5	4.5			10.4
PS	69	M	3	1	27.3	4	135	1.58	0.58	81.93	1.6	high	8.8	5.9	0.05	27	10.3
PS	39	M	3	1	27.3	4.4	175	1.54	1.06	83.52	1.6	high	9.9	7.6	0.1	31	6.7
PS	73	M	2	1	20.8	4.2	167	1.55	0.86	75.96	2.1	high	9.3	6.2	0.02	23	11.6
PS	51	M	26	1	18.1	3.2	107	1.24	0.86	83.66	1.1	high	8.1	5.5	0.16	25	11.2
PS	77	F	16	1	17	3.8	187	1.4	0.97	117.3	1.9	high	8.7	7.4	0.17	35	11.3
PS	57	F	15	1	16.6	4	185	1.36	1.04	99.29	1.9	high	9.3	5.8	0.02	38	9.9
PS	63	F	14	1	17	3.3	148	1.17	0.97	138.7	1.9	high	9	5.2	0.31	30	11.3
PS	71	M	11	1	18.4	3.2	126	1.68	0.63	98.08	1.9	high	9.8	4.6	0.04	27	10.9
PS	59	F	10	1	18.4	3.8	204	1.55	1.01	120.3	1.3	high	9.8	5	0.4	31	9.6
PS	63	F	9	1	21.8	4	176	1.27	0.85	110.4	1.9	high	9.6	4.4	0.02	25	10.9

PS	76	F	9	1	18.2	3.6		1.16	0.84	158.5	2.5	high	9.1	4.5	1	27	12.7
PS	75	M	9	1	23.2	3.5	205	1.12	0.94	101.4	1.8	high	9.4	6.3	0.05	20	11.8
PS	62	M	8	1	23.8	3.5	145	1.32	0.9	150.6	2.1	high	9.1	7			10.3
PS	76	M	7	1	22.3	3.7		1.21	0.76	140.9	1.5	high	8.3	4.2			6.9
PS	67	M	7	1	20.1	3.7	157	1.56	0.72		1.5	high	8.6	4.8			10.2
PS	67	M	6	1	20.1	4	114	2.03	0.82	111.6	1.8	high	8.4	5.2			8.2
PS	67	M	6	1	23.4	3.9	133	1.74	1.03	126.9	2.1	high	8.5	4.7			10.9
PS	62	M	6	1	20.5	3.8	174	1.5	1.08	120.8	1.9	high	9.2	6.4			11.9
PS	68	F	6	1	20.3	4	120	1.56	1.1	122.2	2.1	high		7	0.03	28	10
PS	56	M	5	1	18.9	4.1	110	1.61	0.8	54.22	2.1	high	7.7	4.3		18	12.2
PS	73	F	5	1	23.2	3.9	162	1.54	0.87	95.91	1.6	high	8.9	5.7	0.1	30	11.3
PS	73	M	5	1	21	4.2	155	1.35	0.81	105.4	2.1	high	9.5	6.4	0.3	20	10.7
PS	68	F	4	1	23.2	4.2	159	1.43	0.89	103.5	1.5	high	8.6	4.4	0	10	
PS	72	M	4	1	21.5	4	110	1.06	1.09	102.9	1.8	high	9.3	6.6	0.3	57	8.3
PS	61	M	3	1	23.9	3.7	90	1.46	1.13	131.5	1.5	high	7.4	4.5	1.01	31	9.9
PS	77	M	3	1	22.3	3.4	164	1.48	0.88	98.75	2.1	high	10.1	6.3	0.3	26	11
PS	65	F	3	1	24.7	3.8	111	1.65	0.48	65.02	1.5	high		3.7	1.86	27	10.4
PS	47	M	2	1	25.1	4.3	72	1.35	0.91	106.3	1.5	high	8.5	5.5			10.3
PS	58	M	2	1	27.4	4	134	1.51	1.04	96.8	1.5	high	8.2	3.3	1.28	30	9.4
PS	31	M	2	1	19.5	3.8	182	1.31	0.73	57.04	1.8	high	8.7	6.6			12.5
PS	59	M	2	1	18.8	4	178	1.76	0.72	44.46	1.9	high	9	7.3			10.3
PS	68	M	2	1	19.6	3.8	145	1.27	1	79.67	1.9	high	9.1	6.4			9.5
PS	62	M	2	1	26.9		118	1.42	0.62	58.27	1.6	high	7.7	3.4	0.38	34	10.2
PS	61	M	21	1	20.2	4.1	124	1.46	0.82	102.8	1.9	high		5.8	0.18		9.5
PS	59	M	23	1	18.8	3.9	142	1.55	0.77	103.6	1.6	high	10.8	5.3		17	10.2
PS	58	M	19	1	22.2	4	113	1.56	0.97	121.3	1.4	high	8.9	3.2	0.02	23	12.3
PS	63	M	19	1	21.2	3.7	185	1.54	0.94	134.2	1.8	high	9.4	4.2	0.01	32	10.4
PS	54	F	16	1	16.9	3.5		1.39	0.9	117.7	1.6	high	8.4	6.4	1.86	26	10.6
PS	59	M	14	1	19.5	3.9		1.69	0.86	106	1.6	high	9.4	5.1	0.03	31	10.4
PS	63	F	13	1	19.2	3.6		1.65	0.75	139.2	1.6	high	10.6	3.1	0.34	34	9.6
PS	66	M	12	1	21.7	3.7		1.25	0.78	85.73	1.6	high	8.8	5.6	0.13	23	10.8
PS	48	M	12	1	20.2	4.1	155	1.87	0.77	110.1	1.4	high	9.2	4.9	0.05	30	10.3
PS	71	F	9	1	19.2	3.2	83	1.29	0.89	115.8	1.8	high	10.5	3.2	1	31	9.2
PS	71	M	8	1	18.4	3.2		1.65	0.88	113.4	1.6	high	8.8	6	1.11	36	10.1
PS	54	M	8	1	25.2	3.6	114	2.46	0.97	122.2	2.5	high	9.6	4.4	0.1	21	11.8
PS	55	M	8	1	21.8	4	152	1.35	1.18	146.9	1.4	high	8.1	5.8	0.06	23	10
PS	65	F	8	1	15.7	3.2	213	1.75	0.96	94.58	1	high	9.6	5.3	0.05	21	11.9
PS	65	M	7	1	24.6	4	117	1.67	0.88	80.69	1.3	high	8	2.4	0.2	28	10.7
PS	69	F	7	1	23.2	4.1		1.75	0.76	112	1.6	high	8.7	3.4		33	9.3
PS	45	M	7	1	21.1	4.1	119	1.29	0.63	62.89	1.4	high	7.8	5.1	1.08	27	10.6
PS	76	M	6	1	23.7	3.4		1.03	0.58	131.3	1.8	high		6.1			10.2
PS	66	F	6	1	23.3	4		1.81	0.89	65.83	1.6	high	9.4	7.1	0.06	25	10.8
PS	58	M	6	1	20.5	3.4	158	1.92	0.97	101	1.8	high	8.2	5.3	0.05	22	10.4
PS	71	F	5	1	18.8	3.7	172	1.26	1.02	97.02	1.5	high	9.4	7.4	1.8	29	10.3
PS	64	M	5	1	17.8	3.6	117	1.3	0.72	80.96	2.1	high	9.3	8.8	0.05	54	11
PS	53	F	4	1	22.6	3.5	186	1.1	0.7	117	1.8	high	8.8	6.1	0.21	30	10.2
PS	72	M	4	1	24.5	3.9	132	1.52	0.76	105	1.5	high	9.4	3.5	0.3	31	9.8
PS	74	M	3	1	25.4	3.9	143	1.33	0.84	118	1.5	high	8.4	4.2	0.3	23	10.4
PS	34	M	2	1	21.6	3.9	120	1.27	0.67	62.87	1.4	high	8.9	5.5	0.05	26	12.3
PS	47	F	19	1	21.7	4.9		2.45	0.88	108.3	1.6	high	9.2	5.2	0.13	24	11.8
PS	52	M	14	1	20.6	4.2		1.33	0.91	115.2	1.3	high	8.1	5.9	0.02	34	11.5
PS	63	M	11	1	20.8	2.9	154	1.74	0.71	121.7	1.5	high	9.8	4.7		24	9.5
PS	51	M	11	1	23.9	3.8	164	1.57	0.92	81.91	1.5	high	10.3	3.8	0.03	29	11.9
PS	57	F	10	1	23.3	4.3	145	1.79	1.23	82.5	1.3	high	8.5	1.7	0.38	30	11.2
PS	68	F	5	1	20	3.9		1.26	0.97	128	1.6	high	9.2	5.5	0.71	34	13.2
PS	72	M	5	1	19.8	3.7	109	1.39	0.72	117.7	1.8	high	8.9	5.2	2.23	33	10
PS	55	F	3	1	20.5	4.1		1.2	0.97	69.26	1.8	high	10.1	8.2	0.19	26	9.5
PS	80	F	2	1	22	3.3		1.59	0.77	81.52	1.6	high		4.9			10.3
PS	69	M	20	1	23.9	3.8	132	1.41	0.72	104.4	1.9	high	8.9	3.3	0.05	23	10.4

PS	61	M	6	1	19.8	3.9	126	1.5	1.22	119.7	1.3	high		4.3		17	9.4
PS	83	M	3	1	19.6	4	203	1.84	1.15	92.88	1.6	high	8.6	5.5	0.13		10.4
PS	56	M	27	1	23	3.8	107	1.47	1.03	101.8	2.1	high	8.9	6.4	0.1	30	11.6
PS	67	M	17	1	20	4.1	130	1.58	0.99	124	1.4	high	7.9	5.3	0.05	21	10.7
PS	64	M	12	1	27.9	3.8		0.95	0.96	103.5	1.5	high	8.1	7.8		29	9.8
PS	58	M	11	1	18.8	3.6	157	1.39	0.71	110	1.6	high	8.5	4.8	0.08	28	9.8
PS	63	F	11	1	17.5	4.1	94	1.3	0.87	152.3	1	high	8.6	3.6	1.93		8.5
PS	31	F	11	1	26.4	3.9	192	1.45	1.37	68.87	1.8	high	9.4	3.8	0.05	23	10.7
PS	61	M	8	1	23.8	3.5	105	1.39	0.81	99.64	1.5	high		5.5	0	18	9.3
PS	60	M	7	1	18.9	3.5	90	1.13	0.79	92.75	1.5	high	10.8	3.4	0.44	26	11.5
PS	70	M	6	1	23.9	3.6	122	1.05	0.69	78.35	1.8	high	8	6.7	0.34	25	10
PS	71	M	6	1	20.5	3.5	82	1.18	0.8	114.8	2.1	high	9.2	7.7	0.19	37	12.2
PS	61	M	5	1	22.6	4	165	1.37	0.79	77.16	1.8	high		6.6		22	9.9
PS	61	F	4	1	24.6	3.6	115	1.48	0.93	111.4	1.8	high	9.1	5.2	0.27	20	11.3
PS	69	M	4	1	21.2	4	113	2.2	0.88	85.44	1.3	high		3.5	0.5	33	8.3
PS	71	M	4	1	18.6	3.9	133	2.04	0.87	137.2	1.8	high	8	4.3	1.03	27	8.3
PS	61	M	3	1	20	3.9	198	2.42	0.87	112.5	2.1	high	8.1	5	0.08	27	11.8
PS	84	M	2	1	18.5	3.5	159	2.01	1.11	122.2	1.8	high	7.7	5.8			
PS	67	F	2	1	21.2	3.8	137	1.22	0.86	86.93	1.6	high	8.2	5.4	0	26	10.3
PS	71	F	3	1	20	3.6	201	1.77	1.1	95.44	1.8	high	9.3	5.3	0.01	25	9.4
PS	42	M	5	1	23.2	3.6	94	1.45	0.87	77.1	1.6	high	8.6	5	0.08	25	10.9
PS	56	M	2	1	34.4	3.1	165	1.49	0.97	66.51	1.5	high	8.9	5.9	2.89	30	10.6
PS	40	F	21	1	21.4	4.1		1.57	0.87	99.19	1.5	high	9.1	6.6	7	34	6.2
PS	53	M	18	1	21.6	3.8		1.46	0.94	112.9	1.8	high		4.3	0.25	29	11.1
PS	45	M	6	1	22.8	3	136	1.55	0.95	134.6	1.8	high	10	5.8	0.05	22	5.9
PS	69	F	5	1	29.9	3.6	139	1.26	1.06	93.11	1.8	high	8.6	4.3	0.05	47	9.1
PS	37	F	3	1	26.1	3.7	116	1.46	0.86	98.98	1.3	high	9.3	4.3	1.9	29	6.7
PS	63	F	13	1	21.2	4	160	1.52	0.94	119.7	1.5	high	9.5	5.3			11
PS	61	F	32	1	20.4	3.7	162	1.24	1.04	86.02	1.4	high	8.9	3.7	0.05	24	12
PS	38	M	8	1	23.8	4.8	118	1.61	0.92	83.03	1.5	high	8.3	3.4	0	28	9.4
PS	30	F	6	1	20.7		127	1.21	1.11	93.77	1.6	high		5	0.09	32	10
PS	78	F	4	1	19	4	125	1.41	1		2.1	high	9.2	6	0.5	21	10.2
PS	68	M	8	1	22.1	3.8	111	1.49	0.85	111.4	1.1	high	8	3.4	0.23	28	11.8
PS	74	M	3	1	21.3	3.7	99	1.45	1.05	126.4	1.8	high		5.4	0.25	21	12.4
PS	59	F	26	1	19.5	4.2	130	1.62	1.02	125.7	1.9	high	8.1	3.5	0.06	22	7.5
PS	74	F	21	1	19	3.9	131	1.15	1.02	112.9	1.3	high	8.2	3.1	0.1	22	9.4
PS	49	F	19	1	15.9	4	145	1.25	0.66	85.56	1.6	high	9.5	5	0.09	30	11
PS	58	M	12	1	20.5	4.6	152	2	0.72	108.1	1.6	high	10	5		25	11.7
PS	82	F	9	1	21.3	4	114	1.35	0.86	117.4	1.9	high	8.8	6	0.06	35	13.6
PS	68	M	8	1	19.1	4.3	179	1.12	0.93	91.37	1.8	high	9.4	5.2	0.35		10.3
PS	63	F	7	1	19.8	4.2	146	1.55	1.1	131	1.6	high	9.2	5.8		25	12.5
PS	61	F	5	1	20.3	4.3	120	1.69	0.77	112.6	1.6	high	9.4	8.1	0.31		11.5
PS	68	M	4	1	19	4.2	177	1.16	0.97	79.97	1.8	high	8.2	6.1			12.7
PS	65	M	4	1	21.1	4.6	200	1.83	0.85	108	1	high	8.7	4.9	0.12	34	10.1
PS	60	M	3	1	24.8	4	191	1.45	0.57	62.34	1	high	8.7	3.6	0.04	25	10.3
PS	73	F	2	1	29.6	3.9	118	1.02	0.53	18.18	1.5	high	10.6	7.5	0.8	28	10.6
PS	64	F	20	1	20.6	4.1	202	1.86	1.02	100.2	1.9	high	8.7	5.9	0.1	22	9.8
PS	56	M	19	1	16.7	4.5	102	1.46	0.88	78.72	1.6	high		5.2	0.77	29	8.1
PS	63	F	15	1	25.4	4.4	156	1.35	0.66	110.8	1.5	high	8.3	5.2	0.01	24	
PS	47	F	6	1	21	4.3	168	1.84	0.82	115.3	1.6	high	9.8	6.5	0.5	29	10.7
PS	82	M	5	1	19.7	4.2	175	1.8	0.79	124.7	1.3	high	8.6	7.9	0.1	26	10.2
PS	59	F	5	1	18.9	4.3	148	1.59	0.84	107	1.3	high	9.7	7.5	0.4	28	8.8
PS	59	F	4	1	20.4	4.4	133	1.15	0.97	91.8	1.5	high		3.9	0.31	28	
PS	60	M	4	1	22.1	3.8	199	1.66	0.84	93.55	1.1	high	9	2	1.1	27	9.7
PS	68	M	4	1	21.9	4.1		1.25	0.78	144.4	1.8	high	8.2	3.4	0.1	37	10.6
PS	61	M	2	1	21.6	4.2	141	1.65	0.74	115.3	1.6	high	9.7	5.5	0.1	22	10.8
PS	55	F	23	1	20.1	3.9	143	1.55	1.11	108.8	1.9	high	9.9	5.3	0.05	27	10.6
PS	65	M	19	1	23.3	4	129	1.56	0.95	106.1	1.9	high	9.2	5.8	0.1	34	11.1
PS	36	M	18	1	19.9	4.9	139	1.81	1.08	98.96	1.9	high	7.8	4.6	0.1	21	10.2

PS	43	F	14	1	19.5	4.6	167	1.42	0.86	91.66	1.9	high	7.9	6.6	1	22	13.5
PS	55	M	10	1	22.4	4.7	266	1.47	0.72	129.4	1.9	high	5.6	4.8	0.1	20	10.9
PS	61	F	10	1	17.9	4.1	149	1.44	0.99	115.1	1.6	high	8.4	5.9	0.1	22	9.8
PS	61	F	10	1	24.9	4.4	253	1.9	1.1	123.1	1.6	high	5.9	3.7	0	8	10.2
PS	66	M	5	1	19.8	4.1	176	1.23	0.75	118.5	1.6	high	9.4	5	0.5	21	11.2
PS	60	F	4	1	21.5	4.3	200	1.33	0.51	127.4	1.6	high	9	4	0	20	9.6
PS	48	F	4	1	26.2	3.9	187	1.47	0.93	80.81	1.8	high		6.6		24	10.3
PS	68	F	3	1	20.8	4.5	103	1.36	0.87	88.92	1.9	high	9.7	7	0	20	10
PS	61	M	2	1	27.9	4	159	1.09	0.55	61.05	1.5	high	8.2	5.3	0.33	27	12.6
PS	54	F	21	1	18.2	4.3	150	1.48	1.15	109.8	1.9	high	9.7	4.9	0.05	33	8.9
PS	64	F	18	1	17.7	4.1	174	1.59	0.8	103.5	1.3	high	8.3	4.5	0.3	23	10.7
PS	59	F	11	1	22.2	4.2	158	1.24	1.1	116.6	1.9	high	8.7	5.4	4.9	24	10
PS	46	M	9	1	26.5	4.7	110	1.09	0.83	141	1.8	high	9.2	6	0.16	23	8
PS	62	F	8	1	27	4.7	210	1.24	0.76	41.76	1.3	high	10.5	5.9	1.03	25	11.1
PS	47	F	6	1	24.4	4.3	176	1.7	0.87	105.9	1.3	high	8.5	6	0.1	24	10.9
PS	55	F	5	1	24.7	4.7	133	1.52	0.84	102.3	1.6	high	7.9	3.9	0.7	22	9.2
PS	50	F	5	1	22.2	4.5		1.28	0.76	131.7	1.5	high	8.2	5.6	5.15	40	9.8
PS	78	F	4	1	18.6	4.6	103	1.06	0.83	79.72	1.8	high		8.4	0.08	28	10.1
PS	54	F	2	1	29.2	4.1	194	1.14	1.03	129.6	2.1	high		6.5	0.25	24	11.2
PS	62	M	2	1	22.1	4.1	148	1.65	0.88	109	1.3	high	9.1	6.2	0.35	26	10.8
PS	67	F	9	1	16.5	4	127	0.99	0.99	86.86	1.6	high	7.9	2.6		29	10.5
PS	58	M	17	1	19.4	3.9	214	1.47	1.2	155.4	1.6	high	8.3	4.6	2.7	20	10.5
PS	50	M	13	1	23.6	3.6	125	1.44	0.92	102	1.8	high	9	4.9	0.05	30	10.8
PS	57	F	10	1	24.7	3.7	104	1.46	0.96	129.5	1.8	high	9	4.9	0.05	25	11.6
PS	41	M	8	1	24.9	4	94	1.36	0.9	101.6	1.8	high	8.7	3.2			9.5
PS	63	F	2	1	18.8	3.8	201	1.17	0.77		1.8	high		7.4			10.6
PS	51	F	2	1	24.3	3.9	165	1.34	0.97	113	1.1	high	9.2	6.4		21	10
PS	76	F	2	1	26.4	3.8	148	1.4	0.64	104.2	1.5	high	7.6	3.7	0.15	27	10.8
PS	71	M	7	1	24.5	3.9	184	1.44	0.86	101.9	1.1	high	9.7	6.3	0	24	9.1
PS	47	F	17	1	19.3	3.8	153	1.32	0.98	118.6	1	high	9.4	7	0.03	22	11.9
PS	72	F	3	1	21	3.7	142	1.6	0.95	118.6	1.6	high	9	4.4	0.2	28	10.2
PS	61	F	3	1	19.6	4	102	1.4	1.31	121.8	2.1	high	6.5	5	0.1	31	9.7
PS	49	F	29	1	21	3.6	136	1.32	0.8	104.9	1.5	high	8.8	4.4	0.02	37	9.4
PS	54	F	15	1	18	4	166	1.39	1.17	102.9	1.5	high	9.6	6	0.04	25	9.2
PS	64	F	15	1	25.5	3.7	142	1.29	1.04	110.1	1.3	high	10.6	4.6	0.04	31	11.4
PS	64	M	13	1	24.3	3.7	94	1.25	0.91	118.9	1.8	high	8.1	4.6	0.05	36	11.3
PS	63	F	12	1	15	3.6	148	1.32	0.69	112.4	1.8	high	8.8	5.6	0.02	25	10.3
PS	59	M	10	1	19.6	3.9	158	1.34	0.9	114.4	1.8	high	8.9	2.6	0.7	29	11
PS	28	F	9	1	27	3.9	174	1.85	0.93	116.8	1.8	high	8.8	5.2	0.3	32	11.9
PS	59	M	8	1	18.5	3.8	149	1.69	1.1	118.5	1.5	high	8.7	6.9	0.03	27	8.6
PS	63	F	7	1	27	3.8	113	1.18	0.97	106.9	1.5	high	8.8	6.2		25	9.3
PS	68	M	3	1	17.5	4	179	1.85	0.91	88.75	1.3	high	9.1	6.1	0.01	20	10
PS	56	M	2	1	22	4	129	1.85	0.63	93.87	1.6	high	9.2	4.3		28	12.2
PS	70	M	9	1	21.9	4	107	1.58	0.59	111.3	1.5	high	8.9	7.3	0	42	10.5
PS	54	M	8	1	18.3	3.9		1.38	0.93	122.9	1.8	high	8.8	5.2			8.7
PS	61	F	4	1	14.2	3.9	143	1.48	1.22	84.08	1.6	high	8.5	4.8	0.1	14	10.7
PS	41	F	2	1	21.5	3.9	143	1.34	0.94	80.74	1.3	high		6.1	0.31	25	10.5
PS	49	M	26	1	27.9	4.2		1.17	0.76	93.06	2.1	high	9.8	5	0.82		13
PS	54	F	2	1	22.4	4.1	124	1.51	0.81	69.76	1.3	high	8.8	4.9	0.05	20	10.2
PS	58	F	35	1	15.7	4	135	1.29	1.01	118	1.5	high	9.5	6	0.98	31	10.1
PS	63	F	31	1	22.4	3.2	116	1.32	0.96	124	1.8	high	9.4	7.2	0.7	39	10.9
PS	58	M	28	1	18.9	3.8	227	1.63	0.75	125.8	1.3	high		4.3	0.38	32	10.4
PS	68	F	23	1	22.2	3.8	141	1.79	0.85	112	1.4	high	8.4	4.7	0.05	42	9.3
PS	64	M	17	1	19.6	3.6		1.52	1.01	78.72	1.8	high	8.8	3.7		16	10.3
PS	65	F	17	1	16.2	4	236	1.2	1.01	127.4	2.1	high	7.8	7.1	0	28	11.3
PS	48	F	14	1	22.2	3.3	118	1.33	1.16	137.7	2	high	9.6	2.8	2.5	41	10.4
PS	77	F	6	1	19.1	3.6	170	1.09	1.28	102.4	1.8	high	8.7	5.4		18	10.4
PS	73	M	6	1	19.2	3.9	132	1.12	0.85	124.6	1.8	high	8.5	1.2	0.91	33	10
PS	57	F	6	1	21.6	4.2	113	1.39	0.92	109.2	1.4	high	8.6	5.7	0.53	30	10.1

PS	51	M	4	1	19.7	3.9	209	1.53	0.72	102.8	1.3	high		5.2	0.02	30	11.2
PS	59	M	4	1	26.3	3.6		1.52	1.01	100.4	2.1	high		3.8	0.06	30	10
PS	56	M	3	1	30.5	4.3	167	1.36	0.87	71.05	2.1	high	8.5	3.8	0.01	23	10.3
PS	54	M	6	1	24.1	3.9	140	1.65	1.14	134.5	1.3	high	8.7	5.1	0.03	27	10
PS	47	M	4	1	24.5	4.2	137	1.29	0.88	119.9	2.1	high		5.8	0.75		8.9
PS	65	F	3	1	21.7	4		1.31	0.78	107.9	1.6	high	9.1	3.9	0.04	15	10.2
PS	62	M	10	1	26.9	4	143	1.46	0.89	132.5	2.1	high	9.1	6.7	0.05		10.2
PS	45	M	4	1	21.4	4.2	132	1.45	0.89	90.63	1.4	high	9.2	3.9	0.05	24	11.1
PS	62	F	31	1	23.4	3.4	132	1.27	0.92	118.4	1.8	high	8.4	4	0.03		10.3
PS	50	M	6	1	19.7	4.3	134	1.83	0.88	150.2	1.4	high	8	7.8	0.06	31	11.9
PS	60	F	12	1	15.1	3.7	165	1.68	1.14	81.54	1.6	high	10	7.7	0.04	33	10.3
PS	63	F	10	1	19	3.8		1.75	0.89	107.4	1.6	high		5	0.02	24	11.6
PS	53	M	13	1	17.2	3.8	153	1.39	1.24	79.31	2.1	high	10.3	5.2	0.08	15	10.1
PS	53	M	11	1	21.6	3.9	144	1.13	0.99	125.5	1.8	high	9.8	8.4	0.2		10.9
PS	46	M	6	1	23.1	4.1	141	1.85	0.81	117.6	1.6	high	8.1	5.2	0		10.8
PS	27	M	4	1	21.9	4.5	157	1.6	0.76	67.26	1.1	high	9.4	4.4	1.02	29	11.8
PS	65	F	3	1	19.7	4.1	107	1.44	0.99	100.5	2.1	high	8.4	4.3	0.9		10.5
PS	44	F	16	1	16.7	4.4		1.79	0.57	120.8	1.5	high					
PS	60	F	14	1	20.5	3.9	181	1.36	0.99	111.8	1.5	high	9.8	3.8	0.08		10.1
PS	75	M	2	1	19.5	3.2	139	1.17	0.81	103.5	2.1	high		2.3		47	10.1
PS	69	M	17	1	18.2	3.6	164	1.17	0.75	129.8	1.8	high	8.3	6.9	0.05	30	11.5
PS	74	F	9	1	18.8	3.3	209	1.55	0.86	153.2	1.5	high	8.7	4.6	0.04		10.4
PS	62	M	12	1	20	4.1	189	1.31	0.76	124.3	1.6	high	9.5	5.9	0.01	27	11.1
PS	75	F	10	1	20.1	3.9	207	1.76	0.98	107.7	2.5	high	9.2	6.1	0.04	27	8.4
PS	54	M	5	1	19.9	3.8	144	1.52	0.87	108.2	1.4	high	8.7	4.2	0.2	28	10.8
PS	44	M	3	1	17.5	4.2	79	1.82	0.87	64.46	1.5	high	9.7	1.6	0.02		8.9
PS	51	M	28	1	17.8	3.8	106	1.37	1.1	108.3	2.1	high	9.5	6.6	0.08		9.5
PS	57	M	3	1	19.7	4.1	120	1.4	0.9	122.2	1.8	high	8.9	6.7		22	11.8
PS	62	F	20	1	18.3	3.9	177	1.59	1.06	104.5	1.3	high	9.9	5.4	1.5		10.6
PS	35	F	14	1	22.5	4	140	1.21	1.02	108.5	2.1	high		5.8			8.4
PS	65	F	10	1	21	4.3	109	1.32	1.24	124.6	1.4	high	8.5	3.9	0.09	26	10.3
PS	56	F	8	1	19.6	4	177	0.94	0.8	130.4	1.6	high		2	1.07		9.2
PS	57	M	6	1	20.6	4.1	159	1.66	0.78	85.6	2.1	high		4.9	0.3	31	10.8
PS	53	M	5	1	24.1	4.2	148	1.55	0.87	119.2	1.3	high		4.9	0.01	48	10.4
PS	74	M	2	1	20.7	4	141	1.56	0.89	75.05	1.6	high		3.6	0.62	25	10.2
PS	80	M	2	1	23.8	3.7	173	1.44	0.92	133.1	1.4	high	9.6	4.9	3.06	26	10.9
PS	51	M	6	1	21	4.7	160	1.46	1.08	100.1	1.3	high	7.5	7.7		39	9.9
PS	55	M	6	1	26.8	4.2		1.51	0.97	144.7	2.1	high	8.7	3.9			11.6
PS	57	F	6	1	24.5	4.3	218	1.52	0.97	129.7	1.6	high		5.2	0.3	17	8.8
PS	40	F	22	1	20.6	4.1	160	1.12	0.95	133.6	1.8	high	8.7	4.9	0.24		11.9
PS	71	M	3	1	18.9	3.4	177	1.34	1.11	107.8	1.8	high	10.2	6.7	0.52	20	10.8
PS	56	F	2	1	21.8	4	146	1.03	0.8	87.29	1.6	high	7.5	5	0.2		10.3
PS	40	F	17	1	17.9	3.8	201	1.58	1.26	98.72	2	high	8.6	4.6	0.1	33	10.3
PS	28	F	4	1	22.7	4.1	137	2.06	1.23	115.4	1.5	high	9.2	4.5		28	11.4
PS	54	M	4	1	23.1	4	101	1.38	1.03	135.4	1.6	high	8.8	5.5	0.19	23	10.7
PS	54	M	26	1	18.6	3.7	124	1.45	1.17	119.5	1.5	high	9.3	4.1	2.88	38	11.3
PS	52	M	14	1	21.3	3.6	142	1.18	0.99	108.1	1.5	high		4.9	0.3	24	10.7
PS	65	F	11	1	22.2	3.7	134	1.25	0.98	143.7	1.5	high	8.9	6.1	0.3	27	11.4
PS	67	M	5	1	21.4	3.7	176	1.43	0.96	135.9	1.6	high	8.8	8.5	0.29	32	11.2
PS	59	M	3	1	19.9	4.3	126	1.06	1.03	105.3	1.8	high	10.5	3.5	0		11.3
PS	28	F	5	1	20.4	4.6		1.63	0.87	104.3	2.1	high	8.8	3.2	0.86		11
PS	60	M	15	1	21.2	3.9	151	1.65	0.9	110.6	1.6	high	9.7	6.9	0.12	27	13.5
PS	69	M	12	1	20.2	4.2	99	0.94	0.86	116.1	1.6	high	8.8	5.8	0.11	32	9.7
PS	65	M	8	1	22.7	3.9	129	1.63	0.86	130.2	1.8	high	8.5	4	0.05	24	11.8
PS	38	F	17	1	17.6	4.2	177	1.56	1.24	116.6	1.4	high	7.9	5	0.17	21	10.6
PS	36	M	9	1	23.5	4	157	1.26	0.89	115.2	1.8	high	10.1	4.3	0.4	23	11.2
PS	54	F	19	1	23.1	4.2	182	1.48	1.11	128.8	1.3	high	9	6.9	0.05	31	9
PS	57	F	18	1	17.4	4.3	102	1.04	1.09	134.7	1.5	high	8.2	3.7	0.15	18	10.8
PS	59	M	7	1	21.3	4.3	116	1.18	1.2	132.8	1.8	high	8.9	7.3	0.03		11.4

PS	33	F	21	1	19.2	3.6	221	1.1	0.94	105.1	1.8	high	9.8	4.9	0.1	26	12
PS	56	F	33	1	19.2	3.9	153	1.48	1.05	125.9	1.1	high	9.5	5.5	0.1		12.4
PS	44	M	18	1	16.7	4.1		1.39	0.9	103.3	2.1	high	9.8	4.6		31	9
PS	31	F	13	1	20.9	3.7		1.42	1.14	112.9	2.1	high	8.8	5	7.47		11.5
PS	61	M	9	1	19.9	3	171	1.44	0.66	76.42	1.6	high	8.2	5.8	0.05	21	10.1
PS	66	F	2	1	17.9	3.6	150	1.17	0.77	87.8	1.8	high		4.1	0.95		12.5
PS	58	F	28	1	15.8	3.4	142	1.39	1.04	105.8	1.5	high	8.9	2.9			11.6
PS	69	M	26	1	18	3.9	176	1.11	0.91	90.34	1	high		6.3			10.3
PS	70	F	20	1	14.5	3.2	157	1.88	0.85	79.37	0.8	high	7.8	3.1			8.4
PS	64	F	11	1	21.5	3.9	154	1.5	1.12	135.5	2.1	high	9	4.8		25	10.5
PS	46	F	8	1	22.2	3.7	146	1.82	0.7	125	2.1	high	9	5.8		28	11
PS	45	M	8	1	22.2	4.2	152	1.29	0.85	95.14	1.5	high	10.6	4.3	0.02	29	11.9
PS	68	F	7	1	18	4.2	112	1.44	0.94	104.7	1.3	high	8.1	4.3	0.49	33	10.7
PS	60	F	7	1	18	3.8	133	1.42	1.17	134.8	1.5	high	8.5	3.9			9.7
PS	54	F	7	1	17.3	3.8	155	1.45	0.93	96.96	1.6	high	9.2	4.3		27	10.2
PS	60	F	7	1	23.9	3.8	124	1.16	0.7	120.9	2.1	high	8.5	5.3		22	9.1
PS	61	F	6	1	17.9	3.6	149	1.55	0.78	105.7	1.8	high	8.3	3.2			9.5
PS	73	M	6	1	18.4	3.7	102	0.82	1	77.46	1.6	high	9.3	5	0.25	27	8.9
PS	73	F	6	1	16.3	3.8	129	2.09	0.94	128.7	2	high	9.9	4.6		25	12.2
PS	60	F	5	1	18.8	4.3	180	1.5	0.89	100.9	1.3	high	9.1	5.3	0.09	31	9.8
PS	58	M	5	1	23.1	4	147	1.34	0.8	104.4	2.1	high	8.6	4.7	0.17	31	10
PS	65	F	4	1	17.9	3.6		1.48	1.05	103.1	1.5	high	8.5	4.5		33	9.9
PS	66	M	4	1	20.9	4.2	134	0.96	0.88	74.04	2.1	high	7.9	3.3	0.35	32	9.4
PS	67	M	4	1	18.3	3.7	155	1.18	0.74	88.65	0.8	high		4.7	4.51	25	9.2
PS	67	F	4	1	19.9	3.9	250	1.49	1.24	129	1.3	high		3.1	0.32	14	10.5
PS	67	F	4	1	22.2	3.6	170	1.39	0.96	87.38	2.5	high		4.2		28	9.2
PS	67	M	3	1	24.2	3.9		1.38	0.74	80.73	1.3	high	8.8	4.5		14	10.8
PS	51	F	2	1	23	3.6	112	0.98	0.74	118.7	2.1	high		6		28	9.7
PS	42	M	2	1	24.5	4.2	122	1.44	0.62	100.9	1.8	high	8.3	3.4			9.9
PS	34	M	2	1	27.1	4	188	1.34	1	101.5	1.5	high	9.3	5.1		25	10
PS	60	M	2	1	31.7	4	211	1.66	1.09	107.5	1.3	high	8.8	4.6		31	11.4
PS	51	M	2	1	28.4	3.7	158	1.16	0.9	77.66	1.5	high		3.9	0.57	24	11.2
PS	46	F	2	1	19.2	3.8	168	1.7	0.92	89.87	1.5	high	9	4.5		26	10.5
PS	67	M	2	1	20.6	4	204	1.67	0.76	99.07	1.5	high	10.7	4.4		22	9.4
PS	58	M	4	1	26.1	4.1		1.69	0.9	144.4	2.1	high	9.4	6.3			11.7
PS	73	F	2	1	21.5	3.8	156	1.43	1.02	94.57	2.1	high	9.5	4.4		28	8.8
PS	67	F	3	1	21.8	3.4	167	1.55	0.93	153.5	1.5	high	8.9	3.8		28	8.6
PS	46	F	5	1	22.6	3.5	221	1.43	0.89	111.8	1.3	high		4.9		30	10.7
PS	43	M	7	1	19.6	3.9	114	1.38	1.72	138	1.9	high	9.1	6.7	0.06	26	10.2
PS	57	M	5	1	20.9	3.9	138	1.37	0.61	72.93	1.5	high	9.3	3.6	0.27	20	11.1
PS	61	M	2	1	26.6	4.2	123	1.57	0.87	123.5	1.5	high	11	4.8	0.39		10.8
PS	74	M	10	1	22	3.4	144	1.14	0.6	116.3	1.8	high	10.3	6.2	0.05	26	10.9
PS	59	M	3	1	26.4	4.2		0.8	0.78	77.41	1.5	high	9.5	8.7	17.13		9.2
PS	55	M	10	1	24.7	4.1	197	1.15	1.08	99.44	1.6	high		4.4	0	14	10.8
PS	71	M	4	1	16.6		159	1.04	0.89	115.9	2.1	high	8.8	6.5		31	10.5
PS	76	M	2	1	23.4	3.6	175	1.53	0.96	129.7	2.2	high	8.9	2.3	0.13	31	10.9
PS	67	M	4	1	23.6	3.8	120	1.53	0.79	105.8	1.9	high	9.1	4.1	0.05	26	11.5
PS	63	M	2	1	20.2	3.5	209	1.46	0.67	59.09	1.6	high		5.9	0.06		9
PS	76	M	3	1	26.3	3.9	133	1.71	0.92	80.89	2.1	high	9.5	4.4	0.04	31	12.1
PS	45	M	23	1	20	2.8	139	1.36	0.85	121.8	2.1	high		5.4	0.1	22	12.3
PS	83	F	2	1	15.9	3.7	142	1.51	0.77	63.6	1.9	high	9.3	2.7	0.05	21	10.3
PS	58	M	3	1	28.4	4.3	87	1	0.93	113	1.8	high	9.4	5			8.8
PS	50	F	36	1	20.1	3.9	135	1.42	0.73	84	1.8	high	8.5	6.2	0.1	41	10.6
PS	73	F	13	1	18.1		97	1	1.2	126.1	1.8	high	8.5	3.3	0.34	27	9
PS	74	M	7	1	17.6	3.7	160	1.44	0.93	109.1	1.5	high	9	3	0.1	28	9.4
PS	52	M	4	1	25	3.9	125	1.44	0.85	121.3	1.5	high	8.5	3.5	0.87	18	9.4
PS	67	M	29	1	20.7	3		1.05	0.8	92.63	1	high	10.3	2.3	1.6		9.6
PS	80	F	14	1	16.8	3.3	150	0.81	0.84	116.9	1.8	high		5	0.27	30	6.9
PS	60	M	3	1	22.3	3.5	167	1.36	1.05	111.3	1.8	high	9.2	6.1	0.05	29	11.1

PS	56	M	11	1	24.5	3.7	103	1.44	0.81	94.27	1.3	high	9.7	5.1	0.05	17	9.9
PS	66	M	11	1	20.4	3.4	182	1.19	0.82	91.01	1.3	high	8.5	4.8	0.11	34	12.2
PS	61	M	12	1	21.6	3.6	108	1.22	0.85	109.7	2.1	high	10	4.7	0.1	24	9.2
PS	57	M	12	1	22.8	3.9	180	1.43	1.28	120	1.5	high	9.1	5.3	0.4		10
PS	75	M	9	1	17.5	3.3	108	0.89	1.16	69.07	1.1	high	8.1	5	0.1		10.7
PS	56	M	9	1	20.6	3.8	171	2.01	1.07	124.3	1.5	high	8.7	5.1	1.65	30	6.1
PS	76	F	9	1	18	3.5	160	1.39	1.02	100.2	1.1	high	9.1	4.6		23	10
PS	65	M	8	1	20.9	3.5	186	1.61	1.27	103.8	1.6	high	9.3	5.3	0.1	27	10.1
PS	63	M	4	1	21.4	3.6	158	1.23	0.9	132.2	1.3	high	9.5	4.6	2.1	24	10
PS	55	M	3	1	25.7	3.9	138	1.29	1.07	125	1.5	high	10.8	7.1	0.03	27	10.5
PS	59	M	2	1	23.7	3.2	151	2.33	0.45	19.14	2.1	high	8.4	7.6	0.3	35	9.6
PS	73	F	29	1	20.9	3.4	121	1.36	0.9	81.37	1.9	high	9.6	5.5	0.08	26	10.9
PS	64	M	5	1	21.8	4	208	1.29	1.28	96.73	1.8	high	9.2	4.9	1.04	23	10.2
PS	26	M	2	1	18.2	3.9	143	1.37	0.97	79.12	1.3	high	9.3	4.2	0.69	19	10.3
PS	62	M	2	1	19.7	4	151	1.45	0.86	120.3	1.3	high	10.3	6.6	0.08	23	10
PS	71	M	2	1	20.1	4	163	1.99	0.93	122.6	1.5	high	9.7	8.3	11.1	24	10.6
PS	52	M	2	1	23.5	4.1	145	1.29	0.89	71.52	1.3	high	9.4	4.6		24	9.4
PS	66	M	24	1	26	3.9	123	1.3	1.1	129.1	2.5	high	10.4	6.6	0.06		13.1
PS	35	M	14	1	21	4.8		1.6	0.92	110	1.6	high	9.2	4.9		20	
PS	72	M	13	1	19.5	3.7	171	1.93	0.99	131.4	1.6	high	9.7	5.2			9.9
PS	62	F	13	1	16.4	4	135	1.82	1.12	131.2	1.5	high	8.9	4.2			11.3
PS	70	F	12	1	19.3	3.7	151	1.32	0.78	123.9	1.4	high	9.5	4.8	0.27	35	10
PS	56	F	11	1	32	3.9	146	1.17	0.62	132.7	2.1	high	9.1	4.3	0.38	28	10
PS	34	M	11	1	20.4	4.1	169	1.78	0.92	120.1	1.6	high	9.1	4			9.6
PS	66	F	10	1	20.8	3.8		1.61	0.9	112.6	1.8	high	10	5.1	0.09	30	10.8
PS	41	F	10	1	20.4	4	125	1.87	0.87	121.5	1.3	high	9.5	4			11.6
PS	62	M	10	1	18.3	3.4	138	1.53	0.98	103.6	2.1	high	8.9	3.4	0.8	23	
PS	57	M	10	1	22.2	4.1	142	1.06	1.2	129.2	2.5	high	9.7	7	0.15		11
PS	61	M	9	1	25.4	3.7	86	1.09	0.89	110.8	1.1	high	8.3	2.4		34	11.3
PS	73	M	9	1	20	4.2	124	0.76	1.21	97.47	1.8	high	8.2	4.6	0.2	34	8.4
PS	67	F	9	1	20.6	3.7	129	1.59	1.28	105.5	1.8	high	8.4	2.1	3.11	29	10.1
PS	46	M	8	1	28.3	4.3	195	1.23	0.96	117.5	1.3	high		6.4	0.04	29	11.3
PS	38	M	8	1	20.3	4.2	99	1.45	1.38	110.3	2.1	high	8.4	3.1	0.28	30	9.1
PS	52	F	8	1	20.2	3.9	160	1.23	1.36	89.17	1.8	high	8.1	5.4	0.06	31	11.9
PS	76	F	8	1	16.3	3.7	146	1.22	0.82	101.6	1.6	high	9.9	4.7			10.6
PS	31	M	7	1	23.6	4.1	167	1.39	1	119.3	1.9	high	8.6	5.1	0.07	23	11.5
PS	59	M	7	1	20.6	4.4		1.01	0.98	81.66	1.5	high	8.2	4.2	0.63		11.7
PS	65	M	7	1	17.4	3.7	123	1.96	1.05	109	1.5	high		4.3	0.15		12.6
PS	54	M	7	1	20.1	4.2	176	1.24	1.08	129	1.8	high	8.2	5.1	0.66	26	8.5
PS	60	M	6	1	22	4.3	178	1.48	0.82	116.7	1.5	high	7.9	5.3	0.09	27	10
PS	76	F	6	1	19	3.8	133	1.09	0.72	85.26	1.6	high	7.9	4.4	0.07	24	10
PS	54	M	6	1	19.2	4	164	1.53	1.12	128	1.3	high	8.9	4.5	0.2	29	10.4
PS	41	M	6	1	21.9	4.2	165	1.9	1.17	142.4	1.3	high		7.1	0.05	30	10.9
PS	58	M	5	1	17.4	4.3	160	1.6	1.13	97.45	1.3	high		2.8	0.01	42	9.9
PS	72	M	5	1	24	4	202	1.79	0.79	100.6	1.5	high	9.5	4.8	0.05		10.6
PS	57	F	5	1	20.9	3.7	145	1.33	1.27	127.6	1.9	high	10.8	4.5	0.47	24	9.4
PS	64	M	5	1	23.2	3.8	163	1.48	0.98	98.31	2.1	high	10.2	4	0.08	26	10.7
PS	78	F	5	1	20.1	3.7	195	1.72	0.7	91.14	2	high	10.5	6.6		24	9.6
PS	55	F	5	1	22.3	3.7	147	1.57	1.11	130.4	1.5	high	8.8	3.6	0.12	25	10
PS	61	M	4	1	24.4	4.2	146	1.29	1.26	103.9	1.3	high	8.5	7.6	0.2	25	11.7
PS	46	F	4	1	29.8	4.1	198	1.44	0.85	105.2	1.8	high	8.9	3.5	0.4	23	10
PS	78	M	4	1	19.3	4	129	1.45	1.09	102.2	2.1	high	10.2	3.3	0.19		10
PS	64	M	4	1	25.2	4		1.76	0.74	111.5	1.5	high	9.2	4.5	0.01	2	10.5
PS	59	M	4	1	18.5	3.9	84	1.73	0.9	123.9	1.9	high	7.7	3.5	0.45	25	10.8
PS	55	M	4	1	21	4.2	151	1.07	0.85	103.9	1.5	high	9.1	6.7	5.5	35	10.2
PS	71	M	4	1	19.5	3.9	85	1.89	0.7	121.2	2.1	high	7.4	5.3	7.2	25	12
PS	63	M	3	1	19.7	4.2	99	1.14	1.07	98.07	2.5	high	9.1	4.8	0.29	24	10.5
PS	31	M	3	1	20.1	4	115	1.34	1.09	120.4	2.5	high		5.1	0.1	20	10.8
PS	43	M	3	1	26.4	4.2	129	1.1	0.9	124	2.1	high		8			10.3

PS	61	M	3	1	24.4	4.1	151	1.72	0.84	94.66	1.3	high	8.1	3.7		30	12.6
PS	67	M	2	1	24	3.8	107	1.37	0.99	72.01	1.8	high	8.5	2.4	0.03	31	10.2
PS	78	M	7	1	18.5	3.5	234	1.37	0.81	114.4	1.3	high	8.7	3.2	0.3	30	10.2
PS	50	M	5	1	22.3	3.9	132	1.18	0.98	128.8	2.5	high		7			8.7
PS	84	F	3	1	19.9	3.7	124	1.59	0.83	45.39	1.6	high	9.9	6.4	0.1	20	9.3
PS	40	F	3	1	18.6	3.4	153	1.62	0.72	99.82	1.3	high	9.6	4.5	0.1	30	12.5
PS	82	F	5	1	22.1	3.8	82	1.18	0.54	86.79	1.8	high	8.8	3.6	0.09	32	9.8
PS	52	M	4	1	26.4	3.9	153	2.13	0.86	97.76	1.5	high	9.1	5.6	0.67	31	10.2
PS	48	M	4	1	20.2	3.9	124	1.08	1.03	87.04	1.5	high	9.6	2.8	0.77	26	8.4
PS	56	F	2	1	16.8	3.9	149	1.6	0.9	95.42	1.4	high	8	5.2	0.05	28	10.3
PS	59	M	15	1	32.7	4.1	136	1.23	0.83	147.5	2.5	high	8.9	6.1	0	19	10.3
PS	77	F	2	1	18.7	3.4	137	1.52	0.81	116.2	2.1	high	9.3	3.2	0.06		10.2
PS	79	F	3	1	20.3	4	195	2.21	0.75	147.1	1.5	high	9.5	6.3	0.07		10.8
PS	58	M	11	1	21.3	4	146	1.79	0.7	99.83	1.6	high	9.3	5.1			10.3
PS	61	F	2	1	26.9	4.1	101	1.41	0.69	102	1.3	high	8.5	5.2	0.46	28	8.8
PS	72	F	4	1	21.9	3.7	196	1.23	0.75	102	1.5	high	10.2	4.7	0.14	18	12.4
PS	49	F	11	1	17.9	3.6	98	1.72	0.81	110	2.1	high	7.3	8.1		21	8.9
PS	80	M	3	1	19.2	3.9	126	1.21	0.63	81.32	1.5	high		4.4	1.12		10.5
PS	67	F	6	1	25.8	4.2	183	1.96	0.9	107.9	1.8	high	8.6	1.3	0.05	21	11.2
PS	82	F	4	1	22.9		271	1.23	0.73	102.3	1.8	high	11	5.8		27	10.9
PS	61	M	7	1	19.2	3.8	156	0.81	1.1	122.1	1.5	high	8.6	4.5	0.28	36	11.1
PS	58	M	3	1	22.8	4.2	185	1.17	0.66	85.17	2.1	high	8.9	5.5	0.06	34	10.5
PS	71	F	10	1	25.3	3.3		1.63	0.96	116.6	1	high	9.8	5.7		26	11
PS	79	F	17	1	17.5	3.3	143	1.51	0.77	124.5	1.4	high	9.1	5.3	0.33	29	9.7
PS	70	M	9	1	19.2	3.7	149	1.33	0.76	94.21	1.5	high	8.7	6.8	0.2	23	9.8
PS	69	F	2	1	25.2	3.5	160	1.3	0.55	41.76	1.6	high	9	3.8	3	32	8.1
PS	73	M	4	1	17.9	3.8	180	1.21	1.07	84.12	2.1	high	8.6	4	0.5	30	13.4
PS	73	F	8	1	23.8	3.5	127	1	0.96	115.7	1.3	high		4.9	0.83	34	7.8
PS	74	M	9	1	22.9	3.5	100	1.37	1	115.3	1.9	high	7.9	6.4	0.05	35	10.8
PS	63	M	18	1	17.2	4.1	150	1.52	0.91	109.1	1.6	high	9.1	4.8	0.05	26	10.9
PS	58	F	13	1	21.6	3.5	182	1.91	0.88	109.3	2.1	high	10.6	6.8	0.05		9.7
PS	55	F	8	1	27.6	4	98	1.06	0.83	84.24	1.8	high	8.6	4.4	0.3	43	12.4
PS	58	F	2	1	16.6	4.2	153	0.97	0.75	70.53	1.3	high	8.8	6.8	0.03	19	10.4
PS	72	M	16	1	21	4		1.65	0.78	124.5	1.3	high		4.8	0.17	34	11
PS	61	M	28	1	25.8	3.3	95	1.4	1.06	87.18	1.8	high	8.6	7.4			10.7
PS	60	M	13	1	24.5	4.4	135	1.45	0.92	132.4	1.5	high	8.1	4.9			12
PS	77	F	9	1	16.6	4.1		1.52	0.89	110.4	1.6	high		6	0.16	25	11.1
PS	71	F	8	1	19	3.5	148	1.69	1.17	78.8	1.5	high	9.8	5.1	1.29	24	10.8
PS	52	F	7	1	20.6	4	173	1.33	1.1	98.99	0.8	high	7.9	6.5	0.1	43	10.4
PS	79	F	7	1	26.4	4.1	152	1.28	0.88	109.5	2.1	high	10.3	5.9	0.12		11.4
PS	70	M	6	1	24.4	3.5	111	2.16	0.81	124.5	1.8	high	8.7	4.8			11.6
PS	71	F	6	1	19	3.4	95	1.36	0.68	121.3	2.1	high	9.1	4.3	0.05		10.7
PS	52	M	6	1	21.9	4.6	129	1.3	0.75	87.49	1.5	high	9.9	5.3	0.1	22	9.4
PS	55	F	5	1	26.2	3.7	142	1.75	0.59	87.51	2.1	high	9.6	5.1	0.06		12.5
PS	89	F	4	1	20.9	3.4	194	1.26	0.97	122.7	2.2	high	8.9	4	0	26	10.1
PS	77	M	4	1	20.6	3.7	139	1.36	0.87	122.2	1.7	high	10.7	5.4	0		10.1
PS	44	F	3	1	22.1	4.1	227	1.34	1.02	107.7	2.1	high	9.3	3.9	0		11
PS	58	F	3	1	19.2	3.7	185	1.78	1.01	123.3	2.5	high	10.3	5	0.05		11.3
PS	78	F	3	1	27.4	3.4	148	1.56	0.65	68.2	2.1	high		6.9	0.05	24	10.8
PS	74	M	3	1	22.2	3.8	145	1.5	0.9	127.5	2.1	high	10.1	5	0.1		11.6
PS	62	F	2	1	30.7	4		1.64	0.78	85.27	1.5	high	8.7	3.8	0.06	25	12.2
PS	63	M	2	1	24	4.2	154	1.65	0.93	53.38	1.5	high	10.4	3.8	0		11
PS	52	F	2	1	28	3.8	98	1.06	0.71	71.28	2.1	high		3.8	0.22	27	12.8
PS	59	M	30	1	16.5	3	141	1.66	0.92	46.26	1.6	high	10.5	3.8	0.06	27	10.2
PS	53	F	25	1	20.5	3.9	311	1.17	0.92	104.4	1.3	high	8	5.9	0.67	12	10.3
PS	39	M	21	1	18.8	4.1	157	1.15	1.01	92.82	2.1	high		9.8	0.03	25	10.7
PS	59	M	18	1	17.5	3.7	152	1.22	0.95	99.11	1.6	high	8.9	5.1	0.05	28	10.2
PS	59	M	11	1	19.5	4.1	144	2.11	1.03	116.2	1.9	high	9.4	4.9	0.05	20	11.1
PS	55	F	10	1	36.1	3.9	151	1.44	0.85	91.44	1.9	high	8.1	6.7	0.27	26	11.2

PS	70	M	9	1	20.2	3.7	134	1.25	0.91	116.7	1.5	high		5.2	0.1		10.8
PS	63	M	9	1	23.8	4.5	91	1.67	0.76	111	1.6	high	8.5	2	0.1	23	9.8
PS	43	F	8	1	26	3.9	206	1.94	1	100.7	1.3	high	9.1	3.9	0.9	47	10.8
PS	40	M	8	1	18	3.7	126	2.06	0.68	81.84	1.3	high	9.3	3.5	0.05	31	9.8
PS	53	M	6	1	24.2	6.8	111	1.78	0.95	90.56	1.3	high	10	5.9	0.02	32	9.7
PS	78	F	6	1	19.1	3.6	197	1.17	0.84	41.36	1.9	high	9	4.9	0.3	14	11.6
PS	52	M	4	1	24.9	4.2	112	1.32	0.67	93.56	1.5	high	8.8	6.5	0.3	24	9.3
PS	51	M	4	1	18	3.8	126	1.13	0.93	90.88	2.1	high		4.5			8.9
PS	49	M	3	1	24.2	3.9	133	1.44	0.96	95.52	1.8	high	9.6	5.4	0.33	25	10.7
PS	43	M	2	1	21	3.8	132	1.27	0.72	65.31	2.1	high	8.8	6.1	0.14	31	10.6
PS	62	M	34	1	18.8	3.4	143	1.45	0.97	113.9	1.8	high	9.6	4.3	0.05	28	10.5
PS	55	F	33	1	18.7	3.6	131	1.65	1.06	102.9	2.1	high	8.9	5.9	0.05		11
PS	51	M	31	1	17.6	3.4		1.48	1.12	88.36	1.8	high		1.9	1.45	19	10.9
PS	49	M	26	1	21.5	4.1		1.37	1.05	90.11	1.3	high		2.4	0.02	21	11
PS	55	F	26	1	18.2	3.8		1.1	0.72	115.9	1.5	high		4	0.06	21	14.1
PS	48	M	25	1	21.3	4.1	122	1.48	1.24	96.15	1.8	high		6	0.12	32	10
PS	58	M	24	1	21.7	3.6	180	1.45	1.07	105.8	1.9	high	9	5.4	0.51	27	11.4
PS	43	M	24	1	22.9	3.6	135	1.53	0.98	88.75	1.6	high	9.2	5.9	0	28	9.9
PS	34	F	23	1	22.6	3.8	188	1.26	1	119.5	1.5	high	8.2	5.3	0.3		7.5
PS	55	M	23	1	18.2	3.7	195	1.68	0.98	97.87	1.5	high	8	6.3	0.05	21	10.5
PS	61	M	22	1	22.4	3.6	130	0.97	1.03	132.1	2.2	high	9.4	5.1		26	10
PS	68	F	22	1	18.7	3.8	176	1.35	0.86	99.64	1.1	high	7.9	4.4	0.1	32	10.9
PS	66	F	21	1	16.4	3.5	103	1.17	0.65	70.41	1.8	high	7.7	6.3	1.3	32	12.3
PS	53	M	18	1	20.1	3.6	121	0.97	0.86	100.9	1.8	high		4.9	0.05	25	9.9
PS	65	M	17	1	21.5	3.7	143	1.39	1.14	103.7	1.5	high	9.7	4.6	0.07	33	11.8
PS	56	M	17	1	18.3	3.2	193	1.39	0.96	96.22	0.8	high	8	3.7		24	9.5
PS	60	M	17	1	19.3	3.8	194	1.24	0.96	108.1	1.6	high		5.4	1.65	37	11.8
PS	73	M	16	1	19.6	3.8	182	1.65	0.81	104.8	1.5	high	8.2	7.1	0.05		11.3
PS	50	M	16	1	20.1	3.4	128	1.44	0.92	110.1	1.4	high		3.7	0.05	29	11.7
PS	46	F	16	1	20.3	3.7	140	1.75	0.92	74.17	1.8	high	9.5	5.4	0.34		12.1
PS	58	M	16	1	20.7	3.6	174	1.47	1.03	118.2	1.8	high	9.4	6.4	0.05	27	11
PS	73	F	16	1	21	3.4		0.92	0.96	108.4	2.1	high	9.5	6	13.39		11.3
PS	47	M	15	1	22.5	3.6	187	1.41	0.93	114.7	1.9	high	8.4	4.2		15	11.3
PS	44	F	15	1	21.9	3.5	152	1.38	0.92	119.9	1.5	high	9.9	6.1	0.14	23	11
PS	68	M	14	1	19.4	3.8	207	1.49	0.98	93.36	1.8	high	9.4	4.9	0		10.1
PS	60	M	14	1	23.7	3.5	123	1.37	1.09	104.6	1.8	high	9.5	6.6	0.02	45	6.9
PS	59	M	13	1	22.2	4	179	1.84	1.11	114.5	1.6	high	10.9	4.8	0.04	25	11.4
PS	54	M	13	1	21.7	3.7	134	1.42	0.89	118.8	1.8	high	8.8	3.7	0.51	27	9.3
PS	54	M	13	1	19.9	3.4	133	1.34	0.96	109.6	1.5	high		5	0.2	28	10
PS	65	M	12	1	26.2	3.5	101	1.57	0.66	103.1	1.1	high	7.9	5.2	0.7	30	10.5
PS	72	F	12	1	25.2	3.8	110	1.24	0.95	110.6	1.6	high	8.8	7.1		30	9.1
PS	55	M	10	1	21	3.9	164	1.68	0.98	114.9	1.8	high	10.4	4.8	0.05	27	11.6
PS	49	M	9	1	17.1	3.6	143	1.37	1.03	93.11	1.6	high		5.5	0.47	32	10.1
PS	68	F	9	1	22.8	3.9	149	1.4	1.07	103.5	1.5	high	9.3	6.1	0.3	26	10.6
PS	70	M	9	1	21.2	2.9	141	2.5	1.01	123.4	1.8	high	9.2	5.1	0.05	26	12.1
PS	62	M	8	1	22.3	3.5	204	1.31	0.91	106.3	1.5	high	9.6	5.2	0	16	12.1
PS	59	M	8	1	18.1	3.6	155	1.7	0.76	87.95	1.8	high	9.5	5.1	0.09	29	12.8
PS	50	M	7	1	21.5	3.4	150	1.29	0.97	90.68	1.6	high	10.1	8.2	0.18	30	8
PS	41	M	7	1	21.5	4	159	1.9	0.96	101.6	1.3	high	9.2	4.1	1.28	30	9.6
PS	51	M	6	1	21.6	4	162	1.28	0.86	72.53	1.3	high	7.6	4.6	0.2	19	13.6
PS	60	F	6	1	25.1	3.4	119	1.16	0.69	103.9	1.3	high		6.1	0.7	36	12
PS	57	F	6	1	18.4	3.6		1.78	1.15	121.7	1.5	high		3.3			13.7
PS	61	M	6	1	19.4	3.2	101	1.48	0.79	95.73	2.1	high	9.2	4.7	0.08	45	9.9
PS	65	F	6	1	17.3	3.1	154	1.71	0.93	99.2	1.1	high	7.7	6	0.28	33	11
PS	70	F	6	1	18.5	3.6	88	1.4	1.08	93.98	1.6	high	8.5	3.7		18	10.4
PS	57	M	6	1	24.8	3.8	130	1.03	0.96	111.9	1.8	high	8.9	3.7	0.11	31	8.6
PS	61	M	5	1	25.8	3.7	150	1.08	0.91	138.1	1.5	high	9.4	4.7	0.23	26	11
PS	59	F	5	1	26	3.7	128	1.03	0.96	136.7	1.8	high	9.3	5.4	0.07	24	12
PS	54	F	5	1	24.4	3.8	164	1.29	1.01	101.2	1.8	high	9.5	4.8	0		9.9

PS	70	M	5	1	21.8	3.7	130	1.77	0.9	123.6	1.8	high	8.2	4.4			9.9
PS	68	M	4	1	23	3.7		2.01	0.93	122.5	2.1	high		7.3			10
PS	54	F	4	1	22.3	3.5	113	1.35	0.71	95.27	1.5	high	8.3	5	0.11	27	11
PS	21	F	4	1	21.3	3.6	127	1.26	1.22	95.49	1.8	high		4.3	0.3	17	10
PS	69	M	3	1	26.9	3.9	187	1.48	0.99	110.6	2.1	high	10.8	5.5		30	14
PS	62	M	2	1	23.9	3.7	144	1.66	0.98	99.76	1.5	high	8.3	4.2	0.16	28	10.9
PS	76	F	28	1	17.9	4.3	134	1.59	0.96	139.7	1.6	high	9.4	6.3		22	9.6
PS	63	M	2	1	26.1	3.7	208	1.11	0.93	83.78	2.1	high	9.3	6.1			10.1
PS	59	M	2	1	20.3	3.8	116	1.48	0.93	95.05	1.5	high	9	4.2			9.3
PS	57	F	2	1	18	3.5	163	1.28	0.9	71.88	1.3	high	9.5	3.9	0.14	28	11.2
PS	54	M	2	1	22.9	4	142	0.99	1.16	108.4	2.1	high	8.9	5.4	0		10.5
PS	39	M	7	1	24.1	3.7	199	1.53	0.75	134.2	1.5	high	8.6	4.6	0.1	24	9.8
PS	52	F	19	1	17.4	3.8	142	1.49	1.13	106.4	1.6	high	8.4	5.3		20	9.6
PS	59	M	5	1	23.9	4.1	218	1.68	0.82		1.1	high	10.5	5	0.2	27	11.2
PS	75	F	36	1	17.1	3.6	143	1.54	0.91	82.36	1.6	high		5.4	0	39	10.5
PS	54	F	18	1	24.9	3.7	126	1.21	0.73	75.41	2.1	high	8.3	8.3	0	31	9
PS	64	F	10	1	18.1	3.8	110	1.96	0.99	138.7	1.5	high	8.8	4.8	0.06	24	10.3
PS	68	F	11	1	22.5	3.4	175	1.2	1.17	141.1	1.5	high		5.9	0.13	25	8.5
PS	47	F	6	1	19.1	3.7	153	1.31	1.07	126.3	1.3	high	9.6	5.8	3.3	32	8.8
PS	71	F	6	1	21.1	4.1	166	1.16	1.15	147.8	1.4	high		6	0.6	27	10.3
PS	57	M	5	1	21.7	3.9	169	1.21	0.68	95.35	2.1	high	9.3	4.8	0.5	25	9.9
PS	67	F	4	1	23.9	3.8	139	1.33	1.03	113.4	2.1	high	11.5	6.3		29	10.5
PS	66	F	2	1	24.5	4	182	1.59	0.85	94.83	1.8	high	9.9	6.4		27	9.4
PS	51	F	2	1	21.1	3.8	130	1.9	0.93	124.3	1.4	high	9.1	4.3	0.05	33	10.2
PS	87	F	2	1	18.6	3.9	128	1.42	0.79	57.58	1.5	high	9.2	4.3	0.1	32	7.4
PS	57	M	15	1	19.3	4	164	1.56	0.92	130.3	1.6	high	8.5	2		22	12.7
PS	34	M	12	1	20.2	4	178	1.69	0.57	92.07	1.9	high	10.2	8.2		19	9.9
PS	46	M	8	1	26.1	4.1	270	1.2	0.95	146.9	1.8	high		7.4	0.3	29	9.9
PS	61	M	5	1	23.2	3.6		1.33	0.8	108.8	1.5	high		3.1	0.24	40	10.7
PS	60	M	7	1	24.3	3.5	197	1.66	0.8	110.3	1.5	high	8.2	3	0.03	22	10.7
PS	49	F	16	1	21	3.9	146	1.23	1.13	106.7	1.6	high		7			8.9
PS	34	M	14	1	22.9	4	129	2.14	0.88	138.5	1.8	high	8.7	5.4	1.68	31	7.6
PS	72	M	20	1	22.1	3.8	171	1.47	1.07	110.9	1.9	high	8	5.8		29	10.7
PS	77	F	14	1	16.1	3.5	224	1.37	0.73	131.9	1.6	high	8.5	7.3		34	13.1
PS	36	F	2	1	18.5	4	179	1.69	0.87	118.2	2.1	high	9.1	5.4	0.1	25	9.1
PS	22	M	4	1	16.6	3.9	159	1.28	1.21	102.6	1.6	high	9.3	4.2		22	10.5
PS	48	F	29	1	18.9	4	132	1.5	0.89	123	1.9	high	8.3	5.7		23	11.8
PS	69	M	13	1	21.2	3.5	158	1.17	1.11	117.9	1.5	high	8.2	4.5		23	8.8
PS	61	M	6	1	24.7	3.6	116	1.48	1.16	141.4	1.6	high	8.5	7.3		24	12.9
PS	64	M	14	1	21.4	3.6	99	1.37	0.86	76.49	1.6	high	8.5	4.2		20	11.9
PS	67	M	13	1	20.8	3.8	111	1.3	1.05	136	1.6	high	7.7	3.5		22	9.3
PS	57	M	11	1	19.3	3.9	139	1.29	0.8	110.8	1.9	high	8.7	5.5		18	9.3
PS	63	M	17	1	18.6	3.2	180	1.4	0.93	85.43	1.6	high	8.4	5.2		16	10.1
PS	48	F	17	1	17.1	3.8		1.57	1.08	123.8	1.8	high	8.3	6.4	0	26	14.1
PS	74	M	7	1	21.3	3.6	116	1.3	0.91	107.3	2.1	high	8.4	5.7	0.09	42	11.8
PS	57	M	13	1	24.3	3.2	191	1.31	1.03	145.2	1.6	high	7.7	4.3	0.37		10.4
PS	69	F	3	1	18.6	3.9	115	2.23	0.84	96.97	1.9	high	8.7	5.9	0.42	23	12.2
PS	59	F	7	1	20.7	3.7	180	1.62	0.94	103.5	1.8	high	10	4.6	0.1	28	10.7
PS	52	F	6	1	26.1	3.5	117	1.31	0.95	85.21	1.5	high	8.2	5.9	0.83	31	13.6
PS	62	M	9	1	24.5	4	71	1.85	0.88	128.1	1.6	high	9.1	5.8	0.14	31	11.3
PS	68	M	6	1	24	3.9	158	2.01	0.71	132.2	1.3	high	9.7	5	0.09	35	11.1
PS	62	M	11	1	26.7	3.4	135	2.25	0.66	98.48	1.3	high	9.6	3.7	0.09	33	8.7
PS	71	F	31	1	19.6	3.2	157	1.82	0.89	77.56	1.6	high	8.8	4.7	0.08	32	10.8
PS	56	M	16	1	23.4	3.9	136	1.54	0.93	128.7	1.6	high	7.7	3.9	0.37	31	8.8
PS	61	F	16	1	18.5	4	117	2.05	1.07	116.8	1.5	high	10.3	4.6	1.89	25	10.2
PS	46	F	9	1	17.3	3.7	175	1.57	1.14	111.5	1.5	high	9.3	5.6	0.28	27	9.2
PS	44	M	29	1	22.2	4.4	168	1.15	1.03	105.1	1.4	high		5.9	0.05	28	11.5
PS	37	F	11	1	18.6	4.1	133	1.68	1.35	109.9	1.9	high	8.4	4.4	0.03	33	11.1
PS	43	F	10	1	19.2	4.1		1.19	0.98	138.3	1.3	high	10.5	4.2		31	11

PS	54	M	26	1	20.5	3.7	125	1.92	0.96	153.2	1.5	high	9.5	6.4	0.07	32	10.1
PS	58	F	19	1	19.7	4	128	1.96	1.23	143.2	1.9	high	8	4.9	0.14	35	10
PS	57	M	18	1	22.4	4	167	1.38	0.98	142.5	2.1	high	9.5	6.4	0.16		12.3
PS	71	F	15	1	23	3.7	174	1.23	1.14	109.9	1.4	high	8.5	7.5	3.27	36	10.5
PS	66	F	35	1	20.9	3.7	135	1.71	1.17	105.2	1.6	high	10.1	4.9	0		10.9
PS	72	F	31	1	22.5	3.2	208	1.18	0.68	103.3	1.8	high	8.5	4.6	0	17	12
PS	58	F	23	1	19.3	4.1	116	1.54	1.41	156.2	1.5	high	8.6	5	0.03	25	11
PS	66	M	12	1	19.3	3.9		1.18	1.17	131.1	1.6	high	9.1	6		34	9.6
PS	79	F	6	1	23.6	3.7	153	1.17	0.9	81.77	2.2	high	8.5	5.1	0.9	26	12.1
PS	68	F	21	1	18.3	3.8	121	1.3	1.38	108.6	1.3	high	8.1	2.6	0.03	21	10.1
PS	51	M	18	1	18.4	4.1	167	1.28	1.41	92.65	2.5	high	9.2	5.2	0.1	27	14.9
PS	73	F	6	1	25.3	3.5	120	1.54	1.02	150.8	1.8	high	9.2	3.3	0.37	38	9.8
PS	61	M	14	1	19.6	3.6	137	1.14	1.06	164.6	2.1	high	10.1	7.5	0.05	32	12.2
PS	79	M	18	1	19.9	4.1	189	1.25	1.15	163.4	1.8	high	8.8	4	2.5		9.1
PS	40	M	14	1	16.8	4	210	1.4	1.16	119.3	2.1	high	9.8	8.3	0.12	29	11.4
PS	57	F	33	1	23.7	3.9	92	1.91	1.23	133.3	1.6	high	9.1	3.3	0.39	30	10.2
PS	59	F	26	1	19.1	3.7	177	1.45	1.09	138.2	1.8	high	10.8	7.1	0		12.4
PS	65	F	18	1	25.3	3.5	184	1.2	0.91	141.5	1.5	high	8.7	4.5	0.32	17	9.8
PS	51	M	6	1	33.2	3.8	207	1.51	1.23	107.9	1.5	high		6.5	9.9	21	11.2
PS	47	M	5	1	30.6	3.2	109	1.47	1	107.5	1.3	high	7.5	1.5	1.6	25	10.8
PS	60	M	21	1	21.5	4.3	187	1.14	0.78	92.84	2.1	high	10.3	6.4	0.05		9.6
PS	73	M	18	1	17.8	4.1	149	1.53	1.27	143.5	1.5	high	9.2	3.4			8.7
PS	64	M	6	1	17.2	3.9	211	1.18	1.1	124.6	1.4	high		5	0.38	19	12
PS	64	M	25	1	24.2	3.4	194	1.52	0.89	128.6	1.4	high	9.1	5.7	0.1	32	12
PS	43	M	20	1	26.3	4.1	140	1.43	0.98	116.4	2.1	high	9.2	7.4	0.47		9.5
PS	81	F	14	1	22.6	3.6		1.11	0.96	110.1	2.1	high	8.5	3.4		28	12.9
PS	48	M	4	1	17.8	4.5	114	1.37	1.31	131.6	1.8	high	9.6	5.2	0.05	23	10.9
PS	52	F	32	1	20.9	3.6	134	1.04	0.89	87.32	1.6	high	7.9	4.5	0.24	37	9.9
PS	61	M	28	1	21.2	3.8	72	1.54	0.98	108	1.9	high	9.4	5.4	0.3	30	10.5
PS	45	F	13	1	16.6	4.1	117	1.62	0.94	89.43	2.1	high	9.4	5.5	0.1	24	9
PS	58	M	17	1	24	3.8	110	1.36	1	137.3	2.5	high	9.6	6.1	0.05		13
PS	67	F	16	1	17.3	3.6	163	1.67	1.07	134.8	2.1	high	9.2	7.3	0.16		11.1
PS	60	M	27	1	21.1	3.3	142	1.36	1.2	117.3	1.4	high	8.1	3.7	0.1	29	9.9
PS	63	M	31	1	21.1	3.7	165	1.35	1.31	131.6	1.6	high	9.2	1.8		36	7.5
PS	24	M	4	1	16.5	4.3	142	1.29	1.12	85.45	2.1	high	9.8	8.2	0.08		6.2
PS	60	F	27	1	22.2	3.5	136	1.41	1	117.9	2.1	high	9.7	4.9	0	26	10.3
PS	60	F	14	1	30.2	3.6		1.71	1.01	118.3	1.5	high	8.2	5.8	0.05	26	10.3
PS	44	M	13	1	19.6	4.2	178	1.35	1	92.2	1.8	high	9.3	6.9	0.08		9.9
PS	58	F	5	1	20.8	3.9	164	0.99	0.94	85.85	1.5	high	9.2	3.7	0	27	9.9
PS	73	M	20	1	18.1	3.4	112	1.53	0.93	97.29	2.1	high	9.6	5.7	0.05		10.7
PS	72	F	8	1	27.8	3.4		1.02	0.81	124.3	1.6	high	10	4.3	0.05		9.9
PS	63	M	7	1	21.9	3.8	83	1.67	0.69	113.6	1.5	high	8.7	7		25	10
PS	56	M	5	1	21.6	3.7	168	1.59	0.83	97.35	1.3	high	9.4	0.5	6.1	25	8
PS	58	M	3	1	26.5	4	139	1.68	0.88	112.6	1.5	high	10.6	5.8	0.2	24	8.5
PS	64	F	16	1	17.4		110	1.5	0.65	64.62	2.1	high	9.6	6.5	0.65	30	11.6
PS	43	M	13	1	27.7		159	1.21	0.73	78.48	1.8	high	8.4	4.4	0.21	32	10.3
PS	45	F	12	1	18.7		163	1.4	0.72	106.2	2.1	high	9.9	5.5	0.11		10.9
PS	64	M	6	1	20.9		162	2.35	0.81	100.9	1.8	high	9.2	2.5	0.09		11.1
PS	41	M	2	1	21.6		134	1.33	1.06	81.29	1.5	high	8.9	10.4	0.11	24	8.9
PS	71	F	2	1	25.2	3.8	162	1.7	0.58	91.51	1.9	high	9.4	4.3	0.07	35	11.4
PS	46	M	8	1	19.6	3.2	98	1.15	0.88	107.4	1.8	high	8.2	2.9		16	10.1
PS	62	F	10	1	21.3	2.6	78	1.6	0.93	124.1	1.9	high	9	7.5	1.49	47	12.3
PS	85	M	4	1	19.8	3.9	115	1.41	0.83	147.3	1.3	high	6.2	8.4		35	10.9
PS	59	M	35	1	22.1	3.5	131	1.23	1.02	103.7	2.2	high	8.5	5.9	0.1		10.5
PS	59	F	5	1	22.7	3.8	146	1.11	0.96	122.8	1.1	high		5.6		21	12.8
PS	52	M	9	1	18.9	3.8	170	1.8	1.36	102.7	0.8	high		2.9		34	8.9
PS	44	M	6	1	29.3	4.2		1.61	0.74	98.46	2.1	high	8.6	7		21	11.2
PS	66	M	10	1	21.7	3.9	127	1.43	1.06	134.8	1.5	high	8.2	7	0.3	55	9.7
PS	70	F	5	1	26.7		105	1.5	0.85	118.8	2.1	high	10.2	4.2	0.05		8.2

PS	70	M	14	1	25.3	3.5	224	1.58	0.7	94.53	1.8	high	8.5	5.7	0	22	10.8
PS	73	F	24	1	21.4	3.6	195	1.35	0.8	63.32	1.3	high		5.8	1.37		10
PS	50	F	4	1	26.2	3.7	155	1.4	0.78	108.1	2.1	high	8.5	4.4	0.11	27	10.1
PS	74	M	5	1	20.1	3.4	142	2.05	0.63	101.1	1.9	high	9.7	3.1	0.02	35	11.2
PS	73	M	2	1	19	3.6	123	1.7	1.13	78.47	1.5	high	9.8	6.7	0.71	35	7.6
PS	67	M	20	1	14.8	3.8	146	1.48	0.81	102.7	1.9	high	9.6	7.1	0.03	33	11.6
PS	75	F	14	1	16.7	3.6	100	1.87	0.91	124.5	1.6	high	9.7	5.9	0.04	29	11.4
PS	75	F	2	1	19.4	3.7	175	1.87	0.65	99	1.5	high	9.3	3.9	0.17	31	12
PS	60	F	4	1	27.3	4	148	1.5	0.57	56.83	1.8	high	9.2	6.9	0.3	0	10.3
PS	72	M	16	1	19.9	3.3	187	1.45	0.81	107.2	1.5	high	8.8	8.9	0.1		10.7
PS	63	M	10	1	18.8	4.1	145	1.6	1.05	73.59	1.6	high	9.5	5	0.13	25	10.8
PS	64	M	2	1	20.8	3.9	148	1.33	0.83	89.49	1.6	high	9.6	5.9	0.05	29	11.6
PS	65	F	30	1	15.6	3.5	193	0.87	0.95	70.97	1.3	high	9.7	4.4			12.2
PS	40	M	26	1	21.3	3.7	143	1.55	1.03	87.49	1.5	high	8.9	5.5	0.22	32	12
PS	71	F	18	1	29	3.5	127	1.57	1.25	109.7	1.4	high	9.6	4.4	0.05	21	11.5
PS	65	F	17	1	18.4	3.8	130	1.42	1.27	117	1.8	high	9.5	5.8	0	18	15.2
PS	69	F	15	1	17.7	3.5	129	1.08	1.32	111.4	1.3	high	8.6	5.2	0.08	26	10.2
PS	74	F	15	1	21.8	3.9	146	1.45	0.98	146.9	1.4	high	9.4	6.7	0.08	26	9.5
PS	72	M	11	1	18.3	3.9	102	1.76	0.84	107	1.9	high	8.8	3.9	0.01	30	10.6
PS	55	F	8	1	18.7	3.8	147	1.05	1	107.7	1	high		4.4	0.16	10	10.7
PS	88	F	6	1	18.6	3.7	138	2.2	1	138.4	1.8	high	9.2	4.3	0.09		10.8
PS	55	F	5	1	18.2	4.1	151	1.61	0.99	113.7	1.8	high	10.2	4.9	0		10.5
PS	31	M	5	1	22.5	4	176	1.14	0.72	85.43	1.8	high	8.5	7.7	0.3		10
PS	50	F	5	1	29.7	3.6		1.44	0.91	104.1	1.8	high	8.5	4.7		44	9.3
PS	74	M	5	1	22	3.7	157	1.32	0.91	129.6	2.1	high	9.5	4.2	1.04	25	10.7
PS	62	M	4	1	23.5	3.7	191	1.67	0.85	72.88	2.1	high	9.9	4.8		25	10.8
PS	56	F	4	1	27.8	3.8		1.31	0.95	90.4	2.5	high	9.7	7.8	0.49		9.8
PS	67	M	4	1	19.9	3.7	150	1.3	1.01	135.5	2.1	high	8.4	5.7	0		9.8
PS	62	M	4	1	17.9	4	104	1.48	0.81	124.2	1.9	high	9.1	3.9	0.02	29	10.1
PS	74	F	3	1	26.3	3.3	145	1.92	1.06	64.38	1.3	high	10.3	6.3	0.04	24	11.3
PS	68	M	3	1	20.5	3.7	202	1	1.08	97.31	1.5	high	8.9	5.7			6.1
PS	69	M	3	1	21.3	3.8	114	2.69	0.82	89.2	2.5	high	7.6	4.6	0.03	30	11.5
PS	67	F	3	1	26.6	4.1	106	1.68	0.7	45.69	1.6	high	8.7	5.2	0.29	49	11.4
PS	59	F	2	1	20.2	3.3	140	1.01	0.93	114.3	1.8	high	9.3	5.8	0		10.8
PS	77	M	2	1	19.2	3.9	80	1.34	1	83.58	1.6	high	8.4	4.1	0.06	28	12
PS	59	M	2	1	22.1	3.6		1.41	0.67	71.62	1.5	high	9.4	3.6	3.74		10.9
PS	71	M	2	1	23.1	3.7	97	1.34	0.79	59.65	1.3	high	8.3	7.2	0.14	33	8.1
PS	60	M	2	1	17.4	3.9	109	1.44	1.25	90.08	2.1	high	8.7	6.4	3.03	22	10.4
PS	57	F	2	1	16.6	3.8	130	1.59	1.23	102.6	1.5	high		5.2	0.05	26	9
PS	72	M	7	1	27.4	3.9	142	1.26	0.85	100.3	2.2	high		4.9		23	11.1
PS	58	F	12	1	18.3	3.7	149	1.16	1.33	102.3	2.2	high	9.1	4	0	30	10.3
PS	64	M	2	1	21.1	3.8	164	1.62	1.01	92.35	1.9	high	8.9	5.9	0.04	27	11
PS	61	M	19	1	30.7	3.7	112	1.52	0.99	131.4	1.6	high	9.1	3.4	23.27		10.8
PS	62	M	13	1	24.2	3.8	183	1	1.04	135.8	1.5	high		5.6	0.1	18	9.5
PS	56	M	15	1	18.4	3.8	125	1.29	1.03	96.34	2.1	high	10.3	6.5	0.58	24	10.7
PS	55	F	3	1	24.3	3.5	225	1.72	0.87	49.65	2.1	high		5.6	0.78	27	10.9
PS	67	F	11	1	21.3	3.4	185	2.3	0.79	95.99	1.8	high	9	7.4	0.14	24	8
PS	61	M	7	1	20.7	3.8	124	1.04	0.79	94.88	1.5	high	9.4	4.9	0.1	19	11.1
PS	60	M	4	1	20.2	3.8	126	1.67	1.03	75.82	2.1	high	9.9	5.7	0.05	25	9.5
PS	78	F	4	1	19.7	3.8	115	1.27	1.19	131.8	1.8	high		3.6	0.2		8.9
PS	70	M	2	1	29.1	3.7	217	1.76	0.82	142.6	2	high	9.6	5.2	0.56	27	10.2
PS	79	M	2	1	23.1	3.9	141	1.8	0.84	112.6	1.8	high	10	3.4	1.61	27	11.1
PS	72	F	2	1	23.7	3.6	188	1.77	0.82	50.63	2	high	8.4	4.4		14	10.8
PS	61	M	3	1	20.7	3.7	156	1.39	0.98	95.06	1.5	high	9.2	5.3	0.1	21	
PS	71	F	9	1	19.6	3.9	186	1.26	0.82	104	1.8	high	7.5	4.1	0.6	26	10
PS	45	F	7	1	15.7	2.8	144	1.38	0.88	78.26	1.5	high	8.9	6.3	0	15	9.2
PS	41	M	2	1	22.5	4.2	186	1.32	1.06	127	1.6	high		4.4	0.23	33	14.9
PS	74	F	14	1	24.8	3.8	155	1.46	0.9	98.7	2.5	high	9.1	5	0.06		11.5
PS	33	M	8	1	26.1	4.4	127	1.8	0.86	117.7	1.9	high	8.1	5.1	0.4	31	11.9

PS	42	F	4	1	20.4	3.9	159	1.47	1.03	121.2	1.8	high	8.6	6.3		25	11.1
PS	56	M	3	1	29.3	4.2		1.23	0.69	62.58	2.1	high	9.3	4.2		42	9.6
PS	64	F	15	1	17.8	3.9	148	1.33	1.19	122	1.9	high	9.3	5.2	0.2	21	11.6
PS	65	M	12	1	19.3	3.4	130	1.24	0.97	150.1	1.5	high	8.8	5.6		21	11.9
PS	70	M	18	1	23	3.4	153	1.32	0.91	113.8	2.1	high	9.7	5.4	0.11		12.4
PS	68	M	18	1	19.8	3.7	146	1.36	1.1	108.5	1.9	high	9.3	4.2	0.2	21	11.5
PS	62	M	15	1	19.4	3.9	149	1.7	1.1	130	1.3	high	9.4	4.3	0.1	22	9.8
PS	37	F	10	1	21.1	4	111	1.35	1.19	134.3	1.3	high	8.3	4.8	0.3	28	10.9
PS	35	M	4	1	27.9	4.6	209	1.62	0.94	91.09	1.5	high	9.6	5.9			10
PS	61	F	20	1	17.8	3.2	140	1.54	0.88	86.73	2.1	high	8.8	1.8	0.1	21	9.3
PS	32	F	7	1	24.3	4	139	1.35	1.07	96.93	1.6	high	10	4.3	0.1	16	11.3
PS	62	F	8	1	26.2	3.6	212	1.51	1.15	146.6	2.1	high	10.2	3.5	0.39	28	10.1
PS	39	F	8	1	18.3	3.8	134	1.03	1.33	103.8	1.3	high	7.1	2.5	0.05	26	6.9
PS	43	M	14	1	21.8	3.6	137	1.28	0.82	143.9	2.1	high	9.9	5	0.88	19	9.2
PS	60	M	2	1	17.9	2.5	117	1.61	1.41	118.9	2.1	high	7.6	3.9	1.03	24	9.1
PS	67	M	15	1	18.7	3.6	142	1.9	1.01	92.25	1.5	high	8.7	5.3		23	10.2
PS	73	M	2	1	20.7	3.7	144	1.7	0.84	70.6	1.1	high	9.7	3.5		36	9.1
PS	66	F	23	1	24.7	4	220	1.02	1.13	116.8	1.5	high		3.9	0.08		10.9
PS	59	M	32	1	16.4	3.8	165	1.46	1.01	102.2	1.3	high	7.9	4.1			8.7
PS	32	F	17	1	16.5	4	136	1.56	0.95	91	1.6	high	7.8	4	2.2	22	7.4
PS	64	F	12	1	21.6	3.6	152	1.64	0.8	93.37	1.5	high	8.4	4.3	0.02	23	10.7
PS	71	M	19	1	18.2	3.8	147	1.75	1.1	101.4	1.6	high	9.5	3.1	0.1	24	9.5
PS	64	F	8	1	22.5	3.8	175	1.42	0.94	128.6	2.1	high	9.4	5.8	4.25	29	10.6
PS	43	M	3	1	32.6	3.7	99	1.22	0.77	77.53	1.8	high	8.8	7.9	0.03	38	10.9
PS	68	M	6	1	16.9	3.5		1.07	0.93	97.16	1.5	high	8.7	5.4		26	11.3
PS	60	M	2	1	22.8	4.5	170	1.22	0.81	119	2.1	high	8.1	7.1	0.1		10.6
PS	35	F	3	1	25	4.1	162	1.58	0.96	107.1	1.6	high	10.1	7.6	0.11	27	9.4
PS	72	M	3	1	19.9	3.5	127	0.91	0.98	134.8	2.1	high	8.5	5.3	0.16	30	10.7
PS	60	F	9	1	22.3	3.6	121	1.68	0.79	86.69	1.9	high		6.2		24	10.1
PS	58	M	13	1	21.9	3.6	147	1.64	0.83	112.8	2.1	high	9.5	6.3		26	12.4
PS	71	M	10	1	26	3.5	153	1.16	0.91	118.1	1.8	high	10.5	5.8	0.06	18	8.9
PS	60	M	8	1	19.4	3.8	166	0.88	0.7	109.8	2.1	high	8.6	7.8	0.21	31	13.9
PS	70	M	8	1	21.1	3.8	168	1.61	0.82	114.1	1.5	high	10	5.3	0.02	37	11
PS	53	M	6	1	24.3	3.9	123	1.49	1.13	135.2	1.9	high		6		26	9.9
PS	39	F	5	1	25.9	3.7	144	1.93	1.13	103.9	1.8	high	9.9	3.5	0.05	22	11.6
PS	64	M	4	1	33.2	3.9	98	1.32	0.84	83.89	1.5	high		5.5	0.24	29	10.7
PS	57	M	4	1	26.5	4.1	205	1.57	0.89	119.8	1.3	high	9	4.1	0.1		11.1
PS	65	M	4	1	28.4	3.9	113	1.42	0.95	75.75	2.1	high	8.7	4.6	0	27	10.7
PS	45	M	3	1	20.5	4	124	1.02	0.77	82.09	2.1	high		6.3	0.1	25	10.1
PS	73	F	3	1	23.7	3.7		1.34	0.67	87.55	1.5	high	9.4	5.3		26	10.1
PS	55	M	26	1	18	4	155	1.46	0.91	116.6	1.9	high	8.9	4.8			12.5
PS	84	F	2	1	21.3	3	160	1.12	0.93	87.63	2.5	high	11.4	5.3	0.15	26	11
PS	65	M	9	1	20.6	3.7	119	1.48	0.76	95.51	2.1	high	8.4	4.2	0.1	45	10.5
PS	62	M	8	1	18.9	3.5	140	1.08	0.74	90.69	1.3	high		4.5	0.24	31	10.4
PS	65	F	6	1	20.1	4.2	138	1.49	0.83	106.8	1.8	high	9.7	6.1	0.07	28	10.5
PS	67	M	5	1	20.4	4.1	135	1.41	0.58	84.52	2.1	high	9.5	5.9	2.63	32	9.7
PS	61	M	12	1	18	3.4	160	1.15	0.92	132.9	1.5	high	8.5	3.9	0		10.9
PS	66	F	4	1	22.3	3.9	156	1.82	0.99	136.4	1.4	high		4.1	0.3		11.3
PS	72	M	4	1	17.9	3.6	176	1.48	0.71	87.32	1.8	high	9	4.6	0.11	35	11
PS	66	M	2	1	20	4.2	165	1.54	0.77	115.8	1.3	high		5.4	0.06	26	10.7
PS	72	F	5	1	27.4	3.2		1.45	0.65	58.05	2.1	high	9.4	3.4		23	8.4
PS	74	M	3	1	15.5	3.2	155	0.96	0.8	83.29	1.3	high	7.9	4.8	0.5	37	9.9
PS	55	F	15	1	25.8	3.7	112	1.4	0.87	90.27	1.5	high	8.2	5.5	0.93	11	11.4
PS	73	F	4	1	22.2	3.5	206	1.57	0.9	115.4	0.6	high		5.1	0.05		9.4
PS	60	M	8	1	29.3	3.3	94	1.93	1.02	109	2.1	high	8.7	4.9		19	10.3
PS	71	F	10	1	23.5	3.7	209	1.34	1.27	105.6	1.5	high	8.7	1.7	11.6	24	8.3
PS	67	M	9	1	22.9	3.6	154	1.48	0.81	106.3	1.8	high	9.1	6.1	0.06	27	10.3
PS	66	M	8	1	20.8	3.7	113	1.46	0.89	94.75	1.5	high	7.8	5.2	0.27	15	10.5
PS	69	M	3	1	26.6	4.2	114	0.52	0.57	83.49	1.3	high	8.6	3.8			10

PS	46	M	6	1	27.9	4.3		1.37	1.03	93.9	2.1	high	9.9	6		29	11.7
PS	64	M	3	1	21.6	4.1	186	1.24	0.8	115.7	1.8	high	9.9	7.7		24	12.1
PS	65	M	5	1	17.9	3.9	124	1.19	0.83	132	1.5	high	9	4.7		31	9.1
PS	65	M	4	1	18.8	4.1	128	1.32	0.79	117.6	1.8	high	7.6	6.6	4.6	24	9.9
PS	61	M	6	1	19.9	4	154	1.08	0.71	120.6	2.1	high	9.4	6.7	6.96		11.7
PS	63	M	2	1	21.5	4.5		1.15	0.82	85.47	1.3	high		5.5			8.3
PS	59	M	3	1	24.4	4	223	1.05	1.08	120.9	1.6	high	8.6	3.5	0.82	21	9.9
PS	64	F	3	1	20.5	4	179	1.62	1.05	89.27	1.5	high	9.4	5	0.05	32	11.6
PS	68	F	2	1	26.1	4		0.92	0.75	82.06	1.3	high		3.5	1.59	30	11.2
PS	65	M	5	1	20.8	3.5	145	1.01	0.61	97.52	1.5	high	9.3	3.1	1.73	32	8.2
PS	41	M	2	1	32.5	4.2		1.37	1.03	96.22	1.8	high	9.2	4.2		25	9.2
PS	53	M	2	1	20.2	4.5	132	1.24	1.2	115.7	1.6	high	9.3	7.3	4.63	28	14.5
PS	47	M	31	1	16.7	4	122	1.09	1.26	64.78	1.9	high	9.9	9.1	0.15	25	11.4
PS	40	M	14	1	22.8	3.4	192	1.55	1.27	59.13	1.8	high	9.7	5	0.07	22	10.8
PS	59	F	22	1	18	4.1	114	1.35	1.1	98.41	1.6	high	10.7	9.3		28	11.9
PS	45	M	21	1	21.4	3.8	166	1.45	1.17	110.1	1.5	high	9.4	5.3	0.11		9.2
PS	68	M	27	1	16.7	3.5	143	1.61	0.78	114.1	2.1	high	8.4	5.6	0.11	28	10.6
PS	36	F	12	1	33.4	3.8	153	1.97	0.92	97.04	1.6	high	9.2	4.5	0.16	27	11.4
PS	65	M	12	1	19.8	4.2	106	1.16	1.1	108.9	1.8	high	8.3	3.4	0.12	26	11
PS	62	F	10	1	21.5	4.1	114	1.13	1.2	119.3	1.3	high	8.7	3.3	1.09	31	7
PS	67	F	30	1	16	3.7	165	1.3	0.81	97.88	1.3	high	7.9	6.9		29	10.5
PS	63	F	10	1	19.4	3.8	177	2.23	1.04	139.3	1.6	high	9.6	4	0.11	27	10.4
PS	67	M	4	1	25	4.2	122	1.12	1.06	85.56	1.5	high	8.2	4.9	0.03	38	11.3
PS	71	M	22	1	22.7	4.1		1.58	1.18	106.6	1.6	high	10.5	4.6			10.5
PS	61	M	10	1	19	4.5	105	1.56	0.87	126.2	1.9	high	9.3	5.1	0.09	31	12.4
PS	75	F	10	1	18.7	3.8	128	1.29	0.91	127.9	1.1	high	9.5	4.7	3.6	29	9
PS	80	M	10	1	24.1	3.5	83	1.17	0.9	152.7	1.8	high	8.9	2.2	7.64	37	11.2
PS	65	M	5	1	21.5	4	136	0.91	0.95	116.1	1.8	high	9.5	7.4	0.09	29	11.1
PS	62	M	5	1	23.7	4.1		0.94	0.78	107.2	2.1	high		6.1	0.27		9.2
PS	80	M	3	1	17.9	4.1		1.45	0.77	97.06	2.1	high	9.5	5.2	0.05	25	9.2
PS	48	M	23	1	24.8	4	171	1.56	0.95	113.4	1.4	high	9.8	7.1	0.5	30	9.8
PS	68	M	7	1	21.2	3.9	161	1.14	0.83	92.85	1.5	high	9.9	5.1	0.43		13.3
PS	77	F	6	1	17.9	3.7	130	1.68	0.98	113.9	1.5	high	10.5	5.1		17	11.1
PS	60	F	24	1	17.3	3.5	183	1.01	1.15	125.9	1.6	high		7			10.2
PS	59	F	16	1	19.4	4.1	126	1.61	1.06	128.2	1.9	high	8.2	4	0.09	19	11.2
PS	47	M	16	1	20.3	4.1		1.41	0.91	126	1.8	high	7.4	4.2	0.56	21	11.1
PS	53	M	15	1	23.4	3.6	125	1.98	0.61	115.2	1.9	high	9.2	6	0.05	26	11.3
PS	54	M	14	1	17.8	3.8	140	2.03	0.9	60.58	2.1	high		3.6	0.02	25	10.7
PS	69	M	9	1	17.3	3.5	143	1.57	0.94	111.1	1.6	high	8.1	5.2	0.09	27	10.5
PS	68	M	6	1	22.9	4.1		1.47	0.73	106.8	1.5	high			0.1		7.1
PS	59	M	5	1	22.8	4.2	154	1.68	1.21	153.8	1	high		5.7	0.27		8.7
PS	72	M	4	1	26.4	3.7	127	1.15	0.43	60.23	2.1	high	10.2	4	0.08		10.8
PS	88	F	2	1	20	3.6		1.36	0.79	115.9	2.1	high		5.1	0.1		9.5
PS	67	F	2	1	23.2	4.3	230	1.37	1.28	123.7	1.8	high	8.2	5.5	0.08	14	
PS	75	M	17	1	20.6	3.9	176	1.22	0.71	71.14	1.8	high	9	4.6	0	12	10.2
PS	76	F	10	1	22.1	3.5	136	1.29	0.89	152.8	1.5	high	8.9	5.3	0		9.3
PS	44	M	4	1	20.3	4.3	160	1.65	0.79	100.8	1.3	high	10.3	4.3	0.1	28	11.2
PS	56	F	9	1	20.9	4	149	1.32	0.68	121.6	1.8	high	8.8	4		38	
PS	61	F	10	1	15.5	3.8	181	1.56	1.08	128.4	1.9	high	10.2	5.5	0.05	29	10.5
PS	61	F	6	1	22.1	3.5	88	1.2	1.07	77.99	1.5	high	8.1	6.3		20	9.5
PS	64	M	10	1	21.8	4.1	145	1.97	0.82	110.6	1.5	high	8.8	7.3	0.39		10.9
PS	74	M	6	1	19.6	4	150	1.89	0.94	120.7	1.8	high	8.3	6.2	0.15	28	11.6
PS	71	F	18	1	18.7	3.9	177	1.38	0.96	146.9	1	high		5.3	0.05		8.8
PS	75	M	2	1	24	4.3	129	1.27	1.18	164.7	1.8	high	9.2	4.6	0.1	31	11.1
PS	64	M	4	1	19.2	4	115	1.36	0.96	117.8	1.6	high	7.1	4.3		19	7.8
PS	76	M	3	1	22.7	3.7	166	1.13	0.94	126	1	high		3.9	0.06		8.6
PS	65	M	16	1	21.3	3.9	162	1.03	0.88	125.2	1	high		6.2	0.09		11.5
PS	55	M	8	1	19.7	2.6	194	1.1	0.71	84.21	1.6	high		6.4			9.9
PS	46	M	11	1	21.1	3.7	145	1.56	0.91	103.2	1.9	high	8.8	3.1	0.26	26	9.1

PS	56	M	28	1	22.5	3.3	140	1.2	0.87	77.08	2.2	high	10.4	6.3	0.1	38	11
PS	58	F	24	1	19	3.5	104	1.1	0.89	105.4	1.8	high	8.9	4.9	0.69	23	9
PS	72	M	23	1	18.1	3.7	157	1.38	0.78	103.7	1.8	high	8.9	5.7	0.1	30	9.6
PS	64	M	22	1	18.5	3.4	165	1.02	0.63	91.74	1.5	high	9.5	4.7	0		10.4
PS	58	M	22	1	20.4	3.7	127	1.49	0.89	111.2	1.8	high	10.2	7	0	22	11.4
PS	72	F	20	1	22.3	3.6		1.86	0.85	112.1	1.6	high	9.8	6.4			11.6
PS	65	F	20	1	22.6	3.9	171	1.49	1.18	132.7	1.3	high	8.5	3.8	0.21	28	11.4
PS	66	M	18	1	21.6	3.6	176	1.22	0.71	97.02	1.5	high	8.2	2.9	0		10.1
PS	65	F	16	1	12.7	2.5	201	1.63	0.8	51.28	1.3	high	9	4.7	1		10.9
PS	67	M	15	1	21.8	3.5	122	1.15	0.78	133.8	1.3	high	9.4	2.9	0		8.3
PS	68	M	15	1	23.9	3.5	149	1.82	0.77	93.65	1.8	high	8.8	6.3	0		10.6
PS	70	M	14	1	21	3.4		1.34	0.89	124.2	1.3	high	8.2	4.1			10
PS	71	M	13	1	23.8	3.2	142	1.3	0.87	101.2	2.1	high	10.2	5	0.55		11.2
PS	38	M	13	1	27.7	3.9	162	1.15	0.6	125.5	1.6	high	8.4	6.4	0.4	24	12.3
PS	76	F	13	1	18.3	3.8	124	1.51	0.98	104.2	1.8	high	9.9	4.8	0.05	32	10.3
PS	71	F	12	1	21.9	3.8	119	1.56	1.16	93.26	1.8	high	9.5	6.4	0.11	18	9.2
PS	57	M	11	1	18.1	3.5	149	1.49	0.79	84.01	1.1	high	8.5	4.8	1.5	24	8.2
PS	48	F	11	1	27.1	3.8	189	1.65	1.07	127.9	1.5	high	8	7.1	0.26	27	10.2
PS	81	M	11	1	16.7	2.9	146	1.46	0.55	60.04	1.9	high	8.8	3.6	0.2	27	10.7
PS	79	F	11	1	15.2	4	144	1.44	0.76	113.2	2.5	high	8.1	4.9	0.06	22	10.8
PS	72	F	11	1	25.6	3.4	110	1.27	0.81	85.51	1.1	high		9	0.07	28	10.5
PS	65	F	11	1	18.4	4.2	166	1.9	0.9	127.9	1.6	high	9.6	3.1	7.67	25	10.1
PS	60	M	11	1	18.1	3.9	153	1.24	0.7	97.12	1.6	high	8.4	5.3	0.71	20	12.3
PS	59	M	11	1	26	3.7	132	1.79	0.85	104.5	1.9	high	9	5.3	0.05	26	11.1
PS	77	F	11	1	16.4	3.9	218	1.22	0.97	143.2	1.8	high	9.4	3.4	0.1	24	11.3
PS	58	M	10	1	24.3	3.6	168	1.49	0.82	95.1	2.1	high	9	3.7	0.06	22	9.3
PS	57	F	9	1	14.3	3.3	154	1.81	0.81	85.45	1.6	high	9.3	6.8	0.1	35	9.4
PS	53	M	8	1	27.3	3.8	128	1.15	0.68	138.7	1.3	high	9.6	5.3	0.98	33	12.5
PS	67	F	8	1	16.4	3.7		1.35	1.16	106.4	2.1	high		6.6	0.24	16	12.3
PS	77	M	7	1	21	3.8	140	1.62	0.89	72.84	2	high	10.6	6.6		20	8.8
PS	57	F	7	1	20.2	4	158	1.46	0.7	46.9	2	high	10.6	5.7			10.6
PS	43	M	7	1	16.9	4.4	159	2.01	1.11	78.04	1.8	high	8.4	3.2	0.89	29	10.4
PS	60	M	6	1	17.1	3.3	184	1.14	0.99	63.23	2	high	9.6	6.4		26	11.4
PS	55	F	6	1	23.2	3.4	132	1.29	1.04	88.35	1.6	high	8.2	4.9	0.17	29	9.8
PS	60	M	5	1	20.2	3.3	161	1.26	0.45	48.93	1.6	high	7.8	3.2	1.15		9.5
PS	43	M	5	1	24.3	3.6	102	1.34	1.14	116.9	2	high	8.3	6.7	0.18	31	10.8
PS	41	M	5	1	21.7	4	162	1.77	1.03	158.6	1.5	high	11.4	5.5	0.02	25	10.7
PS	51	M	4	1	28.4	3.8	153	1.63	0.9	97.64	2.1	high	8.3	7.4		35	10.9
PS	39	F	4	1	29	3.2		1.38	1.05	68.38	1.5	high	8.7	3.6		19	10.1
PS	60	F	4	1	17.2	3.9	86	1.4	1.04	103.2	1.5	high	8.8	4.5			9.8
PS	77	M	4	1	18.1	3.3	135	1.3	0.72	74.13	2.5	high	9.3	7	0	23	10.1
PS	66	M	3	1	24.5	3.9	133	1.69	1.33	126.5	2	high	8.6	4.4	0.05	25	10.6
PS	59	M	3	1	22.5	3.7	143	1.54	0.87	23.57	2	high	8.5	5.7		32	10.4
PS	86	F	3	1	21.4	4.1	219	1.13	0.92	111.6	2	high	11.3	7	0.36	30	11
PS	73	M	2	1	22.8	3.8	227	1.43	0.78	74.66	1.8	high	8.2	8.8	0.05	30	10.6
PS	67	M	2	1	24.6	3.9	156	1.61	0.78	126.1	2.1	high	8.9	4.1	0.03		9.4
PS	61	M	2	1	20.3	4	171	1.08	0.96	81.09	1.6	high	8.9	4.5		11	11.5
PS	63	F	6	1	21.7	3.4	222	2.16	0.74	121.1	1.8	high	8.5	5.3	0	30	10.2
PS	60	M	15	1	24.6	3.4	154	1.27	0.9	130.4	2.1	high	8.6	5.6	0.26	33	9.4
PS	56	M	4	1	25.2	3.4	119	1.6	0.76	120.4	1.6	high	8.7	4.9		24	8.9
PS	69	M	12	1	20.3	3.9	208	1.46	1.34	133	1	high		4.8	5.92	33	10.6
PS	34	M	10	1	22.4	4.3	146	1.33	0.92	101.4	1.8	high	9.5	4.9		23	10.9
PS	59	M	7	1	23	3.8	145	1.39	0.92	84.62	1.5	high	9.5	6.8	0.3	10	
PS	60	F	16	1	18.1	4.1	83	1.76	0.79	93.2	2.1	high	9.6	4.7	0.05		9.5
PS	60	F	5	1	25.8	3.5	213	1.38	1.07	99.85	1.8	high	8.6	3.7	0.51	29	7.1
PS	71	F	4	1	20.8	3.9	206	1.29	0.76	97.96	2.1	high	10.3	6.5	1.36	27	10.2
PS	72	M	4	1	21.6	3.6	168	1.79	0.95	97.23	2.1	high	8.1	7.5	1.78	18	11.6
PS	59	M	3	1	17.6	4.1	111	1.34	1.21	62.93	1.8	high	8.5	4.9	0.07	24	11.4
PS	59	M	5	1	20.8	3		1.1	0.61	44.57	2.1	high	9.2	9.4		29	9

PS	78	F	2	1	21.4	3.9		1.27	0.88	125.7	1.8	high	8	4.8	2.5	13	12.2
PS	61	M	7	1	19.9	4.3	139	1.41	0.82	77.14	1.9	high	8.7	4.3	0.2	22	10.6
PS	54	F	10	1	21.8	4.2	217	1.71	1.01	121.2	1.5	high	10.2	4	0.01	24	9.4
PS	56	M	3	1	31.3	3.3	198	1.61	0.84	82.27	1.6	high	8.3	4.1		22	9.2
PS	47	F	2	1	26.7	3.5	141	1.59	0.68	63.46	1.6	high	9.9	6.2		18	9.9
PS	65	M	13	1	22	3.7	124	1.53	1	93.38	2.1	high	8.9	6.7	0.12	28	10.2
PS	52	F	11	1	24.5	3.9	140	1.36	0.94	134.6	1.5	high	10.2	5.9	0.2	26	10
PS	69	F	12	1	22.6	3.5	167	1.17	0.82	106.9	1.5	high	8.4	4.2		17	10.5
PS	56	M	11	1	35.8	3.8	133	2.41	0.85	123	1.5	high	7.4	1.6	0.63	43	9.6
PS	47	M	8	1	33.9	4	150	1.27	0.94	115.2	2.1	high		5.4	0.07	29	10.1
PS	66	F	14	1	13.9	3.6	115	1.49	0.93	120		high	8	3.9			
PS	64	M	18	1	25.8	3.7	182	1.53	1.11	128	2.1	high	9.8	7.5	0	22	9.6
PS	61	M	4	1	22.3	4.2	97	1.23	0.97	109.4	1.5	high	9.6	6.9	0.25	28	11.7
PS	79	F	3	1	21.9	4.1	165	1.47	0.83	107.3	1.1	high		4.8	0.11	24	10.5
PS	66	F	8	1	20.5	3.6		1.09	1.32	129.1	1.5	high					
PS	55	F	11	1	21.8	3.7	143	1.34	1.26	121.8	1.5	high	9.1	6.8	0.16	28	10.3
PS	45	M	7	1	19.4	3.7	154	1.14	0.85	131.2	2.1	high		6.6		34	9.7
PS	43	M	9	1	34.8	4	213	1.16	0.75	99.06	1.8	high	9.7	3.8	0.05	18	9.9
PS	51	M	11	1	28.3	4.3	194	0.94	0.92	117	1.5	high	9	3	0.05	12	10.4
PS	55	F	11	1	23.5	3.6	113	1.45	1.28	114.2	2.1	high	9.7	4.8	0.15		10.4
PS	48	F	4	1	20.2	3.7	145	1.64	0.91	82	1.1	high	8.7	3.1	0.1		10.3
PS	67	F	25	1	18.3	4	239	1.63	0.88	116.1	1.4	high	9.4	4.9	0.1	30	10.8
PS	57	M	9	1	29.3	3.4	213	1.44	0.83	82.95	1.5	high	9.2	5.1	0.16	25	9.9
PS	58	F	19	1	15.4	4.1	166	1.63	1.12	92.05	1.5	high	10	6.8	0.1	34	10.6
PS	62	M	4	1	18.5	3.9	149	1.68	0.98	71.83	1.3	high	9.4	5.8		26	11.3
PS	60	M	22	1	28.8	3.9	167	1.38	0.84	87.75	1.4	high	8.9	6.6	0.4	18	9.9
PS	65	F	3	1	19.8	3.5	119	0.84	0.97	85.41	2.1	high		4.5	0.05	17	9
PS	55	F	2	1	28.5	4.1	160	1.69	0.73	61.75	1.8	high	8.8	3.7	0.2	20	11.4
PS	77	M	4	1	20.8	4	120	1.22	0.77	132.5	2.1	high	8.4	7.6	0.2	28	9.1
PS	79	F	8	1	23.6	3.3	171	1.54	0.8	103.2	1.6	high	8.8	5.3	0.04	24	11.4
PS	60	F	3	1	21.7	4.7	148	0.68	1.18	107	2.1	high	10	8.6	0.05		11.4
PS	53	M	5	1	25.6	3.7	206	1.47	1.53	110.4	2.5	high	8.4	5.6	0.1	27	10.9
PS	61	M	11	1	25	3.9	196	1.47	0.82	93.56	1.5	high	9.9	5.2	0.1		9.4
PS	58	M	9	1	22.3	4.2	123	1.21	1.08	119	1.9	high	11.4	7	0.62	32	11.7
PS	63	M	4	1	26.4	4.1	168	2.07	0.9	107	1.5	high	8.8	4	0.1	35	11.2
PS	55	F	11	1	19.7		150	1.43	0.91	107.8	1.5	high	8.7	4.8	2.8	24	11.1
PS	65	F	9	1	19.5	3.9	169	1.62	0.79	97.21	1.8	high		3.3	0.06		8.8
PS	66	F	5	1	21.7	4.1	185	1.44	0.85	83.93	2.1	high	8.5	4.7	0		10.5
PS	61	M	2	1	22.6	4	172	1.4	1.11	100.1	1.6	high	8.8	6.3	0.29	27	8.3
PS	45	M	10	1	21.4	4		1.6	0.81	61.9	2.1	high	8.7	6.5		28	13.8
PS	32	F	9	1	24.1	3.8	127	1.36	1.21	96.99	2.5	high	8.7	5.8	0.09	28	9.7
PS	56	M	3	1	22.6	3.8	131	1.68	0.88	125.8	1.6	high	8	4.1	0.62	22	9.9
PS	76	M	3	1	23	3.3	113	1.04	0.71	101.9	1.6	high	9.2	2	3.8	35	10.3
PS	58	F	3	1	24.1	3.7	181	0.97	0.88	97.83	1.8	high	10.6	6.6		32	8.2
PS	64	M	9	1	18.5	3.8	187	1.25	1.13	121.5	1.6	high	9.5	4.4	0.27	25	10.7
PS	67	M	10	1	22.1	4.1	87	1.44	0.88	149	2.1	high	10.4	4.2	4.21	35	8.7
PS	39	F	11	1	18	3.9	184	1.35	0.93	114.1	1.6	high	9.8	3.9	0.09	28	9
PS	55	M	12	1	22.6	4.4	201	1.65	1.56	151.5	1.6	high		7.1	0.06		10.2
PS	62	M	15	1	19.7	3.1	163	1.45	0.73	115.7	2.1	high	10	7.5	0.24	29	12.2
PS	54	M	18	1	18.2	3.6	116	1.48	0.87	128.6	1.3	high		6.8	0.84		10.7
PS	63	M	22	1	22.6	3.6	125	1.47	0.68	109.5	2.1	high	10.3	5.3	0.1	21	7.8
PS	59	F	25	1	15.4	3.4	144	1.41	0.94	119.3	1.1	high	8.4	4.3	0.04	32	10.6
PS	76	M	18	1	19.1	3.8	119	1.16	1.16	154	1.5	high	10	7.8	0.18	36	11.1
PS	77	F	5	1	21.5	3.4	179	1.6	0.91	67.85	1.6	high	8.4	1.3	0.03	22	10.5
PS	60	M	31	1	17.8	3.1	126	0.69	0.84	96.63	2.1	high	8.8	7.2	0.3	33	10.4
PS	41	M	11	1	16.7	4.2	113	0.98	0.68	105.2	1.6	high	9.4	4.3	1.07		10.1
PS	62	M	12	1	22.4		139	1.23	0.78	102.6	1.8	high	8.3	6.8			11.3
PS	67	F	29	1	17	3.6	132	1.35	0.83	87.37	2.5	high	9.7	5.5	0.07	24	9.1
PS	79	F	2	1	18.2	3.7	148	1.95	1.04	126.6	1.8	high	9.3	4.7	0.03	21	9.3

PS	75	M	13	1	18.2	4	174	1.22	0.66	106.8	1.9	high	11	4.9	0.03		11.1
PS	71	F	13	1	18.9	3.7	287	1.83	1.15	123.8	1.8	high		2.4	0.15	21	12.1
PS	77	F	13	1	16.9	4	139	1.53	1.03	147	1.5	high	8.9	3.7	0	21	10
PS	77	F	4	1	17.6	4	175	1.15	1	78.77	1.3	high	9.2	3.8	0.12		8.4
PS	77	M	20	1	23.4	4.2	121	1.25	0.8	118.9	1.5	high		6.8	0.1	28	11.2
PS	67	M	13	1	18.2	3.7		2.11	1.12	120	1.5	high	8.6	4.6			10.4
PS	72	F	2	1	21.9	4.3	93	1.27	0.58	50.87	1.8	high	7.8	3.1	2.9	32	9.6
PS	68	F	14	1	22.8	3.5		0.94	1.04	100.2	1.6	high	8.5	3.9	0.28		7.1
PS	67	M	13	1	22.4	3.4	119	1.44	0.84	123.3	1.9	high	10.6	5.5	0.29	28	10.8
PS	76	F	28	1	17.5	3.7	139	1.28	0.86	89.55	2.1	high	9.1	3.2	0.42	26	9.9
PS	53	F	6	1	19.3	3.9	126	1.46	1.02	92.26	1.5	high	8.8	5.4			9.3
PS	67	M	6	1	19.3	4.4	165	1.21	1.06		1.5	high	7.6	5.7		17	11.9
PS	70	M	6	1	23.1	3.7		1.89	0.76	95.6	1	high	9.6	5.2		34	11.1
PS	47	F	5	1	24.5	3.5	251	1.5	1.02	105.7	1.6	high	7.5	5.1		22	9.7
PS	49	F	5	1	21.1	3.9	141	1.37	1.05	38.44	1.5	high	9.7	4.4	0.5		9
PS	70	F	4	1	27.4	3.5	158	1.12	0.77	98.52	1.5	high	9.8	6.1			11.7
PS	58	F	4	1	28.4	4.1	128	1.84	1.23	113	1.5	high	7.4	6.5	4.1	20	8
PS	64	M	3	1	22.8	3.6		1.58	0.89	137	1.8	high	8.7	5	3.73		9.9
PS	62	F	3	1	25.9	3.6		1.56	0.62	79.13	1.6	high	8.8	6		23	9.1
PS	78	F	2	1	23.7	4		1.5	0.73	90.77	2.1	high	10.2	5.5		30	11
PS	64	F	2	1	22.8	3.4		1.09	0.96	77.2	2.1	high	8.1	5.8		22	9.4
PS	74	M	2	1	17.3	3.7		1.59	0.95	41.99	1.6	high	9.6	5.7		20	9
PS	65	F	2	1	19.5	3.6	148	1.08	0.95	86.08	2.1	high	8.4	6.2	3.3	36	9.8
PS	77	F	2	1	19.5	3.9	118	1.47	0.84	88.67	1.6	high	9.5	3.6	0.3	20	8
PS	82	F	3	1	20.9	3.8		1.35	0.78	134.4	1.8	high		3.1	0.27	27	11.4
PS	60	M	17	1	25.8	3.4	135	1.7	1.24	69.34	1.5	high	8.7	3.9	0.05	24	11.4
PS	36	F	17	1	25.5	3.7	116	1.33	1.17	132.8	2.1	high	7.8	7.3		26	10.7
PS	65	M	6	1	18.7	4.2	116	1.27	1.04	158.2	1.8	high	9	6.1	0.2	33	11.4
PS	76	M	11	1	21.3	3.4	174	1.5	0.82	89.33	1.6	high	10.2	7.2	0.05	27	9.1
PS	52	M	10	1	21.6	6.3	186	1.36	0.73	97.3	1.6	high	8.8	4.8	0.25	24	9.8
PS	89	M	9	1	17.7	3.2	131	1.46	0.76	96.3	1.9	high	9.4	6.2	0.05	32	11.7
PS	76	F	6	1	22.3	3.9	171	1.45	1.02	146.3	1.6	high	10	4.3	0.19	28	12.9
PS	77	M	4	1	21.3	3.3	142	1.56	0.97	115.5	1.6	high		4.4		25	12.2
PS	76	M	4	1	21.4	3.5	117	1.25	0.95	77.88	2.1	high	9.1	6.4	0.08	28	10.3
PS	75	F	5	1	17.2		116	1.45	1.18	71.67	2.1	high	8.6	5.3	0.18	34	8.6
PS	65	M	17	1	21.5	3.2	148	0.93	0.69	84.2	1	high		6	0.05		9.1
PS	69	M	11	1	20.2	4.2	156	1.17	0.8	130.7	1	high		6.4	1.14		8.3
PS	66	M	2	1	22.4	3.9	158	1.51	1.03	105.5	1.8	high	9.4	6.4	0.34	29	10.3
PS	65	F	11	1	22.7	3.7	141	1.3	0.88	120.7	1	high		6.1	0.05		8.4
PS	65	F	16	1	22.9	4.7	162	0.94	0.95	103.6	1.5	high	8.4	5.4	1.9	48	10.2
PS	62	F	12	1	24	4	181	1.14	0.94	146.1	1	high		4.8	0.05		9
PS	80	F	12	1	20.6	3.9	217	1.15	0.84	131.3	1	high		5.4	0.21		8.3
PS	65	M	6	1	19.3	3.3	200	1.1	0.67	98.03	1.8	high	9.8	5	0	21	10.2
PS	73	F	9	1	19.7	4.1	147	1.77	1.06	100.3	1.8	high	9.9	3.6	0	25	10.3
PS	84	F	4	1	22.4	4	207	1.47	0.65	117.3	1.5	high	9	3.4	0.8	35	9
PS	70	M	25	1	22.3	4	160	1.3	1.17	118.4	2.2	high	9.2	4.4	0.73	22	9.9
PS	42	M	9	1	18.1	4.8	137	0.99	1.1	112.5	1	high		3.6	0.05		9.1
PS	58	F	2	1	22	3.8	138	1.53	0.74	84.33	1.5	high		4.9	0.09	30	9.2
PS	43	M	21	1	22.7	4.4	150	1.34	1	129.3	1.6	high		8			10
PS	68	M	3	1	21.8	3.8	166	1.43	0.94	89.79	1.6	high	8.2	3.7		26	10.3
PS	77	F	10	1	29.3	3.6	182	1.4	0.97	94.65	1.6	high	8.6	4.6	0		10.5
PS	61	M	7	1	23.5	4.2	172	1.36	1.06	117	1.6	high	8.1	8.6	1.7	39	10.4
PS	66	F	21	1	20.3	4.3	165	1.57	0.82	109.9	1.8	high	9	4	0.13	28	9.8
PS	80	F	5	1	18.4	3.9	220	1.39	0.75	114.9	1.6	high	11	3.9		25	13.1
PS	79	F	3	1	19.7	3.9	156	1.54	0.84	81.72	1.6	high	8.4	4.9	0.3	22	10.4
PS	76	F	9	1	17.2	3.9		1.3	1.22	103.1	2.1	high		4	0.65	33	8.4
PS	82	M	5	1	23.1	3.8	100	1.44	0.66	96.63	1.8	high	7.9	3.2		33	9.3
PS	78	M	7	1	19.2	4.4	136	1.76	0.88	107.7	2.1	high	9.3	4.6	0.56	28	10
PS	59	F	13	1	22.6	3.9		1.61	1	119.4	2.1	high	9.1	5.5	0.07	26	12.9

PS	81	F	10	1	22.5	3.2	147	1.44	0.93	122.7	1.5	high	10.9	3.9	0.02	33	13.2
PS	66	F	10	1	17.3	3.9	166	1.77	1.16	127.4	2.1	high	10.1	4.5	0.05	27	11.1
PS	74	M	8	1	21	3.9		1.35	0.89	140	1.5	high	9.6	5.6			9.5
PS	65	M	8	1	25.9	3.6	174	1.58	0.79	102.7	1.8	high	9.1	4.7	0.21	33	10.7
PS	78	M	7	1	19.1	3.7	155	1.13	0.73	104.7	1.8	high	9.4	6.7	0.04	31	12.6
PS	70	F	6	1	22.5	3.2	149	1.77	0.73	89.1	1.3	high		4.5	0.05	21	10.2
PS	37	M	4	1	25.1	3.9	148	1.54	0.97	134.1	1.6	high	7.7	4.7	0.03	30	11.6
PS	61	M	4	1	23.6	3.7	94	1.76	0.95	104.7	2.1	high	9	2.8	2.07	34	10.2
PS	79	F	3	1	19.8	3.7	123	1.37	0.9	145.4	2.1	high			0.05		
PS	74	F	3	1	19.5	4	183	1.16	0.97	111.5	1.8	high	9.1	3.7	0.05	32	13.7
PS	74	M	3	1	17.2	3.5	184	1.45	0.85	46.01	1.6	high	8.4	3.9	0.01	27	9.4
PS	67	M	3	1	21.3	3.7	103	1.85	0.97	60.6	1.8	high	9.3	5.8	0.25	34	11.1
PS	58	M	3	1	19	4.4	117	1.62	0.78	96.5	2.1	high	10.4	5.4	0.09	29	10.1
PS	65	M	2	1	25.4	3.4	133	1.47	0.78	101.8	2.1	high	12.1	6.8	1.33	25	9.3
PS	50	M	2	1	25.5	4.1	99	1.51	0.97	76.8	1.5	high	8.7	2.9	0.77	26	10.4
PS	50	M	2	1	18.8	3.9	142	1.4	1.03	135	1.5	high	8.7	3.1	0.04	32	11.8
PS	49	M	2	1	31.3	3.7		0.97	0.88	118.5	2.1	high	8.4	5.9		50	10.5
PS	64	F	23	1	21.8	3.8	164	1.83	1.05	104.4	1.6	high		8.9			11.1
PS	59	M	3	1	22.4	3.8	126	1.33	0.96	144	1.8	high	8.2	3.5	0.14	30	10.7
PS	59	M	26	1	21.6	3.4		1.17	0.7	116.6	1.6	high	10	5.7			12.1
PS	55	M	12	1	20	3.9	157	1.58	1.06	118.3	2.1	high	9.2	5.3	0.69	23	10.5
PS	34	M	7	1	26.3	4.1	176	1.22	1.1	104.5	1.5	high	8.8	8.1	1.2		9.6
PS	60	M	2	1	20.2	4.1		1.18	0.97	103.9	2.1	high	9.8	4.4	0.04		12.3
PS	62	M	10	1	22.4	3.8	179	1.98	0.85	95	1.6	high	8.3	5.8	0.27	29	11.4
PS	65	M	5	1	22.4	3.4	134	1.27	0.97	108.2	1.8	high	9.4	2.9	0.28	43	10.5
PS	42	F	25	1	17.2	3.8	240	1.53	0.91	108.5	1.6	high		4.2			11.5
PS	56	F	4	1	18.9	4	140	1.19	0.35	159	1.9	high		6.4			10.3
PS	77	F	3	1	18.8	3.9	119	1.17	0.88	74.71	1.9	high		7.3			11
PS	80	F	5	1	28.1	3.7	108	1.5	0.85	54.47	1.6	high	7.2	3.9	0.16	32	10.3
PS	61	F	17	1	17.9	3.9	118	1.64	1.13	112	1	high		1.7	0.17	17	10.4
PS	68	F	3	1	23.2	3.7	195	1.06	0.82	42.11	1.3	high	10.8	4.5	0.05		10
PS	66	F	37	1	22.4	4	90	1.43	1.05	68.37	1.8	high	9	5.5	4.72	34	10.2
PS	66	F	9	1	21.2	3.5	120	1.27	1	126.1	1.6	high		6.7			10.1
PS	76	F	5	1	17.7	3.7	193	1.38	0.81	122.9	1.5	high		4.2	0.21	23	10
PS	72	M	3	1	21.2	3	148	1.5	0.81	92.96	1.8	high	8.5	5.6		35	11.3
PS	87	M	3	1	21.7	3.6	142	1.17	1.09	135.4	1.8	high	8.7	5.3	0.9	24	10.3
PS	60	M	4	1	23.9	3.5	96	1.2	0.9	90.73	1.1	high		2.1	0.5	18	10.5
PS	46	M	7	1	18.9	4.1	204	1.27	0.72	87.78	1.6	high		8.1			12
PS	58	F	8	1	17.5	4.1	106	1.37	0.9	124.5	1.9	high		7.2			9
PS	59	F	7	1	18.5	3.7	171	1.38	0.85	139.6	1.5	high	10.1	3.8			10.2
PS	65	F	2	1	16.2	4	157	1.45	0.85	132	1.9	high		7.4			11.5
PS	76	F	4	1	22.3	3.4	148	1.34	0.71	117.1	1.6	high		6.3			9.7
PS	76	M	7	1	19	3.8	128	1.88	0.72	58.42	1.3	high	9.7	5.3	0.1	25	10.3
PS	73	M	2	1	21	3.5	148	1.05	0.7	100.9	1.3	high	9.9	6.7	0.12	26	11.2
PS	66	F	7	1	20.8	3.3	171	1.75	0.63	62.26	2.1	high	8.9	2.9	1.5	34	8.3
PS	66	M	5	1	28.9	3.9	201	1.76	0.75	148.6	1.6	high	7.8	6.4	0.3	28	10.8
PS	59	F	5	1	24.7	3.8	155	1.43	0.78	69.21	2.1	high	8.3	5.9		14	10.5
PS	57	F	4	1	16.5	4	214	1.48	1.07	127.7	1.3	high	8.4	3.3	0.1		10.3
PS	50	F	3	1	23.5	4	134	1.21	0.89	59.98	1.5	high	9.1	6.2	2.32	32	11.1
PS	71	M	15	1	17.2	3.9	214	1.37	0.9	100.2	1.5	high	8.1	5.5	0.68	27	10.8
PS	67	F	15	1	21.2	3.8	164	1.35	1.09	121.5	1.8	high	9.7	6.5	0.1	28	9.8
PS	48	M	3	1	23.6	3.4		1.63	0.77	100.4	1.6	high	8.6	4.6	2.59	34	9.8
PS	66	F	24	1	19.6	3.6	105	0.94	0.98	116.6	2.5	high	9.3	6.4	0.04	34	11.5
PS	49	M	9	1	25	3.7	186	1.35	0.77	99.36	1.5	high	9.3	4.8	0.03	27	9
PS	39	M	2	1	29.6	3.8		1.35	0.83	103.1	2.1	high	9	7.3	0	29	9.7
PS	73	F	14	1	17.2	3.8		1.07	0.71	121.5	1.6	high	7.8	5.1	1.07	33	11.2
PS	73	F	5	1	26.4	3.3		1.86	0.67	95.95	1.8	high	8.8	6.4	0	28	11.1
PS	54	F	12	1	17.3	3.2	135	1.05	0.77	92.27	2.5	high	9.9	3.2	1.09	21	9.9
PS	45	M	9	1	24.7	3.5	168	1.55	0.72	116.3	1.5	high	9.1	6	0.3	29	11.2

PS	78	M	4	1	20.6	3.1	142	1.44	0.62	110.1	1.8	high	10.3	6.9	0.05	24	11.3
PS	78	M	15	1	21.1	3.1	140	1.36	0.87	145.8	1.5	high	9.4	5.9	0.06	31	9.2
PS	56	F	4	1	24.2	3.3	148	1.62	0.95	123	1.6	high	9.4	4.6	0.63	34	10.1
PS	90	F	15	1	19.1	3.6	156	2.2	0.56	121.4	1.1	high	9	3.4	0.22	31	11.3
PS	52	M	13	1	23.3	4.1	194	1.31	0.95	129.7	1.5	high	9.8	5.8	0.08	30	8.8
PS	59	M	4	1	26.2	3.5	134	1.6	0.65	143.1	1.5	high	11	4.7			10.6
PS	55	M	29	1	21.1	3.7	154	1.42	0.95	100.8	2.1	high	9.2	4	0.09	38	
PS	68	M	14	1	20.2	3.3	107	1.32	0.82	82.87	2.1	high	9.8	5.2	0.41	29	9.4
PS	58	M	4	1	14.4	3.3	114	1.08	0.98	77.11	2	high	10.3	7.7	1.12	28	9.2
PS	70	F	13	1	21.8	3.3	140	1.52	0.76	121.5	1.8	high	9.6	6.7	0.46	27	10.3
PS	40	M	4	1	23.8	4.2		1.78	0.96	58.87	1.3	high	8.8	5.2	0.03	20	11.5
PS	87	F	5	1	21	3.2		1.71	0.65	119.1	1.6	high	8.6	5.7	0	29	11.2
PS	69	F	31	1	22.6	3		1.87	0.75	62.01	1.8	high	9.2	6.1	3.7		11.5
PS	75	F	4	1	25.4	3.5		1.52	0.76	144	2.1	high	9.6	5.8	0	38	9.5
PS	47	F	2	1	30.2	3.3		1.65	0.74	78.25	1.6	high	8.8	4.3	0	30	9.8
PS	71	F	2	1	21.1	3.5		1.89	0.75	81.97	1.6	high	9.5	8.5	0.03	24	10.7
PS	70	F	11	1	21.2	3.6		1.26	0.74	89.78	2.1	high	8.8	5.2	0	24	10.5
PS	61	F	32	1	16.3	3.1	130	1.16	1.12	123.6	2.1	high	10.1	7.6		30	10.8
PS	50	M	27	1	14.4		103	0.96	0.78	64.6	1.6	high		6.7			11.1
PS	42	M	25	1	19.2		133	1.57	0.79	71.03	2.1	high	9	4.1	0.11	22	8.7
PS	59	M	24	1	19.3		172	1.03	0.98	101.9	1.9	high		8.2			10
PS	49	F	23	1	20.7		169	1.2	0.6	102	2.1	high		5	0.07		10.3
PS	35	M	22	1	22.3		156	1.61	1	80.99	1.6	high		4.8	0.06	18	10.2
PS	65	M	19	1	20.3		161	1.39	0.79	92.71	1.6	high		6	0.18	36	10.4
PS	65	M	17	1	22.5		225	1.5	0.55	71.33	1.6	high	9.6	4.9		22	9.6
PS	58	F	14	1	21.7		165	1.23	0.78	83.04	1.5	high	9.5	10.2	0.1		7.1
PS	70	F	11	1	21.3		112	1.49	0.94	136.1	2.1	high	10.5	8.7		28	10.2
PS	63	F	10	1	21.2	3.7	208	1.6	0.76	102.1	1.8	high	10.7	5.3	0.1	28	10.6
PS	57	M	9	1	17.8		140	1.22	1.19	73.03	2.5	high	9.9	4.8	2.29		10.7
PS	68	F	6	1	16.1		133	1.57	0.91	82.87	1.6	high	7.4	4.9		21	9.5
PS	76	M	6	1	22.7		127	1.5	0.8	110.5	2.1	high		5.3	0.18	30	8.7
PS	52	F	4	1	18.6		94	1.24	0.83	87.03	1.8	high	9.1	2.1	2.83	26	8.3
PS	75	M	3	1	23.9			1.15	0.85	134.4	1.9	high	10.6	8.1	0.1		8
PS	60	M	2	1	17.1		131	1.56	0.9	103.4	1.9	high	10.3	4.9	0.05		9.8
PS	50	F	18	1	15.6	3.7	118	1.5	0.91	77.81	1.5	high	9	4.8		40	9.1
PS	58	M	7	1	24.1	3.9	200	1.84	1.07	126.7	1.6	high	10	6.3		22	10.9
PS	80	F	8	1	22.1	3.6	129	1.58	1	18.7	1.8	high	8.7	3	0.02	27	11.4
PS	68	M	10	1	22.7	3.8	128	1.47	0.99	139.1	1.5	high	8.8	5.1	0.04	21	10.4
PS	58	M	10	1	20.4	3.9	153	1.39	0.97	114.4	1.5	high	8.6	3.6	0.29	31	10.8
PS	33	M	7	1	20.4	4.3	164	1.65	0.94	110.1	1.6	high		7.9		26	11.1
PS	67	M	2	1	21.4	3.5		1.56	0.77	18.61	1.3	high	7.8	4.6	4.9	39	9.4
PS	73	F	3	1	20.3	3.6	113	1.28	0.99	117.7	2.1	high		5.9	0.05	25	16.3
PS	55	M	3	1	22.7	4.2	171	1.6	0.87	85.8	2.1	high	8.6	8.6			12
PS	48	M	7	1	19.8	4.2	146	1.66	0.94	144.9	1.6	high	8.1	4.8	0.57	29	9.9
PS	66	F	2	1	25.1	4.4	207	1.63	0.86	57.37	1.5	high		5.5	0.05	20	9.1
PS	51	M	5	1	20.5	3.7	124	1.47	0.81	72.15	2.1	high		5.2	0.05	32	9.9
PS	78	M	9	1	22.5	3.9	105	0.99	0.73	122.3	1.6	high	9.5	4.7	1.7	18	9.3
PS	62	F	4	1	19	4.1		1.16	0.73	105.2	2.1	high	8.7	3.2	0.14	27	10.6
PS	46	M	26	1	21.3	3.6	177	1.78	0.73	80.4	2.1	high	8.7	4.5	0.05	25	11.4
PS	41	M	12	1	22.4	4.3		1.46	0.91	131.3	1.8	high	9.4	4.2	0.05	24	11.1
PS	66	F	7	1	21.8	3.6	130	1.6	1.09	137.4	2.1	high	11.1	6.8	0.02	27	11.4
PS	38	F	6	1	18.1	3.9	104	1.64	0.98	104.8	2	high	8.6	7.5	0.07		8.4
PS	68	F	12	1	21	3.9	149	1.44	1.16	103.1	1	high	9.6	4.9	0.41	16	10.4
PS	60	M	7	1	23.5	4.2	155	1.26	0.97	112.5	1.8	high	10	3.8	0.1	19	10.8
PS	34	F	18	1	17.2	4.1	128	1.81	1.34	88.74	2.1	high	7.6	5.1	0.06		10.5
PS	47	F	6	1	18.8	3.8	112	1.16	0.87	101.4	2.1	high		6.6	0.48	32	12.7
PS	53	M	3	1	21.3	3.9		1.19	0.97	70.88	2.1	high	9.6	5.6		36	9.6
PS	62	F	2	1	20	4.1	154	1.65	0.97	121	1.8	high	10	6.6			10.7
PS	57	M	12	1	22.4	3.8	123	1.41	1.01	118.9	2.1	high	8.1	5.4	0.05	31	10.1

PS	61	M	9	1	19.7	4	194	1.52	0.73	96.96	1.8	high	8.6	3.6	0.02	15	12.1
PS	64	M	5	1	28.8	4.2	209	1.72	0.96	135.6	2.1	high	9.9	4.9	0.11	34	12.3
PS	62	M	7	1	17.4	3.7	130	1.61	0.86	129.5	1.3	high	9.4	5.1	0.7	26	10.6
PS	49	M	4	1	16.2	3.8	178	1.32	0.87	115.8	1.6	high	9.1	3.8	0.34		10.5
PS	61	F	21	1	16	3.3		1.16	1.24	66.74	2.1	high	9.6	7.4	0.33	28	11.3
PS	36	F	4	1	23.5	4.1	158	0.95	0.84	92.77	1.3	high	9.7	7.8		36	8.3
PS	73	M	6	1	19.4	4.1	86	1.28	0.8	91.4	2.1	high	8.4	8.6		25	9.6
PS	56	M	2	1	24.4	4.5	127	1.41	0.91	120.2	2.1	high	9.9	8.5		43	10.9
PS	76	M	10	1	22.3	3.6	132	1.14	0.93	141.3	1.6	high	11.5	7.8	0.46	34	11.4
PS	61	M	7	1	25.9	3.9	194	1.57	0.81	113.2	1.8	high		6.4	0.33	26	8.6
PS	46	F	4	1	21	4.1	183	1.92	1.06	87.11	1.8	high		6.1	0.05	30	8.5
PS	60	F	11	1	16.7	3.8	160	2.14	0.6	104.9	1.8	high	11.4	5.2	0.03	26	10.5
PS	77	F	17	1	22.9	3.8		1.18	0.96	123.6	1.8	high	9.7	5.1	0.71	19	9.5
PS	79	M	6	1	22.2	4.2	138	1.31	0.89	99.16	1.8	high	9.7	4.6	0.33	27	11.6
PS	61	F	6	1	19.8	4	127	1.44	1.07	113.6	1.5	high	9	5	0.02	33	12.2
PS	56	F	3	1	21.6	4.1	80	1.95	1	83.63	1.3	high	10.1	4.7	0.05	23	14.5
PS	71	F	7	1	19.5	4	164	1.38	0.69	139.3	2.1	high	10	5.7	0.78		11.4
PS	68	F	10	1	23.1	3.1	91	1.07	0.57	109.8	1.1	high	9.8	3.3		39	8.3
PS	68	M	13	1	17.9	4	70	1.24	1.01	99.2	2.1	high	7.8	9.8	0.05	60	15.3
PS	74	M	6	1	22.9	3.7	185	1.27	1	144.2	1.8	high	10.2	5	0.35	25	11.9
PS	52	M	9	1	25.6	3.7		1.71	0.79	119	1.6	high	9.1	5.2	0	21	9.8
PS	60	F	7	1	17.5	3.8	113	1.81	1.13	116.1	1.8	high	8.2	4.4	1.2	24	9.7
PS	68	F	4	1	25.3	4	142	1.54	0.8	109.6	2.2	high	9.5	2.9	0.01	23	10.3
PS	83	M	10	1	21.1	3.5		1.2	0.93	115.3	1.3	high		6.5	0.82		15
PS	60	M	4	1	19.8	3.9	113	1.3	0.95	105.8	1.8	high	9.8	7	1.32	29	11.1
PS	79	M	3	1	19.7	3.6	163	1.35	1.23	101.7	1.6	high	11	5.5	0.09		10.8
PS	49	F	6	1	17	3.9	165	1.66	1.05	79.41	1.8	high	9	5.1			11.8
PS	69	F	25	1	22.4	3.5	184	1.03	0.87	122.3	1	high		5.3	0.07		8.8
PS	68	F	25	1	20.2	3.6	126	1.31	0.93	82.23	1.5	high	8.8	2.4	1	26	9.7
PS	59	F	25	1	17	3.6	166	1.04	0.69	60.16	1	high		3	0.23		8.4
PS	60	F	24	1	19.7	3.8	125	1.47	0.87	100.8	1.8	high	6.6	4.2	0.3		9.5
PS	60	F	23	1	17.6	4	124	0.95	0.78	105.9	1.1	high		5.8		18	9.8
PS	79	F	21	1	19.8	3.7	108	1.43	0.57	106.1	1.4	high	9.8	4.7	1.2	27	7.7
PS	53	F	17	1	17.4	4	111	1.3	0.74	99	2.1	high		3.7	0.6	27	9.9
PS	71	M	15	1	20	3.8	94	0.65	0.68	82.28	1.1	high		8.5		22	9.1
PS	66	F	15	1	20	3.8	148	1.69	0.88	123.3	1.4	high	8.9	6.3	0.1	30	11.4
PS	56	M	12	1	20.4	4	175	1.34	0.9	111.7	1.5	high		7.4	0.1	21	11.6
PS	64	M	11	1	19.1	3.9	129	1.25	1	77.49	1.5	high		5.6	0	16	10.6
PS	81	M	10	1	21.1	3.5	205	1.71	0.68	93.55	1.8	high	8.1	4.7	0.06		11.3
PS	58	F	8	1	16.4	4	103	1.24	1.11	90.14	1.5	high	8.3	4.8	0.1	22	10.4
PS	62	M	4	1	18.8	3.8	137	1.46	0.72	68.56	1.4	high	8.1	6		32	10.1
PS	62	F	3	1	19.7	3.8	163	0.85	1.3	126	1.5	high	11.2	2.6	0.13	11	10.9
PS	73	F	2	1	18.3	3.6	131	1.36	1.3	116.3	2.1	high	9.8	4.1	0.48		8.9
PS	60	M	31	1	17.6	3.4	139	1.73	1.08	64.07	1.9	high	10.6	3.8	0.19		11.2
PS	65	M	6	1	25.4	3.8	214	1.5	0.81	59.05	1.6	high	9.7	7.1	0.17		10.1
PS	47	M	3	1	26.4	4.6		1.12	1.01	94.94	1.3	high		4.7			11.2
PS	65	M	15	1	17.9	3.3	161	1.52	0.93	119	1.8	high		6.8		28	11.3
PS	43	M	5	1	23.6	4.3		1.44	1.14	119.8	1.6	high	11	5.2	0	17	10.5
PS	49	M	25	1	18.3	4.1	192	1.64	1.07	117.2	1.3	high	8.7	5.8	0.59		10.4
PS	57	F	24	1	22	4.1	109	1.07	0.72	103.5	2.2	high	6.5	6.1	0.34		9.5
PS	59	F	23	1	19.3	3.6		1.38	0.98	104.2	1.6	high	9.6	8.1			12.5
PS	74	F	21	1	18.5	3.4	148	1.59	1.15	87.08	1.5	high	9.8	6.6	0.41	31	8.9
PS	58	M	21	1	21.1	3.9		1.26	0.75	114.9	1.6	high	10.3	6.1			11.6
PS	61	F	20	1	20.8	3.9	188	1.77	0.92	115	2.1	high	8.6	3.4	0.3	28	10.2
PS	60	F	19	1	19.5	3.5	156	1.15	0.81	121.5	1.8	high	9.6	5	0.2	25	10.1
PS	34	M	16	1	23.3	3.7	226	1.52	1.32	80.46	1.6	high	9.6	6.8	0.04	29	10.4
PS	39	M	15	1	18.4	4.4	128	2.7	1.14	116.5	1.5	high	9.1	5	0.05	54	11.2
PS	56	M	15	1	17.4	4.1	168	1.54	1.24	143.8	1.5	high	9.8	5.6	0.1	20	10.7
PS	79	M	14	1	16.4	3.9	131	1.37	0.92	127	1.8	high	9.3	5.6	0.11	29	11.5

PS	56	M	12	1	18	3.7	122	1.03	0.98	102.8	1.8	high	9.8	5.8	0.37		10
PS	61	F	12	1	21.8	3.8	193	1.77	1.28	124.4	1.5	high	8.9	6.8		27	9.9
PS	52	F	12	1	18.7	3.9	170	1.3	0.94	126.3	2.5	high	9.4	8.8			10.8
PS	67	M	12	1	25.4	3.8	138	1.41	1.02	121.1	1.8	high	9.8	6.2	0.3	0	10.5
PS	69	F	11	1	27.7	3.7	114	1.44	1.23	98.17	1.8	high	9.3	5.2	1.37		10.4
PS	40	F	11	1	19.1	4	170	1.36	0.92	112.1	1.6	high	8.8	5.6	0.12		7.7
PS	58	F	10	1	18.4	3.9	150	1.5	1.01	110.4	1.5	high	9	5.3	0.05	26	9.3
PS	52	F	9	1	31.2	3.3	156	1.69	0.81	79.68	1.5	high	8.9	5.1	6.96	19	10.6
PS	61	F	8	1	17.6	3.7	194	1.67	0.97	112.3	1.5	high	9.2	5.8	0.09	26	11.1
PS	71	F	8	1	20.2	3.9	151	1.17	1.22	114.6	2.1	high	11.6	5.5	0.01		9.6
PS	46	F	8	1	16.3	3.5	100	1.47	1.08	98.8	1.8	high	8	5.6	0.1	31	9.5
PS	52	M	8	1	22.1	3.9	202	1.19	0.99	106.5	1.8	high	8.8	7.2	0.13		10.5
PS	52	M	7	1	17.1	3.4	189	1.16	0.82	103.6	1.5	high	9.7	4.4			11.2
PS	77	M	7	1	22.8	4.1	171	0.91	0.91	92.59	2.1	high	8.6	6.1	0.19		9.8
PS	55	M	6	1	21	4.2	218	1.93	0.59	84.43	2.1	high	9.3	5.7	0.2	27	10.6
PS	47	M	6	1	20	4.2	159	1.35	1.18	137.7	2.1	high	8.8	5.2		20	10.3
PS	55	M	6	1	20	4.3	134	1.15	0.96	82.7	2.1	high	9.3	4.6	0.08		10.5
PS	72	M	6	1	20.5	4	124	2.73	0.96	109.6	2.1	high	8	3.4	0.05	28	9.6
PS	71	F	5	1	23	3.7	129	1.78	1.26	123.5	2.1	high	9.4	6.3	0.05	24	10
PS	55	M	4	1	22.8	4.2	132	1.45	0.83	109.8	2.1	high	8.3	5.9	0.2	30	10.8
PS	53	M	4	1	21.5	4.1	157	1.12	1.14	105.8	1.5	high	9	4.8	0.03	31	12
PS	62	M	4	1	21.5	4	150	1.09	0.89	117	1.3	high	9	5.2			
PS	66	F	4	1	27.6	3.5	200	1.47	1.07	80.57	1.3	high		6.3	0.06	26	12.1
PS	34	M	3	1	22.5	4.2	223	1.35	1.08	111.5	1.8	high	9.5	10.8		32	9.2
PS	67	M	3	1	19.2	3	99	1.43	0.78	87.29	2.1	high	9.5	4.5	0.05	35	8.9
PS	68	M	3	1	24.2	3.8	144	1.1	0.94	88.93	2.1	high	8.4	6.3	0.13		8.6
PS	80	F	3	1	18.7	3.8	157	1.74	0.9	151.6	2.1	high		4	3.85	32	8.7
PS	24	M	3	1	20	4.3	138	1.99	0.98	108.8	2.1	high	8.3	5.2	0.05	25	9.6
PS	50	M	2	1	29.9	3.9	104	1.19	1.03	133.5	1.8	high	9.2	6	0.41	30	7.8
PS	72	M	2	1	21	3.9	123	1.24	0.99	127.2	1.6	high	10.4	5.6	0.24	25	11.4
PS	76	M	2	1	17.4	3.2	96	0.97	1.12	91.19	2.1	high	10.4	5.3	2.34		10.1
PS	53	M	2	1	23.3	3.9	127	1.02	0.86	124	1.8	high	8.9	6.2	3.43		9.1
PS	44	M	2	1	23.8	4.2	118	1.04	0.89	82.63	1.8	high	9.8	5.1	0	19	10.6
PS	54	F	16	1	21.2	4.1	186	1.87	0.91	141.6	2.1	high	8.5	7.7	0.22	33	10.7
PS	33	M	12	1	20.6	4.1		1.18	1.06	99.27	2.1	high	8.7	4		30	8.9
PS	78	M	6	1	25.9	3.7	122	1.26	0.81	128.1	1.5	high	10	4.4	0.32	42	10.9
PS	58	M	24	1	21	3.9	194	1.27	0.95	124.3	1.4	high	10.8	5.4	0.21	30	11.9
PS	68	F	11	1	26.6	3.7	145	1.16	1.23	106.3	1.8	high	9.6	4.1	0.05		10.2
PS	38	F	3	1	25.4	3.7	141	0.97	1.12	121.5	1.8	high	8.7	7		18	10.3
PS	52	M	4	1	19.5	4.1	144	1.15	0.85	93.54	1.8	high	9.3	3.6	0	17	10.2
PS	78	M	12	1	23.8	3.4	150	1.18	0.93	99.7	2	high		5.4	0.39	18	
PS	58	M	16	1	22.2	4.2		1.81	0.88	103.2	1.8	high	9.7	4.5	0.03	29	10.9
PS	45	F	6	1	21.1	3.6	100	1.57	0.55	80.26	2.1	high	8.3	4.8	0.06	44	10
PS	39	M	8	1	20.3	2.7	151	1.26	0.87	103.6	1.8	high	8.6	4.1	0	16	11.9
PS	47	M	4	1	20.9	4.1	164	1.82	0.78	82.41	1.5	high	8.6	6.5	0	23	10.1
PS	66	F	15	1	17.5	3.9	162	1.25	1.02	101.2	2.1	high	8.9	5.1	2.23	22	10.5
PS	58	F	11	1	18	3.9	94	1.28	0.7	85.28	2.1	high	9.1	7.3	0.05	24	9.6
PS	83	M	7	1	18.7	4.1	125	1.37	0.78	126.5	2.1	high	8.7	5.7	0.05	24	11.6
PS	71	F	6	1	20	3.9	149	1.49	0.9	129.3	1.4	high	9.3	4.9	0.05	32	9.8
PS	59	M	5	1	25	4.3	160	0.98	0.88	109	1.5	high	9.4	5.7	0.03	18	11.7
PS	64	M	4	1	27.1	4.1	130	1.76	0.57	90.63	1.5	high	8.9	6.3	0.1	29	9.5
PS	66	F	3	1	19.9	3.9	102	1.06	0.87	100.5	2.1	high	8.3	6.5	1.69		9.1
PS	41	F	4	1	21.5	4.3	108	1.51	0.7	113.7	2.1	high	8.7	2.9	0.7	30	10.2
PS	80	M	5	1	22.2	3.7	209	2.14	0.71	96.43	1.5	high	9.2	5.4	0.21	20	10.1
PS	74	F	2	1	18.7	3.3	113	1.04	0.69	38.25	1.8	high	9.7	6.9	0.05	29	11.3
PS	43	M	29	1	24.6	3.9	97	1.34	1.3	106.5	1.6	high	8.5	6.4	0.05		10
PS	61	M	11	1	21.8	3.6	152	1.81	0.83	105.1	1.6	high	10	5.2	0.08		10.7
PS	66	M	15	1	21.9	3.6	166	1.38	1.02	120.1	1.8	high	8.2	3.9	0.35	25	10.9
PS	60	M	7	1	25	4.1	185	2.04	0.95	102.7	1.3	high	9	2.9	0.05		9.9

PS	73	M	6	1	23.4	3.8	155	0.86	0.9	101.1	1.3	high	8.6	3.5	1.85		10.8
PS	47	M	4	1	24.6	3.7	128	1.07	0.93	24.4	1.5	high	9.8	7.2	0.34	27	10.4
PS	67	F	4	1	21.5	3.7	140	1.74	0.76	80.79	1.8	high	9.5	6.4	0.12	39	12
PS	62	M	3	1	23.2	3.7	218	2.1	0.9	103.2	1.5	high	8.7	3.6	0.16	31	10.7
PS	85	M	10	1	17	3.9	74	0.97	1.03	109.8	1.3	high	9.5	4.4	1	24	8.1
PS	71	F	14	1	20.1	3.5	91	1.53	0.75	108.9	2.1	high	9.8	4.3	0.07	27	
PS	83	F	6	1	20.4	3.6	77	1.42	1.09	112.2	2.1	high	10.6	6.5	0.03	28	11
PS	71	F	9	1	18.8	3.9	139	1.57	1.41	111.8	2.1	high	8.5	5.5	0.58	31	12.9
PS	63	F	14	1	22.7	3.7	173	2.61	0.78	99.52	1.5	high	7.7	3.9	0.3		12.1
PS	60	M	14	1	22.6	3.8	179	1.5	0.92	152.8	1.6	high	9.3	5.2	0.05		9.7
PS	60	F	18	1	22.1	3.9	184	1.07	0.8	119.4	2.2	high		9	0.3	33	9.8
PS	45	F	11	1	18.1	3.9	194	0.87	1.26	110.9	0.6	high	9.8	3.6		18	9.8
PS	60	F	10	1	23.2	4.1	142	0.83	0.91	85.99	1.9	high	8.7	5.3	0.07		11
PS	56	F	10	1	21.5	4	176	1.57	0.9	101.2	1.6	high	10.6	6.2	0		9.2
PS	52	F	9	1	21.2	3.8	213	0.93	1.3	77.3	2.1	high	9.4	6.6	0.12	26	11.4
PS	68	M	8	1	20.2	4	155	2.05	0.93	106.7	1.5	high	8.9	5.2	0		9.5
PS	67	M	7	1	21.3	3.5	161	1.09	1.15	129.2	1.8	high	9.2	4.9	0.08	27	10.3
PS	56	M	6	1	18.1	3.9	164	1.03	0.74	73.73	2.1	high	10.2	5.9	0		10.1
PS	62	M	5	1	23.6	3.4	168	1.48	0.93	104.7	1.3	high		3.4		25	9.7
PS	86	F	5	1	18.7	3.7		1.85	0.65	113.4	2.1	high	8.5	4.1	0.3	22	9.7
PS	70	F	3	1	19.8	3.8	125	1.47	0.9	115.1	1.5	high	10	5.4	0		10.2
PS	61	M	2	1	25.4	4.3	185	1.65	0.99	109.8	1.5	high	8.3	5.2		26	10
PS	27	M	2	1	18	4.5	177	1.47	0.59	78.28	1	high	9.7	5.2	0.02	31	11.8
PS	79	M	2	1	24.1	3	212	1.82	0.44	50.31	2.1	high	8	6.4		23	13.8
PS	76	M	2	1	24.6	3.6	139	1.59	0.77	73.41	1.5	high	9	6.8		26	9.6
PS	47	M	30	1	19.9	3.7	192	1.54	0.68	97.28	1.5	high	8.3	6.1	0.1	26	11.1
PS	61	M	29	1	24.8	4	186	1.66	0.91	118.6	1.6	high	9.6	7.8	0.09	30	10.7
PS	59	M	25	1	22	4.4	151	1.33	1.14	93.98	1.8	high	8.6	5.6	0.1		11.7
PS	57	M	25	1	19.6	4.2	192	1.96	0.67	95.4	1.5	high	9.1	4.5	0.06	42	11.1
PS	59	M	23	1	18.7	4.2	106	1.17	0.96	126.6	1.8	high	8	3.9	0.22	26	9.4
PS	56	M	19	1	27.5	3.5	247	1.32	0.8	99.6	1.4	high	9	5.8	0.5	29	9.3
PS	54	M	18	1	16.2	3.9	149	1.47	0.96	92.2	1.3	high	6.1	7.5	0.17	28	12.1
PS	69	M	18	1	24.1	3.3	128	1.38	1.07	123.8	1.5	high	7.9	5.6	0.13	38	13.9
PS	63	M	17	1	23.7	3.6	116	0.87	0.78	114.8	2.1	high	9.2	5.8	0.04	22	11
PS	49	M	16	1	20.5	4	143	1.33	0.82	113.5	1.8	high	9.5	3.2	0.02	25	12.2
PS	75	M	16	1	22.1	3.4	120	1.37	0.84	102.3	2.1	high	7.5	5.1	0.27	31	10.7
PS	66	M	14	1	21.1	3.5	129	1.25	0.72	118.8	1.8	high	8.4	2.8	0.31	27	12.3
PS	62	F	13	1	18.6	4.1	126	1.56	0.97	134.7	2.1	high		4	0.12	29	12.2
PS	57	M	13	1	21.2	3.9	179	1.47	0.9	105.9	2.5	high	8	3.9		25	12.3
PS	57	M	12	1	21.7	4.1	194	1.33	0.88	136.2	1.5	high	8.5	3.1	0.02	31	10.8
PS	63	M	11	1	16.8	3.7	115	1.5	0.96	77.4	1.5	high	9.6	3.9	0.09		11.1
PS	68	M	11	1	19.4	4	132	1.37	0.78	137.1	1.8	high	8.7	4.5	0.09		8.9
PS	43	F	10	1	29.5	4	177	2.19	0.79	92.28	1.8	high	9.7	6.5	0.05		7.1
PS	48	M	10	1	26.4	4.3	113	1.51	0.88	137.9	1.5	high	9	3.6	0.95	27	11.4
PS	61	F	8	1	15.3	3.5	140	1.5	0.93	94.15	1.5	high	8.4	5	0.01	27	10.3
PS	72	M	8	1	21	3.7	122	1.28	1.05	111.2	1.8	high	8.4	4.5	0.17		10.5
PS	60	M	8	1	25.4	3.6	128	1.81	0.78	116.1	1.1	high	9.8	4.1	0.2	20	9
PS	45	M	22	1	24.1	4.1	146	1.11	0.87	118.3	1.8	high	9.4	3.8		31	9.7
PS	61	M	8	1	25.7	4	161	1.16	0.88	114.3	2.1	high	10.1	6.8	0.15		11.7
PS	48	F	7	1	23.2	4.2	170	1.13	0.88	80.63	2.1	high	9.6	5.2	0.05		10.1
PS	51	F	7	1	19	4.1	189	2.05	0.9	94.93	1.5	high		4.2	0.13	23	12.1
PS	60	F	6	1	21.2	4.2	145	1.58	0.96	130.2	1.8	high	10.4	5.6	0.3	18	11.3
PS	83	F	6	1	26.3	3.5	109	1.31	0.68	113.8	1.6	high	7.5	4.1	0.21	29	10.2
PS	45	M	6	1	17.7	4.2	98	1.29	0.96	108.4	2.1	high	10.2	3.1	0.25	26	9.8
PS	54	M	5	1	17.5	3.9		1.37	1.11	98.47	1.8	high	9.5	4.5			9.7
PS	42	F	5	1	31.3	3.8	127	1.32	0.46	16.39	2.1	high	9.7	7.1	0.05	23	12
PS	67	M	5	1	19.5	4.1	123	1.4	0.85	120.5	2.1	high	9.4	3.1	0.06		9.9
PS	68	M	4	1	21.4	3.8	234	1.26	1	125.5	2.1	high		5.4	0.16	18	10.5
PS	63	M	4	1	18	3.8	151	1.41	0.83	98.35	1.8	high	8.7	4.2		28	9.5

PS	73	M	4	1	23.1	3.7	147	1.32	0.72	98.96	2	high	9.7	4.7		29	11.4
PS	62	M	4	1	24.8	3.9	118	1.41	0.75	78.98	1.8	high	10.5	7.5		28	10.9
PS	59	F	3	1	23.1	3.8	120	1.81	0.94	88.41	1.1	high	8.3	7.1	0.02	30	10.2
PS	79	F	2	1	18.4	2.7	207	1.93	0.74	130.9	1.3	high	9.3	3.7	0.4	22	10.8
PS	61	F	2	1	18.9	4.4	95	1.31	0.77	105.7	2.1	high		3.7		31	10.7
PS	32	M	2	1	26.8	4.4	202	0.94	0.88	102.5	1.8	high	9.4	3.7	0.13		11
PS	57	M	2	1	19.4	4.1	67	1.3	0.96	111.5	1.5	high	8.3	4.2		24	10.6
PS	82	F	4	1	21.8	3.6	177	1.42	0.98	107.5	2.1	high		6.4	4.75	19	11.7
PS	60	F	2	1	21.6	3.8	224	1.82	0.67	87.59	1.5	high	8.3	3.9	0	25	10.2
PS	72	F	6	1	23	3.8		1.49	1.06	134.1	1	high	9.1	3.7		23	10.5
PS	70	M	2	1	23.1	3.8	164	1.35	0.54	11.12	1.6	high	10	6.5		28	10.9
PS	78	F	2	1	16.4	3.4	134	1.56	0.57	59.41	1.3	high	8.5	4.1	1		7.1
PS	69	F	16	1	17.1	3.8		1.57	0.97	95.55	2.1	high	9.5	6.8			10.7
PS	71	F	2	1	17.4	3.6		1.75	1.03	102	2.1	high	9.9	6.7		25	
PS	67	F	13	1	25	3.5	172	1.61	1.14	71.01	1.5	high	9.2	4.5	0.43	15	10.6
PS	65	M	6	1	20.5	3.8	213	1.37	0.87	98.92	2.1	high	10.9	7	0.06		12.3
PS	62	M	22	1	22.5	3.6		1.57	0.72	106.4	1.2	high	10.1	4.6			9.9
PS	62	F	24	1	19.1	4	147	1.45	1.37	117.9	1.5	high	8.6	5.6	1.2	31	9.8
PS	65	M	21	1	19.5	3.9	105	0.81	0.98	116.1	1	high	8.3	4.3	0.15	29	7.8
PS	44	M	23	1	20.2	4.2	171	1.43	0.98	118.1	1.9	high	9.4	5.2	0.1	21	9.5
PS	57	F	19	1	17.9	3.9		1.48	1.12	131	1.6	high	9.4	5.2	0.3	24	11.3
PS	62	F	19	1	17.6	4		1.75	0.88	130.2	1.4	high		7.7	0.2	33	11.8
PS	66	F	18	1	18.5	4	148	2.68	0.92	112.3	1.1	high	9.9	3.5		21	10
PS	58	F	15	1	21.3	3.9	139	1.48	1.22	140.9	1.4	high	9.2	5	0.27	27	8
PS	53	M	16	1	17.5	4.2	245	1.11	0.78	116	1.3	high	8.5	3.3	0.17	10	10.4
PS	75	M	14	1	23.5	3.6	145	1.56	0.94	156.3	1.5	high	9.9	5.8	0.11	32	10.2
PS	70	F	14	1	15.7	4.1	161	1.5	0.81	111.5	1.4	high	9.9	4.8	0.06	27	11.4
PS	34	M	13	1	28.9	4	115	1.14	0.68	117.2	1.3	high	8.9	5.1	0.22	18	10.5
PS	59	M	12	1	19.9	3.5	209	1.33	0.95	125.3	1.8	high	9	4	0.03	28	10.8
PS	74	M	11	1	19.8	3.2		1.78	0.91	111.6	1.3	high	8.9	3.9	0.06	30	11.6
PS	60	F	29	1	22.1	3	110	0.93	0.71	70.44	2.1	high	8.7	6.2	0.17	49	10.5
PS	65	F	10	1	15.5	3.2		1.51	1.14	69.26	1.5	high	9.4	6.2	0.05	20	10.3
PS	73	M	10	1	25.2	3.7	192	1.26	0.94	154.7	1.8	high	9.7	4.8		33	9.8
PS	48	M	9	1	24.6	4.1	229	1.81	1.1	134.2	1.8	high	8.6	4	0.77		11.3
PS	78	M	8	1	19.3	4		1.21	0.82	119.5	1.5	high	8.1	3.9	0.06	23	11.2
PS	58	M	8	1	24.1	4		1.42	0.79	100.5	2.1	high	10.6	5.7			9.4
PS	67	M	8	1	20.4	3.5	131		0.76	111	2.1	high		9.4	0	21	11.9
PS	39	M	8	1	25.8	3.9	143	1.63	1.15	107.9	1.6	high	9.4	3.2	0.05		11.1
PS	69	M	8	1	19.7	3.4	139	1.2	0.92	113.2	2.1	high	8.6	5.2		27	10.4
PS	36	M	8	1	22.8	4.3	93	1.48	0.68	89.85	1	high	9.9	5.1	0.06	30	10.3
PS	77	M	8	1	18.5	3.5	156	1.57	0.92	106.4	2.1	high	10.1	4.8		27	9.7
PS	55	F	13	1	22.4	4.3	138	1.13	0.98	126.2	2.1	high	8.7	5.9	0.06	20	12.7
PS	75	M	5	1	21.9	4	84	1.64	0.94	143.7	2.1	high	9.4	5.3		39	9.7
PS	50	M	5	1	26.1	3.6	202	1.47	0.78	126.6	1.6	high	7.9	6.3	0.22		9.6
PS	69	F	5	1	23.4	3.9	193	1.57	0.66	117.5	2.1	high	8.8	6.4		29	11.7
PS	75	F	4	1	21.6	3.4	127	1.44	0.53	74.13	2.1	high	8.5	5.9			11.4
PS	44	M	4	1	19.4	3.7	150	1.4	1.01	77.76	2.1	high	10.3	5.7	0.83	24	11.5
PS	67	F	12	1	22.2	3.4	111	1.09	0.96	149.6	2.1	high	9.5	4.7	0.58	19	10.3
PS	79	F	4	1	26.1	3.7	114	1.31	0.7	87.94	1.1	high		6.9	0.2	53	11
PS	71	F	3	1	21	3.5	87	1.16	0.72	87.89	1.1	high		14	0.1	28	10.2
PS	65	M	13	1	19.9	3.8	128	1.52	0.79	127.2	1.4	high	9.1	6.3	0.03	29	12.7
PS	69	F	6	1	26.3	3.9	196	2.01	1.13	92.37	1.8	high	9.1	4.7		28	
PS	53	M	19	1	22.3	4.1	126	1.38	0.98	139	1.9	high	8.9	2.8	1.54		10.6
PS	55	F	2	1	20	4	140	2.55	0.76	71.26	1.6	high	8.3	4	0.21	38	8
PS	40	M	12	1	19.1	4		1.37	0.98	124.3	1.8	high	8.9	4.9	0.05	34	10.9
PS	76	M	11	1	19.6	3.8	270	1.74	1	112.5	2.1	high	9	6.3		30	12
PS	54	M	10	1	28.7	3.6	133	2.14	0.87	120.6	2.1	high	8.7	7.2		28	9.7
PS	44	M	4	1	33.2	4.6	150	1.29	1.27	101.4	1.3	high	9	4.7	0		10.4
PS	71	M	14	1	20.4	4.1	118	1.03	0.75	88.47	1.5	high		4.2	0.78	34	8.9

PS	66	M	5	1	21.5	4.1	108	2.14	0.85	126	2.1	high	10	4.6		25	11.2
PS	62	F	11	1	21	4.2	125	2.43	1.2	92.36	1.5	high	9.5	5.6		28	10.3
PS	52	M	14	1	19.7	4.3	124	1.43	1.15	114.3	1.5	high	9.6	5.8	3.81	22	10
PS	53	F	9	1	16.1	4		1.5	1.18	112.9	1.8	high	9.1	6		24	11.5
PS	49	F	6	1	22.7	3.9	181	1.55	0.89	118.7	1	high	11	5.9	0.09	23	10.6
PS	72	M	3	1	22.5	4.1	204	1.57	1.09	139.1	1	high	8.9	4.4		26	9.3
PS	60	M	3	1	24.7	4	138	1.68	0.66	60.84	2.1	high	9.8	5.5	0.05	43	10.7
PS	54	F	11	1	23	3.8	143	1.44	0.98	93.29	1.5	high	8.9	5.6	2.43	25	9.8
PS	73	F	11	1	21	4.2	130	1.27	0.9	147.3	1.8	high	10	4.1	0.2	19	10.2
PS	50	M	4	1	20.5	4.2	139	1.26	0.63	116.6	1.8	high		5.4	0.11		11.3
PS	59	M	9	1	21.2	4.3	151	1.74	0.81	121.2	1.8	high		3.8	0.12	29	9.8
PS	67	F	2	1	21.4	3.9	157	1.02	1.08	80.75	2.1	high	9.1	8.2	0.2		11
PS	63	F	10	1	19.8	3.4	162	1.57	0.9	129.4	1	high	9	4.9	0.4	29	11.3
PS	60	F	10	1	25.7	3.8	98	1.18	1.21	123.8	2.1	high	7.4	4.9	3		8.5
PS	43	M	8	1	25.5	4.2		1.54	0.95	108.4	1	high	9.3	3.5	2.8		7.8
PS	60	M	5	1	20.9	4.3	167	1.27	1.14	98.18	1.8	high	8.8	6.2	0.03	22	10.6
PS	66	M	2	1	21.4	4.1	160	1.3	0.83	96.08	1.8	high		5.1	0.05	29	10.2
PS	47	M	11	1	28.2	4.1	137	1.65	0.75	72.65	1.5	high	7.6	4.7	0.12	31	11.5
PS	63	F	4	1	15.1	3.7	190	1.56	1.04	53.25	2.1	high	8.8	4.4	0.2	29	10.9
PS	61	F	7	1	17.8	3.6	137	1.26	0.86	96.96	1	high		6.1	0.06		8.9
PS	61	M	7	1	20	4.1	156	1.55	0.94	85.14	2.1	high	8.3	5.7	0.24		9.3
PS	66	F	6	1	34.1	3.8		1.77	0.9	119.6	1.8	high		3.9			10
PS	75	F	7	1	16.9	3.9	124	1.1	0.88	92.57	1.8	high		7.5	1.03	35	10.3
PS	56	F	15	1	25	4	138	1.32	1.19	136.5	2.1	high	8.5	4.9	0.26	30	
PS	71	M	4	1	18.6	3.9	138	1.29	0.78	108.4	2.1	high	9	6.2	0.08	46	8.9
PS	63	M	3	1	21.8	3	139	1.26	0.81	81.33	1.4	high		4.8	1.8	38	12.7
PS	38	F	5	1	22.7	3.8	177	1.35	1.02	129.2	2.1	high	8.5	8.8	0.02	22	10
PS	45	M	4	1	19.4	3.7		1.36	1.17	119.4	1.8	high	9.9	2.8			8.9
PS	81	M	3	1	17.8	3.5	120	1.77	0.9	99.25	1.4	high	8.5	4.3	0.2	44	7.2
PS	62	M	2	1	25.2	4.1	163	1.73	1.12	108.2	1.8	high	8.4	7.5	0.02	19	13.2
PS	74	F	5	1	18.5	4.2	211	1.55	0.73	51.94	1.6	high	9.8	4.7	0.05		10.3
PS	70	F	2	1	25.4	4.4	92	1.23	0.75	80.04	1.6	high	8.5	3.8	0.08		9.1
PS	52	F	15	1	17.7	3.8	121	1.92	0.99	117.6	1.5	high	8.4	7.4	0.4	27	11
PS	58	F	13	1	18.4	4	153	1.69	1.08	101.5	1.9	high	9.9	4	0.52		10
PS	71	F	10	1	18.3	3.7	204	2.07	1.02	126.4	1.6	high	9.6	4.2	0.05		9.9
PS	67	M	9	1	21.2	3.9	104	1.88	0.85	136	1.6	high	8.8	6.1	0.05		10.6
PS	63	F	8	1	16	3.9	132	1.42	0.96	87.7	2.1	high	9.2	7			9
PS	95	F	5	1	17.6	3.3	170	1.19	0.85	95.23	1.6	high	9.2	4.2	0.05		9.8
PS	68	M	3	1	20.4	4.1	178	1.85	0.95	104	1.6	high	9.3	4.2	0.05		9.8
PS	72	M	3	1	20.3	3.5	125	2.21	0.79	91.21	1.8	high		4.4	0.47	23	9.3
PS	77	M	3	1	23.9	3.3	141	1.08	0.95	142.5	1.6	high	9.4	4.3	0.12		9.9
PS	67	F	7	1	19.5	3.4	153	1.88	1.01	77.65	1.3	high	8.6	4.4	0.31		9.7
PS	49	F	23	1	23.3	3.8	106	1.41	1.09	114.5	1.8	high	10.1	4.9	0.1	21	9.5
PS	61	F	21	1	18.1	4.1	114	1.54	1.03	67.76	1.5	high	10.8	8.1	0.1	19	10.7
PS	48	M	20	1	22.2	3.7	126	1.65	1.11	129.9	1.3	high	9.7	2.8	0.1	19	10.2
PS	56	F	18	1	16.7	4.2	162	1.4	1.11	137.7	1.5	high	11	7.5		34	12.3
PS	77	M	17	1	18.8	4	176	1.53	1	97.07	1.6	high	9.9	3.1	0.05		11.2
PS	62	M	15	1	20.1	3.6	176	2.2	0.99	100.9	1.6	high	9.4	5.4	0.05	28	10.3
PS	52	M	13	1	26.9	3.7	107	1.24	1.01	125.1	2.1	high	8.9	3.9	0.12	25	10.5
PS	59	F	12	1	26.9	4.1	171	2	1.19	70.11	1.6	high	8.9	5.5	0.05	27	11
PS	69	M	3	1	25.6	4.4	152	1.95	0.73	82.95	1.8	high	10.5	5.1	0.07	34	10
PS	58	M	3	1	21.6	3.8	112	1.63	0.83	86.85	1.5	high	9.6	7.2			9.9
PS	39	M	3	1	24.5	4.5	188	1.48	1.08	103.7	1.3	high	9.2	5.1	0.1	18	12.6
PS	64	M	27	1	19.8	3.8	137	1.4	1.02	114	1.5	high	8.8	4.2	0.1	22	11.7
PS	51	M	6	1	21.1	3.9	102	0.91	1.03	92.83	1.5	high	8.7	4		27	9.3
PS	68	F	16	1	19.5	4.1	143	1.25	1.01	139.2	1.6	high	9.4	5.3		27	12.6
PS	49	M	13	1	18.7	3.9	218	1.31	0.81	120.3	1.8	high	9.4	6.5		31	8.8
PS	59	M	11	1	17.1	4.1	212	1.69	1.08	130.5	1.4	high	9.5	6.2	0.1	27	12
PS	27	M	10	1	24.9	3.1	150	1.72	1.13	126.7	1.4	high	9.5	3.2	0.1	31	10

PS	58	M	7	1	20.4	3.8	125	1.54	0.78	115.8	2.2	high	9.6	4.5	0.6	30	11.1
PS	66	M	7	1	24.5	4.1	112	1.08	0.86	81.88	1.8	high	9.3	4.7	0.11		9.4
PS	59	M	6	1	19.1	4	213	1.5	0.91	126.8	1.4	high	9.5	5.1	0.2	16	11.4
PS	56	F	6	1	22.4	4.1	235	1.77	1.07	104.4	1	high	8.9	5.2	0.1	25	9.8
PS	60	M	5	1	22.3	4.5	163	1.38	0.91	127.7	1.8	high	7.2	6.8	3.03		10.2
PS	78	M	5	1	21.5	3.9	66	0.91	0.98	127.1	1.3	high	9.8	1.7	8.4		9.2
PS	64	F	5	1	19.2	3.6	202	1.76	0.93	97.86	1.6	high	10	3.5		25	10.8
PS	70	M	4	1	24.2	3.9	111	1.79	0.94	110.9	1.5	high	8.7	5.8	0.2	25	12.1
PS	46	F	4	1	17.7	3.8	203	1.92	0.99	105.3	1.6	high	10.8	8	0.13	28	11.9
PS	76	M	3	1	25	4	153	1.35	0.74	89.57	1.8	high	9.1	5.3	0.1	27	11.8
PS	68	M	3	1	24.7	4	111	1.46	0.97	124.7	2.1	high	8.5	3.5	0.3	31	10.3
PS	75	F	3	1	16.1	3.9	116	1.42	0.96	128.8	1.3	high	9.6	5.1	2.57	30	8.3
PS	71	M	3	1	25.9	4.1	147	1.67	0.8	70.07	1.6	high	10.6	6.2	0.01	21	10.5
PS	42	M	3	1	29.7	4	180	1.04	1	142.4	1.5	high	8	7.8	0	20	8.9
PS	65	M	2	1	20.1	3.8	109	0.81	0.88	68.8	1.5	high	11.9	5.7	0.25	25	10.2
PS	65	F	2	1	28.1	3.8	148	1.21	0.76	71.3	1.8	high	10.5	6	0.1	16	11.5
PS	65	M	6	1	22.1	4.2	180	1.44	1.01	119	1.8	high	9	4.5	0.12		11
PS	69	F	9	1	18.4	4.3		1.34	0.83	108	1.5	high	8.9	4.5	0.06	23	10.8
PS	63	M	31	1	18.1	3.3	204	1.38	0.98	95.66	2.1	high	9.4	6.8	0.03	27	12.4
PS	80	F	32	1	17.7	4	163	1.14	0.7	95.69	1.1	high	9.4	4.1	0.4		9.6
PS	67	M	30	1	22.8	3.5	109	1.31	1.07	129.4	2.5	high	8.2	4.1	0.02	55	10.4
PS	58	F	30	1	17.3	3.8	176	1.44	0.89	103.2	1.8	high	8.5	5.7	0.07	33	10.7
PS	50	F	29	1	23	3.6	167	1.58	0.95	135.1	1.8	high	9.2	4.9		32	10.1
PS	73	F	29	1	17.8	3.6	123	1.3	0.99	79.34	2.1	high	9.4	6.2		37	9.6
PS	74	M	28	1	29.1	3.8	147	1.52	0.85	106.8	1.8	high	9.3	4.6		29	9.6
PS	51	M	26	1	22.3	2.7	119	2.1	0.88	118	2.1	high	9	5.2	0.2	25	10.6
PS	66	F	22	1	24.5	3.9	115	1.39	0.99	131.2	2.5	high	8.4	7.1	0.01	25	10
PS	60	M	22	1	20.2	3.6		1.7	0.92	102.9	2.1	high	10.1	7		25	11.4
PS	82	M	21	1	20.1	3.6	203	1.28	0.8	120.7	1.5	high	8	6.2		28	9.7
PS	62	F	19	1	21.5	3.8	102	1.3	0.83	115.7	1.5	high	9.4	4.6	0.01		9.2
PS	65	M	19	1	23.9	4.2	101	1.3	1.42	244.5	2.1	high	8.4	5.5	0.01	32	10.3
PS	58	M	17	1	25.1	4.1	138	2.91	0.87	112.3	1.5	high	9.6	5.1		27	9.9
PS	72	M	16	1	20.9	3.8	146	2.46	0.79	89.6	2.1	high	9.2	3.7		22	11.5
PS	65	F	16	1	17.7	3.6	165	1.09	0.78	98.84	1.8	high		6	0.3	27	9.9
PS	51	M	15	1	18.4	3.6	187	1.02	0.86	124.1	2.1	high	8.9	4.8	5.74	36	8.6
PS	59	F	15	1	21.3	3.9	144	1.77	0.85	88.55	1.6	high	8.5	5.5		26	
PS	72	F	15	1	20.4	3.7	129	1.64	0.83	193.4	1.8	high	8.4	5.3	0.05	30	10.7
PS	74	F	14	1	20.2	2.9	155	1.03	0.48	71.51	1.3	high	8.3	6	0.05	25	9.2
PS	61	F	14	1	18.7	5		2.03	1.03	71.42	2.5	high					
PS	80	F	13	1	20.5	3.7	155	1.47	0.77	94.82	1.5	high	8.9	6.8		28	11.1
PS	66	F	12	1	27.9	3.6	158	1.28	1	151.7	2.1	high	9.6	6.4	0.1	24	10.4
PS	50	M	11	1	24.5	3.9	131	1.78	0.87	103.6	1.3	high	8.6	3.9	0.04	30	10.7
PS	64	F	11	1	20.7	3.6	181	2.28	1	134.9	2.1	high	9.2	5.2		26	10.5
PS	61	F	11	1	17.9	4.1		1.03	0.9	111.9	1.5	high	9.7	4.2		17	9.7
PS	39	F	11	1	19.1	4.1	156	1.63	1.1	99.81	1.5	high	9	5.3	0.03	30	11.1
PS	83	M	9	1	22.3	3.7	137	1.14	0.73	118.6	1.5	high	8.7	5		20	10.7
PS	65	M	8	1	26.1	3.9	118	1.35	0.99	132.9	2.1	high		3.7			10.1
PS	55	M	8	1	22.1	3.7	202	1.52	0.96	115.7	2.1	high		3.8	0.28	22	9.3
PS	63	M	7	1	21.7	4	131	1.58	0.78	78.43	1.3	high	8.4	5.1	0.06	15	9.2
PS	46	F	6	1	26.3	3.3	186	1.32	0.83	116.9	2.1	high	8.9	4.4	0.07	32	12
PS	84	M	5	1	22.1	4.1	161	1.76	0.72	119.6	2.1	high	9	3.2	0.1		11.5
PS	74	M	5	1	18.2	3.5	141	2	0.77	111.2	1.8	high		5.1			10.4
PS	72	F	5	1	23.6	3.4	151	1.14	0.74	106.7	1.6	high		3.8	0.05	26	9.6
PS	66	M	5	1	20.1	3.5	188	1.37	0.69	78.87	2.1	high	8.8	6.5	1.94	30	10.7
PS	65	M	4	1	22.7	3.9	144	1.52	0.81	98.92	1.6	high	8.5	3.9		24	10.2
PS	75	F	4	1	22.6	4	202	1.45	0.91	83.42	1.8	high	8.9	8.9	4.65	22	10.5
PS	65	M	4	1	29.2	3.9	215	1.97	0.62	68.22	1.3	high		3.6	0.5	24	10.2
PS	33	M	4	1	24.5	3.8	107	1.94	1.07	99.67	1.5	high	8.3	3.9		19	12.9
PS	78	M	4	1	22.4	3.8	177	1.47	0.89	99.96	1.3	high		6.2	0.3	22	10.7

PS	82	M	3	1	17.9	3.6		1.07	0.75	95	1.8	high	9.4	4.9		18	12.6
PS	60	M	3	1	21.6	3.2	107	1.32	0.86	74.63	1.8	high	8.3	4.1	1.28	33	8.9
PS	70	M	3	1	22.2	3.8	120	1.24	0.76	63.77	1.5	high		3.8	0.3	21	11.5
PS	81	M	3	1	25.4	3.4	131	0.93	0.71	121.7	1.8	high	11.3	2	0.78	33	9.5
PS	39	M	2	1	21.1	3.7	152	1.6	0.82	109.8	1.5	high	8.9	4.3	1.49	38	10.2
PS	40	M	2	1	19.8	4.2	110	1.59	0.95	120.3	2.1	high	9.5	3.5	0.36	40	9.2
PS	65	M	2	1	21.8	3.4	152	1.45	0.8	99.75	1.3	high	12.1	6.5		37	11
PS	81	M	2	1	21.9	4	175	1.85	0.99	82.76	1.3	high		2.1	0.1	27	8.8
PS	77	M	2	1	22.3	3.6	210	1.35	0.6	86.86	1.3	high		5.6	0.1	22	9.4
PS	34	F	2	1	16.6	4.2	168	1.65	0.7	69.77	1.3	high		5.5	0.1	29	9.8
PS	53	M	2	1	23	4.1	181	1.72	0.75	109.4	1.3	high		5.3	0.8	22	9.9
PS	62	F	6	1	20.4	3.1	199	1.33	0.83	149.2	1.8	high	7.8	4.7	0.23	52	10.3
PS	59	F	14	1	23.5	3.7	123	1.37	1.04	119.5	2.1	high	8.4	4.3	0.3	22	11.7
PS	69	M	11	1	19.3	3.9	148	1.94	0.95	101.7	1.5	high	9.9	4.8			11.5
PS	55	F	11	1	21.4	4	113	1.08	1.14	102.1	2.1	high	8.1	5.4	0.35		
PS	84	F	8	1	15.1	3.5	107	1.11	1.01	81.8	1.4	high	9.7	5.4	0.47	31	9.6
PS	64	F	6	1	19.8	3.8	177	1.89	0.79	103.5	1	high	9	5	0.1	19	10.4
PS	74	F	4	1	20.1	3.3	81	1.61	0.76	68.75	1.5	high	7.7	6.8	0.1	23	7.1
PS	61	M	3	1	22.4	3.3	207	1.25	0.75	104.4	2.1	high	8.6	6.8	0.02	32	10.1
PS	66	M	3	1	23.4	3.9	144	1.68	0.75	137.9	1.3	high		3.3	0.5	25	10.5
PS	69	F	3	1	18	3.8	194	1.32	0.68	87.23	1.3	high	8.7	5.3	0.84	18	10.6
PS	50	M	14	1	20	4	145	1.84	0.83	90.55	2.1	high	10.2	5.9	0.58	21	11.1
PS	59	F	23	1	19.7	3.1	206	1.27	0.82	81.63	2.1	high	8.5	4	0.07		
PS	44	F	7	1	20.9	3.5	162	0.93	0.93	100.9	1.6	high	10.4	5.3	0.01	12	10.9
PS	60	M	5	1	24.2	3.6	143	0.91	0.73	104.9	1.5	high	8.5	3.7		26	11.2
PS	49	M	4	1	25.7	3.7	121	1.41	0.81	100.2	1.8	high		6.2			9.7
PS	40	M	4	1	22.1	3.6	151	1.47	0.71	96.04	2.1	high	10.7	7.8		32	10.8
PS	67	M	2	1	25.1	2.9	170	1	0.81	71.43	2.1	high	8.4	4.3	0.02	27	10.4
PS	57	M	2	1	21.8	3.6	137	2.2	1.08	120.3	2.1	high	9.6	5		26	9.1
PS	31	M	2	1	23.2	3.7	149	1.53	0.72	69.97	1.3	high		7.5	0.15	27	12
PS	60	M	33	1	21.3	3.9	157	1.73	0.87	51.17	2.1	high	8.8	5.2		28	11.8
PS	69	F	23	1	14.8	4	189	1.19	0.84	87.33	2.1	high	8.5	5.8		23	10.6
PS	74	F	19	1	22.4	3.7	147	1.29	0.88	141.8	1.8	high	8.7	4.6	0.3	27	13.5
PS	59	F	16	1	23.1	3.9	132	1.35	0.76	116.1	2.1	high	7.8	8.6	0.4		11
PS	65	M	12	1	22.5	3.8	114	1	1.01	109.4	2.1	high	9.4	7.5		30	9.5
PS	31	M	11	1	20.8	4.2	123	1.3	1.01	104.6	2.5	high	8.5	5.7		33	9.7
PS	61	M	9	1	20.7	3.6	129	1.36	1	125.8	2.1	high	8	3.1		30	9.9
PS	60	M	8	1	24.1	3.2		1.48	0.64	80.03	1.5	high	8	6.6	0.38	24	9.5
PS	56	F	5	1	20.4	3.3	101	1.22	1.03	104.6	1.8	high	9.4	4.4		26	9.9
PS	71	F	5	1	23.2	3.5		1.28	0.99	129.3	1.5	high	8	3.4	0.42		11.9
PS	31	M	4	1	22.1	4.2	109	1.41	1.16	84.62	2	high		3.8	0.25	36	11.4
PS	35	M	3	1	18.5	4.1	136	1.6	0.82	119.5	1.5	high	8.4	5.5		17	10.9
PS	68	M	2	1	19.2	3.4		1.86	0.85	116.7	2	high		4.6			6.3
PS	53	M	2	1	22	4.3	154	1.37	0.75	45.29	1.3	high		7.9	0.5	37	10.6
PS	68	F	2	1	21	3.5	119	1.16	0.89	4.9	2	high	9	6.8		39	9.5
PS	47	M	14	1	36.4	4.1	159	1.4	0.81	85.57	1.5	high	9.1	5	0.32	27	11.3
PS	39	M	12	1	17		174	1.49	1.22	96.05	1.5	high	8.2	3.1		36	8.3
PS	69	M	9	1	22.7	3.8		1.89	0.88	140.1	1.6	high	9.2	4.9	3.6		10.1
PS	50	M	8	1	20.1	3.8		1.55	0.96	115	1.5	high	8.7	7			7.8
PS	61	F	5	1	18.7	4.6	97	1.39	1.07	104.8	2.5	high	8.4	3.8	0.14	25	10.7
PS	49	F	2	1	19.4	3.9	90	1.52	0.89	102.5	1.8	high	9.1	6.3	0.14	29	10.4
PS	56	M	6	1	20.7	3.5	105	1.44	0.83	88.03	2.1	high	8.9	4	0	25	11.5
PS	48	M	3	1	18.5	4	197	1.37	0.83	111.5	2.1	high	9.7	4.7	0.3	24	12.4
PS	70	M	10	1	21	4.1	127	1.32	0.96	139.9	1.8	high		5	0	25	9.5
PS	61	M	4	1	27.4	3.8	168	0.75	0.96		2.1	high	8.8	6.6	0.44	34	11.1
PS	54	F	2	1	18.1	3.8		1.4	1.11	95.83	2.1	high		5.4		28	11.3
PS	54	M	6	1	18.4	3.4		1.05	1.09	64.79	1.8	high		5.9	0.13	26	10
PS	62	F	23	1	21.1	4	154	1.52	1.08	98.73	1.6	high	8.4	6	0.05	21	9.7
PS	62	F	27	1	20.7	3.4	164	1.06	1.04	115.7	1.6	high	9	2.8	0.1	27	10.3

PS	54	M	15	1	24.1	4.3	109	1.3	1.36	138.7	1.6	high		4.3			8.8
PS	53	M	26	1	17.4	3.4	91	1.13	1.06	95.2	1.8	high	8.6	4.7	0.14	39	10.5
PS	52	F	16	1	14.7	4	153	1.55	0.99	60.68	1.8	high	9.2	3.8	0.06	31	9.2
PS	61	M	13	1	26.5	3.5	187	2.03	0.96	101.8	2.1	high	9.3	6.1		25	11
PS	62	M	10	1	21.4	4	200	1.92	0.96	130.6	2.1	high	9	4.2		33	11.7
PS	56	M	9	1	20.1	4.1	158	1.71	0.9	83.79	1.5	high	8.6	3.1		26	9.1
PS	81	F	5	1	17.9	4.2	137	1.6	1.12	87.58	1.8	high	8.8	4.3	0.05	38	10.3
PS	81	M	5	1	20	3.8	171	1.34	0.98	110.6	1.8	high	8.9	7.1	0.08	26	12.7
PS	73	F	5	1	26.2	3.8	221	1.33	1.1	103.1	1.8	high	10.3	5.6	0.5	17	10.4
PS	57	M	4	1	21.7	4.3	170	2.39	0.94	114.6	1.5	high	8.3	3.8		24	10.8
PS	73	M	4	1	24.8	4	178	1.08	0.91	98.4	1	high	9.3	4.2		25	11.1
PS	59	M	2	1	21.9	4	170	1.45	0.91	74.41	2.1	high	9	6.2	0.2	27	10.6
PS	57	M	2	1	20.9	4	127	1.1	0.92	76.12	1.5	high	10	5.9	0.16	27	9.8
PS	45	M	2	1	27.9	3.9	178	1.31	1.05	65.21	1.5	high	7.4	4.1	0.2		9.7
PS	71	F	17	1	18.2	3.6	168	1.54	0.86	110.1	1.8	high	8.6	4.5			12.1
PS	68	M	14	1	23.6	3.6	190	2.17	0.79	127.7	2.1	high	7.9	6.2		25	10.3
PS	68	F	6	1	19	3.7	113	1.4	1.01	111.2	1.8	high	8	2.5		28	8.4
PS	68	M	22	1	17.1	3.3	127	2.78	0.97	96.41	2.1	high	9.6	4.9		29	11.2
PS	67	M	16	1	22.1	3.8	137	1.56	0.68	113.4	1.6	high	9.1	11.4	0.03	29	10.8
PS	67	M	3	1	25.2	3.4	98	1.24	0.96	153.3	1.1	high	10.3	4.7	0	30	10.9
PS	67	M	5	1	21.7	3.7	156	1.65	0.88	93.92	1.8	high	9.7	4.4	0.4	29	10.6
PS	66	F	11	1	20.3	3.9	95	1.08	0.98		1.5	high	8.8	4.3	4.43	32	10.5
PS	66	M	5	1	21.4	3.9	214	1.86	0.77	133.8	1.4	high	10	6.3	0.3	26	9.3
PS	66	M	19	1	22.2	4	129	1.15	0.86	131.2	1.9	high	9.1	6.8	0.1	24	11.4
PS	64	M	9	1	28.2	3.9	132	1.7	1.02	108.3	1.5	high	9.2	5.1	0.02	36	10.2
PS	63	F	12	1	15.9	3.9	178	1.73	0.82	111.1	1	high	8.8	4.2	0.1	25	10.9
PS	62	M	5	1	18.7	3.8	185	1.72	0.81	115.6	1.3	high		6.1	0.5		10.2
PS	61	F	19	1	18.1	3.8	137	2.13	0.9	127.5	1.8	high	11	5.6	0.03	24	10
PS	61	F	15	1	19.9	3.8	151	1.39	1.04	143.2	2.2	high	9.1	6.5	0.2	29	11.4
PS	60	F	7	1	21.4	3.4	118	1.36	0.91	86.21	1.1	high	8.3	3.7	0.06	24	8.7
PS	59	F	9	1	20.6	3.9	141	1.3	1	114	1.1	high		10.4		29	8.4
PS	59	F	17	1	21.3	4	122	1.34	1.2	101	1.8	high	10.3	4.6	0.05	26	13.8
PS	57	M	6	1	15.1	3.8		1.66	0.85	92.6	1.6	high		4.6	0.14	26	10.7
PS	57	M	4	1	21.9	3.4	141	1.62	0.74	98.9	1.5	high	9.5	2.9		21	10.2
PS	57	F	11	1	15.2	3.6	83	1.44	0.81	105.4	1.5	high		2.7			9.2
PS	57	M	7	1	25.2	4.1	202	1.1	0.8	115.3	1.3	high		5.2	3.94	17	9.5
PS	56	M	20	1	18.8	3.7	189	1.39	0.75	89.03	1.6	high	10.1	5.3	0.2	19	10.4
PS	56	M	2	1	23.5	3.9	172	1.84	0.87	89.77	1.3	high	10.6	4.5	0.2	31	11.9
PS	56	M	16	1	20.6	4	162	1.43	0.88	96.85	1.6	high	9.2	3.7	0.1	19	11.6
PS	55	M	2	1	19.8	3.9		0.9	0.85	93.19	1.5	high	8.2	2.6		18	11
PS	54	M	13	1	19.9	3.7	99	1.41	0.93	132.2	2.1	high		5.7		25	10.6
PS	53	F	31	1	17.8	3.9	187	1.7	0.98	92.62	1.6	high	9.7	4.8	0.1	22	11.5
PS	52	M	2	1	16.9	3.7		1.26	0.77	82.28	1.6	high		4		17	10.4
PS	52	M	5	1	25.7	4.1	145	1.2	0.91	87.28	2.1	high	9	6	0.1	23	10.1
PS	52	F	7	1	17.5	4.1	182	1.43	1.03	97.29	1.5	high	8.5	3.9			10.8
PS	51	F	18	1	18.6	3.8	132	1.54	0.84	106.4	1.6	high		6.8	0		10.5
PS	50	M	21	1	21.6	3.8	167	1.78	0.9	110.5	1.9	high	9.3	5	0.1	21	10.3
PS	48	M	32	1	17.9	3.6	162	1.41	0.72	71.54	1.6	high		5.1	0.3		9.3
PS	48	M	13	1	20.4	3.6	143	2.15	1.03	115.6	2.1	high	8.2	6.5		21	10.2
PS	47	M	6	1	20.1	4	129	1.51	0.76	115.3	2.2	high	9.3	6	0.1	22	10.2
PS	47	M	5	1	17.2	3.8	121	1.33	0.77	80.33	1.8	high	8.6	4.5	0.04		11.1
PS	47	F	13	1	23.3	3.1	119	1.38	0.92	115.4	1.3	high		6.6	1.5		10.4
PS	43	M	18	1	17.6	3.7	136	1.39	0.88	114.9	1.6	high		5.4	0.06		8.5
PS	38	F	9	1	22.6	3.6	157	1.33	0.84	125	1.5	high	7.8	5.5	0.5		9.5
PS	66	F	9	1	17.7	4.3	115	1.6	1.02	131.7	2.5	high	9.8	6.1	0.1	31	12.2
PS	66	F	28	1	15.5	3.6	150	1.66	0.9	90.14	1.6	high	8.1	6.4	0.1	22	12.1
PS	51	M	2	1	19.6	3.9		1.42	0.73	124	1.5	high	9.3	6	0.25	28	10
PS	58	M	20	1	22	4	133	1.98	1.29	121.1	2.1	high	10.4	6.1		23	11
PS	62	F	9	1	19.2	3.7	140	1.57	1.1	85.69	1.6	high	9.5	3.9		16	8.1

PS	62	M	4	1	19.7	3.5	152	1.35	1.53	70.08	1.3	high		5.7	0.1		9.7
PS	46	M	16	1	23	4.3	132	2.48	0.93	125.9	2.1	high	9.3	6		32	12.1
PS	59	M	13	1	20	4.3		1.42	0.82	113.6	1.5	high	8.4	5.6	0.31	30	12
PS	71	M	6	1	19.1	4.1	147	1.62	1.23	127.5	2.1	high	8.8	5.5	0.1	33	10.1
PS	87	M	4	1	24.5	3.4	204	1.35	0.95	133.5	1.5	high		4.6		21	9.5
PS	66	M	6	1	24.6	3.9	142	1.67	1.09	148.9	1.5	high	8.9	4.9	0.28	27	9.7
PS	60	M	6	1	24.6	3.9		1.34	0.89	125.3	1.5	high	9.7	6.2			10.9
PS	82	M	4	1	19.3	3.8	156	1.74	0.96	125.1	1.8	high	9.4	2.9	0.06	34	11.4
PS	61	M	3	1	37.2	3.5	171	1.87	0.82	76.98	1.6	high	7.6	6.5	0.1	37	10.9
PS	49	F	15	1	21.2	4	127	1.64	1.16	106.8	1.6	high		4.7	0.1		9.5
PS	56	M	3	1	21.8	3.8	161	1.25	0.7	83.95	2.1	high	8.6	6.5	0.01		10.7
PS	58	F	6	1	16.1	3.8	188	1.47	1.03	116.6	1.5	high	9.2	4			9.2
PS	55	M	7	1	18.4	4.1	159	1.29	0.98	111.7	1.8	high	8.3	8.6	0.08	29	13.9
PS	66	F	5	1	21.7	3.7	136	1.97	0.86	109.1	1.4	high	10.2	2.6	0.7	38	10.4
PS	87	M	3	1	21.1	3.5	148	1.47	0.8	107	1.6	high	8	7.4	0.65	29	11.2
PS	83	F	12	1	21.8	3.3	113	1.21	0.81	111.6	1	high	8.7	4.6	0.1		9.3
PS	69	M	7	1	24.3	3.8	144	1.28	0.77	101.5	1.1	high	9.6	4	0.23	31	9.9
PS	49	M	2	1	22.7	3.7	126	1.48	0.81	117.8	1.3	high	10.4	5.9	0.05	29	11.8
PS	61	M	14	1	18.2	4.2	150	1.47	0.88	104.1	1.6	high		5.1	0.3		10.8
PS	49	M	21	1	24.2	3.8	171	1.63	1.04	116.6	1.5	high	8.6	4.1		21	9.1
PS	37	M	18	1	21.9	4.1	195	1.77	0.97	123.5	1.5	high	10.6	5.1	0.02	25	9.8
PS	55	M	9	1	22.2	3.9	126	1.33	1.1	122	1.5	high		3.3	0.7	25	10.3
PS	78	M	7	1	21.9	3.5	101	1.48	0.8	102.6	1.5	high	9.1	6.5			10.4
PS	79	M	4	1	19.7	3.8	152	1.06	0.83	105.6	1.5	high		6.1	0.1	20	12.6
PS	41	M	4	1	21.8	4.4	150	1.45	1.08	108.1	1.8	high		5.3	10.55	28	10.9
PS	74	F	2	1	23.6	3.5	185	1.29	0.92	115.3	2.1	high	8	6.1	0.93	28	11.4
PS	59	M	2	1	27.9	3.9	118	1.2	0.79	113.8	1.5	high	8.9	4.1	0	25	6.4
PS	64	F	17	1	27.9	3.9	159	1.2	1.17	90.67	1.8	high	9.6	7	0.37	29	11.1
PS	62	F	11	1	22.2	3.8	151	1.6	1.01	132.8	1.5	high	9.4	4.7	0.18	29	10.6
PS	57	M	10	1	21.5	4	197	1.47	1.2	123.9	1.5	high	9.3	5	0.01	30	11
PS	43	M	3	1	25.7	4	218	0.94	1.09	93.02	2.1	high	8.8	7.2	0.05	24	10.9
PS	69	M	2	1	24.2	3.7	110	0.75	0.9	94.57	2.1	high		9.5	0.14	31	10.6
PS	70	M	2	1	21.5	3.5	190	1.2	0.61	103.1	2.1	high		6	9.2	20	9
PS	83	M	7	1	19.4	3.6	150	1.33	0.73	115.8	2	high	10.8	4.5	0.1	40	14.6
PS	60	M	6	1	23.3	3.9	103	1.75	0.93	102.5	2.1	high	9.9	4.3	0.43	30	10.5
PS	54	M	3	1	26.1	3.3	124	1.61	0.91		1.6	high	9.9	8	0.05	23	10.2
PS	44	M	17	1	22	4.1	141	1.17	0.81	130.8	1.3	high		4.8	0.1		11.6
PS	63	M	9	1	19.9	3.8	167	1.37	0.96	116.7	1.5	high	8.1	5.8	0.05	30	11.3
PS	62	M	5	1	19.9	3.9	97	1.06	0.8	108.2	2.1	high	8	5.1	0.09		8.7
PS	84	M	2	1	24.8	3.9	219	1.72	0.85	126.8	1.6	high	8.4	5.4	0.11	32	10.1
PS	85	M	6	1	21.6	3.6		1.21	0.92	117.5	1.5	high	9.3	5.3	0.2	25	11.5
PS	71	M	6	1	21.9	3.5	111	1.38	0.88	96.72	1	high		9.8	0.18		9.4
PS	68	F	11	1	19.6	3.8	177	1.15	0.98	110.9	1.3	high	9.2	5.4	1.98	17	10.6
PS	61	M	2	1	21.5	3.9	180	1.54	0.99	76.49	2.1	high	8.5	5.5	0.05	44	9.9
PS	70	F	10	1	19.4	3.8	156	1.62	0.87	125.9	1.8	high	8.4	3.2	0.06	24	9.4
PS	48	M	3	1	25.2	2.7	170	1.75	1.18	137.1	1.3	high	9	5.2	0.01	25	11.6
PS	40	M	10	1	22.1	4.2	224	1.77	1.07	131.3	1.5	high	8.2	5.9	0.44	21	11
PS	57	M	5	1	23.2	4.5	188	1.32	0.88	107	1.6	high		5.3	0		10.4
PS	37	M	2	1	17.5	3.6	263	1.47	0.69	67.85	1.6	high	8.8	5.9		26	10
PS	43	M	17	1	22.8	3.6	130	1.41	1.04	122.4	1.6	high	10	9.8	0.1		11
PS	82	F	10	1	20.8	3.9	143	1.5	0.69	119.7	2.5	high	9.6	4.2	0.05	34	11.1
PS	70	F	2	1	24.7	3.8	117	1.09	1.15	121.8	1.8	high	7.5	6.9			12.8
PS	52	F	14	1	22.4	4	140	1.69	0.88	123.8	1.5	high	8.6	4.6	0.03	26	10.5
PS	59	M	14	1	20.5	3.7	218	2.68	1.09	109.3	1.6	high	9.7	11.7	0.1	41	8.4
PS	64	F	7	1	20.8	3.6	156	1.31	0.76	107.7	1.5	high		5.6	0.1	29	11.3
PS	67	F	9	1	20.1	3.7	113	1.64	0.65	113.4	1.6	high	9.2	3.6	0.1		10.8
PS	60	M	5	1	24.3	3.9	131	1.66	1.03	92	2.1	high	9.5	6	1.08	25	10
PS	56	M	19	1	24.3	4.5	117	1.24	1.24	134.3	1.6	high	9.4	6.2	0	33	9.9
PS	55	M	4	1	24.2	3.6	186	1.56	0.74	82.86	1	high		3.7	0.2	22	9.9

PS	59	M	9	1	24.8	3.8	159	1.71	0.87	109.5	1.3	high		4.8	0.1	20	10.9
PS	44	M	11	1	22.3	3.9	161	1.54	1.3	88.16	1.5	high	9.2	4.6			11.5
PS	62	F	2	1	22.9	3.6	184	1.95	0.89	125.2	1.6	high	10	5.9	0.1	23	12
PS	68	F	3	1	17.5	4.3	187	1.29	0.8	134.6	2.1	high	9.8	5.1	0.1	21	10.9
PS	50	M	22	1	16.3	4.2	168	1.43	0.83	96.24	1.6	high	7.9	6.4	0.24	42	9.6
PS	53	M	31	1	19	3.9	162	2.08	0.93	99.21	1.9	high	9.1	4.4	0.06	27	11.2
PS	57	M	29	1	19.8	3.9	205	1.36	1.05	88.94	1.6	high	10.6	5	0.05		12.6
PS	63	M	27	1	18.1	4	122	1.22	0.94	109.8	1.5	high	9.5	5.2	0.09	14	11.3
PS	54	M	26	1	18.3	4.1	185	1.46	1.11	102.9	1.8	high	9	5.1	0	26	11.7
PS	40	M	26	1	20.7	4.2	101	1.61	0.83	106.5	1.6	high	9.1	3.3	0.02	34	11.3
PS	56	M	24	1	21.5	3.8	162	1.57	0.82	111.7	1.3	high	9.3	3.1	0.06	27	10.1
PS	56	F	23	1	23.7	3.7	114	0.98	1	122.3	1.3	high	8.9	3	0.05	10	11.2
PS	48	M	19	1	19.8	3.8	110	1.65	1.13	111.2	1.9	high	9.2	6.7	0.03	29	11.4
PS	40	M	17	1	21	4.2	120	1.71	0.61	109.2	1.3	high	8.6	4.3	0.01	25	10.1
PS	58	M	17	1	22.7	3.6	134	1.61	0.93	129.5	1.3	high	10.8	3.4	0.14	32	9.3
PS	39	M	17	1	22.4	4.1	133	1.41	1.23	123.9	1.9	high		5.9	0	23	11
PS	60	M	16	1	19.5	3.9	177	1.28	1.14	104.3	2.1	high	7.7	6.6			10.3
PS	45	M	16	1	18.2	3.8	150	1.22	0.77	105.2	1.6	high	8.7	3.8	0.07		11.4
PS	61	M	14	1	22.1	3.5	232	1.47	1.12	114.3	1.3	high	8.7	7.6	0.02		10.3
PS	70	M	14	1	21.3	3.9	142	1.71	0.92	119.3	1.3	high	9.5	3.3		36	8.4
PS	33	M	6	1	18.6	4	104	1.05	1.1	84.26	1.5	high	8.4	4.3	0.05	16	11.3
PS	68	M	10	1	22.5	3.5	107	1.57	0.95	120	1.5	high	8	4.5	0.07	27	13.2
PS	54	M	10	1	19.6	4.1	181	1.65	0.93	114.7	1.9	high	8.8	4.6		24	10.7
PS	49	M	17	1	16.7	4	179	1.46	0.82	64	1.6	high	9.7	7.1	0.05	34	10.7
PS	75	M	9	1	18.6	3.7	182	1.59	1.08	99.59	1.9	high	8.8	5.5		13	10.1
PS	77	F	9	1	21	3.6	166	1.73	0.97	122.2	1.8	high	9.8	5.2	0.3	43	9.1
PS	54	F	8	1	17	3.7	201	1.35	1.03	103.9	1.9	high	7.6	7.9		17	10.5
PS	72	M	5	1	21.8	3.9	211	1.39	1.11	136.1	1	high	6.4	6.3	0.1		9.6
PS	56	M	10	1	19.8	3.9	165	1.22	0.88	121.7	1.9	high	10.6	7.4	0.06		10.6
PS	83	F	5	1	21.5	3.7	205	1.08	0.69	77.65	1.6	high	9.4	6.1	0.31		7.5
PS	79	F	5	1	18.4	3.3	210	1.15	0.92	89.65	2.1	high	8.2	6.1	0.3	34	10.2
PS	56	F	5	1	28.2	3.3	129	1.11	0.76	100.8	1.5	high	9.1	3			10.6
PS	49	M	3	1	24.7	3.2	105	0.78	0.84	100.9	1.1	high		6	0.17	25	9.8
PS	63	F	2	1	18.2	3.8	155	1.63	0.67	86.18	1.8	high	9.2	6.6	0.1	17	11.5
PS	46	F	2	1	26	3.7	193	1.13	0.78	93.61	1.6	high	8.9	5.2	0.05		14.9
PS	46	M	2	1	21.1	4.6	101	1.01	0.83	102.2	1.6	high	6.8	4.4	0.08		7.8
PS	58	M	5	1	23.4	3.5	99	0.83	0.94	95.07	1.5	high	8.6	8.4	0.1	26	13.3
PS	45	M	24	1	20.2	3.6	132	1.13	0.87	48.51	1.1	high	9.2	6.7	0.28	28	13.5
PS	46	F	23	1	20.3	3.8	147	1.64	0.83	128.9	2.1	high	9.1	6	0.04	18	6.6
PS	80	M	10	1	21.3	3.7	92	1.6	0.67	139.3	1.5	high		5.4	0.01	23	11.1
PS	80	M	6	1	24.6	3.6	190	1.15	0.81	112.9	2.1	high		5		26	9.4
PS	70	M	5	1	22.2	3.5	200	1.47	0.73	158.7	2.1	high	10	4.4		24	11.4
PS	62	M	4	1	25.9	3.6	135	1.41	0.91	74.08	2.1	high	10.2	6.8			10.4
PS	69	M	4	1	19.5	3.9	110	1.05	0.93	132.9	1.9	high	7	2.8	0.34		10.9
PS	59	M	3	1	26.5	3.5	140	2	0.82	67.91	1.6	high	9.1	6.7	0.05	29	10.9
PS	68	M	2	1	20.4	3.7	100	1.45	0.65	58.72	1.5	high	9.1	4.1		25	10.6
PS	73	M	2	1	23.4	3.7	107	1.26	0.98	111.8	1.8	high		6.2	0.6	28	10.8
PS	61	M	2	1	20.1	3.7	139	1.54	0.86	112	2.5	high	9.8	4.6	0.3	23	10.3
PS	71	M	2	1	21.9	3.6	166	1.06	0.66	90.34	2.1	high	8.9	4.2	0.3	21	8.8
PS	86	F	4	1	24.1	3.3	119	1.55	0.77	103.9	1.3	high	8	2.4	3.67	34	7.9
PS	50	M	3	1	22.5	3.8	139	1.62	0.82	117	1.8	high	8.2	8.3		30	11.7
PS	50	F	3	1	19.7	4.2	147	2.19	0.91	78.03	2.1	high	8.6	5.2	0.26	35	10.6
PS	53	F	3	1	19.8	3.3	132	1.59	0.54	48.61	1.5	high	9.2	4.2			10.5
PS	75	M	10	1	27.2	3.7		1.38	0.68	84.63	1.5	high	8.3	3	0.1	30	10.1
PS	69	F	14	1	16.6	3.9	196	1.87	0.99	130.2	2.1	high	9.5	4.9	0.1	32	11.9
PS	59	F	12	1	16.4	3.8	118	1.12	0.88	120.6	1.6	high	11.2	5.1	0.2	32	9.6
PS	35	M	10	1	14.5	3.5	121	0.97	1.02	82.26	1.9	high	8.2	5.9		35	12.1
PS	51	F	9	1	22.4	3.9	169	1.57	0.75	98.38	1.3	high	8.2	5.5	0.05		10.1
PS	64	F	9	1	23	4.2	141	1.97	1.17	116.4	2.1	high	8.8	5.5		43	10.7

PS	55	F	16	1	20.1	3.3	127	1.98	0.75	112.8	2.1	high	8.7	6.8		52	12.6
PS	54	M	30	1	20.8	3.7	150	1.52	0.81	111	2.1	high	9.6	5.3	0.03	28	11.2
PS	42	F	20	1	18.7	3.8	162	2.86	1.01	95	2.1	high	9.5	4.4		25	10.4
PS	38	F	2	1	24.6	3.7	110	1.49	0.69	103.1	1.8	high	9.6	4.2	0.81	34	7.6
PS	59	F	20	1	19.6	3.6		1.22	0.86	98.87	2.5	high		6.2	0.56	34	10.6
PS	71	F	15	1	22.1	3.9	133	1.56	0.92	85.96	2.1	high		5.8		37	12.3
PS	70	F	11	1	17.6	3.4	111	1.58	0.87	84.97	1.5	high	8.6	4.4	0.08	28	10.1
PS	45	F	10	1	18.1	3.8	126	1.32	0.8	92.24	2.1	high	8.2	5.6	2.04	31	11
PS	70	M	8	1	20.8	4	152	1.55	0.76	122.9	1.1	high	8.7	4.5	0.06	24	10.9
PS	52	F	5	1	21.6	1.6		1.35	1.05	98.16	1.5	high	8.6	2.5	0.59	17	8.9
PS	59	M	2	1	20.8	4	177	1.32	0.73	59.82	1.8	high	8.8	4.9	0.8	31	10.5
PS	50	F	26	1	16.3	4.3	194	1.53	1.03	117	1.6	high	9.6	5.9	0.04	31	9.5
PS	55	F	9	1	17.2	3.7	183	1.94	0.99	135.3	1.8	high	7.8	2.5	0.05		11.4
PS	72	F	3	1	27.6	3.7	177	1.5	0.76	77.87	1.8	high	8.1	2.8	2.69	22	8.7
PS	61	M	3	1	17.1		137	1.41	0.69	93.9	1.5	high	9.9	5.5	0.72	36	11.7
PS	58	F	3	1	23.5	3.9		1.5	0.64	96.67	1.3	high	9.2	3.1		18	
PS	50	F	12	1	18.3	3.7	190	2.59	0.99	112.1	2.1	high	8.3	4.1		31	10.4
PS	60	F	4	1	22.4	4	180	1.09	0.79	118.3	1.6	high	6.7	4.4	0.02	26	10.4
PS	69	M	2	1	20.5	3.5	128	1.45	0.77	74.82	1.8	high	8.4	6.9		48	12.1
PS	47	F	23	1	22.5	3.7	105	1.98	1.19	88.19	2.1	high	8.8	5.6		27	10.2
PS	67	M	3	1	24.3	3.7	96	0.95	0.63	95.26	1.8	high	8.9	7.1	0.47	29	12.1
PS	30	F	4	1	31.5	3.7	153	1.73	0.7	108.6	1.1	high	9.2	4.1	0.11	26	10.4
PS	60	F	17	1	21.7	3.8	133	2.01	0.99	99.46	2.1	high	9.4	5.3		28	10.8
PS	63	F	2	1	35.7	3.5	169	1.25	0.99	89.39	1.6	high	7.8	7.3		23	10.6
PS	60	F	13	1	21.5	3.7	151	1.37	1.33	107.3	1.8	high	9.4	6.9	0.15	35	11.4
PS	67	F	14	1	19.2	3.8	137	1.64	1.21	86	1.1	high	9.8	2.9	0.83	23	5.7
PS	59	F	8	1	18.9	3.3		1.06	1.12	156.1	1.6	high	10.8	5.3		31	10.9
PS	74	F	4	1	25.4	3.6	190	1.22	0.77	100.2	1.1	high	9.9	4.5		40	11.1
PS	49	F	9	1	16.3	3.6	166	1.03	0.7	99.35	2.1	high		5.4	0.05	35	15
PS	65	M	6	1	23.3	3.1	146	1.36	0.77	105.3	1.5	high	8.9	6.2	0.09	22	11.7
PS	50	M	29	1	18.8	3.8	105	2.11	1.08	97.15	2.1	high	9.1	4		26	10.1
PS	53	M	28	1	21.2	3.3	134	2.55	1.09	116.7	2.1	high	9.7	4.8		26	10.3
PS	50	F	9	1	17.5	3.2	189	1.7	0.99	95.39	1.8	high	9.6	7.1	0.9	33	9.7
PS	43	M	20	1	16.5	3.9	131	1.84	0.85	95.85	2.1	high	8.9	5.5		25	10.2
PS	58	M	20	1	24.1	3.4	131	2.22	0.86	102.4	2.1	high	9.5	5.3		38	11.9
PS	58	M	20	1	21.4	3.7	147	1.72	0.93	123.6	2.1	high	9.6	5		25	12.3
PS	53	F	19	1	18.8	3.6	143	1.47	1.02	116.6	1.8	high	10.2	5.3	0.03		10.5
PS	71	M	11	1	18.9		101	0.99	0.84	129.2	1.5	high	8.9	5.2	0.05		11
PS	40	F	11	1	18.8	4	211	1.95	0.82	117.7	1.5	high	9.8	6.6	0.26	24	9.5
PS	69	M	9	1	18.5	4	137	1.08	0.75	89.29	2.1	high	8.8	7.3	0.29	34	7.5
PS	75	M	8	1	23.5	3.2	131	1.37	0.68	92.96	2.1	high	9.4	4.8		28	10.6
PS	35	M	8	1	19.3	3.7	147	1.37	0.95	83.84	1.5	high	8.5	3.9	0.66	27	9.5
PS	65	F	8	1	28.4	3.8	102	1.72	0.84	155.9	1.5	high	8.7	4.9		22	8.7
PS	62	M	7	1	24.7	4.1	200	1.26	0.85	130.3	1.5	high	8.9	5	0.02	28	11.4
PS	64	F	9	1	21.6	4.4	100	1.73	1.09	124.7	2.5	high	10	5.7	0.01	33	11.4
PS	70	M	6	1	19.2	4.1		1.33	1.22	139.1	1.8	high	10.7	5			12.3
PS	64	M	5	1	23.7	4	118	1.77	0.88	83.38	1.5	high	9.6	4.7	0.08	32	13.2
PS	63	F	4	1	21.3	4	131	1.13	0.78	79.83	2.1	high	9	5.7		37	10.3
PS	52	M	3	1	24.3	4	173	1.21	1.08	97.33	2.1	high	8.9	3.2		19	9.6
PS	66	F	2	1	22.5	3.7	172	1.21	0.81	107.3	1.6	high	7.8	8.6		33	12
PS	63	M	2	1	18.4	3.8	145	1.8	0.88	85.34	2.1	high	8.3	5.7	0.04	25	10.2
PS	49	M	2	1	31.3	4.1	169	1.52	0.83	89.1	1	high	9.5	4	0.1	28	10.8
PS	84	M	2	1	18.7	4.1	104	0.9	0.77	85.93	1.3	high	7.9	5	1.1	33	9.4
PS	50	M	2	1	25.2	4	162	1.51	0.96	110.9	2.1	high	9.9	5.7		17	10
PS	67	M	3	1	20.9	4.1	174	1.62	0.88	134.2	1.5	high	9.7	5.6	0.83	34	12.1
PS	59	F	3	1	20.5	3.8	139	1.68	1.18	98.91	1.6	high	9.7	5.5	0.31	31	10.1
PS	59	F	10	1	17.3	3.9	87	1.13	0.89	92.77	1.5	high	8.2	5.2	3.54	43	7.5
PS	29	M	6	1	18.3	4.2	152	1.81	0.96	98.77	1.9	high	10.5	6	0.03	33	10.6
PS	57	F	5	1	22.5	4	126	1.81	0.91	121.5	1.9	high	10.7	4.5	0.08	29	11.8

PS	71	M	2	1	20.3	3.7	189	1.36	0.91	59.19	1.9	high	8.5	4.8	0.04	30	10.2
PS	63	M	6	1	22.7	3.8	149	1.68	1.2	112	1.9	high	9.5	4.2	4.12	36	9.1
PS	81	M	3	1	23	3.8	162	1.06	0.66	75.63	1.6	high	10.4	4.8	0.7	26	11.6
PS	50	M	2	1	20.5	4.1	166	1.26	0.93	92.14	1.3	high	8.9	3.6	0.4	18	11.5
PS	61	M	4	1	21	3.9	170	1.31	0.74	88.16	2.1	high	8.4	5.9		33	9.5
PS	73	F	2	1	24.5	4	170	1.19	0.93	67.72	2.1	high		5.1			8.8
PS	61	M	2	1	27	3.7	188	1.32	0.75	110.7	2.1	high	8.6	5.6	0.2	20	10
PS	65	M	5	1	23.2	3.6		1.48	0.79	129.2	2.1	high	10.1	4.6			9.8
PS	41	M	2	1	21.2	4.4	139	1.62	0.75	112	1.5	high	8	4.3	0.18	32	7.3
PS	58	M	7	1	22.5	4.3	130	1.49	1	86.2	1.5	high	7.6	5.3	0.3	27	11.1
PS	59	M	23	1	17.5	3.4	162	1.8	1	118.9	1.8	high	7.8	5.1	0.05	20	11.5
PS	62	M	20	1	19.2	3.7	219	1.37	0.97	158.9	1.4	high	9.4	6	0	21	11.1
PS	69	M	9	1	24.1	3.8	152	2.45	1.08	130.9	2.1	high	9.4	4.4		20	8.8
PS	60	M	4	1	20	3.5	146	1.36	0.83	92.24	1.8	high	10	4.4	1.08	30	9.9
PS	63	F	6	1	17.5	4.2	137	1.84	0.92	67.07	1.5	high	11.2	5.8	0.06	28	11.6
PS	69	M	5	1	17.9	4	221	1.53	1.02	82.83	1.3	high		4.6	0	30	11.4
PS	57	M	2	1	21.4	4.2	158	1.81	0.69	117.4	1.3	high		6.5	0.1	17	10.7
PS	60	F	2	1	20.4	3.7	171	1.78	0.89	104.2	1.3	high		5.4	0.1	20	9.9
PS	76	F	11	1	17.1	3.5	133	1.29	0.69	123.5	1.5	high	9.8	3.7			
PS	63	F	8	1	21.8	3.9	129	1.73	0.72	118.5	1.6	high		4.9		9	9.9
PS	75	F	6	1	17.2	3.8	122	1.49	0.66	94.14	1.8	high	8.4	5.2	0.08	27	9
PS	50	M	4	1	22.4	4	235	1.23	0.88	130.2	1.8	high	9.9	4.3	0.14	22	10.8
PS	80	F	3	1	23.1	3.7	145	1.66	0.56	85.06	1.6	high	9.6	3.6	0.52	33	11.9
PS	66	M	3	1	18.3	3.7	216	1.09	0.92	84.9	1.1	high	10	4.2	0.07	26	11.4
PS	43	M	17	1	21.1	4.2	121	1.2	1.13	90.51	1.3	high	9	5.9	0.06	38	9.4
PS	81	F	5	1	25	3.4	151	1.3	0.84	112.9	1.9	high	9.6	5.1	0.14	31	10.7
PS	59	F	24	1	18.2	3.9	188	1.11	0.96	121.2	2.1	high	8.7	6	0.16		9.9
PS	54	M	2	1	19.1	3.3		1.17	0.72	73.11	1.5	high	9.6	7.6	0.12	20	9
PS	56	F	17	1	17.4	3.6	145	1.25	1.27	141.3	1.8	high	8	8	0.2	28	9.3
PS	50	M	13	1	28.6	3.6	156	1.32	0.91	130.8	1.5	high	8.4	4.4	0.2	25	11.3
PS	66	F	12	1	17.6	3.6	146	1.21	0.96	104.6	2.2	high	9.7	6.6	0		11.2
PS	62	M	5	1	22.3	3.9	181	1.79	1.13	95.67	1.5	high	9	6.8			9.1
PS	43	F	19	1	22.6	4.1	129	1.65	1.09	99.16	1.6	high	8.6	2.9	0.26	46	10.5
PS	71	M	11	1	19.1	3.4	136	1.48	0.92	120.9	2.1	high	9.1	4.9	0.1	36	9.8
PS	59	M	2	1	30.2	3.8	214	1.66	0.69	57.11	1.5	high	9.6	5.4	0.2	30	10.9
PS	63	M	12	1	19.2	3.7	195	1.59	1.16	125.9	1.5	high	9.7	7	0.2	21	12.5
PS	67	F	4	1	22.2	3.3	110	1.44	0.71	84.91	1.5	high	8.8	5.7		25	11
PS	58	M	3	1	18.4	4.1	148	1.33	0.68	46.75	1.3	high	8.3	7.4	1.17	27	10
PS	70	M	18	1	19.4	3.9	164	1.45	0.98	110.3	1.9	high	9.9	4.2	0.11	35	12.8
PS	43	M	7	1	22.5	3.8	183	1.51	1.13	128.7	2.1	high	10.4	6.1			9.9
PS	38	M	6	1	18	4.2	128	1.45	0.76	111.7	1.6	high	8.3	5.4	0.22		7.8
PS	70	F	5	1	18.1	3.1	136	2.03	0.62	91.94	1.1	high	9	6.2		21	9.2
PS	50	F	18	1	17.7	3.5	173	1.38	1.21		1.6	high	9.2	7.6	0.29		9.3
PS	41	M	14	1	32.7	3.8	190	2.03	1.26	74.89	1.8	high	10	7.5	0.55	31	10.9
PS	61	F	23	1	18.6	3.3	137	1.09	0.86	111.3	2.1	high		7.6	0.21		12.1
PS	40	F	20	1	19.4	3.5	165	1.21	0.91	101.7	1.5	high	10.2	6.1	0	23	10.7
PS	69	F	12	1	16.4	3.9	136	0.97	1.11	102.5	1.6	high	8.8	9.5	0.02		10.4
PS	65	M	2	1	19.2	3.7		1.23	0.96	79.22	1.5	high	8.4	5.4	0.07	19	10.9
PS	55	F	5	1	20	3.6	154	1.44	0.79	74.71	2.1	high	10.1	5.4	0.2	28	8.1
PS	83	M	3	1	20.8	3.1	161	1.47	0.85	118.4	1.6	high	9.6	4.4	0.09		11.4
PS	61	M	2	1	19.5	3.6	50	1.23	0.96	99.16	1.5	high	9.4	3.1	0.53	29	8.9
PS	57	F	27	1	17.4	3.7	144	1.06	1.16	113.2	1.6	high	8.9	5.6	2.4		8.4
PS	69	M	3	1	20.9	3.6	102	1.35	0.91	160.6	1.5	high	9.5	3.7	0.06	29	9.3
PS	57	M	4	1	19.8	3.8	151	1.84	0.74	98.63	1.5	high	8.6	4.1	0.02	20	9.3
PS	67	M	2	1	19.8	3.5	179	1.56	0.75	89.04	1.6	high		3.3		18	9.5
PS	62	M	2	1	23.1	3.5	130	1.35	0.72	123	1.5	high	8	3.3			11
PS	84	F	9	1	17.7	3.6	154	1.64	1.03	98.58	1.3	high	8.6	7.7	0.07	22	11.3
PS	55	M	5	1	20.7	3.9	173	1.43	0.91	117.6	1.6	high	8.1	8.4	0.07		10.9
PS	30	F	11	1	19.9	3.6	150	1.37	1.13	96.84	1.9	high	8.9	3.3	0.65		7.2

PS	51	F	25	1	15.7	4		1.82	0.71	118.1	1.6	high		4.9	0.3	30	8.8
PS	58	M	15	1	20.3	3.7	165	1.6	0.9	124.3	1.8	high	8.9	5.6	0.2		11.7
PS	67	M	11	1	18.8	3.5	210	1.75	0.94	130.7	1.3	high	9.3	6	0	31	11.8
PS	60	M	10	1	17.4	3.4	235	1.41	0.83	113	0.8	high	9.9	4.8	0.1	25	9.9
PS	67	M	5	1	19.1	3.2	206	1.54	1.16	111	1.8	high		2.9	0	25	9.1
PS	67	M	2	1	26.8	3.6	94	1.01	0.92	157.7	1.6	high	9.4	4.8	0.1	36	9.7
PS	68	F	24	1	15.9	3.5	207	1.51	1.07	95.4	1.6	high	8.3	5.4	0.14		11.4
PS	62	M	17	1	19.6	3.8	201	1.55	0.84	90.94	1.5	high	9.2	4.1	0.74		10.3
PS	73	M	11	1	23.2	3.8	198	1.5	0.81	147.4	1.9	high	8.5	4.8	1.5	32	10.4
PS	68	M	10	1	17.2	3.4	182	1.14	0.56	65.38	2.1	high	8.4	5.3	0.97	25	11
PS	83	F	10	1	20.4	3.2	168	1.39	1.03	157.9	1.5	high	9.7	4.1	0.4	26	9.5
PS	61	M	9	1	22	3.8	127	1.45	1	121.8	2.1	high	9.7	4.4	21.5	22	12.3
PS	67	M	5	1	21.8	3.7	161	1.47	0.73	105	2.1	high	8.8	6.9	3.25	29	12.7
PS	53	F	5	1	16.1	3.7	131	1.42	0.68	84.55	1.5	high	8.7	4.2	0.02	27	11.7
PS	55	M	4	1	20.5	3.7	116	1.31	0.89	106.3	1.6	high	7.5	5.3		30	11.2
PS	70	F	4	1	18.3	3.4	117	0.81	0.96	112.9	2.5	high	8.8	5		22	9.2
PS	50	M	4	1	27.3	3.7	93	1.26	0.6	102.6	2.1	high	8.8	5.8	0.31	21	10.6
PS	75	F	4	1	18.3	3.8	200	1.76	0.79	111	2.1	high	9.3	6.7	0.64	27	8.3
PS	83	M	4	1	20.7	3.3	192	1.69	0.73	127	1.9	high	9.3	5.2		31	10.5
PS	67	F	3	1	15.3	3.6	129	1.49	1.07	86.73	1.6	high	8.7	5.1	0.13		10.1
PS	67	M	3	1	19.5	3.8	127	1.33	0.95	111.4	1.5	high	8.2	5.6	0.6		11.6
PS	64	F	3	1	16.9	3.9	199	1.32	0.78	79.4	1.6	high		4.3	0.39	33	9.8
PS	67	M	3	1	18.4	3.6	157	1.68	0.92	78.2	1.5	high	9.8	4.6		31	11.5
PS	74	M	2	1	18	3.3	284	1.8	0.73	124.1	1.5	high	11.1	6.7	0.34		10
PS	46	F	2	1	14.8	3.9		1.07	0.81		2.1	high	9.2	6.2	0.99	33	10.7
PS	63	M	9	1	18.7	4	163	1.28	0.87	100.3	1.9	high	8.7	5.1		38	12.8
PS	50	M	6	1	28.9	4.1	99	1.26	1.17	129.1	2.1	high	9.2	5.4		29	10.5
PS	61	F	2	1	27.6	4.3		1.75	0.9	105.3	1.1	high		5.6			9.1
PS	56	F	14	1	17.1	4.2	159	1.6	0.93	132.2	1.5	high	10	6	1.75	36	10.8
PS	55	M	3	1	21.6	4	130	1.06	1.16	77.08	2.1	high		4	0.15	31	12.2
PS	62	M	7	1	22.4	4.1	172	1.22	0.86	95.15	1.9	high	9.1	6.2	0.1		13.1
PS	65	M	3	1	25.3	3.9	115	1.52	0.84	112.5	1.9	high	9.3	5.6	0.05		9.6
PS	55	M	3	1	28.1	4.2	109	1.87	0.91	110	1.5	high	8.9	5.7			9
PS	51	M	2	1	24.4	4	167	1.32	0.69	91.67	2.1	high	8.7	2.7	1.13	36	10.7
PS	53	M	17	1	20	4.3	165	1.79	0.95	101	1.9	high	9.1	4.5	0.05		8.6
PS	50	M	11	1	19.9	4.2	151	1.94	1.16	114.4	2.1	high	9.3	7		28	10.7
PS	50	M	5	1	24.7	4.3	161	1.14	1.37	141.8	1.9	high	9.3	9.8	1.15		9.1
PS	42	M	6	1	27.1	4.3	181	2.18	0.81	109	1.5	high		4.9		18	10.3
PS	72	M	12	1	19.4	4.1	141	1.19	0.77	108.5	1.9	high	9	4	0.18		9.5
PS	65	F	9	1	21	3.7	72	1.44	1.21	138.3	1.9	high		4.2		33	9.5
PS	66	M	4	1	17.3	3.5	147	1.47	0.88	78.24	1.6	high	9.6	5.3	0.04	29	13
PS	66	F	8	1	21.1	4.1	163	1.4	1.19	134.5	1.9	high		6		28	10.7
PS	45	M	2	1	37.4	3.5	156	1.49	0.74	128.5	1.8	high	7.2	5.1	0	38	8.2
PS	72	F	4	1	22.7	3.7	191	1.78	0.53	84.14	1.3	high	9.1	6.7	0.32	22	10.6
PS	65	M	3	1	24	4	118	1.19	0.75	114.5	1.9	high		4.6		32	10.2
PS	72	M	5	1	21.9	4	127	1.46	0.91	123.6	2.5	high	10.1	6.1	0	28	12.4
PS	71	M	8	1	23.1	3.6	168	1.32	0.85	139.5	1.9	high		5		34	9.6
PS	59	M	5	1	21.2	4.4	192	1.25	0.96	131.9	1.8	high	8.7	5.4	0.08	24	11.1
PS	72	M	11	1	21.3	4	180	1.75	0.77	112.8	1.3	high		6.3		26	9.7
PS	63	M	3	1	24.8	3.6	171	1.75	0.89	158.5	1.3	high		5.5		22	9.2
PS	53	F	2	1	20.3	3.8	137	1.34	0.93	104.6	1.5	high	8.2	3.3		19	8.6
PS	60	F	2	1	24	4.1	150	1.36	0.93	87.29	1.9	high		4.4		30	9.1
PS	71	M	15	1	18.8	3.9	145	1.54	0.99	119.1	1.3	high		7.1		24	9.4
PS	71	M	5	1	18.6	4.6		1.1	0.74	87.48	1.8	high	8.4	7.4		27	8.6
PS	53	M	10	1	20	4.1	162	1.17	1.14	121	2.1	high	9.8	4.2			9.7
PS	83	F	9	1	19.5	4.2	181	1.16	1.02	85.19	1.3	high		5.7		26	9.6
PS	73	F	4	1	18.5	4.2	106	1.16	0.9	98.79	2.1	high	9.9	6.3	0.07	28	14.5
PS	70	M	4	1	22.8	4	168	1.19	1.2	131.9	1.9	high		5.7			9.6
PS	55	M	7	1	22.3	4.4	201	1.08	0.86	133.1	1.6	high		3.6			9.9

PS	62	M	5	1	25.4	4.2	177	1.6	1.17	107.2	1.1	high	8.6	4.5		26	10.3
PS	57	M	17	1	20.2	3.9	150	1.43	1.03	115.3	1.3	high	9.1	3.3	0.1	24	10.3
PS	59	F	5	1	15.6	4.3	151	2	0.98	114.3	1.3	high	9.1	3	0.31	25	11.2
PS	63	F	9	1	22.8	4	136	1.43	0.89	135.3	1	high	8.2	5.8		28	10.6
PS	58	M	2	1	25.7		160	1.25	0.79	95.78	2.1	high	9.9	5.8	0.14	32	
PS	61	M	22	1	22.4			1.52	0.75	105.1	2.2	high	9.3	4.6			11.5
PS	61	M	19	1	19.5		128	1.56	0.74	79.21	1	high	9.9	6.1		22	11.1
PS	55	M	18	1	21		162	1.92	0.75	103.2	1.3	high	9	4.4		30	11.5
PS	55	M	17	1	29		135	1.67	0.8	102.3	1.3	high	9.1	5.1		28	11.9
PS	37	M	16	1	16.7		175	1.92	0.9	99.32	1	high	8.9	6.1		27	10.9
PS	43	M	16	1	16.3		175	1.45	0.78	64.56	1.3	high	10.7	6.7		28	10
PS	61	F	14	1	14.6		124	1.32	1.06	103.8	1.8	high	8.1	3.7	0.06	17	11.2
PS	62	F	13	1	23.7		139	1.49	0.86	116.6	1.3	high	10.7	1.9		26	10.7
PS	44	F	9	1	25.2		95	1.36	0.76	53.27	1.5	high	9.3	4.6		27	9.8
PS	37	M	8	1	20			1.22	0.88	104.1	1.3	high	8.7	5.7		23	10.2
PS	66	M	7	1	23.2		170	1.78	0.98	119	1	high	9.1	4.8		27	10.3
PS	65	M	6	1	20.4		190	1.65	0.72	100.7	1	high	9	3.4		26	9.6
PS	81	F	3	1	18.8			1.4	0.85	92.52	1.6	high	9.7	4.6	0.15	39	8.1
PS	71	F	21	1	18.3	3.9	176	1.43	0.78	119.3	2.1	high	8.4	6.2	0.19	29	10.9
PS	55	F	14	1	19.8	3.8	133	1.07	0.84	124.6	2.1	high	9.9	5.5	0.63	33	10.8
PS	74	M	13	1	16.4	4.4		1.6	0.98	118.7	1.6	high	8.9	5.3	0.41	29	11
PS	77	M	11	1	17.2	4		1.08	0.88	87.11	1.9	high	9.4	5.2	0.02	26	9.8
PS	68	F	7	1	21.6	3.8	147	1.32	0.66	105	2.1	high	8.9	6.4	0.05	25	10.7
PS	88	M	2	1	20.9	5.6		1.6	0.76	100.4	1.6	high		5.2	0.13	26	12.1
PS	54	M	16	1	23.6	4.3	159	1.27	1.04	124.8	1.9	high	8.6	3.5			10.3
PS	75	M	2	1	20.8	3.2		1.3	0.57	50.14	2.1	high	9.1	6.8			11.1
PS	63	M	3	1	22.9	3.6	144	1.9	0.87	82.02	1.3	high	8	3.1	0.4	27	6.3
PS	61	M	34	1	18.7	3.9	134	1.44	0.86	59.54	2.2	high	9.5	6.1	0		10.4
PS	64	F	15	1	15.1	3.1	224	1.08	0.69	81.2	1.5	high	8.6	4.5	0.1		11.2
PS	61	M	14	1	24.1	3.8	158	1.56	0.81	151.4	1.6	high	10.1	5.3	0.1	26	8.6
PS	68	F	3	1	25	3.3	191	2.15	0.77	86.85	1.5	high	9.5	5.3	0.57	29	11.6
PS	82	F	3	1	21.6	3.9	206	1.78	1.04	117.1	1.8	high	10.2	3	0.1	30	13.7
PS	75	F	6	1	22.3	3.8	133	1.55	0.99	102	1.8	high	8.1	5.9	0.35		11.4
PS	66	M	26	1	20.4	3.4	146	2.33	0.88	112.7	1.5	high	8.3	5		31	11.4
PS	61	M	18	1	15.6	3.4	163	1.56	0.7	45.33	1.6	high	10.4	4	0.1	24	8.7
PS	75	F	16	1	15.1	3.6	104	0.99	1.19	105.7	1.4	high	10.5	3.9	1.4	37	10.3
PS	50	F	16	1	19.7	4.2	146	1.39	1.06	65.98	2.1	high		5.9	0.28	23	11.4
PS	60	M	14	1	24.2	3.7	177	1.16	0.87	99.03	2.1	high	9.2	5.9	6.15	27	10.3
PS	59	M	14	1	18	3.7	79	1.66	0.92	119.9	1.4	high		1.4	5.94		4.9
PS	65	F	13	1	20.1	3.6	113	1.39	0.89	119.6	1.8	high	7.9	8.3	0.48	31	10.7
PS	62	M	10	1	18.7	3.9	134	1.84	0.95	109	2.1	high	8.2	6.4	0.2		10.9
PS	75	M	10	1	20.3	3.9	205	0.7	0.8	121.1	1.5	high	9.9	7.8	0.05	23	9.5
PS	74	M	10	1	22.5	3.2	124	0.91	0.66	99.25	1	high		3.6	3.6		10.9
PS	49	F	9	1	20.1	3.2	166	1.09	0.62	75.96	2.1	high		5.1	0.13	26	11.4
PS	61	F	9	1	18.8	3.9	197	1.48	1	75.85	1.6	high		5.6	0.1	29	10.2
PS	65	M	9	1	24.3	3.5	115	1.24	0.82	122.3	1.1	high		4.8	2.4	30	9.4
PS	53	M	9	1	23.4	3.4	149	1.51	0.73	103.2	1.8	high	9.8	4.4	1.11	23	9.8
PS	69	F	8	1	20.6	3.8	110	1.75	0.81	127.5	0.6	high		1.9	6.35	30	11.5
PS	60	M	7	1	23.1	3.5	180	1.2	0.99	110.8	1.5	high	8.4	4.1	0.92	29	12.6
PS	58	F	6	1	18.1	3.9	155	1.52	1.08	124.1	1.5	high	8.4	5.3	0.05	23	11.6
PS	60	F	5	1	17.5	3.7	186	1.38	0.87	115	1.8	high	10.7	6.7	9.8	21	9.5
PS	60	M	4	1	20.8	3.7	169	1.29	0.8	116.6	1.5	high	9	4.6			9.8
PS	68	F	4	1	22.4	3.6	207	1.49	0.77	82.08	1.6	high	10.4	6.3		31	10.4
PS	64	F	4	1	19.2	3.7	99	2.06	0.9	97.93	1.5	high	10.7	4.9	0.3	19	8.4
PS	67	F	3	1	18.5	3.5	137	1.11	0.71	68.35	1.4	high	8.6	1.6	0.2	35	10.1
PS	60	F	3	1	26.2	3.7	133	1.1	0.88	39.89	1.6	high	11	5.3	0.01	22	11.5
PS	85	F	3	1	24.6	3.8	122	2.01	0.73	94.87	2.1	high	9.3	4.8		25	12.2
PS	74	M	2	1	26.1	3.4	88	1.12	0.76	131.7	2.1	high	8.6	9.4	0.3	21	11.6
PS	88	F	2	1	21.3	3.6	134	1.47	0.68	73.18	1.3	high	10.8	5.8	0.03	25	9.3

PS	66	F	2	1	34.5	3.6	137	1.01	0.94	77.19	1	high	10.4	8.4		26	7.7
PS	78	F	2	1	20.5	3.4	148	1.22	0.92	55.98	1.6	high	8.8	5.2		36	11
PS	54	M	2	1	21.2	3.8	106	1.16	0.79	96.51	1.5	high	8.8	5.3	0.25	18	10.2
PS	65	M	11	1	22.4	3.8	187	1.86	0.83	83.44	1.8	high	9.1	4.3	1.85	32	10.8
PS	79	F	8	1	22.4	2.7	166	2.33	0.77	63.39	1.8	high	8.7	4.5		17	9.8
PS	80	M	2	1	21.1	3	142	1.6	0.71	88.65	1.5	high		4.7	0.05	26	10.3
PS	63	F	16	1	20.5	4.1	147	1.9	1.07	102.5	2.1	high	9.2	4.5		22	7.4
PS	50	F	13	1	16.4	3.8	170	1.5	0.96	89.33	2.1	high	9.6	4.8		35	10.5
PS	61	M	10	1	19.3	3.8	223	2.11	0.89	105.1	1.8	high		5.4	0.31	24	12.5
PS	77	M	9	1	22.2	4.2	134	2.13	0.95	109.3	2.1	high	8	8	0.85	31	10.9
PS	66	M	7	1	17	4.4	144	1.12	1	122.2	2.1	high	8.7	9	0.05	24	
PS	69	F	6	1	18.5	3.8	126	1.46	0.86	140.6	1.3	high	9.5	3.2	0.1	23	9.7
PS	86	F	3	1	22.8	3.7	100	1.36	0.71	82.29	2.1	high	9.2	5.8	5.22	38	10.8
PS	63	M	2	1	19.4	3.6	111	2.25	0.81	79.71	1.5	high	9	4.1	4	25	10.5
PS	78	F	15	1	16.8	3.5	108	1.47	1.04	88.76	1	high	9	5.1	0.02	27	11.5
PS	65	F	14	1	19.8	4	113	1.96	0.79	145.8	1.8	high	9.5	6.8	1	37	10.7
PS	80	F	9	1	21.4	4	192	1.58	0.93	81.9	1.3	high	9.9	1.8	0.11	28	12.4
PS	40	F	5	1	19.6	4	95	1.64	1.33	125.8	2.1	high	9	5.1			10.9
PS	65	M	5	1	25.2	3.7	226	1.19	0.71	113.3	1.5	high	9.2	5.5	0.44	24	12
PS	53	M	16	1	31.5	3.9	120	2.15	0.99	97.09	2.1	high	10.6	5.3		20	10.2
PS	61	M	6	1	20.3	3.7	145	1.2	0.88	131.4	1.5	high		4.4			10.8
PS	41	M	13	1	25.1	3.9	195	1.93	0.95	130	2.1	high	8.7	4.3		23	10.8
PS	54	M	18	1	18.3	4		1.25	0.81	103.7	2.1	high	9	4.6	0.08	20	10
PS	75	F	26	1	20.3	3.7	143	1.31	0.82	124	1.5	high		3.5	0.03	31	11.9
PS	63	F	14	1	22.4	3.9	178	1.31	0.85	95.51	2.1	high		5.9	0.05	23	10.7
PS	55	F	8	1	22.6	3.7	154	1.63	1.05	125.7	2	high	8.6	7	0.03	33	10.4
PS	36	M	10	1	15.6	3.9	208	1.86	1.28	102.4	1.8	high	10	7.3			11.3
PS	77	F	16	1	15.8	3.4	146	2.01	0.77	87.2	2.1	high	9	4.1		26	10.2
PS	63	M	2	1	19	3.4	142	1.66	0.82	124.4	2.1	high	8.4	4.7		25	13.3
PS	41	F	16	1	21.9	3.8	155	1.86	0.8	126.5	2.1	high	8.5	5		24	10
PS	84	F	5	1	20.6	3.4	144	1.42	0.56	102.5	1.5	high	8.2	4.5	0.81	25	11.8
PS	38	M	16	1	22.8	4.1	134	1.75	0.67	117.9	1.8	high	9.6	5.7	0.15	28	10.2
PS	39	F	2	1	22.8	4.1	140	1.3	0.9	100.9	2.1	high		5.2	0.05	26	9.3
PS	73	M	7	1	18	4	249	1.51	1.06	85.78	1.5	high	10.9	7.5	0.09	29	10.9
PS	62	M	24	1	17.5	3.7	175	1.61	0.9	105.6	1.6	high	9.3	4.1	0.1	35	9.4
PS	65	M	5	1	18.7	3.6	115	1.23	1	96.42	1	high	9.5	4.4	3.11	26	11.9
PS	49	M	2	1	29.4	3.5	135	1.29	0.7	109.5	1.5	high	8.3	5.2	0.1	17	11.1
PS	59	F	2	1	16.8	4.2		1.7	1.03	141	1	high	8.6	4.5	0.74	41	8.9
PS	64	F	28	1	17.7	3.8	94	0.95	0.98	108.4	1.8	high	9	5.6	0.1	31	10.2
PS	57	M	25	1	20.1	3.8	207	2.48	1.24	105.9	1.5	high	9.2	4.9		22	10.1
PS	52	M	16	1	20.1	3.9	150	2.09	1	122.8	1	high	8.5	4.7	0.95	38	10.1
PS	54	F	15	1	20.8	3.8	185	2.26	0.99	128.5	1.5	high	10.5	4.4		24	10.7
PS	81	M	13	1	26.5	3.8	113	1.18	0.67	128.4	1	high	9	5.1	0.07		11.5
PS	62	M	12	1	23.1	3.5	139	1.46	0.94	131	1.6	high	9.6	6.4	0.29	28	9.1
PS	60	F	10	1	24.2	3.6	119	0.92	1.23	99.93	2.1	high	7.8	6.2	0.3	37	11.7
PS	65	M	10	1	22.7	3.9	139	1.1	0.79	101.4	1.8	high	9.9	4.2		25	8.1
PS	60	F	7	1	25.3	3.7	154	1.37	0.88	159.4	1.1	high		4.9	0.07		11.2
PS	52	M	6	1	21.3	4.1	157	1.62	0.58	94.71	1.6	high	8.2	4.6	0.13	29	9.1
PS	66	F	5	1	20.3	3.7	178	1.35	1.03	120.2	1	high	9.8	2.5	0.1	24	10.8
PS	85	F	5	1	21.9	3.7	153	1.84	1.1	111.2	1.8	high	11.5	6.2	5.48	25	9.8
PS	58	F	5	1	19.4	3.9	150	1.43	0.82	84.53	2.1	high		3.5	0.31	24	10.8
PS	66	F	4	1	19.3	3.6	138	1.27	0.93	135.9	2.1	high	10.3	3.6	0.12	28	10
PS	76	F	2	1	19.6	3.6	94	1.46	1.08	127.7	1.3	high	7.9	6.4	0.5	29	10.3
PS	47	M	2	1	24	3.7	209	1.27	0.77	78.27	1.5	high	8.6	3.9	1.3	31	10
PS	74	M	2	1	22.2	3.8	173	1.73	0.85	108.1	1.3	high	9.6	4.5		26	10.1
PS	47	F	2	1	28.4	3.9	124	1.35	0.85	81.67	2.1	high	9.6	6			10.9
PS	55	F	2	1	21.3	4.1	182	1.16	0.9	94.04	2.1	high	8.2	6.7	0.2	33	11.5
PS	49	F	4	1	23.3	4	133	1.09	0.62	86.11	1.6	high	8.3	2.7	0.1	15	9.3
PS	45	M	20	1	24.7	4	140	1.61	0.59	63.27	1.5	high	7.6	5.2	0.11	27	9.8

PS	64	F	14	1	21	3.7	215	1.3	0.85	91.9	1.5	high	9.2	4.1	0.27	53	10.8
PS	69	M	4	1	23.8	3.8		1.43	0.89	104.5	1.3	high	8.4	5.4	0.15	24	9.3
PS	51	M	4	1	18.8	3.9	150	1.27	0.9	95.25	1.3	high	8.7	4.6	0.1	25	8.1
PS	57	M	14	1	19.8	3.7	147	1.21	0.98	81.72	2.1	high	8.8	8.7	0.6	31	10.4
PS	59	M	6	1	27.5	3.6	126	1.37	0.82	108.1	2.1	high	8.9	7.2	0.1	21	9.9
PS	68	F	14	1	22.5	3.6	116	1.42	0.8	136.4	1	high	9.7	2.8	0.1	29	8.9
PS	58	M	13	1	21.8	3.3	156	1.89	0.75	93.75	1.8	high	8.9	5.4	0.4	31	10
PS	70	M	3	1	23.5	3.7	141	1.24	0.73	103.2	1.6	high	8.2	3.4	0.1	18	10.3
PS	60	F	25	1	22.7	3.2	114	1.4	0.84	97.72	1.8	high	8.9	5.3	1.7	20	14.2
PS	71	F	11	1	23.5	3.3		1.05	0.86	117.4	2.1	high		7	0.1	30	12.5
PS	53	F	2	1	19.4	2.2	142	1.64	1.02	104.6	1.3	high	8.7	3.8	0.7		7.9
PS	53	M	2	1	23.6	4.1	131	2.08	1.13	127.8	1.6	high	9.3	4.3	0.08		9
PS	68	M	5	1	23.8	3.7	156	1.91	0.91	89.26	1.5	high		3.6		23	10
PS	73	M	12	1	14.6	3.4	219	2.02	0.85	97.61	1.8	high	10.3	4.4	0.2		11.1
PS	77	M	6	1	20.6	2.1	162	1.35	0.47	53.1	1.5	high	8	6.4	0.18	34	10.1
PS	61	F	3	1	29.6	3.3	161	1.6	0.78	31.72	2.1	high	10.1	4.1	1.6	36	10.6
PS	79	F	2	1	22.9	3.6	121	1.72	0.73	112.9	1.5	high	8.2	5	0.08	25	8.5
PS	37	M	18	1	21.4	4.1	169	1.39	1.19	110.6	1.3	high		4	0.06	28	10.9
PS	50	F	9	1	19.7	4.1	152	1.34	0.89	120.5	1.8	high	9.8	6.2		18	11
PS	59	M	8	1	19.3	4.2	189	1.71	0.7	126.9	2.1	high	8.1	5.2		17	10.6
PS	51	F	7	1	24.1	5.2	125	1.43	0.91	125.7	2.1	high	9.1	6.8	0.1	23	10.9
PS	60	M	7	1	17.3	4.1	160	1.39	1.11	75.88	1.5	high	10.5	5.7			10.2
PS	52	F	6	1	23.4	3.6	150	1.95	0.86	120.2	1.3	high	10.1	4.2	0.05		10.3
PS	79	M	2	1	20.5	3.8	157	2.37	0.9	75.77	1.3	high	9.1	5.7	0.05		9.6
PS	42	F	2	1	27.3	3.5	187	1.62	0.93	81.73	1.3	high	8.3	5.3	0.05		10.4
PS	56	M	10	1	20.9	3.7	204	1.66	0.61	88.36	1.8	high	9	4.7		35	10.6
PS	76	F	14	1	18.9	3.6	176	2.09	0.65	126.7	1.3	high	9.1	4	0.19		
PS	69	M	2	1	23.4	4.1		1.14	0.67	89.17	2.1	high	9.6	7.2	0.14	27	14.5
PS	79	F	2	1	20	4	137	1.27	1.03	94.96	2.5	high	9.6	5.8	0.2	33	10.1
PS	76	F	7	1	18.5	4.3	165	1.27	0.79	122.8	1.5	high	9.1	6.1	0.04	25	11.9
PS	74	F	6	1	19.5	3	132	1.67	1.11	129.5	1.5	high	9.1	3.7		27	11
PS	43	M	20	1	18.7	4.3	142	1.51	1.28	118	1.5	high	9.8	8	0.3	29	9.8
PS	65	F	17	1	15.5	3.4	129	1.61	0.86	96.53	1.6	high	8.9	4.5	0.05		10
PS	55	M	17	1	20	3.9	129	1	0.84	119.4	1.8	high	8.5	6.5	0.12	25	11
PS	76	M	15	1	18.7	3.7		1.37	0.97	118.4	1.8	high	10.4	3.6	0.1	22	9.9
PS	72	M	12	1	22.9	3.7		1.26	0.85	99.94	2.1	high	10.2	6.8		24	9.8
PS	63	F	9	1	27.6	3.8	187	2.23	1.23	119.9	1.6	high	10.4	5	1.08	23	11.9
PS	71	M	8	1	20.6	3.7	132	1.92	1	106.2	1.3	high	8.5	4.7	0.05		11.3
PS	55	F	6	1	18.1	3.9	159	1.72	0.91	112.2	1.5	high		4.2	0.23		9.7
PS	65	F	5	1	18.9	3.8	162	1.93	0.81	94	1.3	high	8.6	4.2	0.05		9
PS	57	M	3	1	18.7	3.4	157	1.42	1.06	73.71	1.8	high	8.1	3.7	0.1	10	
PS	69	M	3	1	26.1	4.4	113	1.79	0.73	72.32	1.8	high	8.7	3.3	12.9	30	10.7
PS	67	F	3	1	17.5	3.8	99	1.5	0.75	108	2.1	high	9.1	7.4	0.05	30	11.9
PS	72	M	2	1	23	3.6	132	1.53	0.95	93.85	1.5	high	8.3	7.1		31	10.8
PS	60	M	2	1	19.3	3.9	164	1.31	1.01	62.6	1.8	high	9.7	4.7	0.02	26	10.9
PS	52	F	2	1	27.2	3.3	157	1.25	0.85	44.83	2.1	high	7.9	3.7	0.46	25	10.5
PS	63	F	29	1	23.9	3.4	154	1.5	0.96	100.7	1.9	high	9	5.1	0.21		9.7
PS	63	F	29	1	17.2	3.5	205	1.63	1.04	105.8	1.3	high	8	3.9	2.33		10.4
PS	62	M	29	1	19.9	3.3	182	1.56	0.63	104.6	1.5	high	10.3	4.7	0	23	10.8
PS	71	M	28	1	17.8	3.8	128	1.54	0.91	100.9	1.9	high	8.4	6.8	0.1		11
PS	69	F	26	1	16.9	3.8	217	2.33	0.88	81.56	1.6	high	8.6	5	0.38		10.7
PS	59	F	25	1	19.3	4	175	1.59	0.99	137.4	1.9	high	8.6	4.9	0.09		9.5
PS	60	M	24	1	22.9	3.6	201	1.8	0.76	133	1.3	high	9.1	6.1	0.25		11.2
PS	59	M	24	1	21	4.3	166	1.38	0.87	103	1.5	high	9.4	4.7	0.03	24	10.4
PS	51	F	26	1	14.7	3.8	172	2.07	0.72	85.54	1.6	high		5.6		27	7.7
PS	48	F	23	1	19.1	4.3	151	1.55	1.03	105.6	1.8	high	8.9	3.5	0.04	25	11.5
PS	43	M	10	1	21.9	3.7	150	1.48	1.03	92.42	2.1	high	8.7	5.3	0.05	26	11.2
PS	57	M	22	1	18.6	4	196	2.26	0.86	91.52	1.3	high	8.8	4.3	0.05		10.7
PS	77	F	22	1	20.1	4.2	183	1.64	0.76	98.32	1.5	high	9.3	6.2	0.12	31	12.1

PS	68	F	20	1	21.1	4	118	1.39	0.89	124	1.5	high	9.2	5.9	0.02	32	11.7
PS	56	M	20	1	19.7	4.3	138	0.85	0.84	113.6	1.3	high	8.8	6.8	0.05		8.4
PS	54	M	20	1	20.3	3.8	144	2.01	0.81	103.1	2.1	high	10	5.1		25	11.7
PS	55	M	18	1	23.5	3.7	85	1.51	1.08	113.2	2.1	high	7	4.3	0.39	26	10.1
PS	49	M	18	1	17.5	4.3	171	1.86	0.74	129	1.3	high	9	6.6	0.1	32	10.5
PS	48	M	17	1	20.4	3.9		1.06	0.93	109.2	2.1	high	8.7	5.5	0.05	50	11.4
PS	49	M	14	1	20.9	4.1	187	2	0.79	109.1	1.8	high		5.4		27	11.2
PS	59	M	13	1	20.8	4.1		1.78	0.83	128.8	2.1	high	9.1	3.4			10.8
PS	51	F	12	1	19.7	4.1	186	1.48	0.97	117.3	1.5	high	9.2	5.8	0.05	27	10.5
PS	59	M	12	1	21.8	3.9	144	1.45	0.93	136.4	1.5	high	8.1	5.8	0.17	17	11.1
PS	54	M	11	1	22.9	4.4	166	1.03	0.97	121.3	1.3	high	8.8	5.4	0.04	25	10.2
PS	56	M	10	1	25.5	3.8	97	1.34	1.1	102.7	1.8	high	8.9	5.6	0.2	24	9.7
PS	61	F	9	1	19.2	3.8	147	1.54	0.82	116.4	2.5	high	9.4	5.4	0	23	13
PS	60	M	15	1	19.6	3.7		1.1	1.33	104.3	1.8	high	8.7	7.3			8.3
PS	33	M	9	1	21.4	3.7	121	1.34	1.05	105.5	1.8	high	9.6	3.6	0.3	30	10.1
PS	36	M	15	1	19.8	4.4		1.21	1.09	98.14	1.6	high		5.6		34	10.7
PS	70	M	8	1	21.6	3.9	114	1.53	1.13	136.8	1.8	high		7.3			11.1
PS	66	M	8	1	21.1	3.9		1.76	0.96	100.5	2.1	high	9.3	5.9	0.45	28	10.6
PS	75	F	7	1	27	3.7	99	1.08	0.74	87.5	1.5	high		4.3	2.33	44	10.3
PS	63	M	7	1	23.9	3.8	153	1.67	0.91	155.6	1.5	high	10.1	4.8	0.05	27	10.7
PS	57	M	7	1	22	4.2	147	1.67	1.07	122.3	1.5	high	9.6	6.2			9.7
PS	64	F	6	1	18.5	3.9	96	1.26	0.99	142.5	2.1	high	9.5	7.4	0.07	20	10.8
PS	72	M	6	1	22.1	4	143	1.48	1.03	108	1.5	high	9.2	5.1	0.1	19	12.1
PS	52	M	6	1	19	4.2	128	1.36	0.9	132.6	1.5	high	9.8	4.1	0.01	24	11.8
PS	67	F	6	1	23.4	4.1	197	1.61	0.91	152.1	1.6	high	9.4	5.9	0.05	29	9.6
PS	57	F	5	1	26.4	3.5	140	1.35	0.7	94.75	2.1	high	9.2	6.1			10.4
PS	51	M	4	1	20.1	4.7	174	1.24	0.71	101	1.1	high	8.9	5	0.2	21	9.1
PS	30	M	6	1	19.9	4	139	1.54	0.97	92.64	1.8	high		5.4			11.7
PS	73	F	4	1	20.1	3.9	193	1.58	0.84	87.25	1.5	high	8.5	6.2			9.7
PS	66	M	3	1	25.3	4.1		1.49	0.79	93.79	2.5	high	9.3	4.5	0.19	25	13.3
PS	67	M	3	1	23.3	3.9		2.09	0.82	53.97	1.5	high	9.5	5.7	0.2	27	8.7
PS	78	M	3	1	19.1	3.2		1.58	0.71	93.62	1.3	high		5.1	0.53	25	9.9
PS	71	M	3	1	18.1	4.1	107	1.62	0.7	56.44	2.1	high	8.2	4.6			7.8
PS	70	M	2	1	19.9	3.3	128	1.25	1.03	125.4	1.5	high	8.3	6.2	0.06	29	11.1
PS	55	M	2	1	20.6	3.8	84	1.26	0.83	118.3	1.5	high	9.2	6.8	3.5	34	9.3
PS	57	F	4	1	22.4	3.9	115	1.18	0.95	84.23	1.8	high		6			13.1
PS	61	F	23	1	24.1	3.7	191	0.95	0.83	129.9	1.3	high	10.1	6.7	0.19	26	9.7
PS	46	F	33	1	18.5	3.9	199	1.23	1.18	111.9	1.9	high	8.1	6.1	0.07	25	11.3
PS	69	F	14	1	17.5	3.9	155	1.63	0.97	117.9	1	high	8.8	7.3	0.1	25	10.4
PS	67	F	14	1	21.5	3.9	141	1.64	0.89	127.3	1.5	high	9	5.3	0.36	33	12.8
PS	72	M	6	1	23.9	3.7	128	1.55	0.85	120.6	1.5	high	8.6	4	0.07	20	7.8
PS	60	M	5	1	23	3.7	201	1.2	0.97	81.28	1.3	high	10.9	5.1	0.22	22	9.7
PS	65	M	2	1	23.6	3.6	156	2.13	0.84	128.7	1.5	high	8	4.7	0.12		10.5
PS	60	F	3	1	23.1	3.4	132	1.64	0.79	70.41	1.5	high	8.3	5.9		36	11
PS	35	M	4	1	22.4	4.4	115	1.52	0.88	103.8	1.6	high	10.3	4.8	0.04		11
PS	73	F	2	1	18.4	3.3	114	1.04	0.88	64	1.3	high	9	4	0.1	52	10.2
PS	73	M	7	1	21.8	3.6	141	1.33	0.92	135.9	1.6	high	9.4	5.3		28	10.5
PS	49	M	8	1	22.1	4.2	157	1.51	0.92	122.4	1.5	high	9.9	8.5			9.2
PS	37	M	3	1	22.1	3.8	181	1.44	1.05	99.18	1.6	high	10.1	3.5	0.07	29	10.7
PS	69	M	5	1	26.5	3.1	139	1.41	0.69	81.18	1.5	high	9.1	4.1	0.15	16	11.6
PS	60	M	2	1	21.3	4.2	210	1.71	1.27	96.8	1.6	high	10.1	6.7	2.13	29	8.6
PS	51	M	2	1	22.2	3.8	135	1.35	0.98	107.9	1.5	high		5.8	0.25	29	10.3
PS	58	M	6	1	22.2	4.4	248	1.51	0.32		1.8	high	9.4	4.9	1.61	44	9.9
PS	72	F	4	1	26.8	3.4	136	1.47	0.78	109.8	1.1	high	8.6	3.8	0.6		12
PS	76	F	6	1	18.1	3.6	135	1.54	0.73	110.4	2.1	high	8.2	6.9	0.5	28	10.2
PS	59	F	25	1	18.2	3.3	142	0.95	0.88	126.3	1.5	high		4.9	0.16	24	11
PS	57	F	15	1	21.6	3.6		1.88	1.06	146.4	0.9	high	8.8	3.8			10.7
PS	46	F	13	1	17	4	277	1.22	0.99	102.5	1.6	high	9.7	4.8	0.06	20	10.5
PS	70	M	11	1	19	3	143	1.27	0.77	126.1	1.5	high	8.5	7.9		24	11.5

PS	72	F	15	1	14.6	3.9	138	1.01	1.15	108.8	2.1	high	9.2	4.8	0.2	38	9.1
PS	51	M	8	1	27.5	4.5	126	1.25	0.83	97.84	1.3	high	10.6	4.3	0.1		9
PS	60	M	24	1	20.9	3.9	115	1.19	1.16	115	2.5	high	8.9	6.1	0.4	28	11.6
PS	58	M	22	1	21.9	3.6	168	1.98	1.01	84.03	1.5	high	9.1	3.8	0.2	33	10.9
PS	67	M	3	1	20.8	3.9	104	1.23	0.88	107.7	1.6	high	8.3	4.5			
PS	37	M	11	1	20.4	4.5	150	1.33	0.91	116.4	1.5	high		4.5	0.08	21	12.2
PS	30	M	6	1	22	4.4	93	1.38	0.89	120.8	1.9	high	7.6	2.7	0.27	25	9.5
PS	66	F	15	1	17.5	3	152	1.77	1.11	113.9	2.1	high	10	6.4	0		12.7
PS	36	M	5	1	20.7	4	167	1.47	0.9	117.7	1.8	high	9.6	4.9	0	12	10
PS	58	F	24	1	21.2	3.5	118	1.74	1.03	112.2	1.3	high	8.8	4.5	0.19	32	7.7
PS	59	M	17	1	21.7	3.5	146	1.15	0.84	145.3	1.9	high	9.8	5.8	0.06	24	11.4
PS	58	M	15	1	21.9	3.1	183	1.88	0.8	129	2.1	high	9.5	6.8	0.1	24	11.8
PS	35	M	12	1	22.4	4.3	160	1.44	0.73	121.7	1.6	high	9.5	3.7	0	25	11
PS	51	M	6	1	26.1	4	126	1.14	0.96	97.85	1.3	high	7.9	3.3	0.01	23	9.3
PS	39	M	11	1	19.9	4.2	182	1.68	0.99	90.87	1.3	high	9.1	3	0.1	33	9
PS	44	M	8	1	23.7	4.6	184	0.83	1.19	112.5	2.1	high	9.9	4.7	0.1	28	8.7
PS	69	F	5	1	22.5	3.5	145	1.21	0.48	95.67	2.1	high	8.5	5.6	0.22		10.3
PS	53	M	2	1	26	3.8	140	1.86	0.91	99.12	1.3	high	11.6	3.9	8.27	26	9.4
PS	58	M	13	1	23.7	4	217	1.48	0.9	113.1	2.1	high	9.4	5.2			8.8
PS	44	M	6	1	24.4	4	134	1.13	0.83	132.5	1.6	high	8.9	4.1	0.11	17	11.3
PS	68	M	3	1	23.7	3.5	125	1.39	0.83	108.2	1.6	high	8.5	4.9	0.4	31	11.3
PS	70	M	3	1	19.6	3.6	143	1.49	0.77	117.6	2.1	high	8	4.6	0.59	20	8.5
PS	68	F	17	1	19.2	3.7	190	1.78	0.8	121.8	1.5	high		5			11
PS	51	M	10	1	24.6	3.9		1.33	1.15	95.72	1.6	high	7.8	3.3	1.18	39	9.6
PS	53	M	9	1	19.6	4.1	118	1.67	0.97	124.5	1.6	high		6	0.2	28	9.7
PS	65	F	7	1	24.1	3.6	148	1.01	0.78	142	2.1	high		6		33	11.5
PS	64	M	18	1	18.5	4	136	1.79	0.94	107	1.5	high	9.5	4.7	0.3	29	10.5
PS	72	F	16	1	22.7	3.8	111	1.53	0.86	81.42	1.5	high		1.2	0.9	36	9
PS	61	M	14	1	22.4	4.1	127	1.09	0.74	94.79	1.5	high	9.6	5.9	0.81	29	10.2
PS	64	M	19	1	20.6	3.6		1.84	0.94	104.9	2.1	high	8.7	4.7	0.09	30	9.3
PS	44	M	10	1	23.8	4.7	130	1.28	0.67	120.6	1.3	high	8.4	4.6	0.04	34	10.6
PS	78	F	6	1	19.5	3.5	120	1.82	0.51	60.17	1.8	high	9.2	4.4	0.86	27	8.9
PS	54	M	6	1	20.7	3.6	154	1.37	0.82	97.72	1.8	high	8.6	5.5	0.13	23	9.8
PS	72	M	5	1	25.8	3.5		1.34	0.74	77.87	0.6	high	10.1	6.3			11.8
PS	61	M	4	1	17.9	3.7	104	1.16	0.91	84.56	1.5	high	9	6.3	0.07	32	8
PS	77	F	4	1	25.1	4	152	2.03	0.62	68.38	1.5	high	8.7	4.2	0	43	11
PS	74	M	3	1	21.7	4.1	153	1.27	0.79	89.39	1.5	high		3.5			8.9
PS	66	M	2	1	20.2	3.1	155	1.45	0.9	81.65	1.3	high	8.5	5.7	0.2	26	11.5
PS	87	F	2	1	19	2.9	101	1.53	0.67	77.21	1.8	high	8.9	3.9	0.01	30	8.8
PS	65	F	2	1	15.8	3.5	200	1.69	0.83	60.09	1.5	high		5.4			10.9
PS	58	M	2	1	23.1	3.7		1.44	0.75	100.8	2.1	high	9.7	4	0.2	27	11.8
PS	60	M	6	1	19.9	4.1	119	1.32	1.11	125.8	1.3	high	9.2	4.8	1.6	20	10.3
PS	71	M	8	1	22.4	3.8	137	1.52	0.87	89.15	2.1	high	9.3	5.9	2.95	28	9.9
PS	74	M	6	1	18.8	3.6		1.03	0.98	93.02	1.9	high	10.2	4.2			11.1
PS	81	F	9	1	22.4	3.3		1.29	1.01	120.9	1.5	high	8.8	2.9		24	9.4
PS	71	F	6	1	22.4	3.2	108	1.23	0.72	101.6	1.5	high	8.6	4	1.9	31	8.3
PS	59	M	2	1	23.2	3.1	93	1.12	0.84	87.49	1.3	high	7.4	1.8	2.77	31	7
PS	54	F	35	1	18.8	3.7	141	1.53	1.02	80.26	1.6	high	9.8	5.3	0.1	18	9.3
PS	70	M	32	1	14.9	3.8	164	1.86	1.05	76.73	1.9	high	8.3	4.3	0.1	25	9.8
PS	59	M	30	1	20.8	3.8	190	1.81	0.91	110.1	1.9	high	9.7	5.5	0.3	24	11.3
PS	52	F	30	1	22.5	4.1	179	1.33	1.01	124.7	1.6	high	8.2	5.6	0.1	21	10.7
PS	67	M	29	1	18.6	3.5	164	1.14	0.86	105.1	1.6	high	10	4.2	0.1	20	10.9
PS	40	F	28	1	19.7	3.3		1.4	0.76	106.4	1.6	high	9.3	6.7			10.7
PS	62	F	27	1	22.6	3.8	211	1.53	1.06	111.8	1.6	high	8.3	6.4	0.1	21	10.6
PS	53	F	25	1	18.9	4.2	166	1.18	1.07	122.5	1.4	high	9.1	6.5	0	29	11.8
PS	61	F	24	1	18	4		1.12	1.15	91.72	1.9	high	9.7	6.6			13.6
PS	60	M	24	1	18.6	3.5		1.24	1.22	122.6	1.6	high	9.4	4.3			10.1
PS	60	M	23	1	21.4	3.3	265	1.48	1.06	107.6	1.9	high	8.4	5.6	0.1	25	10.2
PS	63	M	21	1	19.8	3.4	175	1.75	0.78	83.38	1.6	high	9.2	4.1	0.1	16	10.4

PS	65	F	20	1	18.9	4.6		1.51	1.17	116.2	1.6	high	9.7	6.1	0	24	10.9
PS	49	F	19	1	21.3	4.5		1.66	0.82	108.4	1.6	high	8.7	5.3	0	19	10.1
PS	61	F	19	1	22.3	4.1		2.05	0.99	121.8	1.6	high	8.8	4.4	1.2	24	9
PS	55	M	17	1	19.9	3.6	146	1.17	0.9	96.43	1.8	high	8.8	4.9	0.05	16	10.6
PS	68	M	16	1	18.2	3.6	120	1.62	0.79	76.58	1.1	high		2	0.2	24	10.6
PS	55	M	16	1	22.7	3.7	181	1.51	1.04	113.4	1.8	high	8.3	5.2			9.2
PS	46	M	15	1	22.4	3.8		0.9	1.01	112.5	1.9	high	8.9	6.5			10.7
PS	58	F	14	1	17.2	3.5	180	1.38	0.83	75.54	1.1	high	9.5	3.4	4.9	32	10.8
PS	54	M	13	1	22.7	4		1.27	1.22	100.7	1.6	high	9.7	4.3			11.5
PS	59	M	13	1	19.4	3.6		1.61	0.92	109.6	1.9	high	9.9	6.8			11.5
PS	71	F	10	1	18.4	4		1.57	1.04	116.7	1.6	high	9	5			11.2
PS	63	F	10	1	17.8	3.9	173	1.55	0.95	148.3	1.8	high		5.4	2.26		11.1
PS	52	M	10	1	31.3	4.2	154	1.48	0.81	111.9	1.5	high	9.3	4.4		32	10.3
PS	50	F	10	1	20.6	4.2		1.51	1.15	132.1	0.9	high	8.2	4.2			11.3
PS	59	M	10	1	22.7	4.2		1.21	1.01	106	1.9	high	9.9	3.6			13.9
PS	72	M	10	1	15.2	4.4		1.28	0.92	132.7	0.9	high	8.9	5.5			11.5
PS	54	M	8	1	23.8	4.5		1.7	1.22	95.66	1.3	high	9.3	4.9	0.9		12.7
PS	54	M	8	1	23.1	3.9	134	1.48	0.94	88.9	2.1	high	9.6	4.6	0.03	19	10.5
PS	65	F	7	1	20.7	4.1		1.38	0.98	115.1	1.6	high	9.3	5.5			11.6
PS	68	M	7	1	29.8	4.4		1.27	1.12	145.9	1.6	high	9.2	4.2			10
PS	60	M	7	1	22.5	3.4		1.49	0.78	101.6	1.8	high	8.5	3.4	0.35	29	10.4
PS	68	M	7	1	23.7	3.9	156	1.41	1.02	104.8	2.1	high		9		33	
PS	55	M	6	1	25.8	4.1	148	1.35	1.05	133.2	2.1	high	7.9	5.9	0.21	18	9.3
PS	73	M	6	1	20.5	3.4	124	1.12	0.64	88.58	1.3	high	9.3	5.6		22	11.1
PS	45	M	6	1	17.7	3.7	146	1.45	0.84	100.4	1.5	high	8.1	3.5	0.17	30	9.6
PS	47	F	6	1	26.4	3.8	123	1.28	0.71	103.3	1.5	high	8	6.9	0.2	23	8.8
PS	65	M	6	1	26.2	4.1		1.3	0.94	104.8	1.9	high	9.3	5.2			11.4
PS	73	F	6	1	23.5	3.5	128	1.63	0.92	92.49	1.5	high	9.7	3.3	0.1	22	10.2
PS	67	M	5	1	21.8	3.8		0.86	0.88	107.6	1.8	high	9.4	2.9	4.06		13.1
PS	51	F	5	1	38.6	3.8	143	1.43	0.96	99.06	1.8	high	9.3	4.8	0.07	41	11
PS	70	M	5	1	30.6	3.9	159	1.11	0.79	124.2	1.8	high		5.6	0.05	33	9.1
PS	59	M	5	1	24.8	4	130	1.5	0.79	91.17	1.3	high	9.3	8.7	0.1	27	11.9
PS	53	M	5	1	23.2	4.1		1.53	0.77	95.35	1.5	high	7.9	3.8			10.6
PS	65	M	5	1	28.5	3.9		1.18	0.91	115.5	1.9	high	8.9	4.8			11.5
PS	43	M	4	1	18.7	4	147	1.63	0.73	78	1.3	high	8.6	3.9		22	8.2
PS	78	M	4	1	19.1	3.6	133	1.42	0.95	87.28	2.2	high	8.7	5.5	0.13	33	10.8
PS	62	M	4	1	24.2	3.7	162	1.3	0.88	115.4	1.5	high		5.3	0.13	31	9.4
PS	49	F	3	1	18.6	3.9		1.25	0.71	76.43	1.9	high	9	4			10.8
PS	65	F	3	1	20.5	3.5		1.37	0.57	73.17	1.9	high	9	5.5			11.3
PS	71	F	3	1	24.2	3.7		1.7	0.7	115.6	2.5	high	9.1	6.2		29	10.7
PS	53	M	3	1	27.6	4.1	115	1.26	0.84	77.41	1.5	high	9.5	7.2			12.4
PS	57	M	3	1	23.7	4.2		1.5	1.18	120.9	1.5	high	9.6	3.9	3.19	25	11.7
PS	54	F	3	1	23.6	3.9		1.48	1.06	118.6	1.6	high	8	3.9			12.4
PS	62	M	3	1	24.3	3.7	149	1.23	0.83	106.7	1.3	high		5.2	0.05	21	11.3
PS	46	M	3	1	25.2	4	106	0.94	0.85	115.9	1.5	high	8.7	4.8	0.6	29	10.9
PS	71	M	3	1	25.2	4.2	122	1.19	0.94	108.6	1.8	high	8.8	6.2	1.01	22	9.7
PS	68	M	3	1	21.8	3	232	1.02	0.72	65.35	2.1	high	7.5	9.3	0.7	29	11.2
PS	61	M	3	1	30.1	4.2	196	1.29	0.79	136.7	1.1	high	8.7	4.7	0.2	17	9.4
PS	71	F	2	1	20.5	3.9	123	1.46	1.26	105.2	1.5	high	7.3	6.3	0.48	45	10.4
PS	72	M	2	1	21.2	3.7	119	1.17	0.78	145.6	2.1	high	8.9	7	1.45	22	10.5
PS	71	M	2	1	23.2	3.5	116	1.02	0.74	96.29	2.1	high		6.8	0.06	27	10.7
PS	54	M	2	1	20.4	4.4	170	1.86	0.87	71.33	2.1	high	8.8	5.6	0.14	31	9.4
PS	40	F	2	1	29.1	4	205	1.66	0.99	62.72	1.3	high	9.2	6.2	0.28	33	11.3
PS	60	F	2	1	25.2	4.4	164	1.32	0.69	59	1.5	high	8.7	5.3	0.3	32	8.9
PS	78	M	2	1	24.9	4.2	147	1.27	0.67	62.9	1.5	high		4.9	0.18	25	10.4
PS	78	M	2	1	23.1	3.7	150	1.24	0.78	100.6	2.1	high	8.9	5.7	0.1	21	8.5
PS	46	M	17	1	21.2	4.3		1.79	1.03	122.9	1.6	high	10	5.8			11.1
PS	55	F	8	1	25.1	3.3	161	1.25	0.79	95.3	1.8	high	8.6	4	0.04		11.1
PS	77	F	22	1	17.4	3.8		1.35	1.04	136.5	1.6	high	9.3	5.4	0.01	29	11

PS	54	F	10	1	21.9	2.9	131	1.4	0.99	108.2	2.1	high		10			7.9
PS	60	M	8	1	18.2	3.5	148	1.47	0.76	113.5	1.5	high	8.2	3.9	1.7	31	10.4
PS	80	F	5	1	18	3.5	192	1.22	1.04	109	1.5	high	9.5	4.5	0.1	16	10.6
PS	78	F	4	1	23.6	3.8	212	1.92	1.09	138.3	1.5	high	8.9	6.9	0.1	26	11.6
PS	68	F	4	1	20.6	3.8	198	1.5	1.12	117.2	1.8	high	8.9	4.2	0	32	10.1
PS	70	F	4	1	17	3.6	215	2.14	0.78	102	1.5	high	8.5	4	0.4	33	10.4
PS	59	F	13	1	20.5	3.4	173	1.43	1.02	106.7	1.8	high	9.3	6.4	0.03	24	10.1
PS	57	F	27	1	21.5	3.8		1.77	1.09	129.1	1.3	high	10.2	4		30	9.1
PS	53	F	13	1	17.9	3.7	139	1.21	1.15	109.1	0.8	high	8.2	5	0.06	19	10.8
PS	34	M	2	1	23.7	4.4	144	1.3	0.68	83.68	2.1	high	9.4	6.8			10.9
PS	68	F	21	1	20.1	3.7	200	1.17	0.93	96.65	1.8	high		2.4	0	23	9.1
PS	61	M	7	1	20.9	4.3	110	1.2	0.68	98.33	1.5	high		5.3	0.22	34	8.9
PS	78	M	6	1	21	3.4	210	1.37	1.14	120.2	1.1	high	9.4	5.7	0.02	20	9.7
PS	77	M	3	1	21.4	3.8	187	1.33	0.72	113.7	1.8	high	10	4.4	0.08	29	12
PS	69	M	7	1	18.2	3.9	193	1.26	0.81	123.1	1.5	high	8.8	8.9	0.51	28	12.9
PS	53	M	8	1	19.4	4.4	103	1.42	0.88	81.27	1.8	high		4.6			10.4
PS	67	F	24	1	18.9	3.8		0.98	1.1	107	1.9	high	9.9	6.6			10.9
PS	60	M	17	1	21	3.9	233	1.63	0.97	117.2	1.3	high	8.2	5.5	0.16	26	10.7
PS	59	M	14	1	20.8	3.7	160	1.26	0.97	116.9	1.3	high	10.9	5.6	0.02	26	10.2
PS	66	M	14	1	20.9	3.7		1.41	0.78	93.79	1.9	high	10.2	6.7			12.3
PS	32	M	6	1	24.5	3.6	127	1.83	0.7	107	1.5	high	9.3	6.1	0.05	43	10.5
PS	76	F	4	1	22	3.8	144	1.15	1.01	96.81	2.1	high	10	5.6	0.15		9.3
PS	48	M	4	1	30.1	3.8	162	1.26	0.94	89.86	1.5	high	9.3	5.3	0.06	28	12.4
PS	59	M	2	1	18.2	3.9	156	1.82	1.48	94.04	1.6	high	8.7	4.3	0.49	36	10
PS	65	M	11	1	20.4	4.1	148	1.32	0.97	126.3	1.5	high		5.9			10.6
PS	67	F	14	1	20.6	3.9	171	1.24	1.28	117.3	1.8	high		4.8			11.8
PS	65	M	2	1	29.9	3.6	127	1.2	0.83	71.77	1.8	high	5.9	10.5		30	10.9
PS	62	M	11	1	18.7	4		1.63	1.2	117.9	1.6	high	9.8	7.3			11.2
PS	90	F	10	1	20.1	3.5	162	1.57	0.97	104.5	1.8	high	10.8	6.1		26	11.7
PS	56	F	9	1	17.5	4.1	176	1.33	1.06	105.2	1.8	high	8.8	4	0.05	9	9.6
PS	59	M	7	1	26.6	4	317	1.99	1.21	107.4	2.1	high		4.5	0.05		12
PS	45	M	6	1	21.9	4.2		1.65	1.05	117.2	1.8	high	8.8	3.8	1.55	31	10.5
PS	58	M	2	1	20.8	3.8	154	1.82	0.89	88.69	1.3	high	8.9	5	0.05	25	9.3
PS	71	M	22	1	20.5	3.9	158	1.16	1.11	116.3	1.8	high	8.2	5.6		30	9.7
PS	61	M	14	1	19.4	4		1.14	0.94	98.57	1.6	high	9	1.3			8.8
PS	71	F	21	1	21.1	3.6	146	1.16	1.15	94.76	1.8	high	8.5	6.4	0.96	29	9.9
PS	71	F	14	1	17.4	3.3	236	1.41	1.1	122.2	1.5	high	8.8	4	0.09	27	11.9
PS	41	F	9	1	17.2	3.1	171	1.42	0.81	118.1	1.8	high		6	0.1	14	9.6
PS	65	M	11	1	16.4	3.1	172	1.48	0.69	68.51	1.6	high	8.8	6.8	0.06	24	10.1
PS	64	F	21	1	17.5	4.1	132	1.66	1.27	113.9	2.1	high		7.6			9.4
PS	60	F	17	1	16.7	4	140	1.66	1.15	107.8	1.5	high		5	0.08		10.1
PS	61	M	19	1	19.4	3.8		1.29	0.83	76.4	1.9	high	9.5	5.7			11.3
PS	68	M	4	1	19	4.2	107	1.34	0.92	126.2	2.1	high	11.3	7.5	0.05	26	11.6
PS	48	F	8	1	16.9	4	174	1.28	0.61	89.09	1.8	high	8.9	5.8	0.55	24	9.9
PS	41	M	9	1	17.9	3.7	121	1.58	0.8	109.3	1.6	high	8.1	5.1	4	17	11.8
PS	59	M	8	1	19.4	3.6	188	1.85	0.65	96.18	1.6	high	8.5	4	0.06	24	10.1
PS	61	M	27	1	21.6	3.6	114	1.3	0.82	115.3	1.8	high	9.7	4.7	0.05	31	11.7
PS	66	M	6	1	21.3	3.8	134	1.32	0.81	78.54	1.8	high	8.2	5.6	0.6	22	10.9
PS	50	M	4	1	24.9	4.3	134	1.33	0.94	38.09	2.1	high	8.4	5.6	0.14	35	10
PS	21	M	2	1	21	4.3	165	1.48	0.75	97.01	1.5	high	8.8	6.4	0.47	30	9.5
PS	55	F	9	1	19.6	4	135	1.38	1	99.7	2.5	high	9.3	9.6		22	12.1
PS	54	M	12	1	18.1	3.8	126	1.17	1.06	93.33	1.8	high	8	5.2	0.39	27	11.6
PS	68	M	11	1	18.8	4.2	210	1.5	1.39	87.52	1.5	high	9.8	5.3	0.56	32	10.9
PS	57	M	11	1	21.6	4	143	1.15	0.82	92.9	1.6	high	9.9	6.1	0.89	26	13.8
PS	55	M	4	1	23.5	4	145	1.23	0.96	145.5	1.8	high	9.3	5.1	0.29	27	11.2
PS	64	M	2	1	19.8	4.1	126	1.64	0.84	67.44	1.6	high	7.9	6.1	0.6	40	10.8
PS	44	M	11	1	21.4	3.9	156	1.35	0.56	92.68	2.1	high	8.2	5.8	0.22	28	12.3
PS	63	F	8	1	22.7	3.6	119	1.17	1.34	94.6	2.1	high		4.9	0.63	25	10.5
PS	59	M	2	1	20.9	3.7	113	1.75	1.05	125.3	1.6	high	8.3	2.6	0.05	24	11.5

PS	58	F	3	1	17.8	4.2	184	1.62	0.87	120.6	1.3	high		4.7	0.14	52	9.5
PS	72	F	30	1	20.7	3.5	108	1.79	0.87	76.81	1.3	high	9	5.9	0	23	10.5
PS	59	F	18	1	18.8	3.7	94	1.31	0.6	78.76	1.8	high	7.6	3.5	0.1	15	6.7
PS	67	F	14	1	21.7	3.6	195	1.6	1.29	108.3	1	high	8.6	9	0.1	24	11.2
PS	59	M	9	1	24.1	3.9	140	1.44	0.88	121.9	2.1	high	10.1	5.7	0.23	30	12.3
PS	64	M	8	1	20.1	3.5	240	1.43	0.87	100.8	1.5	high	9.1	5	0.35	24	11.4
PS	66	M	6	1	20.3	3.4	143	1.44	1.04	112.5	1.5	high		6.3	0	16	10.6
PS	63	M	3	1	13.9	3.4	162	1.31	0.82	70.86	1.5	high	8.6	4.8	0.24	31	10.8
PS	58	F	29	1	16.3	4.1	136	1.16	0.98	71.35	2.1	high	9	6.8	0.21	28	10.1
PS	79	M	5	1	19.9	4	177	1.63	1	103.6	2.1	high	9.2	5.4	0.11	33	10.2
PS	83	F	16	1	19.1	3.7	201	1.84	0.86	106.1	2.1	high	10.8	4.8	0.05	23	10.7
PS	70	M	6	1	17.9	3.5	153	1.45	0.83	108.5	1.5	high	9.5	5	0.1	27	9.1
PS	62	F	26	1	16.1	4	174	1.4	1.04	73.61	1.4	high	8.6	3.7	0.23	14	10
PS	71	F	20	1	19.4	3		1.69	1.06	116.4	1.8	high	9.9	5.6	0.2	31	12.6
PS	67	M	11	1	21.3	3.7	145	1.64	0.89	116	2.1	high	8.6	7	0.22		11.6
PS	58	M	9	1	23	3.7	170	1.42	1	128.5	1.8	high		4.9	0.04	26	10.1
PS	75	F	9	1	23.9	3.5	141	1.59	0.74	133.1	1.3	high	9.8	3.6			11.7
PS	72	F	8	1	21.6	3.1		1.56	0.86	97.39	2.5	high	9.1	5.1	0.01	23	10.7
PS	60	M	17	1	19.4	4.4	147	1.53	1.02	76.19	1.1	high	9.8	7.7	0	25	9.6
PS	59	M	7	1	17.5	3.8	121	1.72	1.55	139.8	2.1	high	8.7	3.8	0.05	41	9.7
PS	64	M	7	1	21.5	3.5	145	1.36	1.07	102.1	1.1	high	9	5.1	0.1	24	9.3
PS	68	M	5	1	17.5	3.4		1.39	0.8	97.1	2.5	high	9.6	6.7	0.4	31	9.8
PS	76	F	4	1	17.7	3.5	148	1.52	0.92	110.1	1.3	high	8.9	5	0.8		9.1
PS	83	M	4	1	23.7	3.4	128	1.52	0.76	108.4	1.8	high		4.7	2.65	26	10.6
PS	79	F	4	1	20.8	3.3	151	1.63	1.06	71.38	1.3	high		3.6	0.04	23	11
PS	73	F	3	1	20.7	3.2	177	1.78	0.63	72.69	1.8	high	8.5	4.7	0.99	17	9.8
PS	64	M	3	1	21.4	3.6	150	1.64	1.13	113.3	1.6	high	8.6	7.3		43	6.6
PS	82	M	3	1	19	3.1	107	1.68	0.6	135.5	1.3	high	8.4	4.6	0.2	38	12.6
PS	65	F	8	1	16.1	4	96	1.35	0.92	97.12	1.8	high	9.8	7		27	10.5
PS	85	M	3	1	18.4	4	131	1.46	1.27	83.91	1.3	high		5.5			11.5
PS	84	F	7	1	27.9	3.5	170	1.3	0.81	78.3	2.1	high	10.2	5.9		37	10.6
PS	70	F	16	1	17.3	3.8	134	1.37	1.23	105.6	2.2	high	8.7	4.6	0.8	21	11.6
PS	66	M	2	1	21.7	3.7	153	1.41	0.62	40.87	1.8	high	9.5	7.2	0.2	24	9.7
PS	76	F	6	1	21.2	3.9		1.28	0.67	89.66	1.5	high	9.5	5			10.3
PS	50	F	7	1	24.4	4.1		1.67	1.19	121.1	1.1	high	9	4.5	0.03		
PS	82	M	10	1	18.1	3.5		1.85	1.13	117.7	1.8	high	8.8	3.8	0.05	27	10.8
PS	49	M	27	1	19.3	4.1	209	1.59	0.88	33.51	1.3	high	9.1	5.2	2.6	35	11.2
PS	75	F	5	1	18.9	3.4	162	1.73	0.64	75.38	1.6	high		4.1	0.04	33	11.8
PS	56	F	3	1	22.2	3.6	120	1.22	0.77	81.94	1.8	high	9.5	5.5	0.91	31	8.6
PS	64	M	14	1	19.4	3.6	137	2.11	0.91	99.59	1.3	high	9	5	0.2	28	8.6
PS	59	F	8	1	18.2	3.8	147	1.41	0.83	108.1	1.8	high	7.9	5.5		20	11.3
PS	52	F	8	1	17.9	3.7	166	1.41	1.02	121.8	2.1	high	9.3	5.5	0.1	18	11.2
PS	87	M	5	1	25	3.9	149	1.18	1.16	133.2	2.2	high	6.8	6.4	0.45	39	6.7
PS	70	F	11	1	21.2	3.4		1.7	0.49	104.2	1.4	high	10.4	5.3			10.3
PS	78	F	3	1	18.2	3.8	123	2.2	0.85	81.14	1.6	high	9.1	3.9	0.05	23	9.2
PS	75	M	5	1	21.8	3.7	124	1.37	0.88	163.4	1.1	high	8.3	9.5	0.2	28	9.1
PS	60	M	30	1	21.2	3.4		1.41	0.97	63.04	1.6	high	9.3	5.5	0.1	26	9
PS	58	M	30	1	18.7	3.9	193	1.3	1.22	111.2	1.8	high	9.2	4.7	0.05	28	12.1
PS	59	F	18	1	17.5	3.4	121	1.58	1.29	80.49	1.8	high		8.5		39	8.5
PS	65	M	17	1	18.6	3.5	105	1.47	0.82	132.3	1.9	high		5.7	0.13		9.3
PS	58	M	14	1	22.1	3.7	181	1.47	1.23	117.4	1.3	high	7	8.1	0.2		11.2
PS	55	F	14	1	20.8	3.9	96	1.23	1.02	140.4	2.1	high	8.3	4.1	0.09	30	9.3
PS	39	F	12	1	18.1	4.1	159	1.46	1.15	116.8	1.9	high	9.4	7.2	0.15	25	10.4
PS	73	M	5	1	24.4	3.6	149	0.62	0.69	107.6	2	high	10.7	3.9	0.48	27	8.1
PS	75	F	4	1	19.6	3.5	212	1.13	1.01	100.9	1.6	high		6.3	0.24	23	9.9
PS	62	M	2	1	17.5	4.4	158	1.37	0.73	88.69	1.9	high	9.2	6.8	0.17	28	10.6
PS	55	M	33	1	21	3.9	177	1.22	1.17	90.56	1.8	high	8.5	4.1	0.1	21	11.5
PS	40	F	16	1	29.3	3.9	152	1.5	1.23	130.3	1.6	high	9.3	8.4	0.53	33	10.6
PS	54	F	11	1	16.7	4.4		0.91	0.95	111.9	1.5	high	9.2	0.8			9.6

PS	80	F	2	1	24.3	2.8	170	1.41	1.5	103.1	1.6	high	8.7	3.8	0.61	29	10.3
PS	65	F	16	1	16.8	3.4	152	1.46	0.85	128.2	1.6	high	9.1	8.1	0.02	29	10.9
PS	56	F	14	1	18.9	3.5	138	1.35	0.79	137.1	1.6	high	9.9	4.9	0.08	29	10.9
PS	69	F	3	1	19.3	3.4	99	1.15	0.78	126.5	1.8	high	9.1	3.5	0.05	35	11.1
PS	62	F	37	1	16.2	3.6	209	1.57	0.94	56.73	1.8	high	9.5	4.8	0.06	29	11
PS	66	M	30	1	22.8	3.7	152	1.02	1.08	105.9	1.9	high	8.7	5.2	0.02	27	11.1
PS	69	M	34	1	20.7	3.4	107	0.8	1.03	91.31	1.8	high	9.4	5.9	0.05	28	10.8
PS	63	M	25	1	18	3.5	80	1.11	1	132.1	1.6	high	8.7	8.3	0.09	26	11.5
PS	66	M	35	1	20	4	123	1.49	0.75	117.8	1.4	high	8.9	4.1	0.66	31	11.7
PS	56	M	21	1	22.5	3.7	130	1.43	0.98	144.1	1.8	high	8.8	5.6	0.13	22	9.9
PS	56	M	17	1	20.9	3.7	162	1.72	1.06	132.5	1.6	high	10.5	6.5	0	24	10.7
PS	81	F	2	1	24.6	3.4	170	1.51	0.72	142.1	1.6	high	9	6.4	0.11	42	11.4
PS	66	M	29	1	19.4	3.7	153	1.07	0.85	86.25	1.6	high	9	8	0.15	28	11
PS	54	F	11	1	16	4.3	110	1.34	1.16	113.8	0.8	high		5.1	0.11		11
PS	46	M	26	1	20.5	4	135	1.22	0.83	99.65	1.6	high		6	0.05	23	11.1
PS	55	M	31	1	19.9	3.7	123	1.37	0.93	101.6	1.4	high	9	6.2	0.25	29	10.2
PS	63	M	30	1	23	3.9	101	1.55	0.79	87.19	1.8	high	10.1	6	0		10.7
PS	70	F	29	1	22.6	3.9	151	1.34	0.81	139.9	1.9	high	9.6	4.5	0.02	26	
PS	65	M	27	1	21.4	3.5	146	1.31	0.94	106.2	1.4	high	9.9	6	0.1	14	11.7
PS	68	M	20	1	22.5	3.5	153	1.56	0.76	112.5	1.9	high	10.5	5.3		28	11.5
PS	56	F	20	1	20.5	3.6	108	1.31	0.85	96.62	1.6	high	9.2	5.1	0.03	27	9.4
PS	52	M	17	1	26	4.4	179	1.29	0.96	141.8	1.6	high	9.2	5.9	0.05	46	8.1
PS	57	F	12	1	18.6	4	118	1.57	0.84	98.71	2.5	high	8.5	5.9	0	25	9.3
PS	60	M	8	1	22.3	4	153	1.33	0.77	111.1	2.1	high	10.5	5.8	0	22	10
PS	62	F	6	1	16.4	3.1	162	1.61	0.86	70.94	1.6	high		3.5	0.29	33	9.5
PS	60	M	26	1	24.4	3.7	170	1.45	1.11	110.4	1.4	high	9.6	6.2	0.1		10.2
PS	73	F	23	1	16.8	3.4	176	1.42	1.14	113.4	1.8	high	8.9	6.9	0.23	23	10.4
PS	55	F	28	1	20	4	141	1.58	1.17	123.2	1.3	high	9.3	6.3		35	9.4
PS	64	M	18	1	23	3.7	170	1.37	0.96	112.7	1.3	high	10.5	3.8	0.03	23	9.4
PS	67	M	19	1	23.1	3.2	186	2.05	1.04	120.3	1.6	high	9	4.4		22	10.7
PS	82	F	5	1	19.3	3.1	146	1.23	0.75	86.49	1.8	high	7.9	6.9	0.34		12.8
PS	56	M	5	1	19.9	3.9	182	1.24	1.08	153.9	1.6	high	9.3	6.1	0.03	40	10.6
PS	55	F	26	1	19.9	3.9	130	1.4	1.03	112.6	1.9	high	10.3	6.1	0	29	10.1
PS	54	F	25	1	19	4.1	179	1.79	1.08	124.4	1.9	high	9.5	4.4	0	38	9.3
PS	55	F	25	1	18.1	3.9	177	1.55	1.12	103.8	1.9	high	8.8	5.9	0.1	31	12.2
PS	56	F	14	1	19.8	3.9	117	1.38	1.19	121.9	2.1	high	8.7	4.9			14.1
PS	45	M	14	1	20.2	3.5	157	1.22	1.13	104.1	2.5	high	8.1	6.5	0.1	22	9.9
PS	48	M	9	1	25.7	4.1	142	1.52	0.91	113.4	1.6	high	8.5	8.3	0.25	41	9.2
PS	55	F	33	1	19.4	4.1	128	1.47	0.85	105.4	1.8	high	9.2	5.5	0.07	29	9.6
PS	54	M	3	1	23.6	3.5	176	1.15	0.94	100.9	1.3	high	10.4	6.8	0.45	44	10.5
PS	61	F	19	1	21.4	3.9	148	0.92	1.05	154.1	2.2	high	9.1	6.3	0.65	26	9.2
PS	67	M	29	1	20.7	3.6	160	1.42	0.96	112.4	1.9	high	10.1	4.5	0	29	11.8
PS	57	M	31	1	20.8	3.7	115	1.33	1.01	134.6	1.6	high		4.4		29	9.7
PS	60	M	27	1	21.6	3.7	135	1.82	0.8	109.2	1.6	high		4.9	2.47	27	8.3
PS	65	M	21	1	21.1	3.8	118	1.75	1.08	116.5	1.8	high		5.1	0.1	29	10
PS	59	F	26	1	22.9	3.7	141	1.8	0.87	97.84	1.8	high	9.8	4.8	0.03	27	10.9
PS	57	F	23	1	19.5	3.7		0.9	0.92	116.6	1.9	high	9.6	3.6		33	9.9
PS	66	F	15	1	18.7	4	158	1.3	1.35	163.6	1.6	high	9.7	7.3	1	32	12.7
PS	50	M	10	1	19.1	4.1	132	1.36	0.76	83.84	2.5	high	8.4	4.6	0	13	10.2
PS	69	F	10	1	22.5	4.3	194	1.54	0.83	102.4	1.5	high		5.6	0.02	12	10.4
PS	48	M	4	1	28.4	3.4	151	1.17	0.95	84.67	2.1	high	8	5.4	0.04	22	10
PS	67	M	13	1	24.2	3.9		1.56	1.02	139.6	1.3	high	9.3	2.9	0.14		
PS	57	M	8	1	19.6	4.1	152	1.24	0.86	94.53	1.8	high	9.2	8.6		25	10.8
PS	73	F	8	1	19.3	4	80	1.28	0.69	83.33	1.3	high	9.1	4.4	0.07	35	9.3
PS	72	F	5	1	18.3	4.1	110	0.79	0.69	88.19	1.5	high	8.5	3.3	0	18	11.1
PS	55	F	18	1	18.6	4	147	1.5	1.08	149.6	1.6	high	8.7	5.5	0.03	21	8.5
PS	68	F	10	1	23.7		166	1.42	0.78	122.1	1.3	high	9.2	3.6	0.02	44	9.2
PS	61	F	2	1	24.9		142	1.41	1.04	79.88	1.5	high	8.8	5.5	0.26	23	
PS	43	M	13	1	23.2	4	150	1.1	0.87	145.1	1.6	high	9.5	4.7	0.76	21	10.5

PS	59	F	16	1	17.8	4.3	112	1.53	0.91	112.9	1.5	high	9.1	4.5	0.2	24	10.1
PS	59	F	9	1	16.8		121	1.03	0.94	96.08	1.8	high	9.2	7.5	1.01		8.7
PS	41	M	19	1	19.3		123	1.25	1.17	93.2	1.6	high	9.4	3.9	0.02	45	9.9
PS	65	M	14	1	17.8		124		1.3	58.3	1.6	high	8.4	5.9	0.06	26	10.5
PS	48	M	5	1	20.6		138	1.07	1.04	113.9	2.1	high	9	4.6	0.05	21	10.1
PS	70	M	2	1	21.8		131	1.28	0.9	99.17	1.5	high	9	3.6	0.34	30	11
PS	65	F	23	1	23	3		1.38	0.79	70.68	1.9	high	9.2	4.8	0.03	31	10.8
PS	50	F	9	1	22.8	3.6		2.08	0.95	136.9	1.5	high	10.5	5.1	0.05	37	9.8
PS	35	F	6	1	20.3	4	181	1.99	0.91	139.9	1.3	high	8.5	4			10.4
PS	44	F	6	1	20.4	3.6	245	1.52	1.03	122.1	1.6	high	10.1	6.3	0.05	23	11.1
PS	69	M	2	1	21.1	2.4	194	1.12	0.97	109.6	2.1	high		5.8	0.31	29	11.3
PS	64	F	33	1	19.5	3.8	145	1.82	1.04	77.06	1.4	high	9.8	4.8	0.16	32	10.6
PS	66	F	31	1	17.9	3.6	234	2.26	1.04	114	1	high	10.1	5.7	0.07	26	10.1
PS	45	F	31	1	16.8	3.7		1.9	0.82	118.5	1.5	high		4.6	0.05	22	12
PS	47	M	30	1	21.6	4.4	121	1.11	1	103.1	2.2	high	8.3	4.9	0.5	28	9.9
PS	66	F	28	1	15.6	3.6		1.14	1.09	101.9	1.8	high	8.4	6.7	0.69	33	10.5
PS	69	F	27	1	18.6	3.8	176	1.54	1.02	99.1	1.4	high	8.7	3.2	0.16	19	9.8
PS	72	F	25	1	23.2	3.5	148	1.57	1.01	107.4	1.4	high	9	6	0.12	16	8.4
PS	40	M	20	1	22.9	4.2	132	1.52	1.21	118.8	1.6	high	7.8	4.4	0.9		11.4
PS	52	M	19	1	20.4	4	152	2.54	1.07	94.11	1.4	high	8.8	4	0.1	20	10
PS	55	M	17	1	21.5	3.9	139	0.93	1.01	125	2	high	9.6	9.9	0.69	30	10.8
PS	54	M	17	1	24.6	3.8		1.28	1.08	127.9	1.6	high	9.1	4.1	0.05	26	9.1
PS	70	F	15	1	18.8	4		1.47	0.76	115.1	1.9	high	8.3	5.8	0	39	10
PS	59	M	14	1	20.1	4	106	1.42	1.16	128.5	1.3	high	8.2	4.5	0.03	31	10.1
PS	69	F	14	1	19.5	3.6	185	1.3	0.75	100.1	1.5	high	8.9	5.2	0.3	28	10
PS	63	F	13	1	18.6	3.9	125	1.29	1.05	82.73	1.3	high		5.4	0.1	25	9.8
PS	66	F	13	1	25.3	2	196	1.28	0.79	115.8	2.1	high	8.5	7.1		29	11.4
PS	70	F	13	1	15.9	4	50	1.33	0.83	89.56	1.6	high	9.3	7.4	0.02	20	10.5
PS	71	M	12	1	20.9	3.9	144	1.01	0.84	102.7	1.8	high	7.8	2.3	9.78		8.2
PS	67	F	10	1	18.1	4.2	221	1.23	0.83	100.4	1.1	high	8.4	4.4	0.1	29	9.5
PS	63	M	9	1	27.7	4	120	1.01	0.99	93.51	1.5	high		7.8	0.1	39	10.5
PS	65	M	9	1	24.4	4.2	49	1.58	0.8	126.2	1.6	high	9.4	5.9	0.02	24	10.1
PS	59	M	7	1	22.8	3.1	159	1.18	1.36	84.48	1.8	high	9.1	7.7	0.13	29	9.9
PS	76	M	6	1	22.9	4.1	101	1.38	0.86	149.7	1.6	high	9	4.6	0.09	27	11
PS	37	M	6	1	23.4	4.2	148	1.32	0.97	118.7	2.1	high	9.5	7.2		35	10.2
PS	70	M	6	1	23.2	4.1	151	1.57	0.82	126.6	1.5	high	9.3	6	0.07	17	10.1
PS	80	F	5	1	19.5	3.8	138	1.06	1.21	130.2	1.5	high	7.5	5	0.22	23	8.6
PS	52	M	4	1	21.1	4.2	185	1.71	0.94	130.8	1.1	high	9.5	3.2	0.35	32	9.7
PS	61	F	4	1	22.5	3.9	108	0.99	0.86	94.79	1.3	high	9.3	3.2	0.07	15	10.6
PS	70	M	3	1	25	3.8		1.22	0.86	125.9	1.6	high	8.8	4.4	3.61	16	9.5
PS	29	M	3	1	23.6	3.9	135	1.55	0.83	106.7	1.3	high		6.3	0.14	27	12.1
PS	76	F	3	1	18.9	3.8	160	1.05	1.19	55.87	1.8	high	8.6	5.7	0.5	13	11.4
PS	49	F	3	1	22	3.9	144	1.89	0.71	83.12	1.3	high		4.8	0.21	21	11
PS	75	M	3	1	23.3	3.9		1.7	0.89	90.25	1.5	high	9.4	2.6		32	10.1
PS	60	M	3	1	24.7	4	152	1.56	1.06	124.6	1.5	high		8.8			12
PS	65	M	2	1	17.8	3.6	88	1.2	0.68	100.8	1.1	high	7.6	4.9	0	44	8.6
PS	45	M	22	1	21.8	4	183	1.88	0.7	107.9	1.6	high	10.7	4.7	0.05	22	9.9
PS	67	F	30	1	18.5	3.8	81	1.59	0.98	97.51	1.6	high	9.5	7.4	0.34	26	13.4
PS	60	M	30	1	19.9	3.6	162	1.63	0.72		1.8	high	10.4	6.5	0.3	26	10.6
PS	60	F	28	1	17.5	3.8	202	1.85	1.13	96.55	1.5	high	9.7	6.1	0.06	24	11
PS	66	F	21	1	19.2	3.5	172	1.07	1.08	130	1.8	high	9.6	6.8	0.3	26	13.1
PS	59	F	19	1	18.2	3.7	137	1.25	1.06	129	2.1	high	9	4.4	0.05	26	10.4
PS	69	F	14	1	21.2	4	34	1.55	0.88	108.5	1.9	high	10.4	8.4	0.05	23	10.4
PS	61	M	13	1	18.7	4	112	1.26	1.37	120.4	1.8	high	8	6.2	1.16	34	11.1
PS	65	M	13	1	21.2	3.8	89	1.43	0.93	127.4	1.6	high	9.5	7	0.03	29	11.1
PS	62	M	13	1	21	4	104	1.39	0.9	130.6	1.6	high	9.7	4.7	4.36	28	10.4
PS	73	M	13	1	20.6	3.9	97	1.72	0.71	135.1	1.5	high	8.2	4.9	0.05	27	11.6
PS	59	M	13	1	20	4.2	71	1.43	0.84	125.7	1.9	high	7.1	6.9	0.07	27	10.4
PS	57	M	12	1	20.1	3.8	125	1.29	0.92	130.7	2.1	high	9.1	5.3			12.1

PS	66	F	10	1	20.9	3.7	163	1.34	0.74	109.4	1.3	high	7.9	3.2	0.05	17	8
PS	71	M	9	1	18.6	3.7	181	1.37	1.05	118.6	1.3	high	10.2	5	0.04	31	12.3
PS	42	F	9	1	21.8	3.6		1.79	1	130.4	1.6	high	9.3	7.1		25	10.7
PS	49	F	9	1	17.8	3.8	98	1.48	1.08	93.98	1.9	high	8.6	5.7	0.02	31	
PS	38	F	9	1	18.4	4.1	188	1.79	0.92	94.71	1.8	high	9.4	5.7			9.2
PS	67	M	6	1	21.6	3.9	188	1.37	0.91	126.4	2.5	high	7.7	4.6	0.15	25	12.2
PS	27	F	6	1	20	4.1	191	1.6	1.38	122.7	1.6	high	9.5	5.5	0.05	22	10.2
PS	70	M	5	1	20.8	3.8	141	1.24	0.83	123.4	2.1	high		4.5	0.02	34	11.6
PS	59	F	5	1	20.2	3.5	139	1.03	0.76	86.46	1.9	high	9.7	6.7	2.44	30	10.9
PS	61	F	5	1	20	3.9	158	1.36	0.87	114.3	1.3	high		3.8	2.83	32	11.3
PS	54	M	4	1	22.3	3.7	199	1.06	0.91	137.5	1.3	high	7.8	3.3	1.12	25	8.7
PS	37	F	4	1	22.8	3.6	145	1.44	0.72	85.58	1.5	high	8.6	5.4	0.2		10.3
PS	57	M	2	1	19.9	4.1	112	1.29	0.84	100.5	1.8	high	7.6	6.6	1.69	33	10.8
PS	66	M	10	1	24.4	3.7	152	1.4	0.91	120.8	1.8	high	7.6	3.7		26	11.6
PS	61	F	20	1	24	4	154	1.62	1.3	126.9	1.8	high	9.5	6.3	0.05		11.2
PS	68	M	8	1	24.4	4.2	127	1.29	1.19	120.4	1.6	high	9.1	5.9	0.07	30	11
PS	30	M	18	1	16.7	4.3	132	1.15	1.32	90.33	1.9	high	10.4	6.7	0.58	35	10.8
PS	59	M	16	1	22.7	3.6	141	1.2	0.96	113.3	1.5	high	7.5	6	1.5	35	10
PS	48	M	4	1	26.8	3.9	140	1.42	1	100.1	1.5	high	8.6	5.8		31	11
PS	58	M	3	1	20.6	3.7		1.38	0.58	91.19	2.1	high	9.1	6.1	0.2	24	12.1
PS	73	M	2	1	18.6	3.3	163	1.81	0.57	90.99	2.1	high	7.6	6.7		27	9.7
PS	58	M	26	1	21	3.9		1.54	1.23	116.4	2.5	high	9.9	4.6	0.15	23	11.2
PS	52	F	16	1	23.1	3.8		1.53	0.95	124.8	2.1	high	9.6	6.6	0.24	25	10.8
PS	66	M	17	1	17.5	3.6		1.53	0.86	104.4	1.8	high	9.4	5.4	1.44	32	11.7
PS	47	F	8	1	21	3.7		1.14	0.87	124.5	2.5	high		7.4	0.39		8.7
PS	57	F	17	1	15.6	3.4	180	2.13	0.78	117.9	1.5	high	9.8	6.1	0.09	29	11.6
PS	64	F	13	1	17.5	3.9		1.4	1.1	111.9	2.5	high	10	10.7	1.17	28	12.6
PS	62	M	28	1	19.2	3.7		1.29	1.28	78.71	2.1	high	9.7	5.2	0.12	26	9.4
PS	65	M	19	1	13	3.3	163	1.25	0.7	49.21	2.2	high	8.5	9.9	0.1	27	9.3
PS	54	F	16	1	18.6	3.8	147	1.13	0.98	81.66	2.1	high	9.9	5.8	0.17	29	10.9
PS	72	F	13	1	20		138	1.5	0.98	152	1.6	high	8.2	5.2	0.05	29	10.6
PS	72	M	13	1	23	3.6	146	1.38	1.02	104.3	2.1	high	9.9	7.8	0.42	23	11.8
PS	72	M	5	1	20.3	4.1	155	1.14	0.71	104.1	1.8	high	9.5	7.5	1.62	43	12
PS	82	M	3	1	20.4	3.4	127	1.61	0.63	106.4	1.6	high		4.6	0.05	30	10.7
PS	77	M	3	1	20.3	3.6	120	0.96	0.71	88.02	1.8	high	8.9	2.3	0.18	29	7
PS	83	F	2	1	30	3.9	138	1.07	0.8	112.9	1.8	high	9.7	5.4		30	10.7
PS	72	F	27	1	20.1	3.5	151	1.76	0.99	73.76	1.5	high	7.2	5.7	0.44	22	12.8
PS	70	F	2	1	22.9		202	1.94	0.95	103.9	1.5	high	8.6	5.1		21	10.1
PS	84	F	5	1	23.1		122	0.86	1.06	129.1	1.5	high	9.1	4.8	0.11	31	11
PS	61	F	22	1	20.3	3.6	157	1.45	0.8	92.68	1.4	high	9.7	5.1	0.1		10.1
PS	66	M	12	1	21.5	3.7	189	2.65	0.74	94.94	2.1	high	11	3.9	0.07	16	8.7
PS	51	F	10	1	14	3	145	1.09	0.85	14.43	1.5	high	9.1	3.5	1.12	29	9.8
PS	70	F	9	1	24.3	3.9	126	0.81	1	91.47	2.1	high		6.4	0.25	31	9.7
PS	59	M	2	1	24.3	3.3	153	1.98	0.59	87.67	1.6	high	9.8	4.3	0.05	32	11.2
PS	62	F	5	1	14.7	4	109	1.2	1.41	74.5	1.8	high	9.4	5.5	0.3	26	9.3
PS	57	M	4	1	21.7	3.6	182	1.46	0.87	120.3	1.5	high	10.4	4.6		29	9
PS	67	M	3	1	20.5	3.6	153	1.44	0.69	121.9	1.5	high		6.2	0.64		11.3
PS	88	M	2	1	23.3	3.6	172	1.92	0.72	64.39	1.4	high	8.7	5.4	0.1	25	10.8
PS	85	M	9	1	21	3.3	192	1.69	0.96	115	1.3	high	9.7	4.9	0.1	30	11.1
PS	68	M	5	1	23.9	4	158	1.3	0.87	79.51	1.5	high	7.8	4.4	0.06	20	10.3
PS	65	F	32	1	18.6	3.7	142	1.55	0.94	117	1.9	high	9	3.6	0.15	33	11.3
PS	83	M	3	1	18.3	3.7	145	1.95	0.87	99.64	1.3	high	8.9	5.4	0.2	31	9.7
PS	59	F	17	1	15.9	4.2	116	1.46	1.2	90.63	1.6	high	9.2	5.5	0.08	27	10
PS	71	M	14	1	17.5	4.2		1.23	1.25	126	2.1	high	8.7	7.1		23	11.3
PS	39	F	13	1	20.6	4	262	1.33	0.82	101.5	1.9	high	9.5	4.5	0.25	25	11.1
PS	38	F	13	1	23.7	4	164	1.44	0.83	120	1.5	high	10.3	6.2			9.5
PS	62	M	12	1	15.9	4.2	98	1.05	0.91	94.96	1.1	high		4.2	0.36	26	8.3
PS	63	F	10	1	16.1	4.3		1.66	0.83	139.6	2.1	high	9.4	8.7	0.06	33	12.6
PS	69	M	7	1	20.1	3.6	134	0.74	0.64	63.01	1.5	high	7.9	4.2	1.03	22	10.2

PS	64	M	6	1	21.1	3.7		1.37	0.96	123.4	1.5	high	10.3	5.1	0.35	20	9.6
PS	55	F	6	1	19.6	3.8	89	1.51	1.02	117.8	1.6	high	11.5	5.1	0.05	22	10.5
PS	58	M	6	1	20.9	3.6		1.34	0.75	124	2.1	high	8.6	6.7	0.08	25	9.8
PS	68	M	6	1	19.9	4.2	127	1.34	1.02	97.89	2.1	high	9.3	6.5	0.59	37	12.5
PS	52	M	5	1	18.7	4	141	1.09	0.86	56.88	2.1	high	7.9	6.1	0	27	11.7
PS	57	M	4	1	21	3.7	138	1.52	0.63	112.4	1.8	high		5.8	0.04		13.8
PS	49	M	4	1	19.3	4.3	33	1.1	0.8	115.3	1.6	high	9.8	4.5	0.27	19	10.5
PS	70	M	3	1	22.9	3.8	79	1.35	0.85	99.11	1.3	high	9.2	3	0.02	23	9.3
PS	63	M	2	1	19.3	3.8		1.98	0.75	68.37	2.5	high	8.5	4.4	1.47	30	10.1
PS	61	F	8	1	21.2	3.6	263	1.73	0.83	121.1	1.5	high	9.9	5.8	2.36	28	10.7
PS	60	M	32	1	21.5	3.8	182	1.75	0.77	91.23	1.6	high	10.7	7.4	0.01	30	9.1
PS	68	F	25	1	15	4	44	1.3	1.01	111.2	1.9	high	10.2	4.4	0.07	35	10.8
PS	62	F	11	1	16.9	3.9	100	1.88	1.19	118.8	1.3	high	9	3.5	0.2	36	10.5
PS	60	F	11	1	19.3	3.5	117	1.24	0.79	116.1	1.6	high	8.7	6.8	0.05	23	10.5
PS	60	F	9	1	16.9	4.3	47	1.69	1.03	104.9	1.3	high	9.7	3.7	0.02	35	11.9
PS	65	M	9	1	17.3	3.8	181	1.36	0.88	128.2	1	high	9.8	3.4	0.26	37	10.7
PS	50	M	9	1	17.4	3.5	138	1.91	0.79	104.2	1.5	high	9.5	8.5			9.1
PS	37	M	9	1	17.4	4.6	194	1.81	1	134.6	1.5	high	8.6	6.2			10.1
PS	76	F	8	1	22.9	3.2		1.15	0.86	143.2	1.6	high		3	4.45	32	9.5
PS	69	M	5	1	24.7	2.7	112	1.39	0.69	104.4	1.4	high	8.9	4.6		26	
PS	76	M	4	1	22.7	3.7	210	1.94	0.86	84.77	1.5	high		5.5	0.23	24	9.8
PS	71	M	2	1	20	3.5	105	1.13	0.83	83.91	1.3	high	8.4	2.8	0.3	30	9.6
PS	52	M	2	1	21	3.8		1.29	0.76	98.41	2.1	high	9.6	5.7	0.17	24	10.5
PS	56	M	27	1	20	3.9	265	1.52	0.82	104.2	1.6	high	9	4	0.07	35	12.2
PS	55	M	18	1	20	3.9	170	0.95	0.92	109.7	2.1	high		4.2	0.02		10.3
PS	60	F	11	1	17.4	4	159	1.55	0.77	118.2	1.9	high	10.4	7.5	0.24	27	11.3
PS	47	M	10	1	20.3	4.3		1.9	0.76	124.8	1.5	high		4.8			10.4
PS	64	F	8	1	24.2	3.6		1.47	1	141	2.1	high	9.8	5.7	0.11	22	11.5
PS	66	M	5	1	20.8	3.5	128	1.13	1.08	134.6	1.8	high	8.6	5.1			9.7
PS	49	F	4	1	20.2	4.1		1.58	1.06	135.5	1.5	high		4.2	0.26		
PS	67	M	2	1	23.9	4	134	1.69	0.87	109.5	1.5	high	9.4	5.7	0.03	33	11.3
PS	56	M	4	1	20.5	4.2	120	1.55	0.89	88.89	1.5	high		5.8	0.43	29	11.3
PS	63	M	4	1	23.6	4.2	70	1.29	0.92	116.6	1.9	high	9.9	5.8	0.23	21	9
PS	55	M	2	1	19.5	4		1.39	0.89	76.58	1.5	high	9.7	4.5	0.2	18	10.9
PS	54	M	3	1	22.6	4.2		1.8	1.03	106	1.5	high	8.1	6	0.21		12
PS	56	F	11	1	28.5	3.4	49	1.57	1.28	153.8	1.9	high	9.5	7.2	0.05	25	11.2
PS	61	F	6	1	29.2	3.9	157	1.26	0.91	135.9	2.1	high	9	5		38	12.1
PS	60	F	4	1	25.2	3.8	24	1.13	0.95	131.5	1.9	high	10.1	5.8	0.07	3	9.5
PS	78	M	2	1	27.1	3.6	145	1.7	0.74	75.46	1.5	high	9.1	5.8	0.08	37	11.1
PS	56	M	4	1	24.3	4.2	178	1.48	1.07	71.76	1.5	high	8.7	6.7	0.59	27	9.1
PS	73	F	10	1	19.3	3.9	82	1.59	0.99	128.7	1.6	high	9.1	5.5	0.68	34	10.7
PS	72	M	19	1	17.1	3.2	155	1.52	0.94	81.99	1.6	high	9.2	7.3	0.05	27	12.4
PS	53	F	4	1	28.5	3.8	168	1.16	0.64	136	1.4	high	9	4.3		31	10
PS	67	M	17	1	17.2	3.9	107	1.02	1.25	132.9	1.9	high	9.3	6.4	1.29	28	10.6
PS	80	F	2	1	21.2	4	161	1.16	1.16	143.7	1.5	high	8.3	9.4	0.07	30	11
PS	46	F	19	1	21.1	4.6	52	1.44	1.09	104.8	1.9	high	8.3	5.4	0.01	28	11.6
PS	57	F	21	1	21.1	4.1	62	1.25	0.96	127.3	1.3	high	9.7	2.8	0.16	19	10.4
PS	64	F	26	1	18.6	3.1	66	1.14	0.94	87.28	1.3	high	10.1	6.1	0.11	25	10.7
PS	67	F	18	1	16.5	4.2	33	1.62	1.01	134.8	1.6	high	8.9	5.2	0.06	29	10.6
PS	52	F	17	1	17.5	3.9	67	1.55	1.3	109.8	1.9	high	10.6	4.5	0.01	29	11.9
PS	81	F	17	1	19.6	3	56	1.19	0.94	89.57	1.6	high	9.2	6.6	0.11	27	14
PS	66	M	21	1	23.7	3.7	167	1.73	0.84	140	1.8	high	8.5	4.4	0.12	21	
PS	67	M	19	1	17.2	3.7	133	1.26	1.08	106.2	1.9	high	11.2	5.2	0.47	24	11.2
PS	58	F	12	1	14.9	3.3	164	1.67	0.66	89.89	1.9	high	9.4	6.2	0.4	26	10.3
PS	52	M	14	1	22.1	4.1	144	1.41	1.05	111.4	1.3	high	8.6	5.7	0.11	18	11.6
PS	79	F	6	1	16.8	3.2	99	0.98	0.95	83.97	2.1	high	8.4	7	3.5	29	9
PS	56	F	5	1	25.5	3.8	119	1.47	0.9	86.11	1.8	high	9.4	6	0.2	26	10.2
PS	53	F	12	1	17.2	3.9	137	1.41	0.92	78.2	1.5	high	8.6	3.4	0.05	27	10.1
PS	69	F	12	1	25.4	3.5	179	1.48	0.96	116.1	1.5	high		6	0.17	27	10.5

PS	65	M	4	1	24.1	4.3		0.75	0.77	99.62	2.1	high	8.9	5.3	0	30	11.6
PS	72	F	11	1	27.1	3.8	186	1.95	0.73	132.9	1.6	high	8.4	5.8	0.1	27	9.1
PS	69	F	9	1	16.8	3.9	141	1.74	0.73	121.7	1.2	high	9.3	6.9	0.1		10.5
PS	38	F	11	1	17.7	4.1	139	1.26	0.93	118.7	2.1	high		5.1	0.37		10.4
PS	69	F	10	1	16.4	2.9	69	1.58	1.27	115.3	1.9	high	9.4	5.6	0.3	36	10.6
PS	60	F	10	1	24.4	4.1	187	1.46	0.88	130.3	1.6	high	8.2	6.2	0.04	25	10.5
PS	66	M	10	1	20.6	3.7		1.49	0.96	109.8	1.1	high	9.5	5.5			9.8
PS	88	F	9	1	15.8	3.8	153	1.55	0.84	56.01	1.5	high	9.5	8	0	23	9.8
PS	81	F	5	1	17.7	3.4	174	1.49	1.02	109.5	1.8	high	9	6.8	0.05	25	9.6
PS	44	F	9	1	17.3	3.8	140	1.24	0.84	88.24	2.1	high	10.1	5.4			10.9
PS	53	M	14	1	21.2	3.8		1.27	1	118.6	2.1	high	9.6	5.2	0.2	14	10.7
PS	78	F	6	1	24.7	3.4	156	1.46	1.07	121	1.3	high	10.7	5.1	0.1	22	11.4
PS	50	F	7	1	20.3	4.2	183	1.33	0.98	106.4	1.8	high		5.2	0.92		10.8
PS	83	M	6	1	20.3	3.9	124	1.25	0.75	94.17	2.1	high	7.9	5.7	0.27	26	10.1
PS	70	M	5	1	21.4	4.4	101	1.27	1.03	84.66	1.5	high	9.4	4	0.2	22	9.9
PS	51	F	5	1	25	4.3	249	1.75	0.96	110.8	1.8	high	8.9	3.4	0.06	27	9.5
PS	59	M	18	1	21.3	4.1	150	1.84	0.75	126.2	0.9	high	8.1	8.2	0.3	53	9.2
PS	94	M	3	1	15.9	3.5	133	2.21	1.05	111	1.5	high	9.7	7.3	0.06	36	12.1
PS	67	M	3	1	25.1	3.6	101	1.32	0.86	96.96	2.1	high		4.7	0.01	28	9
PS	45	M	2	1	19.2	4.2	177	1.26	0.74	80.79	1.1	high	7.8	5.4	0.31	21	10.4
PS	80	M	9	1	21	3.3	98	1.2	0.73	116	1.8	high	7.5	2.5	0.57	25	7.8
PS	55	M	2	1	22.9	4.2	190	1.52	1.05	100.3	1.5	high	8	6.2	0.1	33	10.2
PS	67	F	3	1	18.5	4.7	170	1.33	0.98	105.2	2.2	high	9.1	4.8			
PS	58	M	36	1	21.4	3.1	134	1.4	0.74	81.88	1.3	high	8.5	4	0.02	16	10.8
PS	59	M	6	1	17.7	3.3	163	1.14	1.05	109.9	1.6	high	8	5.5	0.07	26	9.2
PS	50	F	3	1	32	3.4	156	1.63	0.69	74.19	2.1	high		4.4	0.14	30	10.6
PS	74	F	17	1	20	3.6	97	1.27	0.91	102.1	2.1	high	8.1	6	0		9.6
PS	61	M	9	1	17.2	3	133	1.69	0.47	52.91	1.5	high	9.3	4.7	0.06	35	11.3
PS	56	M	6	1	19.5	3.5	159	1.74	0.63	86.76	1.6	high	8.5	6.1	0.2	24	10
PS	62	M	2	1	26.4	3.3	125	1.19	0.64	75.26	2.1	high		3.6			9.5
PS	60	M	8	1	19	3.9	157	1.46	0.78	74.9	1.8	high	9.7	5.2	0.03		10.7
PS	61	M	16	1	21.8	3.6	153	1.41	0.88	131.8	1.6	high	8.7	4.9	0.06	24	9.8
PS	77	F	3	1	19.3	3.3	180	1.22	1.14	93.93	1.5	high	8.4	4.1	0.1	19	9.7
PS	54	M	5	1	21.8	3.7		0.82	0.6	88.72	1.5	high	8.5	3.7		38	11.8
PS	60	M	8	1	18.5	3.8	172	1.47	0.75	106.5	2.1	high	10	4.6		37	13.4
PS	76	M	2	1	18	3.6	110	1.48	0.81	126.4	1	high		3.7	5.06	22	10.1
PS	66	F	2	1	19.1	3.4	154	1.64	0.91	71.89	1.8	high	8	3.6	0	32	11.2
PS	60	M	9	1	18.9	3.1	160	2.09	0.93	93.74	1.6	high	8.8	4.6	0.05	19	10.7
PS	66	M	15	1	34.4	2.8	191	1.44	0.67	61.55	1.5	high	8.7	4.1	0.2	25	11.9
PS	66	F	5	1	24.5	3.7	132	1.41	1.27	139.4	1.8	high		4.9	1.15		10.7
PS	83	M	5	1	20.6	3.1	128	1.19	0.74	102.5	1.8	high	8.7	4.8	0.1	74	6.1
PS	51	M	8	1	20.9	3.5	154	1.48	0.65	93.73	1.6	high	7.6	3.8	0.17	24	10.9
PS	70	M	13	1	18.1	3.3	163	1.27	0.87	67.08	1	high	8.3	4.4	0.89	29	10.8
PS	54	F	11	1	23.3	4.1	114	0.99	0.83	130.3	1.3	high	9.1	6.4	0.07	26	10.2
PS	63	M	6	1	17.5	3.2	175	1.56	0.55	66.12	1.5	high	8.1	3.9	10.25	25	10.7
PS	66	M	4	1	22.4	3.8	176	1.58	1.01	126.3	1.3	high		5	0.42		9.8
PS	62	M	5	1	18.2	3.8	130	1.49	1.03	122.9	1.5	high		9.1			10.7
PS	74	M	8	1	22	3.7	157	1.18	0.73	119.3	1.3	high	7.2	4	0.07	18	10.4
PS	80	M	5	1	21.4	3.5	156	1.63	0.84	101.2	1.5	high	8.8	11.8	0.08	21	10.1
PS	52	M	2	1	22.4	3.7	140	1.23	1.21	101.6	1.3	high	9.3	4.7	0.27	19	9.8
PS	53	F	2	1	22.3	3.8	107	1.32	1.13	102.3	1.8	high	7.9	5.6	0.07	23	9.9
PS	55	M	7	1	18.5	3.8	206	1.37	0.84	91.74	2.1	high	7.9	7.9	0.12	27	10.8
PS	60	F	19	1	19.8	3.6		1.39	0.87	107.2	1.6	high	9.6	3.6	0.26	9	9.8
PS	79	M	9	1	20.2	3.1	133	1.68	0.73	122.7	1.5	high	9.4	6.4			11.4
PS	67	M	2	1	24.5	3.7	179	1.43	0.84	97.38	2.1	high	7.9	5.4	0.2	21	10.8
PS	57	F	3	1	30.6	3.7	147	1.52	0.97	95.07	1.3	high	9.5	6.9	0.68	28	6.7
PS	58	M	4	1	16.2	4.2	115	1.34	0.89	86.85	1.3	high	9.1	6.2	0.01	20	11.6
PS	56	F	34	1	16.1	3.8	152	1.63	1.23	118.1	1.3	high	9.1	4.6	0.03	21	11.3
PS	54	F	33	1	25.1	3.9	109	1.29	0.97	114	1.9	high	8.8	4.9	0.27	29	11.2

PS	65	M	32	1	23.6	3.4	156	1.53	0.87	72.28	1	high	8.2	5.2	0.05	26	10.7
PS	54	F	31	1	17.5	3.7	136	1.47	1.01	99.32	1.4	high	8.9	4.3	0.1	23	10.5
PS	41	M	28	1	24.3	3.8	120	1.05	1.12	94.44	1.8	high	8.7	7.9	0.02	22	10.9
PS	51	F	27	1	21	3.7	156	0.57	0.91	123.9	1	high	8.8	3.8	0.05	10	10.3
PS	53	F	27	1	20.2	4	139	0.81	1.24	105.7	1.8	high		5	1.6	21	11.8
PS	58	M	26	1	21.6	3.9	172	1.48	0.89	121.3	1.9	high	9.3	4.1	0.02	33	10.8
PS	62	M	26	1	20.1	4.1		1.55	0.99	128.7	1.5	high	9	8.8	0.06	23	13.8
PS	59	F	26	1	18.4	3.9	198	1.73	1.53	90.57	1.5	high	8.8	4.6	0.1		10.4
PS	56	M	26	1	24.2	4.1	213	1.45	0.84	95.86	1.3	high	5.2	5.3	0.11	27	11
PS	60	M	25	1	22.8	3.9	139	1.87	1.12	144.5	1	high	8.9	4.2	0.03	32	9.3
PS	52	M	24	1	24.2	4.2		1.45	0.93	112.4	1	high		5.4	0.1	27	10.1
PS	47	F	23	1	17.2	4.2	125	1.06	1.29	113.7	1.5	high	10.9	5.7			13.1
PS	55	F	21	1	18.1	4.2	154	1.47	0.94	111.7	1.3	high	7.8	4.4	0.04	26	11.1
PS	48	F	21	1	19.9	4	150	1.5	1.25	118.6	2.1	high	7.7	7.1	0.09	29	10.6
PS	59	M	20	1	21.1	3.7	114	1.24	0.98	136.2	1.3	high	9.4	6.2	0.1	21	12.8
PS	66	M	19	1	19.4	3.7	164	1.51	0.91	66.22	1.6	high	9.4	4.8	0.05	27	10.9
PS	75	M	19	1	22.7	3.9		2.39	0.9	113	1	high	8.1	4.4	0.27	39	7.3
PS	55	M	17	1	16.7	4	133	1.4	0.98	116.5	1.8	high	7.8	3.7		24	10.9
PS	66	M	16	1	21.9	3.7	153	1.18	0.96	104.5	1.8	high	9.6	5.5	0.53	27	8.9
PS	62	F	15	1	24	3.8	158	1.2	1.16	123.2	1	high	7.1	6.2		21	9.4
PS	71	M	15	1	16.4	3.4	221	1.48	1.01	90.8	1.5	high	9.6	5.5	0.15	27	10.5
PS	32	F	2	1	25.1	3.9	142	1.61	1.04	102.3	2	high	12.5	4	0.1		10.6
PS	54	M	13	1	20.3	4.5		1.27	0.92	113.6	1.8	high	10.3	5.7			
PS	65	M	12	1	24.4	3.7	154	1.78	0.81	135.3	1.5	high	9.8	4.7	0.12	25	11.3
PS	62	M	12	1	25.5	3.9	114	1.08	1	108.2	1.5	high		5.3	0.27	34	11.1
PS	54	F	11	1	19.2	3.8	136	1.34	1.31	134.2	1.6	high	7.7	6.3	0.14	26	10.5
PS	47	M	11	1	24.1	4.4	147	1.23	1.03	133.8	2.5	high	9.8	6.7	2.14	29	10.7
PS	43	F	11	1	21.2	4.1	143	1.3	1.14	125.9	2.1	high		5.2	0.2	23	10.1
PS	55	M	10	1	19.3	4	169	1.8	0.93	117.9	1.8	high	9.3	5	4	28	11.2
PS	33	M	10	1	16.2	4.1	171	1.24	0.85	80.88	1.8	high	9.8	7.1	0.82	38	
PS	60	M	10	1	20.6	4.6	170	1.6	0.96	85.34	2.2	high	9.9	5	0.1	23	9.7
PS	56	M	9	1	21.2	4	171	1.03	0.89	100.1	2.1	high	7.9	6.5	1.85	18	14.7
PS	30	F	9	1	14.7	4.6	163	1.26	1.09	96.27	1.8	high	7.6	7.5			11.5
PS	58	M	9	1	20.6	4.1	161	1.66	1.11	110.2	1.5	high		5.6	0.43	18	9.1
PS	68	M	8	1	22.3	3.6	161	1.86	0.86	145.6	1.1	high	8.6	3.9			6.8
PS	65	F	7	1	27.4	3.7	147	1.13	0.73	138.7	2.1	high	9.5	4.7	0.05	34	11.1
PS	70	M	6	1	21	3.9	194	1.65	1.08	122.4	2.2	high	9.3	4.8	0.24	32	9.9
PS	26	F	6	1	18.1	3.6	183	1.02	1.23	123.4	1.6	high	9.1	6.7	0.4	31	13.9
PS	49	M	6	1	23.7	4		1.34	1.05	128.5	2.5	high	10	5.8	0.28		
PS	43	M	6	1	15.4	4.6	129	1.67	0.97	92.94	1.6	high		5.7			8.6
PS	34	F	5	1	19.3	4.2	118	1.74	0.72	109.3	2.1	high	8.2	4.3	0.1	25	11.2
PS	41	F	5	1	24.6	4		1.38	1.14	104.7	2.1	high	7.6	5.7	0.05	20	10.5
PS	64	M	5	1	22.1	4.2	157	1.48	1.05	114.1	1.8	high	9.2	5.6		31	5.8
PS	60	M	5	1	17.8	3.7	106	1.29	0.69	100.8	1.4	high	7.1	5.6	0.37	23	8.6
PS	33	M	5	1	20.2	4.3		1.57	1.13	143.5	1.1	high	8.6	7.2	0.04		8.6
PS	58	M	5	1	21.2	3.5	149	1.49	0.9	118	1.6	high	8.9	2.7		27	8.8
PS	49	F	5	1	23	3.8	119	1.56	0.74	88.74	1.8	high	9.7	3.8	0.02	47	10
PS	60	F	4	1	21	4.2		1.31	1.03	83.24	1.3	high	8.8	5.9		27	14.7
PS	59	F	4	1	18.1	3.8	133	1.45	1.1	102.1	2.1	high	9.1	8.1	0.05	27	9.5
PS	51	M	4	1	22.9	3.5	141	1.15	0.76	107.5	1.1	high	9.3	3.4		16	10.4
PS	59	M	4	1	25.9	4	108	1.48	0.79	93.4	1.3	high	8.7	5.6	0.04	16	13.7
PS	49	M	4	1	34	4.1	172	1.43	0.91	86.11	1.1	high	8.1	4	1.08	31	8.4
PS	57	M	4	1	22.6	3.5	173	1.28	1.15	137.4	1.3	high		7.6	0.12	30	11.1
PS	54	F	3	1	17.2	3.8	173	1.3	1.06	114.2	1	high	9.2	4.1	0.19	29	8.9
PS	79	F	3	1	19.6	3.7	136	1.1	1.08	130.2	1.5	high	8.8	5.3	0.2	16	10.2
PS	68	F	3	1	18.5	4.4	146	1.75	0.98	93.9	1.5	high	8.8	3.5	0.33	20	11.1
PS	59	M	2	1	22.4	4	163	1.42	0.89	79.48	1.1	high	8.7	5.6	0.17	27	10.3
PS	72	F	2	1	21.2	3.8	102	1.14	1.06	117.7	1.8	high	8.9	6.8	3.29	33	9.8
PS	59	M	2	1	24.4	4.1	104	1.23	0.66	78.75	1.8	high	9.4	5.6	0.7	47	10

PS	65	F	11	1	18.4	3.8	159	1	1.07	114.7	2.2	high		7.1	0.2	24	8.7
PS	60	M	2	1	22.8	4.2	189	1.66	1.02	73.55	1.3	high	8.9	3.5	0.01	22	12
PS	56	M	31	1	22.1	4.2	138	1.21	1.1	122.3	1.3	high	9.4	5.3	1.2	33	9.5
PS	66	F	38	1	16.8	3.6	182	1.57	1	59.24	2.2	high	9.3	5.5	0.1	23	9.4
PS	66	F	8	1	18.4	3.6	87	0.87	0.85	110.5	1.3	high		2.1	1.03		9.9
PS	50	M	20	1	24.4	3.7	163	1.8	0.97	105.5	1.3	high	10.5	7.4	0.01	26	11
PS	63	F	6	1	23.1	3.5	164	1.5	0.85	107.6	0.8	high	7.1	3.1	0.17	32	9.9
PS	35	M	10	1	19.2	3.8	145	1.52	0.86	87.92	1.5	high	8.1	3.3			10.5
PS	79	F	3	1	21.4	3.3	103	1.52	0.79	97.34	1.3	high	6.8	3.9	5.26	38	8.7
PS	69	F	2	1	14.4	3.7	135	1.63	1.02	89.27	1.8	high	9	6.8	0.05	24	12.4
PS	47	M	27	1	21.9		215	1.17	0.76	70.21	1	high	8.8	4.6	0.05	26	10.3
PS	43	M	25	1	20.3		134	1.89	1.02	106.9	1.8	high	9.5	7.2	0.2	23	8.2
PS	61	M	24	1	23.2		142	1.36	0.91	83.25	1.3	high		4.8		24	8.3
PS	65	F	11	1	16.4		200	2.17	0.8	99.71	1.5	high	8.9	4.7	0.07	18	10.5
PS	69	F	10	1	16.7		159	0.5	0.7	80.87	1.4	high	8.2	2.7			8.3
PS	66	F	6	1	20.6	4.1	175	0.89	1.03	95.31	1.4	high	8.4	3.8	0.8	9	
PS	66	M	3	1	21.2	3.9	171	1.2	0.73	109.2	2.1	high	8.5	3.2	0.4	41	9.4
PS	76	F	20	1	17.3	3.7	156	1.38	0.85	113.1	1.9	high		10.7	0.3	30	11.2
PS	74	M	14	1	25.3	3.1	140	1.59	0.97	129.1	1.3	high	8.2	5.5	0.01	28	8.4
PS	88	M	5	1	22.9	3.6	121	1.8	0.73	66.09	1.8	high	10.2	4.9	0.07		10.4
PS	78	M	3	1	28.9	3.2	171	1.71	0.6	66.07	1.5	high	8.4	4.4	0.16	32	7.8
PS	51	F	22	1	20.7	3.9	179	1.25	1.31	84.72	1.3	high	8.1	2.6	1.73	24	10.6
PS	61	F	24	1	22.6	3.8	150	1.26	0.97	89.56	1.8	high	8.7	4.3	0.02	31	10
PS	51	F	24	1	22.8	3.8	170	1.23	1.1	106.3	1.3	high	7.5	6.1	0.05	33	10.2
PS	70	F	14	1	22.9	3.7	166	1.44	0.97	107.2	1	high	7.7	7.1	0.3	14	10.8
PS	71	M	16	1	20	3.5	169	1.51	1.15	116.9	0.6	high	8	4.8	0.02	17	11.5
PS	52	M	16	1	21.4	3.9	189	1.53	1	105.8	2.1	high	10	4.8	1.06	21	10.2
PS	56	M	29	1	25.8	3.9	182	1.61	1.17	92.84	1.3	high	9.3	5.4	3.12	26	9.7
PS	56	M	27	1	23	4	208	1.45	1.07	109.8	1.6	high	9.5	7	0.25	18	12.2
PS	56	F	34	1	24.8	3.9	175	1.05	0.71	65.87	1.9	high	9.9	5.9	0.02	25	10.3
PS	64	F	13	1	19.5	3.8	222	1.51	0.93	126.5	1.8	high		4.8	0.13		10.3
PS	53	F	17	1	21.2	3.6	126	1.23	0.75	92.01	1.3	high		4.3	0.1	43	9.9
PS	57	F	31	1	19.8	3.6	198	1.63	1.16	104.4	1.4	high	8.7	6.2	0.36	33	10.5
PS	73	F	16	1	18.5	3.6	198	1.27	1.15	108.7	1.3	high		6.4	4.95		10.4
PS	74	M	12	1	21.8	3.5	173	1.4	1.07	123.4	1.8	high	10.7	5.6	0.02		8.9
PS	54	F	12	1	20.9	4	158	1.2	0.93	124.1	2.1	high	8	7	0.3	24	11.7
PS	60	M	14	1	20.7	3.8	130	1.16	0.9	110.9	1.3	high	11.1	5.9		27	12.1
PS	71	F	11	1	17.8	3.7	158	1.18	1.14	117.7	0.6	high	8.5	4.9	0.1	23	10.3
PS	69	M	10	1	25.5	3.6	191	1.15	0.89	122.3	2.1	high	8	6.5	0.16	32	9.5
PS	61	F	9	1	18.9	3.7	124	1.22	0.9	77.11	1.3	high	8.2	4.6	0.2	28	7.4
PS	77	M	9	1	22.7	4.1		1.32	0.89	132.8	2.5	high	8.9	6.3	0.1	21	11.2
PS	61	M	8	1	20.8	3.5	237	1.63	1.12	101.7	1.3	high	8.9	5.8	0.1	25	9.6
PS	69	M	8	1	25.3	3.5		1.4	0.79	92.65	1.9	high	8.1	3.6		32	9.7
PS	58	F	8	1	23.5	3.7	176	1.37	0.87	118	1.9	high	10.3	4.7		27	12.3
PS	76	M	7	1	21.3	3.8	160	1.34	1.24	101.6	2.1	high	8.2	5	0.3	21	12.4
PS	59	F	6	1	18.8	3.4	195	1.05	1.03	119.7	1.8	high		4.9	10.25	29	12.5
PS	44	M	6	1	20	3.7	157	2.52	0.97	123	1.6	high	10	4.8		37	11.7
PS	34	F	5	1	22.9	3.3	141	1.82	1.02	106.9	1.9	high	10.8	4.4		18	10.3
PS	57	M	5	1	22.7	3.8	118	1.67	1.01	82.51	1.8	high	9.1	5.8	0.03	27	10.9
PS	58	F	4	1	24.5	3.6	165	1.76	0.91	99.79	1.6	high	9.2	5.5		31	10.8
PS	68	M	5	1	20.5	3.8	122	1.42	0.83	123.7	1.5	high	7.5	4.3	1.2	29	8.7
PS	71	M	3	1	23.4	3.8	184	2.41	0.83	74.35	1.6	high	9.2	5.3		25	10.1
PS	73	F	3	1	26.3	3.6	123	1.63	0.63	87.64	1.9	high	9.3	4.3		29	9.9
PS	57	F	4	1	19.4	3.7	120	1.69	0.83	121.9	1.6	high	9	5.1		27	9.9
PS	33	M	3	1	25.1		153	1.53	0.85	74.99	1.9	high	9.6	6.8		28	10.4
PS	59	F	3	1	24.9	3.6	124	1.39	0.87	96.54	1.5	high	7.8	4.7	0.09	28	10.7
PS	59	F	3	1	25.7	3.5	175	1.49	0.69	91.81	1.5	high	9.7	7.1	0.37	15	9.2
PS	39	M	3	1	29	3.8	124	1.1	0.8	66.42	1.8	high		6.2	0.03	45	11.4
PS	67	M	2	1	23.2	3.2		1.4	0.62	110.2	1.3	high	10.4	4.3	0.44		11

PS	53	M	2	1	27.8	3.7	104	1.19	0.78	117.1	1.5	high		9.3	1.16	30	10.4
PS	49	M	2	1	21.9	3.9	138	1.55	0.92	78.34	1.5	high	9.3	3.9	0.1		11.1
PS	82	M	5	1	21.8	3.3	176	1.43	0.75	111.4	1.5	high	9.3	3.9			10.8
PS	50	M	6	1	28.2	3.8		1.49	1.03	77.64	1.5	high	8.6	4.7	0.3	26	10.7
PS	42	M	5	1	46.3	3.3	138	2.28	0.89	73.91	2.1	high	8.4	3.1	0.1	31	10.3
PS	66	F	4	1	23.9	3.6	127	2.05	0.85	86.66	1.3	high	10.5	4.3	0.05	32	8.8
PS	80	M	2	1	15.7	4.1	133	1.28	1.16	109.5	2.1	high	8.6	5.8	0.2		11.4
PS	60	F	2	1	18	3.8	177	1.99	1.29	130.9	1.6	high	9.4	4.5		29	9.6
PS	69	F	4	1	19	3.7	125	1.37	1.07	122	1.6	high	8.9	5.1	0.71		10
PS	72	M	3	1	24.5	4	126	1.37	0.7	97.85	1.8	high		5.3	0.33		10.7
PS	80	F	7	1	17.3	4	129	1.08	0.82	112.6	1.3	high	7.8	5.2		23	9
PS	70	F	6	1	18.2	4	108	1.58	1.03	113.2	1.5	high	9.1	4.4	0	28	10.1
PS	55	M	3	1	22.4	4	212	1.32	0.82	81.65	1.5	high		10.2	0.19	26	11.2
PS	80	F	2	1	28.5	3	200	1.51	0.98	126.1	1.6	high	8.9	7.4	0.01	23	10.5
PS	78	M	2	1	19	3.9	155	1.54	0.79	64.59	1.8	high	8.4	3.9	0.48	24	10.6
PS	76	M	2	1	20.8	3.9	199	1.29	0.81	106.8	1.3	high	10	6	1.35		12
PS	60	M	4	1	18.8	3.4	172	1.23	0.73	103.6	2.1	high	9.3	4.8	0.06		11.8
PS	71	F	4	1	19.8	4.1	86	1.21	0.81	67.74	2.1	high	8.1	3.7	0.03	21	9.4
PS	77	F	23	1	18.4	3.9	173	1.61	0.96	99.12	1.6	high	8.7	4.7	0	25	10.7
PS	77	M	13	1	18.2	3.9	155	1.52	0.99	85.67	1.8	high	9.6	7.8		23	11.6
PS	78	M	9	1	18.9	3.8	121	1.76	0.73	115.2	1.5	high	10.3	5	0.12		10.4
PS	71	F	30	1	18.7	3.1	91	1.61	0.69	92.18	1.9	high	8.4	6	0.06	26	10.4
PS	66	M	30	1	20.9	3.2	143	1.82	0.87	59.05	1.9	high	9.7	6.1	0.1	22	10.6
PS	40	F	5	1	18.4	3.9	185	1.21	1.13	122.1	2.1	high	9.4	8.2	0.06	29	11.4
PS	59	M	3	1	23.8	3.6	228	1	0.98	99.29	1.5	high		4.8	0.1	27	10.3
PS	48	M	2	1	24.1	4.1	145	1.25	1.02	122.3	2.1	high	10.6	6.5	0.1		11.6
PS	76	M	10	1	20.2	3.8	163	1.56	0.34	14.79	1.6	high	10	5	0.03	30	9.1
PS	57	M	12	1	22.4	3.5	152	1.22	0.84	91.06	1.6	high	9.2	5.7	0.24	31	10.4
PS	32	M	17	1	17.1	3.4	125	1.47	0.9	72.88	2	high	10.4	5.3	0.04	30	11.1
PS	65	F	16	1	24.3	4.2	226	1.35	1.06	133.4	1.6	high	8.3	4.8	0.1	14	12
PS	58	M	12	1	23.1	4.1	177	1.39	0.91	126	1.6	high	9	4.5	0.09	23	11.1
PS	75	M	9	1	20.5	4.1	107	1.25	1.11	148	1.6	high	9.1	6	0.14	24	12.3
PS	68	M	15	1	21.3	3.4	194	1.3	0.48	110.9	1.8	high	9.7	6.7	0.05	19	10.9
PS	57	F	15	1	16.9	3.9	153	1.4	0.79	112.4	1.6	high	8.9	7.1	0.09	20	12.1
PS	75	M	5	1	22.6	3.8		1.25	0.89	90.21	1.3	high	8.8	4.2	0.1	30	11.6
PS	60	M	6	1	21	4.1	129	1.6	0.9	116.9	1.6	high	9	4.9	0.03	29	10.1
PS	71	M	5	1	19.9	4.2	145	1.38	0.86	99.5	1.3	high	9.1	3.5	0.05	26	14
PS	60	M	28	1	18.7	3.9	143	1.17	0.82	107.5	0.6	high	8.1	4.2	0.32	24	10.7
PS	71	M	5	1	20.1	3.8	141	1.8	0.93	146.9	1.8	high		4.6	0.26	30	12.3
PS	59	F	29	1	21.9	3.9	180	1.58	1.01	132.3	1.6	high	8.7	9	0.02	29	10.9
PS	68	M	25	1	19.6	4	212	1.32	0.92	115.5	1.6	high	10	6	0.13	29	11.5
PS	48	M	24	1	19.6	4	134	1.69	0.79	138.2	1.6	high	9.2	3.5	0.37	29	10.2
PS	64	M	16	1	22	4.1	121	1.24	0.86	130.2	1.6	high	8.6	3.8	0.01	22	10.8
PS	56	M	13	1	19.7	4.3	109	1.42	1.36	136.1	1.6	high	9.2	6.3	0.02	32	11.2
PS	73	F	11	1	20.6	4	151	1.41	0.93	130.3	1.6	high	8.5	6.2	0.41	45	7.8
PS	44	M	5	1	21.9	4.1	136	1.3	0.72	99.35	1.5	high	8.8	3.7	1.35	22	9.8
PS	79	M	5	1	24.4	3.6	164	1.8	0.87	112.5	1.1	high		6.3		26	11
PS	80	F	3	1	17.1	4.1	117	1.2	1.24	147.4	1	high		2.2	2.6	27	11.6
PS	61	M	15	1	24.3	3.6	145	1.53	0.85	109.4	1.6	high	8.3	5.7	0.03	30	11.9
PS	60	M	6	1	23	4.2		1.55	0.61	111	1.5	high		6.2			9.8
PS	73	F	14	1	16.9	3.9	125	1.09	1.07	98.36	1.5	high		3.6	0.26	28	9.9
PS	52	M	11	1	19.4	4	77	1.39	1.5	124.1	1.6	high	8.5	5.2	0.02	33	11.2
PS	62	M	5	1	21.8	4.2	132	0.82	1.14	155.7	1.3	high	8.7	4.7		30	9.2
PS	51	M	23	1	23.5	3.9	166	2.06	1.13	105.5	2.1	high	8.3	4.7	0.29		11
PS	56	M	2	1	21.8	4	131	1.54	0.86	128.9	2.1	high	10.2	5.7	0.14		12.3
PS	50	M	22	1	16.8	3.5	171	1.25	0.97	98.47	1.6	high	8.8	2.1	0.14	26	9.3
PS	62	M	18	1	21.4	3.7	187	1.69	0.82	127.9	1.6	high	9.6	5.1	0.23	21	10.7
PS	56	F	18	1	16	4.2	196	1.16	0.85	96.55	1.6	high		5	0.57	22	11.6
PS	67	F	15	1	17.7	4	126	1.39	1.17	125.8	1.6	high	8.6	7.7	1.04		10.7

PS	39	M	2	1	27.1	4		1.15	0.71	29.88	1.6	high		4.7	0.18	28	9.5
PS	62	M	7	1	21.4	3.6	186	1.5	0.98	116.9	1.6	high	7.9	2.9	0.63	39	12.7
PS	66	F	19	1	20.8	4.3	220	1.32	0.98	93.18	1.5	high	8.2	5.9	0.2		9.4
PS	60	F	9	1	28	4.2	113	1.07	0.9	129	1.6	high	8.3	3.9	10.8	27	
PS	55	F	16	1	24.6	4	147	1.52	0.93	136.9	1.9	high	9.5	7.7	0.08	22	10.8
PS	34	F	10	1	25.9	4	114	1.61	1.16	131.4	1.5	high	9	4			10.2
PS	62	M	12	1	17.6	4	111	1.92	0.66	104.4	1.9	high	8.8	5.4		41	12.3
PS	79	F	5	1	22.5	3.7	164	1.59	0.8	155.7	0.8	high	8.9	2.7	0.03	29	10.5
PS	65	F	22	1	16.8	3.4	159	1.33	1.13	112.6	1.9	high	10	5.3	0.7	26	10.2
PS	65	M	20	1	20.4	3.9	136	1.48	0.87	76.04	1.3	high	10.1	7.6	2.5	29	11.5
PS	56	M	3	1	26.5	3.8	189	1.58	0.84	102.4	1.5	high	9.2	4.4	0.7	29	10
PS	57	F	11	1	20.6	4.1	136	1.75	0.76	98.56	1.8	high	6.8	6.1	0.2	21	12.1
PS	71	F	17	1	21.5	3.6	192	1.29	0.81	94.49	1.8	high		5	0.2	32	11
PS	58	F	18	1	18.8	3.5	183	2.01	0.87	118.2	2.1	high	9.2	4.9	0	47	10.9
PS	66	F	16	1	22.8	3.7	123	1.4	1.12	119.5	1.8	high	9.9	5.6	0.03	24	10.8
PS	65	M	2	1	23.7	3.6	161	1.51	0.84	111.8	1.8	high	9.1	6.8	0.06		11.3
PS	65	F	16	1	24	3.4	178	1.71	0.66	130.3	1.4	high	9.2	4.2	0.11	23	10.2
PS	44	M	16	1	18	4	138	1.57	0.87	109.6	1.4	high	9.9	4.9	1.9	20	10.7
PS	58	M	17	1	18.5	3.6	143	1.67	0.86	109.3	1.8	high	9.2	5.8	0.17	34	11.4
PS	57	M	14	1	21	3.7	155	1.87	0.98	134.6	1.8	high	9.9	3.7	0.6	27	10.4
PS	45	F	13	1	16.5	3.5	107	1.17	1.01	83.58	1.8	high		4.8	0.1	19	11.1
PS	74	M	13	1	22.8	3.3		1.29	0.94	137.4	2.1	high	9.6	4.3			11
PS	68	F	13	1	28.3	3.4	152	1.37	0.75	146.6	1.3	high	7.6	4.2	0.13	20	12.2
PS	67	M	13	1	19.5	3.4	153	1.57	1.19	132.6	1.5	high	11.6	4.9	0.03	28	10.7
PS	49	M	14	1	17.5	3.8	186	1.54	1	101.1	1.3	high		6	0.1		11
PS	57	M	12	1	18.9	3.8	209	1.78	0.86	91.4	1.8	high		4.9	0.05	24	9.8
PS	45	M	12	1	22.4	4	141	1.5	0.91	126.6	1.3	high	8.1	5.8	0		11.6
PS	67	M	12	1	19.9	3.8	162	1.74	1.17	107.5	2.1	high		7.3	0.1	25	10.5
PS	54	F	12	1	20	4.1	151	1.09	1.17	103.2	1.5	high		6.2	0.24	25	9.1
PS	78	M	11	1	19.6	3.6	87	1.7	0.75	116.7	1.3	high	8.1	1.7	2.64	45	8.7
PS	69	M	10	1	26.6	3.7	132	1.24	0.91	89.83	1.5	high	8.7	4.5	0		10.4
PS	42	F	9	1	21.6	3.8	224	1.55	0.88	91.28	1.6	high	9.6	5.1	0.05	46	11
PS	83	M	9	1	18.6	3.9	95	1.44	1.04	108.8	1.8	high	9.4	6.1	0.4	31	10.2
PS	58	M	17	1	22.1	4.1	216	1.71	0.93	115	1.5	high	9.5	5.6	0.09	32	10.8
PS	74	M	10	1	19.9	3.9	178	1.22	0.91	125	2.5	high	11	5.6	0.05		11.3
PS	82	M	10	1	18.3	3.7	119	1.21	0.9	116.5	2.1	high	9.9	4.8	0.02		10.9
PS	57	M	10	1	22.7	3.3		1.5	0.82	88.6	2.5	high		8	0.84		10.7
PS	50	M	9	1	17.1	4.7	161	1.3	0.98	111.8	1.5	high		5.3	0	24	11.9
PS	45	F	9	1	18.5	3.9	99	1.67	1.04	112.4	1.8	high	8.7	4.4	0.1	31	8.7
PS	55	F	15	1	20.3	3.6	194	1.22	1.25	129.3	2.2	high	9	3.6	0.1	26	9.4
PS	57	F	9	1	19	4	152	1.31	1.1	129.8	1.3	high	9.5	4.5	0.09	24	9.2
PS	79	M	8	1	19.5	3.1		1.63	0.75	96.83	1.5	high	9.2	3.3	0.3	35	8.8
PS	67	F	8	1	25	3.7	149	1.35	0.86	137.7	1.5	high		3	0.48		9.9
PS	48	M	9	1	22.9	4.4	223	1.36	0.79	108.2	1.1	high	9.1	6.8	0	25	10.7
PS	77	M	8	1	18.3	3.9	197	2.13	0.99	98.59	2.1	high	8.6	4.8		17	11.1
PS	55	M	8	1	17.2	3.8	125	2.37	0.84	134.6	1.6	high	10.4	7.8	0.04	26	10.3
PS	77	F	8	1	23.6	3.5	245	1.55	0.96	105.7	1.5	high	7.9	5.5	0.1	34	10.5
PS	53	M	16	1	19.5	4.2	172	1.46	1.07	125.1	1.5	high	8.4	7.3	0.09	30	11
PS	66	F	17	1	17.6	3.3	121	1.07	1	97.24	1	high	9.3	4.8	0.09	46	10.5
PS	64	M	7	1	18.8	3.5	168	1.81	0.74	117.6	1.6	high	9.4	8.3	0.05	44	9.1
PS	75	F	7	1	16.6	3.7	164	1.3	1.1	125.8	1.8	high	9.7	6.4	0.1	23	10.8
PS	60	F	7	1	20.8	4	125	1.06	1.01	124.6	2.1	high		7	0.19	24	10.9
PS	63	F	14	1	20.9	3.7	81	1.35	0.8	153.3	2.1	high	9.7	7.1	0.06		6.4
PS	71	M	7	1	22.8	3.9	134	1.58	0.97	136.9	2.2	high	9.6	3.6	0.1	33	10.6
PS	83	F	6	1	22.9	3.7	125	1.16	0.7	147.5	1.5	high	9.3	3.8	0.23	26	11.3
PS	70	F	6	1	20.1	3.5	189	1.27	0.7	91.73	1.3	high	9.2	5.6	0.1		9.3
PS	58	M	6	1	26.1	3.6		1.49	0.83	121.6	2.1	high	9.9	3.9	2.14	23	9.9
PS	86	F	6	1	16.9	3.3	139	1.72	0.91	129.2	1.8	high	9	5.6	0.24		8.9
PS	58	M	6	1	20.2	4		1.46	1.02	91.14	1.5	high	9.8	6.4	0.1		9.4

PS	63	F	5	1	27.1	3.5	147	1.38	0.76	113.5	2.2	high	9.3	2.8	1	30	8.4
PS	68	M	5	1	17.3	3.6	144	1.33	1.08	136.3	1.3	high		4.7		36	10.1
PS	61	F	5	1	20.2	3.8	163	1.53	0.89	109.4	1.5	high	9	5	0.2	24	7.6
PS	70	M	5	1	27.2	3.8	172	1.92	0.8	60.34	1.8	high		6.6	0.75	27	10
PS	61	M	4	1	21.9	4.1		1.62	0.64	74.68	1.1	high	9.1	4.1			11
PS	65	M	5	1	22.1	4.2		1.33	0.89	102.3	2.1	high	9.5	5	0.1		10.5
PS	68	M	4	1	26.7	3.8	113	0.91	0.99	137.5	1.3	high	9	6.1	1.02	17	9.3
PS	61	M	4	1	21.5	3.4	199	1.44	0.81	104.7	1.5	high	9.5	4.7	0.4		10.4
PS	71	M	4	1	22.1	3.4		1.76	1.03	115.8	1.5	high	8	4.5	0.06		11.4
PS	59	F	14	1	19.4	4		1.41	1.01	112	1.5	high	8.1	4.4		27	10.1
PS	60	M	4	1	21.2	4.1		1.34	0.72	91.39	2.1	high	9.7	6.7	0.1		11
PS	56	M	4	1	19.5	4		1.71	0.7	126.1	1.5	high	8.5	5.6	0.1		10.3
PS	64	M	7	1	25.2	3.3	140	1.2	0.72	105.2	1.5	high	10.6	4.5	0.21	17	11
PS	49	M	4	1	24	4.1	168	1.41	1.39	121.5	1.8	high	9.3	7.5		28	9.6
PS	49	F	17	1	16.9	4.2	187	1.43	1.02	111.7	1.5	high	9.9	3.5	0.01	15	11.5
PS	80	M	5	1	26.6	3.6		1.39	0.97	98.04	1.8	high	9.3	5.4	0.03	24	12.3
PS	42	F	10	1	26.6	3.5		0.9	1.03	100.7	2.1	high	9.2	4.7	0.01	23	10
PS	67	M	4	1	22.3	3.7	189	1.28	1.1	107.6	1.6	high	7.9	4.5	0.1	23	10.7
PS	76	F	4	1	18.8	3.7		2.05	1.07	111.3	1.5	high	9.3	3.6	0.1		11.2
PS	63	F	3	1	22.1	4	130	1.45	1.26	130.4	2.1	high	9.4	2.3	6.04	27	8.8
PS	69	M	6	1	23.6	3.8	219	1.75	0.87	93.26	2.1	high	10.4	7.3	0.05	29	10.5
PS	70	F	8	1	35.3	2.7	168	1.84	0.73	80.53	1.6	high	9.9	5.9	0.05	24	10.2
PS	55	M	3	1	25.1	4	167	1.16	0.66	125.1	1.8	high	8.8	3.5	0.5	21	10.5
PS	31	F	3	1	17.6	4	156	1.36	0.66	75.27	2.1	high	9.4	6	0		8.6
PS	68	F	11	1	27	3.8	164	1.64	1.03	113.5	1.6	high	10.4	6.7	0.05	24	10.9
PS	69	M	3	1	20.2	3.7	103	1.23	0.89	139.3	2.1	high	9.1	6.6	0.03		11.1
PS	70	M	3	1	22.3	3.8	169	1.39	0.88	98.31	2.1	high	9.2	5.8	0.02		11.4
PS	71	F	2	1	27.7	3.6	184	1.73	0.98	66.51	1.8	high	8.9	4.7			11.8
PS	59	F	15	1	17	4.1	164	1.38	1.03	116.9	1.8	high	8.8	6.2	0.1	22	9.5
PS	79	F	8	1	23.2	3.1	122	1.33	1.05	151.7	1.8	high	9	7.1	0.05		10.8
PS	59	M	3	1	27.3	3.9	145	1.65	0.95	139.2	1.8	high		6.7	0	28	12.3
PS	71	F	6	1	24.9	3.4	139	1.76	0.79	73.81	1.6	high	9.7	5.7	0.22	22	10
PS	46	M	2	1	20.5	4.3	111	0.72	0.78	92.56	2.1	high	10.2	6	0.8	19	10.5
PS	56	M	9	1	25	3.8	110	1.2	0.7	133.3	1.8	high		5.6	0.1	36	11.9
PS	47	M	2	1	26.3	4.3	160	1.06	0.92	108	2.1	high	9.2	5.7	0.05		9.5
PS	88	F	3	1	19.4	3	175	1.32	0.94	129.2	1.5	high	9.1	5.2	0.45	37	10.7
PS	64	M	2	1	18.6	4.1	165	1.63	0.84	84.09	1.6	high	8.1	5	0.19	26	10
PS	67	M	2	1	23.8	3.3	205	1.53	0.94	99.9	1.4	high	8.7	3.3	0.1	26	9.5
PS	65	M	2	1	22.7	4.2	185	1.4	0.87	143.8	1.3	high	9.2	6.1	0.07	24	9
PS	76	M	2	1	17.7	3.3	214	1.73	0.81	81.67	1.4	high	9.5	6.1	1	25	12.1
PS	64	M	17	1	22.4	3.2	170	1.28	0.84	105.7	2.1	high	8.8	5.9	0.2	33	10.1
PS	71	M	17	1	16.9	3.2	195	1.58	0.69	104.5	1.5	high	7.6	5.1	0.21	25	11
PS	51	F	24	1	25.7	3.8	195	2.07	0.88	125.4	1.5	high	9.4	4.2	0.05	27	11.1
PS	42	F	16	1	23	3.8	217	1.85	0.97	128.1	2.2	high	9.2	4.8	0.05	37	11.9
PS	68	F	2	1	19.2	3.4	134	1.62	1.01	128.7	1.5	high	8.8	6.6	0.1	30	10.8
PS	58	F	2	1	18.6	4	99	1.41	1.06	121.5	1.5	high	9.7	6	1.53	24	10.5
PS	74	M	3	1	17.9	3.1	205	1.38	0.76	112.5	1.5	high	9.2	4.6	1	30	11.8
PS	68	F	18	1	18.7	3.7	137	1.2	0.55	117	1.5	high	7.4	3.5	0.06	21	11.5
PS	75	M	4	1	26.3	3.9		1.29	0.77	129.6	1.5	high	9.2	5.2	0.1		9.8
PS	51	F	2	1	22.5	3.4	163	1.2	0.93	76.35	2.1	high	9	5.4	0.3	34	10.1
PS	51	F	3	1	14.7	3.5	111	1.81	0.77	73.24	1.8	high	8.9	8	0.3	26	9.6
PS	78	F	3	1	16.4	3.4	209	1.43	0.77	109.2	1.4	high	10.1	5.2	0.2	28	11.2
PS	68	F	3	1	19.6	3.6	144	1.13	1.05	86.37	1.5	high	9.3	3.4	1.4		10.3
PS	60	M	3	1	26.5	3.5	259	0.74	0.72	82.24	1.5	high	8.9	5.7	0.06	16	9.8
PS	69	F	13	1	23.2	3.9	216	1.03	0.89	91.36	1.8	high		3.3	0.05		
PS	59	M	13	1	19.1	3.9	144	2.32	0.7	117.6	1.8	high	7.4	3.1	0		9.4
PS	51	F	9	1	21.9	2.7		1.45	0.43	51.02	1.5	high		5.4			7.6
PS	42	M	7	1	23.3	3.9	151	1.19	0.95	119.9	1.5	high	9.3	6.9	0.9		10.5
PS	56	M	2	1	20	3.7	175	1.37	0.77	67.65	2.1	high	9.7	7.1	0.3	26	9.9

PS	70	M	6	1	18.6		136	1.6	0.75	138.5	2.1	high	8.6	5.5	0.03		10.1
PS	80	M	3	1	25	3.5		1.81	0.82	111	2.1	high	10.2	4.6	1.05	32	11.1
PS	69	M	4	1	22.6	3.8	156	1.53	1.15	175.7	2.1	high	9.2	4.8	0.33	27	11.1
PS	74	M	7	1	19.6	3.6	164	1.33	0.74	122.4	1.6	high	7.9	4.3	0.07		8.5
PS	72	F	3	1	28.8	3.6	110	1.25	0.71	69.52	1.8	high	7.6	5.6	0.25	44	11.8
PS	59	F	21	1	23.9	4.3	134	1.15	0.82	106.4	1.3	high	9	3.1	0.07	22	10.1
PS	64	F	4	1	27.9	4.1	121	1.72	0.58	47.52	1.5	high	8	6.6	0.28	33	11.1
PS	62	M	15	1	20.2	4.1		1.3	0.95	95.53	2.1	high	9.4	6.1		29	9.5
PS	67	M	5	1	22.9	4	135	1.89	0.68	100.8	1.6	high	9.5	9.3			7.9
PS	65	F	14	1	16.1	4.1	201	1.76	0.89	97.5	1.6	high	8.7	2.7	0.19	24	10.1
PS	64	F	21	1	21.3	4.3	173		0.83	99.63	2.1	high	11.3	4.2	0.06	31	10.9
PS	58	M	8	1	19.3	4.3	99	1.31	1.17	91.27	2.1	high	9.5	3.9	0.22	24	11.2
PS	79	F	4	1	19.3	3.8	155	1.88	0.5	63.04	1.5	high	7.4	4.5	0	34	9.8
PS	58	F	12	1	17.3	4	150	1.35	1.04	114.1	1.9	high	9.2	5.2		22	10.7
PS	62	F	11	1	20.9	4.2	131	1.17	0.87	93.73	1.5	high	7.7	5.6	0.18	31	10.9
PS	67	F	9	1	27.3	3.8	161	1.7	0.68	90.8	1.5	high	8.7	5.4	0.07	25	9.9
PS	79	M	3	1	19.2	3.7		1.07	0.93	44.57	1.8	high	9.5	6.4		17	10.7
PS	68	F	3	1	17.8	4.3		1.29	0.82	81.7	2.1	high	9.7	4.7		34	10.2
PS	70	F	26	1	18.3	3.9	135	1.1	1.21	108.9	1	high	6.3	5.8	1.3	32	10.2
PS	61	M	19	1	18.6	4.2	109	1.31	0.95	89.86	2.1	high	9.1	4.6	0.11	24	10.1
PS	74	M	4	1	22.4	4		1.16	1.09	103.9	1.1	high	9	5.6	0.74		10.4
PS	71	F	12	1	17.7	4	90	0.88	1.03	130.7	1.8	high	9	7.5	0.9	44	9.9
PS	58	F	3	1	17.7	4.3	168	2	1.3	105.5	1.5	high	8	3.5		24	11.6
PS	69	F	23	1	22	3.8	147	1.43	1.14	87.14	1.6	high	9.7	3.5		36	7.6
PS	74	F	18	1	18.8	4.1	117	1.29	1.13	110.2	1.3	high	7.8	4.7	0.07	25	10
PS	42	F	7	1	17.4	4	184	1.67	0.84	102.1	1.5	high	8.9	7.8	0.03	26	10.9
PS	73	F	22	1	22.2	4.1	115	1.67	0.8	101.6	1	high	9.4	4.7	0.05	40	11
PS	56	F	7	1	24.1	4	150	1.31	0.91	95.56	1.6	high	9.3	5.6	0.05		10.9
PS	56	M	13	1	21.3	4.4	122	1.63	0.74	84.96	1.6	high	9.1	6.7	0.1	21	11.1
PS	69	F	12	1	23.8	3.7		0.99	0.81	97.51	1.3	high	8.7	4.2	5.07		9.9
PS	75	M	5	1	22.5	4.5	151	1.5	0.76	99.11	1.5	high	8.9	3.4	0.06	26	10
PS	62	M	11	1	20.4	4.2	130	1.31	0.93	110.3	1.3	high	9	6.4	0.4	19	8.5
PS	63	F	13	1	20.1	4.5	148	1.35	0.95	136.4	2.1	high	9.9	5.4	0.05	19	10.4
PS	67	M	11	1	18.9		168	1.66	1.04	75.09	1.6	high	8.6	6.6	0	26	10
PS	51	M	19	1	18.3	3.6		1.45	1.15	87.44	2.5	high	8.3	4.9	5.2	25	9.9
PS	65	M	9	1	16.5	3.7	150	1.2	0.93	78.94	1.5	high		3.8	0.1	21	8.6
PS	73	M	6	1	23.5	4.4	133	1.13	0.94	124.5	1.9	high	9.4	6.1	0.1	30	10.4
PS	65	F	7	1	24.1	3.6	99	0.9	0.79	121.2	2	high	8.8	6.1		27	9.7
PS	64	M	5	0	17.1	3.5	175	1.56	0.81	51.04	1.5	high	9.2	4.7	0.01	23	10.6
PS	66	M	11	1	21.9	4	205	1.21	0.82	129.8	1.8	high	10.2	7.4	0.14	17	13.1
PS	52	F	2	1	25.7	3.9		1.88	1.33	98.17	1.1	high	9.6	4.2			9.8
PS	49	F	3	1	20.4	3.7	119	1.14	1.13	122.8	2.1	high	7.7	6.7			11.1
PS	46	F	4	1	19.6	4.5	121	1.27	1.1	117.2	1.5	high	8.8	5	0.3	35	9.8
PS	71	M	11	1	23.6	4	118	1.74	0.7	103.9	1.6	high	9.8	3.2	0.4	28	10.3
PS	60	M	18	1	18.3			1.74	0.97	98.35	1.3	high	10.7	3.5		26	11.1
PS	59	M	17	1	21.6		147	2.94	1.29	135	1.8	high	9	4	0		10.9
PS	58	F	22	1	21.3			1.3	1.11	124.1	1.6	high	8.3	4.6	0.17	24	10.4
PS	60	M	16	1	23.4		155	1.27	0.95	107.2	1.6	high	9.2	3.8	0.05	21	11
PS	56	F	15	1	18.5		163	2.88	0.98	139.5	1.8	high	8.2	3.8	0		9.6
PS	55	F	28	1	16.1		173	1.29	1.1	88.95	1.6	high	10	4.5	0.06	27	10.6
PS	52	F	15	1	16.9		190	2.59	1.1	122	1.8	high	9.8	5.8	0.1		10.6
PS	66	F	14	1	18.4		170	1.76	1.04	117.1	1.8	high	8.8	5.7	0.1	22	10.2
PS	45	M	18	1	17.3		183	1.51	0.93	87.13	1.3	high	8.1	4.9	0.05	22	10.1
PS	70	M	14	1	17.5		136	1.59	0.98	88.11	1.8	high	8.1	3.1	0.1	36	9.5
PS	40	F	19	1	21.1		182	2.81	0.96	140.9	1.8	high	8	4.8	0		10.5
PS	71	M	8	1	22.7		144	1.71	1.09	26.24	1.3	high	8.1	4.7	0.05	28	9.6
PS	61	M	7	1	22.2		132	1.67	0.77	102.9	1.8	high	8.2	5.6	0.12	28	10
PS	44	M	7	1	24		103	1.21	1.1	57.9	1.8	high	9	5.7	0.08	31	10.6
PS	71	F	6	1	18		182	2.13	1.08	139.9	1.6	high	8.7	5.3	0.06		9.6

PS	43	M	5	1	22		117	1.47	0.89	153.6	1.6	high	8.1	6.2	0.14	25	10
PS	59	M	5	1	38.1	3.8	147	1.49	0.78	110.5	2.1	high	10	4.6	0.3	30	10.2
PS	54	F	6	1	25.2		118	1.48	0.95	107.9	2.1	high		4			7.5
PS	62	M	8	1	23		176	1.86	0.77	94.19	1.5	high	9.5	7.8	0.07	22	12
PS	64	M	3	1	21.2		154	0.71	0.78	117.3	1.8	high		9.5		19	11.1
PS	63	M	4	1	21.1		179	1.13	0.89	106.5	2.1	high	9	4.7	1.06	24	10.7
PS	51	M	2	1	23.5		157	1.2	0.83	117.7	1.5	high	8.8	4.6	0.57	26	10.5
PS	65	M	2	1	21.1	3.7	150	1.55	1.03	129.3	1.6	high	7.9	5.3	0.05	23	11.5
PS	36	M	2	1	26		119	1.47	1.01	91.65	1.5	high	8.1	3.6	0.54	27	9.3
PS	53	F	11	1	21.6		207	1.1	1.24	104.3	1.3	high	10.2	5.9	0.17	29	
PS	71	F	6	1	32.2	3.7	168	1.41	0.92	109	1.3	high	9.3	2.6		32	12.1
PS	71	M	10	1	20.9	3.5		1.38	1.1	90.24	2.1	high		5.6	0.1	29	10.8
PS	60	M	9	1	24.3	4.1	197	1.27	0.84	90.57	1.5	high	9.5	4.6		31	12.3
PS	68	F	7	1	18	4.1	153	1.18	0.84	106.7	1.6	high	9.6	5.2	0.5	29	12.2
PS	65	F	25	1	23.1	3.5	198	1.65	0.95	105.2	1.3	high	9.9	7.5	0.1	27	11.6
PS	57	F	5	1	24.8	3.9	144	1.64	0.56	117.6	1.5	high	7.7	5.9		21	10.9
PS	60	F	18	1	21.6	3.7	161	1.4	0.84	108	1.1	high	10.2	9.5		19	11.7
PS	60	M	32	1	20.9	3.1	160	1.41	1.05	78.03	1.9	high	9	5.5	0.1	18	10.1
PS	60	M	29	1	22.3	3.5	177	1.67	0.84	81.38	1.9	high	9.9	5.3	0	22	11.4
PS	54	M	27	1	20.7	4.1	194	1.64	1.07	128.4	1.3	high	9.7	5.9	0	24	10.2
PS	69	M	24	1	20.9	3.3	149	1.7	0.86	129.8	1.6	high	8.7	5.5	0	29	9.6
PS	66	M	19	1	25.6	3.5	149	1.42	1	131.2	1.9	high	9.6	5.4	0.1	33	12.2
PS	41	M	17	1	22.5	4.1	141	1.21	0.97	116.9	1.5	high	9.6	7.4	0.17	24	13.8
PS	60	F	17	1	20.8	3.7	275	1.39	1.02	115.3	1.6	high	9.7	4.1	2.42	21	7.8
PS	70	F	16	1	21	3.3	141	1.74	0.9	126.7	1.5	high		5.4	0.05	31	10.8
PS	73	F	14	1	17.4	3.9	163	1.69	1.09	122.3	1.6	high	9.3	5.4	0	30	11
PS	53	M	13	1	22.7	3.7	156	1.4	0.96	113.2	1.5	high	10.2	7.8	0.5	28	11.8
PS	80	M	11	1	21.5	3.5	135	1.44	0.72	130.8	1.5	high	9.6	6.8	0.1	28	10.8
PS	61	M	7	1	21.2	3.8		1.2	0.93	112.1	1.8	high	9.2	5.8	0.2	28	11
PS	41	F	2	1	18.8	4.1		1.1	1.07	106.3	1.5	high	7.9	6.3	0	24	10.5
PS	61	F	32	1	20.1	3.8	160	1.26	0.95	99.12	2.1	high	8.8	6.6			9.4
PS	55	F	10	1	22	4	126	1.33	1.14	109.4	2.1	high		5.2	0.67	27	11.4
PS	51	M	23	1	20.4	3.8	189	1.35	0.89	100.7	1.9	high	9.5	6.8	0.1	24	11.5
PS	68	M	20	1	20.5	3.9	142	1.25	0.78	127.5	1.9	high	8.7	4.4	0	18	10.2
PS	53	M	20	1	20	3.8	175	1.6	0.74	112.8	1.9	high	9.1	5.5	0.8	27	10.3
PS	59	M	18	1	21.5	4	123	1.38	0.89	98.77	1.9	high	10.3	5.1	0	41	9.9
PS	54	M	17	1	19.3	3.8	103	1.35	0.89	129.9	1.9	high	9.6	5.4	0.42	21	9.1
PS	67	F	13	1	22.6	4	165	1.24	0.94	97.3	1.9	high	8.9	4.6	1.06	30	9.8
PS	64	M	11	1	23.2	3.9	192	1.58	1.1	148.5	1.3	high	9.5	5.3	0.2	21	10.5
PS	53	M	5	1	19.1	4.1	137	1.68	0.99	143.3	1.9	high	8.9	5	0.44	27	9.7
PS	67	M	5	1	20.5	4.2	154	1.3	0.98	95.13	1.9	high	9.5	6.6	0.22	28	11.2
PS	76	M	5	1	23.5	3.8	185	1.2	0.74	123.6	1.8	high		6.3	2.2	22	10.8
PS	76	F	3	1	26.5	3.9	116	1.24	1.18	96.66	1.9	high	9.7	4.6	0.02	23	10.1
PS	68	F	3	1	18.5	3.8	112	2.07	1.41	121.6	1.9	high	9.8	6.3	0.03	26	10.2
PS	28	F	2	1	16.1	4.3	187	1.44	0.95	71.27	1.8	high	10.4	7.2		32	8.7
PS	46	F	2	1	21.1	4.8	144	1.45	0.74	90.83	1.9	high	9.6	7	0.06	30	10.3
PS	59	M	2	1	20.1	4.3	159	1.76	1.12	101	2.1	high	8.6	5.1	0.14	24	10.1
PS	42	M	2	1	21.5	4	118	1.58	0.76	123.4	1.6	high	10.1	5.1	0.11	31	10.4
PS	65	M	2	1	20.3	4.5	131	1.31	1.24	136.1	1.9	high	9.8	4.5	0.03	29	10.7
PS	81	F	7	1	20.6	3.6	128	1.18	1.27	96.9	1.9	high	8.5	2.9	0.06	23	9.3
PS	55	M	26	1	22.4	3.9	163	1.38	1.02	142.9	1.9	high	8.6	6.4	0.05	31	10.5
PS	53	M	23	1	23.3	3.8	153	1.62	0.9	130.3	1.5	high		3.6	1.77	50	11.6
PS	60	F	21	1	18.9	3.5		1.75	0.98	128.8	1.8	high	9.1	6.3	0.12	22	11.2
PS	54	M	15	1	25	3.4	177	1.44	0.91	110.5	1.8	high	9	4.6	0.04	18	10.8
PS	58	F	13	1	16.3	3.4	127	1.32	1.02	74.51	1.1	high	9	4	0.1	29	9.5
PS	70	F	10	1	16.2	3.6		1.42	1.12	100.1	1.8	high	10	4.3	0.44	23	11.4
PS	49	F	7	1	19.6	3.5	140	0.85	0.82	139.8	2.1	high	8.9	5.5	1.5		7.8
PS	77	M	7	1	24.3	3.5	119	1.83	0.94	106.8	1.3	high	8.5	4.2	0.14		10.8
PS	60	F	7	1	22	3.8	72	1.73	1.15	129.4	2	high	9	3.5	12.36	40	8.3

PS	35	M	6	1	14.1	4.2	184	1.36	1.45	114.4	2	high	8.5	6.6		17	9.6
PS	52	F	5	1	18.5	3.9		1.14	1.04	137.5	2.1	high	9	6	0.05	23	11.1
PS	72	M	5	1	26.8	3.9	119	1.61	0.96	115.4	1.6	high	9.2	4.6	0.07	26	10.4
PS	74	M	4	1	18.8	3.2	177	1.29	0.91	125.4	1.8	high	8.6	5.7		44	11
PS	78	M	4	1	21.7	4.1	72	1.76	0.92	103.6	1.8	high	8.2	3.9		29	11.6
PS	36	F	3	1	27.8	3.7	188	1.86	1.08	135.4	1.9	high	10.1	6.4	0.04	23	10.5
PS	61	F	2	1	22.6	4	180	1.59	1	126.7	1.6	high	9.5	3.5	0.2		
PS	69	M	2	1	20.3	3.9	78	0.95	0.82	104.7	0.8	high	8.6	4.3			11.3
PS	46	F	2	1	17.1	3.5	133	1.37	1.19	108	1.5	high	8.3	4.3	2.6	26	11.3
PS	65	F	32	1	16.7	3.9	142	1.45	1.07	94.72	2.2	high	8.6	4.5	0.06	29	10.8
PS	72	M	21	1	19.1	3.2	86	1.14	0.82	83.9	2.1	high	8.3	7.4	0.1	46	11.5
PS	57	M	21	1	19.2	4.1	262	1.35	0.78	123.8	1.8	high	10.2	5.9	0.1		9.2
PS	58	F	13	1	21.4	3.7	131	1.34	1.14	142.7	2.1	high	8.6	6.1	0.1	25	11.3
PS	71	M	11	1	22.9	3.7	139	1.15	0.8	135.6	1.6	high	8.4	4.1	0.48	28	11.5
PS	73	F	10	1	21	4.2	129	1.09	0.82	109.4	2.1	high	9.2	3.5	0.54	31	10.2
PS	52	M	9	1	15.5	4.2	137	1.33	1.16	110	1.8	high	8.2	8.1		36	11.3
PS	54	M	9	1	20.3	4.1	209	1.33	0.92	120	2.1	high		6.3	0.19	29	11.7
PS	68	F	8	1	17.8	3.8	134	1.76	0.82	86.75	1.5	high	8.7	4.1	0.17	32	11.7
PS	57	F	7	1	26.1	4.2	220	1.5	0.93	105.2	1.6	high	9.6	6.1		27	10.4
PS	58	M	7	1	21.3	4.1	231	0.94	0.76	84.44	2.1	high	10.1	6.6	0.11	29	10.9
PS	64	F	6	1	21.6	3.7	212	1.53	0.97	142.5	1.5	high	9.7	5.8	0.06	24	10.7
PS	55	M	5	1	22.8	4.1	135	1.44	0.94	134	1.5	high	9	5.5	0.02	32	10
PS	61	M	4	1	20	3.9	81	1.08	1.03	81.87	1.6	high	8.6	6.2	0.2	25	7.7
PS	78	M	4	1	19.9	4.2	218	1.57	1.09	133.6	1.6	high	7.7	4.3	0	20	10.6
PS	64	M	4	1	22.8	4	102	0.84	1.1	120.2	2.5	high	7.9	7.2	0.11	31	10.8
PS	43	M	3	1	18.3	4	149	1.27	0.97	123.9	1.6	high	10	4.8	0	38	10.7
PS	35	F	13	1	22.8	3.8	101	1.27	0.71	81.36	1.9	high	8.1	5.7	0.07	23	10
PS	60	F	12	1	27.6	3.5	164	1.03	0.93	122.1	1.8	high	7.6	5	0.02	18	11.2
PS	47	F	9	1	17.6	3.4	180	1.19	0.83	105.8	1.9	high	9.2	5.4	1.31	27	9.8
PS	48	F	8	1	20.5	3.6	154	1.06	0.93	97.97	2.1	high	9.4	4.8	0.02	28	11.7
PS	40	M	6	1	21.1	3.8	178	1.4	0.95	101.8	1.3	high	10.4	5.6			12.2
PS	70	F	5	1	19.5	3.6	156	1.28	0.8	86.73	1.8	high	9.7	4.2	0.78		9.9
PS	65	F	3	1	21.3	3.4	157	1.85	0.55	101.7	1.6	high	8.3	4.4	0.08	25	10
PS	75	M	2	1	22	3.3	121	1.1	0.9	103.8	1.3	high	9.4	5	1.24	29	10.1
PS	68	M	21	1	20.1	3.2	114	1.2	0.78	118.8	1.9	high	9.8	6.7	0.26	17	10.3
PS	47	M	14	1	21.9	3.7	104	1.48	0.88	97.61	1.9	high	9.4	6.9	0.07	23	11
PS	45	M	14	1	19.7	4	139	1.51	0.88	91.06	1.3	high		7	0.17	32	10.8
PS	84	M	11	1	18.3	3.8	128	1.55	1.07	112.9	1.5	high	9	5.3	1		9.8
PS	73	M	10	1	15.2	3.9	153	1.48	0.88	75.98	1.5	high	9.9	5.3	1.28	22	11.4
PS	77	M	4	1	22.8	3.5	203	2.05	0.73	107.3	1.1	high	9	4.6			9.9
PS	76	M	4	1	20.7	3.8	145	1.54	0.82	102.5	1.5	high	9.1	4.2		38	9.9
PS	48	M	3	1	24	3.5	226	1.52	0.93	63.33	1.6	high	9.1	4.9	0.1	27	10.1
PS	64	F	2	1	22.9	3.7		1.41	0.66	107.7	1.3	high	7.9	4.6	0.38	30	9.3
PS	50	F	9	1	22.6	3.9	175	1.73	0.79	75.33	1.5	high		5.4		24	10
PS	33	F	5	1	22.8	2.7	105	1.45	0.92	79.93	2	high		5.2	0.21	26	9.3
PS	43	M	2	1	22.1	3.4	127	1.42	0.65	71.24	1.8	high	9	4.4		27	10.5
PS	52	M	2	1	24.5	4.1	152	1.31	0.64	71.39	1.5	high	8.6	4.3		17	10.7
PS	67	F	2	1	23.9	3.2	100	1.35	0.75	85.34	1.8	high	7.5	5	0.15	24	12.3
PS	60	F	21	1	17.7	2.9	155	1.3	0.9	82.84	1.3	high	9.3	6.5	0.06	22	10.7
PS	65	M	11	1	14.9	3.5	133	1.33	0.78	85.03	1.5	high	8.3	6.3	0.1	26	11.2
PS	52	F	9	1	18.3	3.8	116	1.53	0.82	76.13	1.9	high	8.9	5.2	0.18	23	9.8
PS	69	M	6	1	21.5	3.7	153	1.53	1.06	121.1	2.1	high	9.1	8.8			9.3
PS	67	F	3	1		3.7	157	1.53	1.03	77.14	2.1	high	8.9	6.8	0.8		12
PS	64	M	11	1	20.8	3.7	143	1.69	0.96	116.9	2.1	high	8.9	4.7	0.04	23	10.5
PS	64	F	6	1	18.8	4	157	2.5	0.83	115.7	1.8	high	8.2	4.6	0.1		10.4
PS	73	M	10	1	19.8	4.1		1.15	1.21	149.7	1.5	high			6.04		9.5
PS	74	F	7	1	20.1	3.4	142	2.64	0.76	124.7	1.8	high	8	4.2	0		9.8
PS	42	M	9	1	25.5	4	204	1.57	0.63	88.64	1.5	high	10.1	7.6		30	9.3
PS	64	M	5	1	24.2	3.9		1.77	1.02	141.3	1.8	high	8.8	4.5			10.1

PS	61	M	11	1	21.5	4.2	139	1.14	0.95	125.8	1.8	high	9.9	3.9	0.11	13	10.8
PS	58	F	9	1	17.6	4.3	202	2.95	0.97	132.8	1.8	high	9.6	3.5	0.1		8.6
PS	76	F	12	1	22.1	2.5	133	1.01	0.58	56.52	1.5	high		4.7	0.82	30	9.9
PS	53	M	19	1	18.3	3.9	139	1.36	0.95	150.3	1.8	high	9.4	5.4	0.2	33	9.1
PS	62	M	6	1	22	3.9	159	1.52	0.69	89.2	2.1	high	8.2	3.9		26	10.7
PS	63	F	12	1	17.9	4.1	133	1.52	0.9	136.2	1.1	high		4.6	0.25	27	10.3
PS	74	F	8	1	18.1	3.2	189	2.69	0.79	98.17	1.8	high	8.1	6.1	0.3		6.9
PS	63	F	19	1	25.5	3.8	142	1.87	1.05	110.6	1.4	high	8.4	3.6	0.07	35	10.8
PS	60	F	3	1	19.1	3.5	163	1.73	0.81	109	1.4	high	11.3	3.1	0.1	25	7
PS	65	M	27	1	19.5	3.3	131	1.43	1.06	110.1	1.5	high	9	4.7	0.1	17	10.4
PS	64	F	24	1	20	4.3	143	1.83	1.17	106.5	2.1	high	7.5	5.4	0.1	26	11
PS	58	F	21	1	23.6	4.2	146	1.88	1.01	132.2	1.4	high	9.4	7.5	0.1		11.2
PS	66	M	4	1	21.4	3.5	162	0.95	0.79	105.6	1.1	high	8.5	0.8	0.36	44	8.7
PS	71	F	5	1	22	3.3	150	1.92	1.05	113.6	1.5	high	10.2	4.2	0.27	28	11.7
PS	73	M	4	1	19.8	3.4	131	1.71	0.96	45.26	1.6	high	9.1	5.3	0.05	23	11
PS	55	F	2	1	16.1	3.6	157	1.81	0.94	72.11	2.1	high		8.3	0.06	42	12.8
PS	64	M	14	1	19.6	3.8	79	1.19	1.04	118.9	2.1	high		4.8	4.44	26	10.2
PS	60	M	6	1	31.3	3.9	148	1.49	1.07	86.04	1.5	high	9.3	6.3	3.84	21	10.4
PS	76	M	10	1	22.3	3.6	76	1.3	0.84	100.7	1.8	high	9.1	5.3	0.06	37	10.3
PS	63	F	7	1	16.8	3.8	133	1.02	1.09	107.1	2.1	high	9.8	5.7	0.15	29	9.7
PS	58	F	3	1	28.7	3.6	153	1.62	0.71	54.7	1.8	high	8.5	4.2	1.22	34	11.4
PS	73	M	4	1	24.6	3.5	176	1.62	1.19	112.5	1.6	high	8.2	6.6	0.08	24	10.6
PS	61	M	18	1	21.7	3.8	140	2.04	0.77	90.04	1	high	9.1	4.2	2.12	25	8.3
PS	56	M	3	1	23.1	3.7	198	1.58	0.96	85.19	1	high	9	3.9	0.11	25	12.2
PS	68	F	8	1	18.2	3.8	187	1.12	1.28	106.1	1.9	high	8.9	6	0.1		9.8
PS	62	M	9	1	25.7	3.9	116	1.34	0.87	131.5	1.9	high	9.2	5.1	0.53	27	9.2
PS	63	F	12	1	18.5	3.8	197	1.45	1.1	125.5	2.1	high	7.6	6.1	0.22	29	10.3
PS	71	M	11	1	23.8	3.3	133	1.35	0.74	198.4	1.9	high	9.1	3.9	0.08	26	9.3
PS	64	M	11	1	24.4	3.7		1.37	0.86	139	1.6	high	9.1	2.7	0	18	9.7
PS	59	M	26	1	19.6	3.3	145	1.19	0.89	110.9	1.9	high	9.7	7.8	0.03	23	12.6
PS	69	F	10	1	21.7	3.6	103	1.43	0.83	127	1.5	high		3.8		28	7.9
PS	66	M	8	1	21.7	3.8	116	1.24	0.93	131.6	1.9	high	9	7.1	0.04	24	10.3
PS	57	M	8	1	24.2	3.9	181	1.66	1.1	160.3	1.9	high	8.7	4.3	0.03	18	9.4
PS	80	M	7	1	24.5	3.6	103	1.54	0.9	127.7	1.5	high	8	6.1			9.7
PS	74	M	10	1	19.7	3.9	151	1.64	0.94	137.4	1.8	high		5.9		27	10.4
PS	71	M	7	1	21.9	3.6	146	1.13	0.77	110.4	1.8	high	8.8	4.8			11.5
PS	59	M	9	1	22.4	3.6	121	1.29	0.82	118.2	1.6	high	9	4.9	3.69	36	7.2
PS	75	F	20	1	17	3.5	200	1.67	0.86	98.58	1.9	high	7.9	5.5	0.01	21	10.5
PS	75	M	7	1	24.2	3.6	163	1.12	0.93	128.9	1.6	high		6.9	0.1	25	11.1
PS	57	M	10	1	25.9	3.7	144	1.4	0.97	155.9	1.3	high	9.2	3.7	0.1	30	12.2
PS	55	M	9	1	17.2	3.7	159	1.81	1.05	122.4	1.6	high	8.4	4.6	0.1	31	14.8
PS	36	M	7	1	18.3	4.1	153	1.27	0.89	79.13	1.6	high	10.2	4.1	0.1	25	10.8
PS	58	M	7	1	20	3.9	68	1.62	0.94	148.1	1.5	high	7.6	6.7	1.53	30	9.3
PS	51	M	7	1	21.8	4.2	166	1.05	0.79	125.6	1.8	high	6.9	5.3	0.3	28	11.4
PS	58	M	7	1	20.2	3.3	162	1.35	0.64	124.9	1.8	high	7.9	3			10.7
PS	81	M	12	1	17.9	3.4		1.23	1.15	90.86	2.1	high	9.8	6.9			11.7
PS	60	F	5	1	29.4	3.6	121	1.38	0.87	113.9	2.1	high		8.2		28	8.5
PS	54	M	6	1	23.3	3.6	128	1.14	1.06	128.9	1.1	high	7.9	4.3			11.1
PS	71	M	4	1	21.3	3.8	231	1.62	0.78	127.6	1.6	high	8.8	5.7	0.3	24	9.6
PS	60	F	5	1	17	4	142	1.71	0.94	114.8	1.3	high	8.7	3.6	0.1	31	10.5
PS	62	M	2	1	17.3	3.7	108	1.47	1.08	108.1	1.3	high	9.2	3.8	0.38	27	9.6
PS	69	F	6	1	21.4	3.2		1.31	0.92	106.3	0.6	high	9.3	3.3	0.05		10
PS	76	M	3	1	21.8	3.6	120	1.52	0.73	98.55	1.6	high		5	0.1	31	12.2
PS	40	M	6	1	26	3.9		0.89	0.82	123.6	0.6	high	9.1	5.3	0.06		9.4
PS	56	M	6	1	21.2	3.7	143	1.33	1.01	101.8	0.6	high	8.1	4.4	1.62		9.9
PS	73	M	5	1	24.1	3.8	165	1.27	0.93	120.5	1.1	high	8.3	4.7	0.16	34	10
PS	63	F	6	1	22.1	3.6	168	1.17	1.18	112.6	2.1	high	9	5.1	0.73	34	10.6
PS	67	F	6	1	22.3	4.2	109	1.49	0.81	127.6	1.5	high	8.7	6.3		18	11.8
PS	61	M	15	1	22.9	3.8	112	1.06	0.95	97.56	1.6	high		4.7	2.71	39	6.6

PS	61	M	5	1	21.3	3.9	185	1.28	0.67	128.7	1.5	high		6.3		14	9
PS	60	M	5	1	20.8	3.7	166	1.36	0.79	94.11	1.8	high	8.2	3.9		25	11.5
PS	62	M	5	1	23.8	4.1	219	1.13	0.79	117.1	1.3	high	9.1	5.4	0.04	22	10.6
PS	61	M	5	1	21.3	3.9	140	0.93	0.84	113.6	1.5	high	8.9	4.1	0.02	23	
PS	28	M	5	1	18.3	4.1	122	1.52	0.66	106.1	1.8	high	9.7	4.1	0.4	29	10.2
PS	63	M	4	1	22.6	3.7	167	1.29	1.02	156.3	1.5	high	8.7	3.7	0.83	23	9.1
PS	69	F	2	1	23.8	3.9	151	1.09	0.77	128.5	1.3	high	7.3	5.9	8.53	16	7.7
PS	57	M	6	1	25.9	3.6	113	1.34	0.95	102.8	0.6	high	8	4.6			10.4
PS	64	M	4	1	29.1	3.4	142	1.34	0.94	101.1	1.5	high		5.7	0.2	32	10.4
PS	56	F	3	1	18.6	4	185	0.97	1.01	106.7	1.5	high	9.3	6.2	0.65	26	11.1
PS	74	M	4	1	21.7	3.6	126	1.62	0.62	134.7	1.9	high	9.6	4.9	0.7	3	12.8
PS	62	M	4	1	20.4	3.6	126	1.66	0.89	119	2.1	high	8.8	4.7	0.04	22	8.8
PS	57	M	4	1	25.4	3.6	184	1.26	1.05	129.5	1.6	high	9.9	6.3	0.2	22	10.3
PS	60	M	4	1	23.2	4.1	189	1.23	0.91	120.6	1.9	high	8.7	6.1		25	9.6
PS	61	F	4	1	23.2	4.3	175	1.95	1.22	106.7	1.8	high	8.5	4.7	0.1	26	11.8
PS	79	F	4	1	23.4	3.5		1.29	0.98	129.7	2.1	high	10.6	3.9	0.1	27	11.3
PS	63	M	3	1	24.4	4.1		1.16	0.84	97.13	2.1	high		3.2	0.12		
PS	54	M	3	1	22.3	3.9	112	1.24	0.81	84.75	2.1	high	8	4.1	0.02	34	9.2
PS	60	M	3	1	18.3	3.7	120	1.45	0.97	123.3	2.1	high		4.5	0.05	30	10
PS	75	M	3	1	20.8	3.6	129	1.16	0.92	109.1	1.6	high	8.3	5.3		27	9.7
PS	85	M	2	1	22.2	3.7	184	1.3	1.01	116.6	1.6	high	8	6.2		25	9.5
PS	85	M	2	1	21.3	3.3		1.65	0.83	85.17	1.6	high	9.7	6.2		34	10.6
PS	60	M	2	1	25.3	3.9	118	1.46	0.71	98.1	1.8	high	9.3	4.6		45	12.6
PS	53	M	3	1	24	3.9		0.99	0.72	81.55	1.8	high	7.7	5.3		33	9.3
PS	52	F	4	1	18.5	4.1		1.66	0.97	108.8	1.9	high	10.5	3.7		31	9.6
PS	60	M	2	1	27.2	3.5	103	1.11	0.99	101.4	1.6	high		4.2	0.3	37	10.4
PS	66	M	2	1	26.8	3.4	121	1.15	0.82	89.77	2.1	high	8.3	7	0.11	25	10.6
PS	66	F	2	1	15.3	3.2	146	1.53	0.85	52.35	1.5	high	7.9	5.9	0.38	29	11.2
PS	35	M	4	1	30.7	4.2	146	1.95	0.95	120	1.1	high	7.8	4.6	6.39	22	10.1
PS	61	M	2	1	20.7	3.8	118	1.8	0.91	125.5	1.8	high	8.7	5.8	19.7		9.2
PS	70	F	11	1	20.8	3.7		1.17	0.95	100.1	1.9	high	9.3	5		33	9.6
PS	64	F	10	1	19.7	3.7	206	1.44	0.89	111.2	1.6	high	8.5	5.2		22	10.3
PS	51	M	11	1	21.7	3.8		1.01	0.9	124	0.9	high	9.5	6.1			13.5
PS	59	F	10	1	21.8	3.9	142	1.19	0.9	111.9	1.5	high		5.5	0.03	22	10.3
PS	39	F	9	1	22.1	3.7	114	1.45	0.98	133.4	1.6	high	9.4	5	0.3		10.6
PS	66	M	14	1	22.6	3.6	174	2.02	0.87	125.2	1.5	high	9.5	5.5	0.1	28	11
PS	59	M	18	1	24.3	3.6	94	1.98	0.92	112.2	1.6	high	9.6	5.5	0		9.7
PS	67	M	14	1	21.3	4	215	1.66	0.81	128	1.6	high		3.2	0.05		10.2
PS	64	M	22	1	20.7	3.6	173	1.75	0.76	87.15	1.6	high		3.6	0.02		10.6
PS	66	F	28	1	22.2	4	222	1.33	0.9	121.9	1.5	high	9.2	4.8	0.08	23	11.5
PS	58	M	17	1	19.7	4		1.69	1.06	125	1.8	high	9.3	6.3		20	9.4
PS	65	M	13	1	24.8	4.6	160	2.2	0.85	124.3	1.1	high	9.1	4.5	0.03	30	10.8
PS	65	F	32	1	24.7	3.3	147	1.25	0.94	118.9	1.8	high		4.2	0.05		9.3
PS	80	M	18	1	18.9	3.3	193	1.07	0.91	124.9	1.6	high		5.5	0.06		11.7
PS	65	M	27	1	22.5	3.7	205	1.06	0.72	89.76	1.3	high		7.5			11.2
PS	62	F	20	1	22.2	3.6	115	1.6	0.93	124.6	1.3	high		6.6		27	9.7
PS	45	M	28	1	16.2	3.2	213	1.71	1.06	78.82	2.1	high	9.4	4.8	0.37	30	11.5
PS	58	M	25	1	19.3	3.6		1.53	0.91	115.1	2.5	high	8.4	6.9		25	10.4
PS	56	M	18	1	22.6	3.8		1.63	0.83	118.1	2.1	high	9.9	4.1		30	9.2
PS	57	M	33	1	17.5	4.2	107	1.05	1.09	112.1	1.9	high		4.9		32	10.4
PS	58	M	17	1	18	4.1		1.48	0.86	100.5	1.5	high	9.9	4.5		18	10.6
PS	67	M	16	1	21.3	3.7	153	1.5	0.69	112.1	1.5	high	10.8	4.7		37	11.8
PS	53	M	16	1	25.2	3.7	133	1.14	1.1	124.9	1.6	high		5.6	0.1	29	10.1
PS	61	F	29	1	22.2	3.2	161	1.28	0.8	125	1.3	high	11	5.1	0.1		10.1
PS	48	F	26	1	18.5	3.9	125	1.83	1.45	124.6	1.8	high	8.9	5.7	0.87	27	10.2
PS	71	F	17	1	20.9	4		1.65	0.71	105.9	2.1	high	9.9	4.7		25	12.6
PS	62	M	16	1	19.7	3.4	137	1.32	0.87	139.8	1.3	high		6.1		28	9.8
PS	67	M	13	1	19.5	4		1.54	0.92	97.6	1.5	high	9.6	5.7		25	10.1
PS	64	M	18	1	22.5	3.7		1.45	0.92	101.2	1.5	high	13.1	1.8		25	9.8

PS	59	M	18	1	18.2	3.5	181	1.51	0.96	118.2	1.6	high	8.9	7	0.05	26	10.4
PS	55	M	16	1	22.9	4.2	191	1.53	0.76	100.8	1.6	high	9	6.5	0.05	25	11.8
PS	68	F	30	1	17.6	3.5	152	1.03	0.83	69.76	2.1	high	9.1	5.5			11.3
PS	66	F	27	1	20.6	3.7		1.29	0.9	121.8	2.1	high	10.5	5		24	10.3
PS	67	F	26	1	23.8	3.5	129	1.31	0.83	77.26	1.5	high	9.5	6.9			11.7
PS	64	F	13	1	23.2	3.9	156	1.62	1.17	154.7	1.5	high	8.8	2.9			9.9
PS	72	M	13	1	21	3.6	206	2.27	0.73	111.3	1.5	high		5.6	0.16	26	11.6
PS	61	M	13	1	19.8	3.8	113	1.39	1	108.8	1.2	high	9.6	5.3	0.27	29	11.1
PS	63	F	10	1	17.8	3.8	102	1.18	0.98	124.8	2.1	high	8.3	6.4	0.05	29	11.8
PS	61	M	12	1	17.1	3.6	162	1.29	1.03	109.3	1.5	high	11.5	7.9			12.6
PS	70	M	12	1	23.6	3.9	157	1.24	0.91	109	1.5	high	9.4	5.4			10.3
PS	37	M	12	1	20.1	4	210	1.79	1.08	139.1	1.6	high	9.4	3.9	0.55	29	10
PS	74	M	6	1	17.8	3.3	129	1.12	1.08	89.91	1.8	high	9.2	3.2	0.19	26	11.7
PS	61	F	9	1	21.8	4	197	1.5	0.89	139.4	1.6	high	9.5	4.2	0.07	20	10.6
PS	70	F	8	1	22.1	3.7	156	1.6	1.05	131.2	1.8	high	7.9	4.5			8.8
PS	58	F	8	1	21.5	3.2	160	1.56	0.75	94.87	1.6	high	9.3	4.1		36	
PS	70	M	8	1	24.2	3.6	137	2.22	0.73	102.8	1.8	high		7.8		28	11.4
PS	78	F	12	1	21.6	3.1	121	1.58	1	110.5	1.1	high	9.1	3.8	0.14		10.2
PS	57	F	27	1	24.2	4	175	1.35	1	103	1.8	high		5.4	0.05		10.8
PS	61	M	25	1	20	4	96	2	1.32	146.4	1.8	high		5.4	0.06	34	10.8
PS	71	F	24	1	20.9	3.8	173	1.15	0.95	106.5	1.9	high	9.1	7.5	0.66	38	11
PS	69	F	24	1	17.5	4.2	214	1.54	1.23	104.4	1.6	high	9	6.3	0.08	25	10.3
PS	66	F	22	1	21.6	3.8	141	0.69	1.02	101.2	1.3	high	8.7	5.7	0.02	20	11
PS	65	M	22	1	20.5	3.9	160	1.32	0.98	110.8	1.8	high		5.1	0.11	21	10.7
PS	71	M	22	1	23.1	3.9	133	1.56	1.45	126.6	1.4	high	7.8	5.6	0.02	32	11.1
PS	65	M	21	1	22.8	2.7	138	0.95	0.87	113.3	1.8	high		5		24	11.8
PS	70	M	21	1	17.5	3.6	141	1.55	0.86	94.12	1.6	high	9.5	5.9		27	10.8
PS	59	M	20	1	19	4.1		1.11	1.17	106.8	1.6	high		6.3	0.1	29	9.6
PS	68	M	19	1	19.5	4.1	172	1.42	0.95	135.5	1.6	high	9	5.7	0.05	31	11.6
PS	73	F	16	1	22.6	4.3	164	1.37	0.94	108.9	1.6	high	8.5	3.1	0.31	19	12.1
PS	52	M	16	1	21.6	4.3	136	0.97	0.84	95.53	1.5	high		3	0.42	16	11.1
PS	48	M	15	1	19.7	4.6	146	1.33	1.22	128.3	2.2	high	9.1	5.3	0.08	28	10.6
PS	53	M	14	1	20.4	4.4	174	1.58	1.06	112.8	2.1	high		5.1		36	11.5
PS	69	F	13	1	21	4	200	2	1.15	113.4	1.3	high	8.9	8.9	0.1		10.2
PS	65	F	13	1	19.4	4	166	1.46	1	111.9	1.6	high	9.3	6.2	0.14	26	10.5
PS	59	M	12	1	21.9	4.4	111	1.49	1.14	122.9	1.6	high	9.2	4.9	0.2	36	10.8
PS	75	M	12	1	18.7	4.2	177	1.53	0.93	153.2	1.6	high	9.5	4.4	0.09	23	9.7
[truncated: 635,615 more chars]
